# Supplementary material for: Multi-modal molecular programs regulate melanoma cell state
Source: Nat Commun. 2022 Jul 9;13:4000. doi: 10.1038/s41467-022-31510-1 (PMC9271073; doi:10.1038/s41467-022-31510-1)
Supplement: Supplementary file 1 — Supplementary Information [file 41467_2022_31510_MOESM1_ESM.pdf]

Multi-modal molecular programs regulate melanoma cell state and clinical outcomes.

Authors: Miles C. Andrews, Junna Oba, Chang-Jiun Wu, Haifeng Zhu, Tatiana Karpinets, Caitlin A. Creasy, Marie-Andrée Forget, Xiaoxing Yu, Xingzhi Song, Xizeng Mao, A. Gordon Robertson, Gabriele Romano, Peng Li, Elizabeth M. Burton, Yiling Lu, Robert Szczepaniak Sloane, Khalida M. Wani, Kunal Rai, Alexander J. Lazar, Lauren E. Haydu, Matias A. Bustos, Jianjun Shen, Yueping Chen, Margaret B. Morgan, Jennifer A. Wargo, Lawrence N. Kwong, Cara L. Haymaker, Elizabeth A. Grimm, Patrick Hwu, Dave S.B. Hoon, Jianhua Zhang, Jeffrey E. Gershenwald, Michael A. Davies, P. Andrew Futreal, Chantale Bernatchez, Scott E. Woodman.

Supplementary Information

Supplementary Figure 1.

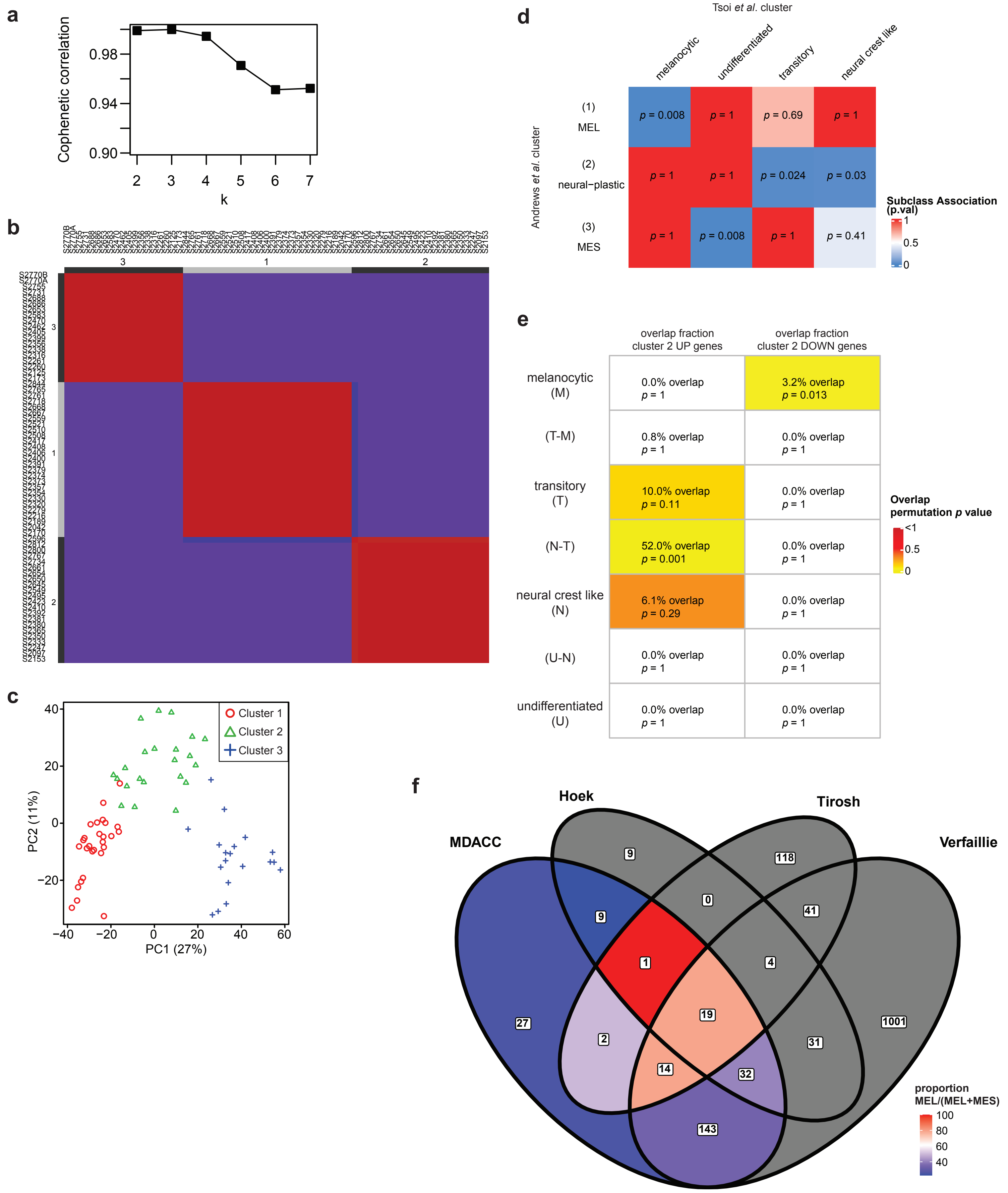

Supplementary Figure 1: Unsupervised cNMF clustering and gene set overlaps of early-passage melanoma MDACC cell lines. Using the 1500 most-variably expressed genes across a cohort of early-passage melanoma cell lines (n=68), cNMF clustering was optimized by cophenetic correlation (**a**) and consensus clustering (**b**) with 1000 iterations revealing 3 distinct mRNA expression-based clusters. (**c**) Principal component analysis demonstrates the clear separation between clusters. (**d**) Heatmap of cross-cohort class similarities measured by SubMap and (**e**) gene set overlaps between the MEL and MES-based clusters and transcriptomic states described by Tsoi *et al*. Shown in (d) are summary subclass association *p* values derived from Bonferroni-corrected one-sided Fisher inverse chi-square statistics estimated on subclass gene set enrichment scores, and in (d) one-sided *p* values of gene set overlap using permutation testing with 1000 iterations. (**f**) Gene set overlaps between MEL and MES genes and the published melanoma transcriptome type gene sets indicated. The relative proportion of MEL to MES genes within each overlap segment is indicated by color, with red representing more MEL and blue representing more MES. Source data are provided as a Source Data file.

Supplementary Figure 2.

a

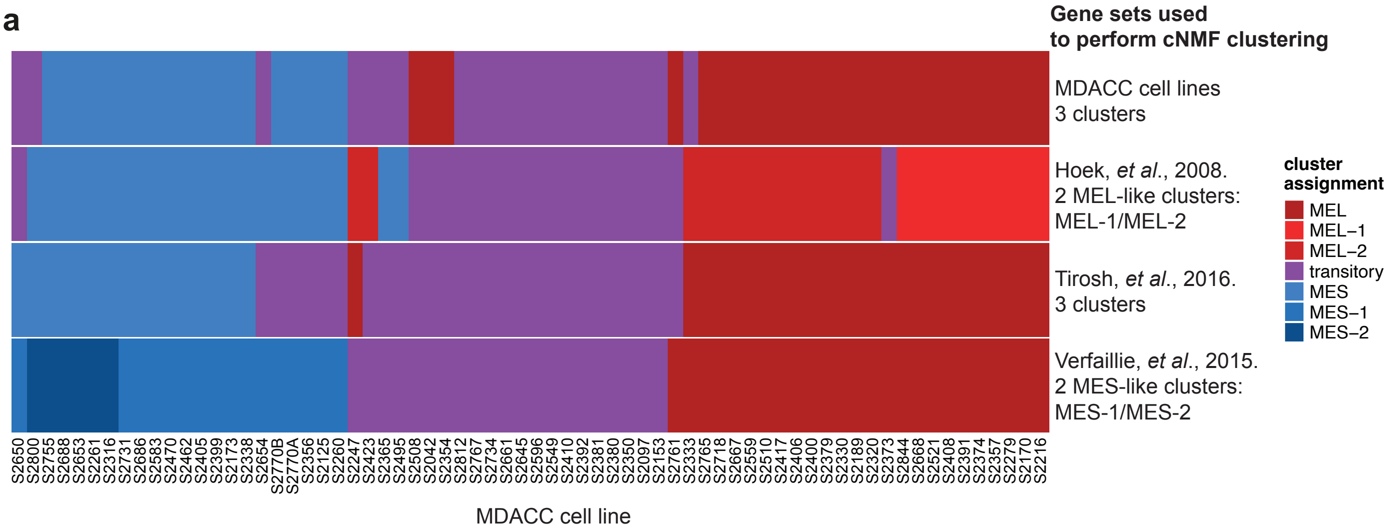

b

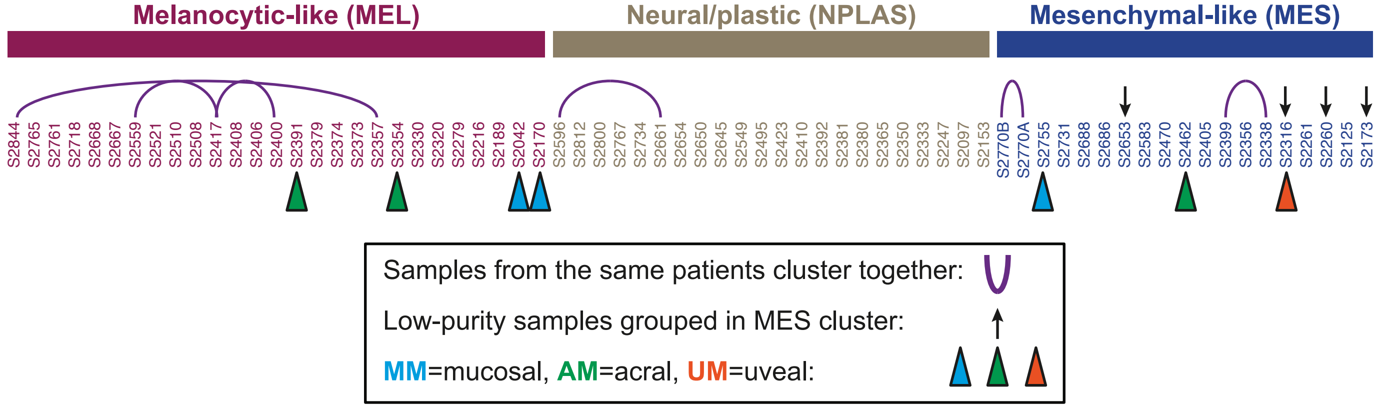

c

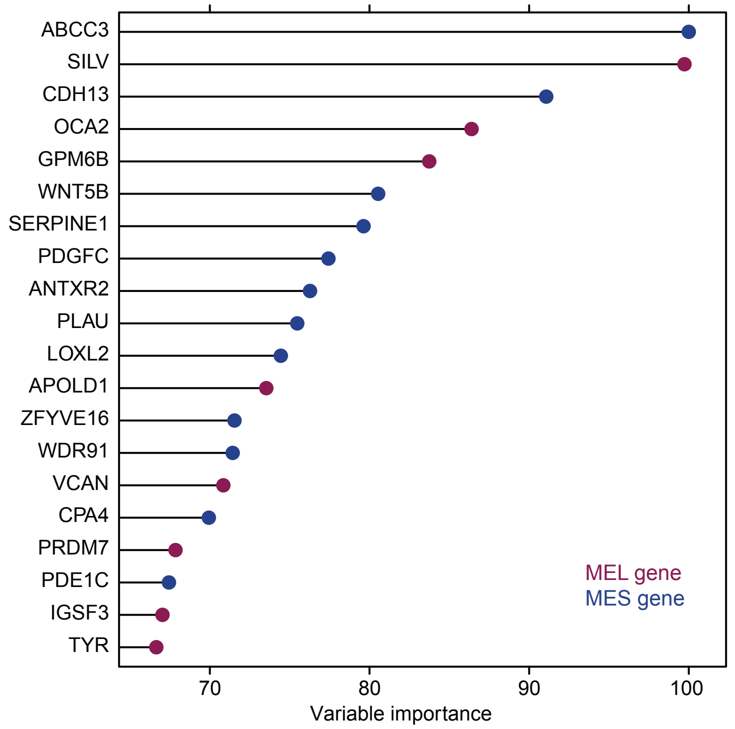

d

	**Full cohort** (n=68 lines from 62 patients)	**Refined cohort** (n=53 lines from 53 patients)
	Number (%)	Number (%)
**Sex**		
Male	37 (60%)	33 (62%)
Female	25 (40%)	20 (38%)
**Subtype**		
Non-acral	50 (74%)	41 (77%)
Acral lentiginous	3 (4%)	1 (2%)
Mucosal	3 (4%)	-
Uveal	1 (2%)	-
Unknown primary	11 (16%)	11 (21%)

Supplementary Figure 2: Comparative gene set clustering and cohort refinement. (**a**) Comparison of cell line clustering based on MEL and MES genes and three other published melanoma transcriptomic types. Optimal cNMF-based clustering solutions for different cohorts produced 2 MEL-like clusters or 2 MES-like clusters as indicated in the figure. (**b**) Based on clustering derived from the cNMF approach, cell lines derived separately from tumors arising in the same patient remain closely related, and lower-purity cell lines appear within the MES cluster. Cell line names are colored according to the cNMF clustering (red, Cluster 1; khaki, Cluster 2; blue, Cluster 3). Cell lines derived from mucosal and uveal melanomas are also indicated. (**c**) Variable importance plot of the top 20 most informative genes used in the random forest cluster classifier model. (**d**) Full cohort composition used in initial cluster discovery, and refined cohort used for subsequent molecular analyses considering only non-mucosal/uveal subtypes, higher-purity samples, and only one cell lines derived from an individual patient. Source data are provided as a Source Data file.

Supplementary Figure 3.

a

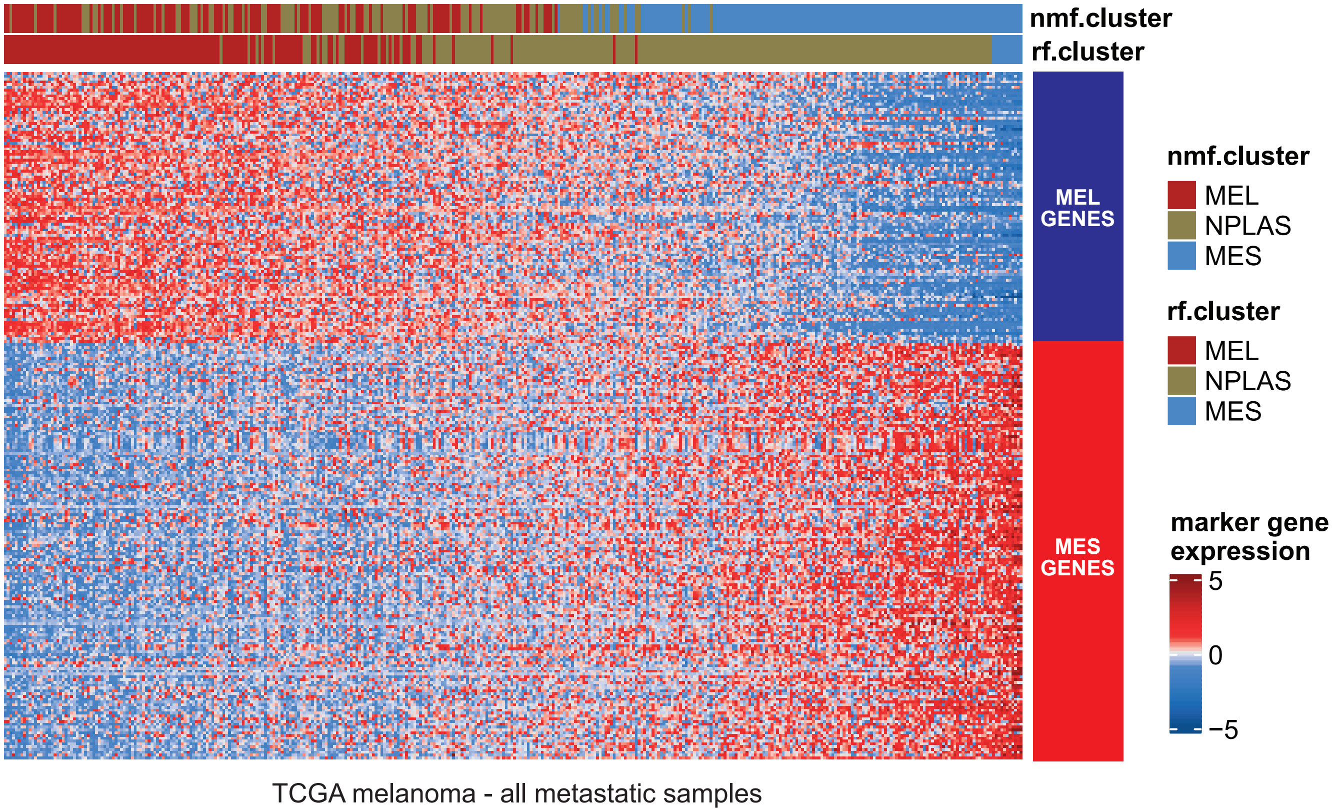

b

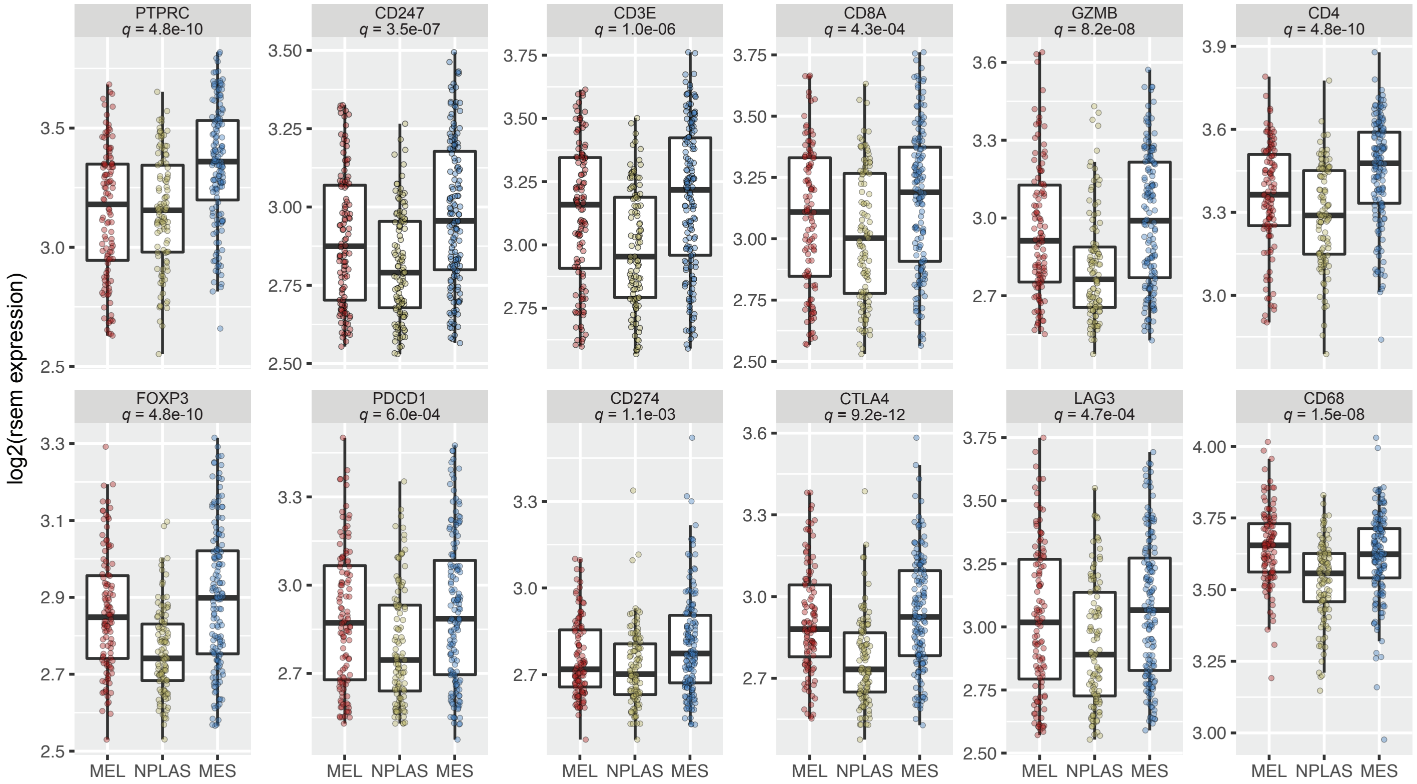

Supplementary Figure 3: MCS clusters and key immune gene expression differences in TCGA melanoma samples. (**a**) Heatmap and unsupervised cNMF clustering analysis was performed on all metastatic TCGA melanoma samples (n=368, purity ≥0.50) based on MEL and MES marker gene expression, showing overall preserved separation into predominantly MEL-like or predominantly MES-like clusters, although with less distinct separation than was seen in only high-purity samples. cNMF-based clusters (nmf.cluster) and the cluster prediction from the cell line-trained random forest model (rf.cluster) are shown above; samples are ordered left-to-right by decreasing MEL-MES score (see Methods). (**b**) Specific marker genes supportive of leukocyte subsets within all metastatic TCGA melanoma samples (n=368) reveal differential abundances between MCS groups. Boxplots indicate the median (thick bar), first and third quartiles (lower and upper bounds of the box, respectively), lowest and highest data value within 1.5 times the interquartile range (lower and upper bounds of the whisker), and all individual data points are shown. The q-values shown are Benjamini-Hochberg corrected two-sided Kruskal-Wallis p-values comparing marker gene expression across all three sample classes. Source data are provided as a Source Data file.

Supplementary Figure 4.

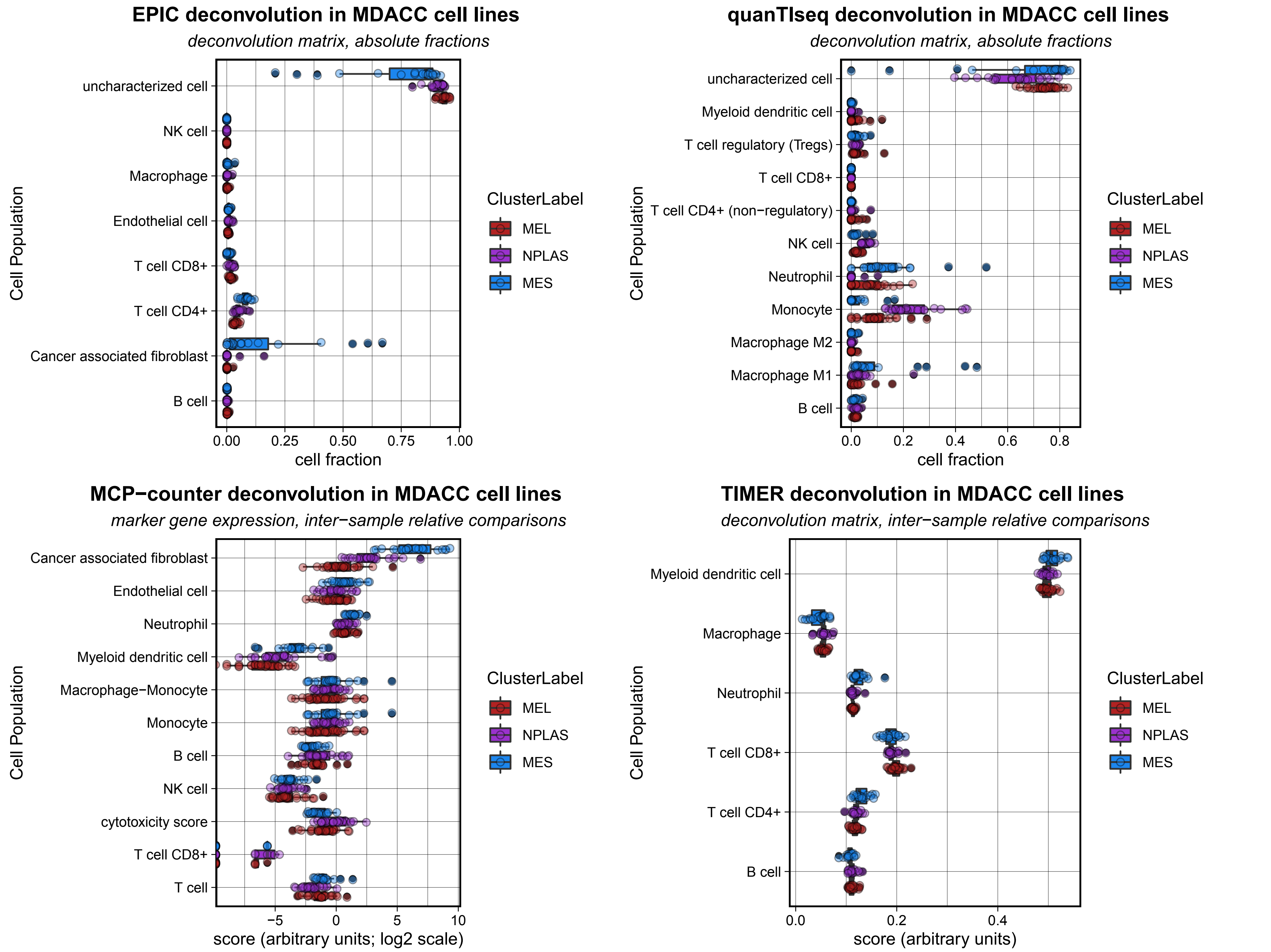

Supplementary Figure 4: Transcriptomic immune deconvolution in MDACC cell lines. Four immune deconvolution methods were applied to gene expression data of the MDACC melanoma cell line panel (n=53), with cell lines grouped by MEL/NPLAS/MES clusters, showing evidence of spurious myeloid and/or stromal signatures particularly in MES samples. The cell populations evaluated by each algorithm are shown along the y-axis with output scores/fractions along the x-axis. Horizontal boxplots indicate the median (thick bar), first and third quartiles (lower and upper bounds of the box, respectively), lowest and highest data value within 1.5 times the interquartile range (lower and upper bounds of the whisker), and all individual data points are shown. The deconvolution strategy and output type are indicated above each graph: EPIC, quanTIseq and TIMER utilize a gene expression deconvolution matrix, whereas MCP-counter employs subset-defining marker gene expression values. EPIC and quanTIseq output absolute cell fractions whereas MCP-counter and TIMER output arbitrary unit values which are suitable for inter-sample relative abundance comparisons. Source data are provided as a Source Data file.

Supplementary Figure 5.

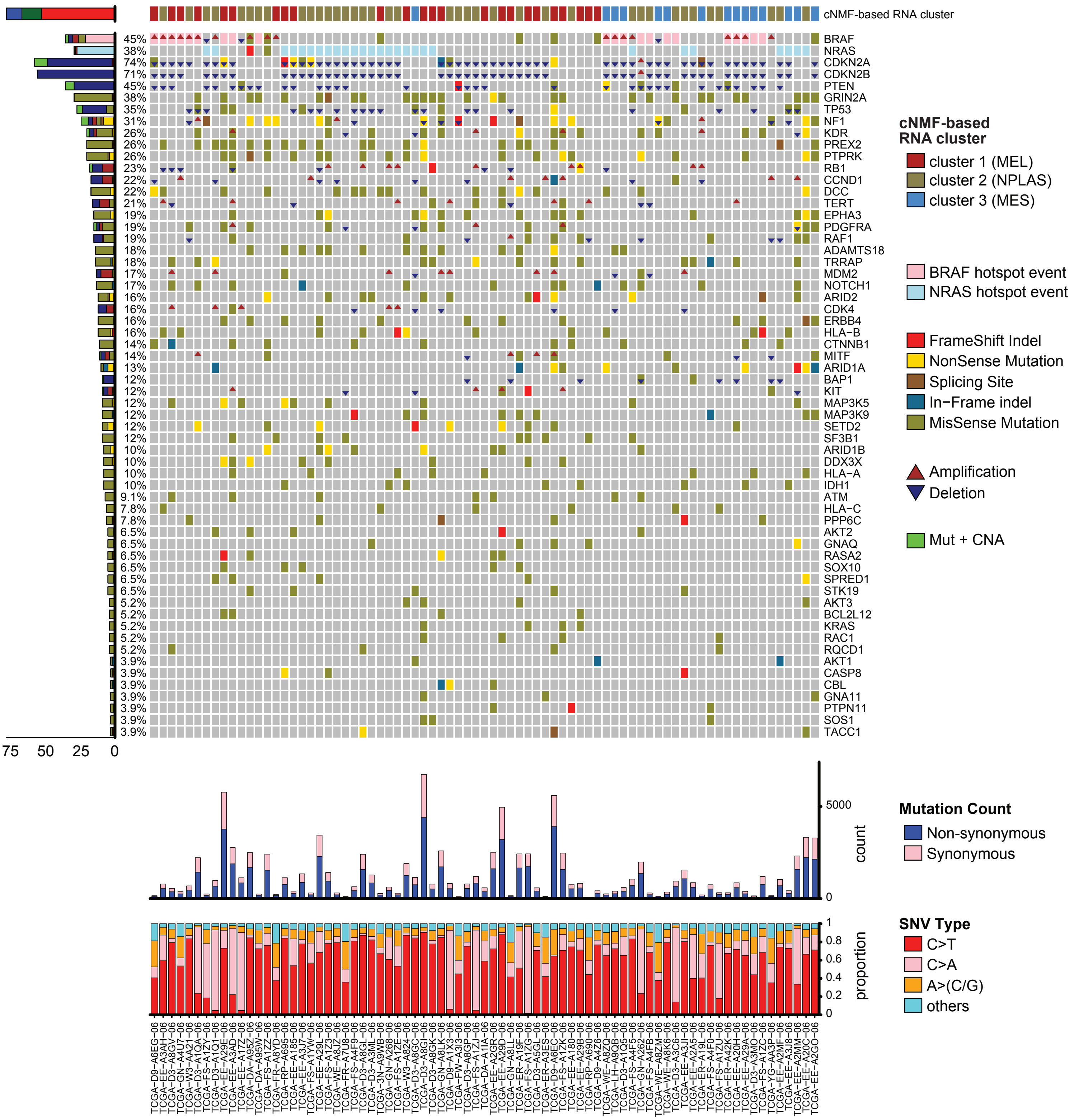

TCGA melanoma - high purity metastatic samples

Supplementary Figure 5: Genomic features of high-purity TCGA melanoma metastases. Oncomap displaying somatic variants identified from whole-exome sequencing affecting a panel of genes frequently altered in melanoma. High-purity metastatic samples of the TCGA melanoma cohort (n=77) are shown indicating the cluster assignments assigned by cNMF performed on all TCGA melanoma samples (primaries and all metastatic samples), the presence of *BRAF* and *NRAS* mutations, and other mutations or copy number alterations (CNA). The histogram at left indicates the cumulative frequency of genomic alterations (all types) for each gene. Total synonymous and non-synonymous mutation count (number) and type of single nucleotide variants (proportion) are indicated in the bar graph and stacked bar graph below, respectively. Source data are provided as a Source Data file.

Supplementary Figure 6.

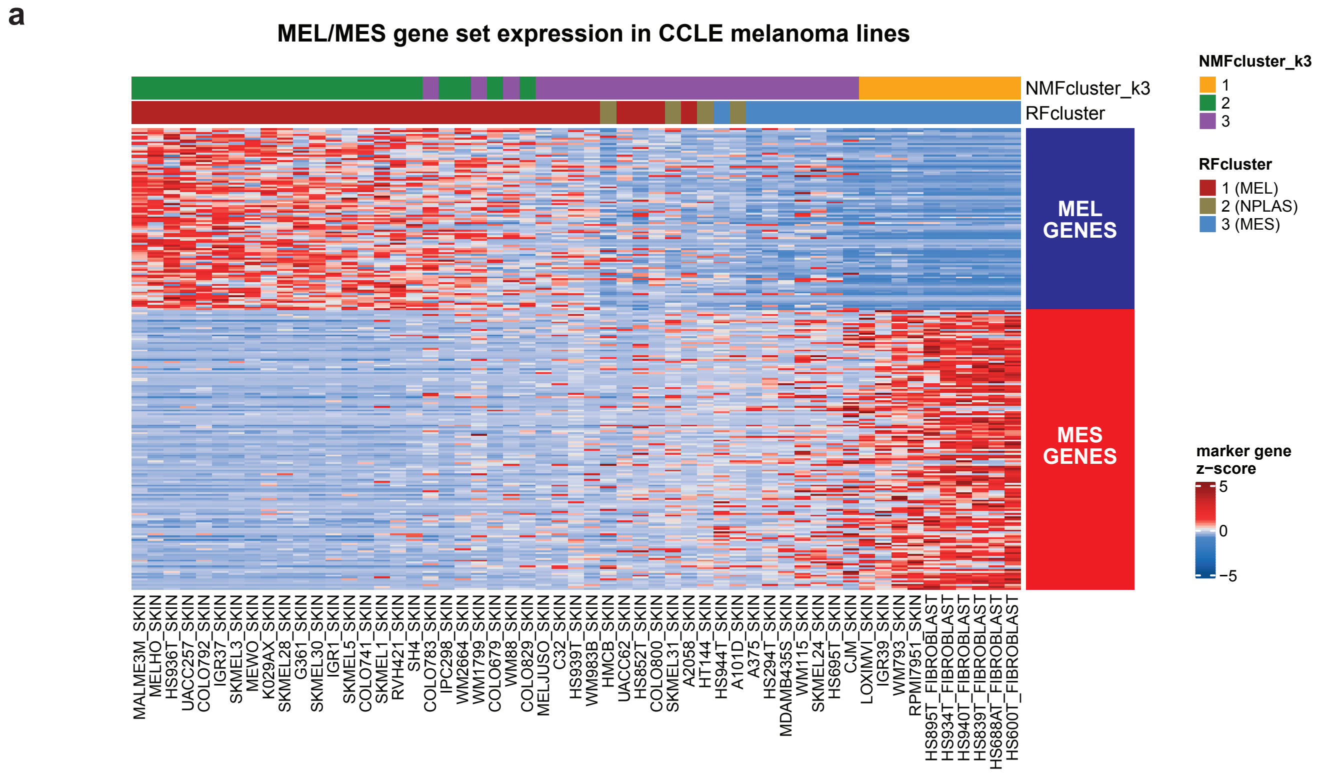

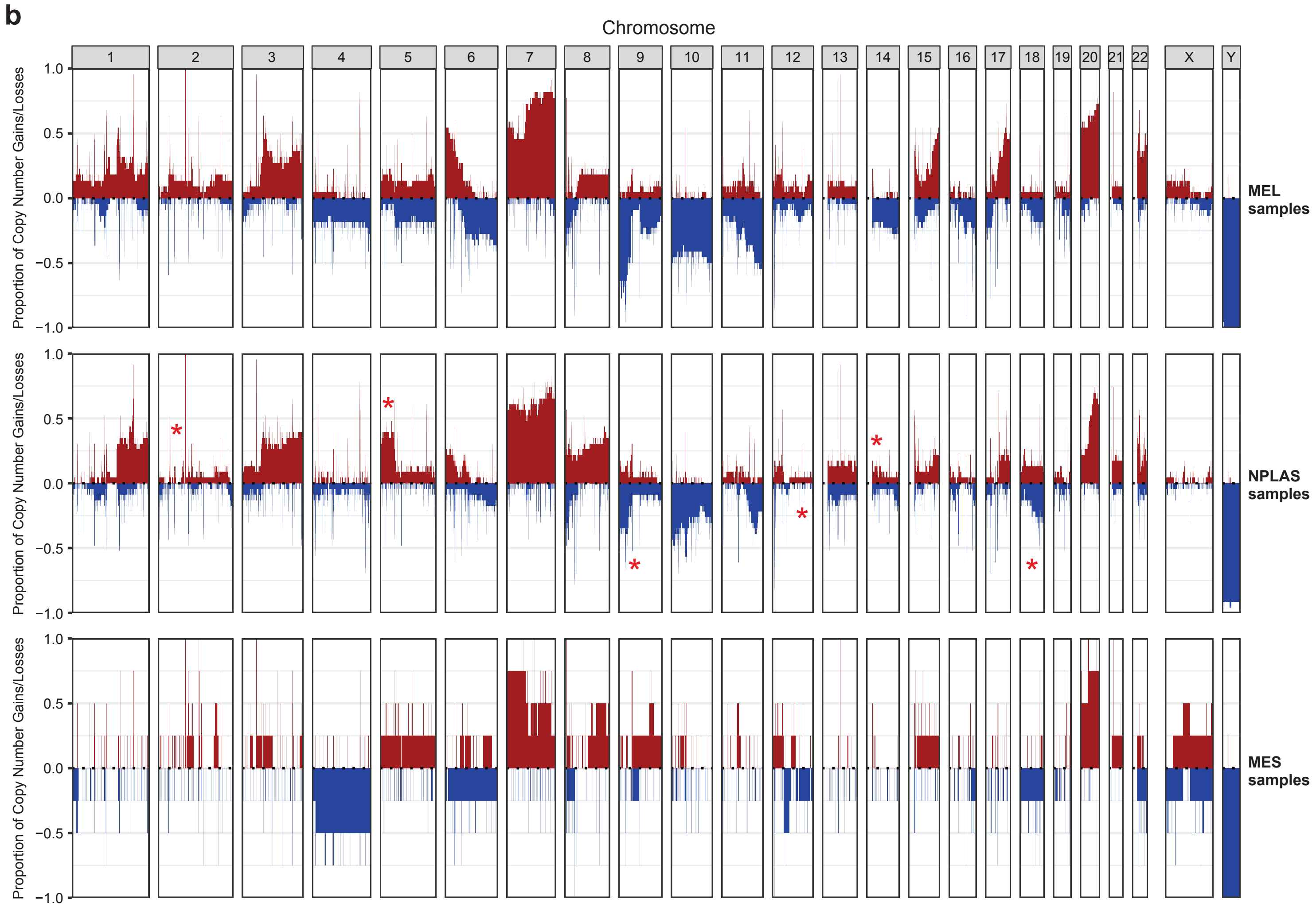

Supplementary Figure 6: MCS and copy number alteration of Cancer Cell Line Encyclopedia (CCLE) melanoma samples. (**a**) Heatmap of MEL and MES gene expression in CCLE melanoma samples with available gene expression data demonstrating clear segregation into MCS groups. cNMF-based clusters (k=3; NMFcluster_k3) and random forest cluster predictions (RFcluster) are shown above; samples are ordered left-to-right by decreasing MEL-MES score (see Methods). (**b**) Genome-wide copy number alteration surveyed as segmented data loss/gain frequency plots contrasting profiles between the three cNMF-derived MCS identified as in (a). Regions of particular note displaying differential CNA profiles between MCS groups are indicated by red asterisks. Source data are provided as a Source Data file.

Supplementary Figure 7.

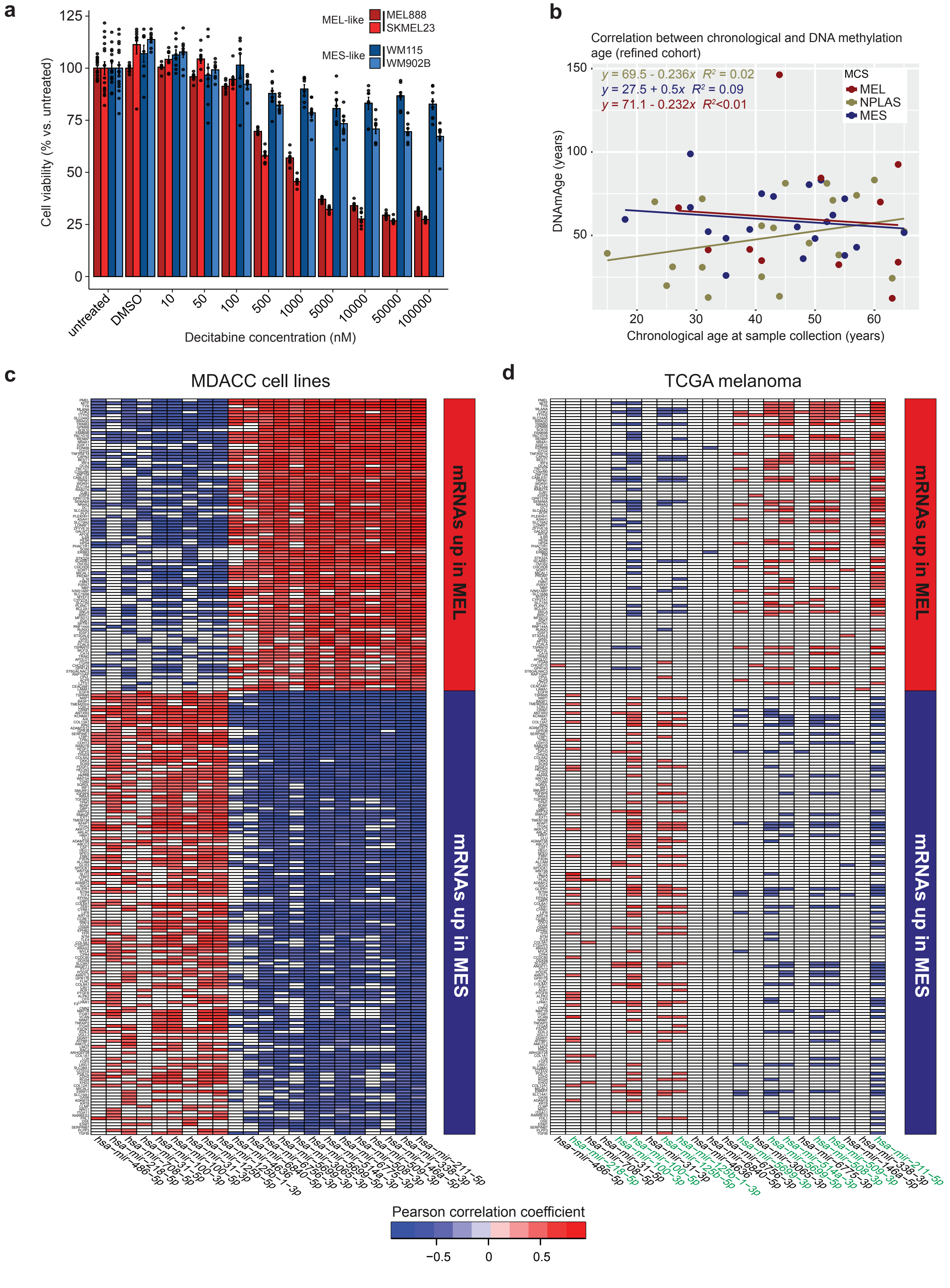

Supplementary Figure 7: Methylation effects and microRNA targeting of MEL and MES genes in MDACC cell lines and TCGA melanoma samples. (**a**) Melanoma cell lines exposed to increasing concentrations of the demethylating agent decitabine reveal greater sensitivity in MEL-like (high ECAD versus NCAD/ZEB1 expression) lines compared with MES-like (low ECAD versus NCAD/ZEB1 expression) lines. Bars show mean ± SEM of biological replicates (n=16 for untreated, n=8 for the other conditions), normalized to untreated cells for each line indicated. (**b**) Correlation between DNA methylation age (DNAmAge) and chronological age of the patient at the time the tumor from which each early-passage melanoma cell line was created was sampled, indicating MCS of each sample, and linear regression lines for each MCS. (**c-d**) Heatmaps of the Pearson correlation between expression of individual microRNAs (columns) and MEL/MES marker gene transcript abundances (rows) in (**c**) the cell line panel and (**d**) TCGA melanoma samples showing preservation of most cell line miR-gene correlations in tissue samples being particularly consistent for half of the marker miRs indicated by green labels in (b). Source data are provided as a Source Data file.

Supplementary Figure 8.

a

MEL (n=21) vs MES (n=11)

	FC	mean TPM **MEL**	mean TPM **MES**
LHFPL3-AS1	157.6	119.5	0.8
RP3-527G5.1	63.1	10.7	0.2
RP11-429E11.2	60.8	8.2	0.1
RP11-1055B8.3	49	23.8	0.5
LINC00518	37.4	16.6	0.4
AC009495.3	33.1	4.1	0.1
RP11-529E10.7	29.1	3.6	0.1
LINC00520	27.7	24.7	0.9
RP11-374M1.2	22.3	3.7	0.2
LINC00589	21.5	2.8	0.1
AC145110.1	20.2	4.6	0.2
RP4-718J7.4	17.6	5.1	0.3
RP11-420N3.2	14.9	2.9	0.2
RP1-155D22.2	14.7	2.9	0.2
RP11-161M6.2	12.9	1.4	0.1
RP3-395M20.8	10.5	8.3	0.8
AC005786.7	10.5	3.8	0.4
RP1-90G24.10	10.1	1.8	0.2
CELF2-AS1	9.3	1.1	0.1
LINC00473	9.2	23.2	2.5
RP11-561B11.6	-6.3	0.2	1.2
NR2F1-AS1	-6.5	0.8	5.4
SERTAD4-AS1	-6.5	0.2	1.5
RP11-274H2.3	-6.5	0.2	1.5
RP11-274H2.5	-6.9	0.2	1
RP11-426C22.4	-7.5	0.2	1.4
LINC00973	-7.6	0.9	6.8
RP11-356J5.12	-7.9	0.3	2.5
STARD4-AS1	-8.4	0.2	1.7
RP11-177H13.2	-9.2	0.2	1.9
RP11-879F14.2	-9.5	0.3	3.1
AC003092.1	-10.4	0.4	4.4
CTD-2541J13.2	-11.2	0.2	2.4
LINC00944	-11.6	0.1	1.5
BDNF-AS	-12.2	0.1	1.7
EMX2OS	-16.4	0.1	2.2
FENDRR	-20.5	0.2	3.4
CTD-2354A18.1	-21.8	0.3	7.2
RP11-244F12.3	-22.1	0.2	3.7
MIR137HG	-31.6	0.1	3.7

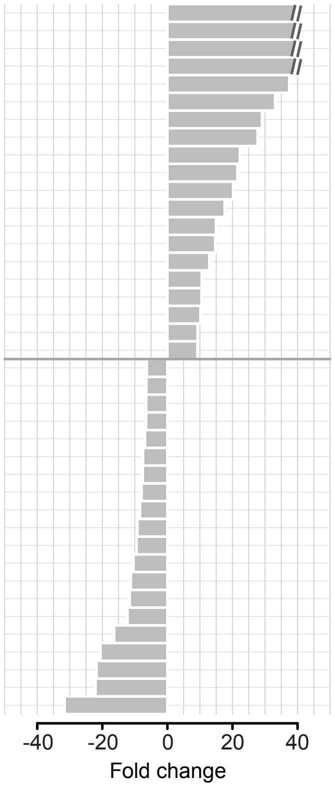

b

MES (n=11) vs other (n=42)

	FC	mean TPM **MES**	mean TPM **other**
RP11-334E6.12	56.9	7.4	0.1
APCDD1L-AS1	43	5.2	0.1
MIR31HG	14.3	2.5	0.2
CTD-2541J13.2	14.2	2.4	0.2
GS1-600G8.5	12.3	1.5	0.1
LINC01085	12.1	1.2	0.1
FENDRR	10.8	3.4	0.3
RP11-490M8.1	9.7	1.2	0.1
AC007319.1	9.3	1.2	0.1
MIR137HG	9.1	3.7	0.4
LINC00973	7.9	6.8	0.9
BDNF-AS	6.3	1.7	0.3
RP11-879F14.2	6.1	3.1	0.5
AC003092.1	5.2	4.4	0.8
GAS6-AS2	4.7	1.4	0.3
HAS2-AS1	4.7	2.7	0.6
LOXL1-AS1	4.7	3.3	0.7
RP11-244F12.3	4.3	3.7	0.9
RP11-618G20.1	4.3	4	0.9
RP11-356J5.12	4.2	2.5	0.6
RP1-90G24.10	-7.8	0.2	1.4
RP11-143A12.3	-8	0.2	1.6
RP11-3L21.2	-8.1	0.3	2.7
RP11-347E10.1	-8.8	0.4	3.5
RP11-420N3.2	-11.1	0.2	2.1
AC145110.1	-11.9	0.2	2.7
RP1-155D22.2	-12.7	0.2	2.5
RP11-141M1.3	-12.7	0.2	2
RP4-718J7.4	-13.5	0.3	3.9
LINC00589	-13.7	0.1	1.8
LINC00520	-16.1	0.9	14.3
RP11-161M6.2	-17.7	0.1	1.9
AC009495.3	-20.1	0.1	2.5
RP11-374M1.2	-21.6	0.2	3.6
LINC00518	-25.5	0.4	11.3
RP11-529E10.7	-27.4	0.1	3.4
RP3-527G5.1	-31.7	0.2	5.4
RP11-429E11.2	-34.3	0.1	4.6
RP11-1055B8.3	-35.6	0.5	17.3
LHFPL3-AS1	-94	0.8	71.3

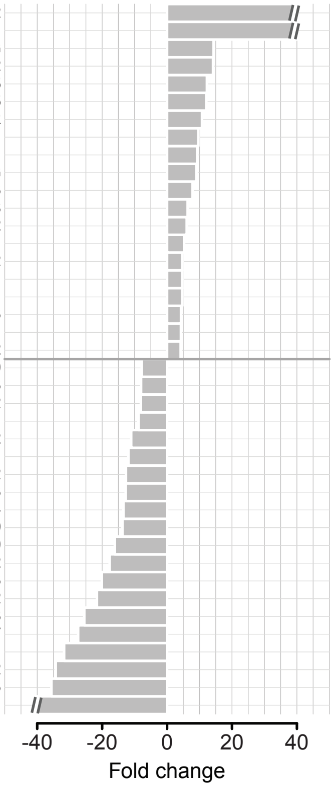

c

MEL (n=21) vs other (n=32)

	FC	mean TPM **MEL**	mean TPM **other**
LINC00698	17.8	2.2	0.1
AC104655.3	16	2.9	0.2
RP3-395M20.7	14.2	1.5	0.1
RP4-529N6.1	11.8	2.5	0.2
RP11-429E11.2	11.2	8.2	0.7
RP11-94H18.1	11	1.2	0.1
RP11-84D1.1	10.7	1.5	0.1
LINC00702	10.4	4.4	0.4
RP11-252C15.1	8.6	1.2	0.1
LINC00520	8.5	24.7	2.9
AC009495.4	8	2.5	0.3
ST8SIA6-AS1	8	1.1	0.1
LHFPL3-AS1	7.7	119.5	15.4
RP11-115J16.1	7.6	1.7	0.2
AC145110.1	7.4	4.6	0.6
RP11-217B1.2	7.4	1.6	0.2
FEZF1-AS1	7.4	1.4	0.2
RP11-164N3.2	6.7	1	0.2
AC009495.3	6.6	4.1	0.6
RP11-400N13.3	6.3	2.9	0.5
RP11-366L20.3	-4.5	0.3	1.3
RP11-879F14.2	-4.7	0.3	1.5
RP11-37L2.1	-4.8	0.4	1.8
STARD4-AS1	-5.1	0.2	1.1
SERTAD4-AS1	-5.1	0.2	1.2
AC003092.1	-5.5	0.4	2.3
RP11-141M1.3	-5.5	0.4	2.5
AL035610.1	-5.8	0.2	1.1
RP1-79C4.4	-5.8	0.2	1.3
RP11-177H13.2	-5.9	0.2	1.2
GDNF-AS1	-6.1	0.4	2.2
AF121898.3	-7.2	0.2	1.3
RP11-586K2.1	-7.3	0.4	2.6
CTD-2354A18.1	-8.7	0.3	2.9
FENDRR	-8.9	0.2	1.5
EMX2OS	-9	0.1	1.2
ADAMTS9-AS1	-11.2	0.1	1.5
RP11-244F12.3	-13.7	0.2	2.3
MIR137HG	-14.8	0.1	1.7
H19	-16	1	15.3

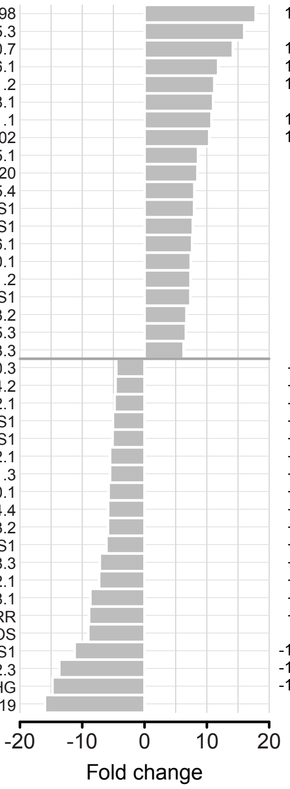

d

NPLAS (n=21) vs other (n=32)

	FC	mean TPM **NPLAS**	mean TPM **other**
RP11-141M1.3	11.6	3.5	0.3
RP11-713M15.2	11.4	1.2	0.1
RP11-37L2.1	11.3	2.6	0.2
RP1-142L7.5	7.1	1.3	0.2
ADAMTS9-AS1	6.9	1.9	0.3
RP11-9L18.3	6.5	2.2	0.3
CHRM3-AS2	6.2	1.2	0.2
RP11-586K2.1	5.7	3.5	0.6
RP11-6O2.4	5.6	1.4	0.2
GDNF-AS1	5.5	2.9	0.5
HPN-AS1	5.3	1.5	0.3
LINC00499	4.1	1.1	0.3
RP11-472N13.3	4	1.5	0.4
AC064875.2	3.9	8.3	2.1
RP11-474O21.5	3.8	2.7	0.7
RP11-370B11.3	3.8	1.6	0.4
RP11-128P17.4	3.6	1.3	0.4
RP11-549B18.1	3.5	3	0.8
RP11-34A14.3	3.5	2	0.6
CTC-529P8.1	3.4	3.8	1.1
CTD-2319I12.4	-1.7	0.7	1.2
AP001258.4	-1.7	0.9	1.6
RP11-298J20.4	-1.9	1	1.8
LINC00152	-1.9	12.3	23.2
AC017002.2	-2.1	2.6	5.6
AC017002.1	-2.2	1.2	2.7
TERC	-2.4	30.6	74.5
RP11-400N13.3	-5.9	0.3	1.9
LINC00702	-8.5	0.3	2.9
CTD-2541J13.2	-10.8	0.1	1.3

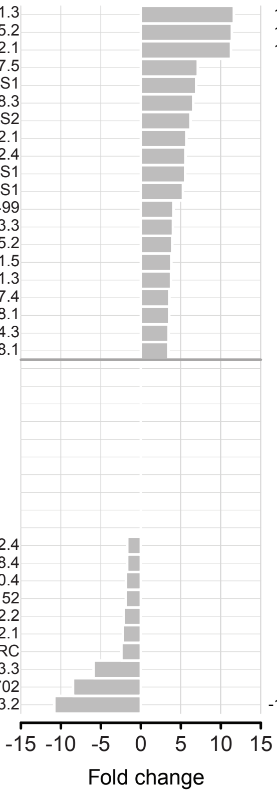

Supplementary Figure 8: Differential lncRNA expression between MCS in MDACC cell lines. Pairwise comparisons of lncRNA expression for (**a**) MEL versus MES, (**b**) MES versus (MEL or NPLAS), (**c**) MEL versus (MES or NPLAS) and (**d**) NPLAS vs (MEL or MES), expressed as fold change showing the top 20 differentially expressed lncRNAs in either direction, with a minimum of 1.5 absolute fold change between the two groups. TPM: transcripts per million. Source data are provided as a Source Data file.

Supplementary Figure 9.

a

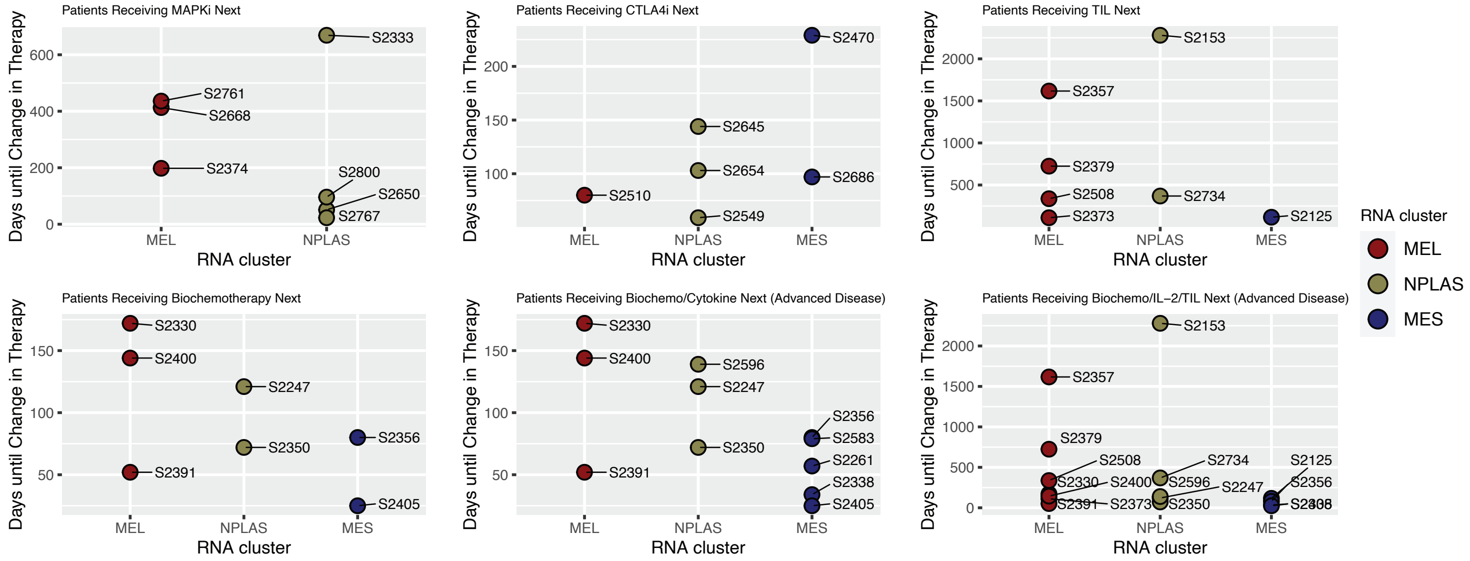

b

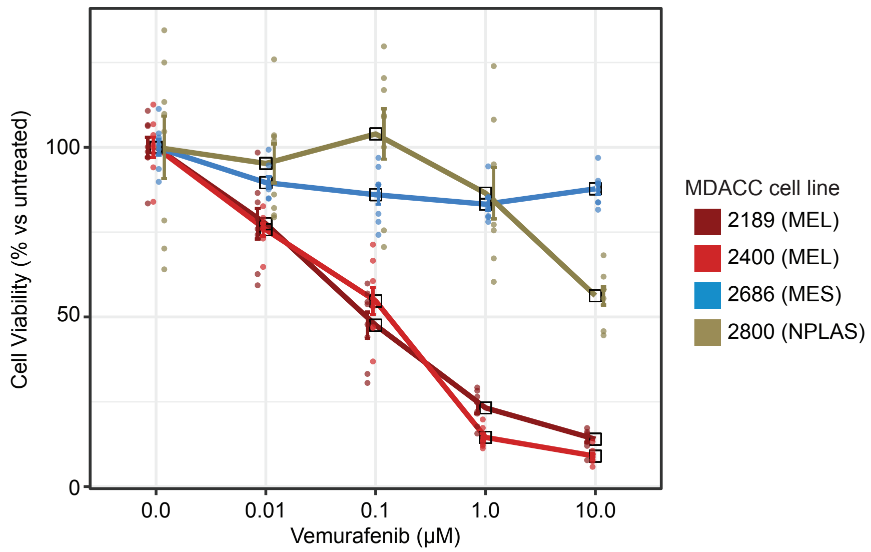

Supplementary Figure 9: MCS are associated with differential clinical outcomes. (**a**) Duration from initiation of systemic therapy until requirement for a change in therapy, stratified by MCS for patients receiving the next category of systemic therapy as indicated in each graph following harvest of the tumor from which a melanoma cell line was derived. (**b**) Dose-response viability curves measured by MTT assay for melanoma cell lines exposed to vemurafenib for 24 hours at the doses indicated. Shown are mean ± SD for biological replicates (n=8 each) normalized to untreated controls. Source data are provided as a Source Data file.

Supplementary Figure 10.

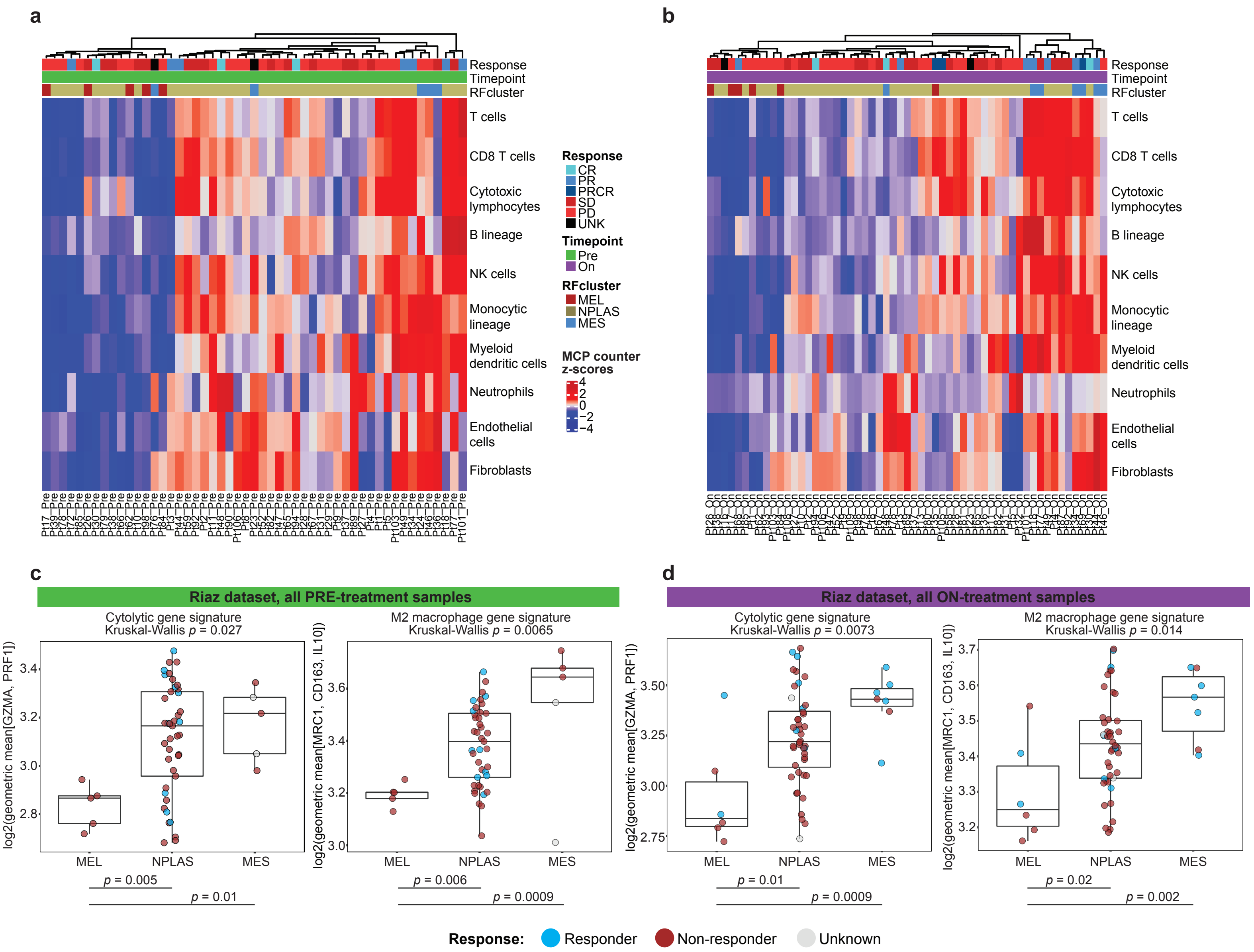

Supplementary Figure 10: (a) Pre-treatment (n=51) and (**b**) on-treatment (n=57) samples of the Riaz PD-1 inhibitor treated cohort demonstrate distinct immune/stromal content between samples, with relative lack of immune infiltrates in samples classified as MEL by the cell line-trained random forest classifier model compared with relatively diverse immune and stromal representation in MES samples, similarly to observations in TCGA melanoma samples based on MCP-counter transcriptomic deconvolution. In the same dataset, MEL samples also show lower cytolytic and M2 macrophage-associated markers compared with NPLAS/MES samples at both (**c**) pre-treatment and (**d**) on-treatment time points. Boxplots indicate the median (thick bar), first and third quartiles (lower and upper bounds of the box, respectively), lowest and highest data value within 1.5 times the interquartile range (lower and upper bounds of the whisker), and all individual data points are shown. Two-sided Kruskal-Wallis *p* values comparing across all sample classes are shown with post-hoc Dunn tests indicating pairwise comparisons. Response (binary) is indicated by color (cyan=responder, red=non-responder, gray=unknown) within each cluster group. Source data are provided as a Source Data file.
