# Supplementary material for: Multi-modal molecular programs regulate melanoma cell state
Source: Nat Commun. 2022 Jul 9;13:4000. doi: 10.1038/s41467-022-31510-1 (PMC9271073; doi:10.1038/s41467-022-31510-1)
Supplement: Supplementary file 3 — Description of Additional Supplementary Files [file 41467_2022_31510_MOESM3_ESM.pdf]

Description of Additional Supplementary Files

Supplementary Data 1: Cell line associated clinical data.

Supplementary Data 2: List of cluster-defining genes.

Supplementary Data 3: GO term and KEGG pathway enrichment of cluster-defining genes.

Supplementary Data 4: Random Forest MCS classifier model-retained genes.

Supplementary Data 5: Reverse-phase protein array data.

Supplementary Data 6: Multi-modal regulation summary for MEL and MES genes.

Supplementary Data 7: MEL gene regulatory influences – MDACC cell lines.

Supplementary Data 8: MEL gene regulatory influences – TCGA melanomas.

Supplementary Data 9: MES gene regulatory influences – MDACC cell lines.

Supplementary Data 10: MES gene regulatory influences – TCGA melanomas.
