# Supplementary material for: Multi-modal molecular programs regulate melanoma cell state
Source: Nat Commun. 2022 Jul 9;13:4000. doi: 10.1038/s41467-022-31510-1 (PMC9271073; doi:10.1038/s41467-022-31510-1)
Supplement: Supplementary file 10 — Supplementary Data 7 [file 41467_2022_31510_MOESM10_ESM.pdf]

Supplementary Data 7: MEL gene regulatory influences - MDACC cell lines.

PMEL

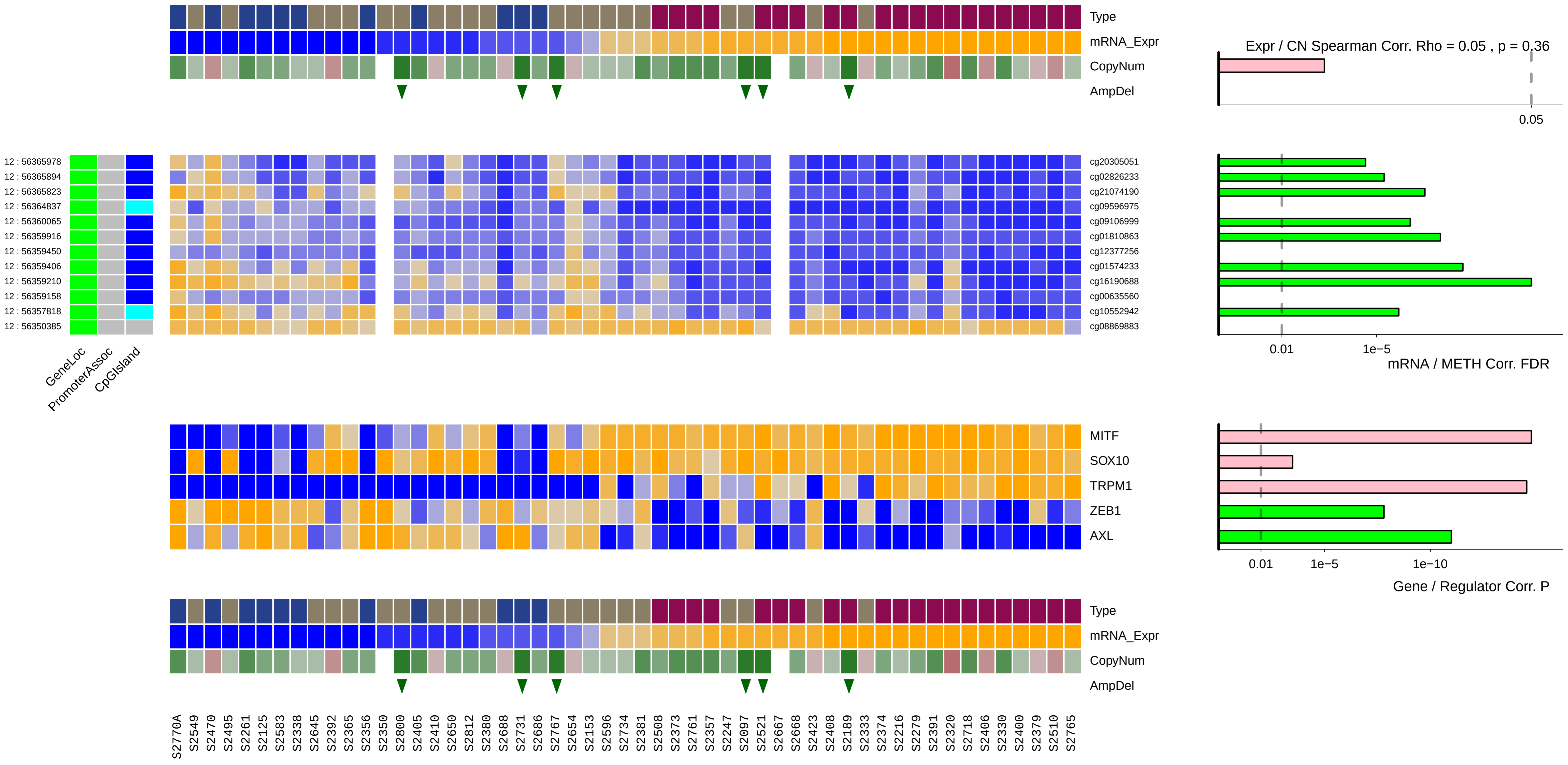
Type
mRNA_Expr
CopyNum
AmpDel
Expr / CN Spearman Corr. Rho = 0.05 , p = 0.36
0.05
12 : 56365978
12 : 56365894
12 : 56365823
12 : 56364837
12 : 56360065
12 : 56359916
12 : 56359450
12 : 56359406
12 : 56359210
12 : 56359158
12 : 56357818
12 : 56350385
cg20305051
cg02826233
cg21074190
cg09596975
cg09106999
cg01810863
cg12377256
cg01574233
cg16190688
cg00635560
cg10552942
cg08869883
0.01
1e−5
mRNA / METH Corr. FDR
GeneLoc
PromoterAssoc
CpGIsland
MITF
SOX10
TRPM1
ZEB1
AXL
0.01
1e−5
1e−10
Gene / Regulator Corr. P
Type
mRNA_Expr
CopyNum
AmpDel
S2770A S2549 S2470 S2495 S2261 S2125 S2583 S2338 S2645 S2392 S2365 S2356 S2350 S2800 S2405 S2410 S2650 S2812 S2380 S2688 S2731 S2686 S2767 S2654 S2153 S2596 S2734 S2381 S2508 S2373 S2761 S2357 S2247 S2097 S2521 S2667 S2668 S2423 S2408 S2189 S2333 S2374 S2216 S2279 S2391 S2320 S2718 S2406 S2330 S2400 S2379 S2510 S2765

MITF

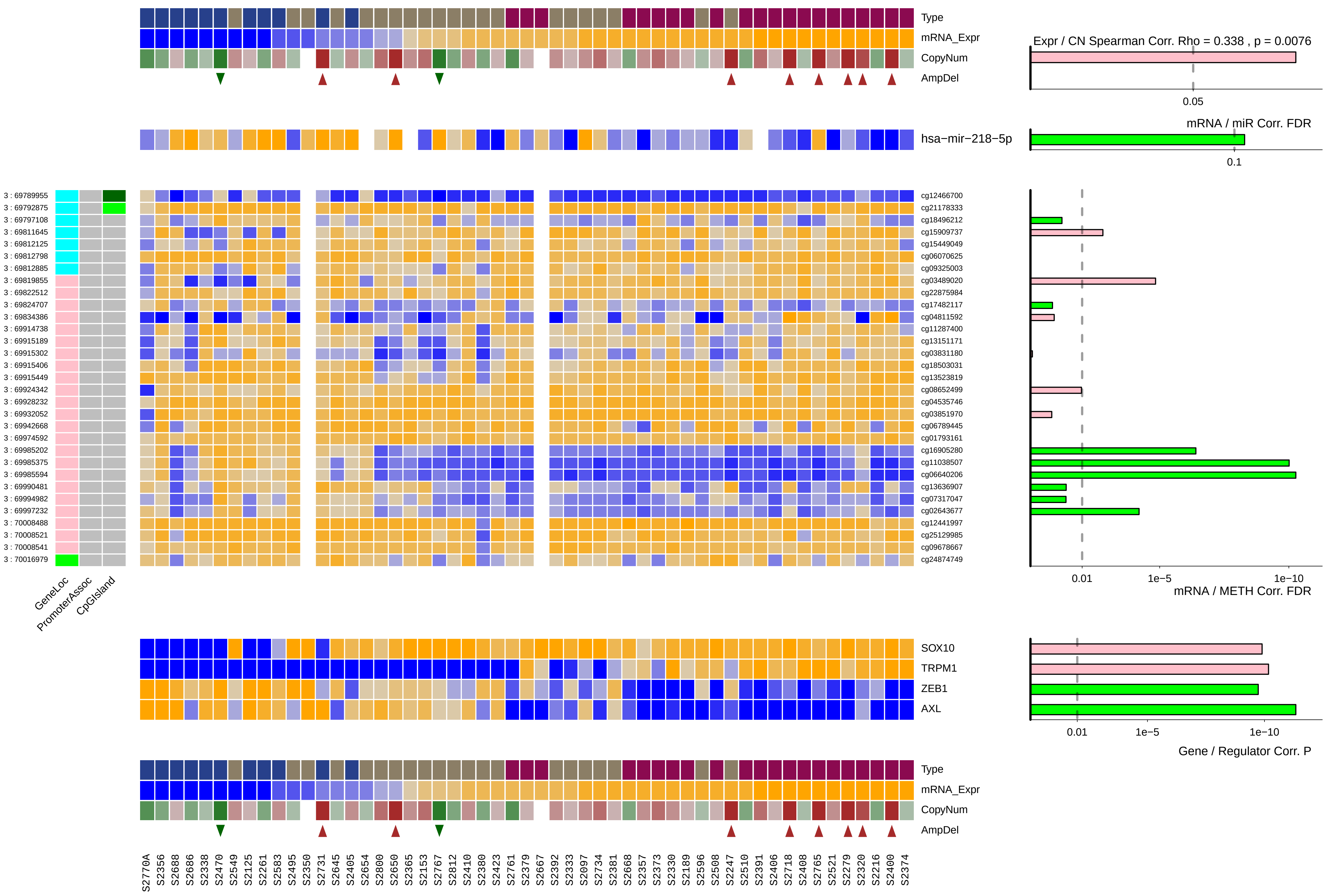
Type
mRNA_Expr
CopyNum
AmpDel
Expr / CN Spearman Corr. Rho = 0.338 , p = 0.0076
0.05
hsa−mir−218−5p
mRNA / miR Corr. FDR
0.1
3 : 69789955
3 : 69792875
3 : 69797108
3 : 69811645
3 : 69812125
3 : 69812798
3 : 69812885
3 : 69819855
3 : 69822512
3 : 69824707
3 : 69834386
3 : 69914738
3 : 69915189
3 : 69915302
3 : 69915406
3 : 69915449
3 : 69924342
3 : 69928232
3 : 69932052
3 : 69942668
3 : 69974592
3 : 69985202
3 : 69985375
3 : 69985594
3 : 69990481
3 : 69994982
3 : 69997232
3 : 70008488
3 : 70008521
3 : 70008541
3 : 70016979
cg12466700
cg21178333
cg18496212
cg15909737
cg15449049
cg06070625
cg09325003
cg03489020
cg22875984
cg17482117
cg04811592
cg11287400
cg13151171
cg03831180
cg18503031
cg13523819
cg08652499
cg04535746
cg03851970
cg06789445
cg01793161
cg16905280
cg11038507
cg06640206
cg13636907
cg07317047
cg02643677
cg12441997
cg25129985
cg09678667
cg24874749
GeneLoc
PromoterAssoc
CpGIsland
0.01
1e−5
1e−10
mRNA / METH Corr. FDR
SOX10
TRPM1
ZEB1
AXL
0.01
1e−5
1e−10
Gene / Regulator Corr. P
Type
mRNA_Expr
CopyNum
AmpDel
S2770A
S2356
S2688
S2686
S2338
S2470
S2549
S2125
S2261
S2583
S2495
S2350
S2731
S2645
S2405
S2654
S2800
S2650
S2365
S2153
S2767
S2812
S2410
S2380
S2423
S2761
S2379
S2667
S2392
S2333
S2097
S2734
S2381
S2668
S2357
S2373
S2330
S2189
S2596
S2508
S2247
S2510
S2391
S2406
S2718
S2408
S2765
S2521
S2279
S2320
S2216
S2400
S2374

TYR

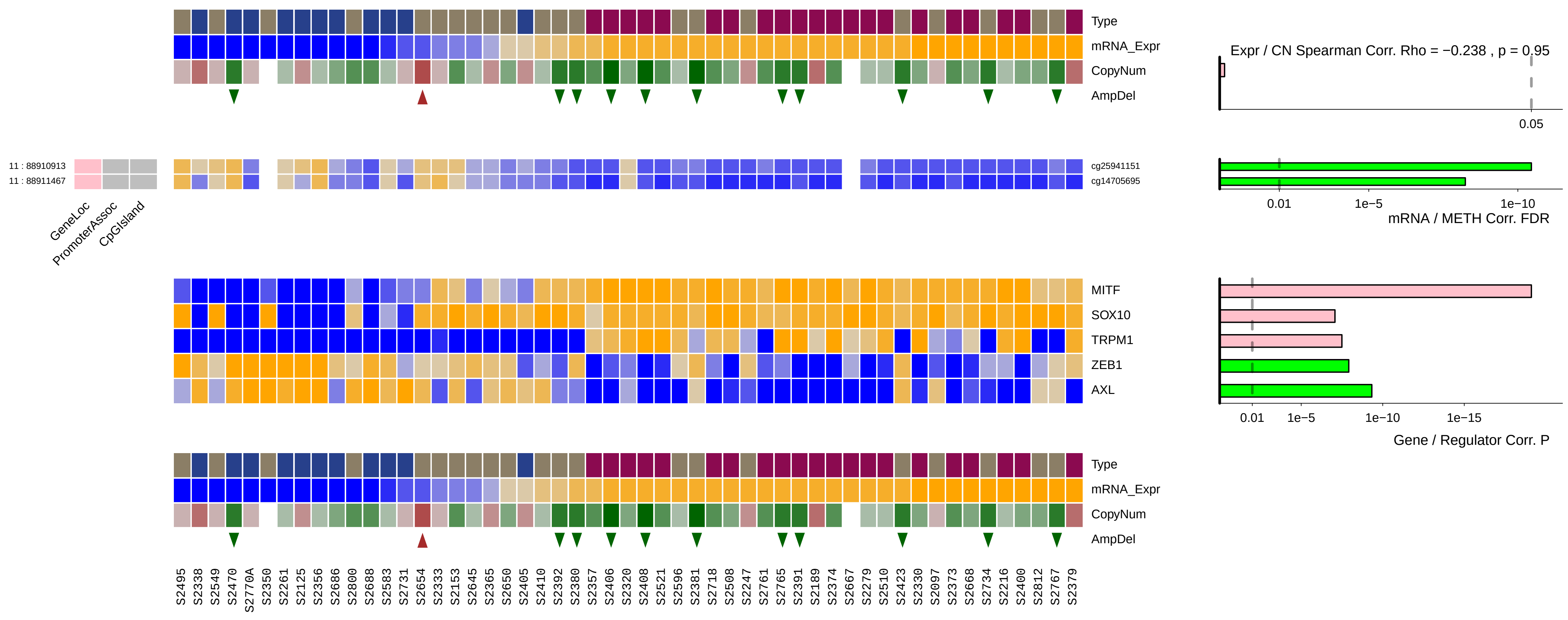

Type
mRNA_Expr
CopyNum
AmpDel
Expr / CN Spearman Corr. Rho = −0.238 , p = 0.95
0.05
11 : 88910913
11 : 88911467
GeneLoc
PromoterAssoc
CpGIsland
cg25941151
cg14705695
0.01
1e−5
1e−10
mRNA / METH Corr. FDR
MITF
SOX10
TRPM1
ZEB1
AXL
0.01
1e−5
1e−10
1e−15
Gene / Regulator Corr. P
Type
mRNA_Expr
CopyNum
AmpDel
S2495 S2338 S2549 S2470 S2770A S2350 S2261 S2125 S2356 S2686 S2800 S2688 S2583 S2731 S2654 S2333 S2153 S2645 S2365 S2650 S2405 S2410 S2392 S2380 S2357 S2406 S2320 S2408 S2521 S2596 S2381 S2718 S2508 S2247 S2761 S2765 S2391 S2189 S2374 S2667 S2279 S2510 S2423 S2330 S2097 S2373 S2668 S2734 S2216 S2400 S2812 S2767 S2379

MLANA

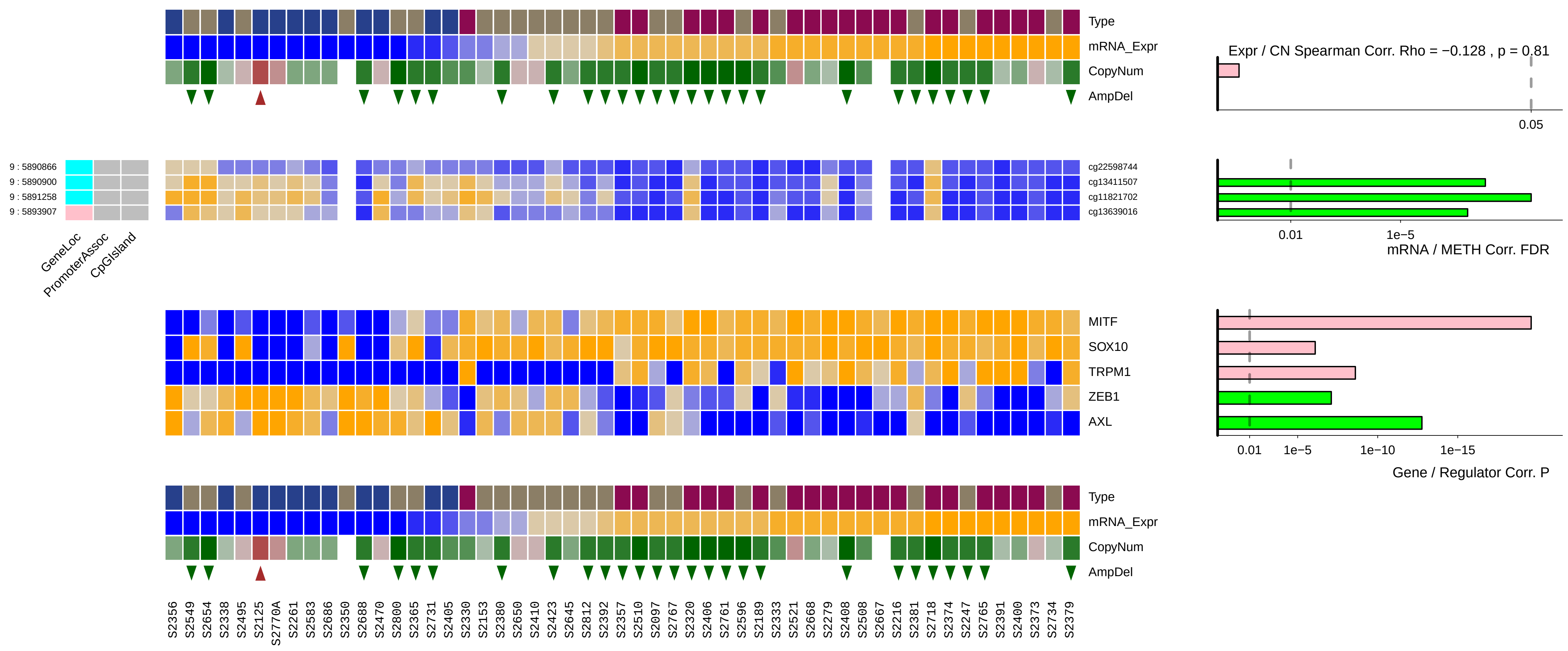
Type
mRNA_Expr
CopyNum
AmpDel
Expr / CN Spearman Corr. Rho = −0.128 , p = 0.81
0.05
9 : 5890866
9 : 5890900
9 : 5891258
9 : 5893907
GeneLoc
PromoterAssoc
CpGIsland
cg22598744
cg13411507
cg11821702
cg13639016
0.01
1e−5
mRNA / METH Corr. FDR
MITF
SOX10
TRPM1
ZEB1
AXL
0.01
1e−5
1e−10
1e−15
Gene / Regulator Corr. P
Type
mRNA_Expr
CopyNum
AmpDel
S2356 S2549 S2654 S2338 S2495 S2125 S2770A S2261 S2583 S2686 S2350 S2688 S2470 S2800 S2365 S2731 S2405 S2330 S2153 S2380 S2650 S2410 S2423 S2645 S2812 S2392 S2357 S2510 S2097 S2767 S2320 S2406 S2761 S2596 S2189 S2333 S2521 S2668 S2279 S2408 S2508 S2667 S2216 S2381 S2718 S2374 S2247 S2765 S2391 S2400 S2373 S2734 S2379

CDK2

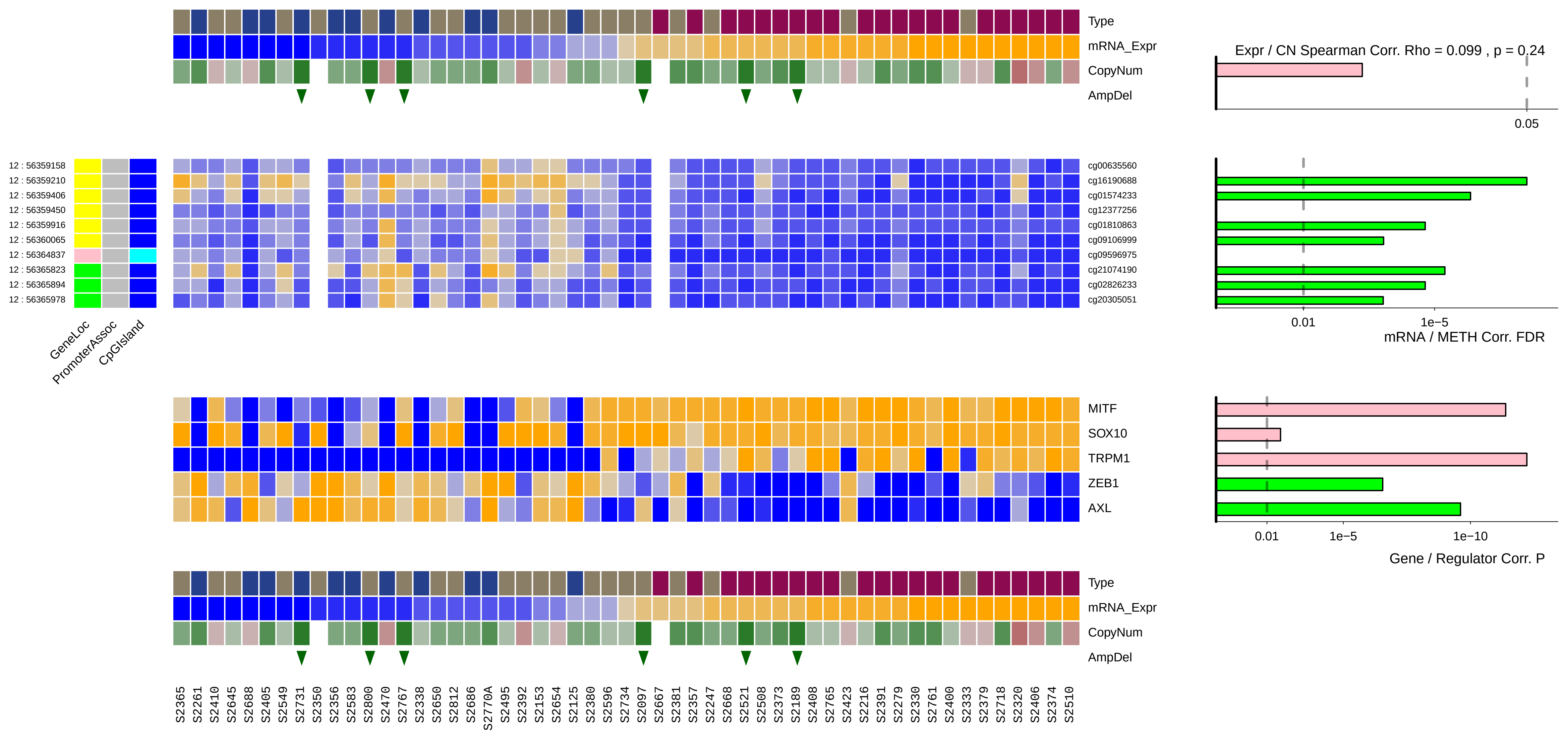

Type
mRNA_Expr
CopyNum
AmpDel
Expr / CN Spearman Corr. Rho = 0.099 , p = 0.24
0.05
12 : 56359158
12 : 56359210
12 : 56359406
12 : 56359450
12 : 56359916
12 : 56360065
12 : 56364837
12 : 56365823
12 : 56365894
12 : 56365978
GeneLoc
PromoterAssoc
CpGIsland
cg00635560
cg16190688
cg01574233
cg12377256
cg01810863
cg09106999
cg09596975
cg21074190
cg02826233
cg20305051
0.01
1e−5
mRNA / METH Corr. FDR
MITF
SOX10
TRPM1
ZEB1
AXL
0.01
1e−5
1e−10
Gene / Regulator Corr. P
Type
mRNA_Expr
CopyNum
AmpDel
S2365 S2261 S2410 S2645 S2688 S2405 S2549 S2731 S2350 S2356 S2583 S2800 S2470 S2767 S2338 S2650 S2812 S2686 S2770A S2495 S2392 S2153 S2654 S2125 S2380 S2596 S2734 S2097 S2667 S2381 S2357 S2247 S2668 S2521 S2508 S2373 S2189 S2408 S2765 S2423 S2216 S2391 S2279 S2330 S2761 S2400 S2333 S2379 S2718 S2320 S2406 S2374 S2510

TTYH2

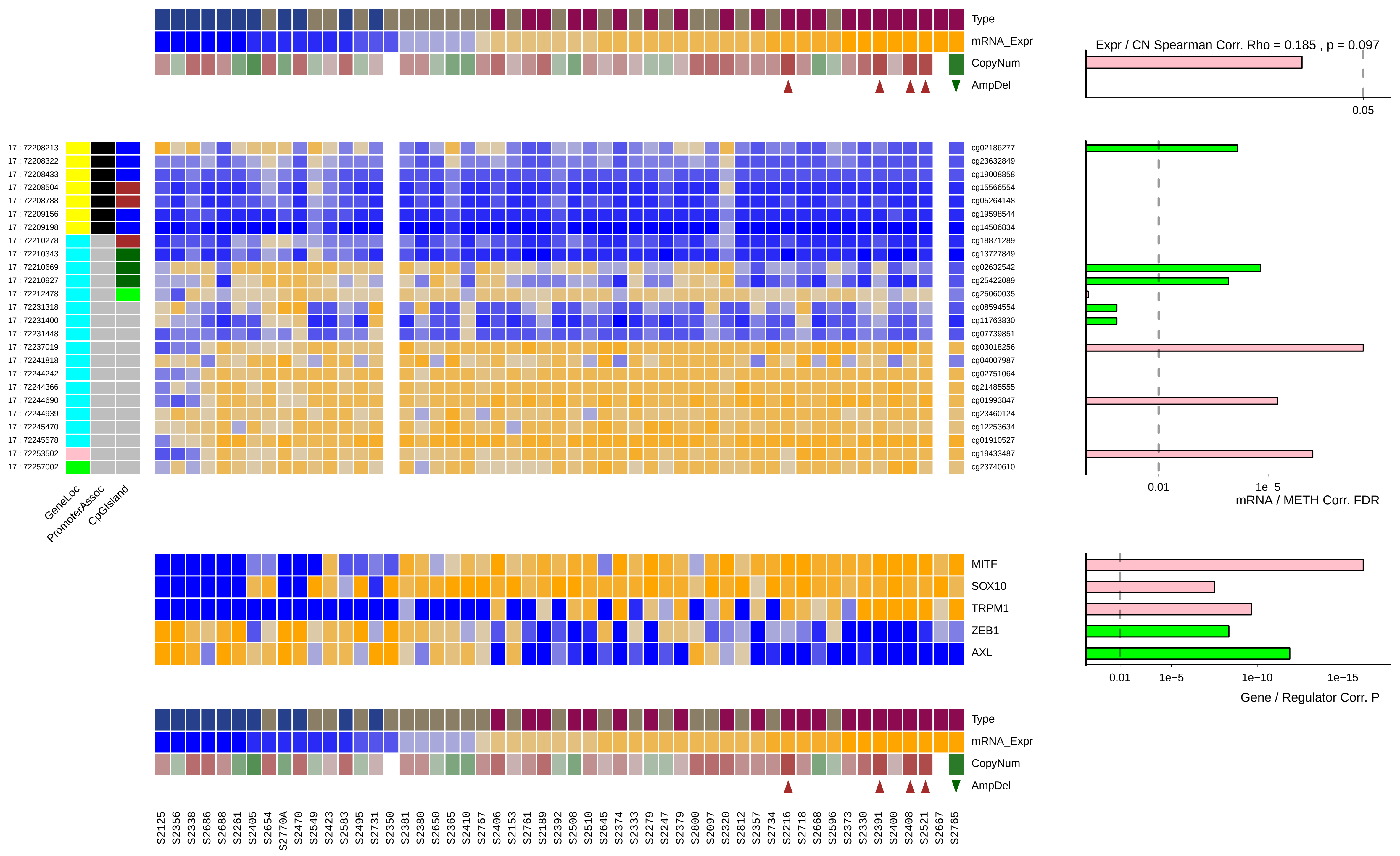

Type
mRNA_Expr
CopyNum
AmpDel
Expr / CN Spearman Corr. Rho = 0.185 , p = 0.097
0.05
17 : 72208213
17 : 72208322
17 : 72208433
17 : 72208504
17 : 72208788
17 : 72209156
17 : 72209198
17 : 72210278
17 : 72210343
17 : 72210669
17 : 72210927
17 : 72212478
17 : 72231318
17 : 72231400
17 : 72231448
17 : 72237019
17 : 72241818
17 : 72244242
17 : 72244366
17 : 72244690
17 : 72244939
17 : 72245470
17 : 72245578
17 : 72253502
17 : 72257002
cg02186277
cg23632849
cg19008858
cg15566554
cg05264148
cg19598544
cg14506834
cg18871289
cg13727849
cg02632542
cg25422089
cg25060035
cg08594554
cg11763830
cg07739851
cg03018256
cg04007987
cg02751064
cg21485555
cg01993847
cg23460124
cg12253634
cg01910527
cg19433487
cg23740610
0.01
1e−5
mRNA / METH Corr. FDR
GeneLoc
PromoterAssoc
CpGIsland
MITF
SOX10
TRPM1
ZEB1
AXL
0.01
1e−5
1e−10
1e−15
Gene / Regulator Corr. P
Type
mRNA_Expr
CopyNum
AmpDel
S2125
S2356
S2338
S2686
S2688
S2261
S2405
S2654
S2770A
S2470
S2549
S2423
S2583
S2495
S2731
S2350
S2381
S2380
S2650
S2365
S2410
S2767
S2406
S2153
S2761
S2189
S2392
S2508
S2510
S2645
S2374
S2333
S2279
S2247
S2379
S2800
S2097
S2320
S2812
S2357
S2734
S2216
S2718
S2668
S2596
S2373
S2330
S2391
S2400
S2408
S2521
S2667
S2765

SLC24A5

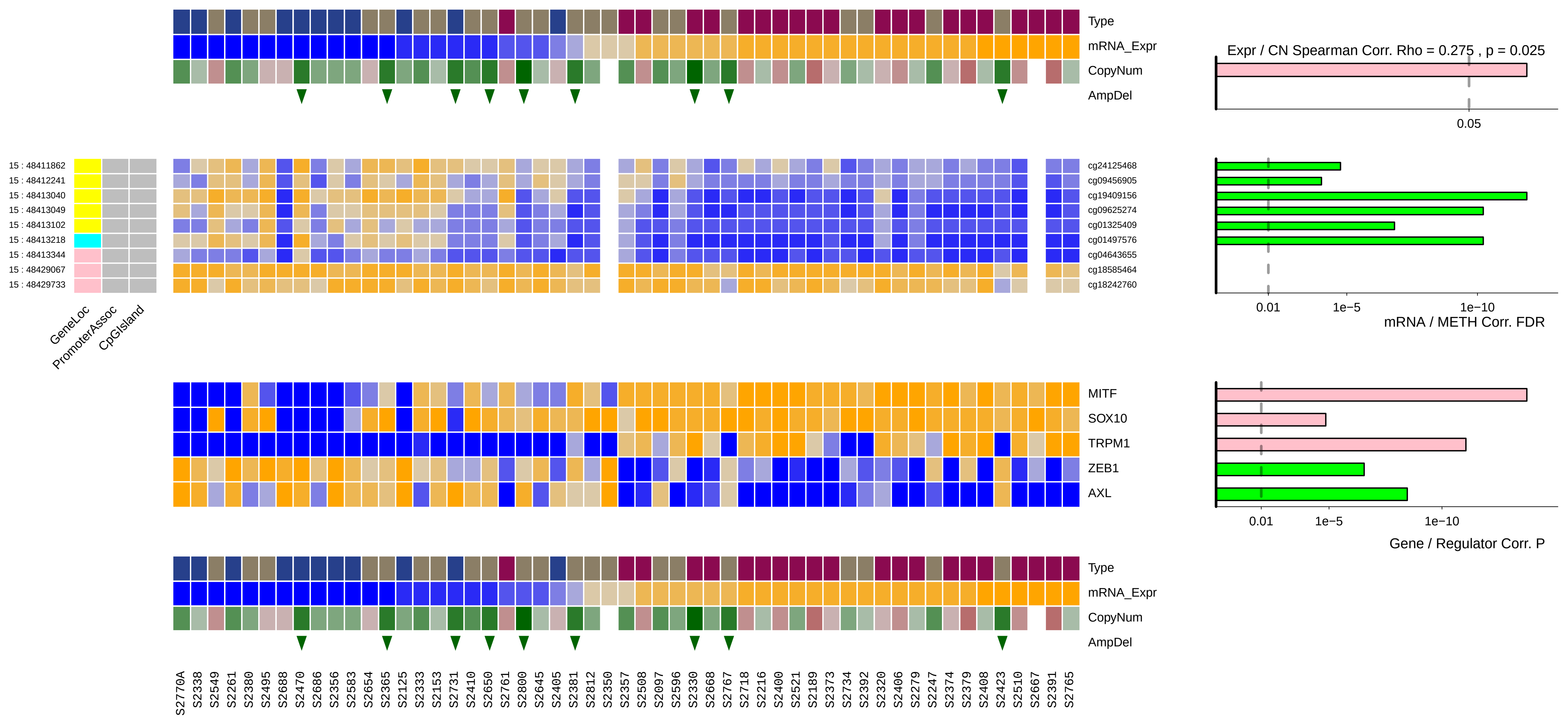
Type
mRNA_Expr
CopyNum
AmpDel
Expr / CN Spearman Corr. Rho = 0.275 , p = 0.025
0.05
15 : 48411862
15 : 48412241
15 : 48413040
15 : 48413049
15 : 48413102
15 : 48413218
15 : 48413344
15 : 48429067
15 : 48429733
GeneLoc
PromoterAssoc
CpGIsland
cg24125468
cg09456905
cg19409156
cg09625274
cg01325409
cg01497576
cg04643655
cg18585464
cg18242760
0.01
1e−5
1e−10
mRNA / METH Corr. FDR
MITF
SOX10
TRPM1
ZEB1
AXL
Gene / Regulator Corr. P
S2770A S2338 S2549 S2261 S2380 S2495 S2688 S2470 S2686 S2356 S2583 S2654 S2365 S2125 S2333 S2153 S2731 S2410 S2650 S2761 S2800 S2645 S2405 S2381 S2812 S2350 S2357 S2508 S2097 S2596 S2330 S2668 S2767 S2718 S2216 S2400 S2521 S2189 S2373 S2734 S2392 S2320 S2406 S2279 S2247 S2374 S2379 S2408 S2423 S2510 S2667 S2391 S2765

RRAGD

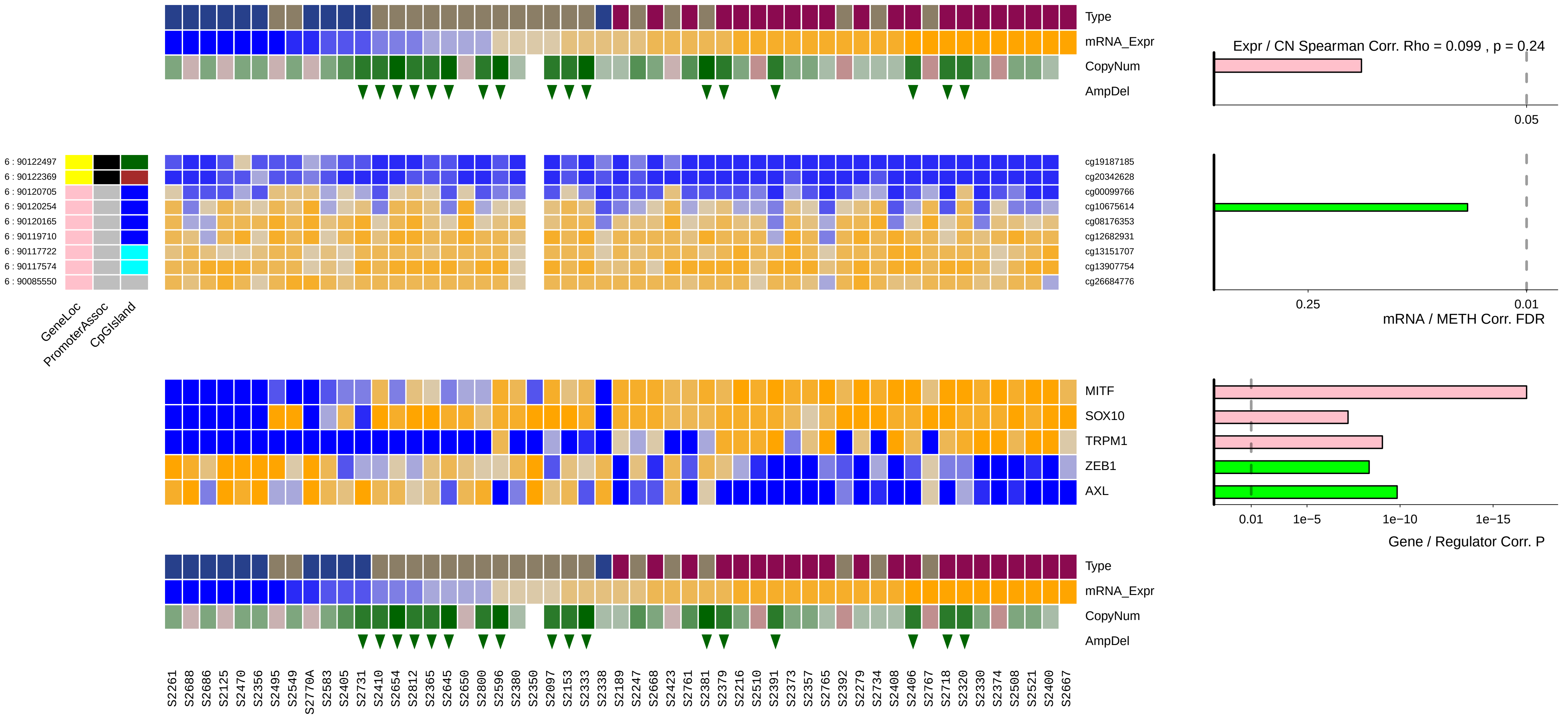
Type
mRNA_Expr
CopyNum
AmpDel
Expr / CN Spearman Corr. Rho = 0.099 , p = 0.24
0.05
6 : 90122497
6 : 90122369
6 : 90120705
6 : 90120254
6 : 90120165
6 : 90119710
6 : 90117722
6 : 90117574
6 : 90085550
GeneLoc
PromoterAssoc
CpGIsland
cg19187185
cg20342628
cg00099766
cg10675614
cg08176353
cg12682931
cg13151707
cg13907754
cg26684776
0.25
0.01
mRNA / METH Corr. FDR
MITF
SOX10
TRPM1
ZEB1
AXL
0.01
1e−5
1e−10
1e−15
Gene / Regulator Corr. P
S2261 S2688 S2686 S2125 S2470 S2356 S2495 S2549 S2770A S2583 S2405 S2731 S2410 S2654 S2812 S2365 S2645 S2650 S2800 S2596 S2380 S2350 S2097 S2153 S2333 S2338 S2189 S2247 S2668 S2423 S2761 S2381 S2379 S2216 S2510 S2391 S2373 S2357 S2765 S2392 S2279 S2734 S2408 S2406 S2767 S2718 S2320 S2330 S2374 S2508 S2521 S2400 S2667

TRIM63

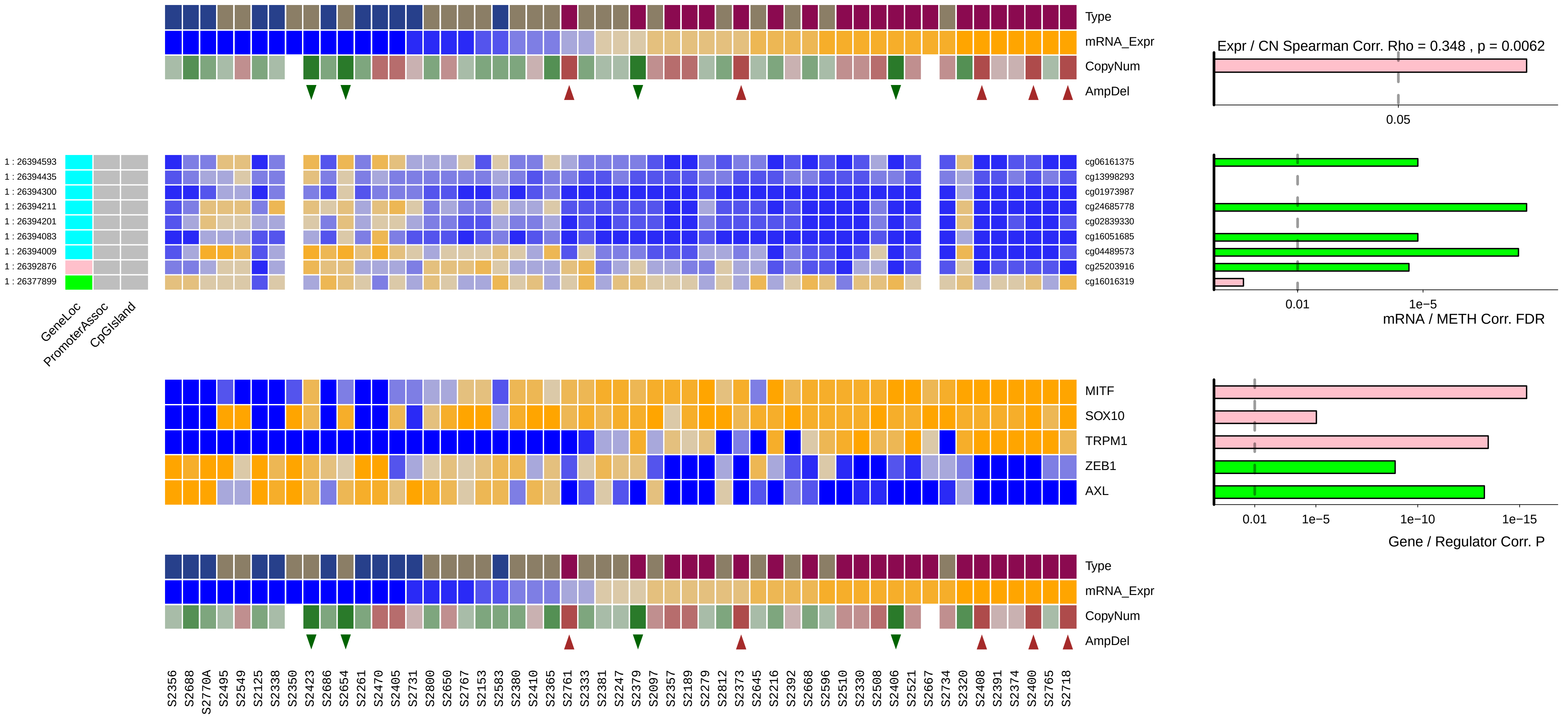

Type
mRNA_Expr
CopyNum
AmpDel
Expr / CN Spearman Corr. Rho = 0.348 , p = 0.0062
0.05
1 : 26394593
1 : 26394435
1 : 26394300
1 : 26394211
1 : 26394201
1 : 26394083
1 : 26394009
1 : 26392876
1 : 26377899
GeneLoc
PromoterAssoc
CpGIsland
cg06161375
cg13998293
cg01973987
cg24685778
cg02839330
cg16051685
cg04489573
cg25203916
cg16016319
0.01
1e−5
mRNA / METH Corr. FDR
MITF
SOX10
TRPM1
ZEB1
AXL
0.01
1e−5
1e−10
1e−15
Gene / Regulator Corr. P
Type
mRNA_Expr
CopyNum
AmpDel
S2356 S2688 S2770A S2495 S2549 S2125 S2338 S2350 S2423 S2686 S2654 S2261 S2470 S2405 S2731 S2800 S2650 S2767 S2153 S2583 S2380 S2410 S2365 S2761 S2333 S2381 S2247 S2379 S2097 S2357 S2189 S2279 S2812 S2373 S2645 S2216 S2392 S2668 S2596 S2510 S2330 S2508 S2406 S2521 S2667 S2734 S2320 S2408 S2391 S2374 S2400 S2765 S2718

GPM6B

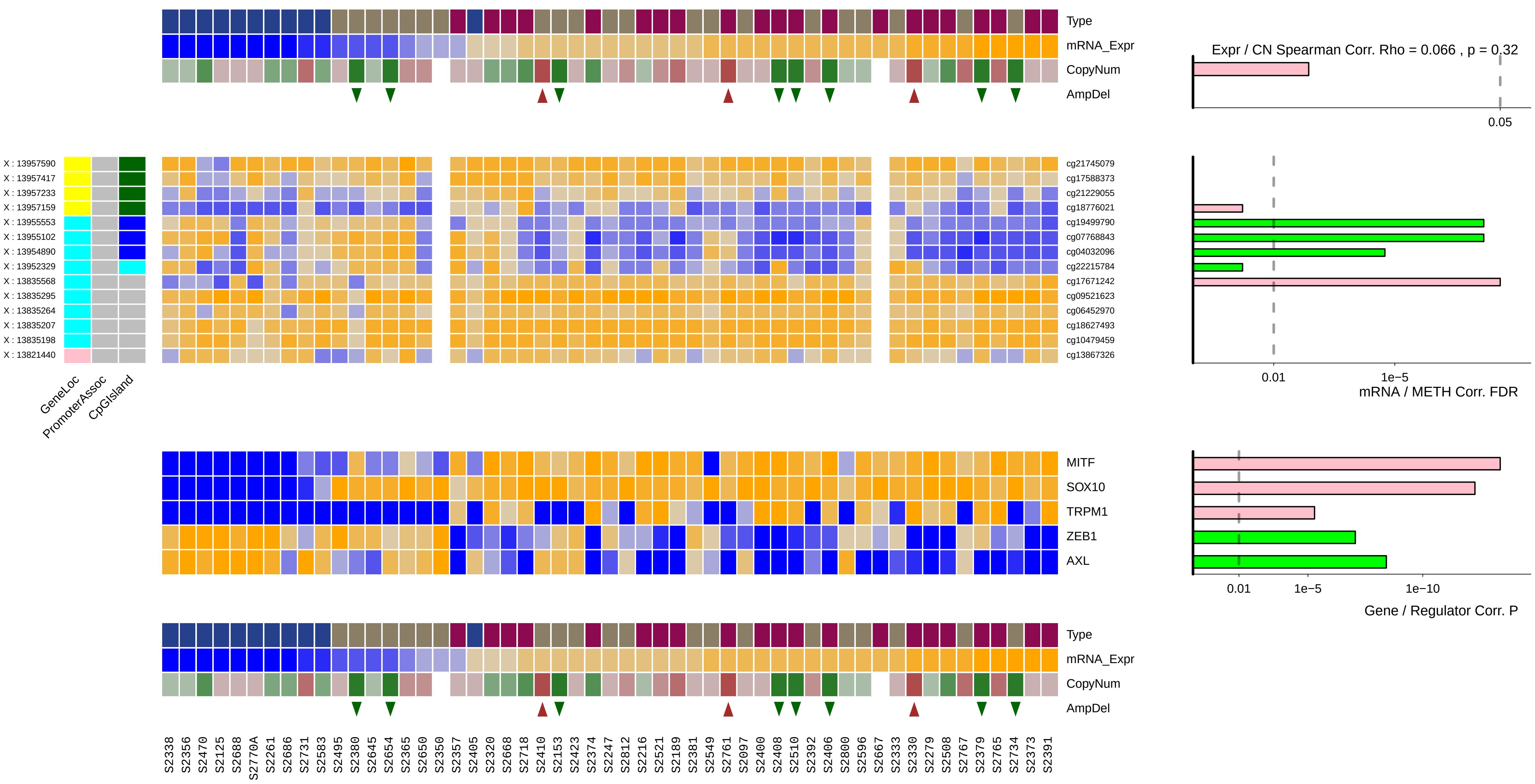

Type
mRNA_Expr
CopyNum
AmpDel
Expr / CN Spearman Corr. Rho = 0.066 , p = 0.32
0.05
X : 13957590
X : 13957417
X : 13957233
X : 13957159
X : 13955553
X : 13955102
X : 13954890
X : 13952329
X : 13835568
X : 13835295
X : 13835264
X : 13835207
X : 13835198
X : 13821440
GeneLoc
PromoterAssoc
CpGIsland
cg21745079
cg17588373
cg21229055
cg18776021
cg19499790
cg07768843
cg04032096
cg22215784
cg17671242
cg09521623
cg06452970
cg18627493
cg10479459
cg13867326
0.01
1e−5
mRNA / METH Corr. FDR
MITF
SOX10
TRPM1
ZEB1
AXL
0.01
1e−5
1e−10
Gene / Regulator Corr. P
Type
mRNA_Expr
CopyNum
AmpDel
S2338 S2356 S2470 S2125 S2688 S2770A S2261 S2686 S2731 S2583 S2495 S2380 S2645 S2654 S2365 S2650 S2350 S2357 S2405 S2320 S2668 S2718 S2410 S2153 S2423 S2374 S2247 S2812 S2216 S2521 S2189 S2381 S2549 S2761 S2097 S2400 S2408 S2510 S2392 S2406 S2800 S2596 S2667 S2333 S2330 S2279 S2508 S2767 S2379 S2765 S2734 S2373 S2391

SOX10

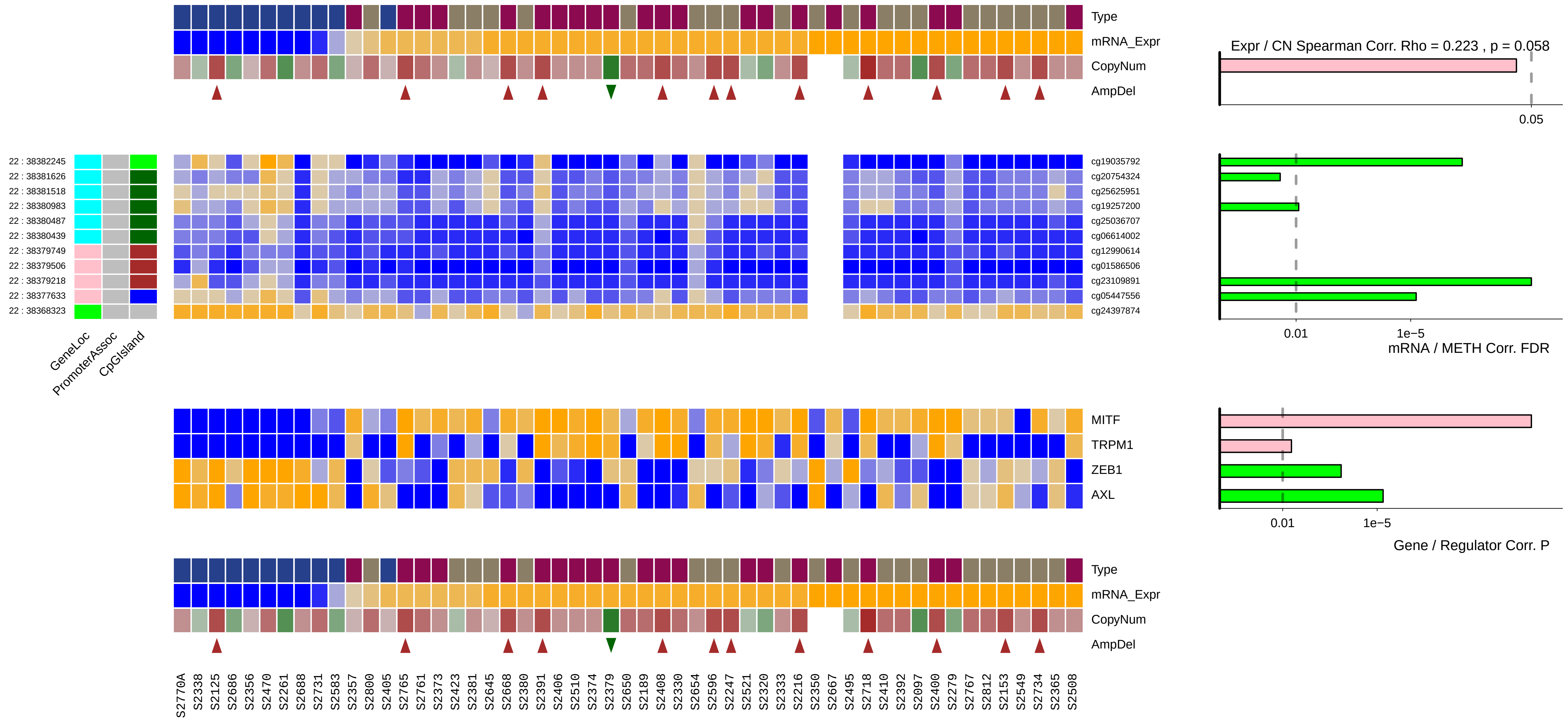
Type
mRNA_Expr
CopyNum
AmpDel
Expr / CN Spearman Corr. Rho = 0.223 , p = 0.058
0.05
GeneLoc
PromoterAssoc
CpGIsland
22 : 38382245
22 : 38381626
22 : 38381518
22 : 38380983
22 : 38380487
22 : 38380439
22 : 38379749
22 : 38379506
22 : 38379218
22 : 38377633
22 : 38368323
cg19035792
cg20754324
cg25625951
cg19257200
cg25036707
cg06614002
cg12990614
cg01586506
cg23109891
cg05447556
cg24397874
0.01
1e−5
mRNA / METH Corr. FDR
MITF
TRPM1
ZEB1
AXL
Gene / Regulator Corr. P
S2770A S2338 S2125 S2686 S2356 S2470 S2261 S2688 S2731 S2583 S2357 S2800 S2405 S2765 S2761 S2373 S2423 S2381 S2645 S2668 S2380 S2391 S2406 S2510 S2374 S2379 S2650 S2189 S2408 S2330 S2654 S2596 S2247 S2521 S2320 S2333 S2216 S2350 S2667 S2495 S2718 S2410 S2392 S2097 S2400 S2279 S2767 S2812 S2153 S2549 S2734 S2365 S2508

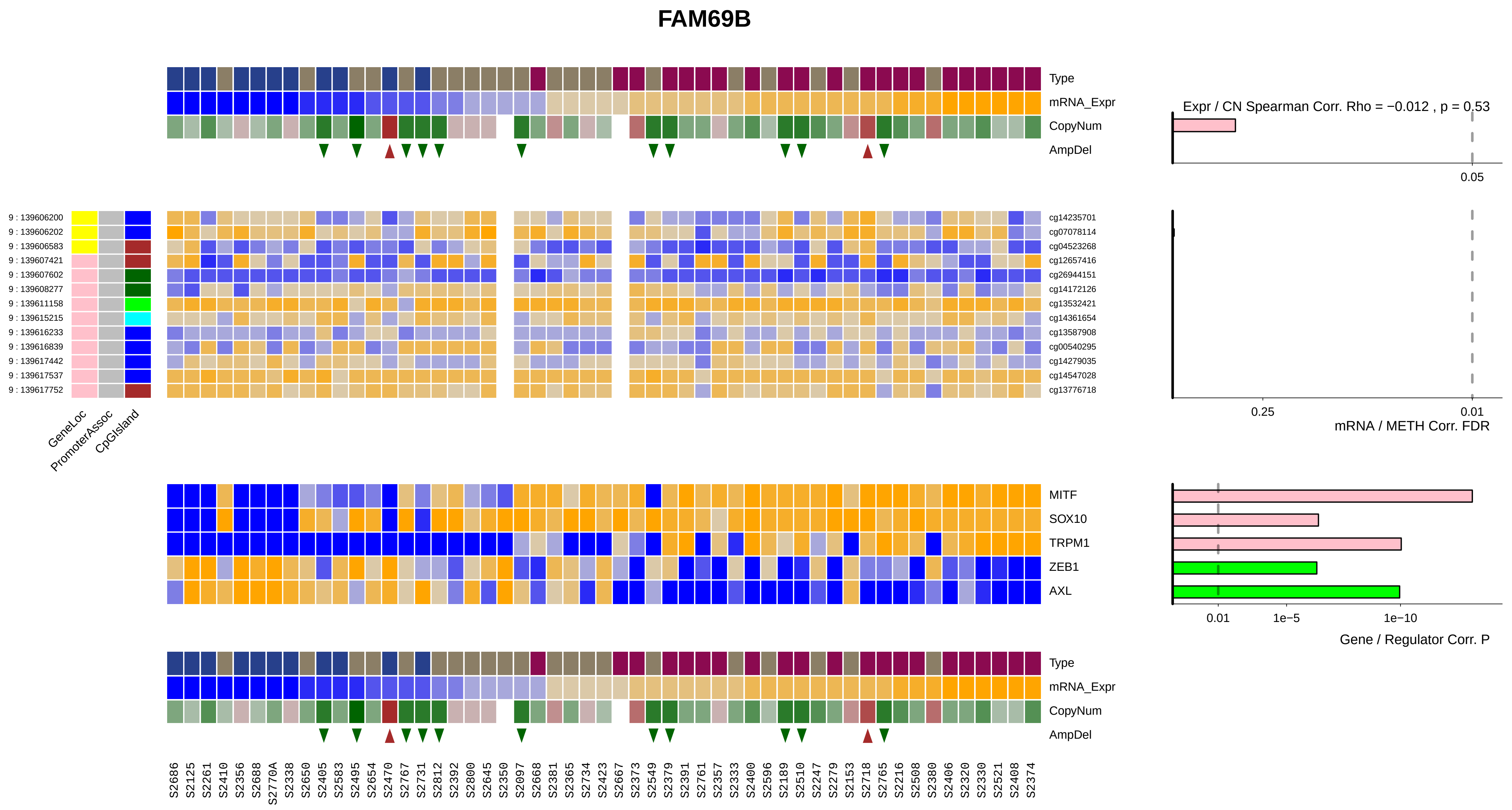
FAM69B
Type
mRNA_Expr
CopyNum
AmpDel
Expr / CN Spearman Corr. Rho = −0.012 , p = 0.53
0.05
cg14235701
cg07078114
cg04523268
cg12657416
cg26944151
cg14172126
cg13532421
cg14361654
cg13587908
cg00540295
cg14279035
cg14547028
cg13776718
9 : 139606200
9 : 139606202
9 : 139606583
9 : 139607421
9 : 139607602
9 : 139608277
9 : 139611158
9 : 139615215
9 : 139616233
9 : 139616839
9 : 139617442
9 : 139617537
9 : 139617752
GeneLoc
PromoterAssoc
CpGIsland
0.25
0.01
mRNA / METH Corr. FDR
MITF
SOX10
TRPM1
ZEB1
AXL
0.01
1e−5
1e−10
Gene / Regulator Corr. P
S2686 S2125 S2261 S2410 S2356 S2688 S2770A S2338 S2650 S2405 S2583 S2495 S2654 S2470 S2767 S2731 S2812 S2392 S2800 S2645 S2350 S2097 S2668 S2381 S2365 S2734 S2423 S2667 S2373 S2549 S2379 S2391 S2761 S2357 S2333 S2400 S2596 S2189 S2510 S2247 S2279 S2153 S2718 S2765 S2216 S2508 S2380 S2406 S2320 S2330 S2521 S2408 S2374

TBC1D16

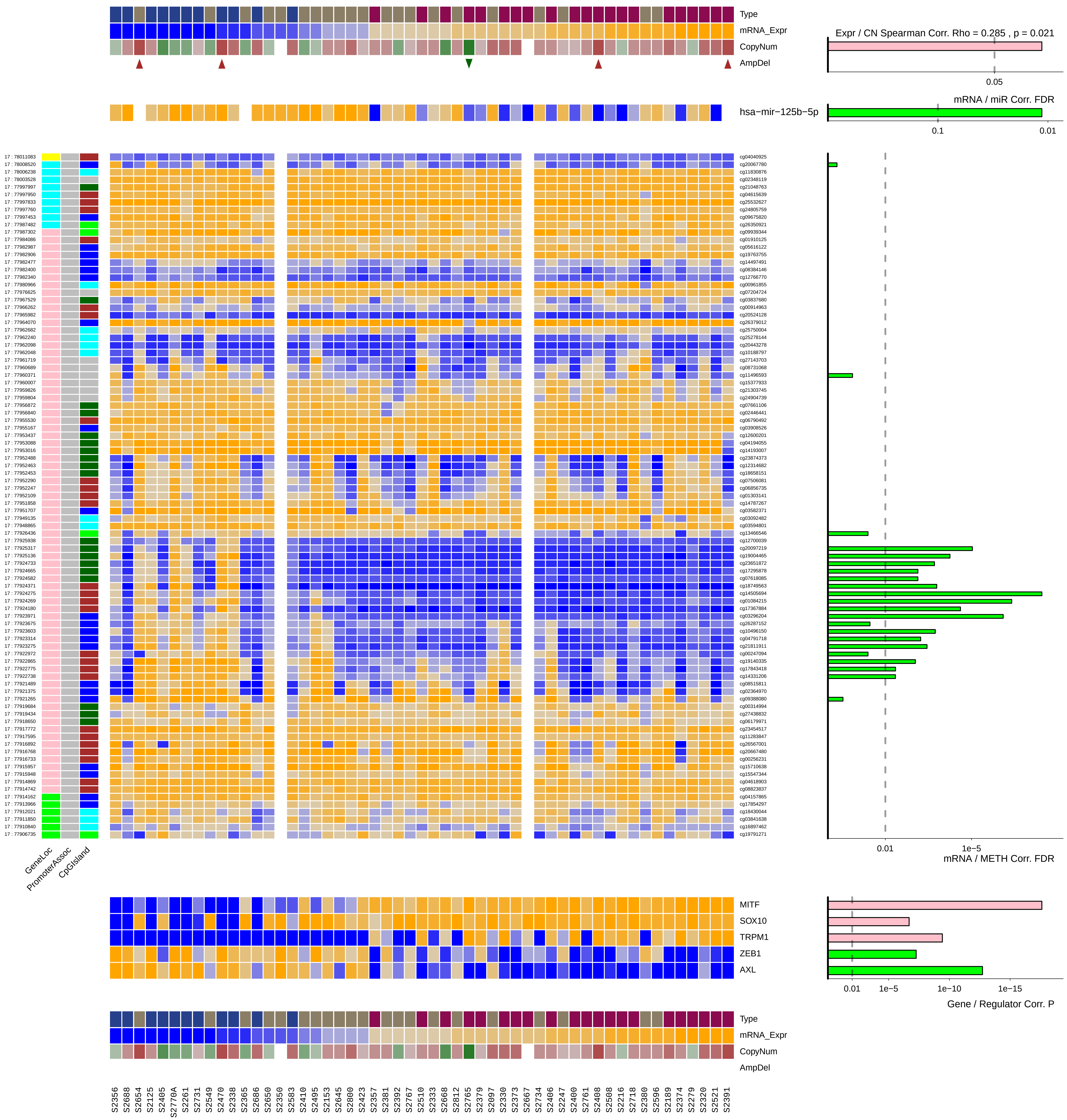
Type
mRNA_Expr
CopyNum
AmpDel
Expr / CN Spearman Corr. Rho = 0.285 , p = 0.021
0.05
hsa−mir−125b−5p
mRNA / miR Corr. FDR
0.1
0.01
GeneLoc
PromoterAssoc
CpGIsland
0.01
1e−5
mRNA / METH Corr. FDR
MITF
SOX10
TRPM1
ZEB1
AXL
0.01
1e−5
1e−10
1e−15
Gene / Regulator Corr. P
Type
mRNA_Expr
CopyNum
AmpDel
S2356 S2688 S2654 S2125 S2405 S2770A S2261 S2731 S2549 S2470 S2338 S2365 S2686 S2650 S2350 S2583 S2410 S2495 S2153 S2645 S2800 S2423 S2357 S2381 S2392 S2767 S2510 S2333 S2668 S2812 S2765 S2379 S2097 S2330 S2373 S2667 S2734 S2406 S2247 S2400 S2761 S2408 S2508 S2216 S2718 S2380 S2596 S2189 S2374 S2279 S2320 S2521 S2391

RENBP

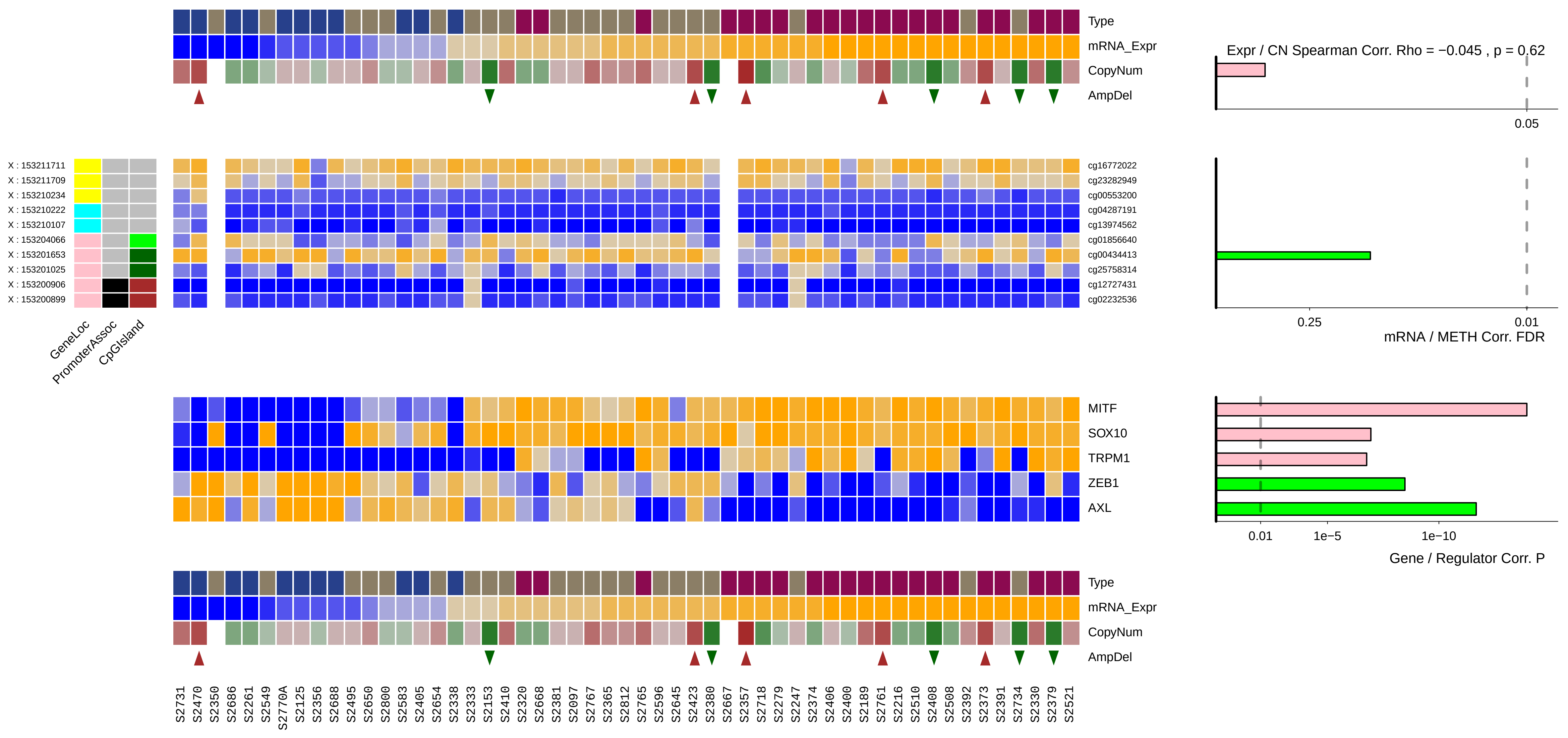

Type
mRNA_Expr
CopyNum
AmpDel
Expr / CN Spearman Corr. Rho = −0.045 , p = 0.62
0.05
X : 153211711
X : 153211709
X : 153210234
X : 153210222
X : 153210107
X : 153204066
X : 153201653
X : 153201025
X : 153200906
X : 153200899
GeneLoc
PromoterAssoc
CpGIsland
cg16772022
cg23282949
cg00553200
cg04287191
cg13974562
cg01856640
cg00434413
cg25758314
cg12727431
cg02232536
0.25
0.01
mRNA / METH Corr. FDR
MITF
SOX10
TRPM1
ZEB1
AXL
0.01
1e−5
1e−10
Gene / Regulator Corr. P
S2731 S2470 S2350 S2686 S2261 S2549 S2770A S2125 S2356 S2688 S2495 S2650 S2800 S2583 S2405 S2654 S2338 S2333 S2153 S2410 S2320 S2668 S2381 S2097 S2767 S2365 S2812 S2765 S2596 S2645 S2423 S2380 S2667 S2357 S2718 S2279 S2247 S2374 S2406 S2400 S2189 S2761 S2216 S2510 S2408 S2508 S2392 S2373 S2391 S2734 S2330 S2379 S2521

NR4A1

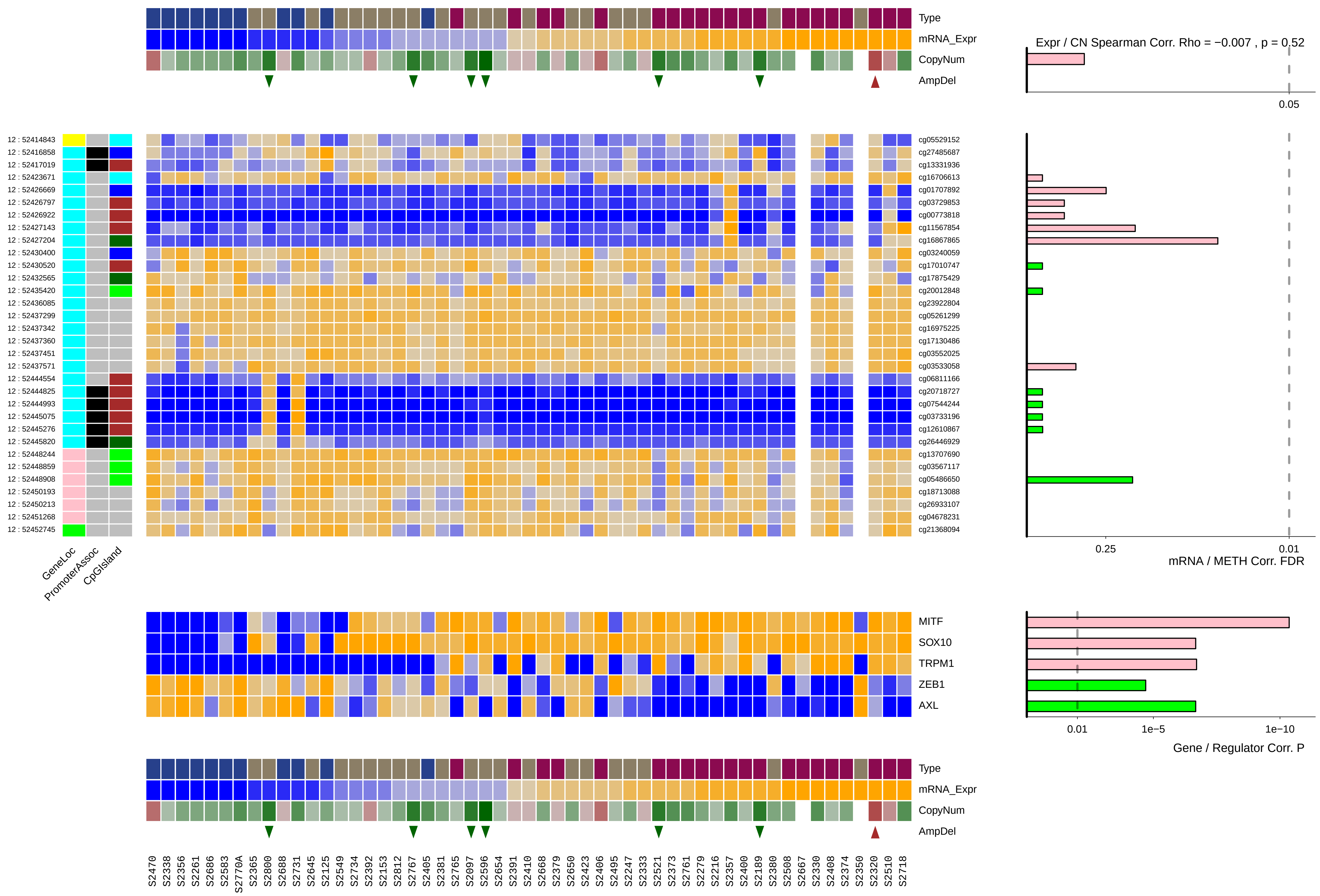
Type
mRNA_Expr
CopyNum
AmpDel
Expr / CN Spearman Corr. Rho = −0.007 , p = 0.52
0.05
12 : 52414843
12 : 52416858
12 : 52417019
12 : 52423671
12 : 52426669
12 : 52426797
12 : 52426922
12 : 52427143
12 : 52427204
12 : 52430400
12 : 52430520
12 : 52432565
12 : 52435420
12 : 52436085
12 : 52437299
12 : 52437342
12 : 52437360
12 : 52437451
12 : 52437571
12 : 52444554
12 : 52444825
12 : 52444993
12 : 52445075
12 : 52445276
12 : 52445820
12 : 52448244
12 : 52448859
12 : 52448908
12 : 52450193
12 : 52450213
12 : 52451268
12 : 52452745
cg05529152
cg27485687
cg13331936
cg16706613
cg01707892
cg03729853
cg00773818
cg11567854
cg16867865
cg03240059
cg17010747
cg17875429
cg20012848
cg23922804
cg05261299
cg16975225
cg17130486
cg03552025
cg03533058
cg06811166
cg20718727
cg07544244
cg03733196
cg12610867
cg26446929
cg13707690
cg03567117
cg05486650
cg18713088
cg26933107
cg04678231
cg21368094
GeneLoc
PromoterAssoc
CpGIsland
0.25
0.01
mRNA / METH Corr. FDR
MITF
SOX10
TRPM1
ZEB1
AXL
0.01
1e−5
1e−10
Gene / Regulator Corr. P
Type
mRNA_Expr
CopyNum
AmpDel
S2470 S2338 S2356 S2261 S2686 S2583 S2770A S2365 S2800 S2688 S2731 S2645 S2125 S2549 S2734 S2392 S2153 S2812 S2767 S2405 S2381 S2765 S2097 S2596 S2654 S2391 S2410 S2668 S2379 S2650 S2423 S2406 S2495 S2247 S2333 S2521 S2373 S2761 S2279 S2216 S2357 S2400 S2189 S2380 S2508 S2667 S2330 S2408 S2374 S2350 S2320 S2510 S2718

IGSF11

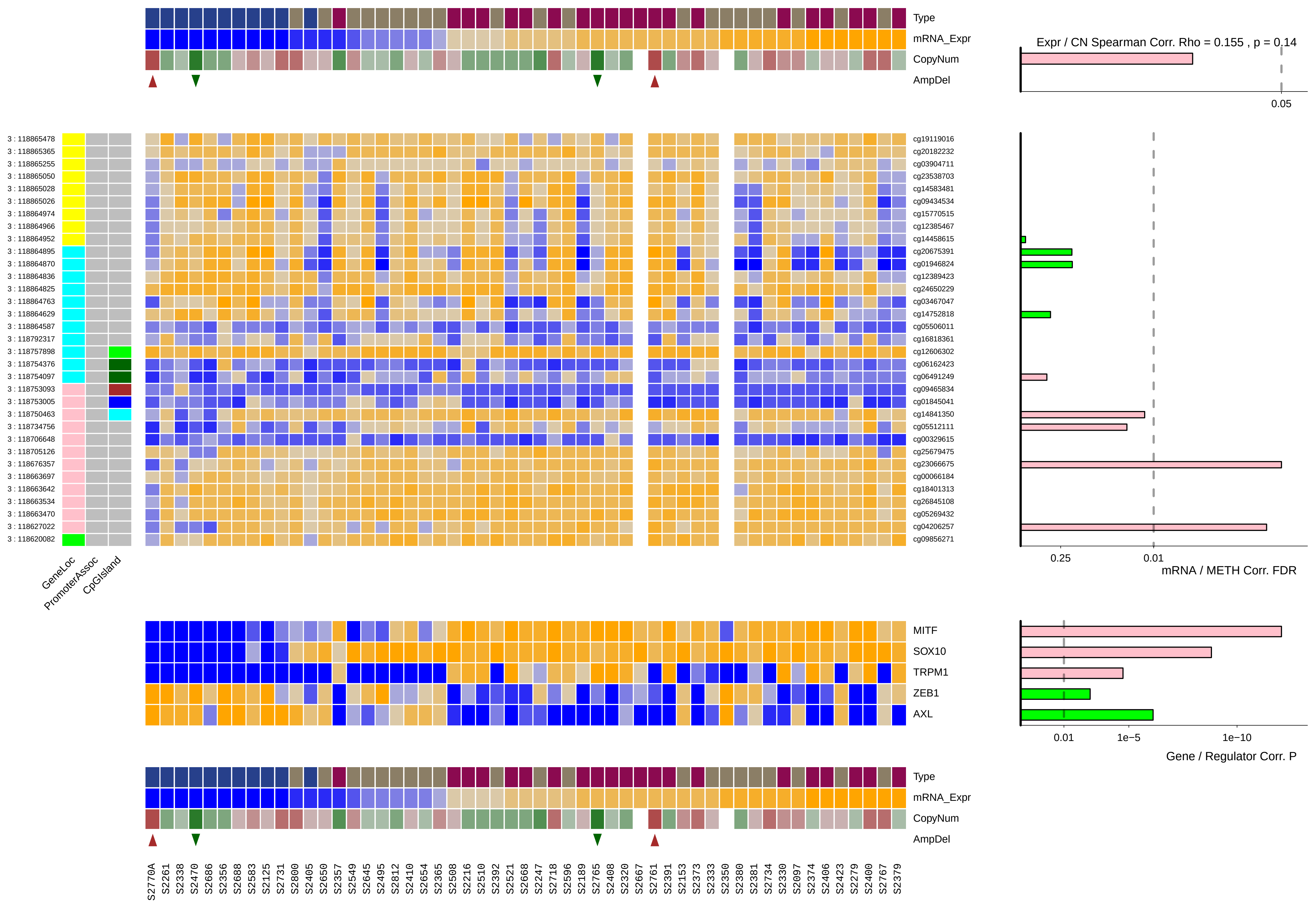

EDNRB

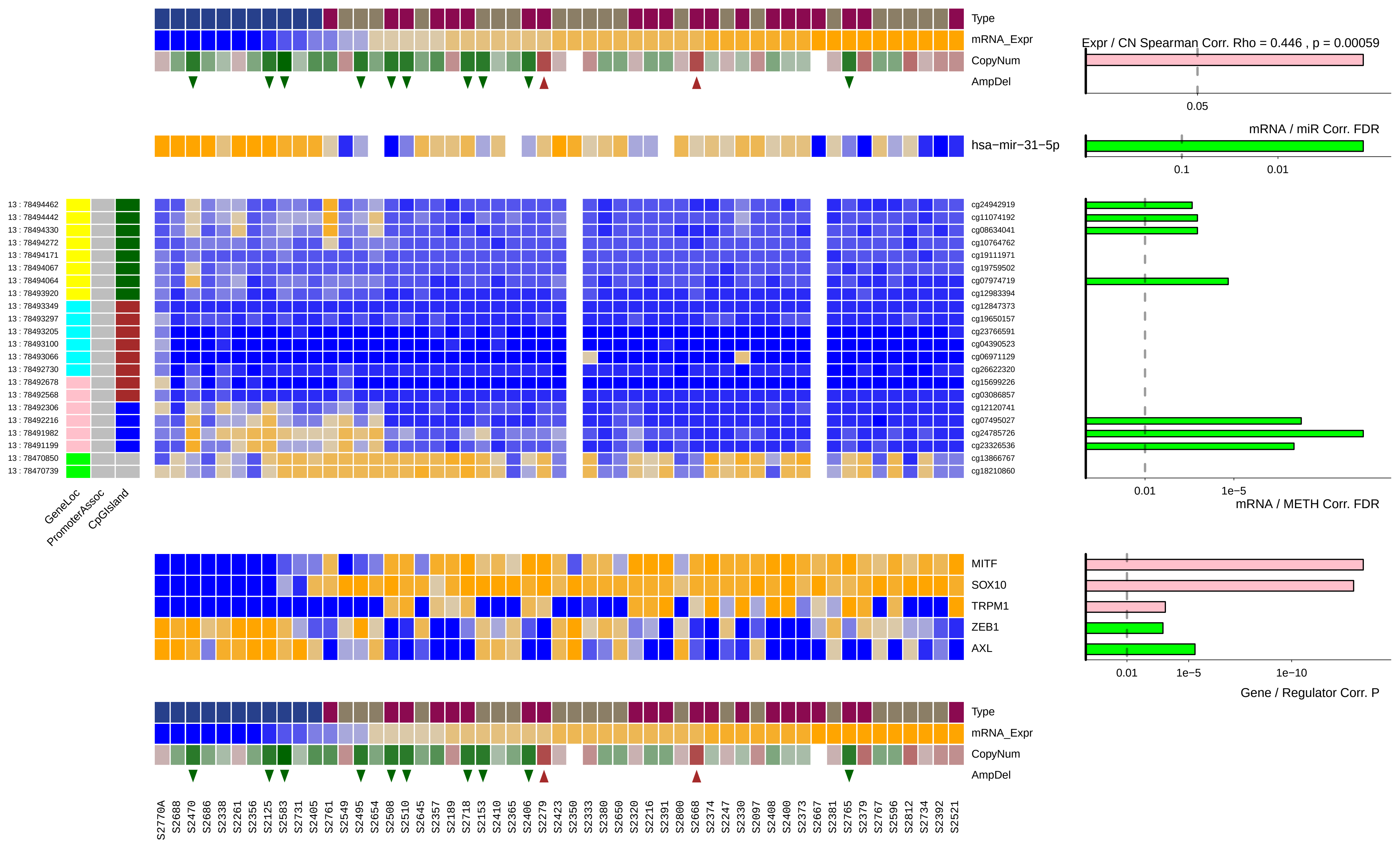

Type
mRNA_Expr
CopyNum
AmpDel
Expr / CN Spearman Corr. Rho = 0.446 , p = 0.00059
0.05
mRNA / miR Corr. FDR
hsa−mir−31−5p
0.1
0.01
13 : 78494462
13 : 78494442
13 : 78494330
13 : 78494272
13 : 78494171
13 : 78494067
13 : 78494064
13 : 78493920
13 : 78493349
13 : 78493297
13 : 78493205
13 : 78493100
13 : 78493066
13 : 78492730
13 : 78492678
13 : 78492568
13 : 78492306
13 : 78492216
13 : 78491982
13 : 78491199
13 : 78470850
13 : 78470739
cg24942919
cg11074192
cg08634041
cg10764762
cg19111971
cg19759502
cg07974719
cg12983394
cg12847373
cg19650157
cg23766591
cg04390523
cg06971129
cg26622320
cg15699226
cg03086857
cg12120741
cg07495027
cg24785726
cg23326536
cg13866767
cg18210860
GeneLoc
PromoterAssoc
CpGIsland
0.01
1e−5
mRNA / METH Corr. FDR
MITF
SOX10
TRPM1
ZEB1
AXL
0.01
1e−5
1e−10
Gene / Regulator Corr. P
Type
mRNA_Expr
CopyNum
AmpDel
S2770A S2688 S2470 S2686 S2338 S2261 S2356 S2125 S2583 S2731 S2405 S2761 S2549 S2495 S2654 S2508 S2510 S2645 S2357 S2189 S2718 S2153 S2410 S2365 S2406 S2279 S2423 S2350 S2333 S2380 S2650 S2320 S2216 S2391 S2800 S2668 S2374 S2247 S2330 S2097 S2408 S2400 S2373 S2667 S2381 S2765 S2379 S2767 S2596 S2812 S2734 S2392 S2521

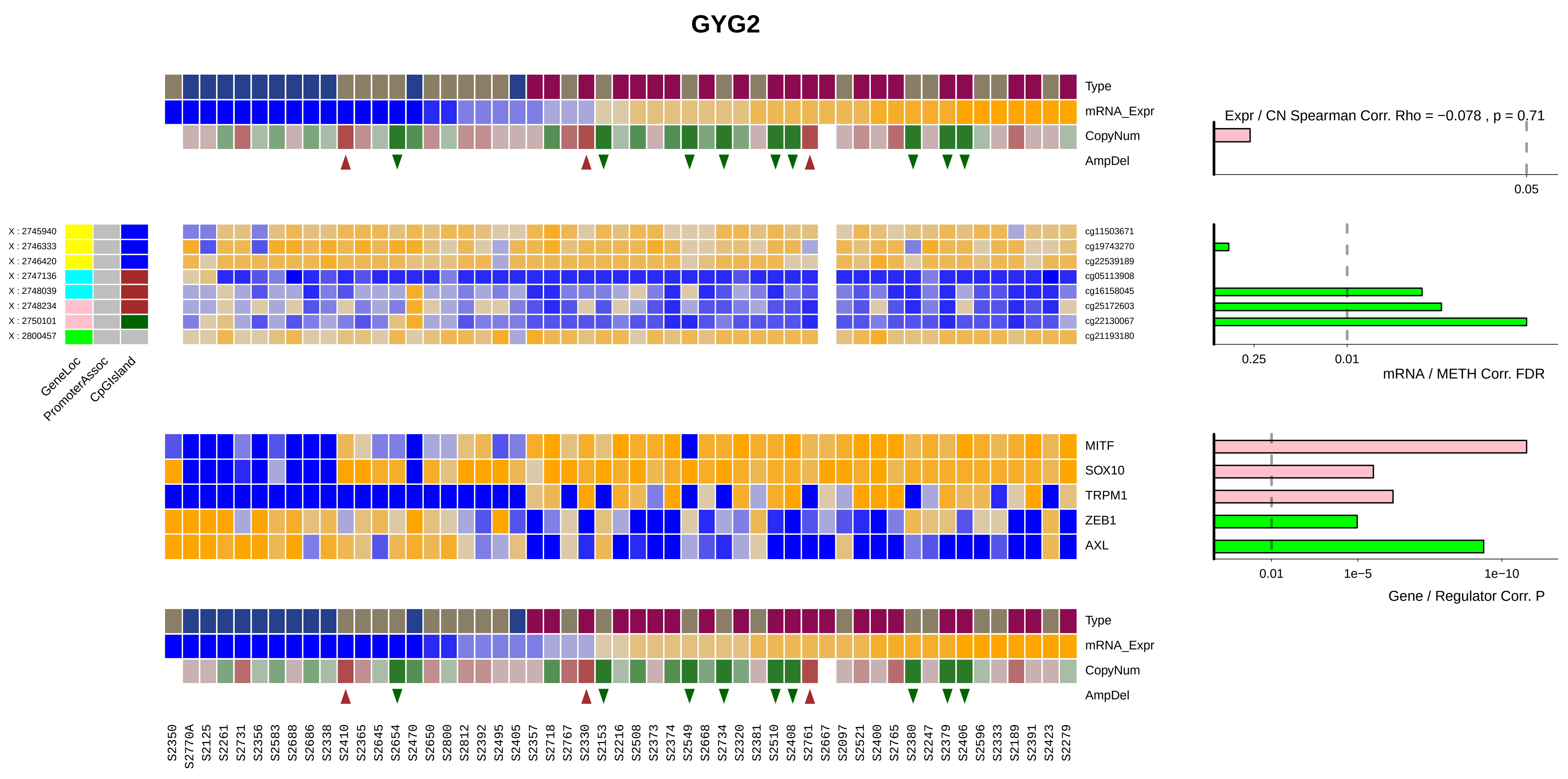

GYG2
Type
mRNA_Expr
CopyNum
AmpDel
Expr / CN Spearman Corr. Rho = −0.078 , p = 0.71
0.05
X : 2745940
X : 2746333
X : 2746420
X : 2747136
X : 2748039
X : 2748234
X : 2750101
X : 2800457
GeneLoc
PromoterAssoc
CpGIsland
cg11503671
cg19743270
cg22539189
cg05113908
cg16158045
cg25172603
cg22130067
cg21193180
0.25
0.01
mRNA / METH Corr. FDR
MITF
SOX10
TRPM1
ZEB1
AXL
0.01
1e−5
1e−10
Gene / Regulator Corr. P
S2350 S2770A S2125 S2261 S2731 S2356 S2583 S2688 S2686 S2338 S2410 S2365 S2645 S2654 S2470 S2650 S2800 S2812 S2392 S2495 S2405 S2357 S2718 S2767 S2330 S2153 S2216 S2508 S2373 S2374 S2549 S2668 S2734 S2320 S2381 S2510 S2408 S2761 S2667 S2097 S2521 S2400 S2765 S2380 S2247 S2379 S2406 S2596 S2333 S2189 S2391 S2423 S2279

CAPN3

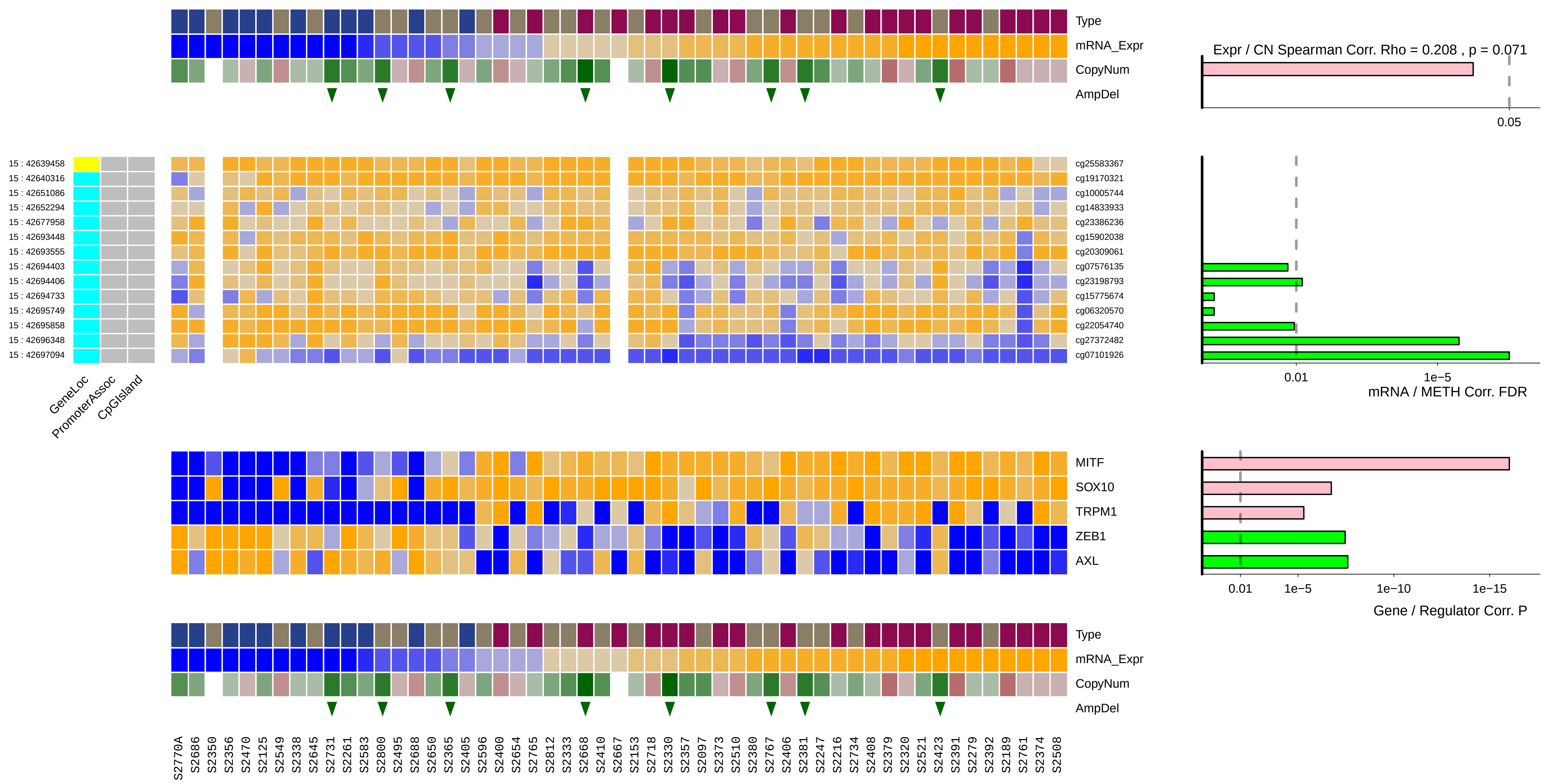
Type
mRNA_Expr
CopyNum
AmpDel
Expr / CN Spearman Corr. Rho = 0.208 , p = 0.071
0.05
15 : 42639458
15 : 42640316
15 : 42651086
15 : 42652294
15 : 42677958
15 : 42693448
15 : 42693555
15 : 42694403
15 : 42694406
15 : 42694733
15 : 42695749
15 : 42695858
15 : 42696348
15 : 42697094
GeneLoc
PromoterAssoc
CpGIsland
cg25583367
cg19170321
cg10005744
cg14833933
cg23386236
cg15902038
cg20309061
cg07576135
cg23198793
cg15775674
cg06320570
cg22054740
cg27372482
cg07101926
0.01
1e−5
mRNA / METH Corr. FDR
MITF
SOX10
TRPM1
ZEB1
AXL
0.01
1e−5
1e−10
1e−15
Gene / Regulator Corr. P
Type
mRNA_Expr
CopyNum
AmpDel
S2770A
S2686
S2350
S2356
S2470
S2125
S2549
S2338
S2645
S2731
S2261
S2583
S2800
S2495
S2688
S2650
S2365
S2405
S2596
S2400
S2654
S2765
S2812
S2333
S2668
S2410
S2667
S2153
S2718
S2330
S2357
S2097
S2373
S2510
S2380
S2767
S2406
S2381
S2247
S2216
S2734
S2408
S2379
S2320
S2521
S2423
S2391
S2279
S2392
S2189
S2761
S2374
S2508

BEST1

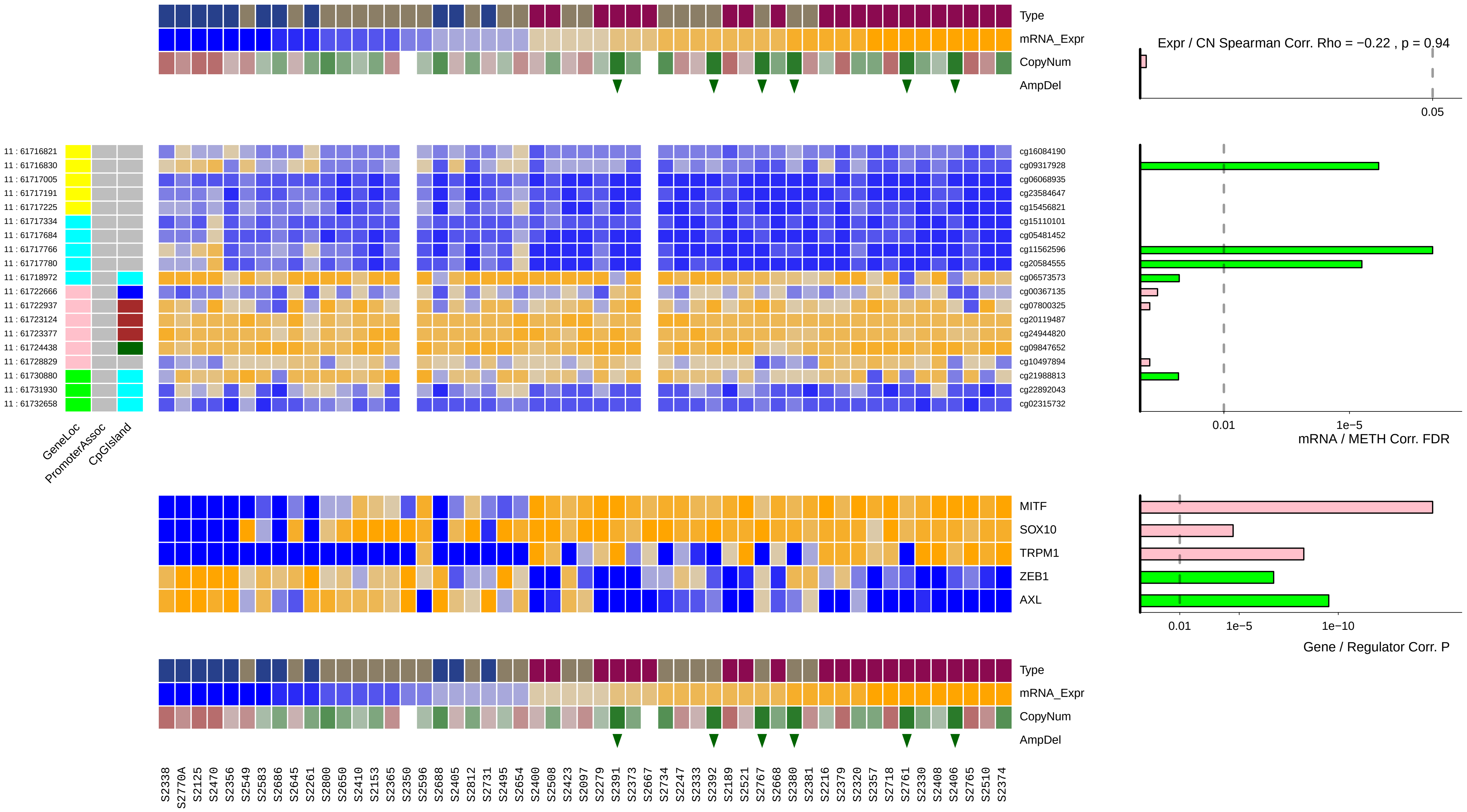

Type
mRNA_Expr
CopyNum
AmpDel
Expr / CN Spearman Corr. Rho = −0.22 , p = 0.94
0.05
11 : 61716821
11 : 61716830
11 : 61717005
11 : 61717191
11 : 61717225
11 : 61717334
11 : 61717684
11 : 61717766
11 : 61717780
11 : 61718972
11 : 61722666
11 : 61722937
11 : 61723124
11 : 61723377
11 : 61724438
11 : 61728829
11 : 61730880
11 : 61731930
11 : 61732658
cg16084190
cg09317928
cg06068935
cg23584647
cg15456821
cg15110101
cg05481452
cg11562596
cg20584555
cg06573573
cg00367135
cg07800325
cg20119487
cg24944820
cg09847652
cg10497894
cg21988813
cg22892043
cg02315732
0.01
1e−5
mRNA / METH Corr. FDR
GeneLoc
PromoterAssoc
CpGIsland
MITF
SOX10
TRPM1
ZEB1
AXL
0.01
1e−5
1e−10
Gene / Regulator Corr. P
Type
mRNA_Expr
CopyNum
AmpDel
S2338 S2770A S2125 S2470 S2356 S2549 S2583 S2686 S2645 S2261 S2800 S2650 S2410 S2153 S2365 S2350 S2596 S2688 S2405 S2812 S2731 S2495 S2654 S2400 S2508 S2423 S2097 S2279 S2391 S2373 S2667 S2734 S2247 S2333 S2392 S2189 S2521 S2767 S2668 S2380 S2381 S2216 S2379 S2320 S2357 S2718 S2761 S2330 S2408 S2406 S2765 S2510 S2374

IRF4

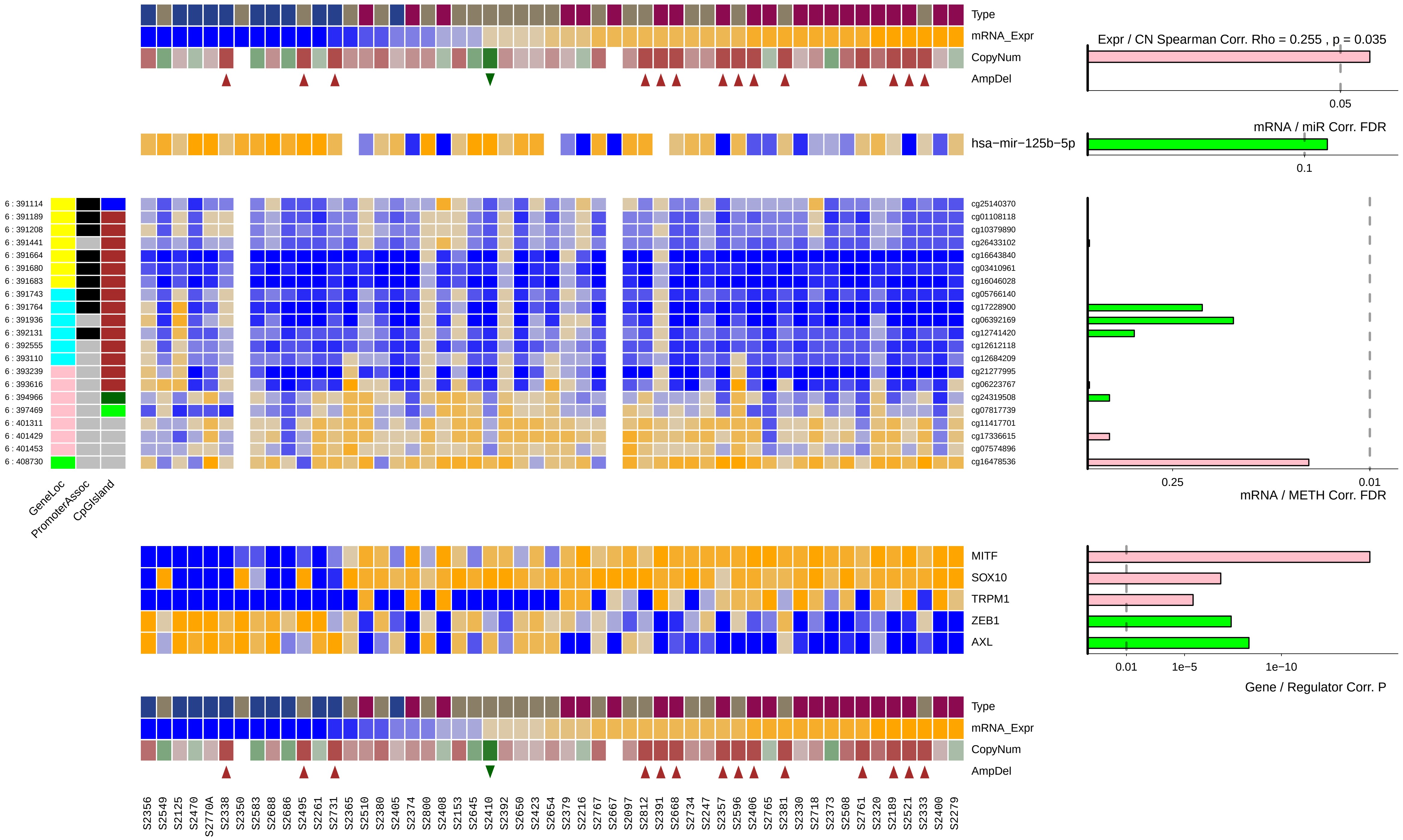
Type
mRNA_Expr
CopyNum
AmpDel
Expr / CN Spearman Corr. Rho = 0.255 , p = 0.035
0.05
hsa−mir−125b−5p
mRNA / miR Corr. FDR
0.1
6 : 391114
6 : 391189
6 : 391208
6 : 391441
6 : 391664
6 : 391680
6 : 391683
6 : 391743
6 : 391764
6 : 391936
6 : 392131
6 : 392555
6 : 393110
6 : 393239
6 : 393616
6 : 394966
6 : 397469
6 : 401311
6 : 401429
6 : 401453
6 : 408730
GeneLoc
PromoterAssoc
CpGIsland
cg25140370
cg01108118
cg10379890
cg26433102
cg16643840
cg03410961
cg16046028
cg05766140
cg17228900
cg06392169
cg12741420
cg12612118
cg12684209
cg21277995
cg06223767
cg24319508
cg07817739
cg11417701
cg17336615
cg07574896
cg16478536
0.25
0.01
mRNA / METH Corr. FDR
MITF
SOX10
TRPM1
ZEB1
AXL
0.01
1e−5
1e−10
Gene / Regulator Corr. P
Type
mRNA_Expr
CopyNum
AmpDel
S2356 S2549 S2125 S2470 S2770A S2338 S2350 S2583 S2688 S2686 S2495 S2261 S2731 S2365 S2510 S2380 S2405 S2374 S2800 S2408 S2153 S2645 S2410 S2392 S2650 S2423 S2654 S2379 S2216 S2767 S2667 S2097 S2812 S2391 S2668 S2734 S2247 S2357 S2596 S2406 S2765 S2381 S2330 S2718 S2373 S2508 S2761 S2320 S2189 S2521 S2333 S2400 S2279

OCA2

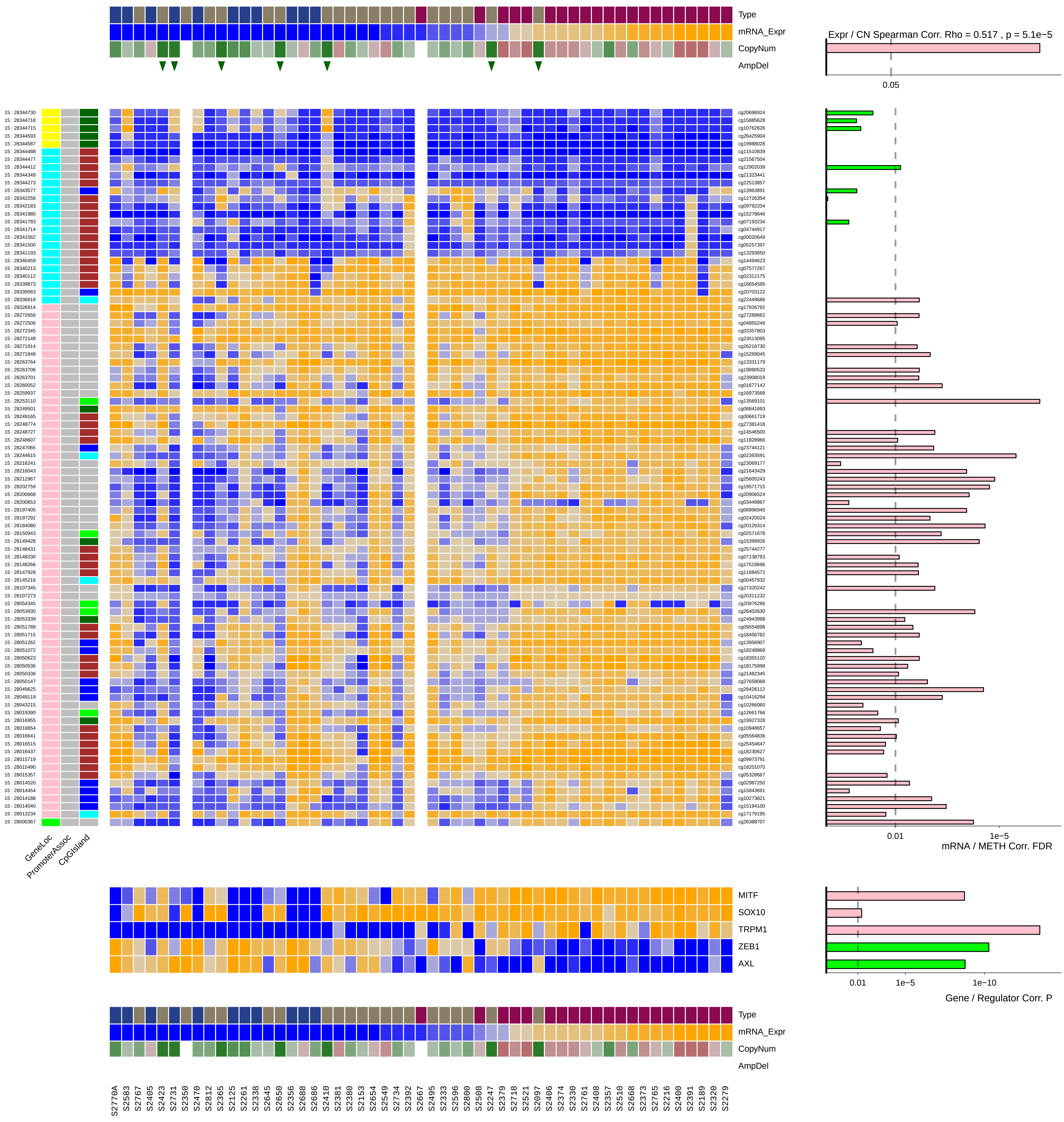

Type
mRNA_Expr
CopyNum
AmpDel
Expr / CN Spearman Corr. Rho = 0.517 , p = 5.1e−5
0.05
GeneLoc
PromoterAssoc
CpGIsland
0.01
1e−5
mRNA / METH Corr. FDR
MITF
SOX10
TRPM1
ZEB1
AXL
0.01
1e−5
1e−10
Gene / Regulator Corr. P
Type
mRNA_Expr
CopyNum
AmpDel
S2770A S2583 S2767 S2405 S2423 S2731 S2350 S2470 S2812 S2365 S2125 S2261 S2338 S2645 S2650 S2356 S2688 S2686 S2410 S2381 S2380 S2153 S2654 S2549 S2734 S2392 S2667 S2495 S2333 S2596 S2800 S2508 S2247 S2379 S2718 S2521 S2097 S2406 S2374 S2330 S2761 S2408 S2357 S2510 S2668 S2373 S2765 S2216 S2400 S2391 S2189 S2320 S2279

TBC1D7

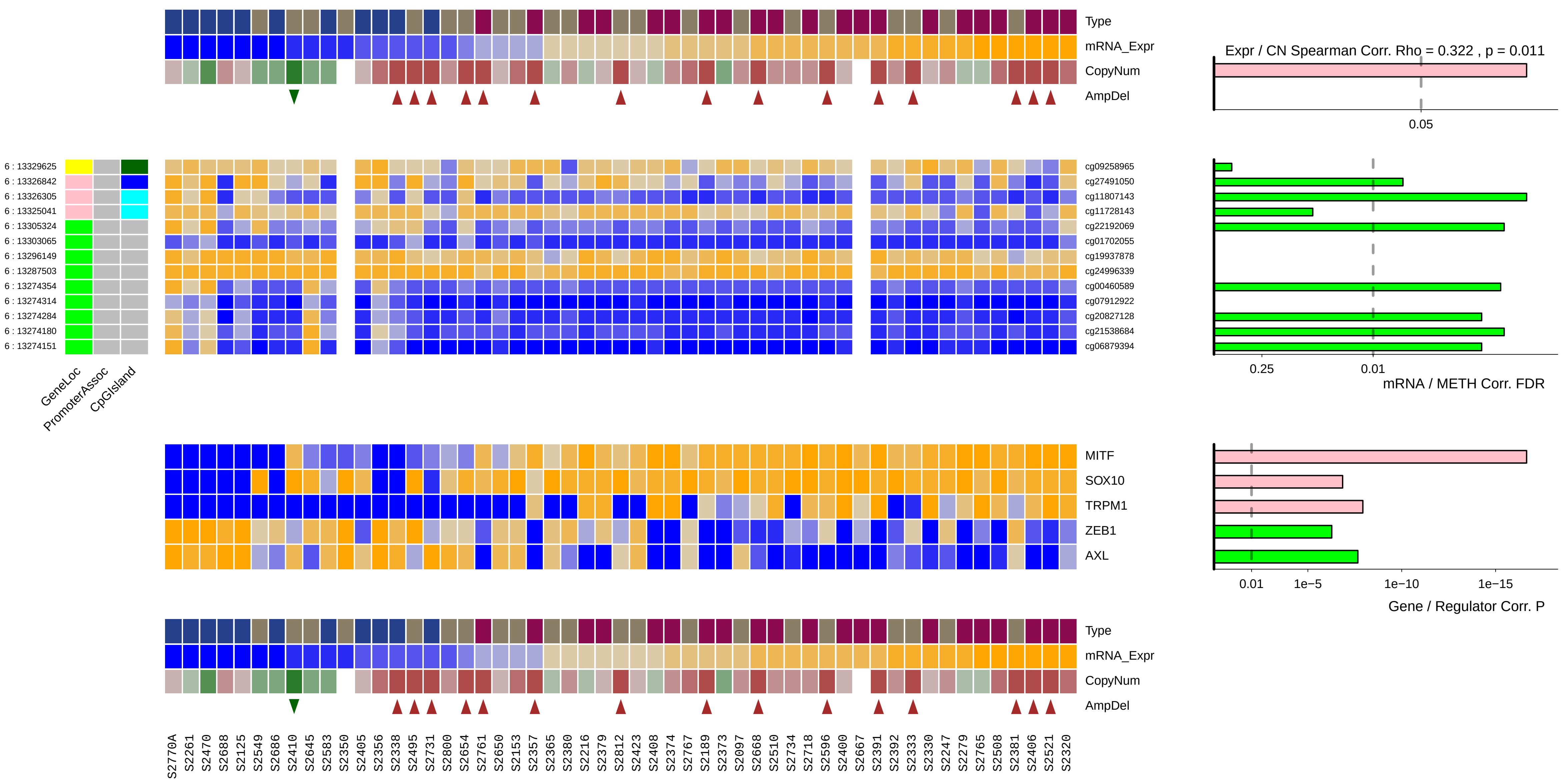

Type
mRNA_Expr
CopyNum
AmpDel
Expr / CN Spearman Corr. Rho = 0.322 , p = 0.011
0.05
6 : 13329625
6 : 13326842
6 : 13326305
6 : 13325041
6 : 13305324
6 : 13303065
6 : 13296149
6 : 13287503
6 : 13274354
6 : 13274314
6 : 13274284
6 : 13274180
6 : 13274151
GeneLoc
PromoterAssoc
CpGIsland
cg09258965
cg27491050
cg11807143
cg11728143
cg22192069
cg01702055
cg19937878
cg24996339
cg00460589
cg07912922
cg20827128
cg21538684
cg06879394
0.25
0.01
mRNA / METH Corr. FDR
MITF
SOX10
TRPM1
ZEB1
AXL
0.01
1e−5
1e−10
1e−15
Gene / Regulator Corr. P
Type
mRNA_Expr
CopyNum
AmpDel
S2770A S2261 S2470 S2688 S2125 S2549 S2686 S2410 S2645 S2583 S2350 S2405 S2356 S2338 S2495 S2731 S2800 S2654 S2761 S2650 S2153 S2357 S2365 S2380 S2216 S2379 S2812 S2423 S2408 S2374 S2767 S2189 S2373 S2097 S2668 S2510 S2734 S2718 S2596 S2400 S2667 S2391 S2392 S2333 S2330 S2247 S2279 S2765 S2508 S2381 S2406 S2521 S2320

C10ORF90

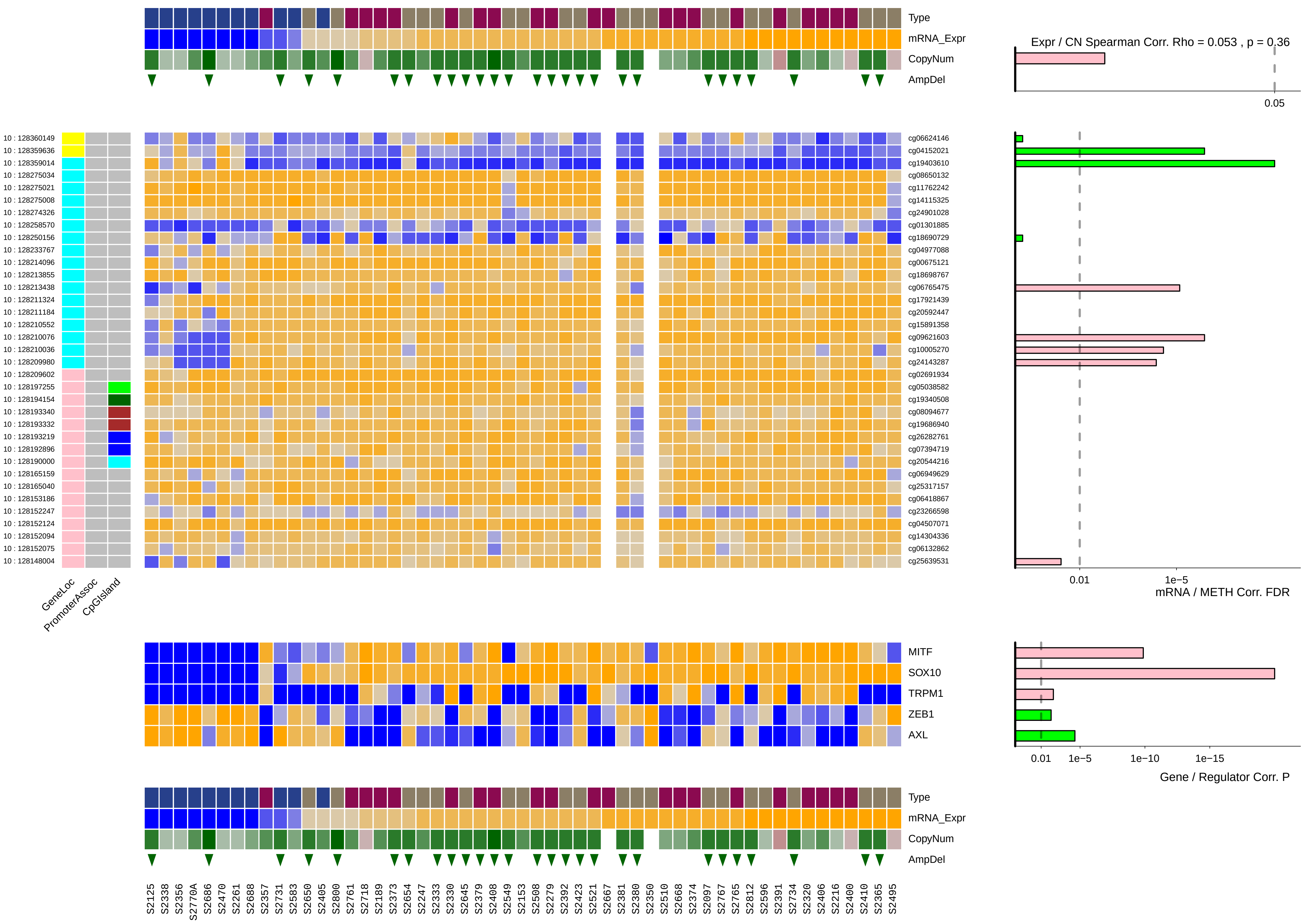
Type
mRNA_Expr
CopyNum
AmpDel
Expr / CN Spearman Corr. Rho = 0.053 , p = 0.36
0.05
10 : 128360149
10 : 128359636
10 : 128359014
10 : 128275034
10 : 128275021
10 : 128275008
10 : 128274326
10 : 128258570
10 : 128250156
10 : 128233767
10 : 128214096
10 : 128213855
10 : 128213438
10 : 128211324
10 : 128211184
10 : 128210552
10 : 128210076
10 : 128210036
10 : 128209980
10 : 128209602
10 : 128197255
10 : 128194154
10 : 128193340
10 : 128193332
10 : 128193219
10 : 128192896
10 : 128190000
10 : 128165159
10 : 128165040
10 : 128153186
10 : 128152247
10 : 128152124
10 : 128152094
10 : 128152075
10 : 128148004
cg06624146
cg04152021
cg19403610
cg08650132
cg11762242
cg14115325
cg24901028
cg01301885
cg18690729
cg04977088
cg00675121
cg18698767
cg06765475
cg17921439
cg20592447
cg15891358
cg09621603
cg10005270
cg24143287
cg02691934
cg05038582
cg19340508
cg08094677
cg19686940
cg26282761
cg07394719
cg20544216
cg06949629
cg25317157
cg06418867
cg23266598
cg04507071
cg14304336
cg06132862
cg25639531
0.01
1e−5
mRNA / METH Corr. FDR
GeneLoc
PromoterAssoc
CpGIsland
MITF
SOX10
TRPM1
ZEB1
AXL
0.01
1e−5
1e−10
1e−15
Gene / Regulator Corr. P
Type
mRNA_Expr
CopyNum
AmpDel
S2125
S2338
S2356
S2770A
S2686
S2470
S2261
S2688
S2357
S2731
S2583
S2650
S2405
S2800
S2761
S2718
S2189
S2373
S2654
S2247
S2333
S2330
S2645
S2379
S2408
S2549
S2153
S2508
S2279
S2392
S2423
S2521
S2667
S2381
S2380
S2350
S2510
S2668
S2374
S2097
S2767
S2765
S2812
S2596
S2391
S2734
S2320
S2406
S2216
S2400
S2410
S2365
S2495

ZNF704

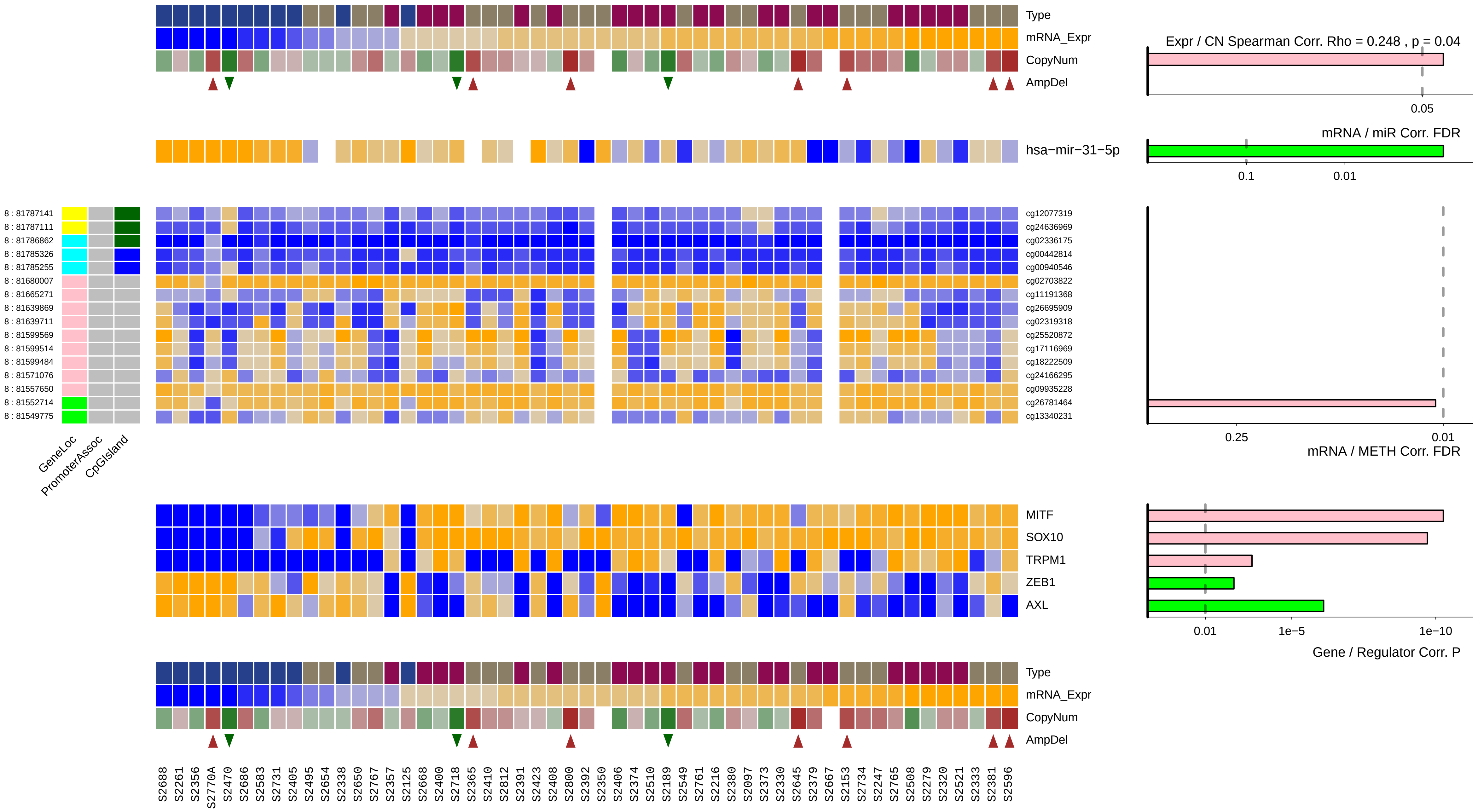
Type
mRNA_Expr
CopyNum
AmpDel
Expr / CN Spearman Corr. Rho = 0.248 , p = 0.04
0.05
hsa−mir−31−5p
mRNA / miR Corr. FDR
0.1
0.01
0.25
0.01
mRNA / METH Corr. FDR
GeneLoc
PromoterAssoc
CpGIsland
MITF
SOX10
TRPM1
ZEB1
AXL
0.01
1e−5
1e−10
Gene / Regulator Corr. P

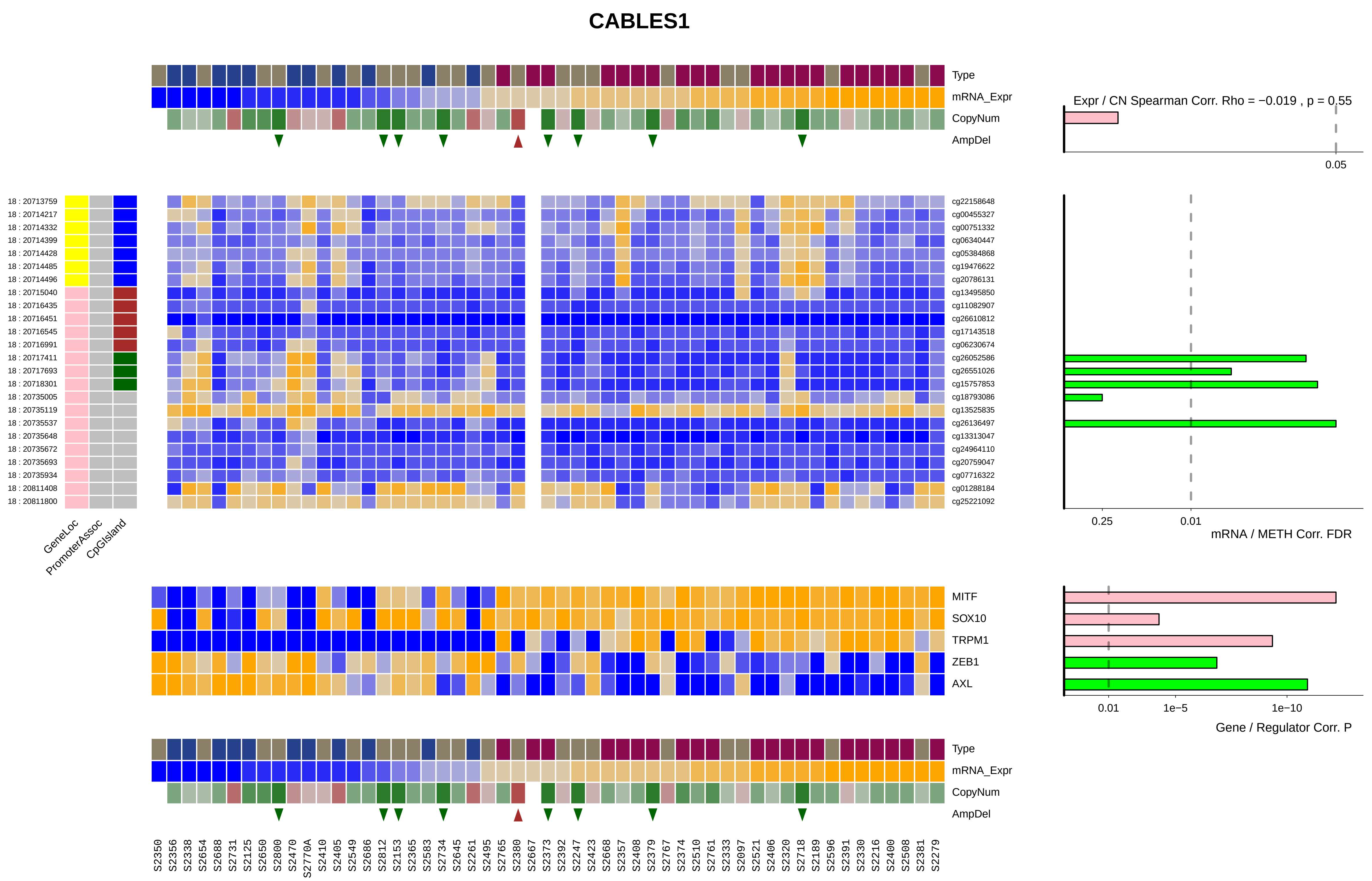

CABLES1
Type
mRNA_Expr
CopyNum
AmpDel
Expr / CN Spearman Corr. Rho = −0.019 , p = 0.55
0.05
mRNA / METH Corr. FDR
0.25
0.01
GeneLoc
PromoterAssoc
CpGIsland
MITF
SOX10
TRPM1
ZEB1
AXL
Gene / Regulator Corr. P
1e−5
1e−10

TRPM1

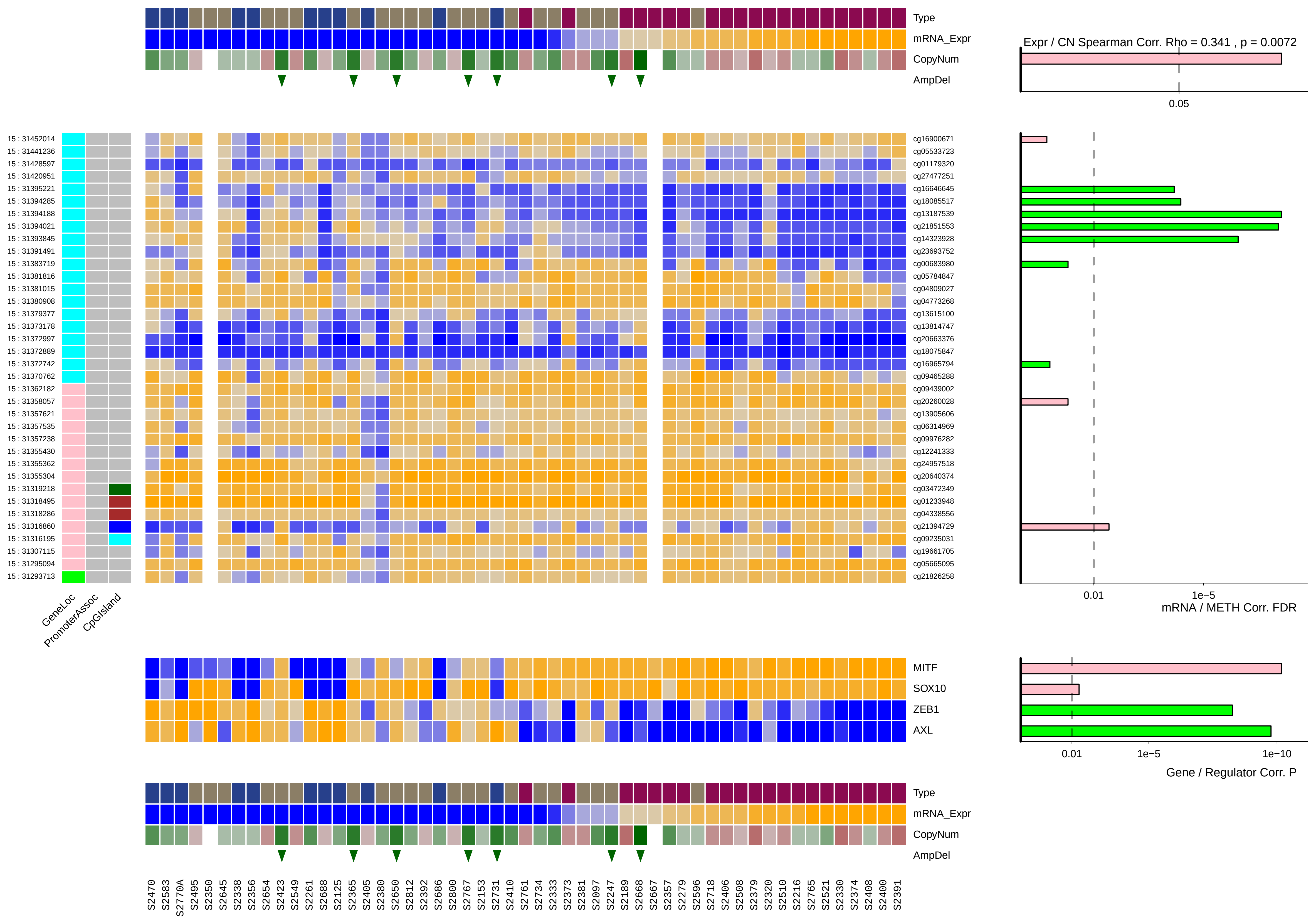
Type
mRNA_Expr
CopyNum
AmpDel
Expr / CN Spearman Corr. Rho = 0.341 , p = 0.0072
0.05
mRNA / METH Corr. FDR
0.01
1e−5
GeneLoc
PromoterAssoc
CpGIsland
MITF
SOX10
ZEB1
AXL
Gene / Regulator Corr. P
1e−10

WDR91

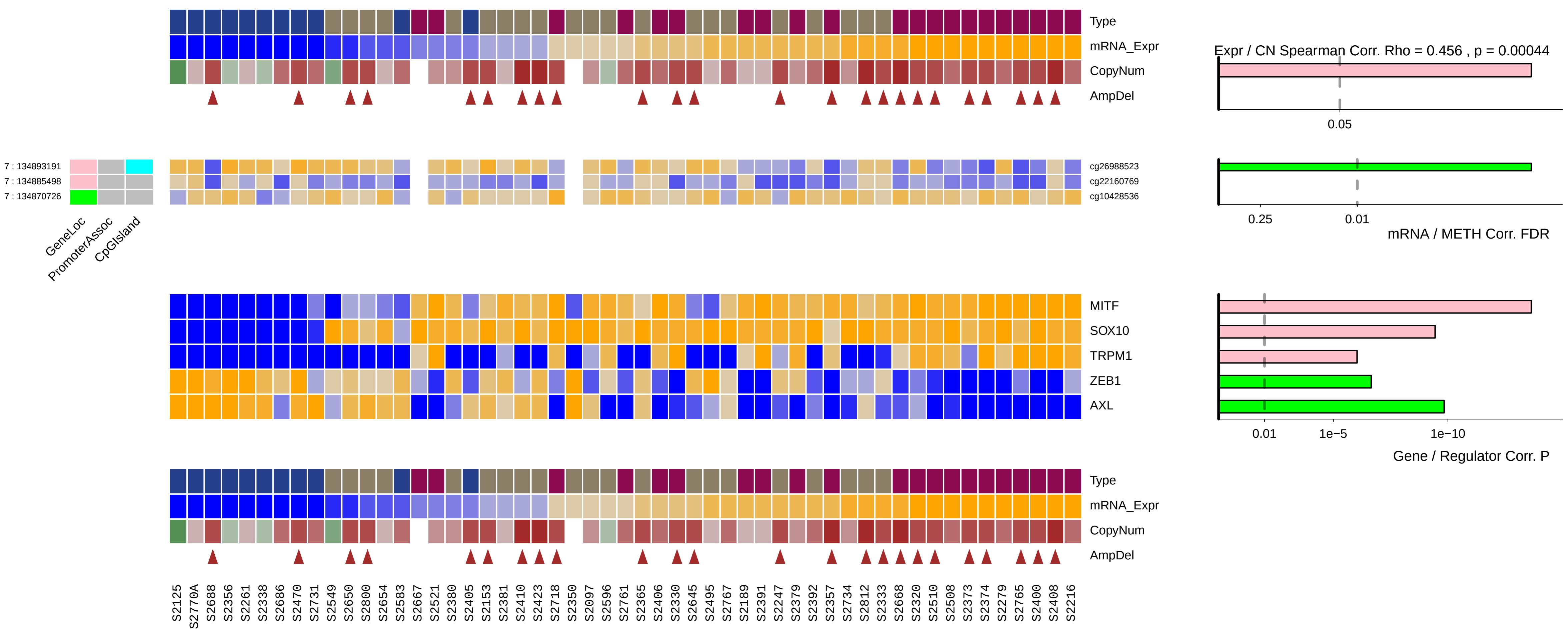

Type
mRNA_Expr
CopyNum
AmpDel
Expr / CN Spearman Corr. Rho = 0.456 , p = 0.00044
0.05
7 : 134893191
7 : 134885498
7 : 134870726
cg26988523
cg22160769
cg10428536
0.25
0.01
mRNA / METH Corr. FDR
GeneLoc
PromoterAssoc
CpGIsland
MITF
SOX10
TRPM1
ZEB1
AXL
0.01
1e−5
1e−10
Gene / Regulator Corr. P
S2125 S2770A S2688 S2356 S2261 S2338 S2686 S2470 S2731 S2549 S2650 S2800 S2654 S2583 S2667 S2521 S2380 S2405 S2153 S2381 S2410 S2423 S2718 S2350 S2097 S2596 S2761 S2365 S2406 S2330 S2645 S2495 S2767 S2189 S2391 S2247 S2379 S2392 S2357 S2734 S2812 S2333 S2668 S2320 S2510 S2508 S2373 S2374 S2279 S2765 S2400 S2408 S2216

SLC1A4

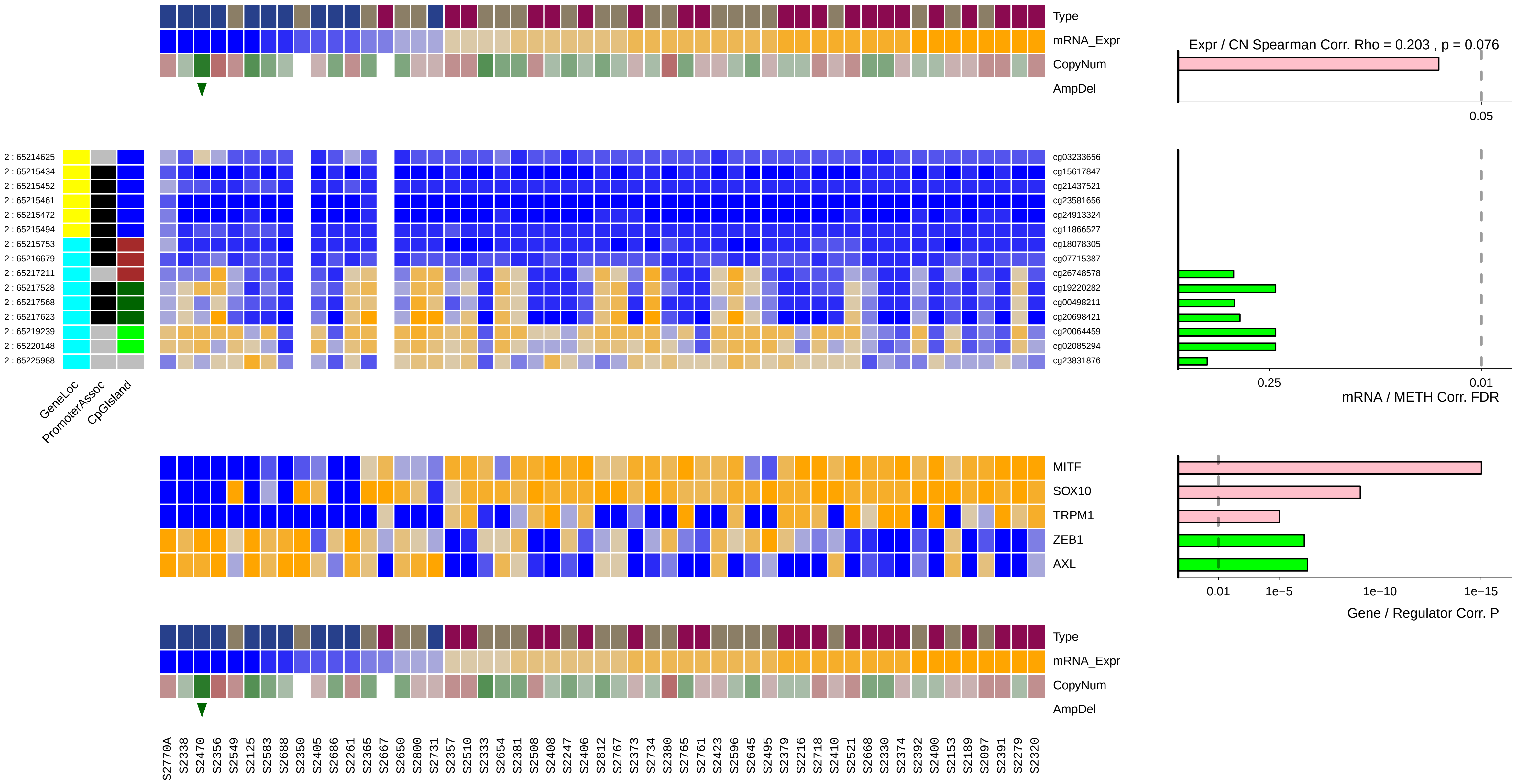

Type
mRNA_Expr
CopyNum
AmpDel
Expr / CN Spearman Corr. Rho = 0.203 , p = 0.076
0.05
2 : 65214625
2 : 65215434
2 : 65215452
2 : 65215461
2 : 65215472
2 : 65215494
2 : 65215753
2 : 65216679
2 : 65217211
2 : 65217528
2 : 65217568
2 : 65217623
2 : 65219239
2 : 65220148
2 : 65225988
cg03233656
cg15617847
cg21437521
cg23581656
cg24913324
cg11866527
cg18078305
cg07715387
cg26748578
cg19220282
cg00498211
cg20698421
cg20064459
cg02085294
cg23831876
GeneLoc
PromoterAssoc
CpGIsland
0.25
0.01
mRNA / METH Corr. FDR
MITF
SOX10
TRPM1
ZEB1
AXL
0.01
1e−5
1e−10
1e−15
Gene / Regulator Corr. P
Type
mRNA_Expr
CopyNum
AmpDel
S2770A S2338 S2470 S2356 S2549 S2125 S2583 S2688 S2350 S2405 S2686 S2261 S2365 S2667 S2650 S2800 S2731 S2357 S2510 S2333 S2654 S2381 S2508 S2408 S2247 S2406 S2812 S2767 S2373 S2734 S2380 S2765 S2761 S2423 S2596 S2645 S2495 S2379 S2216 S2718 S2410 S2521 S2668 S2330 S2374 S2392 S2400 S2153 S2189 S2097 S2391 S2279 S2320

RAB27A

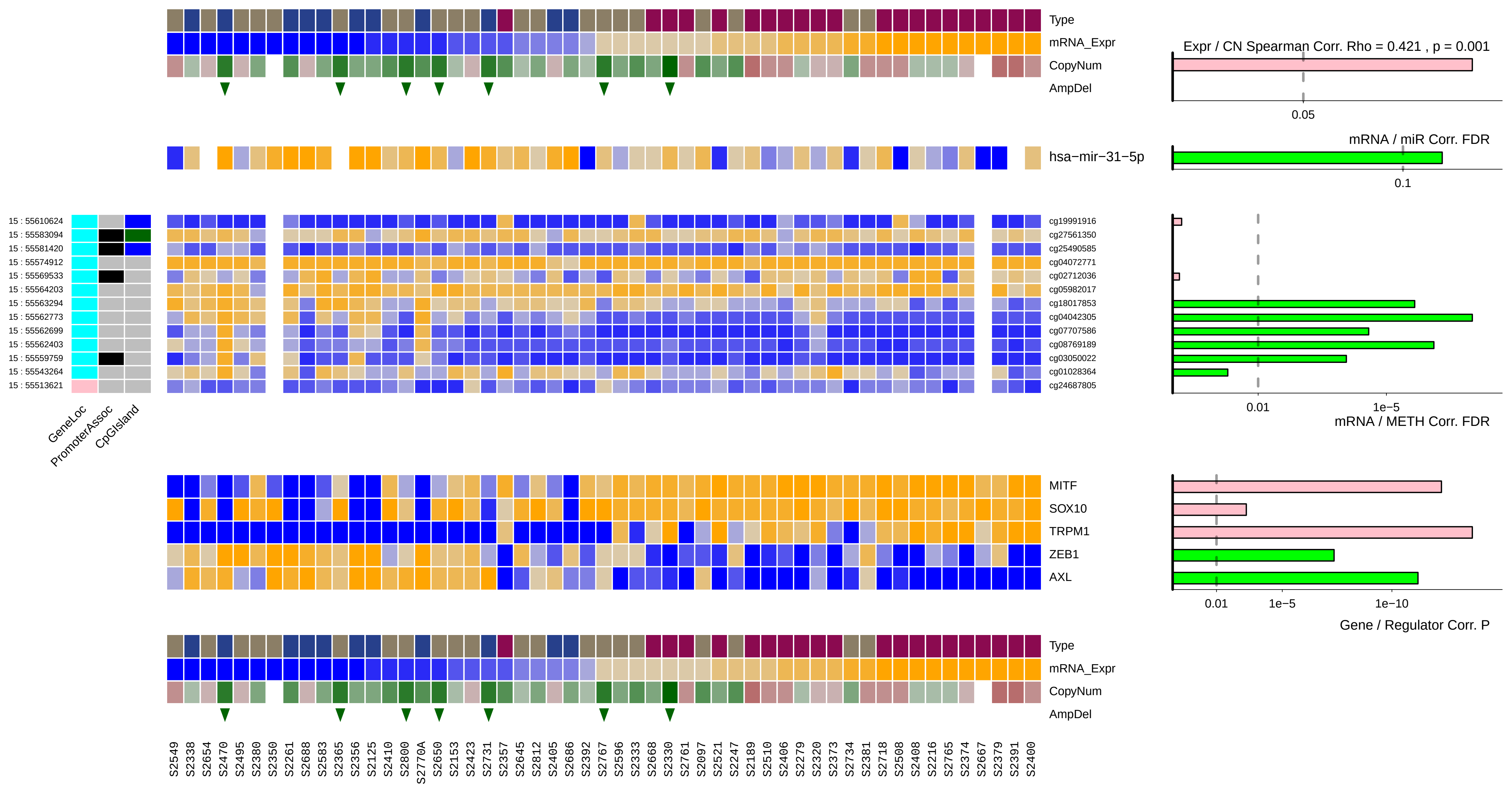

Type
mRNA_Expr
CopyNum
AmpDel
Expr / CN Spearman Corr. Rho = 0.421 , p = 0.001
0.05
hsa−mir−31−5p
mRNA / miR Corr. FDR
0.1
mRNA / METH Corr. FDR
0.01
1e−5
GeneLoc
PromoterAssoc
CpGIsland
MITF
SOX10
TRPM1
ZEB1
AXL
Gene / Regulator Corr. P
1e−10

GJB1

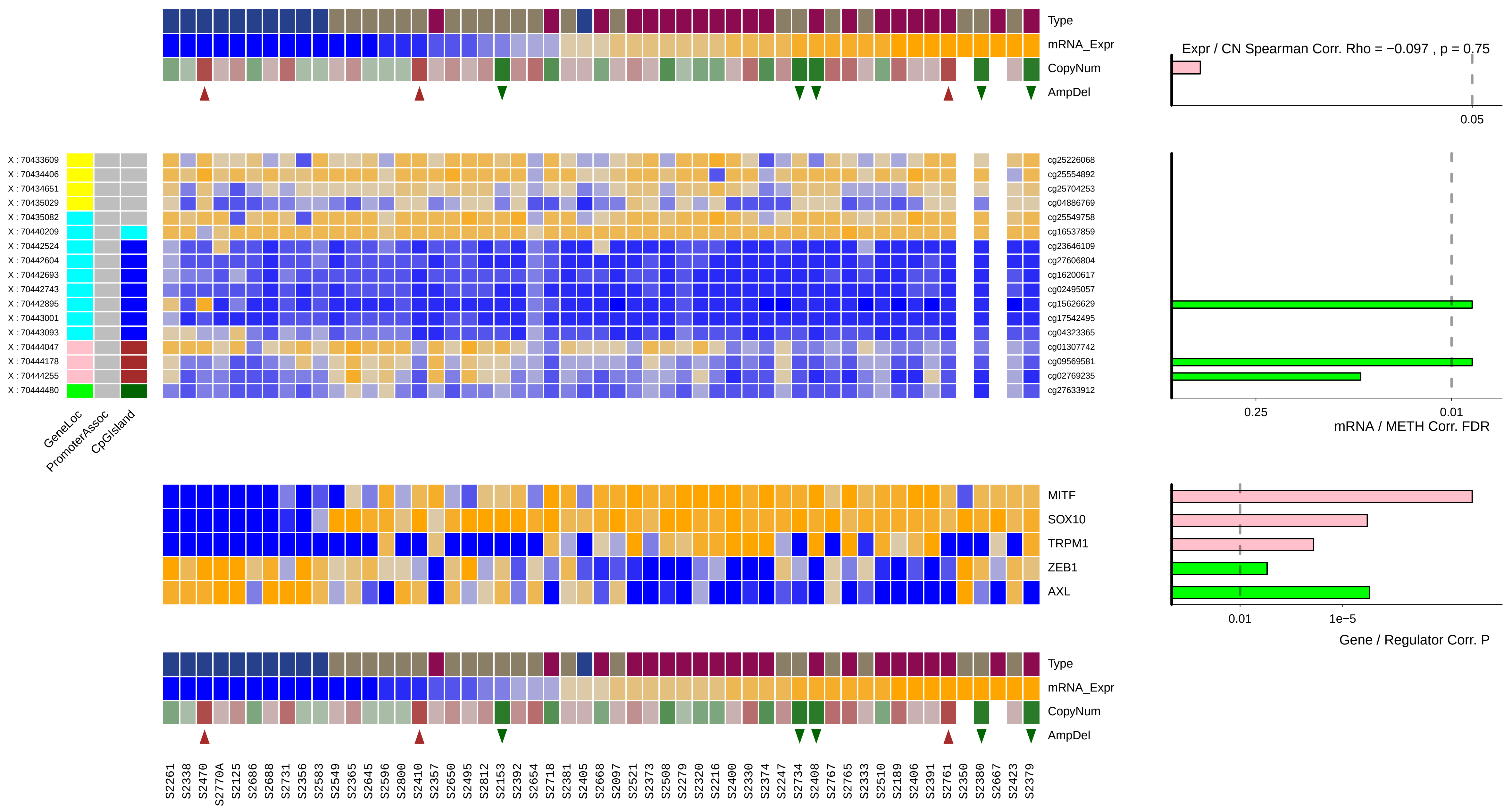
Type
mRNA_Expr
CopyNum
AmpDel
Expr / CN Spearman Corr. Rho = −0.097 , p = 0.75
0.05
X : 70433609
X : 70434406
X : 70434651
X : 70435029
X : 70435082
X : 70440209
X : 70442524
X : 70442604
X : 70442693
X : 70442743
X : 70442895
X : 70443001
X : 70443093
X : 70444047
X : 70444178
X : 70444255
X : 70444480
cg25226068
cg25554892
cg25704253
cg04886769
cg25549758
cg16537859
cg23646109
cg27606804
cg16200617
cg02495057
cg15626629
cg17542495
cg04323365
cg01307742
cg09569581
cg02769235
cg27633912
GeneLoc
PromoterAssoc
CpGIsland
0.25
0.01
mRNA / METH Corr. FDR
MITF
SOX10
TRPM1
ZEB1
AXL
0.01
1e−5
Gene / Regulator Corr. P
S2261 S2338 S2470 S2770A S2125 S2686 S2688 S2731 S2356 S2583 S2549 S2365 S2645 S2596 S2800 S2410 S2357 S2650 S2495 S2812 S2153 S2392 S2654 S2718 S2381 S2405 S2668 S2097 S2521 S2373 S2508 S2279 S2320 S2216 S2400 S2330 S2374 S2247 S2734 S2408 S2767 S2765 S2333 S2510 S2189 S2406 S2391 S2761 S2350 S2380 S2667 S2423 S2379

IGSF8

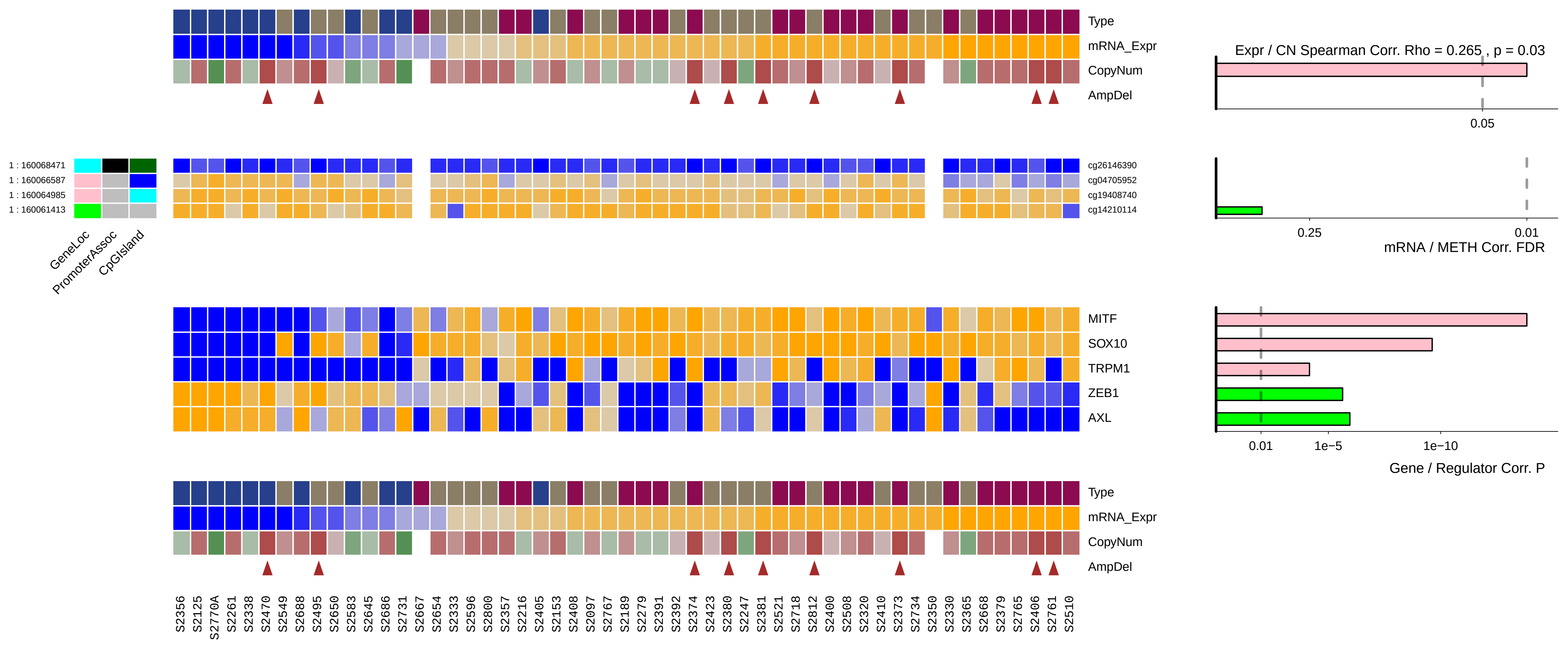

Type
mRNA_Expr
CopyNum
AmpDel
Expr / CN Spearman Corr. Rho = 0.265 , p = 0.03
0.05
1 : 160068471
1 : 160066587
1 : 160064985
1 : 160061413
GeneLoc
PromoterAssoc
CpGIsland
cg26146390
cg04705952
cg19408740
cg14210114
0.25
0.01
mRNA / METH Corr. FDR
MITF
SOX10
TRPM1
ZEB1
AXL
0.01
1e−5
1e−10
Gene / Regulator Corr. P
Type
mRNA_Expr
CopyNum
AmpDel
S2356 S2125 S2770A S2261 S2338 S2470 S2549 S2688 S2495 S2650 S2583 S2645 S2686 S2731 S2667 S2654 S2333 S2596 S2800 S2357 S2216 S2405 S2153 S2408 S2097 S2767 S2189 S2279 S2391 S2392 S2374 S2423 S2380 S2247 S2381 S2521 S2718 S2812 S2400 S2508 S2320 S2410 S2373 S2734 S2350 S2330 S2365 S2668 S2379 S2765 S2406 S2761 S2510

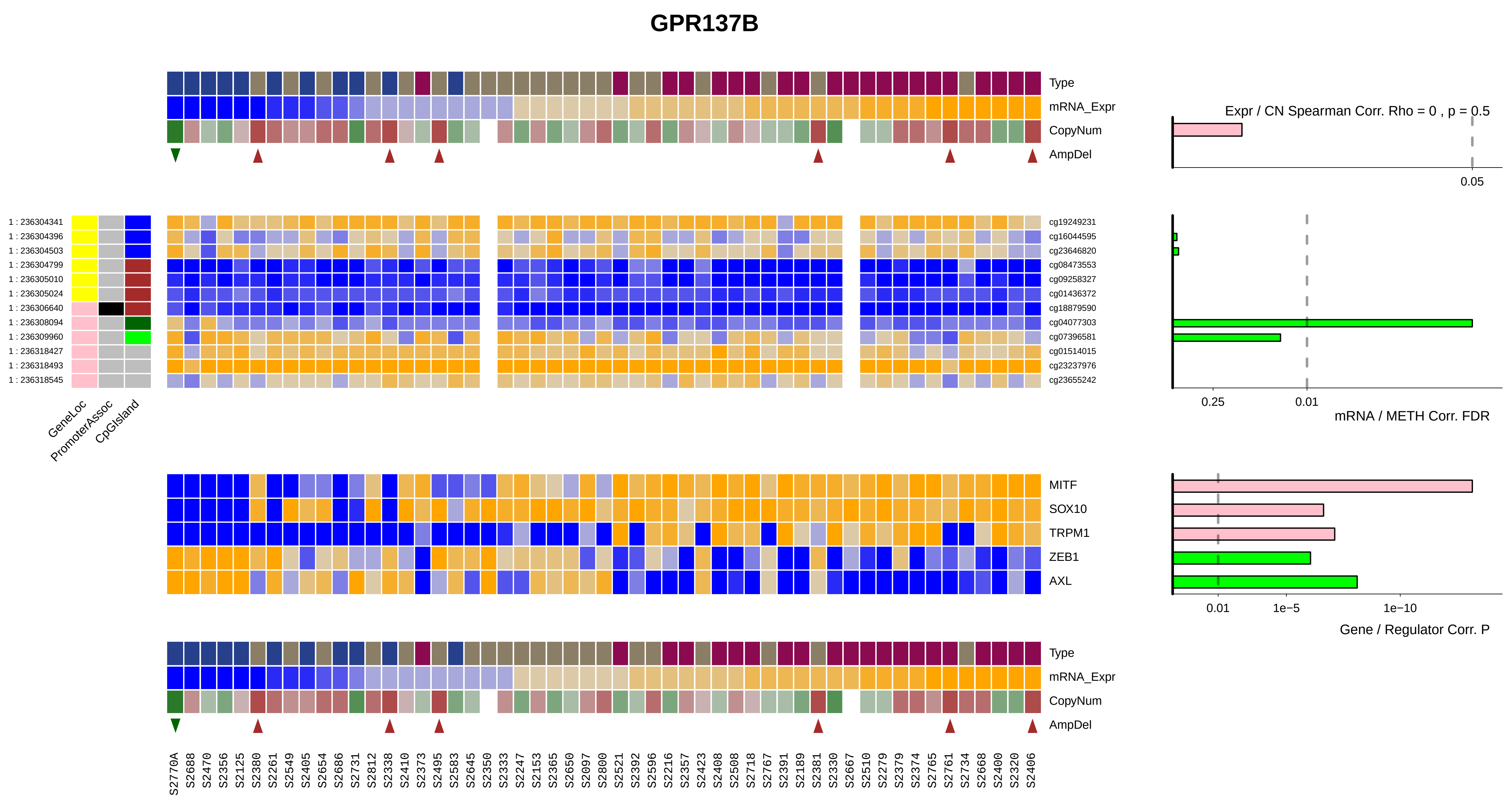
GPR137B
Type
mRNA_Expr
CopyNum
AmpDel
Expr / CN Spearman Corr. Rho = 0 , p = 0.5
0.05
1 : 236304341
1 : 236304396
1 : 236304503
1 : 236304799
1 : 236305010
1 : 236305024
1 : 236306640
1 : 236308094
1 : 236309960
1 : 236318427
1 : 236318493
1 : 236318545
cg19249231
cg16044595
cg23646820
cg08473553
cg09258327
cg01436372
cg18879590
cg04077303
cg07396581
cg01514015
cg23237976
cg23655242
0.25
0.01
mRNA / METH Corr. FDR
GeneLoc
PromoterAssoc
CpGIsland
MITF
SOX10
TRPM1
ZEB1
AXL
0.01
1e−5
1e−10
Gene / Regulator Corr. P
S2770A S2688 S2470 S2356 S2125 S2380 S2261 S2549 S2405 S2654 S2686 S2731 S2812 S2338 S2410 S2373 S2495 S2583 S2645 S2350 S2333 S2247 S2153 S2365 S2650 S2097 S2800 S2521 S2392 S2596 S2216 S2357 S2423 S2408 S2508 S2718 S2767 S2391 S2189 S2381 S2330 S2667 S2510 S2279 S2379 S2374 S2765 S2761 S2734 S2668 S2400 S2320 S2406

SEMA6A

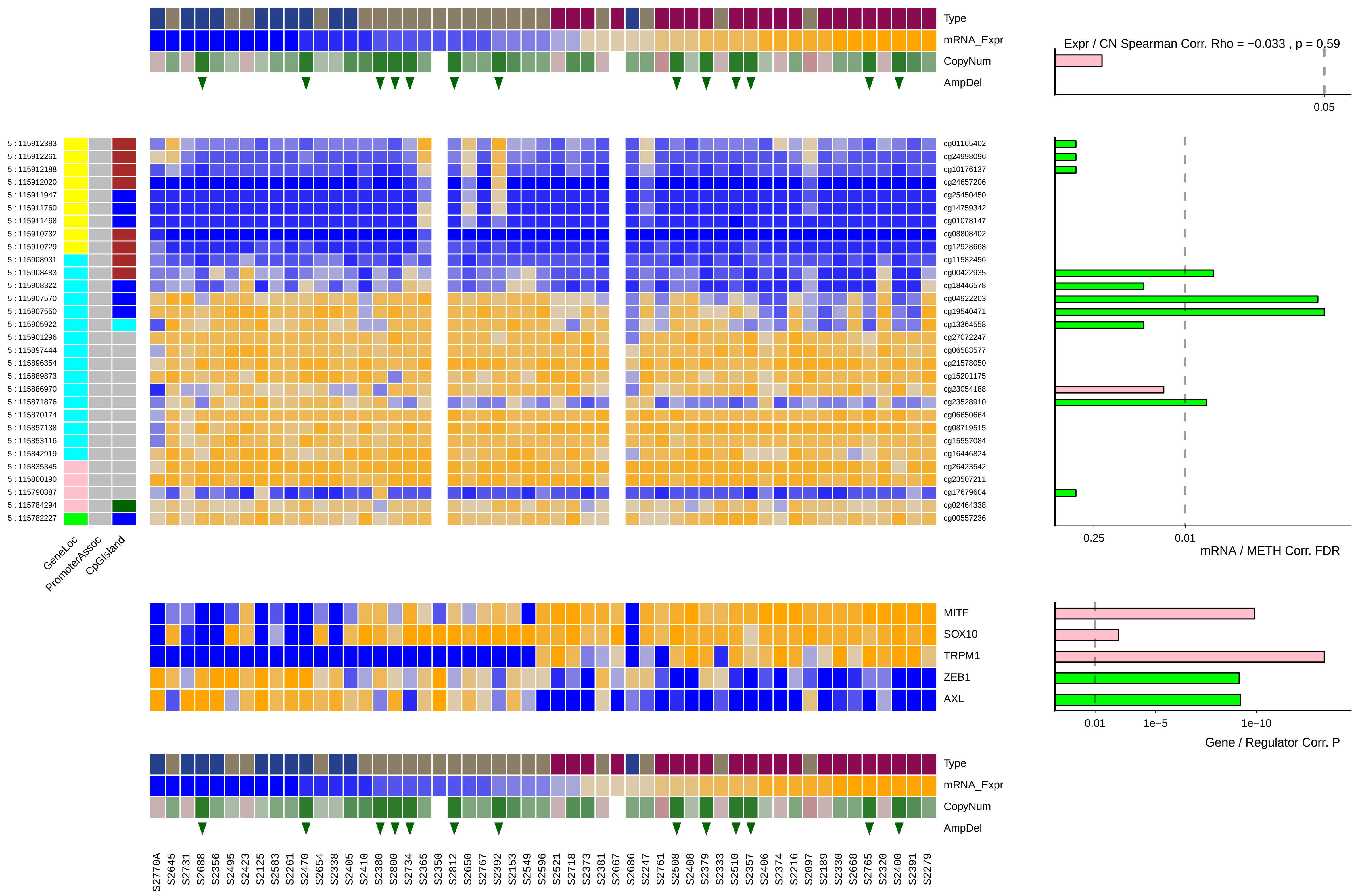
Type
mRNA_Expr
CopyNum
AmpDel
Expr / CN Spearman Corr. Rho = −0.033 , p = 0.59
0.05
GeneLoc
PromoterAssoc
CpGIsland
mRNA / METH Corr. FDR
0.25
0.01
MITF
SOX10
TRPM1
ZEB1
AXL
Gene / Regulator Corr. P
1e−5
1e−10

NR4A3

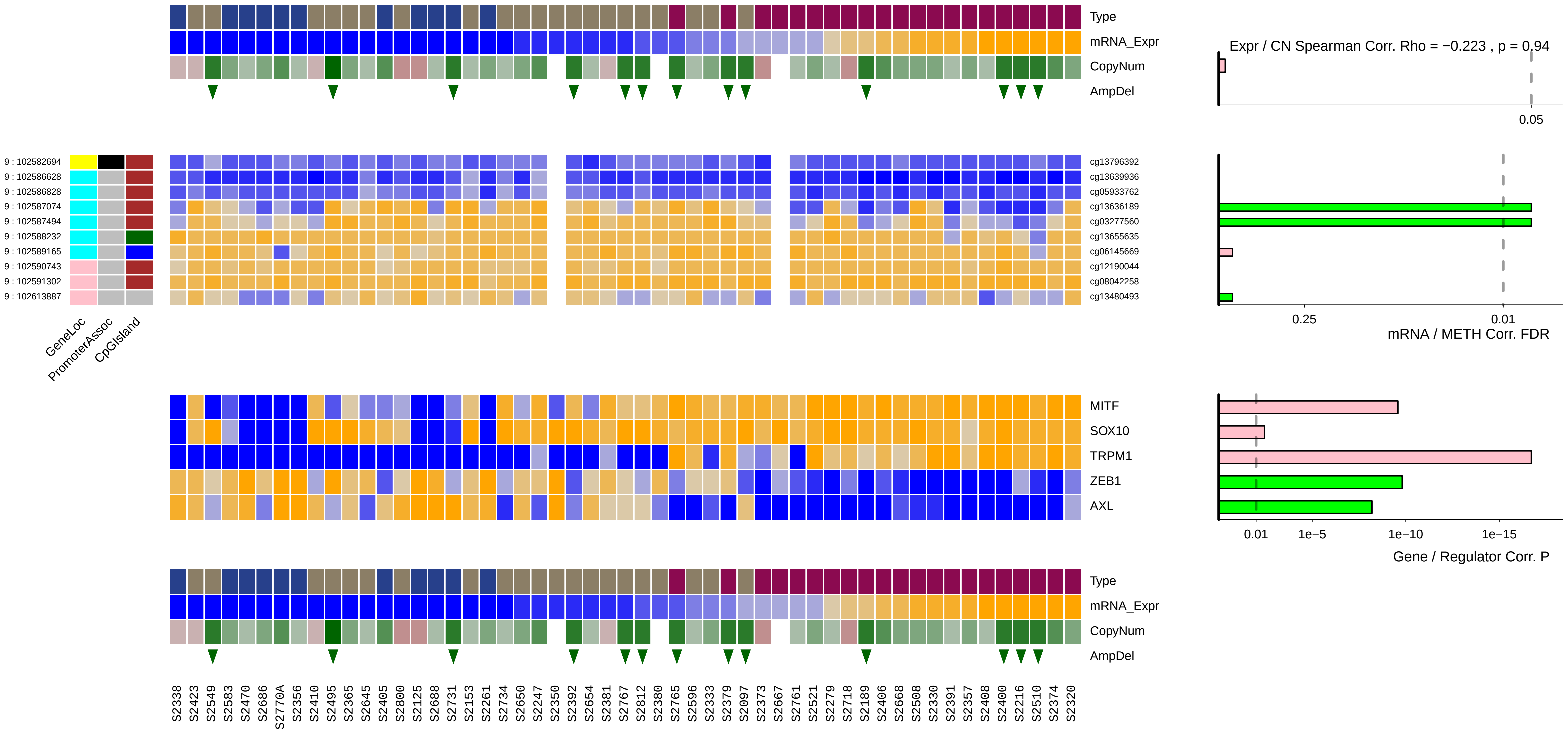
Type
mRNA_Expr
CopyNum
AmpDel
Expr / CN Spearman Corr. Rho = −0.223 , p = 0.94
0.05
9 : 102582694
9 : 102586628
9 : 102586828
9 : 102587074
9 : 102587494
9 : 102588232
9 : 102589165
9 : 102590743
9 : 102591302
9 : 102613887
GeneLoc
PromoterAssoc
CpGIsland
cg13796392
cg13639936
cg05933762
cg13636189
cg03277560
cg13655635
cg06145669
cg12190044
cg08042258
cg13480493
0.25
0.01
mRNA / METH Corr. FDR
MITF
SOX10
TRPM1
ZEB1
AXL
0.01
1e−5
1e−10
1e−15
Gene / Regulator Corr. P
S2338 S2423 S2549 S2583 S2470 S2686 S2770A S2356 S2410 S2495 S2365 S2645 S2405 S2800 S2125 S2688 S2731 S2153 S2261 S2734 S2650 S2247 S2350 S2392 S2654 S2381 S2767 S2812 S2380 S2765 S2596 S2333 S2379 S2097 S2373 S2667 S2761 S2521 S2279 S2718 S2189 S2406 S2668 S2508 S2330 S2391 S2357 S2408 S2400 S2216 S2510 S2374 S2320

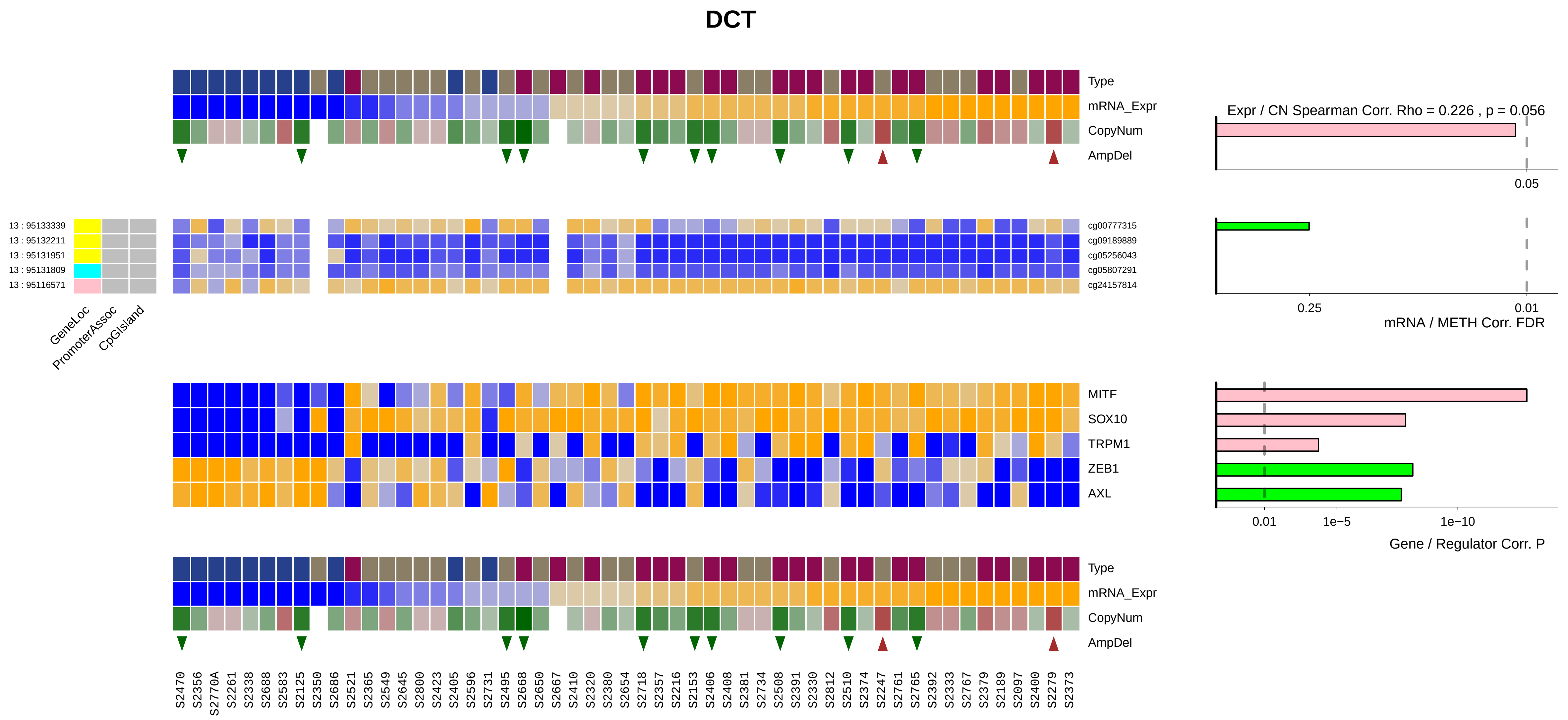
DCT
Type
mRNA_Expr
CopyNum
AmpDel
Expr / CN Spearman Corr. Rho = 0.226 , p = 0.056
0.05
13 : 95133339
13 : 95132211
13 : 95131951
13 : 95131809
13 : 95116571
cg00777315
cg09189889
cg05256043
cg05807291
cg24157814
0.25
0.01
mRNA / METH Corr. FDR
GeneLoc
PromoterAssoc
CpGIsland
MITF
SOX10
TRPM1
ZEB1
AXL
0.01
1e−5
1e−10
Gene / Regulator Corr. P
S2470 S2356 S2770A S2261 S2338 S2688 S2583 S2125 S2350 S2686 S2521 S2365 S2549 S2645 S2800 S2423 S2405 S2596 S2731 S2495 S2668 S2650 S2667 S2410 S2320 S2380 S2654 S2718 S2357 S2216 S2153 S2406 S2408 S2381 S2734 S2508 S2391 S2330 S2812 S2510 S2374 S2247 S2761 S2765 S2392 S2333 S2767 S2379 S2189 S2097 S2400 S2279 S2373

SLC45A2

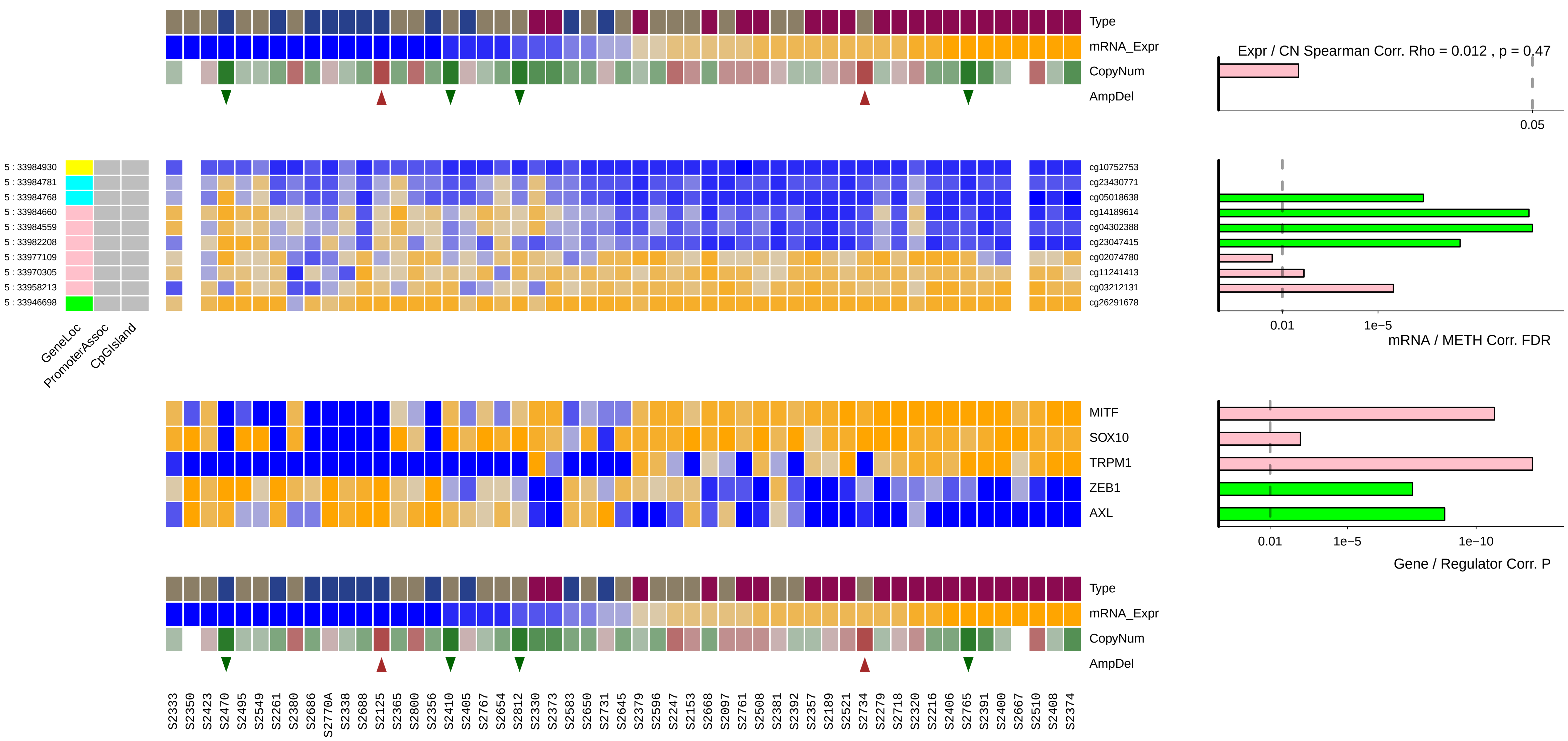

Type
mRNA_Expr
CopyNum
AmpDel
Expr / CN Spearman Corr. Rho = 0.012 , p = 0.47
0.05
5 : 33984930
5 : 33984781
5 : 33984768
5 : 33984660
5 : 33984559
5 : 33982208
5 : 33977109
5 : 33970305
5 : 33958213
5 : 33946698
GeneLoc
PromoterAssoc
CpGIsland
cg10752753
cg23430771
cg05018638
cg14189614
cg04302388
cg23047415
cg02074780
cg11241413
cg03212131
cg26291678
0.01
1e−5
mRNA / METH Corr. FDR
MITF
SOX10
TRPM1
ZEB1
AXL
0.01
1e−5
1e−10
Gene / Regulator Corr. P
Type
mRNA_Expr
CopyNum
AmpDel
S2333 S2350 S2423 S2470 S2495 S2549 S2261 S2380 S2686 S2770A S2338 S2688 S2125 S2365 S2800 S2356 S2410 S2405 S2767 S2654 S2812 S2330 S2373 S2583 S2650 S2731 S2645 S2379 S2596 S2247 S2153 S2668 S2097 S2761 S2508 S2381 S2392 S2357 S2189 S2521 S2734 S2279 S2718 S2320 S2216 S2406 S2765 S2391 S2400 S2667 S2510 S2408 S2374

PLP1

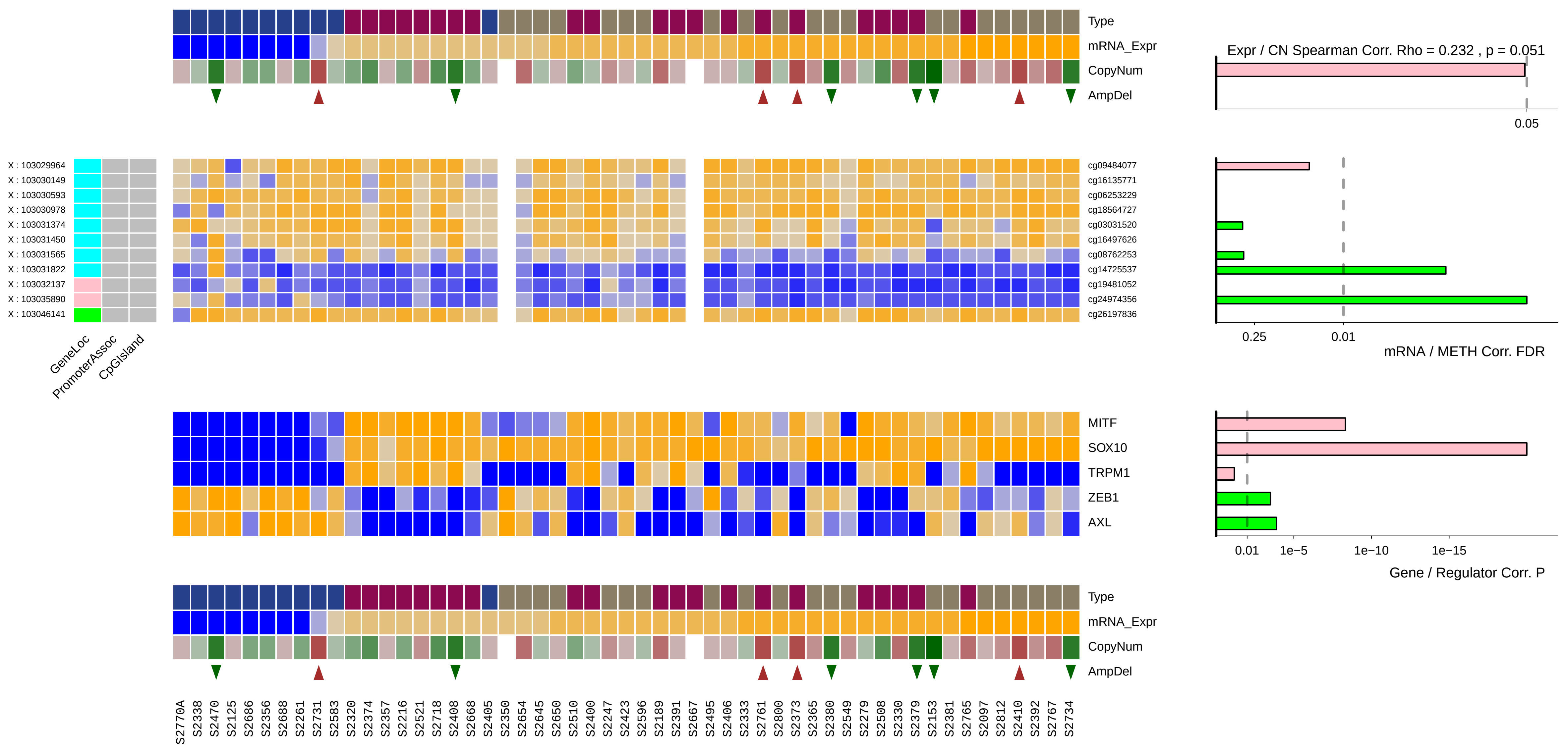
Type
mRNA_Expr
CopyNum
AmpDel
Expr / CN Spearman Corr. Rho = 0.232 , p = 0.051
0.05
X : 103029964
X : 103030149
X : 103030593
X : 103030978
X : 103031374
X : 103031450
X : 103031565
X : 103031822
X : 103032137
X : 103035890
X : 103046141
GeneLoc
PromoterAssoc
CpGIsland
cg09484077
cg16135771
cg06253229
cg18564727
cg03031520
cg16497626
cg08762253
cg14725537
cg19481052
cg24974356
cg26197836
0.25
0.01
mRNA / METH Corr. FDR
MITF
SOX10
TRPM1
ZEB1
AXL
0.01
1e−5
1e−10
1e−15
Gene / Regulator Corr. P
S2770A S2338 S2470 S2125 S2686 S2356 S2688 S2261 S2731 S2583 S2320 S2374 S2357 S2216 S2521 S2718 S2408 S2668 S2405 S2350 S2654 S2645 S2650 S2510 S2400 S2247 S2423 S2596 S2189 S2391 S2667 S2495 S2406 S2333 S2761 S2800 S2373 S2365 S2380 S2549 S2279 S2508 S2330 S2379 S2153 S2381 S2765 S2097 S2812 S2410 S2392 S2767 S2734

PLEKHH1

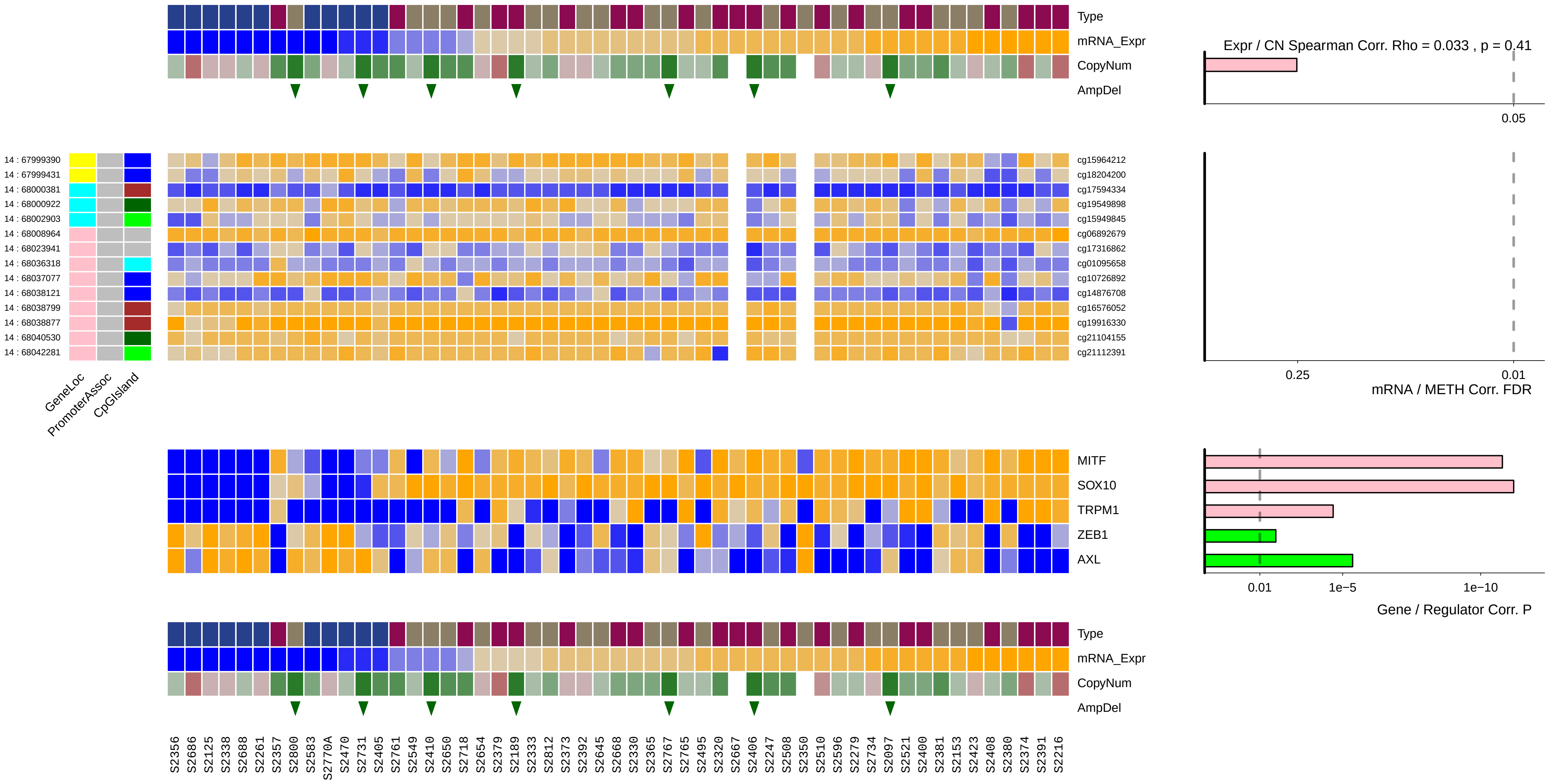
Type
mRNA_Expr
CopyNum
AmpDel
Expr / CN Spearman Corr. Rho = 0.033 , p = 0.41
0.05
14 : 67999390
14 : 67999431
14 : 68000381
14 : 68000922
14 : 68002903
14 : 68008964
14 : 68023941
14 : 68036318
14 : 68037077
14 : 68038121
14 : 68038799
14 : 68038877
14 : 68040530
14 : 68042281
cg15964212
cg18204200
cg17594334
cg19549898
cg15949845
cg06892679
cg17316862
cg01095658
cg10726892
cg14876708
cg16576052
cg19916330
cg21104155
cg21112391
0.25
0.01
mRNA / METH Corr. FDR
GeneLoc
PromoterAssoc
CpGIsland
MITF
SOX10
TRPM1
ZEB1
AXL
0.01
1e−5
1e−10
Gene / Regulator Corr. P
Type
mRNA_Expr
CopyNum
AmpDel
S2356 S2686 S2125 S2338 S2688 S2261 S2357 S2800 S2583 S2770A S2470 S2731 S2405 S2761 S2549 S2410 S2650 S2718 S2654 S2379 S2189 S2333 S2812 S2373 S2392 S2645 S2668 S2330 S2365 S2767 S2765 S2495 S2320 S2667 S2406 S2247 S2508 S2350 S2510 S2596 S2279 S2734 S2097 S2521 S2400 S2381 S2153 S2423 S2408 S2380 S2374 S2391 S2216

ASAH1

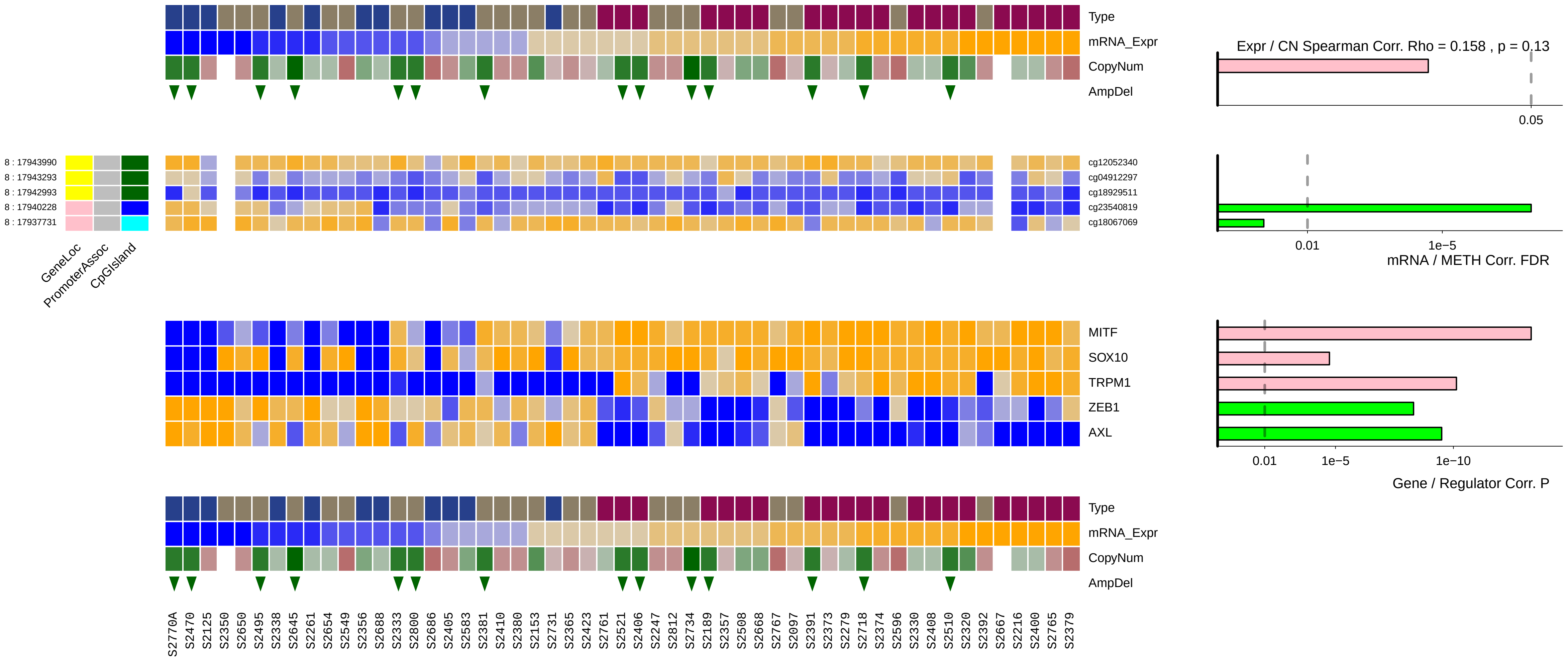
Type
mRNA_Expr
CopyNum
AmpDel
Expr / CN Spearman Corr. Rho = 0.158 , p = 0.13
0.05
8 : 17943990
8 : 17943293
8 : 17942993
8 : 17940228
8 : 17937731
GeneLoc
PromoterAssoc
CpGIsland
cg12052340
cg04912297
cg18929511
cg23540819
cg18067069
0.01
1e−5
mRNA / METH Corr. FDR
MITF
SOX10
TRPM1
ZEB1
AXL
0.01
1e−5
1e−10
Gene / Regulator Corr. P
Type
mRNA_Expr
CopyNum
AmpDel
S2770A S2470 S2125 S2350 S2650 S2495 S2338 S2645 S2261 S2654 S2549 S2356 S2688 S2333 S2800 S2686 S2405 S2583 S2381 S2410 S2380 S2153 S2731 S2365 S2423 S2761 S2521 S2406 S2247 S2812 S2734 S2189 S2357 S2508 S2668 S2767 S2097 S2391 S2373 S2279 S2718 S2374 S2596 S2330 S2408 S2510 S2320 S2392 S2667 S2216 S2400 S2765 S2379

SLC19A2

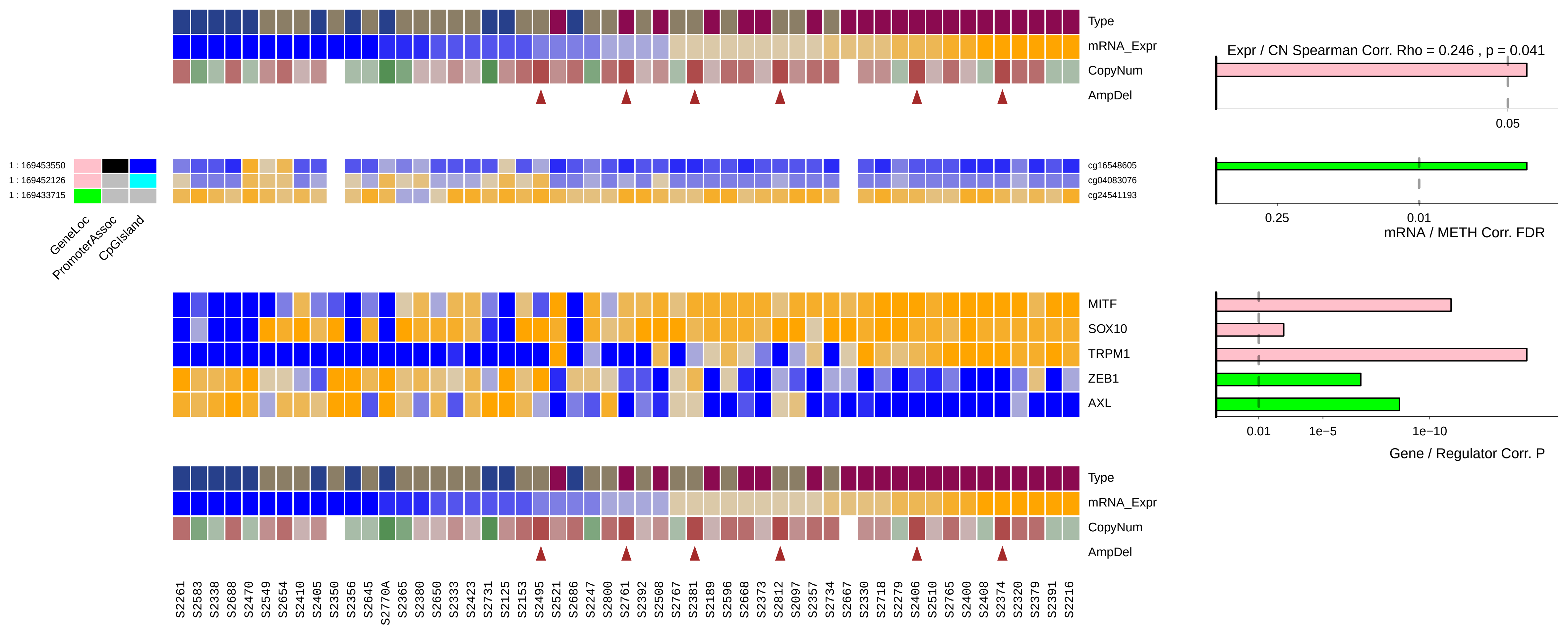
Type
mRNA_Expr
CopyNum
AmpDel
Expr / CN Spearman Corr. Rho = 0.246 , p = 0.041
0.05
1 : 169453550
1 : 169452126
1 : 169433715
GeneLoc
PromoterAssoc
CpGIsland
cg16548605
cg04083076
cg24541193
0.25
0.01
mRNA / METH Corr. FDR
MITF
SOX10
TRPM1
ZEB1
AXL
0.01
1e−5
1e−10
Gene / Regulator Corr. P
S2261 S2583 S2338 S2688 S2470 S2549 S2654 S2410 S2405 S2350 S2356 S2645 S2770A S2365 S2380 S2650 S2333 S2423 S2731 S2125 S2153 S2495 S2521 S2686 S2247 S2800 S2761 S2392 S2508 S2767 S2381 S2189 S2596 S2668 S2373 S2812 S2097 S2357 S2734 S2667 S2330 S2718 S2279 S2406 S2510 S2765 S2400 S2408 S2374 S2320 S2379 S2391 S2216

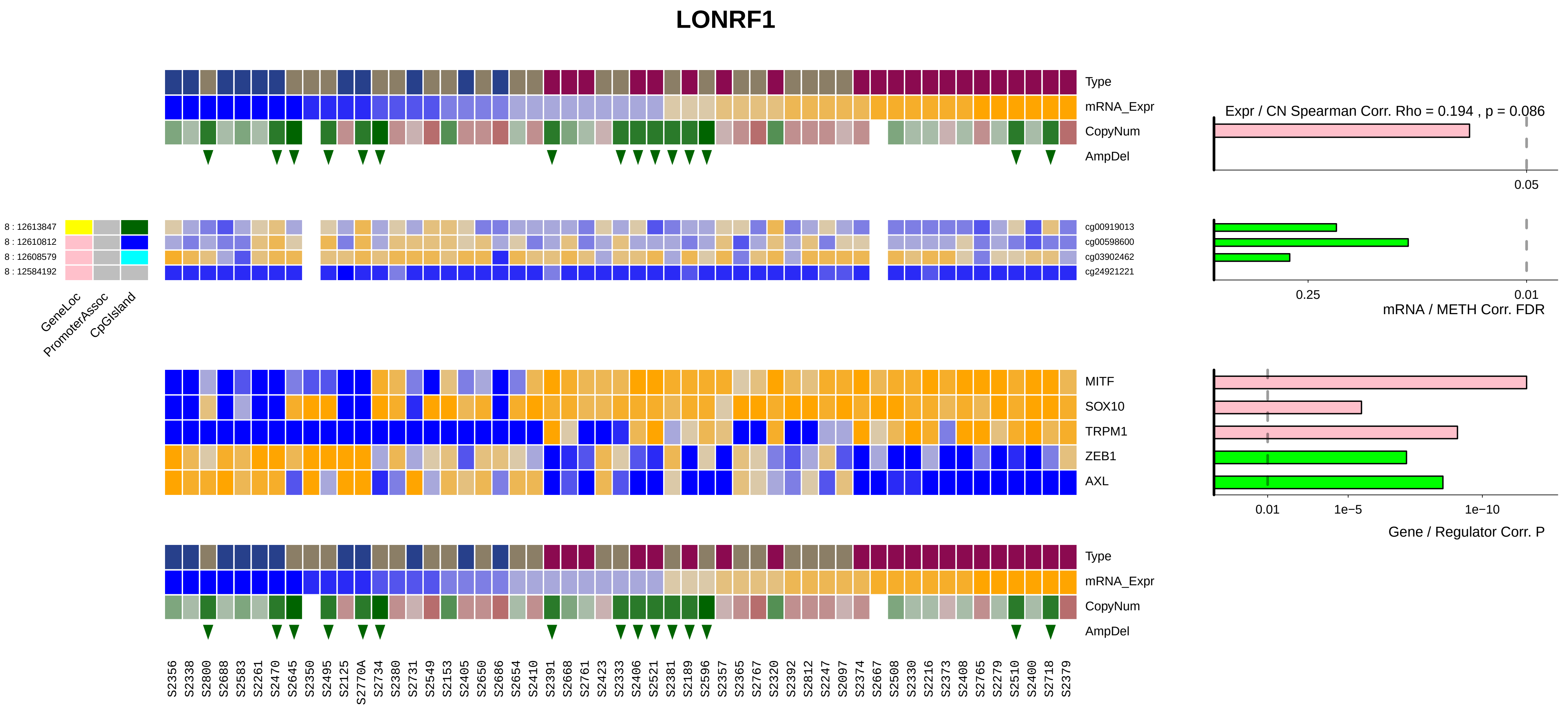

LONRF1
Type
mRNA_Expr
CopyNum
AmpDel
Expr / CN Spearman Corr. Rho = 0.194 , p = 0.086
0.05
8 : 12613847
8 : 12610812
8 : 12608579
8 : 12584192
cg00919013
cg00598600
cg03902462
cg24921221
0.25
0.01
mRNA / METH Corr. FDR
GeneLoc
PromoterAssoc
CpGIsland
MITF
SOX10
TRPM1
ZEB1
AXL
0.01
1e−5
1e−10
Gene / Regulator Corr. P
Type
mRNA_Expr
CopyNum
AmpDel
S2356 S2338 S2800 S2688 S2583 S2261 S2470 S2645 S2350 S2495 S2125 S2770A S2734 S2380 S2731 S2549 S2153 S2405 S2650 S2686 S2654 S2410 S2391 S2668 S2761 S2423 S2333 S2406 S2521 S2381 S2189 S2596 S2357 S2365 S2767 S2320 S2392 S2812 S2247 S2097 S2374 S2667 S2508 S2330 S2216 S2373 S2408 S2765 S2279 S2510 S2400 S2718 S2379

ZFYVE16

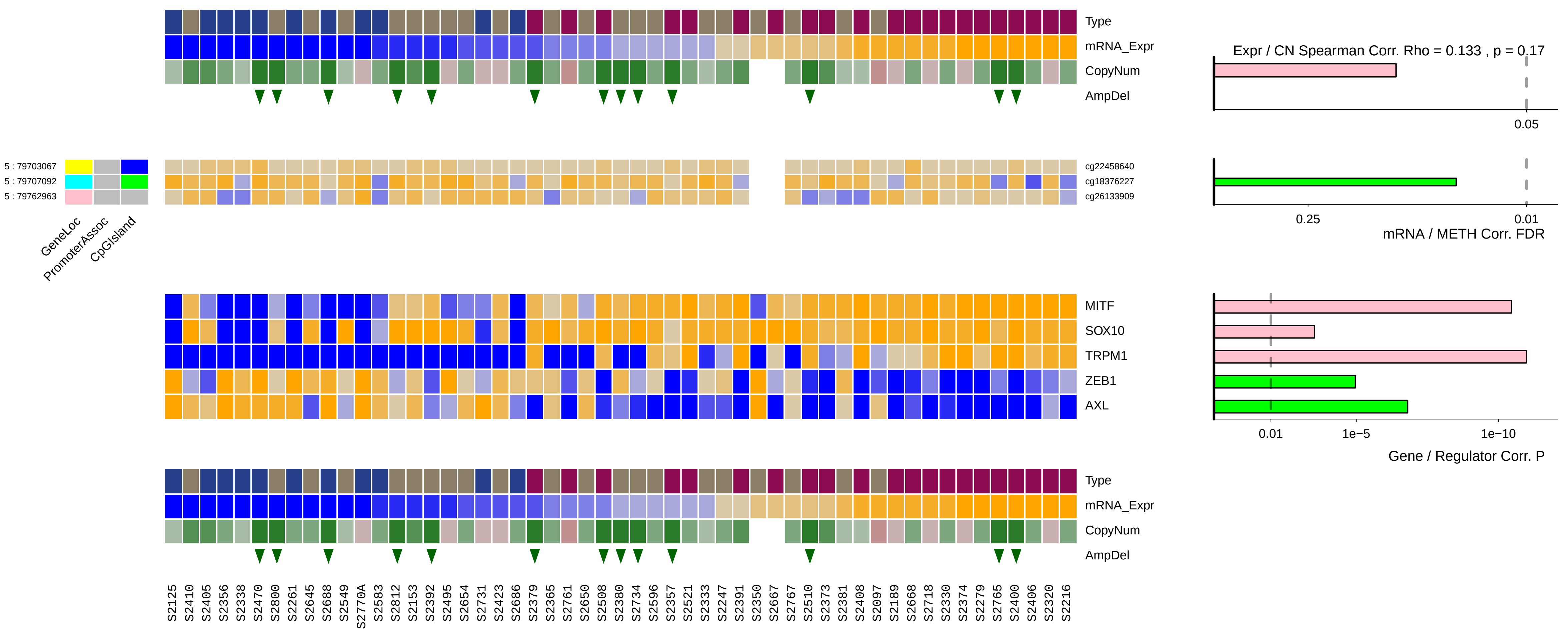
Type
mRNA_Expr
CopyNum
AmpDel
Expr / CN Spearman Corr. Rho = 0.133 , p = 0.17
0.05
5 : 79703067
5 : 79707092
5 : 79762963
GeneLoc
PromoterAssoc
CpGIsland
cg22458640
cg18376227
cg26133909
0.25
0.01
mRNA / METH Corr. FDR
MITF
SOX10
TRPM1
ZEB1
AXL
0.01
1e−5
1e−10
Gene / Regulator Corr. P
S2125 S2410 S2405 S2356 S2338 S2470 S2800 S2261 S2645 S2688 S2549 S2770A S2583 S2812 S2153 S2392 S2495 S2654 S2731 S2423 S2686 S2379 S2365 S2761 S2650 S2508 S2380 S2734 S2596 S2357 S2521 S2333 S2247 S2391 S2350 S2667 S2767 S2510 S2373 S2381 S2408 S2097 S2189 S2668 S2718 S2330 S2374 S2279 S2765 S2400 S2406 S2320 S2216

GALNT3

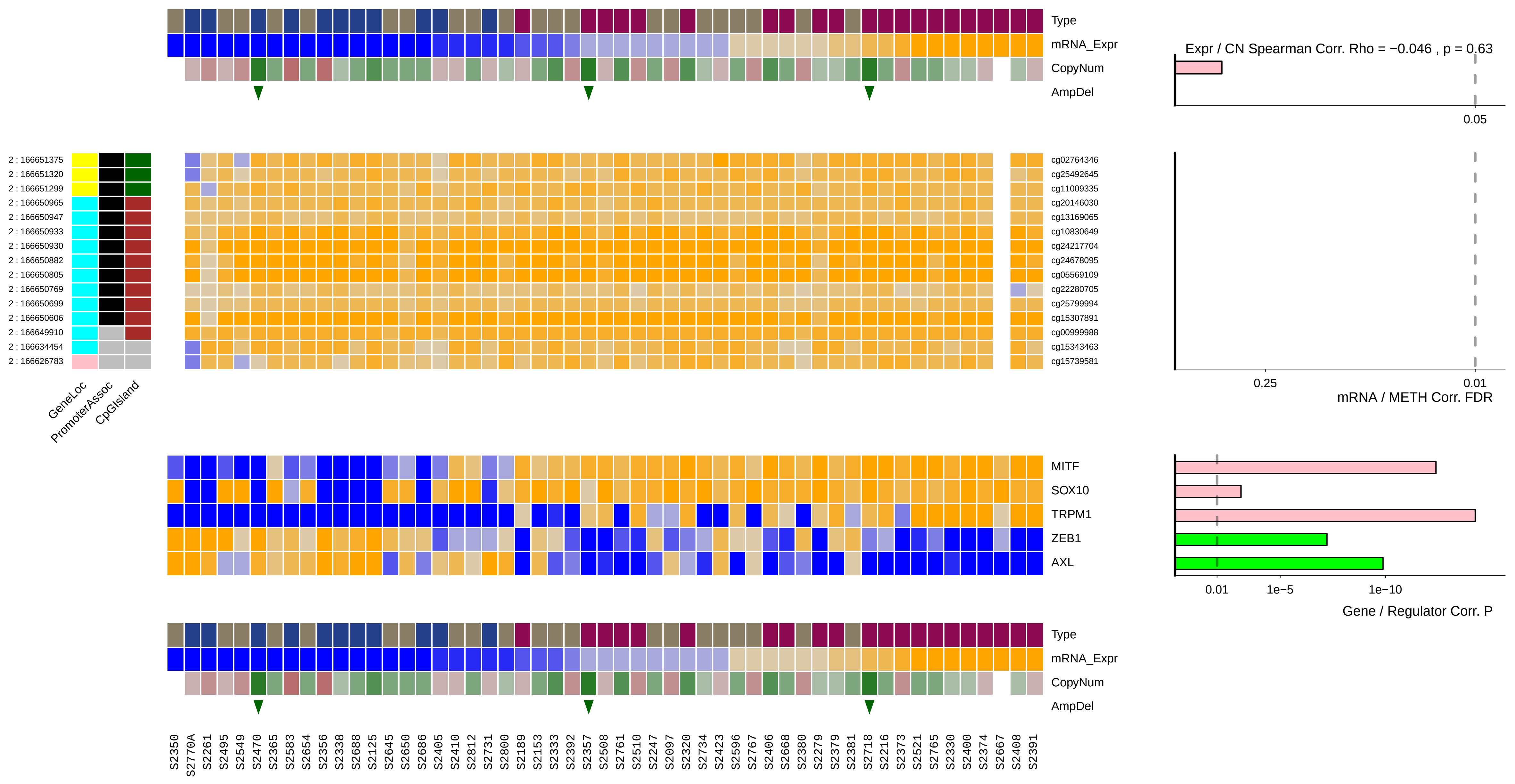
Type
mRNA_Expr
CopyNum
AmpDel
Expr / CN Spearman Corr. Rho = −0.046 , p = 0.63
0.05
2 : 166651375
2 : 166651320
2 : 166651299
2 : 166650965
2 : 166650947
2 : 166650933
2 : 166650930
2 : 166650882
2 : 166650805
2 : 166650769
2 : 166650699
2 : 166650606
2 : 166649910
2 : 166634454
2 : 166626783
cg02764346
cg25492645
cg11009335
cg20146030
cg13169065
cg10830649
cg24217704
cg24678095
cg05569109
cg22280705
cg25799994
cg15307891
cg00999988
cg15343463
cg15739581
GeneLoc
PromoterAssoc
CpGIsland
0.25
0.01
mRNA / METH Corr. FDR
MITF
SOX10
TRPM1
ZEB1
AXL
0.01
1e−5
1e−10
Gene / Regulator Corr. P
Type
mRNA_Expr
CopyNum
AmpDel
S2350 S2770A S2261 S2495 S2549 S2470 S2365 S2583 S2654 S2356 S2338 S2688 S2125 S2645 S2650 S2686 S2405 S2410 S2812 S2731 S2800 S2189 S2153 S2333 S2392 S2357 S2508 S2761 S2510 S2247 S2097 S2320 S2734 S2423 S2596 S2767 S2406 S2668 S2380 S2279 S2379 S2381 S2718 S2216 S2373 S2521 S2765 S2330 S2400 S2374 S2667 S2408 S2391

APOE

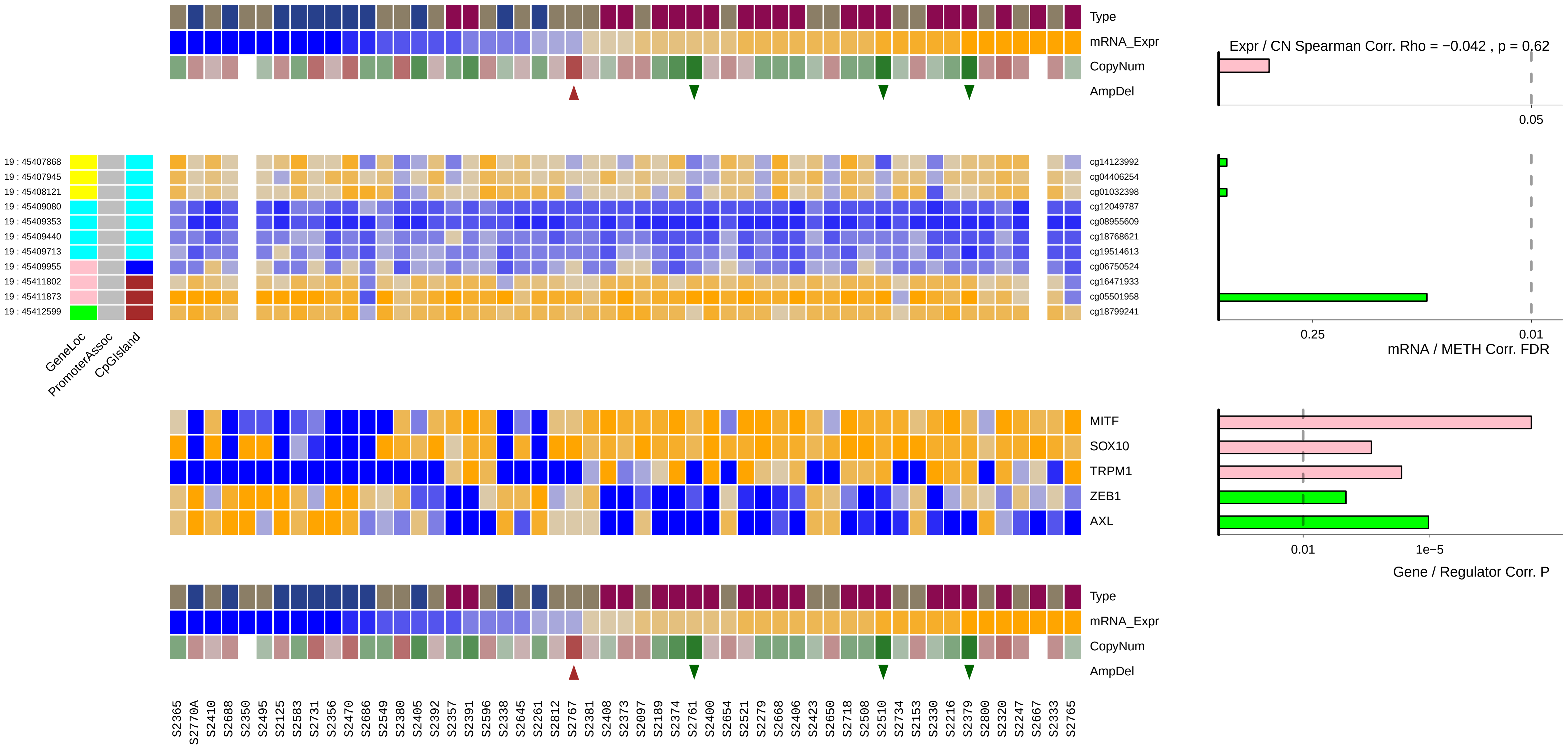

Type
mRNA_Expr
CopyNum
AmpDel
Expr / CN Spearman Corr. Rho = −0.042 , p = 0.62
0.05
19 : 45407868
19 : 45407945
19 : 45408121
19 : 45409080
19 : 45409353
19 : 45409440
19 : 45409713
19 : 45409955
19 : 45411802
19 : 45411873
19 : 45412599
cg14123992
cg04406254
cg01032398
cg12049787
cg08955609
cg18768621
cg19514613
cg06750524
cg16471933
cg05501958
cg18799241
GeneLoc
PromoterAssoc
CpGIsland
0.25
0.01
mRNA / METH Corr. FDR
MITF
SOX10
TRPM1
ZEB1
AXL
0.01
1e−5
Gene / Regulator Corr. P
Type
mRNA_Expr
CopyNum
AmpDel
S2365 S2770A S2410 S2688 S2350 S2495 S2125 S2583 S2731 S2356 S2470 S2686 S2549 S2380 S2405 S2392 S2357 S2391 S2596 S2338 S2645 S2261 S2812 S2767 S2381 S2408 S2373 S2097 S2189 S2374 S2761 S2400 S2654 S2521 S2279 S2668 S2406 S2423 S2650 S2718 S2508 S2510 S2734 S2153 S2330 S2216 S2379 S2800 S2320 S2247 S2667 S2333 S2765

IL6R

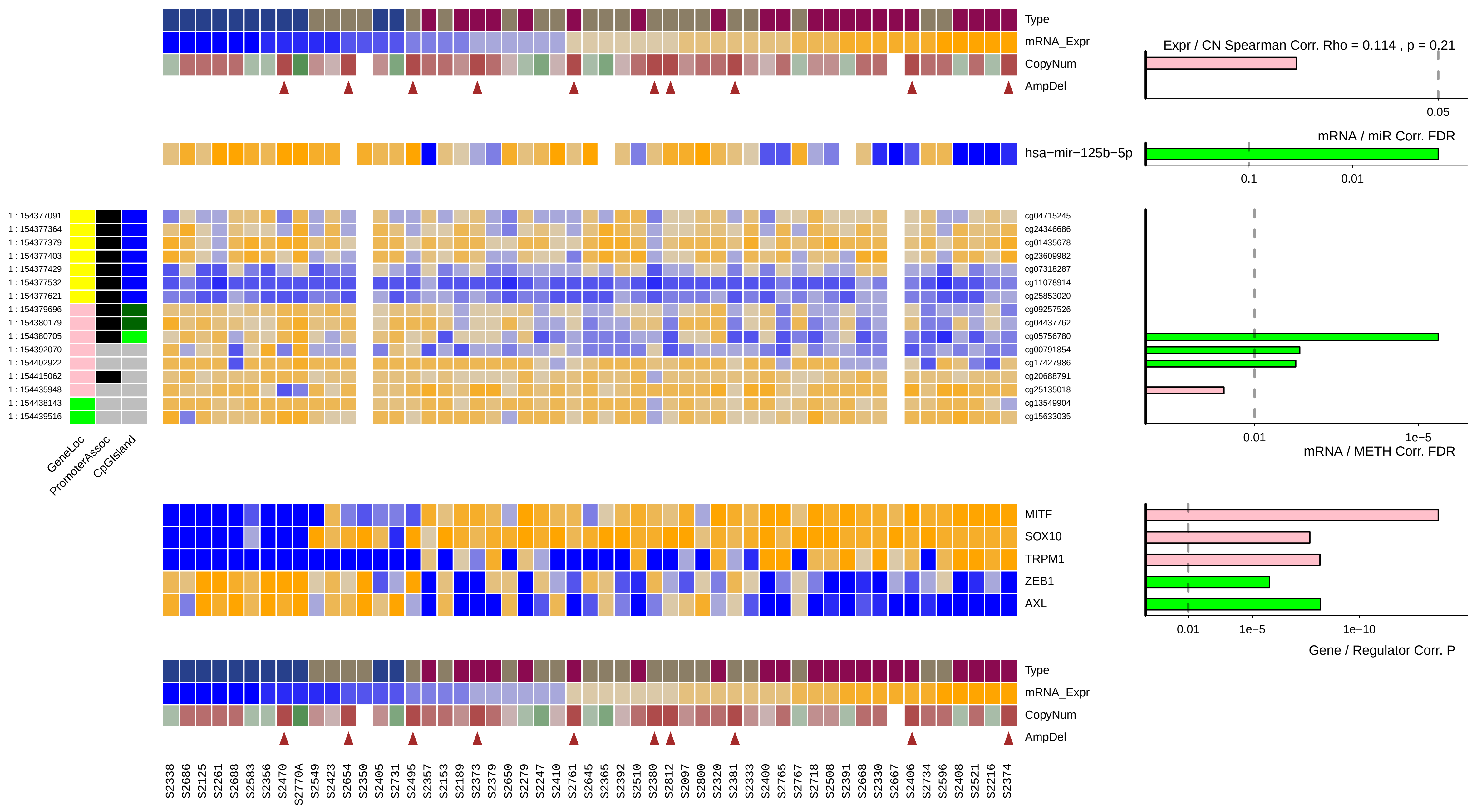

Type
mRNA_Expr
CopyNum
AmpDel
Expr / CN Spearman Corr. Rho = 0.114 , p = 0.21
0.05
hsa−mir−125b−5p
mRNA / miR Corr. FDR
0.1
0.01
1 : 154377091
1 : 154377364
1 : 154377379
1 : 154377403
1 : 154377429
1 : 154377532
1 : 154377621
1 : 154379696
1 : 154380179
1 : 154380705
1 : 154392070
1 : 154402922
1 : 154415062
1 : 154435948
1 : 154438143
1 : 154439516
GeneLoc
PromoterAssoc
CpGIsland
cg04715245
cg24346686
cg01435678
cg23609982
cg07318287
cg11078914
cg25853020
cg09257526
cg04437762
cg05756780
cg00791854
cg17427986
cg20688791
cg25135018
cg13549904
cg15633035
0.01
1e−5
mRNA / METH Corr. FDR
MITF
SOX10
TRPM1
ZEB1
AXL
0.01
1e−5
1e−10
Gene / Regulator Corr. P
S2338 S2686 S2125 S2261 S2688 S2583 S2356 S2470 S2770A S2549 S2423 S2654 S2350 S2405 S2731 S2495 S2357 S2153 S2189 S2373 S2379 S2650 S2279 S2247 S2410 S2761 S2645 S2365 S2392 S2510 S2380 S2812 S2097 S2800 S2320 S2381 S2333 S2400 S2765 S2767 S2718 S2508 S2391 S2668 S2330 S2667 S2406 S2734 S2596 S2408 S2521 S2216 S2374

HES6

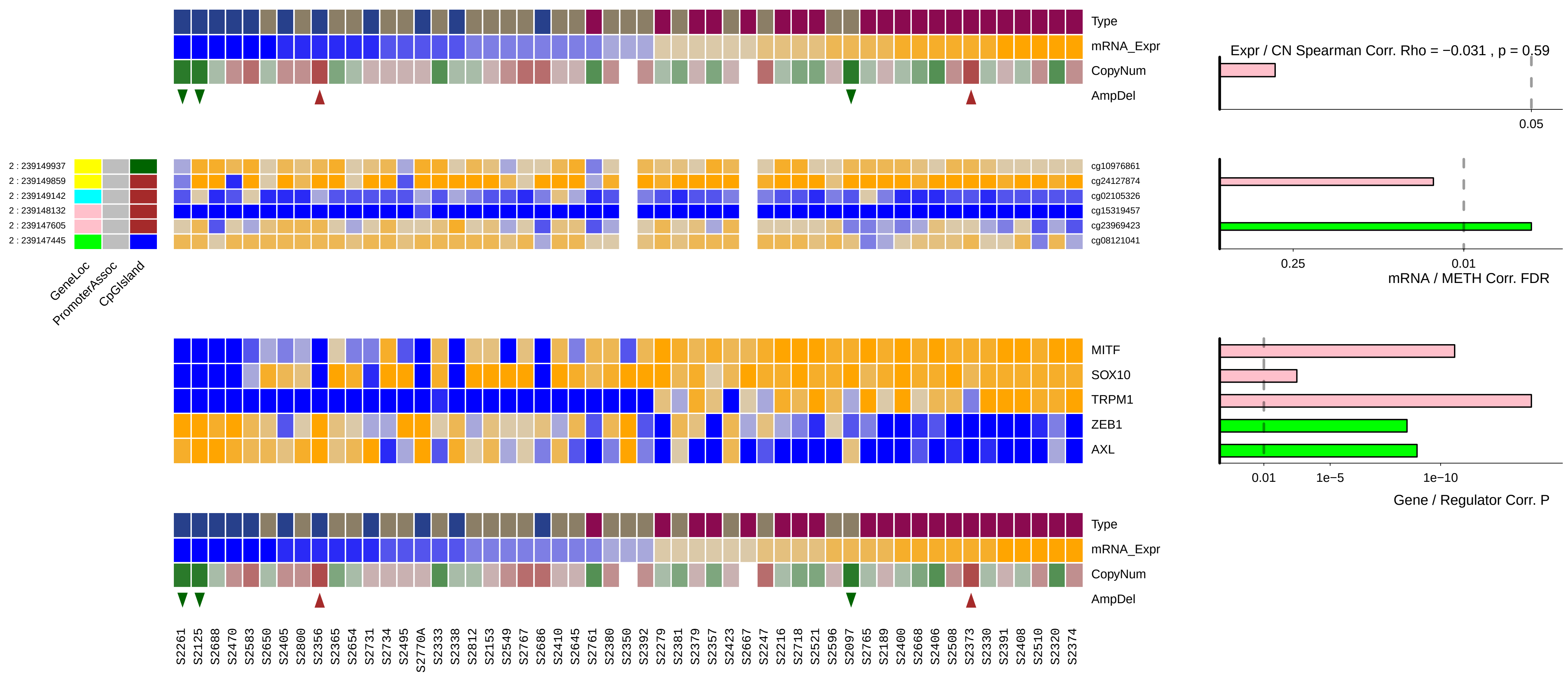
Type
mRNA_Expr
CopyNum
AmpDel
Expr / CN Spearman Corr. Rho = −0.031 , p = 0.59
0.05
2 : 239149937
2 : 239149859
2 : 239149142
2 : 239148132
2 : 239147605
2 : 239147445
GeneLoc
PromoterAssoc
CpGIsland
cg10976861
cg24127874
cg02105326
cg15319457
cg23969423
cg08121041
0.25
0.01
mRNA / METH Corr. FDR
MITF
SOX10
TRPM1
ZEB1
AXL
0.01
1e−5
1e−10
Gene / Regulator Corr. P
Type
mRNA_Expr
CopyNum
AmpDel
S2261 S2125 S2688 S2470 S2583 S2650 S2405 S2800 S2356 S2365 S2654 S2731 S2734 S2495 S2770A S2333 S2338 S2812 S2153 S2549 S2767 S2686 S2410 S2645 S2761 S2380 S2350 S2392 S2279 S2381 S2379 S2357 S2423 S2667 S2247 S2216 S2718 S2521 S2596 S2097 S2765 S2189 S2400 S2668 S2406 S2508 S2373 S2330 S2391 S2408 S2510 S2320 S2374

HPS4

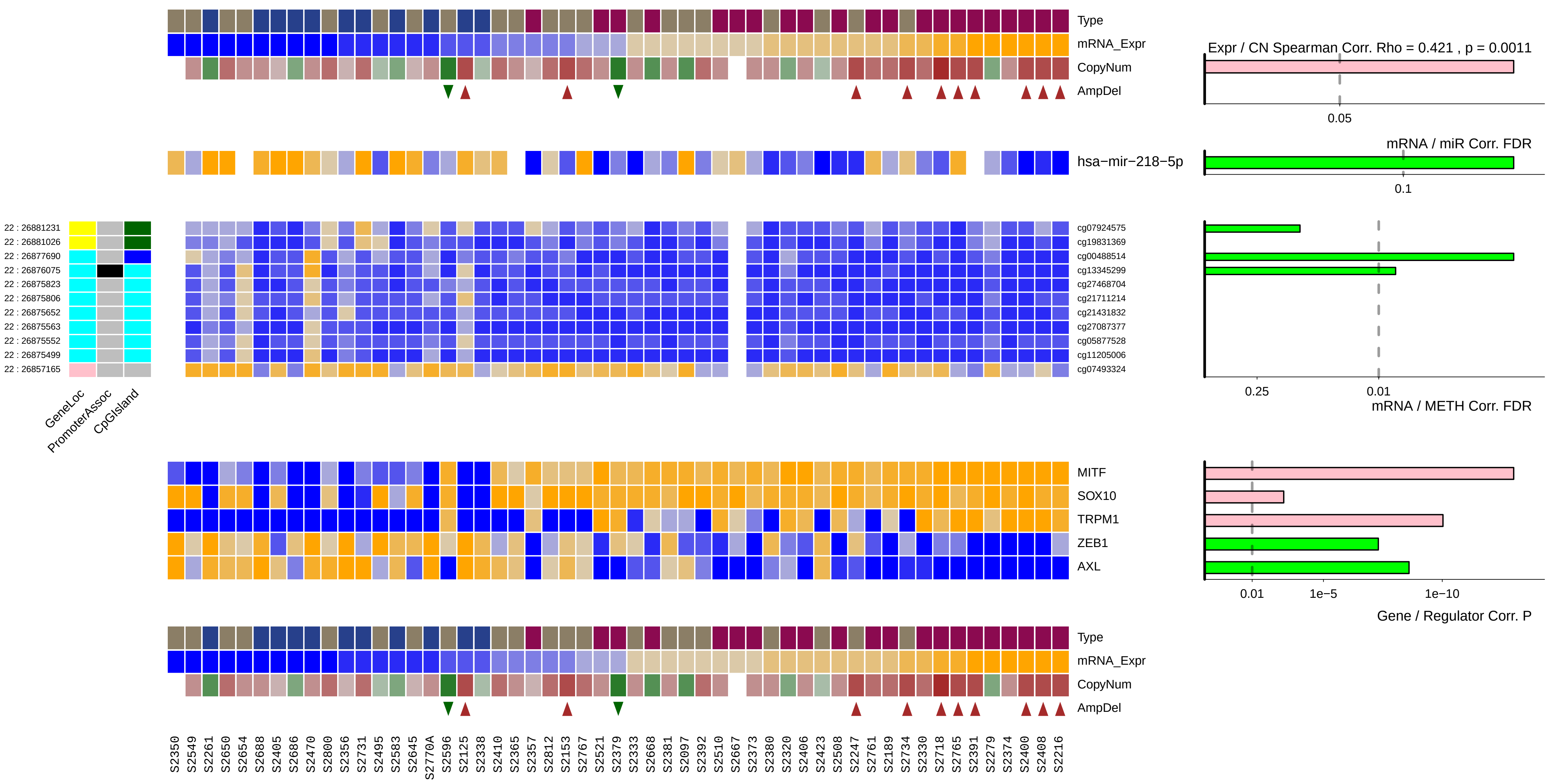

Type
mRNA_Expr
CopyNum
AmpDel
Expr / CN Spearman Corr. Rho = 0.421 , p = 0.0011
0.05
hsa−mir−218−5p
mRNA / miR Corr. FDR
0.1
22 : 26881231
22 : 26881026
22 : 26877690
22 : 26876075
22 : 26875823
22 : 26875806
22 : 26875652
22 : 26875563
22 : 26875552
22 : 26875499
22 : 26857165
GeneLoc
PromoterAssoc
CpGIsland
cg07924575
cg19831369
cg00488514
cg13345299
cg27468704
cg21711214
cg21431832
cg27087377
cg05877528
cg11205006
cg07493324
0.25
0.01
mRNA / METH Corr. FDR
MITF
SOX10
TRPM1
ZEB1
AXL
0.01
1e−5
1e−10
Gene / Regulator Corr. P
Type
mRNA_Expr
CopyNum
AmpDel
S2350 S2549 S2261 S2650 S2654 S2688 S2405 S2686 S2470 S2800 S2356 S2731 S2495 S2583 S2645 S2770A S2596 S2125 S2338 S2410 S2365 S2357 S2812 S2153 S2767 S2521 S2379 S2333 S2668 S2381 S2097 S2392 S2510 S2667 S2373 S2380 S2320 S2406 S2423 S2508 S2247 S2761 S2189 S2734 S2330 S2718 S2765 S2391 S2279 S2374 S2400 S2408 S2216

PHACTR1

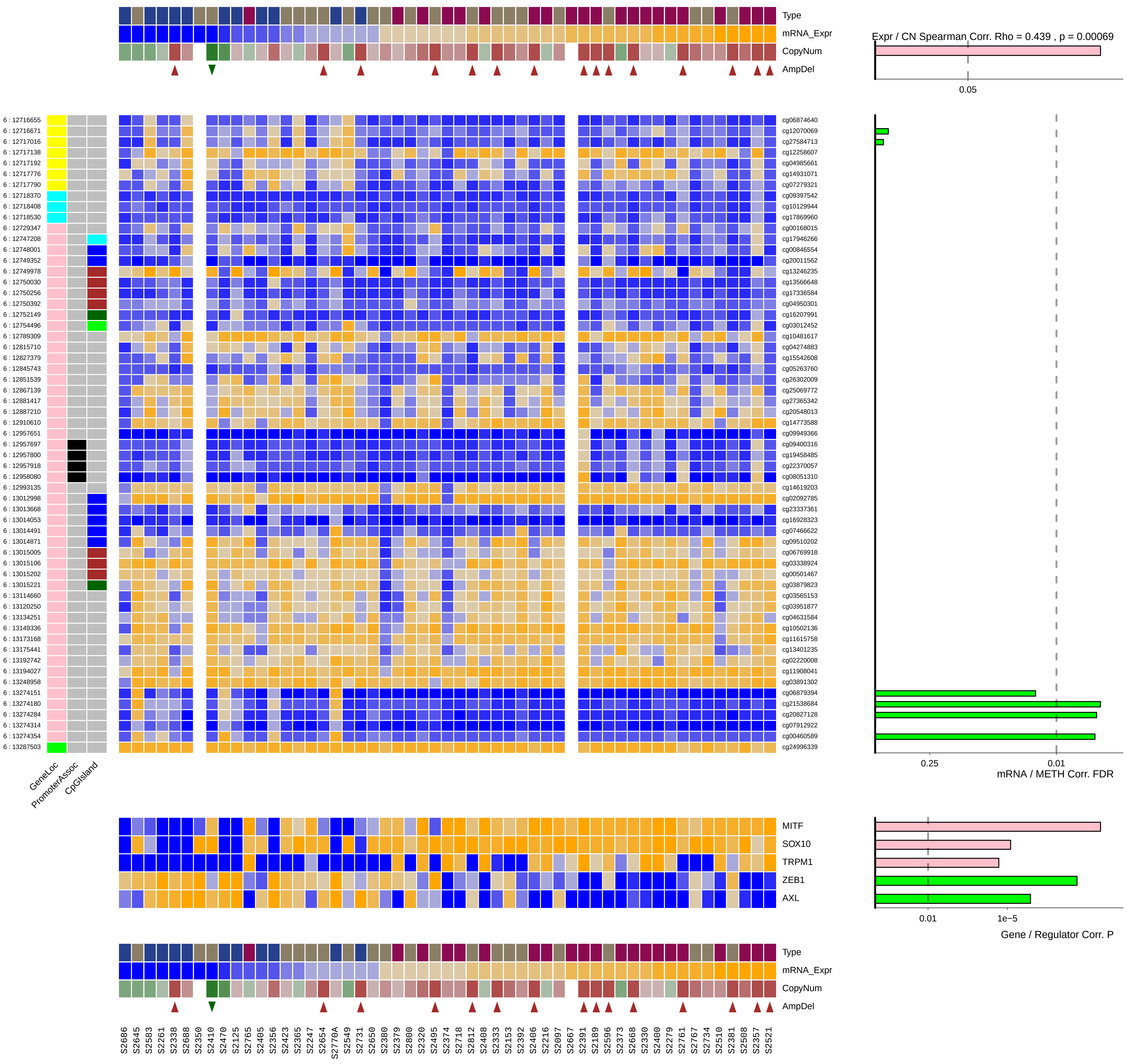
Type
mRNA_Expr
CopyNum
AmpDel
Expr / CN Spearman Corr. Rho = 0.439 , p = 0.00069
0.05
6 : 12716655
6 : 12716671
6 : 12717016
6 : 12717138
6 : 12717192
6 : 12717776
6 : 12717790
6 : 12718370
6 : 12718408
6 : 12718530
6 : 12729347
6 : 12747208
6 : 12748001
6 : 12749352
6 : 12749978
6 : 12750030
6 : 12750256
6 : 12750392
6 : 12752149
6 : 12754496
6 : 12789309
6 : 12815710
6 : 12827379
6 : 12845743
6 : 12851539
6 : 12867139
6 : 12881417
6 : 12887210
6 : 12910610
6 : 12957651
6 : 12957697
6 : 12957800
6 : 12957918
6 : 12958080
6 : 12993135
6 : 13012998
6 : 13013668
6 : 13014053
6 : 13014491
6 : 13014871
6 : 13015005
6 : 13015106
6 : 13015202
6 : 13015221
6 : 13114660
6 : 13120250
6 : 13134251
6 : 13149336
6 : 13173168
6 : 13175441
6 : 13192742
6 : 13194027
6 : 13248958
6 : 13274151
6 : 13274180
6 : 13274284
6 : 13274314
6 : 13274354
6 : 13287503
cg06874640
cg12070069
cg27584713
cg12258607
cg04985661
cg14931071
cg07279321
cg09397542
cg10129944
cg17869960
cg00168015
cg17946266
cg00846554
cg20011562
cg13246235
cg13566648
cg17336584
cg04950301
cg16207991
cg03012452
cg10481617
cg04274883
cg15542608
cg05263760
cg26302009
cg25069772
cg27365342
cg20548013
cg14773588
cg09949366
cg09400316
cg19458485
cg22370057
cg08051310
cg14619203
cg02092785
cg23337361
cg16928323
cg07466622
cg09510202
cg06769918
cg03338924
cg00501467
cg03879823
cg03565153
cg03951877
cg04631584
cg10502136
cg11615758
cg13401235
cg02220008
cg11908041
cg03891302
cg06879394
cg21538684
cg20827128
cg07912922
cg00460589
cg24996339
0.25
0.01
mRNA / METH Corr. FDR
GeneLoc
PromoterAssoc
CpGIsland
MITF
SOX10
TRPM1
ZEB1
AXL
0.01
1e−5
Gene / Regulator Corr. P
Type
mRNA_Expr
CopyNum
AmpDel
S2686
S2645
S2583
S2261
S2338
S2688
S2350
S2410
S2470
S2125
S2765
S2405
S2356
S2423
S2365
S2247
S2654
S2770A
S2549
S2731
S2650
S2380
S2379
S2800
S2320
S2495
S2374
S2718
S2812
S2408
S2333
S2153
S2392
S2406
S2216
S2097
S2667
S2391
S2189
S2596
S2373
S2668
S2330
S2400
S2279
S2761
S2767
S2734
S2510
S2381
S2508
S2357
S2521

SOX6

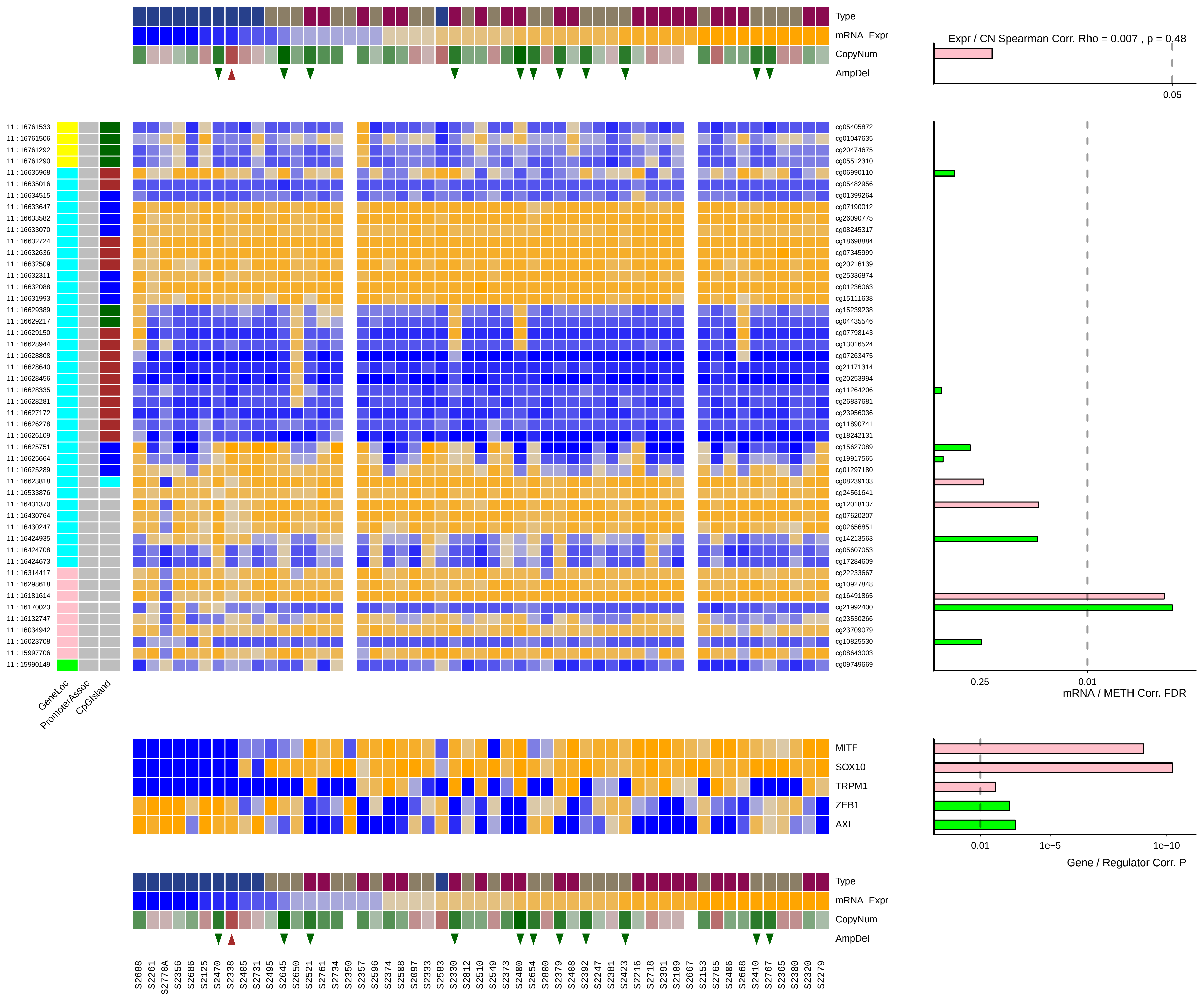
Type
mRNA_Expr
CopyNum
AmpDel
Expr / CN Spearman Corr. Rho = 0.007 , p = 0.48
0.05
11 : 16761533
11 : 16761506
11 : 16761292
11 : 16761290
11 : 16635968
11 : 16635016
11 : 16634515
11 : 16633647
11 : 16633582
11 : 16633070
11 : 16632724
11 : 16632636
11 : 16632509
11 : 16632311
11 : 16632088
11 : 16631993
11 : 16629389
11 : 16629217
11 : 16629150
11 : 16628944
11 : 16628808
11 : 16628640
11 : 16628456
11 : 16628335
11 : 16628281
11 : 16627172
11 : 16626278
11 : 16626109
11 : 16625751
11 : 16625664
11 : 16625289
11 : 16623818
11 : 16533876
11 : 16431370
11 : 16430764
11 : 16430247
11 : 16424935
11 : 16424708
11 : 16424673
11 : 16314417
11 : 16298618
11 : 16181614
11 : 16170023
11 : 16132747
11 : 16034942
11 : 16023708
11 : 15997706
11 : 15990149
cg05405872
cg01047635
cg20474675
cg05512310
cg06990110
cg05482956
cg01399264
cg07190012
cg26090775
cg08245317
cg18698884
cg07345999
cg20216139
cg25336874
cg01236063
cg15111638
cg15239238
cg04435546
cg07798143
cg13016524
cg07263475
cg21171314
cg20253994
cg11264206
cg26837681
cg23956036
cg11890741
cg18242131
cg15627089
cg19917565
cg01297180
cg08239103
cg24561641
cg12018137
cg07620207
cg02656851
cg14213563
cg05607053
cg17284609
cg22233667
cg10927848
cg16491865
cg21992400
cg23530266
cg23709079
cg10825530
cg08643003
cg09749669
0.25
0.01
mRNA / METH Corr. FDR
GeneLoc
PromoterAssoc
CpGIsland
MITF
SOX10
TRPM1
ZEB1
AXL
0.01
1e−5
1e−10
Gene / Regulator Corr. P
Type
mRNA_Expr
CopyNum
AmpDel
S2688
S2261
S2770A
S2356
S2686
S2125
S2470
S2338
S2405
S2731
S2495
S2645
S2650
S2521
S2761
S2734
S2350
S2357
S2596
S2374
S2508
S2097
S2333
S2583
S2330
S2812
S2510
S2549
S2373
S2400
S2654
S2800
S2379
S2408
S2392
S2247
S2381
S2423
S2216
S2718
S2391
S2189
S2667
S2153
S2765
S2406
S2668
S2410
S2767
S2365
S2380
S2320
S2279

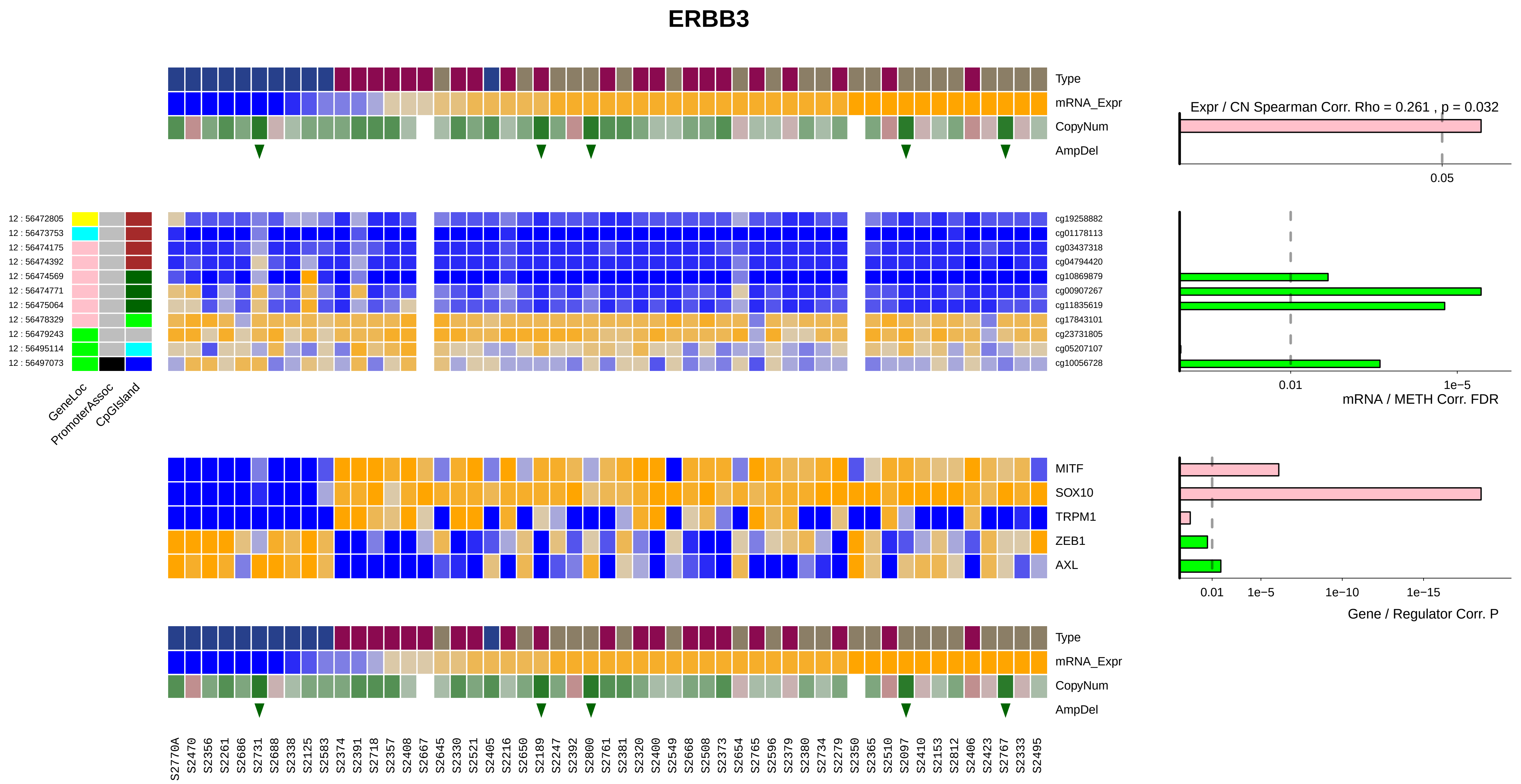
ERBB3
Type
mRNA_Expr
CopyNum
AmpDel
Expr / CN Spearman Corr. Rho = 0.261 , p = 0.032
0.05
12 : 56472805
12 : 56473753
12 : 56474175
12 : 56474392
12 : 56474569
12 : 56474771
12 : 56475064
12 : 56478329
12 : 56479243
12 : 56495114
12 : 56497073
GeneLoc
PromoterAssoc
CpGIsland
cg19258882
cg01178113
cg03437318
cg04794420
cg10869879
cg00907267
cg11835619
cg17843101
cg23731805
cg05207107
cg10056728
0.01
1e−5
mRNA / METH Corr. FDR
MITF
SOX10
TRPM1
ZEB1
AXL
0.01
1e−5
1e−10
1e−15
Gene / Regulator Corr. P
S2770A
S2470
S2356
S2261
S2686
S2731
S2688
S2338
S2125
S2583
S2374
S2391
S2718
S2357
S2408
S2667
S2645
S2330
S2521
S2405
S2216
S2650
S2189
S2247
S2392
S2800
S2761
S2381
S2320
S2400
S2549
S2668
S2508
S2373
S2654
S2765
S2596
S2379
S2380
S2734
S2279
S2350
S2365
S2510
S2097
S2410
S2153
S2812
S2406
S2423
S2767
S2333
S2495

PIR

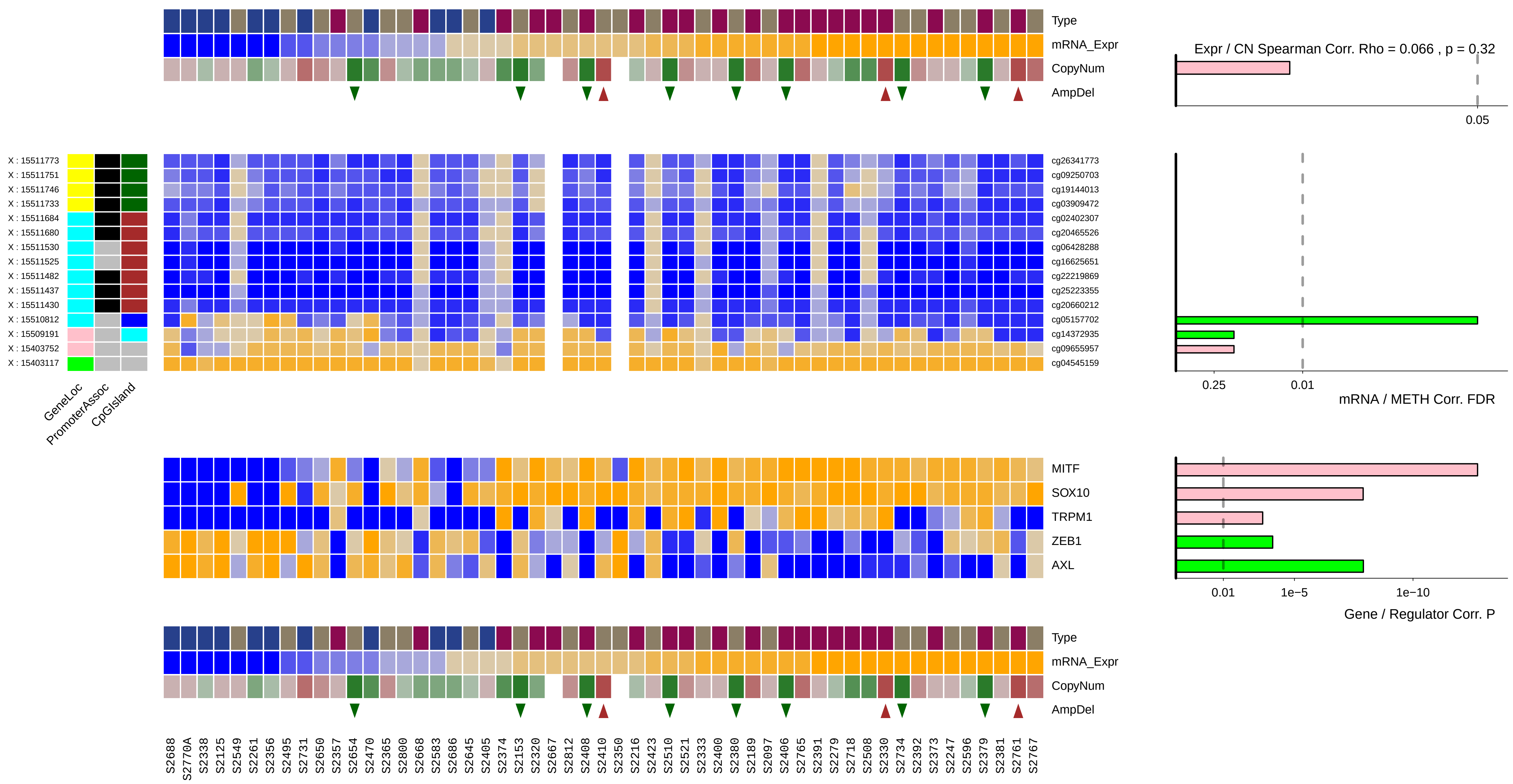
Type
mRNA_Expr
CopyNum
AmpDel
Expr / CN Spearman Corr. Rho = 0.066 , p = 0.32
0.05
mRNA / METH Corr. FDR
0.25
0.01
GeneLoc
PromoterAssoc
CpGIsland
MITF
SOX10
TRPM1
ZEB1
AXL
Gene / Regulator Corr. P
1e−5
1e−10

STK32A

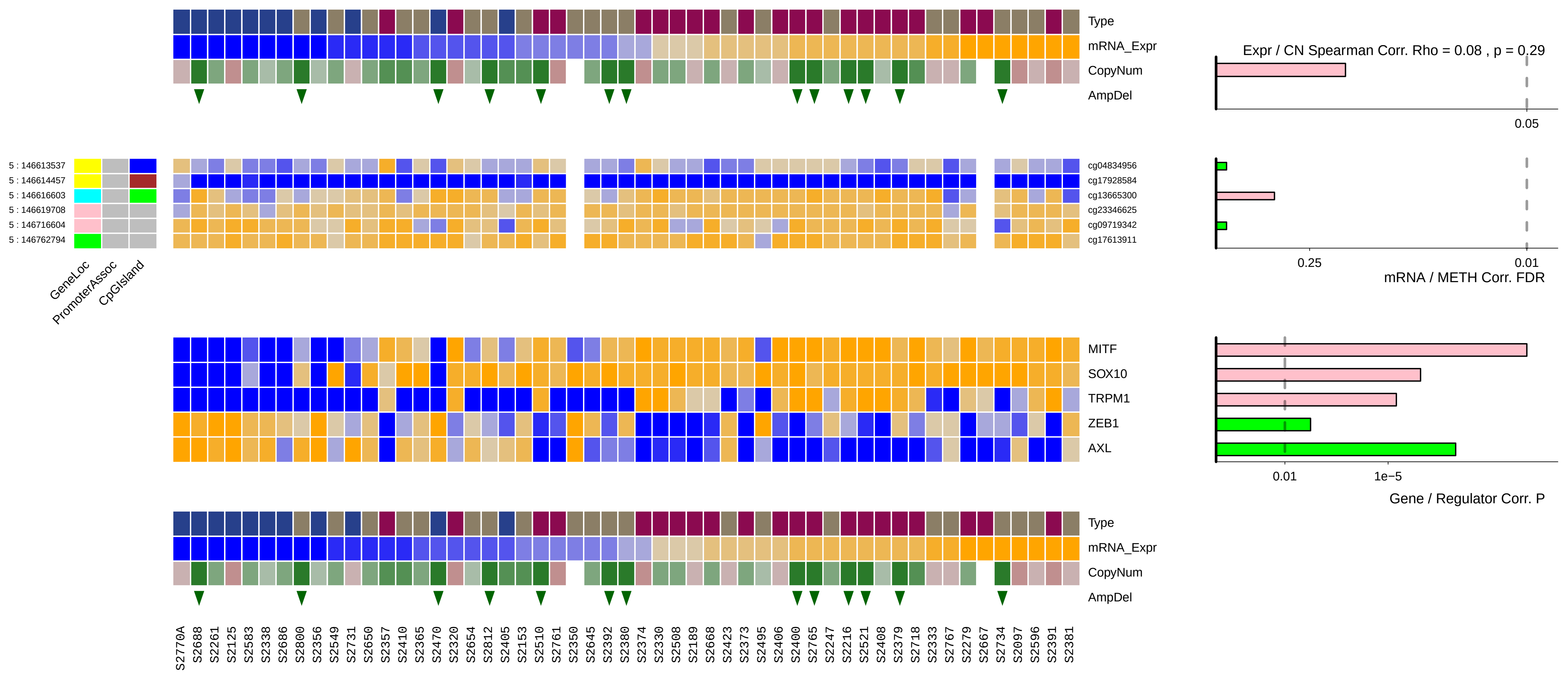
Type
mRNA_Expr
CopyNum
AmpDel
Expr / CN Spearman Corr. Rho = 0.08 , p = 0.29
0.05
5 : 146613537
5 : 146614457
5 : 146616603
5 : 146619708
5 : 146716604
5 : 146762794
GeneLoc
PromoterAssoc
CpGIsland
cg04834956
cg17928584
cg13665300
cg23346625
cg09719342
cg17613911
0.25
0.01
mRNA / METH Corr. FDR
MITF
SOX10
TRPM1
ZEB1
AXL
0.01
1e−5
Gene / Regulator Corr. P
Type
mRNA_Expr
CopyNum
AmpDel
S2770A S2688 S2261 S2125 S2583 S2338 S2686 S2800 S2356 S2549 S2731 S2650 S2357 S2410 S2365 S2470 S2320 S2654 S2812 S2405 S2153 S2510 S2761 S2350 S2645 S2392 S2380 S2374 S2330 S2508 S2189 S2668 S2423 S2373 S2495 S2406 S2400 S2765 S2247 S2216 S2521 S2408 S2379 S2718 S2333 S2767 S2279 S2667 S2734 S2097 S2596 S2391 S2381

SCARB1

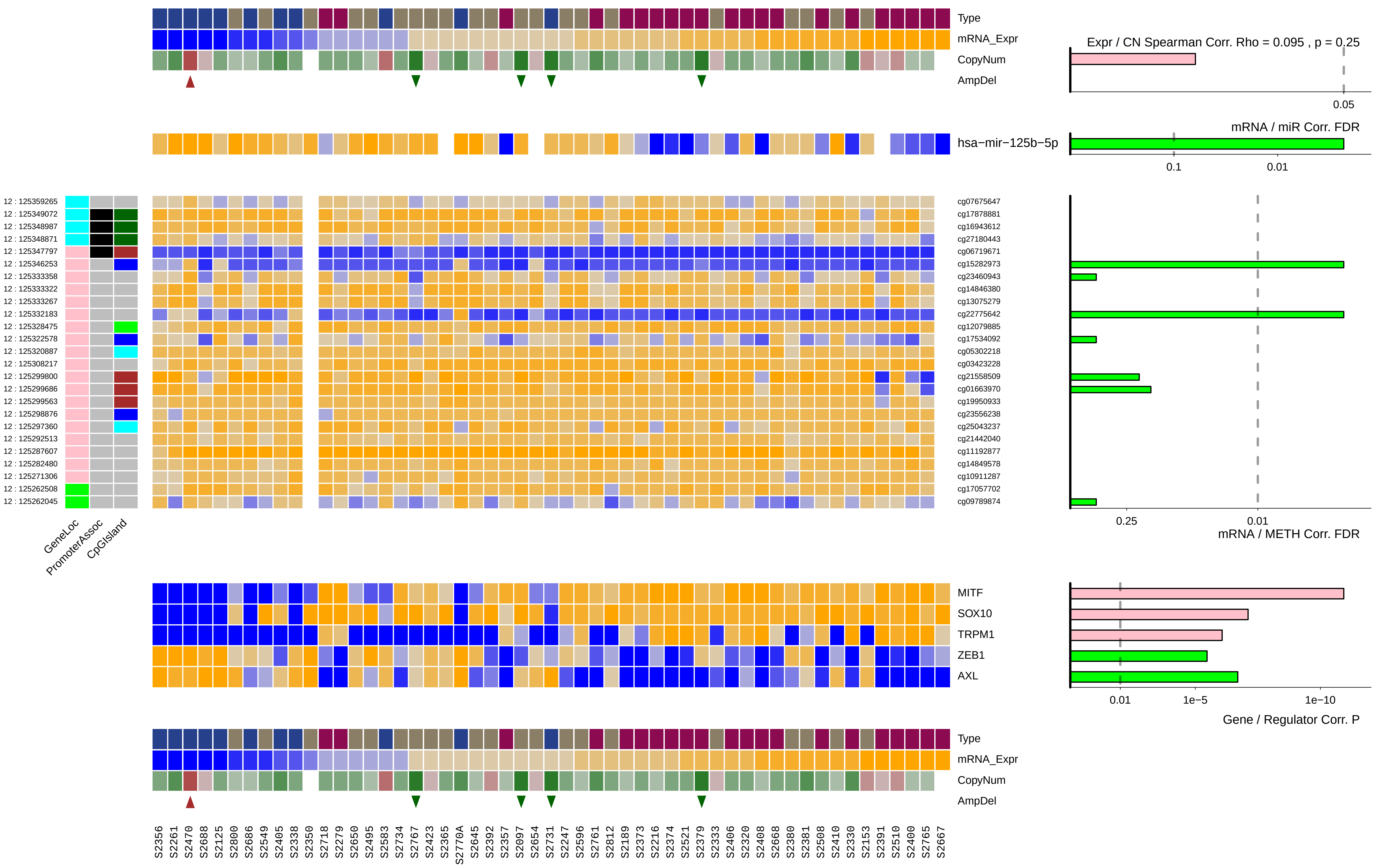

Type
mRNA_Expr
CopyNum
AmpDel
Expr / CN Spearman Corr. Rho = 0.095 , p = 0.25
0.05
hsa−mir−125b−5p
mRNA / miR Corr. FDR
0.1
0.01
mRNA / METH Corr. FDR
0.25
0.01
GeneLoc
PromoterAssoc
CpGIsland
MITF
SOX10
TRPM1
ZEB1
AXL
Gene / Regulator Corr. P
1e−5
1e−10

OVOS2

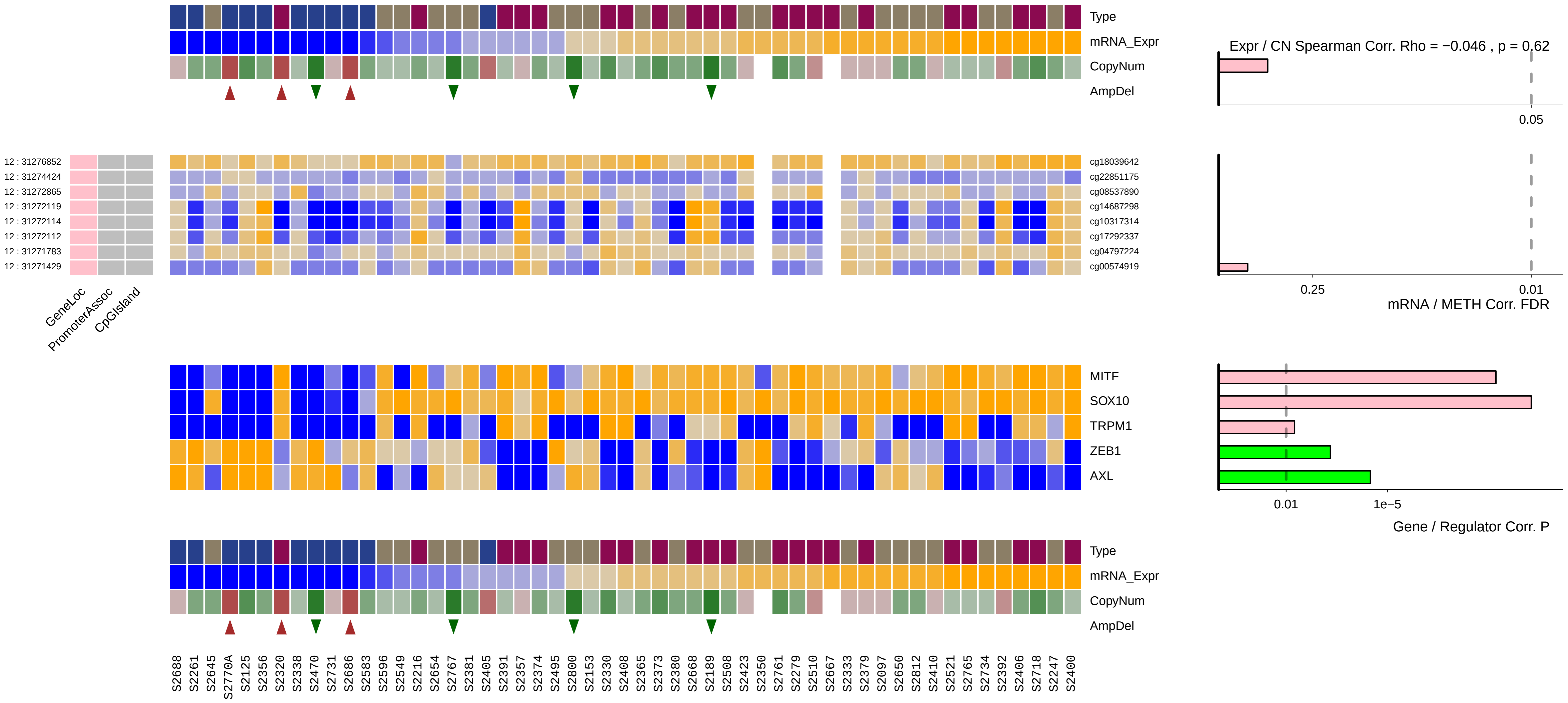
Type
mRNA_Expr
CopyNum
AmpDel
Expr / CN Spearman Corr. Rho = −0.046 , p = 0.62
0.05
12 : 31276852
12 : 31274424
12 : 31272865
12 : 31272119
12 : 31272114
12 : 31272112
12 : 31271783
12 : 31271429
GeneLoc
PromoterAssoc
CpGIsland
cg18039642
cg22851175
cg08537890
cg14687298
cg10317314
cg17292337
cg04797224
cg00574919
0.25
0.01
mRNA / METH Corr. FDR
MITF
SOX10
TRPM1
ZEB1
AXL
0.01
1e−5
Gene / Regulator Corr. P
S2688 S2261 S2645 S2770A S2125 S2356 S2320 S2338 S2470 S2731 S2686 S2583 S2596 S2549 S2216 S2654 S2767 S2381 S2405 S2391 S2357 S2374 S2495 S2800 S2153 S2330 S2408 S2365 S2373 S2380 S2668 S2189 S2508 S2423 S2350 S2761 S2279 S2510 S2667 S2333 S2379 S2097 S2650 S2812 S2410 S2521 S2765 S2734 S2392 S2406 S2718 S2247 S2400

CHCHD6

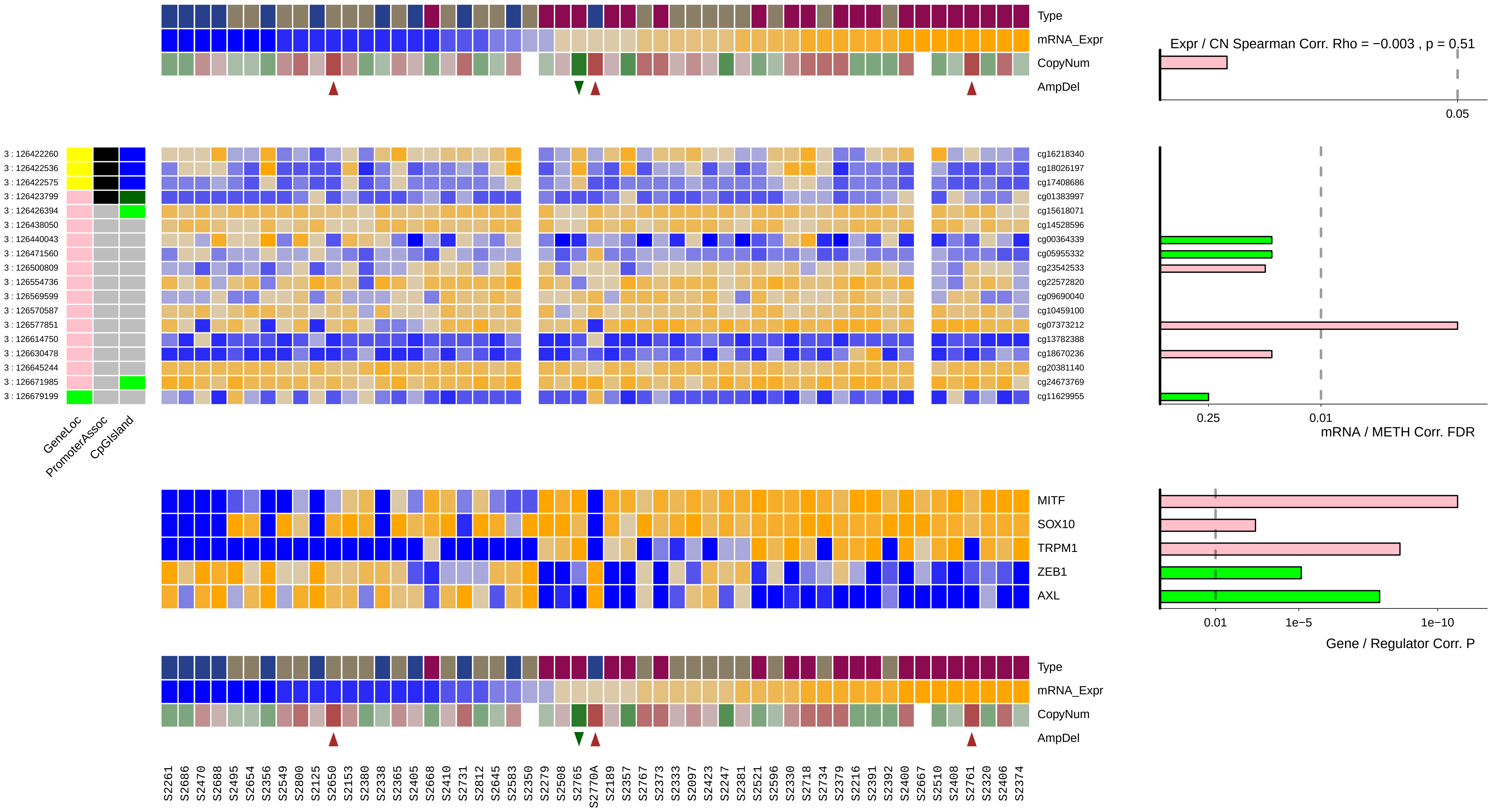

Type
mRNA_Expr
CopyNum
AmpDel
Expr / CN Spearman Corr. Rho = −0.003 , p = 0.51
0.05
GeneLoc
PromoterAssoc
CpGIsland
mRNA / METH Corr. FDR
0.25
0.01
MITF
SOX10
TRPM1
ZEB1
AXL
0.01
1e−5
1e−10
Gene / Regulator Corr. P

SORT1

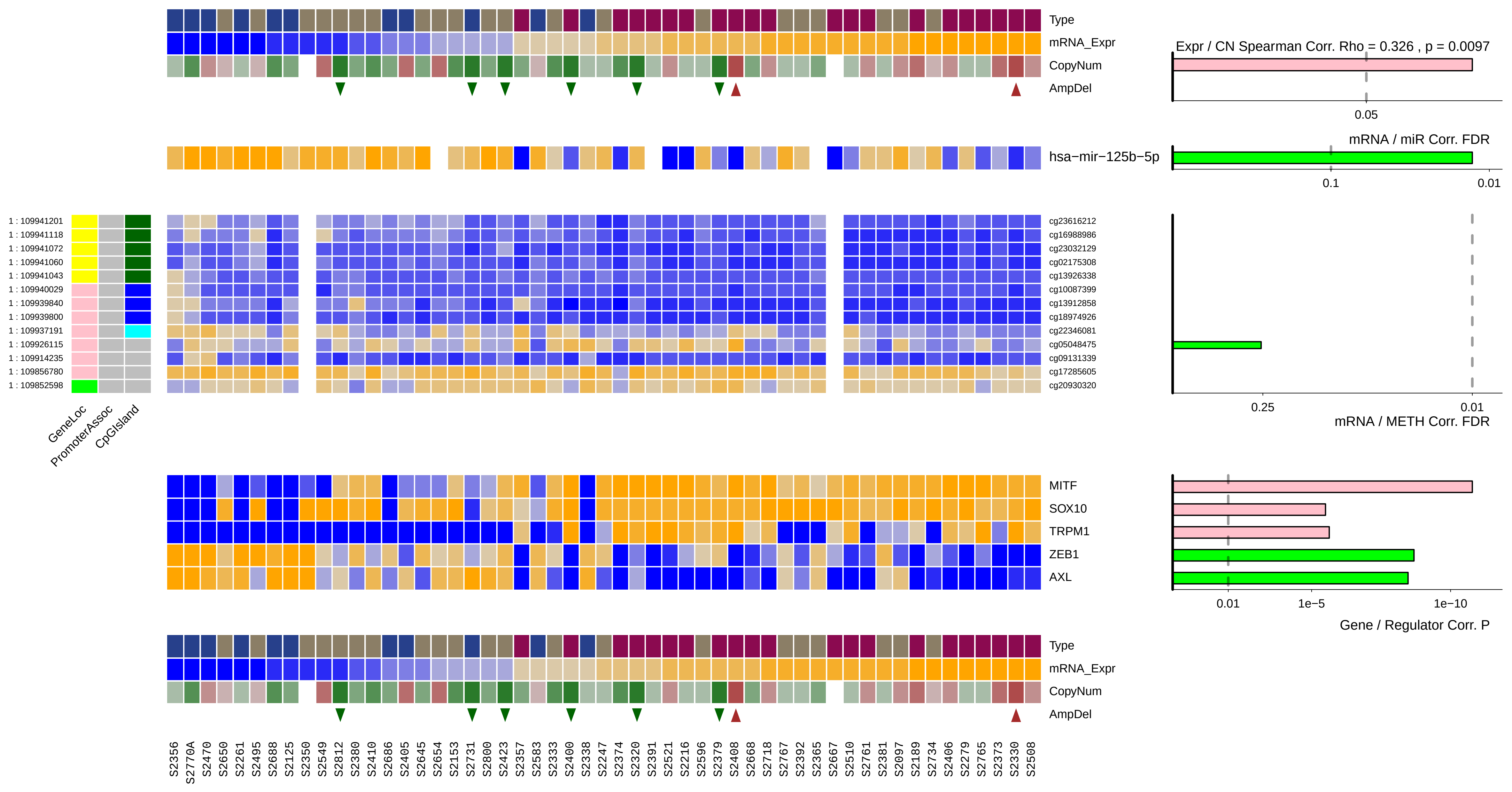

Type
mRNA_Expr
CopyNum
AmpDel
Expr / CN Spearman Corr. Rho = 0.326 , p = 0.0097
0.05
hsa−mir−125b−5p
mRNA / miR Corr. FDR
0.1
0.01
GeneLoc
PromoterAssoc
CpGIsland
0.25
0.01
mRNA / METH Corr. FDR
MITF
SOX10
TRPM1
ZEB1
AXL
0.01
1e−5
1e−10
Gene / Regulator Corr. P

MICAL1

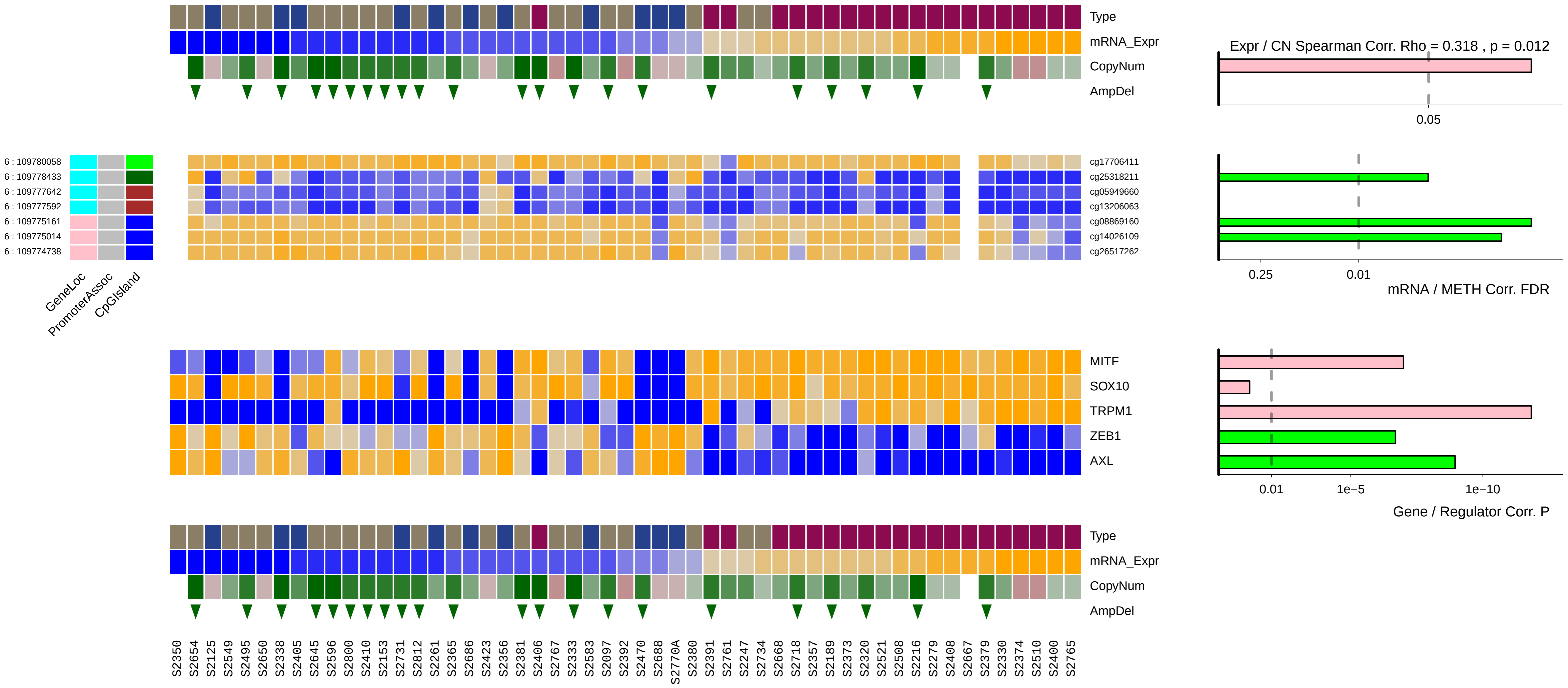

Type
mRNA_Expr
CopyNum
AmpDel
Expr / CN Spearman Corr. Rho = 0.318 , p = 0.012
0.05
6 : 109780058
6 : 109778433
6 : 109777642
6 : 109777592
6 : 109775161
6 : 109775014
6 : 109774738
cg17706411
cg25318211
cg05949660
cg13206063
cg08869160
cg14026109
cg26517262
GeneLoc
PromoterAssoc
CpGIsland
0.25
0.01
mRNA / METH Corr. FDR
MITF
SOX10
TRPM1
ZEB1
AXL
0.01
1e−5
1e−10
Gene / Regulator Corr. P
Type
mRNA_Expr
CopyNum
AmpDel
S2350 S2654 S2125 S2549 S2495 S2650 S2338 S2405 S2645 S2596 S2800 S2410 S2153 S2731 S2812 S2261 S2365 S2686 S2423 S2356 S2381 S2406 S2767 S2333 S2583 S2097 S2392 S2470 S2688 S2770A S2380 S2391 S2761 S2247 S2734 S2668 S2718 S2357 S2189 S2373 S2320 S2521 S2508 S2216 S2279 S2408 S2667 S2379 S2330 S2374 S2510 S2400 S2765

PRDM7

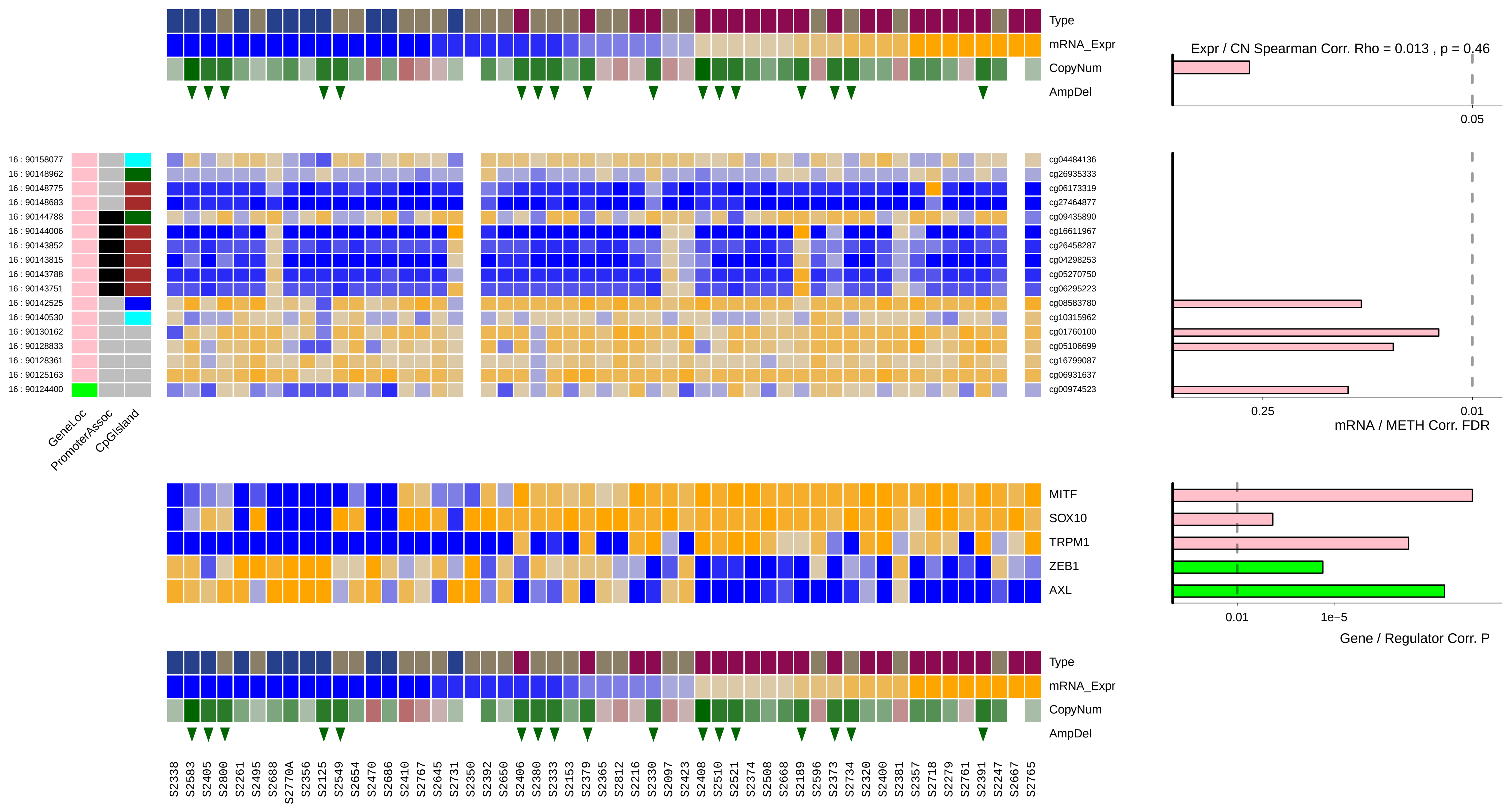
Type
mRNA_Expr
CopyNum
AmpDel
Expr / CN Spearman Corr. Rho = 0.013 , p = 0.46
0.05
cg04484136
cg26935333
cg06173319
cg27464877
cg09435890
cg16611967
cg26458287
cg04298253
cg05270750
cg06295223
cg08583780
cg10315962
cg01760100
cg05106699
cg16799087
cg06931637
cg00974523
16 : 90158077
16 : 90148962
16 : 90148775
16 : 90148683
16 : 90144788
16 : 90144006
16 : 90143852
16 : 90143815
16 : 90143788
16 : 90143751
16 : 90142525
16 : 90140530
16 : 90130162
16 : 90128833
16 : 90128361
16 : 90125163
16 : 90124400
GeneLoc
PromoterAssoc
CpGIsland
0.25
0.01
mRNA / METH Corr. FDR
MITF
SOX10
TRPM1
ZEB1
AXL
0.01
1e−5
Gene / Regulator Corr. P
S2338 S2583 S2405 S2800 S2261 S2495 S2688 S2770A S2356 S2125 S2549 S2654 S2470 S2686 S2410 S2767 S2645 S2731 S2350 S2392 S2650 S2406 S2380 S2333 S2153 S2379 S2365 S2812 S2216 S2330 S2097 S2423 S2408 S2510 S2521 S2374 S2508 S2668 S2189 S2596 S2373 S2734 S2320 S2400 S2381 S2357 S2718 S2279 S2761 S2391 S2247 S2667 S2765

IL16

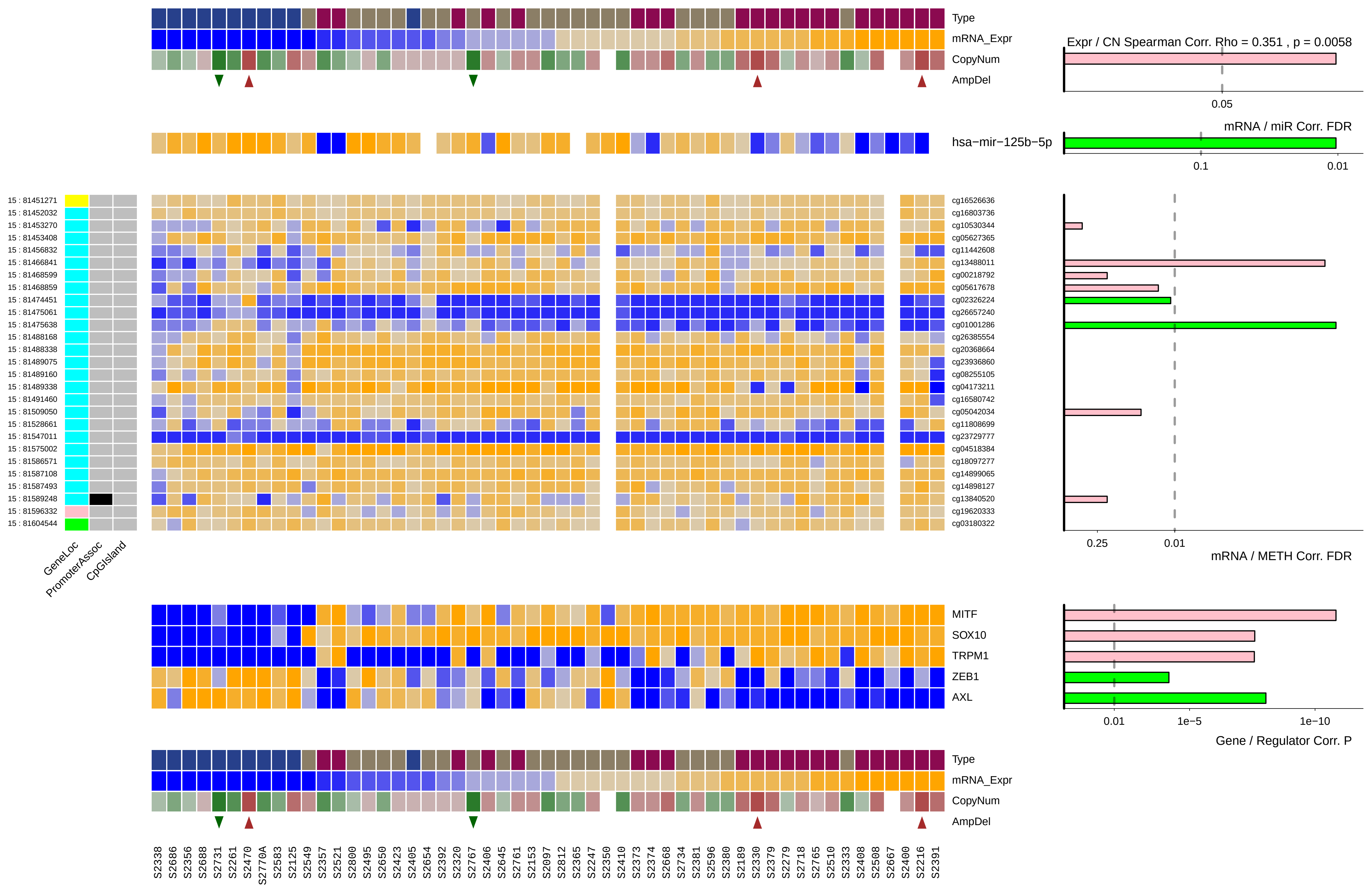

Type
mRNA_Expr
CopyNum
AmpDel
Expr / CN Spearman Corr. Rho = 0.351 , p = 0.0058
0.05
mRNA / miR Corr. FDR
hsa−mir−125b−5p
0.1
0.01
15 : 81451271
15 : 81452032
15 : 81453270
15 : 81453408
15 : 81456832
15 : 81466841
15 : 81468599
15 : 81468859
15 : 81474451
15 : 81475061
15 : 81475638
15 : 81488168
15 : 81488338
15 : 81489075
15 : 81489160
15 : 81489338
15 : 81491460
15 : 81509050
15 : 81528661
15 : 81547011
15 : 81575002
15 : 81586571
15 : 81587108
15 : 81587493
15 : 81589248
15 : 81596332
15 : 81604544
cg16526636
cg16803736
cg10530344
cg05627365
cg11442608
cg13488011
cg00218792
cg05617678
cg02326224
cg26657240
cg01001286
cg26385554
cg20368664
cg23936860
cg08255105
cg04173211
cg16580742
cg05042034
cg11808699
cg23729777
cg04518384
cg18097277
cg14899065
cg14898127
cg13840520
cg19620333
cg03180322
GeneLoc
PromoterAssoc
CpGIsland
0.25
0.01
mRNA / METH Corr. FDR
MITF
SOX10
TRPM1
ZEB1
AXL
0.01
1e−5
1e−10
Gene / Regulator Corr. P
Type
mRNA_Expr
CopyNum
AmpDel
S2338
S2686
S2356
S2688
S2731
S2261
S2470
S2770A
S2583
S2125
S2549
S2357
S2521
S2800
S2495
S2650
S2423
S2405
S2654
S2392
S2320
S2767
S2406
S2645
S2761
S2153
S2097
S2812
S2365
S2247
S2350
S2410
S2373
S2374
S2668
S2734
S2381
S2596
S2380
S2189
S2330
S2379
S2279
S2718
S2765
S2510
S2333
S2408
S2508
S2667
S2400
S2216
S2391

FMN1

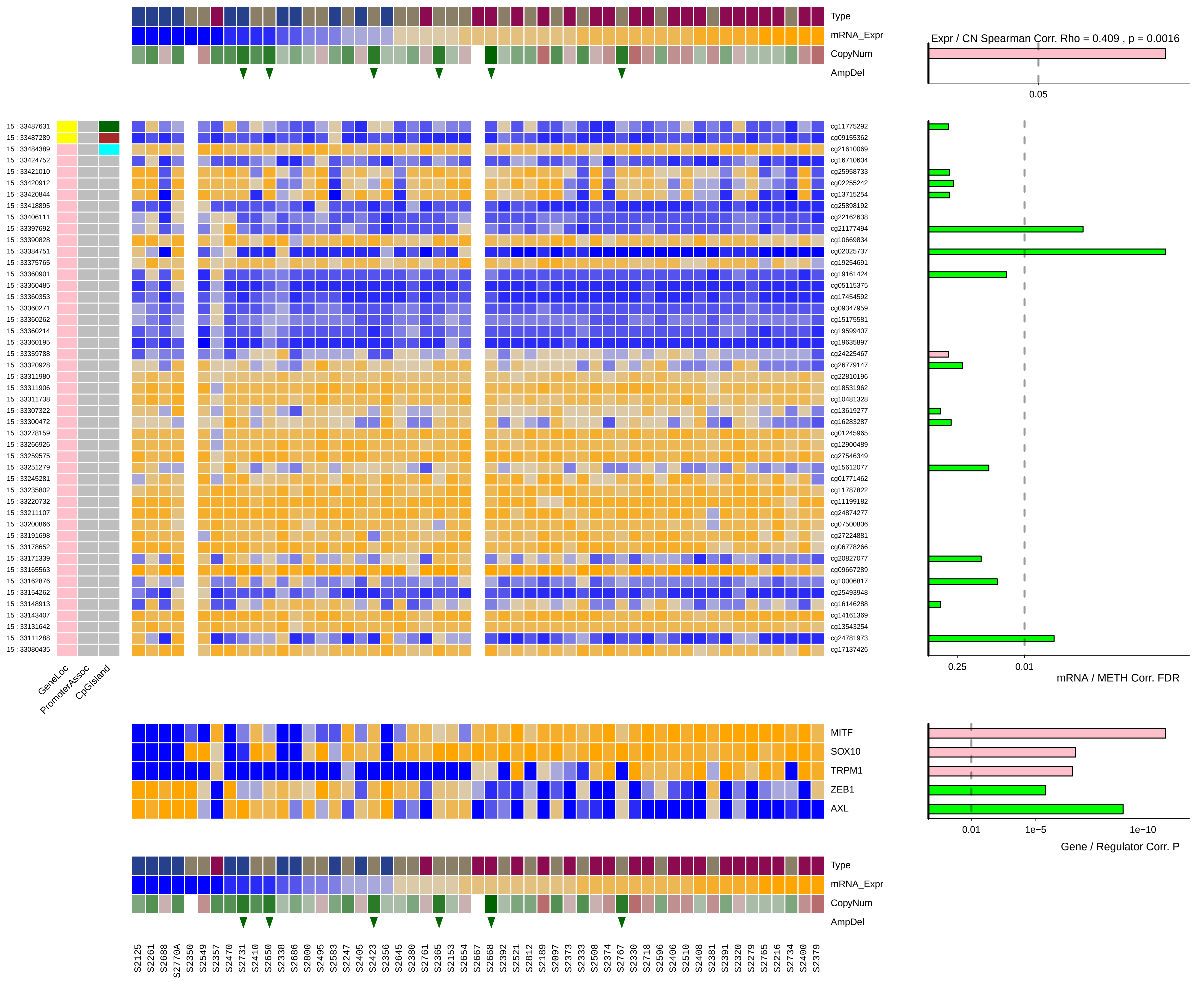
Type
mRNA_Expr
CopyNum
AmpDel
Expr / CN Spearman Corr. Rho = 0.409 , p = 0.0016
0.05
GeneLoc
PromoterAssoc
CpGIsland
0.25
0.01
mRNA / METH Corr. FDR
MITF
SOX10
TRPM1
ZEB1
AXL
0.01
1e−5
1e−10
Gene / Regulator Corr. P

P2RX7

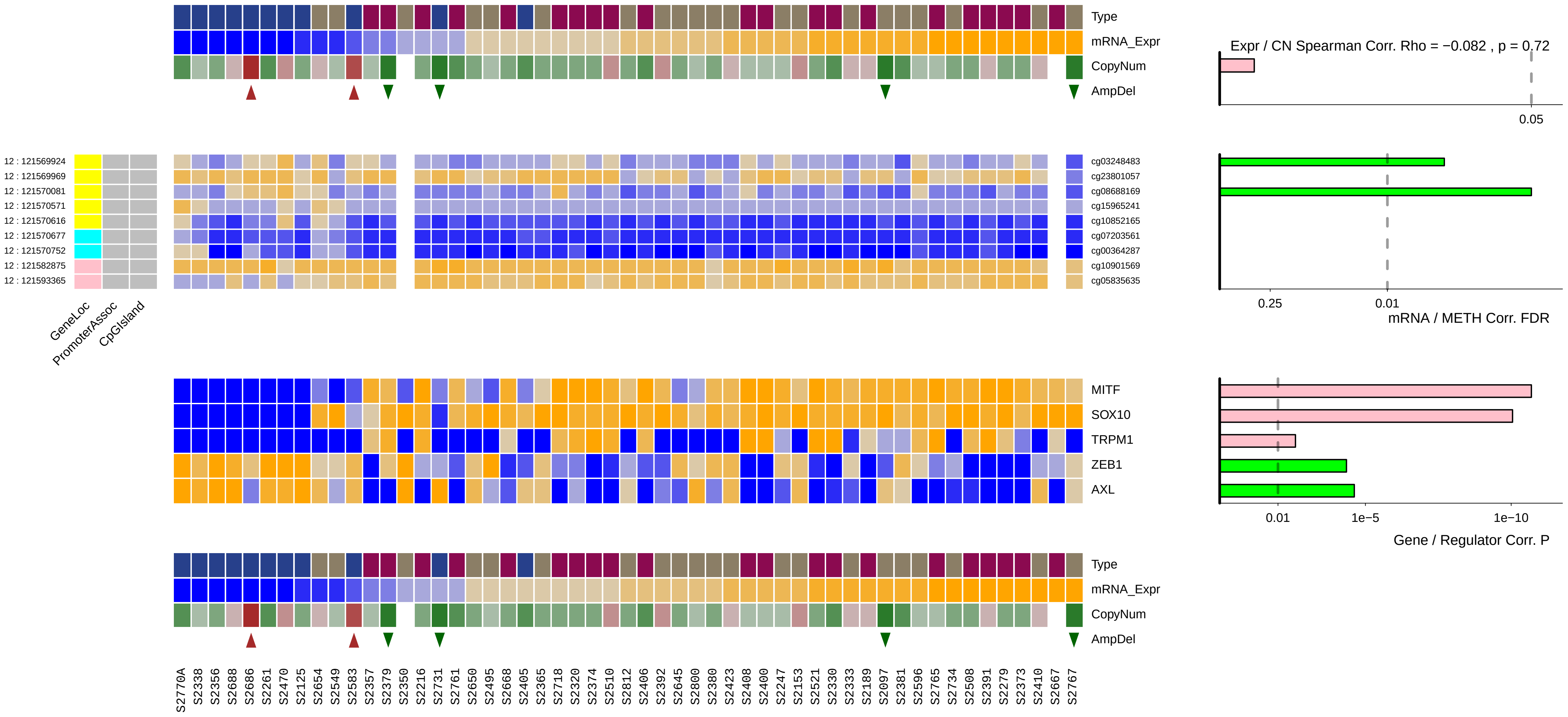

Type
mRNA_Expr
CopyNum
AmpDel
Expr / CN Spearman Corr. Rho = −0.082 , p = 0.72
0.05
12 : 121569924
12 : 121569969
12 : 121570081
12 : 121570571
12 : 121570616
12 : 121570677
12 : 121570752
12 : 121582875
12 : 121593365
GeneLoc
PromoterAssoc
CpGIsland
cg03248483
cg23801057
cg08688169
cg15965241
cg10852165
cg07203561
cg00364287
cg10901569
cg05835635
0.25
0.01
mRNA / METH Corr. FDR
MITF
SOX10
TRPM1
ZEB1
AXL
0.01
1e−5
1e−10
Gene / Regulator Corr. P
S2770A
S2338
S2356
S2688
S2686
S2261
S2470
S2125
S2654
S2549
S2583
S2357
S2379
S2350
S2216
S2731
S2761
S2650
S2495
S2668
S2405
S2365
S2718
S2320
S2374
S2510
S2812
S2406
S2392
S2645
S2800
S2380
S2423
S2408
S2400
S2247
S2153
S2521
S2330
S2333
S2189
S2097
S2381
S2596
S2765
S2734
S2508
S2391
S2279
S2373
S2410
S2667
S2767

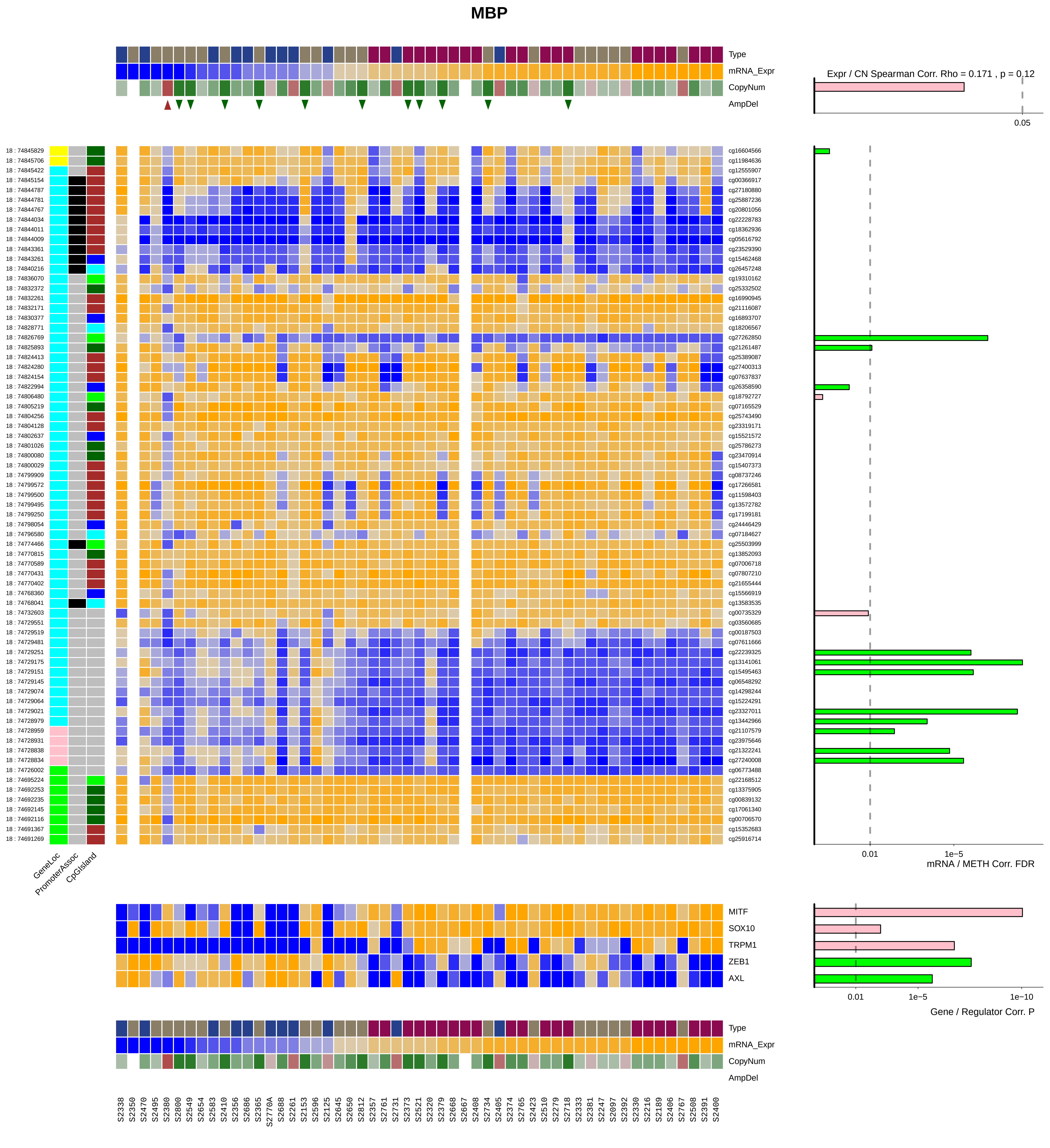
MBP
Type
mRNA_Expr
CopyNum
AmpDel
Expr / CN Spearman Corr. Rho = 0.171 , p = 0.12
0.05
18 : 74845829 cg16604566
18 : 74845706 cg11984636
18 : 74845422 cg12555907
18 : 74845154 cg00366917
18 : 74844787 cg27180880
18 : 74844781 cg25887236
18 : 74844767 cg20801056
18 : 74844034 cg22228783
18 : 74844011 cg18362936
18 : 74844009 cg05616792
18 : 74843361 cg23529390
18 : 74843261 cg15462468
18 : 74840216 cg26457248
18 : 74836070 cg19310162
18 : 74832372 cg25332502
18 : 74832261 cg16990945
18 : 74832171 cg21116087
18 : 74830377 cg16893707
18 : 74828771 cg18206567
18 : 74826769 cg27262850
18 : 74825893 cg21261487
18 : 74824413 cg25389087
18 : 74824280 cg27400313
18 : 74824154 cg07637837
18 : 74822994 cg26358590
18 : 74806480 cg18792727
18 : 74805219 cg07165529
18 : 74804256 cg25743490
18 : 74804128 cg23319171
18 : 74802637 cg15521572
18 : 74801026 cg25786273
18 : 74800080 cg23470914
18 : 74800029 cg15407373
18 : 74799909 cg08737246
18 : 74799572 cg17266581
18 : 74799500 cg11598403
18 : 74799495 cg13572782
18 : 74799250 cg17199181
18 : 74798054 cg24446429
18 : 74796580 cg07184627
18 : 74774466 cg25503999
18 : 74770815 cg13852093
18 : 74770589 cg07006718
18 : 74770431 cg07807210
18 : 74770402 cg21655444
18 : 74768360 cg15566919
18 : 74768041 cg13583535
18 : 74732603 cg00735329
18 : 74729551 cg03560685
18 : 74729519 cg00187503
18 : 74729481 cg07611666
18 : 74729251 cg22239325
18 : 74729175 cg13141061
18 : 74729151 cg15495463
18 : 74729145 cg06548292
18 : 74729074 cg14298244
18 : 74729064 cg15224291
18 : 74729021 cg23327011
18 : 74728979 cg13442966
18 : 74728959 cg21107579
18 : 74728931 cg23975646
18 : 74728838 cg21322241
18 : 74728834 cg27240008
18 : 74726002 cg06773488
18 : 74695224 cg22168512
18 : 74692253 cg13375905
18 : 74692235 cg00839132
18 : 74692145 cg17061340
18 : 74692116 cg00706570
18 : 74691367 cg15352683
18 : 74691269 cg25916714
GeneLoc
PromoterAssoc
CpGIsland
0.01
1e−5
mRNA / METH Corr. FDR
MITF
SOX10
TRPM1
ZEB1
AXL
0.01
1e−5
1e−10
Gene / Regulator Corr. P
Type
mRNA_Expr
CopyNum
AmpDel
S2338 S2350 S2470 S2495 S2380 S2800 S2549 S2654 S2583 S2410 S2356 S2686 S2365 S2770A S2688 S2261 S2153 S2596 S2125 S2645 S2650 S2812 S2357 S2761 S2731 S2373 S2521 S2320 S2379 S2668 S2667 S2408 S2734 S2405 S2374 S2765 S2423 S2510 S2279 S2718 S2333 S2381 S2247 S2097 S2392 S2330 S2216 S2189 S2406 S2767 S2508 S2391 S2400

IVNS1ABP

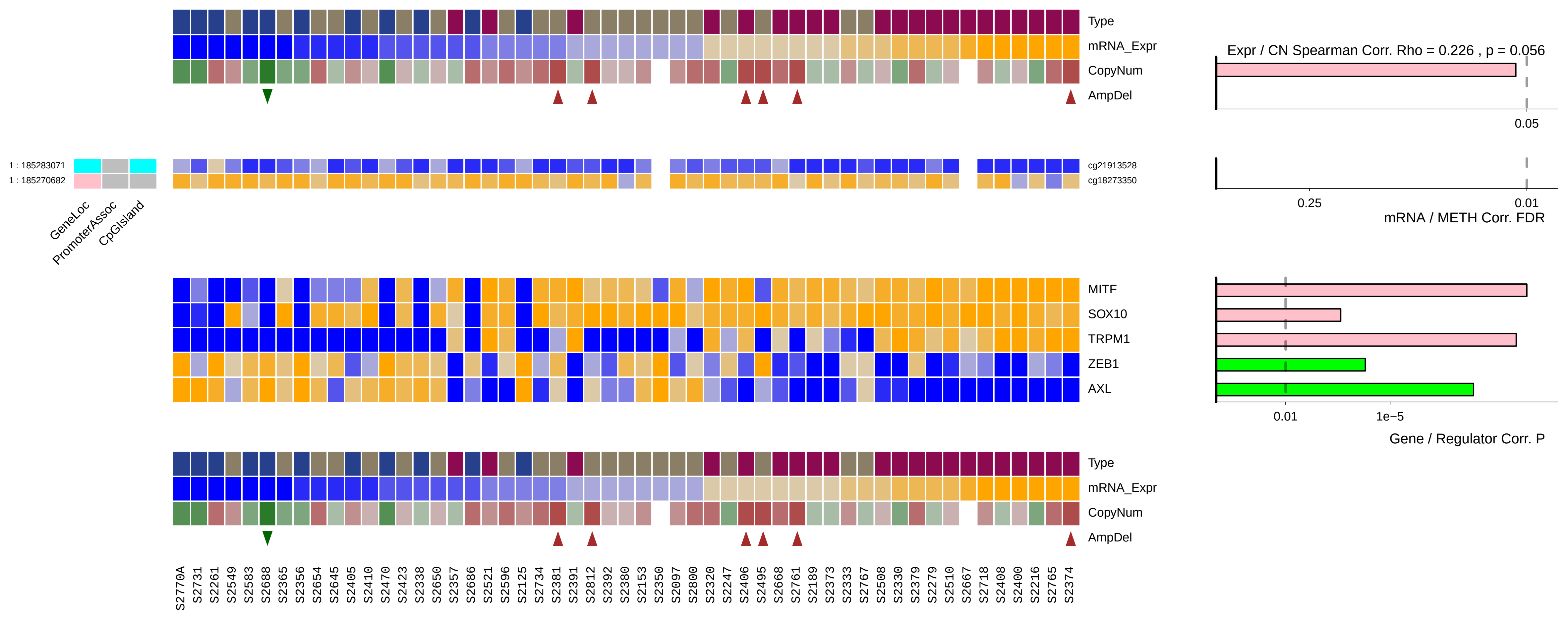

Type
mRNA_Expr
CopyNum
AmpDel
Expr / CN Spearman Corr. Rho = 0.226 , p = 0.056
0.05
1 : 185283071
1 : 185270682
cg21913528
cg18273350
GeneLoc
PromoterAssoc
CpGIsland
0.25
0.01
mRNA / METH Corr. FDR
MITF
SOX10
TRPM1
ZEB1
AXL
0.01
1e−5
Gene / Regulator Corr. P
S2770A S2731 S2261 S2549 S2583 S2688 S2365 S2356 S2654 S2645 S2405 S2410 S2470 S2423 S2338 S2650 S2357 S2686 S2521 S2596 S2125 S2734 S2381 S2391 S2812 S2392 S2380 S2153 S2350 S2097 S2800 S2320 S2247 S2406 S2495 S2668 S2761 S2189 S2373 S2333 S2767 S2508 S2330 S2379 S2279 S2510 S2667 S2718 S2408 S2400 S2216 S2765 S2374

SLC16A6

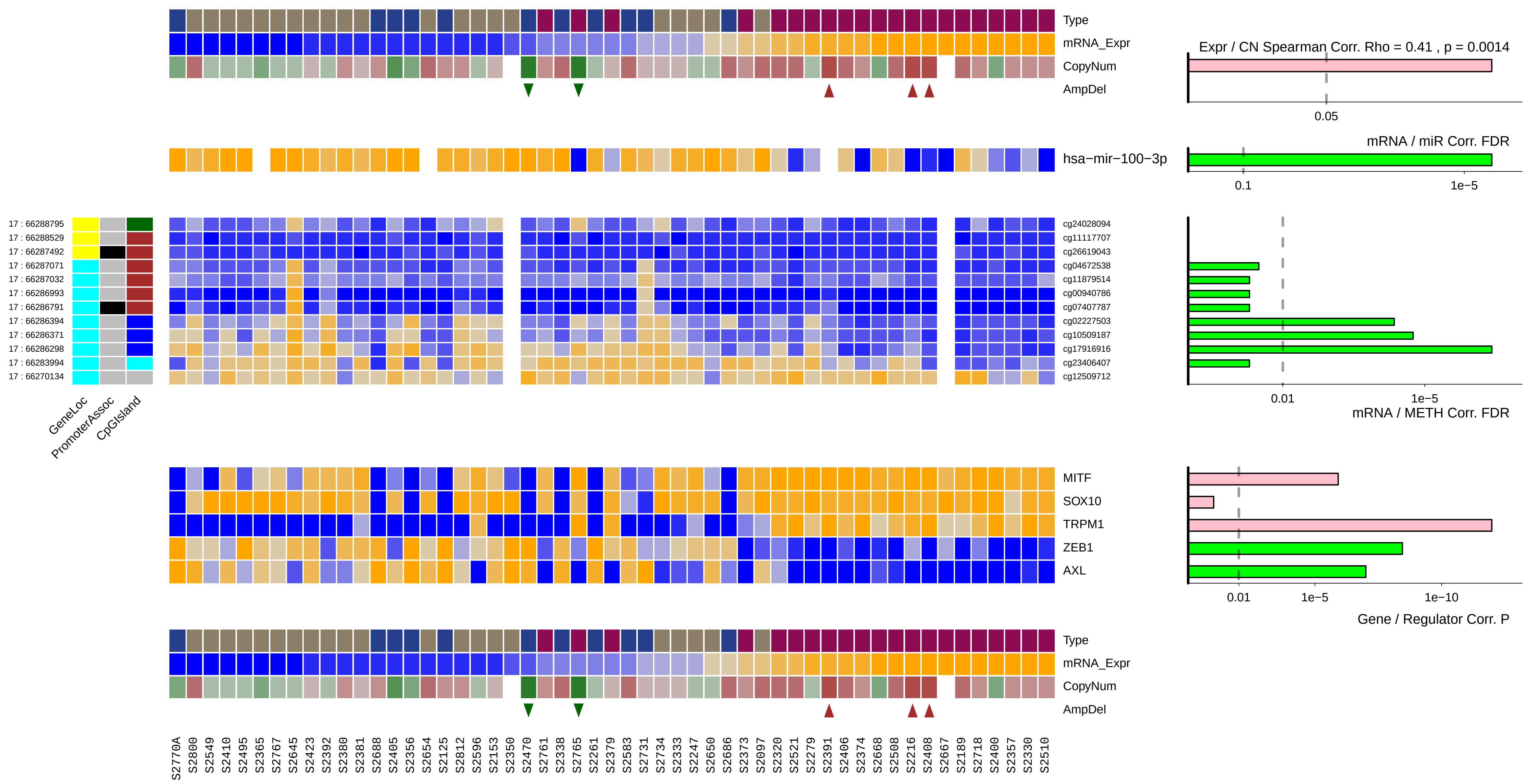
Type
mRNA_Expr
CopyNum
AmpDel
Expr / CN Spearman Corr. Rho = 0.41 , p = 0.0014
0.05
hsa−mir−100−3p
mRNA / miR Corr. FDR
0.1
1e−5
17 : 66288795
17 : 66288529
17 : 66287492
17 : 66287071
17 : 66287032
17 : 66286993
17 : 66286791
17 : 66286394
17 : 66286371
17 : 66286298
17 : 66283994
17 : 66270134
cg24028094
cg11117707
cg26619043
cg04672538
cg11879514
cg00940786
cg07407787
cg02227503
cg10509187
cg17916916
cg23406407
cg12509712
GeneLoc
PromoterAssoc
CpGIsland
0.01
1e−5
mRNA / METH Corr. FDR
MITF
SOX10
TRPM1
ZEB1
AXL
0.01
1e−5
1e−10
Gene / Regulator Corr. P
S2770A S2800 S2549 S2410 S2495 S2365 S2767 S2645 S2423 S2392 S2380 S2381 S2688 S2405 S2356 S2654 S2125 S2812 S2596 S2153 S2350 S2470 S2761 S2338 S2765 S2261 S2379 S2583 S2731 S2734 S2333 S2247 S2650 S2686 S2373 S2097 S2320 S2521 S2279 S2391 S2406 S2374 S2668 S2508 S2216 S2408 S2667 S2189 S2718 S2400 S2357 S2330 S2510

MYEF2

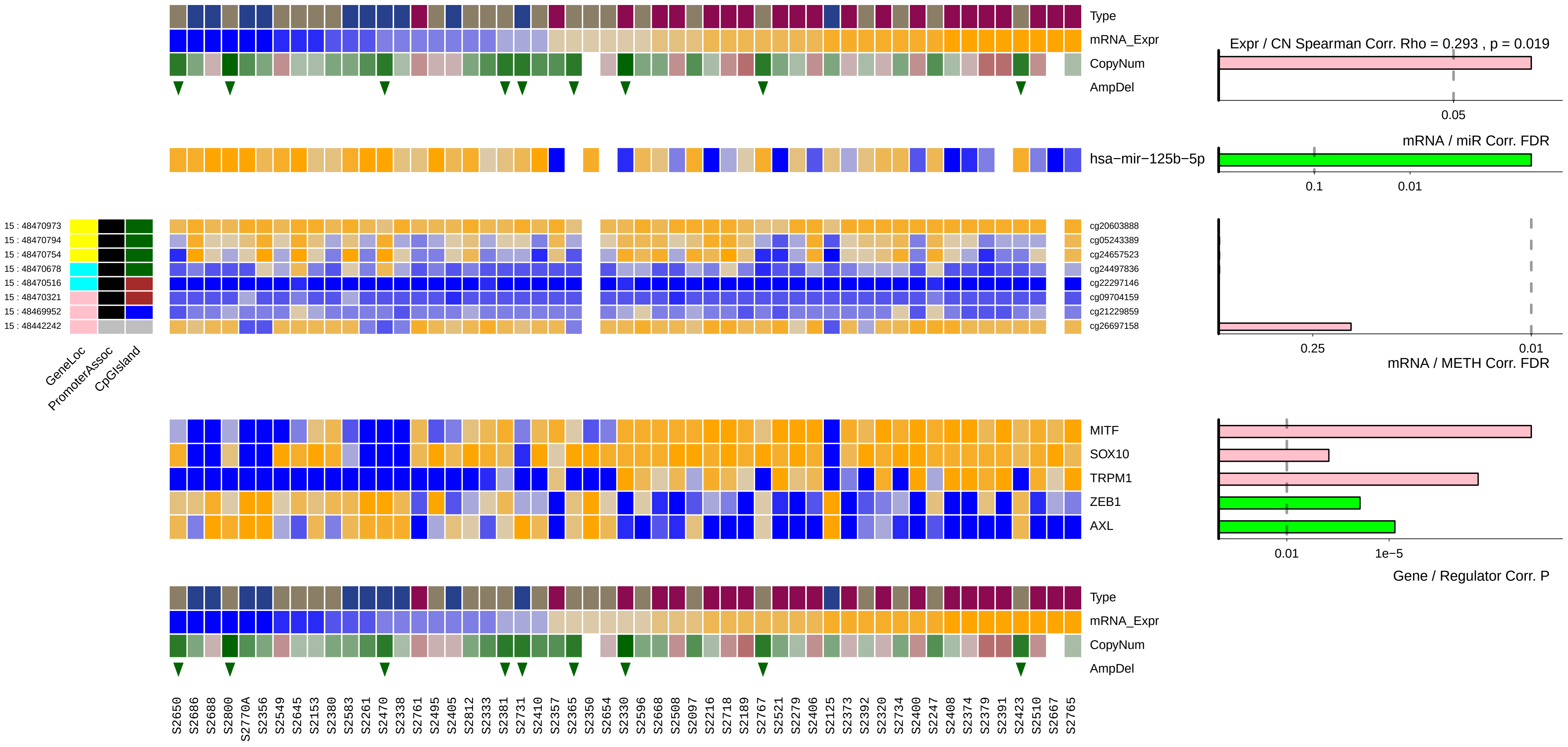

Type
mRNA_Expr
CopyNum
AmpDel
Expr / CN Spearman Corr. Rho = 0.293 , p = 0.019
0.05
mRNA / miR Corr. FDR
hsa−mir−125b−5p
0.1
0.01
15 : 48470973
15 : 48470794
15 : 48470754
15 : 48470678
15 : 48470516
15 : 48470321
15 : 48469952
15 : 48442242
cg20603888
cg05243389
cg24657523
cg24497836
cg22297146
cg09704159
cg21229859
cg26697158
0.25
0.01
mRNA / METH Corr. FDR
GeneLoc
PromoterAssoc
CpGIsland
MITF
SOX10
TRPM1
ZEB1
AXL
0.01
1e−5
Gene / Regulator Corr. P
S2650 S2686 S2688 S2800 S2770A S2356 S2549 S2645 S2153 S2380 S2583 S2261 S2470 S2338 S2761 S2495 S2405 S2812 S2333 S2381 S2731 S2410 S2357 S2365 S2350 S2654 S2330 S2596 S2668 S2508 S2097 S2216 S2718 S2189 S2767 S2521 S2279 S2406 S2125 S2373 S2392 S2320 S2734 S2400 S2247 S2408 S2374 S2379 S2391 S2423 S2510 S2667 S2765

CYP27A1

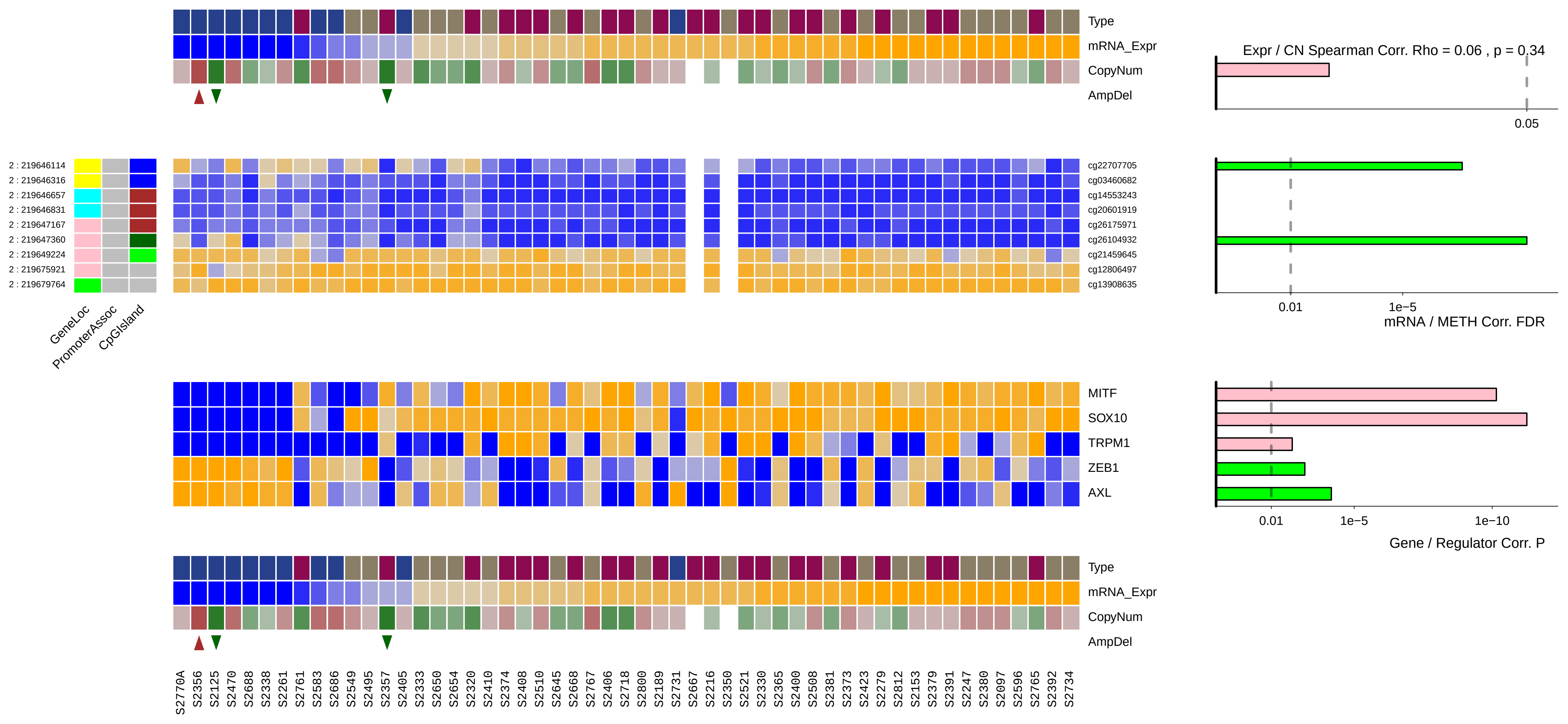

Type
mRNA_Expr
CopyNum
AmpDel
Expr / CN Spearman Corr. Rho = 0.06 , p = 0.34
0.05
2 : 219646114
2 : 219646316
2 : 219646657
2 : 219646831
2 : 219647167
2 : 219647360
2 : 219649224
2 : 219675921
2 : 219679764
GeneLoc
PromoterAssoc
CpGIsland
cg22707705
cg03460682
cg14553243
cg20601919
cg26175971
cg26104932
cg21459645
cg12806497
cg13908635
0.01
1e−5
mRNA / METH Corr. FDR
MITF
SOX10
TRPM1
ZEB1
AXL
0.01
1e−5
1e−10
Gene / Regulator Corr. P
Type
mRNA_Expr
CopyNum
AmpDel
S2770A S2356 S2125 S2470 S2688 S2338 S2261 S2761 S2583 S2686 S2549 S2495 S2357 S2405 S2333 S2650 S2654 S2320 S2410 S2374 S2408 S2510 S2645 S2668 S2767 S2406 S2718 S2800 S2189 S2731 S2667 S2216 S2350 S2521 S2330 S2365 S2400 S2508 S2381 S2373 S2423 S2279 S2812 S2153 S2379 S2391 S2247 S2380 S2097 S2596 S2765 S2392 S2734

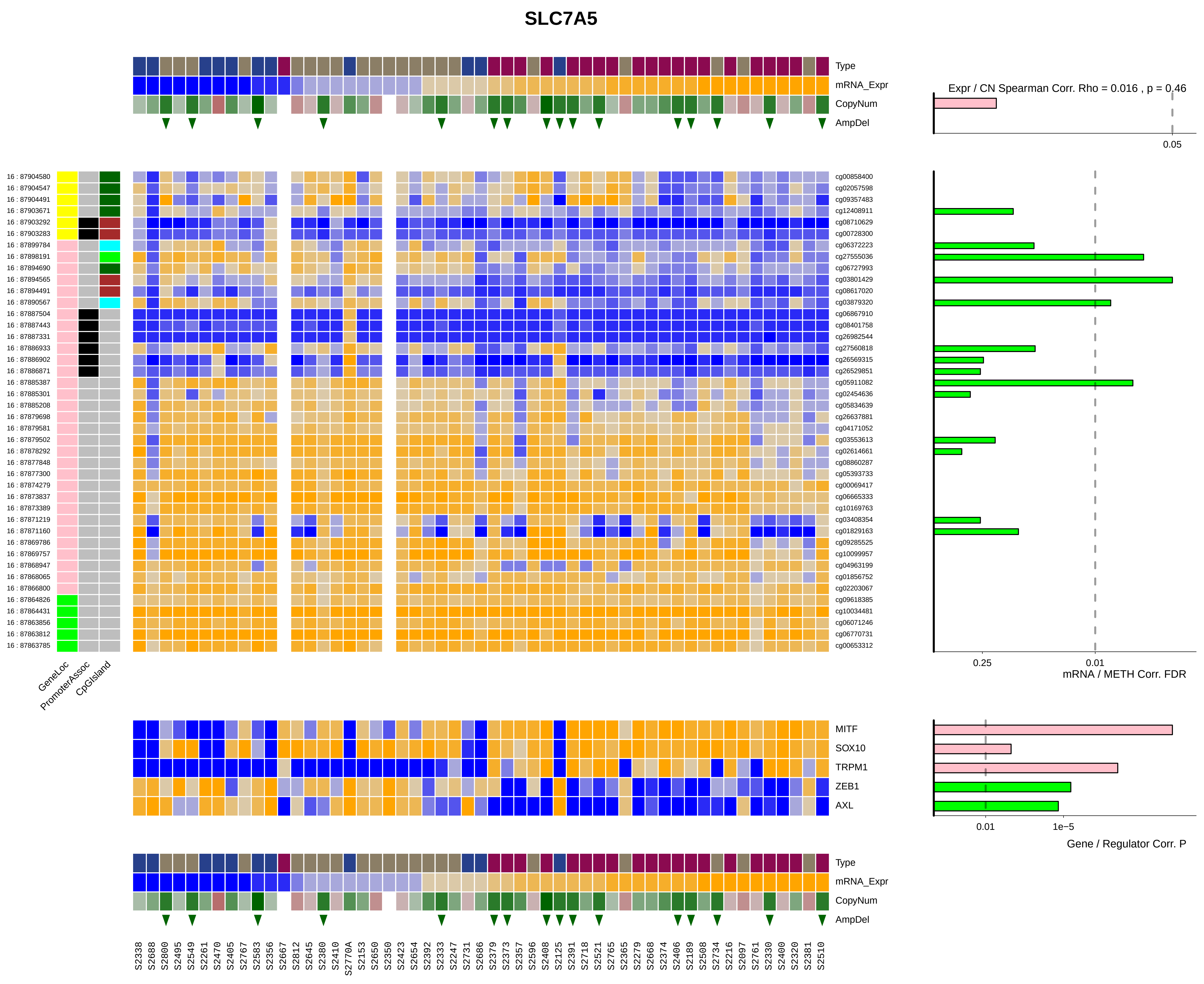
SLC7A5
Type
mRNA_Expr
CopyNum
AmpDel
Expr / CN Spearman Corr. Rho = 0.016 , p = 0.46
0.05
GeneLoc
PromoterAssoc
CpGIsland
mRNA / METH Corr. FDR
0.25
0.01
MITF
SOX10
TRPM1
ZEB1
AXL
Gene / Regulator Corr. P
1e−5

PLXNC1

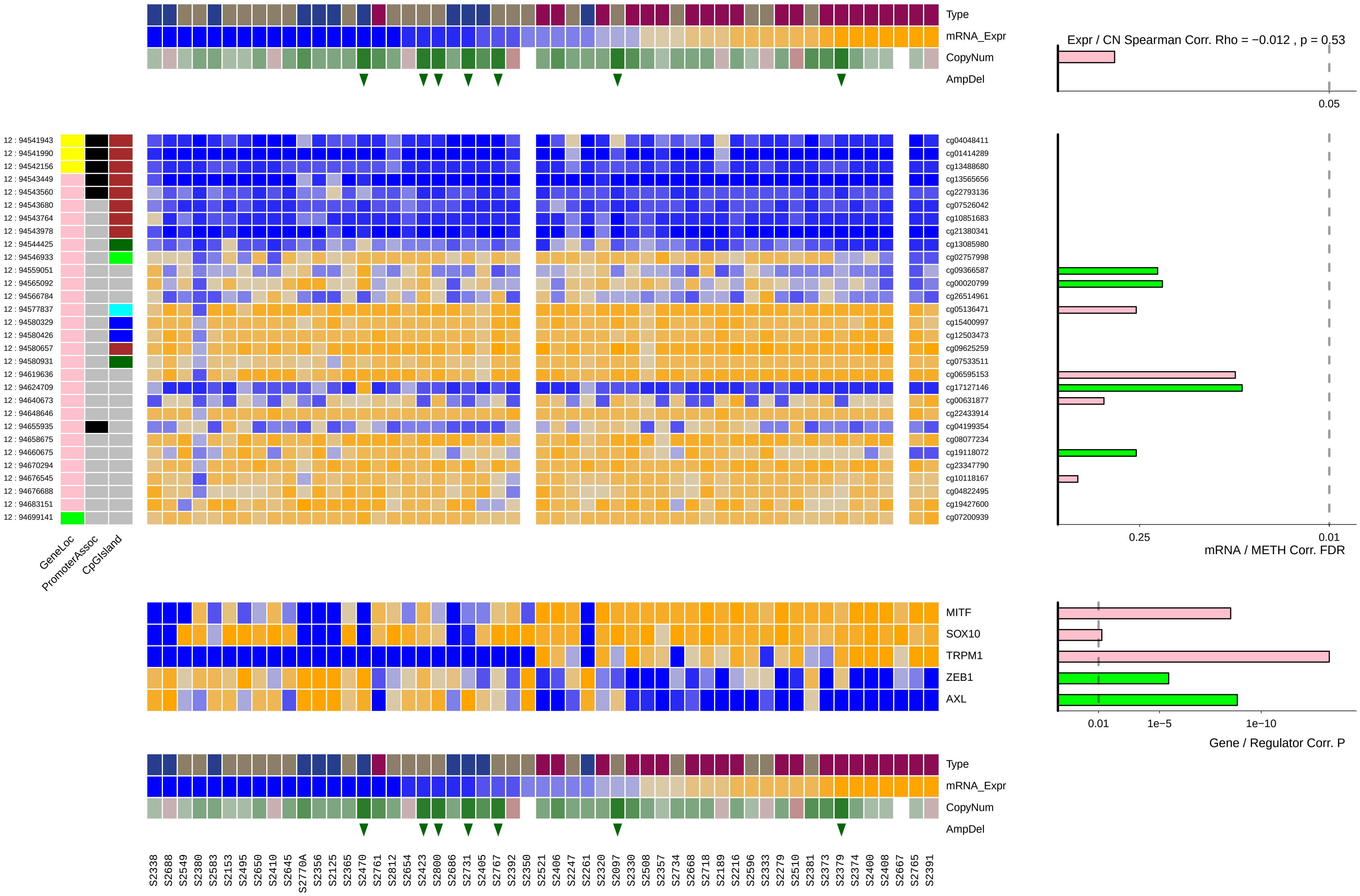

Type
mRNA_Expr
CopyNum
AmpDel
Expr / CN Spearman Corr. Rho = −0.012 , p = 0.53
0.05
12 : 94541943
12 : 94541990
12 : 94542156
12 : 94543449
12 : 94543560
12 : 94543680
12 : 94543764
12 : 94543978
12 : 94544425
12 : 94546933
12 : 94559051
12 : 94565092
12 : 94566784
12 : 94577837
12 : 94580329
12 : 94580426
12 : 94580657
12 : 94580931
12 : 94619636
12 : 94624709
12 : 94640673
12 : 94648646
12 : 94655935
12 : 94658675
12 : 94660675
12 : 94670294
12 : 94676545
12 : 94676688
12 : 94683151
12 : 94699141
cg04048411
cg01414289
cg13488680
cg13565656
cg22793136
cg07526042
cg10851683
cg21380341
cg13085980
cg02757998
cg09366587
cg00020799
cg26514961
cg05136471
cg15400997
cg12503473
cg09625259
cg07533511
cg06595153
cg17127146
cg00631877
cg22433914
cg04199354
cg08077234
cg19118072
cg23347790
cg10118167
cg04822495
cg19427600
cg07200939
GeneLoc
PromoterAssoc
CpGIsland
0.25
0.01
mRNA / METH Corr. FDR
MITF
SOX10
TRPM1
ZEB1
AXL
0.01
1e−5
1e−10
Gene / Regulator Corr. P
Type
mRNA_Expr
CopyNum
AmpDel
S2338 S2688 S2549 S2380 S2583 S2153 S2495 S2650 S2410 S2645 S2770A S2356 S2125 S2365 S2470 S2761 S2812 S2654 S2423 S2800 S2686 S2731 S2405 S2767 S2392 S2350 S2521 S2406 S2247 S2261 S2320 S2097 S2330 S2508 S2357 S2734 S2668 S2718 S2189 S2216 S2596 S2333 S2279 S2510 S2381 S2373 S2379 S2374 S2400 S2408 S2667 S2765 S2391

BCL2A1

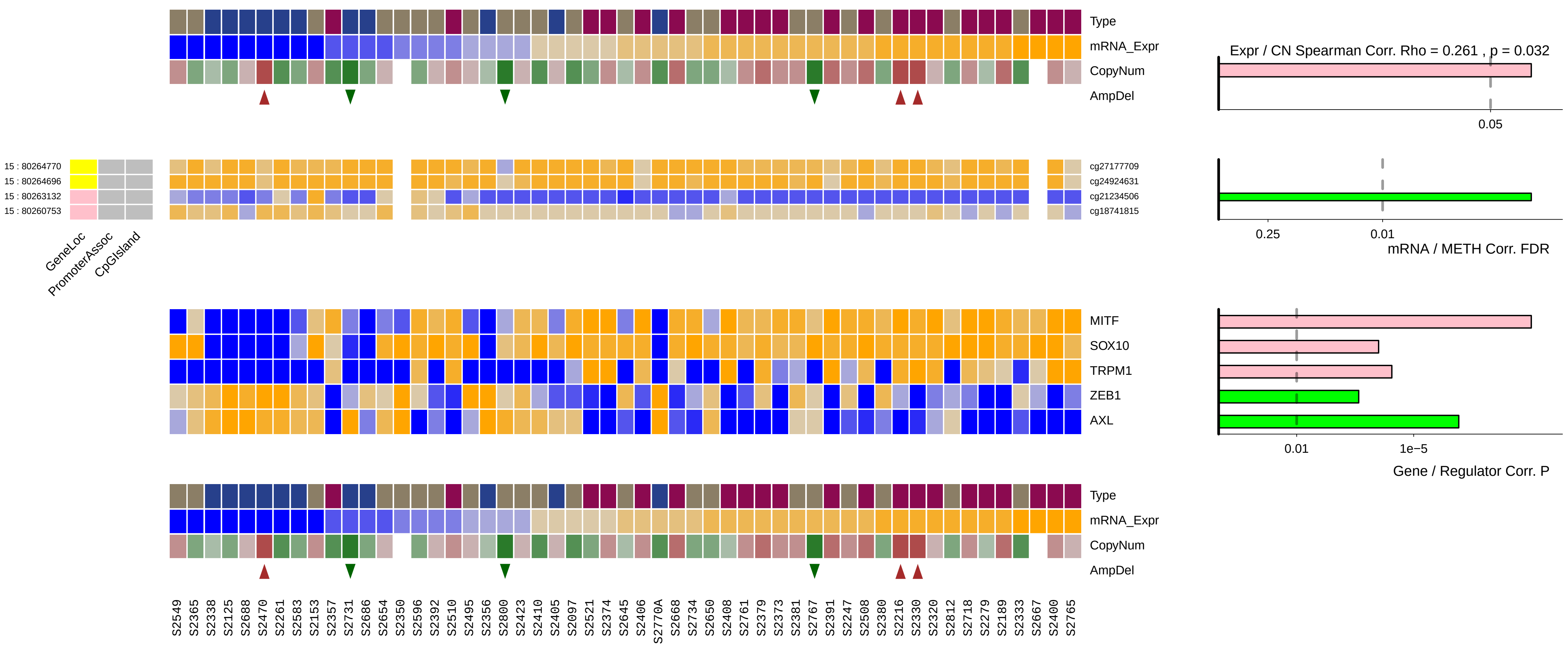
Type
mRNA_Expr
CopyNum
AmpDel
Expr / CN Spearman Corr. Rho = 0.261 , p = 0.032
0.05
15 : 80264770
15 : 80264696
15 : 80263132
15 : 80260753
cg27177709
cg24924631
cg21234506
cg18741815
GeneLoc
PromoterAssoc
CpGIsland
0.25
0.01
mRNA / METH Corr. FDR
MITF
SOX10
TRPM1
ZEB1
AXL
0.01
1e−5
Gene / Regulator Corr. P
S2549 S2365 S2338 S2125 S2688 S2470 S2261 S2583 S2153 S2357 S2731 S2686 S2654 S2350 S2596 S2392 S2510 S2495 S2356 S2800 S2423 S2410 S2405 S2097 S2521 S2374 S2645 S2406 S2770A S2668 S2734 S2650 S2408 S2761 S2379 S2373 S2381 S2767 S2391 S2247 S2508 S2380 S2216 S2330 S2320 S2812 S2718 S2279 S2189 S2333 S2667 S2400 S2765

SNCA

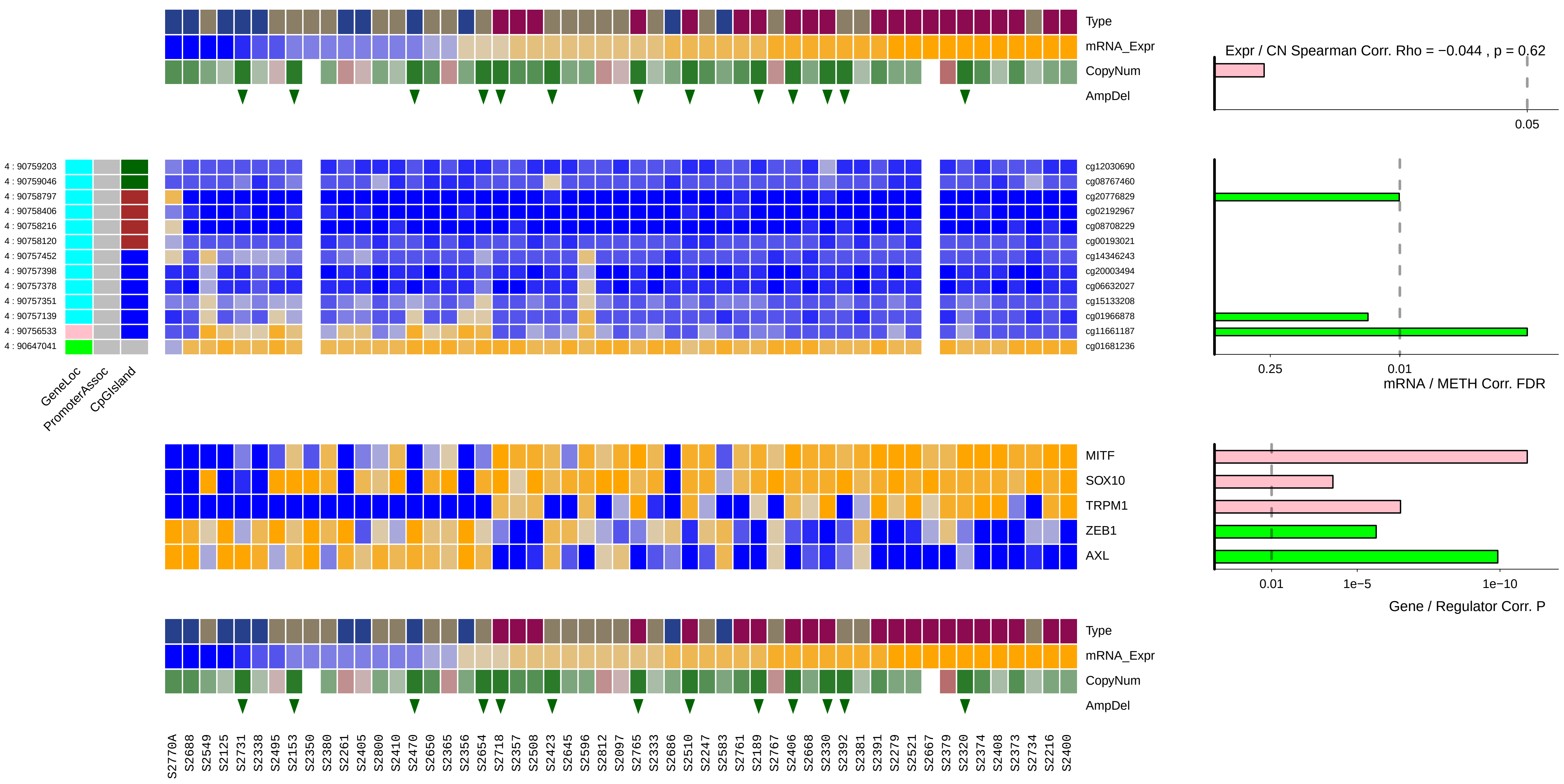
Type
mRNA_Expr
CopyNum
AmpDel
Expr / CN Spearman Corr. Rho = −0.044 , p = 0.62
0.05
4 : 90759203
4 : 90759046
4 : 90758797
4 : 90758406
4 : 90758216
4 : 90758120
4 : 90757452
4 : 90757398
4 : 90757378
4 : 90757351
4 : 90757139
4 : 90756533
4 : 90647041
cg12030690
cg08767460
cg20776829
cg02192967
cg08708229
cg00193021
cg14346243
cg20003494
cg06632027
cg15133208
cg01966878
cg11661187
cg01681236
GeneLoc
PromoterAssoc
CpGIsland
0.25
0.01
mRNA / METH Corr. FDR
MITF
SOX10
TRPM1
ZEB1
AXL
0.01
1e−5
1e−10
Gene / Regulator Corr. P
Type
mRNA_Expr
CopyNum
AmpDel
S2770A S2688 S2549 S2125 S2731 S2338 S2495 S2153 S2350 S2380 S2261 S2405 S2800 S2410 S2470 S2650 S2365 S2356 S2654 S2718 S2357 S2508 S2423 S2645 S2596 S2812 S2097 S2765 S2333 S2686 S2510 S2247 S2583 S2761 S2189 S2767 S2406 S2668 S2330 S2392 S2381 S2391 S2279 S2521 S2667 S2379 S2320 S2374 S2408 S2373 S2734 S2216 S2400

BIRC7

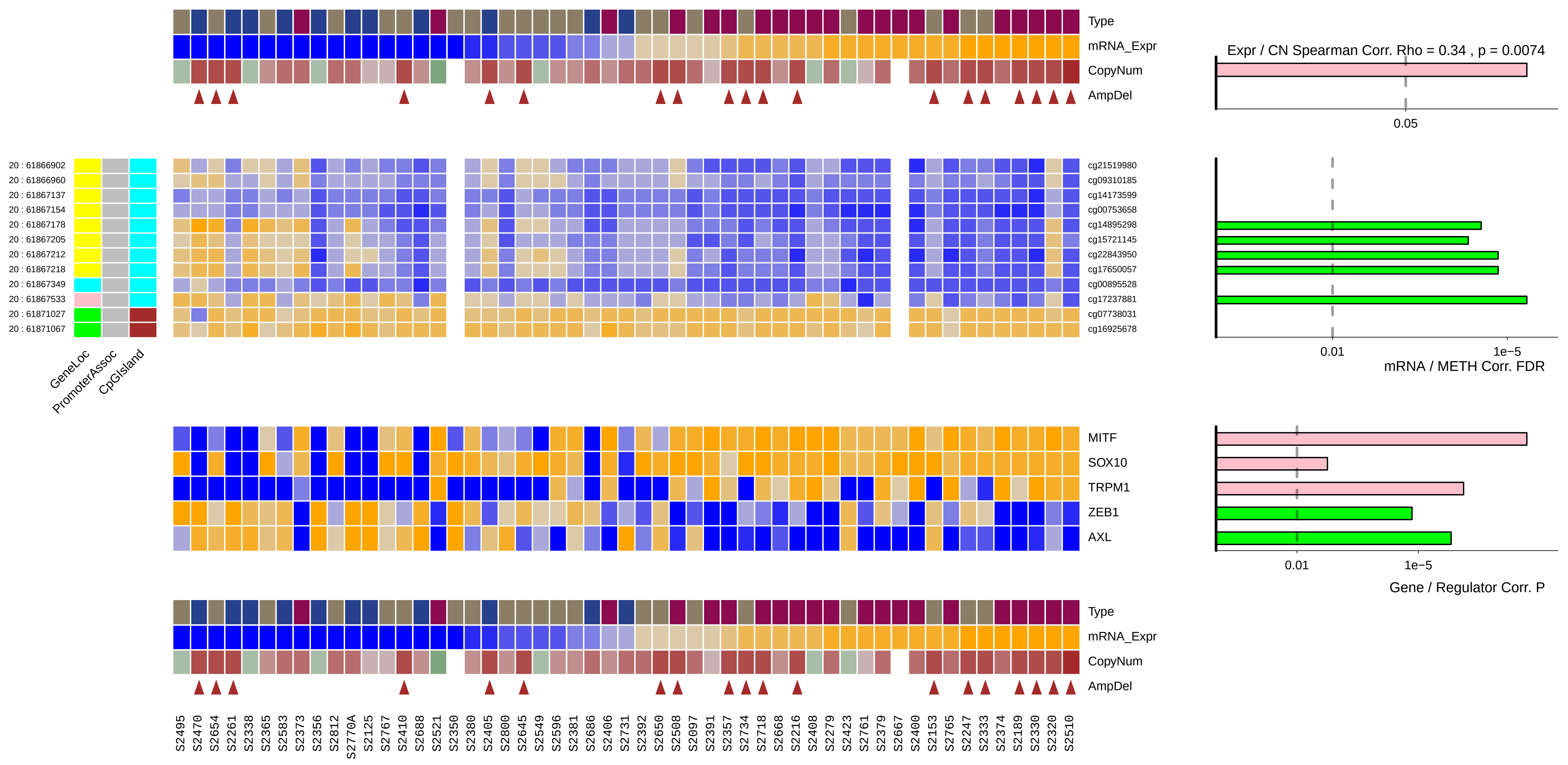
Type
mRNA_Expr
CopyNum
AmpDel
Expr / CN Spearman Corr. Rho = 0.34 , p = 0.0074
0.05
20 : 61866902
20 : 61866960
20 : 61867137
20 : 61867154
20 : 61867178
20 : 61867205
20 : 61867212
20 : 61867218
20 : 61867349
20 : 61867533
20 : 61871027
20 : 61871067
GeneLoc
PromoterAssoc
CpGIsland
cg21519980
cg09310185
cg14173599
cg00753658
cg14895298
cg15721145
cg22843950
cg17650057
cg00895528
cg17237881
cg07738031
cg16925678
0.01
1e−5
mRNA / METH Corr. FDR
MITF
SOX10
TRPM1
ZEB1
AXL
0.01
1e−5
Gene / Regulator Corr. P
Type
mRNA_Expr
CopyNum
AmpDel
S2495 S2470 S2654 S2261 S2338 S2365 S2583 S2373 S2356 S2812 S2770A S2125 S2767 S2410 S2688 S2521 S2350 S2380 S2405 S2800 S2645 S2549 S2596 S2381 S2686 S2406 S2731 S2392 S2650 S2508 S2097 S2391 S2357 S2734 S2718 S2668 S2216 S2408 S2279 S2423 S2761 S2379 S2667 S2400 S2153 S2765 S2247 S2333 S2374 S2189 S2330 S2320 S2510

MFSD12

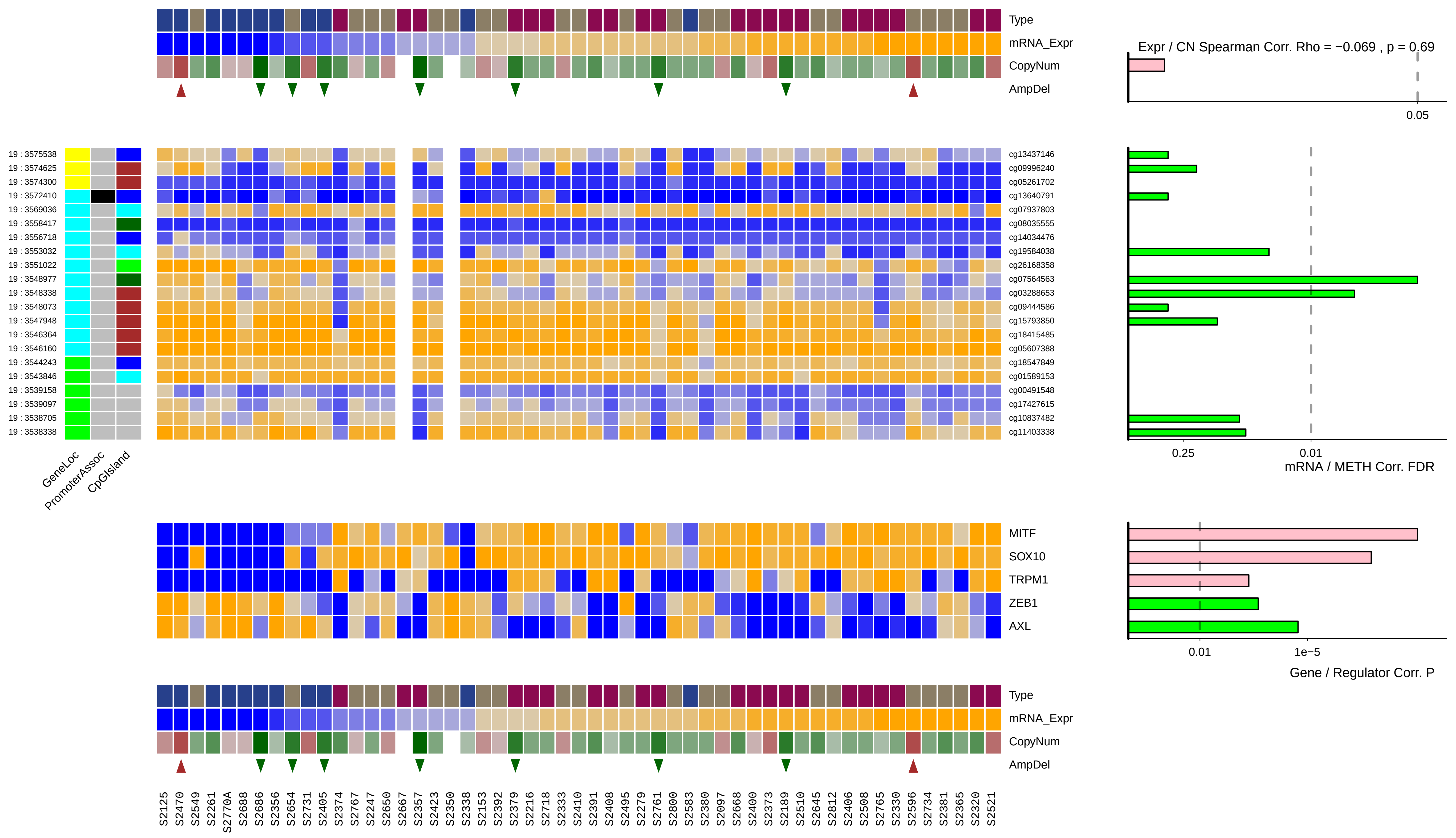
Type
mRNA_Expr
CopyNum
AmpDel
Expr / CN Spearman Corr. Rho = −0.069 , p = 0.69
0.05
mRNA / METH Corr. FDR
0.25
0.01
GeneLoc
PromoterAssoc
CpGIsland
MITF
SOX10
TRPM1
ZEB1
AXL
Gene / Regulator Corr. P
0.01
1e−5

SGK1

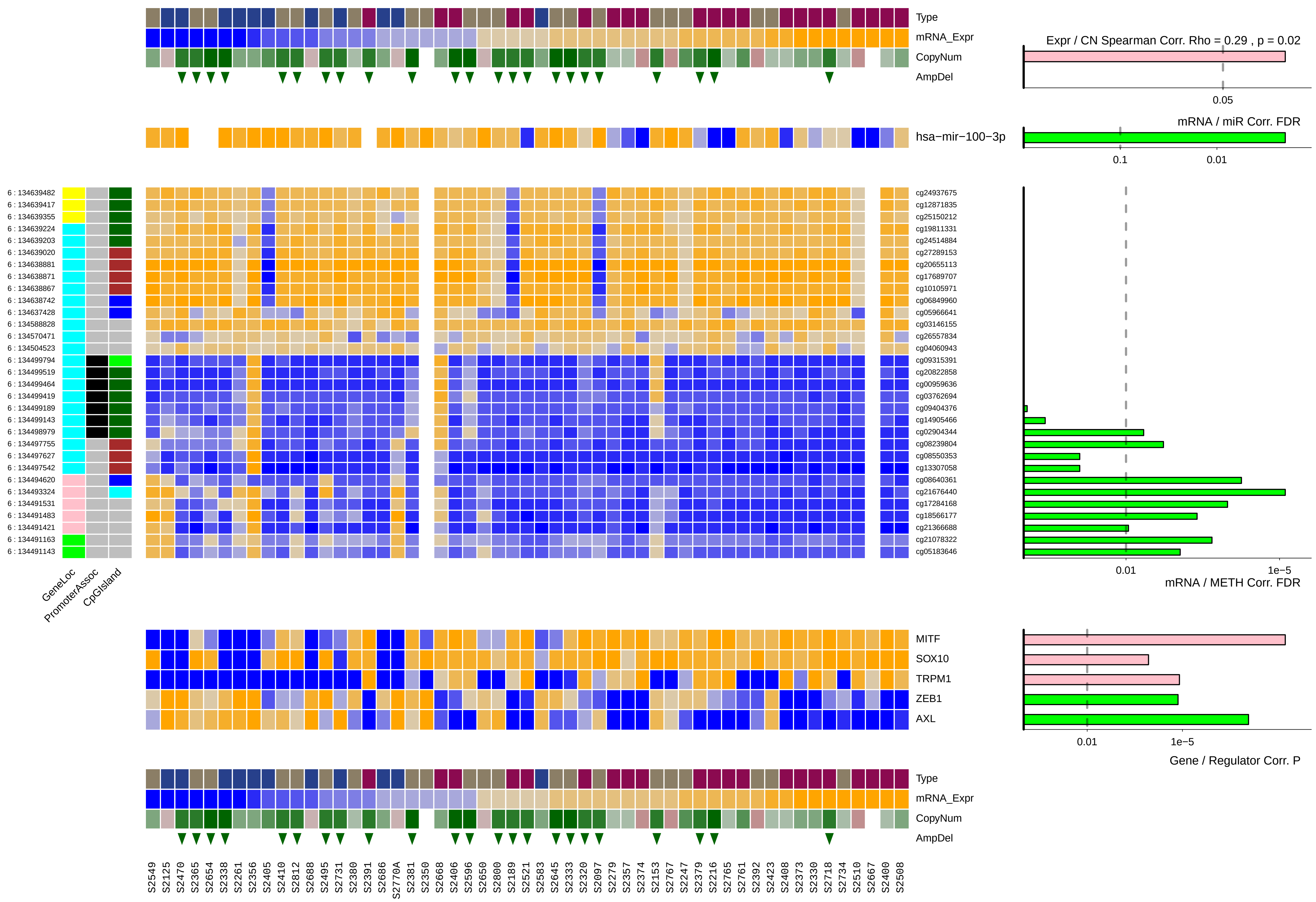
Type
mRNA_Expr
CopyNum
AmpDel
Expr / CN Spearman Corr. Rho = 0.29 , p = 0.02
0.05
mRNA / miR Corr. FDR
hsa−mir−100−3p
0.1
0.01
6 : 134639482
6 : 134639417
6 : 134639355
6 : 134639224
6 : 134639203
6 : 134639020
6 : 134638881
6 : 134638871
6 : 134638867
6 : 134638742
6 : 134637428
6 : 134588828
6 : 134570471
6 : 134504523
6 : 134499794
6 : 134499519
6 : 134499464
6 : 134499419
6 : 134499189
6 : 134499143
6 : 134498979
6 : 134497755
6 : 134497627
6 : 134497542
6 : 134494620
6 : 134493324
6 : 134491531
6 : 134491483
6 : 134491421
6 : 134491163
6 : 134491143
cg24937675
cg12871835
cg25150212
cg19811331
cg24514884
cg27289153
cg20655113
cg17689707
cg10105971
cg06849960
cg05966641
cg03146155
cg26557834
cg04060943
cg09315391
cg20822858
cg00959636
cg03762694
cg09404376
cg14905466
cg02904344
cg08239804
cg08550353
cg13307058
cg08640361
cg21676440
cg17284168
cg18566177
cg21366688
cg21078322
cg05183646
0.01
1e−5
mRNA / METH Corr. FDR
GeneLoc
PromoterAssoc
CpGIsland
MITF
SOX10
TRPM1
ZEB1
AXL
0.01
1e−5
Gene / Regulator Corr. P
Type
mRNA_Expr
CopyNum
AmpDel
S2549 S2125 S2470 S2365 S2654 S2338 S2261 S2356 S2405 S2410 S2812 S2688 S2495 S2731 S2380 S2391 S2686 S2770A S2381 S2350 S2668 S2406 S2596 S2650 S2800 S2189 S2521 S2583 S2645 S2333 S2320 S2097 S2279 S2357 S2374 S2153 S2767 S2247 S2379 S2216 S2765 S2761 S2392 S2423 S2408 S2373 S2330 S2718 S2734 S2510 S2667 S2400 S2508

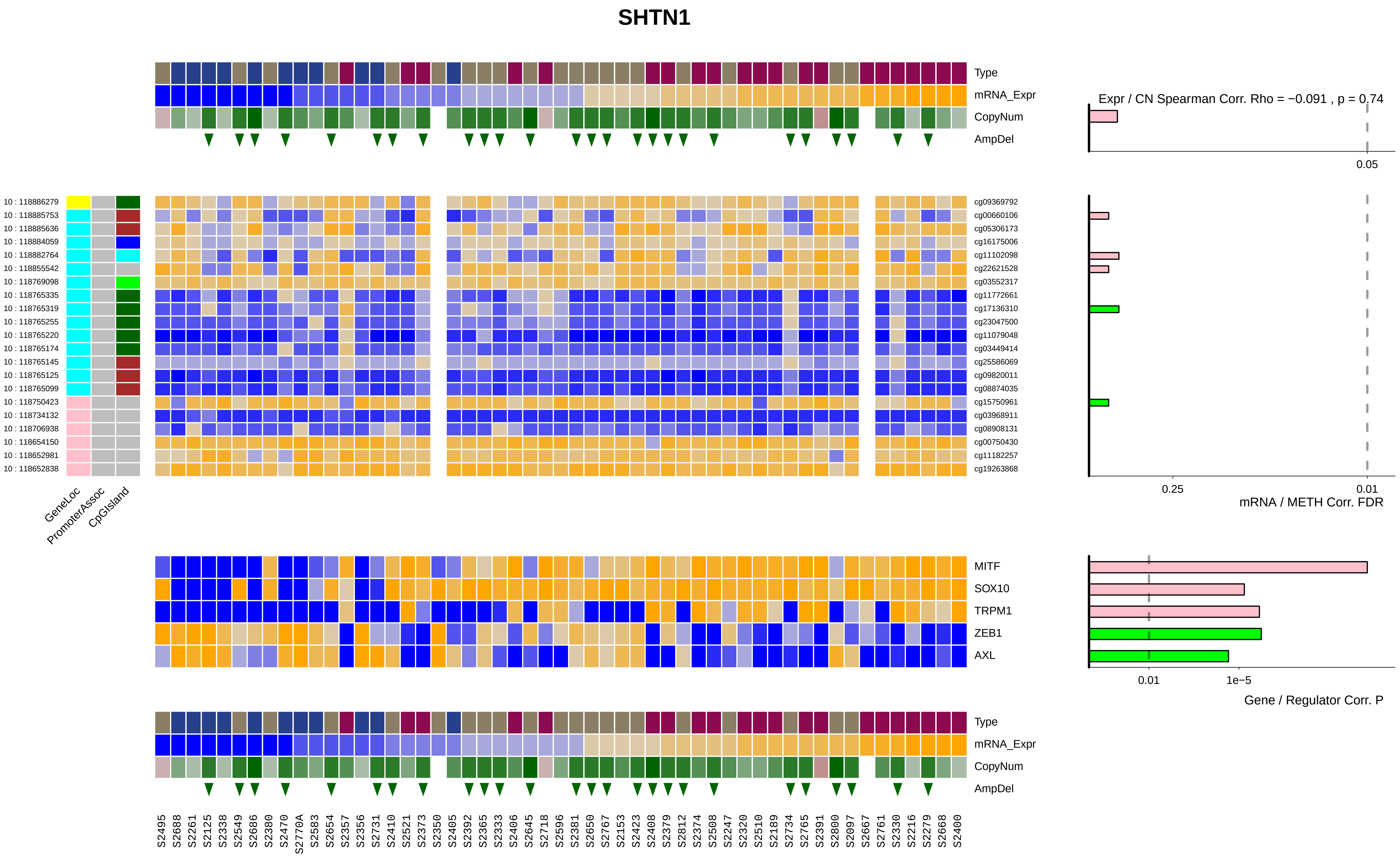
SHTN1
Type
mRNA_Expr
CopyNum
AmpDel
Expr / CN Spearman Corr. Rho = −0.091 , p = 0.74
0.05
10 : 118886279
10 : 118885753
10 : 118885636
10 : 118884059
10 : 118882764
10 : 118855542
10 : 118769098
10 : 118765335
10 : 118765319
10 : 118765255
10 : 118765220
10 : 118765174
10 : 118765145
10 : 118765125
10 : 118765099
10 : 118750423
10 : 118734132
10 : 118706938
10 : 118654150
10 : 118652981
10 : 118652838
cg09369792
cg00660106
cg05306173
cg16175006
cg11102098
cg22621528
cg03552317
cg11772661
cg17136310
cg23047500
cg11079048
cg03449414
cg25586069
cg09820011
cg08874035
cg15750961
cg03968911
cg08908131
cg00750430
cg11182257
cg19263868
GeneLoc
PromoterAssoc
CpGIsland
0.25
0.01
mRNA / METH Corr. FDR
MITF
SOX10
TRPM1
ZEB1
AXL
0.01
1e−5
Gene / Regulator Corr. P
Type
mRNA_Expr
CopyNum
AmpDel
S2495 S2688 S2261 S2125 S2338 S2549 S2686 S2380 S2470 S2770A S2583 S2654 S2357 S2356 S2731 S2410 S2521 S2373 S2350 S2405 S2392 S2365 S2333 S2406 S2645 S2718 S2596 S2381 S2650 S2767 S2153 S2423 S2408 S2379 S2812 S2374 S2508 S2247 S2320 S2510 S2189 S2734 S2765 S2391 S2800 S2097 S2667 S2761 S2330 S2216 S2279 S2668 S2400

RNF144A

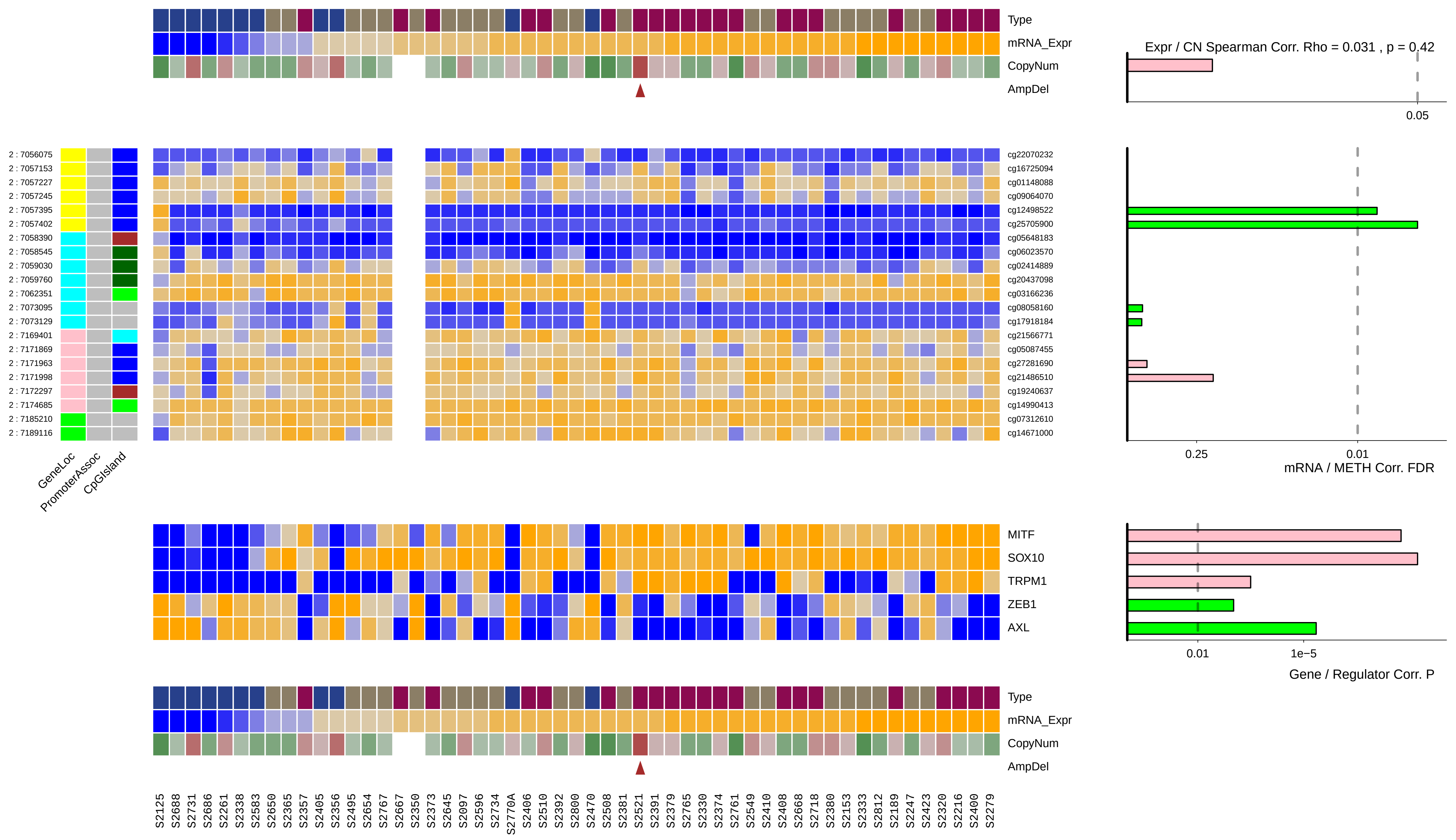
Type
mRNA_Expr
CopyNum
AmpDel
Expr / CN Spearman Corr. Rho = 0.031 , p = 0.42
0.05
2 : 7056075
2 : 7057153
2 : 7057227
2 : 7057245
2 : 7057395
2 : 7057402
2 : 7058390
2 : 7058545
2 : 7059030
2 : 7059760
2 : 7062351
2 : 7073095
2 : 7073129
2 : 7169401
2 : 7171869
2 : 7171963
2 : 7171998
2 : 7172297
2 : 7174685
2 : 7185210
2 : 7189116
cg22070232
cg16725094
cg01148088
cg09064070
cg12498522
cg25705900
cg05648183
cg06023570
cg02414889
cg20437098
cg03166236
cg08058160
cg17918184
cg21566771
cg05087455
cg27281690
cg21486510
cg19240637
cg14990413
cg07312610
cg14671000
0.25
0.01
mRNA / METH Corr. FDR
GeneLoc
PromoterAssoc
CpGIsland
MITF
SOX10
TRPM1
ZEB1
AXL
0.01
1e−5
Gene / Regulator Corr. P
Type
mRNA_Expr
CopyNum
AmpDel
S2125 S2688 S2731 S2686 S2261 S2338 S2583 S2650 S2365 S2357 S2405 S2356 S2495 S2654 S2767 S2667 S2350 S2373 S2645 S2097 S2596 S2734 S2770A S2406 S2510 S2392 S2800 S2470 S2508 S2381 S2521 S2391 S2379 S2765 S2330 S2374 S2761 S2549 S2410 S2408 S2668 S2718 S2380 S2153 S2333 S2812 S2189 S2247 S2423 S2320 S2216 S2400 S2279

RUNX3

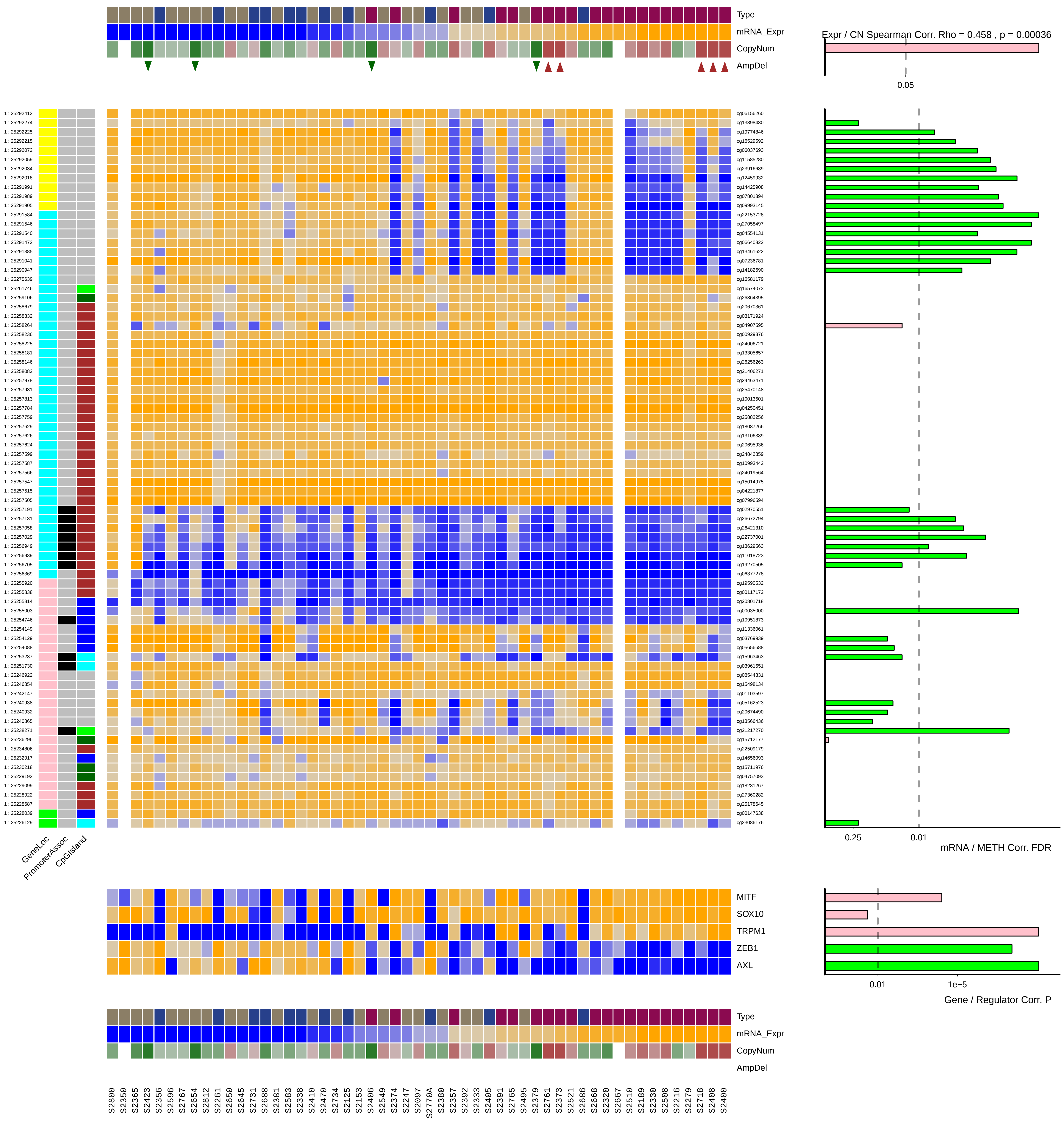

Type
mRNA_Expr
CopyNum
AmpDel
Expr / CN Spearman Corr. Rho = 0.458 , p = 0.00036
0.05
0.25
0.01
mRNA / METH Corr. FDR
GeneLoc
PromoterAssoc
CpGIsland
MITF
SOX10
TRPM1
ZEB1
AXL
0.01
1e−5
Gene / Regulator Corr. P
Type
mRNA_Expr
CopyNum
AmpDel
S2800 S2350 S2365 S2423 S2356 S2596 S2767 S2654 S2812 S2261 S2650 S2645 S2731 S2688 S2381 S2583 S2338 S2410 S2470 S2734 S2125 S2153 S2406 S2549 S2374 S2247 S2097 S2770A S2380 S2357 S2392 S2333 S2405 S2391 S2765 S2495 S2379 S2761 S2373 S2521 S2686 S2668 S2320 S2667 S2510 S2189 S2330 S2508 S2216 S2279 S2718 S2408 S2400

IGSF3

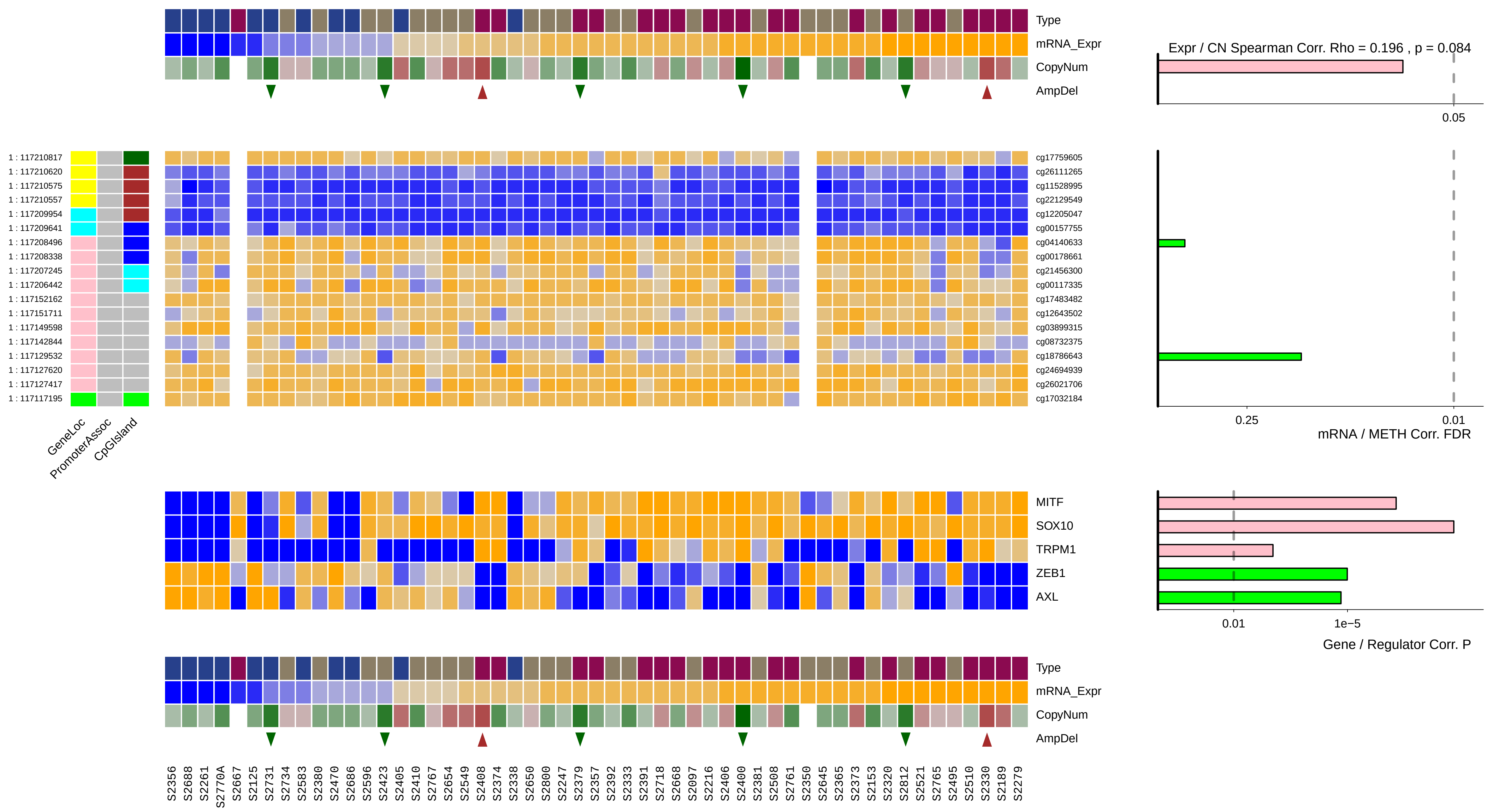

Type
mRNA_Expr
CopyNum
AmpDel
Expr / CN Spearman Corr. Rho = 0.196 , p = 0.084
0.05
GeneLoc
PromoterAssoc
CpGIsland
0.25
0.01
mRNA / METH Corr. FDR
MITF
SOX10
TRPM1
ZEB1
AXL
0.01
1e−5
Gene / Regulator Corr. P

ST3GAL6

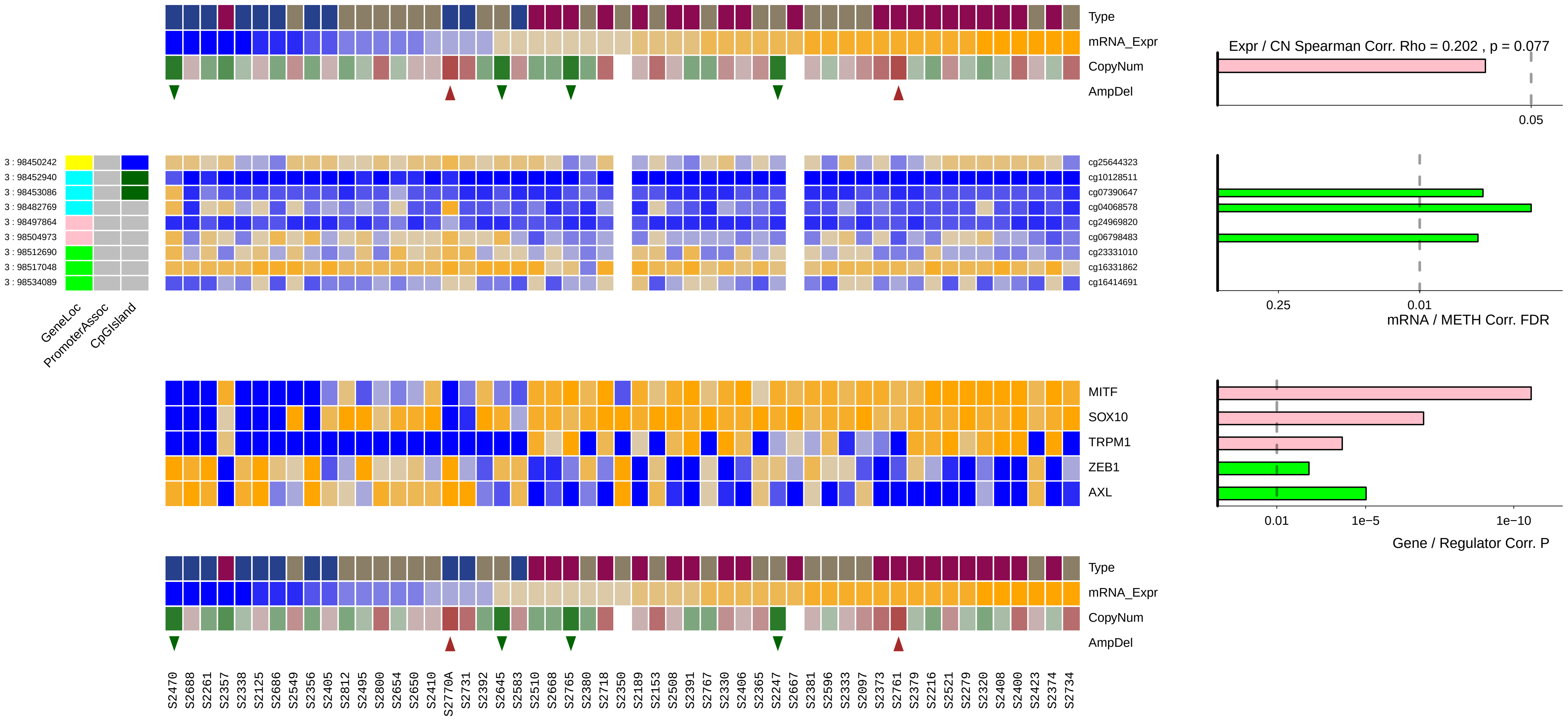

Type
mRNA_Expr
CopyNum
AmpDel
Expr / CN Spearman Corr. Rho = 0.202 , p = 0.077
0.05
3 : 98450242
3 : 98452940
3 : 98453086
3 : 98482769
3 : 98497864
3 : 98504973
3 : 98512690
3 : 98517048
3 : 98534089
GeneLoc
PromoterAssoc
CpGIsland
cg25644323
cg10128511
cg07390647
cg04068578
cg24969820
cg06798483
cg23331010
cg16331862
cg16414691
0.25
0.01
mRNA / METH Corr. FDR
MITF
SOX10
TRPM1
ZEB1
AXL
0.01
1e−5
1e−10
Gene / Regulator Corr. P
S2470 S2688 S2261 S2357 S2338 S2125 S2686 S2549 S2356 S2405 S2812 S2495 S2800 S2654 S2650 S2410 S2770A S2731 S2392 S2645 S2583 S2510 S2668 S2765 S2380 S2718 S2350 S2189 S2153 S2508 S2391 S2767 S2330 S2406 S2365 S2247 S2667 S2381 S2596 S2333 S2097 S2373 S2761 S2379 S2216 S2521 S2279 S2320 S2408 S2400 S2423 S2374 S2734

GAS7

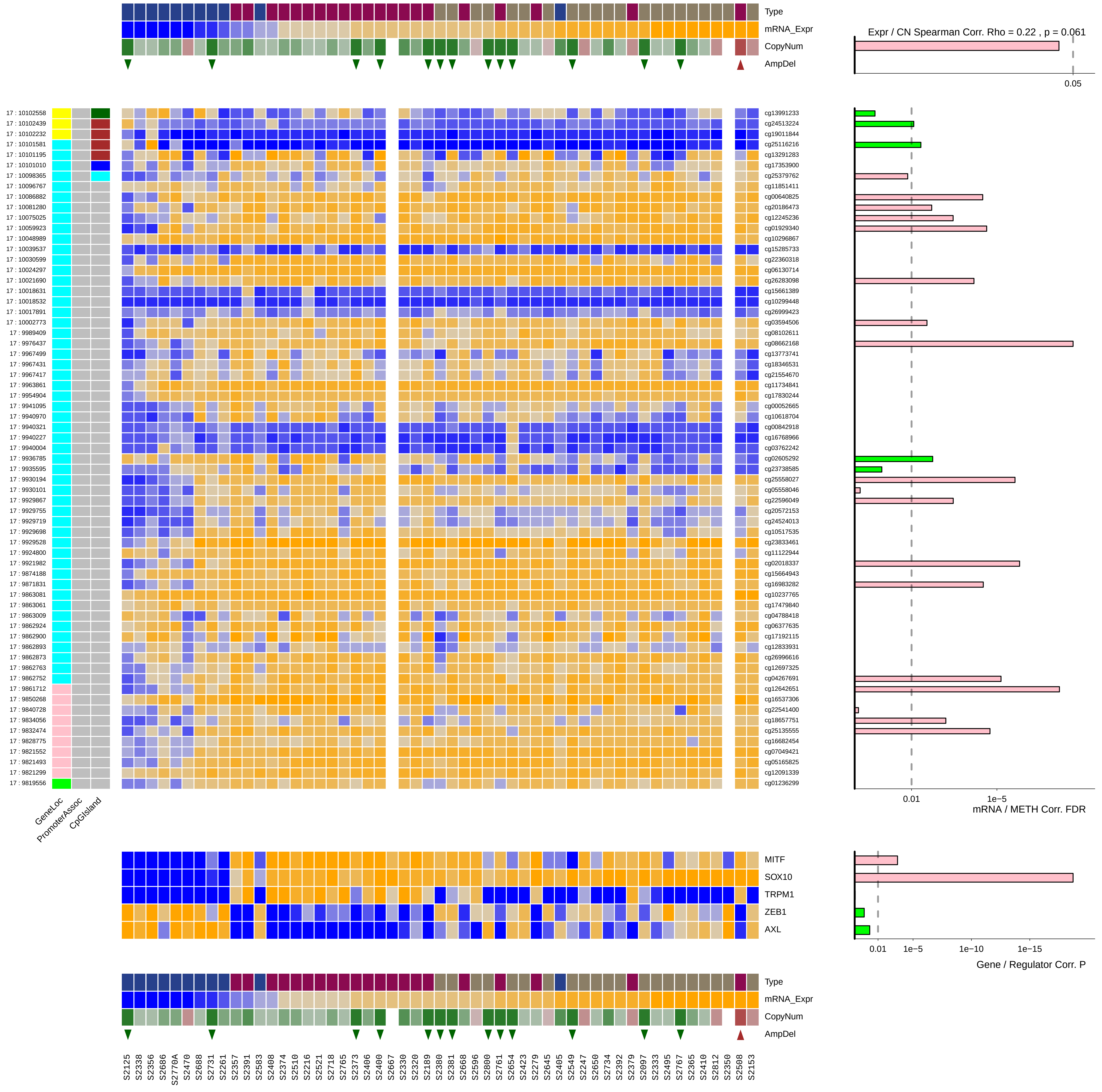

Type
mRNA_Expr
CopyNum
AmpDel
Expr / CN Spearman Corr. Rho = 0.22 , p = 0.061
0.05
17 : 10102558
17 : 10102439
17 : 10102232
17 : 10101581
17 : 10101195
17 : 10101010
17 : 10098365
17 : 10096767
17 : 10086882
17 : 10081280
17 : 10075025
17 : 10059923
17 : 10048989
17 : 10039537
17 : 10030599
17 : 10024297
17 : 10021690
17 : 10018631
17 : 10018532
17 : 10017891
17 : 10002773
17 : 9989409
17 : 9976437
17 : 9967499
17 : 9967431
17 : 9967417
17 : 9963861
17 : 9954904
17 : 9941095
17 : 9940970
17 : 9940321
17 : 9940227
17 : 9940004
17 : 9936785
17 : 9935595
17 : 9930194
17 : 9930101
17 : 9929867
17 : 9929755
17 : 9929719
17 : 9929698
17 : 9929528
17 : 9924800
17 : 9921982
17 : 9874188
17 : 9871831
17 : 9863081
17 : 9863061
17 : 9863009
17 : 9862924
17 : 9862900
17 : 9862893
17 : 9862873
17 : 9862763
17 : 9862752
17 : 9861712
17 : 9850268
17 : 9840728
17 : 9834056
17 : 9832474
17 : 9828775
17 : 9821552
17 : 9821493
17 : 9821299
17 : 9819556
cg13991233
cg24513224
cg19011844
cg25116216
cg13291283
cg17353900
cg25379762
cg11851411
cg00640825
cg20186473
cg12245236
cg01929340
cg10296867
cg15285733
cg22360318
cg06130714
cg26283098
cg15661389
cg10299448
cg26999423
cg03594506
cg08102611
cg08662168
cg13773741
cg18346531
cg21554670
cg11734841
cg17830244
cg00052665
cg10618704
cg00842918
cg16768966
cg03762242
cg02605292
cg23738585
cg25558027
cg05558046
cg22596049
cg20572153
cg24524013
cg10517535
cg23833461
cg11122944
cg02018337
cg15664943
cg16983282
cg10237765
cg17479840
cg04788418
cg06377635
cg17192115
cg12833931
cg26996616
cg12697325
cg04267691
cg12642651
cg16537306
cg22541400
cg18657751
cg25135555
cg16682454
cg07049421
cg05165825
cg12091339
cg01236299
GeneLoc
PromoterAssoc
CpGIsland
0.01
1e−5
mRNA / METH Corr. FDR
MITF
SOX10
TRPM1
ZEB1
AXL
0.01
1e−5
1e−10
1e−15
Gene / Regulator Corr. P
Type
mRNA_Expr
CopyNum
AmpDel
S2125
S2338
S2356
S2686
S2770A
S2470
S2688
S2731
S2261
S2357
S2391
S2583
S2408
S2374
S2510
S2216
S2521
S2718
S2765
S2373
S2406
S2400
S2667
S2330
S2320
S2189
S2380
S2381
S2668
S2596
S2800
S2761
S2654
S2423
S2279
S2645
S2405
S2549
S2247
S2650
S2734
S2392
S2379
S2097
S2333
S2495
S2767
S2365
S2410
S2812
S2350
S2508
S2153

AP1S2

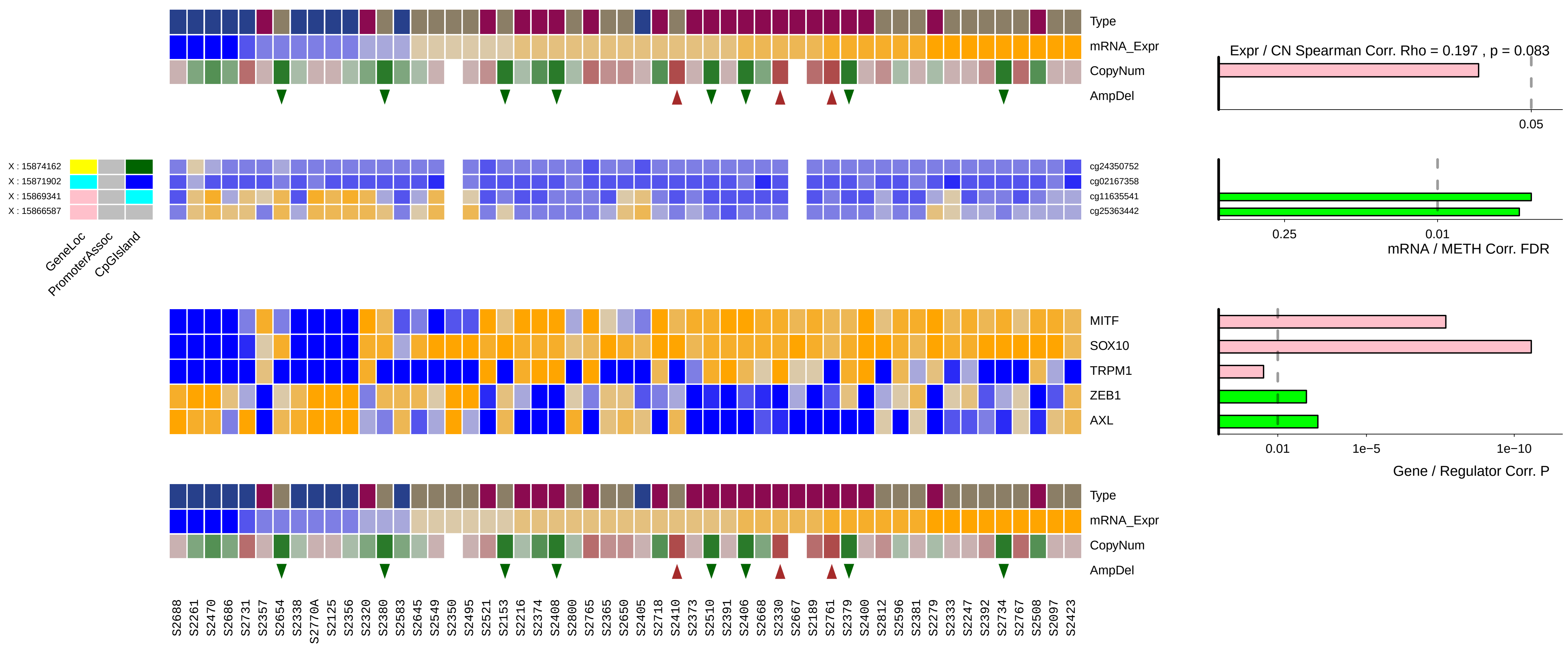

Type
mRNA_Expr
CopyNum
AmpDel
Expr / CN Spearman Corr. Rho = 0.197 , p = 0.083
0.05
X : 15874162
X : 15871902
X : 15869341
X : 15866587
GeneLoc
PromoterAssoc
CpGIsland
cg24350752
cg02167358
cg11635541
cg25363442
0.25
0.01
mRNA / METH Corr. FDR
MITF
SOX10
TRPM1
ZEB1
AXL
0.01
1e−5
1e−10
Gene / Regulator Corr. P
Type
mRNA_Expr
CopyNum
AmpDel
S2688 S2261 S2470 S2686 S2731 S2357 S2654 S2338 S2770A S2125 S2356 S2320 S2380 S2583 S2645 S2549 S2350 S2495 S2521 S2153 S2216 S2374 S2408 S2800 S2765 S2365 S2650 S2405 S2718 S2410 S2373 S2510 S2391 S2406 S2668 S2330 S2667 S2189 S2761 S2379 S2400 S2812 S2596 S2381 S2279 S2333 S2247 S2392 S2734 S2767 S2508 S2097 S2423

FCRLA

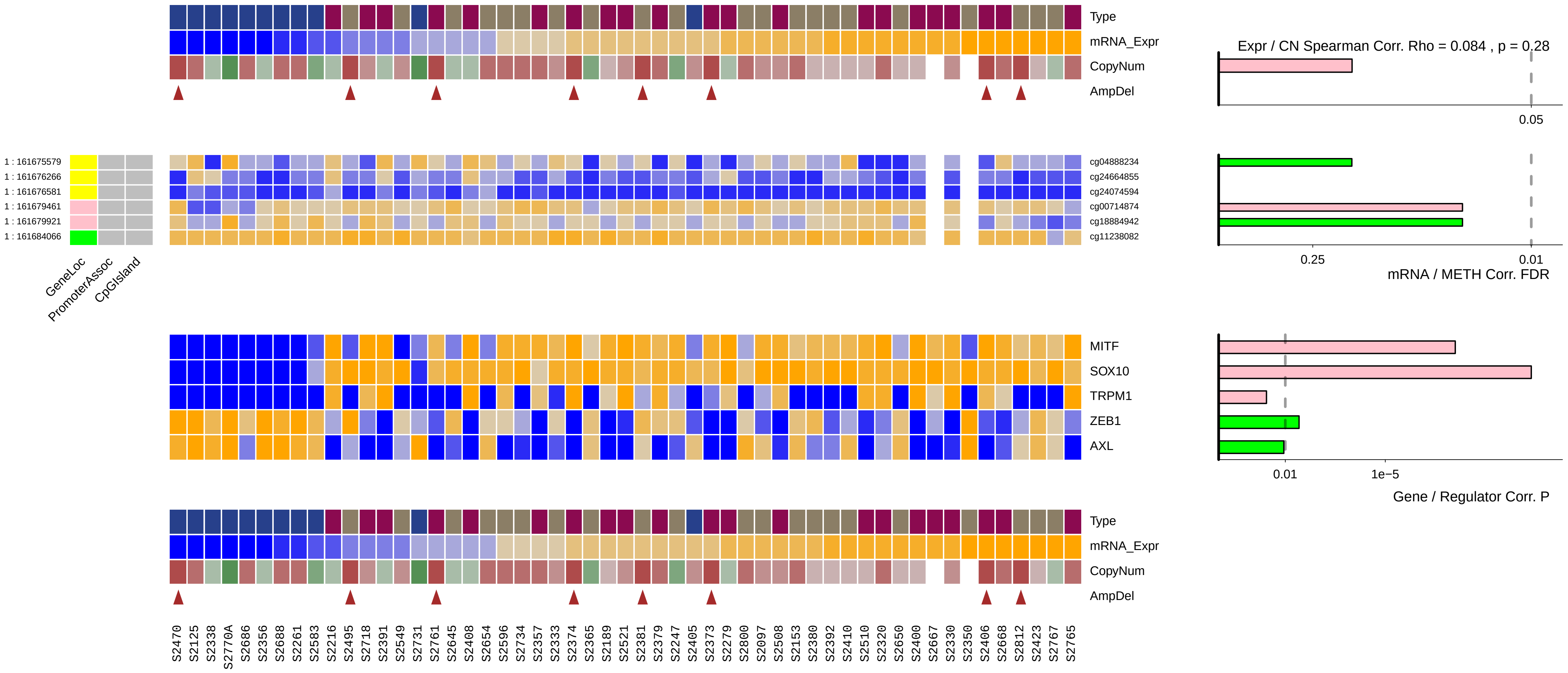
Type
mRNA_Expr
CopyNum
AmpDel
Expr / CN Spearman Corr. Rho = 0.084 , p = 0.28
0.05
1 : 161675579
1 : 161676266
1 : 161676581
1 : 161679461
1 : 161679921
1 : 161684066
GeneLoc
PromoterAssoc
CpGIsland
cg04888234
cg24664855
cg24074594
cg00714874
cg18884942
cg11238082
0.25
0.01
mRNA / METH Corr. FDR
MITF
SOX10
TRPM1
ZEB1
AXL
0.01
1e−5
Gene / Regulator Corr. P
S2470 S2125 S2338 S2770A S2686 S2356 S2688 S2261 S2583 S2216 S2495 S2718 S2391 S2549 S2731 S2761 S2645 S2408 S2654 S2596 S2734 S2357 S2333 S2374 S2365 S2189 S2521 S2381 S2379 S2247 S2405 S2373 S2279 S2800 S2097 S2508 S2153 S2380 S2392 S2410 S2510 S2320 S2650 S2400 S2667 S2330 S2350 S2406 S2668 S2812 S2423 S2767 S2765

TSPAN10

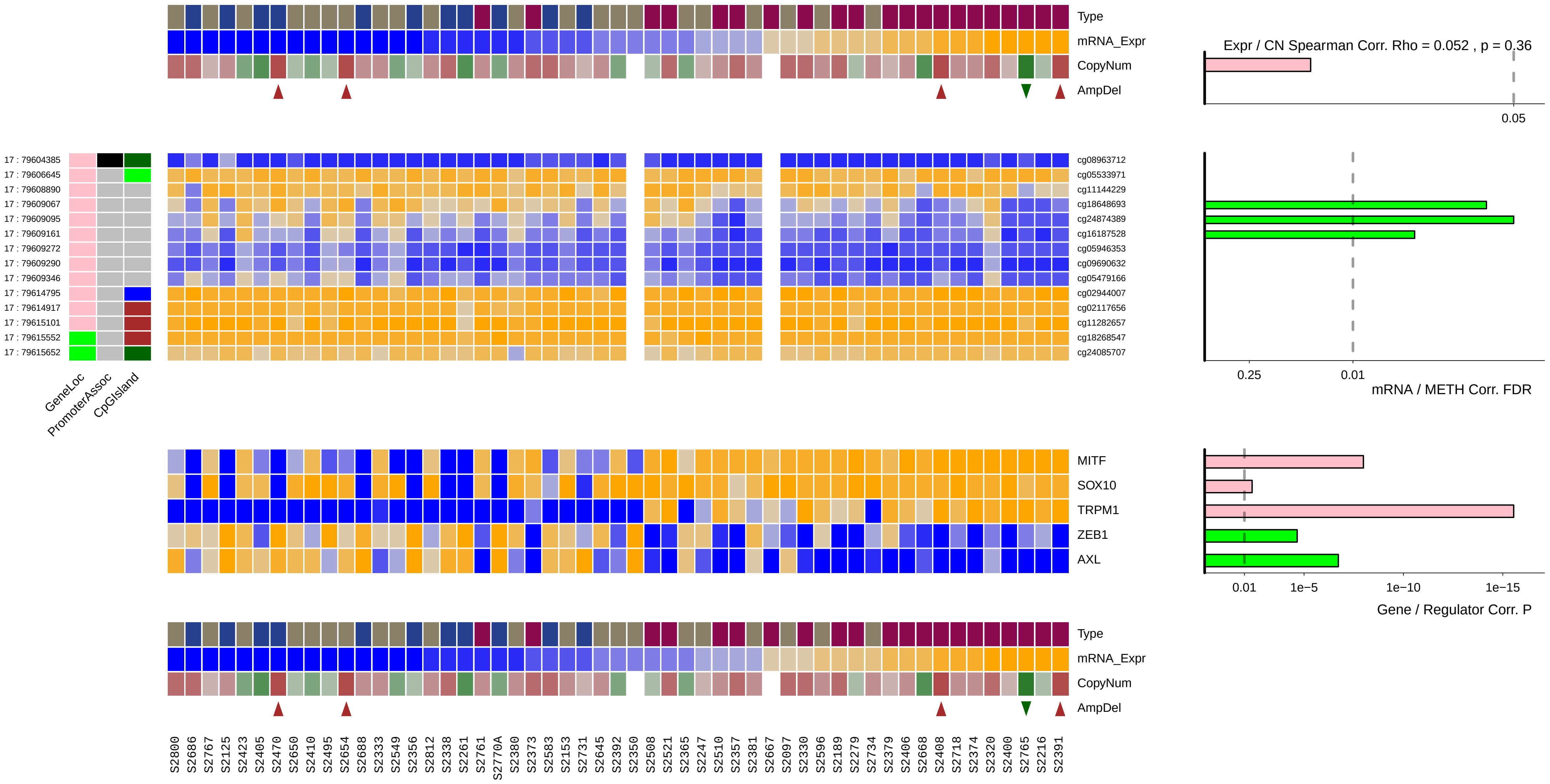

Type
mRNA_Expr
CopyNum
AmpDel
Expr / CN Spearman Corr. Rho = 0.052 , p = 0.36
0.05
cg08963712
cg05533971
cg11144229
cg18648693
cg24874389
cg16187528
cg05946353
cg09690632
cg05479166
cg02944007
cg02117656
cg11282657
cg18268547
cg24085707
17 : 79604385
17 : 79606645
17 : 79608890
17 : 79609067
17 : 79609095
17 : 79609161
17 : 79609272
17 : 79609290
17 : 79609346
17 : 79614795
17 : 79614917
17 : 79615101
17 : 79615552
17 : 79615652
GeneLoc
PromoterAssoc
CpGIsland
0.25
0.01
mRNA / METH Corr. FDR
MITF
SOX10
TRPM1
ZEB1
AXL
0.01
1e−5
1e−10
1e−15
Gene / Regulator Corr. P
S2800 S2686 S2767 S2125 S2423 S2405 S2470 S2650 S2410 S2495 S2654 S2688 S2333 S2549 S2356 S2812 S2338 S2261 S2761 S2770A S2380 S2373 S2583 S2153 S2731 S2645 S2392 S2350 S2508 S2521 S2365 S2247 S2510 S2357 S2381 S2667 S2097 S2330 S2596 S2189 S2279 S2734 S2379 S2406 S2668 S2408 S2718 S2374 S2320 S2400 S2765 S2216 S2391

MCF2L

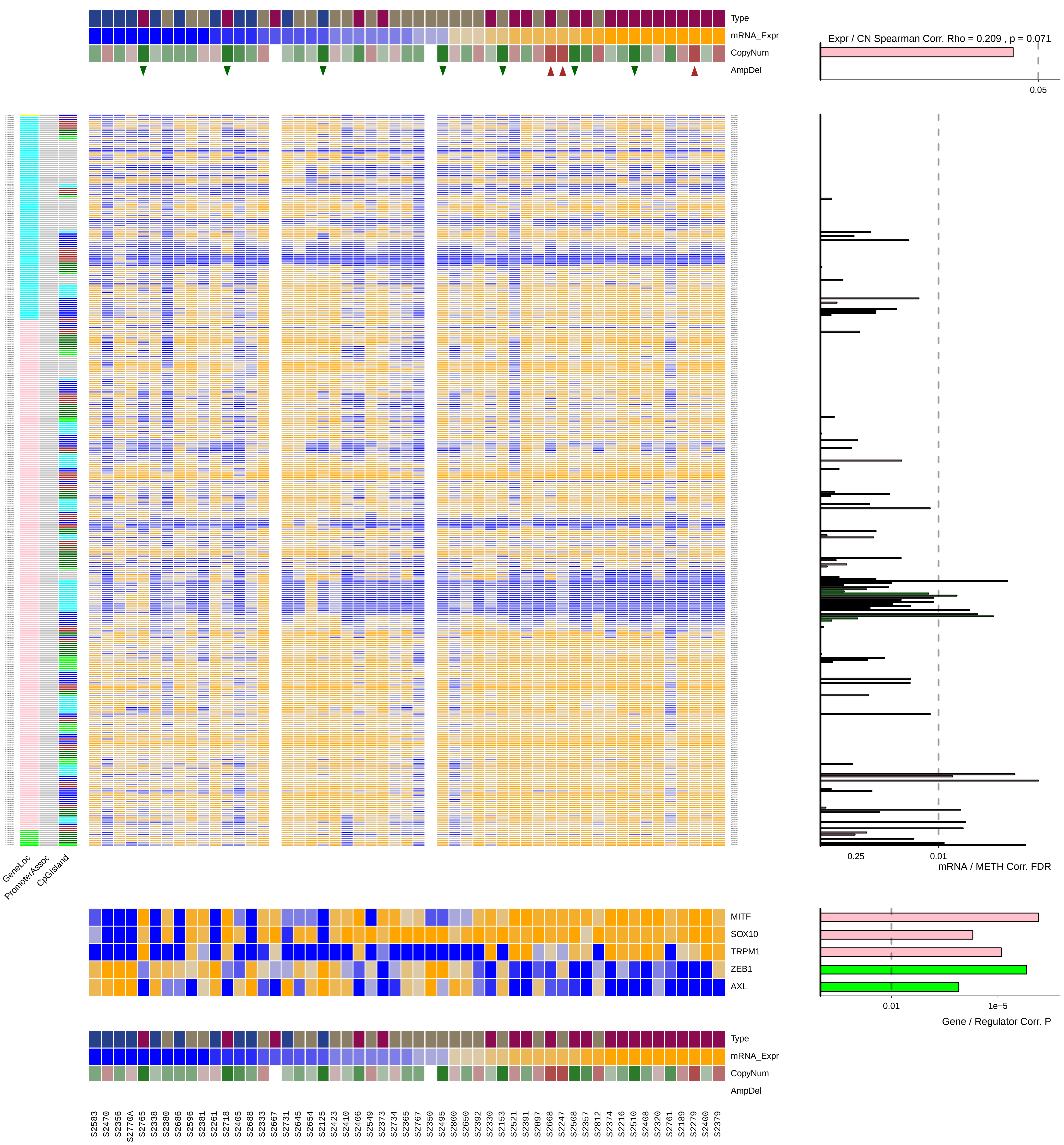

Type
mRNA_Expr
CopyNum
AmpDel
Expr / CN Spearman Corr. Rho = 0.209 , p = 0.071
0.05
GeneLoc
PromoterAssoc
CpGIsland
0.25
0.01
mRNA / METH Corr. FDR
MITF
SOX10
TRPM1
ZEB1
AXL
0.01
1e−5
Gene / Regulator Corr. P
S2583 S2470 S2356 S2770A S2765 S2338 S2380 S2686 S2596 S2381 S2261 S2718 S2405 S2688 S2333 S2667 S2731 S2645 S2654 S2125 S2423 S2410 S2406 S2549 S2373 S2734 S2365 S2767 S2350 S2495 S2800 S2650 S2392 S2330 S2153 S2521 S2391 S2097 S2668 S2247 S2508 S2357 S2812 S2374 S2216 S2510 S2408 S2320 S2761 S2189 S2279 S2400 S2379

CA14

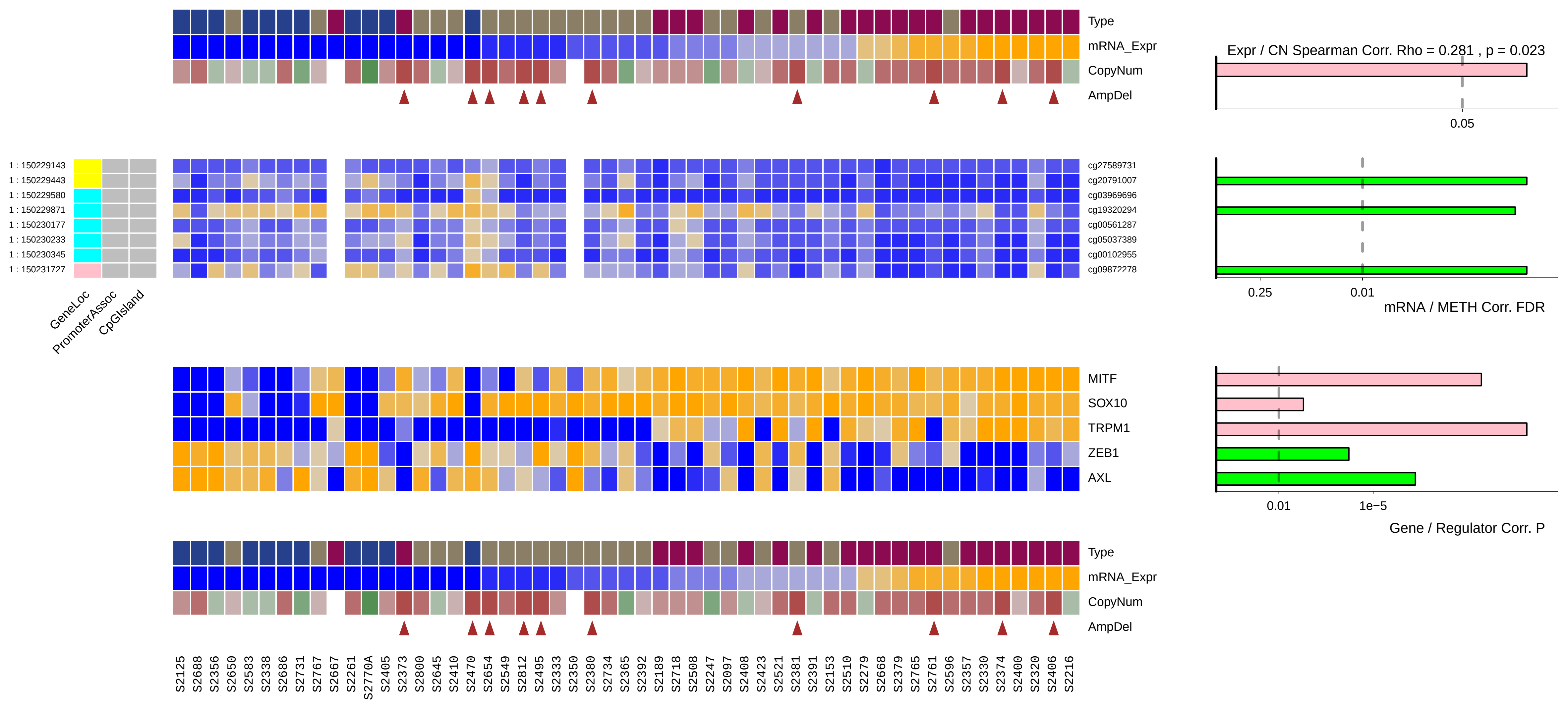

Type
mRNA_Expr
CopyNum
AmpDel
Expr / CN Spearman Corr. Rho = 0.281 , p = 0.023
0.05
1 : 150229143
1 : 150229443
1 : 150229580
1 : 150229871
1 : 150230177
1 : 150230233
1 : 150230345
1 : 150231727
GeneLoc
PromoterAssoc
CpGIsland
cg27589731
cg20791007
cg03969696
cg19320294
cg00561287
cg05037389
cg00102955
cg09872278
0.25
0.01
mRNA / METH Corr. FDR
MITF
SOX10
TRPM1
ZEB1
AXL
0.01
1e−5
Gene / Regulator Corr. P
S2125 S2688 S2356 S2650 S2583 S2338 S2686 S2731 S2767 S2667 S2261 S2770A S2405 S2373 S2800 S2645 S2410 S2470 S2654 S2549 S2812 S2495 S2333 S2350 S2380 S2734 S2365 S2392 S2189 S2718 S2508 S2247 S2097 S2408 S2423 S2521 S2381 S2391 S2153 S2510 S2279 S2668 S2379 S2765 S2761 S2596 S2357 S2330 S2374 S2400 S2320 S2406 S2216

TRIM2

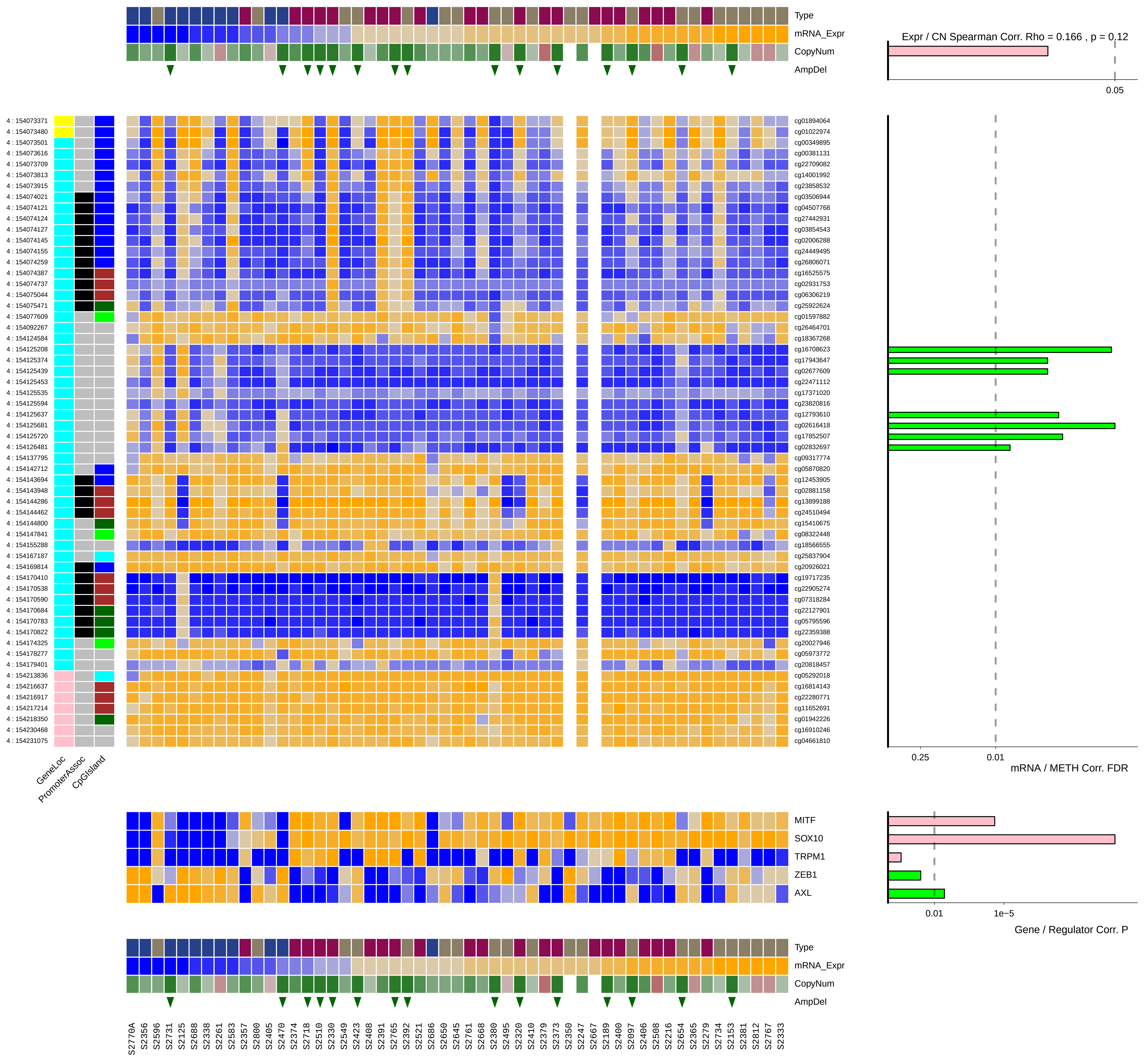

Type
mRNA_Expr
CopyNum
AmpDel
Expr / CN Spearman Corr. Rho = 0.166 , p = 0.12
0.05
4 : 154073371
4 : 154073480
4 : 154073501
4 : 154073616
4 : 154073709
4 : 154073813
4 : 154073915
4 : 154074021
4 : 154074121
4 : 154074124
4 : 154074127
4 : 154074145
4 : 154074155
4 : 154074259
4 : 154074387
4 : 154074737
4 : 154075044
4 : 154075471
4 : 154077609
4 : 154092267
4 : 154124584
4 : 154125208
4 : 154125374
4 : 154125439
4 : 154125453
4 : 154125535
4 : 154125594
4 : 154125637
4 : 154125681
4 : 154125720
4 : 154126481
4 : 154137795
4 : 154142712
4 : 154143694
4 : 154143948
4 : 154144286
4 : 154144462
4 : 154144800
4 : 154147841
4 : 154155288
4 : 154167187
4 : 154169814
4 : 154170410
4 : 154170538
4 : 154170590
4 : 154170684
4 : 154170783
4 : 154170822
4 : 154174325
4 : 154178277
4 : 154179401
4 : 154213836
4 : 154216637
4 : 154216917
4 : 154217214
4 : 154218350
4 : 154230468
4 : 154231075
cg01894064
cg01022974
cg00349895
cg00381131
cg22709082
cg14001992
cg23858532
cg03506944
cg04507768
cg27442931
cg03854543
cg02006288
cg24449495
cg26806071
cg16525575
cg02931753
cg06306219
cg25922624
cg01597882
cg26464701
cg18367268
cg16708623
cg17943647
cg02677609
cg22471112
cg17371020
cg23820816
cg12793610
cg02616418
cg17852507
cg02832697
cg09317774
cg05870820
cg12453905
cg02881158
cg13899188
cg24510494
cg15410675
cg08322448
cg18566555
cg25837904
cg20926021
cg19717235
cg22905274
cg07318284
cg22127901
cg05795596
cg22359388
cg20027946
cg05973772
cg20818457
cg05292018
cg16814143
cg22280771
cg11652691
cg01942226
cg16910246
cg04661810
0.25
0.01
mRNA / METH Corr. FDR
GeneLoc
PromoterAssoc
CpGIsland
MITF
SOX10
TRPM1
ZEB1
AXL
0.01
1e−5
Gene / Regulator Corr. P
Type
mRNA_Expr
CopyNum
AmpDel
S2770A S2356 S2596 S2731 S2125 S2688 S2338 S2261 S2583 S2357 S2800 S2405 S2470 S2374 S2718 S2510 S2330 S2549 S2423 S2408 S2391 S2765 S2392 S2521 S2686 S2650 S2645 S2761 S2668 S2380 S2495 S2320 S2410 S2379 S2373 S2350 S2247 S2667 S2189 S2400 S2097 S2406 S2508 S2216 S2654 S2365 S2279 S2734 S2153 S2381 S2812 S2767 S2333

APOLD1

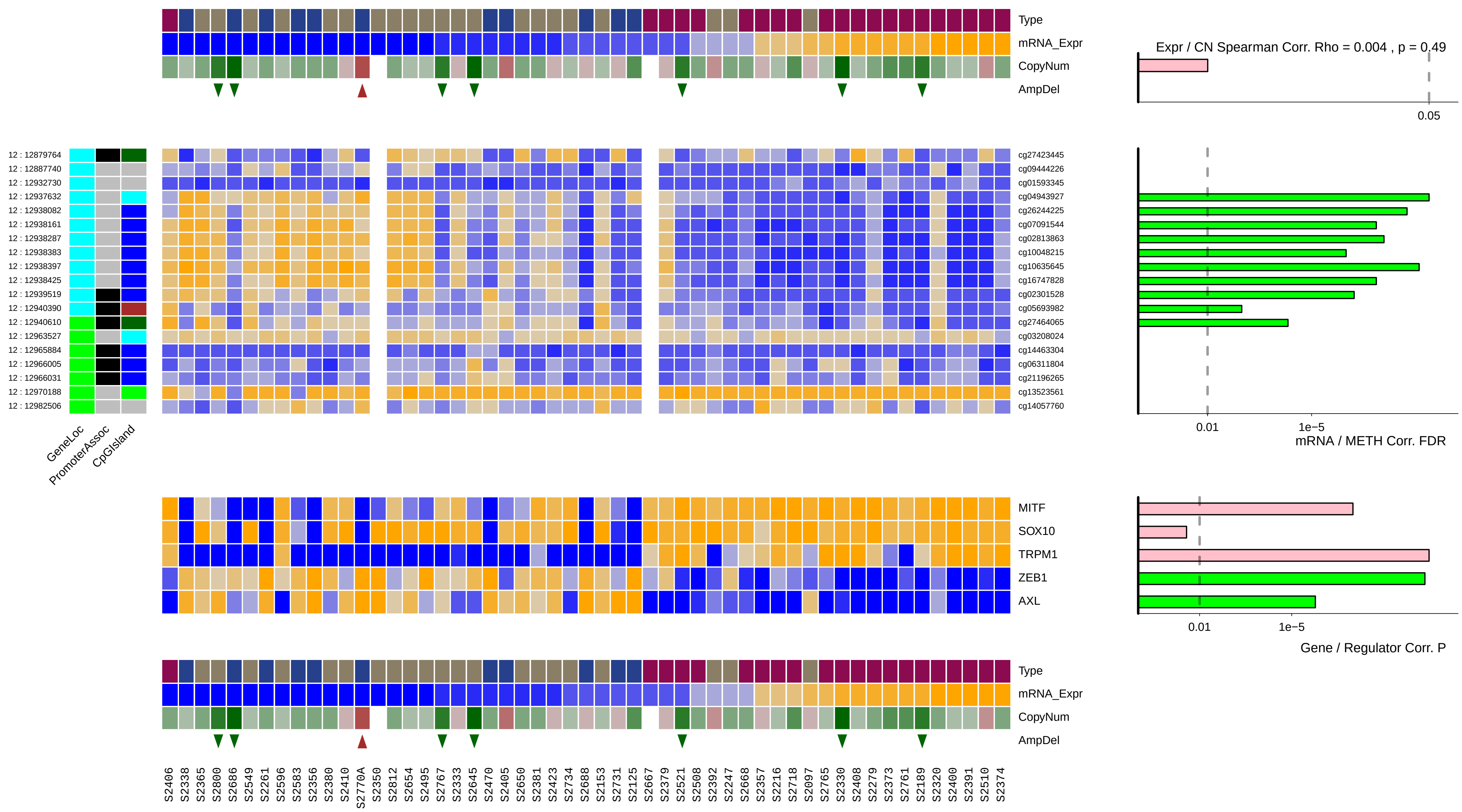

Type
mRNA_Expr
CopyNum
AmpDel
Expr / CN Spearman Corr. Rho = 0.004 , p = 0.49
0.05
12 : 12879764
12 : 12887740
12 : 12932730
12 : 12937632
12 : 12938082
12 : 12938161
12 : 12938287
12 : 12938383
12 : 12938397
12 : 12938425
12 : 12939519
12 : 12940390
12 : 12940610
12 : 12963527
12 : 12965884
12 : 12966005
12 : 12966031
12 : 12970188
12 : 12982506
GeneLoc
PromoterAssoc
CpGIsland
cg27423445
cg09444226
cg01593345
cg04943927
cg26244225
cg07091544
cg02813863
cg10048215
cg10635645
cg16747828
cg02301528
cg05693982
cg27464065
cg03208024
cg14463304
cg06311804
cg21196265
cg13523561
cg14057760
0.01
1e−5
mRNA / METH Corr. FDR
MITF
SOX10
TRPM1
ZEB1
AXL
0.01
1e−5
Gene / Regulator Corr. P
Type
mRNA_Expr
CopyNum
AmpDel
S2406 S2338 S2365 S2800 S2686 S2549 S2261 S2596 S2583 S2356 S2380 S2410 S2770A S2350 S2812 S2654 S2495 S2767 S2333 S2645 S2470 S2405 S2650 S2381 S2423 S2734 S2688 S2153 S2731 S2125 S2667 S2379 S2521 S2508 S2392 S2247 S2668 S2357 S2216 S2718 S2097 S2765 S2330 S2408 S2279 S2373 S2761 S2189 S2320 S2400 S2391 S2510 S2374

ITGA9

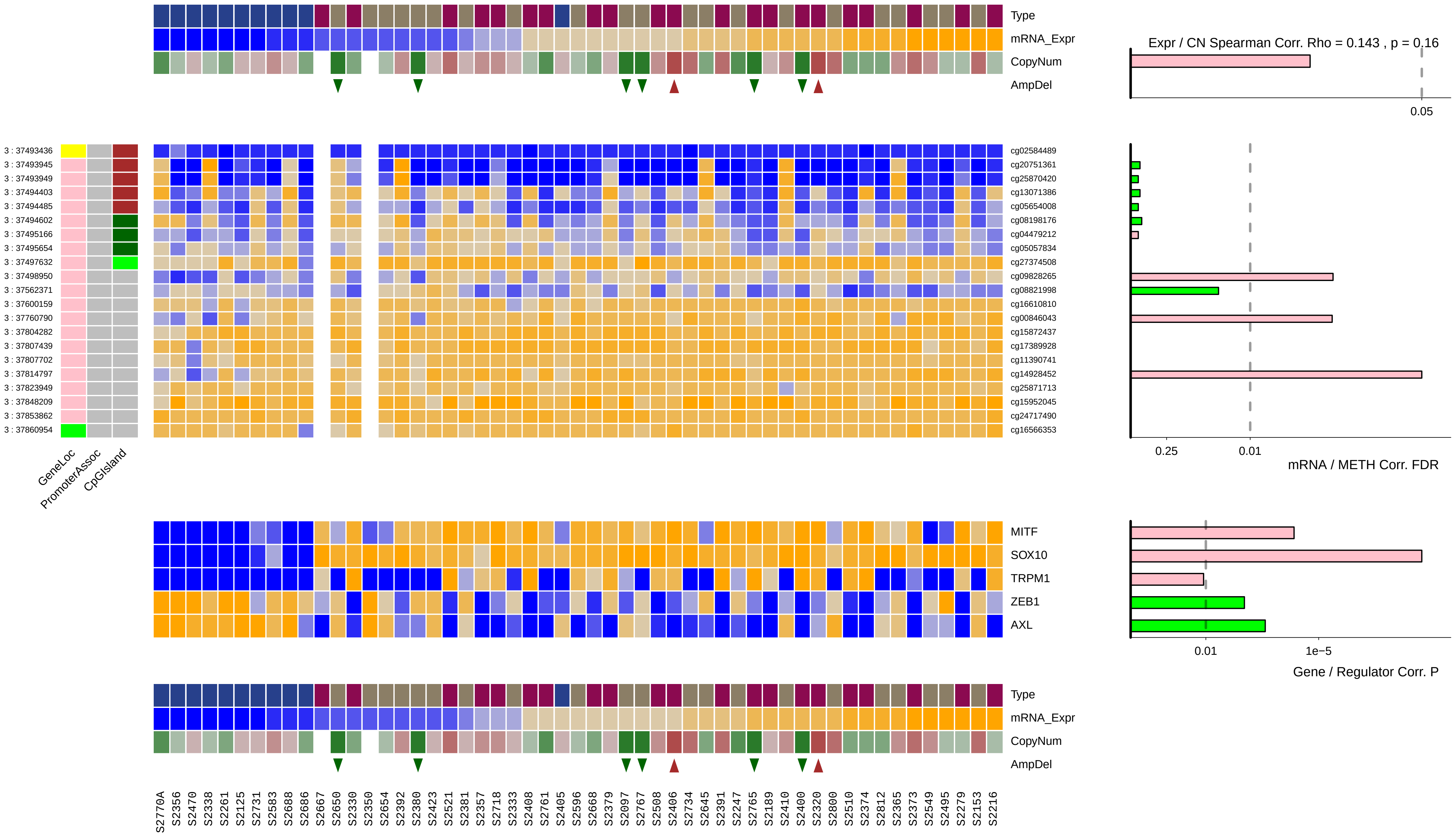

Type
mRNA_Expr
CopyNum
AmpDel
Expr / CN Spearman Corr. Rho = 0.143 , p = 0.16
0.05
mRNA / METH Corr. FDR
0.25
0.01
GeneLoc
PromoterAssoc
CpGIsland
MITF
SOX10
TRPM1
ZEB1
AXL
Gene / Regulator Corr. P
1e−5

CHCHD10

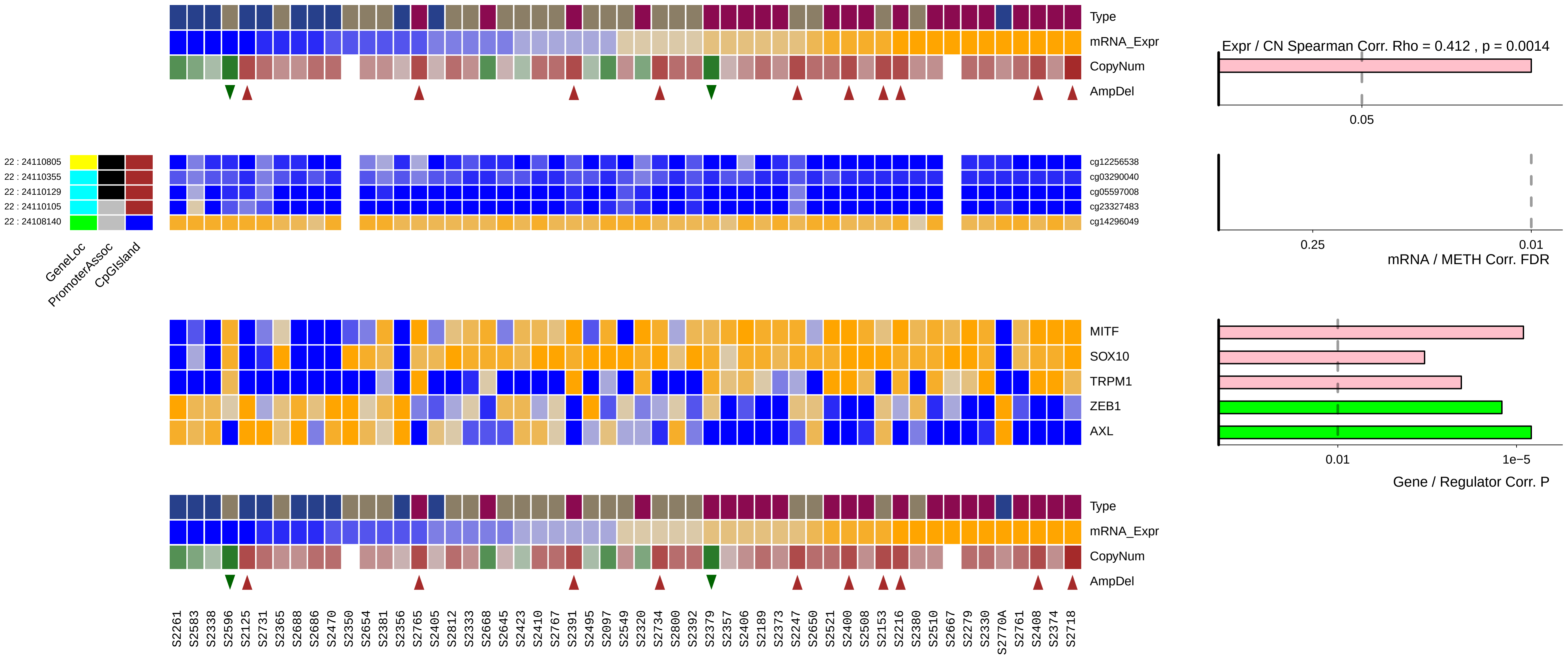
Type
mRNA_Expr
CopyNum
AmpDel
Expr / CN Spearman Corr. Rho = 0.412 , p = 0.0014
0.05
22 : 24110805
22 : 24110355
22 : 24110129
22 : 24110105
22 : 24108140
cg12256538
cg03290040
cg05597008
cg23327483
cg14296049
0.25
0.01
mRNA / METH Corr. FDR
GeneLoc
PromoterAssoc
CpGIsland
MITF
SOX10
TRPM1
ZEB1
AXL
0.01
1e−5
Gene / Regulator Corr. P
S2261 S2583 S2338 S2596 S2125 S2731 S2365 S2688 S2686 S2470 S2350 S2654 S2381 S2356 S2765 S2405 S2812 S2333 S2668 S2645 S2423 S2410 S2767 S2391 S2495 S2097 S2549 S2320 S2734 S2800 S2392 S2379 S2357 S2406 S2189 S2373 S2247 S2650 S2521 S2400 S2508 S2153 S2216 S2380 S2510 S2667 S2279 S2330 S2770A S2761 S2408 S2374 S2718

GPR143

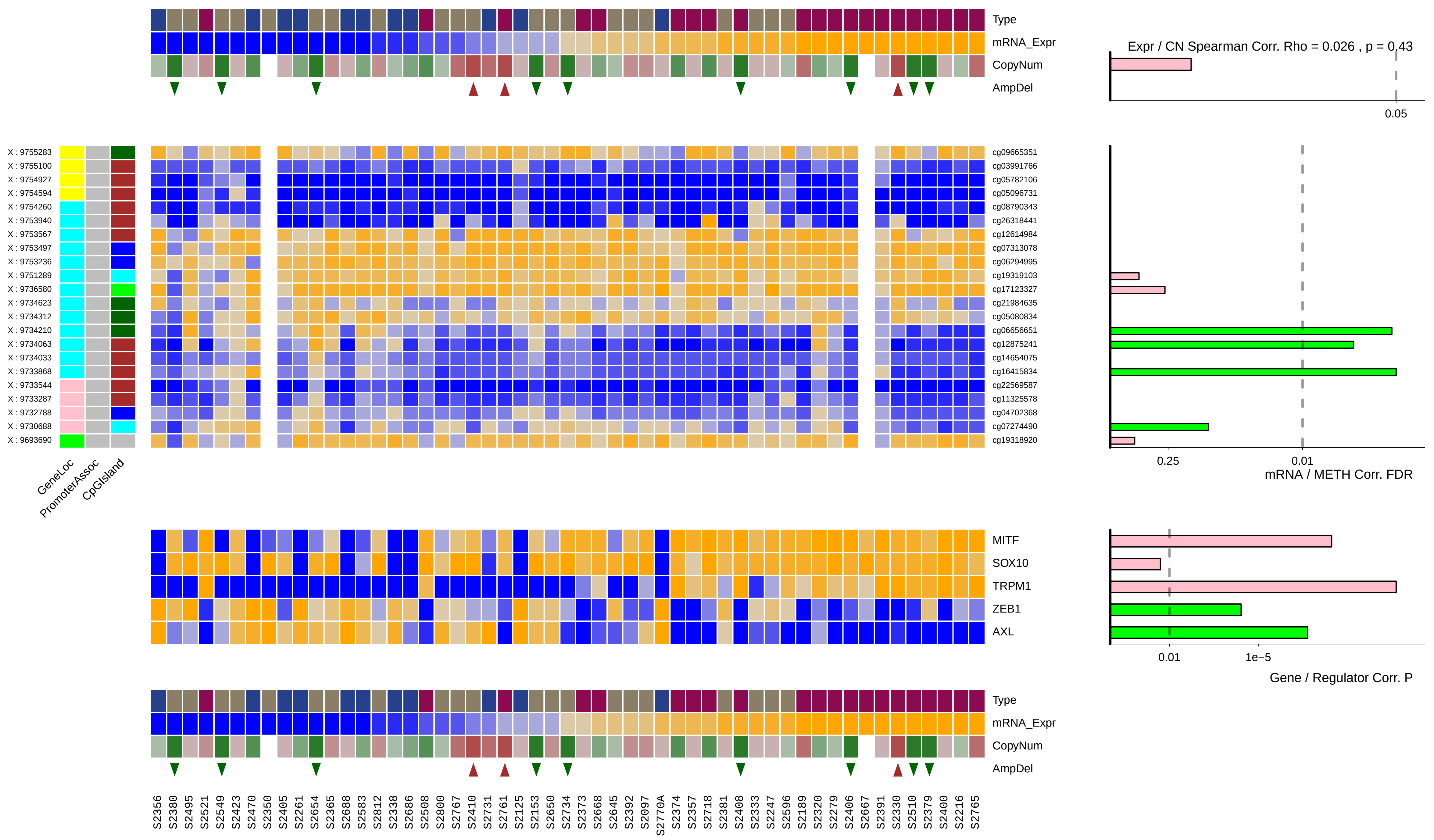
Type
mRNA_Expr
CopyNum
AmpDel
Expr / CN Spearman Corr. Rho = 0.026 , p = 0.43
0.05
GeneLoc
PromoterAssoc
CpGIsland
mRNA / METH Corr. FDR
0.25
0.01
MITF
SOX10
TRPM1
ZEB1
AXL
Gene / Regulator Corr. P
1e−5

ST6GALNAC3

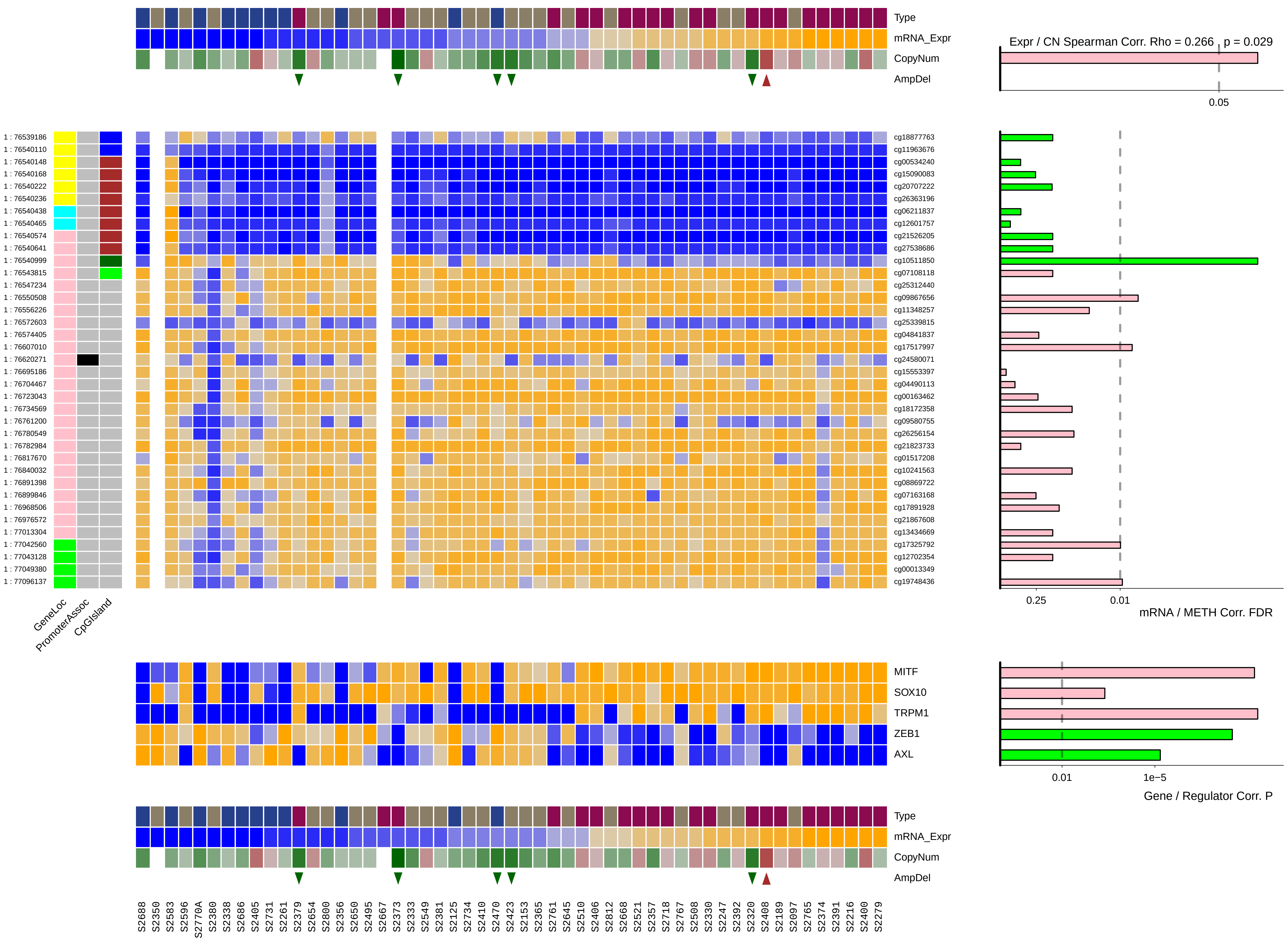

Type
mRNA_Expr
CopyNum
AmpDel
Expr / CN Spearman Corr. Rho = 0.266 , p = 0.029
0.05
1 : 76539186
1 : 76540110
1 : 76540148
1 : 76540168
1 : 76540222
1 : 76540236
1 : 76540438
1 : 76540465
1 : 76540574
1 : 76540641
1 : 76540999
1 : 76543815
1 : 76547234
1 : 76550508
1 : 76556226
1 : 76572603
1 : 76574405
1 : 76607010
1 : 76620271
1 : 76695186
1 : 76704467
1 : 76723043
1 : 76734569
1 : 76761200
1 : 76780549
1 : 76782984
1 : 76817670
1 : 76840032
1 : 76891398
1 : 76899846
1 : 76968506
1 : 76976572
1 : 77013304
1 : 77042560
1 : 77043128
1 : 77049380
1 : 77096137
GeneLoc
PromoterAssoc
CpGIsland
cg18877763
cg11963676
cg00534240
cg15090083
cg20707222
cg26363196
cg06211837
cg12601757
cg21526205
cg27538686
cg10511850
cg07108118
cg25312440
cg09867656
cg11348257
cg25339815
cg04841837
cg17517997
cg24580071
cg15553397
cg04490113
cg00163462
cg18172358
cg09580755
cg26256154
cg21823733
cg01517208
cg10241563
cg08869722
cg07163168
cg17891928
cg21867608
cg13434669
cg17325792
cg12702354
cg00013349
cg19748436
0.25
0.01
mRNA / METH Corr. FDR
MITF
SOX10
TRPM1
ZEB1
AXL
0.01
1e−5
Gene / Regulator Corr. P
Type
mRNA_Expr
CopyNum
AmpDel
S2688
S2350
S2583
S2596
S2770A
S2380
S2338
S2686
S2405
S2731
S2261
S2379
S2654
S2800
S2356
S2650
S2495
S2667
S2373
S2333
S2549
S2381
S2125
S2734
S2410
S2470
S2423
S2153
S2365
S2761
S2645
S2510
S2406
S2812
S2668
S2521
S2357
S2718
S2767
S2508
S2330
S2247
S2392
S2320
S2408
S2189
S2097
S2765
S2374
S2391
S2216
S2400
S2279

RAP1GAP

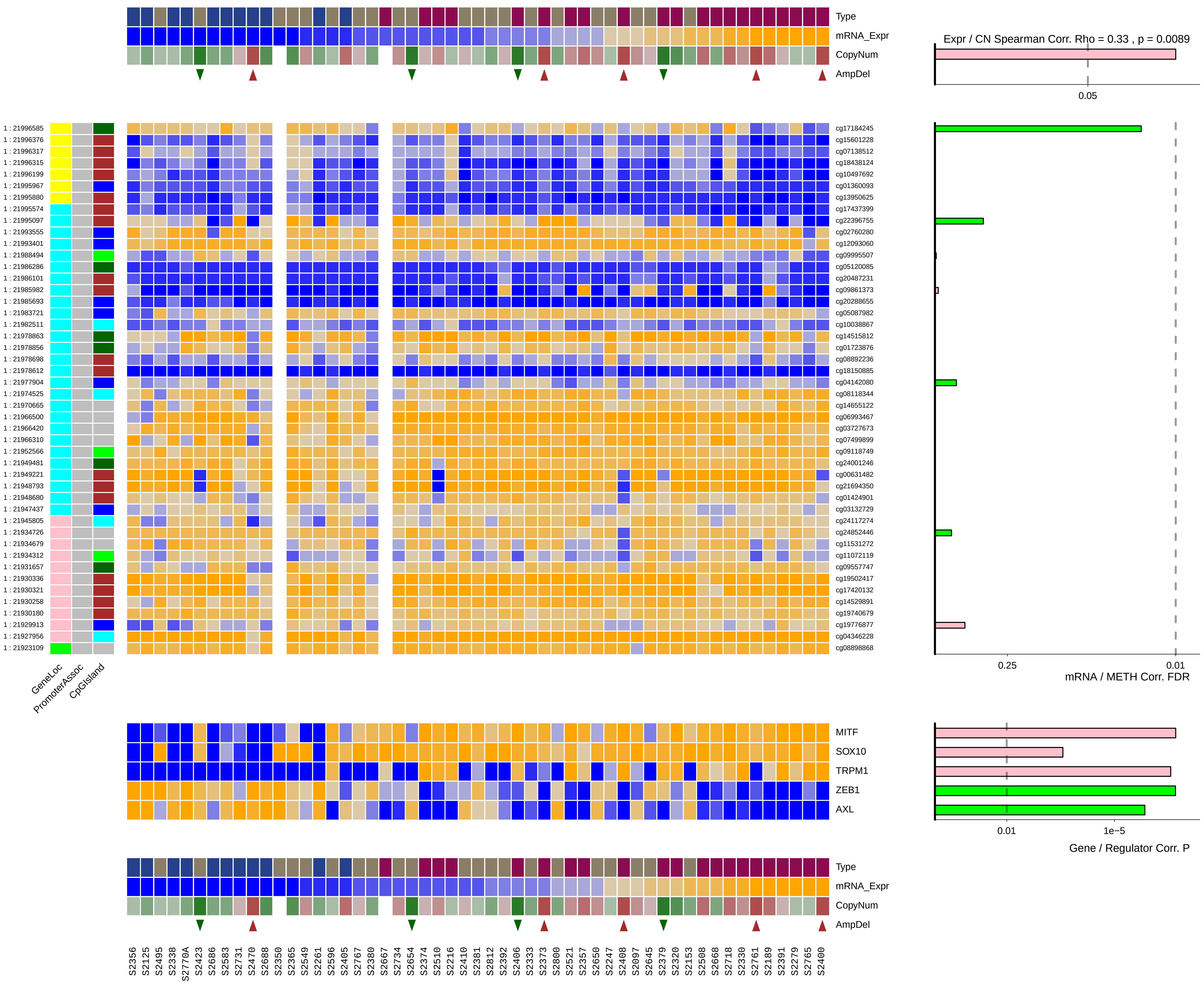

Type
mRNA_Expr
CopyNum
AmpDel
Expr / CN Spearman Corr. Rho = 0.33 , p = 0.0089
0.05
GeneLoc
PromoterAssoc
CpGIsland
0.25
0.01
mRNA / METH Corr. FDR
MITF
SOX10
TRPM1
ZEB1
AXL
0.01
1e−5
Gene / Regulator Corr. P

CPVL

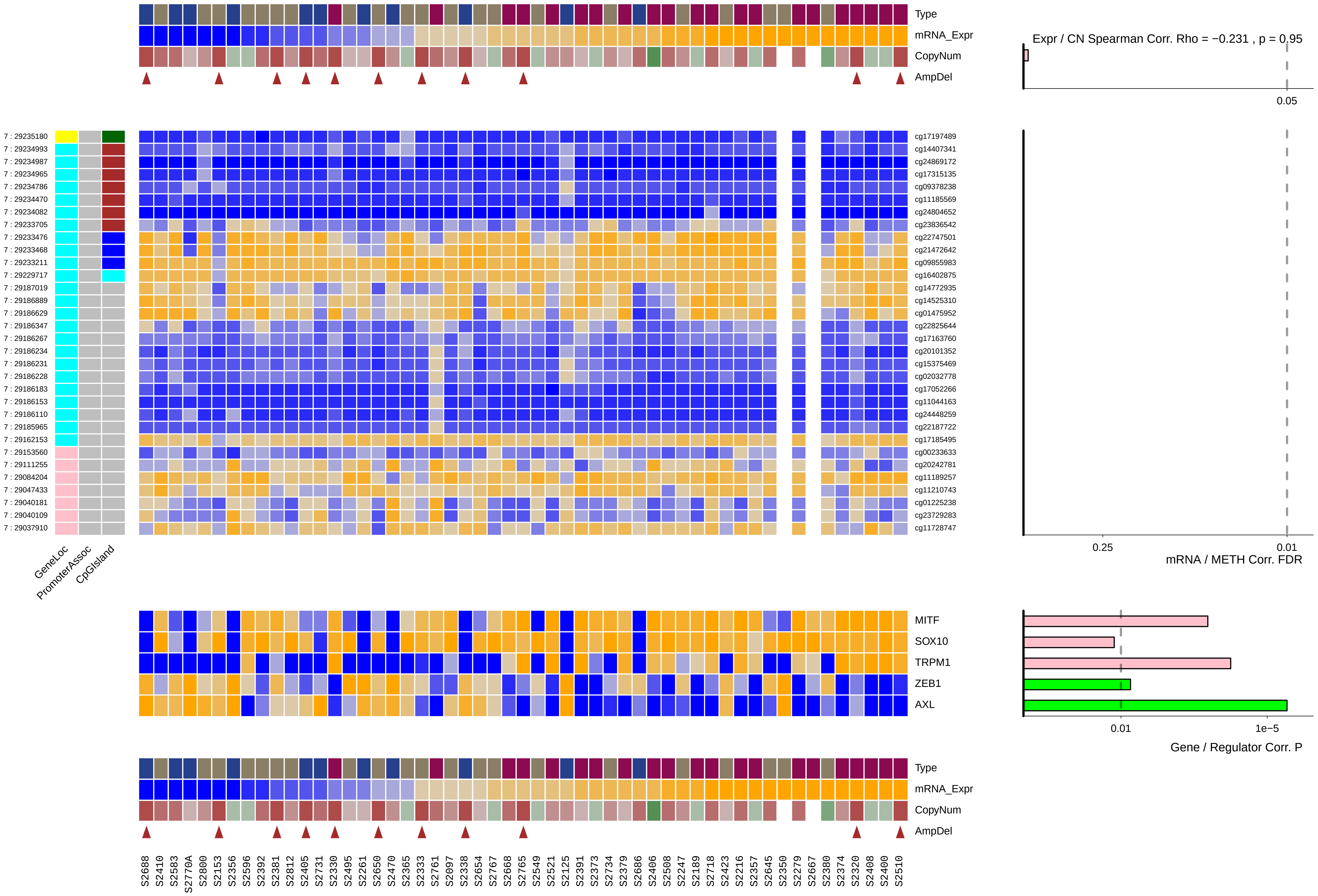

ACP5

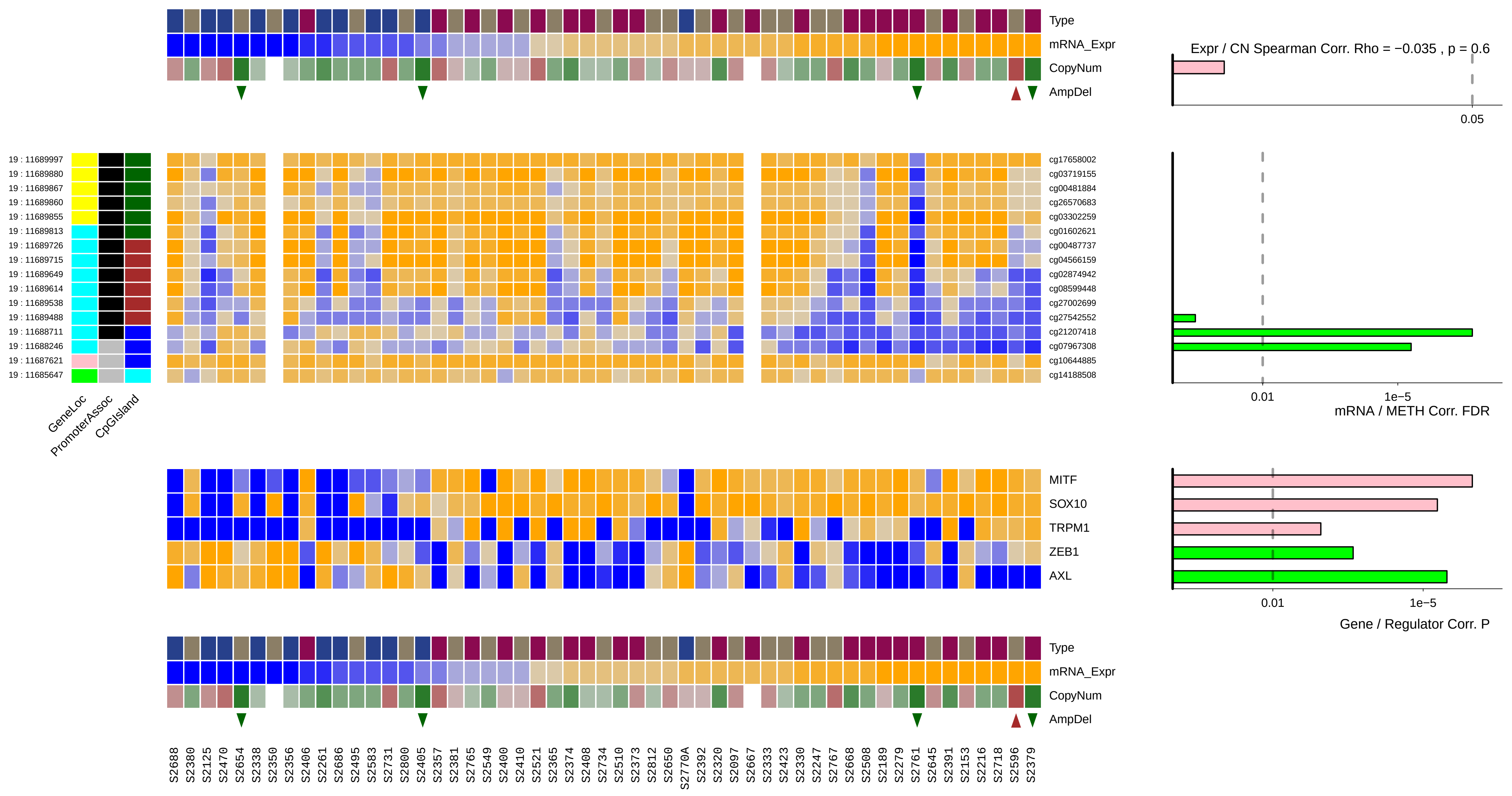
Type
mRNA_Expr
CopyNum
AmpDel
Expr / CN Spearman Corr. Rho = −0.035 , p = 0.6
0.05
cg17658002
cg03719155
cg00481884
cg26570683
cg03302259
cg01602621
cg00487737
cg04566159
cg02874942
cg08599448
cg27002699
cg27542552
cg21207418
cg07967308
cg10644885
cg14188508
19 : 11689997
19 : 11689880
19 : 11689867
19 : 11689860
19 : 11689855
19 : 11689813
19 : 11689726
19 : 11689715
19 : 11689649
19 : 11689614
19 : 11689538
19 : 11689488
19 : 11688711
19 : 11688246
19 : 11687621
19 : 11685647
GeneLoc
PromoterAssoc
CpGIsland
0.01
1e−5
mRNA / METH Corr. FDR
MITF
SOX10
TRPM1
ZEB1
AXL
Gene / Regulator Corr. P
S2688 S2380 S2125 S2470 S2654 S2338 S2350 S2356 S2406 S2261 S2686 S2495 S2583 S2731 S2800 S2405 S2357 S2381 S2765 S2549 S2400 S2410 S2521 S2365 S2374 S2408 S2734 S2510 S2373 S2812 S2650 S2770A S2392 S2320 S2097 S2667 S2333 S2423 S2330 S2247 S2767 S2668 S2508 S2189 S2279 S2761 S2645 S2391 S2153 S2216 S2718 S2596 S2379

LZTS1

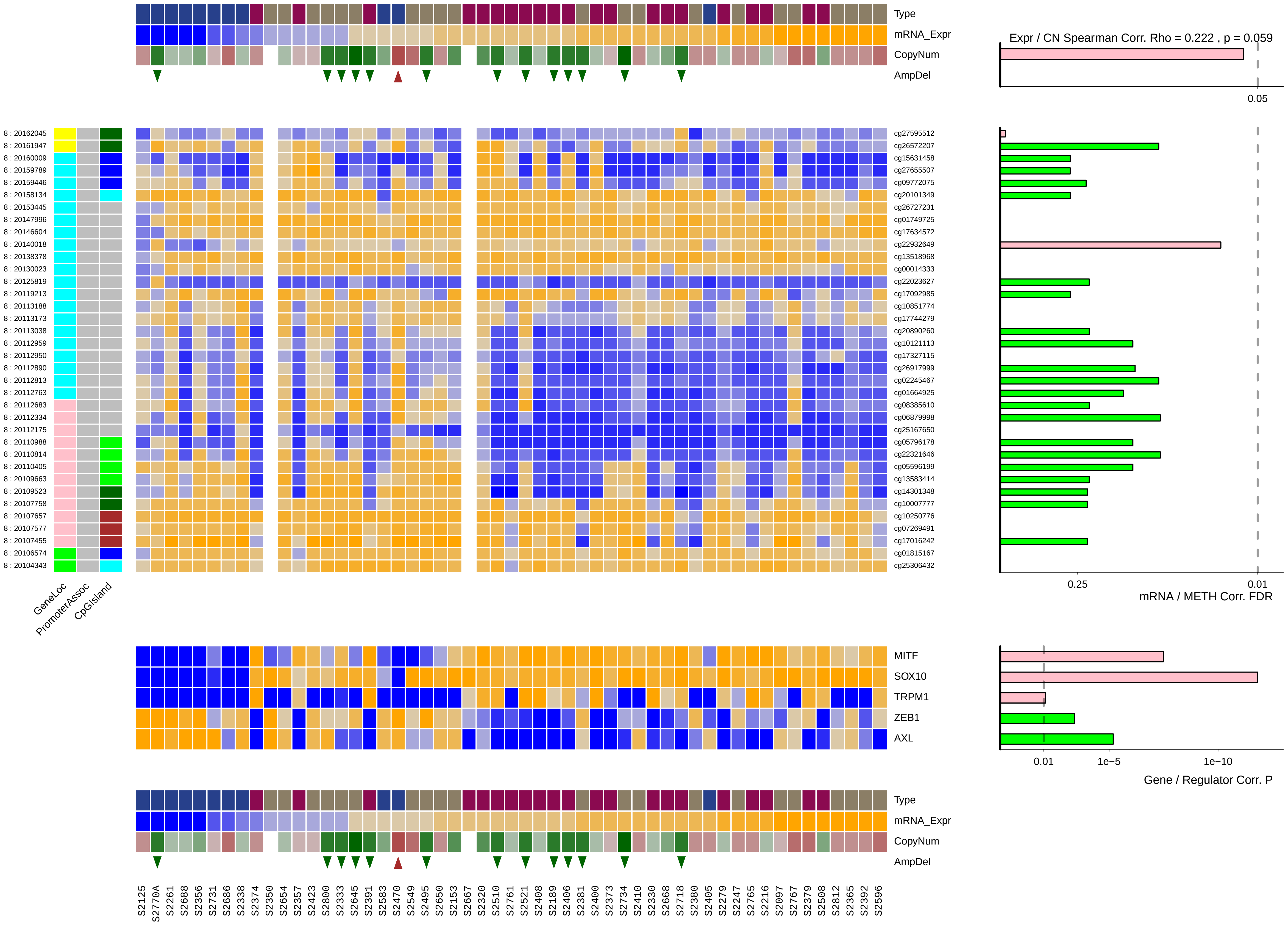

Type
mRNA_Expr
CopyNum
AmpDel
Expr / CN Spearman Corr. Rho = 0.222 , p = 0.059
0.05
GeneLoc
PromoterAssoc
CpGIsland
0.25
0.01
mRNA / METH Corr. FDR
MITF
SOX10
TRPM1
ZEB1
AXL
0.01
1e−5
1e−10
Gene / Regulator Corr. P

CEACAM1

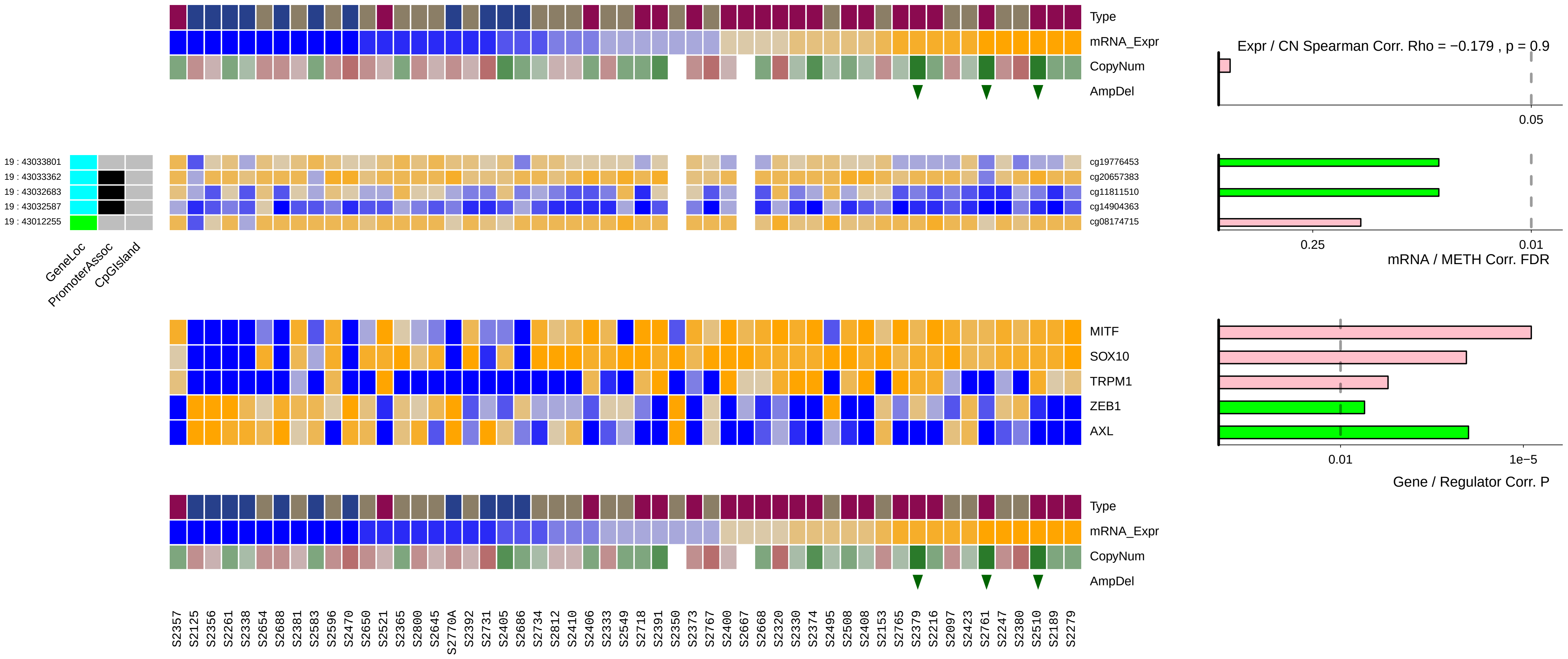

Type
mRNA_Expr
CopyNum
AmpDel
Expr / CN Spearman Corr. Rho = −0.179 , p = 0.9
0.05
19 : 43033801
19 : 43033362
19 : 43032683
19 : 43032587
19 : 43012255
GeneLoc
PromoterAssoc
CpGIsland
cg19776453
cg20657383
cg11811510
cg14904363
cg08174715
0.25
0.01
mRNA / METH Corr. FDR
MITF
SOX10
TRPM1
ZEB1
AXL
0.01
1e−5
Gene / Regulator Corr. P
S2357 S2125 S2356 S2261 S2338 S2654 S2688 S2381 S2583 S2596 S2470 S2650 S2521 S2365 S2800 S2645 S2770A S2392 S2731 S2405 S2686 S2734 S2812 S2410 S2406 S2333 S2549 S2718 S2391 S2350 S2373 S2767 S2400 S2667 S2668 S2320 S2330 S2374 S2495 S2508 S2408 S2153 S2765 S2379 S2216 S2097 S2423 S2761 S2247 S2380 S2510 S2189 S2279

LAMA1

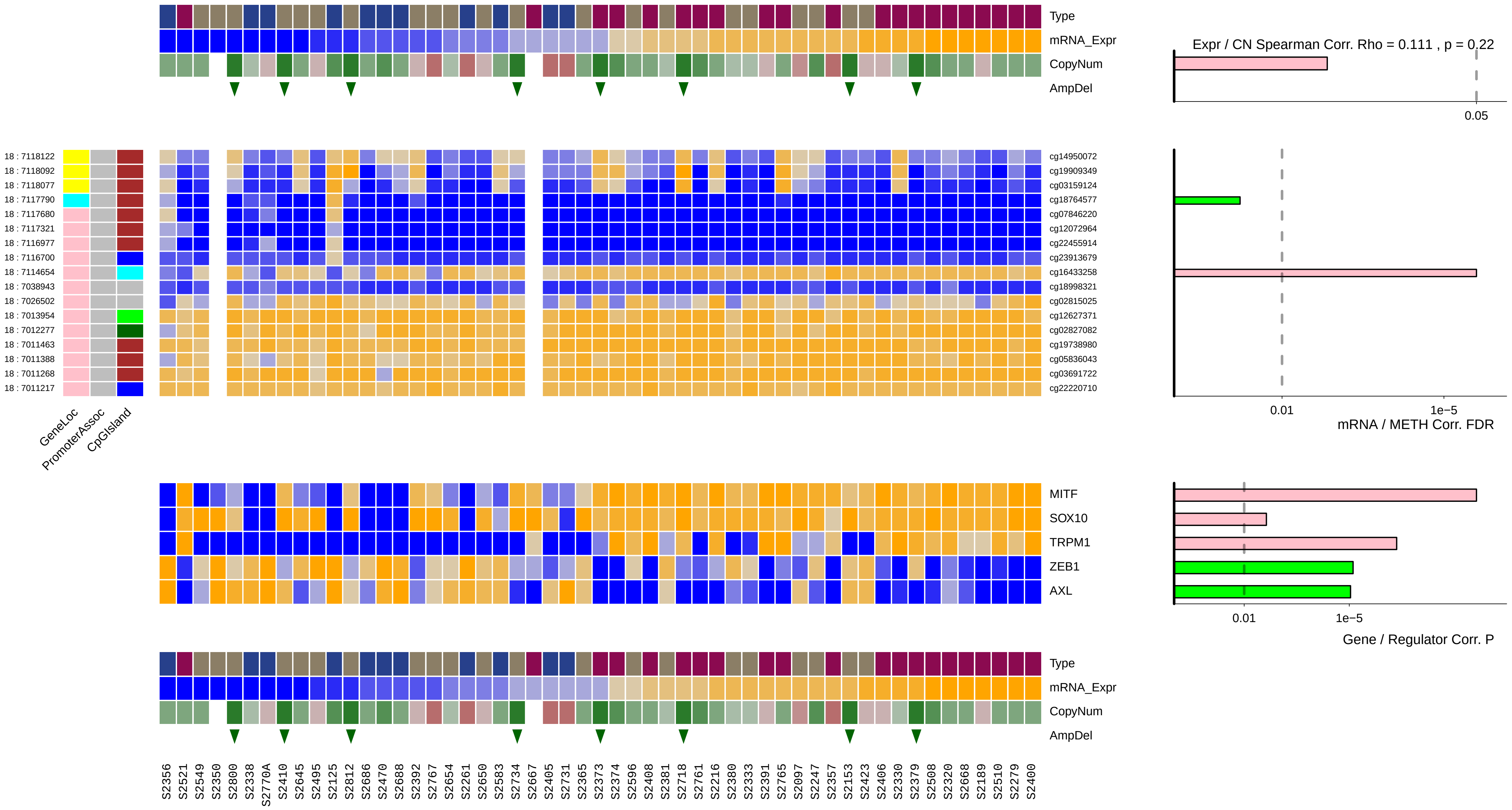

Type
mRNA_Expr
CopyNum
AmpDel
Expr / CN Spearman Corr. Rho = 0.111 , p = 0.22
0.05
18 : 7118122
18 : 7118092
18 : 7118077
18 : 7117790
18 : 7117680
18 : 7117321
18 : 7116977
18 : 7116700
18 : 7114654
18 : 7038943
18 : 7026502
18 : 7013954
18 : 7012277
18 : 7011463
18 : 7011388
18 : 7011268
18 : 7011217
cg14950072
cg19909349
cg03159124
cg18764577
cg07846220
cg12072964
cg22455914
cg23913679
cg16433258
cg18998321
cg02815025
cg12627371
cg02827082
cg19738980
cg05836043
cg03691722
cg22220710
GeneLoc
PromoterAssoc
CpGIsland
0.01
1e−5
mRNA / METH Corr. FDR
MITF
SOX10
TRPM1
ZEB1
AXL
0.01
1e−5
Gene / Regulator Corr. P
Type
mRNA_Expr
CopyNum
AmpDel
S2356 S2521 S2549 S2350 S2800 S2338 S2770A S2410 S2645 S2495 S2125 S2812 S2686 S2470 S2688 S2392 S2767 S2654 S2261 S2650 S2583 S2734 S2667 S2405 S2731 S2365 S2373 S2374 S2596 S2408 S2381 S2718 S2761 S2216 S2380 S2333 S2391 S2765 S2097 S2247 S2357 S2153 S2423 S2406 S2330 S2379 S2508 S2320 S2668 S2189 S2510 S2279 S2400
