# Supplementary material for: Multi-modal molecular programs regulate melanoma cell state
Source: Nat Commun. 2022 Jul 9;13:4000. doi: 10.1038/s41467-022-31510-1 (PMC9271073; doi:10.1038/s41467-022-31510-1)
Supplement: Supplementary file 11 — Supplementary Data 8 [file 41467_2022_31510_MOESM11_ESM.pdf]

Supplementary Data 8: MEL gene regulatory influences - TCGA melanomas.

PMEL

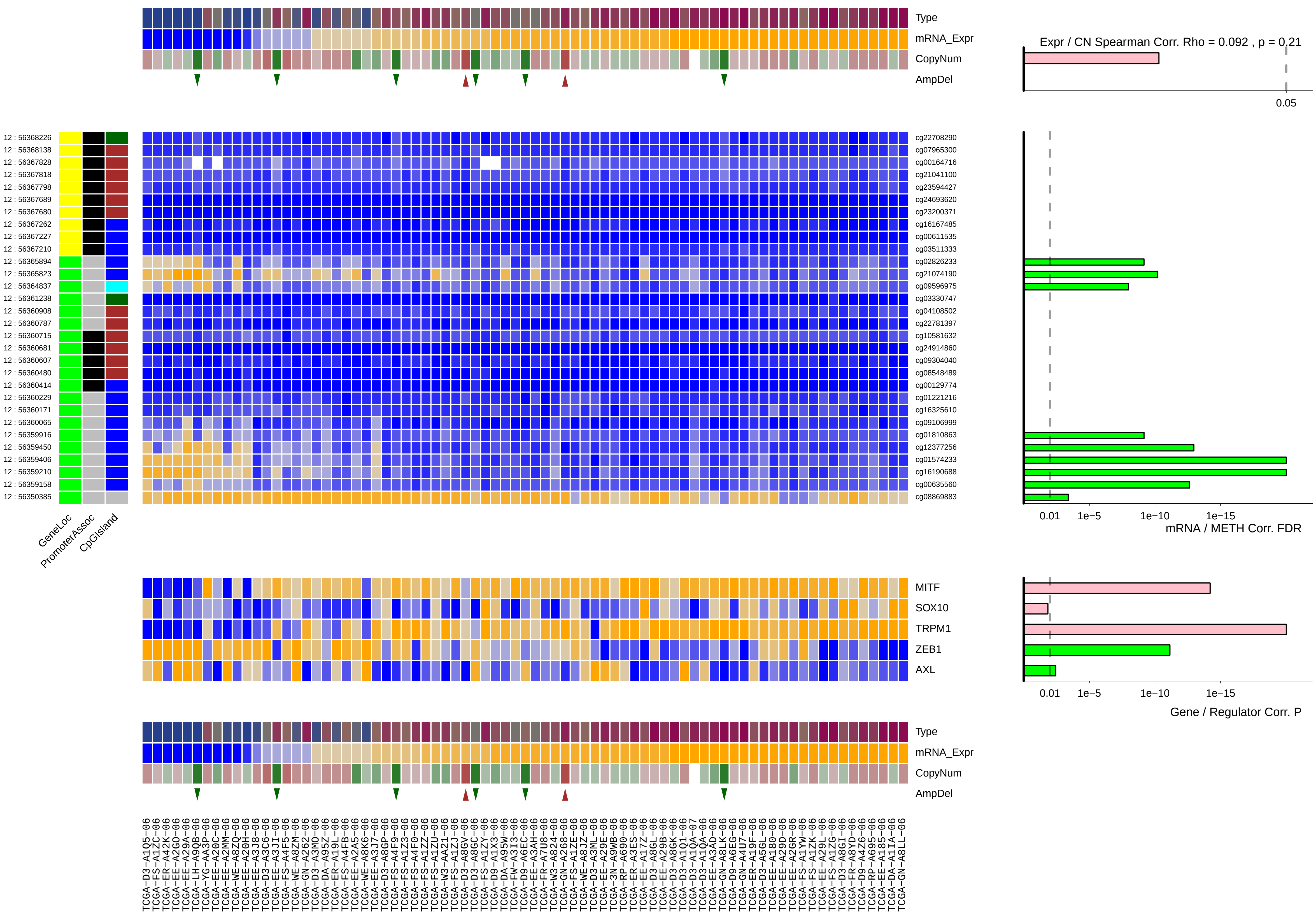

Type
mRNA_Expr
CopyNum
AmpDel
Expr / CN Spearman Corr. Rho = 0.092 , p = 0.21
0.05
cg22708290
cg07965300
cg00164716
cg21041100
cg23594427
cg24693620
cg23200371
cg16167485
cg00611535
cg03511333
cg02826233
cg21074190
cg09596975
cg03330747
cg04108502
cg22781397
cg10581632
cg24914860
cg09304040
cg08548489
cg00129774
cg01221216
cg16325610
cg09106999
cg01810863
cg12377256
cg01574233
cg16190688
cg00635560
cg08869883
12 : 56368226
12 : 56368138
12 : 56367828
12 : 56367818
12 : 56367798
12 : 56367689
12 : 56367680
12 : 56367262
12 : 56367227
12 : 56367210
12 : 56365894
12 : 56365823
12 : 56364837
12 : 56361238
12 : 56360908
12 : 56360787
12 : 56360715
12 : 56360681
12 : 56360607
12 : 56360480
12 : 56360414
12 : 56360229
12 : 56360171
12 : 56360065
12 : 56359916
12 : 56359450
12 : 56359406
12 : 56359210
12 : 56359158
12 : 56350385
GeneLoc
PromoterAssoc
CpGIsland
0.01
1e−5
1e−10
1e−15
mRNA / METH Corr. FDR
MITF
SOX10
TRPM1
ZEB1
AXL
Gene / Regulator Corr. P

MITF

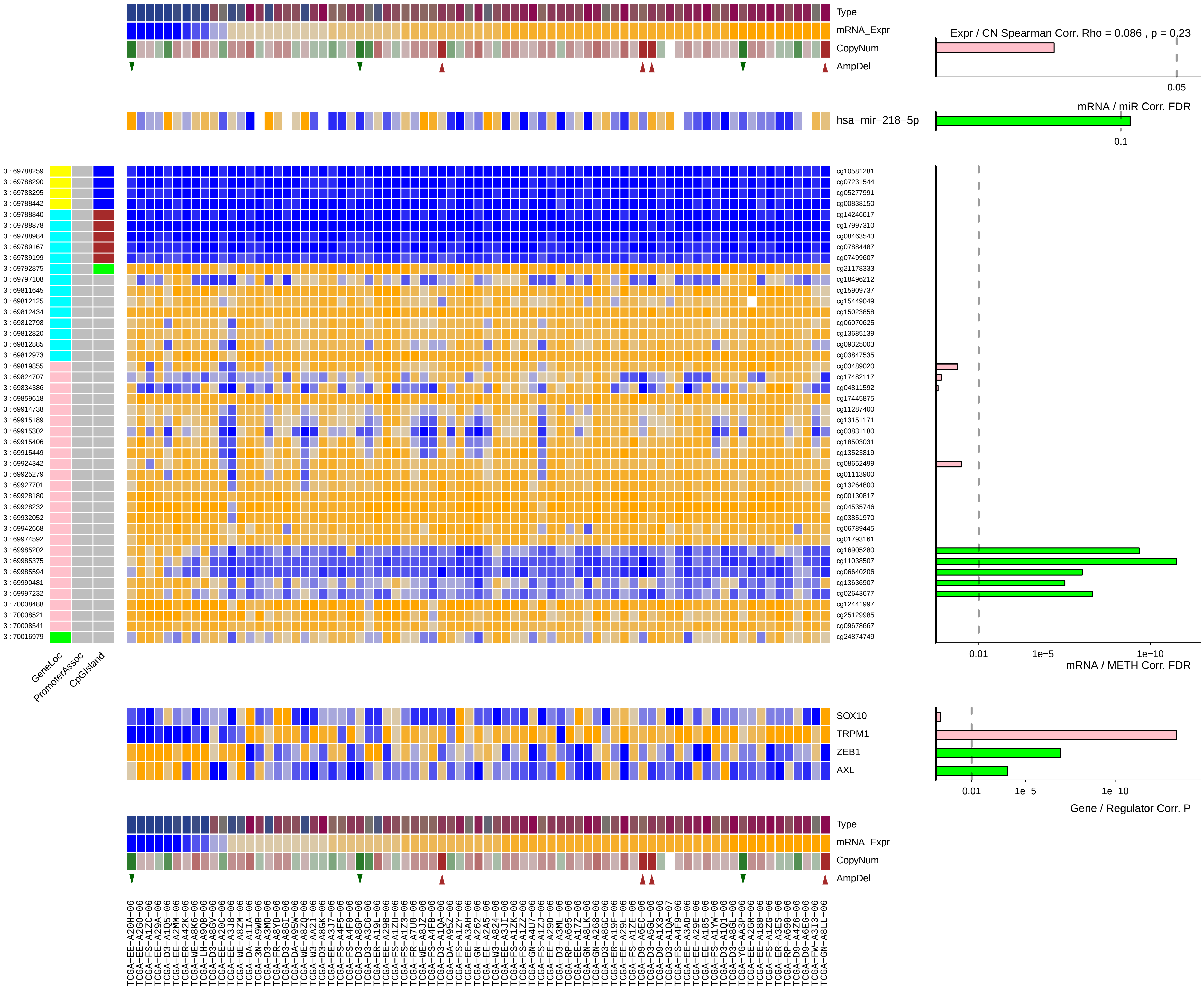

Type
mRNA_Expr
CopyNum
AmpDel
Expr / CN Spearman Corr. Rho = 0.086 , p = 0.23
0.05
hsa−mir−218−5p
mRNA / miR Corr. FDR
0.1
3 : 69788259
3 : 69788290
3 : 69788295
3 : 69788442
3 : 69788840
3 : 69788878
3 : 69788984
3 : 69789167
3 : 69789199
3 : 69792875
3 : 69797108
3 : 69811645
3 : 69812125
3 : 69812434
3 : 69812798
3 : 69812820
3 : 69812885
3 : 69812973
3 : 69819855
3 : 69824707
3 : 69834386
3 : 69859618
3 : 69914738
3 : 69915189
3 : 69915302
3 : 69915406
3 : 69915449
3 : 69924342
3 : 69925279
3 : 69927701
3 : 69928180
3 : 69928232
3 : 69932052
3 : 69942668
3 : 69974592
3 : 69985202
3 : 69985375
3 : 69985594
3 : 69990481
3 : 69997232
3 : 70008488
3 : 70008521
3 : 70008541
3 : 70016979
cg10581281
cg07231544
cg05277991
cg00838150
cg14246617
cg17997310
cg08463543
cg07884487
cg07499607
cg21178333
cg18496212
cg15909737
cg15449049
cg15023858
cg06070625
cg13685139
cg09325003
cg03847535
cg03489020
cg17482117
cg04811592
cg17445875
cg11287400
cg13151171
cg03831180
cg18503031
cg13523819
cg08652499
cg01113900
cg13264800
cg00130817
cg04535746
cg03851970
cg06789445
cg01793161
cg16905280
cg11038507
cg06640206
cg13636907
cg02643677
cg12441997
cg25129985
cg09678667
cg24874749
GeneLoc
PromoterAssoc
CpGIsland
0.01
1e−5
1e−10
mRNA / METH Corr. FDR
SOX10
TRPM1
ZEB1
AXL
0.01
1e−5
1e−10
Gene / Regulator Corr. P
Type
mRNA_Expr
CopyNum
AmpDel

TYR

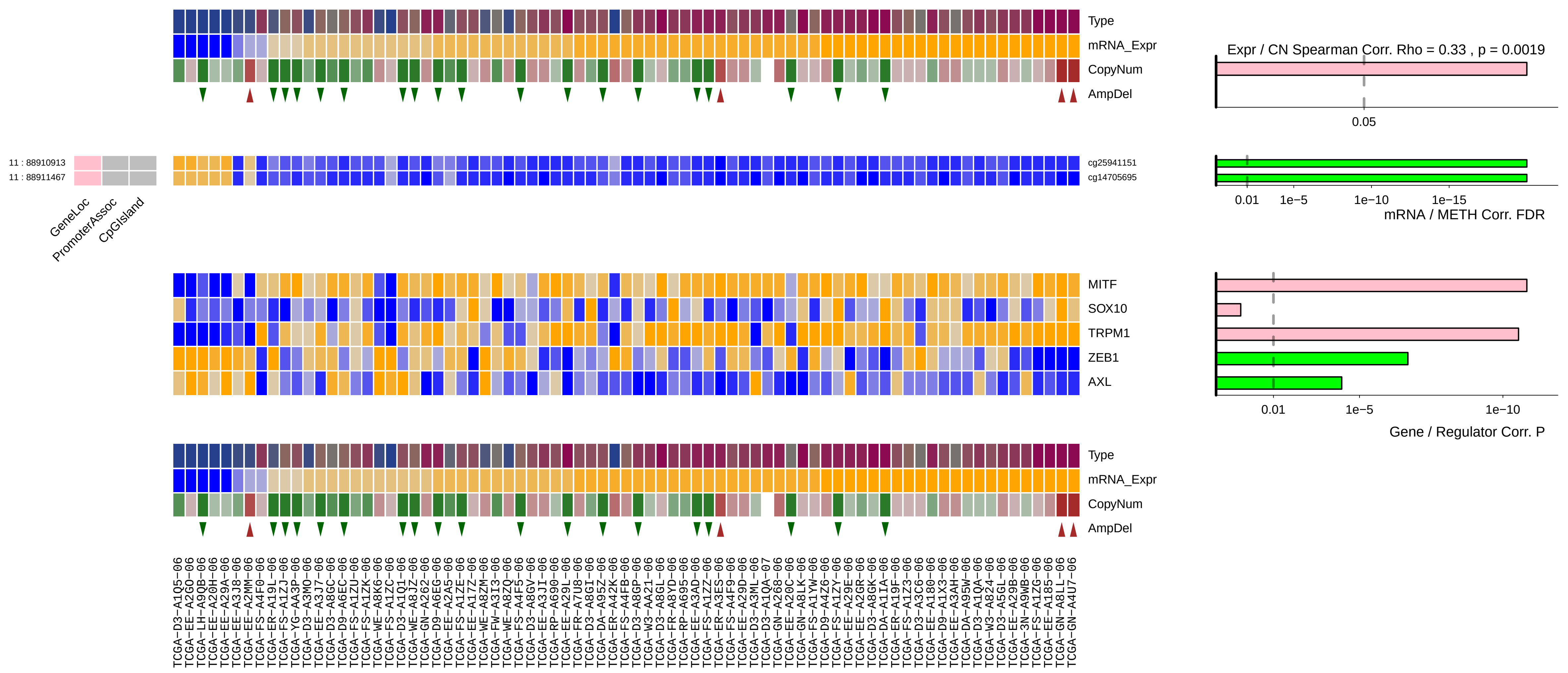
Type
mRNA_Expr
CopyNum
AmpDel
Expr / CN Spearman Corr. Rho = 0.33 , p = 0.0019
0.05
11 : 88910913
11 : 88911467
GeneLoc
PromoterAssoc
CpGIsland
cg25941151
cg14705695
0.01
1e−5
1e−10
1e−15
mRNA / METH Corr. FDR
MITF
SOX10
TRPM1
ZEB1
AXL
0.01
1e−5
1e−10
Gene / Regulator Corr. P
Type
mRNA_Expr
CopyNum
AmpDel
TCGA−D3−A1Q5−06
TCGA−EE−A2GO−06
TCGA−LH−A9QB−06
TCGA−EE−A20H−06
TCGA−EE−A29A−06
TCGA−EE−A3J8−06
TCGA−EE−A2MM−06
TCGA−FS−A4F0−06
TCGA−ER−A19L−06
TCGA−FS−A1ZJ−06
TCGA−YG−AA3P−06
TCGA−D3−A3MO−06
TCGA−EE−A3J7−06
TCGA−D3−A8GC−06
TCGA−D9−A6EC−06
TCGA−FS−A1ZU−06
TCGA−FS−A1ZK−06
TCGA−WE−A8K6−06
TCGA−FS−A1ZC−06
TCGA−D3−A1Q1−06
TCGA−WE−A8JZ−06
TCGA−GN−A262−06
TCGA−D9−A6EG−06
TCGA−EE−A2A5−06
TCGA−FS−A1ZE−06
TCGA−EE−A17Z−06
TCGA−WE−A8ZM−06
TCGA−FW−A3I3−06
TCGA−WE−A8ZQ−06
TCGA−FS−A4F5−06
TCGA−D3−A8GV−06
TCGA−EE−A3JI−06
TCGA−RP−A690−06
TCGA−EE−A29L−06
TCGA−FR−A7U8−06
TCGA−D3−A8GI−06
TCGA−DA−A95Z−06
TCGA−ER−A42K−06
TCGA−FS−A4FB−06
TCGA−D3−A8GP−06
TCGA−W3−AA21−06
TCGA−D3−A8GL−06
TCGA−FR−A8YD−06
TCGA−RP−A695−06
TCGA−EE−A3AD−06
TCGA−FS−A1ZZ−06
TCGA−ER−A3ES−06
TCGA−FS−A4F9−06
TCGA−EE−A29D−06
TCGA−D3−A3ML−06
TCGA−D3−A1QA−07
TCGA−GN−A268−06
TCGA−EE−A20C−06
TCGA−GN−A8LK−06
TCGA−FS−A1YW−06
TCGA−D9−A4Z6−06
TCGA−FS−A1ZY−06
TCGA−EE−A29E−06
TCGA−EE−A2GR−06
TCGA−D3−A8GK−06
TCGA−DA−A1IA−06
TCGA−ER−A19F−06
TCGA−FS−A1Z3−06
TCGA−D3−A3C6−06
TCGA−EE−A180−06
TCGA−D9−A1X3−06
TCGA−EE−A3AH−06
TCGA−DA−A95W−06
TCGA−D3−A1QA−06
TCGA−W3−A824−06
TCGA−D3−A5GL−06
TCGA−EE−A29B−06
TCGA−3N−A9WB−06
TCGA−FS−A1ZG−06
TCGA−EE−A185−06
TCGA−GN−A8LL−06
TCGA−GN−A4U7−06

MLANA

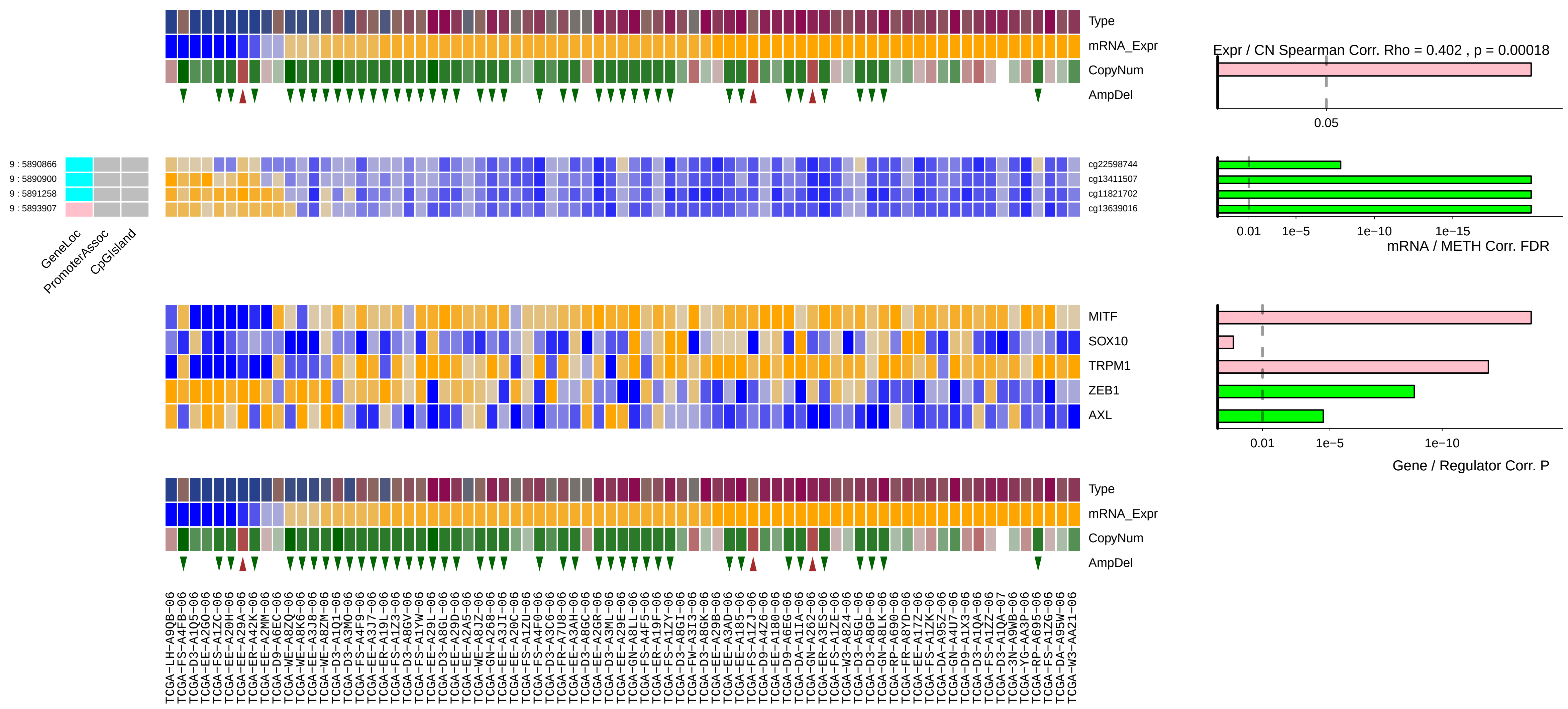

Type
mRNA_Expr
CopyNum
AmpDel
Expr / CN Spearman Corr. Rho = 0.402 , p = 0.00018
0.05
9 : 5890866
9 : 5890900
9 : 5891258
9 : 5893907
GeneLoc
PromoterAssoc
CpGIsland
cg22598744
cg13411507
cg11821702
cg13639016
0.01
1e−5
1e−10
1e−15
mRNA / METH Corr. FDR
MITF
SOX10
TRPM1
ZEB1
AXL
0.01
1e−5
1e−10
Gene / Regulator Corr. P
Type
mRNA_Expr
CopyNum
AmpDel

CDK2

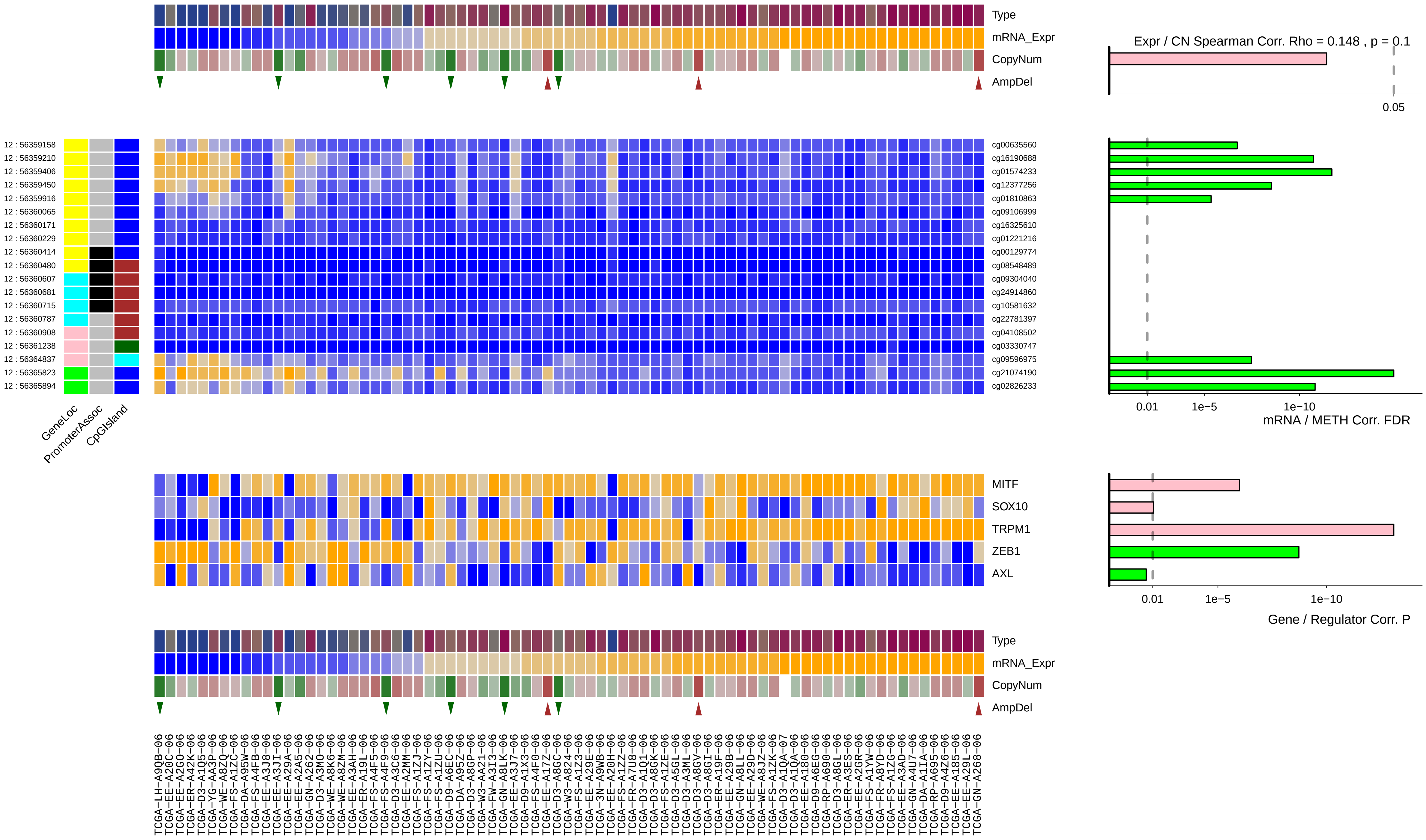

Type
mRNA_Expr
CopyNum
AmpDel
Expr / CN Spearman Corr. Rho = 0.148 , p = 0.1
0.05
12 : 56359158
12 : 56359210
12 : 56359406
12 : 56359450
12 : 56359916
12 : 56360065
12 : 56360171
12 : 56360229
12 : 56360414
12 : 56360480
12 : 56360607
12 : 56360681
12 : 56360715
12 : 56360787
12 : 56360908
12 : 56361238
12 : 56364837
12 : 56365823
12 : 56365894
cg00635560
cg16190688
cg01574233
cg12377256
cg01810863
cg09106999
cg16325610
cg01221216
cg00129774
cg08548489
cg09304040
cg24914860
cg10581632
cg22781397
cg04108502
cg03330747
cg09596975
cg21074190
cg02826233
0.01
1e−5
1e−10
mRNA / METH Corr. FDR
GeneLoc
PromoterAssoc
CpGIsland
MITF
SOX10
TRPM1
ZEB1
AXL
0.01
1e−5
1e−10
Gene / Regulator Corr. P
Type
mRNA_Expr
CopyNum
AmpDel

TTYH2

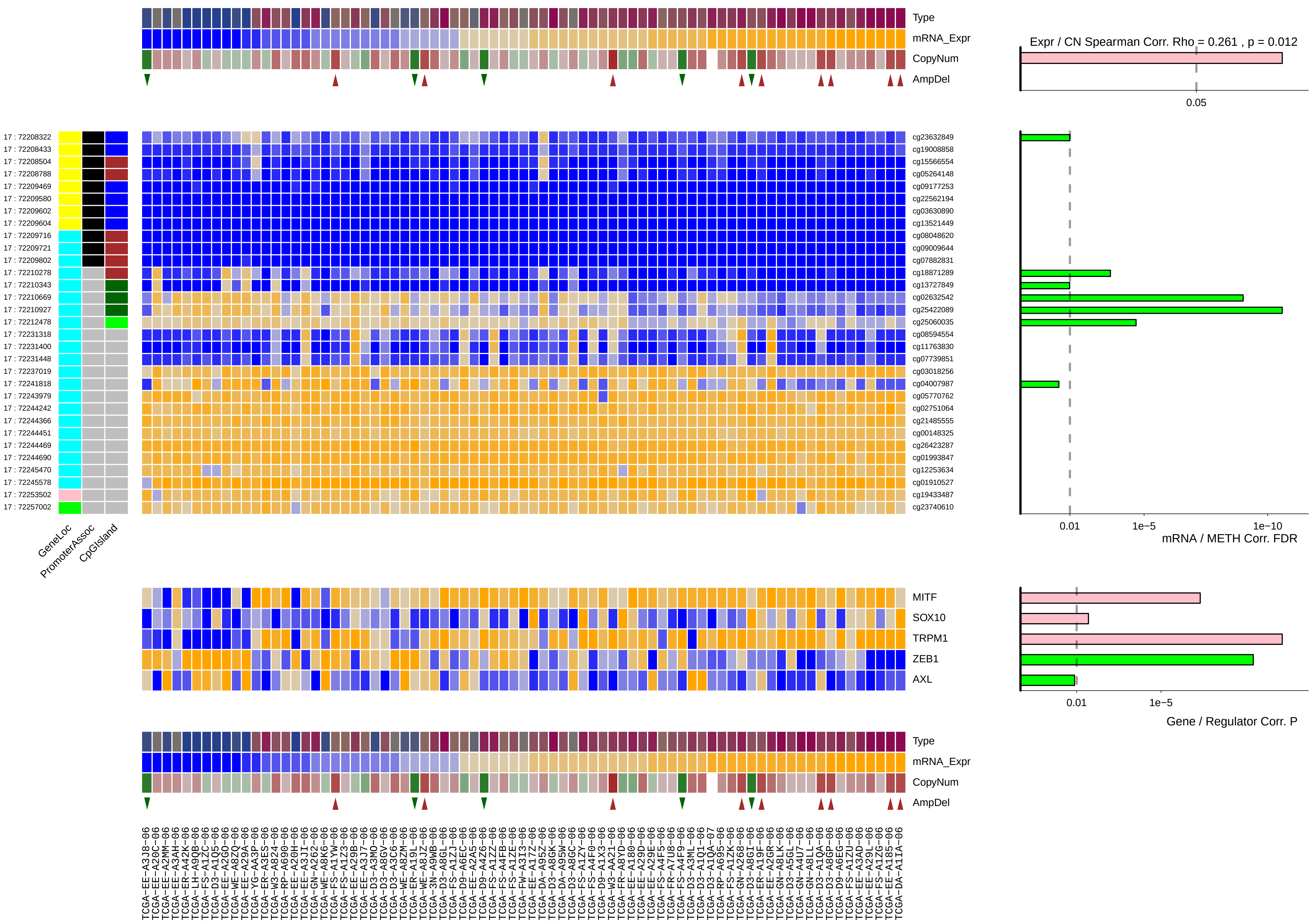

Type
mRNA_Expr
CopyNum
AmpDel
Expr / CN Spearman Corr. Rho = 0.261 , p = 0.012
0.05
17 : 72208322 cg23632849
17 : 72208433 cg19008858
17 : 72208504 cg15566554
17 : 72208788 cg05264148
17 : 72209469 cg09177253
17 : 72209580 cg22562194
17 : 72209602 cg03630890
17 : 72209604 cg13521449
17 : 72209716 cg08048620
17 : 72209721 cg09009644
17 : 72209802 cg07882831
17 : 72210278 cg18871289
17 : 72210343 cg13727849
17 : 72210669 cg02632542
17 : 72210927 cg25422089
17 : 72212478 cg25060035
17 : 72231318 cg08594554
17 : 72231400 cg11763830
17 : 72231448 cg07739851
17 : 72237019 cg03018256
17 : 72241818 cg04007987
17 : 72243979 cg05770762
17 : 72244242 cg02751064
17 : 72244366 cg21485555
17 : 72244451 cg00148325
17 : 72244469 cg26423287
17 : 72244690 cg01993847
17 : 72245470 cg12253634
17 : 72245578 cg01910527
17 : 72253502 cg19433487
17 : 72257002 cg23740610
GeneLoc
PromoterAssoc
CpGIsland
0.01
1e−5
1e−10
mRNA / METH Corr. FDR
MITF
SOX10
TRPM1
ZEB1
AXL
0.01
1e−5
Gene / Regulator Corr. P
Type
mRNA_Expr
CopyNum
AmpDel

SLC24A5

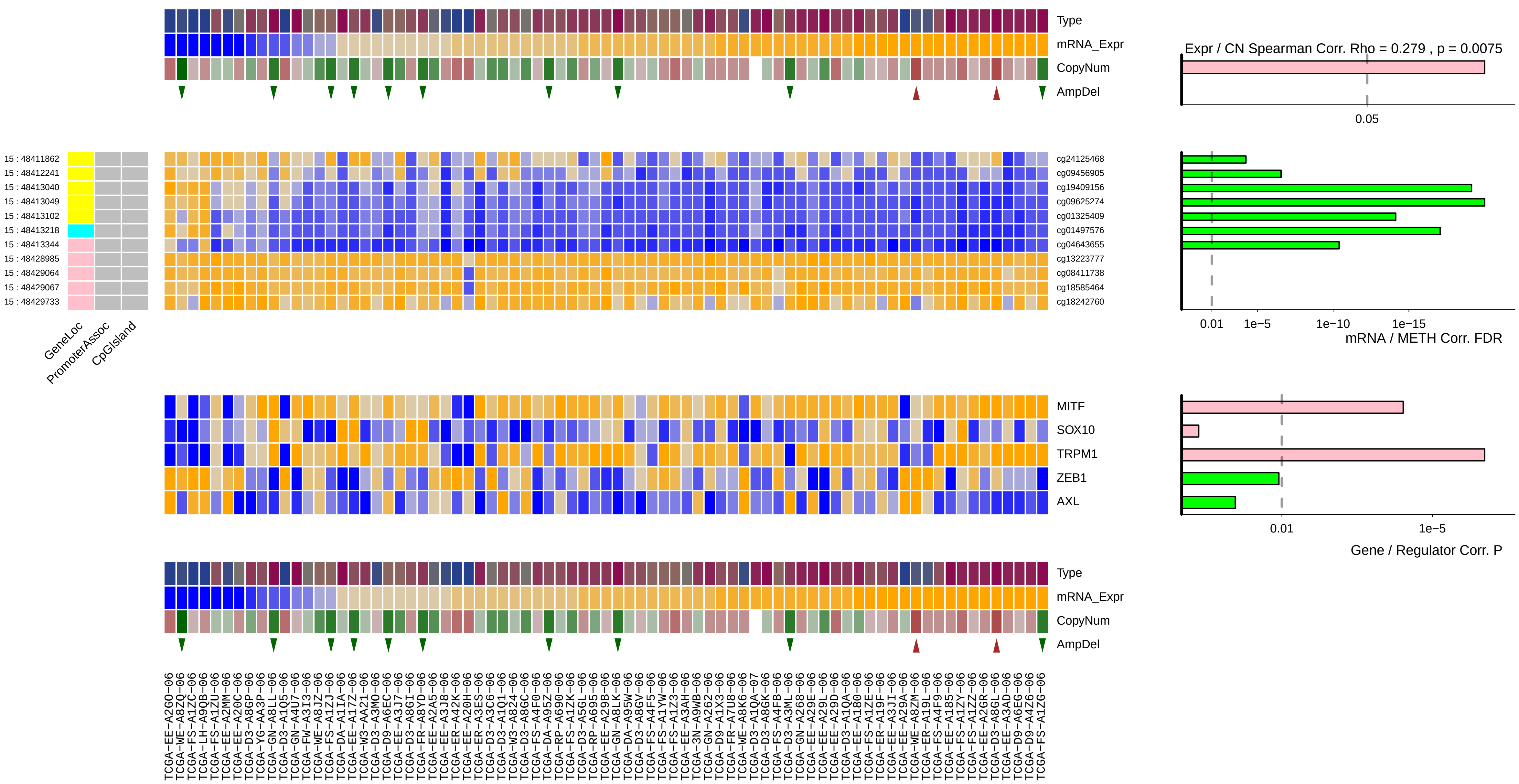

Type
mRNA_Expr
CopyNum
AmpDel
Expr / CN Spearman Corr. Rho = 0.279 , p = 0.0075
0.05
15 : 48411862
15 : 48412241
15 : 48413040
15 : 48413049
15 : 48413102
15 : 48413218
15 : 48413344
15 : 48428985
15 : 48429064
15 : 48429067
15 : 48429733
cg24125468
cg09456905
cg19409156
cg09625274
cg01325409
cg01497576
cg04643655
cg13223777
cg08411738
cg18585464
cg18242760
GeneLoc
PromoterAssoc
CpGIsland
0.01
1e−5
1e−10
1e−15
mRNA / METH Corr. FDR
MITF
SOX10
TRPM1
ZEB1
AXL
0.01
1e−5
Gene / Regulator Corr. P
Type
mRNA_Expr
CopyNum
AmpDel

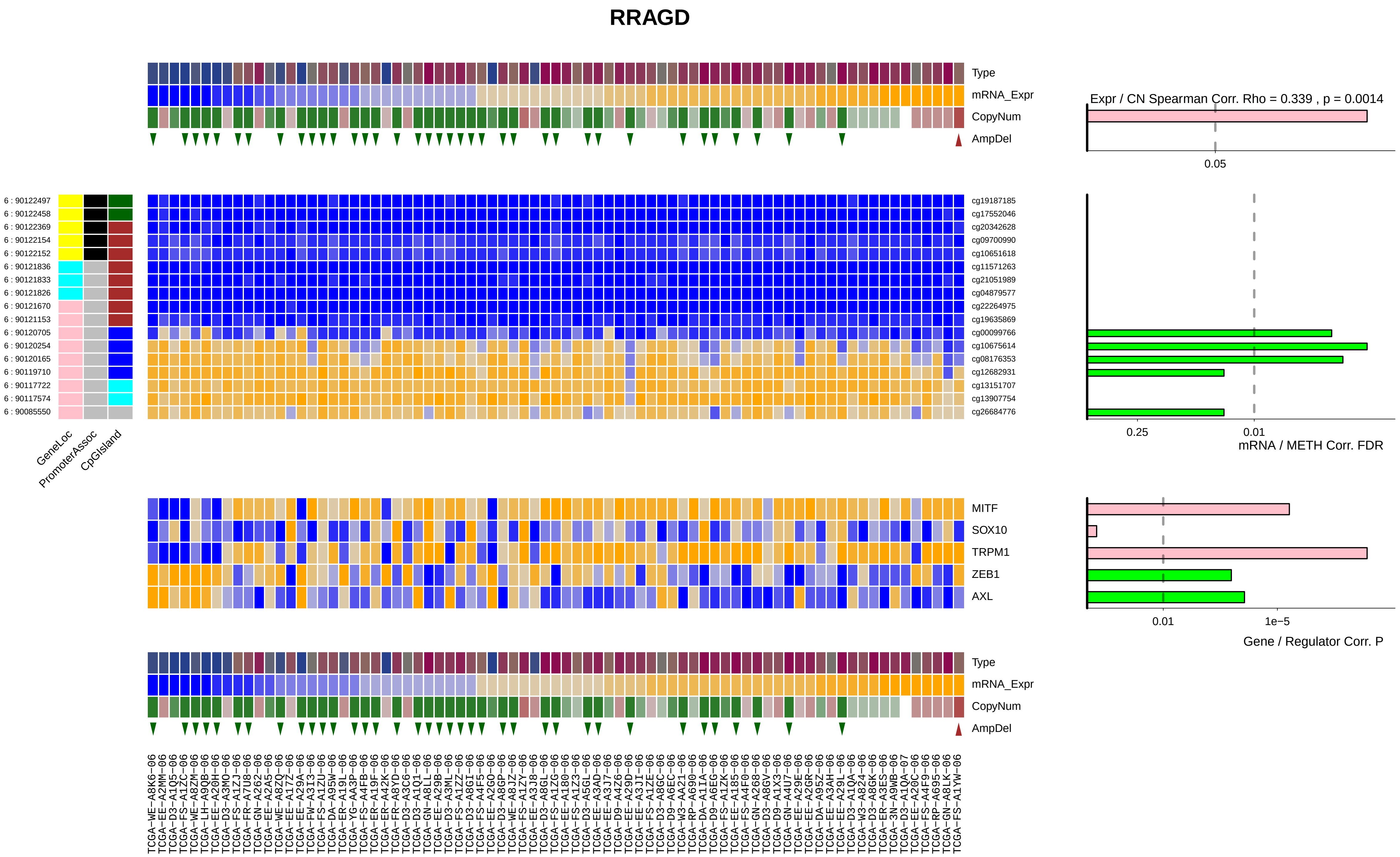

RRAGD
Type
mRNA_Expr
CopyNum
AmpDel
Expr / CN Spearman Corr. Rho = 0.339 , p = 0.0014
0.05
6 : 90122497
6 : 90122458
6 : 90122369
6 : 90122154
6 : 90122152
6 : 90121836
6 : 90121833
6 : 90121826
6 : 90121670
6 : 90121153
6 : 90120705
6 : 90120254
6 : 90120165
6 : 90119710
6 : 90117722
6 : 90117574
6 : 90085550
cg19187185
cg17552046
cg20342628
cg09700990
cg10651618
cg11571263
cg21051989
cg04879577
cg22264975
cg19635869
cg00099766
cg10675614
cg08176353
cg12682931
cg13151707
cg13907754
cg26684776
0.25
0.01
mRNA / METH Corr. FDR
GeneLoc
PromoterAssoc
CpGIsland
MITF
SOX10
TRPM1
ZEB1
AXL
0.01
1e−5
Gene / Regulator Corr. P
Type
mRNA_Expr
CopyNum
AmpDel

TRIM63

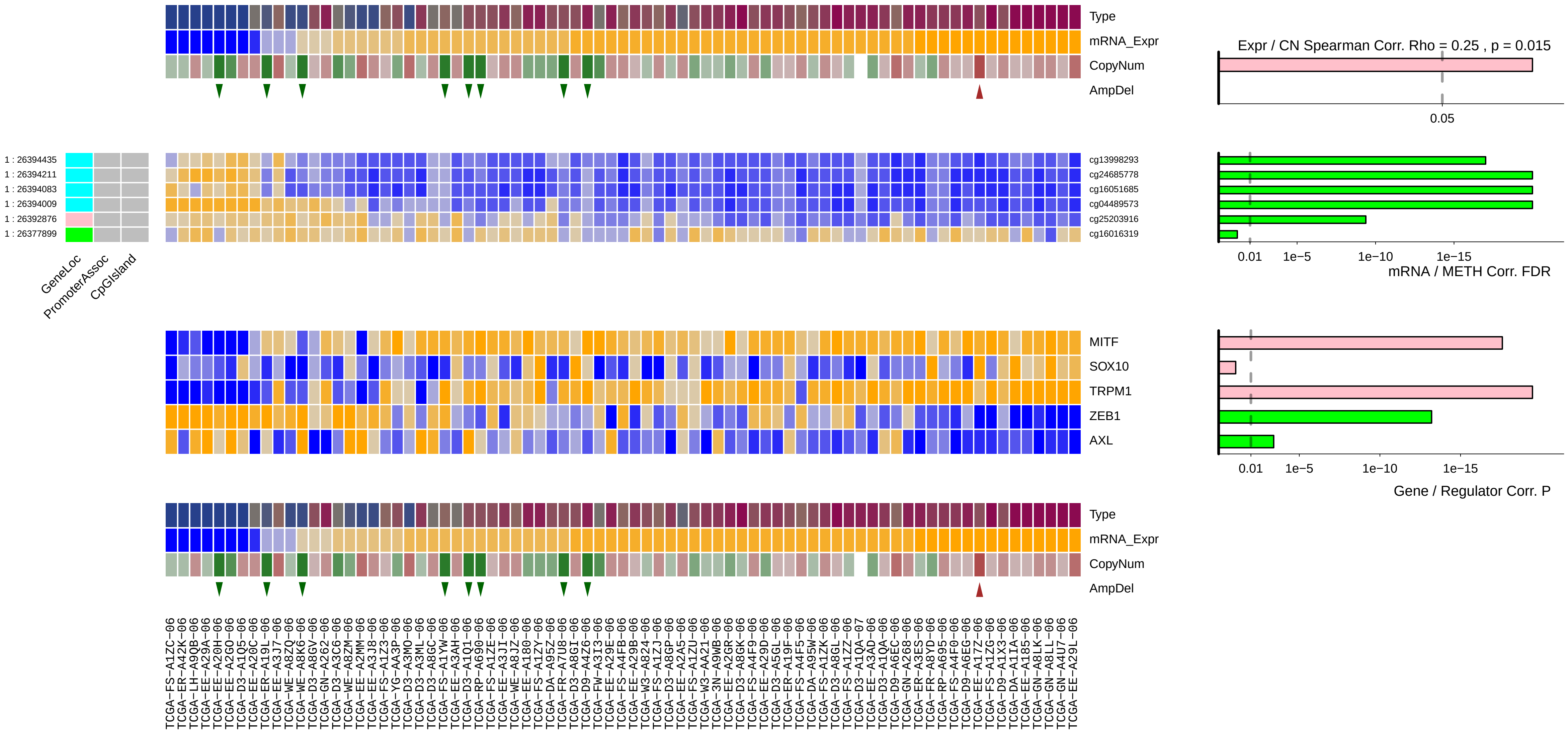

Type
mRNA_Expr
CopyNum
AmpDel
Expr / CN Spearman Corr. Rho = 0.25 , p = 0.015
0.05
cg13998293
cg24685778
cg16051685
cg04489573
cg25203916
cg16016319
1 : 26394435
1 : 26394211
1 : 26394083
1 : 26394009
1 : 26392876
1 : 26377899
GeneLoc
PromoterAssoc
CpGIsland
mRNA / METH Corr. FDR
MITF
SOX10
TRPM1
ZEB1
AXL
Gene / Regulator Corr. P

GPM6B

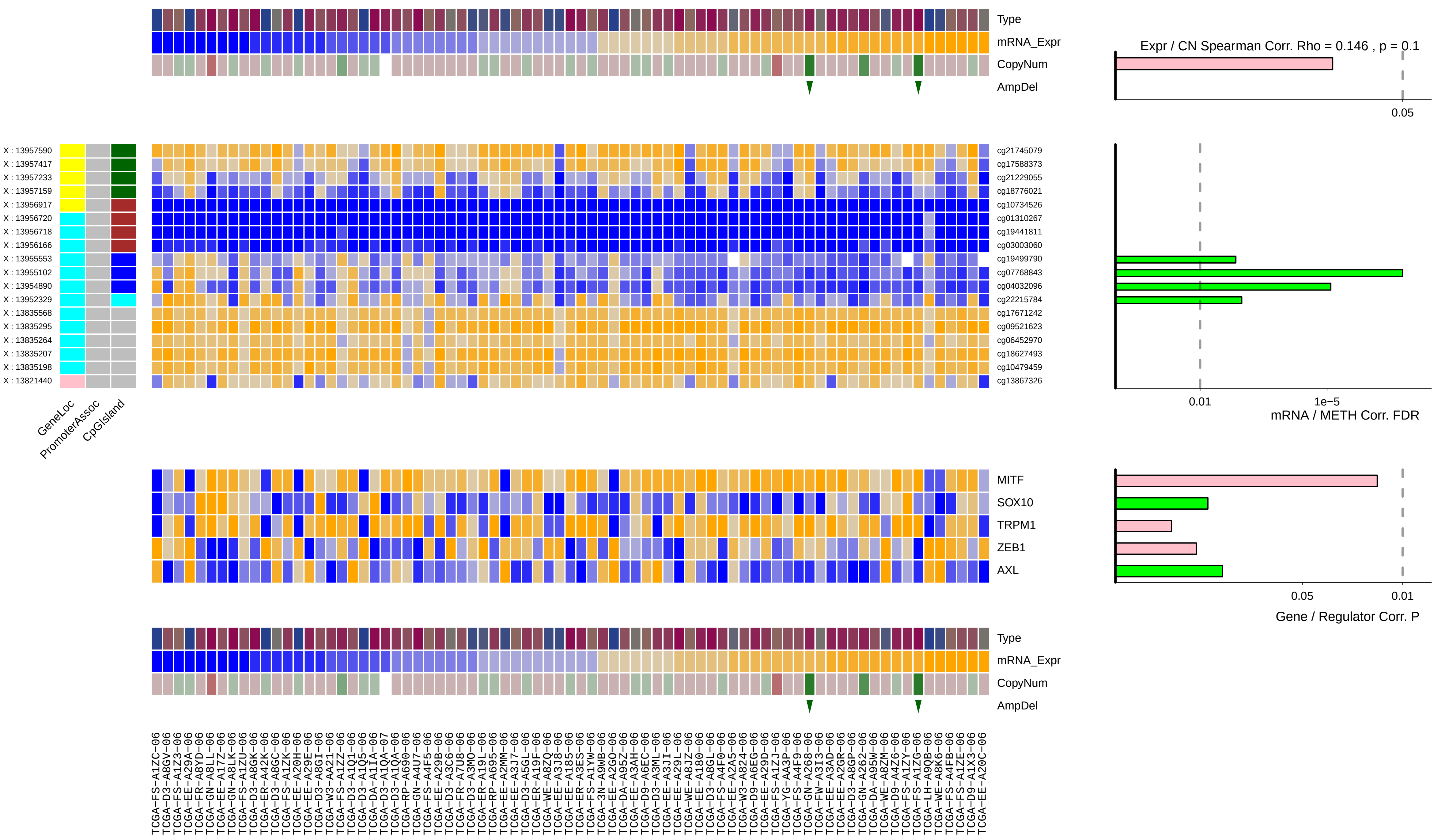

Type
mRNA_Expr
CopyNum
AmpDel
Expr / CN Spearman Corr. Rho = 0.146 , p = 0.1
0.05
X : 13957590
X : 13957417
X : 13957233
X : 13957159
X : 13956917
X : 13956720
X : 13956718
X : 13956166
X : 13955553
X : 13955102
X : 13954890
X : 13952329
X : 13835568
X : 13835295
X : 13835264
X : 13835207
X : 13835198
X : 13821440
GeneLoc
PromoterAssoc
CpGIsland
cg21745079
cg17588373
cg21229055
cg18776021
cg10734526
cg01310267
cg19441811
cg03003060
cg19499790
cg07768843
cg04032096
cg22215784
cg17671242
cg09521623
cg06452970
cg18627493
cg10479459
cg13867326
0.01
1e−5
mRNA / METH Corr. FDR
MITF
SOX10
TRPM1
ZEB1
AXL
0.05
0.01
Gene / Regulator Corr. P
Type
mRNA_Expr
CopyNum
AmpDel

SOX10

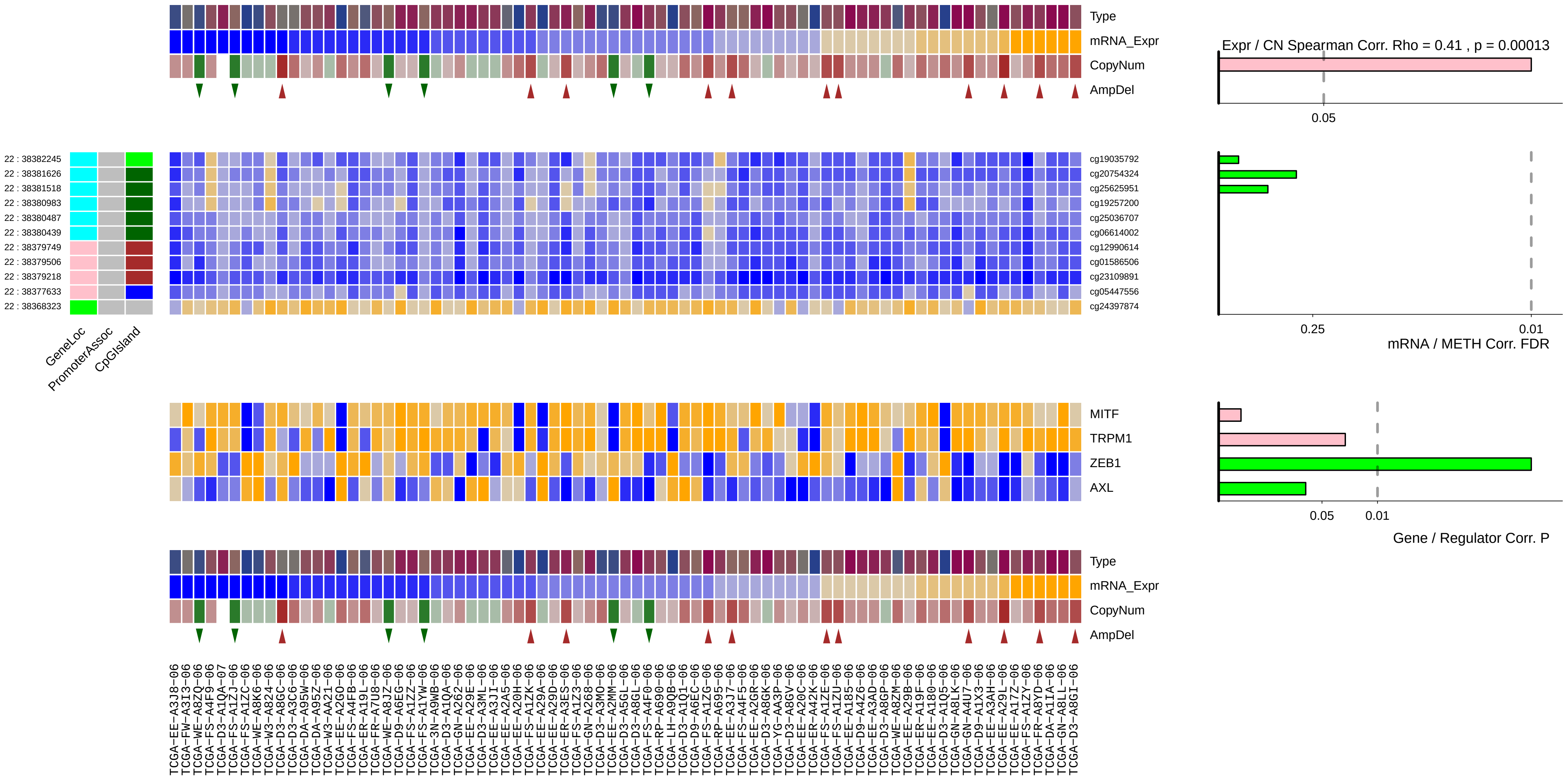

Type
mRNA_Expr
CopyNum
AmpDel
Expr / CN Spearman Corr. Rho = 0.41 , p = 0.00013
0.05
22 : 38382245
22 : 38381626
22 : 38381518
22 : 38380983
22 : 38380487
22 : 38380439
22 : 38379749
22 : 38379506
22 : 38379218
22 : 38377633
22 : 38368323
cg19035792
cg20754324
cg25625951
cg19257200
cg25036707
cg06614002
cg12990614
cg01586506
cg23109891
cg05447556
cg24397874
GeneLoc
PromoterAssoc
CpGIsland
0.25
0.01
mRNA / METH Corr. FDR
MITF
TRPM1
ZEB1
AXL
0.05
0.01
Gene / Regulator Corr. P
Type
mRNA_Expr
CopyNum
AmpDel

FAM69B

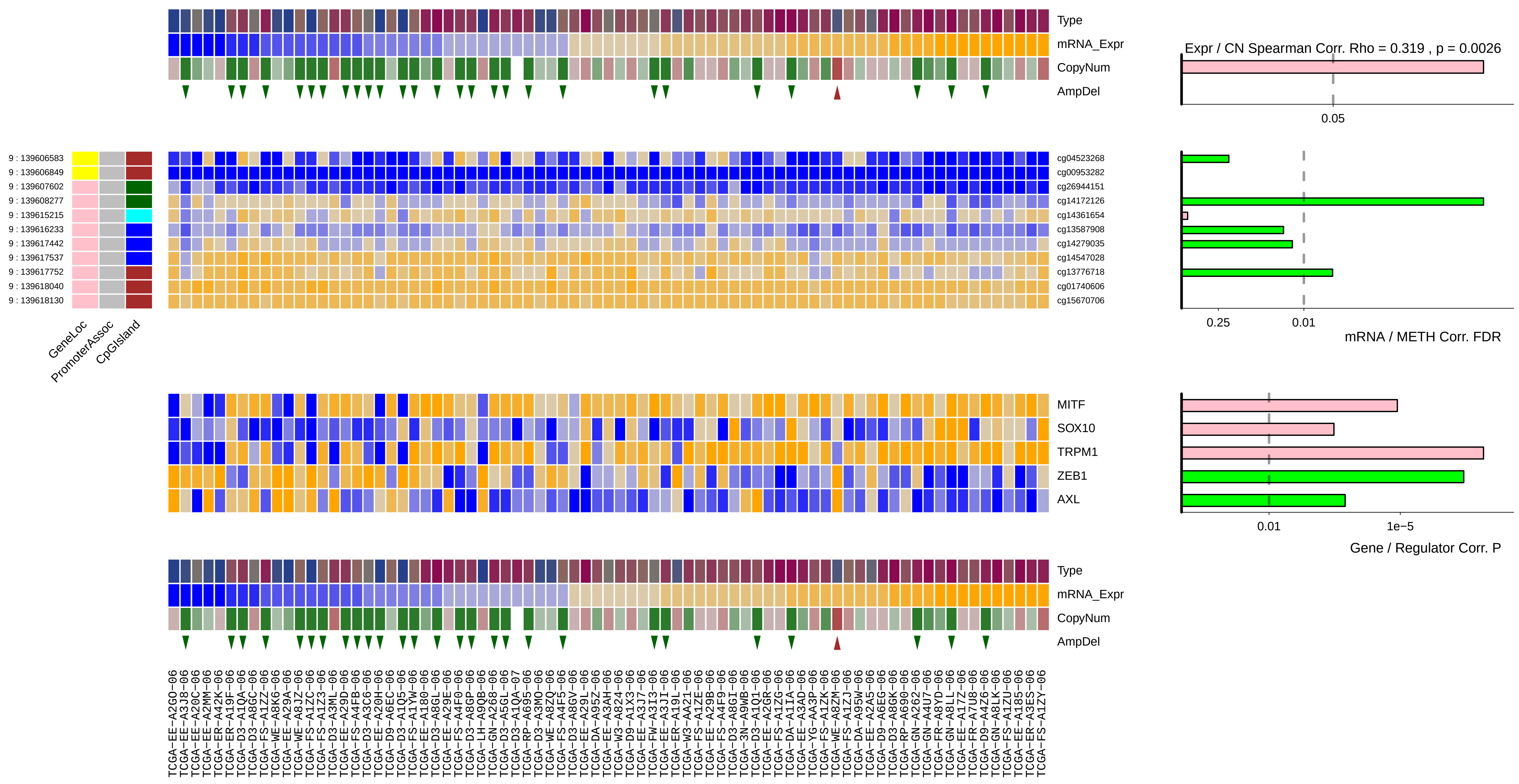

Type
mRNA_Expr
CopyNum
AmpDel
Expr / CN Spearman Corr. Rho = 0.319 , p = 0.0026
0.05
9 : 139606583
9 : 139606849
9 : 139607602
9 : 139608277
9 : 139615215
9 : 139616233
9 : 139617442
9 : 139617537
9 : 139617752
9 : 139618040
9 : 139618130
cg04523268
cg00953282
cg26944151
cg14172126
cg14361654
cg13587908
cg14279035
cg14547028
cg13776718
cg01740606
cg15670706
0.25
0.01
mRNA / METH Corr. FDR
GeneLoc
PromoterAssoc
CpGIsland
MITF
SOX10
TRPM1
ZEB1
AXL
0.01
1e−5
Gene / Regulator Corr. P
Type
mRNA_Expr
CopyNum
AmpDel

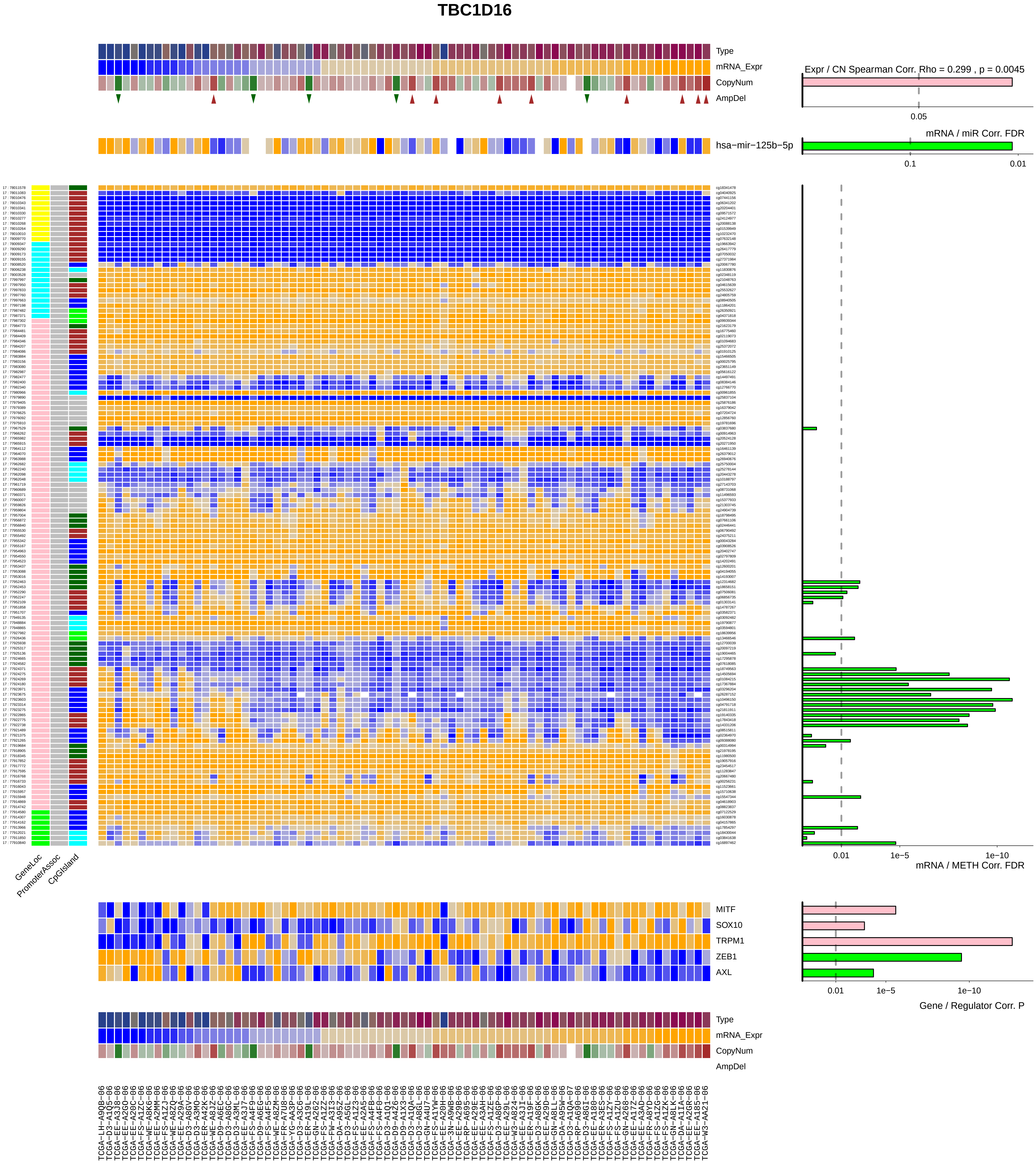
TBC1D16
Type
mRNA_Expr
CopyNum
AmpDel
Expr / CN Spearman Corr. Rho = 0.299 , p = 0.0045
0.05
mRNA / miR Corr. FDR
hsa−mir−125b−5p
0.1
0.01
0.01
1e−5
1e−10
mRNA / METH Corr. FDR
GeneLoc
PromoterAssoc
CpGIsland
MITF
SOX10
TRPM1
ZEB1
AXL
0.01
1e−5
1e−10
Gene / Regulator Corr. P
Type
mRNA_Expr
CopyNum
AmpDel

RENBP

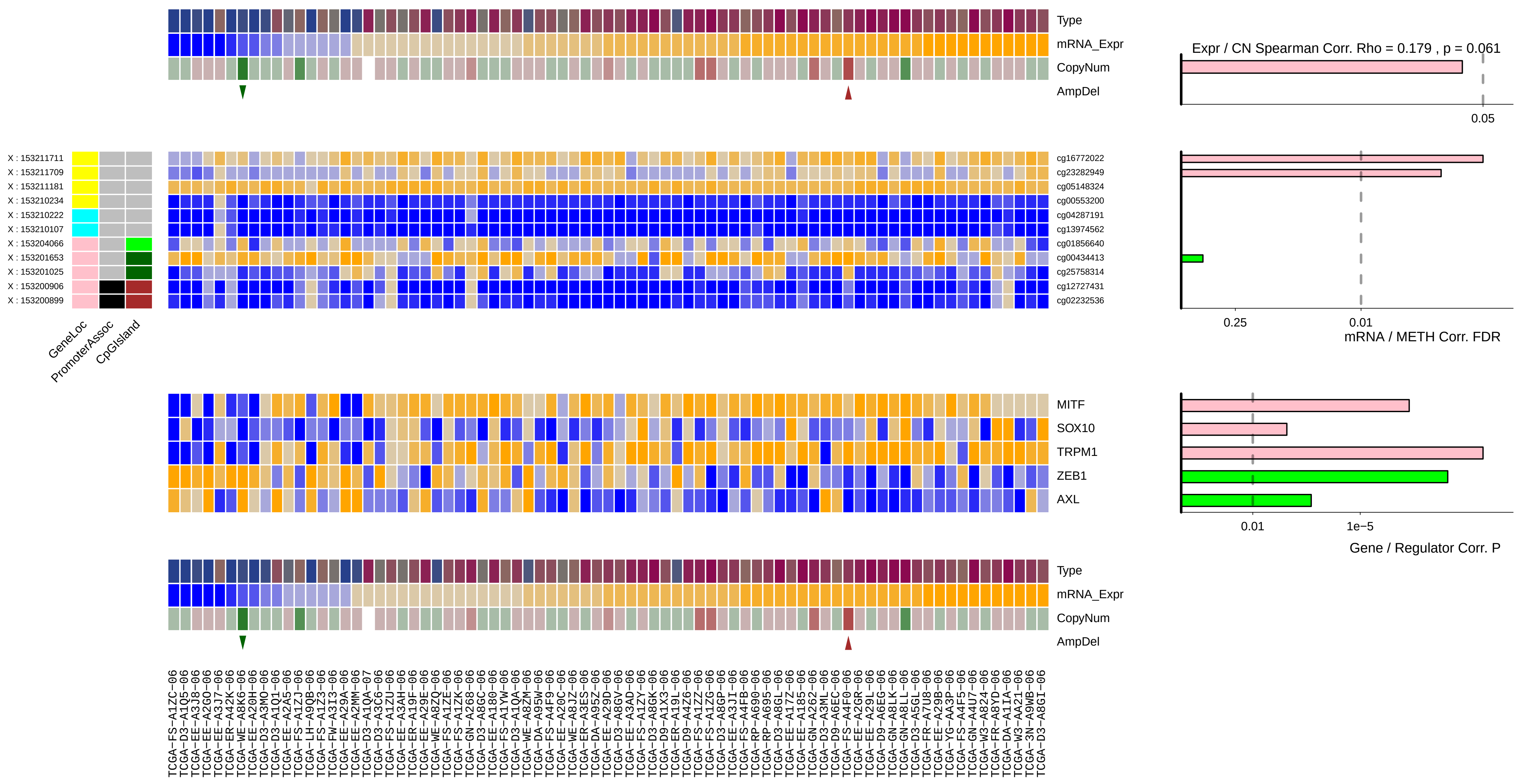
Type
mRNA_Expr
CopyNum
AmpDel
Expr / CN Spearman Corr. Rho = 0.179 , p = 0.061
0.05
X : 153211711
X : 153211709
X : 153211181
X : 153210234
X : 153210222
X : 153210107
X : 153204066
X : 153201653
X : 153201025
X : 153200906
X : 153200899
cg16772022
cg23282949
cg05148324
cg00553200
cg04287191
cg13974562
cg01856640
cg00434413
cg25758314
cg12727431
cg02232536
GeneLoc
PromoterAssoc
CpGIsland
0.25
0.01
mRNA / METH Corr. FDR
MITF
SOX10
TRPM1
ZEB1
AXL
0.01
1e−5
Gene / Regulator Corr. P
Type
mRNA_Expr
CopyNum
AmpDel

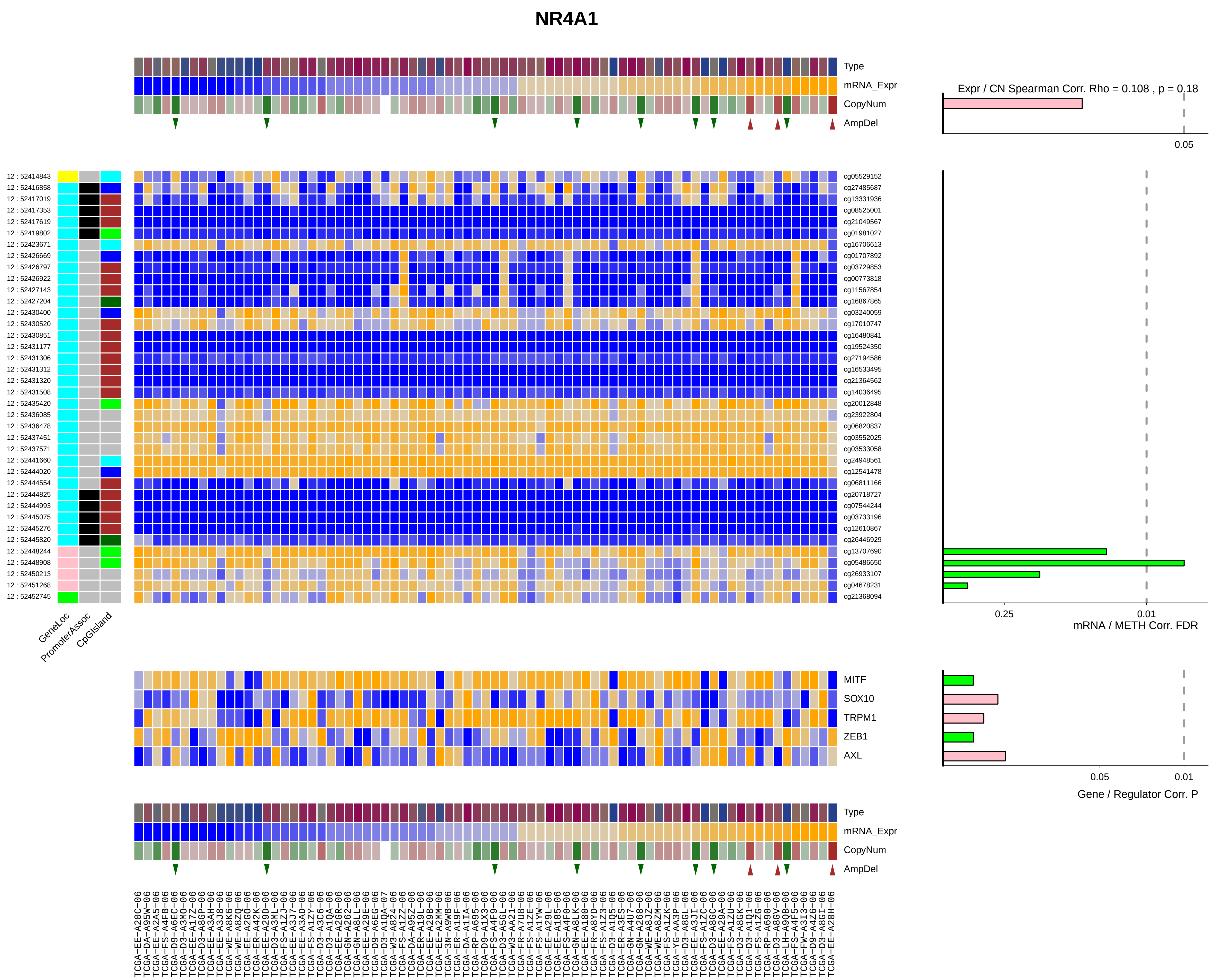
NR4A1
Type
mRNA_Expr
CopyNum
AmpDel
Expr / CN Spearman Corr. Rho = 0.108 , p = 0.18
0.05
12 : 52414843
12 : 52416858
12 : 52417019
12 : 52417353
12 : 52417619
12 : 52419802
12 : 52423671
12 : 52426669
12 : 52426797
12 : 52426922
12 : 52427143
12 : 52427204
12 : 52430400
12 : 52430520
12 : 52430851
12 : 52431177
12 : 52431306
12 : 52431312
12 : 52431320
12 : 52431508
12 : 52435420
12 : 52436085
12 : 52436478
12 : 52437451
12 : 52437571
12 : 52441660
12 : 52444020
12 : 52444554
12 : 52444825
12 : 52444993
12 : 52445075
12 : 52445276
12 : 52445820
12 : 52448244
12 : 52448908
12 : 52450213
12 : 52451268
12 : 52452745
cg05529152
cg27485687
cg13331936
cg08525001
cg21049567
cg01981027
cg16706613
cg01707892
cg03729853
cg00773818
cg11567854
cg16867865
cg03240059
cg17010747
cg16480841
cg19524350
cg27194586
cg16533495
cg21364562
cg14036495
cg20012848
cg23922804
cg06820837
cg03552025
cg03533058
cg24948561
cg12541478
cg06811166
cg20718727
cg07544244
cg03733196
cg12610867
cg26446929
cg13707690
cg05486650
cg26933107
cg04678231
cg21368094
GeneLoc
PromoterAssoc
CpGIsland
0.25
0.01
mRNA / METH Corr. FDR
MITF
SOX10
TRPM1
ZEB1
AXL
0.05
0.01
Gene / Regulator Corr. P
Type
mRNA_Expr
CopyNum
AmpDel

IGSF11

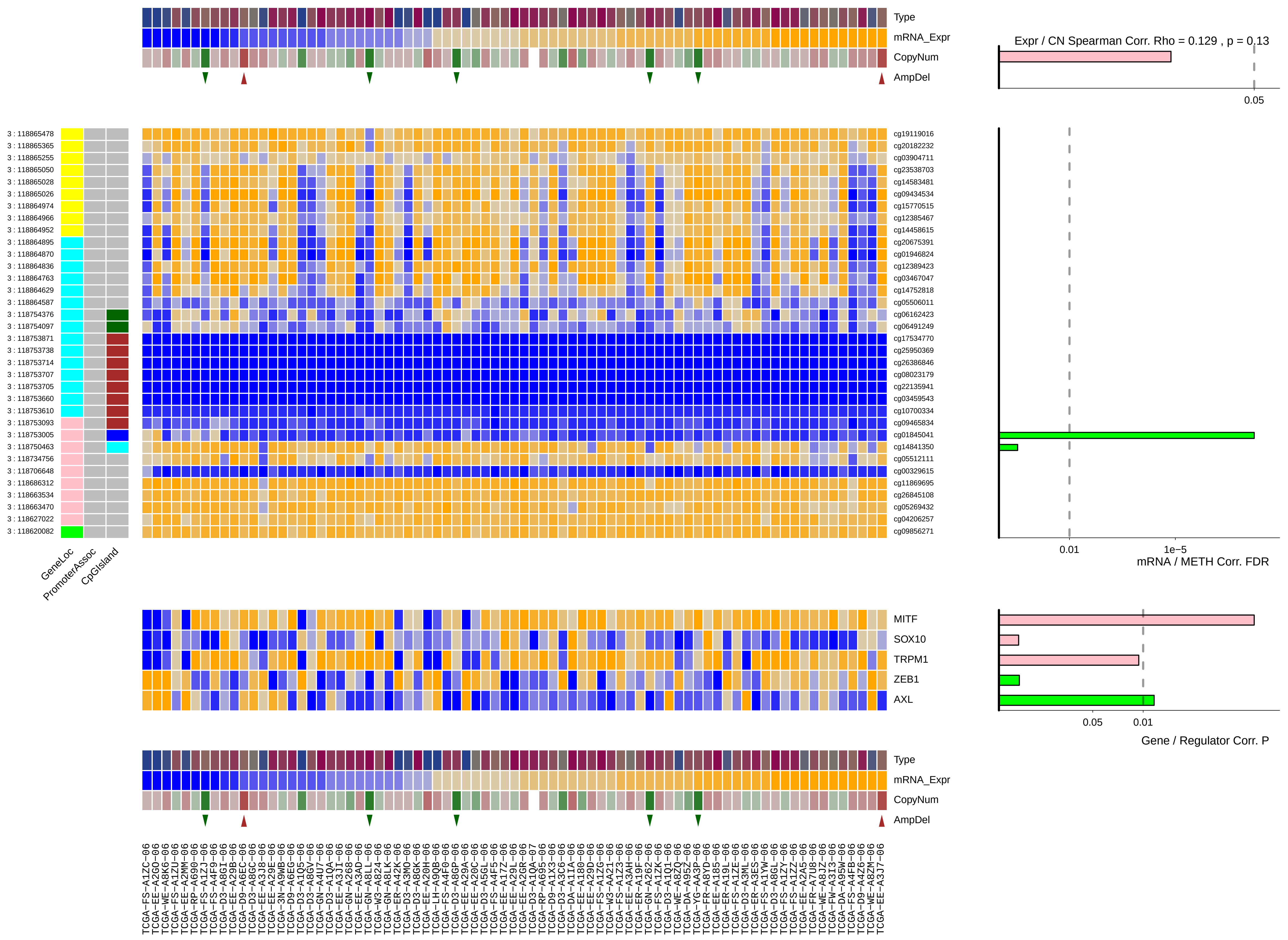
Type
mRNA_Expr
CopyNum
AmpDel
Expr / CN Spearman Corr. Rho = 0.129 , p = 0.13
0.05
0.01
1e−5
mRNA / METH Corr. FDR
GeneLoc
PromoterAssoc
CpGIsland
MITF
SOX10
TRPM1
ZEB1
AXL
0.05
0.01
Gene / Regulator Corr. P

EDNRB

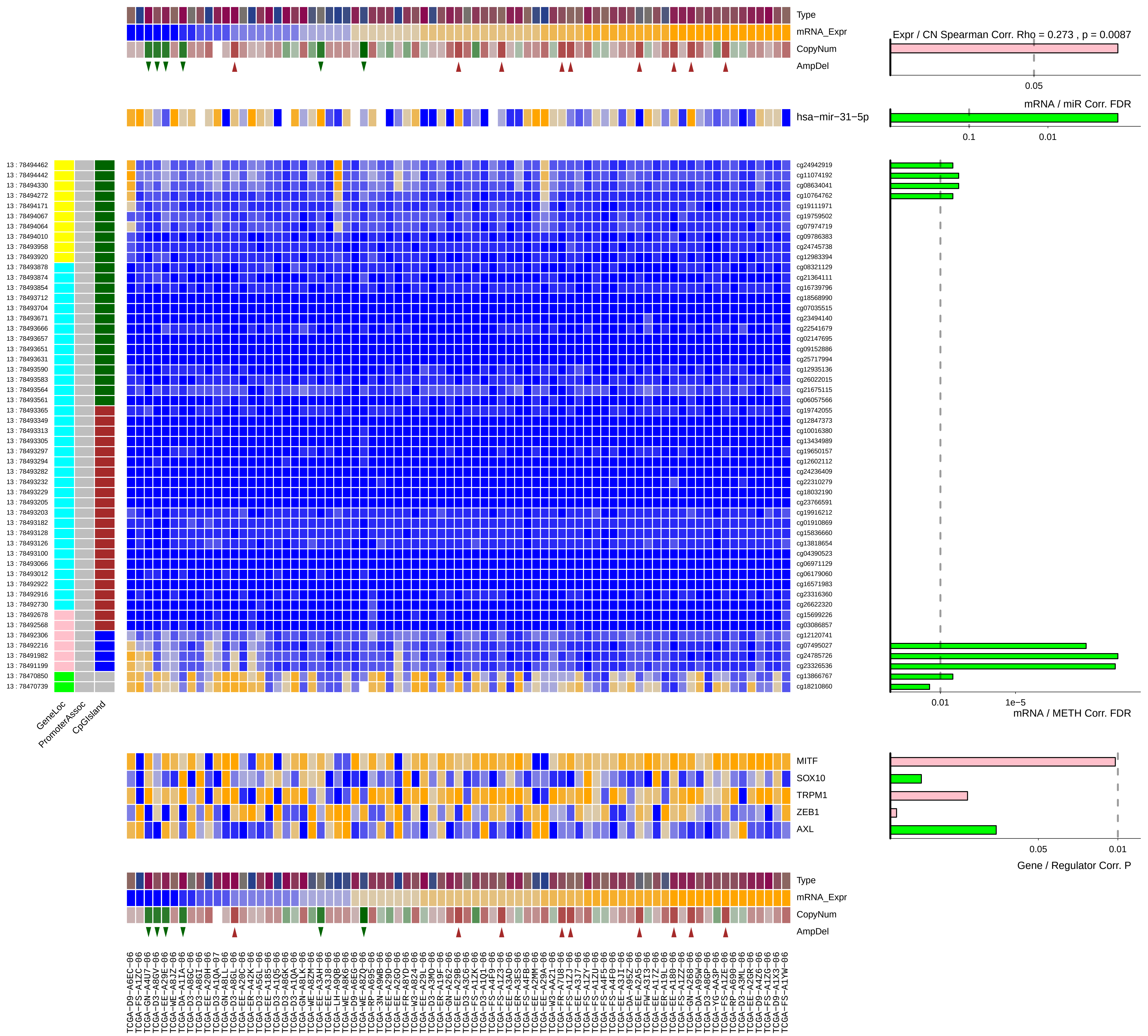

Type
mRNA_Expr
CopyNum
AmpDel
Expr / CN Spearman Corr. Rho = 0.273 , p = 0.0087
0.05
mRNA / miR Corr. FDR
hsa−mir−31−5p
0.1
0.01
GeneLoc
PromoterAssoc
CpGIsland
0.01
1e−5
mRNA / METH Corr. FDR
MITF
SOX10
TRPM1
ZEB1
AXL
0.05
0.01
Gene / Regulator Corr. P
Type
mRNA_Expr
CopyNum
AmpDel

GYG2

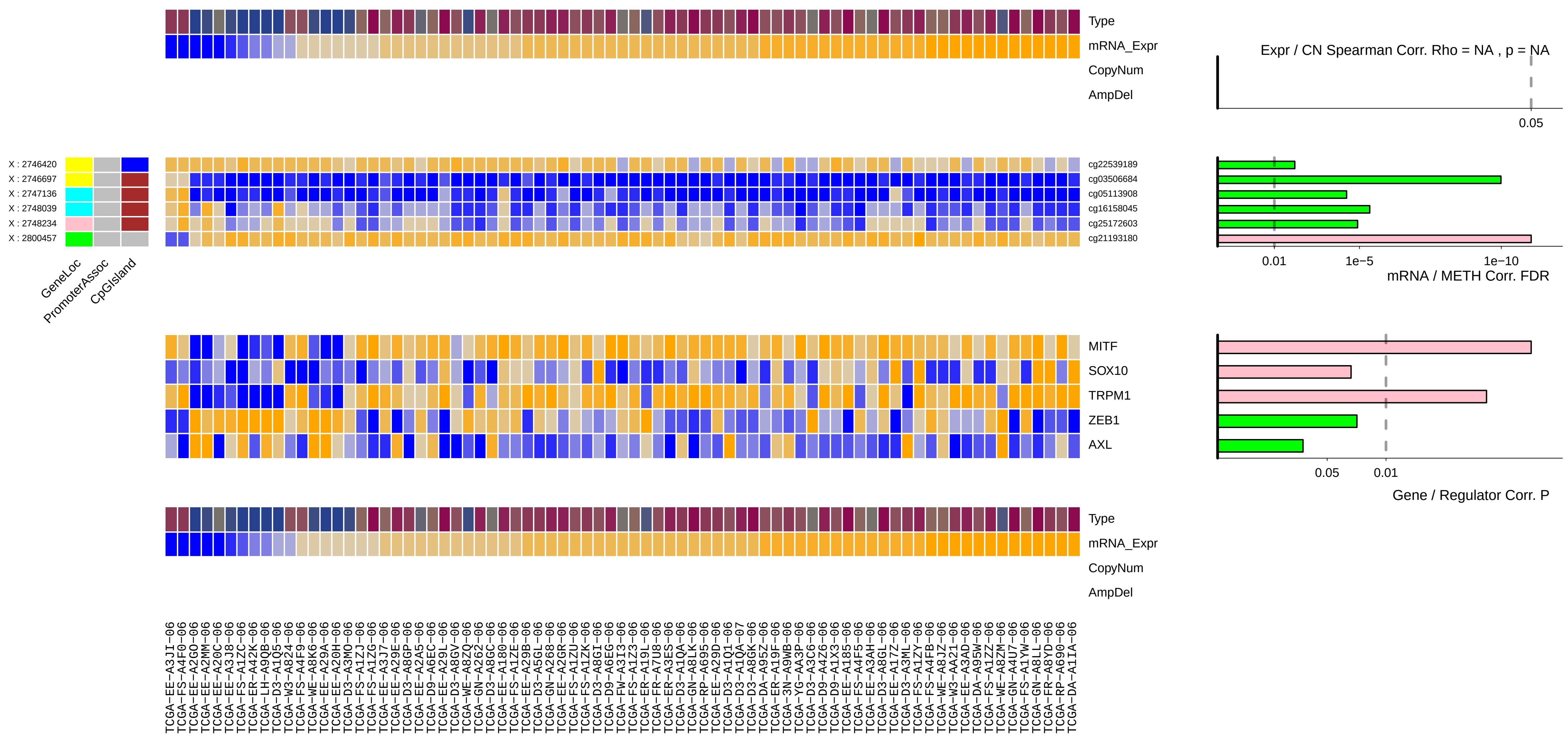

Type
mRNA_Expr
CopyNum
AmpDel
Expr / CN Spearman Corr. Rho = NA , p = NA
0.05
X : 2746420
X : 2746697
X : 2747136
X : 2748039
X : 2748234
X : 2800457
GeneLoc
PromoterAssoc
CpGIsland
cg22539189
cg03506684
cg05113908
cg16158045
cg25172603
cg21193180
0.01
1e−5
1e−10
mRNA / METH Corr. FDR
MITF
SOX10
TRPM1
ZEB1
AXL
0.05
0.01
Gene / Regulator Corr. P
Type
mRNA_Expr
CopyNum
AmpDel

CAPN3

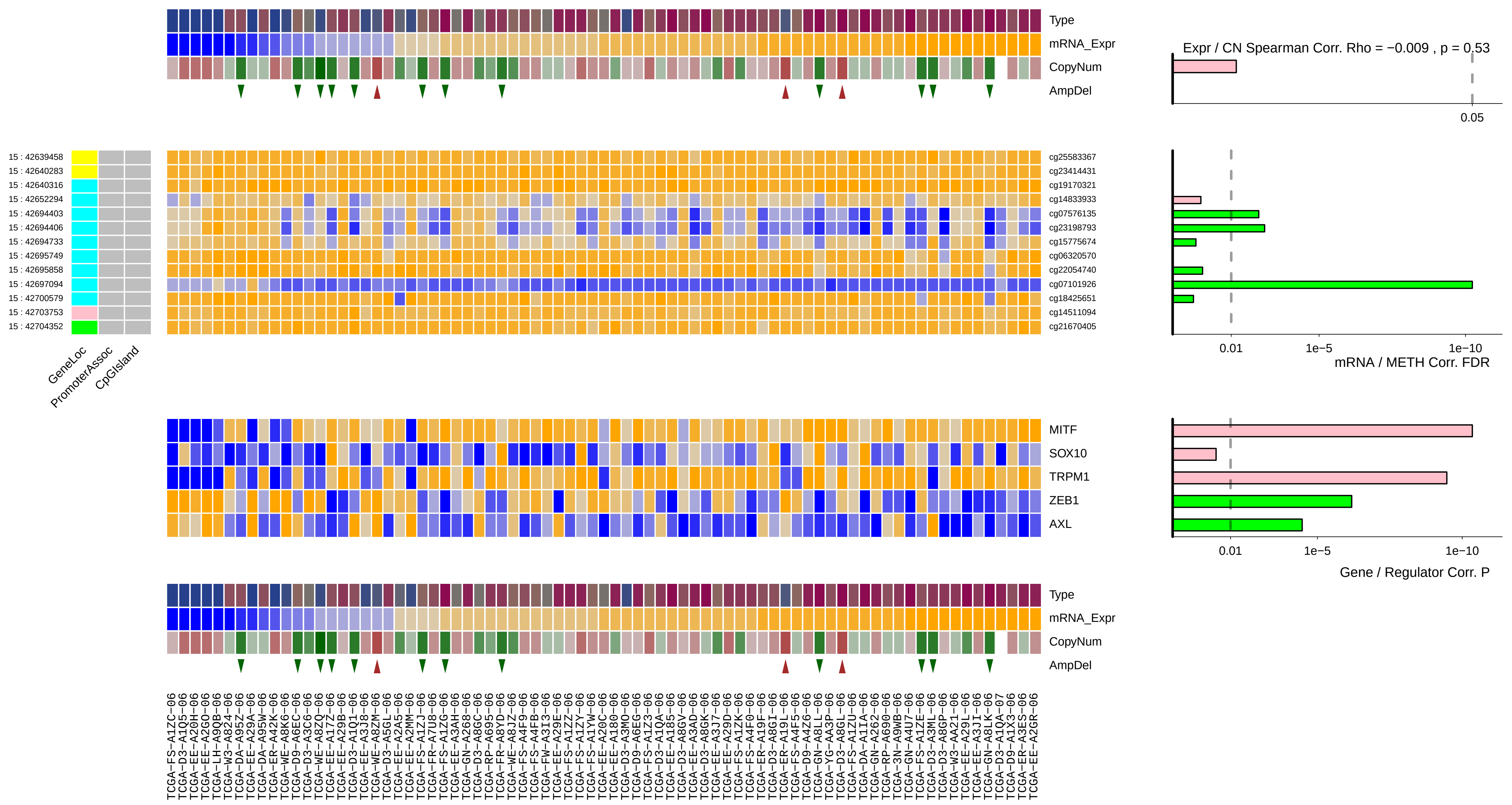
Type
mRNA_Expr
CopyNum
AmpDel
Expr / CN Spearman Corr. Rho = −0.009 , p = 0.53
0.05
15 : 42639458
15 : 42640283
15 : 42640316
15 : 42652294
15 : 42694403
15 : 42694406
15 : 42694733
15 : 42695749
15 : 42695858
15 : 42697094
15 : 42700579
15 : 42703753
15 : 42704352
GeneLoc
PromoterAssoc
CpGIsland
cg25583367
cg23414431
cg19170321
cg14833933
cg07576135
cg23198793
cg15775674
cg06320570
cg22054740
cg07101926
cg18425651
cg14511094
cg21670405
0.01
1e−5
1e−10
mRNA / METH Corr. FDR
MITF
SOX10
TRPM1
ZEB1
AXL
0.01
1e−5
1e−10
Gene / Regulator Corr. P
Type
mRNA_Expr
CopyNum
AmpDel

BEST1

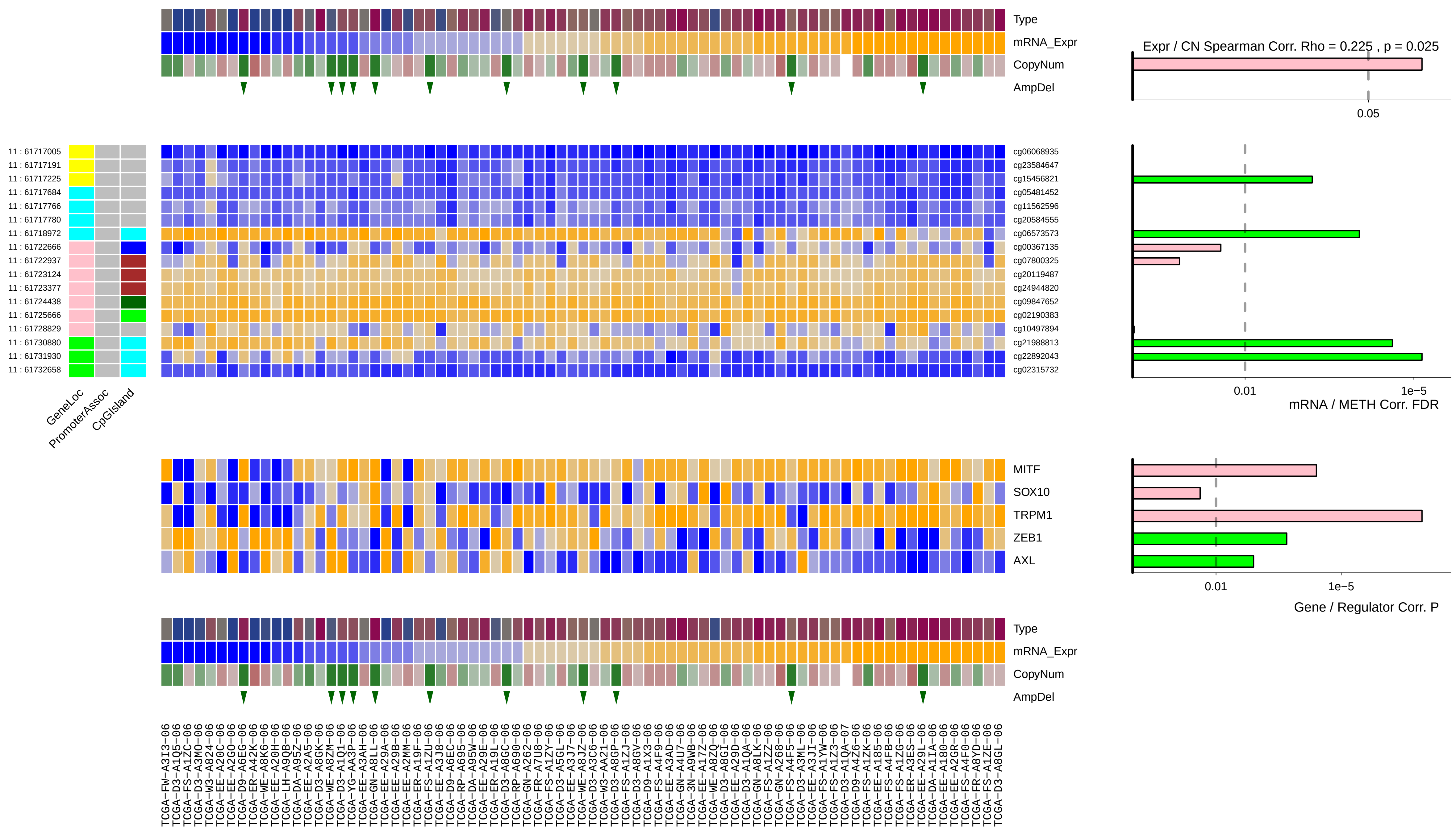

Type
mRNA_Expr
CopyNum
AmpDel
Expr / CN Spearman Corr. Rho = 0.225 , p = 0.025
0.05
11 : 61717005
11 : 61717191
11 : 61717225
11 : 61717684
11 : 61717766
11 : 61717780
11 : 61718972
11 : 61722666
11 : 61722937
11 : 61723124
11 : 61723377
11 : 61724438
11 : 61725666
11 : 61728829
11 : 61730880
11 : 61731930
11 : 61732658
GeneLoc
PromoterAssoc
CpGIsland
cg06068935
cg23584647
cg15456821
cg05481452
cg11562596
cg20584555
cg06573573
cg00367135
cg07800325
cg20119487
cg24944820
cg09847652
cg02190383
cg10497894
cg21988813
cg22892043
cg02315732
0.01
1e−5
mRNA / METH Corr. FDR
MITF
SOX10
TRPM1
ZEB1
AXL
0.01
1e−5
Gene / Regulator Corr. P
Type
mRNA_Expr
CopyNum
AmpDel

IRF4

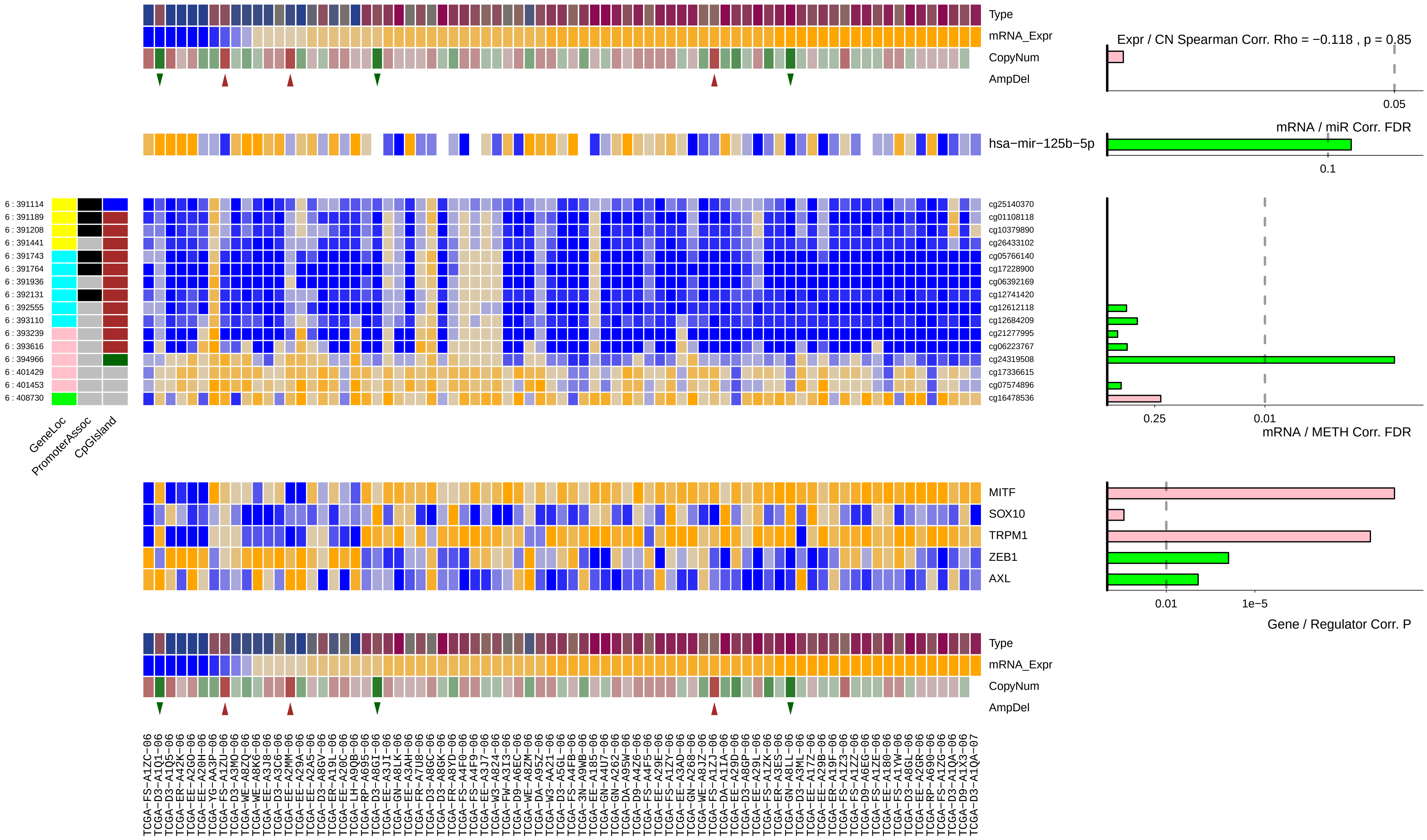
Type
mRNA_Expr
CopyNum
AmpDel
Expr / CN Spearman Corr. Rho = −0.118 , p = 0.85
0.05
hsa−mir−125b−5p
mRNA / miR Corr. FDR
0.1
6 : 391114
6 : 391189
6 : 391208
6 : 391441
6 : 391743
6 : 391764
6 : 391936
6 : 392131
6 : 392555
6 : 393110
6 : 393239
6 : 393616
6 : 394966
6 : 401429
6 : 401453
6 : 408730
cg25140370
cg01108118
cg10379890
cg26433102
cg05766140
cg17228900
cg06392169
cg12741420
cg12612118
cg12684209
cg21277995
cg06223767
cg24319508
cg17336615
cg07574896
cg16478536
GeneLoc
PromoterAssoc
CpGIsland
0.25
0.01
mRNA / METH Corr. FDR
MITF
SOX10
TRPM1
ZEB1
AXL
0.01
1e−5
Gene / Regulator Corr. P
Type
mRNA_Expr
CopyNum
AmpDel

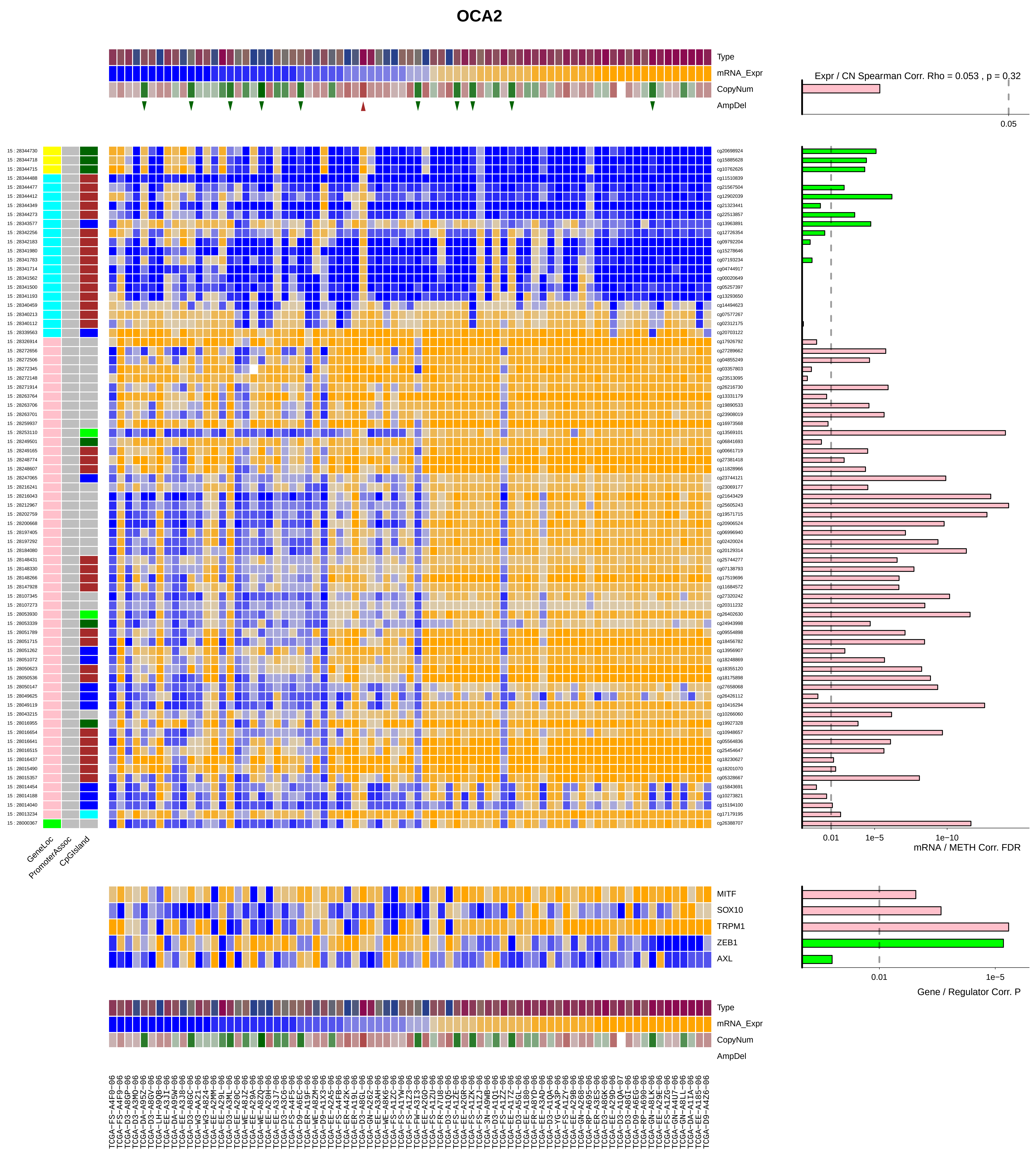

OCA2
Type
mRNA_Expr
CopyNum
AmpDel
Expr / CN Spearman Corr. Rho = 0.053 , p = 0.32
0.05
0.01
1e−5
1e−10
mRNA / METH Corr. FDR
GeneLoc
PromoterAssoc
CpGIsland
MITF
SOX10
TRPM1
ZEB1
AXL
Gene / Regulator Corr. P

TBC1D7

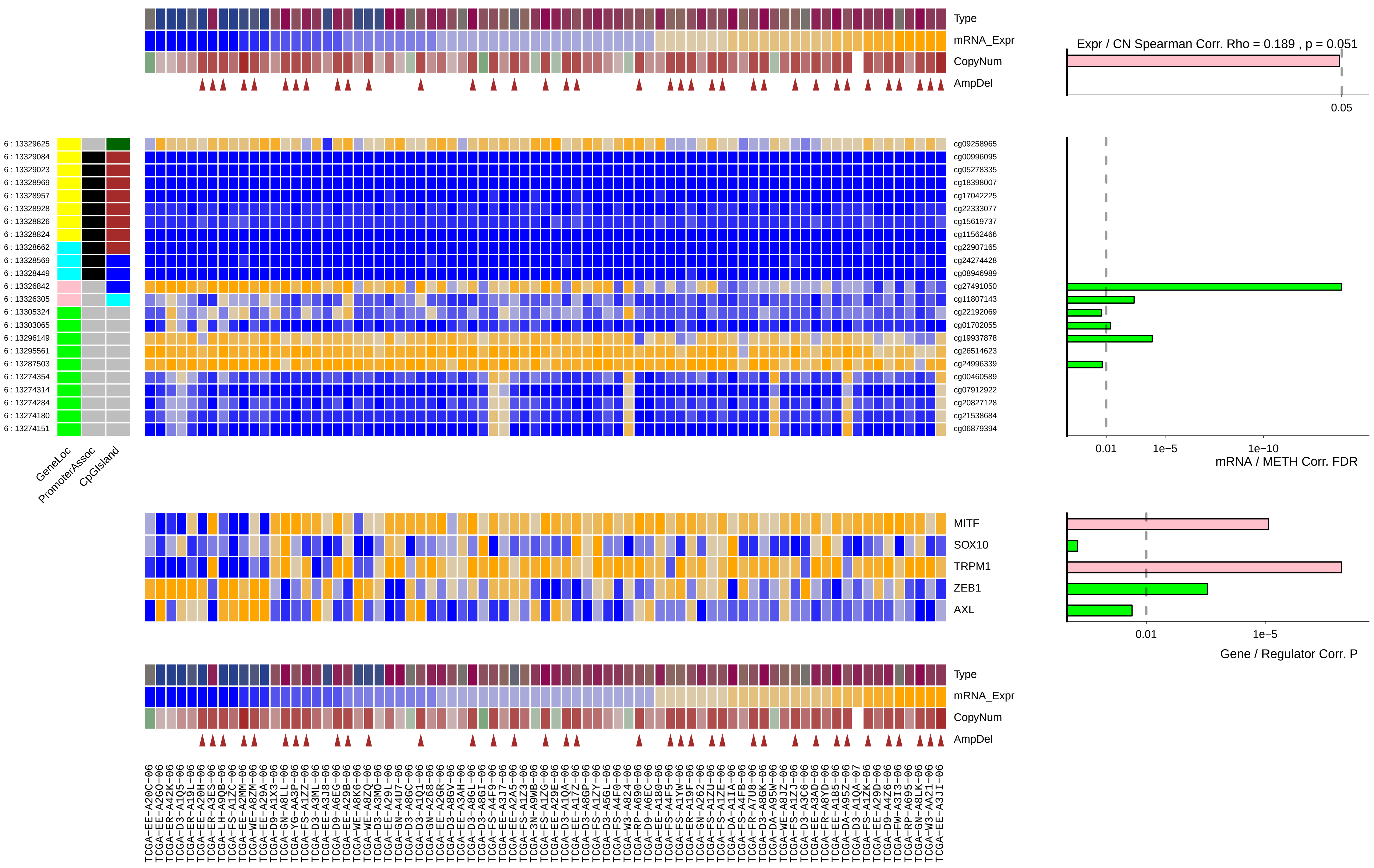
Type
mRNA_Expr
CopyNum
AmpDel
Expr / CN Spearman Corr. Rho = 0.189 , p = 0.051
0.05
GeneLoc
PromoterAssoc
CpGIsland
0.01
1e−5
1e−10
mRNA / METH Corr. FDR
MITF
SOX10
TRPM1
ZEB1
AXL
Gene / Regulator Corr. P

C10ORF90

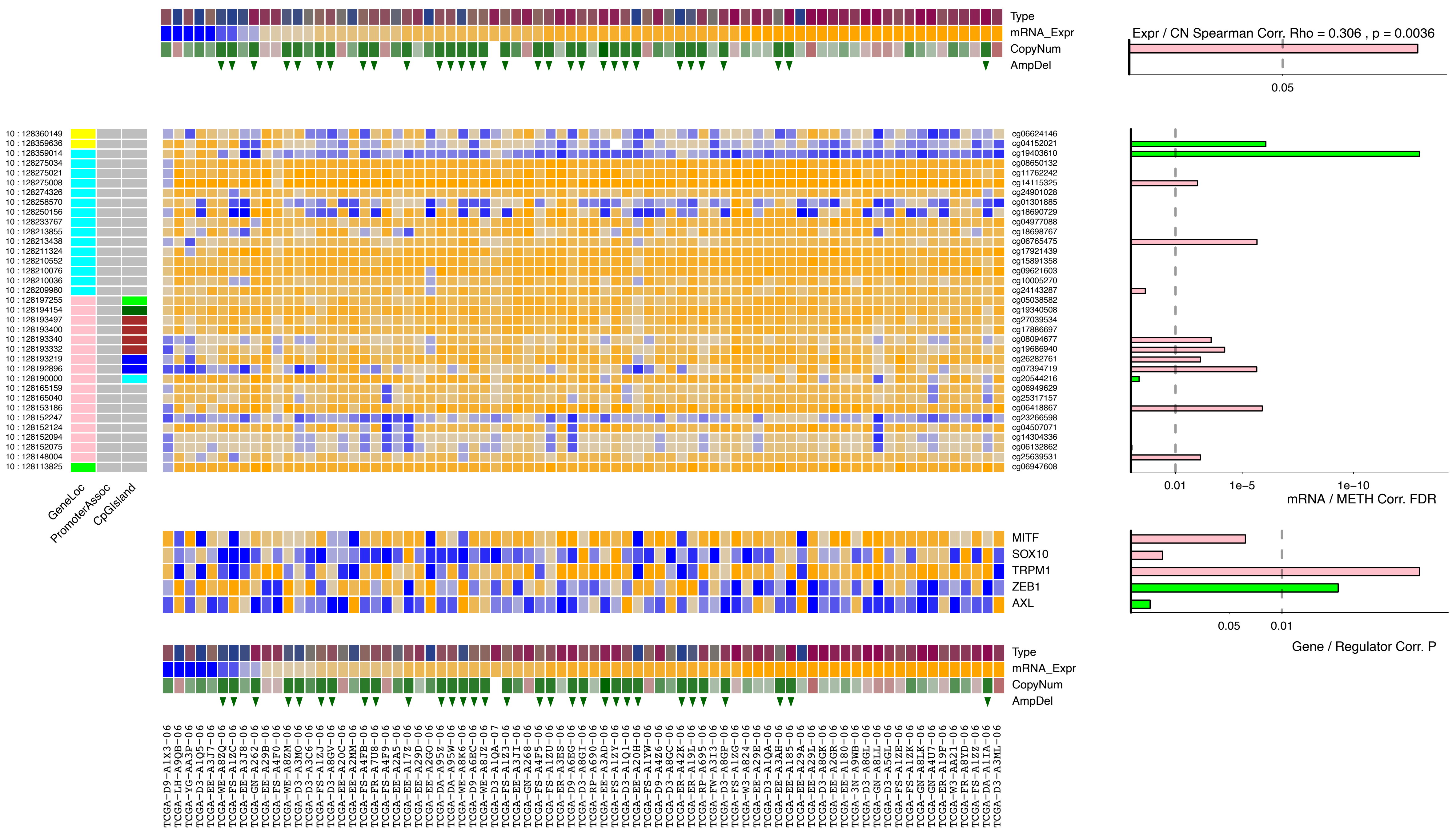

Type
mRNA_Expr
CopyNum
AmpDel
Expr / CN Spearman Corr. Rho = 0.306 , p = 0.0036
0.05
10 : 128360149
10 : 128359636
10 : 128359014
10 : 128275034
10 : 128275021
10 : 128275008
10 : 128274326
10 : 128258570
10 : 128250156
10 : 128233767
10 : 128213855
10 : 128213438
10 : 128211324
10 : 128210552
10 : 128210076
10 : 128210036
10 : 128209980
10 : 128197255
10 : 128194154
10 : 128193497
10 : 128193400
10 : 128193340
10 : 128193332
10 : 128193219
10 : 128192896
10 : 128190000
10 : 128165159
10 : 128165040
10 : 128153186
10 : 128152247
10 : 128152124
10 : 128152094
10 : 128152075
10 : 128148004
10 : 128113825
cg06624146
cg04152021
cg19403610
cg08650132
cg11762242
cg14115325
cg24901028
cg01301885
cg18690729
cg04977088
cg18698767
cg06765475
cg17921439
cg15891358
cg09621603
cg10005270
cg24143287
cg05038582
cg19340508
cg27039534
cg17886697
cg08094677
cg19686940
cg26282761
cg07394719
cg20544216
cg06949629
cg25317157
cg06418867
cg23266598
cg04507071
cg14304336
cg06132862
cg25639531
cg06947608
GeneLoc
PromoterAssoc
CpGIsland
0.01
1e−5
1e−10
mRNA / METH Corr. FDR
MITF
SOX10
TRPM1
ZEB1
AXL
0.05
0.01
Gene / Regulator Corr. P
Type
mRNA_Expr
CopyNum
AmpDel

ZNF704

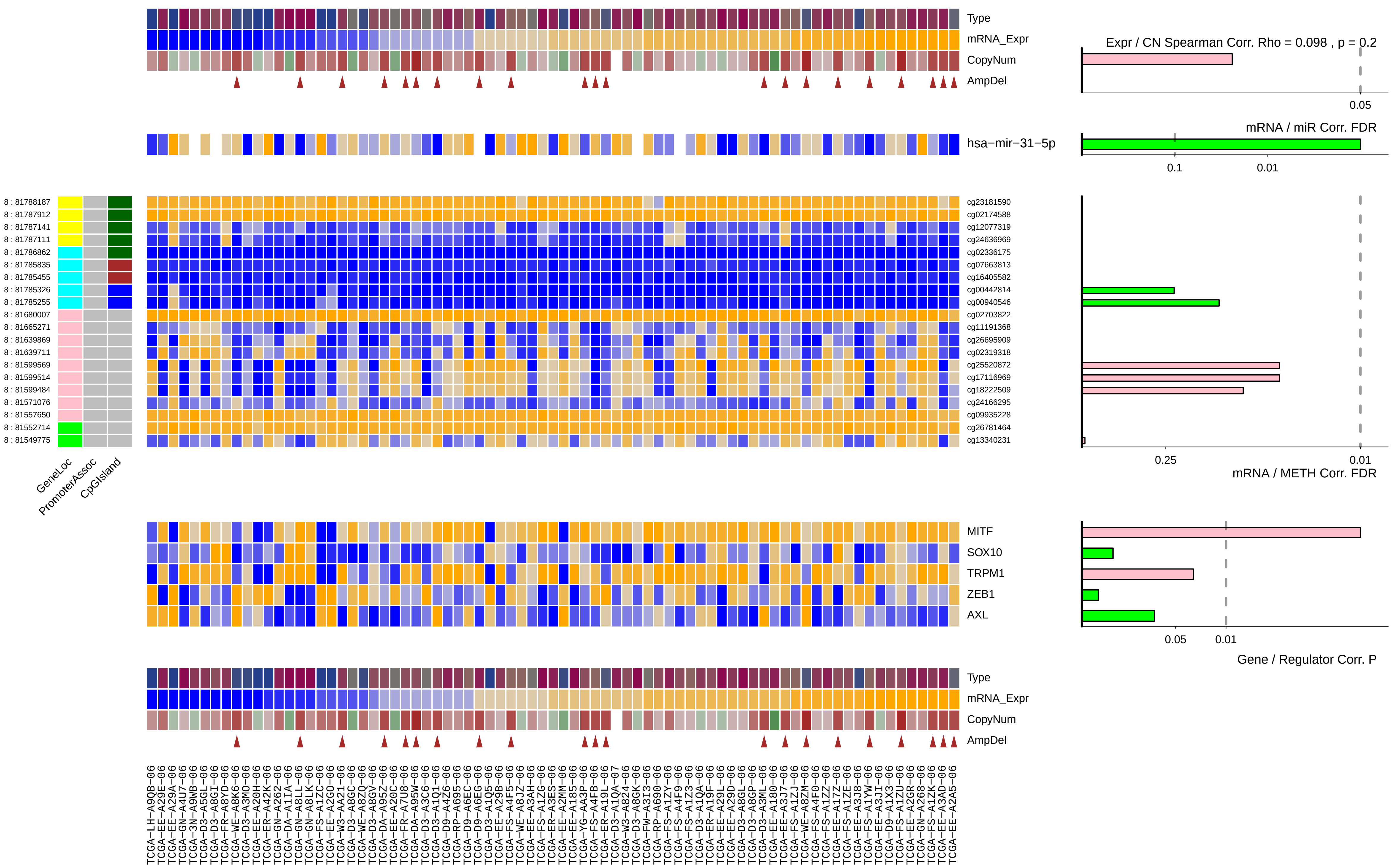
Type
mRNA_Expr
CopyNum
AmpDel
Expr / CN Spearman Corr. Rho = 0.098 , p = 0.2
0.05
hsa−mir−31−5p
mRNA / miR Corr. FDR
0.1
0.01
8 : 81788187
8 : 81787912
8 : 81787141
8 : 81787111
8 : 81786862
8 : 81785835
8 : 81785455
8 : 81785326
8 : 81785255
8 : 81680007
8 : 81665271
8 : 81639869
8 : 81639711
8 : 81599569
8 : 81599514
8 : 81599484
8 : 81571076
8 : 81557650
8 : 81552714
8 : 81549775
cg23181590
cg02174588
cg12077319
cg24636969
cg02336175
cg07663813
cg16405582
cg00442814
cg00940546
cg02703822
cg11191368
cg26695909
cg02319318
cg25520872
cg17116969
cg18222509
cg24166295
cg09935228
cg26781464
cg13340231
GeneLoc
PromoterAssoc
CpGIsland
0.25
0.01
mRNA / METH Corr. FDR
MITF
SOX10
TRPM1
ZEB1
AXL
0.05
0.01
Gene / Regulator Corr. P
Type
mRNA_Expr
CopyNum
AmpDel

CABLES1

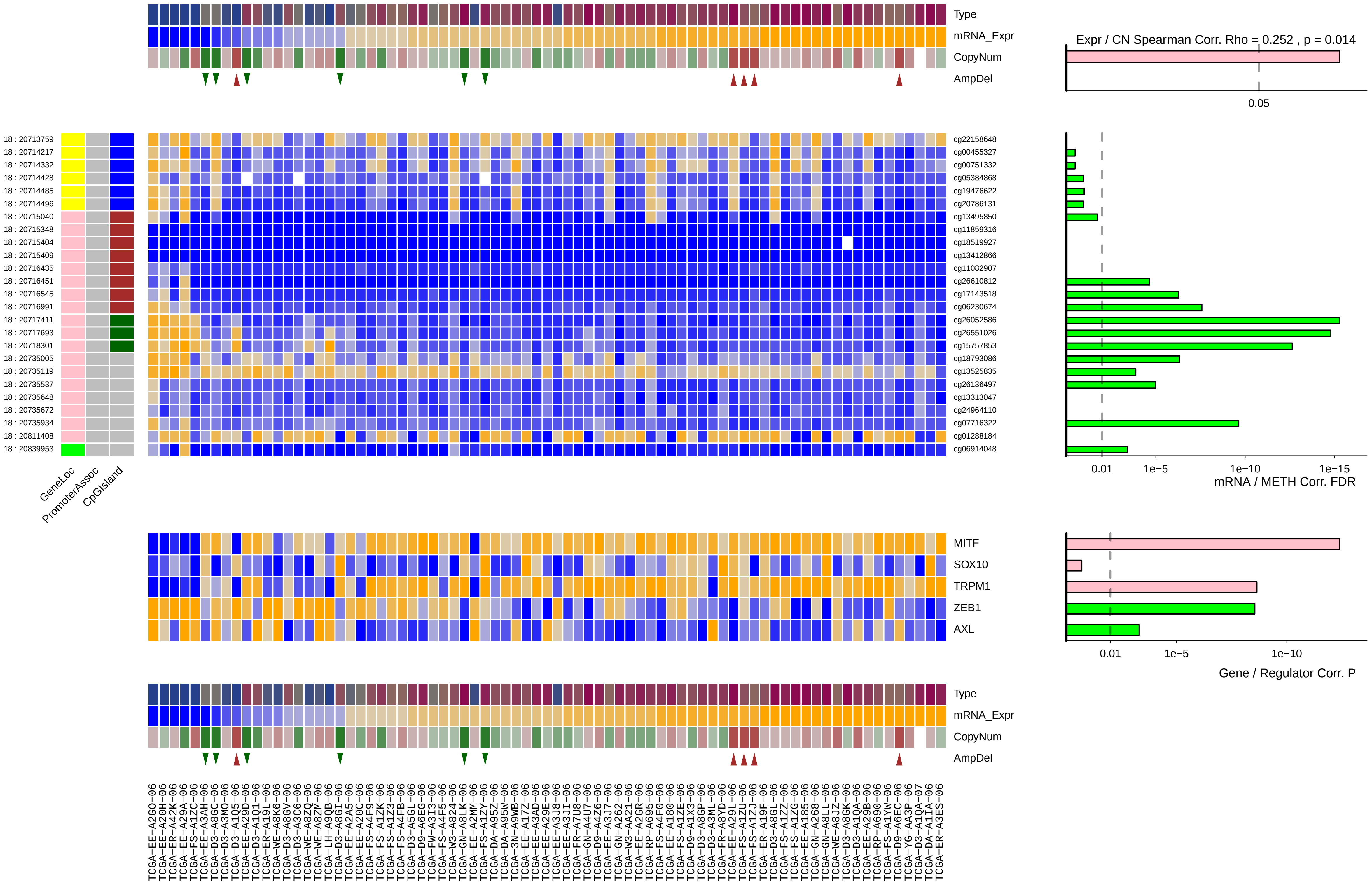

Type
mRNA_Expr
CopyNum
AmpDel
Expr / CN Spearman Corr. Rho = 0.252 , p = 0.014
0.05
GeneLoc
PromoterAssoc
CpGIsland
cg22158648
cg00455327
cg00751332
cg05384868
cg19476622
cg20786131
cg13495850
cg11859316
cg18519927
cg13412866
cg11082907
cg26610812
cg17143518
cg06230674
cg26052586
cg26551026
cg15757853
cg18793086
cg13525835
cg26136497
cg13313047
cg24964110
cg07716322
cg01288184
cg06914048
0.01
1e−5
1e−10
1e−15
mRNA / METH Corr. FDR
MITF
SOX10
TRPM1
ZEB1
AXL
Gene / Regulator Corr. P

TRPM1

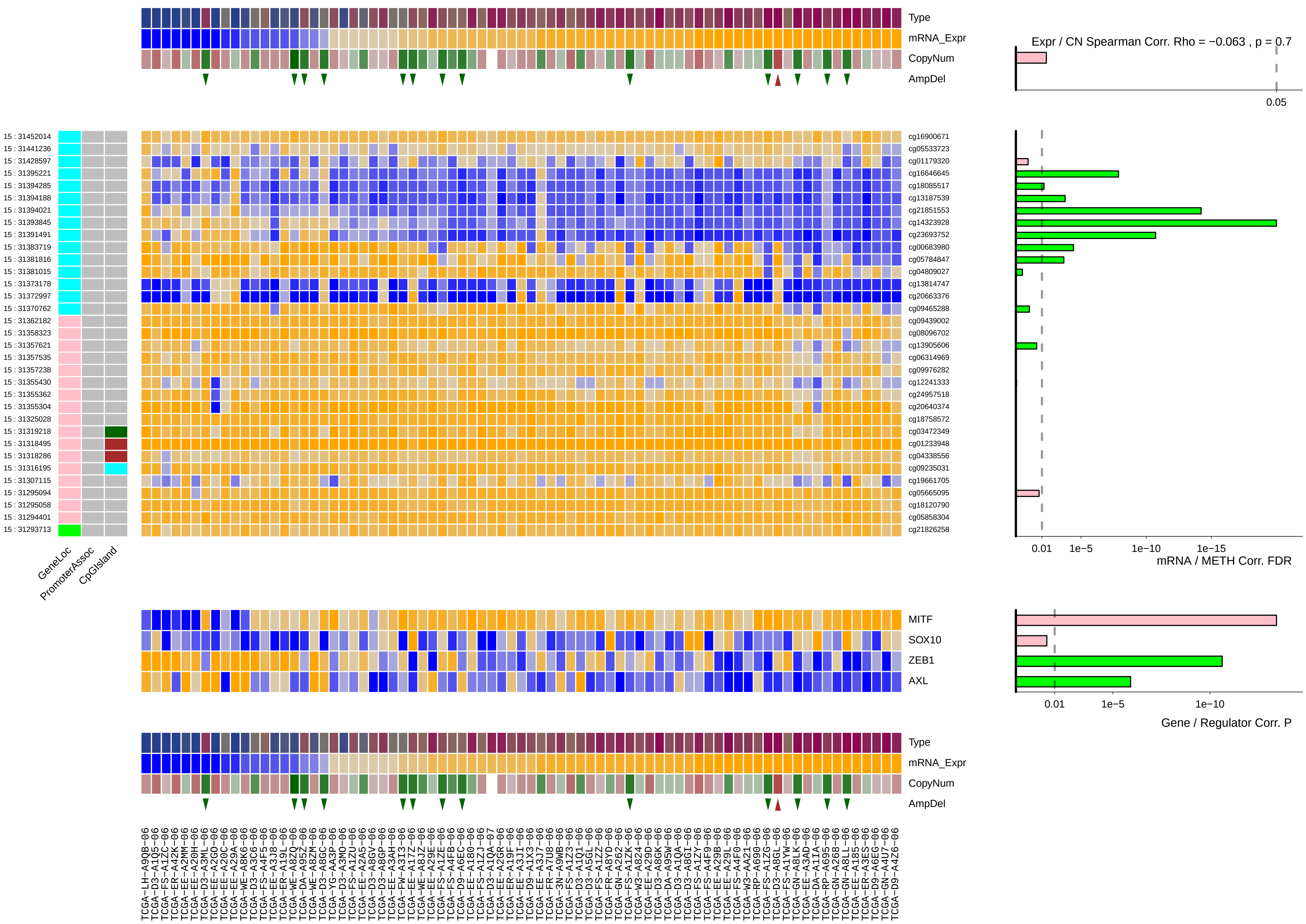

Type
mRNA_Expr
CopyNum
AmpDel
Expr / CN Spearman Corr. Rho = −0.063 , p = 0.7
0.05
15 : 31452014
15 : 31441236
15 : 31428597
15 : 31395221
15 : 31394285
15 : 31394188
15 : 31394021
15 : 31393845
15 : 31391491
15 : 31383719
15 : 31381816
15 : 31381015
15 : 31373178
15 : 31372997
15 : 31370762
15 : 31362182
15 : 31358323
15 : 31357621
15 : 31357535
15 : 31357238
15 : 31355430
15 : 31355362
15 : 31355304
15 : 31325028
15 : 31319218
15 : 31318495
15 : 31318286
15 : 31316195
15 : 31307115
15 : 31295094
15 : 31295058
15 : 31294401
15 : 31293713
cg16900671
cg05533723
cg01179320
cg16646645
cg18085517
cg13187539
cg21851553
cg14323928
cg23693752
cg00683980
cg05784847
cg04809027
cg13814747
cg20663376
cg09465288
cg09439002
cg08096702
cg13905606
cg06314969
cg09976282
cg12241333
cg24957518
cg20640374
cg18758572
cg03472349
cg01233948
cg04338556
cg09235031
cg19661705
cg05665095
cg18120790
cg05858304
cg21826258
GeneLoc
PromoterAssoc
CpGIsland
0.01
1e−5
1e−10
1e−15
mRNA / METH Corr. FDR
MITF
SOX10
ZEB1
AXL
0.01
1e−5
1e−10
Gene / Regulator Corr. P
Type
mRNA_Expr
CopyNum
AmpDel

WDR91

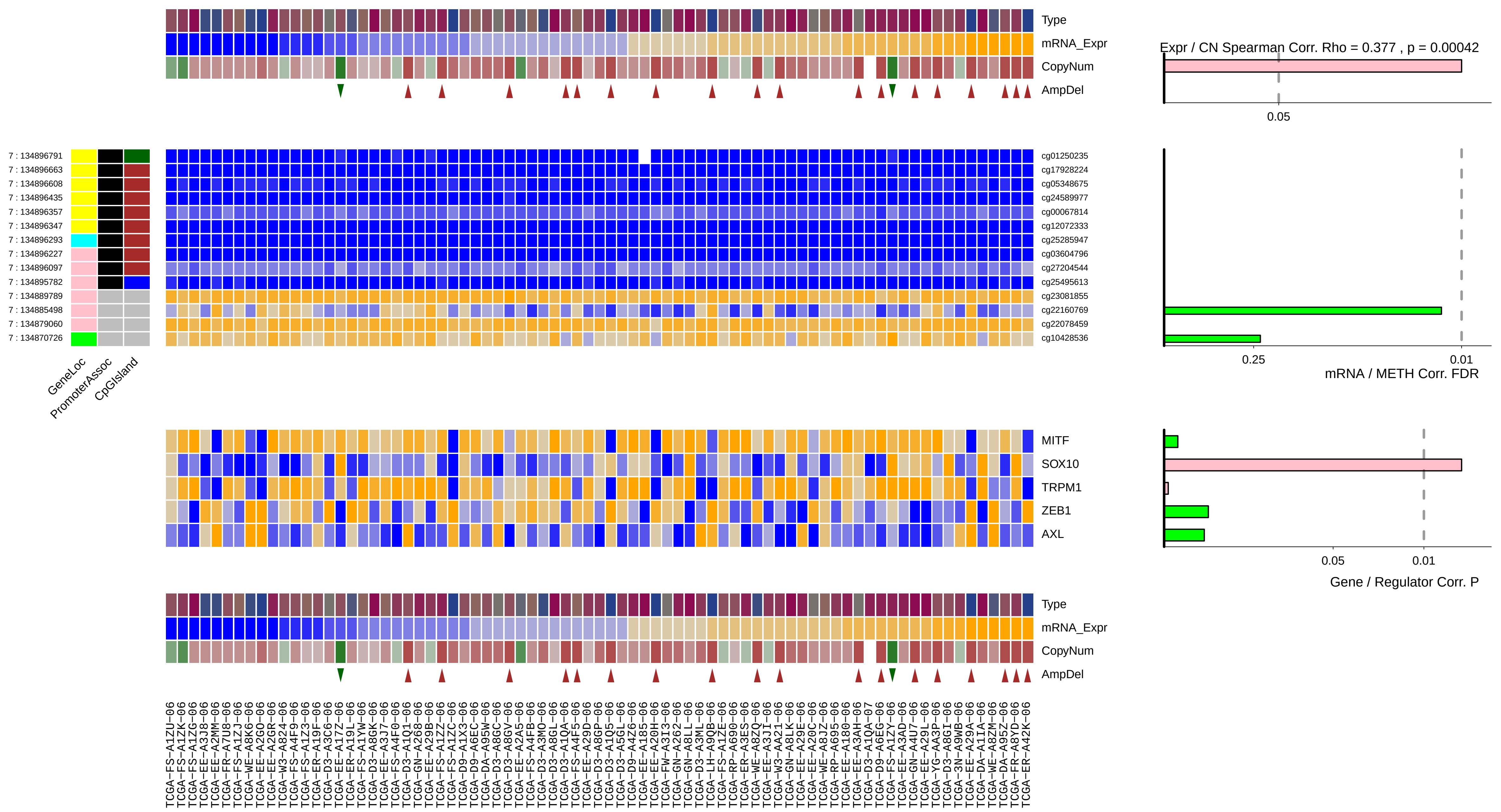

Type
mRNA_Expr
CopyNum
AmpDel
Expr / CN Spearman Corr. Rho = 0.377 , p = 0.00042
0.05
7 : 134896791
7 : 134896663
7 : 134896608
7 : 134896435
7 : 134896357
7 : 134896347
7 : 134896293
7 : 134896227
7 : 134896097
7 : 134895782
7 : 134889789
7 : 134885498
7 : 134879060
7 : 134870726
cg01250235
cg17928224
cg05348675
cg24589977
cg00067814
cg12072333
cg25285947
cg03604796
cg27204544
cg25495613
cg23081855
cg22160769
cg22078459
cg10428536
0.25
0.01
mRNA / METH Corr. FDR
GeneLoc
PromoterAssoc
CpGIsland
MITF
SOX10
TRPM1
ZEB1
AXL
0.05
0.01
Gene / Regulator Corr. P
Type
mRNA_Expr
CopyNum
AmpDel

SLC1A4

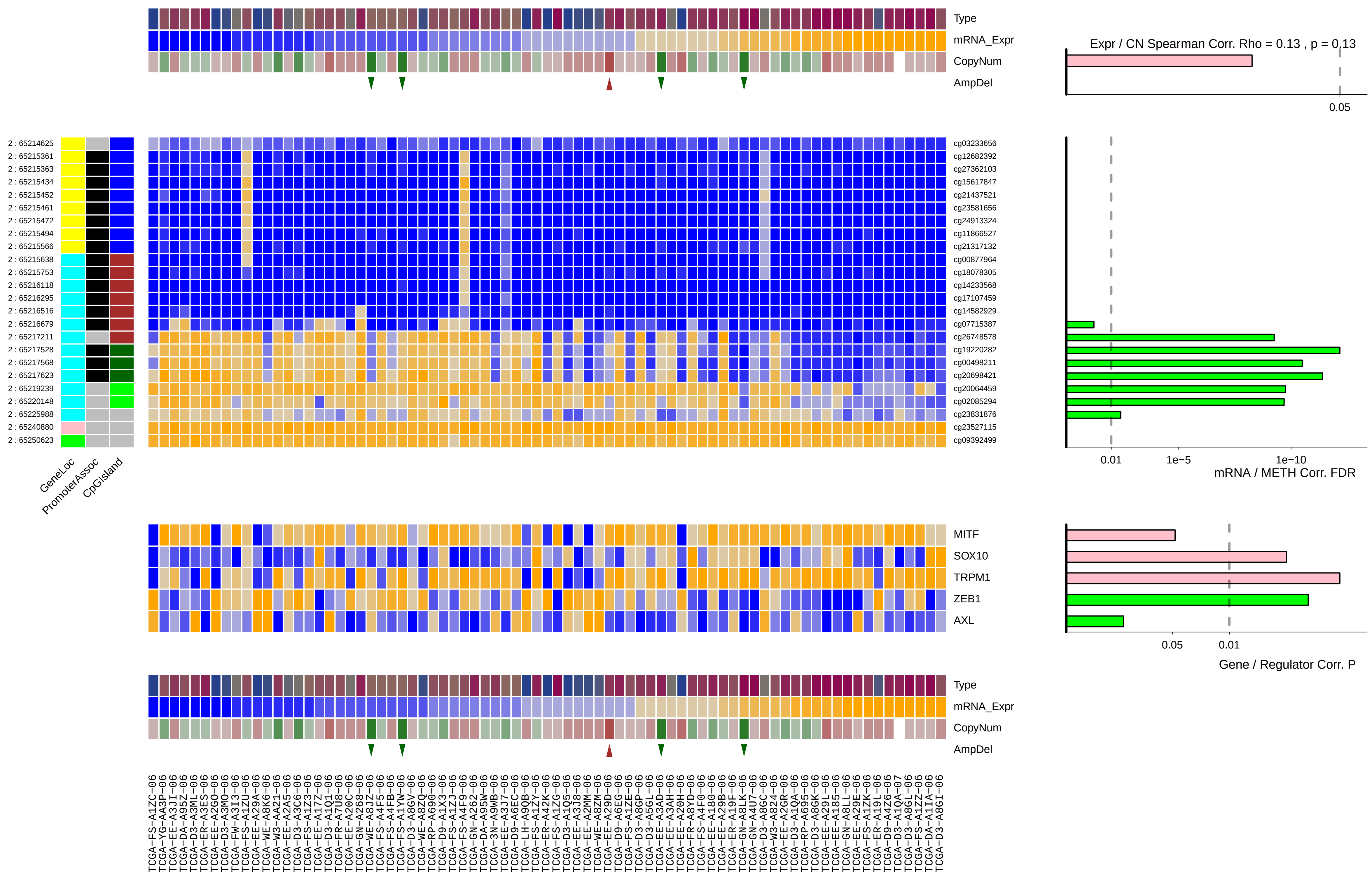

Type
mRNA_Expr
CopyNum
AmpDel
Expr / CN Spearman Corr. Rho = 0.13 , p = 0.13
0.05
2 : 65214625
2 : 65215361
2 : 65215363
2 : 65215434
2 : 65215452
2 : 65215461
2 : 65215472
2 : 65215494
2 : 65215566
2 : 65215638
2 : 65215753
2 : 65216118
2 : 65216295
2 : 65216516
2 : 65216679
2 : 65217211
2 : 65217528
2 : 65217568
2 : 65217623
2 : 65219239
2 : 65220148
2 : 65225988
2 : 65240880
2 : 65250623
cg03233656
cg12682392
cg27362103
cg15617847
cg21437521
cg23581656
cg24913324
cg11866527
cg21317132
cg00877964
cg18078305
cg14233568
cg17107459
cg14582929
cg07715387
cg26748578
cg19220282
cg00498211
cg20698421
cg20064459
cg02085294
cg23831876
cg23527115
cg09392499
GeneLoc
PromoterAssoc
CpGIsland
0.01
1e−5
1e−10
mRNA / METH Corr. FDR
MITF
SOX10
TRPM1
ZEB1
AXL
0.05
0.01
Gene / Regulator Corr. P
Type
mRNA_Expr
CopyNum
AmpDel

RAB27A

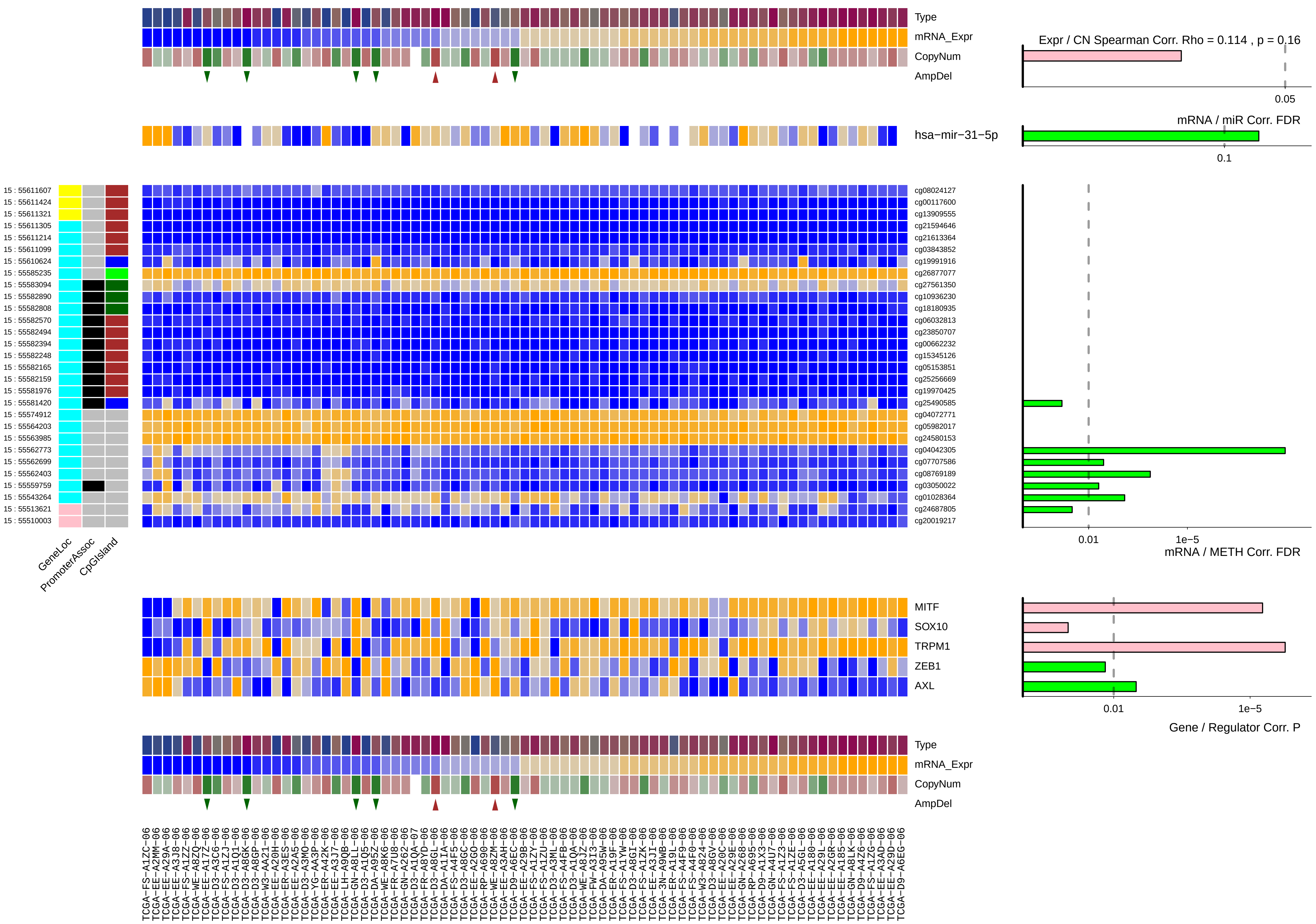

Type
mRNA_Expr
CopyNum
AmpDel
Expr / CN Spearman Corr. Rho = 0.114 , p = 0.16
0.05
hsa−mir−31−5p
mRNA / miR Corr. FDR
0.1
15 : 55611607
15 : 55611424
15 : 55611321
15 : 55611305
15 : 55611214
15 : 55611099
15 : 55610624
15 : 55585235
15 : 55583094
15 : 55582890
15 : 55582808
15 : 55582570
15 : 55582494
15 : 55582394
15 : 55582248
15 : 55582165
15 : 55582159
15 : 55581976
15 : 55581420
15 : 55574912
15 : 55564203
15 : 55563985
15 : 55562773
15 : 55562699
15 : 55562403
15 : 55559759
15 : 55543264
15 : 55513621
15 : 55510003
cg08024127
cg00117600
cg13909555
cg21594646
cg21613364
cg03843852
cg19991916
cg26877077
cg27561350
cg10936230
cg18180935
cg06032813
cg23850707
cg00662232
cg15345126
cg05153851
cg25256669
cg19970425
cg25490585
cg04072771
cg05982017
cg24580153
cg04042305
cg07707586
cg08769189
cg03050022
cg01028364
cg24687805
cg20019217
0.01
1e−5
mRNA / METH Corr. FDR
GeneLoc
PromoterAssoc
CpGIsland
MITF
SOX10
TRPM1
ZEB1
AXL
0.01
1e−5
Gene / Regulator Corr. P
Type
mRNA_Expr
CopyNum
AmpDel

GJB1

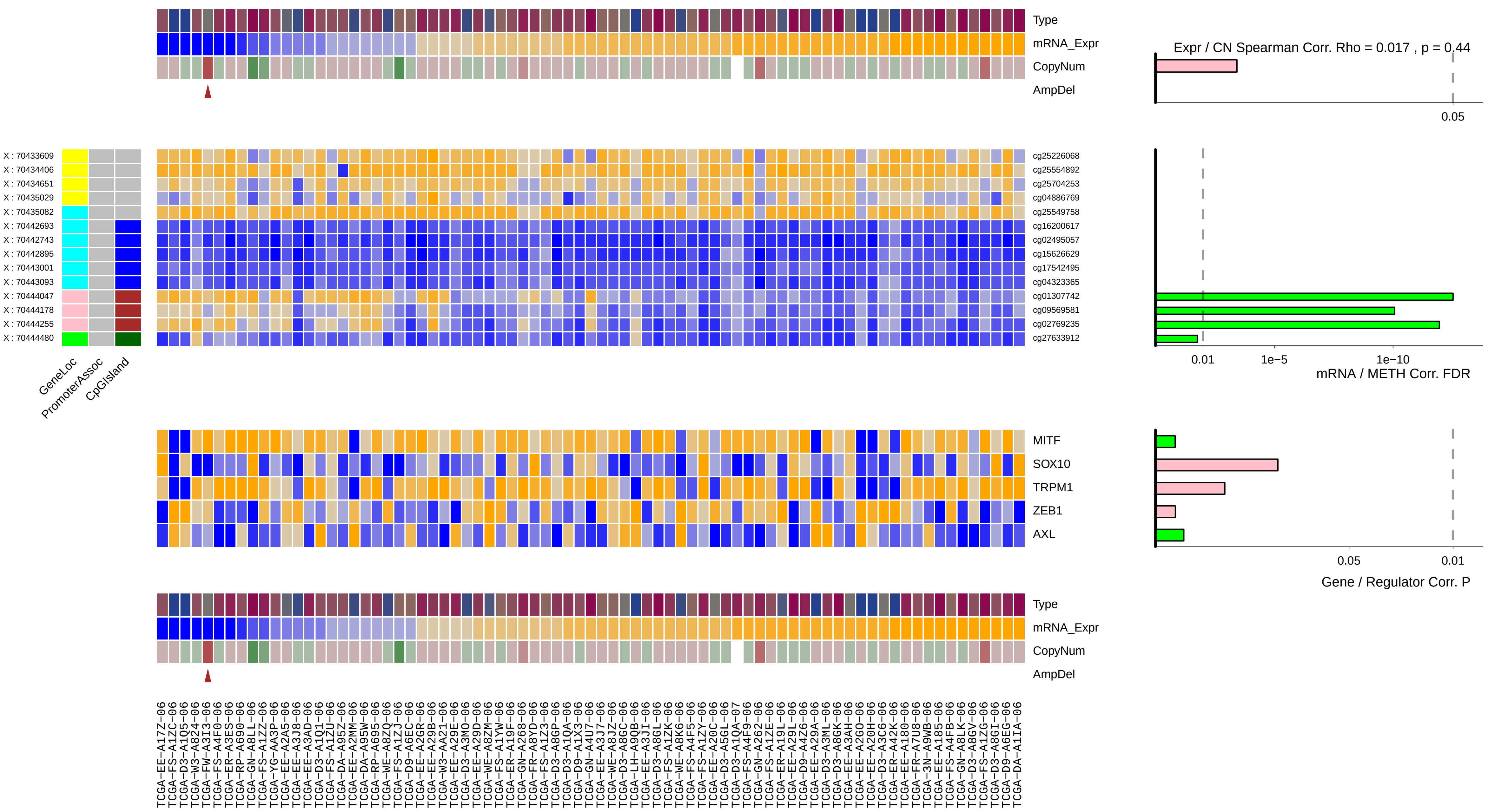
Type
mRNA_Expr
CopyNum
AmpDel
Expr / CN Spearman Corr. Rho = 0.017 , p = 0.44
0.05
X : 70433609
X : 70434406
X : 70434651
X : 70435029
X : 70435082
X : 70442693
X : 70442743
X : 70442895
X : 70443001
X : 70443093
X : 70444047
X : 70444178
X : 70444255
X : 70444480
GeneLoc
PromoterAssoc
CpGIsland
cg25226068
cg25554892
cg25704253
cg04886769
cg25549758
cg16200617
cg02495057
cg15626629
cg17542495
cg04323365
cg01307742
cg09569581
cg02769235
cg27633912
0.01
1e−5
1e−10
mRNA / METH Corr. FDR
MITF
SOX10
TRPM1
ZEB1
AXL
0.05
0.01
Gene / Regulator Corr. P
Type
mRNA_Expr
CopyNum
AmpDel

IGSF8

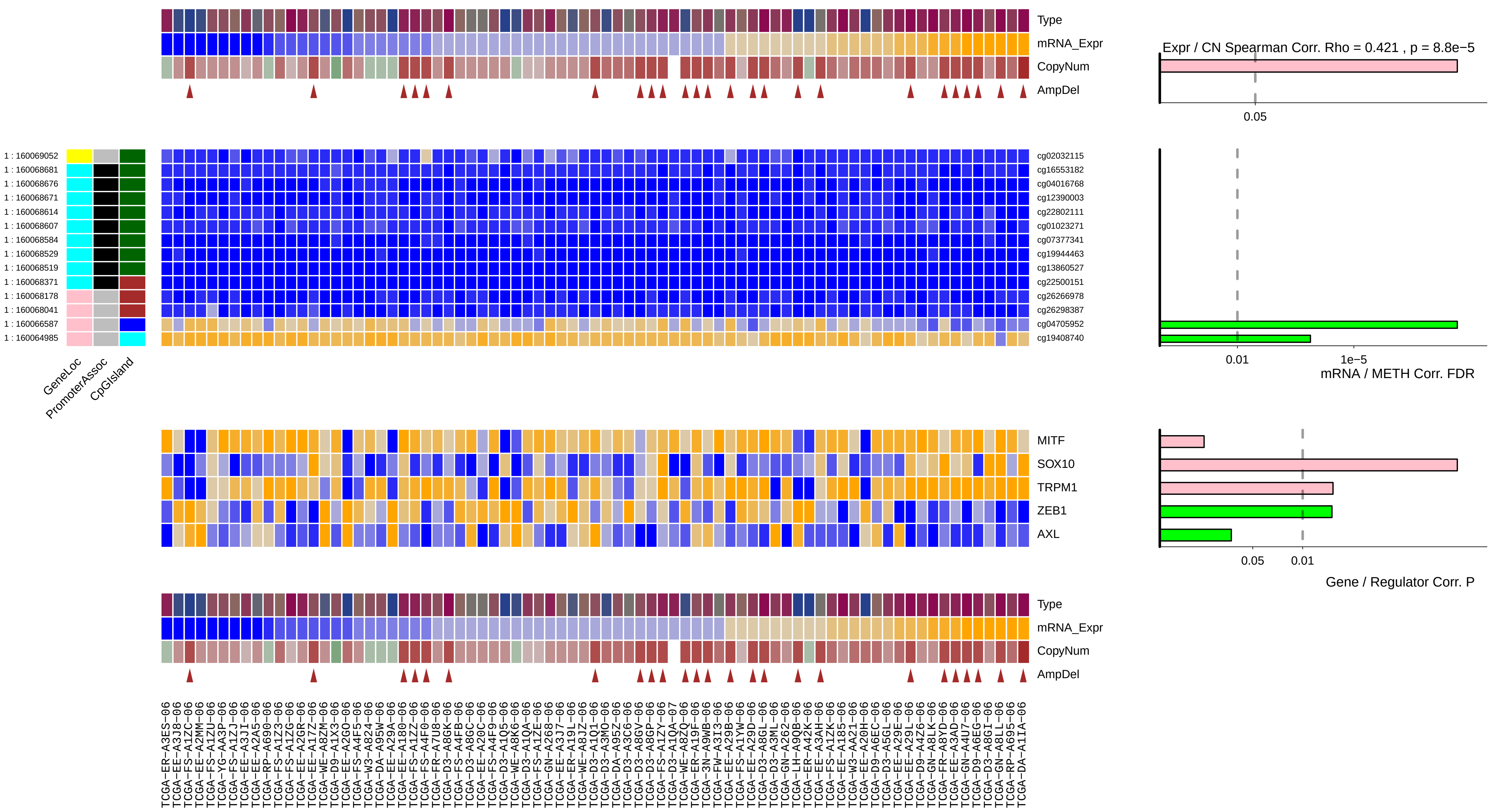
Type
mRNA_Expr
CopyNum
AmpDel
Expr / CN Spearman Corr. Rho = 0.421 , p = 8.8e−5
0.05
1 : 160069052
1 : 160068681
1 : 160068676
1 : 160068671
1 : 160068614
1 : 160068607
1 : 160068584
1 : 160068529
1 : 160068519
1 : 160068371
1 : 160068178
1 : 160068041
1 : 160066587
1 : 160064985
GeneLoc
PromoterAssoc
CpGIsland
cg02032115
cg16553182
cg04016768
cg12390003
cg22802111
cg01023271
cg07377341
cg19944463
cg13860527
cg22500151
cg26266978
cg26298387
cg04705952
cg19408740
0.01
1e−5
mRNA / METH Corr. FDR
MITF
SOX10
TRPM1
ZEB1
AXL
0.05
0.01
Gene / Regulator Corr. P
Type
mRNA_Expr
CopyNum
AmpDel

GPR137B

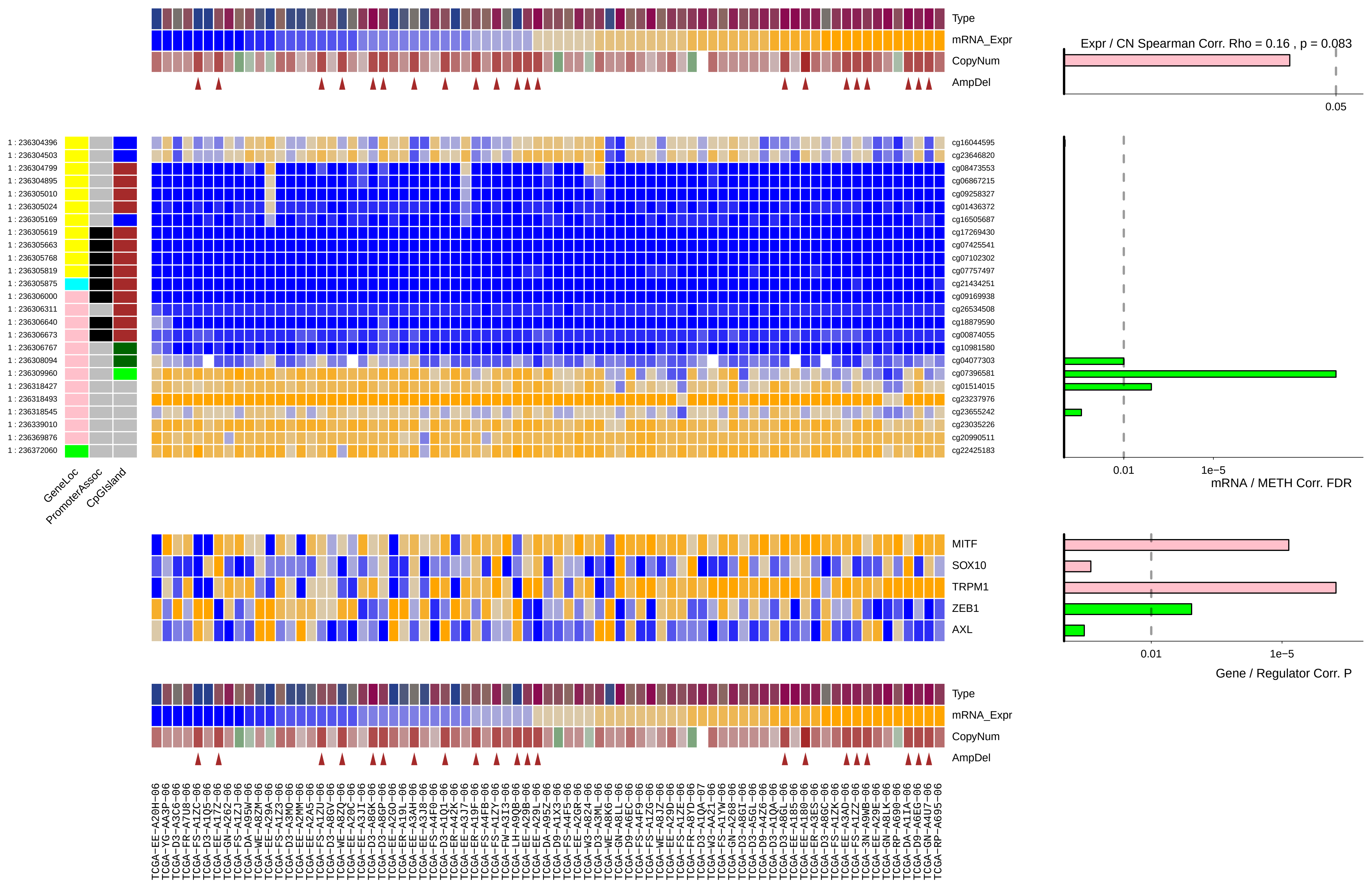
Type
mRNA_Expr
CopyNum
AmpDel
Expr / CN Spearman Corr. Rho = 0.16 , p = 0.083
0.05
1 : 236304396
1 : 236304503
1 : 236304799
1 : 236304895
1 : 236305010
1 : 236305024
1 : 236305169
1 : 236305619
1 : 236305663
1 : 236305768
1 : 236305819
1 : 236305875
1 : 236306000
1 : 236306311
1 : 236306640
1 : 236306673
1 : 236306767
1 : 236308094
1 : 236309960
1 : 236318427
1 : 236318493
1 : 236318545
1 : 236339010
1 : 236369876
1 : 236372060
cg16044595
cg23646820
cg08473553
cg06867215
cg09258327
cg01436372
cg16505687
cg17269430
cg07425541
cg07102302
cg07757497
cg21434251
cg09169938
cg26534508
cg18879590
cg00874055
cg10981580
cg04077303
cg07396581
cg01514015
cg23237976
cg23655242
cg23035226
cg20990511
cg22425183
0.01
1e−5
mRNA / METH Corr. FDR
GeneLoc
PromoterAssoc
CpGIsland
MITF
SOX10
TRPM1
ZEB1
AXL
0.01
1e−5
Gene / Regulator Corr. P
Type
mRNA_Expr
CopyNum
AmpDel
TCGA-EE-A20H-06
TCGA-YG-AA3P-06
TCGA-D3-A3CF-06
TCGA-FR-A7U8-06
TCGA-FS-A1ZC-06
TCGA-D3-A1Q5-06
TCGA-EE-A17Z-06
TCGA-GN-A262-06
TCGA-FS-A1ZJ-06
TCGA-DA-A95W-06
TCGA-WE-A8ZM-06
TCGA-EE-A29A-06
TCGA-FS-A1Z3-06
TCGA-D3-A3MO-06
TCGA-EE-A2MM-06
TCGA-EE-A2A5-06
TCGA-FS-A1ZU-06
TCGA-D3-A8GV-06
TCGA-WE-A8ZQ-06
TCGA-EE-A20C-06
TCGA-EE-A3JI-06
TCGA-D3-A8GK-06
TCGA-D3-A8GP-06
TCGA-EE-A2GO-06
TCGA-ER-A19L-06
TCGA-EE-A3AH-06
TCGA-EE-A3J8-06
TCGA-FS-A4F0-06
TCGA-D3-A1Q1-06
TCGA-ER-A42K-06
TCGA-EE-A3J7-06
TCGA-ER-A19F-06
TCGA-FS-A4FB-06
TCGA-FS-A1ZY-06
TCGA-FW-A3I3-06
TCGA-LH-A9QB-06
TCGA-EE-A29L-06
TCGA-EE-A29B-06
TCGA-DA-A95Z-06
TCGA-D9-A1X3-06
TCGA-FS-A4F5-06
TCGA-EE-A2GR-06
TCGA-W3-A824-06
TCGA-D3-A3ML-06
TCGA-WE-A8K6-06
TCGA-GN-A8LL-06
TCGA-D9-A6EC-06
TCGA-FS-A4F8-06
TCGA-FS-A1ZG-06
TCGA-WE-A8JZ-06
TCGA-EE-A29D-06
TCGA-FS-A1ZE-06
TCGA-FR-A8YD-06
TCGA-D3-A1QA-07
TCGA-W3-AA21-06
TCGA-FS-A1YW-06
TCGA-GN-A268-06
TCGA-D3-A8GI-06
TCGA-D3-A5GL-06
TCGA-D9-A4Z6-06
TCGA-D3-A1QA-06
TCGA-D3-A8GL-06
TCGA-EE-A185-06
TCGA-EE-A180-06
TCGA-ER-A3ES-06
TCGA-D3-A8GC-06
TCGA-FS-A1ZK-06
TCGA-EE-A3AD-06
TCGA-FS-A1ZZ-06
TCGA-3N-A9WB-06
TCGA-EE-A29E-06
TCGA-GN-A8LK-06
TCGA-RP-A690-06
TCGA-DA-A1IA-06
TCGA-D9-A6EG-06
TCGA-GN-A4U7-06
TCGA-RP-A695-06

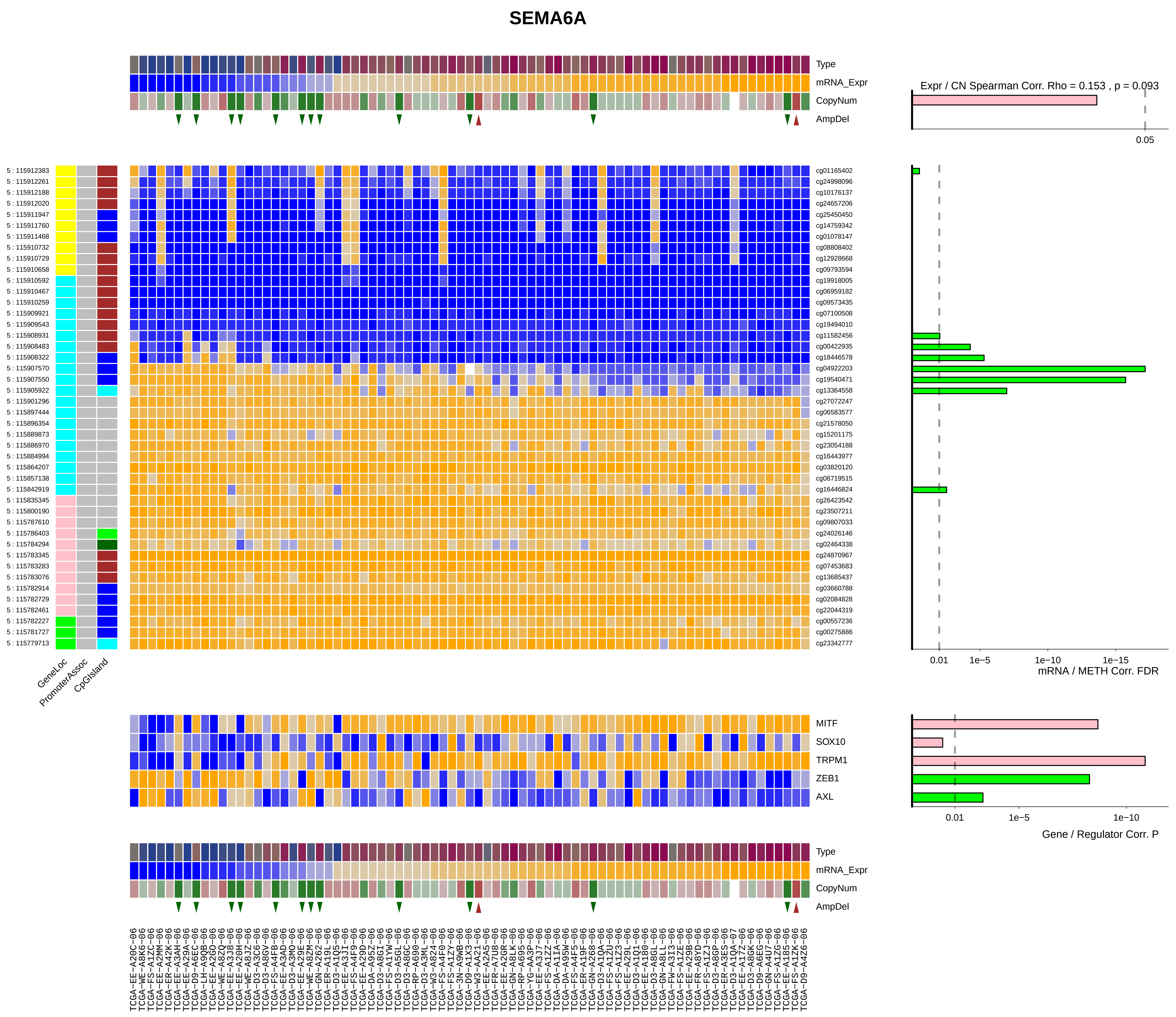
SEMA6A
Type
mRNA_Expr
CopyNum
AmpDel
Expr / CN Spearman Corr. Rho = 0.153 , p = 0.093
0.05
GeneLoc
PromoterAssoc
CpGIsland
0.01
1e−5
1e−10
1e−15
mRNA / METH Corr. FDR
MITF
SOX10
TRPM1
ZEB1
AXL
Gene / Regulator Corr. P

NR4A3

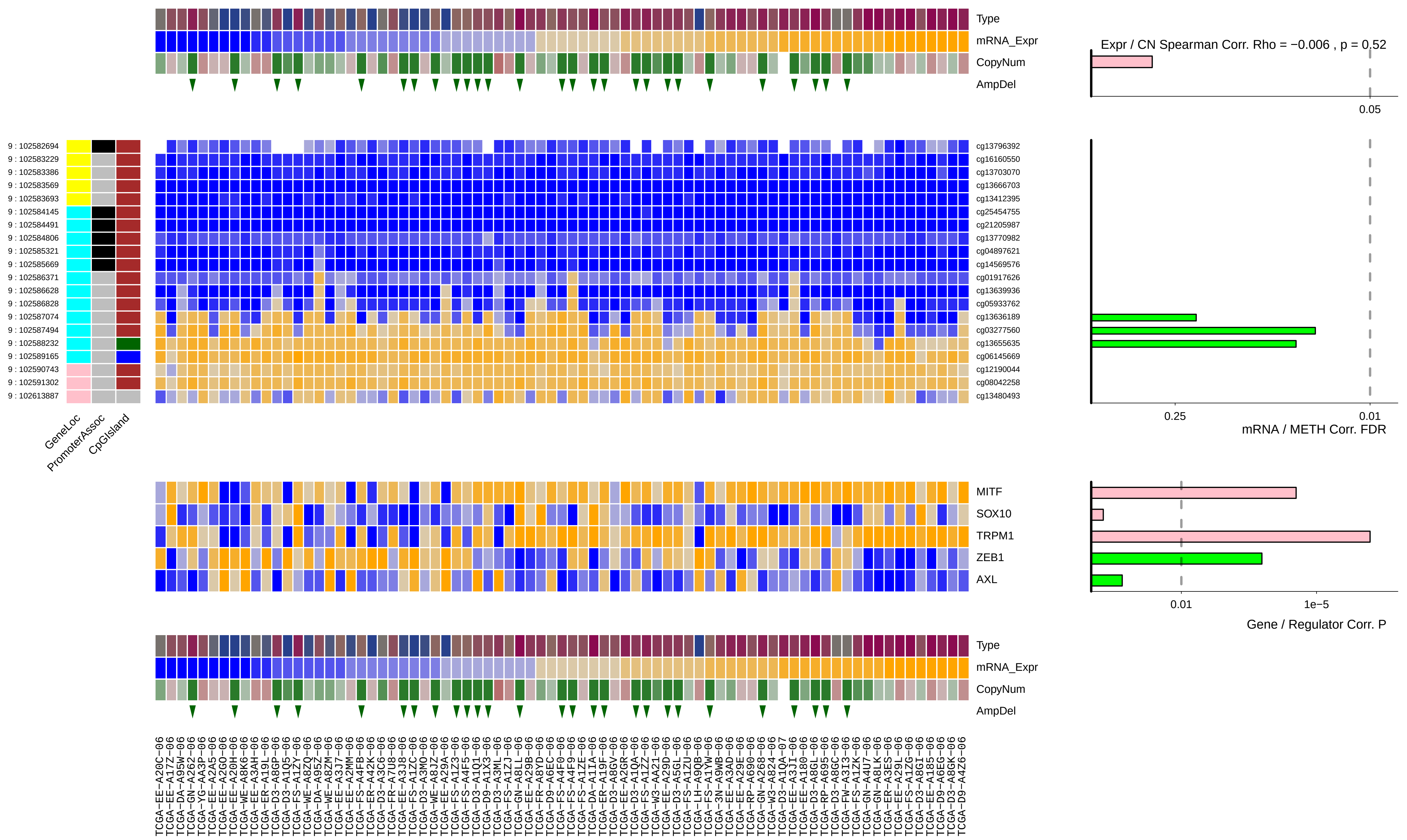

Type
mRNA_Expr
CopyNum
AmpDel
Expr / CN Spearman Corr. Rho = −0.006 , p = 0.52
0.05
9 : 102582694
9 : 102583229
9 : 102583386
9 : 102583569
9 : 102583693
9 : 102584145
9 : 102584491
9 : 102584806
9 : 102585321
9 : 102585669
9 : 102586371
9 : 102586628
9 : 102586828
9 : 102587074
9 : 102587494
9 : 102588232
9 : 102589165
9 : 102590743
9 : 102591302
9 : 102613887
GeneLoc
PromoterAssoc
CpGIsland
cg13796392
cg16160550
cg13703070
cg13666703
cg13412395
cg25454755
cg21205987
cg13770982
cg04897621
cg14569576
cg01917626
cg13639936
cg05933762
cg13636189
cg03277560
cg13655635
cg06145669
cg12190044
cg08042258
cg13480493
0.25
0.01
mRNA / METH Corr. FDR
MITF
SOX10
TRPM1
ZEB1
AXL
0.01
1e−5
Gene / Regulator Corr. P
Type
mRNA_Expr
CopyNum
AmpDel

DCT

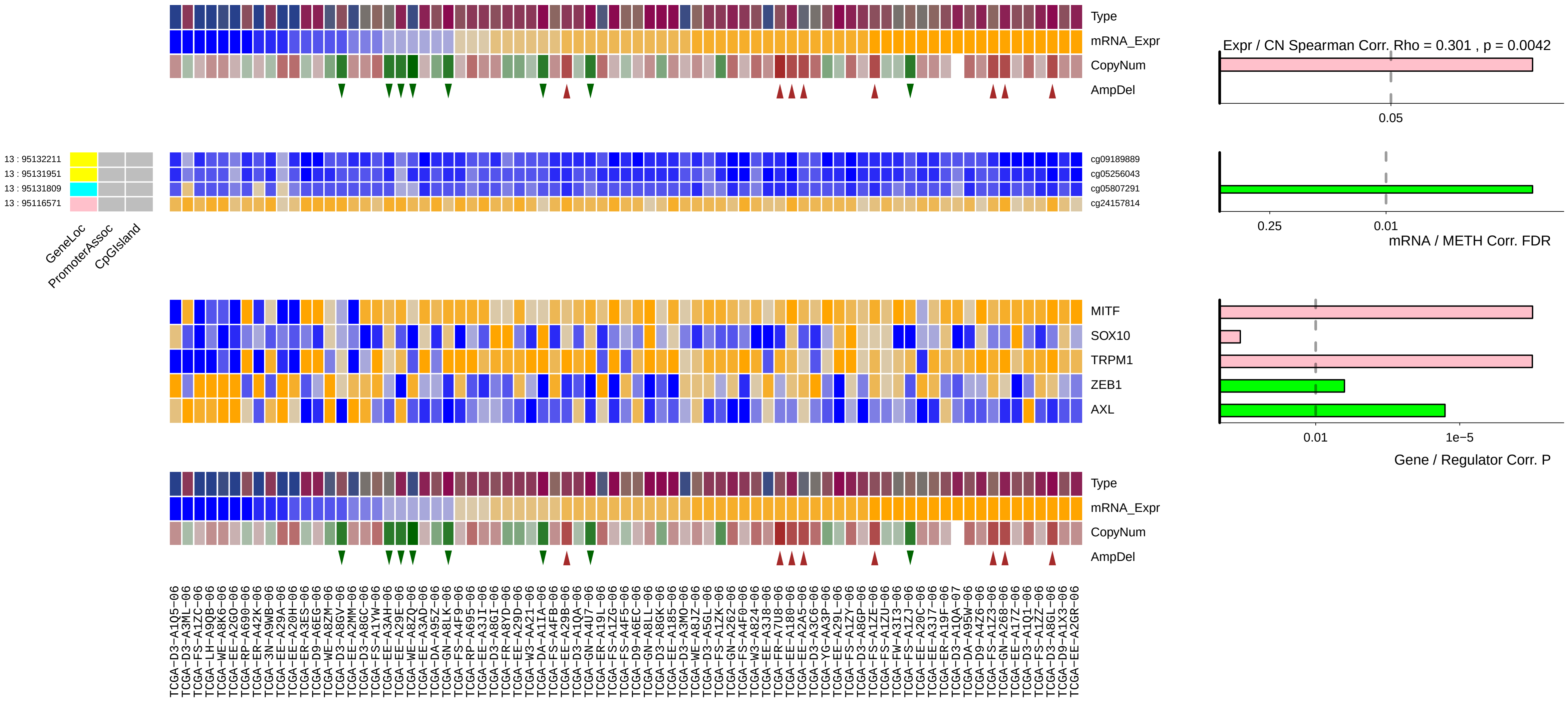
Type
mRNA_Expr
CopyNum
AmpDel
Expr / CN Spearman Corr. Rho = 0.301 , p = 0.0042
0.05
13 : 95132211
13 : 95131951
13 : 95131809
13 : 95116571
GeneLoc
PromoterAssoc
CpGIsland
cg09189889
cg05256043
cg05807291
cg24157814
0.25
0.01
mRNA / METH Corr. FDR
MITF
SOX10
TRPM1
ZEB1
AXL
0.01
1e−5
Gene / Regulator Corr. P
Type
mRNA_Expr
CopyNum
AmpDel

SLC45A2

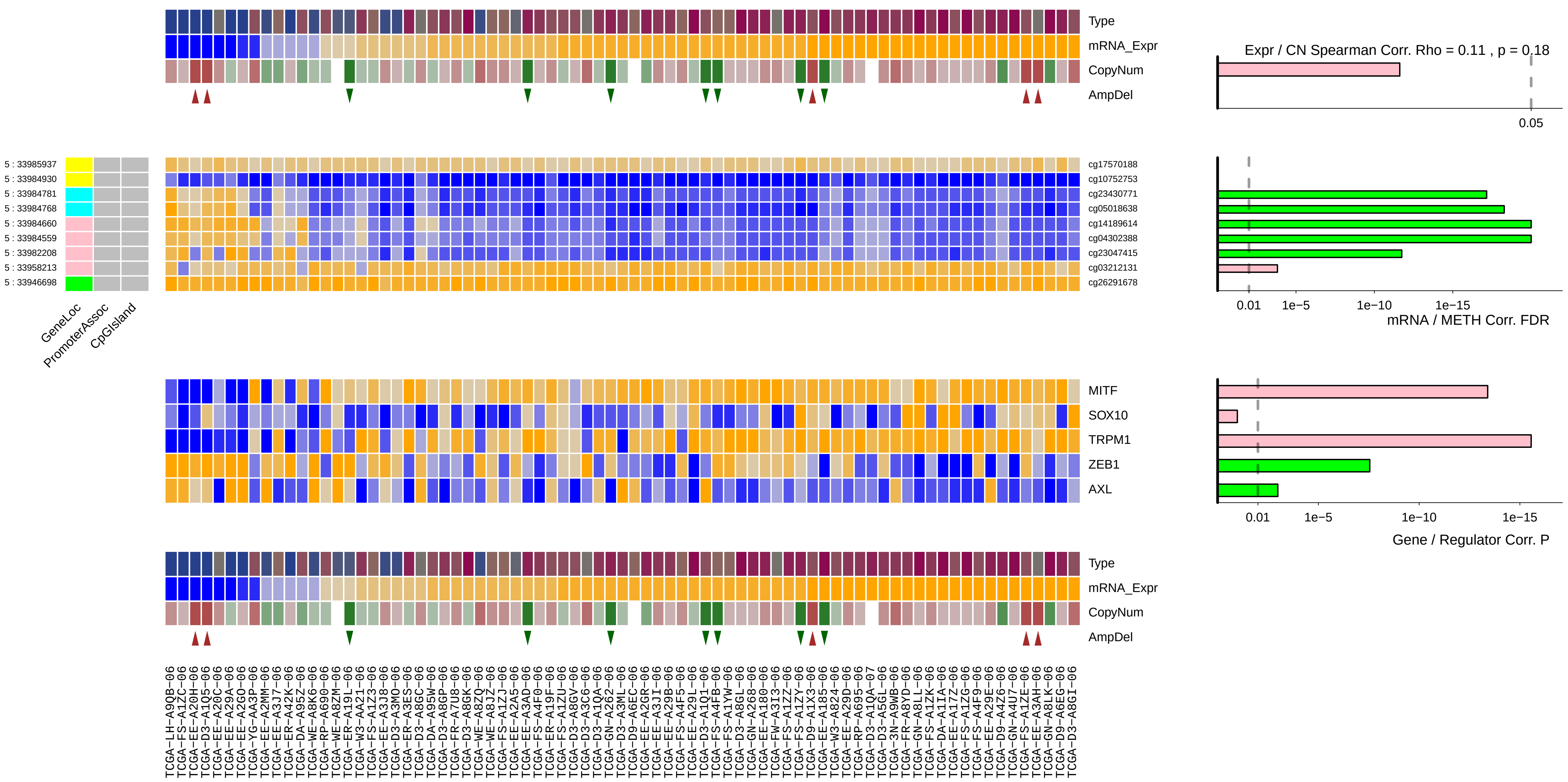
Type
mRNA_Expr
CopyNum
AmpDel
Expr / CN Spearman Corr. Rho = 0.11 , p = 0.18
0.05
5 : 33985937
5 : 33984930
5 : 33984781
5 : 33984768
5 : 33984660
5 : 33984559
5 : 33982208
5 : 33958213
5 : 33946698
GeneLoc
PromoterAssoc
CpGIsland
cg17570188
cg10752753
cg23430771
cg05018638
cg14189614
cg04302388
cg23047415
cg03212131
cg26291678
0.01
1e−5
1e−10
1e−15
mRNA / METH Corr. FDR
MITF
SOX10
TRPM1
ZEB1
AXL
0.01
1e−5
1e−10
1e−15
Gene / Regulator Corr. P
Type
mRNA_Expr
CopyNum
AmpDel

PLP1

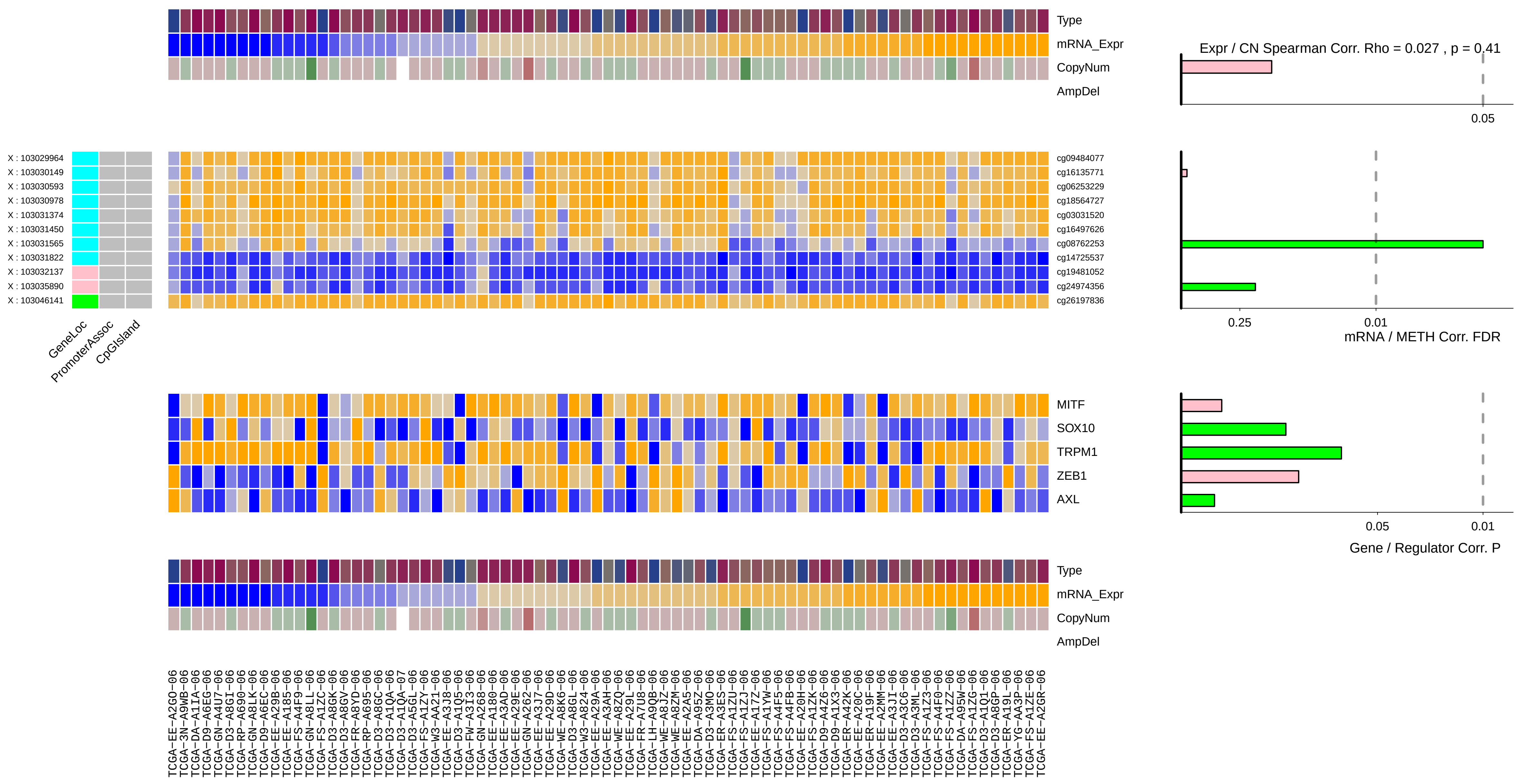
Type
mRNA_Expr
CopyNum
AmpDel
Expr / CN Spearman Corr. Rho = 0.027 , p = 0.41
0.05
X : 103029964
X : 103030149
X : 103030593
X : 103030978
X : 103031374
X : 103031450
X : 103031565
X : 103031822
X : 103032137
X : 103035890
X : 103046141
GeneLoc
PromoterAssoc
CpGIsland
cg09484077
cg16135771
cg06253229
cg18564727
cg03031520
cg16497626
cg08762253
cg14725537
cg19481052
cg24974356
cg26197836
0.25
0.01
mRNA / METH Corr. FDR
MITF
SOX10
TRPM1
ZEB1
AXL
0.05
0.01
Gene / Regulator Corr. P
Type
mRNA_Expr
CopyNum
AmpDel

PLEKHH1

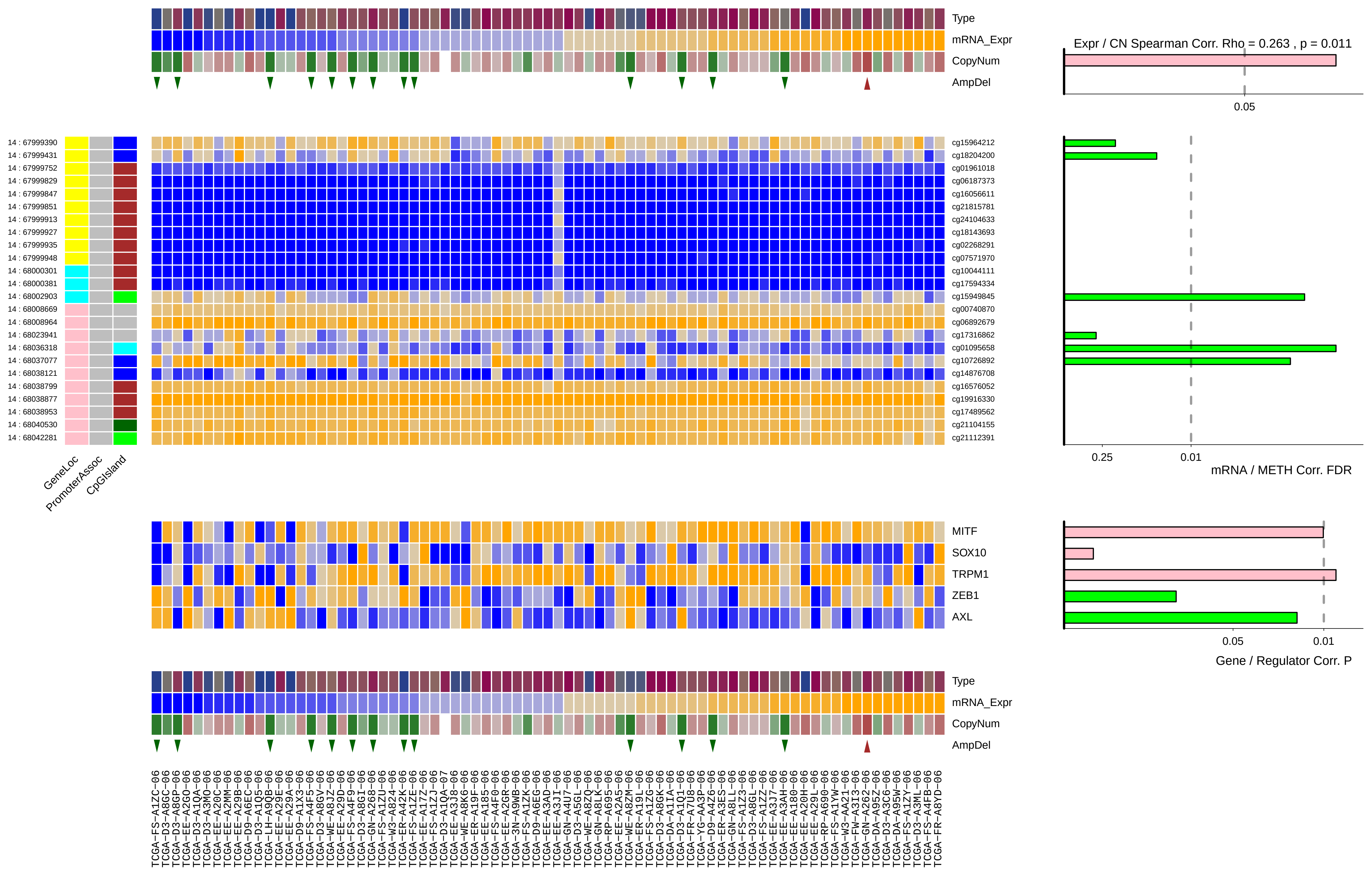
Type
mRNA_Expr
CopyNum
AmpDel
Expr / CN Spearman Corr. Rho = 0.263 , p = 0.011
0.05
14 : 67999390
14 : 67999431
14 : 67999752
14 : 67999829
14 : 67999847
14 : 67999851
14 : 67999913
14 : 67999927
14 : 67999935
14 : 67999948
14 : 68000301
14 : 68000381
14 : 68002903
14 : 68008669
14 : 68008964
14 : 68023941
14 : 68036318
14 : 68037077
14 : 68038121
14 : 68038799
14 : 68038877
14 : 68038953
14 : 68040530
14 : 68042281
cg15964212
cg18204200
cg01961018
cg06187373
cg16056611
cg21815781
cg24104633
cg18143693
cg02268291
cg07571970
cg10044111
cg17594334
cg15949845
cg00740870
cg06892679
cg17316862
cg01095658
cg10726892
cg14876708
cg16576052
cg19916330
cg17489562
cg21104155
cg21112391
GeneLoc
PromoterAssoc
CpGIsland
0.25
0.01
mRNA / METH Corr. FDR
MITF
SOX10
TRPM1
ZEB1
AXL
0.05
0.01
Gene / Regulator Corr. P
Type
mRNA_Expr
CopyNum
AmpDel
TCGA-FS-A1ZC-06
TCGA-D3-A8GC-06
TCGA-D3-A8GP-06
TCGA-EE-A2GO-06
TCGA-D3-A1QA-06
TCGA-D3-A3MO-06
TCGA-EE-A20C-06
TCGA-EE-A2MM-06
TCGA-EE-A29B-06
TCGA-D9-A6EC-06
TCGA-D3-A1Q5-06
TCGA-LH-A9QB-06
TCGA-EE-A29E-06
TCGA-EE-A29A-06
TCGA-D9-A1X3-06
TCGA-FS-A4F5-06
TCGA-D3-A8GV-06
TCGA-WE-A8JZ-06
TCGA-EE-A29D-06
TCGA-FS-A4F9-06
TCGA-D3-A8GI-06
TCGA-GN-A268-06
TCGA-FS-A1ZU-06
TCGA-W3-A824-06
TCGA-ER-A42K-06
TCGA-EE-A17Z-06
TCGA-FS-A1ZJ-06
TCGA-D3-A1QA-07
TCGA-EE-A3J8-06
TCGA-WE-A8K6-06
TCGA-ER-A19F-06
TCGA-EE-A185-06
TCGA-FS-A4F0-06
TCGA-EE-A2GR-06
TCGA-3N-A9WB-06
TCGA-FS-A1ZK-06
TCGA-D9-A6EG-06
TCGA-EE-A3AD-06
TCGA-EE-A3JI-06
TCGA-GN-A4U7-06
TCGA-D3-A5GL-06
TCGA-WE-A8ZQ-06
TCGA-GN-A8LK-06
TCGA-RP-A695-06
TCGA-EE-A2A5-06
TCGA-WE-A8ZM-06
TCGA-ER-A19T-06
TCGA-FS-A1ZG-06
TCGA-D3-A8GK-06
TCGA-DA-A1IA-06
TCGA-D3-A1Q1-06
TCGA-FR-A7U8-06
TCGA-YG-AA3P-06
TCGA-D9-A4Z6-06
TCGA-ER-A3ES-06
TCGA-GN-A8LL-06
TCGA-FS-A1Z3-06
TCGA-D3-A8GL-06
TCGA-FS-A1ZT-06
TCGA-EE-A3J7-06
TCGA-EE-A3AH-06
TCGA-EE-A180-06
TCGA-EE-A20H-06
TCGA-EE-A29L-06
TCGA-RP-A690-06
TCGA-FS-A1YW-06
TCGA-W3-AA21-06
TCGA-FW-A3I3-06
TCGA-GN-A262-06
TCGA-DA-A95Z-06
TCGA-D3-A3C6-06
TCGA-DA-A95W-06
TCGA-FS-A1ZY-06
TCGA-D3-A3ML-06
TCGA-FS-A4FB-06
TCGA-FR-A8YD-06

ASAH1

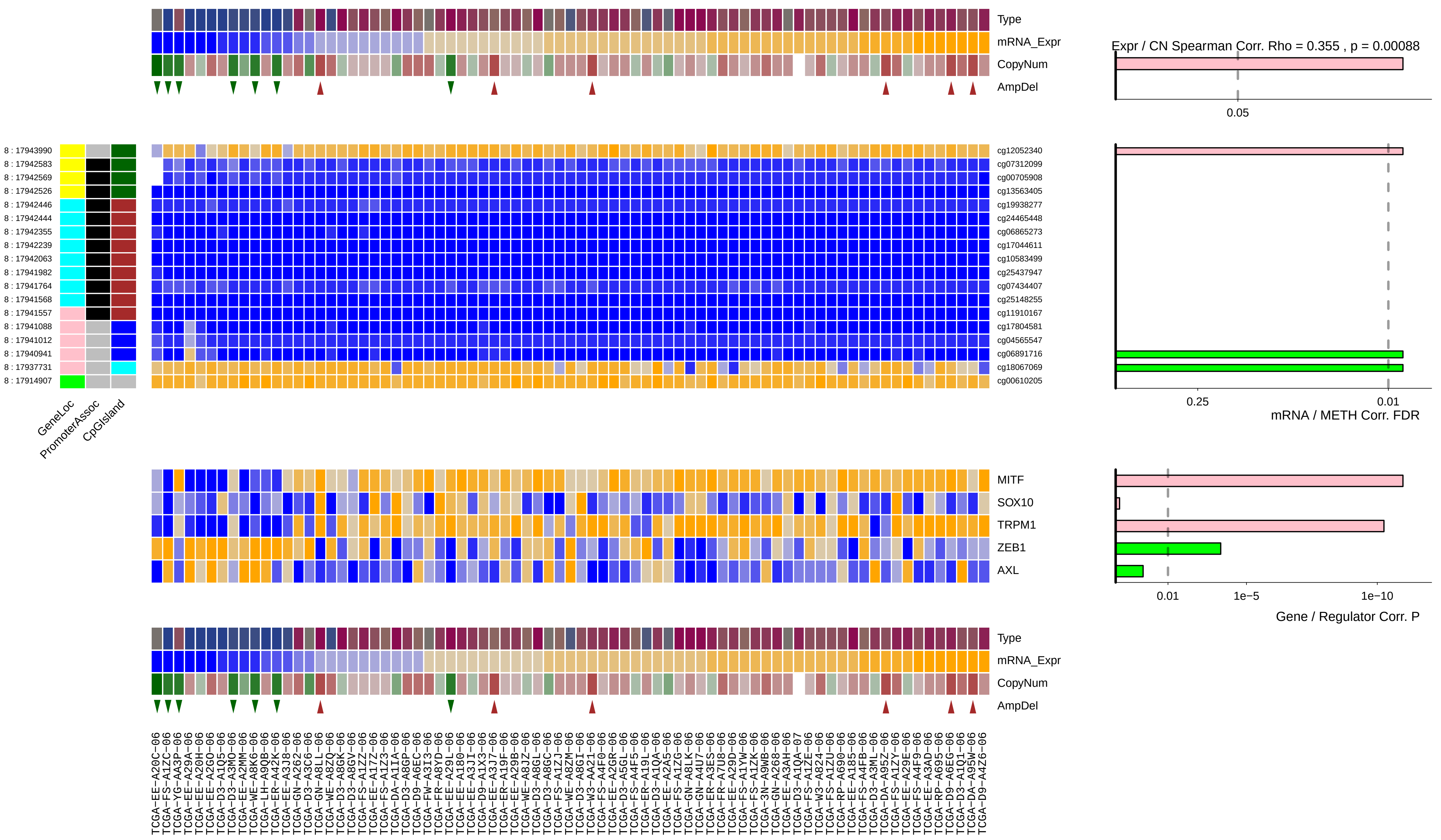

Type
mRNA_Expr
CopyNum
AmpDel
Expr / CN Spearman Corr. Rho = 0.355 , p = 0.00088
0.05
8 : 17943990
8 : 17942583
8 : 17942569
8 : 17942526
8 : 17942446
8 : 17942444
8 : 17942355
8 : 17942239
8 : 17942063
8 : 17941982
8 : 17941764
8 : 17941568
8 : 17941557
8 : 17941088
8 : 17941012
8 : 17940941
8 : 17937731
8 : 17914907
GeneLoc
PromoterAssoc
CpGIsland
cg12052340
cg07312099
cg00705908
cg13563405
cg19938277
cg24465448
cg06865273
cg17044611
cg10583499
cg25437947
cg07434407
cg25148255
cg11910167
cg17804581
cg04565547
cg06891716
cg18067069
cg00610205
0.25
0.01
mRNA / METH Corr. FDR
MITF
SOX10
TRPM1
ZEB1
AXL
0.01
1e−5
1e−10
Gene / Regulator Corr. P
Type
mRNA_Expr
CopyNum
AmpDel

SLC19A2

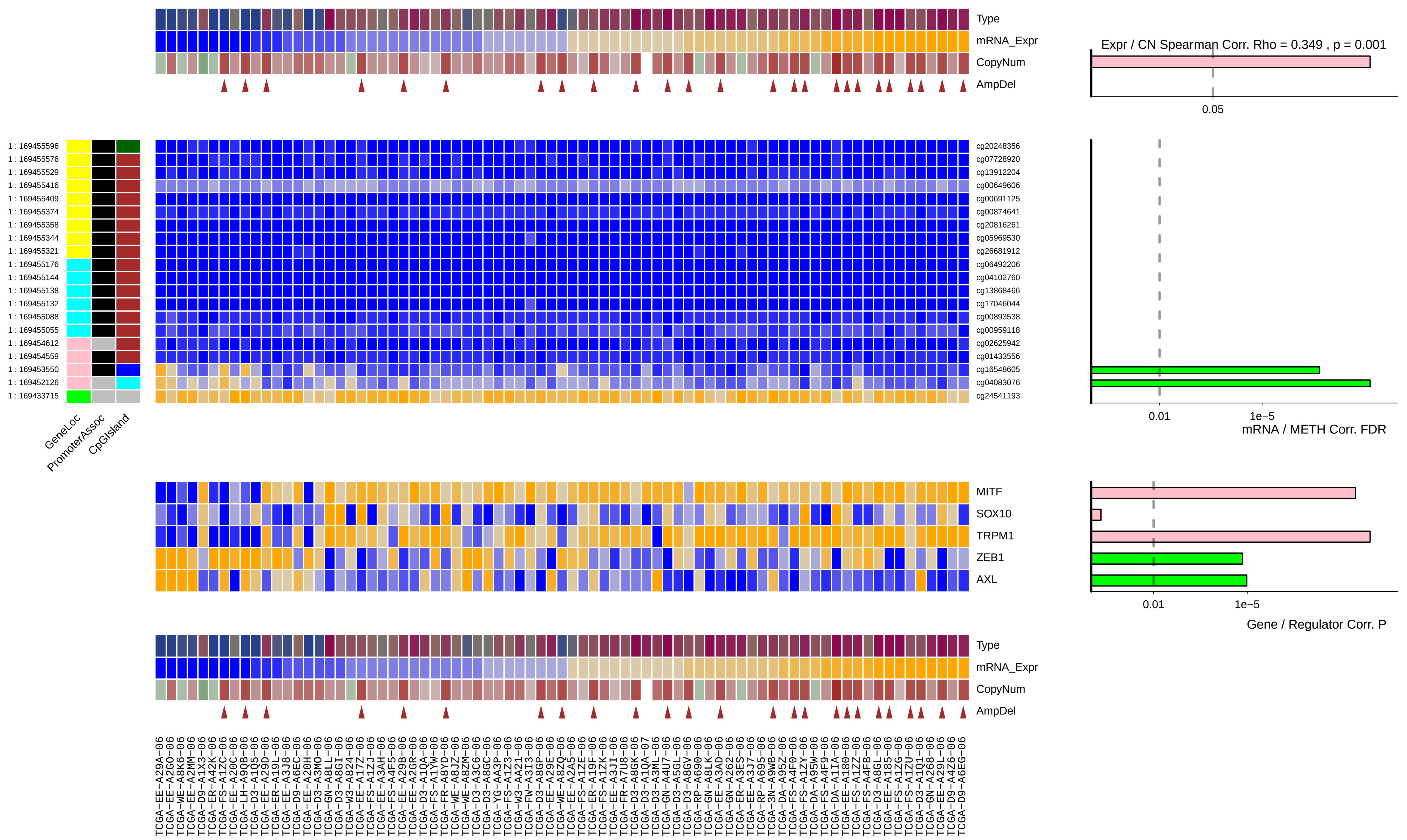

Type
mRNA_Expr
CopyNum
AmpDel
Expr / CN Spearman Corr. Rho = 0.349 , p = 0.001
0.05
GeneLoc
PromoterAssoc
CpGIsland
0.01
1e−5
mRNA / METH Corr. FDR
MITF
SOX10
TRPM1
ZEB1
AXL
Gene / Regulator Corr. P

LONRF1

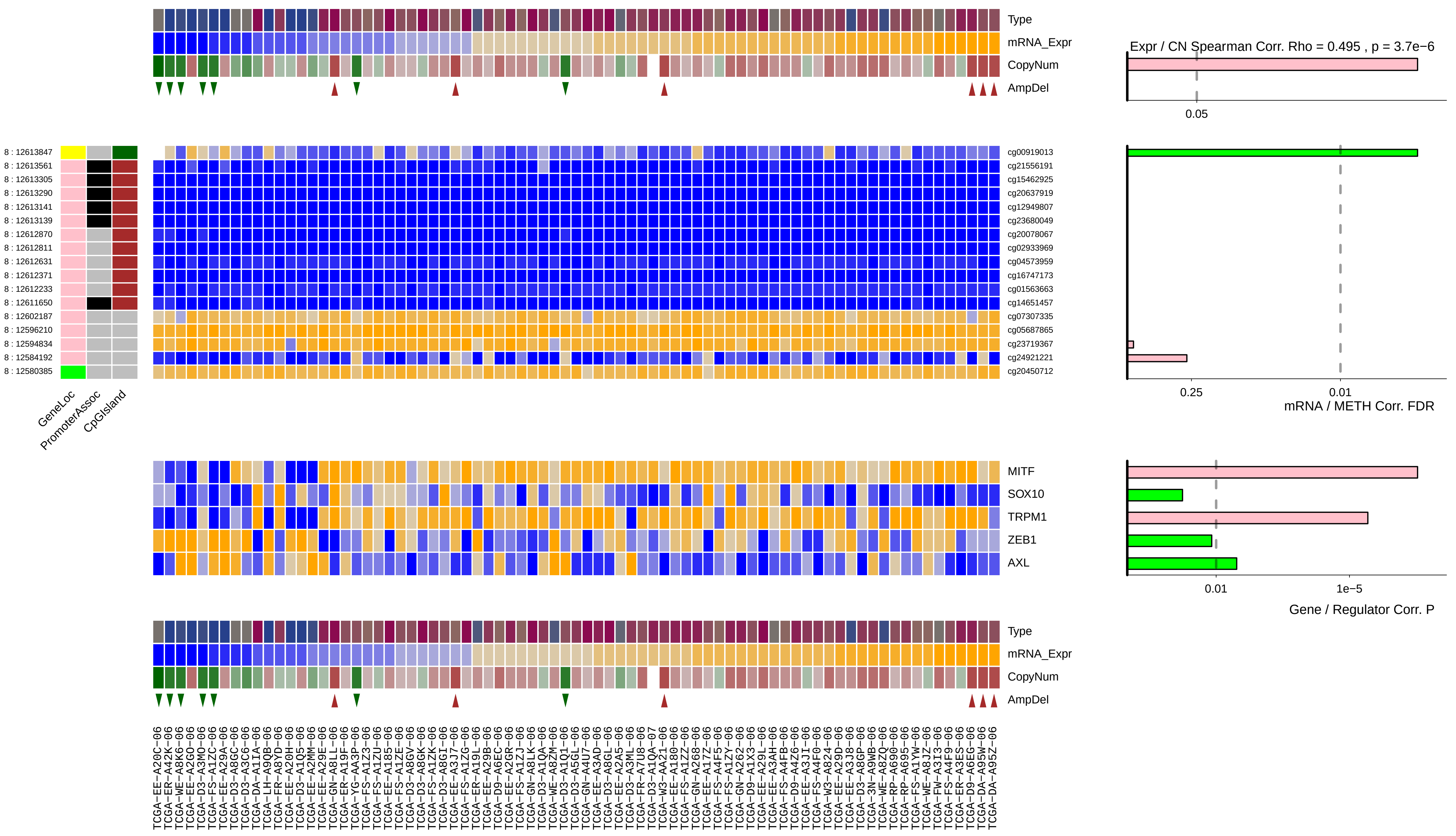

Type
mRNA_Expr
CopyNum
AmpDel
Expr / CN Spearman Corr. Rho = 0.495 , p = 3.7e−6
0.05
8 : 12613847
8 : 12613561
8 : 12613305
8 : 12613290
8 : 12613141
8 : 12613139
8 : 12612870
8 : 12612811
8 : 12612631
8 : 12612371
8 : 12612233
8 : 12611650
8 : 12602187
8 : 12596210
8 : 12594834
8 : 12584192
8 : 12580385
cg00919013
cg21556191
cg15462925
cg20637919
cg12949807
cg23680049
cg20078067
cg02933969
cg04573959
cg16747173
cg01563663
cg14651457
cg07307335
cg05687865
cg23719367
cg24921221
cg20450712
GeneLoc
PromoterAssoc
CpGIsland
0.25
0.01
mRNA / METH Corr. FDR
MITF
SOX10
TRPM1
ZEB1
AXL
0.01
1e−5
Gene / Regulator Corr. P
Type
mRNA_Expr
CopyNum
AmpDel

ZFYVE16

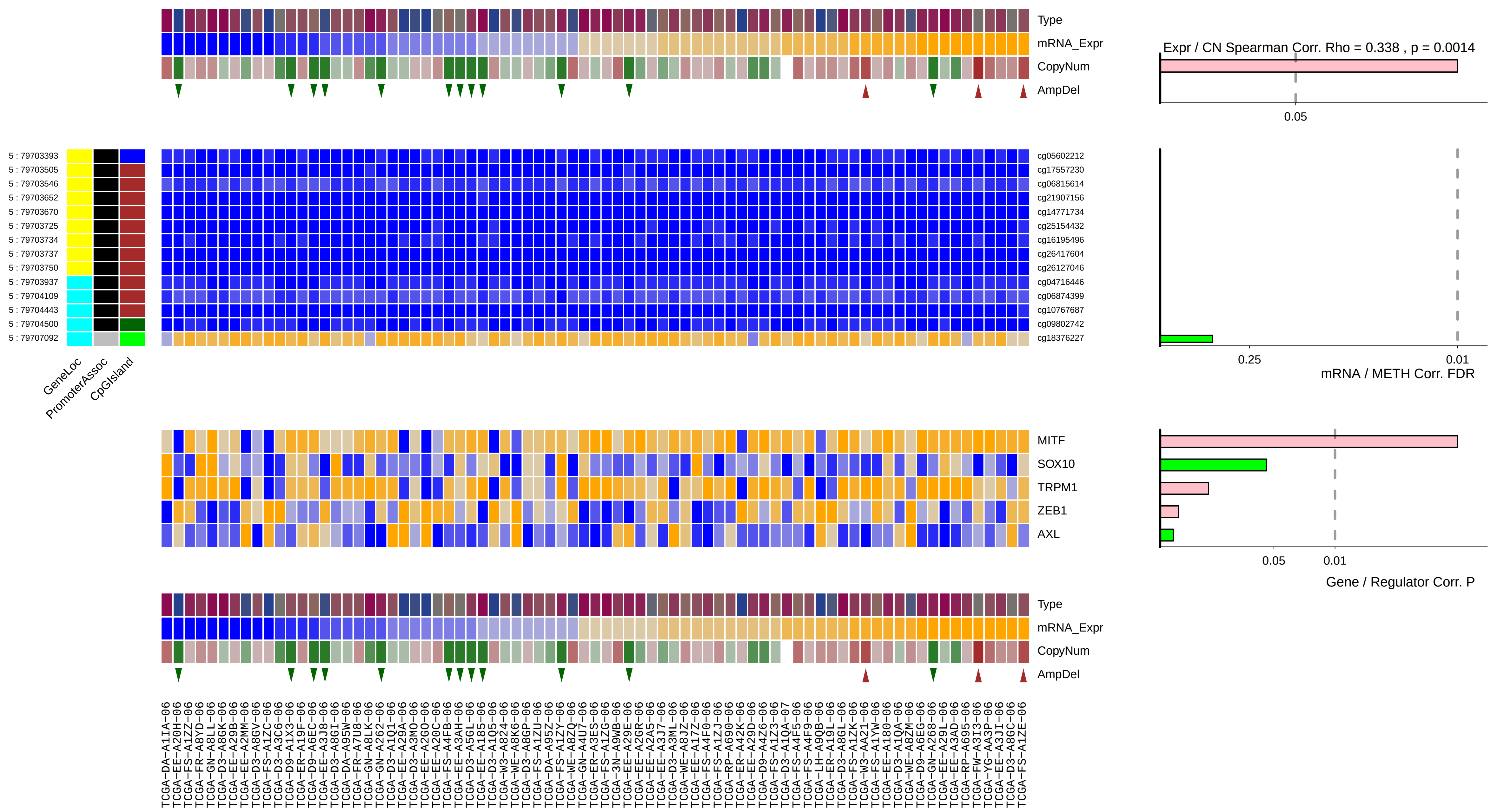

Type
mRNA_Expr
CopyNum
AmpDel
Expr / CN Spearman Corr. Rho = 0.338 , p = 0.0014
0.05
mRNA / METH Corr. FDR
0.25
0.01
GeneLoc
PromoterAssoc
CpGIsland
MITF
SOX10
TRPM1
ZEB1
AXL
Gene / Regulator Corr. P

GALNT3

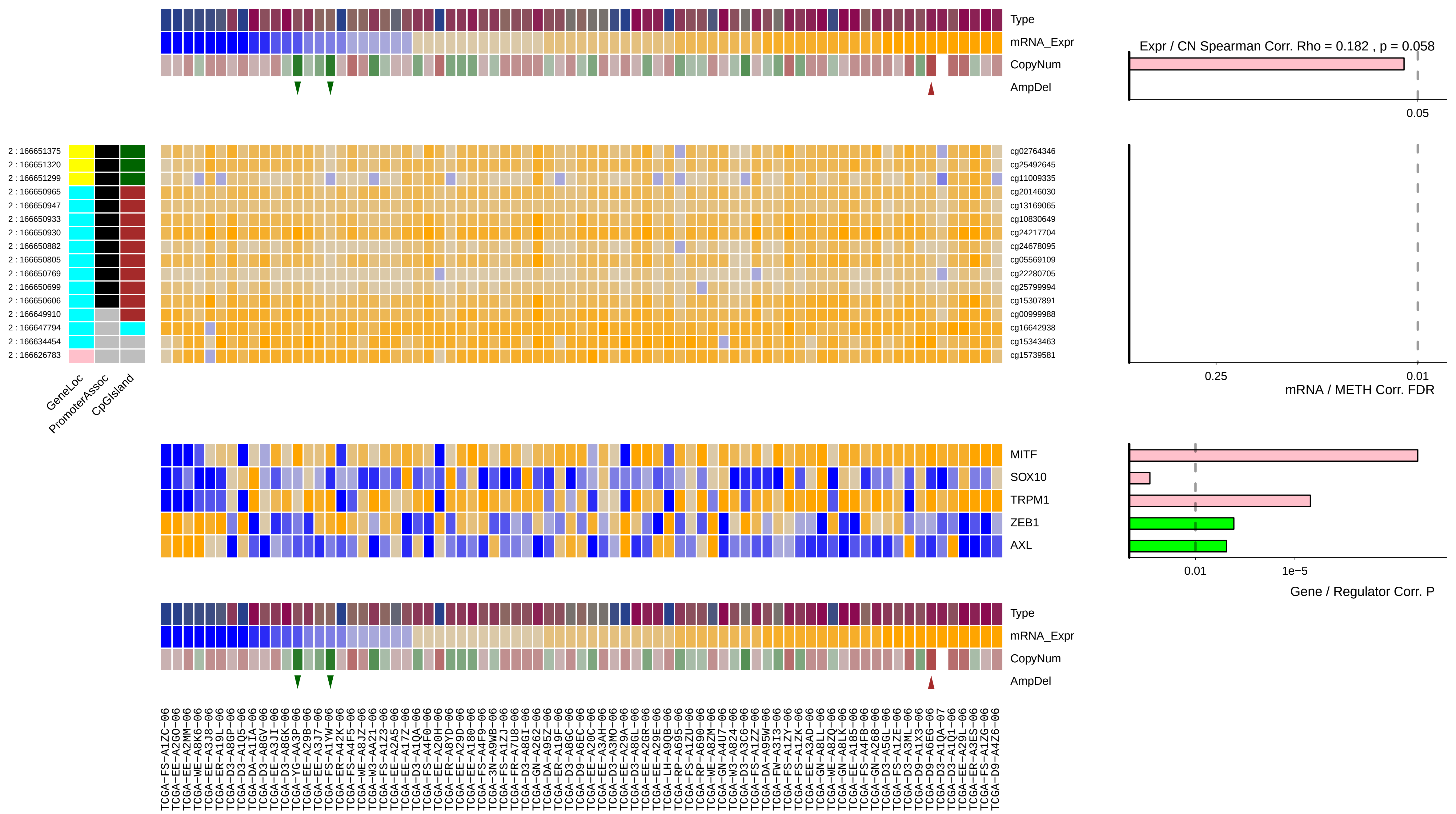

Type
mRNA_Expr
CopyNum
AmpDel
Expr / CN Spearman Corr. Rho = 0.182 , p = 0.058
0.05
2 : 166651375
2 : 166651320
2 : 166651299
2 : 166650965
2 : 166650947
2 : 166650933
2 : 166650930
2 : 166650882
2 : 166650805
2 : 166650769
2 : 166650699
2 : 166650606
2 : 166649910
2 : 166647794
2 : 166634454
2 : 166626783
cg02764346
cg25492645
cg11009335
cg20146030
cg13169065
cg10830649
cg24217704
cg24678095
cg05569109
cg22280705
cg25799994
cg15307891
cg00999988
cg16642938
cg15343463
cg15739581
GeneLoc
PromoterAssoc
CpGIsland
0.25
0.01
mRNA / METH Corr. FDR
MITF
SOX10
TRPM1
ZEB1
AXL
0.01
1e−5
Gene / Regulator Corr. P
Type
mRNA_Expr
CopyNum
AmpDel

APOE

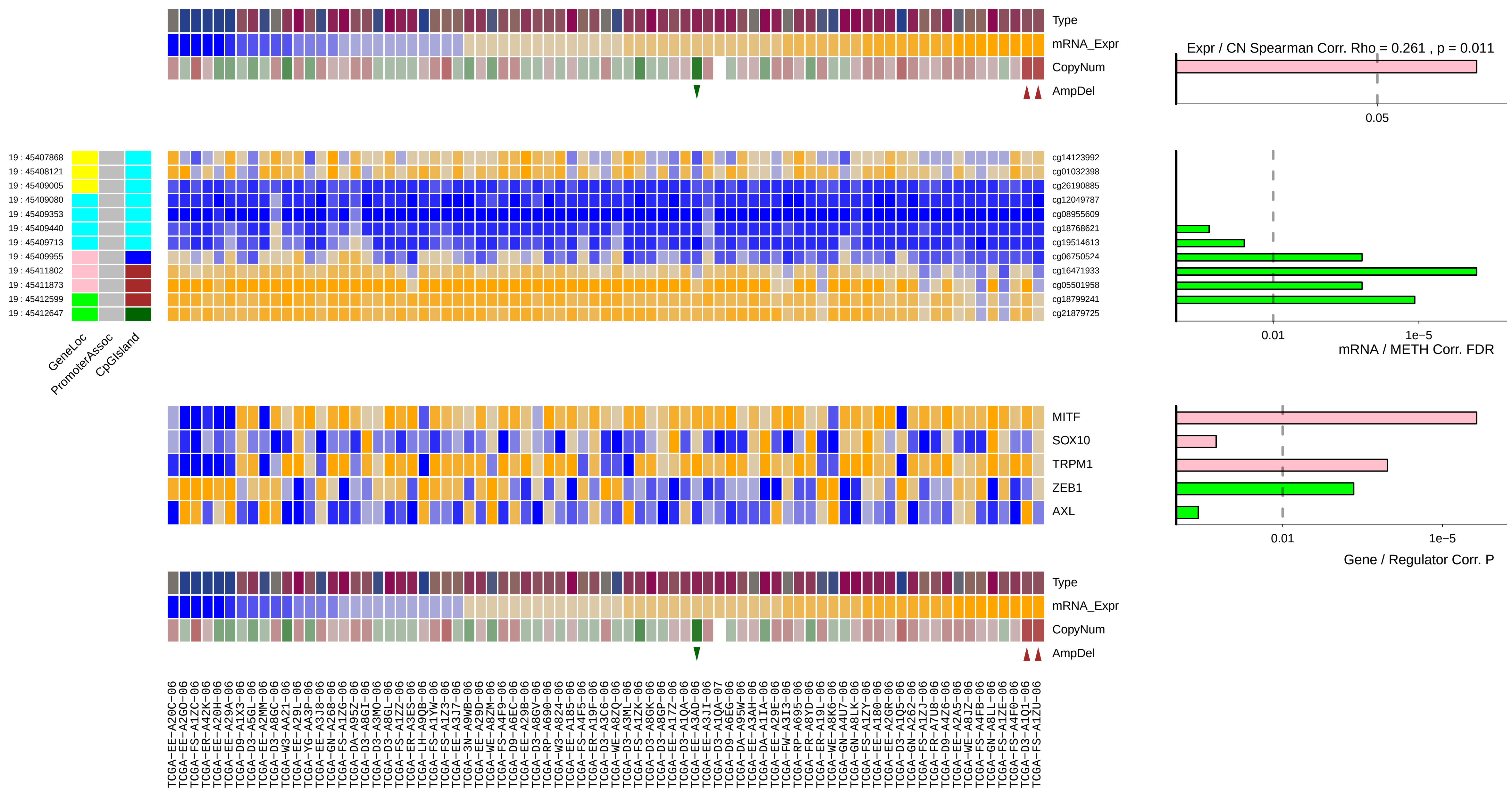

Type
mRNA_Expr
CopyNum
AmpDel
Expr / CN Spearman Corr. Rho = 0.261 , p = 0.011
0.05
19 : 45407868
19 : 45408121
19 : 45409005
19 : 45409080
19 : 45409353
19 : 45409440
19 : 45409713
19 : 45409955
19 : 45411802
19 : 45411873
19 : 45412599
19 : 45412647
cg14123992
cg01032398
cg26190885
cg12049787
cg08955609
cg18768621
cg19514613
cg06750524
cg16471933
cg05501958
cg18799241
cg21879725
GeneLoc
PromoterAssoc
CpGIsland
0.01
1e−5
mRNA / METH Corr. FDR
MITF
SOX10
TRPM1
ZEB1
AXL
0.01
1e−5
Gene / Regulator Corr. P
Type
mRNA_Expr
CopyNum
AmpDel

IL6R

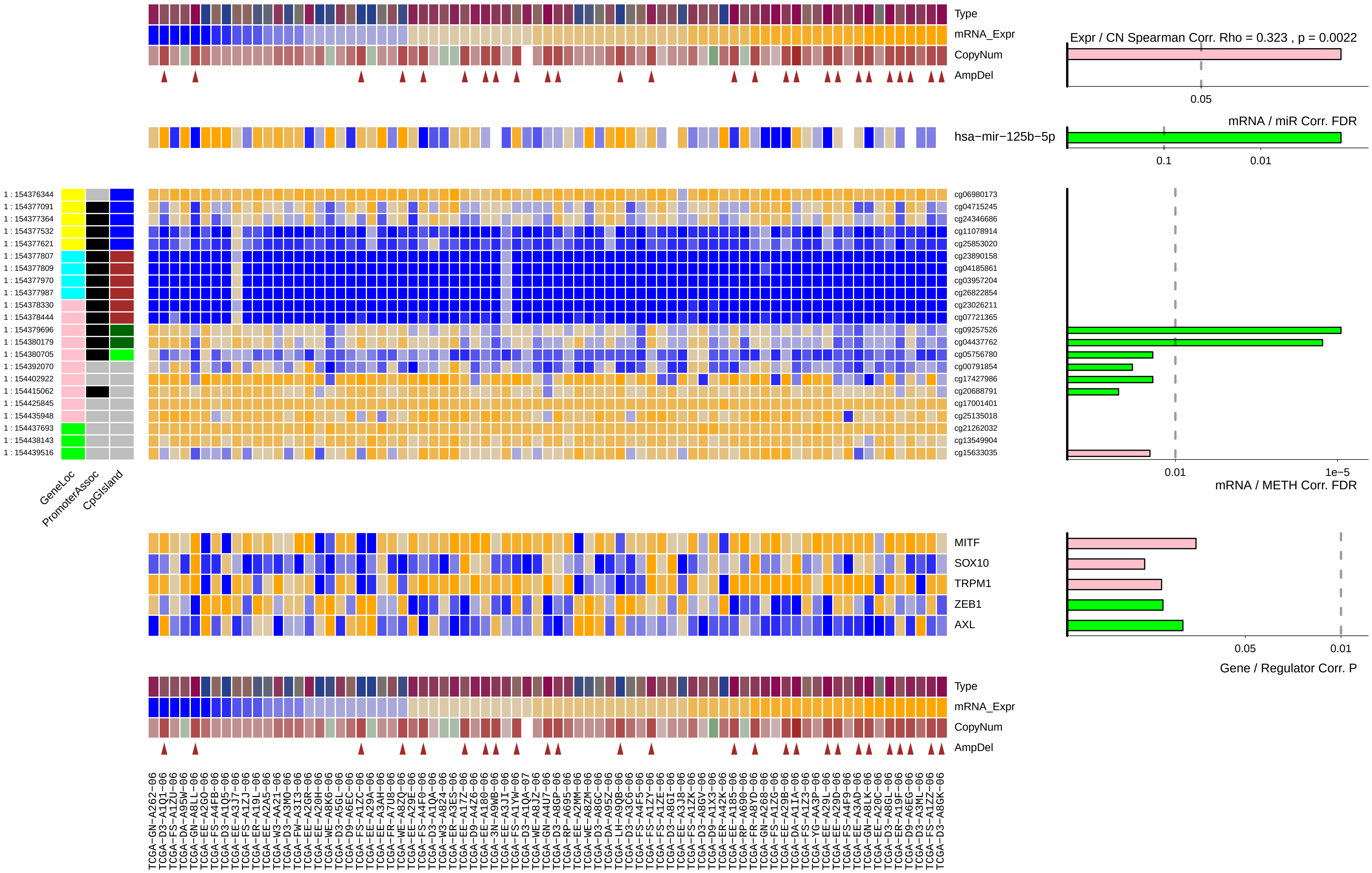

Type
mRNA_Expr
CopyNum
AmpDel
Expr / CN Spearman Corr. Rho = 0.323 , p = 0.0022
0.05
mRNA / miR Corr. FDR
hsa−mir−125b−5p
0.1
0.01
1 : 154376344
1 : 154377091
1 : 154377364
1 : 154377532
1 : 154377621
1 : 154377807
1 : 154377809
1 : 154377970
1 : 154377987
1 : 154378330
1 : 154378444
1 : 154379696
1 : 154380179
1 : 154380705
1 : 154392070
1 : 154402922
1 : 154415062
1 : 154425845
1 : 154435948
1 : 154437693
1 : 154438143
1 : 154439516
cg06980173
cg04715245
cg24346686
cg11078914
cg25853020
cg23890158
cg04185861
cg03957204
cg26822854
cg23026211
cg07721365
cg09257526
cg04437762
cg05756780
cg00791854
cg17427986
cg20688791
cg17001401
cg25135018
cg21262032
cg13549904
cg15633035
0.01
1e−5
mRNA / METH Corr. FDR
GeneLoc
PromoterAssoc
CpGIsland
MITF
SOX10
TRPM1
ZEB1
AXL
0.05
0.01
Gene / Regulator Corr. P
Type
mRNA_Expr
CopyNum
AmpDel

HES6

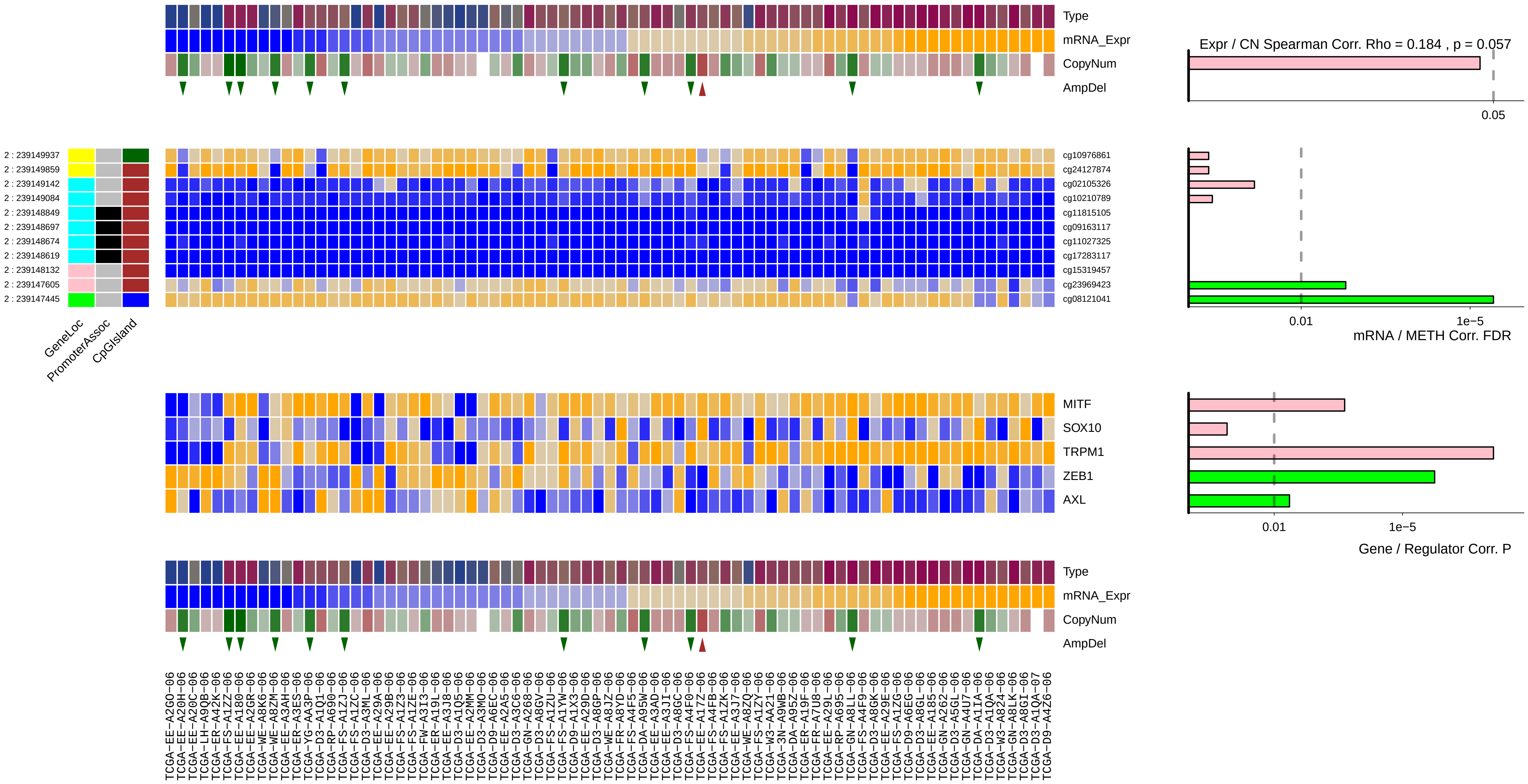
Type
mRNA_Expr
CopyNum
AmpDel
Expr / CN Spearman Corr. Rho = 0.184 , p = 0.057
0.05
2 : 239149937
2 : 239149859
2 : 239149142
2 : 239149084
2 : 239148849
2 : 239148697
2 : 239148674
2 : 239148619
2 : 239148132
2 : 239147605
2 : 239147445
cg10976861
cg24127874
cg02105326
cg10210789
cg11815105
cg09163117
cg11027325
cg17283117
cg15319457
cg23969423
cg08121041
GeneLoc
PromoterAssoc
CpGIsland
0.01
1e−5
mRNA / METH Corr. FDR
MITF
SOX10
TRPM1
ZEB1
AXL
0.01
1e−5
Gene / Regulator Corr. P
Type
mRNA_Expr
CopyNum
AmpDel

HPS4

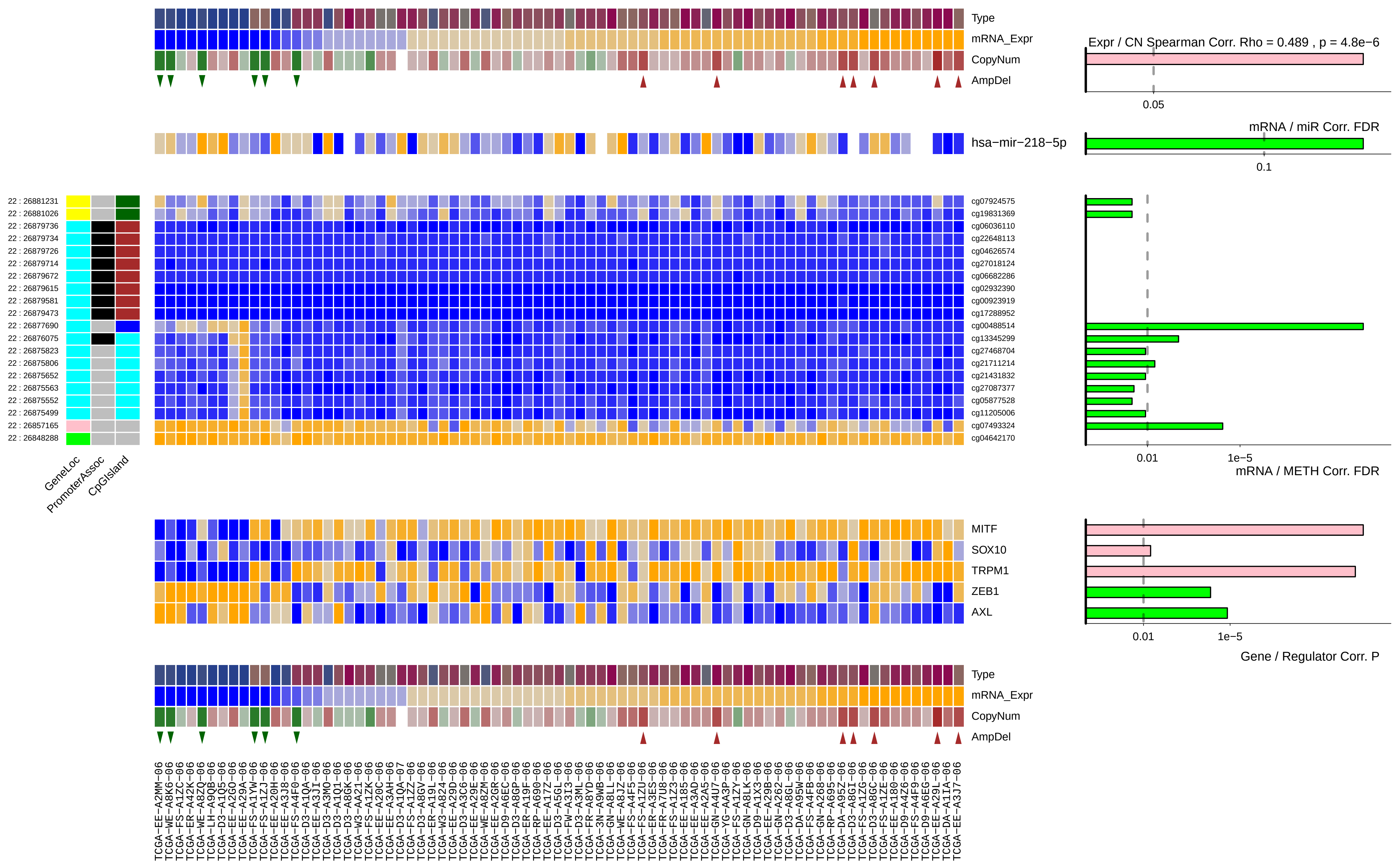
Type
mRNA_Expr
CopyNum
AmpDel
Expr / CN Spearman Corr. Rho = 0.489 , p = 4.8e−6
0.05
mRNA / miR Corr. FDR
hsa−mir−218−5p
0.1
22 : 26881231
22 : 26881026
22 : 26879736
22 : 26879734
22 : 26879726
22 : 26879714
22 : 26879672
22 : 26879615
22 : 26879581
22 : 26879473
22 : 26877690
22 : 26876075
22 : 26875823
22 : 26875806
22 : 26875652
22 : 26875563
22 : 26875552
22 : 26875499
22 : 26857165
22 : 26848288
cg07924575
cg19831369
cg06036110
cg22648113
cg04626574
cg27018124
cg06682286
cg02932390
cg00923919
cg17288952
cg00488514
cg13345299
cg27468704
cg21711214
cg21431832
cg27087377
cg05877528
cg11205006
cg07493324
cg04642170
GeneLoc
PromoterAssoc
CpGIsland
0.01
1e−5
mRNA / METH Corr. FDR
MITF
SOX10
TRPM1
ZEB1
AXL
0.01
1e−5
Gene / Regulator Corr. P
Type
mRNA_Expr
CopyNum
AmpDel

PHACTR1

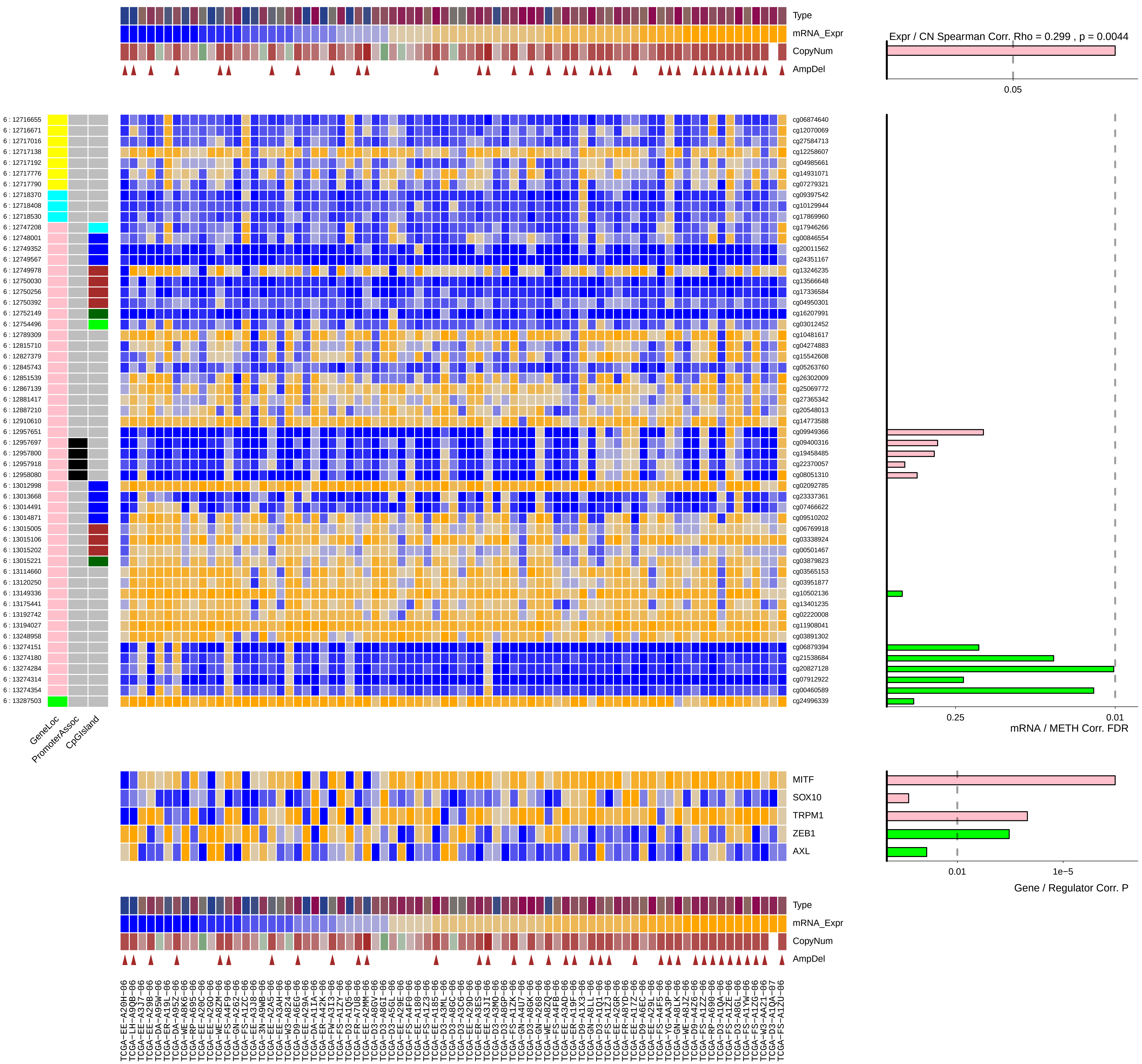

Type
mRNA_Expr
CopyNum
AmpDel
Expr / CN Spearman Corr. Rho = 0.299 , p = 0.0044
0.05
6 : 12716655
6 : 12716671
6 : 12717016
6 : 12717138
6 : 12717192
6 : 12717776
6 : 12717790
6 : 12718370
6 : 12718408
6 : 12718530
6 : 12747208
6 : 12748001
6 : 12749352
6 : 12749567
6 : 12749978
6 : 12750030
6 : 12750256
6 : 12750392
6 : 12752149
6 : 12754496
6 : 12789309
6 : 12815710
6 : 12827379
6 : 12845743
6 : 12851539
6 : 12867139
6 : 12881417
6 : 12887210
6 : 12910610
6 : 12957651
6 : 12957697
6 : 12957800
6 : 12957918
6 : 12958080
6 : 13012998
6 : 13013668
6 : 13014491
6 : 13014871
6 : 13015005
6 : 13015106
6 : 13015202
6 : 13015221
6 : 13114660
6 : 13120250
6 : 13149336
6 : 13175441
6 : 13192742
6 : 13194027
6 : 13248958
6 : 13274151
6 : 13274180
6 : 13274284
6 : 13274314
6 : 13274354
6 : 13287503
cg06874640
cg12070069
cg27584713
cg12258607
cg04985661
cg14931071
cg07279321
cg09397542
cg10129944
cg17869960
cg17946266
cg00846554
cg20011562
cg24351167
cg13246235
cg13566648
cg17336584
cg04950301
cg16207991
cg03012452
cg10481617
cg04274883
cg15542608
cg05263760
cg26302009
cg25069772
cg27365342
cg20548013
cg14773588
cg09949366
cg09400316
cg19458485
cg22370057
cg08051310
cg02092785
cg23337361
cg07466622
cg09510202
cg06769918
cg03338924
cg00501467
cg03879823
cg03565153
cg03951877
cg10502136
cg13401235
cg02220008
cg11908041
cg03891302
cg06879394
cg21538684
cg20827128
cg07912922
cg00460589
cg24996339
0.25
0.01
mRNA / METH Corr. FDR
GeneLoc
PromoterAssoc
CpGIsland
MITF
SOX10
TRPM1
ZEB1
AXL
0.01
1e−5
Gene / Regulator Corr. P
Type
mRNA_Expr
CopyNum
AmpDel

SOX6

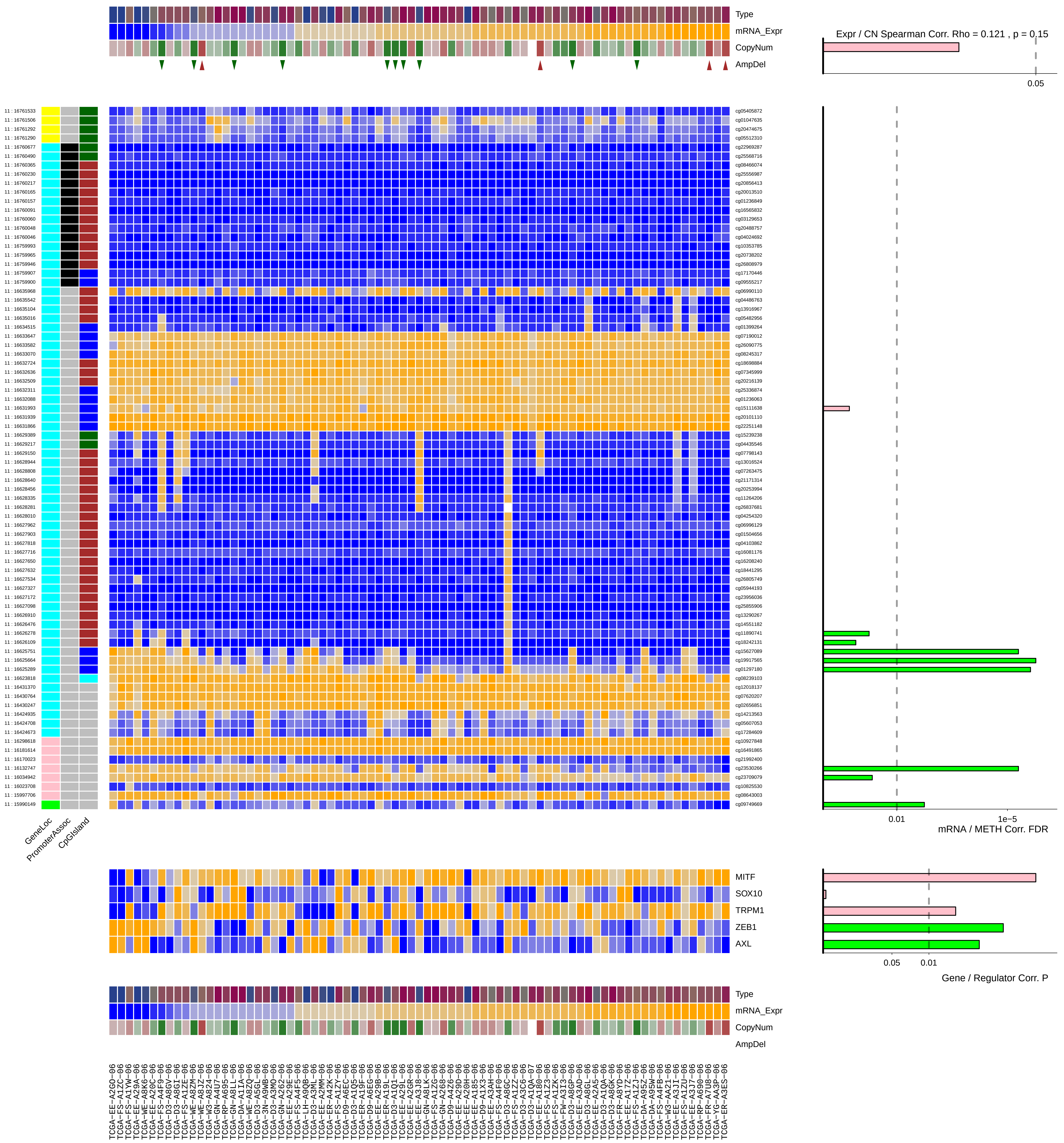

Type
mRNA_Expr
CopyNum
AmpDel
Expr / CN Spearman Corr. Rho = 0.121 , p = 0.15
0.05
GeneLoc
PromoterAssoc
CpGIsland
0.01
1e−5
mRNA / METH Corr. FDR
MITF
SOX10
TRPM1
ZEB1
AXL
0.05
0.01
Gene / Regulator Corr. P
Type
mRNA_Expr
CopyNum
AmpDel

ERBB3

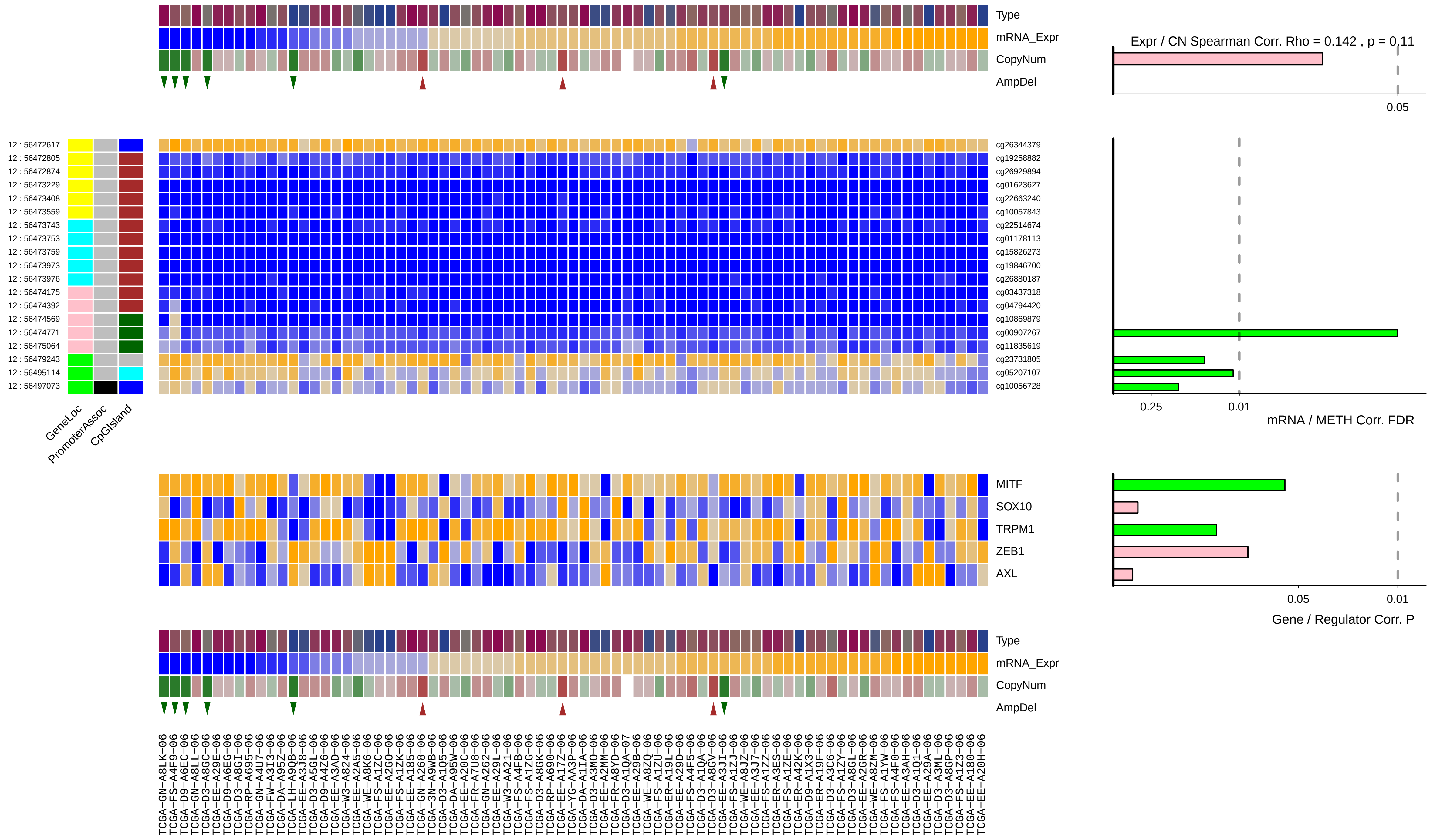

Type
mRNA_Expr
CopyNum
AmpDel
Expr / CN Spearman Corr. Rho = 0.142 , p = 0.11
0.05
12 : 56472617
12 : 56472805
12 : 56472874
12 : 56473229
12 : 56473408
12 : 56473559
12 : 56473743
12 : 56473753
12 : 56473759
12 : 56473973
12 : 56473976
12 : 56474175
12 : 56474392
12 : 56474569
12 : 56474771
12 : 56475064
12 : 56479243
12 : 56495114
12 : 56497073
cg26344379
cg19258882
cg26929894
cg01623627
cg22663240
cg10057843
cg22514674
cg01178113
cg15826273
cg19846700
cg26880187
cg03437318
cg04794420
cg10869879
cg00907267
cg11835619
cg23731805
cg05207107
cg10056728
GeneLoc
PromoterAssoc
CpGIsland
0.25
0.01
mRNA / METH Corr. FDR
MITF
SOX10
TRPM1
ZEB1
AXL
0.05
0.01
Gene / Regulator Corr. P
Type
mRNA_Expr
CopyNum
AmpDel

PIR

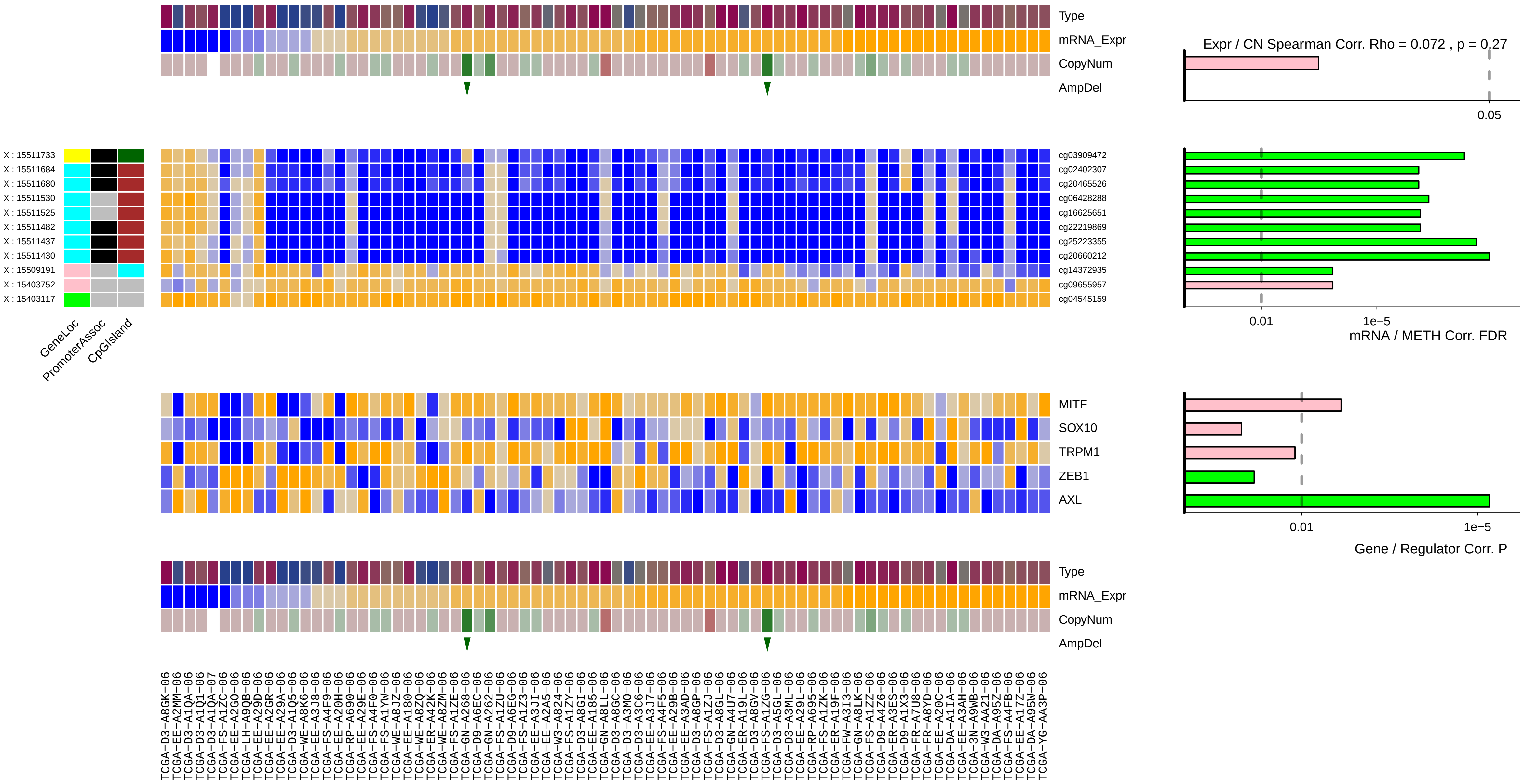

Type
mRNA_Expr
CopyNum
AmpDel
Expr / CN Spearman Corr. Rho = 0.072 , p = 0.27
0.05
X : 15511733
X : 15511684
X : 15511680
X : 15511530
X : 15511525
X : 15511482
X : 15511437
X : 15511430
X : 15509191
X : 15403752
X : 15403117
GeneLoc
PromoterAssoc
CpGIsland
cg03909472
cg02402307
cg20465526
cg06428288
cg16625651
cg22219869
cg25223355
cg20660212
cg14372935
cg09655957
cg04545159
0.01
1e−5
mRNA / METH Corr. FDR
MITF
SOX10
TRPM1
ZEB1
AXL
0.01
1e−5
Gene / Regulator Corr. P

STK32A

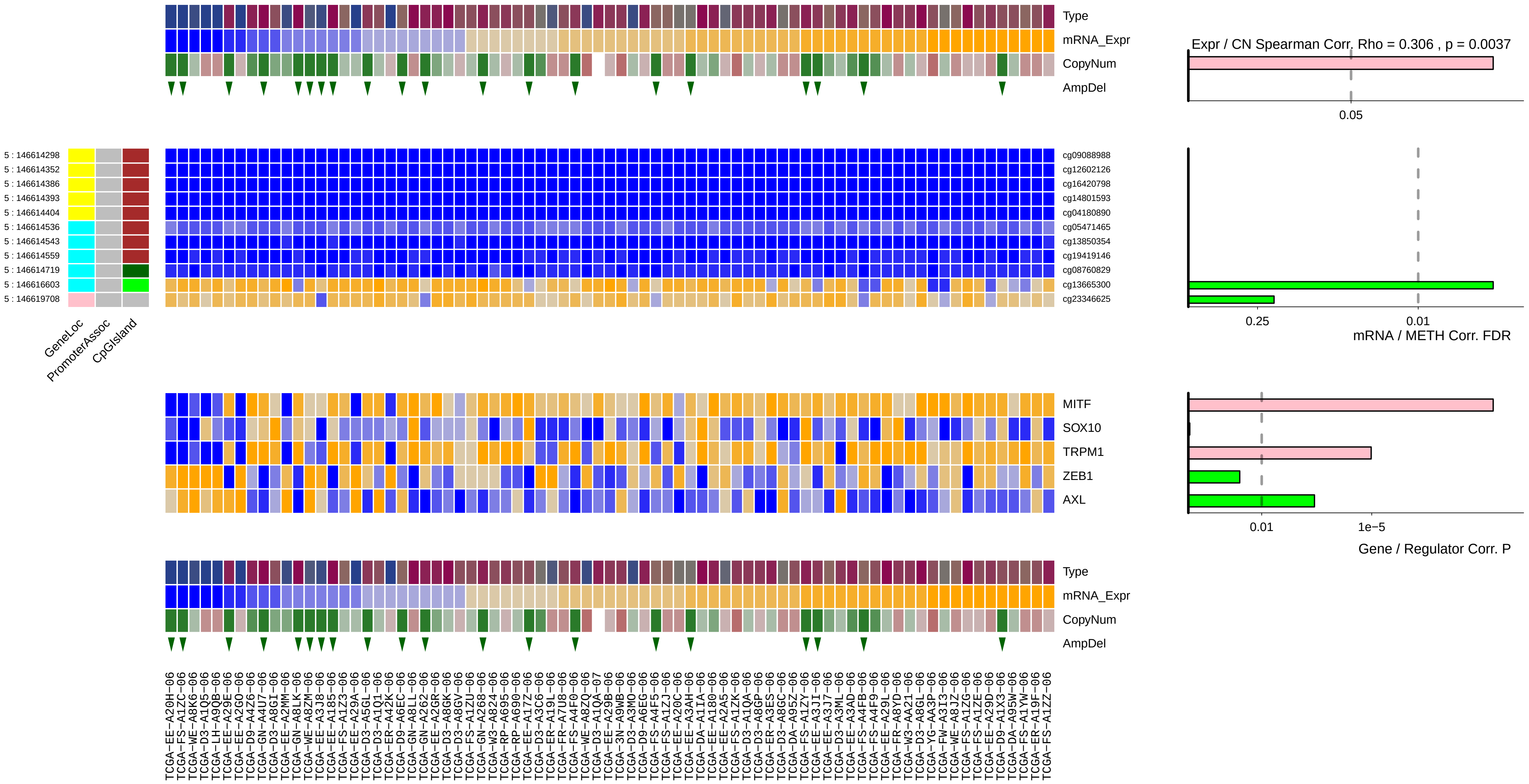

Type
mRNA_Expr
CopyNum
AmpDel
Expr / CN Spearman Corr. Rho = 0.306 , p = 0.0037
0.05
0.25
0.01
mRNA / METH Corr. FDR
GeneLoc
PromoterAssoc
CpGIsland
MITF
SOX10
TRPM1
ZEB1
AXL
0.01
1e−5
Gene / Regulator Corr. P

SCARB1

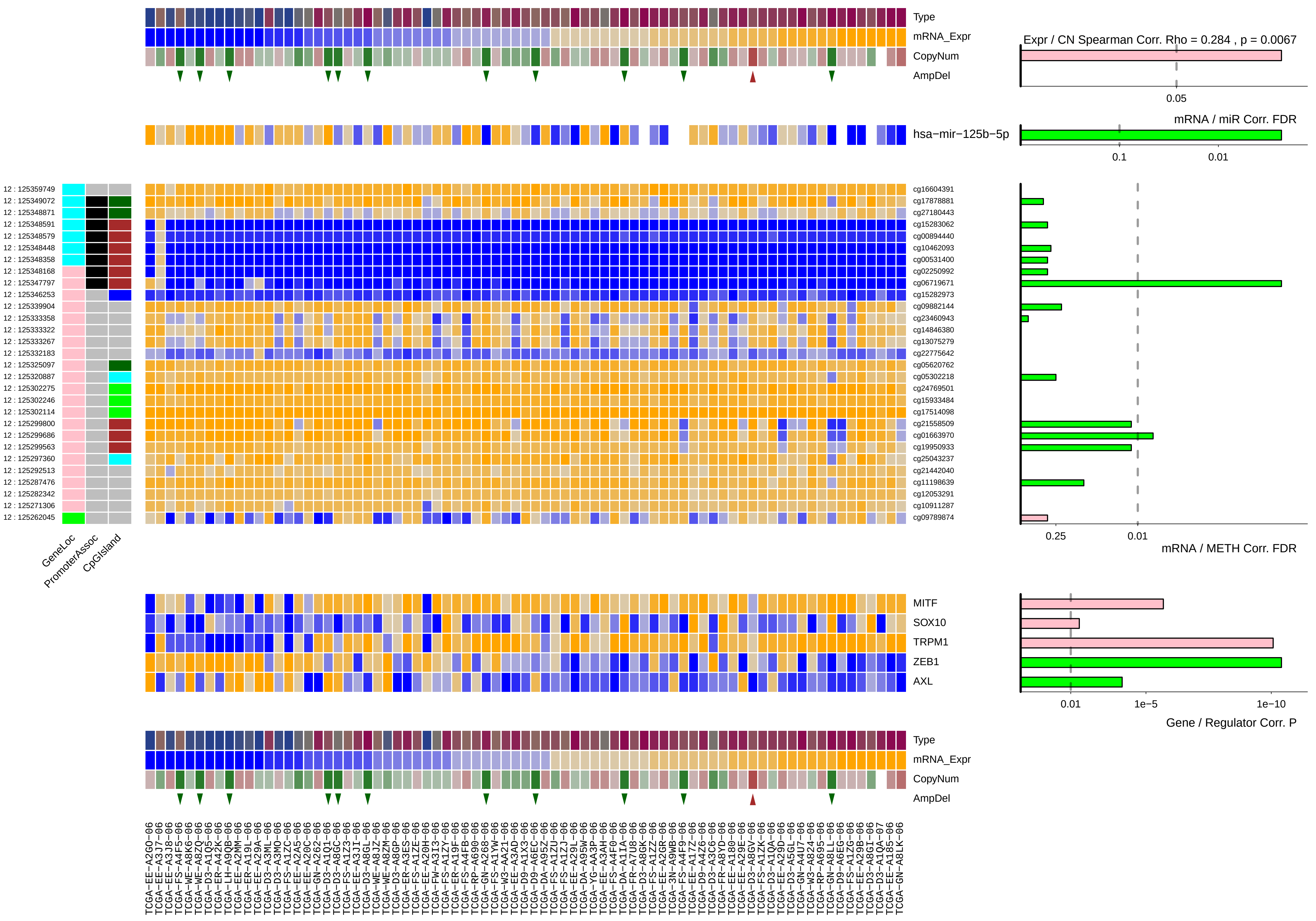

Type
mRNA_Expr
CopyNum
AmpDel
Expr / CN Spearman Corr. Rho = 0.284 , p = 0.0067
0.05
hsa−mir−125b−5p
mRNA / miR Corr. FDR
0.1
0.01
GeneLoc
PromoterAssoc
CpGIsland
0.25
0.01
mRNA / METH Corr. FDR
MITF
SOX10
TRPM1
ZEB1
AXL
0.01
1e−5
1e−10
Gene / Regulator Corr. P

OVOS2

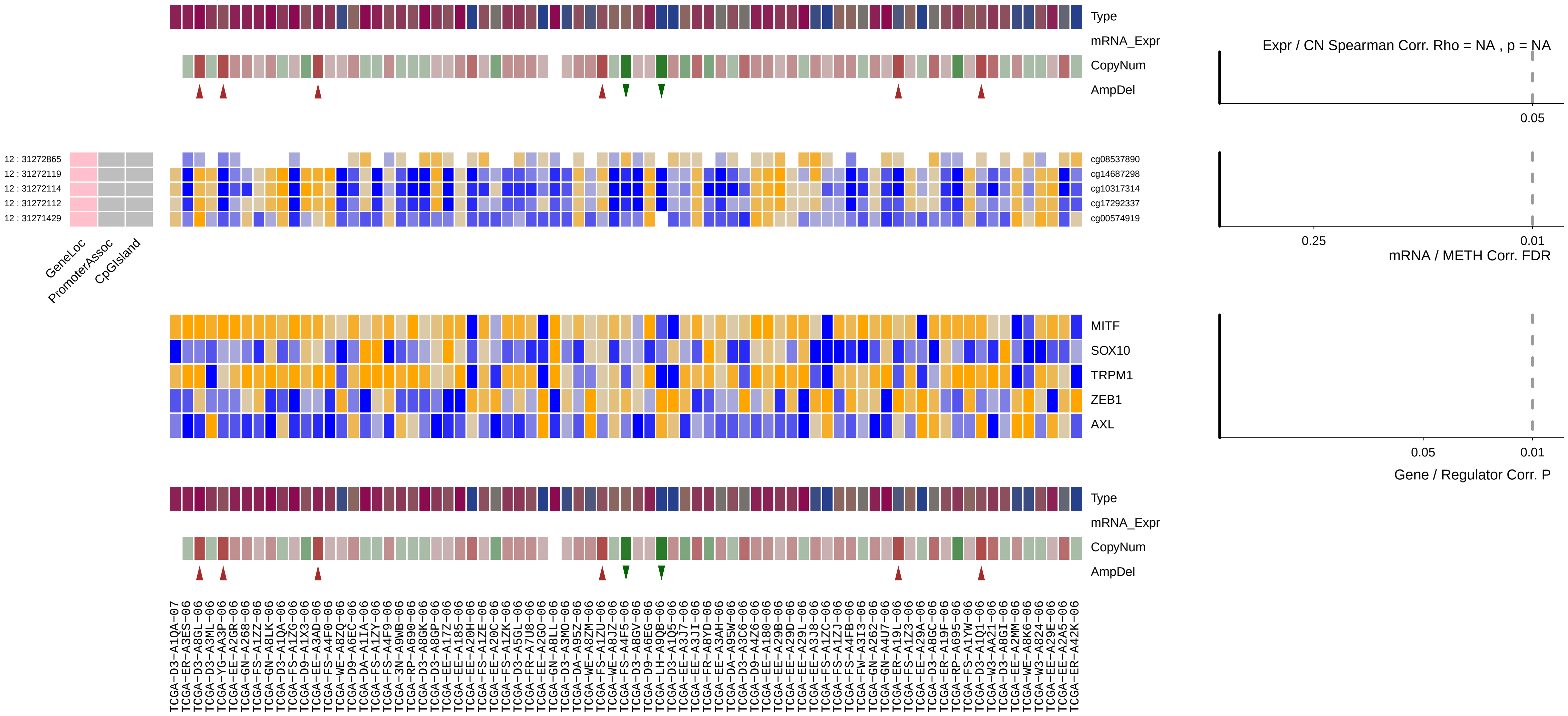
Type
mRNA_Expr
CopyNum
AmpDel
Expr / CN Spearman Corr. Rho = NA , p = NA
0.05
12 : 31272865
12 : 31272119
12 : 31272114
12 : 31272112
12 : 31271429
cg08537890
cg14687298
cg10317314
cg17292337
cg00574919
GeneLoc
PromoterAssoc
CpGIsland
0.25
0.01
mRNA / METH Corr. FDR
MITF
SOX10
TRPM1
ZEB1
AXL
0.05
0.01
Gene / Regulator Corr. P

CHCHD6

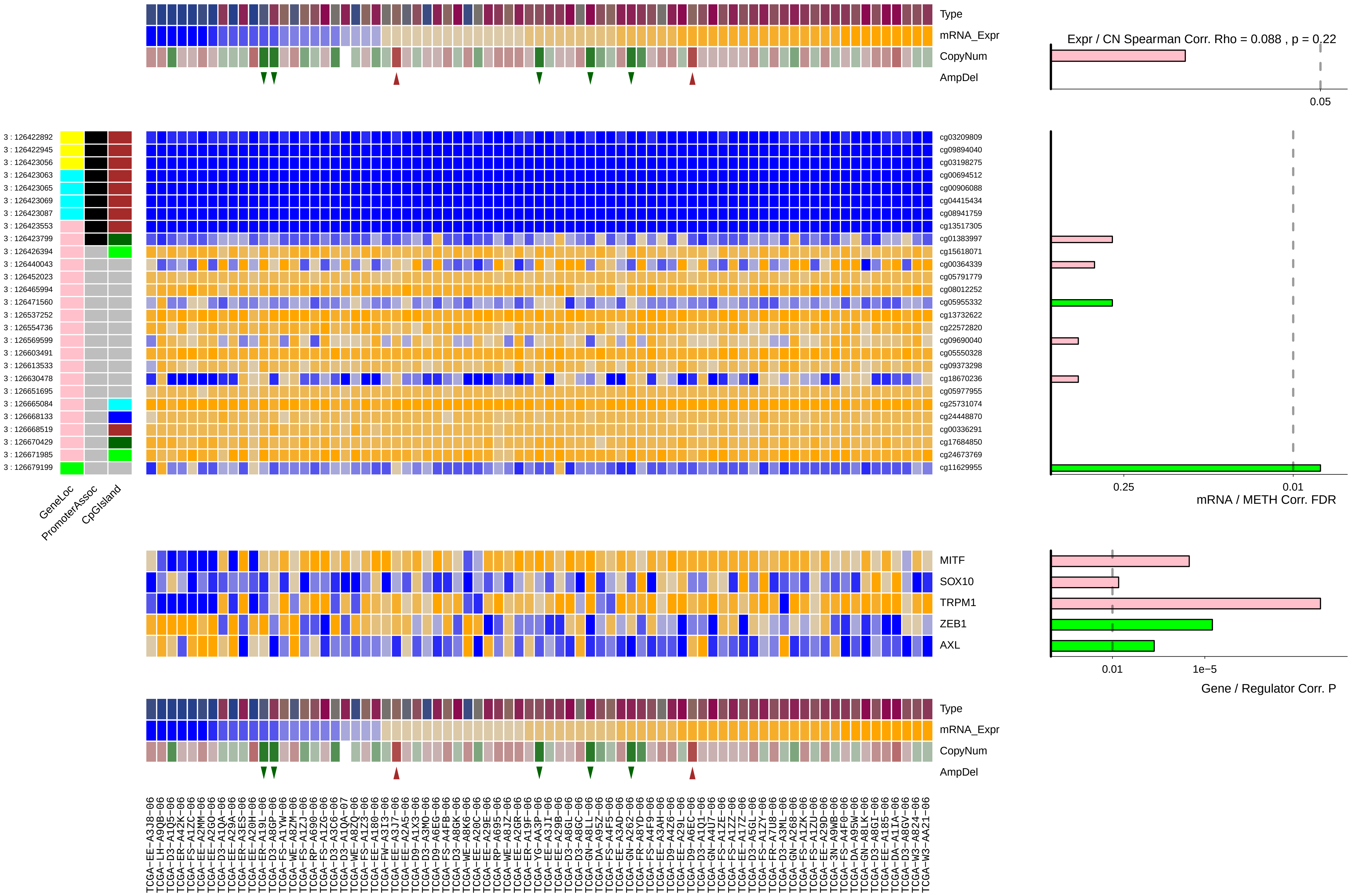
Type
mRNA_Expr
CopyNum
AmpDel
Expr / CN Spearman Corr. Rho = 0.088 , p = 0.22
0.05
3 : 126422892
3 : 126422945
3 : 126423056
3 : 126423063
3 : 126423065
3 : 126423069
3 : 126423087
3 : 126423553
3 : 126423799
3 : 126426394
3 : 126440043
3 : 126452023
3 : 126465994
3 : 126471560
3 : 126537252
3 : 126554736
3 : 126569599
3 : 126603491
3 : 126613533
3 : 126630478
3 : 126651695
3 : 126665084
3 : 126668133
3 : 126668519
3 : 126670429
3 : 126671985
3 : 126679199
cg03209809
cg09894040
cg03198275
cg00694512
cg00906088
cg04415434
cg08941759
cg13517305
cg01383997
cg15618071
cg00364339
cg05791779
cg08012252
cg05955332
cg13732622
cg22572820
cg09690040
cg05550328
cg09373298
cg18670236
cg05977955
cg25731074
cg24448870
cg00336291
cg17684850
cg24673769
cg11629955
0.25
0.01
mRNA / METH Corr. FDR
GeneLoc
PromoterAssoc
CpGIsland
MITF
SOX10
TRPM1
ZEB1
AXL
0.01
1e−5
Gene / Regulator Corr. P
Type
mRNA_Expr
CopyNum
AmpDel

SORT1

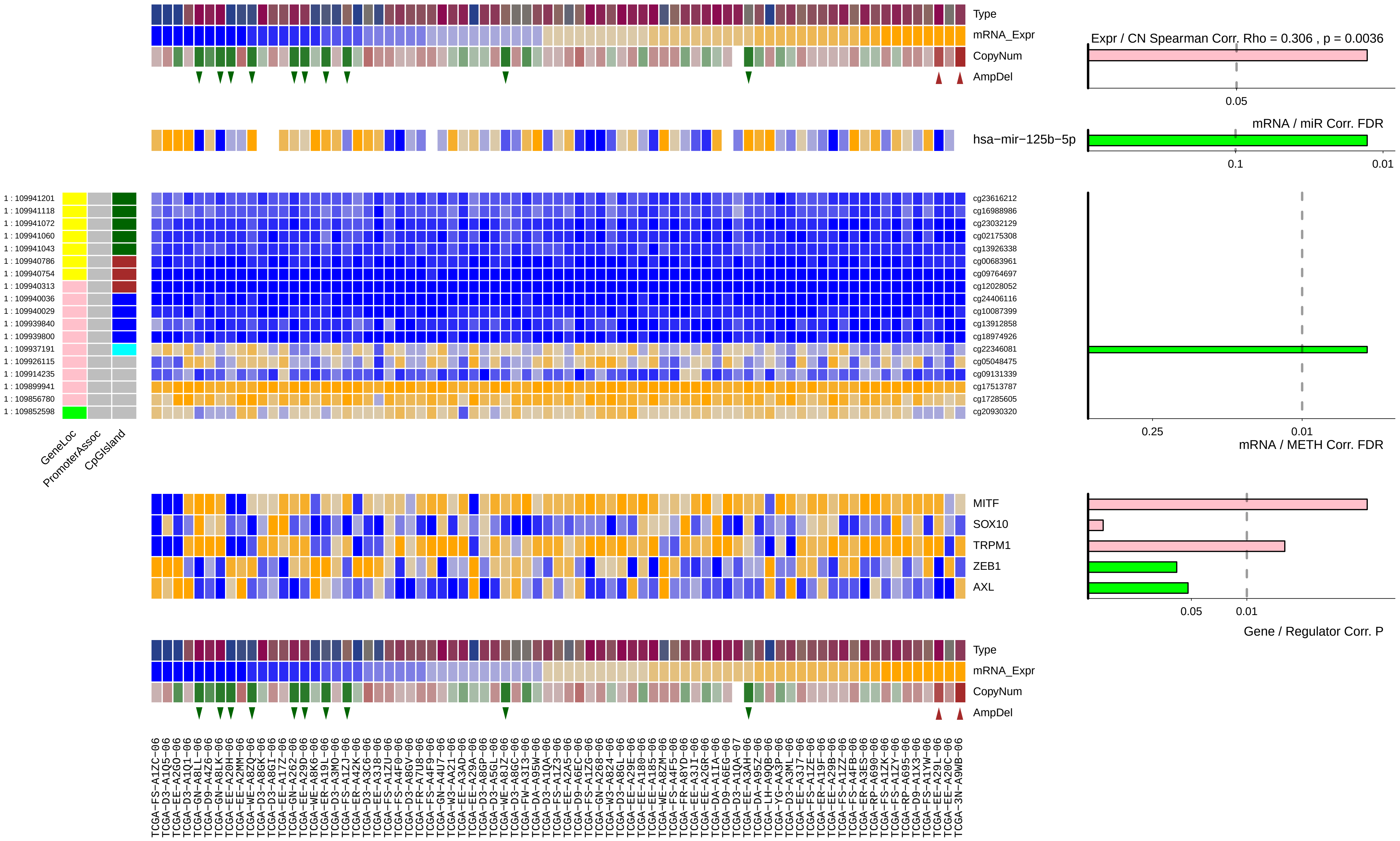

Type
mRNA_Expr
CopyNum
AmpDel
Expr / CN Spearman Corr. Rho = 0.306 , p = 0.0036
0.05
mRNA / miR Corr. FDR
hsa−mir−125b−5p
0.1
0.01
1 : 109941201
1 : 109941118
1 : 109941072
1 : 109941060
1 : 109941043
1 : 109940786
1 : 109940754
1 : 109940313
1 : 109940036
1 : 109940029
1 : 109939840
1 : 109939800
1 : 109937191
1 : 109926115
1 : 109914235
1 : 109899941
1 : 109856780
1 : 109852598
cg23616212
cg16988986
cg23032129
cg02175308
cg13926338
cg00683961
cg09764697
cg12028052
cg24406116
cg10087399
cg13912858
cg18974926
cg22346081
cg05048475
cg09131339
cg17513787
cg17285605
cg20930320
GeneLoc
PromoterAssoc
CpGIsland
0.25
0.01
mRNA / METH Corr. FDR
MITF
SOX10
TRPM1
ZEB1
AXL
0.05
0.01
Gene / Regulator Corr. P
Type
mRNA_Expr
CopyNum
AmpDel

MICAL1

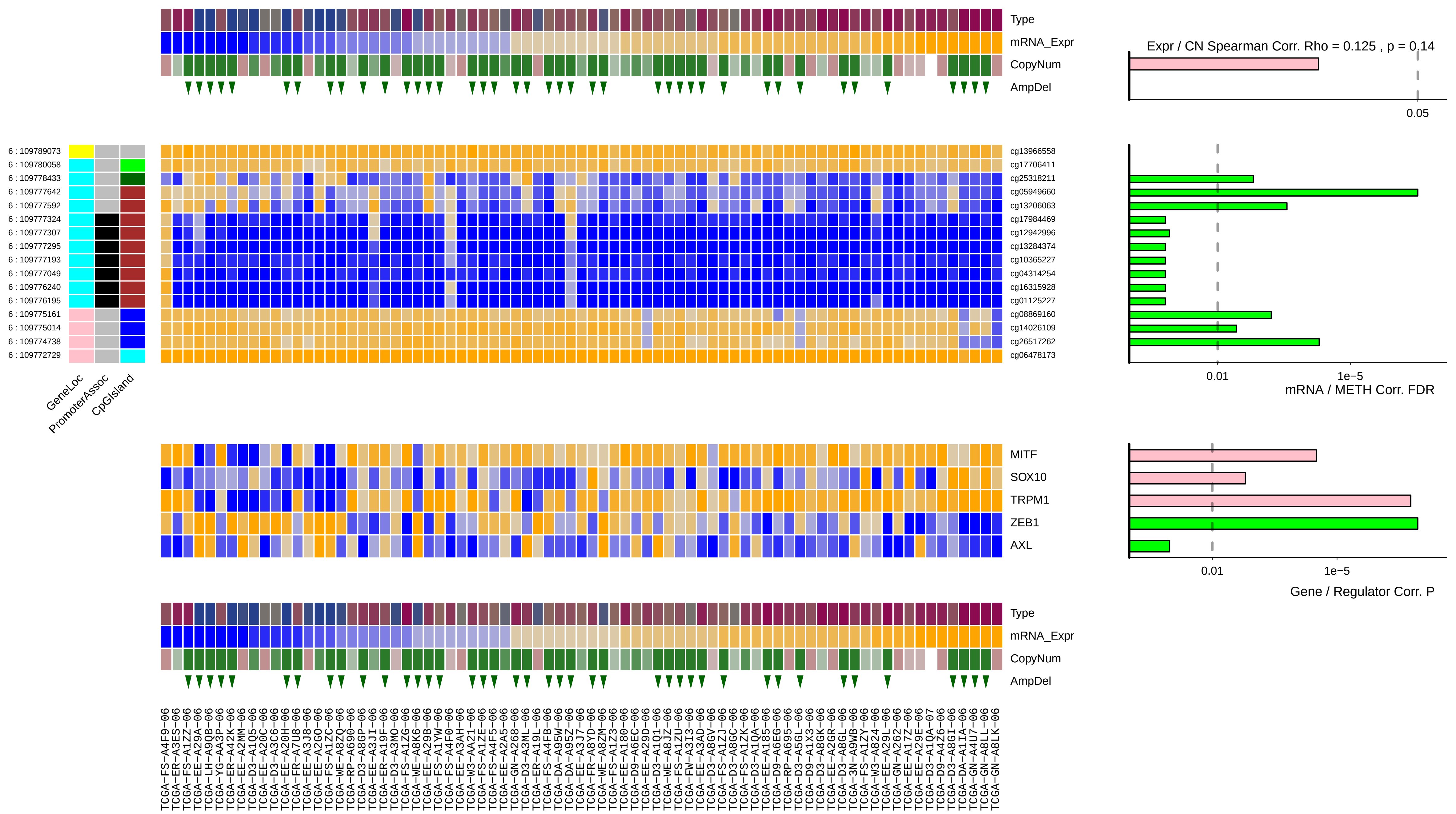

Type
mRNA_Expr
CopyNum
AmpDel
Expr / CN Spearman Corr. Rho = 0.125 , p = 0.14
0.05
6 : 109789073
6 : 109780058
6 : 109778433
6 : 109777642
6 : 109777592
6 : 109777324
6 : 109777307
6 : 109777295
6 : 109777193
6 : 109777049
6 : 109776240
6 : 109776195
6 : 109775161
6 : 109775014
6 : 109774738
6 : 109772729
cg13966558
cg17706411
cg25318211
cg05949660
cg13206063
cg17984469
cg12942996
cg13284374
cg10365227
cg04314254
cg16315928
cg01125227
cg08869160
cg14026109
cg26517262
cg06478173
GeneLoc
PromoterAssoc
CpGIsland
0.01
1e−5
mRNA / METH Corr. FDR
MITF
SOX10
TRPM1
ZEB1
AXL
0.01
1e−5
Gene / Regulator Corr. P
Type
mRNA_Expr
CopyNum
AmpDel

PRDM7

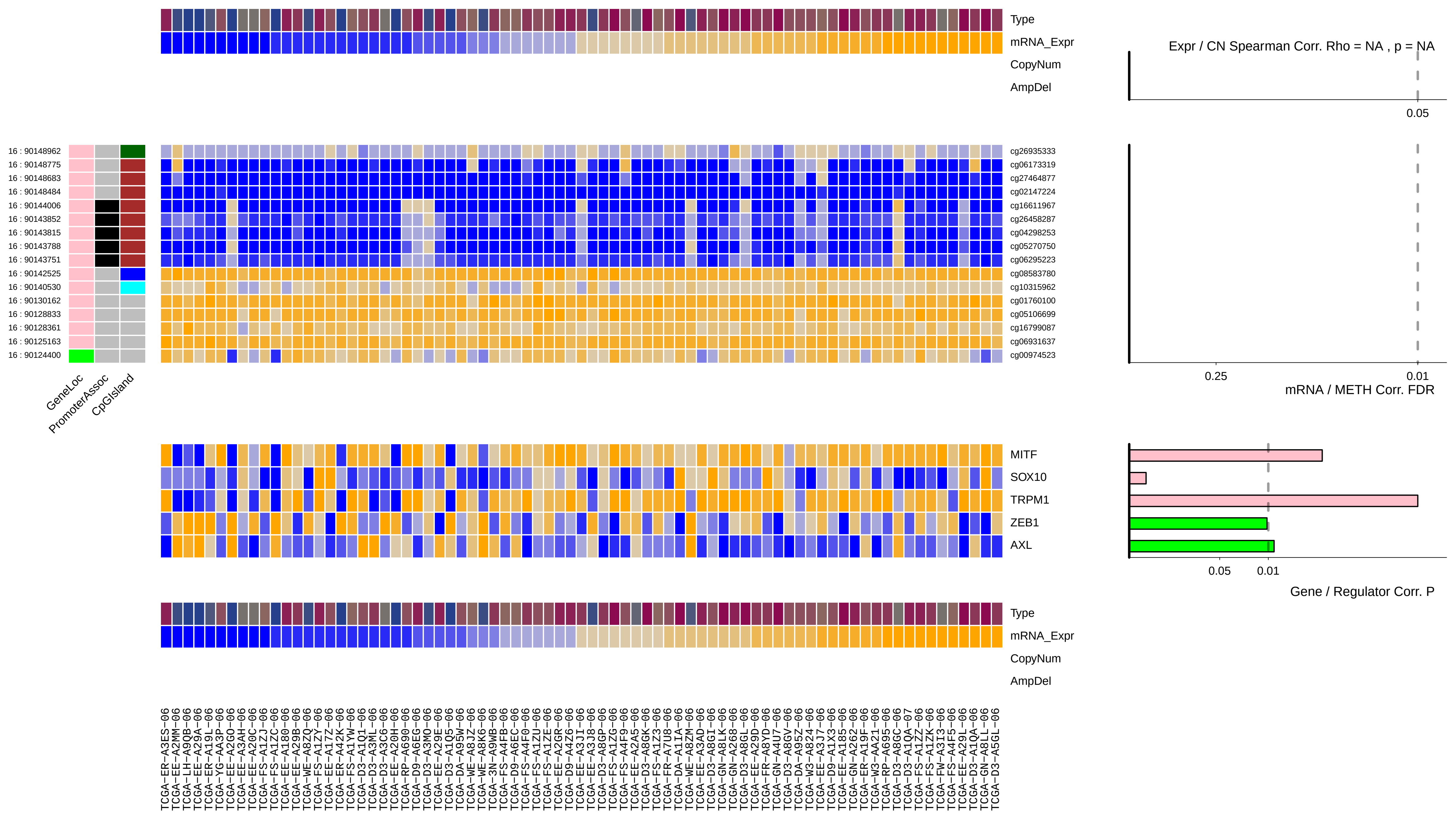

Type
mRNA_Expr
CopyNum
AmpDel
Expr / CN Spearman Corr. Rho = NA , p = NA
0.05
16 : 90148962
16 : 90148775
16 : 90148683
16 : 90148484
16 : 90144006
16 : 90143852
16 : 90143815
16 : 90143788
16 : 90143751
16 : 90142525
16 : 90140530
16 : 90130162
16 : 90128833
16 : 90128361
16 : 90125163
16 : 90124400
cg26935333
cg06173319
cg27464877
cg02147224
cg16611967
cg26458287
cg04298253
cg05270750
cg06295223
cg08583780
cg10315962
cg01760100
cg05106699
cg16799087
cg06931637
cg00974523
GeneLoc
PromoterAssoc
CpGIsland
0.25
0.01
mRNA / METH Corr. FDR
MITF
SOX10
TRPM1
ZEB1
AXL
0.05
0.01
Gene / Regulator Corr. P
Type
mRNA_Expr
CopyNum
AmpDel

IL16

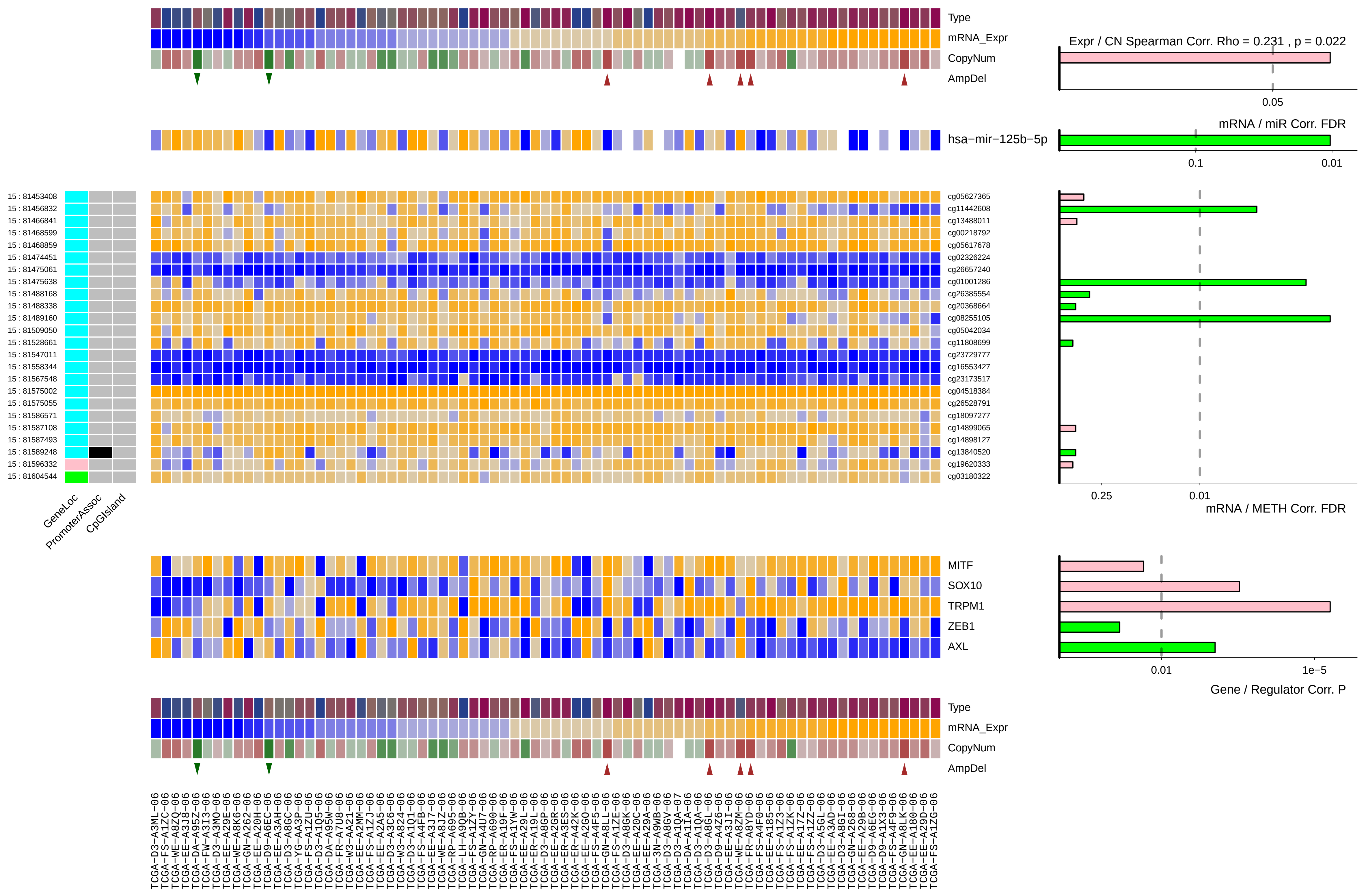

Type
mRNA_Expr
CopyNum
AmpDel
Expr / CN Spearman Corr. Rho = 0.231 , p = 0.022
0.05
hsa−mir−125b−5p
mRNA / miR Corr. FDR
0.1
0.01
15 : 81453408
15 : 81456832
15 : 81466841
15 : 81468599
15 : 81468859
15 : 81474451
15 : 81475061
15 : 81475638
15 : 81488168
15 : 81488338
15 : 81489160
15 : 81509050
15 : 81528661
15 : 81547011
15 : 81558344
15 : 81567548
15 : 81575002
15 : 81575055
15 : 81586571
15 : 81587108
15 : 81587493
15 : 81589248
15 : 81596332
15 : 81604544
GeneLoc
PromoterAssoc
CpGIsland
cg05627365
cg11442608
cg13488011
cg00218792
cg05617678
cg02326224
cg26657240
cg01001286
cg26385554
cg20368664
cg08255105
cg05042034
cg11808699
cg23729777
cg16553427
cg23173517
cg04518384
cg26528791
cg18097277
cg14899065
cg14898127
cg13840520
cg19620333
cg03180322
0.25
0.01
mRNA / METH Corr. FDR
MITF
SOX10
TRPM1
ZEB1
AXL
0.01
1e−5
Gene / Regulator Corr. P
Type
mRNA_Expr
CopyNum
AmpDel

FMN1

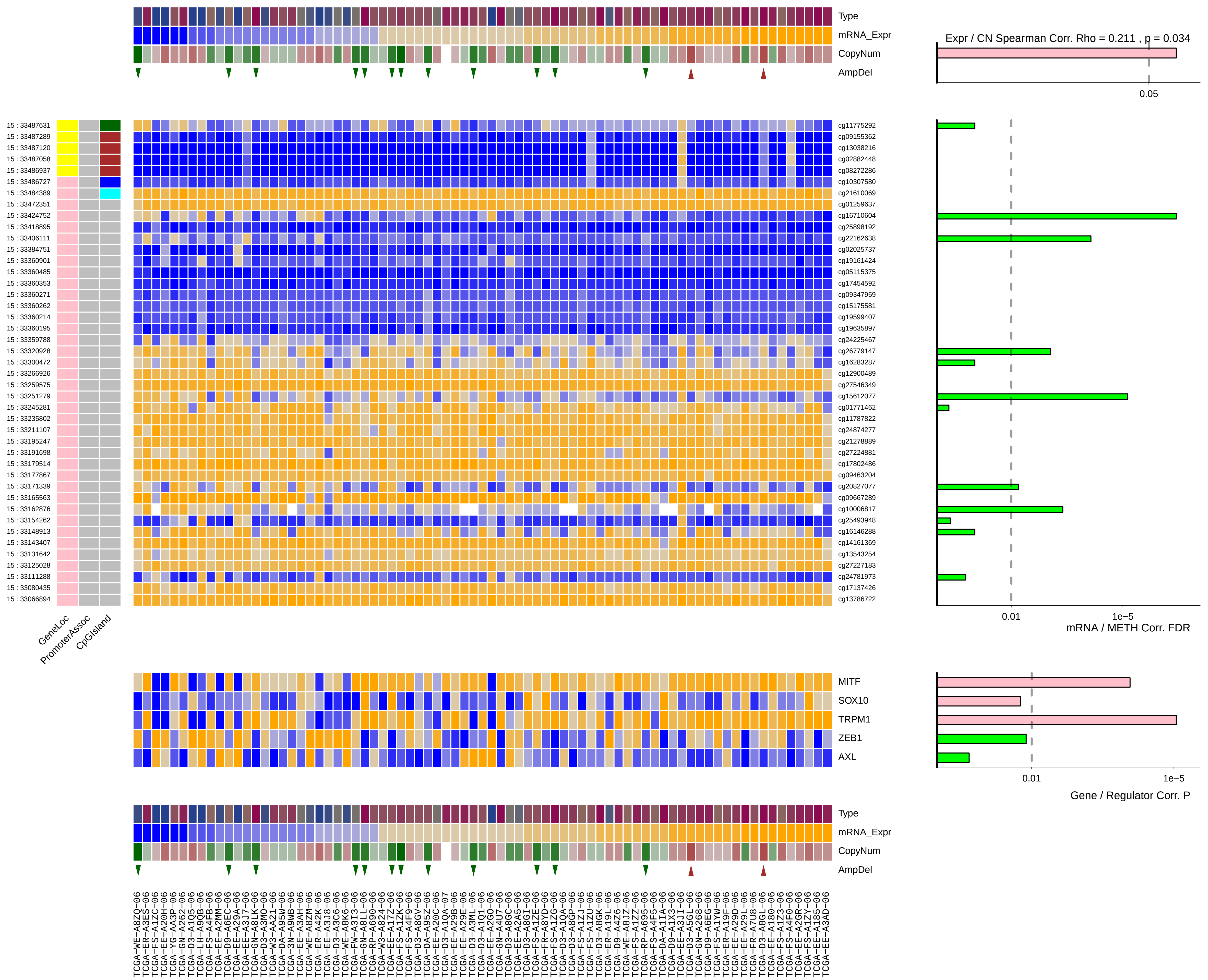

Type
mRNA_Expr
CopyNum
AmpDel
Expr / CN Spearman Corr. Rho = 0.211 , p = 0.034
0.05
GeneLoc
PromoterAssoc
CpGIsland
mRNA / METH Corr. FDR
0.01
1e−5
MITF
SOX10
TRPM1
ZEB1
AXL
Gene / Regulator Corr. P

P2RX7

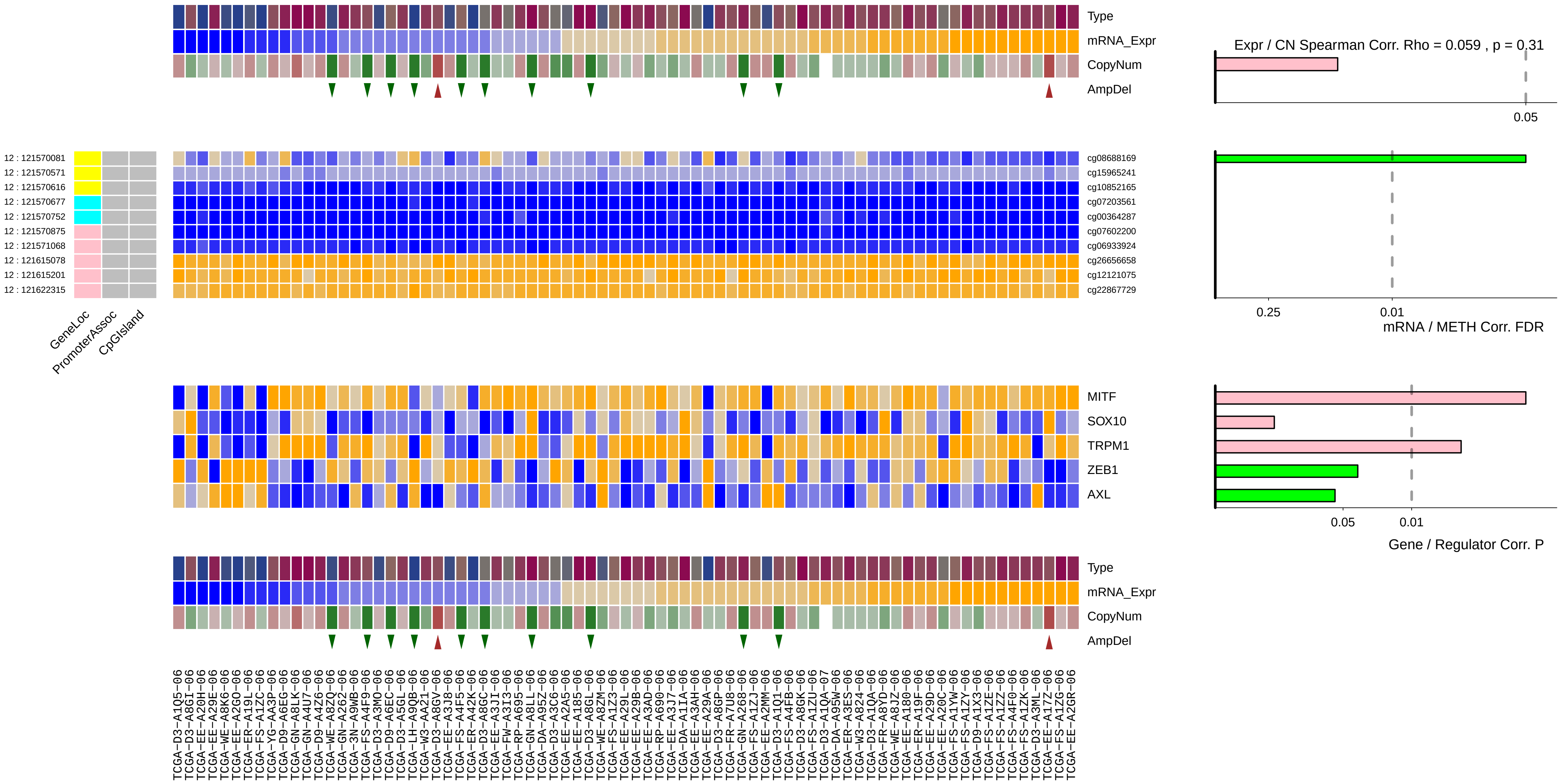

Type
mRNA_Expr
CopyNum
AmpDel
Expr / CN Spearman Corr. Rho = 0.059 , p = 0.31
0.05
12 : 121570081
12 : 121570571
12 : 121570616
12 : 121570677
12 : 121570752
12 : 121570875
12 : 121571068
12 : 121615078
12 : 121615201
12 : 121622315
cg08688169
cg15965241
cg10852165
cg07203561
cg00364287
cg07602200
cg06933924
cg26656658
cg12121075
cg22867729
0.25
0.01
mRNA / METH Corr. FDR
GeneLoc
PromoterAssoc
CpGIsland
MITF
SOX10
TRPM1
ZEB1
AXL
0.05
0.01
Gene / Regulator Corr. P
Type
mRNA_Expr
CopyNum
AmpDel

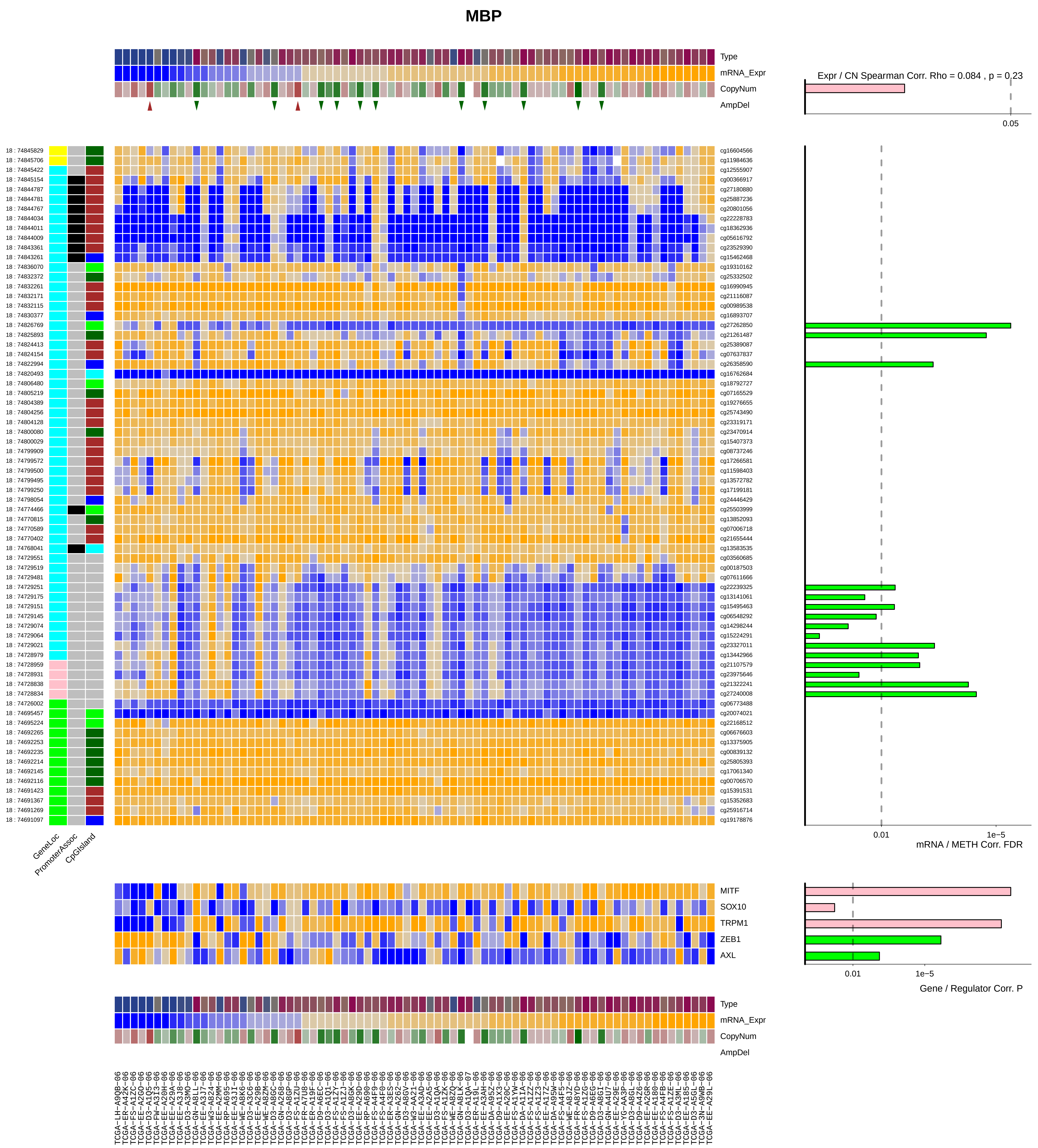
MBP
Type
mRNA_Expr
CopyNum
AmpDel
Expr / CN Spearman Corr. Rho = 0.084 , p = 0.23
0.05
18 : 74845829 cg16604566
18 : 74845706 cg11984636
18 : 74845422 cg12555907
18 : 74845154 cg00366917
18 : 74844787 cg27180880
18 : 74844781 cg25887236
18 : 74844767 cg20801056
18 : 74844034 cg22228783
18 : 74844011 cg18362936
18 : 74844009 cg05616792
18 : 74843361 cg23529390
18 : 74843261 cg15462468
18 : 74836070 cg19310162
18 : 74832372 cg25332502
18 : 74832261 cg16990945
18 : 74832171 cg21116087
18 : 74832115 cg00989538
18 : 74830377 cg16893707
18 : 74826769 cg27262850
18 : 74825893 cg21261487
18 : 74824413 cg25389087
18 : 74824154 cg07637837
18 : 74822994 cg26358590
18 : 74820493 cg16762684
18 : 74806480 cg18792727
18 : 74805219 cg07165529
18 : 74804389 cg19276655
18 : 74804256 cg25743490
18 : 74804128 cg23319171
18 : 74800080 cg23470914
18 : 74800029 cg15407373
18 : 74799909 cg08737246
18 : 74799572 cg17266581
18 : 74799500 cg11598403
18 : 74799495 cg13572782
18 : 74799250 cg17199181
18 : 74798054 cg24446429
18 : 74774466 cg25503999
18 : 74770815 cg13852093
18 : 74770589 cg07006718
18 : 74770402 cg21655444
18 : 74768041 cg13583535
18 : 74729551 cg03560685
18 : 74729519 cg00187503
18 : 74729481 cg07611666
18 : 74729251 cg22239325
18 : 74729175 cg13141061
18 : 74729151 cg15495463
18 : 74729145 cg06548292
18 : 74729074 cg14298244
18 : 74729064 cg15224291
18 : 74729021 cg23327011
18 : 74728979 cg13442966
18 : 74728959 cg21107579
18 : 74728931 cg23975646
18 : 74728838 cg21322241
18 : 74728834 cg27240008
18 : 74726002 cg06773488
18 : 74695457 cg20074021
18 : 74695224 cg22168512
18 : 74692265 cg06676603
18 : 74692253 cg13375905
18 : 74692235 cg00839132
18 : 74692214 cg25805393
18 : 74692145 cg17061340
18 : 74692116 cg00706570
18 : 74691423 cg15391531
18 : 74691367 cg15352683
18 : 74691269 cg25916714
18 : 74691097 cg19178876
GeneLoc
PromoterAssoc
CpGIsland
0.01
1e−5
mRNA / METH Corr. FDR
MITF
SOX10
TRPM1
ZEB1
AXL
0.01
1e−5
Gene / Regulator Corr. P
Type
mRNA_Expr
CopyNum
AmpDel

IVNS1ABP

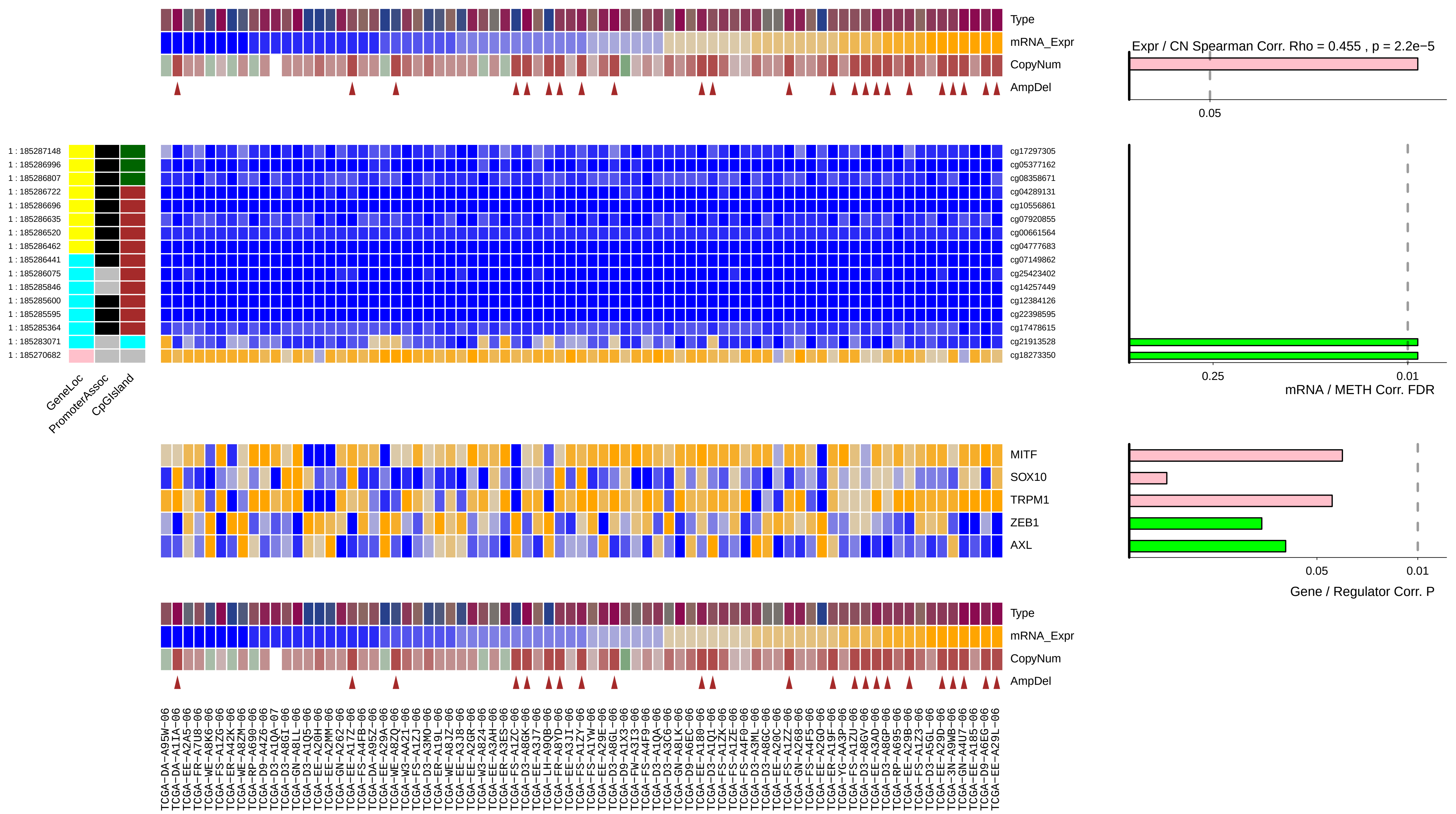

Type
mRNA_Expr
CopyNum
AmpDel
Expr / CN Spearman Corr. Rho = 0.455 , p = 2.2e−5
0.05
1 : 185287148
1 : 185286996
1 : 185286807
1 : 185286722
1 : 185286696
1 : 185286635
1 : 185286520
1 : 185286462
1 : 185286441
1 : 185286075
1 : 185285846
1 : 185285600
1 : 185285595
1 : 185285364
1 : 185283071
1 : 185270682
GeneLoc
PromoterAssoc
CpGIsland
cg17297305
cg05377162
cg08358671
cg04289131
cg10556861
cg07920855
cg00661564
cg04777683
cg07149862
cg25423402
cg14257449
cg12384126
cg22398595
cg17478615
cg21913528
cg18273350
0.25
0.01
mRNA / METH Corr. FDR
MITF
SOX10
TRPM1
ZEB1
AXL
0.05
0.01
Gene / Regulator Corr. P
Type
mRNA_Expr
CopyNum
AmpDel

SLC16A6

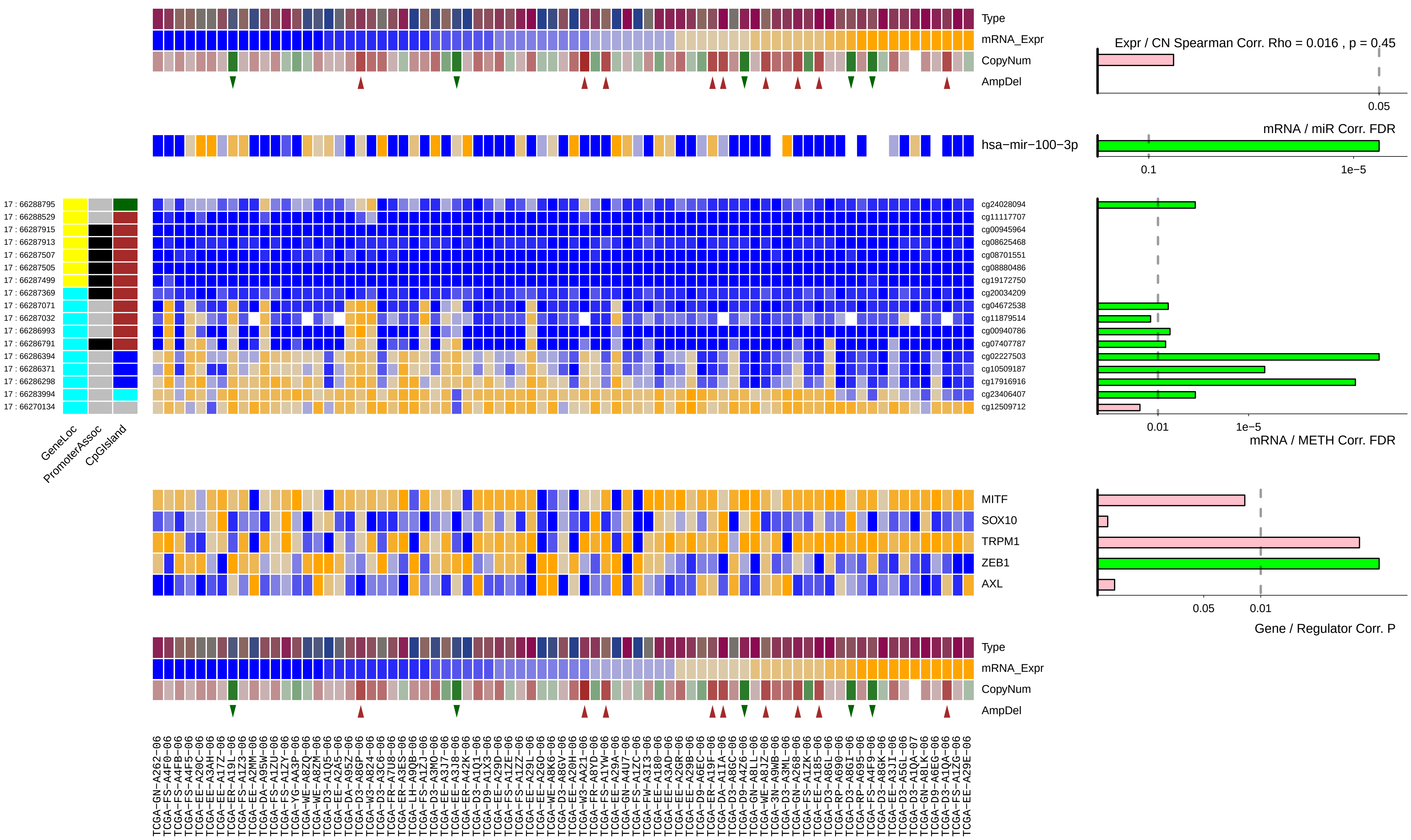

Type
mRNA_Expr
CopyNum
AmpDel
Expr / CN Spearman Corr. Rho = 0.016 , p = 0.45
0.05
hsa−mir−100−3p
mRNA / miR Corr. FDR
0.1
1e−5
17 : 66288795
17 : 66288529
17 : 66287915
17 : 66287913
17 : 66287507
17 : 66287505
17 : 66287499
17 : 66287369
17 : 66287071
17 : 66287032
17 : 66286993
17 : 66286791
17 : 66286394
17 : 66286371
17 : 66286298
17 : 66283994
17 : 66270134
GeneLoc
PromoterAssoc
CpGIsland
cg24028094
cg11117707
cg00945964
cg08625468
cg08701551
cg08880486
cg19172750
cg20034209
cg04672538
cg11879514
cg00940786
cg07407787
cg02227503
cg10509187
cg17916916
cg23406407
cg12509712
0.01
1e−5
mRNA / METH Corr. FDR
MITF
SOX10
TRPM1
ZEB1
AXL
0.05
0.01
Gene / Regulator Corr. P
Type
mRNA_Expr
CopyNum
AmpDel

MYEF2

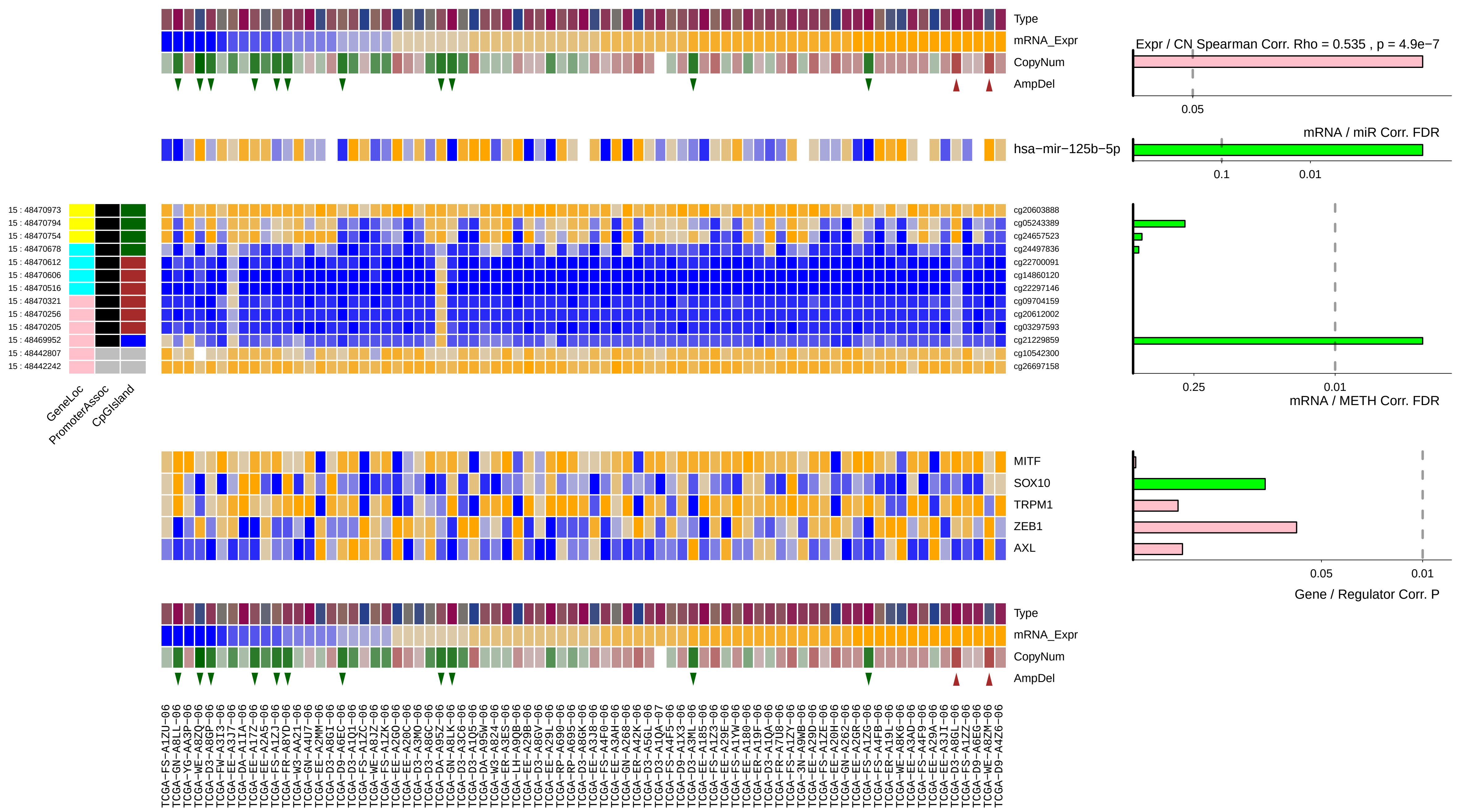

Type
mRNA_Expr
CopyNum
AmpDel
Expr / CN Spearman Corr. Rho = 0.535 , p = 4.9e−7
0.05
hsa−mir−125b−5p
mRNA / miR Corr. FDR
0.1
0.01
GeneLoc
PromoterAssoc
CpGIsland
0.25
0.01
mRNA / METH Corr. FDR
MITF
SOX10
TRPM1
ZEB1
AXL
0.05
0.01
Gene / Regulator Corr. P

CYP27A1

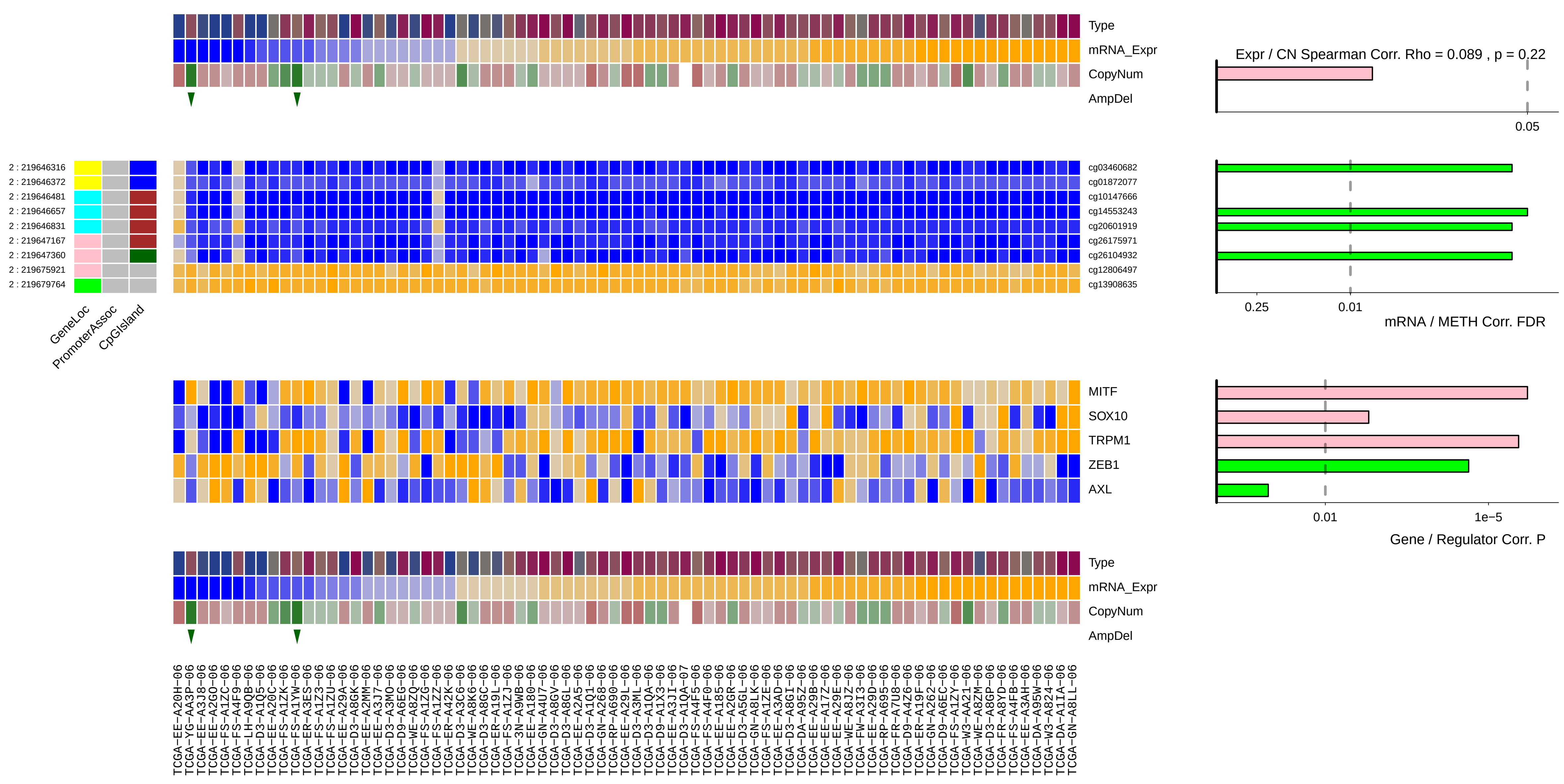
Type
mRNA_Expr
CopyNum
AmpDel
Expr / CN Spearman Corr. Rho = 0.089 , p = 0.22
0.05
2 : 219646316
2 : 219646372
2 : 219646481
2 : 219646657
2 : 219646831
2 : 219647167
2 : 219647360
2 : 219675921
2 : 219679764
GeneLoc
PromoterAssoc
CpGIsland
cg03460682
cg01872077
cg10147666
cg14553243
cg20601919
cg26175971
cg26104932
cg12806497
cg13908635
0.25
0.01
mRNA / METH Corr. FDR
MITF
SOX10
TRPM1
ZEB1
AXL
0.01
1e−5
Gene / Regulator Corr. P
Type
mRNA_Expr
CopyNum
AmpDel
TCGA-EE-A20H-06
TCGA-YG-AA3P-06
TCGA-EE-A3J8-06
TCGA-EE-A2GO-06
TCGA-FS-A1ZC-06
TCGA-FS-A4F9-06
TCGA-LH-A9QB-06
TCGA-D3-A1Q5-06
TCGA-EE-A20C-06
TCGA-FS-A1ZK-06
TCGA-FS-A1YW-06
TCGA-ER-A3ES-06
TCGA-FS-A1Z3-06
TCGA-FS-A1ZU-06
TCGA-EE-A29A-06
TCGA-D3-A8GK-06
TCGA-EE-A2MM-06
TCGA-EE-A3J7-06
TCGA-D3-A3MO-06
TCGA-D9-A6EG-06
TCGA-WE-A8ZQ-06
TCGA-FS-A1ZG-06
TCGA-FS-A1ZZ-06
TCGA-ER-A42K-06
TCGA-D3-A3C6-06
TCGA-WE-A8KC-06
TCGA-D3-A8GC-06
TCGA-ER-A19L-06
TCGA-FS-A1ZJ-06
TCGA-3N-A9WB-06
TCGA-EE-A180-06
TCGA-GN-A4U7-06
TCGA-D3-A8GV-06
TCGA-D3-A8GL-06
TCGA-EE-A2A5-06
TCGA-D3-A1Q1-06
TCGA-GN-A26B-06
TCGA-RP-A690-06
TCGA-EE-A29L-06
TCGA-D3-A3ML-06
TCGA-D3-A1QA-06
TCGA-D9-A1X3-06
TCGA-EE-A3JI-06
TCGA-D3-A1QA-07
TCGA-FS-A4F5-06
TCGA-FS-A4F0-06
TCGA-EE-A185-06
TCGA-EE-A2GP-06
TCGA-D3-A5GL-06
TCGA-GN-A8LK-06
TCGA-FS-A1ZE-06
TCGA-EE-A3AD-06
TCGA-D3-A8GI-06
TCGA-DA-A95Z-06
TCGA-EE-A29B-06
TCGA-EE-A17Z-06
TCGA-EE-A29E-06
TCGA-WE-A8JZ-06
TCGA-FW-A3I3-06
TCGA-EE-A29D-06
TCGA-RP-A695-06
TCGA-FR-A7U8-06
TCGA-D9-A4Z6-06
TCGA-ER-A19F-06
TCGA-GN-A262-06
TCGA-D9-A6EC-06
TCGA-FS-A1ZY-06
TCGA-W3-AA21-06
TCGA-WE-A8ZM-06
TCGA-D3-A8GP-06
TCGA-FR-A8YD-06
TCGA-FS-A4FB-06
TCGA-EE-A3AH-06
TCGA-DA-A95W-06
TCGA-W3-A824-06
TCGA-DA-A1IA-06
TCGA-GN-A8LL-06

SLC7A5

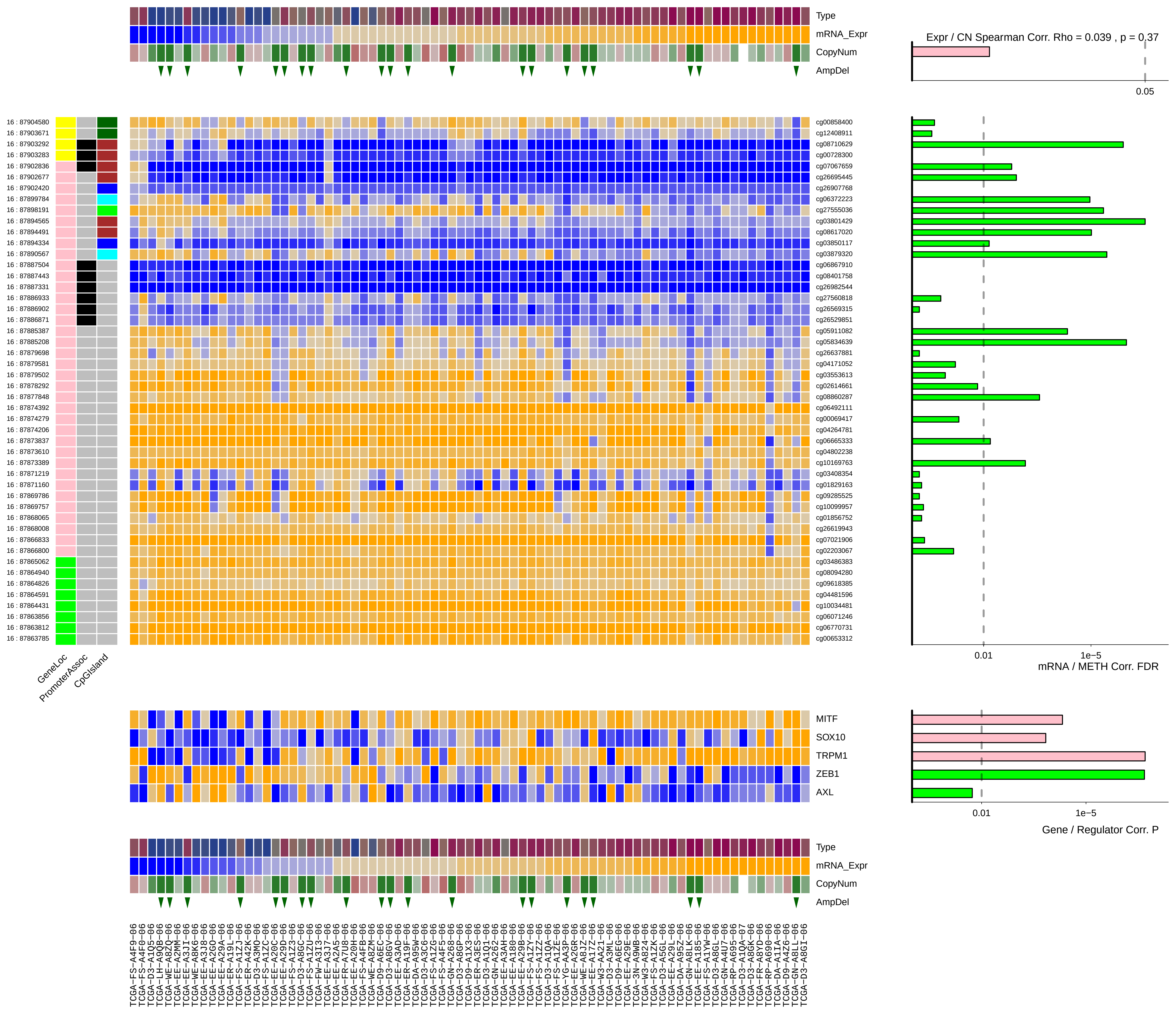

Type
mRNA_Expr
CopyNum
AmpDel
Expr / CN Spearman Corr. Rho = 0.039 , p = 0.37
0.05
GeneLoc
PromoterAssoc
CpGIsland
0.01
1e−5
mRNA / METH Corr. FDR
MITF
SOX10
TRPM1
ZEB1
AXL
Gene / Regulator Corr. P

PLXNC1

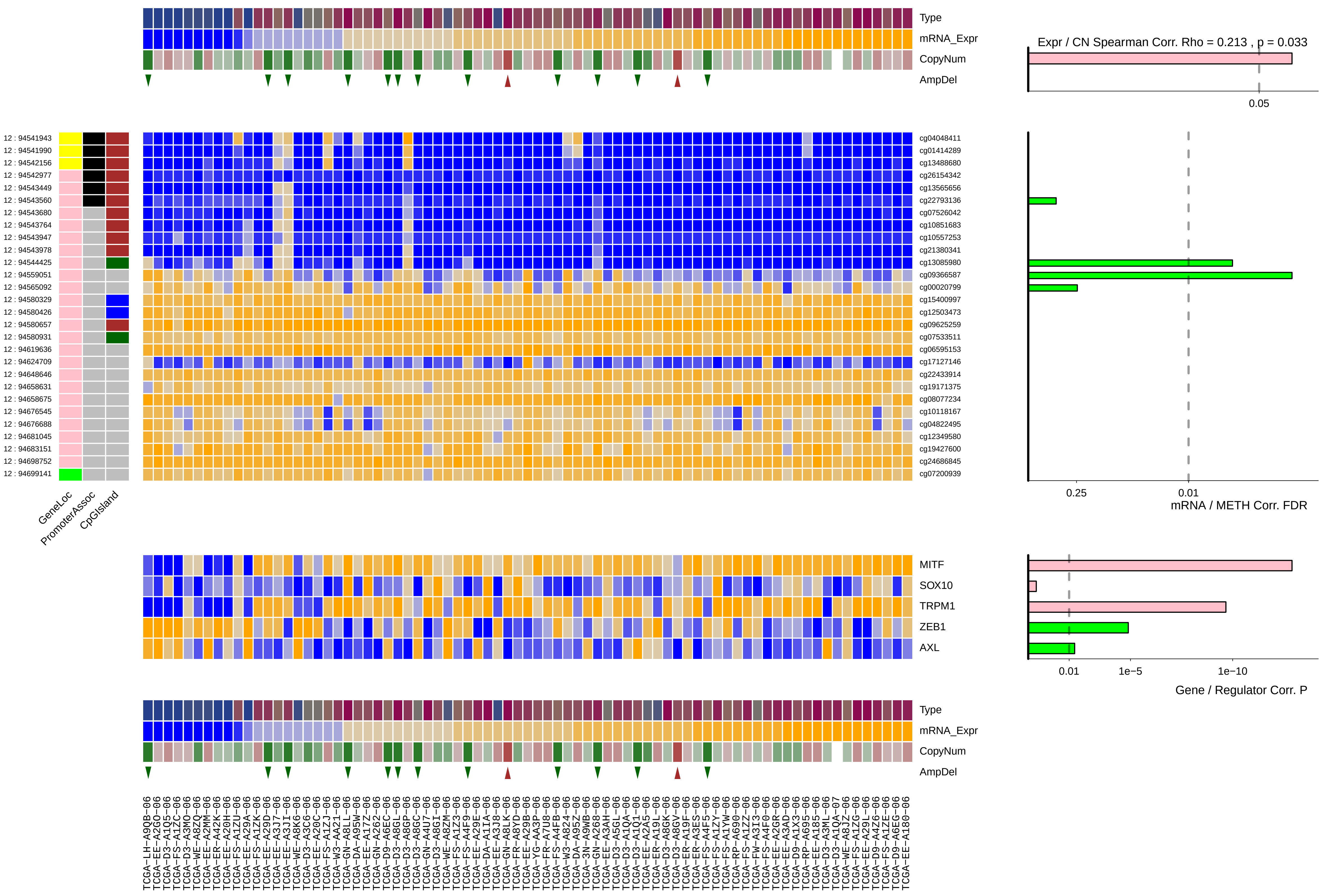

Type
mRNA_Expr
CopyNum
AmpDel
Expr / CN Spearman Corr. Rho = 0.213 , p = 0.033
0.05
12 : 94541943
12 : 94541990
12 : 94542156
12 : 94542977
12 : 94543449
12 : 94543560
12 : 94543680
12 : 94543764
12 : 94543947
12 : 94543978
12 : 94544425
12 : 94559051
12 : 94565092
12 : 94580329
12 : 94580426
12 : 94580657
12 : 94580931
12 : 94619636
12 : 94624709
12 : 94648646
12 : 94658631
12 : 94658675
12 : 94676545
12 : 94676688
12 : 94681045
12 : 94683151
12 : 94698752
12 : 94699141
cg04048411
cg01414289
cg13488680
cg26154342
cg13565656
cg22793136
cg07526042
cg10851683
cg10557253
cg21380341
cg13085980
cg09366587
cg00020799
cg15400997
cg12503473
cg09625259
cg07533511
cg06595153
cg17127146
cg22433914
cg19171375
cg08077234
cg10118167
cg04822495
cg12349580
cg19427600
cg24686845
cg07200939
0.25
0.01
mRNA / METH Corr. FDR
GeneLoc
PromoterAssoc
CpGIsland
MITF
SOX10
TRPM1
ZEB1
AXL
0.01
1e−5
1e−10
Gene / Regulator Corr. P
Type
mRNA_Expr
CopyNum
AmpDel

BCL2A1

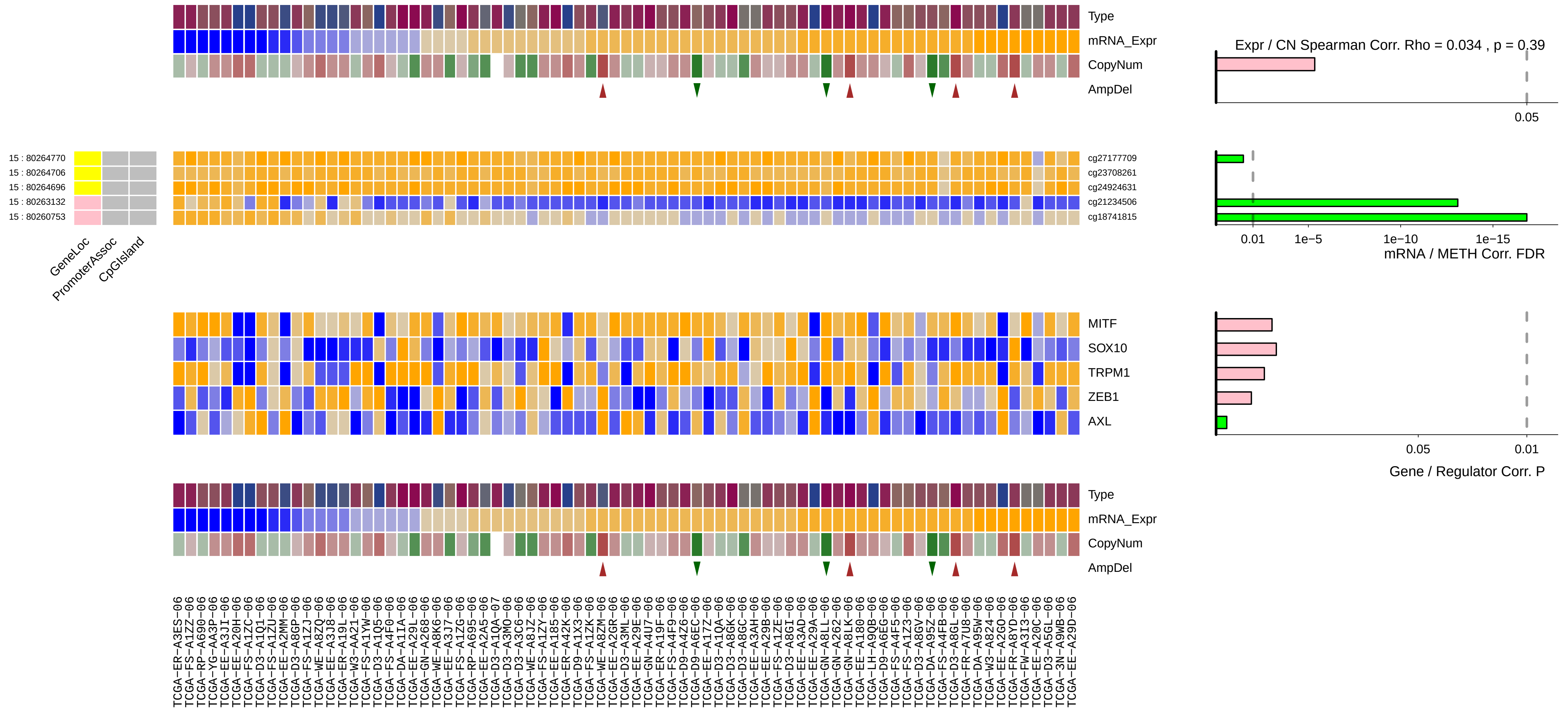

Type
mRNA_Expr
CopyNum
AmpDel
Expr / CN Spearman Corr. Rho = 0.034 , p = 0.39
0.05
15 : 80264770
15 : 80264706
15 : 80264696
15 : 80263132
15 : 80260753
cg27177709
cg23708261
cg24924631
cg21234506
cg18741815
GeneLoc
PromoterAssoc
CpGIsland
0.01
1e−5
1e−10
1e−15
mRNA / METH Corr. FDR
MITF
SOX10
TRPM1
ZEB1
AXL
0.05
0.01
Gene / Regulator Corr. P

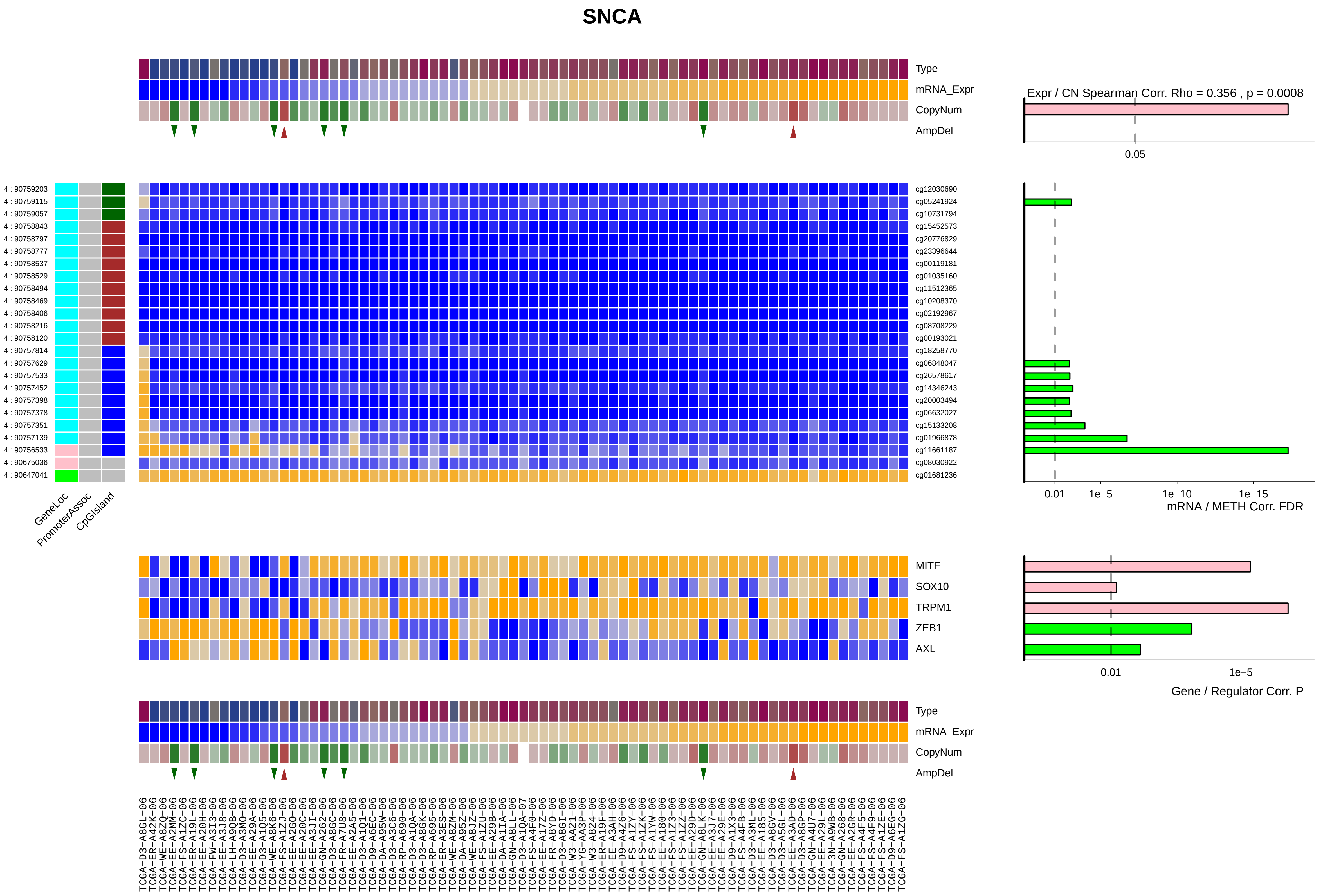
SNCA
Type
mRNA_Expr
CopyNum
AmpDel
Expr / CN Spearman Corr. Rho = 0.356 , p = 0.0008
0.05
4 : 90759203
4 : 90759115
4 : 90759057
4 : 90758843
4 : 90758797
4 : 90758777
4 : 90758537
4 : 90758529
4 : 90758494
4 : 90758469
4 : 90758406
4 : 90758216
4 : 90758120
4 : 90757814
4 : 90757629
4 : 90757533
4 : 90757452
4 : 90757398
4 : 90757378
4 : 90757351
4 : 90757139
4 : 90756533
4 : 90675036
4 : 90647041
cg12030690
cg05241924
cg10731794
cg15452573
cg20776829
cg23396644
cg00119181
cg01035160
cg11512365
cg10208370
cg02192967
cg08708229
cg00193021
cg18258770
cg06848047
cg26578617
cg14346243
cg20003494
cg06632027
cg15133208
cg01966878
cg11661187
cg08030922
cg01681236
GeneLoc
PromoterAssoc
CpGIsland
0.01
1e−5
1e−10
1e−15
mRNA / METH Corr. FDR
MITF
SOX10
TRPM1
ZEB1
AXL
0.01
1e−5
Gene / Regulator Corr. P
Type
mRNA_Expr
CopyNum
AmpDel
TCGA-D3-A8GL-06
TCGA-ER-A42K-06
TCGA-WE-A8ZQ-06
TCGA-EE-A2MM-06
TCGA-FS-A1ZC-06
TCGA-ER-A19L-06
TCGA-EE-A20H-06
TCGA-FW-A3I3-06
TCGA-EE-A3J8-06
TCGA-LH-A9QB-06
TCGA-D3-A3MO-06
TCGA-EE-A29A-06
TCGA-D3-A1Q5-06
TCGA-WE-A8K6-06
TCGA-FS-A1ZJ-06
TCGA-EE-A2GO-06
TCGA-EE-A20C-06
TCGA-EE-A3JI-06
TCGA-GN-A262-06
TCGA-D3-A8GC-06
TCGA-FR-A7U8-06
TCGA-EE-A2A5-06
TCGA-D3-A1Q1-06
TCGA-D9-A6EC-06
TCGA-DA-A95W-06
TCGA-D3-A3C6-06
TCGA-RP-A690-06
TCGA-D3-A1QA-06
TCGA-D3-A8GK-06
TCGA-RP-A695-06
TCGA-ER-A3ES-06
TCGA-WE-A8ZM-06
TCGA-DA-A95Z-06
TCGA-WE-A8JZ-06
TCGA-FS-A1ZU-06
TCGA-EE-A29B-06
TCGA-DA-A1IA-06
TCGA-GN-A8LL-06
TCGA-D3-A1QA-07
TCGA-FS-A4F0-06
TCGA-EE-A17Z-06
TCGA-FR-A8YD-06
TCGA-D3-A8GI-06
TCGA-W3-AA21-06
TCGA-YG-AA3P-06
TCGA-W3-A824-06
TCGA-ER-A19F-06
TCGA-EE-A3AH-06
TCGA-D9-A4Z6-06
TCGA-FS-A1ZY-06
TCGA-FS-A1ZK-06
TCGA-FS-A1YW-06
TCGA-EE-A180-06
TCGA-FS-A1Z3-06
TCGA-FS-A1ZZ-06
TCGA-EE-A29D-06
TCGA-GN-A8LK-06
TCGA-EE-A3J7-06
TCGA-EE-A29E-06
TCGA-D9-A1X3-06
TCGA-FS-A4FB-06
TCGA-D3-A3ML-06
TCGA-EE-A185-06
TCGA-D3-A8GV-06
TCGA-D3-A5GL-06
TCGA-EE-A3AD-06
TCGA-D3-A8GP-06
TCGA-GN-A4U7-06
TCGA-EE-A29L-06
TCGA-3N-A9WB-06
TCGA-GN-A268-06
TCGA-EE-A2GR-06
TCGA-FS-A4F5-06
TCGA-FS-A4F9-06
TCGA-FS-A1ZE-06
TCGA-D9-A6EG-06
TCGA-FS-A1ZG-06

BIRC7

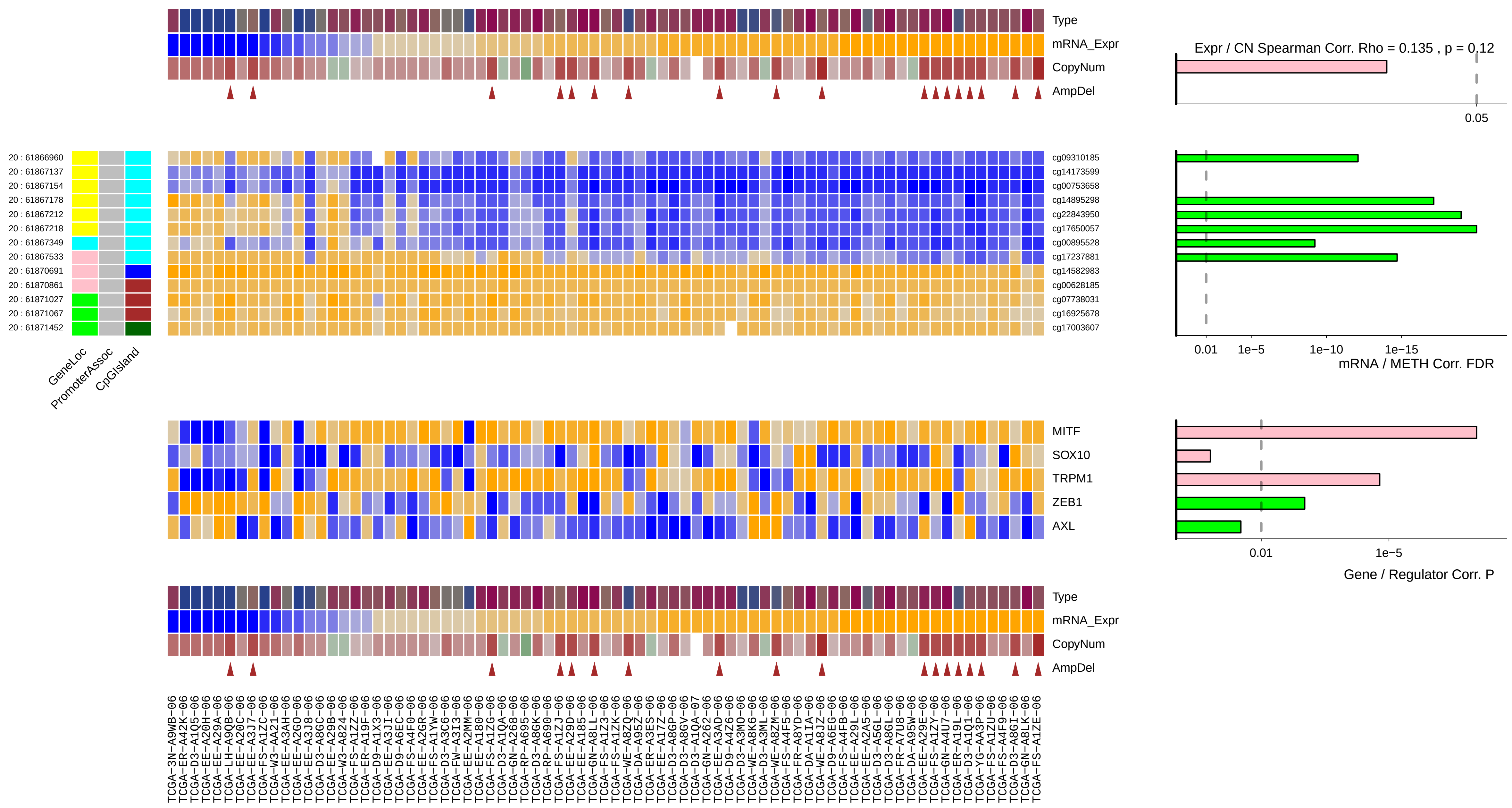

Type
mRNA_Expr
CopyNum
AmpDel
Expr / CN Spearman Corr. Rho = 0.135 , p = 0.12
0.05
20 : 61866960
20 : 61867137
20 : 61867154
20 : 61867178
20 : 61867212
20 : 61867218
20 : 61867349
20 : 61867533
20 : 61870691
20 : 61870861
20 : 61871027
20 : 61871067
20 : 61871452
GeneLoc
PromoterAssoc
CpGIsland
cg09310185
cg14173599
cg00753658
cg14895298
cg22843950
cg17650057
cg00895528
cg17237881
cg14582983
cg00628185
cg07738031
cg16925678
cg17003607
0.01
1e−5
1e−10
1e−15
mRNA / METH Corr. FDR
MITF
SOX10
TRPM1
ZEB1
AXL
0.01
1e−5
Gene / Regulator Corr. P
Type
mRNA_Expr
CopyNum
AmpDel

MFSD12

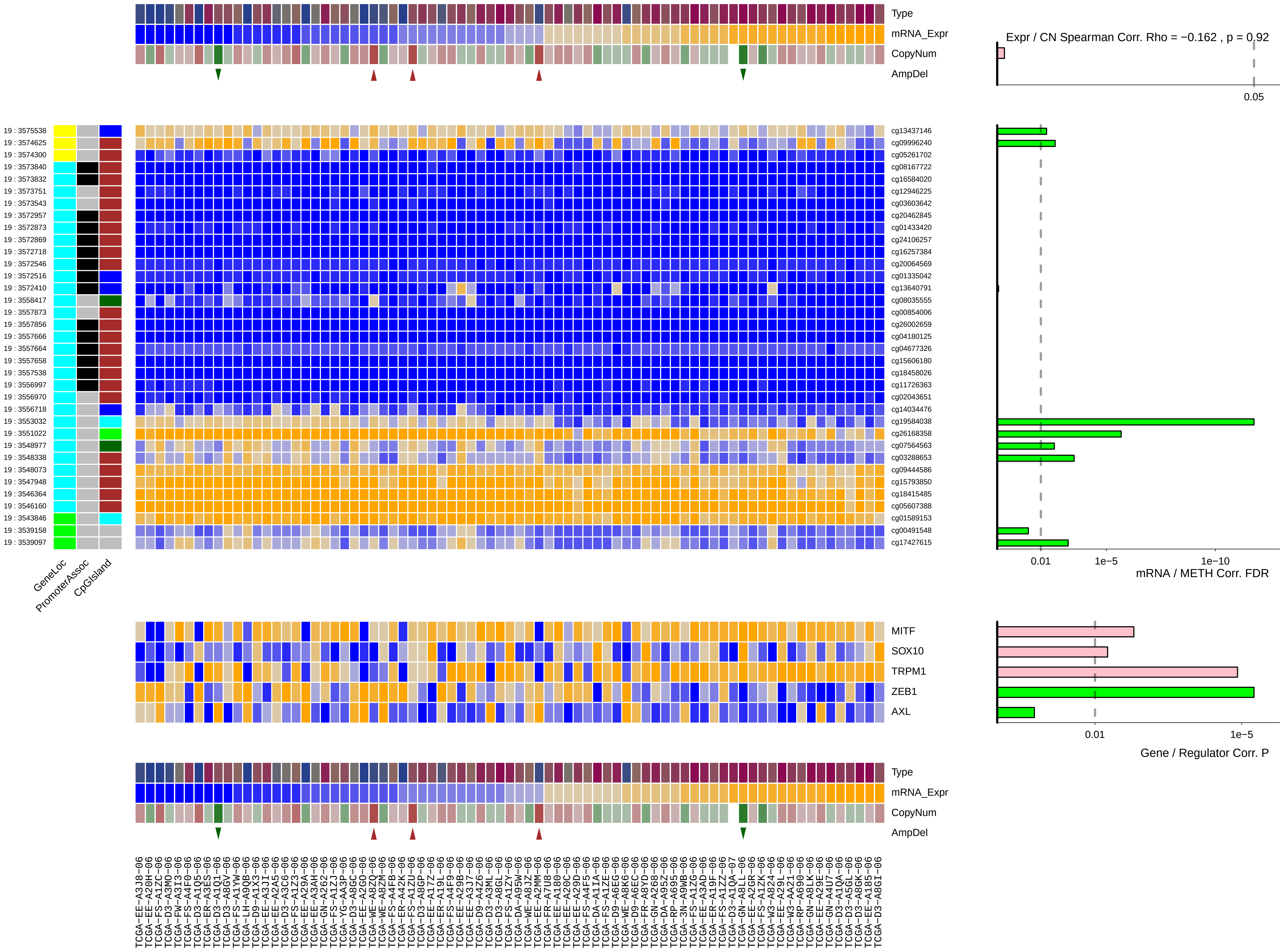
Type
mRNA_Expr
CopyNum
AmpDel
Expr / CN Spearman Corr. Rho = −0.162 , p = 0.92
0.05
GeneLoc
PromoterAssoc
CpGIsland
0.01
1e−5
1e−10
mRNA / METH Corr. FDR
MITF
SOX10
TRPM1
ZEB1
AXL
0.01
1e−5
Gene / Regulator Corr. P

SGK1

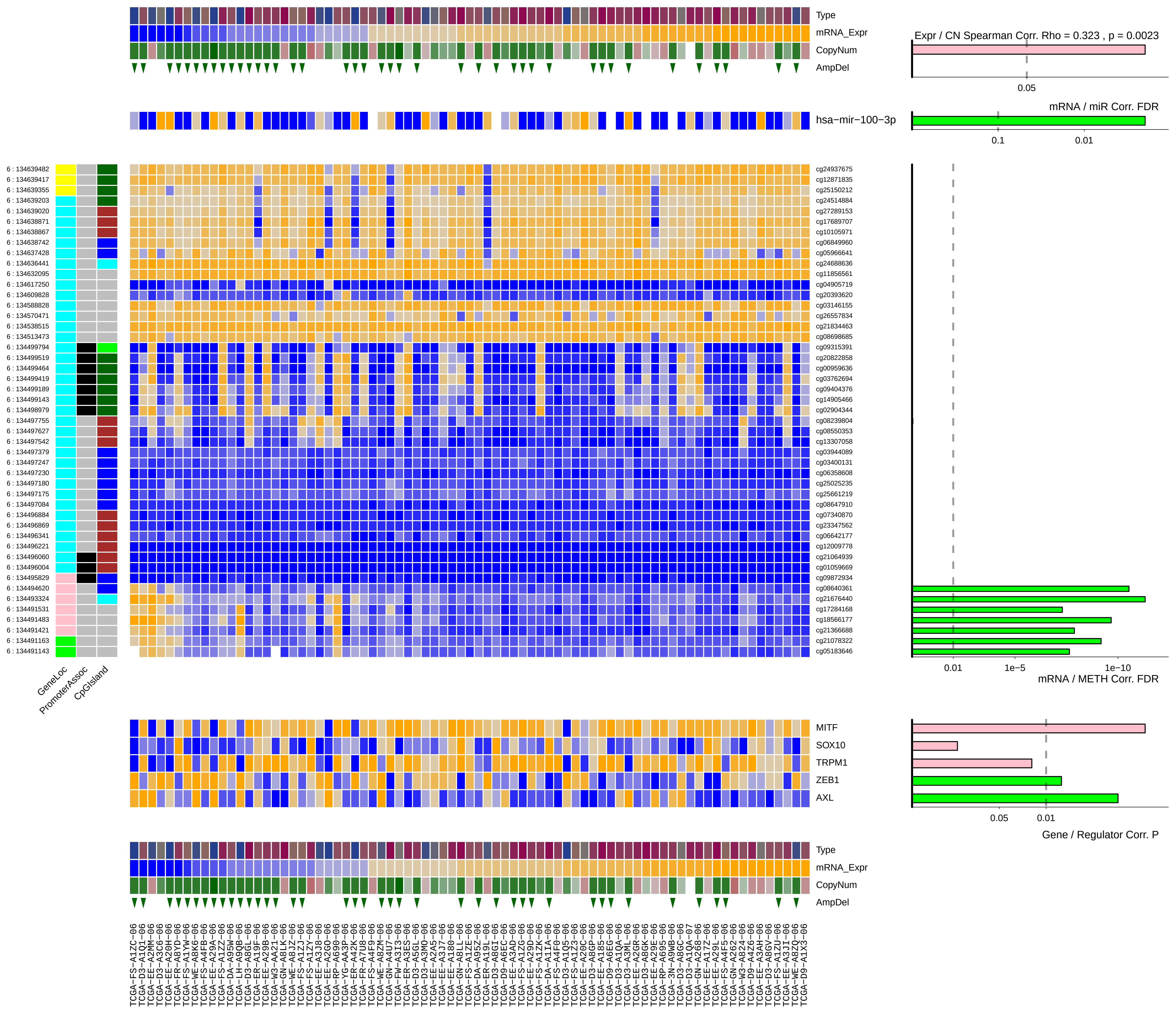

Type
mRNA_Expr
CopyNum
AmpDel
Expr / CN Spearman Corr. Rho = 0.323 , p = 0.0023
0.05
mRNA / miR Corr. FDR
hsa−mir−100−3p
0.1
0.01
cg24937675
cg12871835
cg25150212
cg24514884
cg27289153
cg17689707
cg10105971
cg06849960
cg05966641
cg24688636
cg11856561
cg04905719
cg20393620
cg03146155
cg26557834
cg21834463
cg08698685
cg09315391
cg20822858
cg00959636
cg03762694
cg09404376
cg14905466
cg02904344
cg08239804
cg08550353
cg13307058
cg03944089
cg03400131
cg06358608
cg25025235
cg25661219
cg08647910
cg07340870
cg23347562
cg06642177
cg12009778
cg21064939
cg01059669
cg09872934
cg08640361
cg21676440
cg17284168
cg18566177
cg21366688
cg21078322
cg05183646
0.01
1e−5
1e−10
mRNA / METH Corr. FDR
GeneLoc
PromoterAssoc
CpGIsland
MITF
SOX10
TRPM1
ZEB1
AXL
0.05
0.01
Gene / Regulator Corr. P

SHTN1

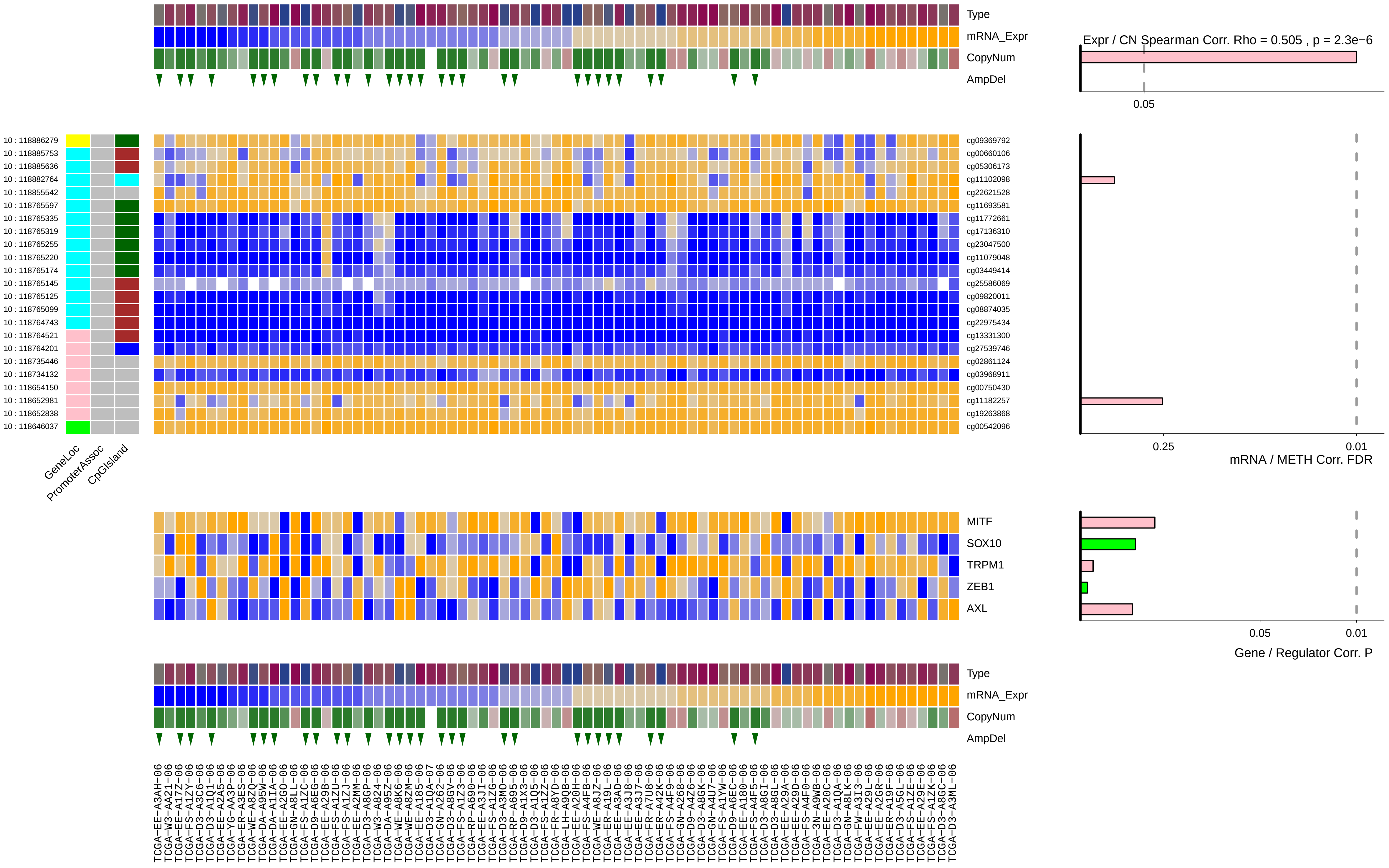
Type
mRNA_Expr
CopyNum
AmpDel
Expr / CN Spearman Corr. Rho = 0.505 , p = 2.3e−6
0.05
10 : 118886279
10 : 118885753
10 : 118885636
10 : 118882764
10 : 118855542
10 : 118765597
10 : 118765335
10 : 118765319
10 : 118765255
10 : 118765220
10 : 118765174
10 : 118765145
10 : 118765125
10 : 118765099
10 : 118764743
10 : 118764521
10 : 118764201
10 : 118735446
10 : 118734132
10 : 118654150
10 : 118652981
10 : 118652838
10 : 118646037
cg09369792
cg00660106
cg05306173
cg11102098
cg22621528
cg11693581
cg11772661
cg17136310
cg23047500
cg11079048
cg03449414
cg25586069
cg09820011
cg08874035
cg22975434
cg13331300
cg27539746
cg02861124
cg03968911
cg00750430
cg11182257
cg19263868
cg00542096
GeneLoc
PromoterAssoc
CpGIsland
0.25
0.01
mRNA / METH Corr. FDR
MITF
SOX10
TRPM1
ZEB1
AXL
0.05
0.01
Gene / Regulator Corr. P
Type
mRNA_Expr
CopyNum
AmpDel
TCGA-EE-A3AH-06
TCGA-W3-AA21-06
TCGA-EE-A17Z-06
TCGA-FS-A1Z7-06
TCGA-D3-A3C6-06
TCGA-D3-A1Q1-06
TCGA-EE-A2A5-06
TCGA-YG-AA3P-06
TCGA-ER-A3ES-06
TCGA-WE-A8ZQ-06
TCGA-DA-A95W-06
TCGA-DA-A1IA-06
TCGA-EE-A2GO-06
TCGA-GN-A8LL-06
TCGA-FS-A1ZC-06
TCGA-D9-A6EG-06
TCGA-EE-A29B-06
TCGA-FS-A1ZU-06
TCGA-FS-A1ZJ-06
TCGA-EE-A2MM-06
TCGA-D3-A8GP-06
TCGA-W3-A824-06
TCGA-DA-A95Z-06
TCGA-WE-A8K6-06
TCGA-WE-A8ZM-06
TCGA-EE-A185-06
TCGA-D3-A1QA-07
TCGA-GN-A262-06
TCGA-D3-A8GV-06
TCGA-FS-A1Z3-06
TCGA-RP-A690-06
TCGA-EE-A3JI-06
TCGA-FS-A1ZG-06
TCGA-D3-A3MO-06
TCGA-RP-A695-06
TCGA-D9-A1X3-06
TCGA-EE-A1QS-06
TCGA-FS-A1ZZ-06
TCGA-FR-A8YD-06
TCGA-LH-A9QB-06
TCGA-EE-A20H-06
TCGA-FS-A4FB-06
TCGA-WE-A8JZ-06
TCGA-ER-A19L-06
TCGA-EE-A3AD-06
TCGA-EE-A3J8-06
TCGA-EE-A3J7-06
TCGA-FR-A7U8-06
TCGA-ER-A42K-06
TCGA-FS-A4F9-06
TCGA-GN-A268-06
TCGA-D9-A4Z6-06
TCGA-D3-A8GK-06
TCGA-GN-A4U7-06
TCGA-FS-A1YW-06
TCGA-D9-A6EC-06
TCGA-EE-A180-06
TCGA-FS-A4F5-06
TCGA-D3-A8GL-06
TCGA-D3-A8GI-06
TCGA-EE-A29A-06
TCGA-EE-A29D-06
TCGA-FS-A4F0-06
TCGA-3N-A9WB-06
TCGA-EE-A20C-06
TCGA-D3-A1QA-06
TCGA-GN-A8LK-06
TCGA-FW-A3I3-06
TCGA-EE-A29L-06
TCGA-EE-A29R-06
TCGA-ER-A19P-06
TCGA-D3-A5GL-06
TCGA-FS-A1ZE-06
TCGA-EE-A29E-06
TCGA-FS-A1ZK-06
TCGA-D3-A8GC-06
TCGA-D3-A3ML-06

RNF144A

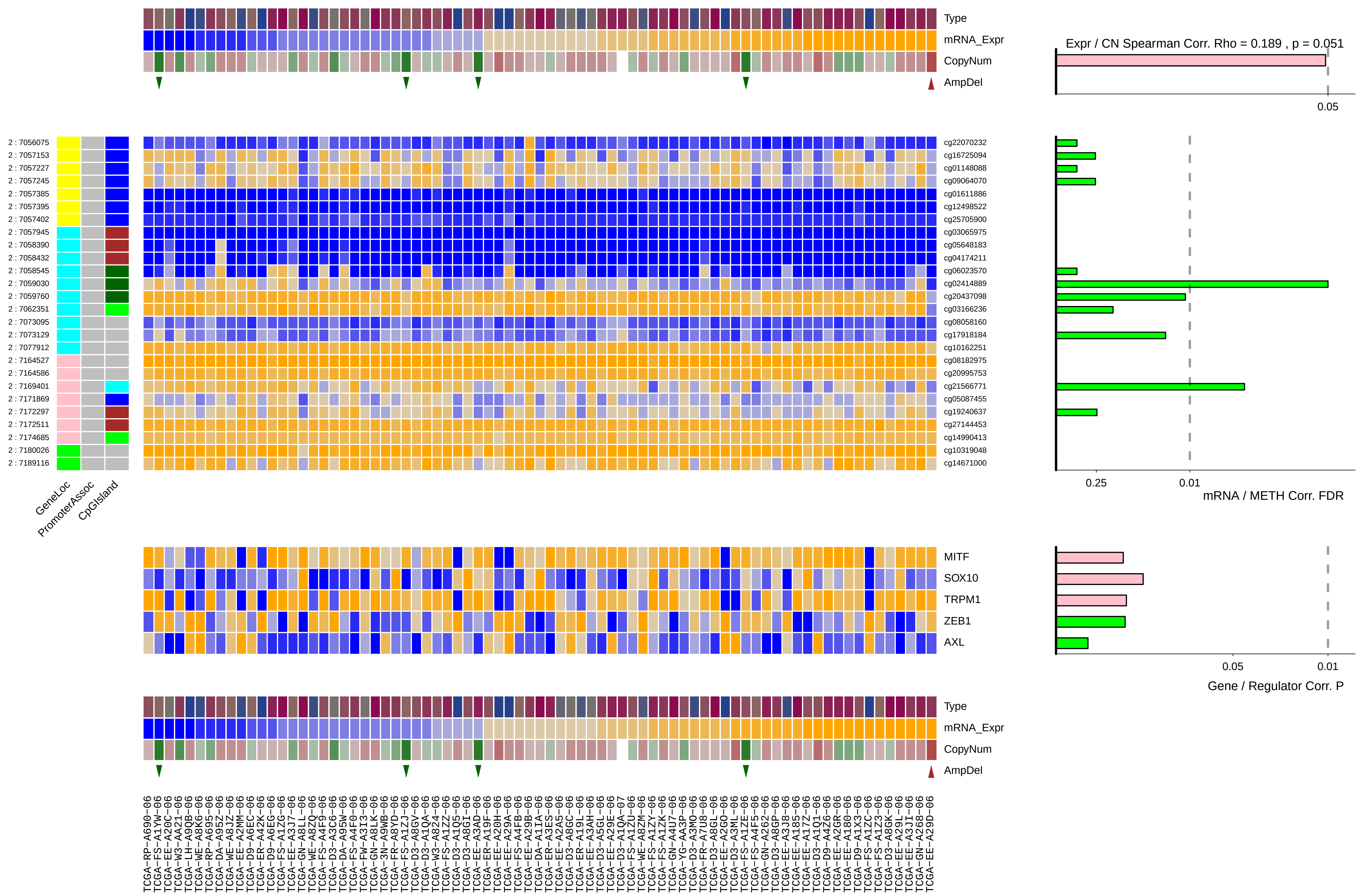
Type
mRNA_Expr
CopyNum
AmpDel
Expr / CN Spearman Corr. Rho = 0.189 , p = 0.051
0.05
mRNA / METH Corr. FDR
0.25
0.01
GeneLoc
PromoterAssoc
CpGIsland
MITF
SOX10
TRPM1
ZEB1
AXL
Gene / Regulator Corr. P
0.05
0.01

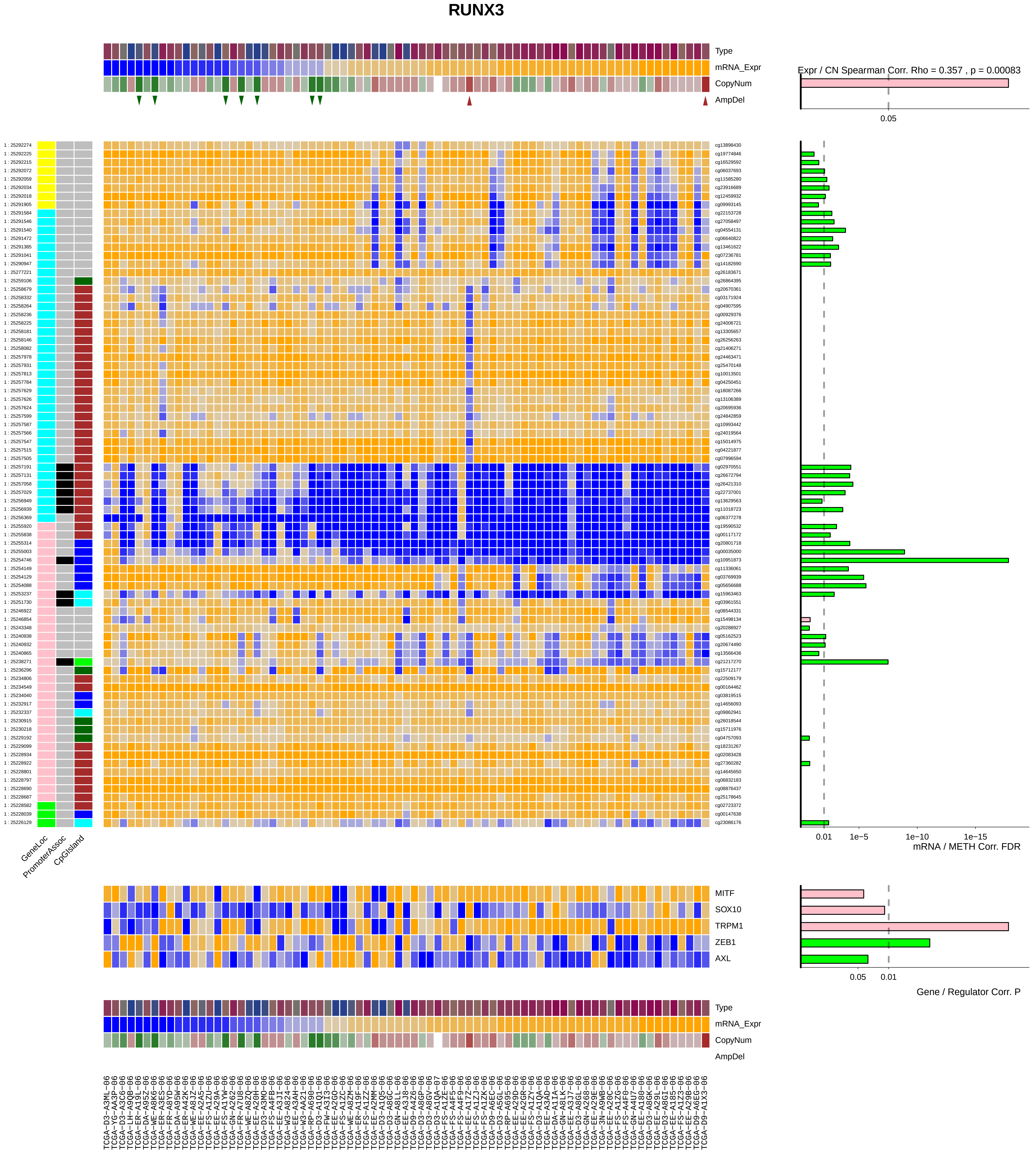

RUNX3
Type
mRNA_Expr
CopyNum
AmpDel
Expr / CN Spearman Corr. Rho = 0.357 , p = 0.00083
0.05
GeneLoc
PromoterAssoc
CpGIsland
0.01
1e−5
1e−10
1e−15
mRNA / METH Corr. FDR
MITF
SOX10
TRPM1
ZEB1
AXL
0.05
0.01
Gene / Regulator Corr. P
Type
mRNA_Expr
CopyNum
AmpDel

IGSF3

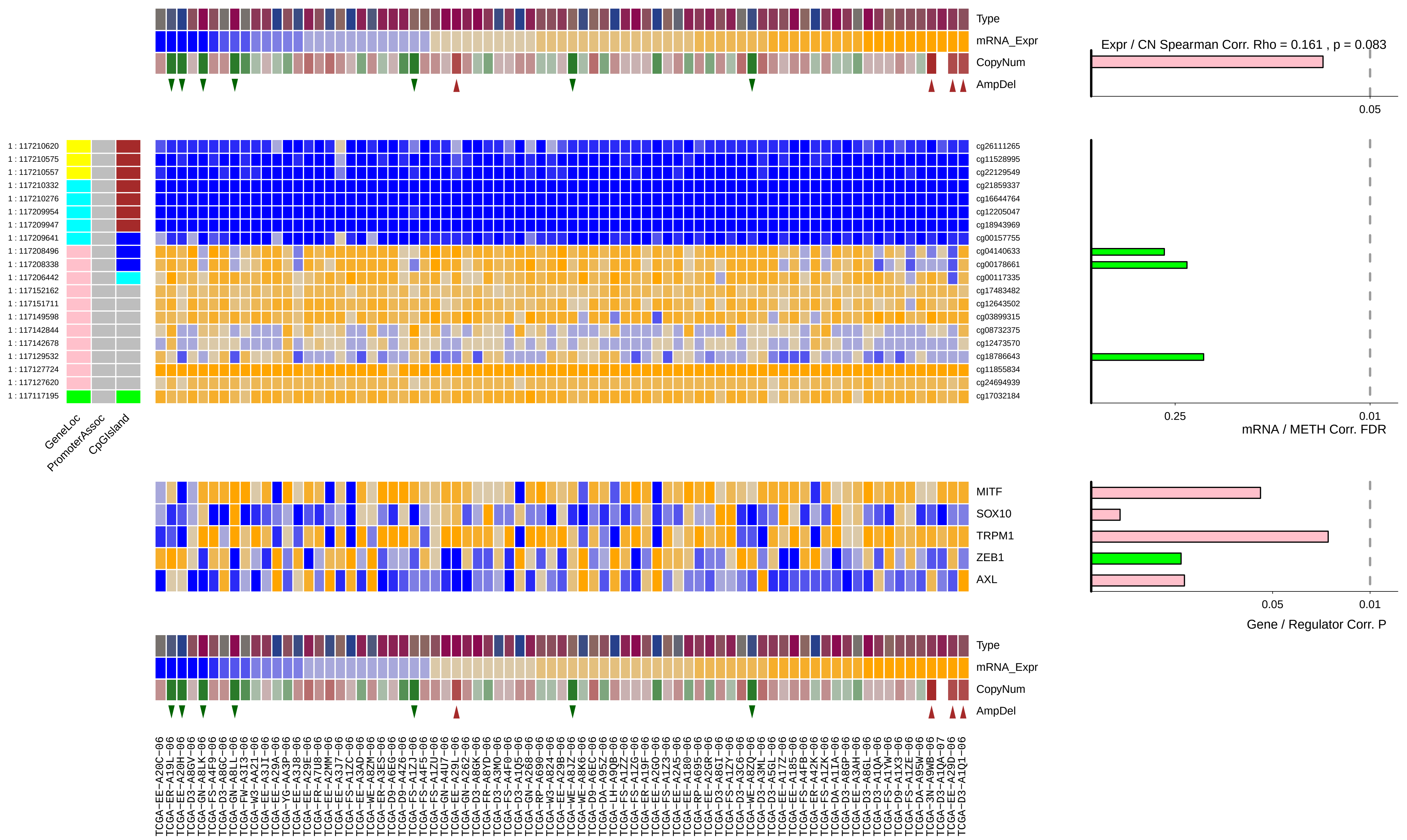

Type
mRNA_Expr
CopyNum
AmpDel
Expr / CN Spearman Corr. Rho = 0.161 , p = 0.083
0.05
1 : 117210620
1 : 117210575
1 : 117210557
1 : 117210332
1 : 117210276
1 : 117209954
1 : 117209947
1 : 117209641
1 : 117208496
1 : 117208338
1 : 117206442
1 : 117152162
1 : 117151711
1 : 117149598
1 : 117142844
1 : 117142678
1 : 117129532
1 : 117127724
1 : 117127620
1 : 117117195
cg26111265
cg11528995
cg22129549
cg21859337
cg16644764
cg12205047
cg18943969
cg00157755
cg04140633
cg00178661
cg00117335
cg17483482
cg12643502
cg03899315
cg08732375
cg12473570
cg18786643
cg11855834
cg24694939
cg17032184
GeneLoc
PromoterAssoc
CpGIsland
0.25
0.01
mRNA / METH Corr. FDR
MITF
SOX10
TRPM1
ZEB1
AXL
0.05
0.01
Gene / Regulator Corr. P
Type
mRNA_Expr
CopyNum
AmpDel

ST3GAL6

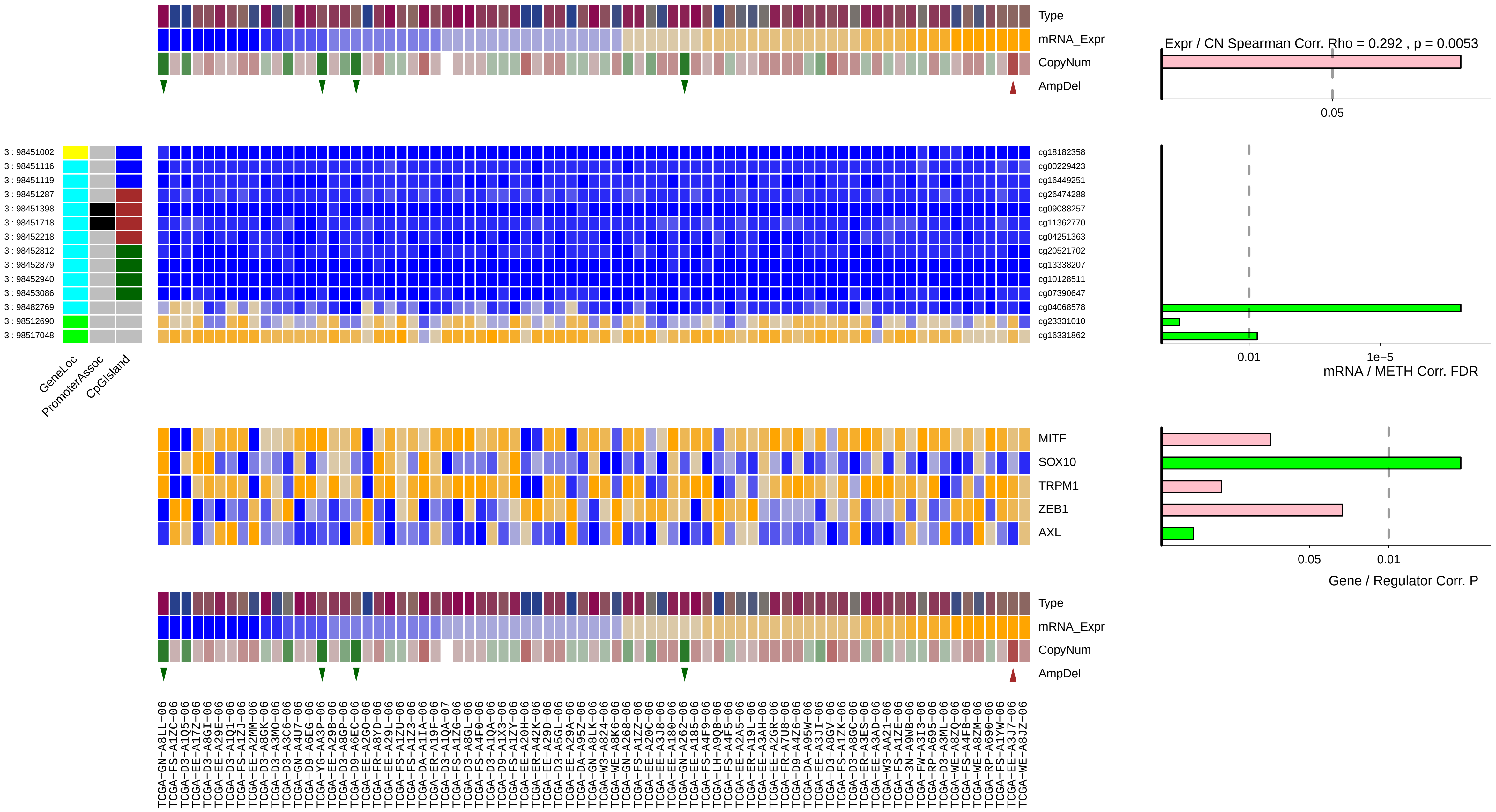

Type
mRNA_Expr
CopyNum
AmpDel
Expr / CN Spearman Corr. Rho = 0.292 , p = 0.0053
0.05
3 : 98451002
3 : 98451116
3 : 98451119
3 : 98451287
3 : 98451398
3 : 98451718
3 : 98452218
3 : 98452812
3 : 98452879
3 : 98452940
3 : 98453086
3 : 98482769
3 : 98512690
3 : 98517048
cg18182358
cg00229423
cg16449251
cg26474288
cg09088257
cg11362770
cg04251363
cg20521702
cg13338207
cg10128511
cg07390647
cg04068578
cg23331010
cg16331862
GeneLoc
PromoterAssoc
CpGIsland
0.01
1e−5
mRNA / METH Corr. FDR
MITF
SOX10
TRPM1
ZEB1
AXL
0.05
0.01
Gene / Regulator Corr. P
Type
mRNA_Expr
CopyNum
AmpDel

GAS7

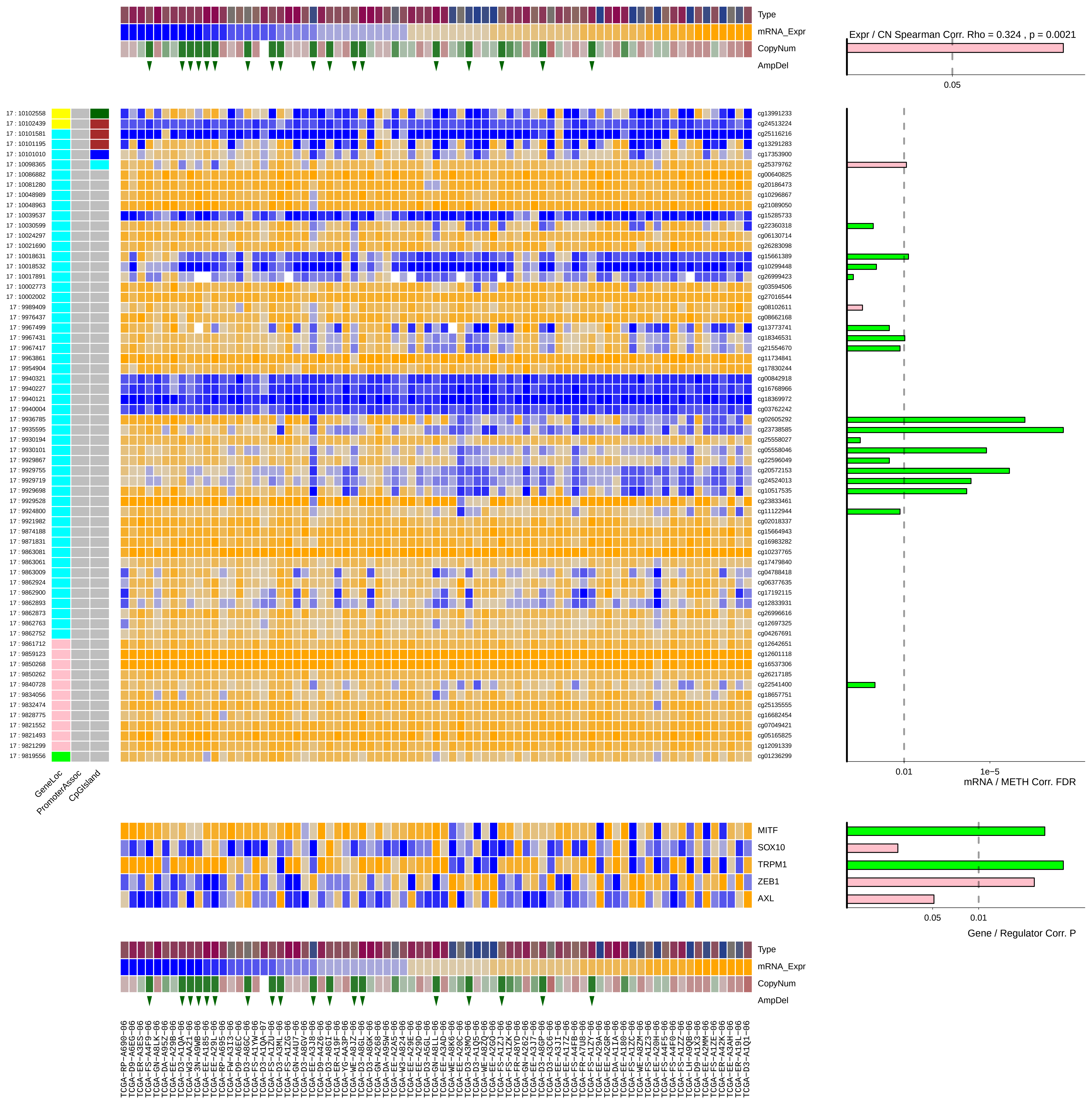
Type
mRNA_Expr
CopyNum
AmpDel
Expr / CN Spearman Corr. Rho = 0.324 , p = 0.0021
0.05
17 : 10102558 cg13991233
17 : 10102439 cg24513224
17 : 10101581 cg25116216
17 : 10101195 cg13291283
17 : 10101010 cg17353900
17 : 10098365 cg25379762
17 : 10086882 cg00640825
17 : 10081280 cg20186473
17 : 10048989 cg10296867
17 : 10048963 cg21089050
17 : 10039537 cg15285733
17 : 10030599 cg22360318
17 : 10024297 cg06130714
17 : 10021690 cg26283098
17 : 10018631 cg15661389
17 : 10018532 cg10299448
17 : 10017891 cg26999423
17 : 10002773 cg03594506
17 : 10002002 cg27016544
17 : 9989409 cg08102611
17 : 9976437 cg08662168
17 : 9967499 cg13773741
17 : 9967431 cg18346531
17 : 9967417 cg21554670
17 : 9963861 cg11734841
17 : 9954904 cg17830244
17 : 9940321 cg00842918
17 : 9940227 cg16768966
17 : 9940121 cg18369972
17 : 9940004 cg03762242
17 : 9936785 cg02605292
17 : 9935595 cg23738585
17 : 9930194 cg25558027
17 : 9930101 cg05558046
17 : 9929867 cg22596049
17 : 9929755 cg20572153
17 : 9929719 cg24524013
17 : 9929698 cg10517535
17 : 9929528 cg23833461
17 : 9924800 cg11122944
17 : 9921982 cg02018337
17 : 9874188 cg15664943
17 : 9871831 cg16983282
17 : 9863081 cg10237765
17 : 9863061 cg17479840
17 : 9863009 cg04788418
17 : 9862924 cg06377635
17 : 9862900 cg17192115
17 : 9862893 cg12833931
17 : 9862873 cg26996616
17 : 9862763 cg12697325
17 : 9862752 cg04267691
17 : 9861712 cg12642651
17 : 9859123 cg12601118
17 : 9850268 cg16537306
17 : 9850262 cg26217185
17 : 9840728 cg22541400
17 : 9834056 cg18657751
17 : 9832474 cg25135555
17 : 9828775 cg16682454
17 : 9821552 cg07049421
17 : 9821493 cg05165825
17 : 9821299 cg12091339
17 : 9819556 cg01236299
GeneLoc
PromoterAssoc
CpGIsland
0.01
1e−5
mRNA / METH Corr. FDR
MITF
SOX10
TRPM1
ZEB1
AXL
0.05
0.01
Gene / Regulator Corr. P
Type
mRNA_Expr
CopyNum
AmpDel

AP1S2

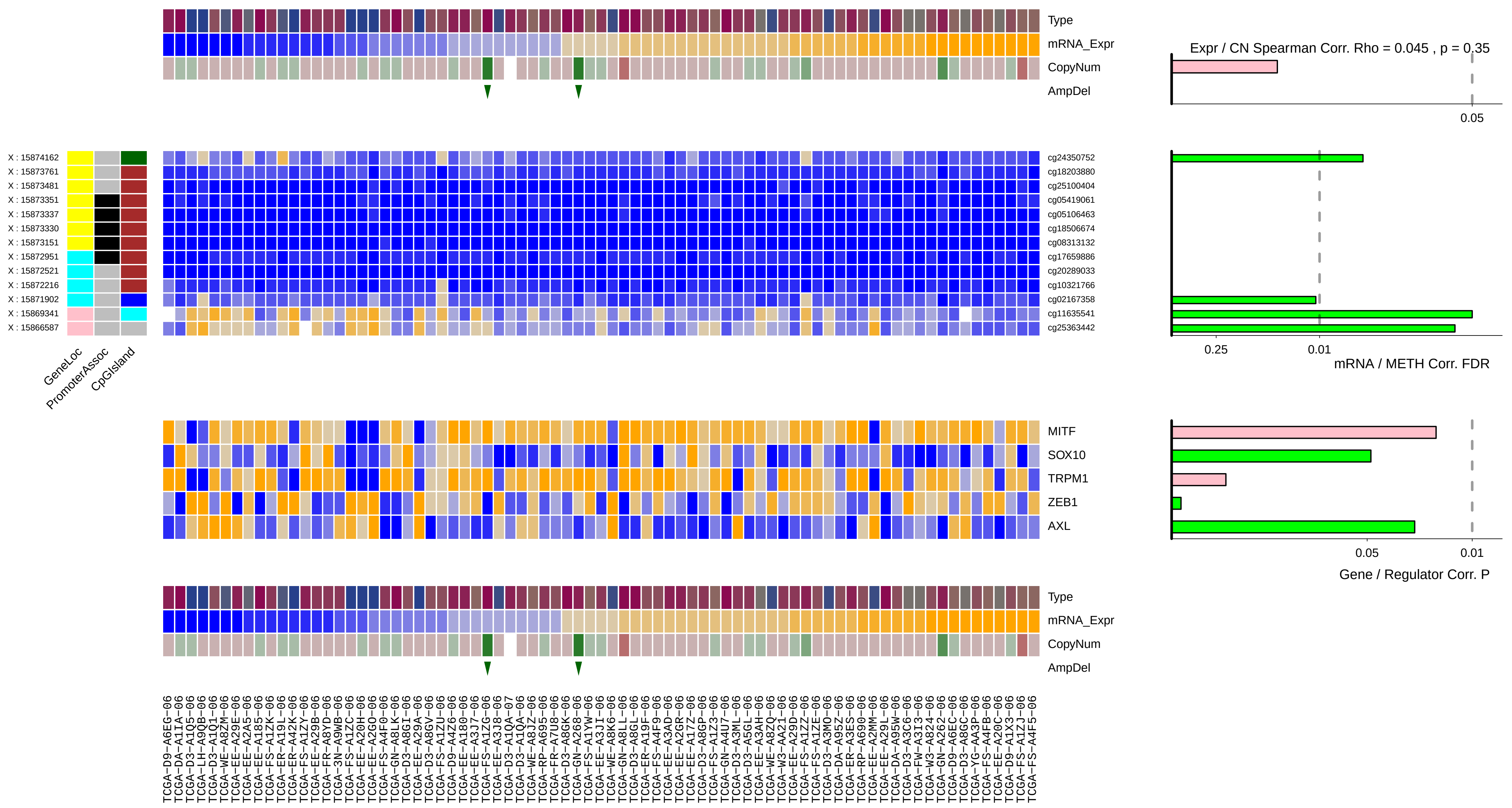
Type
mRNA_Expr
CopyNum
AmpDel
Expr / CN Spearman Corr. Rho = 0.045 , p = 0.35
0.05
mRNA / METH Corr. FDR
0.25
0.01
GeneLoc
PromoterAssoc
CpGIsland
MITF
SOX10
TRPM1
ZEB1
AXL
Gene / Regulator Corr. P
0.05
0.01

FCRLA

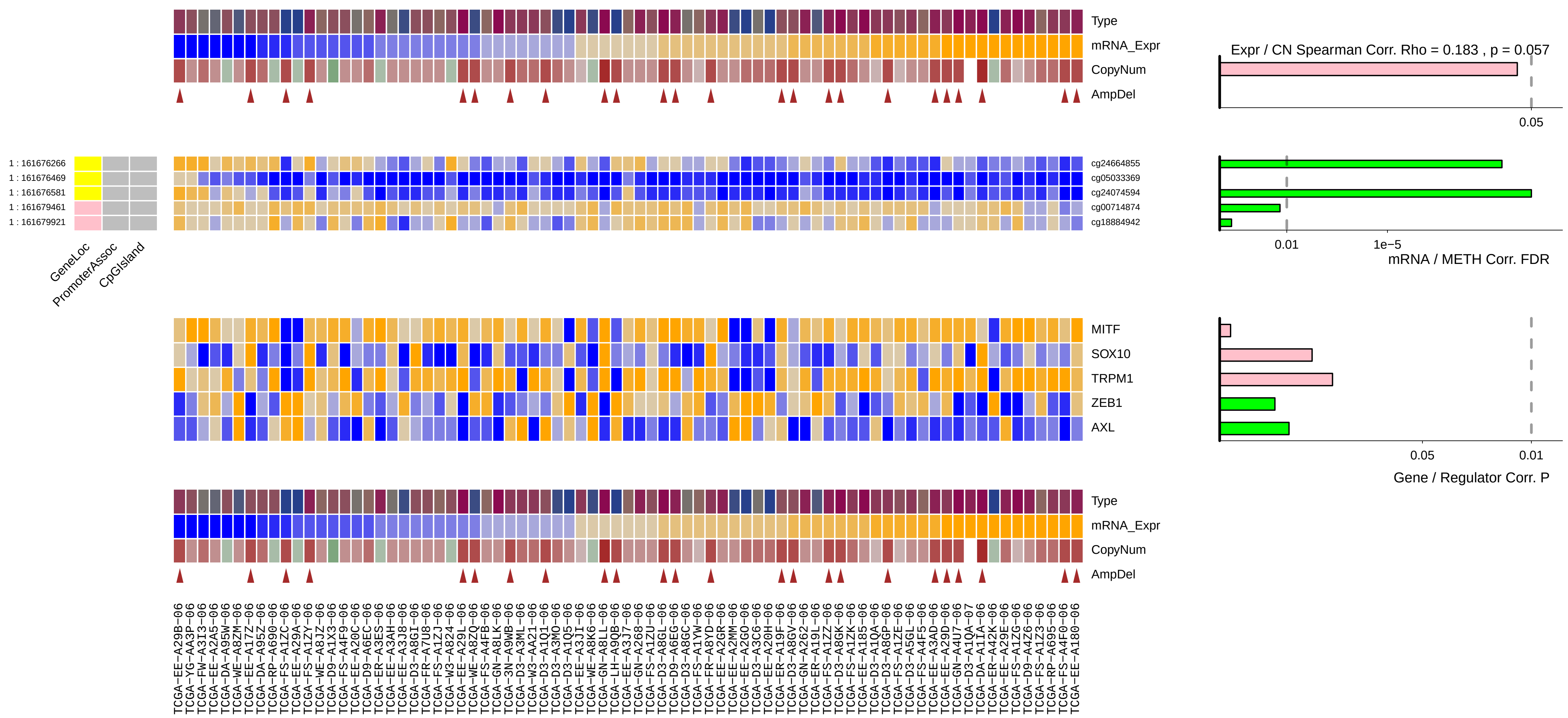
Type
mRNA_Expr
CopyNum
AmpDel
Expr / CN Spearman Corr. Rho = 0.183 , p = 0.057
0.05
1 : 161676266
1 : 161676469
1 : 161676581
1 : 161679461
1 : 161679921
GeneLoc
PromoterAssoc
CpGIsland
cg24664855
cg05033369
cg24074594
cg00714874
cg18884942
0.01
1e−5
mRNA / METH Corr. FDR
MITF
SOX10
TRPM1
ZEB1
AXL
0.05
0.01
Gene / Regulator Corr. P
Type
mRNA_Expr
CopyNum
AmpDel

TSPAN10

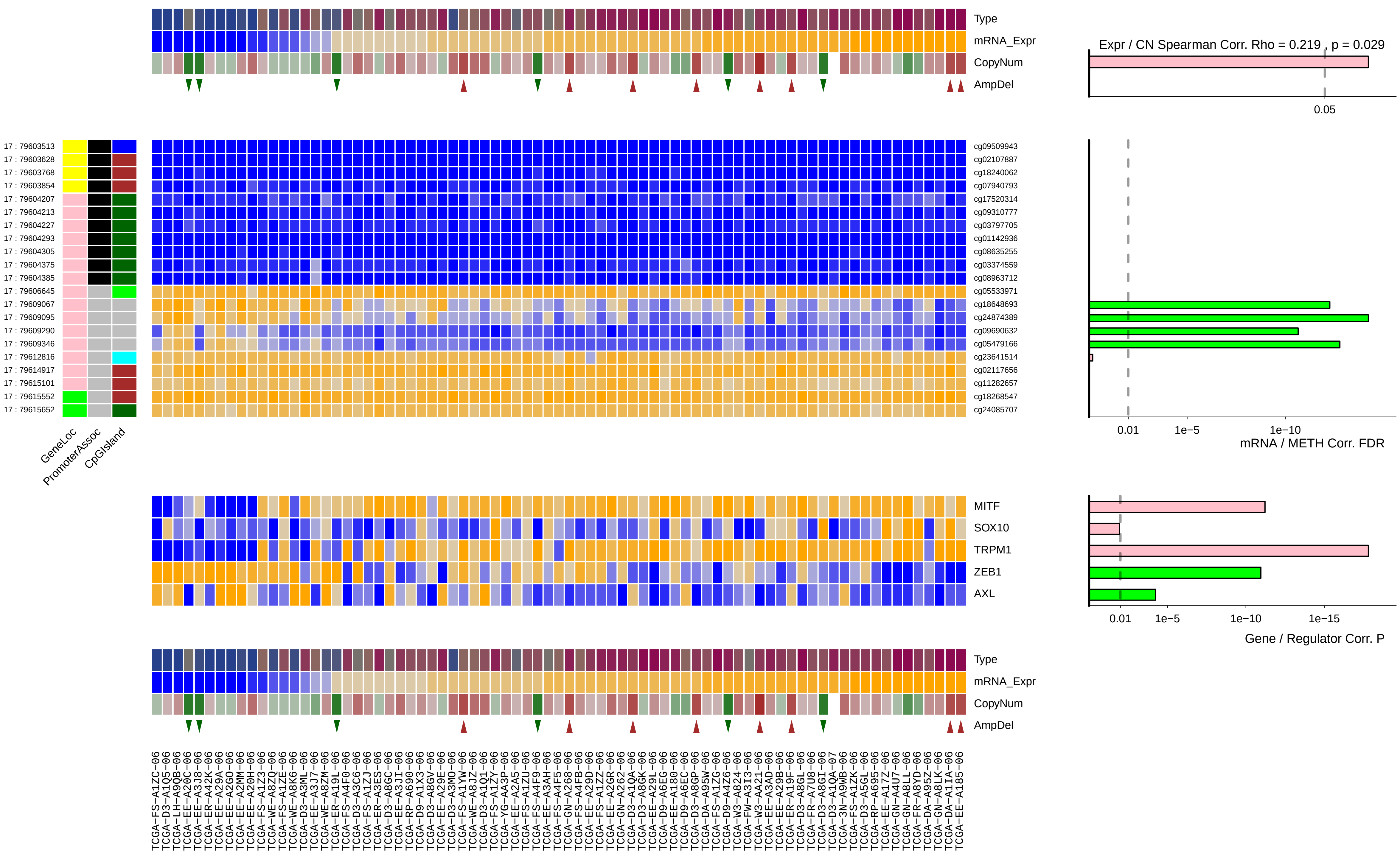

Type
mRNA_Expr
CopyNum
AmpDel
Expr / CN Spearman Corr. Rho = 0.219 , p = 0.029
0.05
17 : 79603513
17 : 79603628
17 : 79603768
17 : 79603854
17 : 79604207
17 : 79604213
17 : 79604227
17 : 79604293
17 : 79604305
17 : 79604375
17 : 79604385
17 : 79606645
17 : 79609067
17 : 79609095
17 : 79609290
17 : 79609346
17 : 79612816
17 : 79614917
17 : 79615101
17 : 79615552
17 : 79615652
cg09509943
cg02107887
cg18240062
cg07940793
cg17520314
cg09310777
cg03797705
cg01142936
cg08635255
cg03374559
cg08963712
cg05533971
cg18648693
cg24874389
cg09690632
cg05479166
cg23641514
cg02117656
cg11282657
cg18268547
cg24085707
GeneLoc
PromoterAssoc
CpGIsland
0.01
1e−5
1e−10
mRNA / METH Corr. FDR
MITF
SOX10
TRPM1
ZEB1
AXL
0.01
1e−5
1e−10
1e−15
Gene / Regulator Corr. P
Type
mRNA_Expr
CopyNum
AmpDel

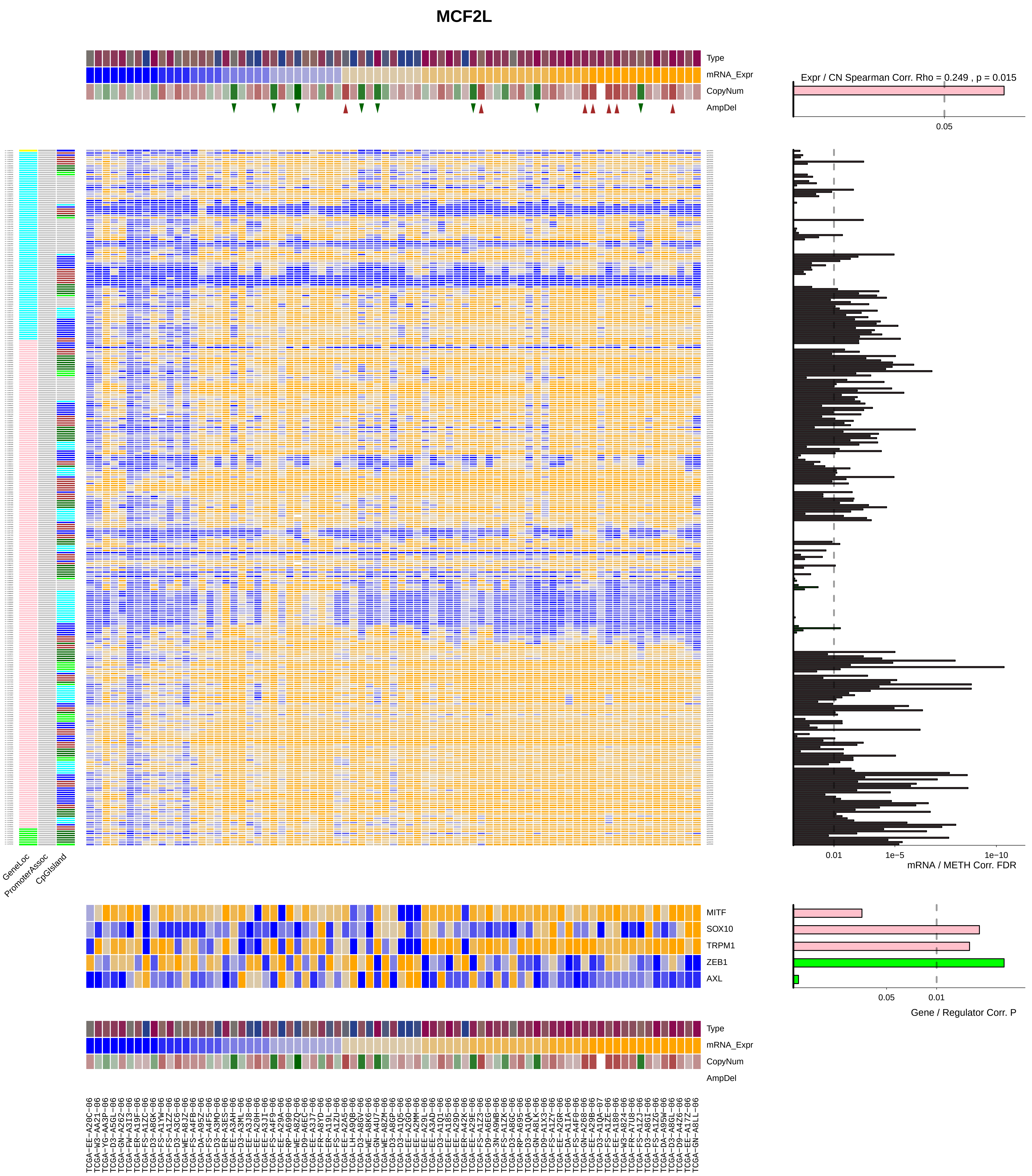

MCF2L
Type
mRNA_Expr
CopyNum
AmpDel
Expr / CN Spearman Corr. Rho = 0.249 , p = 0.015
0.05
GeneLoc
PromoterAssoc
CpGIsland
0.01
1e−5
1e−10
mRNA / METH Corr. FDR
MITF
SOX10
TRPM1
ZEB1
AXL
0.05
0.01
Gene / Regulator Corr. P
Type
mRNA_Expr
CopyNum
AmpDel
TCGA−EE−A20C−06
TCGA−W3−AA21−06
TCGA−YG−AA3P−06
TCGA−D3−A5GL−06
TCGA−GN−A262−06
TCGA−FW−A3I3−06
TCGA−ER−A19F−06
TCGA−FS−A1ZC−06
TCGA−D3−A8GK−06
TCGA−FS−A1YW−06
TCGA−FS−A1ZZ−06
TCGA−D3−A3C6−06
TCGA−WE−A8JZ−06
TCGA−FS−A4FB−06
TCGA−DA−A95Z−06
TCGA−FS−A4F5−06
TCGA−D3−A3MO−06
TCGA−ER−A3ES−06
TCGA−EE−A3AH−06
TCGA−D3−A3ML−06
TCGA−EE−A3J8−06
TCGA−EE−A20H−06
TCGA−EE−A3JI−06
TCGA−FS−A4F9−06
TCGA−EE−A29A−06
TCGA−RP−A690−06
TCGA−WE−A8ZQ−06
TCGA−D9−A6EC−06
TCGA−EE−A3J7−06
TCGA−FR−A8YD−06
TCGA−ER−A19L−06
TCGA−FS−A1ZU−06
TCGA−EE−A2A5−06
TCGA−LH−A9QB−06
TCGA−D3−A8GV−06
TCGA−WE−A8K6−06
TCGA−GN−A4U7−06
TCGA−WE−A8ZM−06
TCGA−D3−A8GP−06
TCGA−D3−A1Q5−06
TCGA−EE−A2GO−06
TCGA−EE−A2MM−06
TCGA−EE−A29L−06
TCGA−EE−A3AD−06
TCGA−D3−A1Q1−06
TCGA−EE−A185−06
TCGA−EE−A29D−06
TCGA−ER−A42K−06
TCGA−EE−A29E−06
TCGA−FS−A1Z3−06
TCGA−D9−A6EG−06
TCGA−3N−A9WB−06
TCGA−FS−A1ZK−06
TCGA−D3−A8GC−06
TCGA−RP−A695−06
TCGA−D3−A1QA−06
TCGA−GN−A8LK−06
TCGA−D9−A1X3−06
TCGA−FS−A1ZY−06
TCGA−EE−A2GR−06
TCGA−DA−A1IA−06
TCGA−FS−A4F0−06
TCGA−GN−A268−06
TCGA−EE−A29B−06
TCGA−D3−A1QA−07
TCGA−FS−A1ZE−06
TCGA−EE−A180−06
TCGA−W3−A824−06
TCGA−FR−A7U8−06
TCGA−FS−A1ZJ−06
TCGA−D3−A8GI−06
TCGA−FS−A1ZG−06
TCGA−DA−A95W−06
TCGA−D3−A8GL−06
TCGA−D9−A4Z6−06
TCGA−EE−A17Z−06
TCGA−GN−A8LL−06

CA14

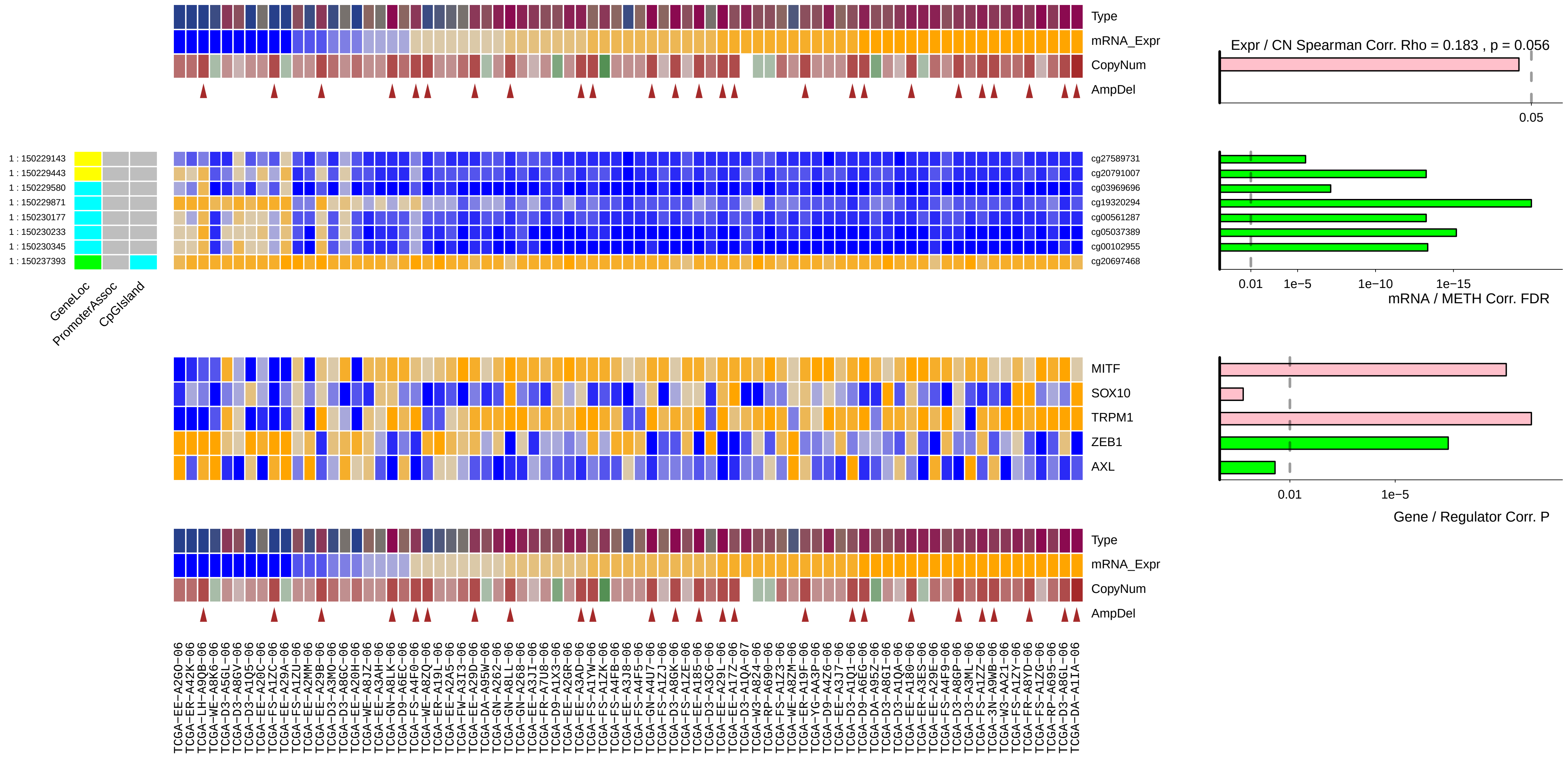
Type
mRNA_Expr
CopyNum
AmpDel
Expr / CN Spearman Corr. Rho = 0.183 , p = 0.056
0.05
1 : 150229143
1 : 150229443
1 : 150229580
1 : 150229871
1 : 150230177
1 : 150230233
1 : 150230345
1 : 150237393
GeneLoc
PromoterAssoc
CpGIsland
cg27589731
cg20791007
cg03969696
cg19320294
cg00561287
cg05037389
cg00102955
cg20697468
0.01
1e−5
1e−10
1e−15
mRNA / METH Corr. FDR
MITF
SOX10
TRPM1
ZEB1
AXL
0.01
1e−5
Gene / Regulator Corr. P

TRIM2

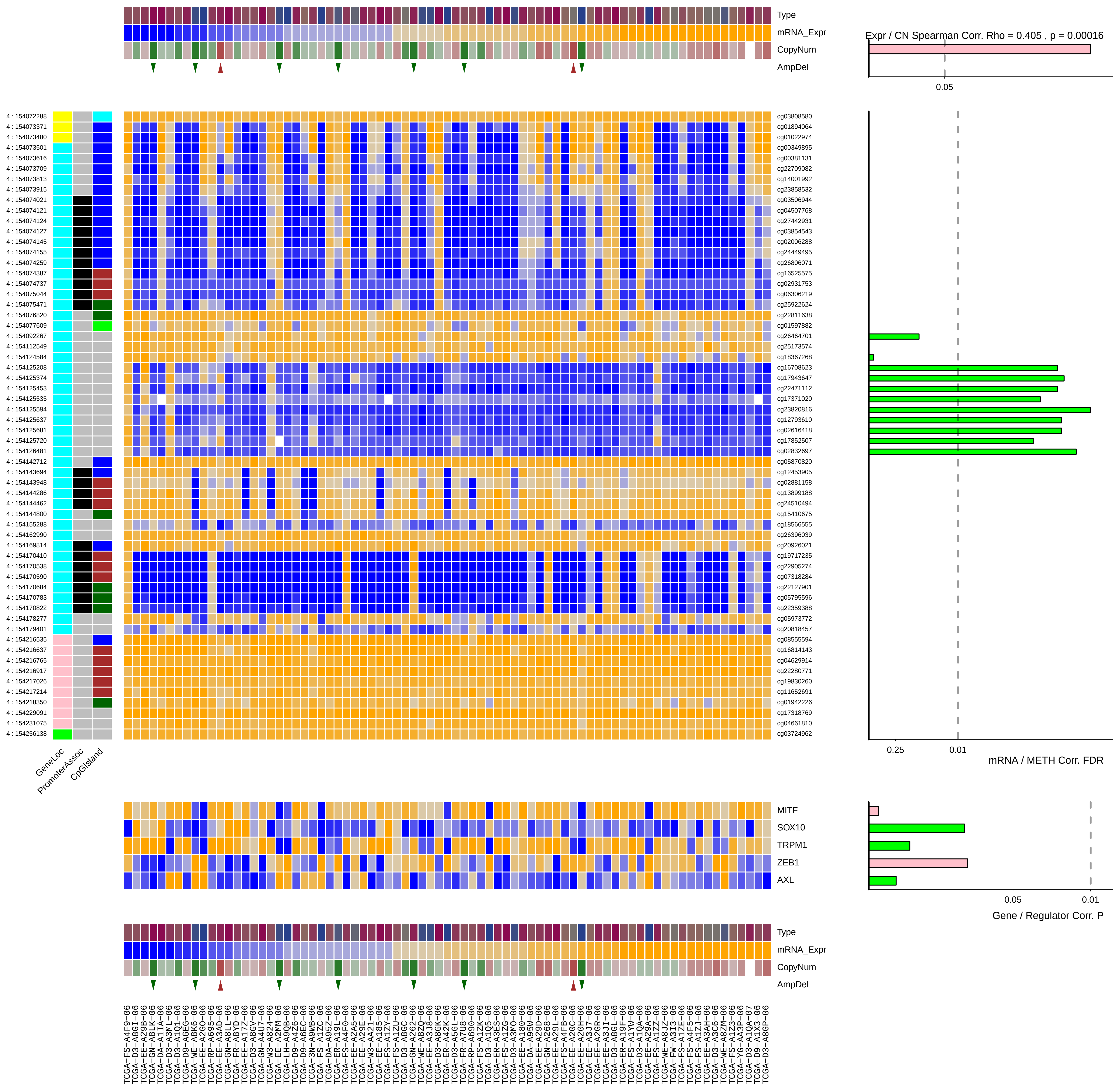

Type
mRNA_Expr
CopyNum
AmpDel
Expr / CN Spearman Corr. Rho = 0.405 , p = 0.00016
0.05
mRNA / METH Corr. FDR
0.25
0.01
GeneLoc
PromoterAssoc
CpGIsland
MITF
SOX10
TRPM1
ZEB1
AXL
Gene / Regulator Corr. P
0.05
0.01

APOLD1

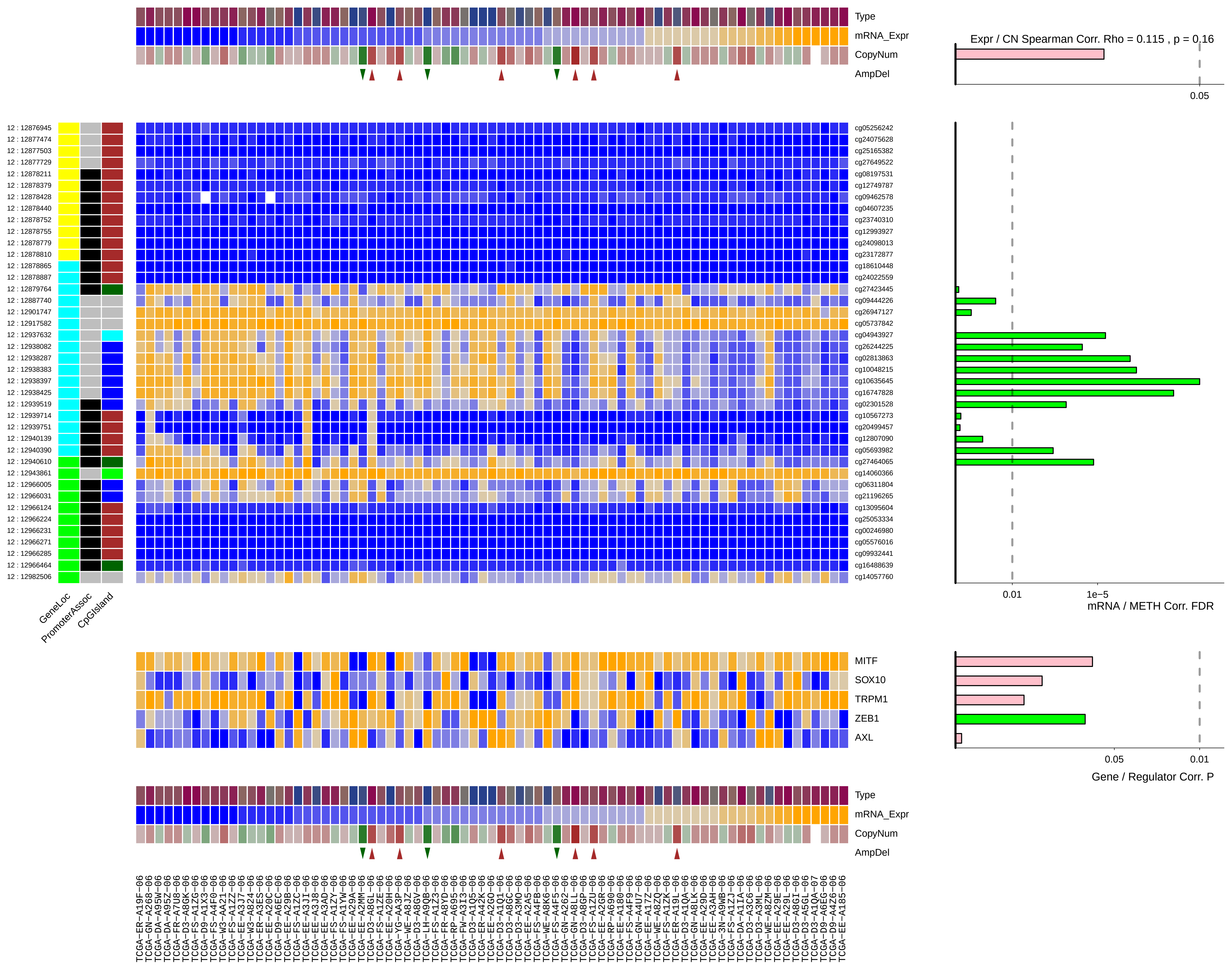

Type
mRNA_Expr
CopyNum
AmpDel
Expr / CN Spearman Corr. Rho = 0.115 , p = 0.16
0.05
12 : 12876945
12 : 12877474
12 : 12877503
12 : 12877729
12 : 12878211
12 : 12878379
12 : 12878428
12 : 12878440
12 : 12878752
12 : 12878755
12 : 12878779
12 : 12878810
12 : 12878865
12 : 12878887
12 : 12879764
12 : 12887740
12 : 12901747
12 : 12917582
12 : 12937632
12 : 12938082
12 : 12938287
12 : 12938383
12 : 12938397
12 : 12938425
12 : 12939519
12 : 12939714
12 : 12939751
12 : 12940139
12 : 12940390
12 : 12940610
12 : 12943861
12 : 12966005
12 : 12966031
12 : 12966124
12 : 12966224
12 : 12966231
12 : 12966271
12 : 12966285
12 : 12966464
12 : 12982506
cg05256242
cg24075628
cg25165382
cg27649522
cg08197531
cg12749787
cg09462578
cg04607235
cg23740310
cg12993927
cg24098013
cg23172877
cg18610448
cg24022559
cg27423445
cg09444226
cg26947127
cg05737842
cg04943927
cg26244225
cg02813863
cg10048215
cg10635645
cg16747828
cg02301528
cg10567273
cg20499457
cg12807090
cg05693982
cg27464065
cg14060366
cg06311804
cg21196265
cg13095604
cg25053334
cg00246980
cg05576016
cg09932441
cg16488639
cg14057760
0.01
1e−5
mRNA / METH Corr. FDR
GeneLoc
PromoterAssoc
CpGIsland
MITF
SOX10
TRPM1
ZEB1
AXL
0.05
0.01
Gene / Regulator Corr. P
Type
mRNA_Expr
CopyNum
AmpDel

ITGA9

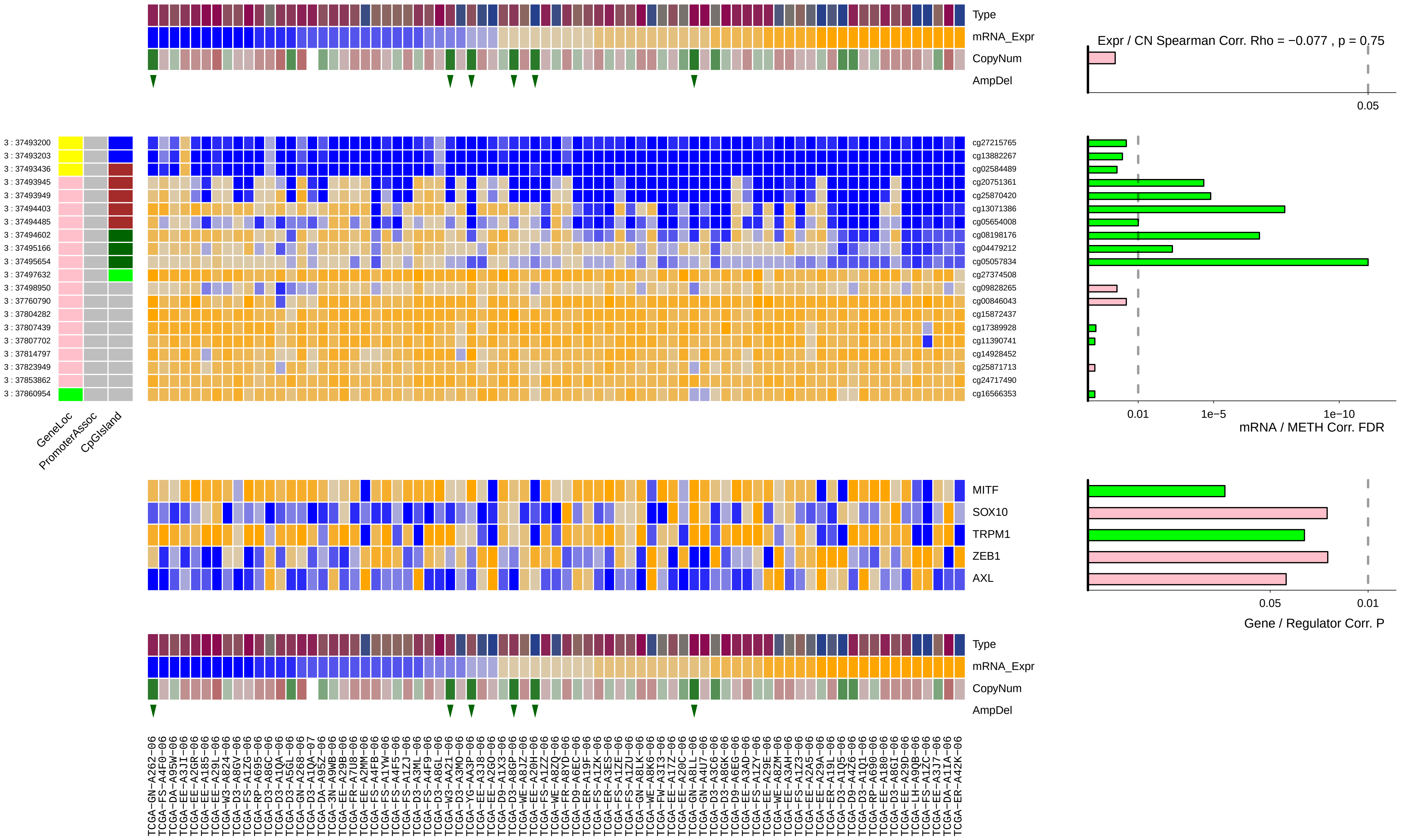

Type
mRNA_Expr
CopyNum
AmpDel
Expr / CN Spearman Corr. Rho = −0.077 , p = 0.75
0.05
3 : 37493200
3 : 37493203
3 : 37493436
3 : 37493945
3 : 37493949
3 : 37494403
3 : 37494485
3 : 37494602
3 : 37495166
3 : 37495654
3 : 37497632
3 : 37498950
3 : 37760790
3 : 37804282
3 : 37807439
3 : 37807702
3 : 37814797
3 : 37823949
3 : 37853862
3 : 37860954
cg27215765
cg13882267
cg02584489
cg20751361
cg25870420
cg13071386
cg05654008
cg08198176
cg04479212
cg05057834
cg27374508
cg09828265
cg00846043
cg15872437
cg17389928
cg11390741
cg14928452
cg25871713
cg24717490
cg16566353
GeneLoc
PromoterAssoc
CpGIsland
0.01
1e−5
1e−10
mRNA / METH Corr. FDR
MITF
SOX10
TRPM1
ZEB1
AXL
0.05
0.01
Gene / Regulator Corr. P
Type
mRNA_Expr
CopyNum
AmpDel
TCGA−GN−A262−06
TCGA−FS−A4F0−06
TCGA−DA−A95W−06
TCGA−EE−A3JI−06
TCGA−EE−A2GR−06
TCGA−EE−A185−06
TCGA−EE−A29L−06
TCGA−W3−A824−06
TCGA−D3−A8GV−06
TCGA−FS−A1ZG−06
TCGA−RP−A695−06
TCGA−D3−A8GC−06
TCGA−D3−A1QA−06
TCGA−EE−A3AG−06
TCGA−GN−A268−06
TCGA−D3−A1QA−07
TCGA−DA−A95Z−06
TCGA−3N−A9WB−06
TCGA−EE−A29B−06
TCGA−FR−A7U8−06
TCGA−EE−A2MM−06
TCGA−FS−A4FB−06
TCGA−FS−A1YW−06
TCGA−FS−A4F5−06
TCGA−FS−A1ZJ−06
TCGA−D3−A3ML−06
TCGA−FS−A4F9−06
TCGA−D3−A8GL−06
TCGA−W3−AA21−06
TCGA−D3−A3MO−06
TCGA−YG−AA3P−06
TCGA−EE−A3J8−06
TCGA−EE−A2GO−06
TCGA−D9−A1X3−06
TCGA−D3−A8GP−06
TCGA−WE−A8JZ−06
TCGA−FS−A1ZZ−06
TCGA−WE−A8ZQ−06
TCGA−FR−A8YD−06
TCGA−D9−A6EC−06
TCGA−ER−A19F−06
TCGA−FS−A1ZK−06
TCGA−ER−A3ES−06
TCGA−FS−A1ZE−06
TCGA−FS−A1ZU−06
TCGA−GN−A8LK−06
TCGA−WE−A8K6−06
TCGA−FW−A3I3−06
TCGA−EE−A17Z−06
TCGA−EE−A20C−06
TCGA−GN−A8LL−06
TCGA−GN−A4U7−06
TCGA−D3−A3C6−06
TCGA−D3−A8GK−06
TCGA−D9−A6EG−06
TCGA−EE−A3AD−06
TCGA−FS−A1ZY−06
TCGA−WE−A8ZM−06
TCGA−EE−A3AH−06
TCGA−FS−A1Z3−06
TCGA−EE−A2A5−06
TCGA−EE−A29A−06
TCGA−ER−A19L−06
TCGA−D3−A1Q5−06
TCGA−D9−A4Z6−06
TCGA−D3−A1Q1−06
TCGA−RP−A690−06
TCGA−EE−A180−06
TCGA−D3−A8GI−06
TCGA−EE−A29D−06
TCGA−LH−A9QB−06
TCGA−FS−A1ZC−06
TCGA−EE−A3J7−06
TCGA−DA−A1IA−06
TCGA−ER−A42K−06

CHCHD10

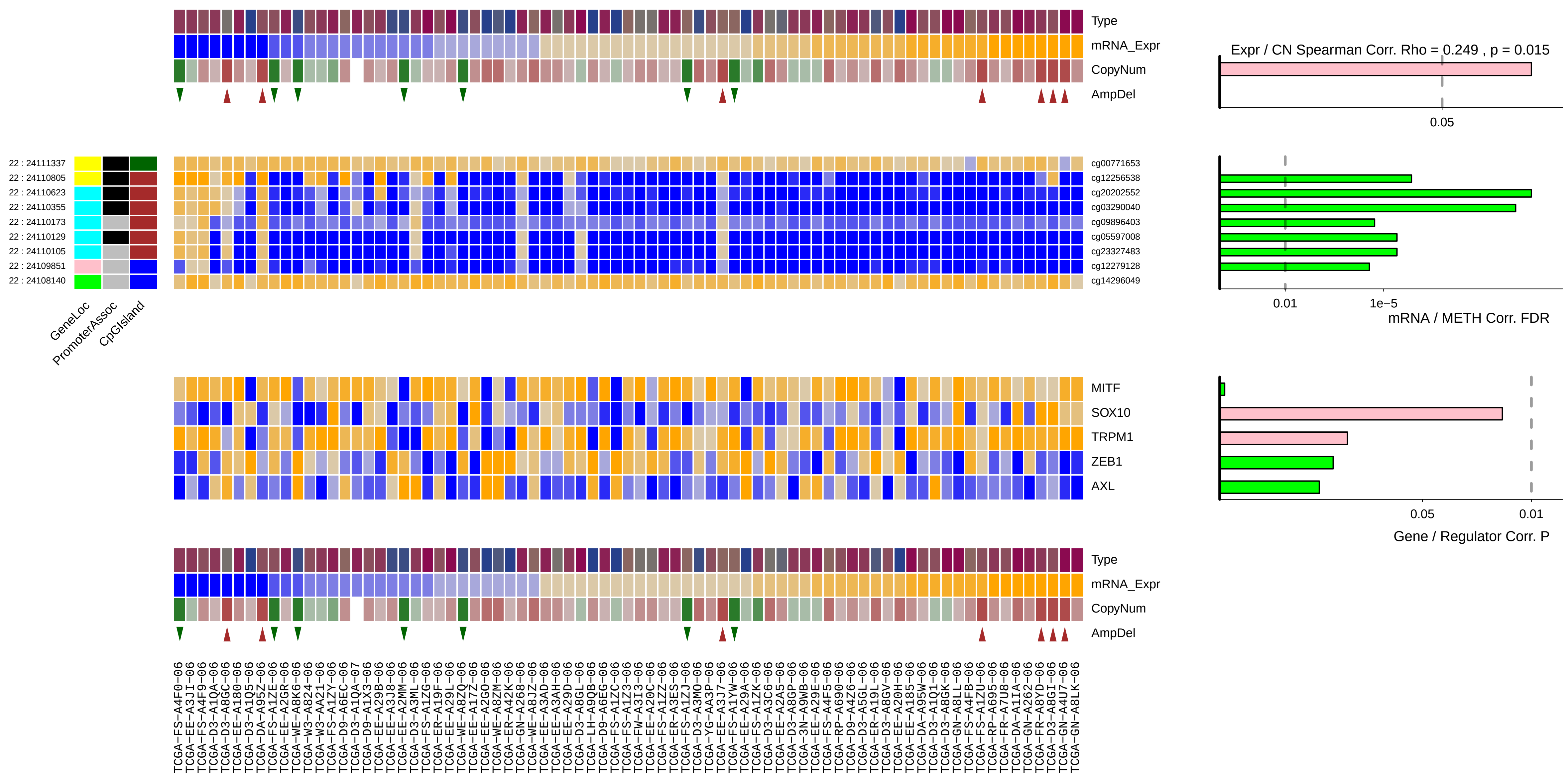

Type
mRNA_Expr
CopyNum
AmpDel
Expr / CN Spearman Corr. Rho = 0.249 , p = 0.015
0.05
22 : 24111337
22 : 24110805
22 : 24110623
22 : 24110355
22 : 24110173
22 : 24110129
22 : 24110105
22 : 24109851
22 : 24108140
GeneLoc
PromoterAssoc
CpGIsland
cg00771653
cg12256538
cg20202552
cg03290040
cg09896403
cg05597008
cg23327483
cg12279128
cg14296049
0.01
1e−5
mRNA / METH Corr. FDR
MITF
SOX10
TRPM1
ZEB1
AXL
0.05
0.01
Gene / Regulator Corr. P
Type
mRNA_Expr
CopyNum
AmpDel

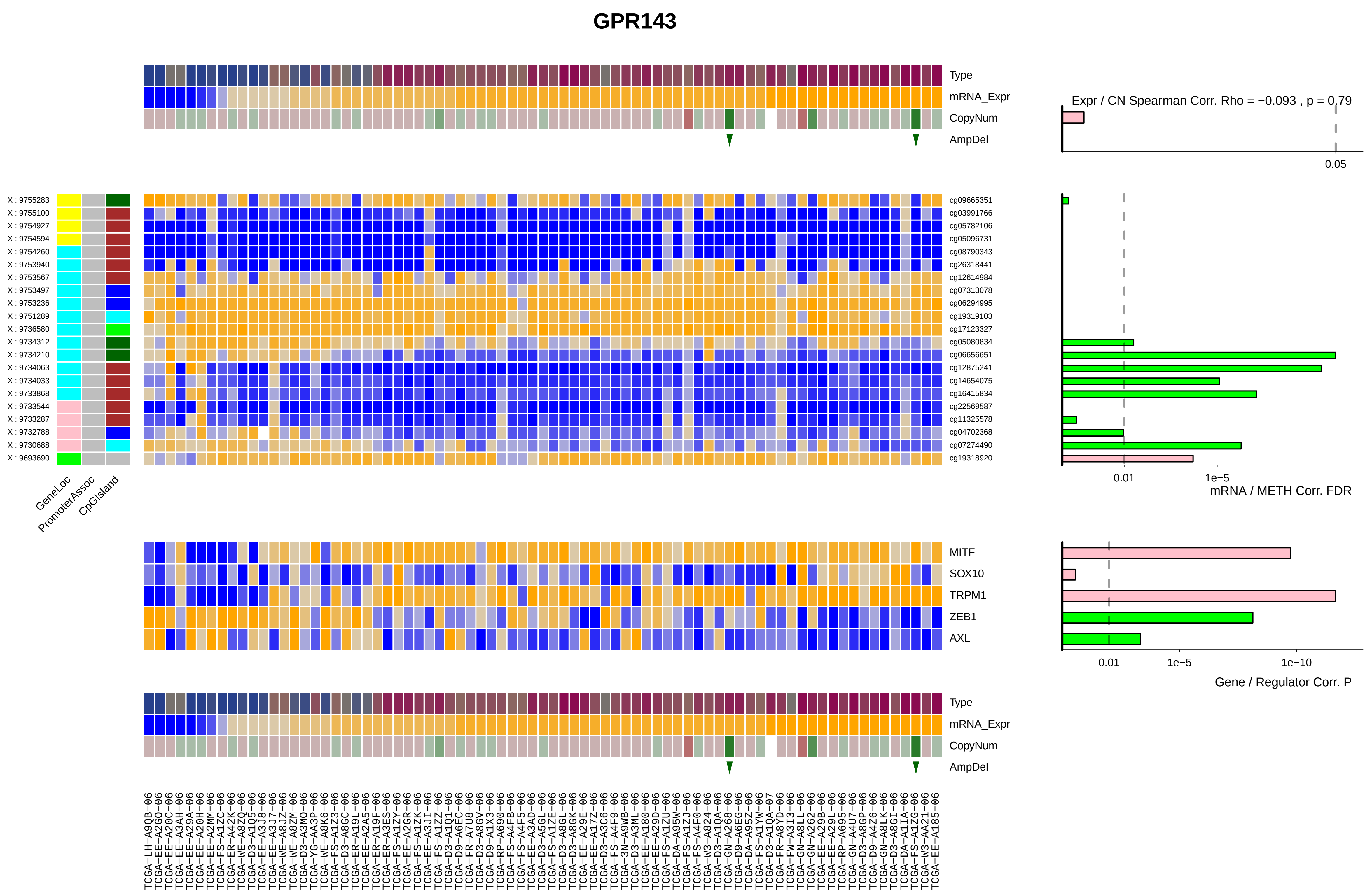
GPR143
Type
mRNA_Expr
CopyNum
AmpDel
Expr / CN Spearman Corr. Rho = −0.093 , p = 0.79
0.05
X : 9755283
X : 9755100
X : 9754927
X : 9754594
X : 9754260
X : 9753940
X : 9753567
X : 9753497
X : 9753236
X : 9751289
X : 9736580
X : 9734312
X : 9734210
X : 9734063
X : 9734033
X : 9733868
X : 9733544
X : 9733287
X : 9732788
X : 9730688
X : 9693690
GeneLoc
PromoterAssoc
CpGIsland
cg09665351
cg03991766
cg05782106
cg05096731
cg08790343
cg26318441
cg12614984
cg07313078
cg06294995
cg19319103
cg17123327
cg05080834
cg06656651
cg12875241
cg14654075
cg16415834
cg22569587
cg11325578
cg04702368
cg07274490
cg19318920
0.01
1e−5
mRNA / METH Corr. FDR
MITF
SOX10
TRPM1
ZEB1
AXL
0.01
1e−5
1e−10
Gene / Regulator Corr. P
Type
mRNA_Expr
CopyNum
AmpDel

ST6GALNAC3

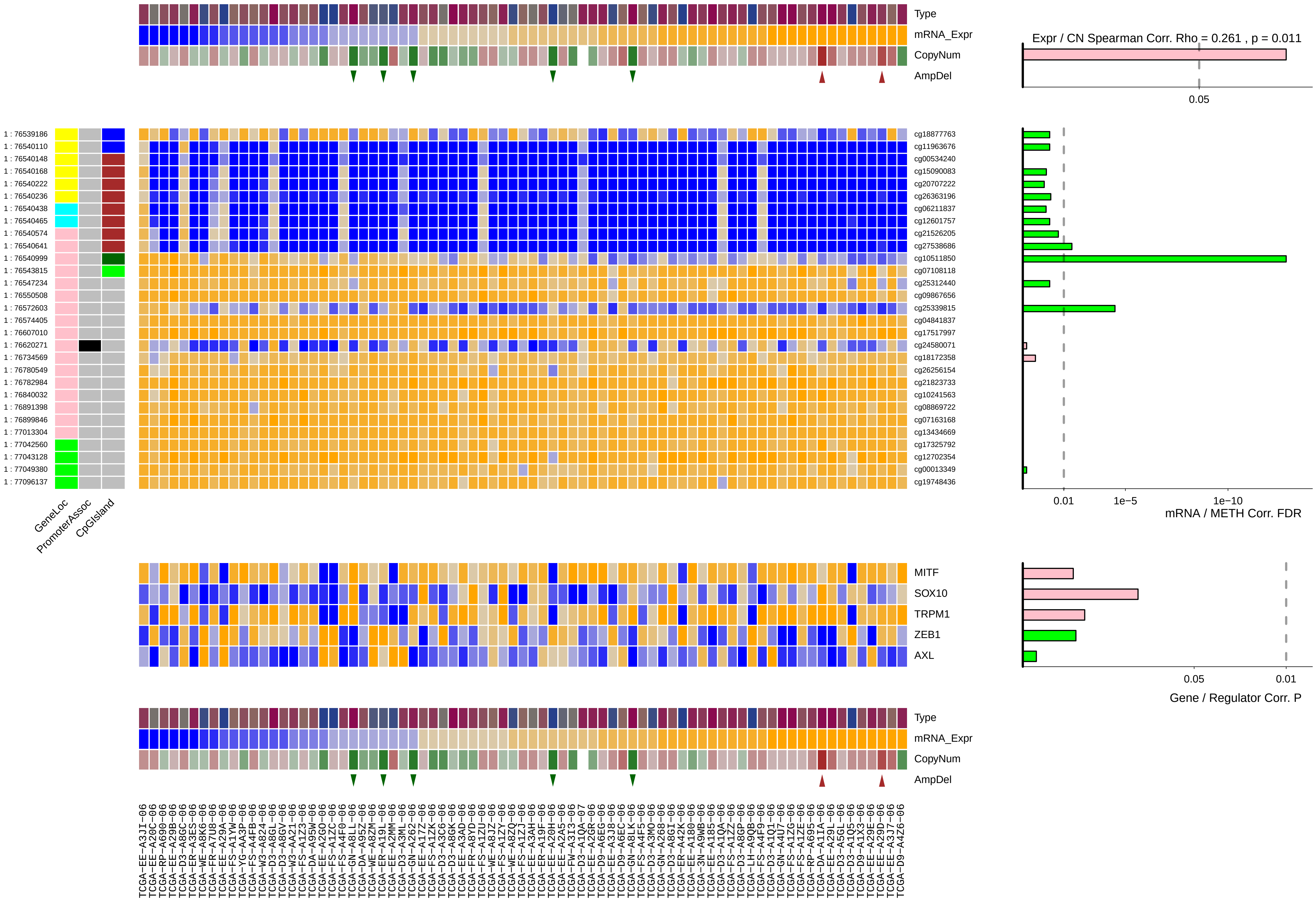

Type
mRNA_Expr
CopyNum
AmpDel
Expr / CN Spearman Corr. Rho = 0.261 , p = 0.011
0.05
mRNA / METH Corr. FDR
0.01
1e−5
1e−10
GeneLoc
PromoterAssoc
CpGIsland
MITF
SOX10
TRPM1
ZEB1
AXL
Gene / Regulator Corr. P

RAP1GAP

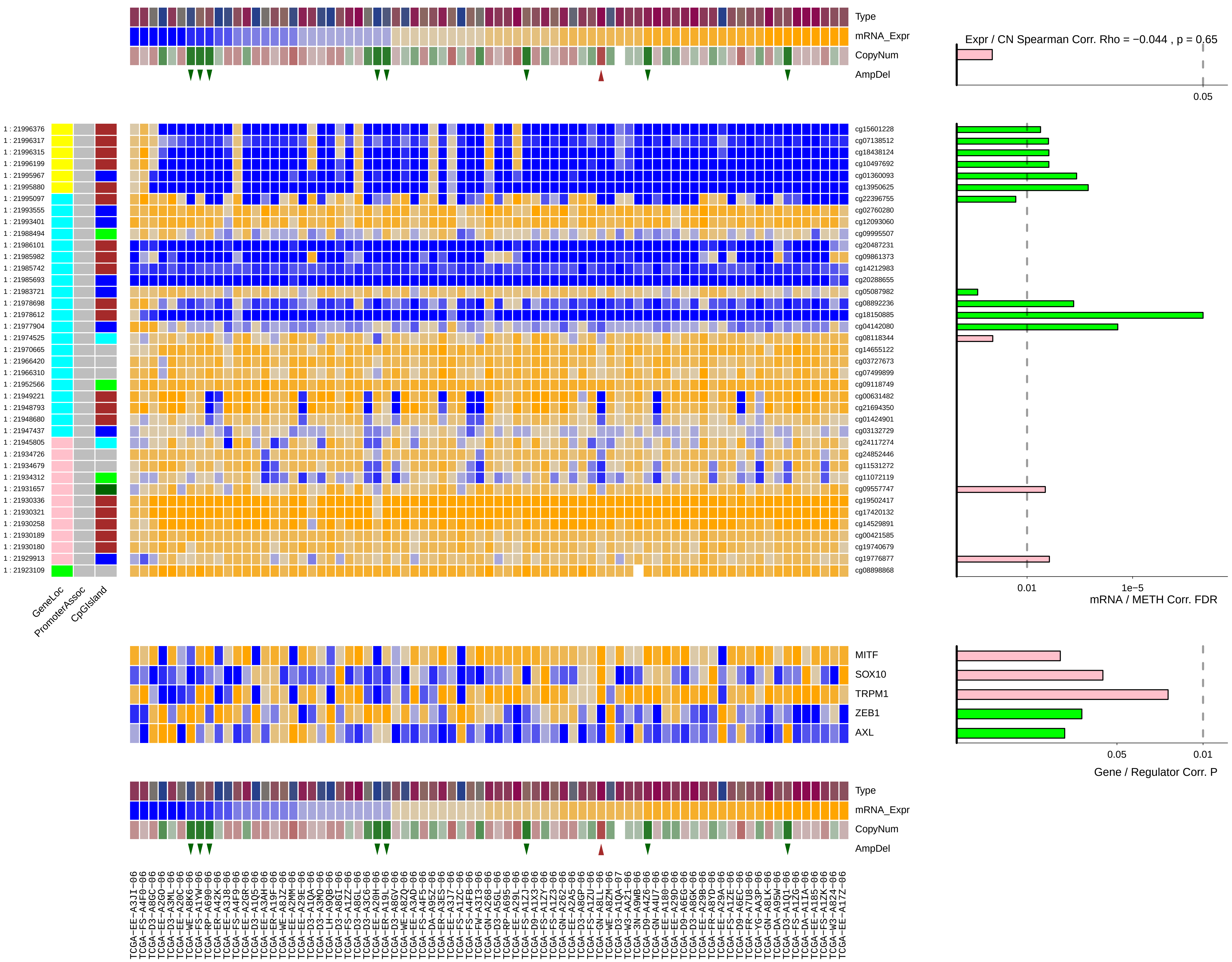
Type
mRNA_Expr
CopyNum
AmpDel
Expr / CN Spearman Corr. Rho = −0.044 , p = 0.65
0.05
1 : 21996376 cg15601228
1 : 21996317 cg07138512
1 : 21996315 cg18438124
1 : 21996199 cg10497692
1 : 21995967 cg01360093
1 : 21995880 cg13950625
1 : 21995097 cg22396755
1 : 21993555 cg02760280
1 : 21993401 cg12093060
1 : 21988494 cg09995507
1 : 21986101 cg20487231
1 : 21985982 cg09861373
1 : 21985742 cg14212983
1 : 21985693 cg20288655
1 : 21983721 cg05087982
1 : 21978698 cg08892236
1 : 21978612 cg18150885
1 : 21977904 cg04142080
1 : 21974525 cg08118344
1 : 21970665 cg14655122
1 : 21966420 cg03727673
1 : 21966310 cg07499899
1 : 21952566 cg09118749
1 : 21949221 cg00631482
1 : 21948793 cg21694350
1 : 21948680 cg01424901
1 : 21947437 cg03132729
1 : 21945805 cg24117274
1 : 21934726 cg24852446
1 : 21934679 cg11531272
1 : 21934312 cg11072119
1 : 21931657 cg09557747
1 : 21930336 cg19502417
1 : 21930321 cg17420132
1 : 21930258 cg14529891
1 : 21930189 cg00421585
1 : 21930180 cg19740679
1 : 21929913 cg19776877
1 : 21923109 cg08898868
GeneLoc
PromoterAssoc
CpGIsland
0.01
1e−5
mRNA / METH Corr. FDR
MITF
SOX10
TRPM1
ZEB1
AXL
0.05
0.01
Gene / Regulator Corr. P
Type
mRNA_Expr
CopyNum
AmpDel

CPVL

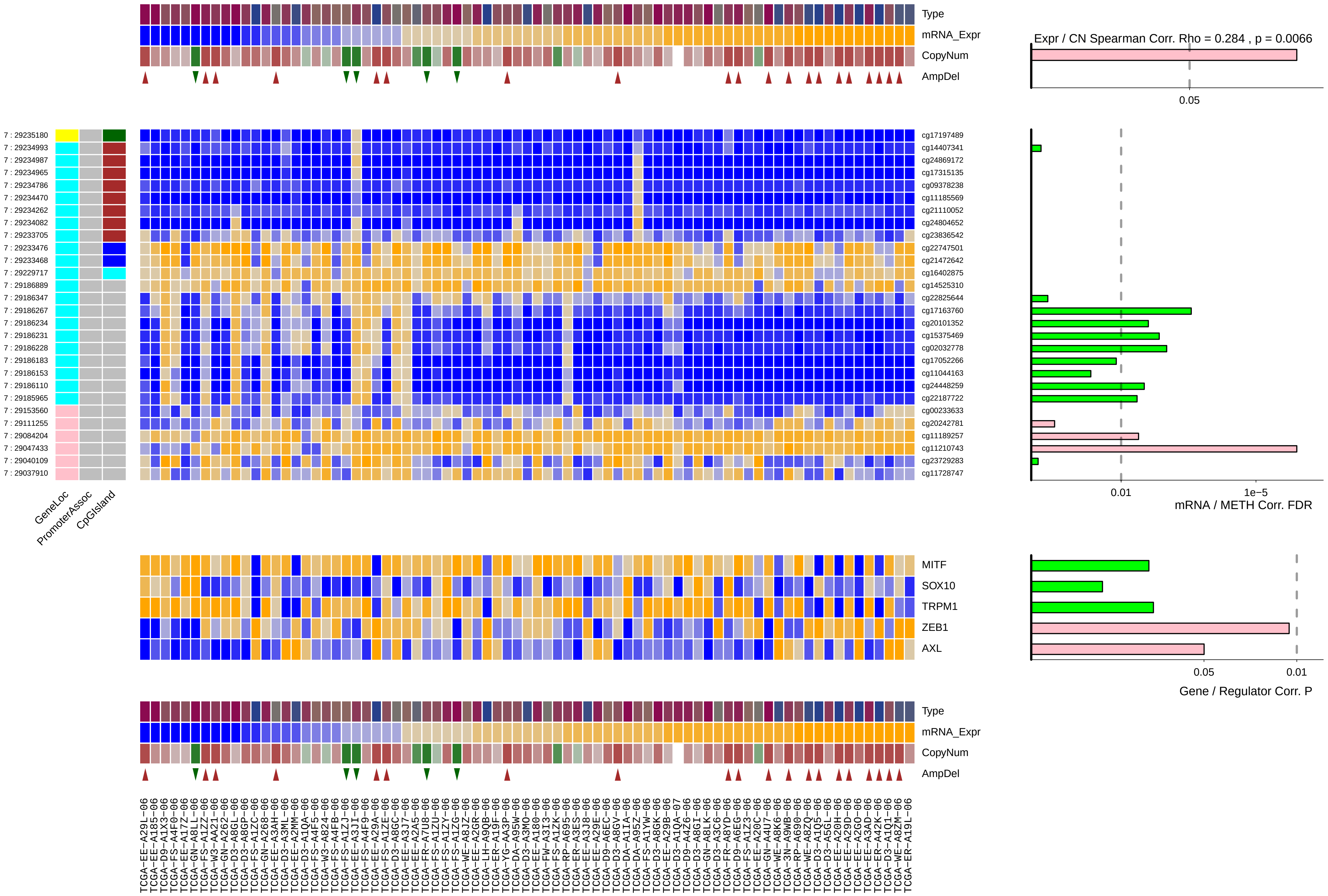
Type
mRNA_Expr
CopyNum
AmpDel
Expr / CN Spearman Corr. Rho = 0.284 , p = 0.0066
0.05
7 : 29235180
7 : 29234993
7 : 29234987
7 : 29234965
7 : 29234786
7 : 29234470
7 : 29234262
7 : 29234082
7 : 29233705
7 : 29233476
7 : 29233468
7 : 29229717
7 : 29186889
7 : 29186347
7 : 29186267
7 : 29186234
7 : 29186231
7 : 29186228
7 : 29186183
7 : 29186153
7 : 29186110
7 : 29185965
7 : 29153560
7 : 29111255
7 : 29084204
7 : 29047433
7 : 29040109
7 : 29037910
cg17197489
cg14407341
cg24869172
cg17315135
cg09378238
cg11185569
cg21110052
cg24804652
cg23836542
cg22747501
cg21472642
cg16402875
cg14525310
cg22825644
cg17163760
cg20101352
cg15375469
cg02032778
cg17052266
cg11044163
cg24448259
cg22187722
cg00233633
cg20242781
cg11189257
cg11210743
cg23729283
cg11728747
0.01
1e−5
mRNA / METH Corr. FDR
GeneLoc
PromoterAssoc
CpGIsland
MITF
SOX10
TRPM1
ZEB1
AXL
0.05
0.01
Gene / Regulator Corr. P
Type
mRNA_Expr
CopyNum
AmpDel

ACP5

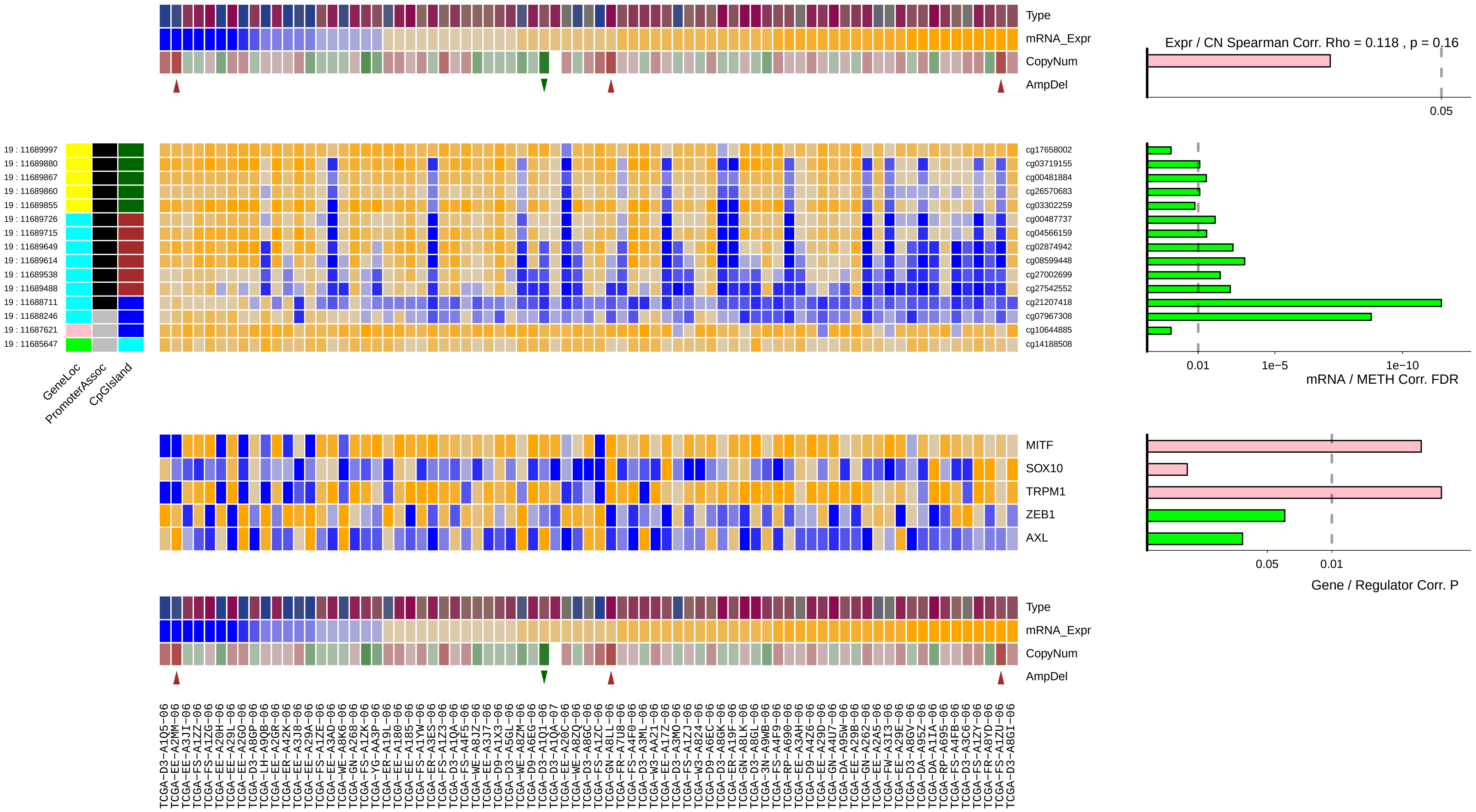

Type
mRNA_Expr
CopyNum
AmpDel
Expr / CN Spearman Corr. Rho = 0.118 , p = 0.16
0.05
19 : 11689997
19 : 11689880
19 : 11689867
19 : 11689860
19 : 11689855
19 : 11689726
19 : 11689715
19 : 11689649
19 : 11689614
19 : 11689538
19 : 11689488
19 : 11688711
19 : 11688246
19 : 11687621
19 : 11685647
GeneLoc
PromoterAssoc
CpGIsland
cg17658002
cg03719155
cg00481884
cg26570683
cg03302259
cg00487737
cg04566159
cg02874942
cg08599448
cg27002699
cg27542552
cg21207418
cg07967308
cg10644885
cg14188508
0.01
1e−5
1e−10
mRNA / METH Corr. FDR
MITF
SOX10
TRPM1
ZEB1
AXL
0.05
0.01
Gene / Regulator Corr. P
Type
mRNA_Expr
CopyNum
AmpDel

LZTS1

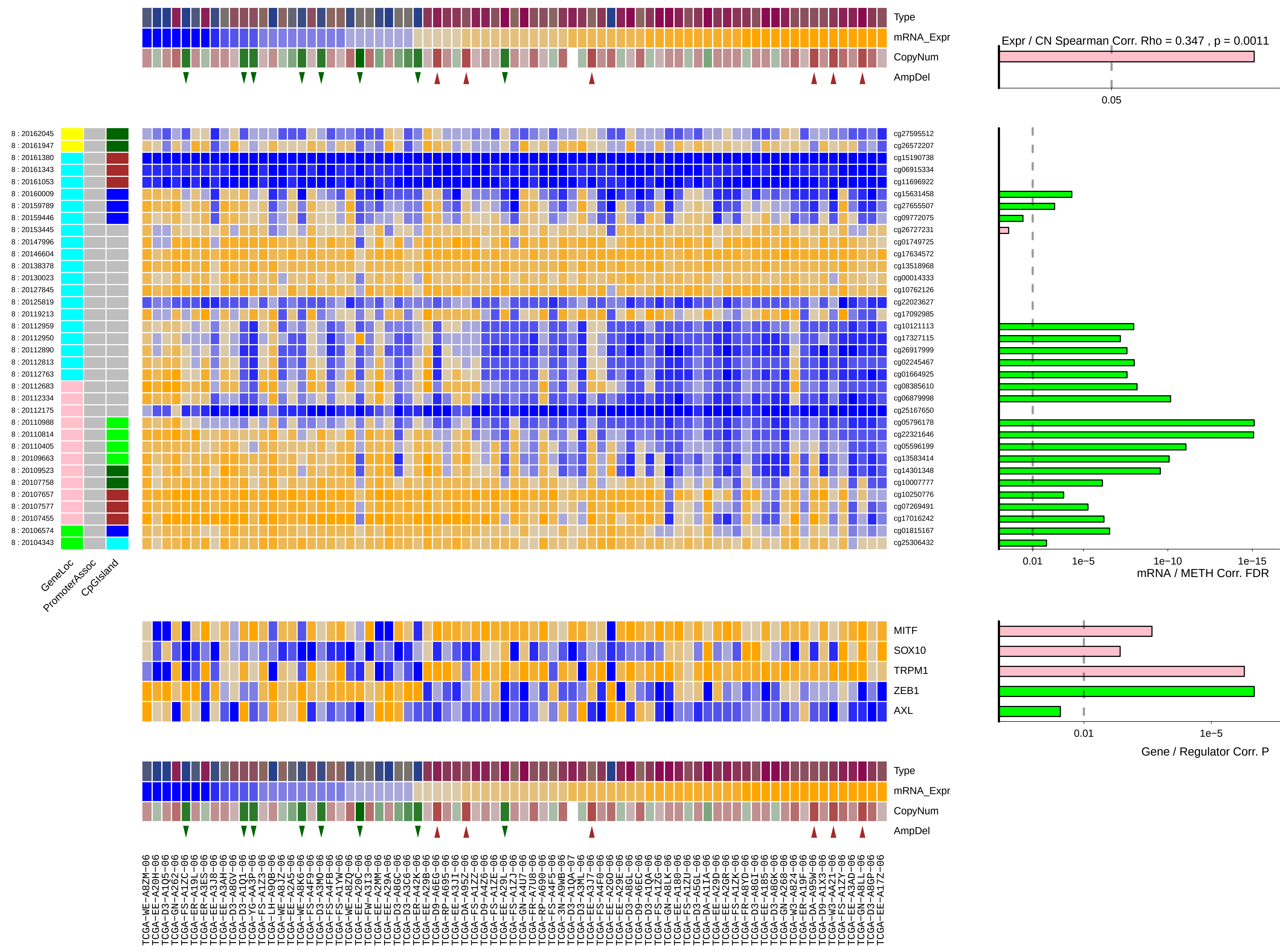
Type
mRNA_Expr
CopyNum
AmpDel
Expr / CN Spearman Corr. Rho = 0.347 , p = 0.0011
0.05
GeneLoc
PromoterAssoc
CpGIsland
0.01
1e−5
1e−10
1e−15
mRNA / METH Corr. FDR
MITF
SOX10
TRPM1
ZEB1
AXL
0.01
1e−5
Gene / Regulator Corr. P

CEACAM1

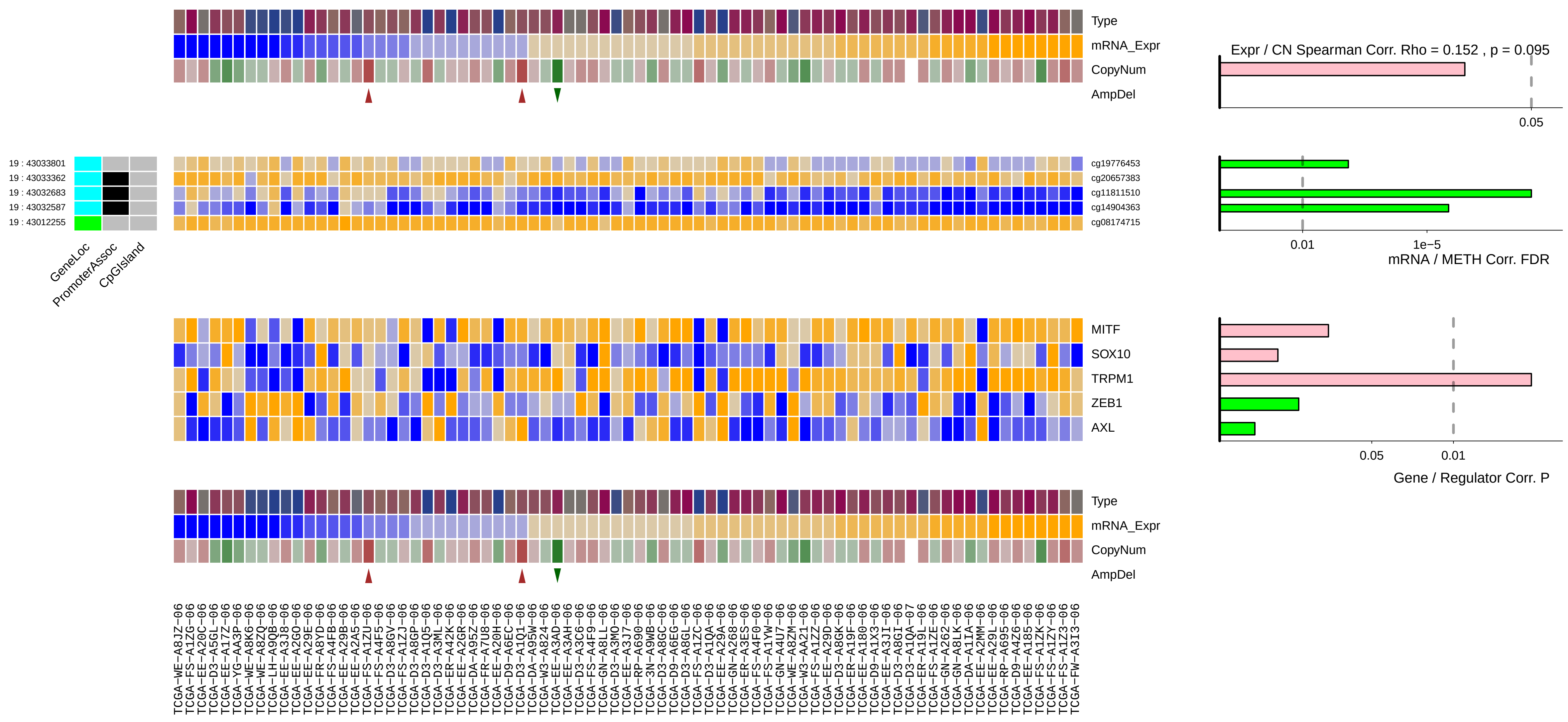
Type
mRNA_Expr
CopyNum
AmpDel
Expr / CN Spearman Corr. Rho = 0.152 , p = 0.095
0.05
19 : 43033801
19 : 43033362
19 : 43032683
19 : 43032587
19 : 43012255
GeneLoc
PromoterAssoc
CpGIsland
cg19776453
cg20657383
cg11811510
cg14904363
cg08174715
0.01
1e−5
mRNA / METH Corr. FDR
MITF
SOX10
TRPM1
ZEB1
AXL
0.05
0.01
Gene / Regulator Corr. P
Type
mRNA_Expr
CopyNum
AmpDel

LAMA1

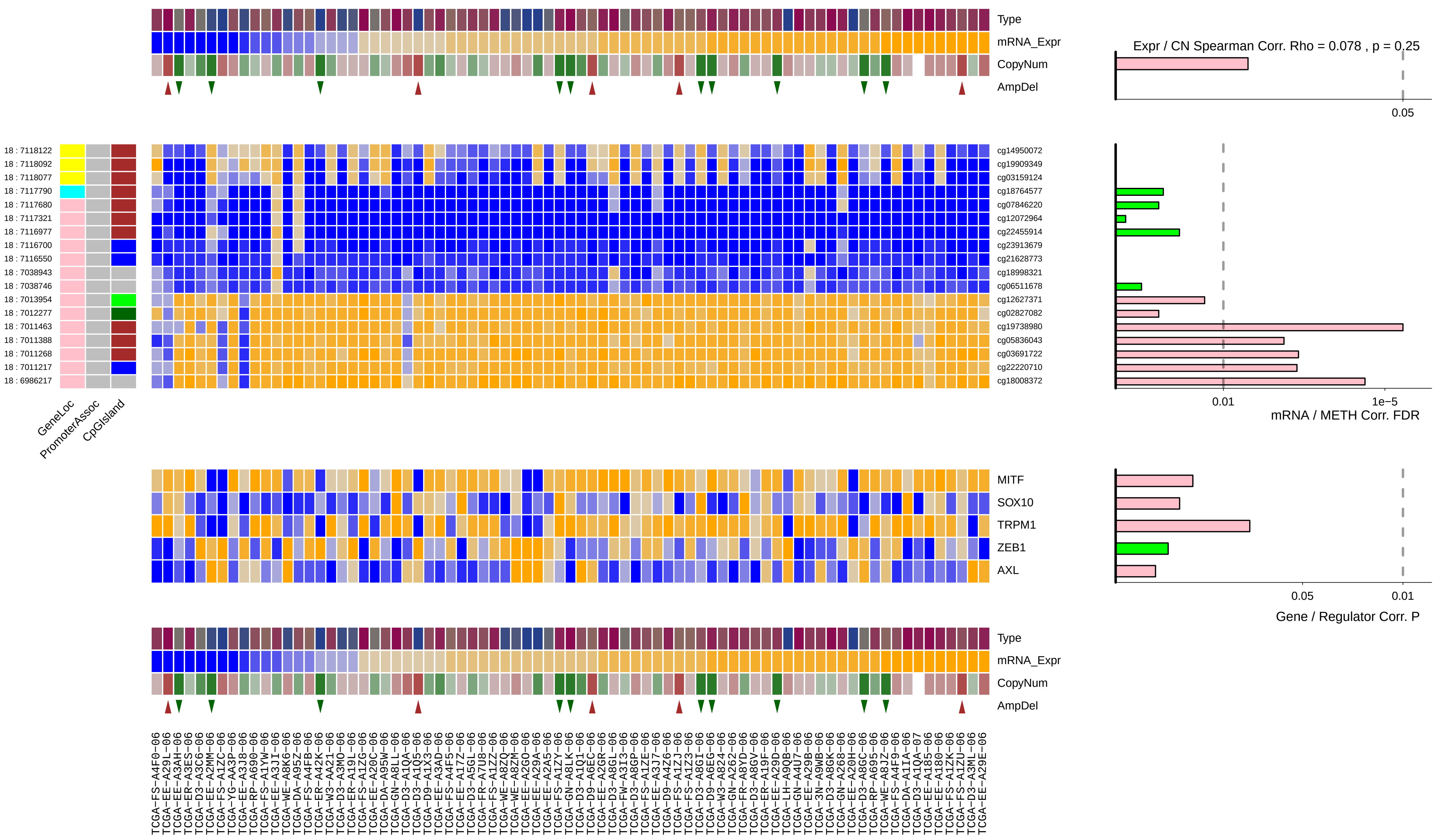

Type
mRNA_Expr
CopyNum
AmpDel
Expr / CN Spearman Corr. Rho = 0.078 , p = 0.25
0.05
18 : 7118122
18 : 7118092
18 : 7118077
18 : 7117790
18 : 7117680
18 : 7117321
18 : 7116977
18 : 7116700
18 : 7116550
18 : 7038943
18 : 7038746
18 : 7013954
18 : 7012277
18 : 7011463
18 : 7011388
18 : 7011268
18 : 7011217
18 : 6986217
cg14950072
cg19909349
cg03159124
cg18764577
cg07846220
cg12072964
cg22455914
cg23913679
cg21628773
cg18998321
cg06511678
cg12627371
cg02827082
cg19738980
cg05836043
cg03691722
cg22220710
cg18008372
GeneLoc
PromoterAssoc
CpGIsland
0.01
1e−5
mRNA / METH Corr. FDR
MITF
SOX10
TRPM1
ZEB1
AXL
0.05
0.01
Gene / Regulator Corr. P
Type
mRNA_Expr
CopyNum
AmpDel
