# Supplementary material for: Multi-modal molecular programs regulate melanoma cell state
Source: Nat Commun. 2022 Jul 9;13:4000. doi: 10.1038/s41467-022-31510-1 (PMC9271073; doi:10.1038/s41467-022-31510-1)
Supplement: Supplementary file 12 — Supplementary Data 9 [file 41467_2022_31510_MOESM12_ESM.pdf]

Supplementary Data 9: MES gene regulatory influences - MDACC cell lines.

EGFR

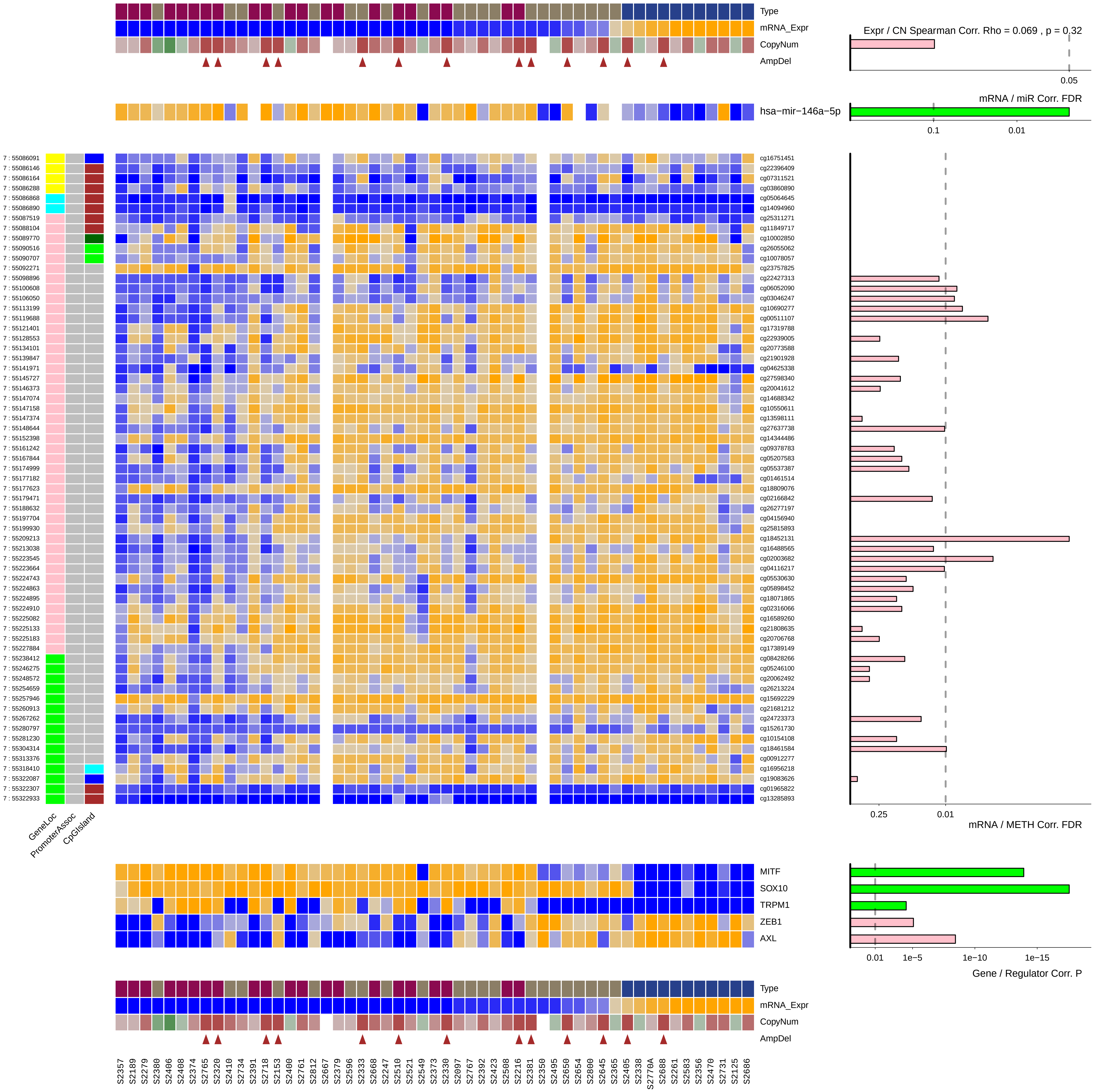

Type
mRNA_Expr
CopyNum
AmpDel
Expr / CN Spearman Corr. Rho = 0.069 , p = 0.32
0.05
hsa−mir−146a−5p
mRNA / miR Corr. FDR
0.1
0.01
7 : 55086091
7 : 55086146
7 : 55086164
7 : 55086288
7 : 55086868
7 : 55086890
7 : 55087519
7 : 55088104
7 : 55089770
7 : 55090516
7 : 55090707
7 : 55092271
7 : 55098896
7 : 55100608
7 : 55106050
7 : 55113199
7 : 55119688
7 : 55121401
7 : 55128553
7 : 55134101
7 : 55139847
7 : 55141971
7 : 55145727
7 : 55146373
7 : 55147074
7 : 55147158
7 : 55147374
7 : 55148644
7 : 55152398
7 : 55161242
7 : 55167844
7 : 55174999
7 : 55177182
7 : 55177623
7 : 55179471
7 : 55188632
7 : 55197704
7 : 55199930
7 : 55209213
7 : 55213038
7 : 55223545
7 : 55223664
7 : 55224743
7 : 55224863
7 : 55224895
7 : 55224910
7 : 55225082
7 : 55225133
7 : 55225183
7 : 55227884
7 : 55238412
7 : 55246275
7 : 55248572
7 : 55254659
7 : 55257946
7 : 55260913
7 : 55267262
7 : 55280797
7 : 55281230
7 : 55304314
7 : 55313376
7 : 55318410
7 : 55322087
7 : 55322307
7 : 55322933
cg16751451
cg22396409
cg07311521
cg03860890
cg05064645
cg14094960
cg25311271
cg11849717
cg10002850
cg26055062
cg10078057
cg23757825
cg22427313
cg06052090
cg03046247
cg10690277
cg00511107
cg17319788
cg22939005
cg20773588
cg21901928
cg04625338
cg27598340
cg20041612
cg14688342
cg10550611
cg13598111
cg27637738
cg14344486
cg09378783
cg05207583
cg05537387
cg01461514
cg18809076
cg02166842
cg26277197
cg04156940
cg25815893
cg18452131
cg16488565
cg02003682
cg04116217
cg05530630
cg05898452
cg18071865
cg02316066
cg16589260
cg21808635
cg20706768
cg17389149
cg08428266
cg05246100
cg20062492
cg26213224
cg15692229
cg21681212
cg24723373
cg15261730
cg10154108
cg18461584
cg00912277
cg16956218
cg19083626
cg01965822
cg13285893
0.25
0.01
mRNA / METH Corr. FDR
GeneLoc
PromoterAssoc
CpGIsland
MITF
SOX10
TRPM1
ZEB1
AXL
0.01
1e−5
1e−10
1e−15
Gene / Regulator Corr. P
Type
mRNA_Expr
CopyNum
AmpDel
S2357
S2189
S2279
S2380
S2406
S2408
S2374
S2765
S2320
S2410
S2734
S2391
S2718
S2153
S2400
S2761
S2812
S2667
S2379
S2596
S2333
S2668
S2247
S2510
S2521
S2549
S2373
S2330
S2097
S2767
S2392
S2423
S2508
S2216
S2381
S2350
S2495
S2650
S2654
S2800
S2645
S2365
S2405
S2338
S2770A
S2688
S2261
S2583
S2356
S2470
S2731
S2125
S2686

TSPAN5

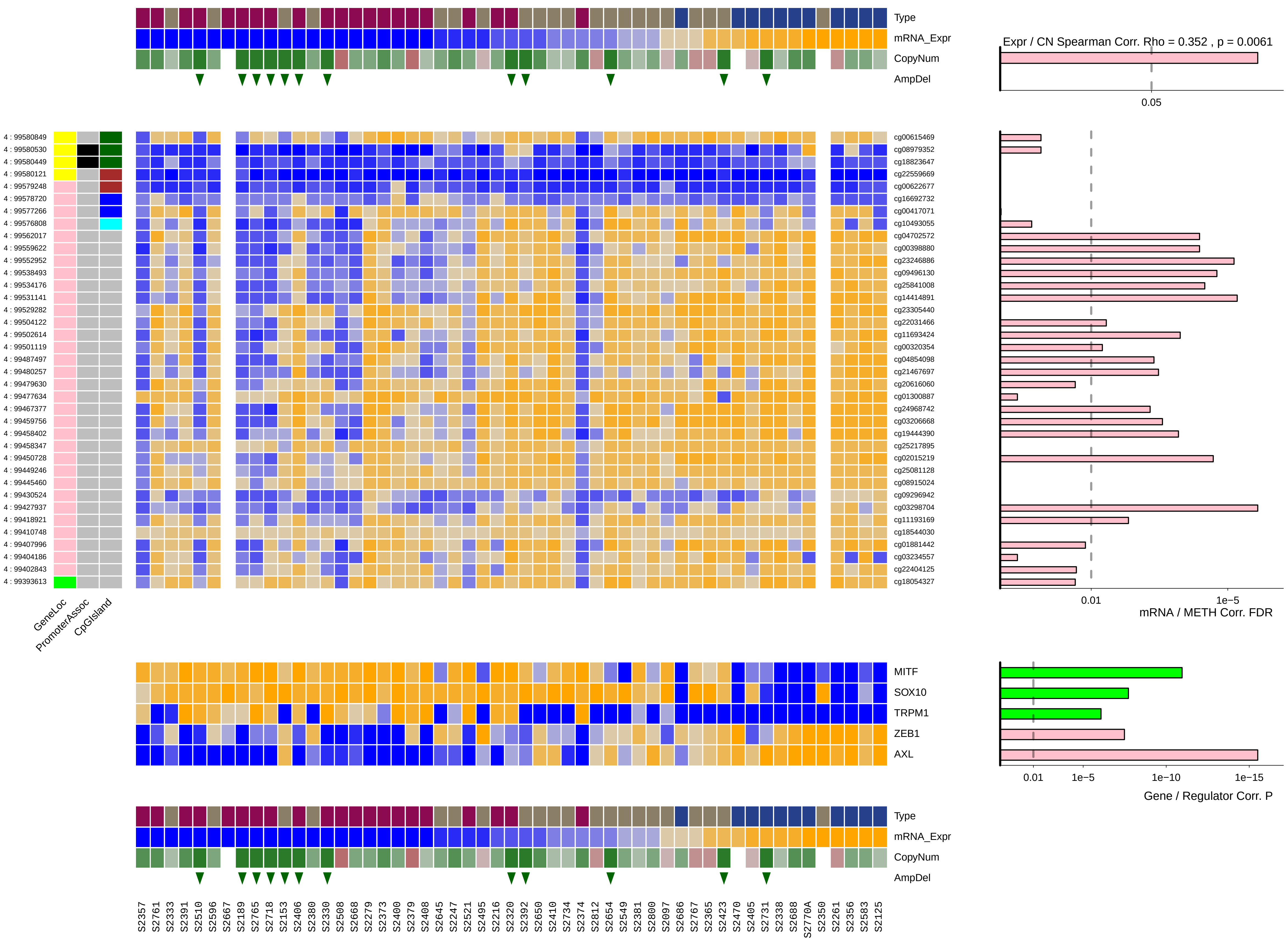
Type
mRNA_Expr
CopyNum
AmpDel
Expr / CN Spearman Corr. Rho = 0.352 , p = 0.0061
0.05
GeneLoc
PromoterAssoc
CpGIsland
0.01
1e−5
mRNA / METH Corr. FDR
MITF
SOX10
TRPM1
ZEB1
AXL
0.01
1e−5
1e−10
1e−15
Gene / Regulator Corr. P
S2357 S2761 S2333 S2391 S2510 S2596 S2667 S2189 S2765 S2718 S2153 S2406 S2380 S2330 S2508 S2668 S2279 S2373 S2400 S2379 S2408 S2645 S2247 S2521 S2495 S2216 S2320 S2392 S2650 S2410 S2734 S2374 S2812 S2654 S2549 S2381 S2800 S2097 S2686 S2767 S2365 S2423 S2470 S2405 S2731 S2338 S2688 S2770A S2350 S2261 S2356 S2583 S2125

NRP1

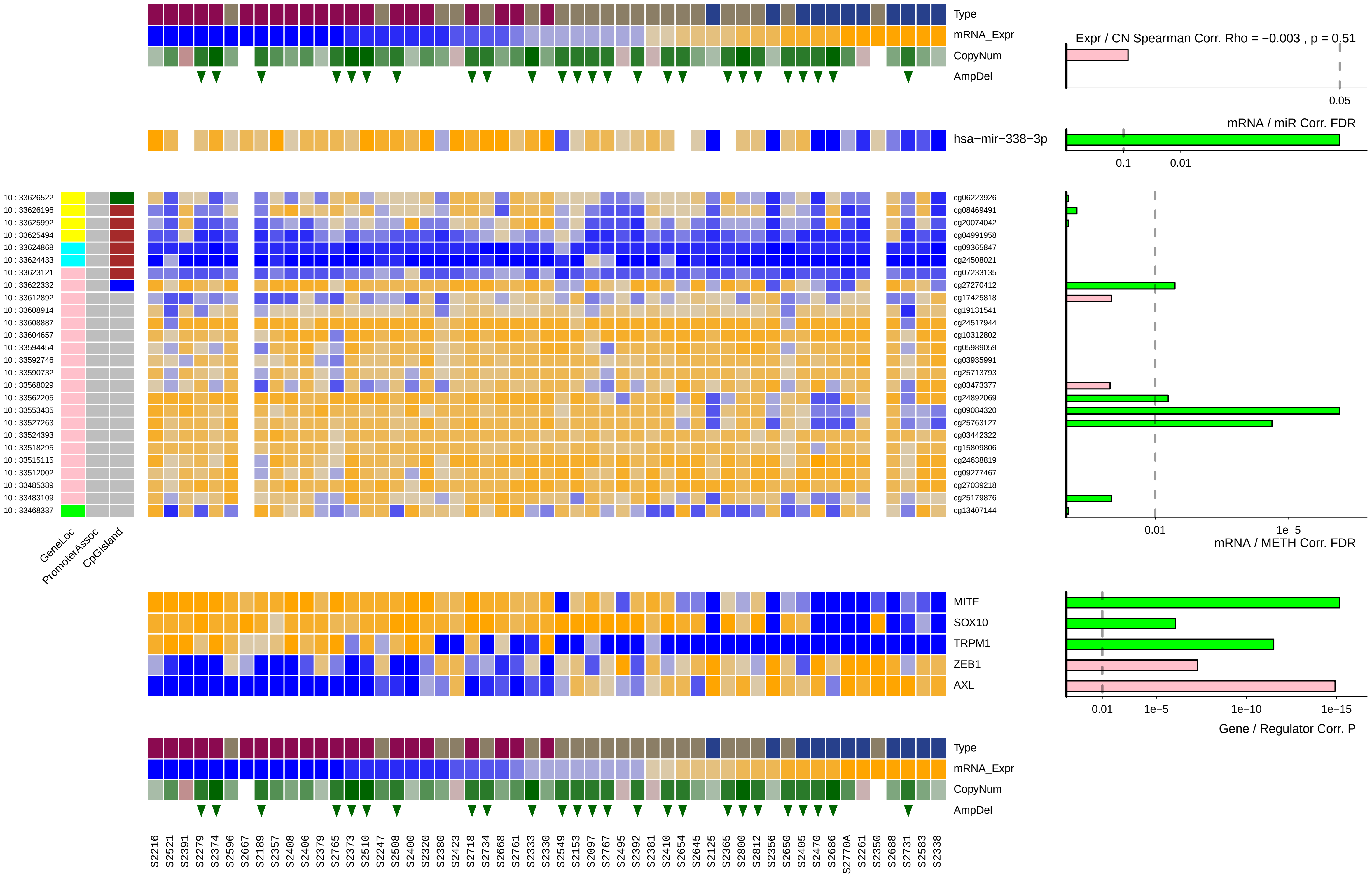

Type
mRNA_Expr
CopyNum
AmpDel
Expr / CN Spearman Corr. Rho = −0.003 , p = 0.51
0.05
mRNA / miR Corr. FDR
hsa−mir−338−3p
0.1
0.01
mRNA / METH Corr. FDR
1e−5
GeneLoc
PromoterAssoc
CpGIsland
MITF
SOX10
TRPM1
ZEB1
AXL
Gene / Regulator Corr. P
1e−10
1e−15

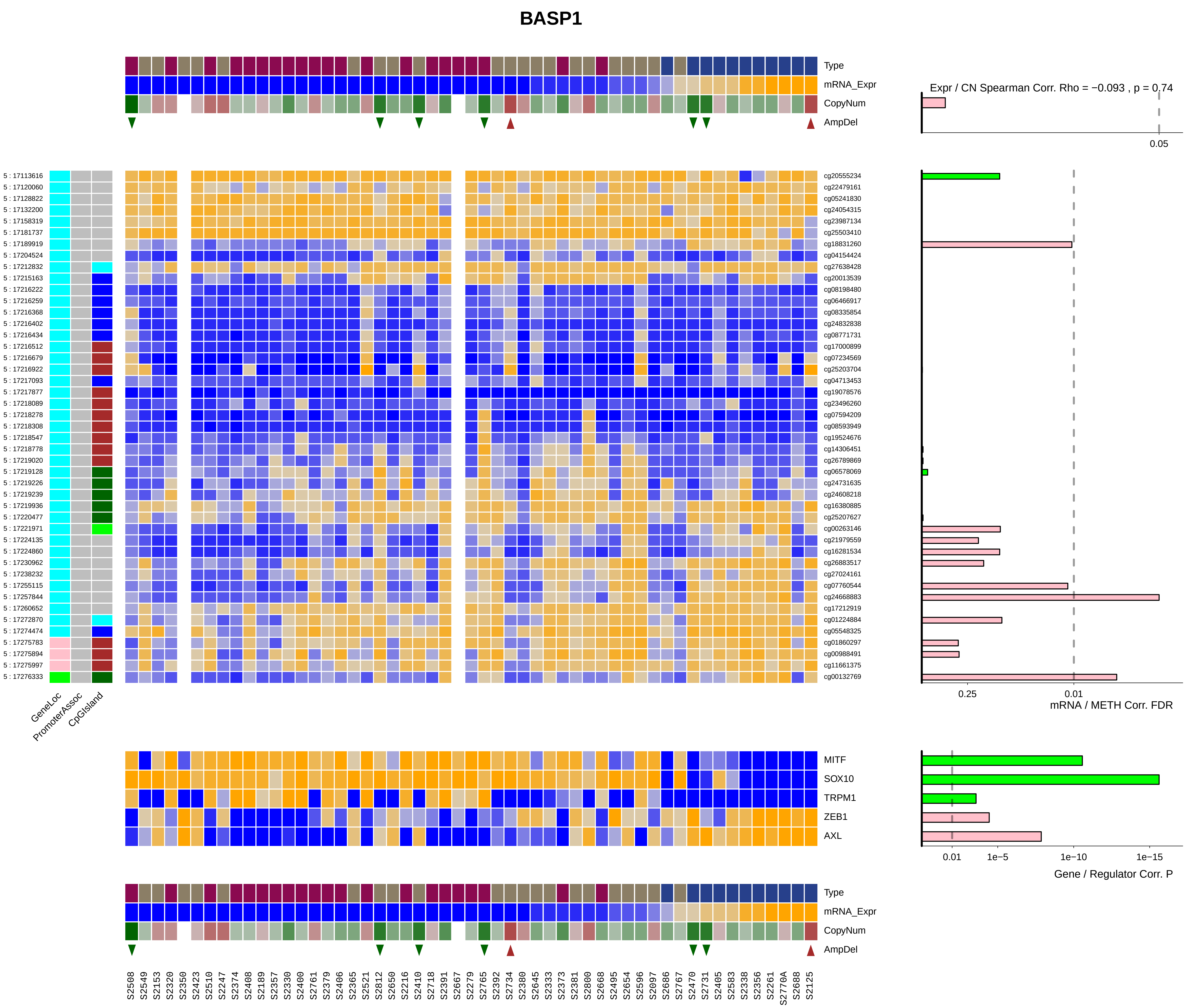
BASP1
Type
mRNA_Expr
CopyNum
AmpDel
Expr / CN Spearman Corr. Rho = −0.093 , p = 0.74
0.05
5 : 17113616
5 : 17120060
5 : 17128822
5 : 17132200
5 : 17158319
5 : 17181737
5 : 17189919
5 : 17204524
5 : 17212832
5 : 17215163
5 : 17216222
5 : 17216259
5 : 17216368
5 : 17216402
5 : 17216434
5 : 17216512
5 : 17216679
5 : 17216922
5 : 17217093
5 : 17217877
5 : 17218089
5 : 17218278
5 : 17218308
5 : 17218547
5 : 17218778
5 : 17219020
5 : 17219128
5 : 17219226
5 : 17219239
5 : 17219936
5 : 17220477
5 : 17221971
5 : 17224135
5 : 17224860
5 : 17230962
5 : 17238232
5 : 17255115
5 : 17257844
5 : 17260652
5 : 17272870
5 : 17274474
5 : 17275783
5 : 17275894
5 : 17275997
5 : 17276333
cg20555234
cg22479161
cg05241830
cg24054315
cg23987134
cg25503410
cg18831260
cg04154424
cg27638428
cg20013539
cg08198480
cg06466917
cg08335854
cg24832838
cg08771731
cg17000899
cg07234569
cg25203704
cg04713453
cg19078576
cg23496260
cg07594209
cg08593949
cg19524676
cg14306451
cg26789869
cg06578069
cg24731635
cg24608218
cg16380885
cg25207627
cg00263146
cg21979559
cg16281534
cg26883517
cg27024161
cg07760544
cg24668883
cg17212919
cg01224884
cg05548325
cg01860297
cg00988491
cg11661375
cg00132769
0.25
0.01
mRNA / METH Corr. FDR
GeneLoc
PromoterAssoc
CpGIsland
MITF
SOX10
TRPM1
ZEB1
AXL
0.01
1e−5
1e−10
1e−15
Gene / Regulator Corr. P
Type
mRNA_Expr
CopyNum
AmpDel
S2508
S2549
S2153
S2320
S2350
S2423
S2510
S2247
S2374
S2408
S2189
S2357
S2330
S2400
S2761
S2379
S2406
S2365
S2521
S2812
S2650
S2216
S2410
S2718
S2391
S2667
S2279
S2765
S2392
S2734
S2380
S2645
S2333
S2373
S2381
S2800
S2668
S2495
S2654
S2596
S2097
S2686
S2767
S2470
S2731
S2405
S2583
S2338
S2356
S2261
S2770A
S2688
S2125

TMEM200A

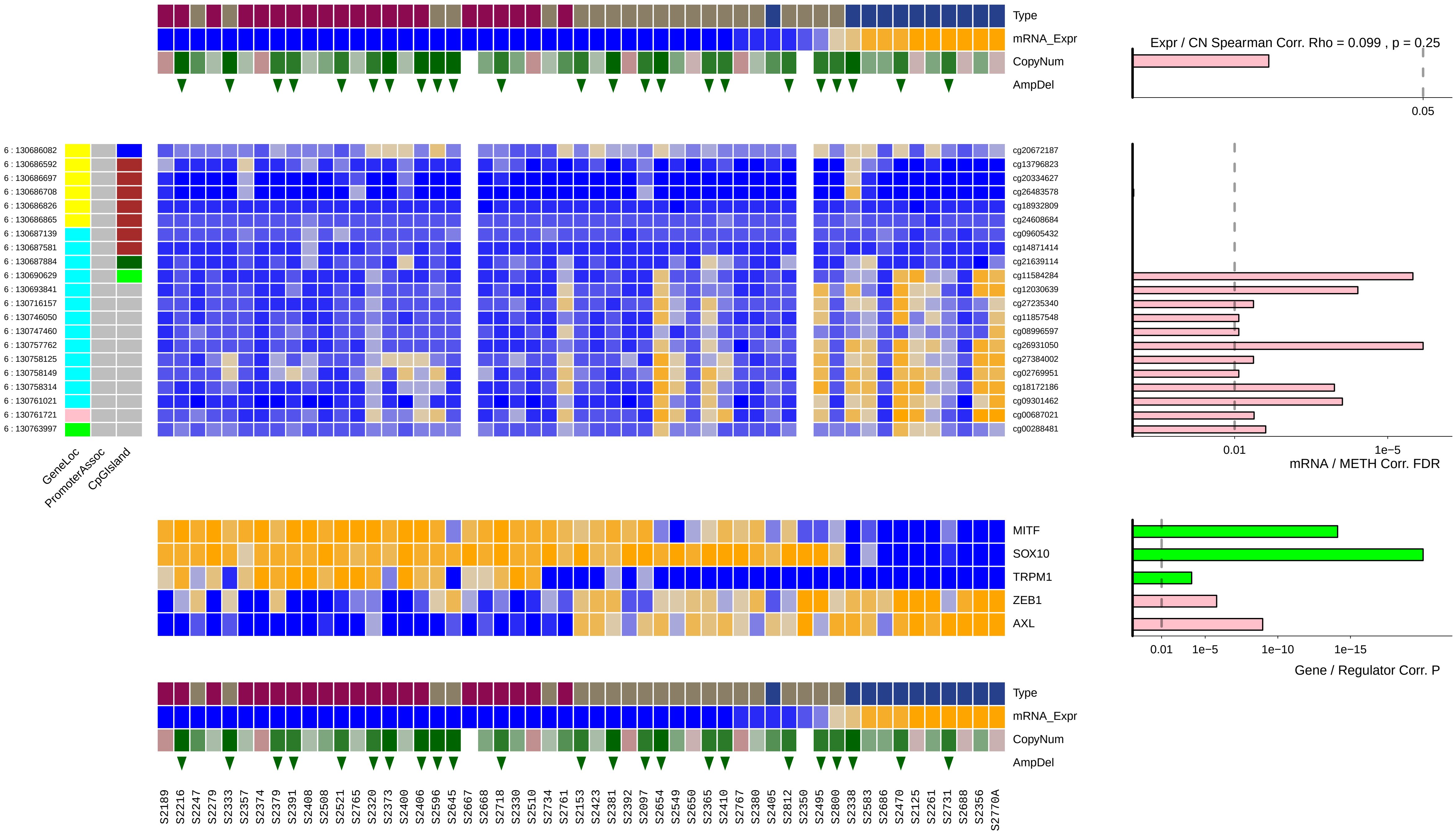

Type
mRNA_Expr
CopyNum
AmpDel
Expr / CN Spearman Corr. Rho = 0.099 , p = 0.25
0.05
6 : 130686082
6 : 130686592
6 : 130686697
6 : 130686708
6 : 130686826
6 : 130686865
6 : 130687139
6 : 130687581
6 : 130687884
6 : 130690629
6 : 130693841
6 : 130716157
6 : 130746050
6 : 130747460
6 : 130757762
6 : 130758125
6 : 130758149
6 : 130758314
6 : 130761021
6 : 130761721
6 : 130763997
cg20672187
cg13796823
cg20334627
cg26483578
cg18932809
cg24608684
cg09605432
cg14871414
cg21639114
cg11584284
cg12030639
cg27235340
cg11857548
cg08996597
cg26931050
cg27384002
cg02769951
cg18172186
cg09301462
cg00687021
cg00288481
GeneLoc
PromoterAssoc
CpGIsland
0.01
1e−5
mRNA / METH Corr. FDR
MITF
SOX10
TRPM1
ZEB1
AXL
0.01
1e−5
1e−10
1e−15
Gene / Regulator Corr. P
Type
mRNA_Expr
CopyNum
AmpDel
S2189 S2216 S2247 S2279 S2333 S2357 S2374 S2379 S2391 S2408 S2508 S2521 S2765 S2320 S2373 S2400 S2406 S2596 S2645 S2667 S2668 S2718 S2330 S2510 S2734 S2761 S2153 S2423 S2381 S2392 S2097 S2654 S2549 S2650 S2365 S2410 S2767 S2380 S2405 S2812 S2350 S2495 S2800 S2338 S2583 S2686 S2470 S2125 S2261 S2731 S2688 S2356 S2770A

LOXL2

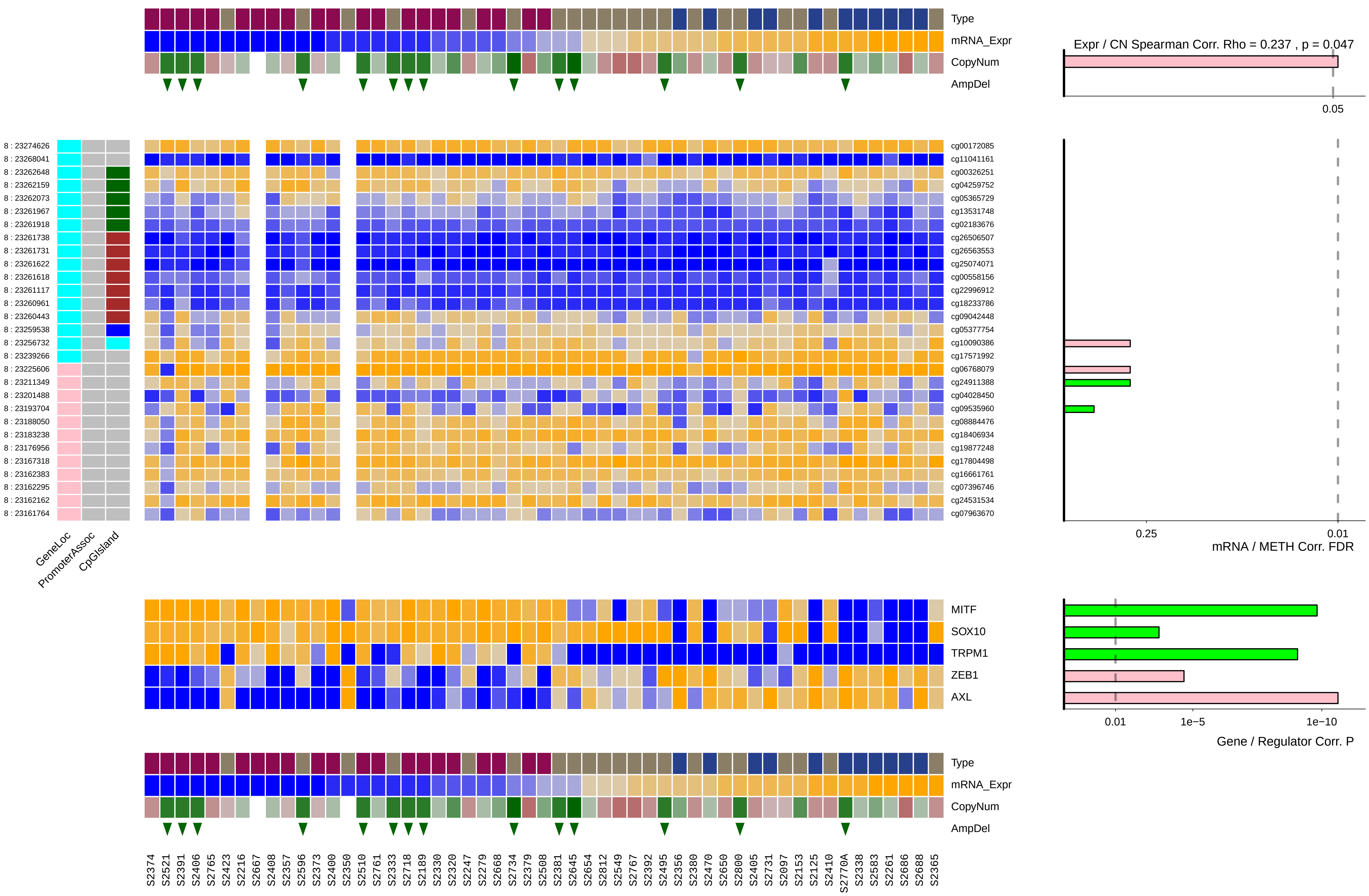

Type
mRNA_Expr
CopyNum
AmpDel
Expr / CN Spearman Corr. Rho = 0.237 , p = 0.047
0.05
8 : 23274626
8 : 23268041
8 : 23262648
8 : 23262159
8 : 23262073
8 : 23261967
8 : 23261918
8 : 23261738
8 : 23261731
8 : 23261622
8 : 23261618
8 : 23261117
8 : 23260961
8 : 23260443
8 : 23259538
8 : 23256732
8 : 23239266
8 : 23225606
8 : 23211349
8 : 23201488
8 : 23193704
8 : 23188050
8 : 23183238
8 : 23176956
8 : 23167318
8 : 23162383
8 : 23162295
8 : 23162162
8 : 23161764
cg00172085
cg11041161
cg00326251
cg04259752
cg05365729
cg13531748
cg02183676
cg26506507
cg26563553
cg25074071
cg00558156
cg22996912
cg18233786
cg09042448
cg05377754
cg10090386
cg17571992
cg06768079
cg24911388
cg04028450
cg09535960
cg08884476
cg18406934
cg19877248
cg17804498
cg16661761
cg07396746
cg24531534
cg07963670
0.25
0.01
mRNA / METH Corr. FDR
GeneLoc
PromoterAssoc
CpGIsland
MITF
SOX10
TRPM1
ZEB1
AXL
0.01
1e−5
1e−10
Gene / Regulator Corr. P
Type
mRNA_Expr
CopyNum
AmpDel
S2374 S2521 S2391 S2406 S2765 S2423 S2216 S2667 S2408 S2357 S2596 S2373 S2400 S2350 S2510 S2761 S2333 S2718 S2189 S2330 S2320 S2247 S2279 S2668 S2734 S2379 S2508 S2381 S2645 S2654 S2812 S2549 S2767 S2392 S2495 S2356 S2380 S2470 S2650 S2800 S2405 S2731 S2097 S2153 S2125 S2410 S2770A S2338 S2583 S2261 S2686 S2688 S2365

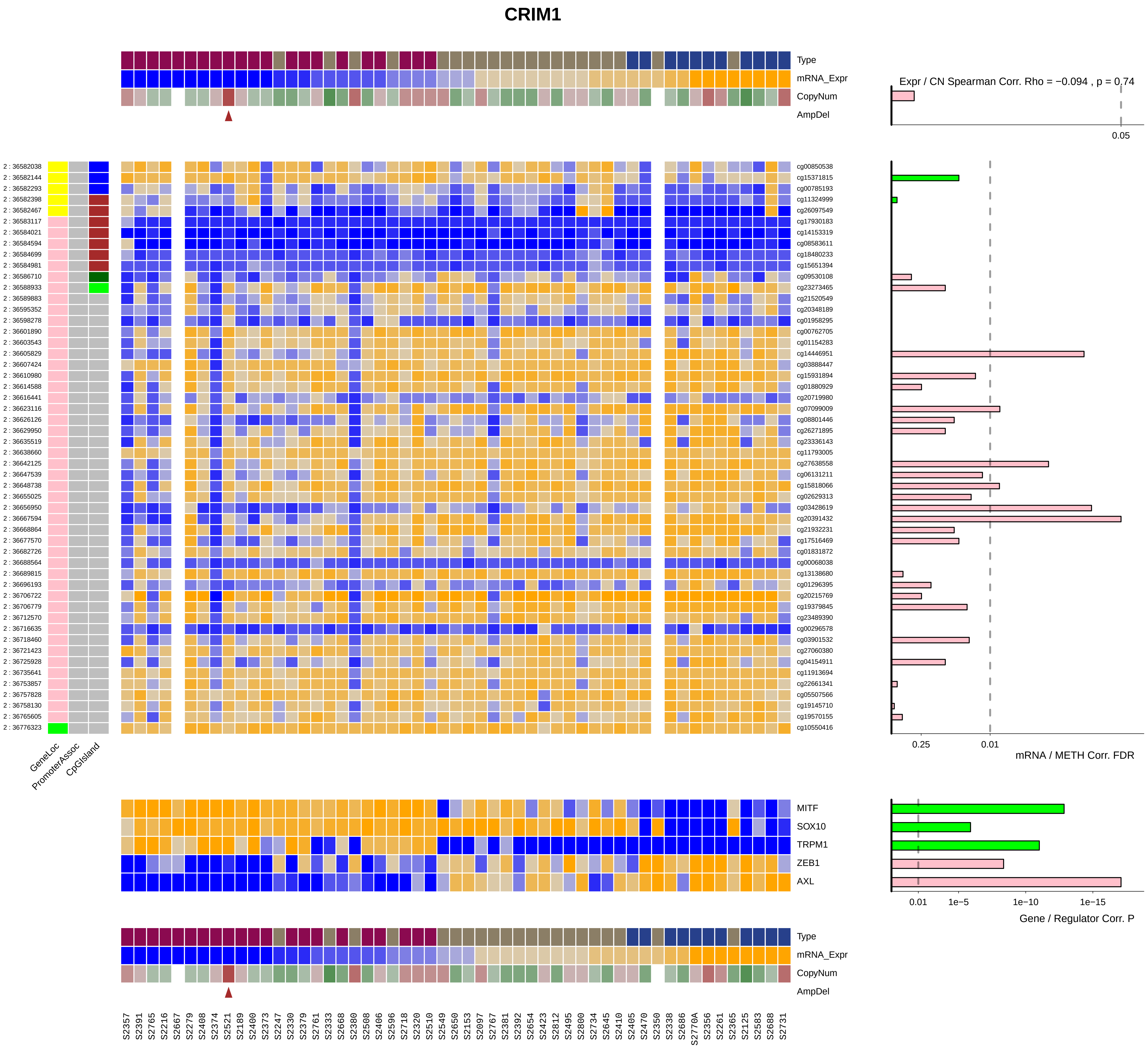

CRIM1
Type
mRNA_Expr
CopyNum
AmpDel
Expr / CN Spearman Corr. Rho = −0.094 , p = 0.74
0.05
2 : 36582038
2 : 36582144
2 : 36582293
2 : 36582398
2 : 36582467
2 : 36583117
2 : 36584021
2 : 36584594
2 : 36584699
2 : 36584981
2 : 36586710
2 : 36588933
2 : 36589883
2 : 36595352
2 : 36598278
2 : 36601890
2 : 36603543
2 : 36605829
2 : 36607424
2 : 36610980
2 : 36614588
2 : 36616441
2 : 36623116
2 : 36626126
2 : 36629950
2 : 36635519
2 : 36638660
2 : 36642125
2 : 36647539
2 : 36648738
2 : 36655025
2 : 36656950
2 : 36667594
2 : 36668864
2 : 36677570
2 : 36682726
2 : 36688564
2 : 36689815
2 : 36696193
2 : 36706722
2 : 36706779
2 : 36712570
2 : 36716635
2 : 36718460
2 : 36721423
2 : 36725928
2 : 36735641
2 : 36753857
2 : 36757828
2 : 36758130
2 : 36765605
2 : 36776323
cg00850538
cg15371815
cg00785193
cg11324999
cg26097549
cg17930183
cg14153319
cg08583611
cg18480233
cg15651394
cg09530108
cg23273465
cg21520549
cg20348189
cg01958295
cg00762705
cg01154283
cg14446951
cg03888447
cg15931894
cg01880929
cg20719980
cg07099009
cg08801446
cg26271895
cg23336143
cg11793005
cg27638558
cg06131211
cg15818066
cg02629313
cg03428619
cg20391432
cg21932231
cg17516469
cg01831872
cg00068038
cg13138680
cg01296395
cg20215769
cg19379845
cg23489390
cg00296578
cg03901532
cg27060380
cg04154911
cg11913694
cg22661341
cg05507566
cg19145710
cg19570155
cg10550416
GeneLoc
PromoterAssoc
CpGIsland
0.25
0.01
mRNA / METH Corr. FDR
MITF
SOX10
TRPM1
ZEB1
AXL
0.01
1e−5
1e−10
1e−15
Gene / Regulator Corr. P
Type
mRNA_Expr
CopyNum
AmpDel
S2357
S2391
S2765
S2216
S2667
S2279
S2408
S2374
S2521
S2189
S2400
S2373
S2247
S2330
S2379
S2761
S2333
S2668
S2380
S2508
S2406
S2596
S2718
S2320
S2510
S2549
S2650
S2153
S2097
S2767
S2381
S2392
S2654
S2423
S2812
S2495
S2800
S2734
S2645
S2410
S2405
S2470
S2350
S2338
S2686
S2770A
S2356
S2261
S2365
S2125
S2583
S2688
S2731

ANTXR2

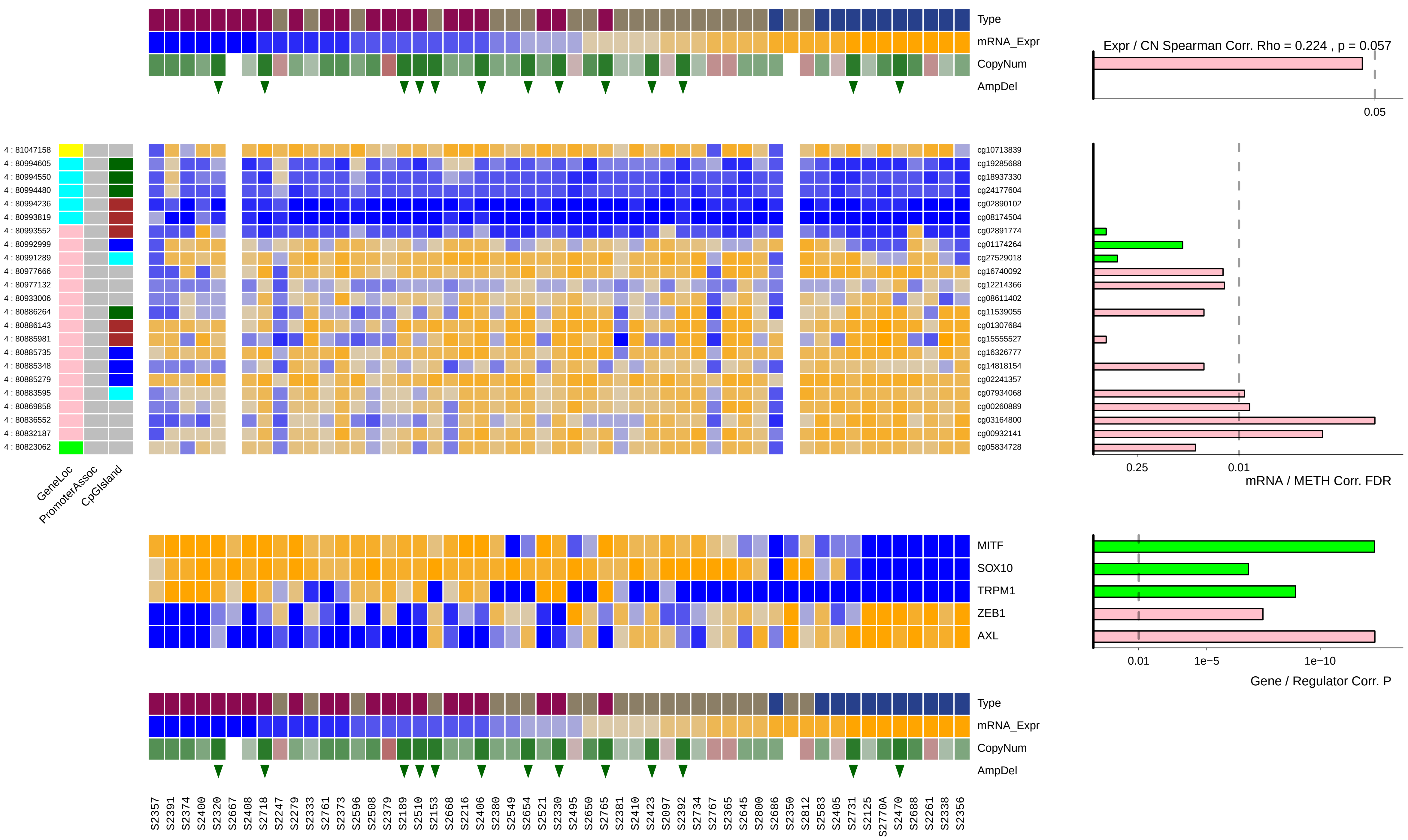

Type
mRNA_Expr
CopyNum
AmpDel
Expr / CN Spearman Corr. Rho = 0.224 , p = 0.057
0.05
4 : 81047158
4 : 80994605
4 : 80994550
4 : 80994480
4 : 80994236
4 : 80993819
4 : 80993552
4 : 80992999
4 : 80991289
4 : 80977666
4 : 80977132
4 : 80933006
4 : 80886264
4 : 80886143
4 : 80885981
4 : 80885735
4 : 80885348
4 : 80885279
4 : 80883595
4 : 80869858
4 : 80836552
4 : 80832187
4 : 80823062
cg10713839
cg19285688
cg18937330
cg24177604
cg02890102
cg08174504
cg02891774
cg01174264
cg27529018
cg16740092
cg12214366
cg08611402
cg11539055
cg01307684
cg15555527
cg16326777
cg14818154
cg02241357
cg07934068
cg00260889
cg03164800
cg00932141
cg05834728
GeneLoc
PromoterAssoc
CpGIsland
0.25
0.01
mRNA / METH Corr. FDR
MITF
SOX10
TRPM1
ZEB1
AXL
0.01
1e−5
1e−10
Gene / Regulator Corr. P
Type
mRNA_Expr
CopyNum
AmpDel
S2357
S2391
S2374
S2400
S2320
S2667
S2408
S2718
S2247
S2279
S2333
S2761
S2373
S2596
S2508
S2379
S2189
S2510
S2153
S2668
S2216
S2406
S2380
S2549
S2654
S2521
S2330
S2495
S2650
S2765
S2381
S2410
S2423
S2097
S2392
S2734
S2767
S2365
S2645
S2800
S2686
S2350
S2812
S2583
S2405
S2731
S2125
S2770A
S2470
S2688
S2261
S2338
S2356

KCNMA1

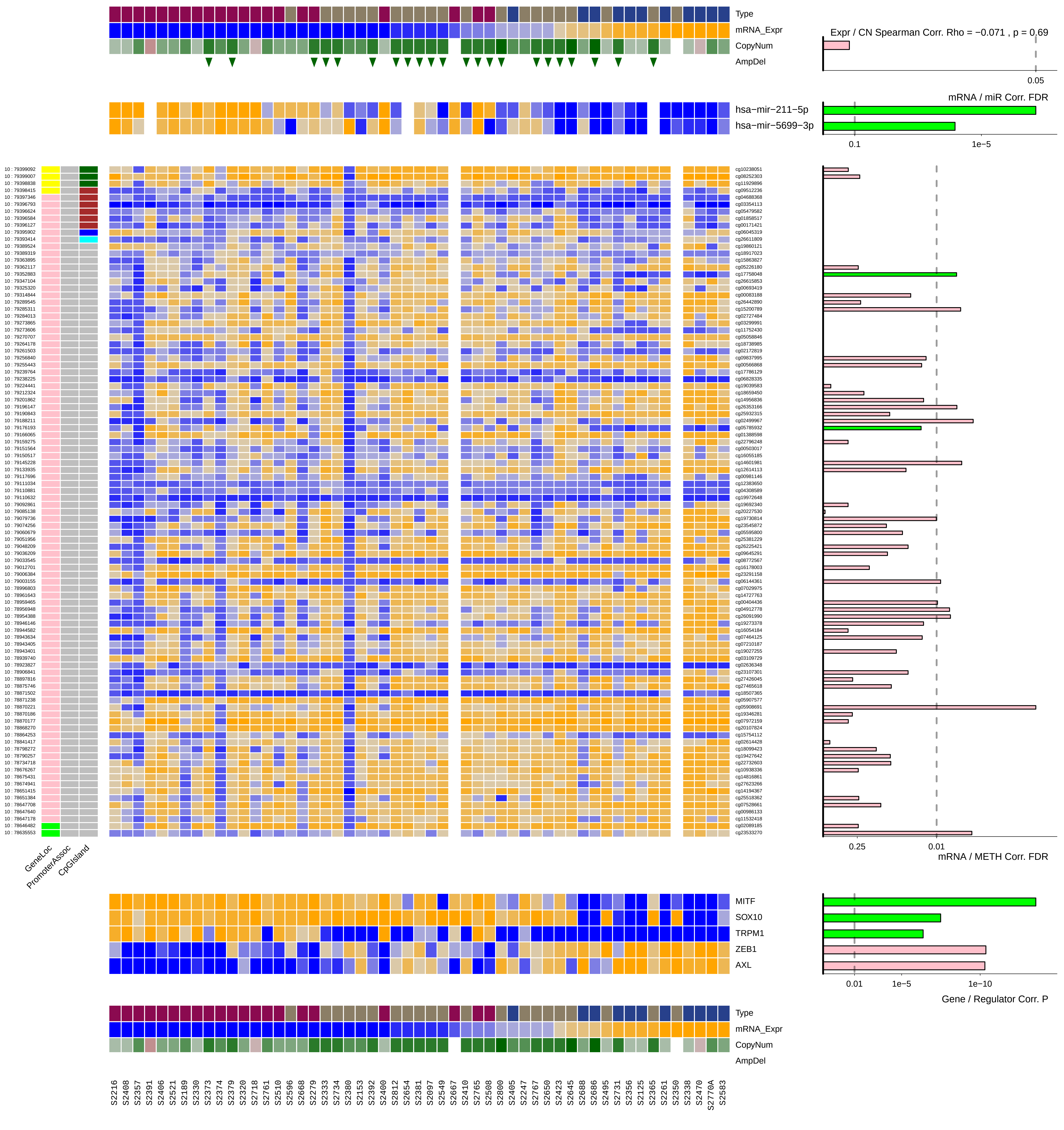

Type
mRNA_Expr
CopyNum
AmpDel
Expr / CN Spearman Corr. Rho = −0.071 , p = 0.69
0.05
mRNA / miR Corr. FDR
hsa−mir−211−5p
hsa−mir−5699−3p
0.1
1e−5
0.25
0.01
mRNA / METH Corr. FDR
GeneLoc
PromoterAssoc
CpGIsland
MITF
SOX10
TRPM1
ZEB1
AXL
0.01
1e−5
1e−10
Gene / Regulator Corr. P
S2216
S2408
S2357
S2391
S2406
S2521
S2189
S2330
S2373
S2374
S2379
S2320
S2718
S2761
S2510
S2596
S2668
S2279
S2333
S2734
S2380
S2153
S2392
S2400
S2812
S2654
S2381
S2097
S2549
S2667
S2410
S2765
S2508
S2800
S2405
S2247
S2767
S2650
S2423
S2645
S2688
S2686
S2495
S2731
S2356
S2125
S2365
S2261
S2350
S2338
S2470
S2770A
S2583

AXL

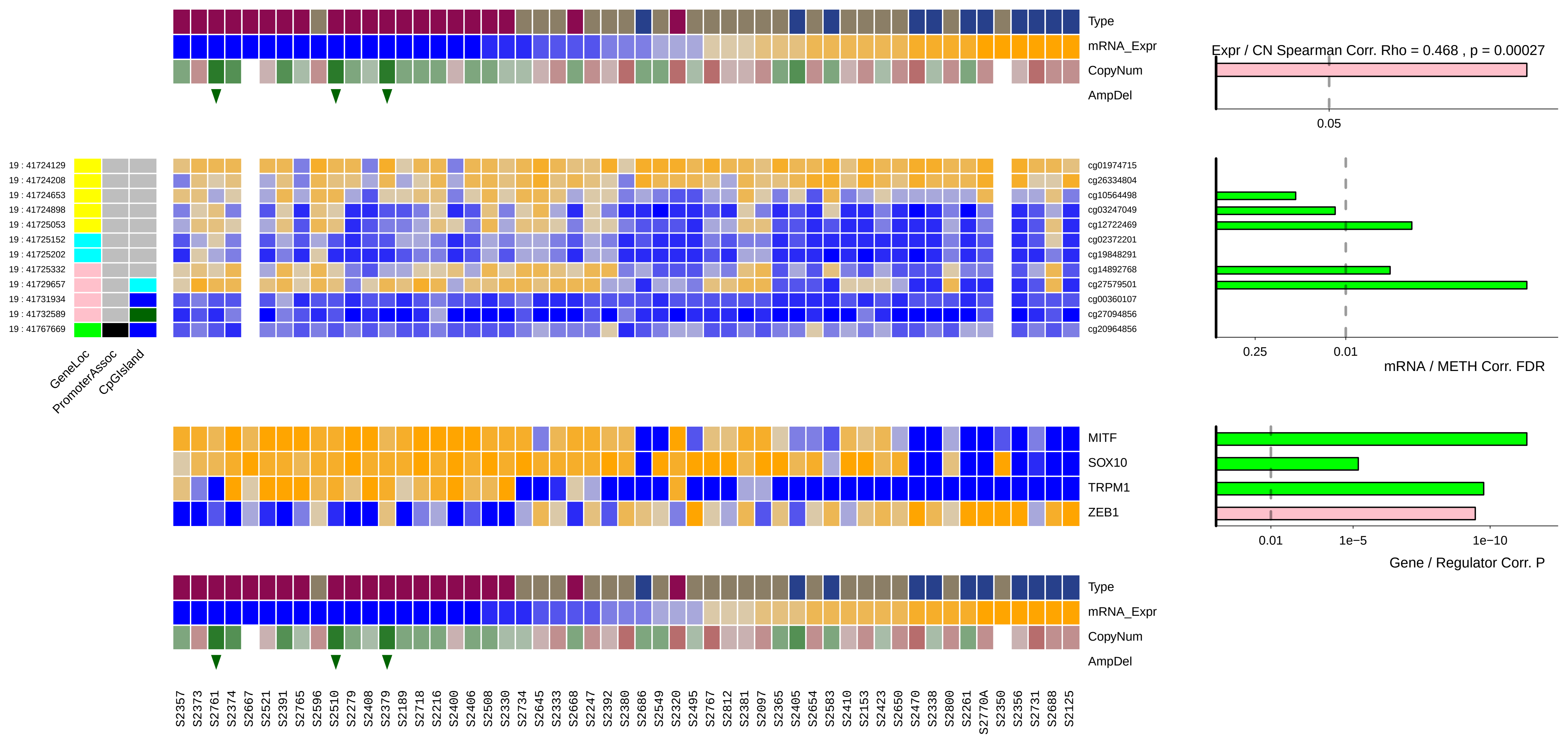

Type
mRNA_Expr
CopyNum
AmpDel
Expr / CN Spearman Corr. Rho = 0.468 , p = 0.00027
0.05
19 : 41724129
19 : 41724208
19 : 41724653
19 : 41724898
19 : 41725053
19 : 41725152
19 : 41725202
19 : 41725332
19 : 41729657
19 : 41731934
19 : 41732589
19 : 41767669
cg01974715
cg26334804
cg10564498
cg03247049
cg12722469
cg02372201
cg19848291
cg14892768
cg27579501
cg00360107
cg27094856
cg20964856
0.25
0.01
mRNA / METH Corr. FDR
GeneLoc
PromoterAssoc
CpGIsland
MITF
SOX10
TRPM1
ZEB1
0.01
1e−5
1e−10
Gene / Regulator Corr. P
S2357 S2373 S2761 S2374 S2667 S2521 S2391 S2765 S2596 S2510 S2279 S2408 S2379 S2189 S2718 S2216 S2400 S2406 S2508 S2330 S2734 S2645 S2333 S2668 S2247 S2392 S2380 S2686 S2549 S2320 S2495 S2767 S2812 S2381 S2097 S2365 S2405 S2654 S2583 S2410 S2153 S2423 S2650 S2470 S2338 S2800 S2261 S2770A S2350 S2356 S2731 S2688 S2125

COL13A1

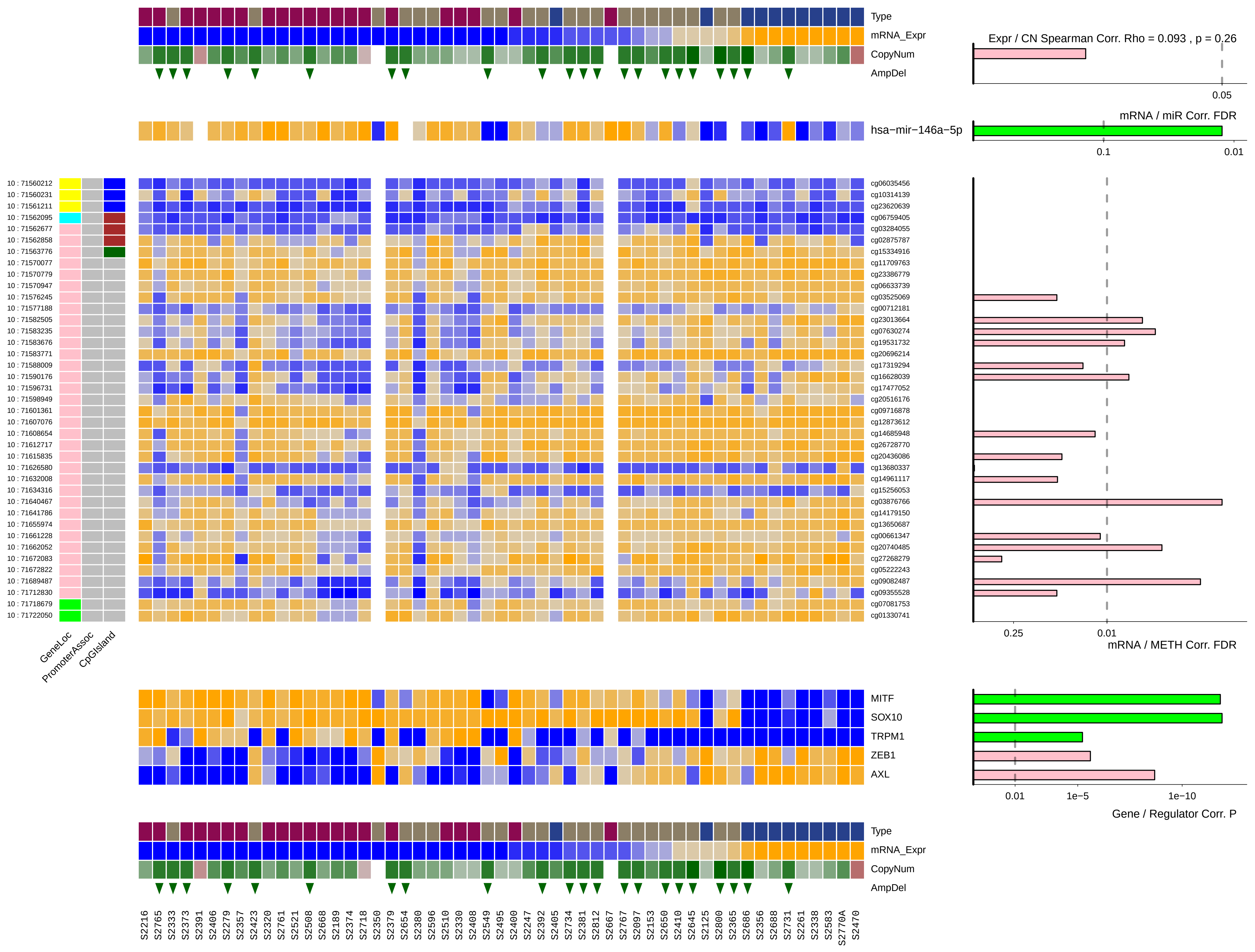

Type
mRNA_Expr
CopyNum
AmpDel
Expr / CN Spearman Corr. Rho = 0.093 , p = 0.26
0.05
hsa−mir−146a−5p
mRNA / miR Corr. FDR
0.1
0.01
GeneLoc
PromoterAssoc
CpGIsland
mRNA / METH Corr. FDR
0.25
0.01
MITF
SOX10
TRPM1
ZEB1
AXL
Gene / Regulator Corr. P
0.01
1e−5
1e−10

RIN2

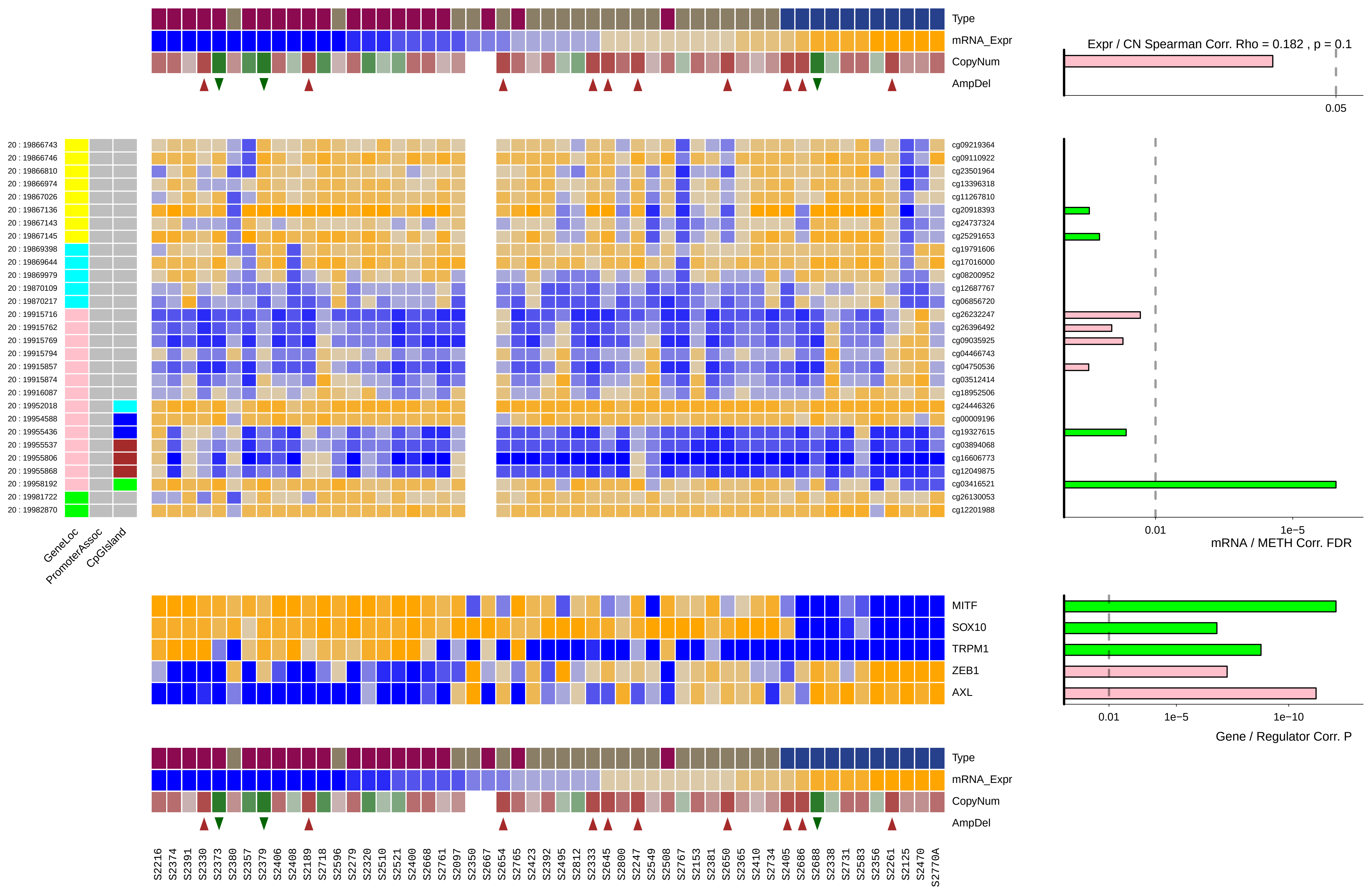

Type
mRNA_Expr
CopyNum
AmpDel
Expr / CN Spearman Corr. Rho = 0.182 , p = 0.1
0.05
20 : 19866743
20 : 19866746
20 : 19866810
20 : 19866974
20 : 19867026
20 : 19867136
20 : 19867143
20 : 19867145
20 : 19869398
20 : 19869644
20 : 19869979
20 : 19870109
20 : 19870217
20 : 19915716
20 : 19915762
20 : 19915769
20 : 19915794
20 : 19915857
20 : 19915874
20 : 19916087
20 : 19952018
20 : 19954588
20 : 19955436
20 : 19955537
20 : 19955806
20 : 19955868
20 : 19958192
20 : 19981722
20 : 19982870
GeneLoc
PromoterAssoc
CpGIsland
cg09219364
cg09110922
cg23501964
cg13396318
cg11267810
cg20918393
cg24737324
cg25291653
cg19791606
cg17016000
cg08200952
cg12687767
cg06856720
cg26232247
cg26396492
cg09035925
cg04466743
cg04750536
cg03512414
cg18952506
cg24446326
cg00009196
cg19327615
cg03894068
cg16606773
cg12049875
cg03416521
cg26130053
cg12201988
0.01
1e−5
mRNA / METH Corr. FDR
MITF
SOX10
TRPM1
ZEB1
AXL
0.01
1e−5
1e−10
Gene / Regulator Corr. P
Type
mRNA_Expr
CopyNum
AmpDel
S2216 S2374 S2391 S2330 S2373 S2380 S2357 S2379 S2406 S2408 S2189 S2718 S2596 S2279 S2320 S2510 S2521 S2400 S2668 S2761 S2097 S2350 S2667 S2654 S2765 S2423 S2392 S2495 S2812 S2333 S2645 S2800 S2247 S2549 S2508 S2767 S2153 S2381 S2650 S2365 S2410 S2734 S2405 S2686 S2688 S2338 S2731 S2583 S2356 S2261 S2125 S2470 S2770A

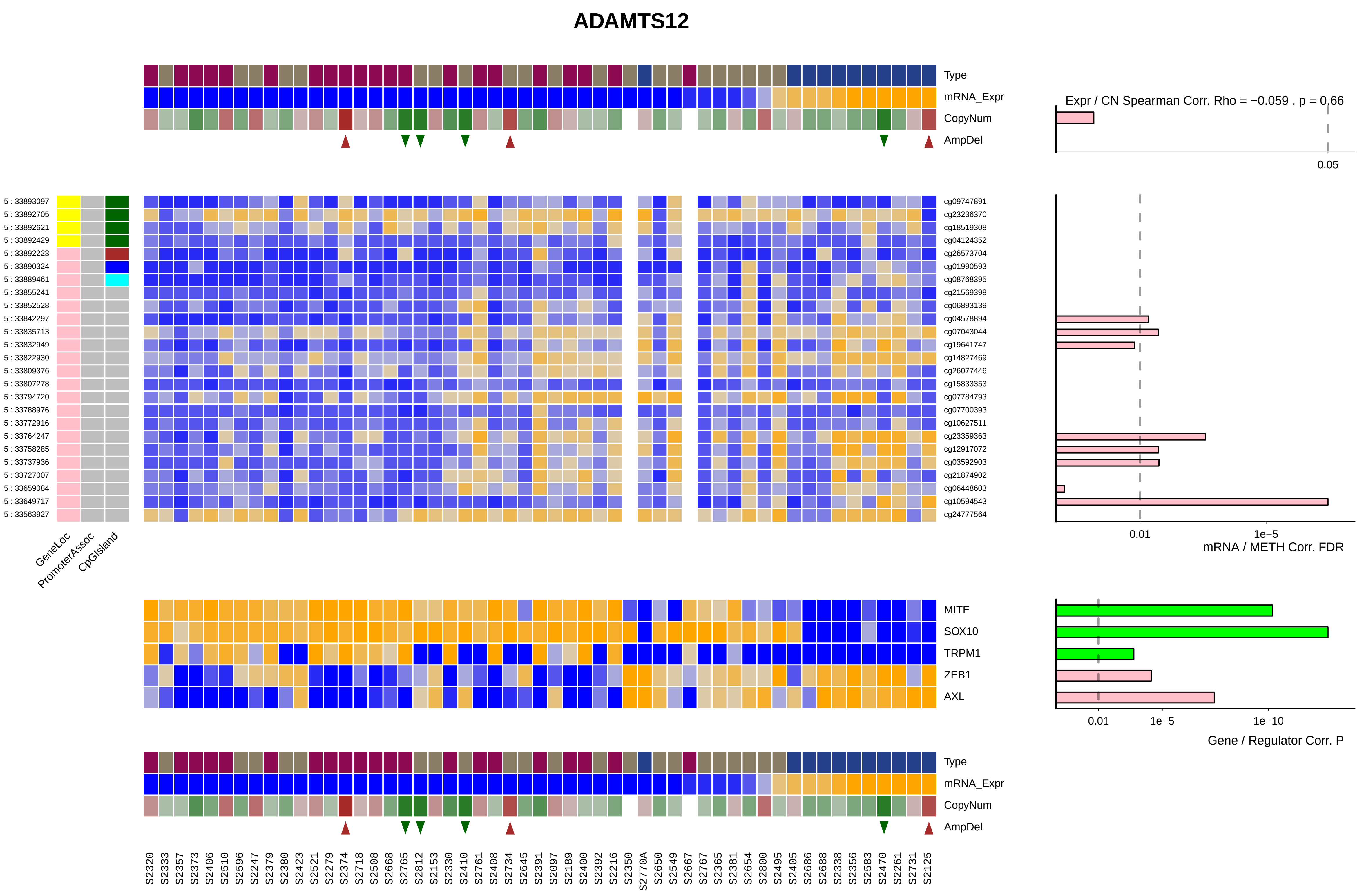
ADAMTS12
Type
mRNA_Expr
CopyNum
AmpDel
Expr / CN Spearman Corr. Rho = −0.059 , p = 0.66
0.05
GeneLoc
PromoterAssoc
CpGIsland
0.01
1e−5
mRNA / METH Corr. FDR
MITF
SOX10
TRPM1
ZEB1
AXL
1e−10
Gene / Regulator Corr. P

GPR39

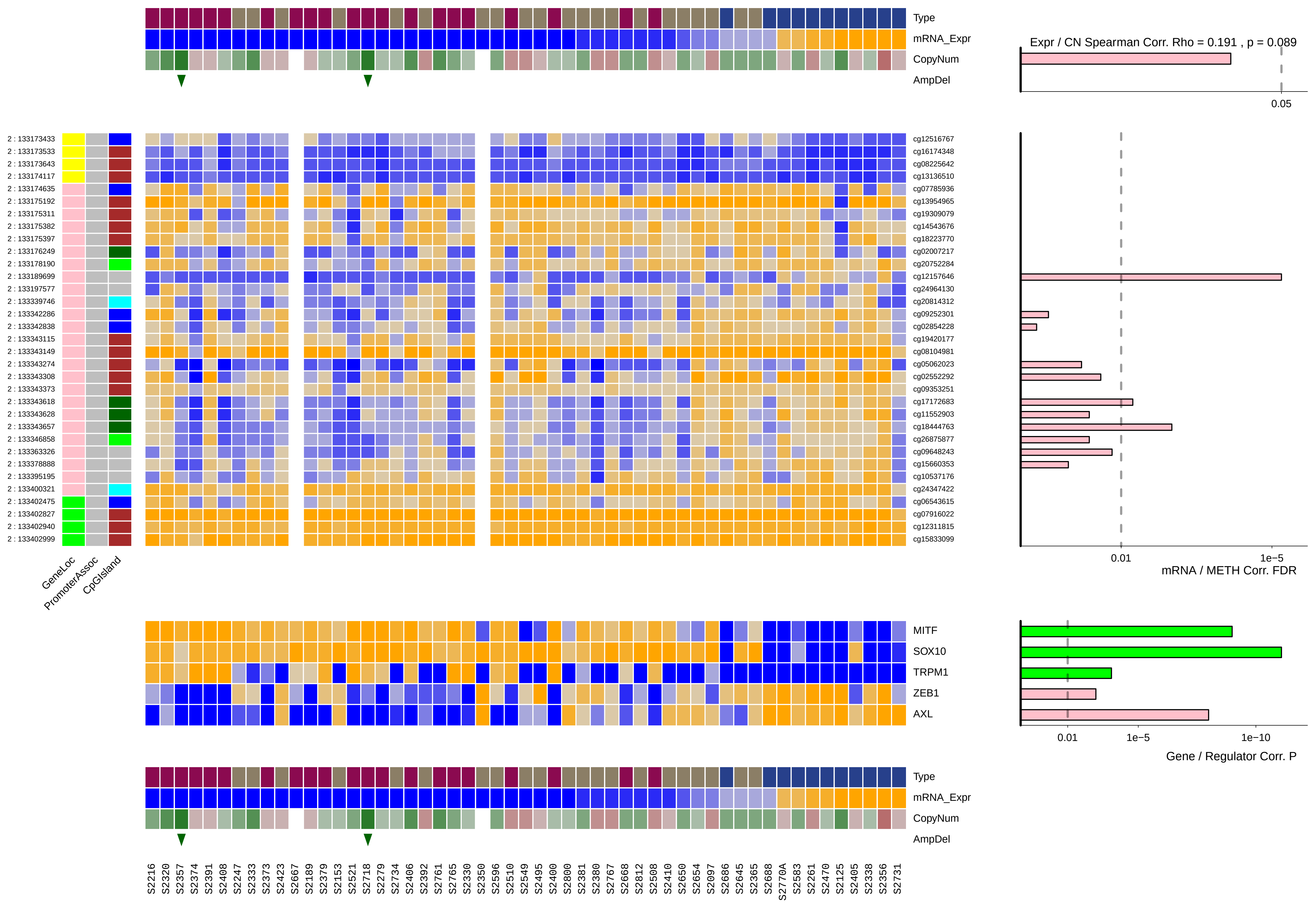
Type
mRNA_Expr
CopyNum
AmpDel
Expr / CN Spearman Corr. Rho = 0.191 , p = 0.089
0.05
2 : 133173433
2 : 133173533
2 : 133173643
2 : 133174117
2 : 133174635
2 : 133175192
2 : 133175311
2 : 133175382
2 : 133175397
2 : 133176249
2 : 133178190
2 : 133189699
2 : 133197577
2 : 133339746
2 : 133342286
2 : 133342838
2 : 133343115
2 : 133343149
2 : 133343274
2 : 133343308
2 : 133343373
2 : 133343618
2 : 133343628
2 : 133343657
2 : 133346858
2 : 133363326
2 : 133378888
2 : 133395195
2 : 133400321
2 : 133402475
2 : 133402827
2 : 133402940
2 : 133402999
cg12516767
cg16174348
cg08225642
cg13136510
cg07785936
cg13954965
cg19309079
cg14543676
cg18223770
cg02007217
cg20752284
cg12157646
cg24964130
cg20814312
cg09252301
cg02854228
cg19420177
cg08104981
cg05062023
cg02552292
cg09353251
cg17172683
cg11552903
cg18444763
cg26875877
cg09648243
cg15660353
cg10537176
cg24347422
cg06543615
cg07916022
cg12311815
cg15833099
0.01
1e−5
mRNA / METH Corr. FDR
GeneLoc
PromoterAssoc
CpGIsland
MITF
SOX10
TRPM1
ZEB1
AXL
0.01
1e−5
1e−10
Gene / Regulator Corr. P
Type
mRNA_Expr
CopyNum
AmpDel
S2216 S2320 S2357 S2374 S2391 S2408 S2247 S2333 S2373 S2423 S2667 S2189 S2379 S2153 S2521 S2718 S2279 S2734 S2406 S2392 S2761 S2765 S2330 S2350 S2596 S2510 S2549 S2495 S2400 S2800 S2381 S2380 S2767 S2668 S2812 S2508 S2410 S2650 S2654 S2097 S2686 S2645 S2365 S2688 S2770A S2583 S2261 S2470 S2125 S2405 S2338 S2356 S2731

SERPINE1

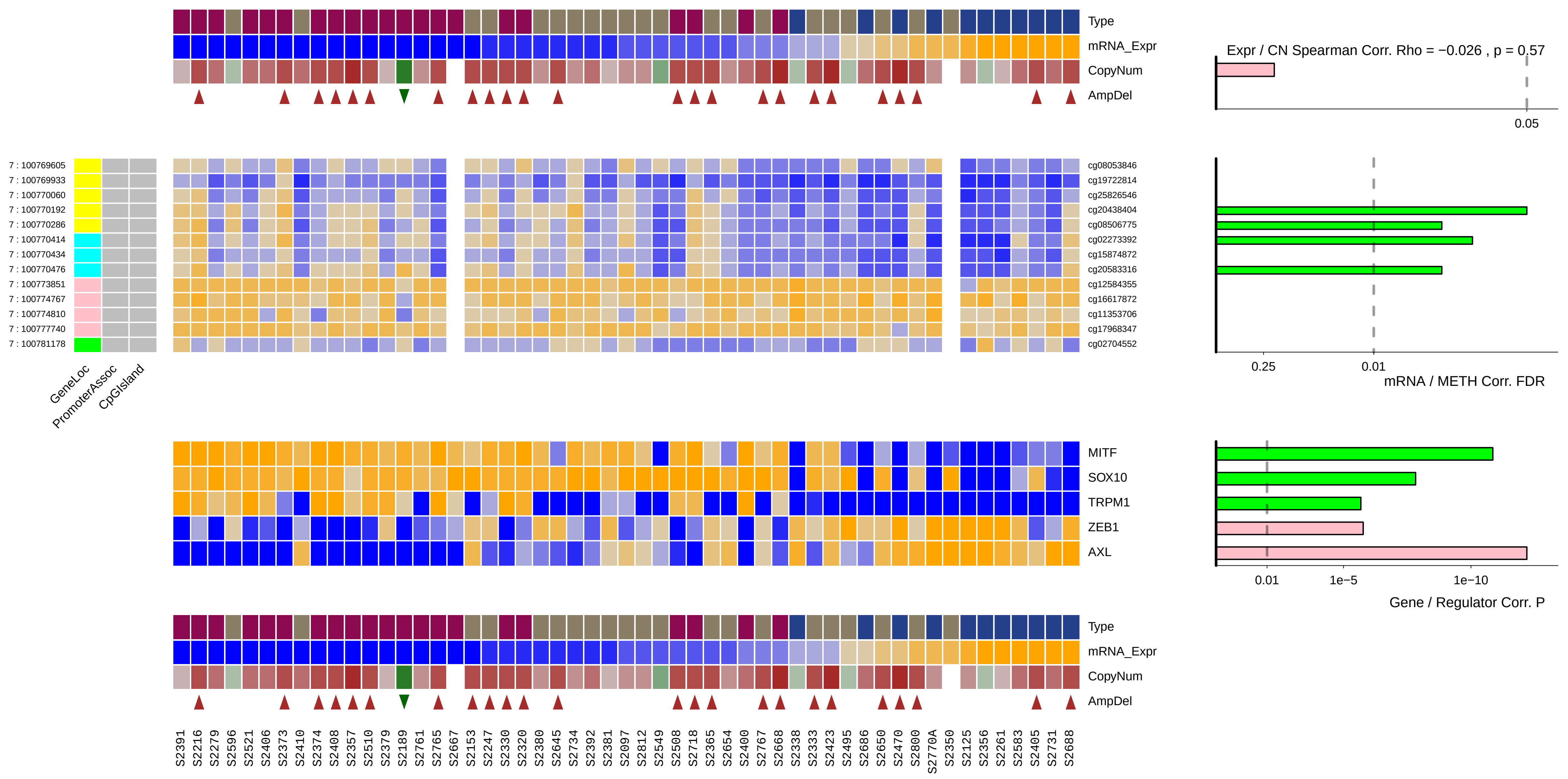
Type
mRNA_Expr
CopyNum
AmpDel
Expr / CN Spearman Corr. Rho = −0.026 , p = 0.57
0.05
GeneLoc
PromoterAssoc
CpGIsland
mRNA / METH Corr. FDR
0.25
0.01
MITF
SOX10
TRPM1
ZEB1
AXL
Gene / Regulator Corr. P
1e−5
1e−10

LTBP1

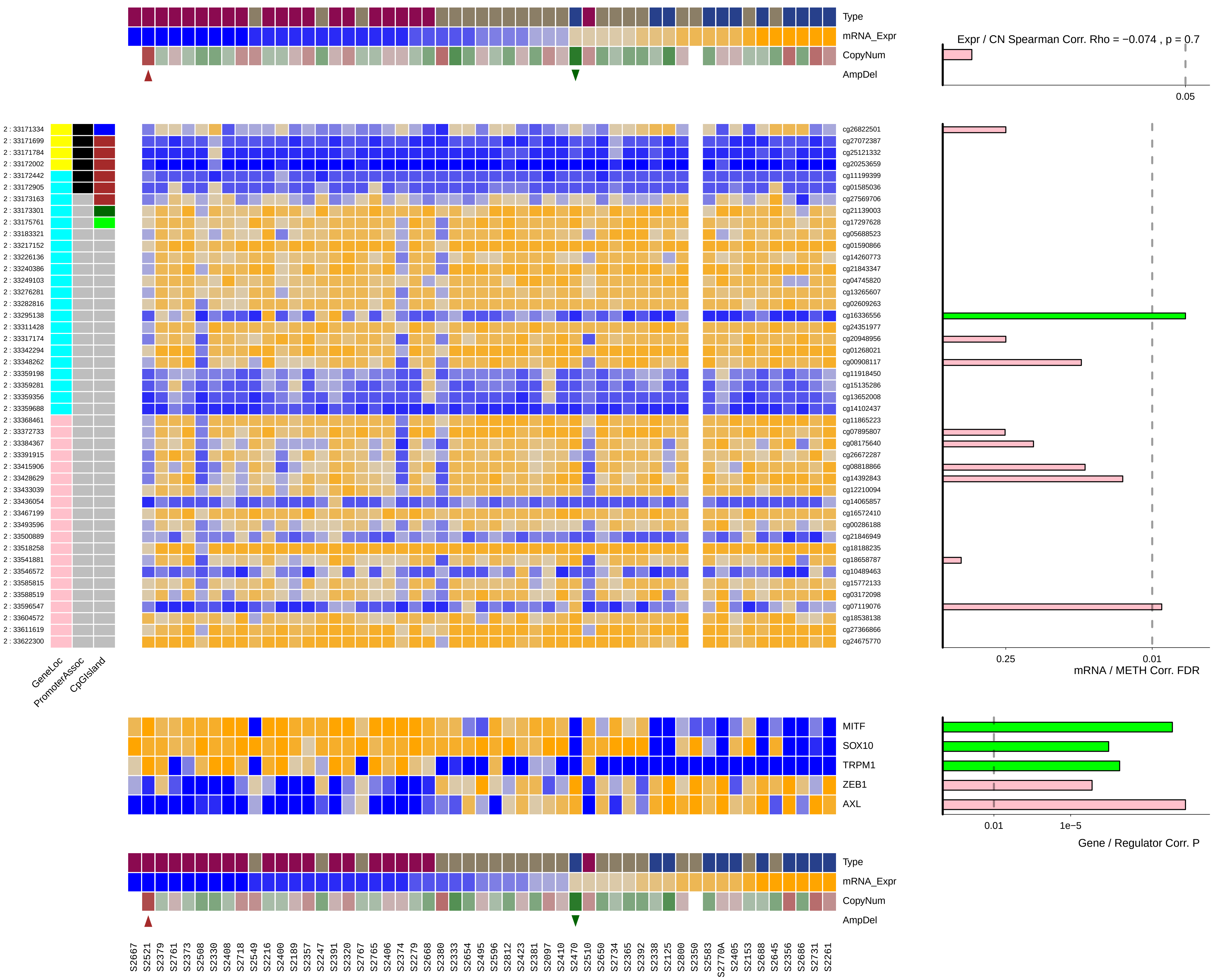
Type
mRNA_Expr
CopyNum
AmpDel
Expr / CN Spearman Corr. Rho = −0.074 , p = 0.7
0.05
GeneLoc
PromoterAssoc
CpGIsland
0.25
0.01
mRNA / METH Corr. FDR
MITF
SOX10
TRPM1
ZEB1
AXL
0.01
1e−5
Gene / Regulator Corr. P

TPBG

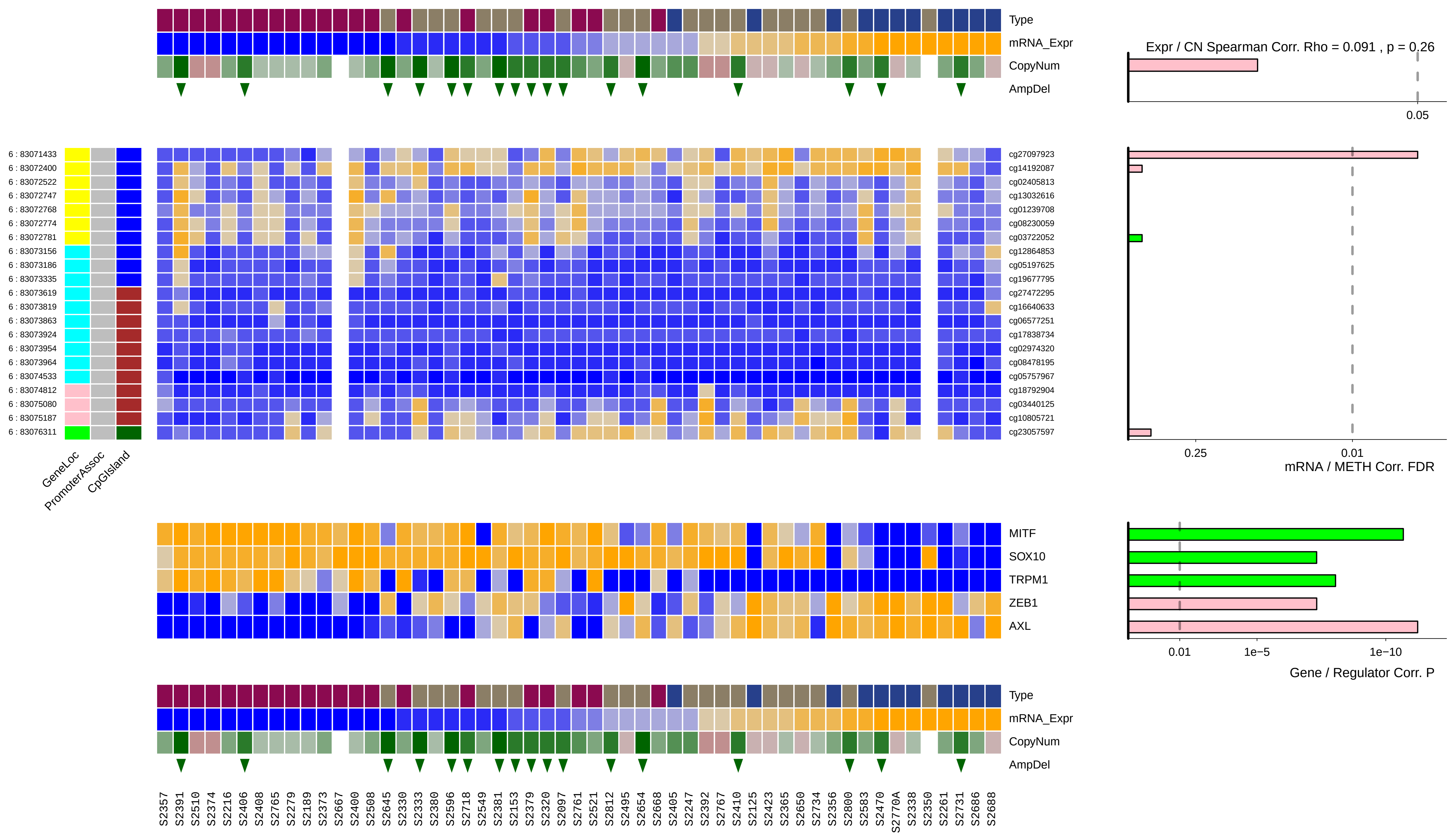
Type
mRNA_Expr
CopyNum
AmpDel
Expr / CN Spearman Corr. Rho = 0.091 , p = 0.26
0.05
6 : 83071433
6 : 83072400
6 : 83072522
6 : 83072747
6 : 83072768
6 : 83072774
6 : 83072781
6 : 83073156
6 : 83073186
6 : 83073335
6 : 83073619
6 : 83073819
6 : 83073863
6 : 83073924
6 : 83073954
6 : 83073964
6 : 83074533
6 : 83074812
6 : 83075080
6 : 83075187
6 : 83076311
cg27097923
cg14192087
cg02405813
cg13032616
cg01239708
cg08230059
cg03722052
cg12864853
cg05197625
cg19677795
cg27472295
cg16640633
cg06577251
cg17838734
cg02974320
cg08478195
cg05757967
cg18792904
cg03440125
cg10805721
cg23057597
0.25
0.01
mRNA / METH Corr. FDR
GeneLoc
PromoterAssoc
CpGIsland
MITF
SOX10
TRPM1
ZEB1
AXL
0.01
1e−5
1e−10
Gene / Regulator Corr. P
Type
mRNA_Expr
CopyNum
AmpDel
S2357
S2391
S2510
S2374
S2216
S2406
S2408
S2765
S2279
S2189
S2373
S2667
S2400
S2508
S2645
S2330
S2333
S2380
S2596
S2718
S2549
S2381
S2153
S2379
S2320
S2097
S2761
S2521
S2812
S2495
S2654
S2668
S2405
S2247
S2392
S2767
S2410
S2125
S2423
S2365
S2650
S2734
S2356
S2800
S2583
S2470
S2770A
S2338
S2350
S2261
S2731
S2686
S2688

CDH13

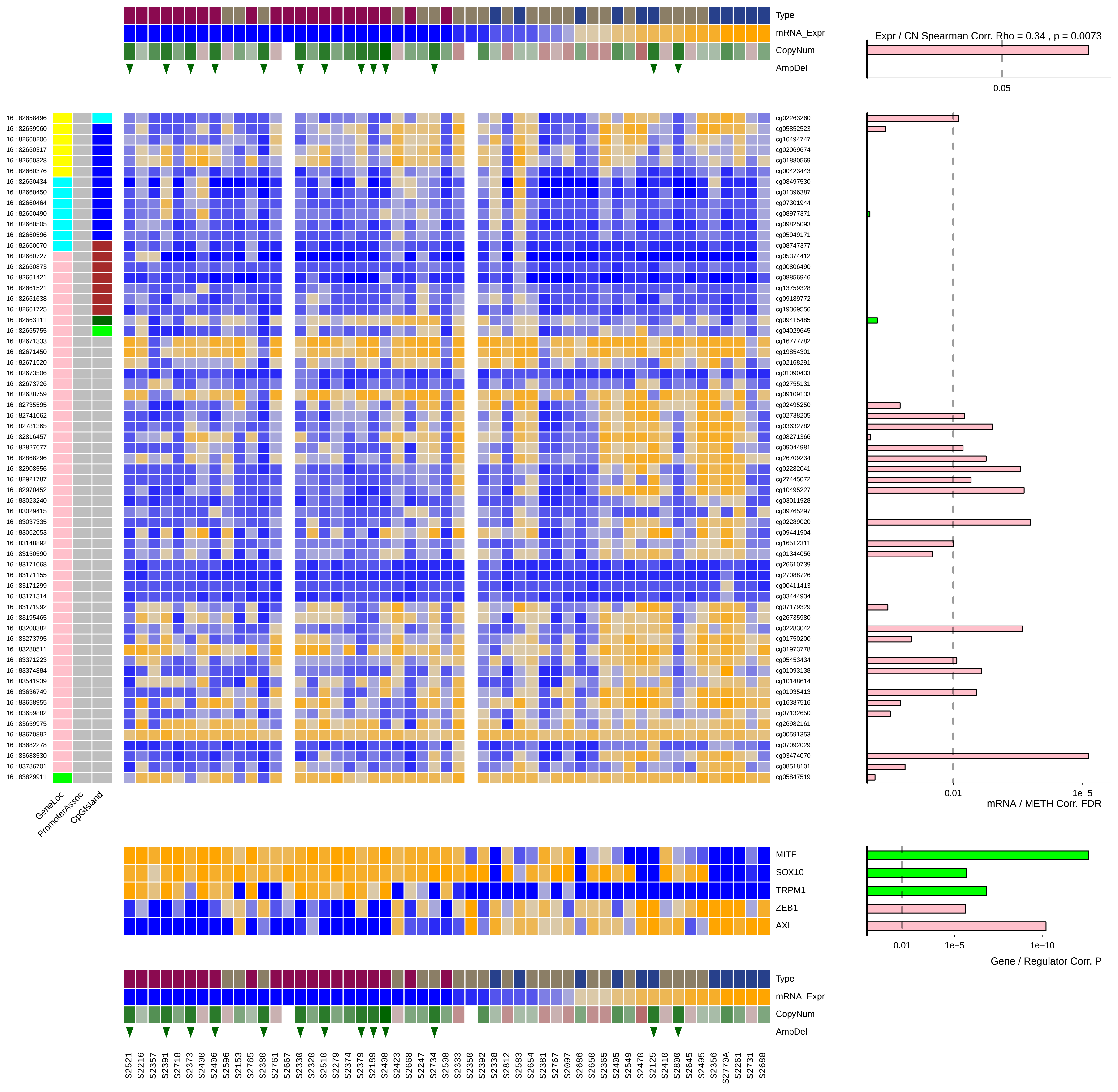
Type
mRNA_Expr
CopyNum
AmpDel
Expr / CN Spearman Corr. Rho = 0.34 , p = 0.0073
0.05
16 : 82658496
16 : 82659960
16 : 82660206
16 : 82660317
16 : 82660328
16 : 82660376
16 : 82660434
16 : 82660450
16 : 82660464
16 : 82660490
16 : 82660505
16 : 82660596
16 : 82660670
16 : 82660727
16 : 82660873
16 : 82661421
16 : 82661521
16 : 82661638
16 : 82661725
16 : 82663111
16 : 82665755
16 : 82671333
16 : 82671450
16 : 82671520
16 : 82673506
16 : 82673726
16 : 82688759
16 : 82735595
16 : 82741062
16 : 82781365
16 : 82816457
16 : 82827677
16 : 82868296
16 : 82908556
16 : 82921787
16 : 82970452
16 : 83023240
16 : 83029415
16 : 83037335
16 : 83062053
16 : 83148892
16 : 83150590
16 : 83171068
16 : 83171155
16 : 83171299
16 : 83171314
16 : 83171992
16 : 83195465
16 : 83200382
16 : 83273795
16 : 83280511
16 : 83371223
16 : 83374884
16 : 83541939
16 : 83636749
16 : 83658955
16 : 83659882
16 : 83659975
16 : 83670892
16 : 83682278
16 : 83688530
16 : 83786701
16 : 83829911
cg02263260
cg05852523
cg16494747
cg02069674
cg01880569
cg00423443
cg08497530
cg01396387
cg07301944
cg08977371
cg09825093
cg05949171
cg08747377
cg05374412
cg00806490
cg08856946
cg13759328
cg09189772
cg19369556
cg09415485
cg04029645
cg16777782
cg19854301
cg02168291
cg01090433
cg02755131
cg09109133
cg02495250
cg02738205
cg03632782
cg08271366
cg09044981
cg26709234
cg02282041
cg27445072
cg10495227
cg03011928
cg09765297
cg02289020
cg09441904
cg16512311
cg01344056
cg26610739
cg27088726
cg00411413
cg03444934
cg07179329
cg26735980
cg02283042
cg01750200
cg01973778
cg05453434
cg01093138
cg10148614
cg01935413
cg16387516
cg07132650
cg26982161
cg00591353
cg07092029
cg03474070
cg08518101
cg05847519
0.01
1e−5
mRNA / METH Corr. FDR
GeneLoc
PromoterAssoc
CpGIsland
MITF
SOX10
TRPM1
ZEB1
AXL
0.01
1e−5
1e−10
Gene / Regulator Corr. P
Type
mRNA_Expr
CopyNum
AmpDel
S2521 S2216 S2357 S2391 S2718 S2373 S2400 S2406 S2596 S2153 S2765 S2380 S2761 S2667 S2330 S2320 S2510 S2279 S2374 S2379 S2189 S2408 S2423 S2668 S2247 S2734 S2508 S2333 S2350 S2392 S2338 S2812 S2583 S2654 S2381 S2767 S2097 S2686 S2650 S2365 S2405 S2549 S2470 S2125 S2410 S2800 S2645 S2495 S2356 S2770A S2261 S2731 S2688

RAB27B

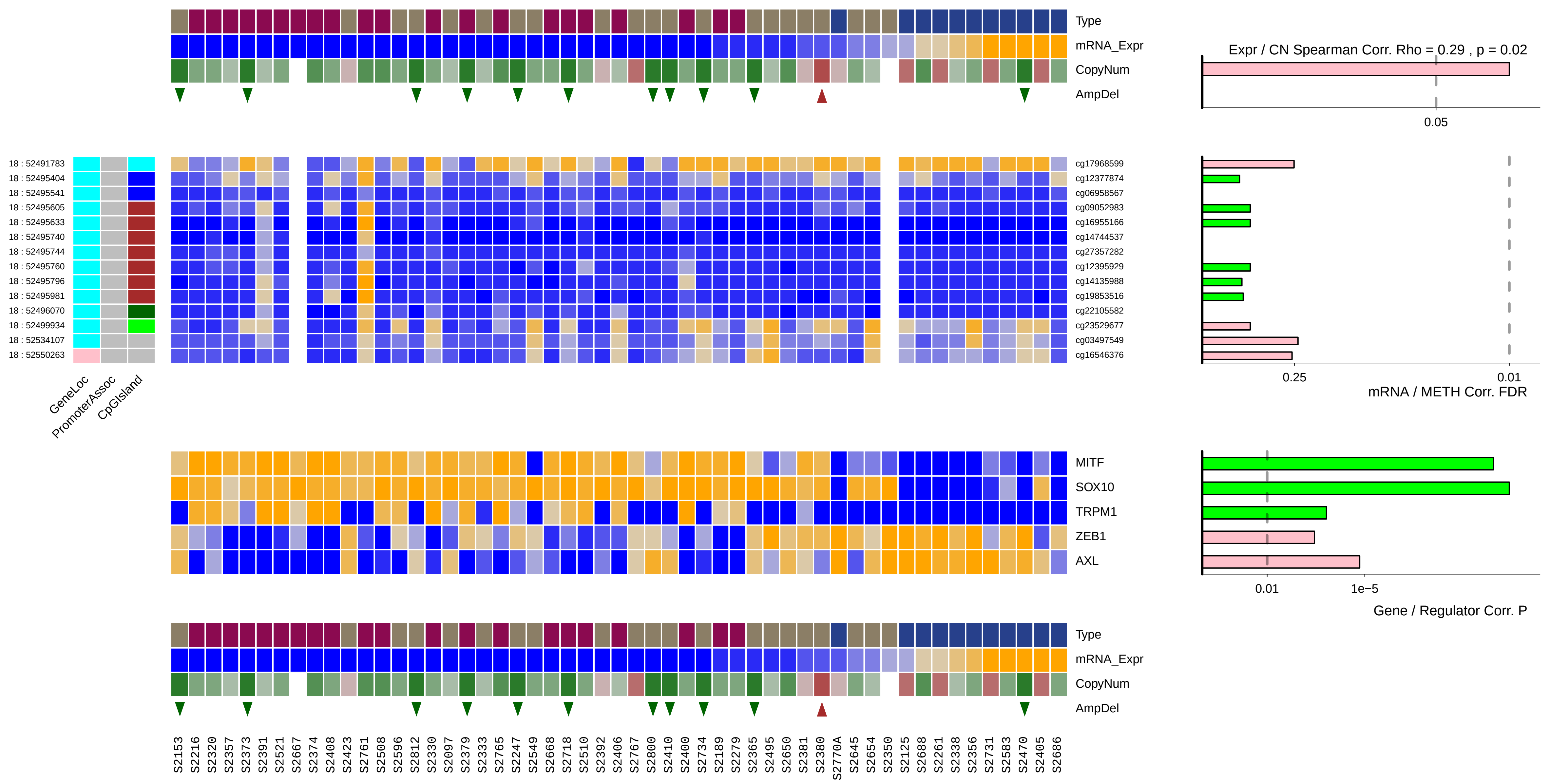
Type
mRNA_Expr
CopyNum
AmpDel
Expr / CN Spearman Corr. Rho = 0.29 , p = 0.02
0.05
18 : 52491783
18 : 52495404
18 : 52495541
18 : 52495605
18 : 52495633
18 : 52495740
18 : 52495744
18 : 52495760
18 : 52495796
18 : 52495981
18 : 52496070
18 : 52499934
18 : 52534107
18 : 52550263
cg17968599
cg12377874
cg06958567
cg09052983
cg16955166
cg14744537
cg27357282
cg12395929
cg14135988
cg19853516
cg22105582
cg23529677
cg03497549
cg16546376
GeneLoc
PromoterAssoc
CpGIsland
0.25
0.01
mRNA / METH Corr. FDR
MITF
SOX10
TRPM1
ZEB1
AXL
0.01
1e−5
Gene / Regulator Corr. P
Type
mRNA_Expr
CopyNum
AmpDel
S2153 S2216 S2320 S2357 S2373 S2391 S2521 S2667 S2374 S2408 S2423 S2761 S2508 S2596 S2812 S2330 S2097 S2379 S2333 S2765 S2247 S2549 S2668 S2718 S2510 S2392 S2406 S2767 S2800 S2410 S2400 S2734 S2189 S2279 S2365 S2495 S2650 S2381 S2380 S2770A S2645 S2654 S2350 S2125 S2688 S2261 S2338 S2356 S2731 S2583 S2470 S2405 S2686

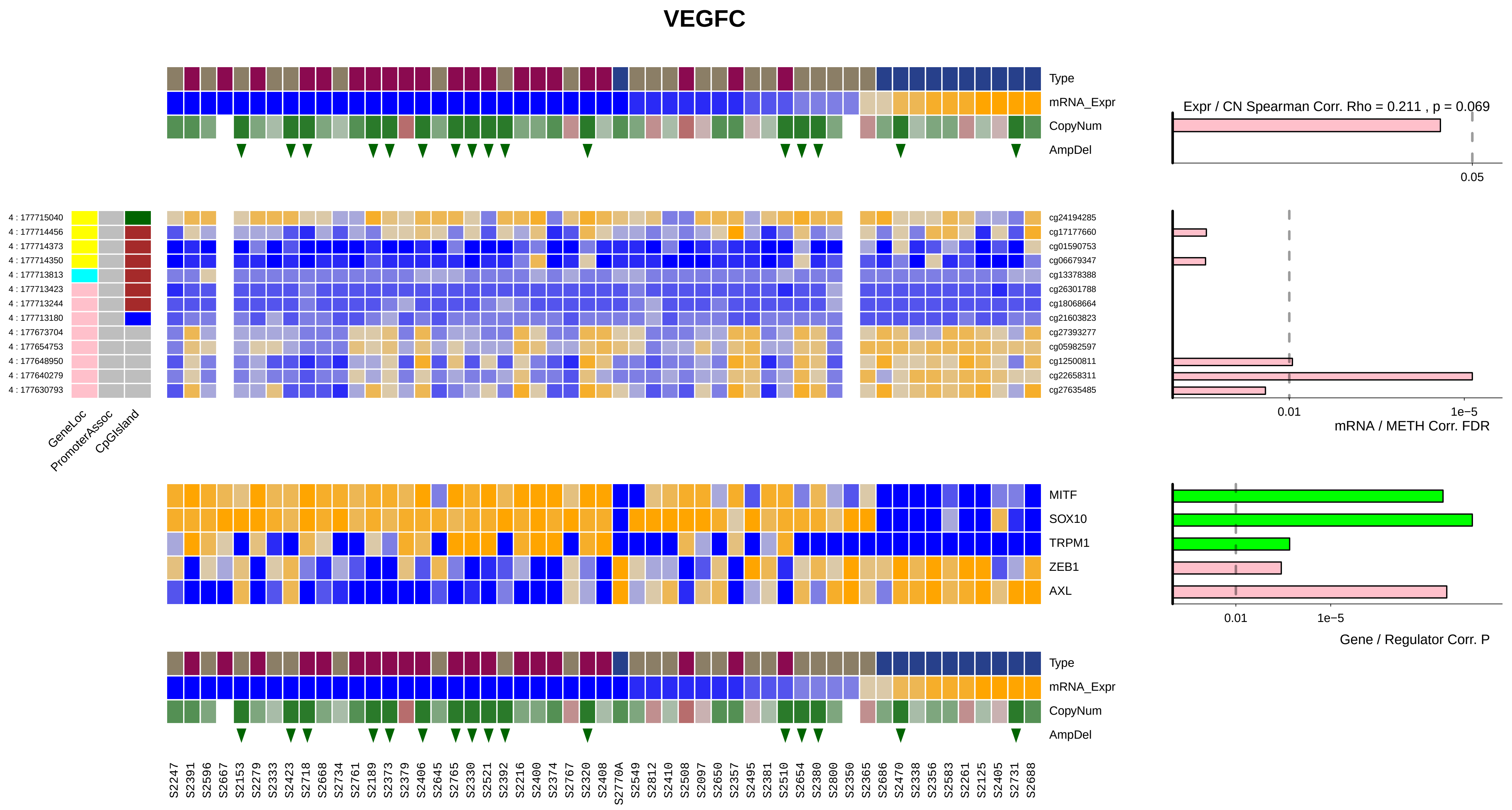
VEGFC
Type
mRNA_Expr
CopyNum
AmpDel
Expr / CN Spearman Corr. Rho = 0.211 , p = 0.069
0.05
4 : 177715040
4 : 177714456
4 : 177714373
4 : 177714350
4 : 177713813
4 : 177713423
4 : 177713244
4 : 177713180
4 : 177673704
4 : 177654753
4 : 177648950
4 : 177640279
4 : 177630793
GeneLoc
PromoterAssoc
CpGIsland
cg24194285
cg17177660
cg01590753
cg06679347
cg13378388
cg26301788
cg18068664
cg21603823
cg27393277
cg05982597
cg12500811
cg22658311
cg27635485
0.01
1e−5
mRNA / METH Corr. FDR
MITF
SOX10
TRPM1
ZEB1
AXL
0.01
1e−5
Gene / Regulator Corr. P
Type
mRNA_Expr
CopyNum
AmpDel
S2247 S2391 S2596 S2667 S2153 S2279 S2333 S2423 S2718 S2668 S2734 S2761 S2189 S2373 S2379 S2406 S2645 S2765 S2330 S2521 S2392 S2216 S2400 S2374 S2767 S2320 S2408 S2770A S2549 S2812 S2410 S2508 S2097 S2650 S2357 S2495 S2381 S2510 S2654 S2380 S2800 S2350 S2365 S2686 S2470 S2338 S2356 S2583 S2261 S2125 S2405 S2731 S2688

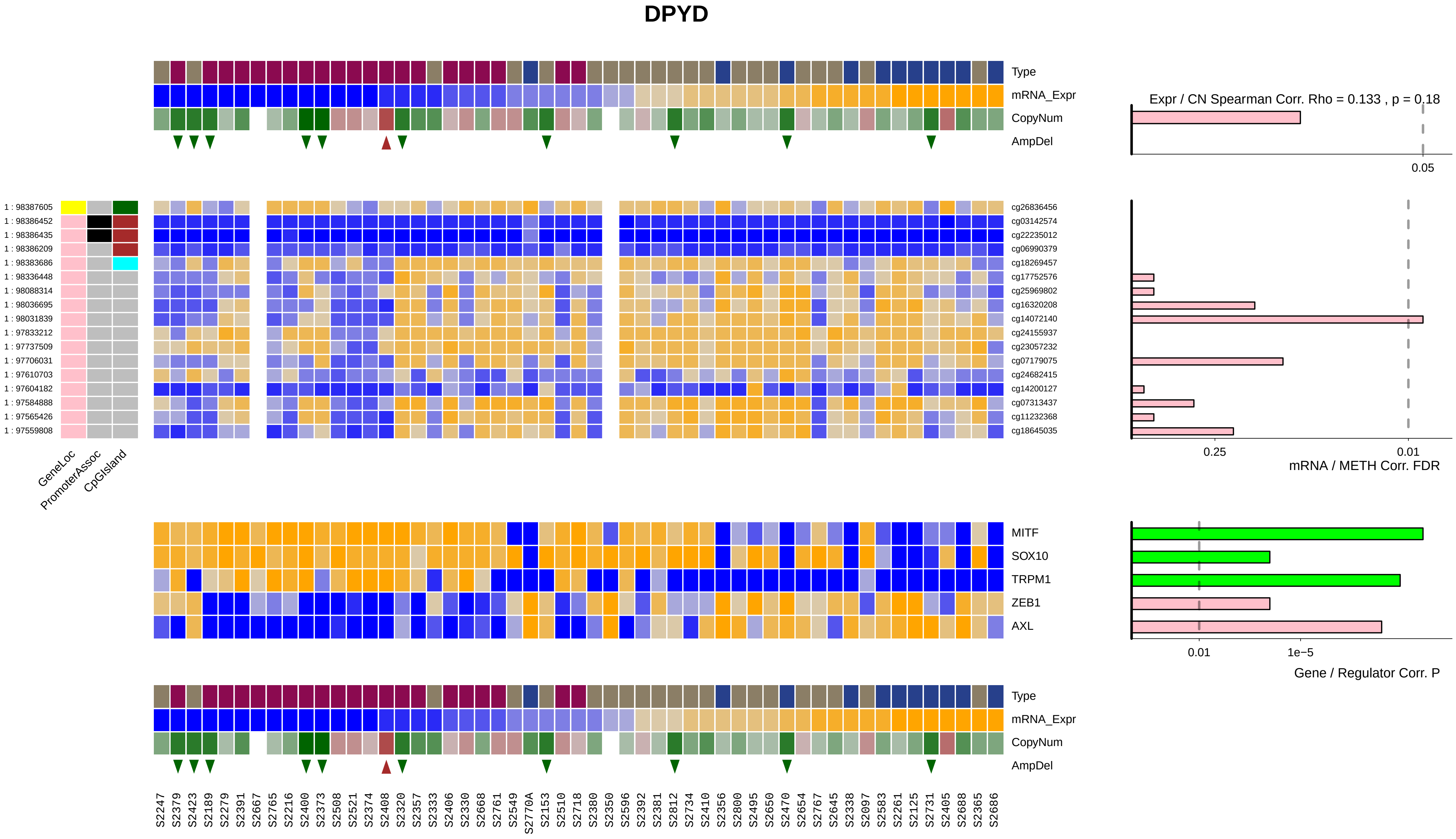
DPYD
Type
mRNA_Expr
CopyNum
AmpDel
Expr / CN Spearman Corr. Rho = 0.133 , p = 0.18
0.05
1 : 98387605
1 : 98386452
1 : 98386435
1 : 98386209
1 : 98383686
1 : 98336448
1 : 98088314
1 : 98036695
1 : 98031839
1 : 97833212
1 : 97737509
1 : 97706031
1 : 97610703
1 : 97604182
1 : 97584888
1 : 97565426
1 : 97559808
GeneLoc
PromoterAssoc
CpGIsland
cg26836456
cg03142574
cg22235012
cg06990379
cg18269457
cg17752576
cg25969802
cg16320208
cg14072140
cg24155937
cg23057232
cg07179075
cg24682415
cg14200127
cg07313437
cg11232368
cg18645035
0.25
0.01
mRNA / METH Corr. FDR
MITF
SOX10
TRPM1
ZEB1
AXL
0.01
1e−5
Gene / Regulator Corr. P
Type
mRNA_Expr
CopyNum
AmpDel
S2247 S2379 S2423 S2189 S2279 S2391 S2667 S2765 S2216 S2400 S2373 S2508 S2521 S2374 S2408 S2320 S2357 S2333 S2406 S2330 S2668 S2761 S2549 S2770A S2153 S2510 S2718 S2380 S2350 S2596 S2392 S2381 S2812 S2734 S2410 S2356 S2800 S2495 S2650 S2470 S2654 S2767 S2645 S2338 S2097 S2583 S2261 S2125 S2731 S2405 S2688 S2365 S2686

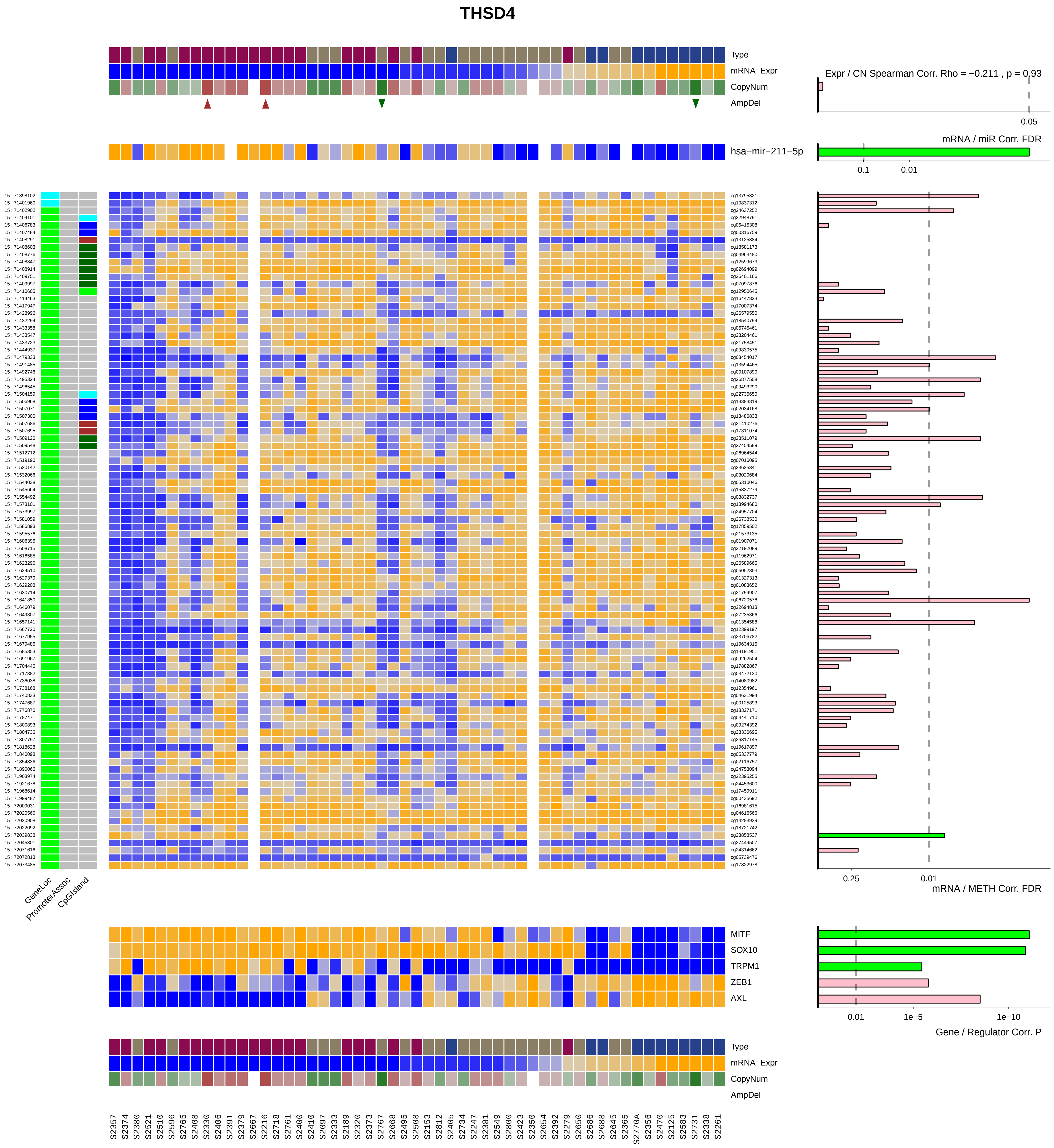
THSD4
Type
mRNA_Expr
CopyNum
AmpDel
Expr / CN Spearman Corr. Rho = −0.211 , p = 0.93
0.05
mRNA / miR Corr. FDR
hsa−mir−211−5p
0.1
0.01
GeneLoc
PromoterAssoc
CpGIsland
0.25
0.01
mRNA / METH Corr. FDR
MITF
SOX10
TRPM1
ZEB1
AXL
0.01
1e−5
1e−10
Gene / Regulator Corr. P
S2357 S2374 S2380 S2521 S2510 S2596 S2765 S2408 S2330 S2406 S2391 S2379 S2667 S2216 S2718 S2761 S2400 S2410 S2097 S2333 S2189 S2320 S2373 S2767 S2668 S2495 S2508 S2153 S2812 S2405 S2734 S2247 S2381 S2549 S2800 S2423 S2350 S2654 S2392 S2279 S2650 S2686 S2688 S2645 S2365 S2770A S2356 S2470 S2125 S2583 S2731 S2338 S2261

COL6A2

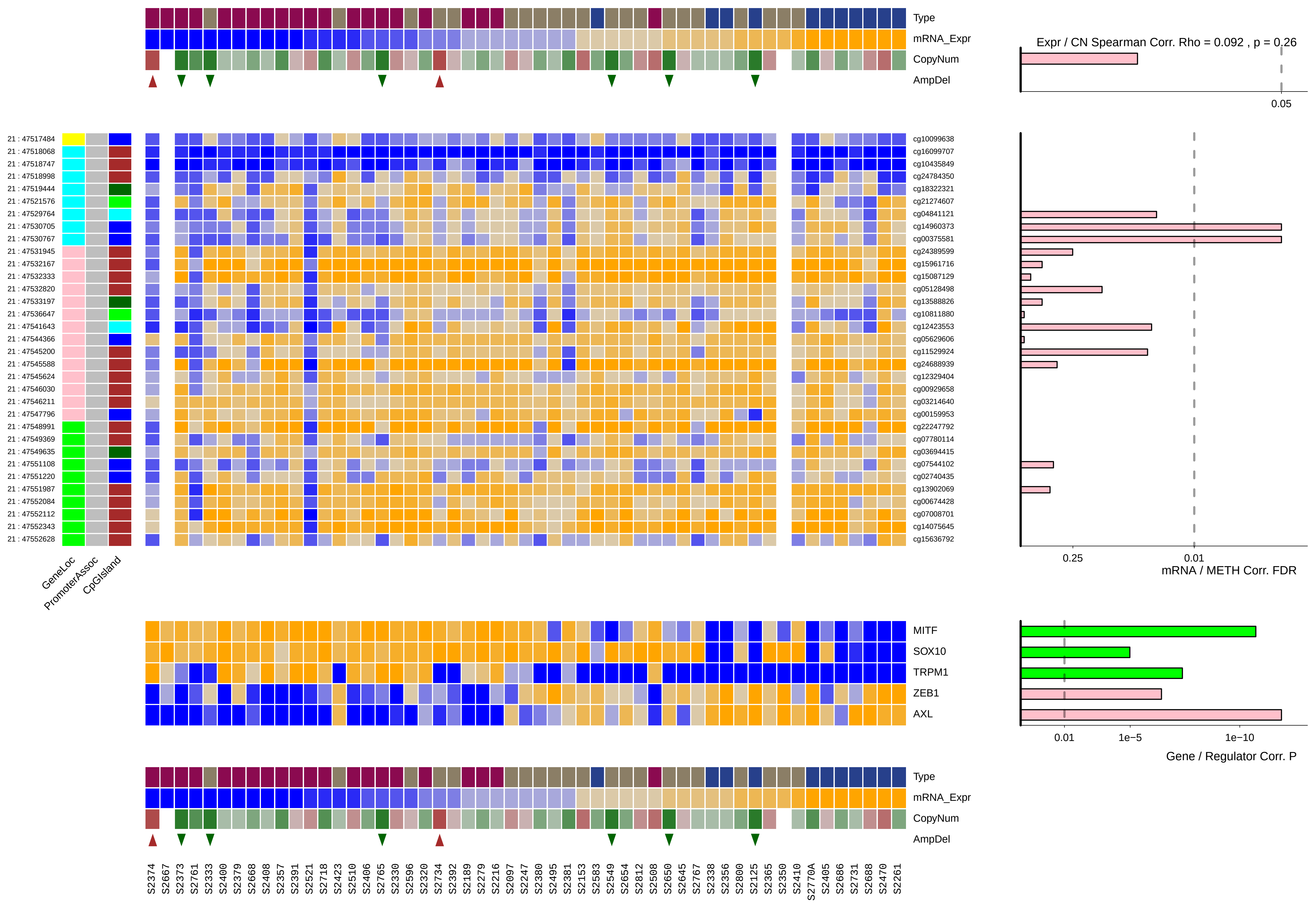

Type
mRNA_Expr
CopyNum
AmpDel
Expr / CN Spearman Corr. Rho = 0.092 , p = 0.26
0.05
GeneLoc
PromoterAssoc
CpGIsland
mRNA / METH Corr. FDR
0.25
0.01
MITF
SOX10
TRPM1
ZEB1
AXL
Gene / Regulator Corr. P
1e−5
1e−10

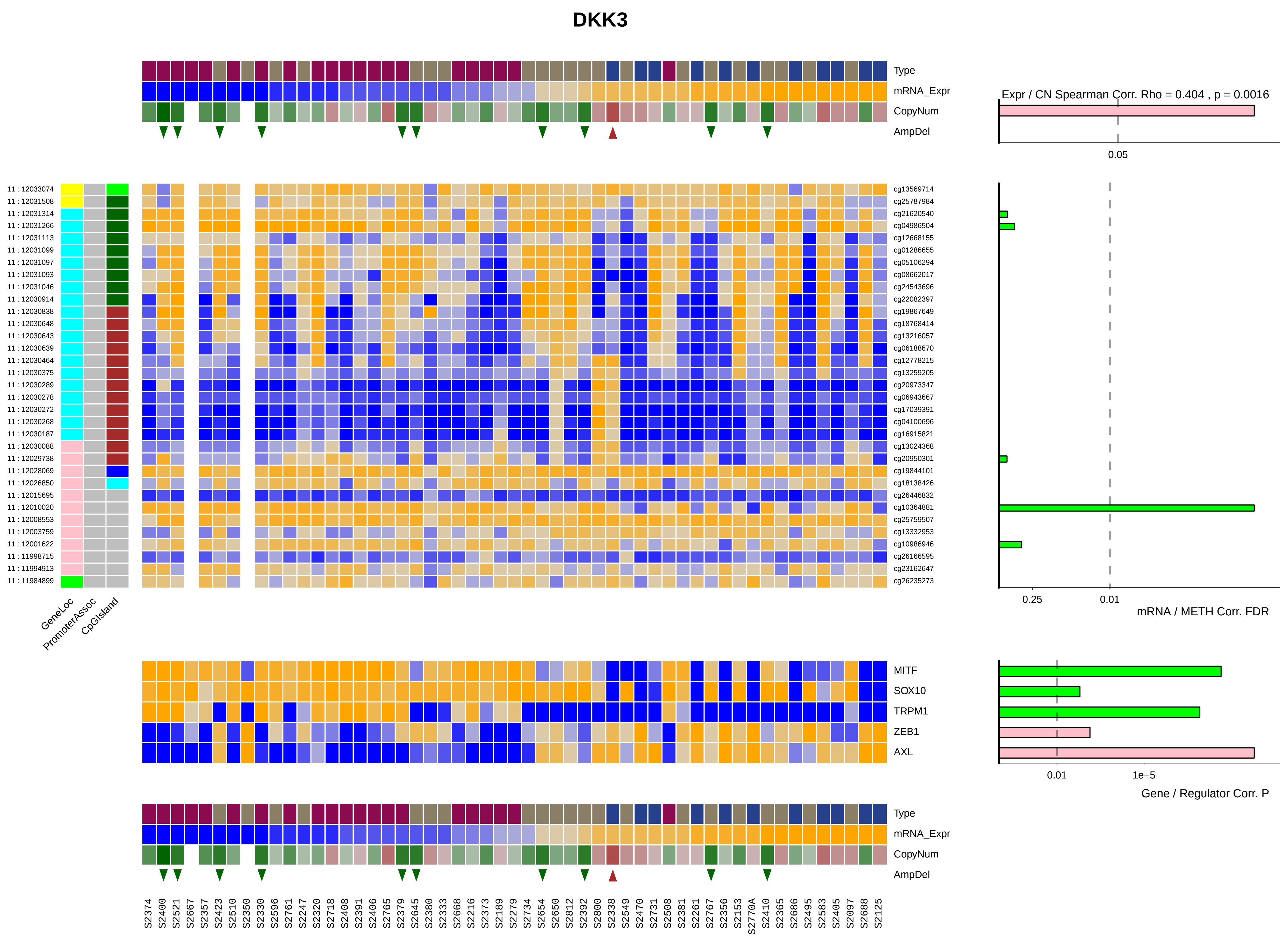
DKK3
Type
mRNA_Expr
CopyNum
AmpDel
Expr / CN Spearman Corr. Rho = 0.404 , p = 0.0016
0.05
11 : 12033074
11 : 12031508
11 : 12031314
11 : 12031266
11 : 12031113
11 : 12031099
11 : 12031097
11 : 12031093
11 : 12031046
11 : 12030914
11 : 12030838
11 : 12030648
11 : 12030643
11 : 12030639
11 : 12030464
11 : 12030375
11 : 12030289
11 : 12030278
11 : 12030272
11 : 12030268
11 : 12030187
11 : 12030088
11 : 12029738
11 : 12028069
11 : 12026850
11 : 12015695
11 : 12010020
11 : 12008553
11 : 12003759
11 : 12001622
11 : 11998715
11 : 11994913
11 : 11984899
cg13569714
cg25787984
cg21620540
cg04986504
cg12668155
cg01286655
cg05106294
cg08662017
cg24543696
cg22082397
cg19867649
cg18768414
cg13216057
cg06188670
cg12778215
cg13259205
cg20973347
cg06943667
cg17039391
cg04100696
cg16915821
cg13024368
cg20950301
cg19844101
cg18138426
cg26446832
cg10364881
cg25759507
cg13332953
cg10986946
cg26166595
cg23162647
cg26235273
0.25
0.01
mRNA / METH Corr. FDR
GeneLoc
PromoterAssoc
CpGIsland
MITF
SOX10
TRPM1
ZEB1
AXL
0.01
1e−5
Gene / Regulator Corr. P
Type
mRNA_Expr
CopyNum
AmpDel
S2374
S2400
S2521
S2667
S2357
S2423
S2510
S2350
S2330
S2596
S2761
S2247
S2320
S2718
S2408
S2391
S2406
S2765
S2379
S2645
S2380
S2333
S2668
S2216
S2373
S2189
S2279
S2734
S2654
S2650
S2812
S2392
S2800
S2338
S2549
S2470
S2731
S2508
S2381
S2261
S2767
S2356
S2153
S2770A
S2410
S2365
S2686
S2495
S2583
S2405
S2097
S2688
S2125

SOX9

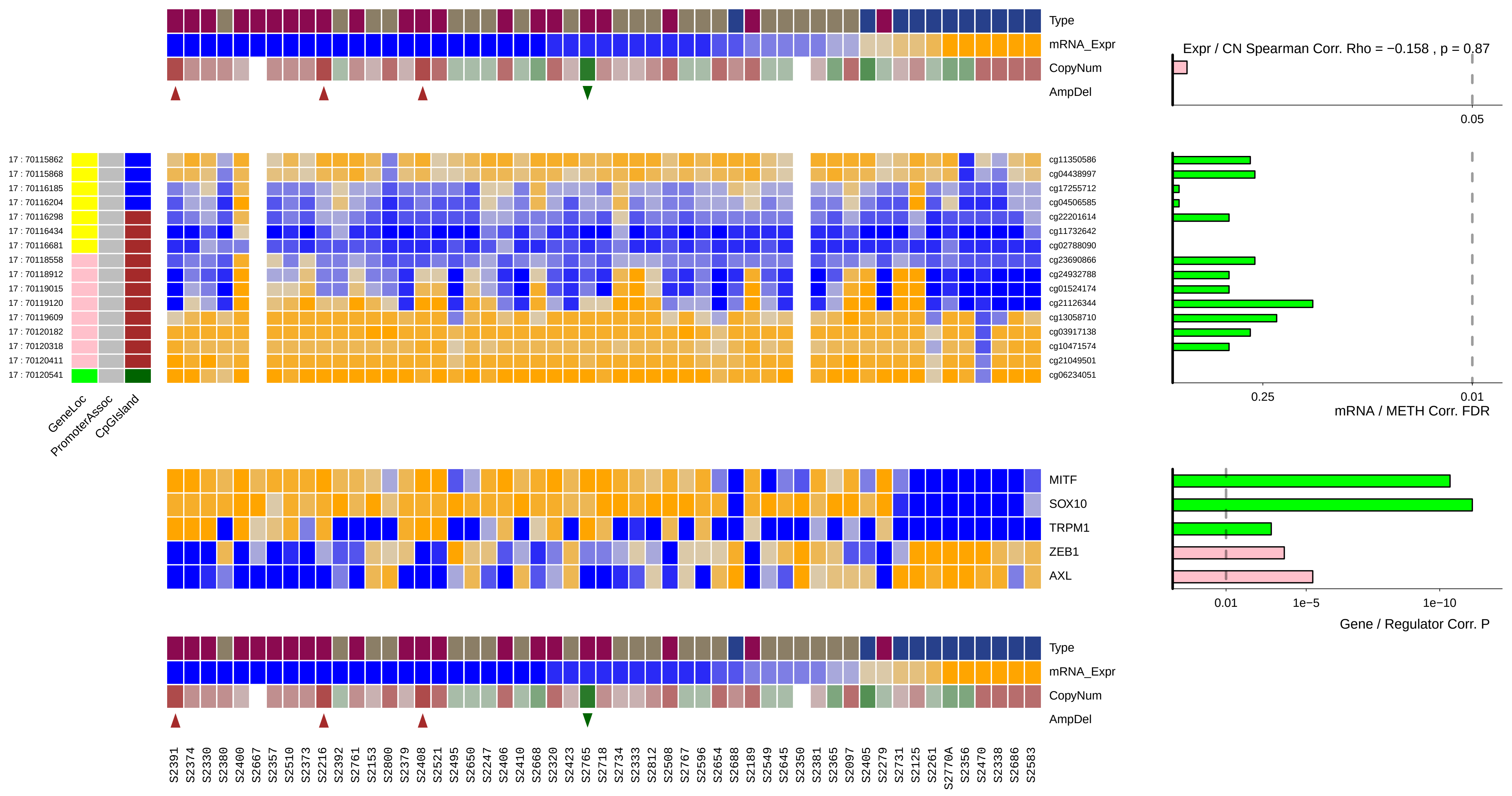
Type
mRNA_Expr
CopyNum
AmpDel
Expr / CN Spearman Corr. Rho = −0.158 , p = 0.87
0.05
17 : 70115862
17 : 70115868
17 : 70116185
17 : 70116204
17 : 70116298
17 : 70116434
17 : 70116681
17 : 70118558
17 : 70118912
17 : 70119015
17 : 70119120
17 : 70119609
17 : 70120182
17 : 70120318
17 : 70120411
17 : 70120541
cg11350586
cg04438997
cg17255712
cg04506585
cg22201614
cg11732642
cg02788090
cg23690866
cg24932788
cg01524174
cg21126344
cg13058710
cg03917138
cg10471574
cg21049501
cg06234051
GeneLoc
PromoterAssoc
CpGIsland
0.25
0.01
mRNA / METH Corr. FDR
MITF
SOX10
TRPM1
ZEB1
AXL
0.01
1e−5
1e−10
Gene / Regulator Corr. P
S2391 S2374 S2330 S2380 S2400 S2667 S2357 S2510 S2373 S2216 S2392 S2761 S2153 S2800 S2379 S2408 S2521 S2495 S2650 S2247 S2406 S2410 S2668 S2320 S2423 S2765 S2718 S2734 S2333 S2812 S2508 S2767 S2596 S2654 S2688 S2189 S2549 S2645 S2350 S2381 S2365 S2097 S2405 S2279 S2731 S2125 S2261 S2770A S2356 S2470 S2338 S2686 S2583

PDGFC

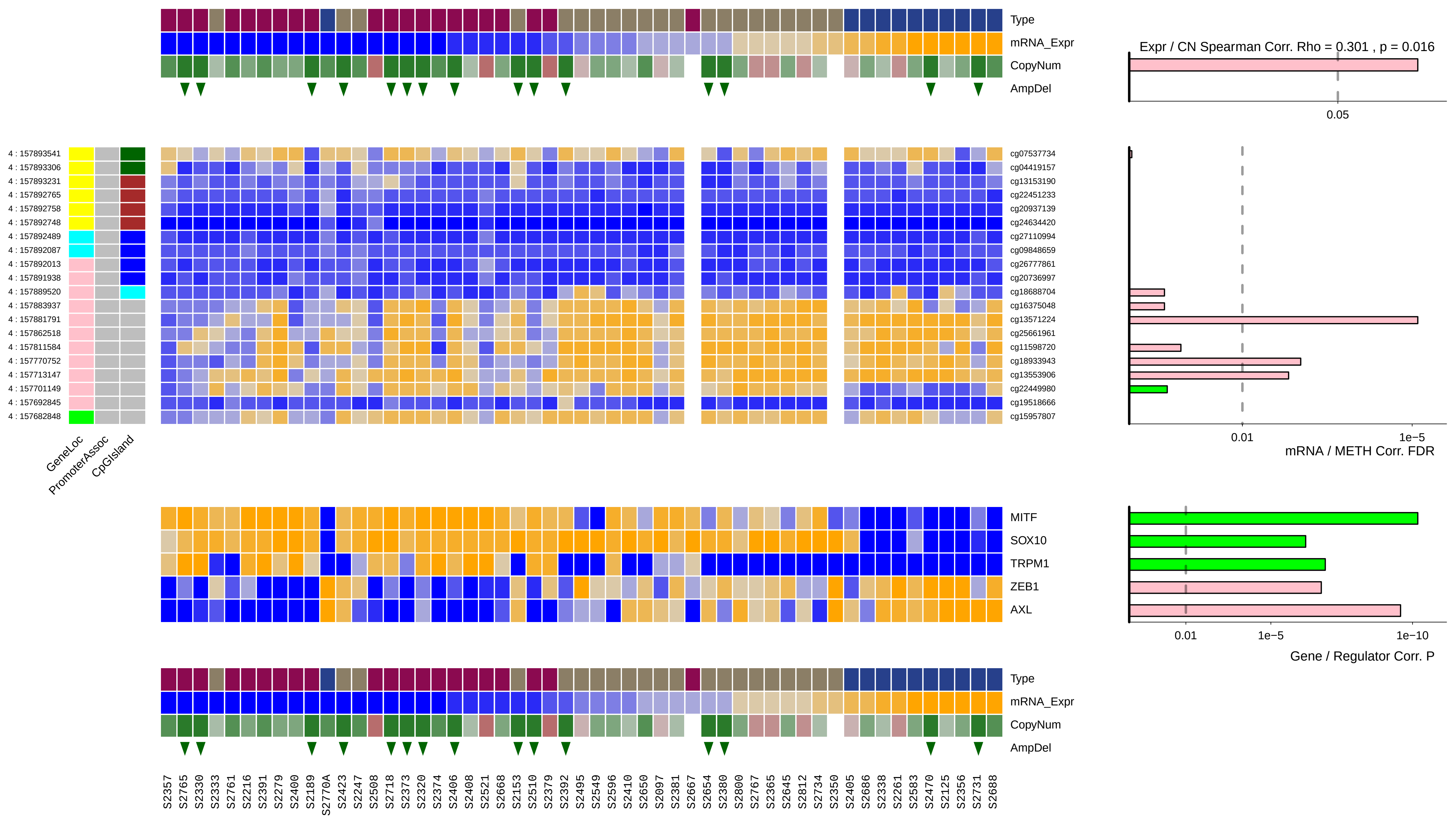
Type
mRNA_Expr
CopyNum
AmpDel
Expr / CN Spearman Corr. Rho = 0.301 , p = 0.016
0.05
mRNA / METH Corr. FDR
0.01
1e−5
GeneLoc
PromoterAssoc
CpGIsland
MITF
SOX10
TRPM1
ZEB1
AXL
Gene / Regulator Corr. P
1e−10

HECW2

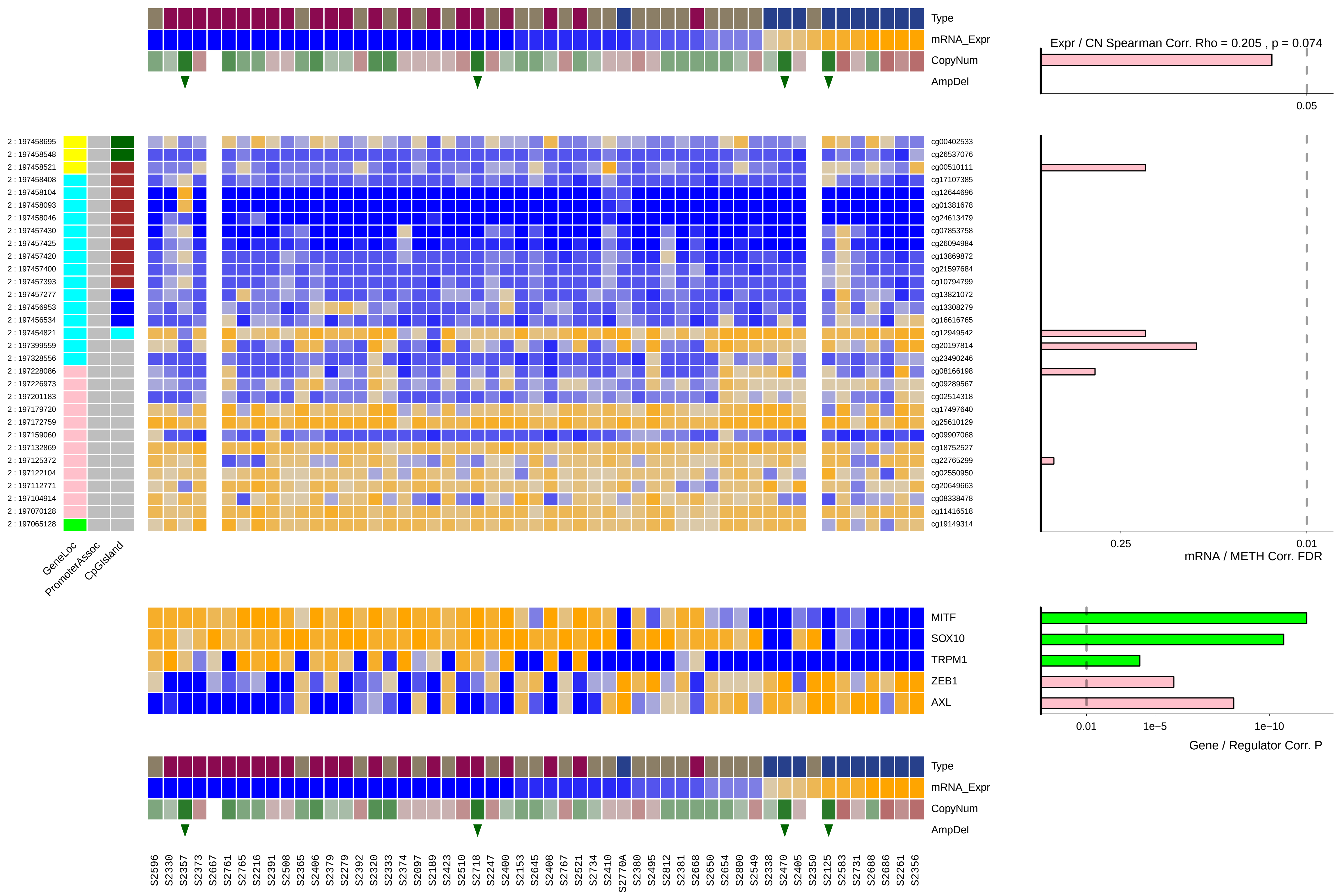

Type
mRNA_Expr
CopyNum
AmpDel
Expr / CN Spearman Corr. Rho = 0.205 , p = 0.074
0.05
2 : 197458695
2 : 197458548
2 : 197458521
2 : 197458408
2 : 197458104
2 : 197458093
2 : 197458046
2 : 197457430
2 : 197457425
2 : 197457420
2 : 197457400
2 : 197457393
2 : 197457277
2 : 197456953
2 : 197456534
2 : 197454821
2 : 197399559
2 : 197328556
2 : 197228086
2 : 197226973
2 : 197201183
2 : 197179720
2 : 197172759
2 : 197159060
2 : 197132869
2 : 197125372
2 : 197122104
2 : 197112771
2 : 197104914
2 : 197070128
2 : 197065128
cg00402533
cg26537076
cg00510111
cg17107385
cg12644696
cg01381678
cg24613479
cg07853758
cg26094984
cg13869872
cg21597684
cg10794799
cg13821072
cg13308279
cg16616765
cg12949542
cg20197814
cg23490246
cg08166198
cg09289567
cg02514318
cg17497640
cg25610129
cg09907068
cg18752527
cg22765299
cg02550950
cg20649663
cg08338478
cg11416518
cg19149314
0.25
0.01
mRNA / METH Corr. FDR
GeneLoc
PromoterAssoc
CpGIsland
MITF
SOX10
TRPM1
ZEB1
AXL
0.01
1e−5
1e−10
Gene / Regulator Corr. P
Type
mRNA_Expr
CopyNum
AmpDel
S2596
S2330
S2357
S2373
S2667
S2761
S2765
S2216
S2391
S2508
S2365
S2406
S2379
S2279
S2392
S2320
S2333
S2374
S2097
S2189
S2423
S2510
S2718
S2247
S2400
S2153
S2645
S2408
S2767
S2521
S2734
S2410
S2770A
S2380
S2495
S2812
S2381
S2668
S2650
S2654
S2800
S2549
S2338
S2470
S2405
S2350
S2125
S2583
S2731
S2688
S2686
S2261
S2356

FZD2

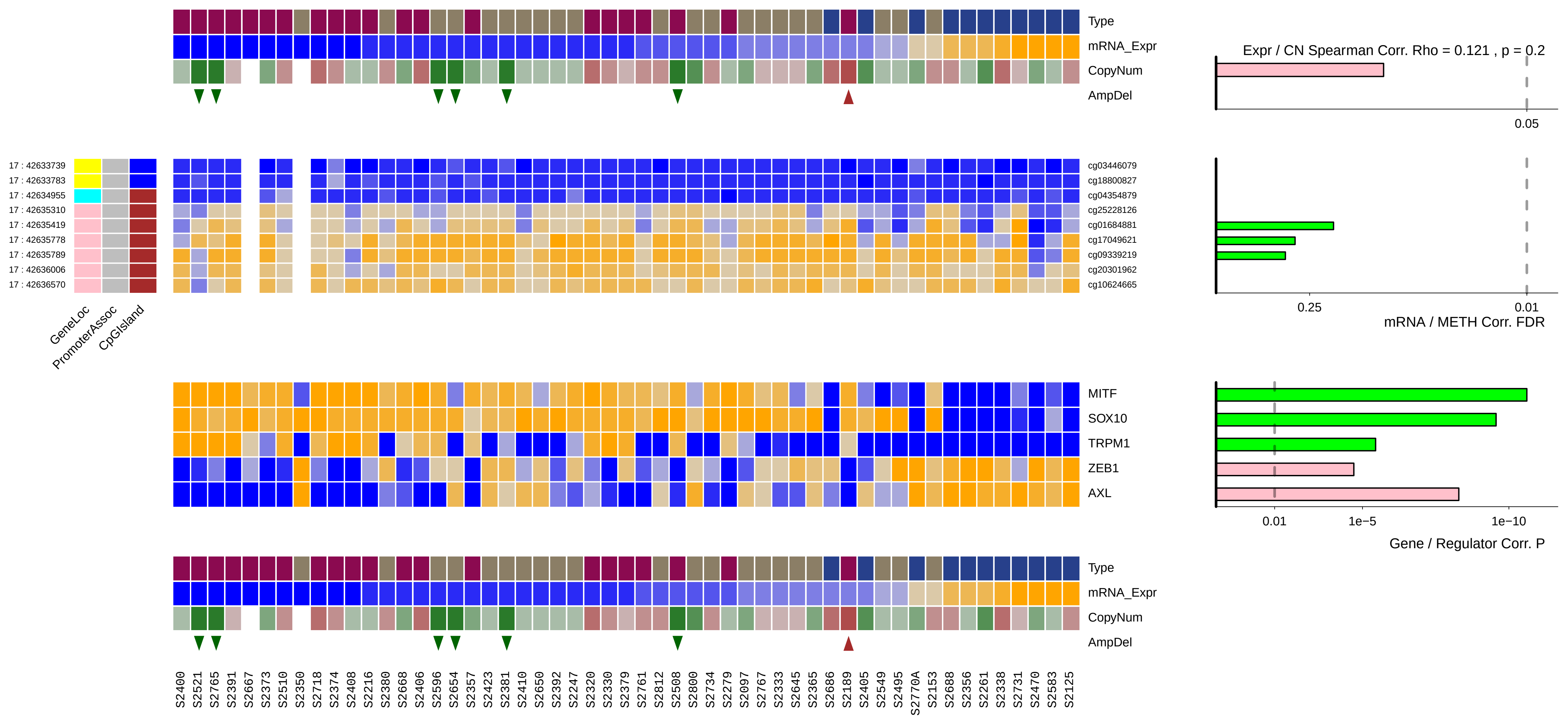
Type
mRNA_Expr
CopyNum
AmpDel
Expr / CN Spearman Corr. Rho = 0.121 , p = 0.2
0.05
17 : 42633739
17 : 42633783
17 : 42634955
17 : 42635310
17 : 42635419
17 : 42635778
17 : 42635789
17 : 42636006
17 : 42636570
GeneLoc
PromoterAssoc
CpGIsland
cg03446079
cg18800827
cg04354879
cg25228126
cg01684881
cg17049621
cg09339219
cg20301962
cg10624665
0.25
0.01
mRNA / METH Corr. FDR
MITF
SOX10
TRPM1
ZEB1
AXL
0.01
1e−5
1e−10
Gene / Regulator Corr. P
S2400 S2521 S2765 S2391 S2667 S2373 S2510 S2350 S2718 S2374 S2408 S2216 S2380 S2668 S2406 S2596 S2654 S2357 S2423 S2381 S2410 S2650 S2392 S2247 S2320 S2330 S2379 S2761 S2812 S2508 S2800 S2734 S2279 S2097 S2767 S2333 S2645 S2365 S2686 S2189 S2405 S2549 S2495 S2770A S2153 S2688 S2356 S2261 S2338 S2731 S2470 S2583 S2125

PAPPA

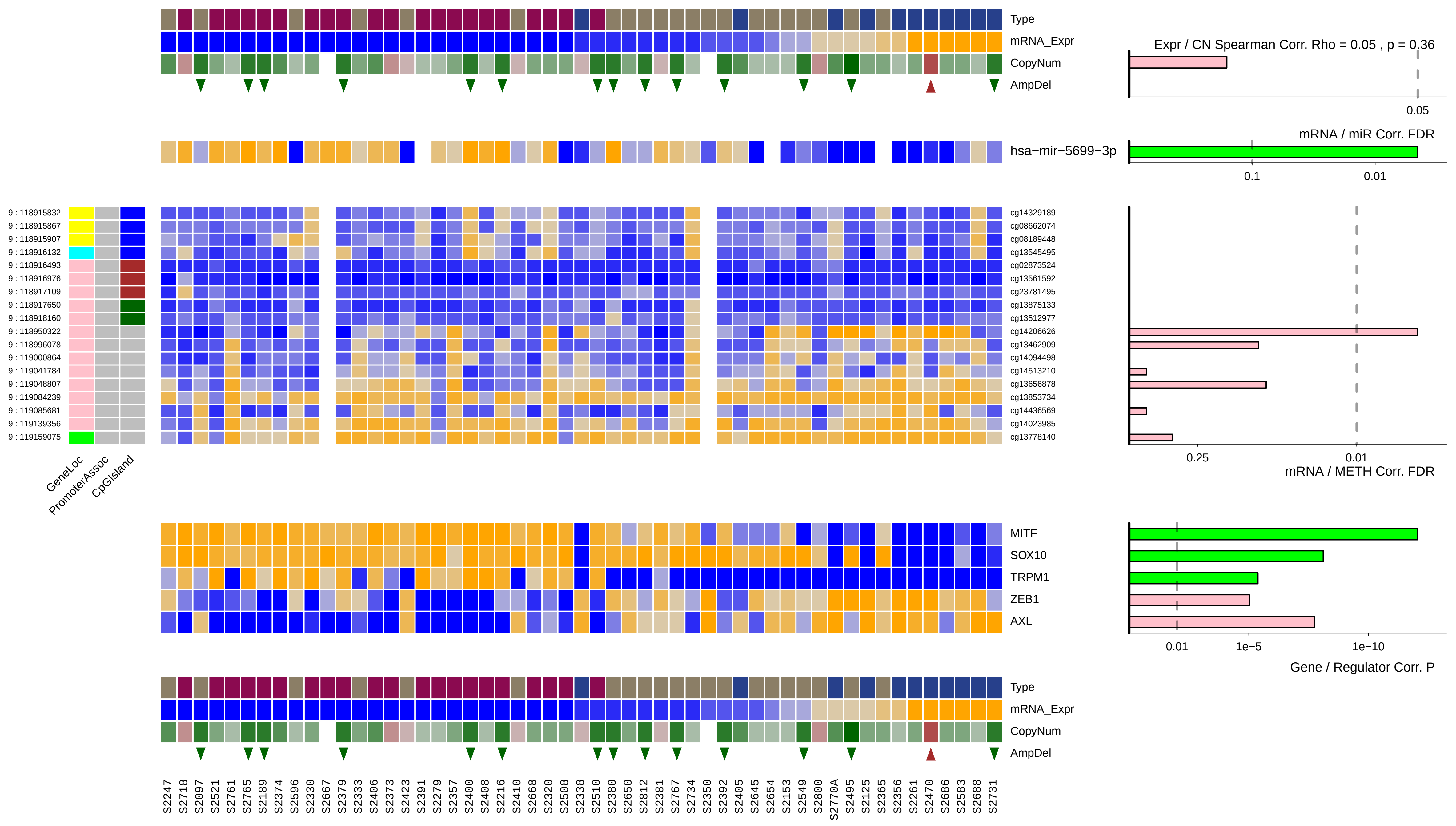
Type
mRNA_Expr
CopyNum
AmpDel
Expr / CN Spearman Corr. Rho = 0.05 , p = 0.36
0.05
mRNA / miR Corr. FDR
hsa−mir−5699−3p
0.1
0.01
9 : 118915832
9 : 118915867
9 : 118915907
9 : 118916132
9 : 118916493
9 : 118916976
9 : 118917109
9 : 118917650
9 : 118918160
9 : 118950322
9 : 118996078
9 : 119000864
9 : 119041784
9 : 119048807
9 : 119084239
9 : 119085681
9 : 119139356
9 : 119159075
cg14329189
cg08662074
cg08189448
cg13545495
cg02873524
cg13561592
cg23781495
cg13875133
cg13512977
cg14206626
cg13462909
cg14094498
cg14513210
cg13656878
cg13853734
cg14436569
cg14023985
cg13778140
GeneLoc
PromoterAssoc
CpGIsland
0.25
0.01
mRNA / METH Corr. FDR
MITF
SOX10
TRPM1
ZEB1
AXL
0.01
1e−5
1e−10
Gene / Regulator Corr. P
Type
mRNA_Expr
CopyNum
AmpDel
S2247 S2718 S2097 S2521 S2761 S2765 S2189 S2374 S2596 S2330 S2667 S2379 S2333 S2406 S2373 S2423 S2391 S2279 S2357 S2400 S2408 S2216 S2410 S2668 S2320 S2508 S2338 S2510 S2380 S2650 S2812 S2381 S2767 S2734 S2350 S2392 S2405 S2645 S2654 S2153 S2549 S2800 S2770A S2495 S2125 S2365 S2356 S2261 S2470 S2686 S2583 S2688 S2731

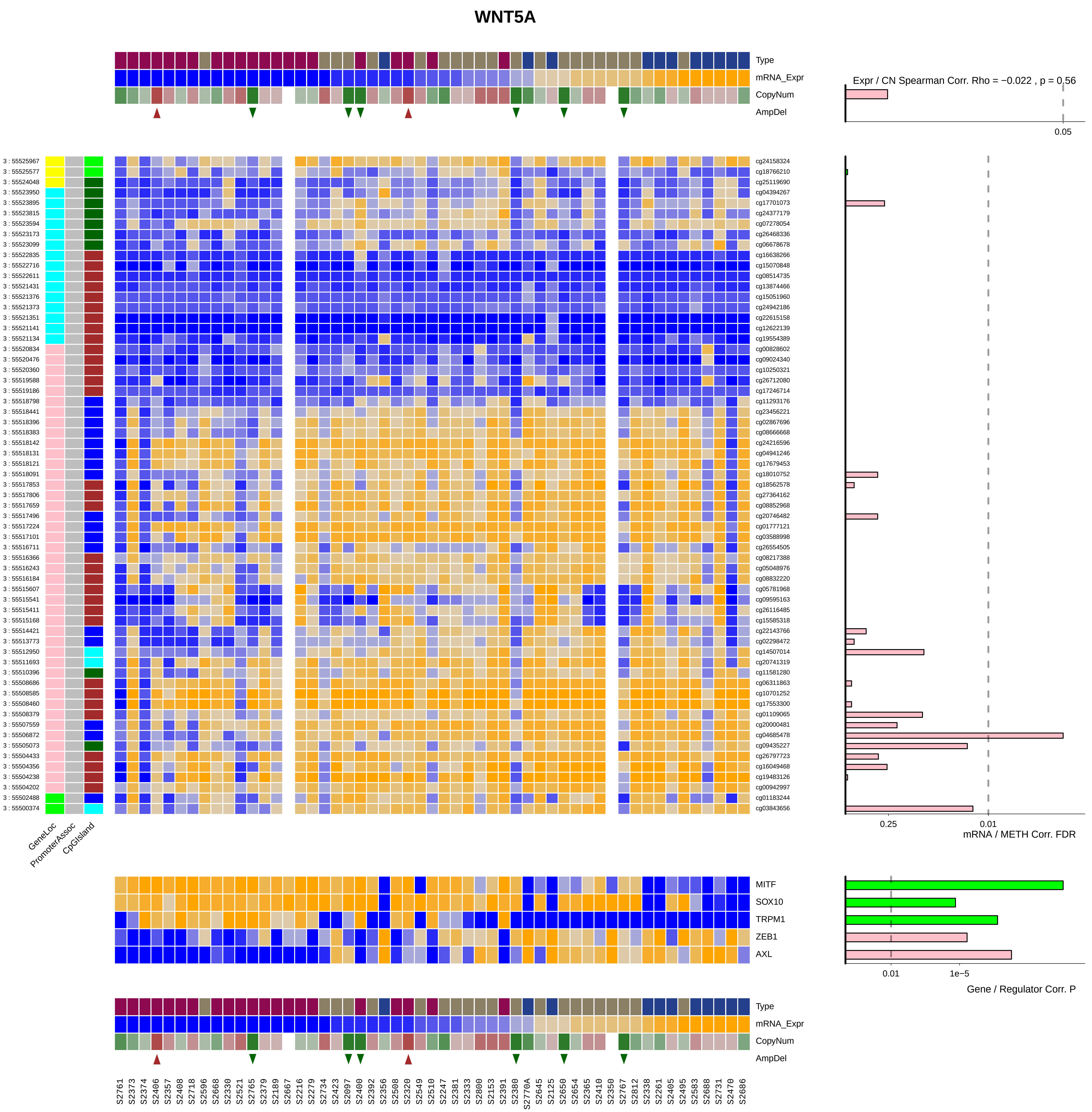
WNT5A
Type
mRNA_Expr
CopyNum
AmpDel
Expr / CN Spearman Corr. Rho = −0.022 , p = 0.56
0.05
GeneLoc
PromoterAssoc
CpGIsland
0.25
0.01
mRNA / METH Corr. FDR
MITF
SOX10
TRPM1
ZEB1
AXL
0.01
1e−5
Gene / Regulator Corr. P

TGM2

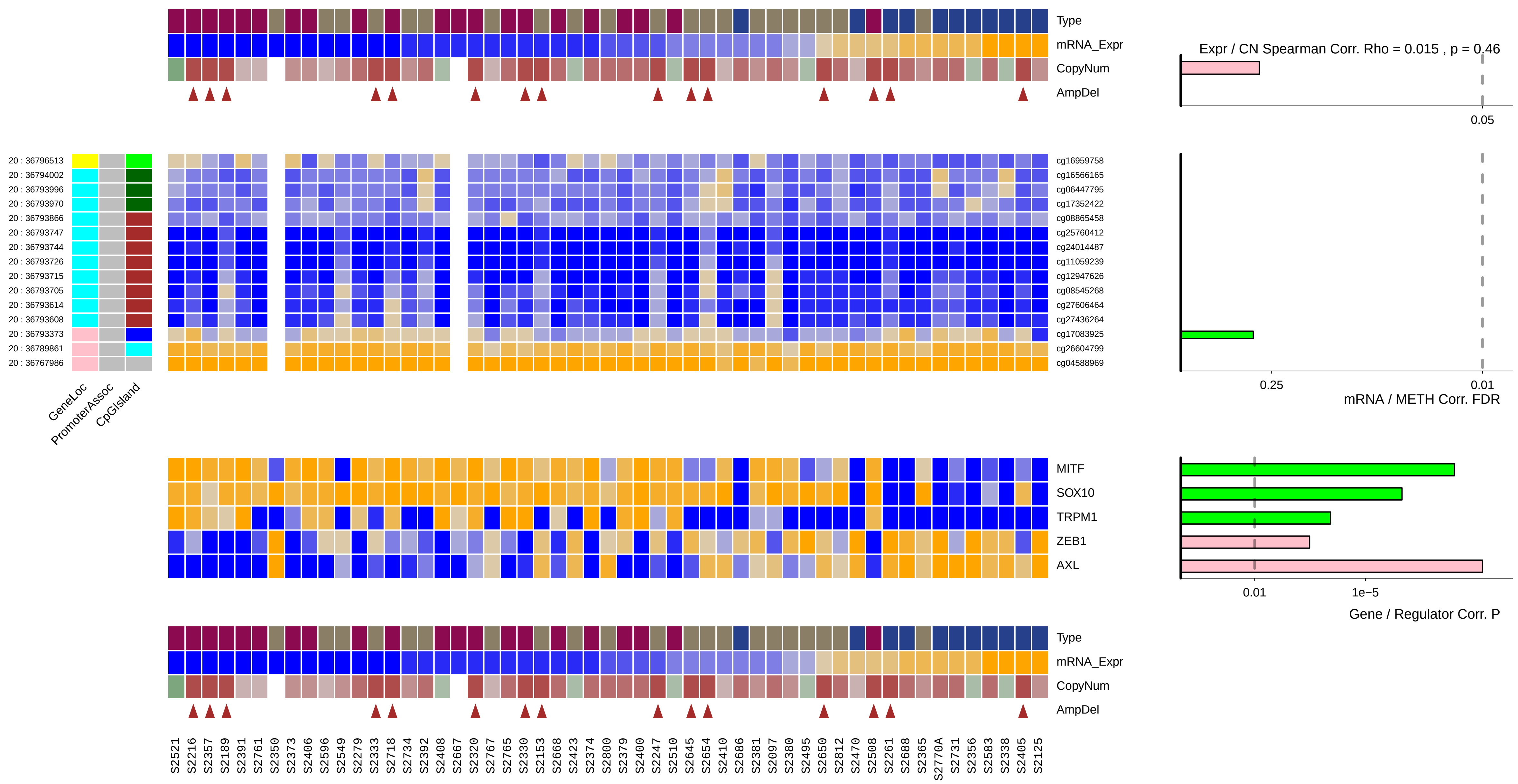
Type
mRNA_Expr
CopyNum
AmpDel
Expr / CN Spearman Corr. Rho = 0.015 , p = 0.46
0.05
20 : 36796513
20 : 36794002
20 : 36793996
20 : 36793970
20 : 36793866
20 : 36793747
20 : 36793744
20 : 36793726
20 : 36793715
20 : 36793705
20 : 36793614
20 : 36793608
20 : 36793373
20 : 36789861
20 : 36767986
cg16959758
cg16566165
cg06447795
cg17352422
cg08865458
cg25760412
cg24014487
cg11059239
cg12947626
cg08545268
cg27606464
cg27436264
cg17083925
cg26604799
cg04588969
GeneLoc
PromoterAssoc
CpGIsland
0.25
0.01
mRNA / METH Corr. FDR
MITF
SOX10
TRPM1
ZEB1
AXL
0.01
1e−5
Gene / Regulator Corr. P
Type
mRNA_Expr
CopyNum
AmpDel
S2521 S2216 S2357 S2189 S2391 S2761 S2350 S2373 S2406 S2596 S2549 S2279 S2333 S2718 S2734 S2392 S2408 S2667 S2320 S2767 S2765 S2330 S2153 S2668 S2423 S2374 S2800 S2379 S2400 S2247 S2510 S2645 S2654 S2410 S2686 S2381 S2097 S2380 S2495 S2650 S2812 S2470 S2508 S2261 S2688 S2365 S2770A S2731 S2356 S2583 S2338 S2405 S2125

SQRDL

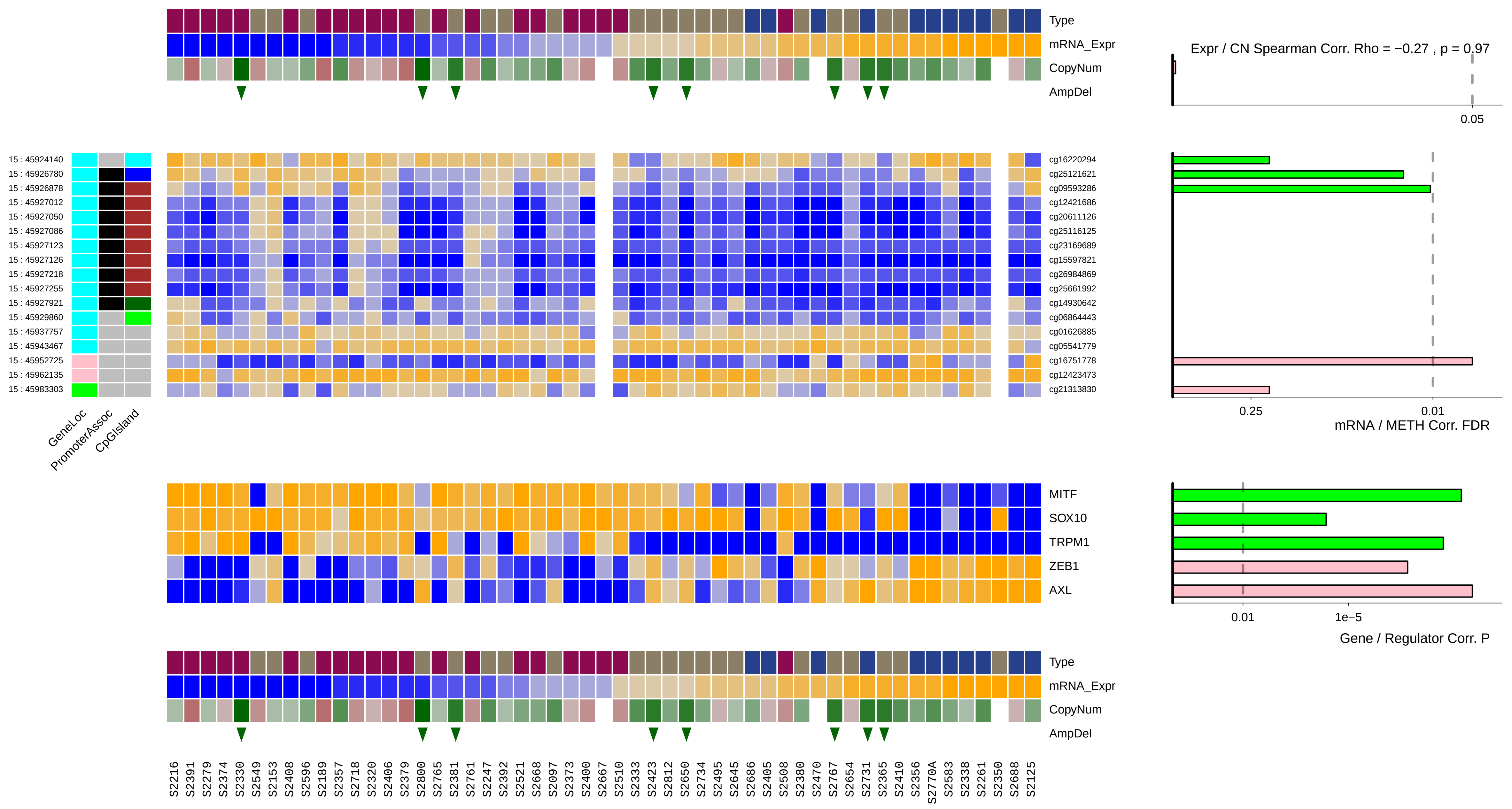

Type
mRNA_Expr
CopyNum
AmpDel
Expr / CN Spearman Corr. Rho = −0.27 , p = 0.97
0.05
15 : 45924140
15 : 45926780
15 : 45926878
15 : 45927012
15 : 45927050
15 : 45927086
15 : 45927123
15 : 45927126
15 : 45927218
15 : 45927255
15 : 45927921
15 : 45929860
15 : 45937757
15 : 45943467
15 : 45952725
15 : 45962135
15 : 45983303
cg16220294
cg25121621
cg09593286
cg12421686
cg20611126
cg25116125
cg23169689
cg15597821
cg26984869
cg25661992
cg14930642
cg06864443
cg01626885
cg05541779
cg16751778
cg12423473
cg21313830
GeneLoc
PromoterAssoc
CpGIsland
0.25
0.01
mRNA / METH Corr. FDR
MITF
SOX10
TRPM1
ZEB1
AXL
0.01
1e−5
Gene / Regulator Corr. P
S2216 S2391 S2279 S2374 S2330 S2549 S2153 S2408 S2596 S2189 S2357 S2718 S2320 S2406 S2379 S2800 S2765 S2381 S2761 S2247 S2392 S2521 S2668 S2097 S2373 S2400 S2667 S2510 S2333 S2423 S2812 S2650 S2734 S2495 S2645 S2686 S2405 S2508 S2380 S2470 S2767 S2654 S2731 S2365 S2410 S2356 S2770A S2583 S2338 S2261 S2350 S2688 S2125

IRF1

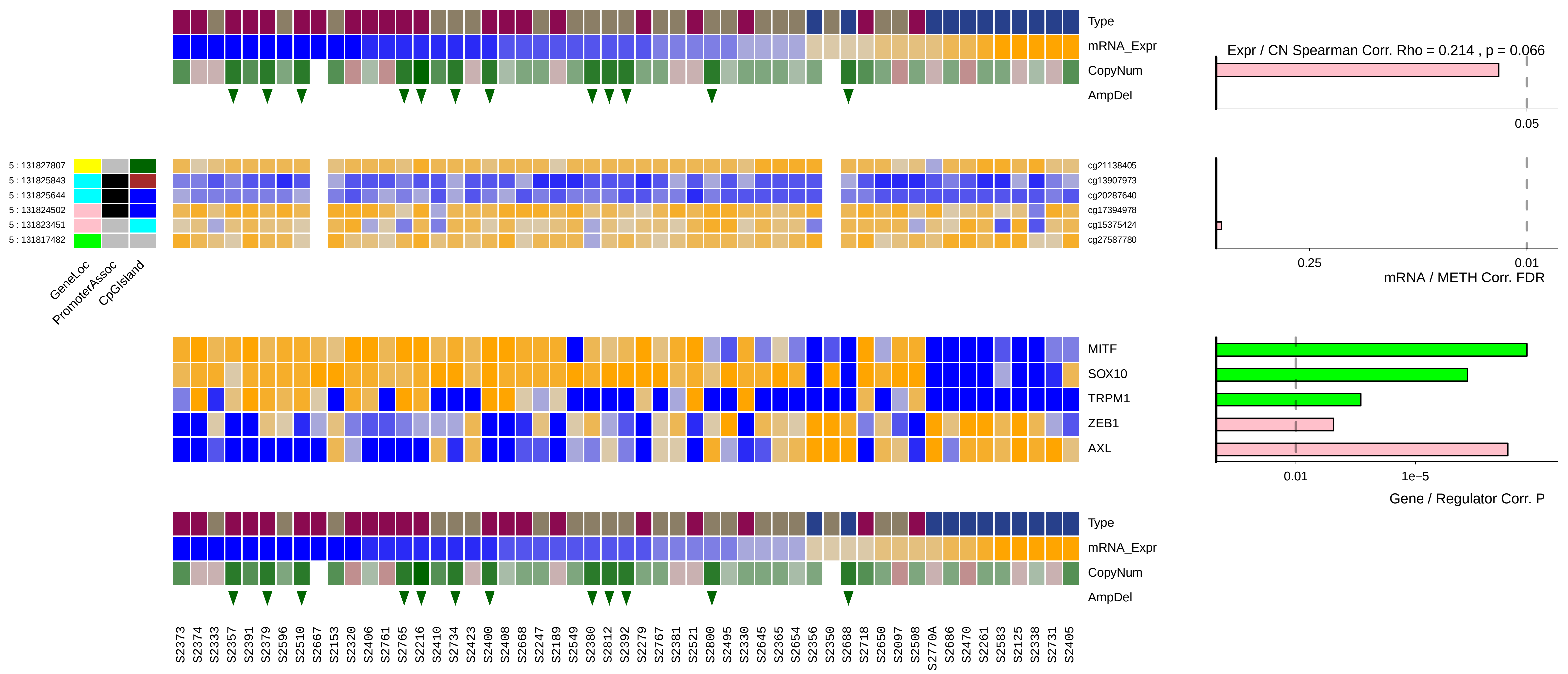
Type
mRNA_Expr
CopyNum
AmpDel
Expr / CN Spearman Corr. Rho = 0.214 , p = 0.066
0.05
5 : 131827807
5 : 131825843
5 : 131825644
5 : 131824502
5 : 131823451
5 : 131817482
GeneLoc
PromoterAssoc
CpGIsland
cg21138405
cg13907973
cg20287640
cg17394978
cg15375424
cg27587780
0.25
0.01
mRNA / METH Corr. FDR
MITF
SOX10
TRPM1
ZEB1
AXL
0.01
1e−5
Gene / Regulator Corr. P
S2373 S2374 S2333 S2357 S2391 S2379 S2596 S2510 S2667 S2153 S2320 S2406 S2761 S2765 S2216 S2410 S2734 S2423 S2400 S2408 S2668 S2247 S2189 S2549 S2380 S2812 S2392 S2279 S2767 S2381 S2521 S2800 S2495 S2330 S2645 S2365 S2654 S2356 S2350 S2688 S2718 S2650 S2097 S2508 S2770A S2686 S2470 S2261 S2583 S2125 S2338 S2731 S2405

SMURF2

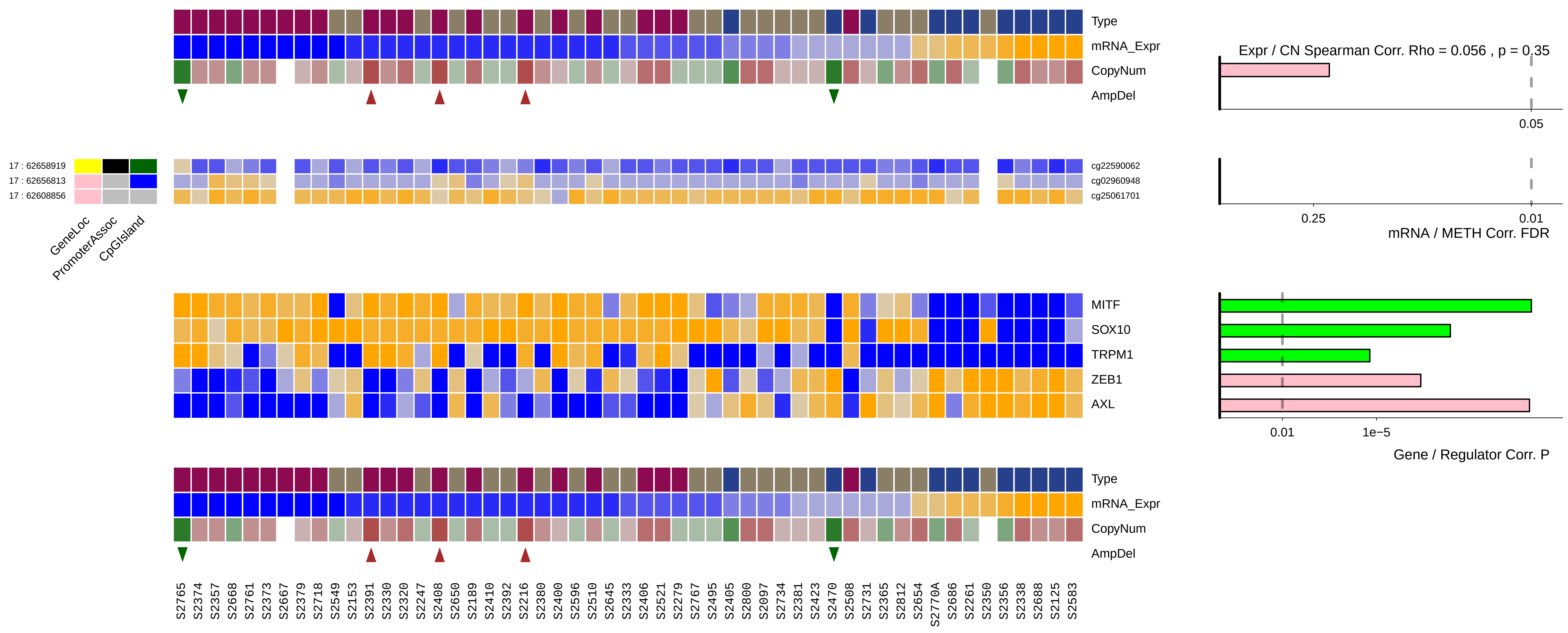
Type
mRNA_Expr
CopyNum
AmpDel
Expr / CN Spearman Corr. Rho = 0.056 , p = 0.35
0.05
17 : 62658919
17 : 62656813
17 : 62608856
GeneLoc
PromoterAssoc
CpGIsland
cg22590062
cg02960948
cg25061701
0.25
0.01
mRNA / METH Corr. FDR
MITF
SOX10
TRPM1
ZEB1
AXL
0.01
1e−5
Gene / Regulator Corr. P
Type
mRNA_Expr
CopyNum
AmpDel
S2765 S2374 S2357 S2668 S2761 S2373 S2667 S2379 S2718 S2549 S2153 S2391 S2330 S2320 S2247 S2408 S2650 S2189 S2410 S2392 S2216 S2380 S2400 S2596 S2510 S2645 S2333 S2406 S2521 S2279 S2767 S2495 S2405 S2800 S2097 S2734 S2381 S2423 S2470 S2508 S2731 S2365 S2812 S2654 S2770A S2686 S2261 S2350 S2356 S2338 S2688 S2125 S2583

IGFBP6

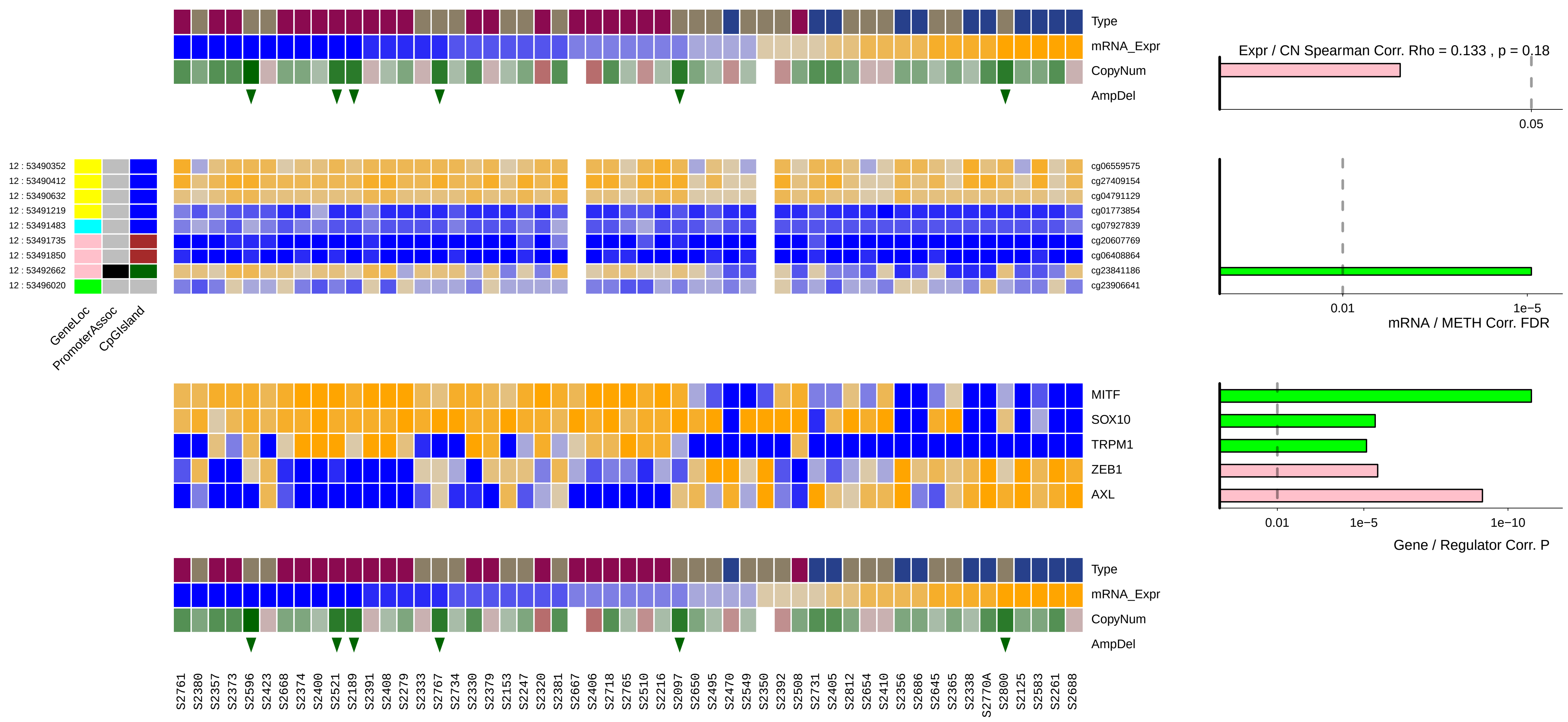

Type
mRNA_Expr
CopyNum
AmpDel
Expr / CN Spearman Corr. Rho = 0.133 , p = 0.18
0.05
12 : 53490352
12 : 53490412
12 : 53490632
12 : 53491219
12 : 53491483
12 : 53491735
12 : 53491850
12 : 53492662
12 : 53496020
GeneLoc
PromoterAssoc
CpGIsland
cg06559575
cg27409154
cg04791129
cg01773854
cg07927839
cg20607769
cg06408864
cg23841186
cg23906641
0.01
1e−5
mRNA / METH Corr. FDR
MITF
SOX10
TRPM1
ZEB1
AXL
0.01
1e−5
1e−10
Gene / Regulator Corr. P
Type
mRNA_Expr
CopyNum
AmpDel
S2761 S2380 S2357 S2373 S2596 S2423 S2668 S2374 S2400 S2521 S2189 S2391 S2408 S2279 S2333 S2767 S2734 S2330 S2379 S2153 S2247 S2320 S2381 S2667 S2406 S2718 S2765 S2510 S2216 S2097 S2650 S2495 S2470 S2549 S2350 S2392 S2508 S2731 S2405 S2812 S2654 S2410 S2356 S2686 S2645 S2365 S2338 S2770A S2800 S2125 S2583 S2261 S2688

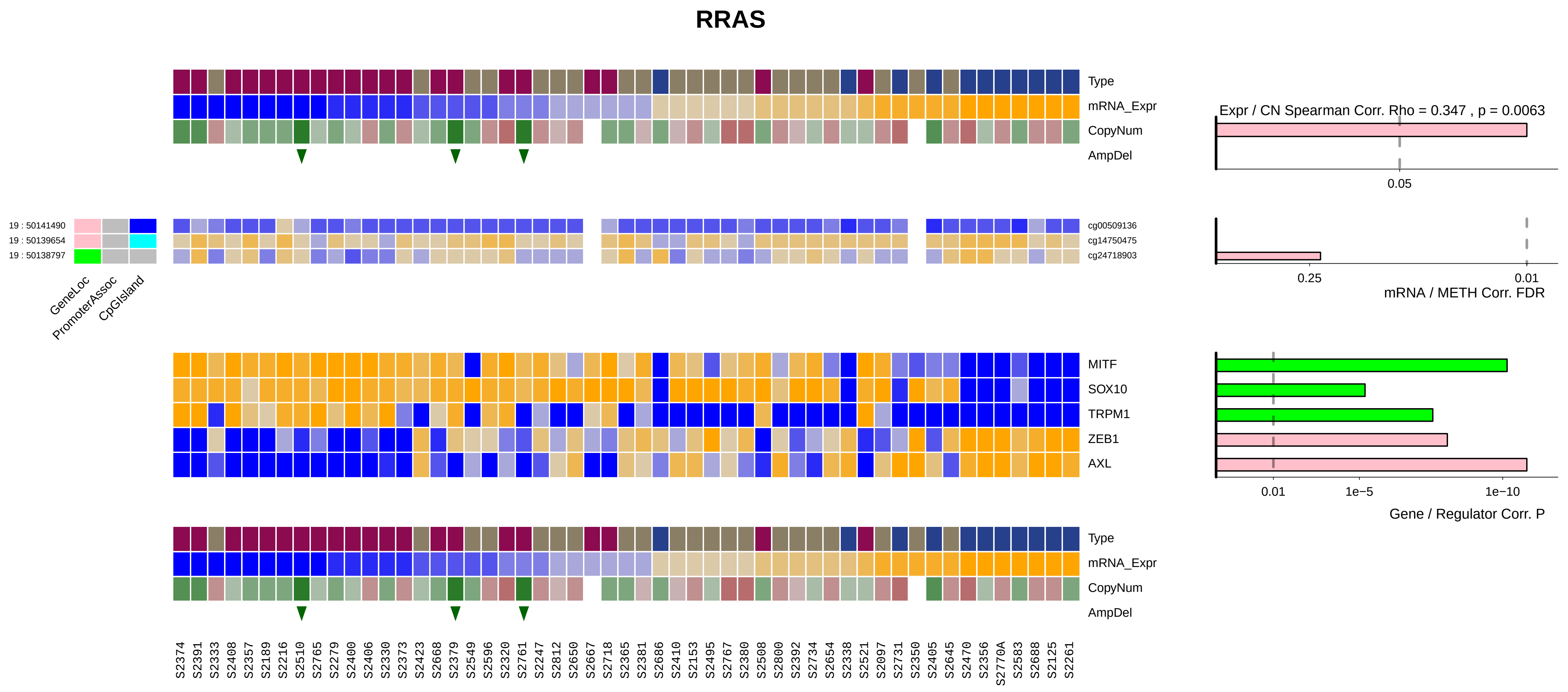
RRAS
Type
mRNA_Expr
CopyNum
AmpDel
Expr / CN Spearman Corr. Rho = 0.347 , p = 0.0063
0.05
19 : 50141490
19 : 50139654
19 : 50138797
cg00509136
cg14750475
cg24718903
0.25
0.01
mRNA / METH Corr. FDR
GeneLoc
PromoterAssoc
CpGIsland
MITF
SOX10
TRPM1
ZEB1
AXL
0.01
1e−5
1e−10
Gene / Regulator Corr. P
Type
mRNA_Expr
CopyNum
AmpDel
S2374 S2391 S2333 S2408 S2357 S2189 S2216 S2510 S2765 S2279 S2400 S2406 S2330 S2373 S2423 S2668 S2379 S2549 S2596 S2320 S2761 S2247 S2812 S2650 S2667 S2718 S2365 S2381 S2686 S2410 S2153 S2495 S2767 S2380 S2508 S2800 S2392 S2734 S2654 S2338 S2521 S2097 S2731 S2350 S2405 S2645 S2470 S2356 S2770A S2583 S2688 S2125 S2261

TGFBR2

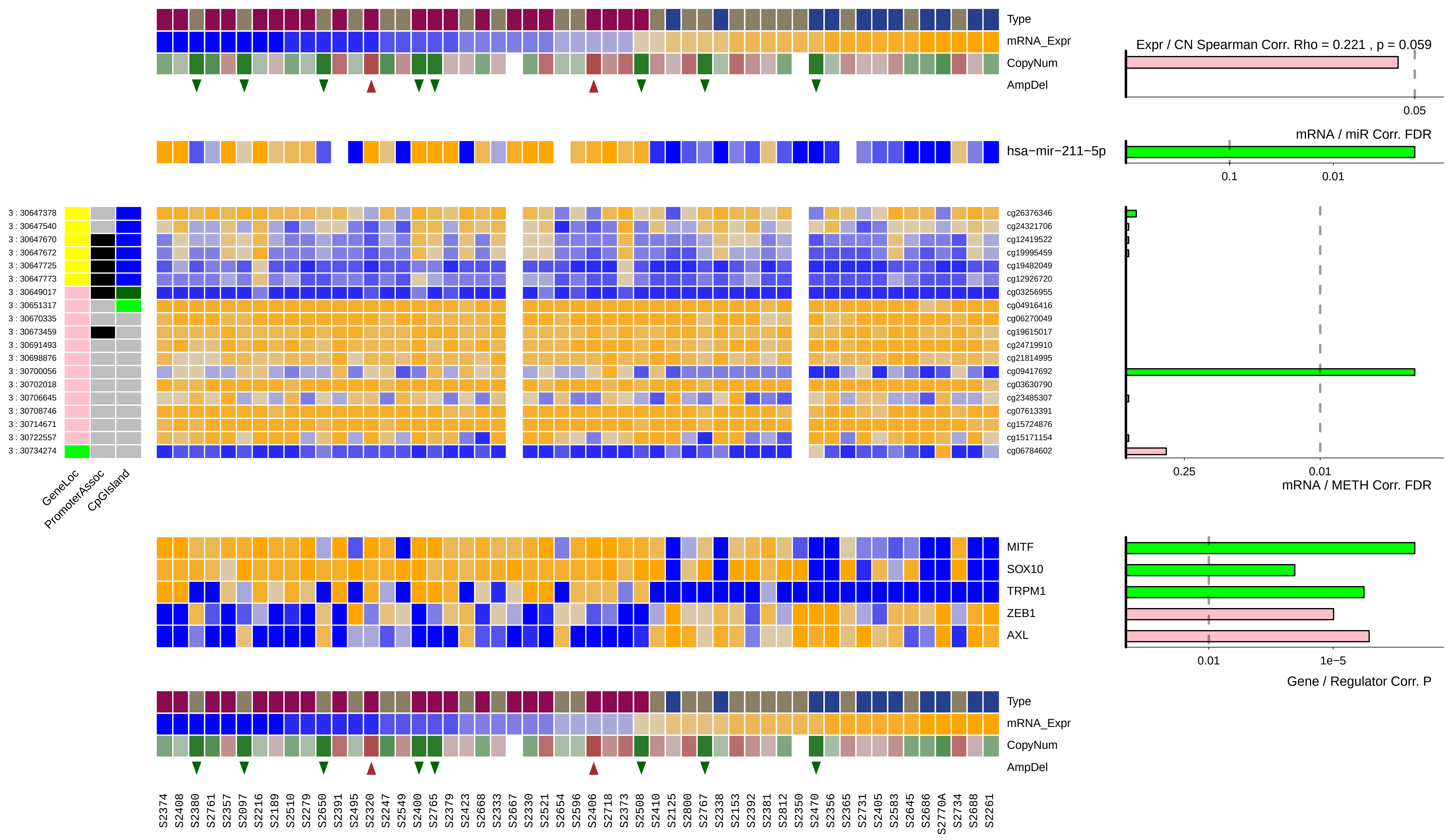

Type
mRNA_Expr
CopyNum
AmpDel
Expr / CN Spearman Corr. Rho = 0.221 , p = 0.059
0.05
hsa−mir−211−5p
mRNA / miR Corr. FDR
0.1
0.01
3 : 30647378
3 : 30647540
3 : 30647670
3 : 30647672
3 : 30647725
3 : 30647773
3 : 30649017
3 : 30651317
3 : 30670335
3 : 30673459
3 : 30691493
3 : 30698876
3 : 30700056
3 : 30702018
3 : 30706645
3 : 30708746
3 : 30714671
3 : 30722557
3 : 30734274
GeneLoc
PromoterAssoc
CpGIsland
cg26376346
cg24321706
cg12419522
cg19995459
cg19482049
cg12926720
cg03256955
cg04916416
cg06270049
cg19615017
cg24719910
cg21814995
cg09417692
cg03630790
cg23485307
cg07613391
cg15724876
cg15171154
cg06784602
0.25
0.01
mRNA / METH Corr. FDR
MITF
SOX10
TRPM1
ZEB1
AXL
0.01
1e−5
Gene / Regulator Corr. P
Type
mRNA_Expr
CopyNum
AmpDel
S2374 S2408 S2380 S2761 S2357 S2097 S2216 S2189 S2510 S2279 S2650 S2391 S2495 S2320 S2247 S2549 S2400 S2765 S2379 S2423 S2668 S2333 S2667 S2330 S2521 S2654 S2596 S2406 S2718 S2373 S2508 S2410 S2125 S2800 S2767 S2338 S2153 S2392 S2381 S2812 S2350 S2470 S2356 S2365 S2731 S2405 S2583 S2645 S2686 S2770A S2734 S2688 S2261

TPM1

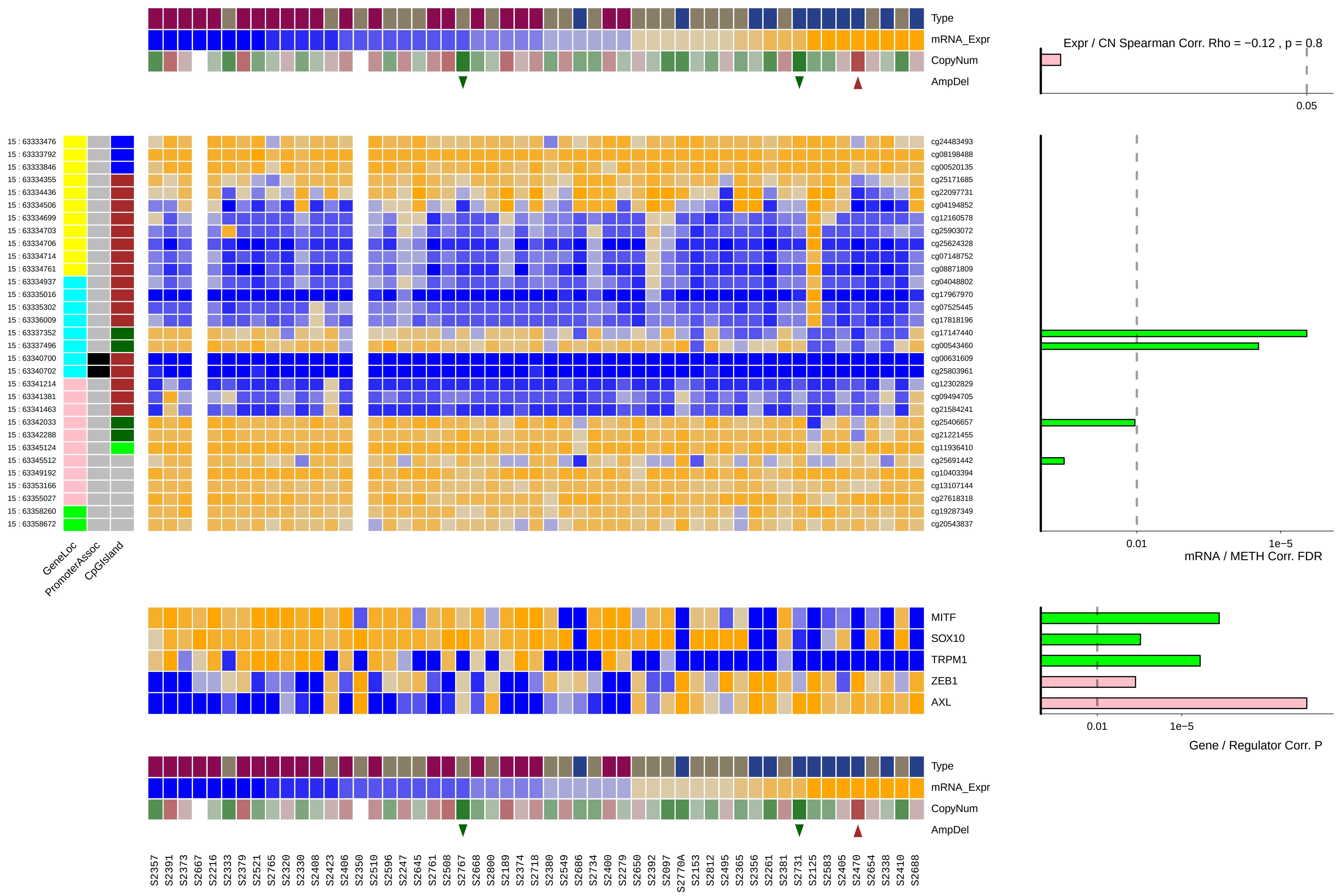

Type
mRNA_Expr
CopyNum
AmpDel
Expr / CN Spearman Corr. Rho = −0.12 , p = 0.8
0.05
15 : 63333476
15 : 63333792
15 : 63333846
15 : 63334355
15 : 63334436
15 : 63334506
15 : 63334699
15 : 63334703
15 : 63334706
15 : 63334714
15 : 63334761
15 : 63334937
15 : 63335016
15 : 63335302
15 : 63336009
15 : 63337352
15 : 63337496
15 : 63340700
15 : 63340702
15 : 63341214
15 : 63341381
15 : 63341463
15 : 63342033
15 : 63342288
15 : 63345124
15 : 63345512
15 : 63349192
15 : 63353166
15 : 63355027
15 : 63358260
15 : 63358672
cg24483493
cg08198488
cg00520135
cg25171685
cg22097731
cg04194852
cg12160578
cg25903072
cg25624328
cg07148752
cg08871809
cg04048802
cg17967970
cg07525445
cg17818196
cg17147440
cg00543460
cg00631609
cg25803961
cg12302829
cg09494705
cg21584241
cg25406657
cg21221455
cg11936410
cg25691442
cg10403394
cg13107144
cg27618318
cg19287349
cg20543837
GeneLoc
PromoterAssoc
CpGIsland
0.01
1e−5
mRNA / METH Corr. FDR
MITF
SOX10
TRPM1
ZEB1
AXL
0.01
1e−5
Gene / Regulator Corr. P
Type
mRNA_Expr
CopyNum
AmpDel
S2357 S2391 S2373 S2667 S2216 S2333 S2379 S2521 S2765 S2320 S2330 S2408 S2423 S2406 S2350 S2510 S2596 S2247 S2645 S2761 S2508 S2767 S2668 S2800 S2189 S2374 S2718 S2380 S2549 S2686 S2734 S2400 S2279 S2650 S2392 S2097 S2770A S2153 S2812 S2495 S2365 S2356 S2261 S2381 S2731 S2125 S2583 S2405 S2470 S2654 S2338 S2410 S2688

BDNF

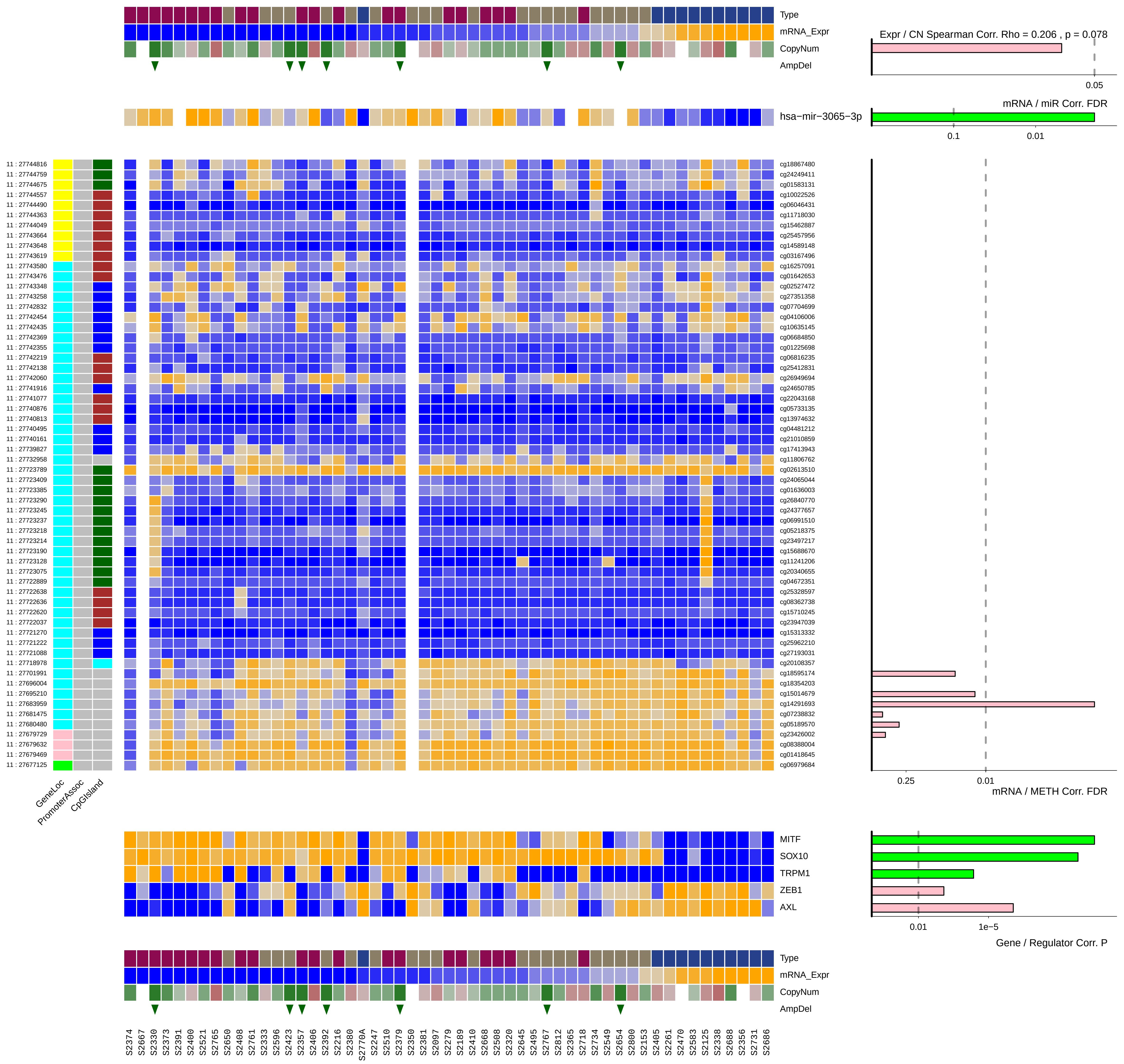
Type
mRNA_Expr
CopyNum
AmpDel
Expr / CN Spearman Corr. Rho = 0.206 , p = 0.078
0.05
hsa−mir−3065−3p
mRNA / miR Corr. FDR
0.1
0.01
0.25
0.01
mRNA / METH Corr. FDR
GeneLoc
PromoterAssoc
CpGIsland
MITF
SOX10
TRPM1
ZEB1
AXL
0.01
1e−5
Gene / Regulator Corr. P

GBP1

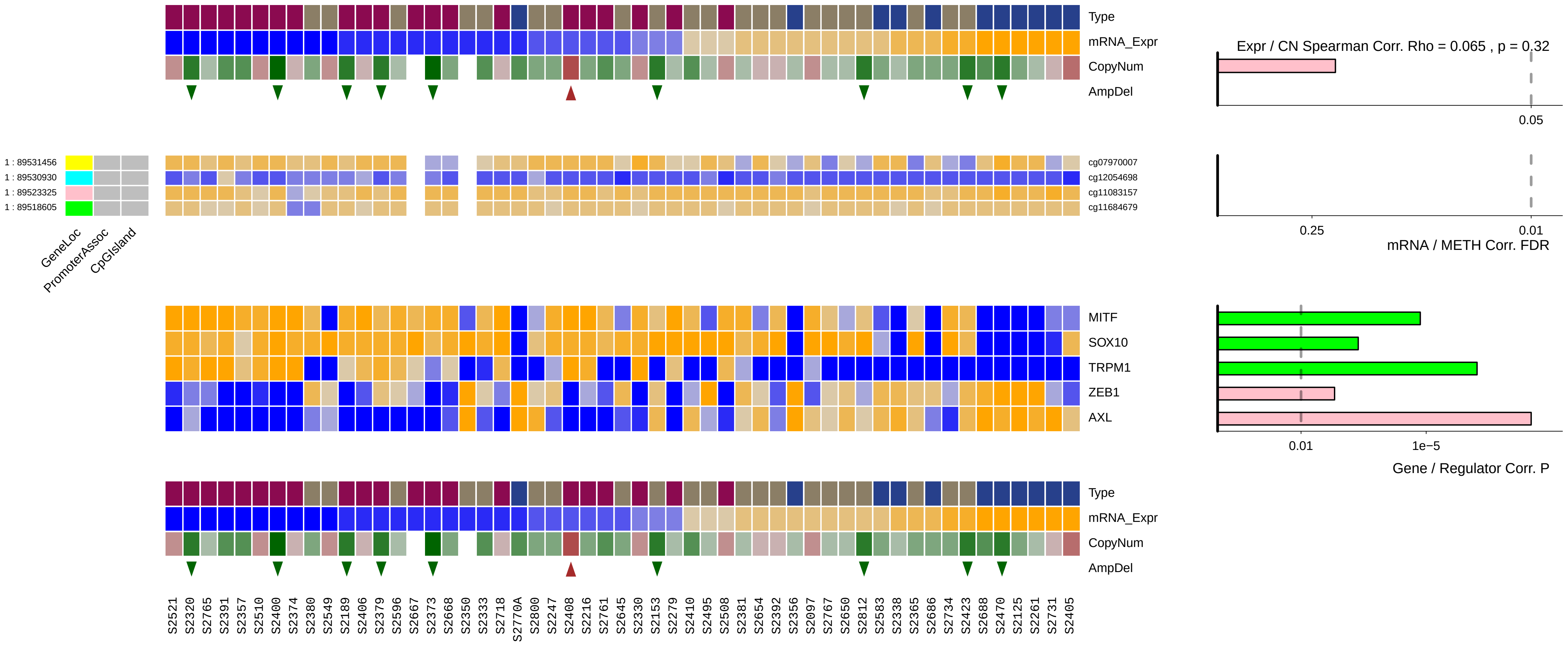

Type
mRNA_Expr
CopyNum
AmpDel
Expr / CN Spearman Corr. Rho = 0.065 , p = 0.32
0.05
1 : 89531456
1 : 89530930
1 : 89523325
1 : 89518605
cg07970007
cg12054698
cg11083157
cg11684679
GeneLoc
PromoterAssoc
CpGIsland
0.25
0.01
mRNA / METH Corr. FDR
MITF
SOX10
TRPM1
ZEB1
AXL
0.01
1e−5
Gene / Regulator Corr. P
S2521 S2320 S2765 S2391 S2357 S2510 S2400 S2374 S2380 S2549 S2189 S2406 S2379 S2596 S2667 S2373 S2668 S2350 S2333 S2718 S2770A S2800 S2247 S2408 S2216 S2761 S2645 S2330 S2153 S2279 S2410 S2495 S2508 S2381 S2654 S2392 S2356 S2097 S2767 S2650 S2812 S2583 S2338 S2365 S2686 S2734 S2423 S2688 S2470 S2125 S2261 S2731 S2405

SRPX2

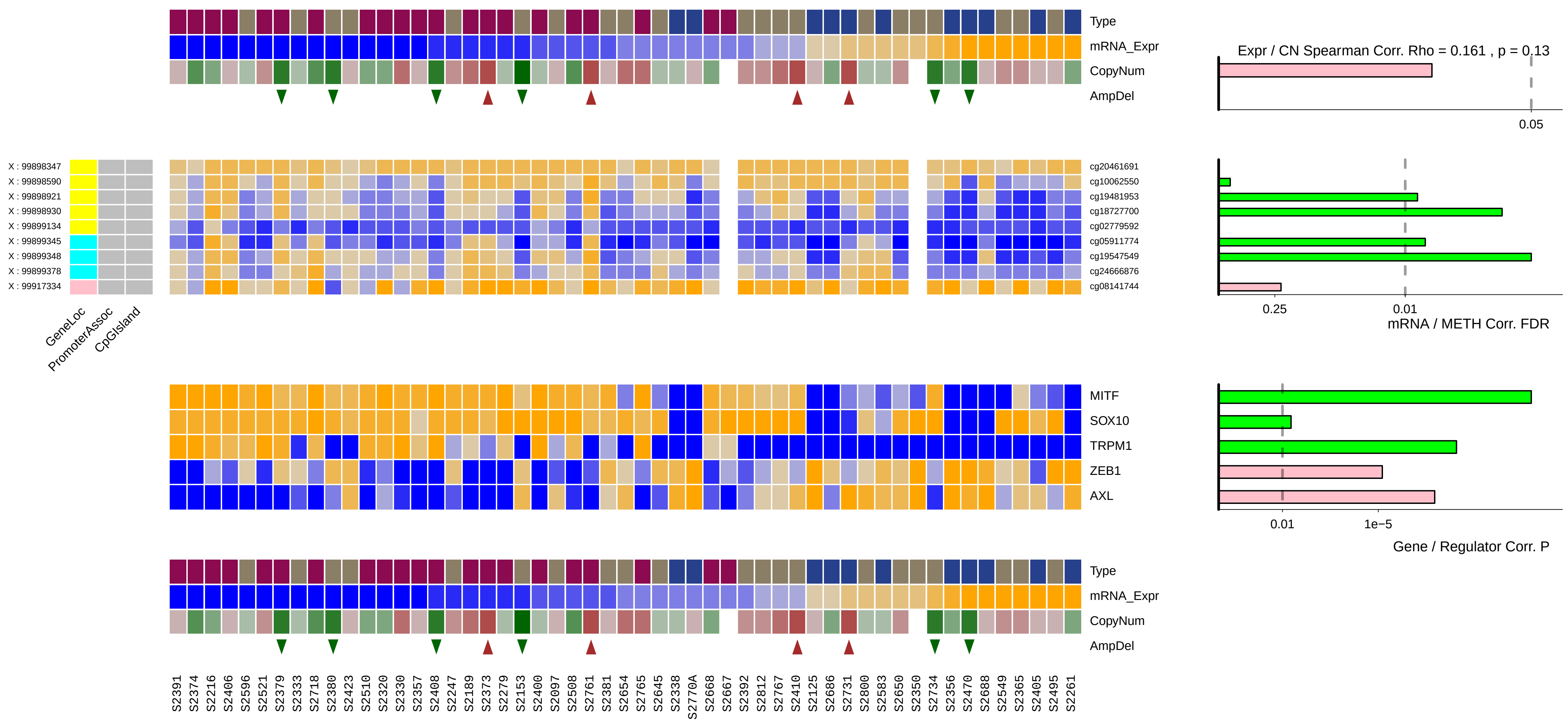

Type
mRNA_Expr
CopyNum
AmpDel
Expr / CN Spearman Corr. Rho = 0.161 , p = 0.13
0.05
X : 99898347
X : 99898590
X : 99898921
X : 99898930
X : 99899134
X : 99899345
X : 99899348
X : 99899378
X : 99917334
GeneLoc
PromoterAssoc
CpGIsland
cg20461691
cg10062550
cg19481953
cg18727700
cg02779592
cg05911774
cg19547549
cg24666876
cg08141744
0.25
0.01
mRNA / METH Corr. FDR
MITF
SOX10
TRPM1
ZEB1
AXL
0.01
1e−5
Gene / Regulator Corr. P
S2391 S2374 S2216 S2406 S2596 S2521 S2379 S2333 S2718 S2380 S2423 S2510 S2320 S2330 S2357 S2408 S2247 S2189 S2373 S2279 S2153 S2400 S2097 S2508 S2761 S2381 S2654 S2765 S2645 S2338 S2770A S2668 S2667 S2392 S2812 S2767 S2410 S2125 S2686 S2731 S2800 S2583 S2650 S2350 S2734 S2356 S2470 S2688 S2549 S2365 S2405 S2495 S2261

SMAGP

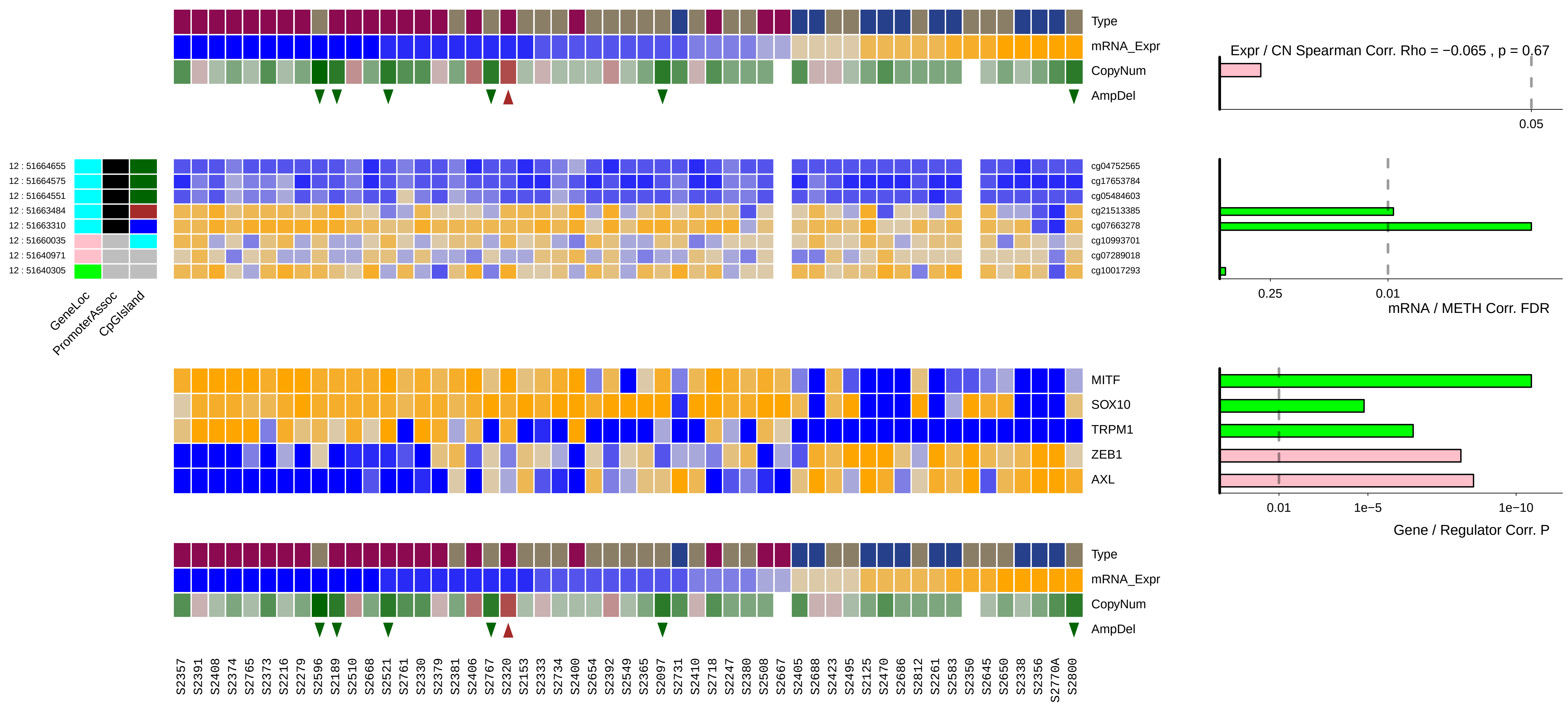

Type
mRNA_Expr
CopyNum
AmpDel
Expr / CN Spearman Corr. Rho = −0.065 , p = 0.67
0.05
12 : 51664655
12 : 51664575
12 : 51664551
12 : 51663484
12 : 51663310
12 : 51660035
12 : 51640971
12 : 51640305
GeneLoc
PromoterAssoc
CpGIsland
cg04752565
cg17653784
cg05484603
cg21513385
cg07663278
cg10993701
cg07289018
cg10017293
0.25
0.01
mRNA / METH Corr. FDR
MITF
SOX10
TRPM1
ZEB1
AXL
0.01
1e−5
1e−10
Gene / Regulator Corr. P
Type
mRNA_Expr
CopyNum
AmpDel
S2357 S2391 S2408 S2374 S2765 S2373 S2216 S2279 S2596 S2189 S2510 S2668 S2521 S2761 S2330 S2379 S2381 S2406 S2767 S2320 S2153 S2333 S2734 S2400 S2654 S2392 S2549 S2365 S2097 S2731 S2410 S2718 S2247 S2380 S2508 S2667 S2405 S2688 S2423 S2495 S2125 S2470 S2686 S2812 S2261 S2583 S2350 S2645 S2650 S2338 S2356 S2770A S2800

EXT1

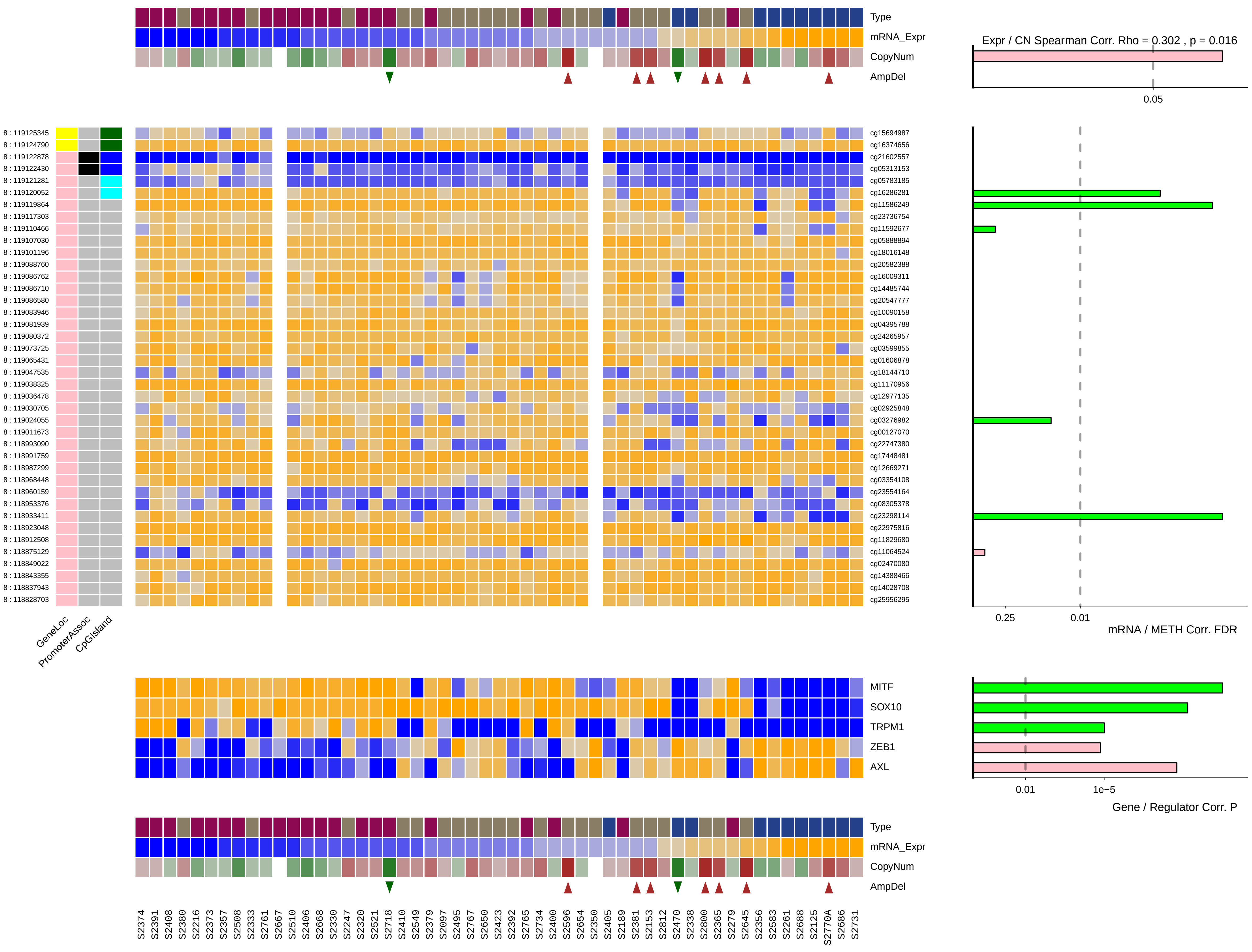

Type
mRNA_Expr
CopyNum
AmpDel
Expr / CN Spearman Corr. Rho = 0.302 , p = 0.016
0.05
8 : 119125345
8 : 119124790
8 : 119122878
8 : 119122430
8 : 119121281
8 : 119120052
8 : 119119864
8 : 119117303
8 : 119110466
8 : 119107030
8 : 119101196
8 : 119088760
8 : 119086762
8 : 119086710
8 : 119086580
8 : 119083946
8 : 119081939
8 : 119080372
8 : 119073725
8 : 119065431
8 : 119047535
8 : 119038325
8 : 119036478
8 : 119030705
8 : 119024055
8 : 119011673
8 : 118993090
8 : 118991759
8 : 118987299
8 : 118968448
8 : 118960159
8 : 118953376
8 : 118933411
8 : 118923048
8 : 118912508
8 : 118875129
8 : 118849022
8 : 118843355
8 : 118837943
8 : 118828703
cg15694987
cg16374656
cg21602557
cg05313153
cg05783185
cg16286281
cg11586249
cg23736754
cg11592677
cg05888894
cg18016148
cg20582388
cg16009311
cg14485744
cg20547777
cg10090158
cg04395788
cg24265957
cg03599855
cg01606878
cg18144710
cg11170956
cg12977135
cg02925848
cg03276982
cg00127070
cg22747380
cg17448481
cg12669271
cg03354108
cg23554164
cg08305378
cg23298114
cg22975816
cg11829680
cg11064524
cg02470080
cg14388466
cg14028708
cg25956295
0.25
0.01
mRNA / METH Corr. FDR
GeneLoc
PromoterAssoc
CpGIsland
MITF
SOX10
TRPM1
ZEB1
AXL
0.01
1e−5
Gene / Regulator Corr. P
Type
mRNA_Expr
CopyNum
AmpDel
S2374
S2391
S2408
S2380
S2216
S2373
S2357
S2508
S2333
S2761
S2667
S2510
S2406
S2668
S2330
S2247
S2320
S2521
S2718
S2410
S2549
S2379
S2097
S2495
S2767
S2650
S2423
S2392
S2765
S2734
S2400
S2596
S2654
S2350
S2405
S2189
S2381
S2153
S2812
S2470
S2338
S2800
S2365
S2279
S2645
S2356
S2583
S2261
S2688
S2125
S2770A
S2686
S2731

TMEM158

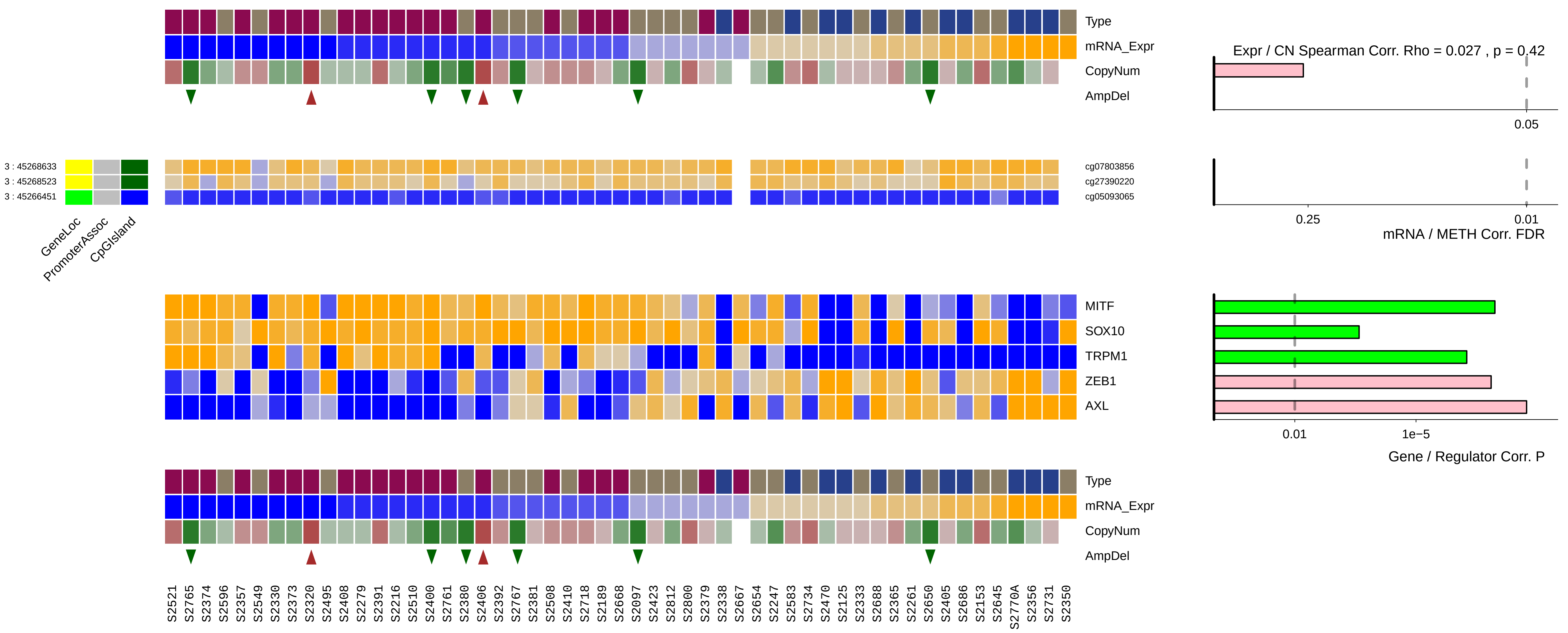

Type
mRNA_Expr
CopyNum
AmpDel
Expr / CN Spearman Corr. Rho = 0.027 , p = 0.42
0.05
3 : 45268633
3 : 45268523
3 : 45266451
GeneLoc
PromoterAssoc
CpGIsland
cg07803856
cg27390220
cg05093065
0.25
0.01
mRNA / METH Corr. FDR
MITF
SOX10
TRPM1
ZEB1
AXL
0.01
1e−5
Gene / Regulator Corr. P
Type
mRNA_Expr
CopyNum
AmpDel
S2521 S2765 S2374 S2596 S2357 S2549 S2330 S2373 S2320 S2495 S2408 S2279 S2391 S2216 S2510 S2400 S2761 S2380 S2406 S2392 S2767 S2381 S2508 S2410 S2718 S2189 S2668 S2097 S2423 S2812 S2800 S2379 S2338 S2667 S2654 S2247 S2583 S2734 S2470 S2125 S2333 S2688 S2365 S2261 S2650 S2405 S2686 S2153 S2645 S2770A S2356 S2731 S2350

AFAP1

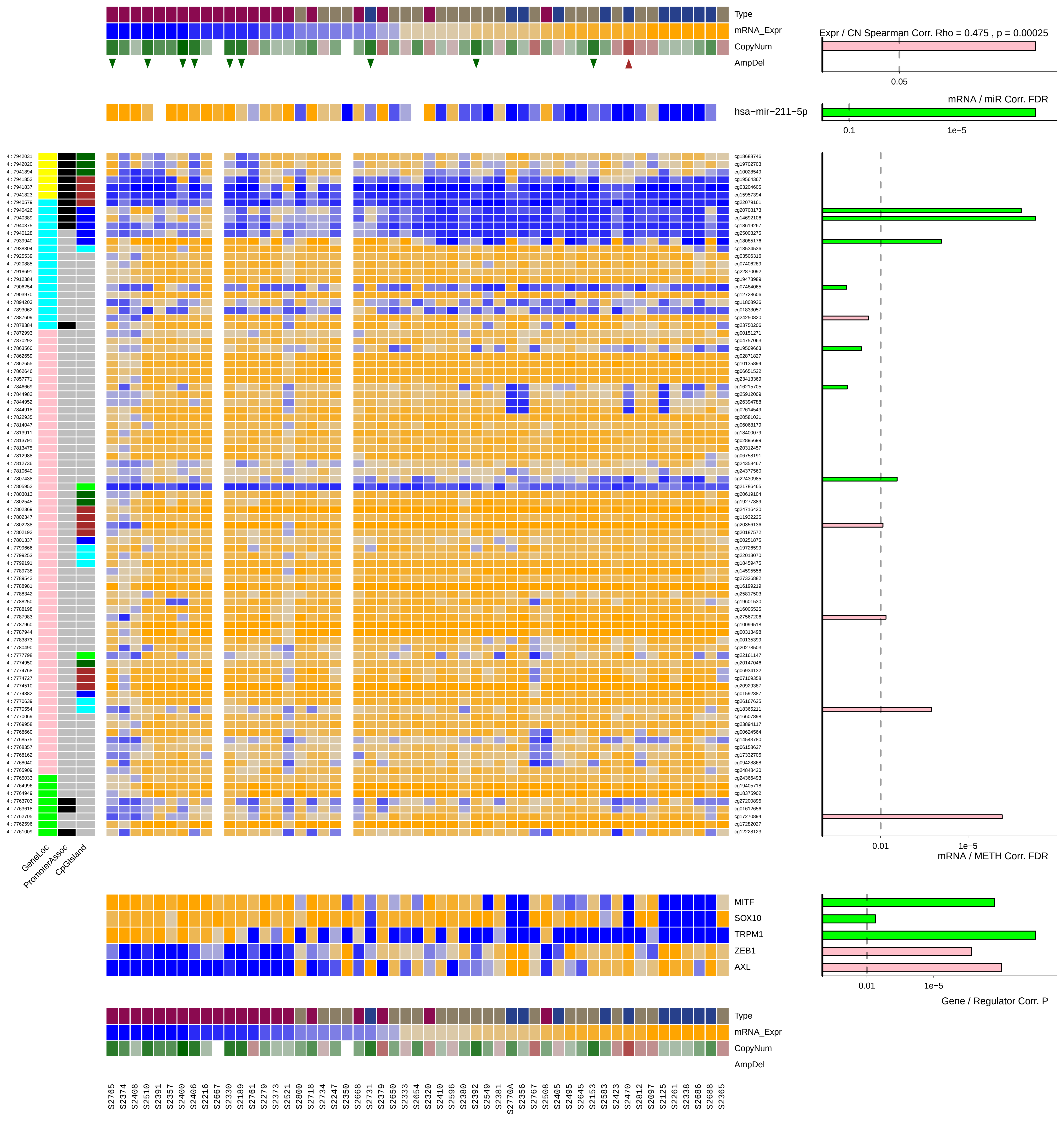

Type
mRNA_Expr
CopyNum
AmpDel
Expr / CN Spearman Corr. Rho = 0.475 , p = 0.00025
0.05
mRNA / miR Corr. FDR
hsa−mir−211−5p
0.1
1e−5
GeneLoc
PromoterAssoc
CpGIsland
0.01
1e−5
mRNA / METH Corr. FDR
MITF
SOX10
TRPM1
ZEB1
AXL
0.01
1e−5
Gene / Regulator Corr. P
Type
mRNA_Expr
CopyNum
AmpDel
S2765
S2374
S2408
S2510
S2391
S2357
S2400
S2406
S2216
S2667
S2330
S2189
S2761
S2279
S2373
S2521
S2800
S2718
S2734
S2247
S2350
S2668
S2731
S2379
S2650
S2333
S2654
S2320
S2410
S2596
S2380
S2392
S2549
S2381
S2770A
S2356
S2767
S2508
S2405
S2495
S2645
S2153
S2583
S2423
S2470
S2812
S2097
S2125
S2261
S2338
S2686
S2688
S2365

ITGA2

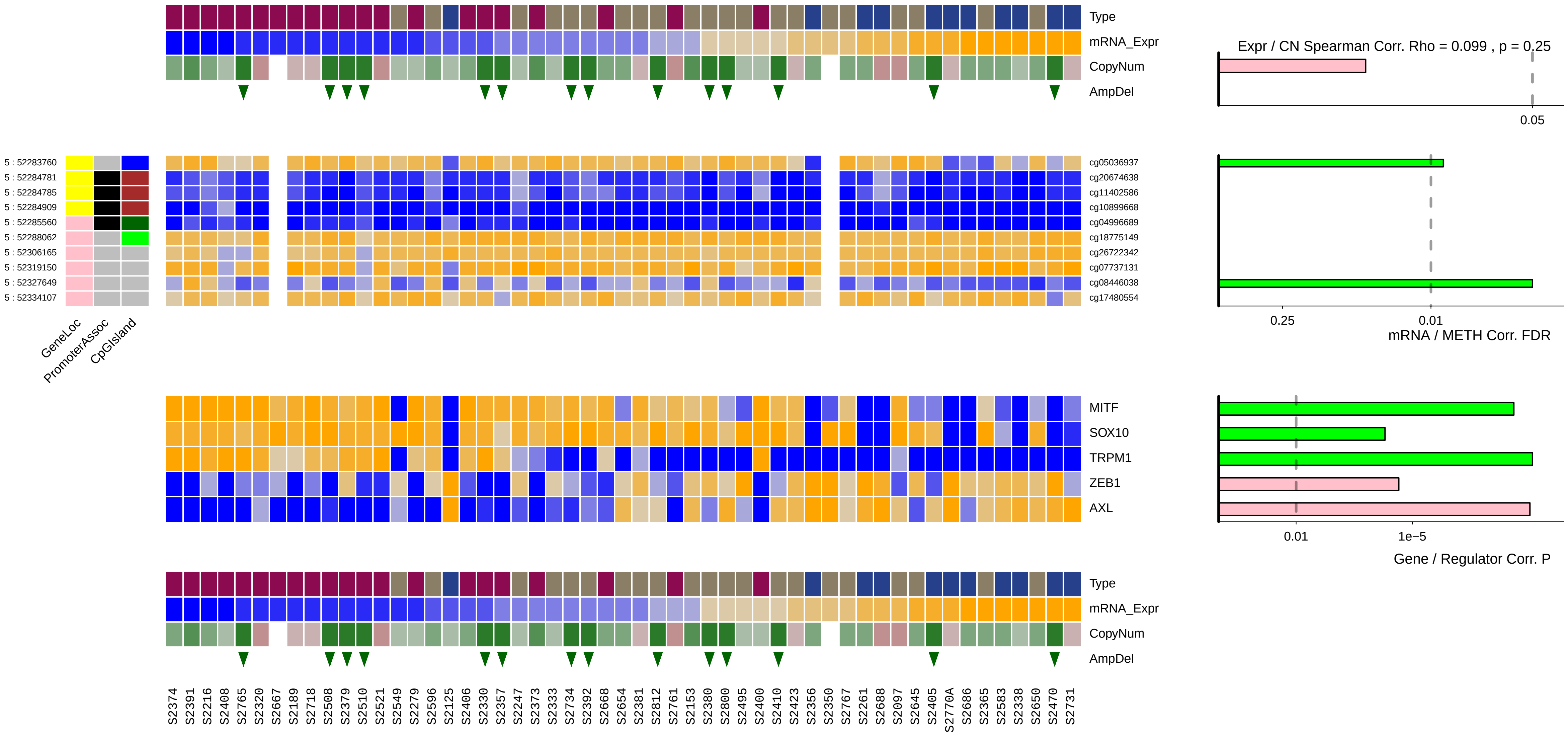

Type
mRNA_Expr
CopyNum
AmpDel
Expr / CN Spearman Corr. Rho = 0.099 , p = 0.25
0.05
5 : 52283760
5 : 52284781
5 : 52284785
5 : 52284909
5 : 52285560
5 : 52288062
5 : 52306165
5 : 52319150
5 : 52327649
5 : 52334107
GeneLoc
PromoterAssoc
CpGIsland
cg05036937
cg20674638
cg11402586
cg10899668
cg04996689
cg18775149
cg26722342
cg07737131
cg08446038
cg17480554
0.25
0.01
mRNA / METH Corr. FDR
MITF
SOX10
TRPM1
ZEB1
AXL
0.01
1e−5
Gene / Regulator Corr. P
Type
mRNA_Expr
CopyNum
AmpDel
S2374 S2391 S2216 S2408 S2765 S2320 S2667 S2189 S2718 S2508 S2379 S2510 S2521 S2549 S2279 S2596 S2125 S2406 S2330 S2357 S2247 S2373 S2333 S2734 S2392 S2668 S2654 S2381 S2812 S2761 S2153 S2380 S2800 S2495 S2400 S2410 S2423 S2356 S2350 S2767 S2261 S2688 S2097 S2645 S2405 S2770A S2686 S2365 S2583 S2338 S2650 S2470 S2731

AKR1C3

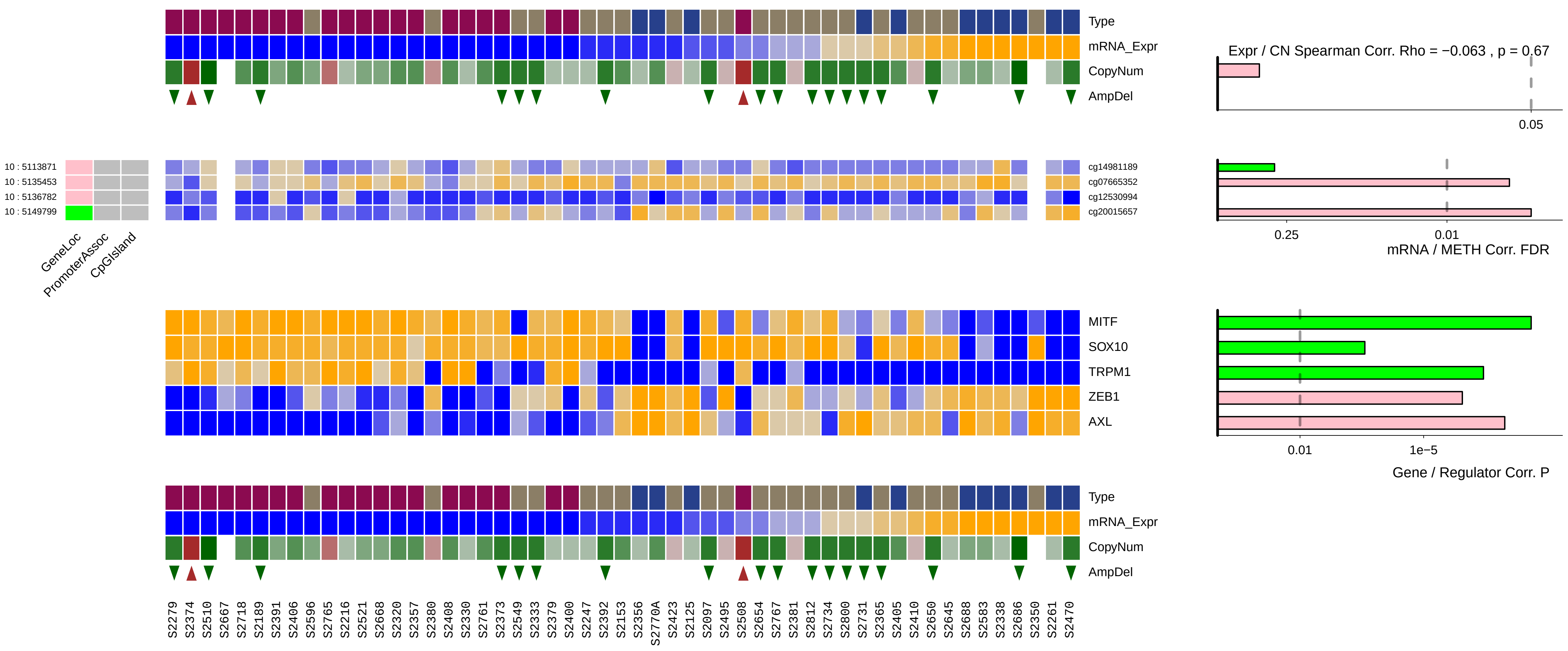

Type
mRNA_Expr
CopyNum
AmpDel
Expr / CN Spearman Corr. Rho = −0.063 , p = 0.67
0.05
10 : 5113871
10 : 5135453
10 : 5136782
10 : 5149799
cg14981189
cg07665352
cg12530994
cg20015657
GeneLoc
PromoterAssoc
CpGIsland
0.25
0.01
mRNA / METH Corr. FDR
MITF
SOX10
TRPM1
ZEB1
AXL
0.01
1e−5
Gene / Regulator Corr. P
S2279 S2374 S2510 S2667 S2718 S2189 S2391 S2406 S2596 S2765 S2216 S2521 S2668 S2320 S2357 S2380 S2408 S2330 S2761 S2373 S2549 S2333 S2379 S2400 S2247 S2392 S2153 S2356 S2770A S2423 S2125 S2097 S2495 S2508 S2654 S2767 S2381 S2812 S2734 S2800 S2731 S2365 S2405 S2410 S2650 S2645 S2688 S2583 S2338 S2686 S2350 S2261 S2470

ARL4C

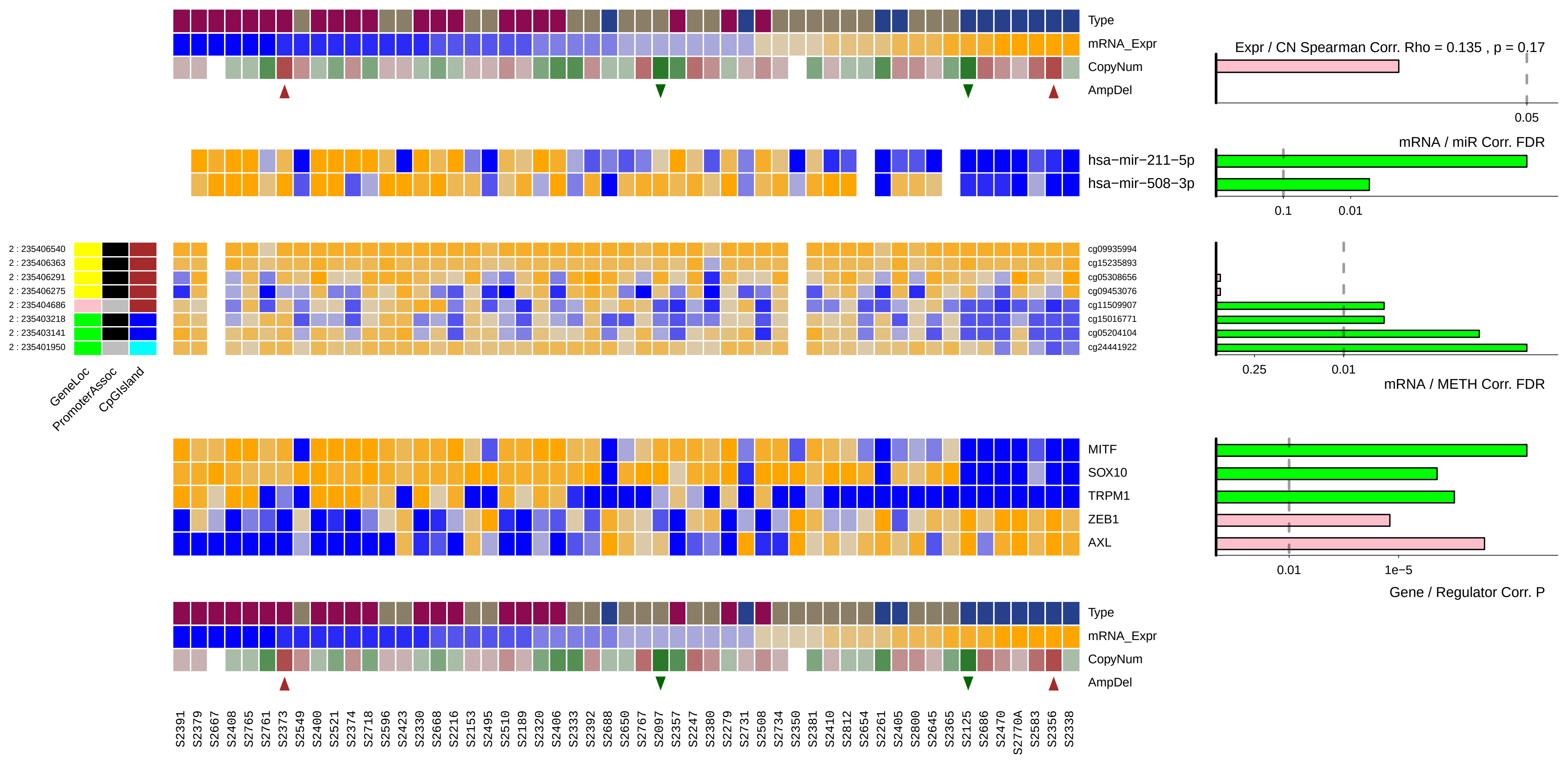
Type
mRNA_Expr
CopyNum
AmpDel
Expr / CN Spearman Corr. Rho = 0.135 , p = 0.17
0.05
hsa−mir−211−5p
hsa−mir−508−3p
mRNA / miR Corr. FDR
0.1
0.01
2 : 235406540
2 : 235406363
2 : 235406291
2 : 235406275
2 : 235404686
2 : 235403218
2 : 235403141
2 : 235401950
GeneLoc
PromoterAssoc
CpGIsland
cg09935994
cg15235893
cg05308656
cg09453076
cg11509907
cg15016771
cg05204104
cg24441922
0.25
0.01
mRNA / METH Corr. FDR
MITF
SOX10
TRPM1
ZEB1
AXL
0.01
1e−5
Gene / Regulator Corr. P
S2391 S2379 S2667 S2408 S2765 S2761 S2373 S2549 S2400 S2521 S2374 S2718 S2596 S2423 S2330 S2668 S2216 S2153 S2495 S2510 S2189 S2320 S2406 S2333 S2392 S2688 S2650 S2767 S2097 S2357 S2247 S2380 S2279 S2731 S2508 S2734 S2350 S2381 S2410 S2812 S2654 S2261 S2405 S2800 S2645 S2365 S2125 S2686 S2470 S2770A S2583 S2356 S2338

HRH1

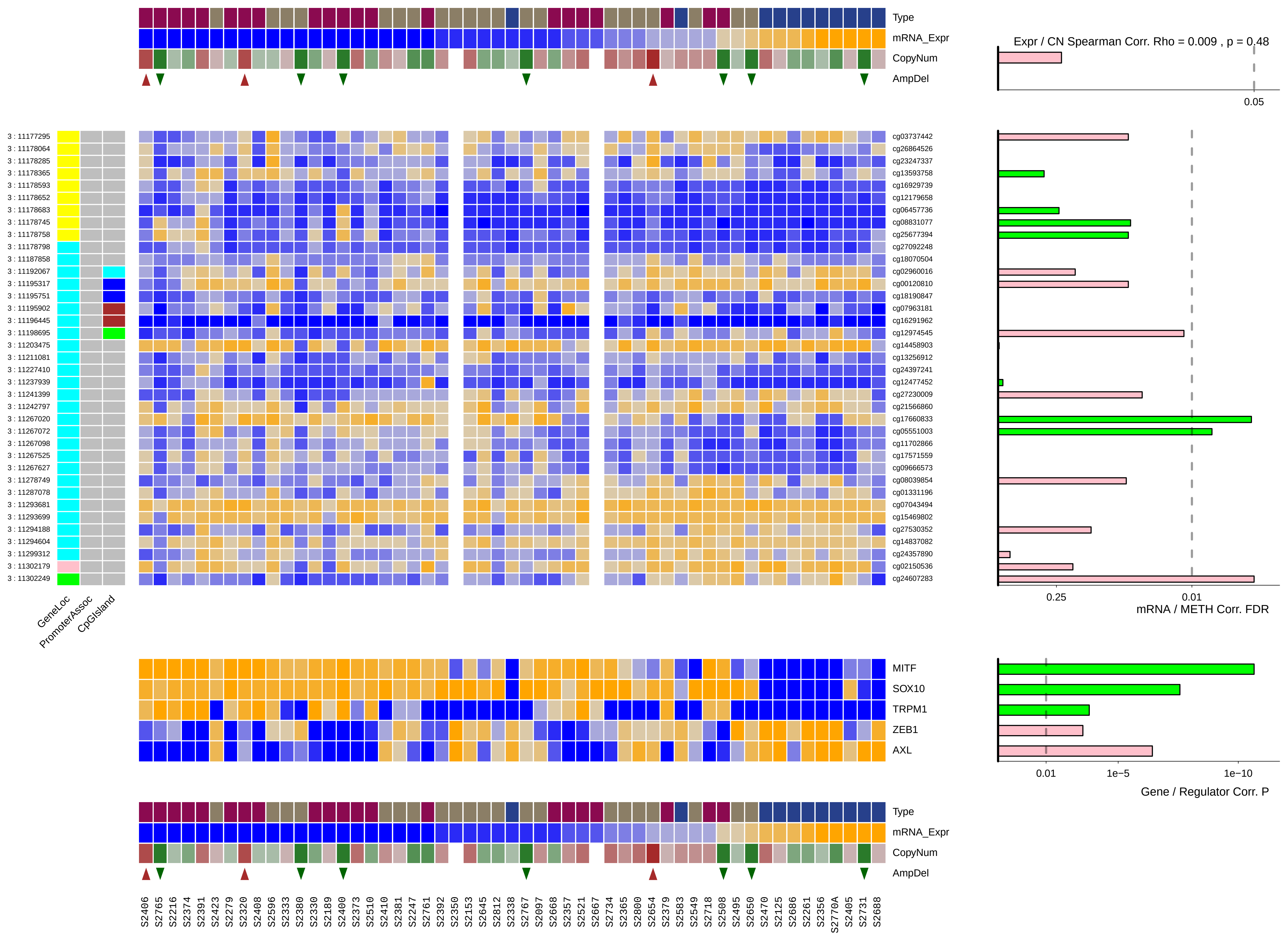

Type
mRNA_Expr
CopyNum
AmpDel
Expr / CN Spearman Corr. Rho = 0.009 , p = 0.48
0.05
3 : 11177295
3 : 11178064
3 : 11178285
3 : 11178365
3 : 11178593
3 : 11178652
3 : 11178683
3 : 11178745
3 : 11178758
3 : 11178798
3 : 11187858
3 : 11192067
3 : 11195317
3 : 11195751
3 : 11195902
3 : 11196445
3 : 11198695
3 : 11203475
3 : 11211081
3 : 11227410
3 : 11237939
3 : 11241399
3 : 11242797
3 : 11267020
3 : 11267072
3 : 11267098
3 : 11267525
3 : 11267627
3 : 11278749
3 : 11287078
3 : 11293681
3 : 11293699
3 : 11294188
3 : 11294604
3 : 11299312
3 : 11302179
3 : 11302249
GeneLoc
PromoterAssoc
CpGIsland
cg03737442
cg26864526
cg23247337
cg13593758
cg16929739
cg12179658
cg06457736
cg08831077
cg25677394
cg27092248
cg18070504
cg02960016
cg00120810
cg18190847
cg07963181
cg16291962
cg12974545
cg14458903
cg13256912
cg24397241
cg12477452
cg27230009
cg21566860
cg17660833
cg05551003
cg11702866
cg17571559
cg09666573
cg08039854
cg01331196
cg07043494
cg15469802
cg27530352
cg14837082
cg24357890
cg02150536
cg24607283
0.25
0.01
mRNA / METH Corr. FDR
MITF
SOX10
TRPM1
ZEB1
AXL
0.01
1e−5
1e−10
Gene / Regulator Corr. P
Type
mRNA_Expr
CopyNum
AmpDel
S2406 S2765 S2216 S2374 S2391 S2423 S2279 S2320 S2408 S2596 S2333 S2380 S2330 S2189 S2400 S2373 S2510 S2410 S2381 S2247 S2761 S2392 S2350 S2153 S2645 S2812 S2338 S2767 S2097 S2668 S2357 S2521 S2667 S2734 S2365 S2800 S2654 S2379 S2583 S2549 S2718 S2508 S2495 S2650 S2470 S2125 S2686 S2261 S2356 S2770A S2405 S2731 S2688

CLU

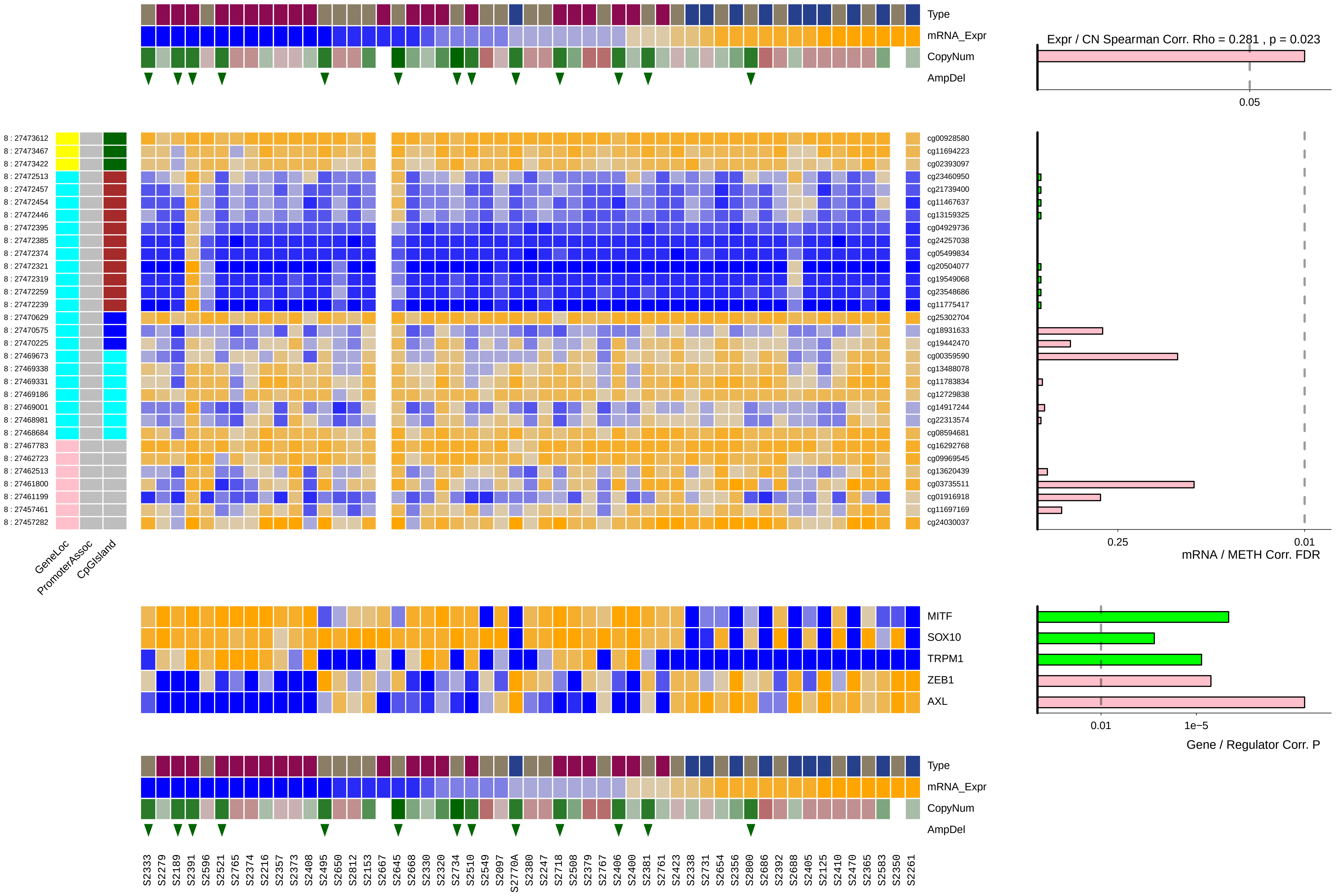
Type
mRNA_Expr
CopyNum
AmpDel
Expr / CN Spearman Corr. Rho = 0.281 , p = 0.023
0.05
8 : 27473612
8 : 27473467
8 : 27473422
8 : 27472513
8 : 27472457
8 : 27472454
8 : 27472446
8 : 27472395
8 : 27472385
8 : 27472374
8 : 27472321
8 : 27472319
8 : 27472259
8 : 27472239
8 : 27470629
8 : 27470575
8 : 27470225
8 : 27469673
8 : 27469338
8 : 27469331
8 : 27469186
8 : 27469001
8 : 27468981
8 : 27468684
8 : 27467783
8 : 27462723
8 : 27462513
8 : 27461800
8 : 27461199
8 : 27457461
8 : 27457282
cg00928580
cg11694223
cg02393097
cg23460950
cg21739400
cg11467637
cg13159325
cg04929736
cg24257038
cg05499834
cg20504077
cg19549068
cg23548686
cg11775417
cg25302704
cg18931633
cg19442470
cg00359590
cg13488078
cg11783834
cg12729838
cg14917244
cg22313574
cg08594681
cg16292768
cg09969545
cg13620439
cg03735511
cg01916918
cg11697169
cg24030037
GeneLoc
PromoterAssoc
CpGIsland
0.25
0.01
mRNA / METH Corr. FDR
MITF
SOX10
TRPM1
ZEB1
AXL
0.01
1e−5
Gene / Regulator Corr. P
Type
mRNA_Expr
CopyNum
AmpDel
S2333 S2279 S2189 S2391 S2596 S2521 S2765 S2374 S2216 S2357 S2373 S2408 S2495 S2650 S2812 S2153 S2667 S2645 S2668 S2330 S2320 S2734 S2510 S2549 S2097 S2770A S2380 S2247 S2718 S2508 S2379 S2767 S2406 S2400 S2381 S2761 S2423 S2338 S2731 S2654 S2356 S2800 S2686 S2392 S2688 S2405 S2125 S2410 S2470 S2365 S2583 S2350 S2261

ADAMTS6

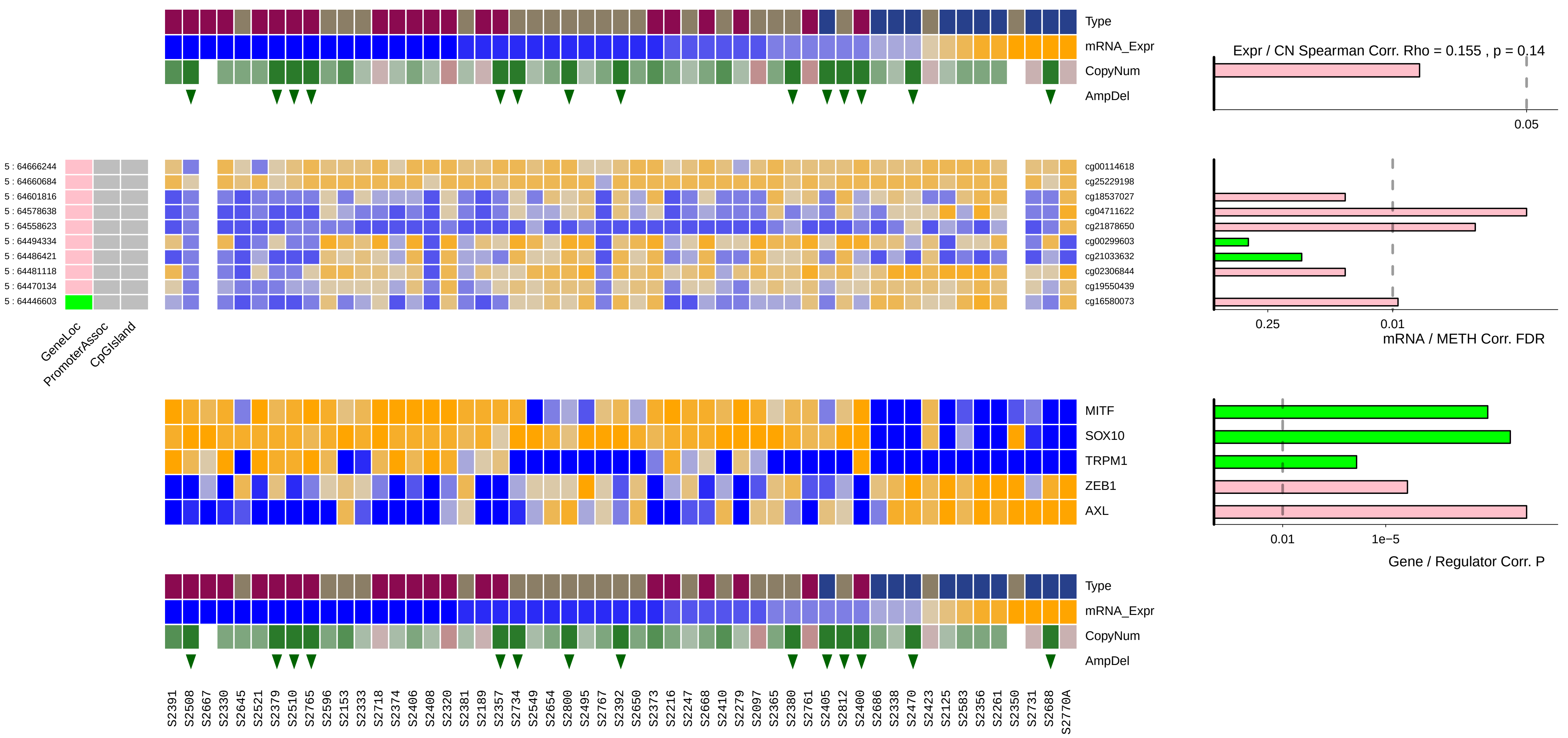
Type
mRNA_Expr
CopyNum
AmpDel
Expr / CN Spearman Corr. Rho = 0.155 , p = 0.14
0.05
5 : 64666244
5 : 64660684
5 : 64601816
5 : 64578638
5 : 64558623
5 : 64494334
5 : 64486421
5 : 64481118
5 : 64470134
5 : 64446603
GeneLoc
PromoterAssoc
CpGIsland
cg00114618
cg25229198
cg18537027
cg04711622
cg21878650
cg00299603
cg21033632
cg02306844
cg19550439
cg16580073
0.25
0.01
mRNA / METH Corr. FDR
MITF
SOX10
TRPM1
ZEB1
AXL
0.01
1e−5
Gene / Regulator Corr. P
S2391 S2508 S2667 S2330 S2645 S2521 S2379 S2510 S2765 S2596 S2153 S2333 S2718 S2374 S2406 S2408 S2320 S2381 S2189 S2357 S2734 S2549 S2654 S2800 S2495 S2767 S2392 S2650 S2373 S2216 S2247 S2668 S2410 S2279 S2097 S2365 S2380 S2761 S2405 S2812 S2400 S2686 S2338 S2470 S2423 S2125 S2583 S2356 S2261 S2350 S2731 S2688 S2770A

ABCC3

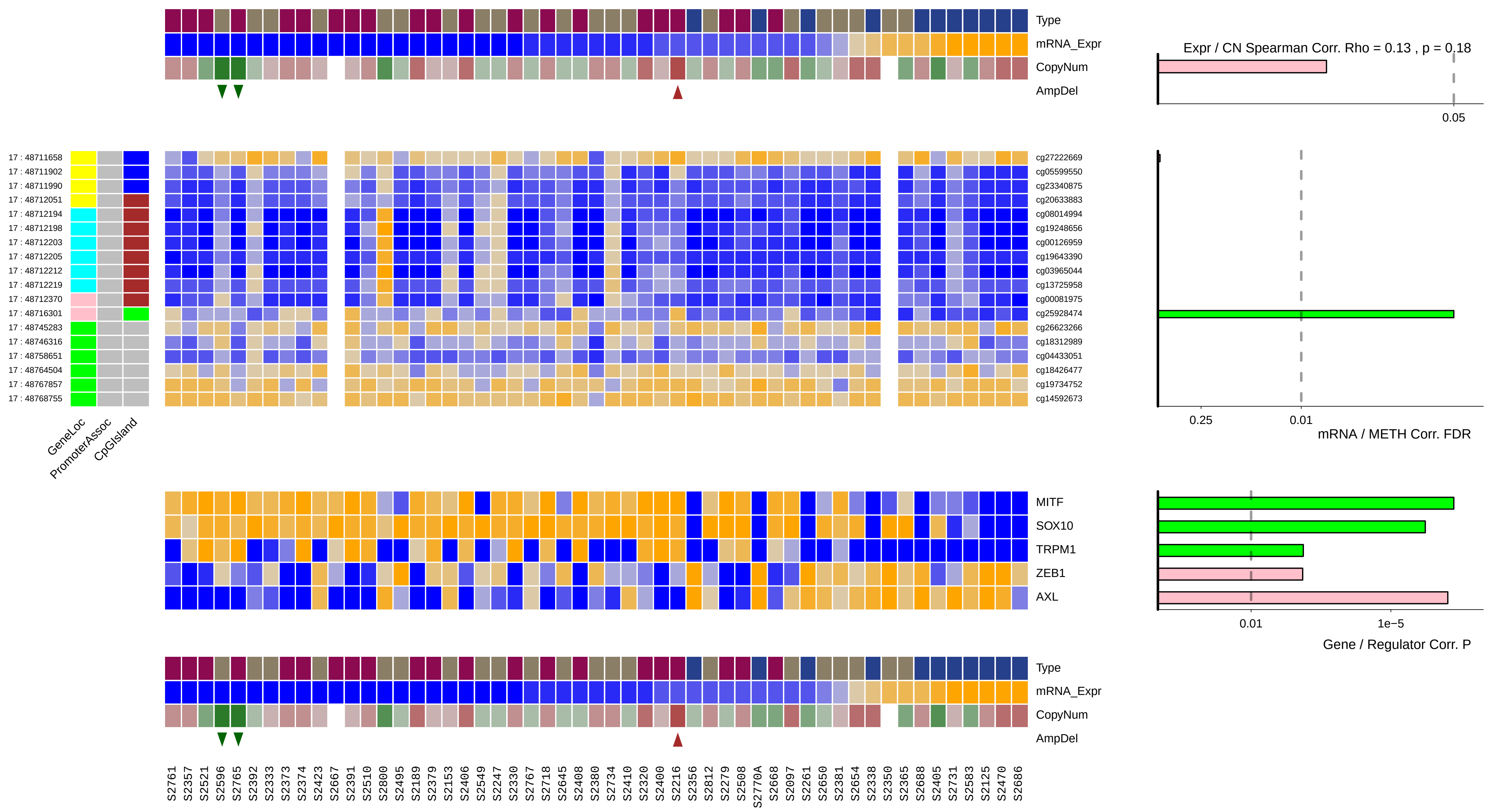
Type
mRNA_Expr
CopyNum
AmpDel
Expr / CN Spearman Corr. Rho = 0.13 , p = 0.18
0.05
17 : 48711658
17 : 48711902
17 : 48711990
17 : 48712051
17 : 48712194
17 : 48712198
17 : 48712203
17 : 48712205
17 : 48712212
17 : 48712219
17 : 48712370
17 : 48716301
17 : 48745283
17 : 48746316
17 : 48758651
17 : 48764504
17 : 48767857
17 : 48768755
cg27222669
cg05599550
cg23340875
cg20633883
cg08014994
cg19248656
cg00126959
cg19643390
cg03965044
cg13725958
cg00081975
cg25928474
cg26623266
cg18312989
cg04433051
cg18426477
cg19734752
cg14592673
GeneLoc
PromoterAssoc
CpGIsland
0.25
0.01
mRNA / METH Corr. FDR
MITF
SOX10
TRPM1
ZEB1
AXL
0.01
1e−5
Gene / Regulator Corr. P
Type
mRNA_Expr
CopyNum
AmpDel
S2761
S2357
S2521
S2596
S2765
S2392
S2333
S2373
S2374
S2423
S2667
S2391
S2510
S2800
S2495
S2189
S2379
S2153
S2406
S2549
S2247
S2330
S2767
S2718
S2645
S2408
S2380
S2734
S2410
S2320
S2400
S2216
S2356
S2812
S2279
S2508
S2770A
S2668
S2097
S2261
S2650
S2381
S2654
S2338
S2350
S2365
S2688
S2405
S2731
S2583
S2125
S2470
S2686

CSF1

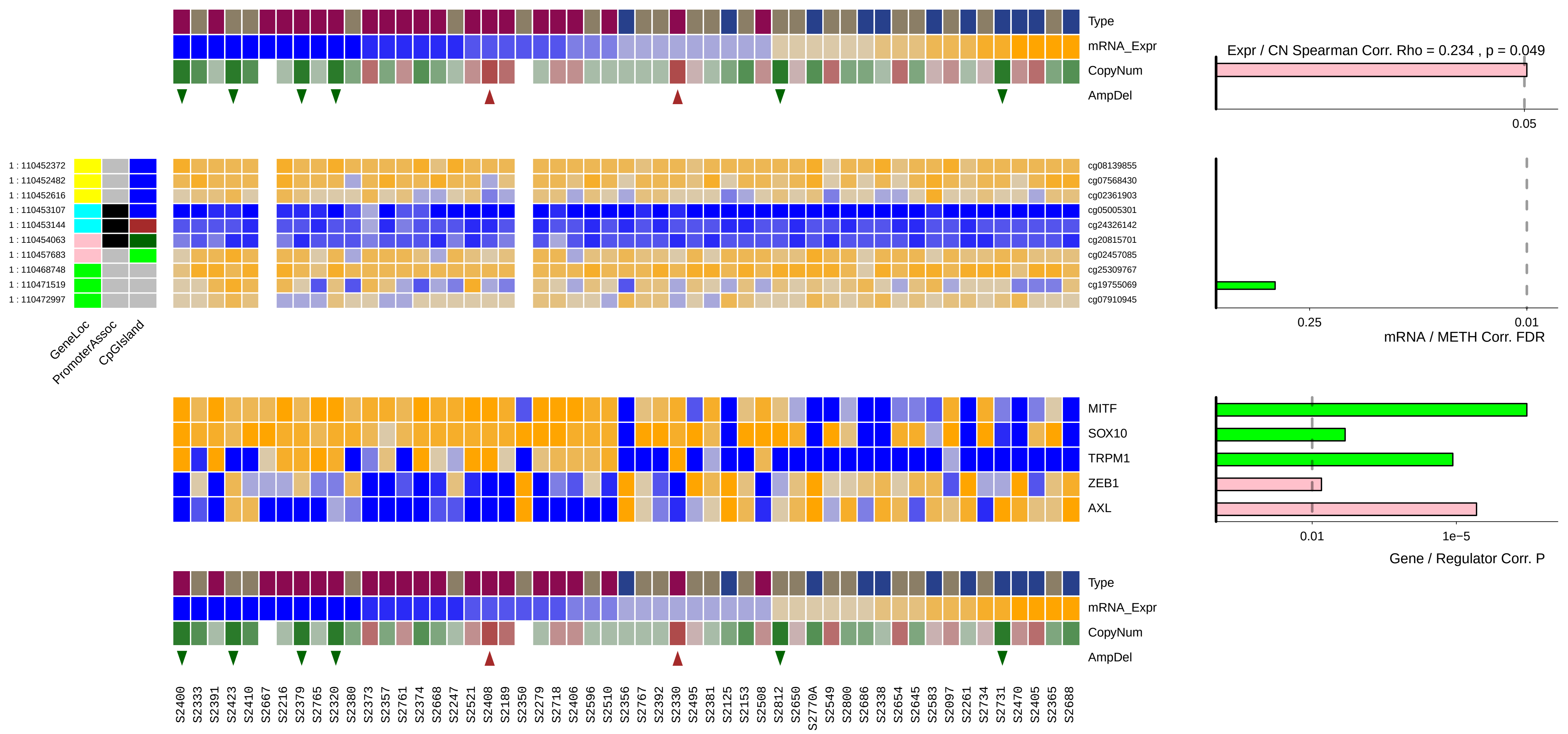
Type
mRNA_Expr
CopyNum
AmpDel
Expr / CN Spearman Corr. Rho = 0.234 , p = 0.049
0.05
1 : 110452372
1 : 110452482
1 : 110452616
1 : 110453107
1 : 110453144
1 : 110454063
1 : 110457683
1 : 110468748
1 : 110471519
1 : 110472997
GeneLoc
PromoterAssoc
CpGIsland
cg08139855
cg07568430
cg02361903
cg05005301
cg24326142
cg20815701
cg02457085
cg25309767
cg19755069
cg07910945
0.25
0.01
mRNA / METH Corr. FDR
MITF
SOX10
TRPM1
ZEB1
AXL
0.01
1e−5
Gene / Regulator Corr. P
S2400 S2333 S2391 S2423 S2410 S2667 S2216 S2379 S2765 S2320 S2380 S2373 S2357 S2761 S2374 S2668 S2247 S2521 S2408 S2189 S2350 S2279 S2718 S2406 S2596 S2510 S2356 S2767 S2392 S2330 S2495 S2381 S2125 S2153 S2508 S2812 S2650 S2770A S2549 S2800 S2686 S2338 S2654 S2645 S2583 S2097 S2261 S2734 S2731 S2470 S2405 S2365 S2688

HEG1

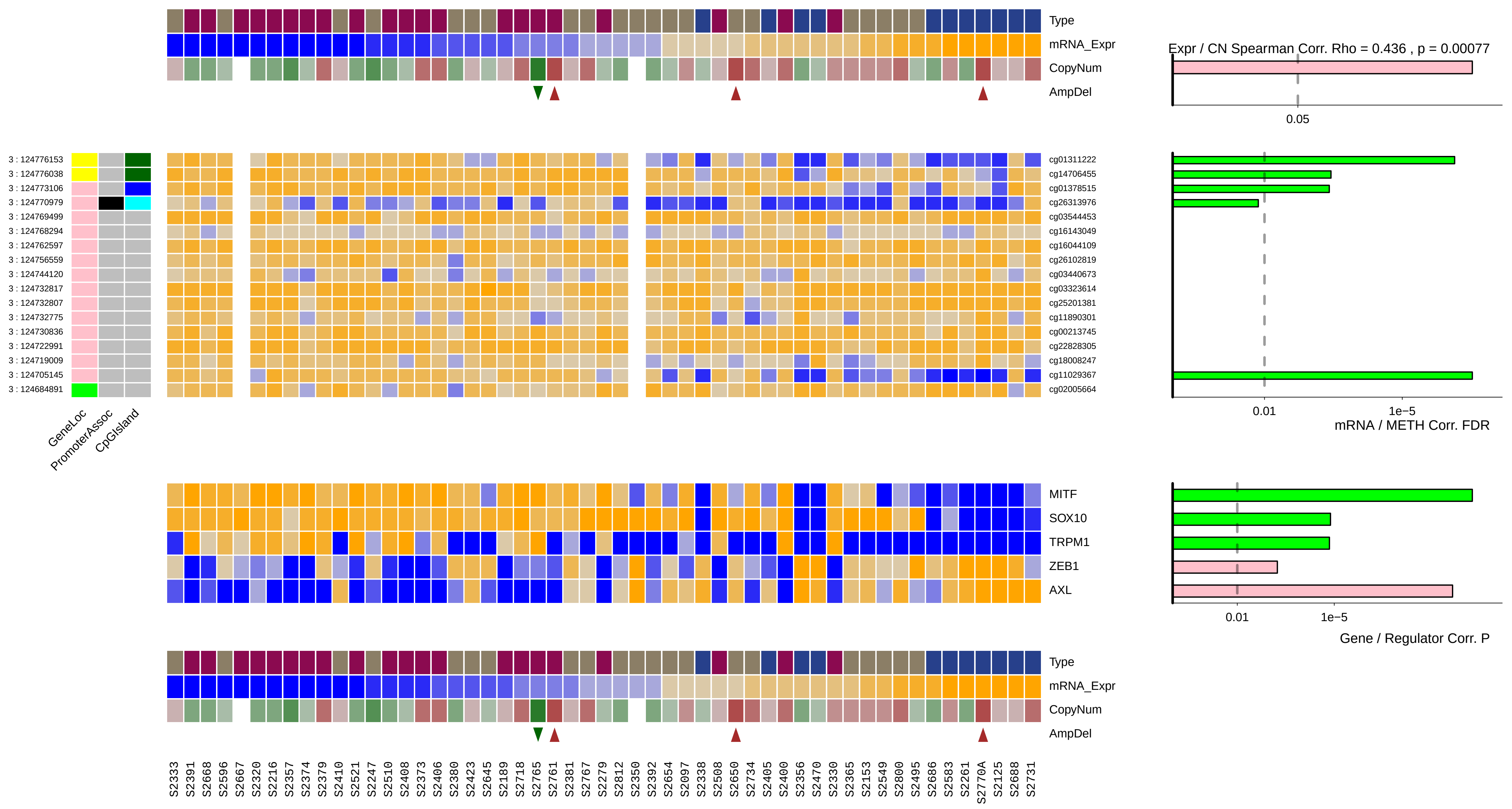
Type
mRNA_Expr
CopyNum
AmpDel
Expr / CN Spearman Corr. Rho = 0.436 , p = 0.00077
0.05
3 : 124776153
3 : 124776038
3 : 124773106
3 : 124770979
3 : 124769499
3 : 124768294
3 : 124762597
3 : 124756559
3 : 124744120
3 : 124732817
3 : 124732807
3 : 124732775
3 : 124730836
3 : 124722991
3 : 124719009
3 : 124705145
3 : 124684891
cg01311222
cg14706455
cg01378515
cg26313976
cg03544453
cg16143049
cg16044109
cg26102819
cg03440673
cg03323614
cg25201381
cg11890301
cg00213745
cg22828305
cg18008247
cg11029367
cg02005664
GeneLoc
PromoterAssoc
CpGIsland
0.01
1e−5
mRNA / METH Corr. FDR
MITF
SOX10
TRPM1
ZEB1
AXL
0.01
1e−5
Gene / Regulator Corr. P
Type
mRNA_Expr
CopyNum
AmpDel
S2333 S2391 S2668 S2596 S2667 S2320 S2216 S2357 S2374 S2379 S2410 S2521 S2247 S2510 S2408 S2373 S2406 S2380 S2423 S2645 S2189 S2718 S2765 S2761 S2381 S2767 S2279 S2812 S2350 S2392 S2654 S2097 S2338 S2508 S2650 S2734 S2405 S2400 S2356 S2470 S2330 S2365 S2153 S2549 S2800 S2495 S2686 S2583 S2261 S2770A S2125 S2688 S2731

SPEG

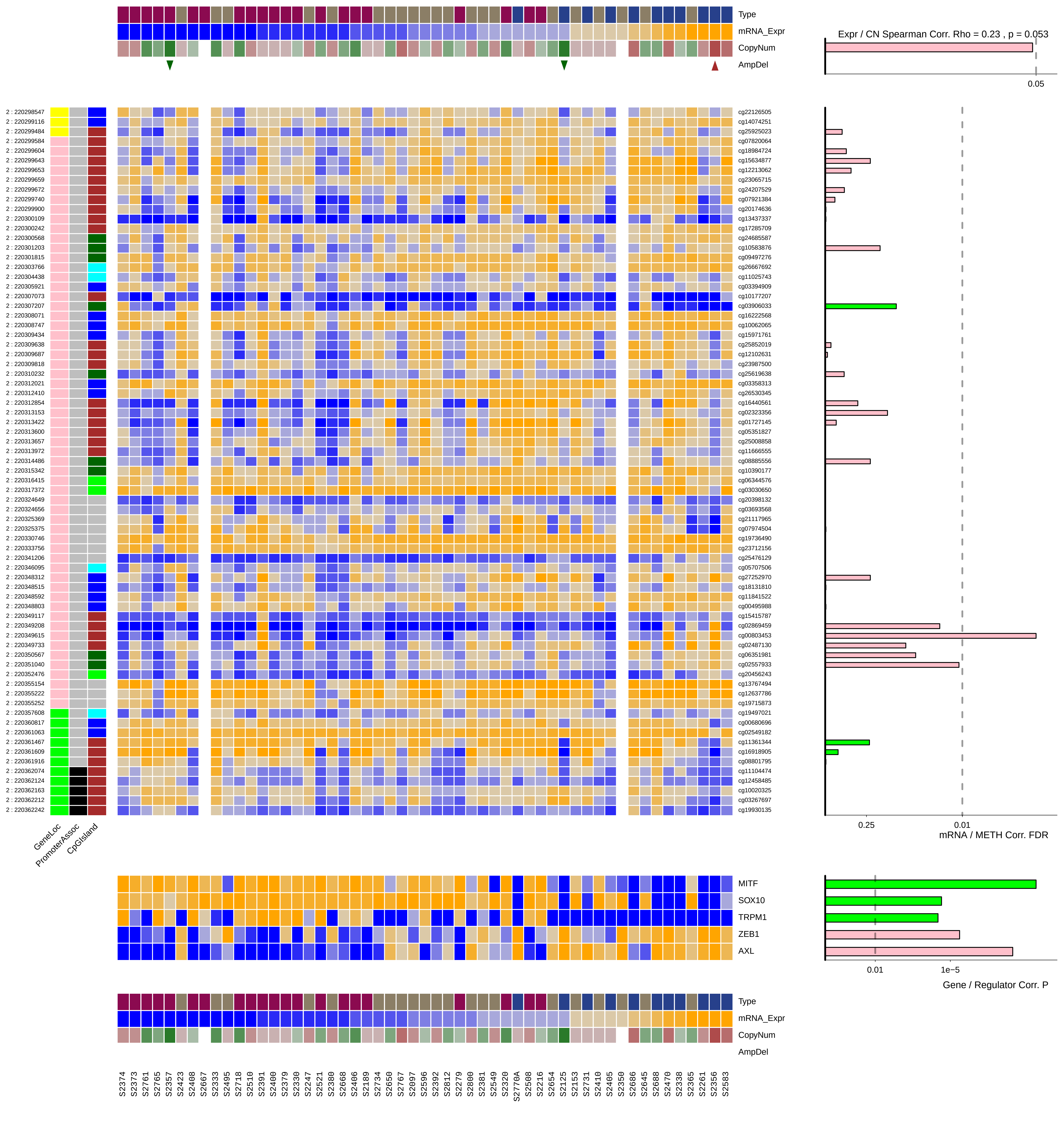

Type
mRNA_Expr
CopyNum
AmpDel
Expr / CN Spearman Corr. Rho = 0.23 , p = 0.053
0.05
0.25
0.01
mRNA / METH Corr. FDR
GeneLoc
PromoterAssoc
CpGIsland
MITF
SOX10
TRPM1
ZEB1
AXL
0.01
1e−5
Gene / Regulator Corr. P

FBN1

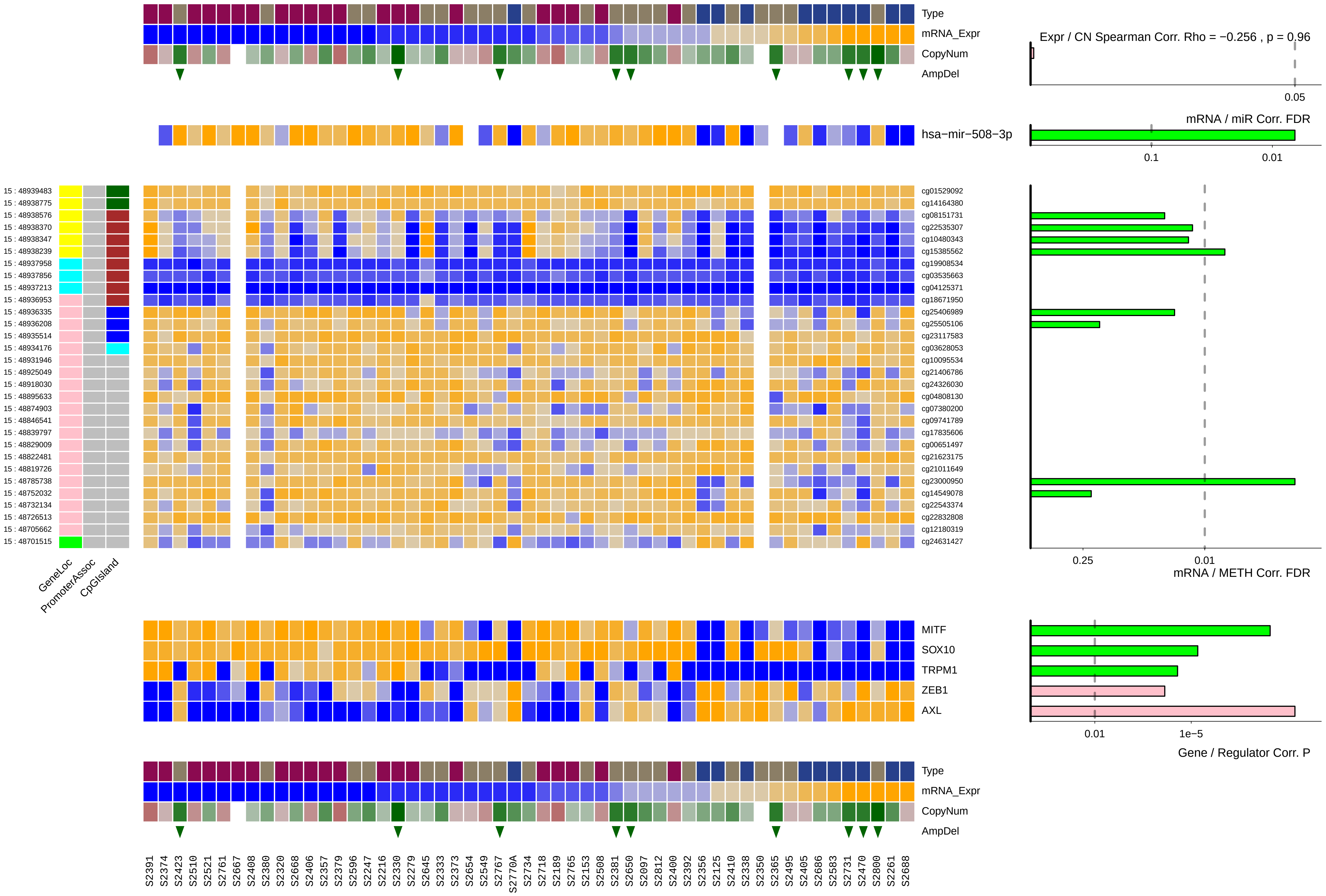

Type
mRNA_Expr
CopyNum
AmpDel
Expr / CN Spearman Corr. Rho = −0.256 , p = 0.96
0.05
hsa−mir−508−3p
mRNA / miR Corr. FDR
0.1
0.01
15 : 48939483
15 : 48938775
15 : 48938576
15 : 48938370
15 : 48938347
15 : 48938239
15 : 48937958
15 : 48937856
15 : 48937213
15 : 48936953
15 : 48936335
15 : 48936208
15 : 48935514
15 : 48934176
15 : 48931946
15 : 48925049
15 : 48918030
15 : 48895633
15 : 48874903
15 : 48846541
15 : 48839797
15 : 48829009
15 : 48822481
15 : 48819726
15 : 48785738
15 : 48752032
15 : 48732134
15 : 48726513
15 : 48705662
15 : 48701515
cg01529092
cg14164380
cg08151731
cg22535307
cg10480343
cg15385562
cg19908534
cg03535663
cg04125371
cg18671950
cg25406989
cg25505106
cg23117583
cg03628053
cg10095534
cg21406786
cg24326030
cg04808130
cg07380200
cg09741789
cg17835606
cg00651497
cg21623175
cg21011649
cg23000950
cg14549078
cg22543374
cg22832808
cg12180319
cg24631427
GeneLoc
PromoterAssoc
CpGIsland
0.25
0.01
mRNA / METH Corr. FDR
MITF
SOX10
TRPM1
ZEB1
AXL
0.01
1e−5
Gene / Regulator Corr. P
Type
mRNA_Expr
CopyNum
AmpDel
S2391 S2374 S2423 S2510 S2521 S2761 S2667 S2408 S2380 S2320 S2668 S2406 S2357 S2379 S2596 S2247 S2216 S2330 S2279 S2645 S2333 S2373 S2654 S2549 S2767 S2770A S2734 S2718 S2189 S2765 S2153 S2508 S2381 S2650 S2097 S2812 S2400 S2392 S2356 S2125 S2410 S2338 S2350 S2365 S2495 S2405 S2686 S2583 S2731 S2470 S2800 S2261 S2688

PXDN

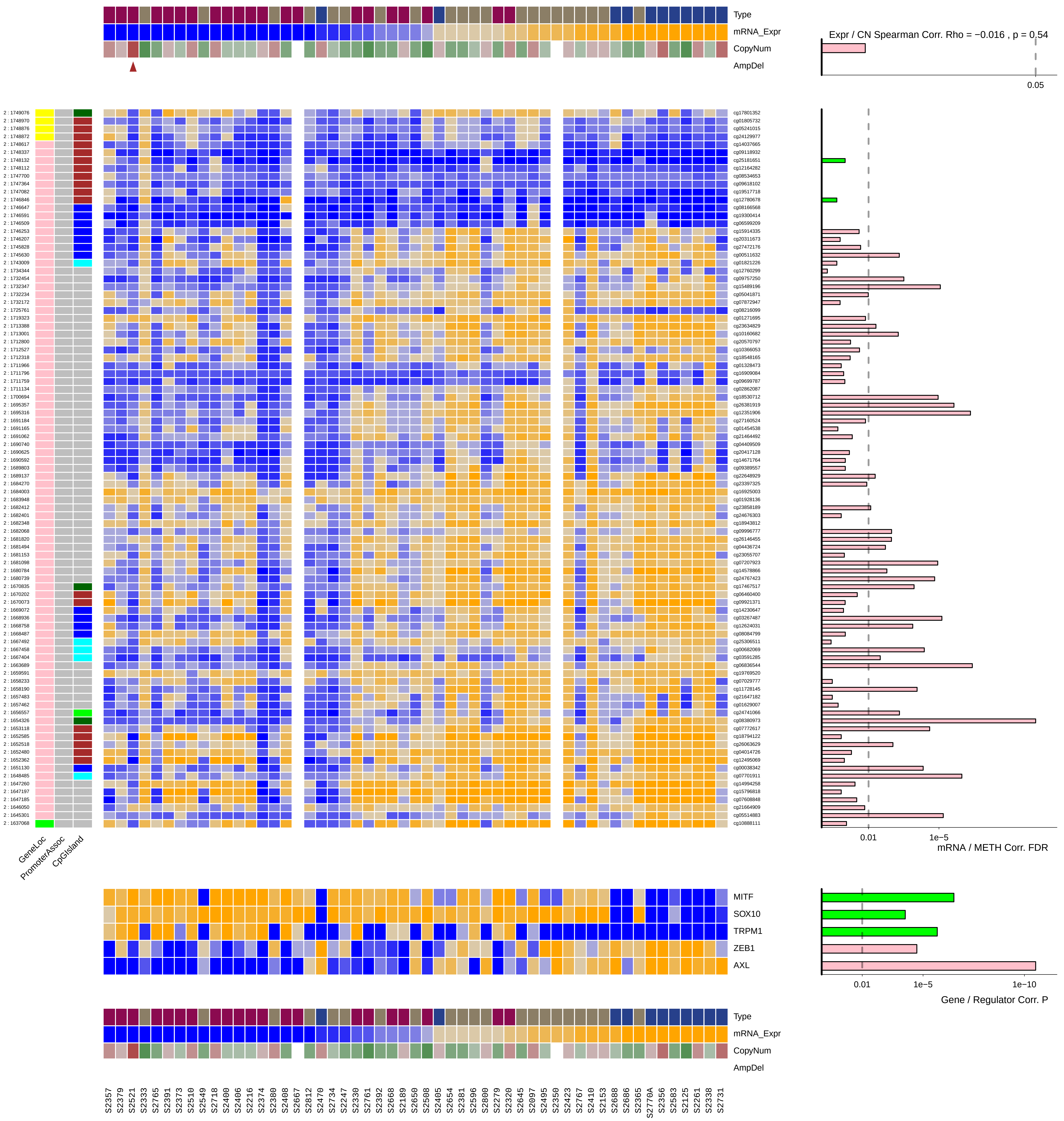
Type
mRNA_Expr
CopyNum
AmpDel
Expr / CN Spearman Corr. Rho = −0.016 , p = 0.54
0.05
GeneLoc
PromoterAssoc
CpGIsland
0.01
1e−5
mRNA / METH Corr. FDR
MITF
SOX10
TRPM1
ZEB1
AXL
0.01
1e−5
1e−10
Gene / Regulator Corr. P
Type
mRNA_Expr
CopyNum
AmpDel
S2357 S2379 S2521 S2333 S2765 S2391 S2373 S2510 S2549 S2718 S2400 S2406 S2216 S2374 S2380 S2408 S2667 S2812 S2470 S2734 S2247 S2330 S2761 S2392 S2668 S2189 S2650 S2508 S2405 S2654 S2381 S2596 S2800 S2279 S2320 S2645 S2097 S2495 S2350 S2423 S2767 S2410 S2153 S2688 S2686 S2365 S2770A S2356 S2583 S2125 S2261 S2338 S2731

ALCAM

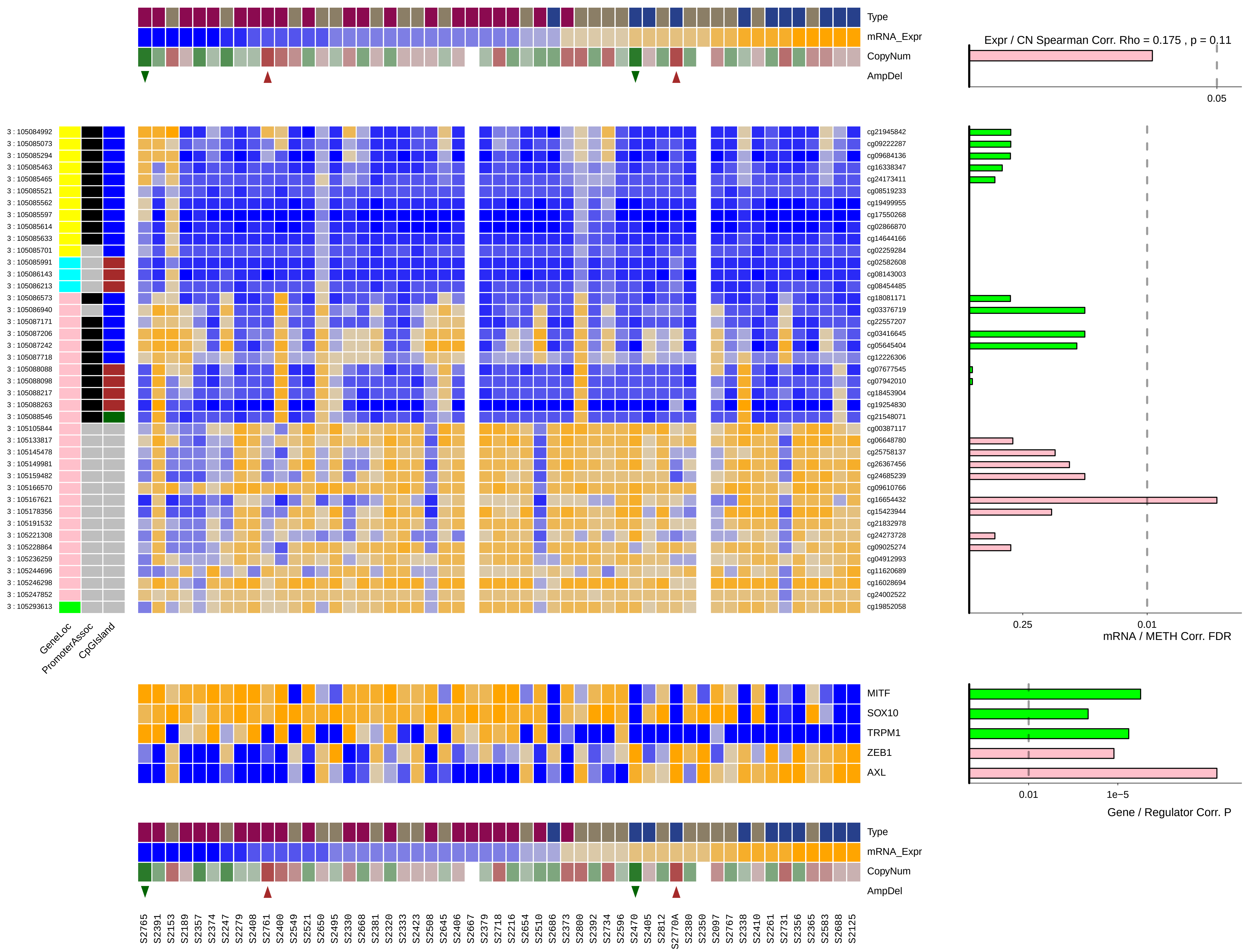

Type
mRNA_Expr
CopyNum
AmpDel

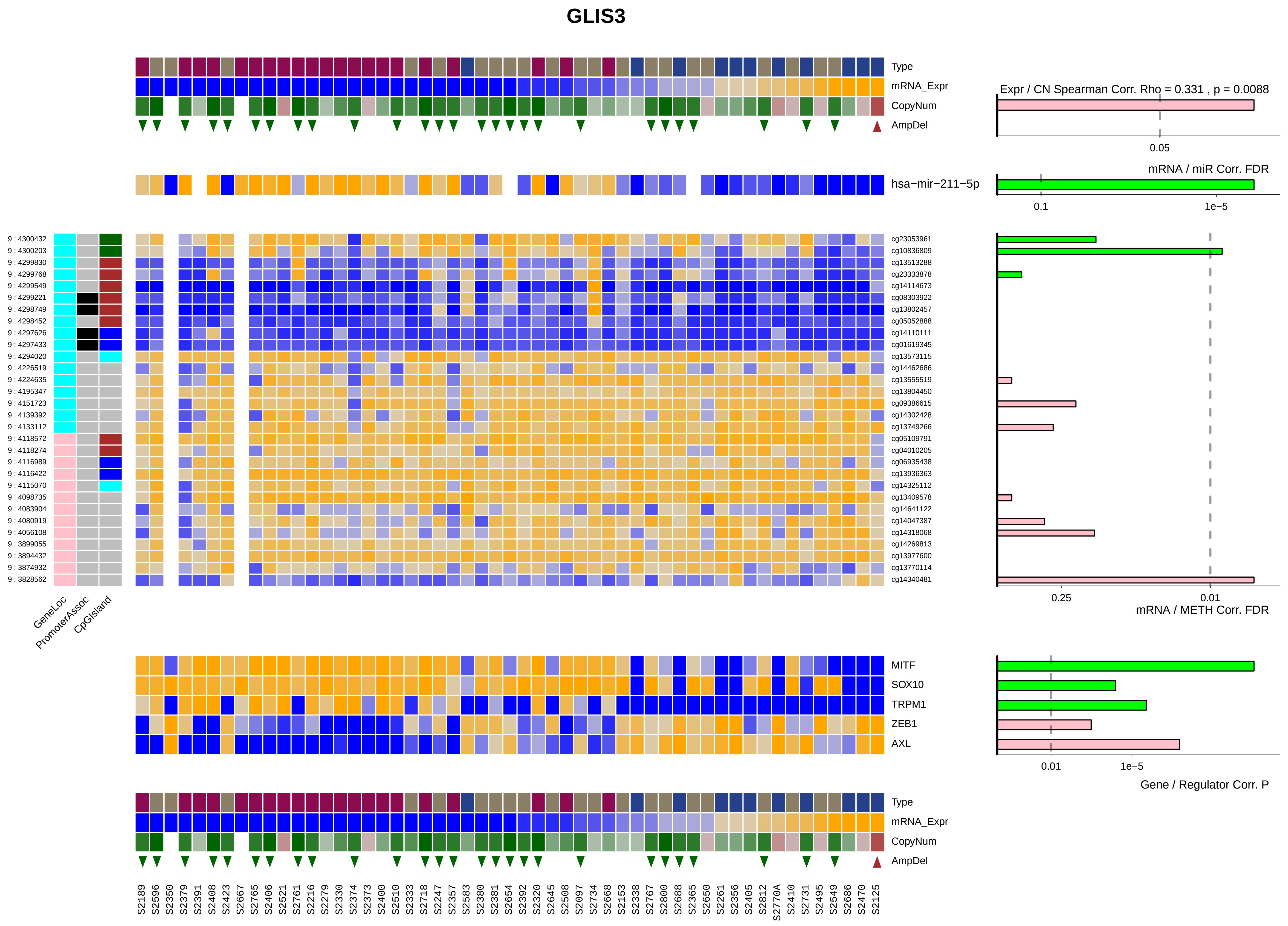
GLIS3
Type
mRNA_Expr
CopyNum
AmpDel
Expr / CN Spearman Corr. Rho = 0.331 , p = 0.0088
0.05
hsa−mir−211−5p
mRNA / miR Corr. FDR
0.1
1e−5
GeneLoc
PromoterAssoc
CpGIsland
0.25
0.01
mRNA / METH Corr. FDR
MITF
SOX10
TRPM1
ZEB1
AXL
0.01
1e−5
Gene / Regulator Corr. P

SPOCK1

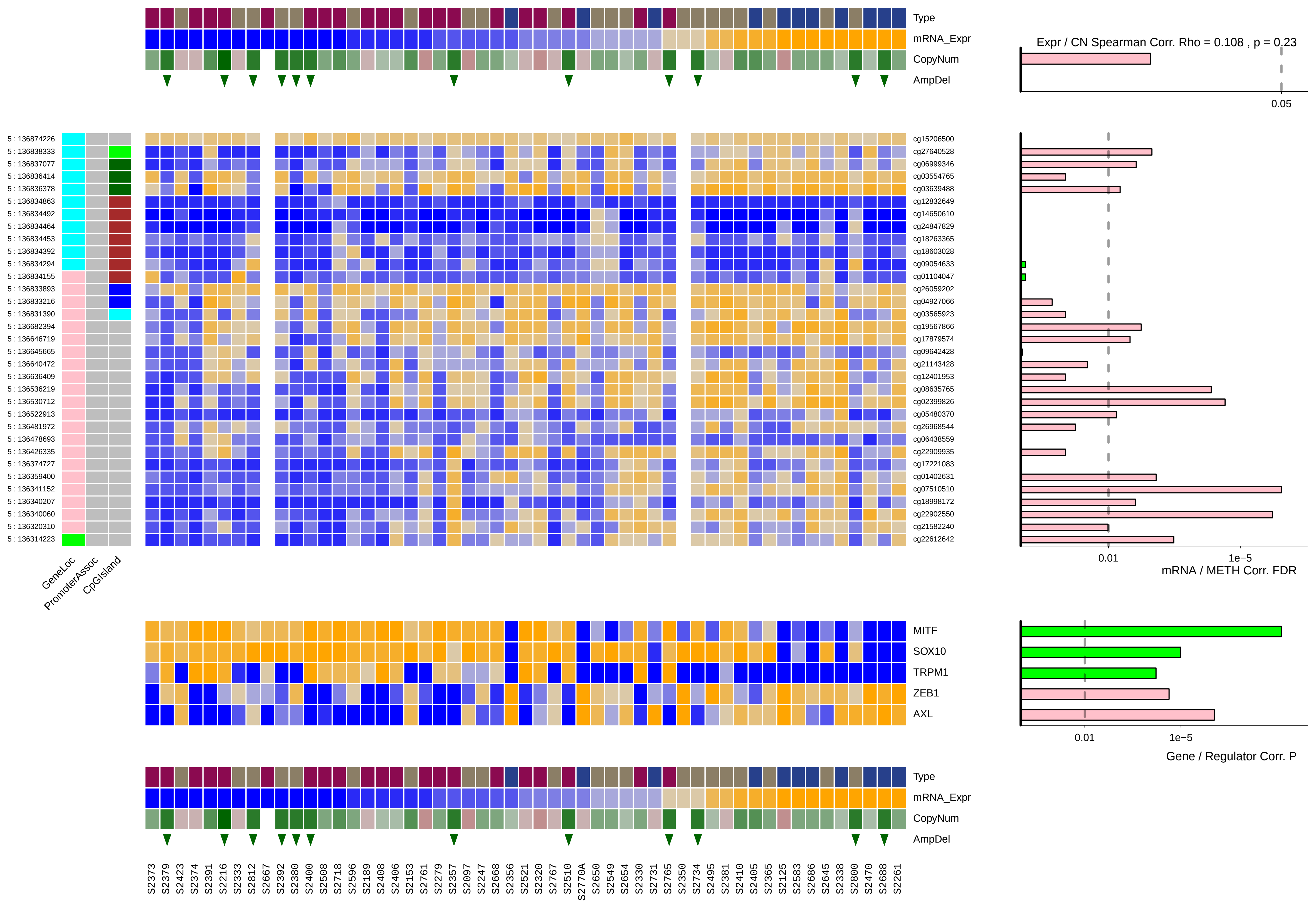
Type
mRNA_Expr
CopyNum
AmpDel
Expr / CN Spearman Corr. Rho = 0.108 , p = 0.23
0.05
mRNA / METH Corr. FDR
0.01
1e−5
GeneLoc
PromoterAssoc
CpGIsland
MITF
SOX10
TRPM1
ZEB1
AXL
Gene / Regulator Corr. P

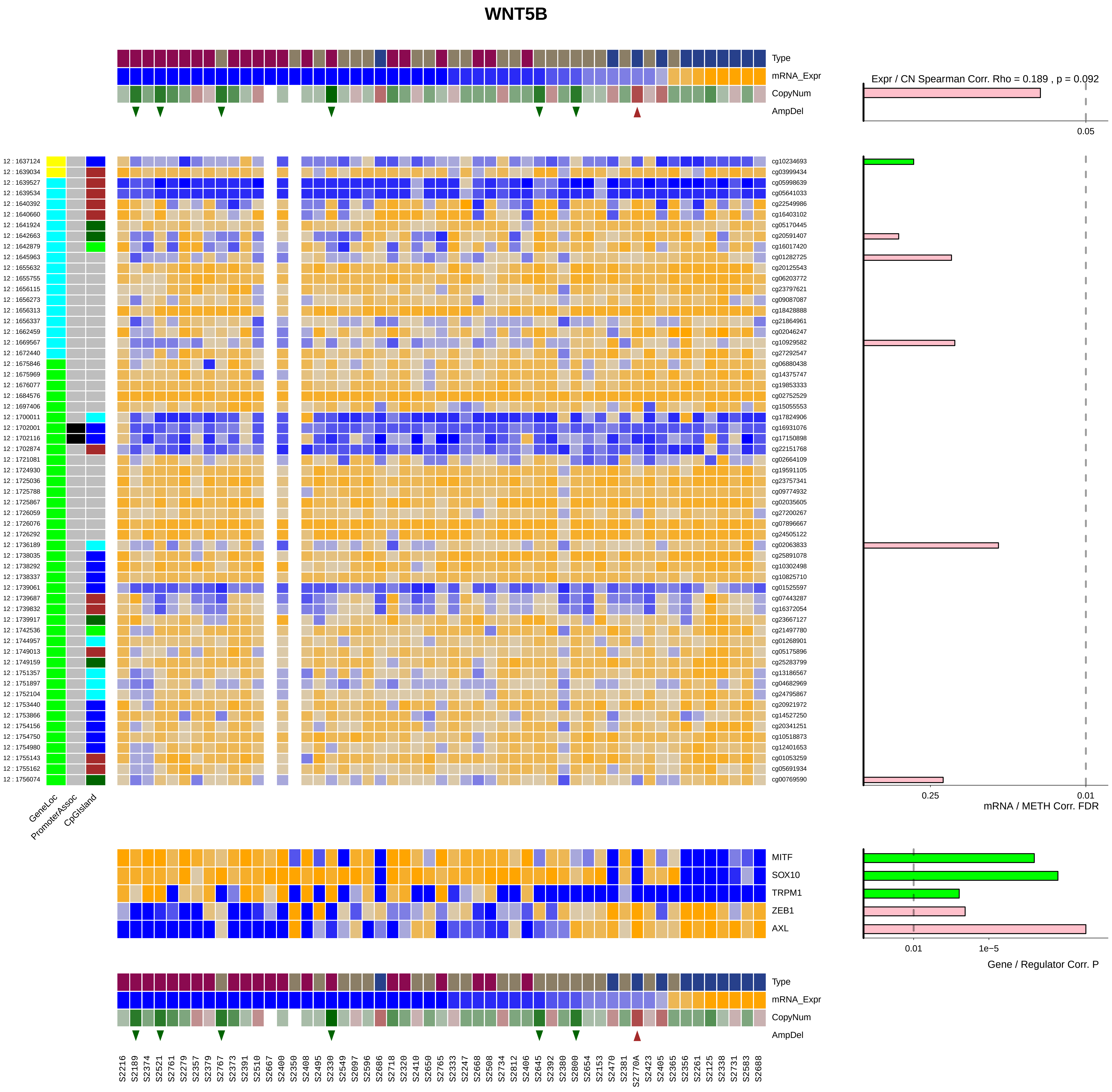

WNT5B
Type
mRNA_Expr
CopyNum
AmpDel
Expr / CN Spearman Corr. Rho = 0.189 , p = 0.092
0.05
12 : 1637124
12 : 1639034
12 : 1639527
12 : 1639534
12 : 1640392
12 : 1640660
12 : 1641924
12 : 1642663
12 : 1642879
12 : 1645963
12 : 1655632
12 : 1655755
12 : 1656115
12 : 1656273
12 : 1656313
12 : 1656337
12 : 1662459
12 : 1669567
12 : 1672440
12 : 1675846
12 : 1675969
12 : 1676077
12 : 1684576
12 : 1697406
12 : 1700011
12 : 1702001
12 : 1702116
12 : 1702874
12 : 1721081
12 : 1724930
12 : 1725036
12 : 1725788
12 : 1725867
12 : 1726059
12 : 1726076
12 : 1726292
12 : 1736189
12 : 1738035
12 : 1738292
12 : 1738337
12 : 1739061
12 : 1739687
12 : 1739832
12 : 1739917
12 : 1742536
12 : 1744957
12 : 1749013
12 : 1749159
12 : 1751357
12 : 1751897
12 : 1752104
12 : 1753440
12 : 1753866
12 : 1754156
12 : 1754750
12 : 1754980
12 : 1755143
12 : 1755162
12 : 1756074
cg10234693
cg03999434
cg05998639
cg05641033
cg22549986
cg16403102
cg05170445
cg20591407
cg16017420
cg01282725
cg20125543
cg06203772
cg23797621
cg09087087
cg18428888
cg21864961
cg02046247
cg10929582
cg27292547
cg06880438
cg14375747
cg19853333
cg02752529
cg15055553
cg17824906
cg16931076
cg17150898
cg22151768
cg02664109
cg19591105
cg23757341
cg09774932
cg02035605
cg27200267
cg07896667
cg24505122
cg02063833
cg25891078
cg10302498
cg10825710
cg01525597
cg07443287
cg16372054
cg23667127
cg21497780
cg01268901
cg05175896
cg25283799
cg13186567
cg04682969
cg24795867
cg20921972
cg14527250
cg20341251
cg10518873
cg12401653
cg01053259
cg05691934
cg00769590
0.25
0.01
mRNA / METH Corr. FDR
GeneLoc
PromoterAssoc
CpGIsland
MITF
SOX10
TRPM1
ZEB1
AXL
0.01
1e−5
Gene / Regulator Corr. P
Type
mRNA_Expr
CopyNum
AmpDel
S2216
S2189
S2374
S2521
S2761
S2279
S2357
S2379
S2767
S2373
S2391
S2510
S2667
S2400
S2350
S2408
S2495
S2330
S2549
S2097
S2596
S2686
S2718
S2320
S2410
S2650
S2765
S2333
S2247
S2668
S2508
S2734
S2812
S2406
S2645
S2392
S2380
S2800
S2654
S2153
S2470
S2381
S2770A
S2423
S2405
S2365
S2356
S2261
S2125
S2338
S2731
S2583
S2688

SLIT2

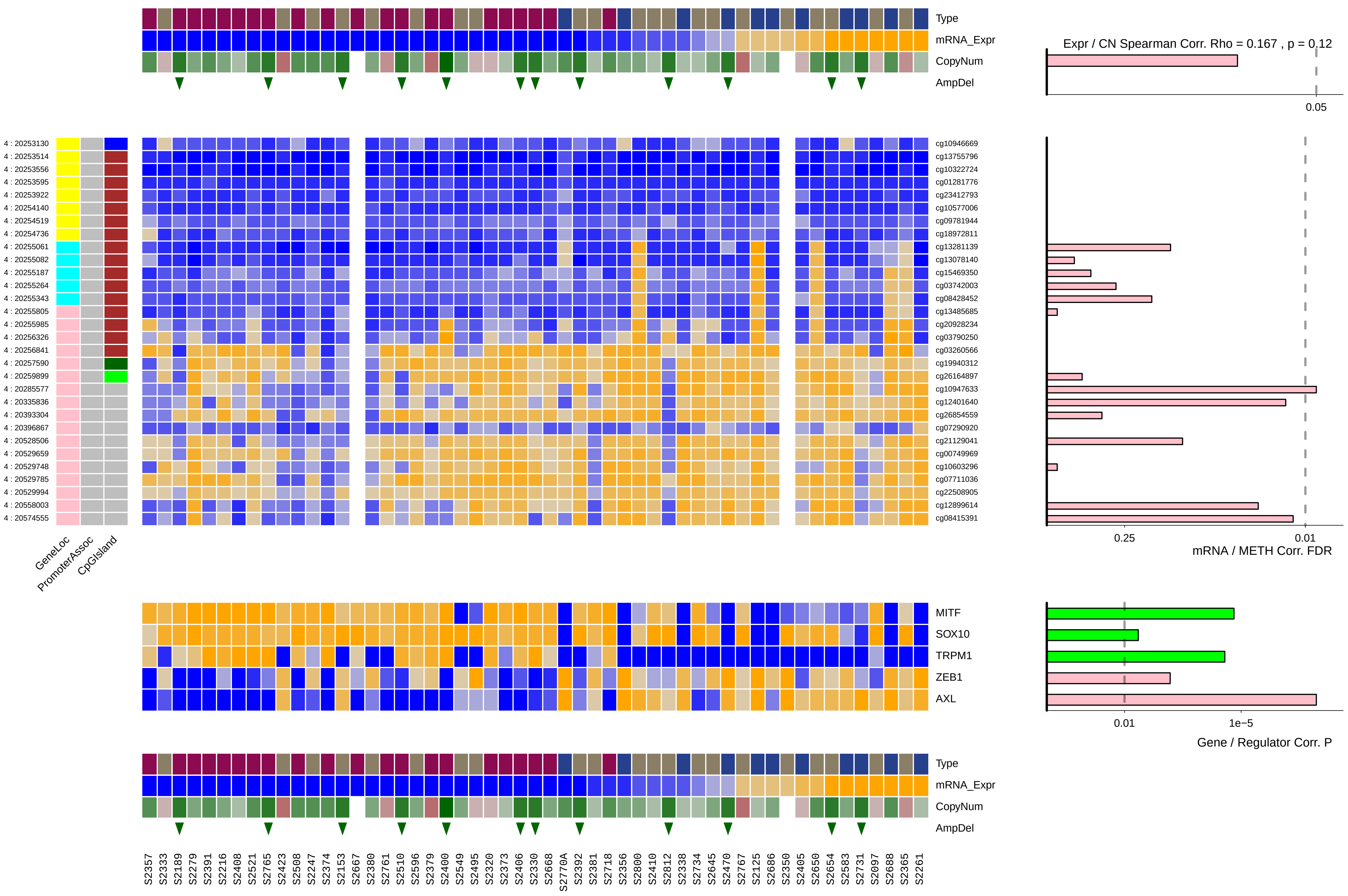
Type
mRNA_Expr
CopyNum
AmpDel
Expr / CN Spearman Corr. Rho = 0.167 , p = 0.12
0.05
GeneLoc
PromoterAssoc
CpGIsland
0.25
0.01
mRNA / METH Corr. FDR
MITF
SOX10
TRPM1
ZEB1
AXL
0.01
1e−5
Gene / Regulator Corr. P

LTBP2

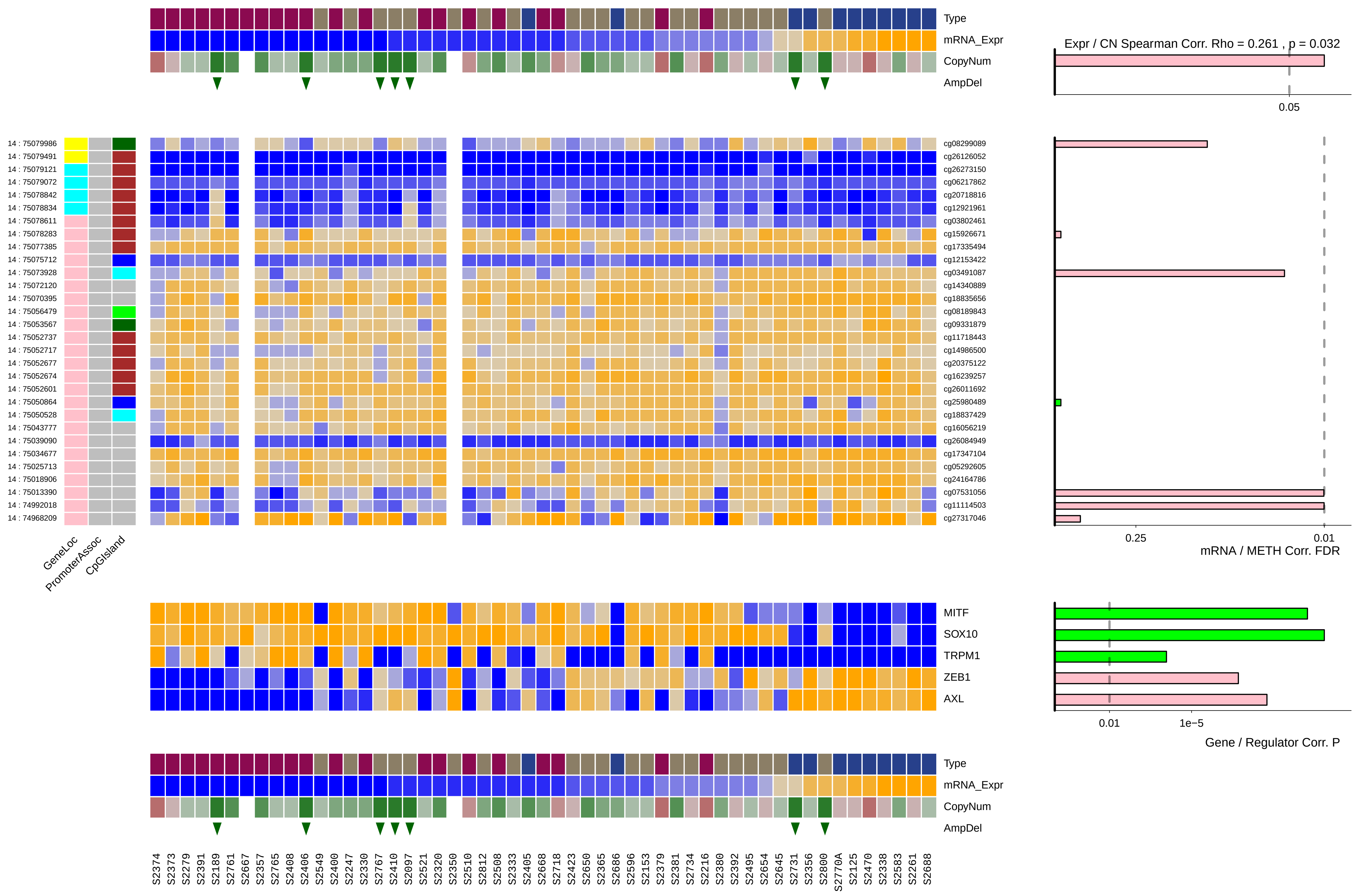

Type
mRNA_Expr
CopyNum
AmpDel
Expr / CN Spearman Corr. Rho = 0.261 , p = 0.032
0.05
GeneLoc
PromoterAssoc
CpGIsland
0.25
0.01
mRNA / METH Corr. FDR
MITF
SOX10
TRPM1
ZEB1
AXL
0.01
1e−5
Gene / Regulator Corr. P

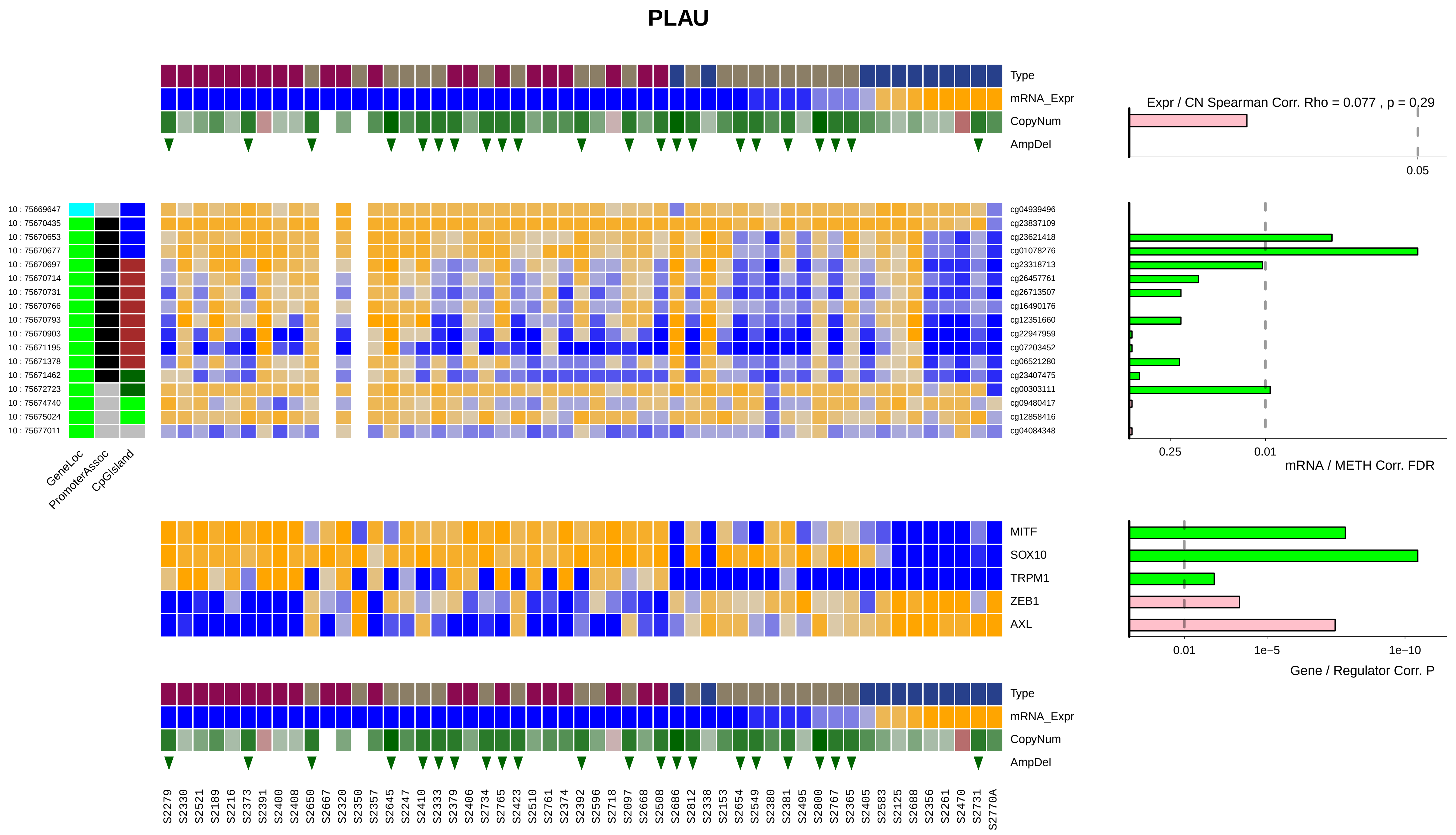
PLAU
Type
mRNA_Expr
CopyNum
AmpDel
Expr / CN Spearman Corr. Rho = 0.077 , p = 0.29
0.05
10 : 75669647
10 : 75670435
10 : 75670653
10 : 75670677
10 : 75670697
10 : 75670714
10 : 75670731
10 : 75670766
10 : 75670793
10 : 75670903
10 : 75671195
10 : 75671378
10 : 75671462
10 : 75672723
10 : 75674740
10 : 75675024
10 : 75677011
cg04939496
cg23837109
cg23621418
cg01078276
cg23318713
cg26457761
cg26713507
cg16490176
cg12351660
cg22947959
cg07203452
cg06521280
cg23407475
cg00303111
cg09480417
cg12858416
cg04084348
0.25
0.01
mRNA / METH Corr. FDR
GeneLoc
PromoterAssoc
CpGIsland
MITF
SOX10
TRPM1
ZEB1
AXL
0.01
1e−5
1e−10
Gene / Regulator Corr. P
Type
mRNA_Expr
CopyNum
AmpDel
S2279 S2330 S2521 S2189 S2216 S2373 S2391 S2400 S2408 S2650 S2667 S2320 S2350 S2357 S2645 S2247 S2410 S2333 S2379 S2406 S2734 S2765 S2423 S2510 S2761 S2374 S2392 S2596 S2718 S2097 S2668 S2508 S2686 S2812 S2338 S2153 S2654 S2549 S2380 S2381 S2495 S2800 S2767 S2365 S2405 S2583 S2125 S2688 S2356 S2261 S2470 S2731 S2770A

ADAM12

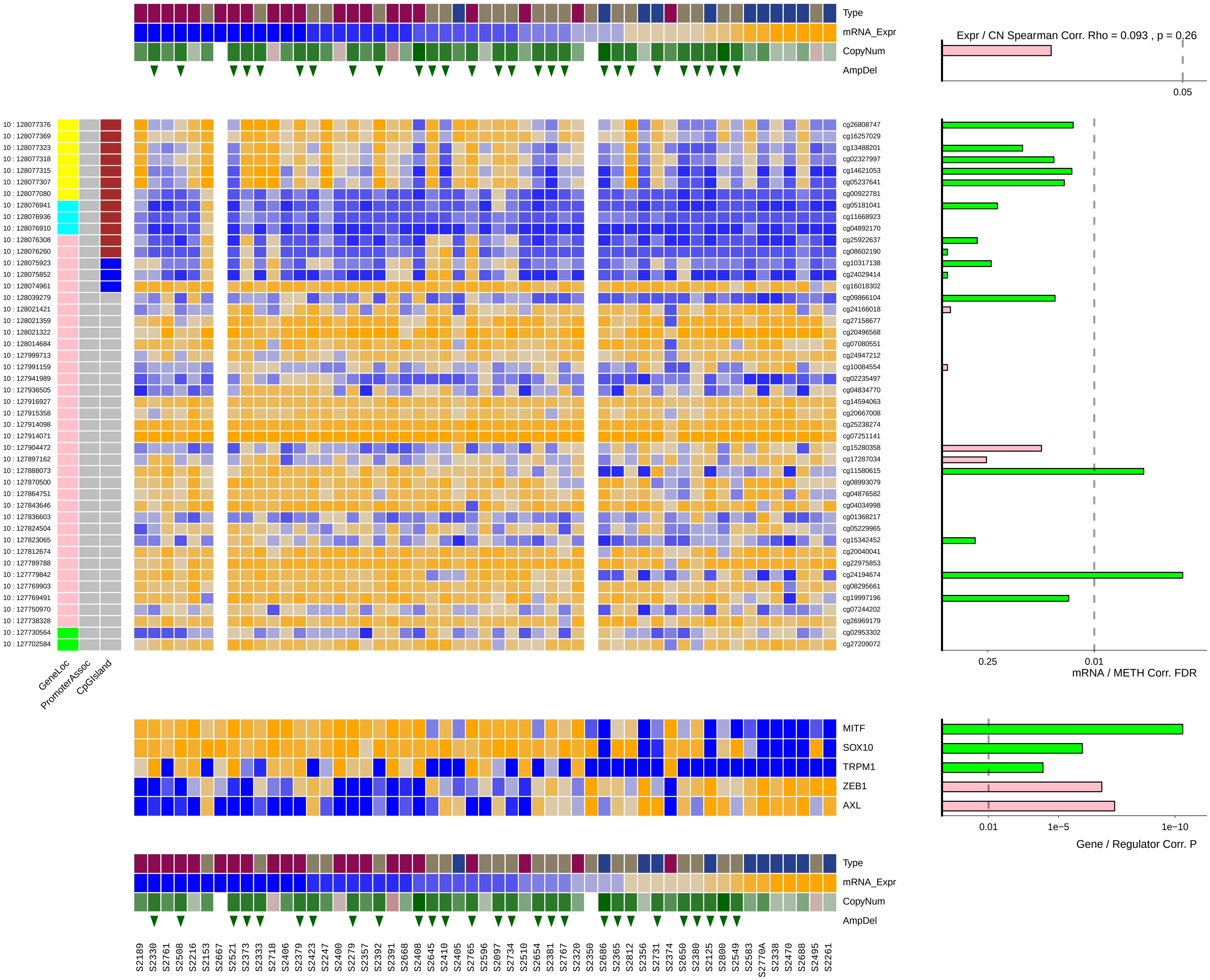

Type
mRNA_Expr
CopyNum
AmpDel
Expr / CN Spearman Corr. Rho = 0.093 , p = 0.26
0.05
10 : 128077376
10 : 128077369
10 : 128077323
10 : 128077318
10 : 128077315
10 : 128077307
10 : 128077080
10 : 128076941
10 : 128076936
10 : 128076910
10 : 128076308
10 : 128076260
10 : 128075923
10 : 128075852
10 : 128074961
10 : 128039279
10 : 128021421
10 : 128021359
10 : 128021322
10 : 128014684
10 : 127999713
10 : 127991159
10 : 127941989
10 : 127936505
10 : 127916927
10 : 127915358
10 : 127914098
10 : 127914071
10 : 127904472
10 : 127897162
10 : 127888073
10 : 127870500
10 : 127864751
10 : 127843646
10 : 127836603
10 : 127824504
10 : 127823065
10 : 127812674
10 : 127789788
10 : 127779842
10 : 127769903
10 : 127769491
10 : 127750970
10 : 127738328
10 : 127730564
10 : 127702584
cg26808747
cg16257029
cg13488201
cg02327997
cg14621053
cg05237641
cg00922781
cg05181041
cg11668923
cg04892170
cg25922637
cg08602190
cg10317138
cg24029414
cg16018302
cg09866104
cg24166018
cg27158677
cg20496568
cg07080551
cg24947212
cg10084554
cg02235497
cg04834770
cg14594063
cg20667008
cg25238274
cg07251141
cg15280358
cg17287034
cg11580615
cg08993079
cg04876582
cg04034998
cg01368217
cg05229965
cg15342452
cg20040041
cg22975853
cg24194674
cg08295661
cg19997196
cg07244202
cg26969179
cg02953302
cg27209072
0.25
0.01
mRNA / METH Corr. FDR
GeneLoc
PromoterAssoc
CpGIsland
MITF
SOX10
TRPM1
ZEB1
AXL
0.01
1e−5
1e−10
Gene / Regulator Corr. P
Type
mRNA_Expr
CopyNum
AmpDel
S2189 S2330 S2761 S2508 S2216 S2153 S2667 S2521 S2373 S2333 S2718 S2406 S2379 S2423 S2247 S2400 S2279 S2357 S2392 S2391 S2668 S2408 S2645 S2410 S2405 S2765 S2596 S2097 S2734 S2510 S2654 S2381 S2767 S2320 S2350 S2686 S2365 S2812 S2356 S2731 S2374 S2650 S2380 S2125 S2800 S2549 S2583 S2770A S2338 S2470 S2688 S2495 S2261

SDC4

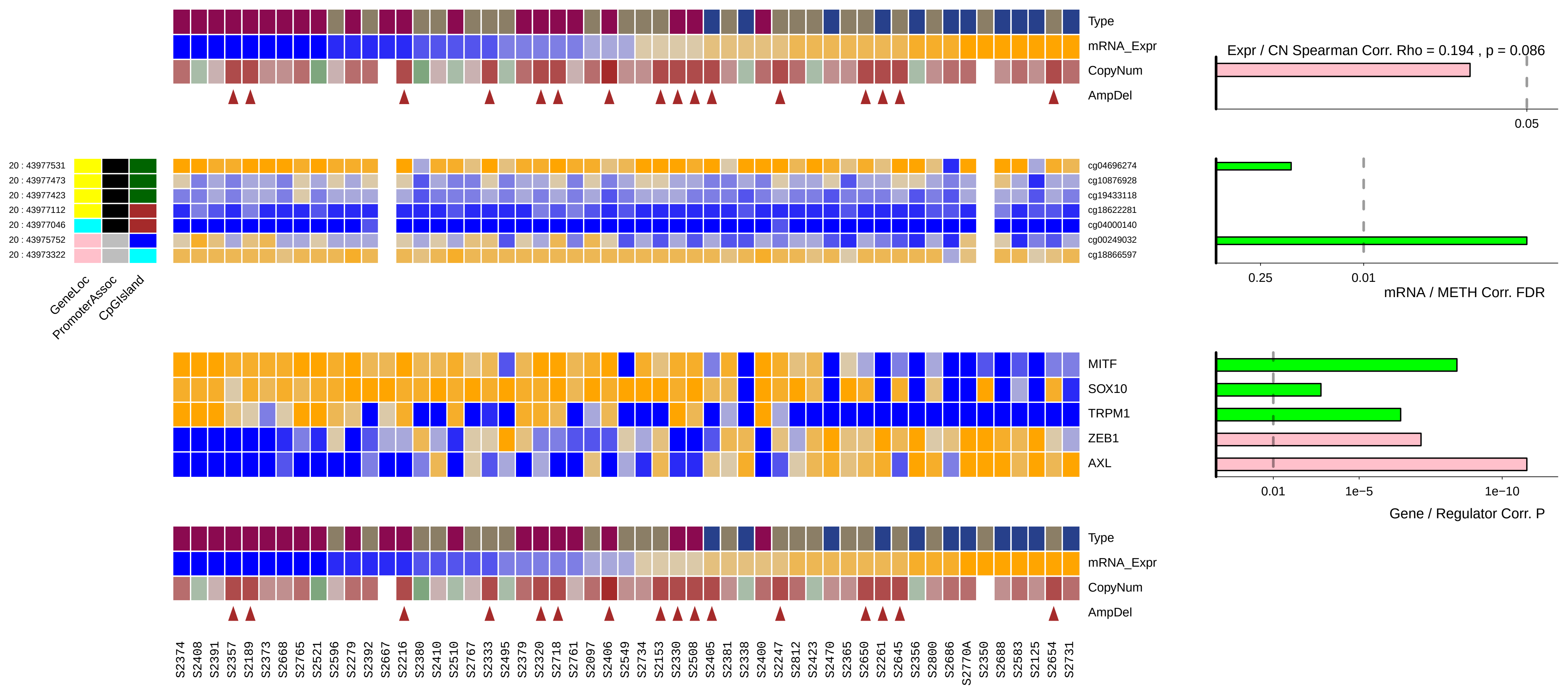

Type
mRNA_Expr
CopyNum
AmpDel
Expr / CN Spearman Corr. Rho = 0.194 , p = 0.086
0.05
20 : 43977531
20 : 43977473
20 : 43977423
20 : 43977112
20 : 43977046
20 : 43975752
20 : 43973322
cg04696274
cg10876928
cg19433118
cg18622281
cg04000140
cg00249032
cg18866597
GeneLoc
PromoterAssoc
CpGIsland
0.25
0.01
mRNA / METH Corr. FDR
MITF
SOX10
TRPM1
ZEB1
AXL
0.01
1e−5
1e−10
Gene / Regulator Corr. P
S2374 S2408 S2391 S2357 S2189 S2373 S2668 S2765 S2521 S2596 S2279 S2392 S2667 S2216 S2380 S2410 S2510 S2767 S2333 S2495 S2379 S2320 S2718 S2761 S2097 S2406 S2549 S2734 S2153 S2330 S2508 S2405 S2381 S2338 S2400 S2247 S2812 S2423 S2470 S2365 S2650 S2261 S2645 S2356 S2800 S2686 S2770A S2350 S2688 S2583 S2125 S2654 S2731

GLIPR1

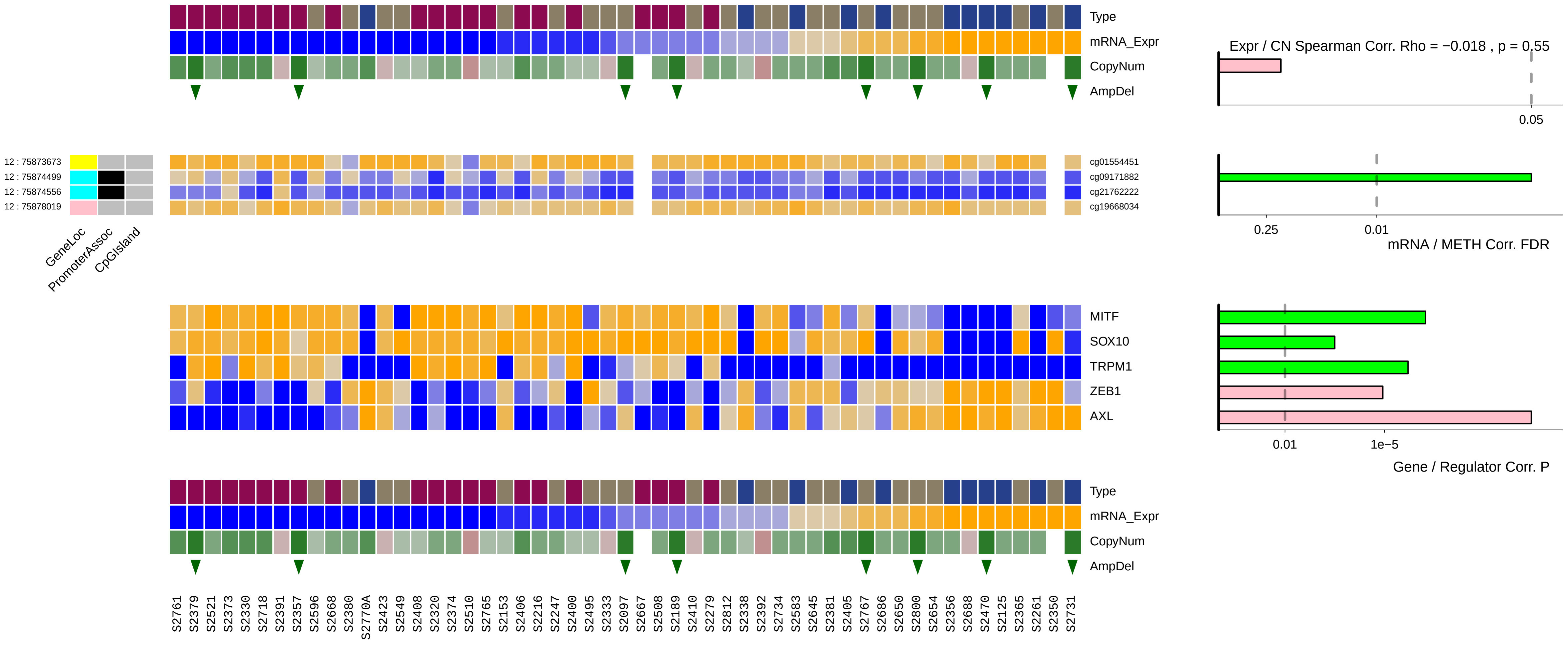
Type
mRNA_Expr
CopyNum
AmpDel
Expr / CN Spearman Corr. Rho = −0.018 , p = 0.55
0.05
12 : 75873673
12 : 75874499
12 : 75874556
12 : 75878019
GeneLoc
PromoterAssoc
CpGIsland
cg01554451
cg09171882
cg21762222
cg19668034
0.25
0.01
mRNA / METH Corr. FDR
MITF
SOX10
TRPM1
ZEB1
AXL
0.01
1e−5
Gene / Regulator Corr. P
S2761 S2379 S2521 S2373 S2330 S2718 S2391 S2357 S2596 S2668 S2380 S2770A S2423 S2549 S2408 S2320 S2374 S2510 S2765 S2153 S2406 S2216 S2247 S2400 S2495 S2333 S2097 S2667 S2508 S2189 S2410 S2279 S2812 S2338 S2392 S2734 S2583 S2645 S2381 S2405 S2767 S2686 S2650 S2800 S2654 S2356 S2688 S2470 S2125 S2365 S2261 S2350 S2731

NTN4

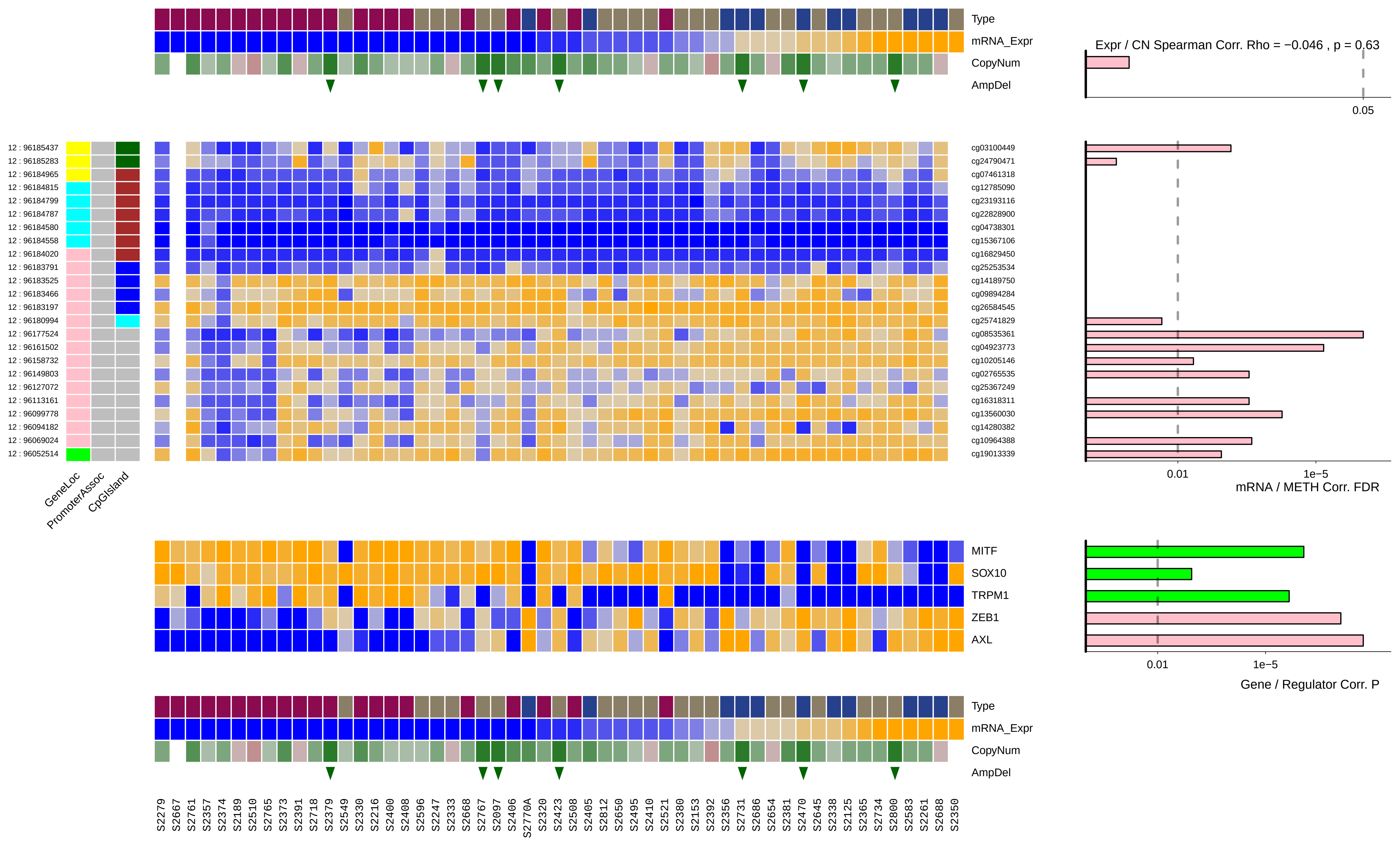

Type
mRNA_Expr
CopyNum
AmpDel
Expr / CN Spearman Corr. Rho = −0.046 , p = 0.63
0.05
12 : 96185437
12 : 96185283
12 : 96184965
12 : 96184815
12 : 96184799
12 : 96184787
12 : 96184580
12 : 96184558
12 : 96184020
12 : 96183791
12 : 96183525
12 : 96183466
12 : 96183197
12 : 96180994
12 : 96177524
12 : 96161502
12 : 96158732
12 : 96149803
12 : 96127072
12 : 96113161
12 : 96099778
12 : 96094182
12 : 96069024
12 : 96052514
cg03100449
cg24790471
cg07461318
cg12785090
cg23193116
cg22828900
cg04738301
cg15367106
cg16829450
cg25253534
cg14189750
cg09894284
cg26584545
cg25741829
cg08535361
cg04923773
cg10205146
cg02765535
cg25367249
cg16318311
cg13560030
cg14280382
cg10964388
cg19013339
0.01
1e−5
mRNA / METH Corr. FDR
GeneLoc
PromoterAssoc
CpGIsland
MITF
SOX10
TRPM1
ZEB1
AXL
0.01
1e−5
Gene / Regulator Corr. P
Type
mRNA_Expr
CopyNum
AmpDel
S2279 S2667 S2761 S2357 S2374 S2189 S2510 S2765 S2373 S2391 S2718 S2379 S2549 S2330 S2216 S2400 S2408 S2596 S2247 S2333 S2668 S2767 S2097 S2406 S2770A S2320 S2423 S2508 S2405 S2812 S2650 S2495 S2410 S2521 S2380 S2153 S2392 S2356 S2731 S2686 S2654 S2381 S2470 S2645 S2338 S2125 S2365 S2734 S2800 S2583 S2261 S2688 S2350

TCF4

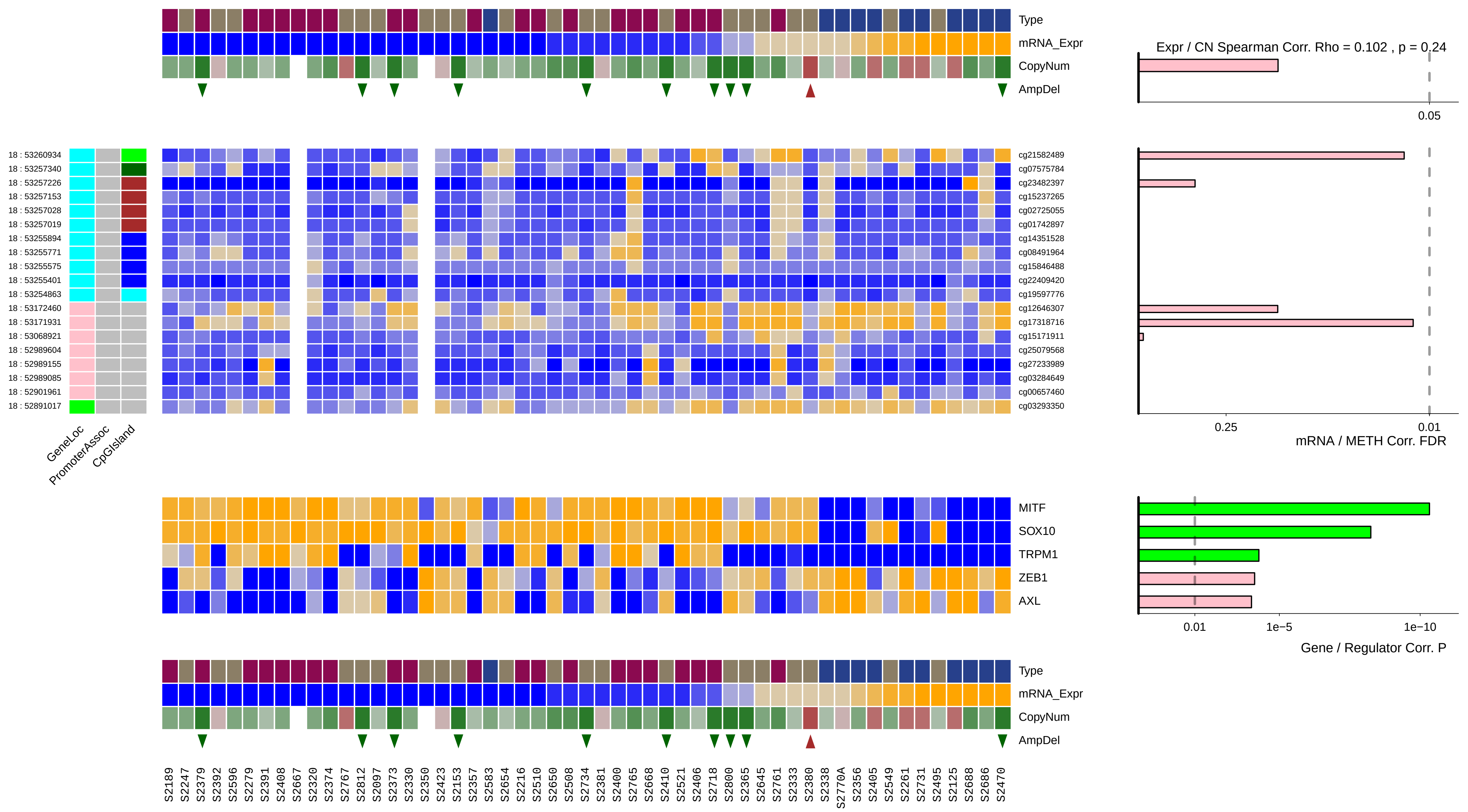

Type
mRNA_Expr
CopyNum
AmpDel
Expr / CN Spearman Corr. Rho = 0.102 , p = 0.24
0.05
18 : 53260934
18 : 53257340
18 : 53257226
18 : 53257153
18 : 53257028
18 : 53257019
18 : 53255894
18 : 53255771
18 : 53255575
18 : 53255401
18 : 53254863
18 : 53172460
18 : 53171931
18 : 53068921
18 : 52989604
18 : 52989155
18 : 52989085
18 : 52901961
18 : 52891017
GeneLoc
PromoterAssoc
CpGIsland
cg21582489
cg07575784
cg23482397
cg15237265
cg02725055
cg01742897
cg14351528
cg08491964
cg15846488
cg22409420
cg19597776
cg12646307
cg17318716
cg15171911
cg25079568
cg27233989
cg03284649
cg00657460
cg03293350
0.25
0.01
mRNA / METH Corr. FDR
MITF
SOX10
TRPM1
ZEB1
AXL
0.01
1e−5
1e−10
Gene / Regulator Corr. P
Type
mRNA_Expr
CopyNum
AmpDel
S2189
S2247
S2379
S2392
S2596
S2279
S2391
S2408
S2667
S2320
S2374
S2767
S2812
S2097
S2373
S2330
S2350
S2423
S2153
S2357
S2583
S2654
S2216
S2510
S2650
S2508
S2734
S2381
S2400
S2765
S2668
S2410
S2521
S2406
S2718
S2800
S2365
S2645
S2761
S2333
S2380
S2338
S2770A
S2356
S2405
S2549
S2261
S2731
S2495
S2125
S2688
S2686
S2470

EPHA2

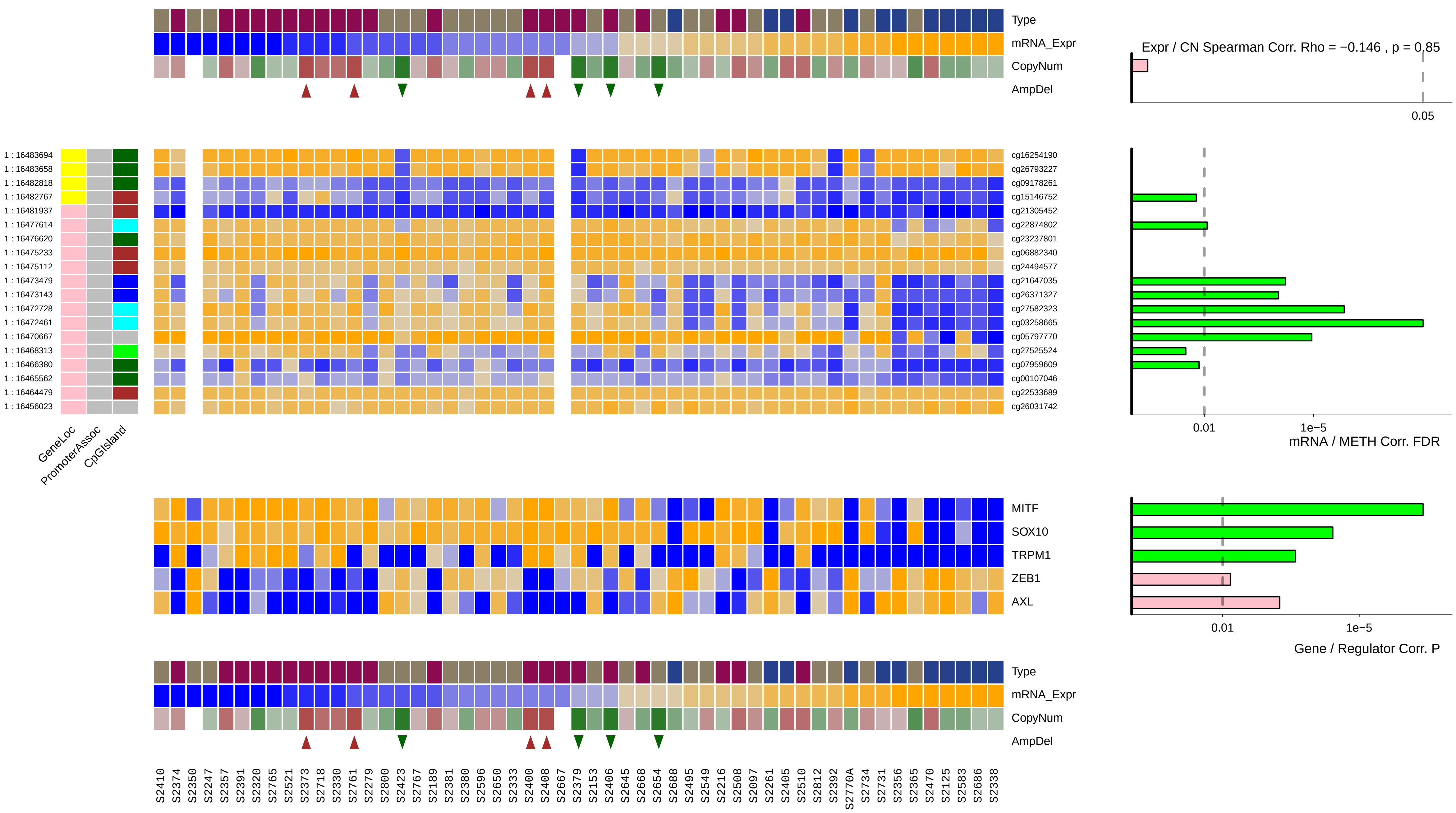

Type
mRNA_Expr
CopyNum
AmpDel
Expr / CN Spearman Corr. Rho = −0.146 , p = 0.85
0.05
1 : 16483694
1 : 16483658
1 : 16482818
1 : 16482767
1 : 16481937
1 : 16477614
1 : 16476620
1 : 16475233
1 : 16475112
1 : 16473479
1 : 16473143
1 : 16472728
1 : 16472461
1 : 16470667
1 : 16468313
1 : 16466380
1 : 16465562
1 : 16464479
1 : 16456023
cg16254190
cg26793227
cg09178261
cg15146752
cg21305452
cg22874802
cg23237801
cg06882340
cg24494577
cg21647035
cg26371327
cg27582323
cg03258665
cg05797770
cg27525524
cg07959609
cg00107046
cg22533689
cg26031742
0.01
1e−5
mRNA / METH Corr. FDR
GeneLoc
PromoterAssoc
CpGIsland
MITF
SOX10
TRPM1
ZEB1
AXL
0.01
1e−5
Gene / Regulator Corr. P
Type
mRNA_Expr
CopyNum
AmpDel
S2410 S2374 S2350 S2247 S2357 S2391 S2320 S2765 S2521 S2373 S2718 S2330 S2761 S2279 S2800 S2423 S2767 S2189 S2381 S2380 S2596 S2650 S2333 S2400 S2408 S2667 S2379 S2153 S2406 S2645 S2668 S2654 S2688 S2495 S2549 S2216 S2508 S2097 S2261 S2405 S2510 S2812 S2392 S2770A S2734 S2731 S2356 S2365 S2470 S2125 S2583 S2686 S2338

GBP3

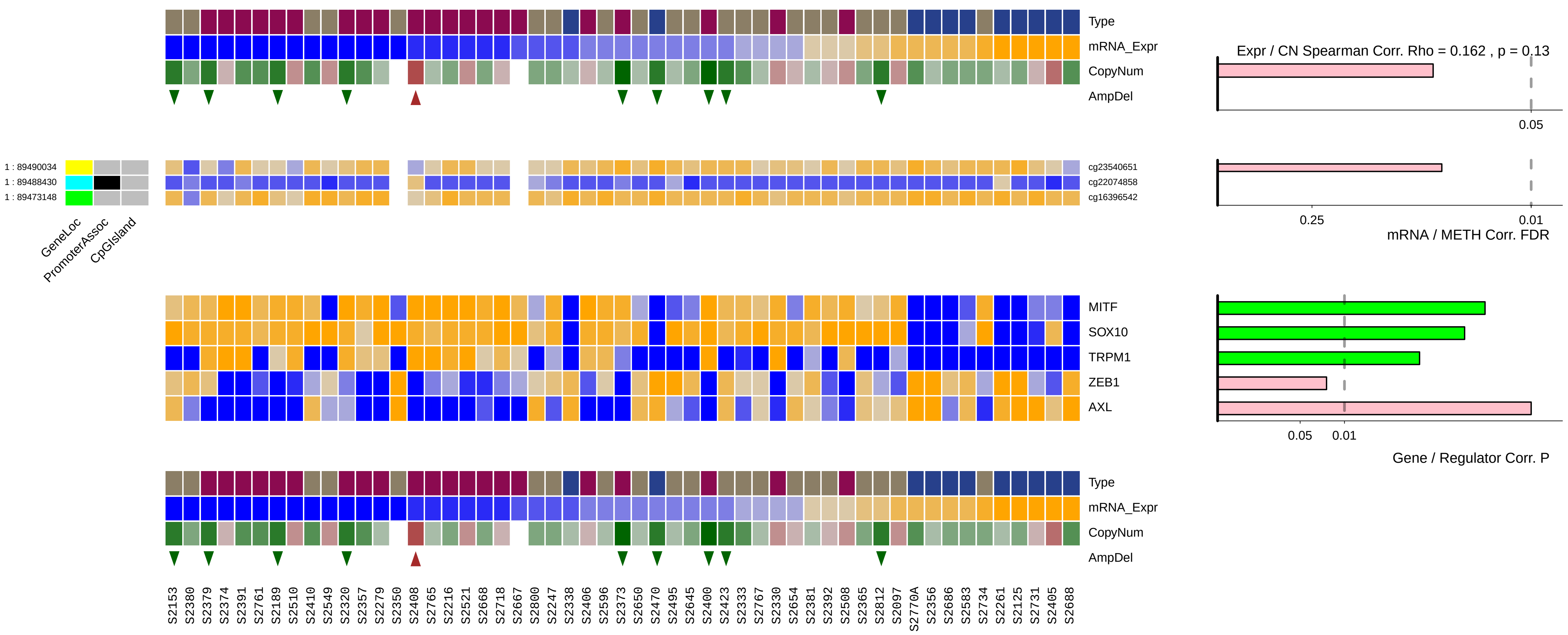
Type
mRNA_Expr
CopyNum
AmpDel
Expr / CN Spearman Corr. Rho = 0.162 , p = 0.13
0.05
1 : 89490034
1 : 89488430
1 : 89473148
cg23540651
cg22074858
cg16396542
0.25
0.01
mRNA / METH Corr. FDR
GeneLoc
PromoterAssoc
CpGIsland
MITF
SOX10
TRPM1
ZEB1
AXL
0.05
0.01
Gene / Regulator Corr. P
S2153 S2380 S2379 S2374 S2391 S2761 S2189 S2510 S2410 S2549 S2320 S2357 S2279 S2350 S2408 S2765 S2216 S2521 S2668 S2718 S2667 S2800 S2247 S2338 S2406 S2596 S2373 S2650 S2470 S2495 S2645 S2400 S2423 S2333 S2767 S2330 S2654 S2381 S2392 S2508 S2365 S2812 S2097 S2770A S2356 S2686 S2583 S2734 S2261 S2125 S2731 S2405 S2688

COL6A1

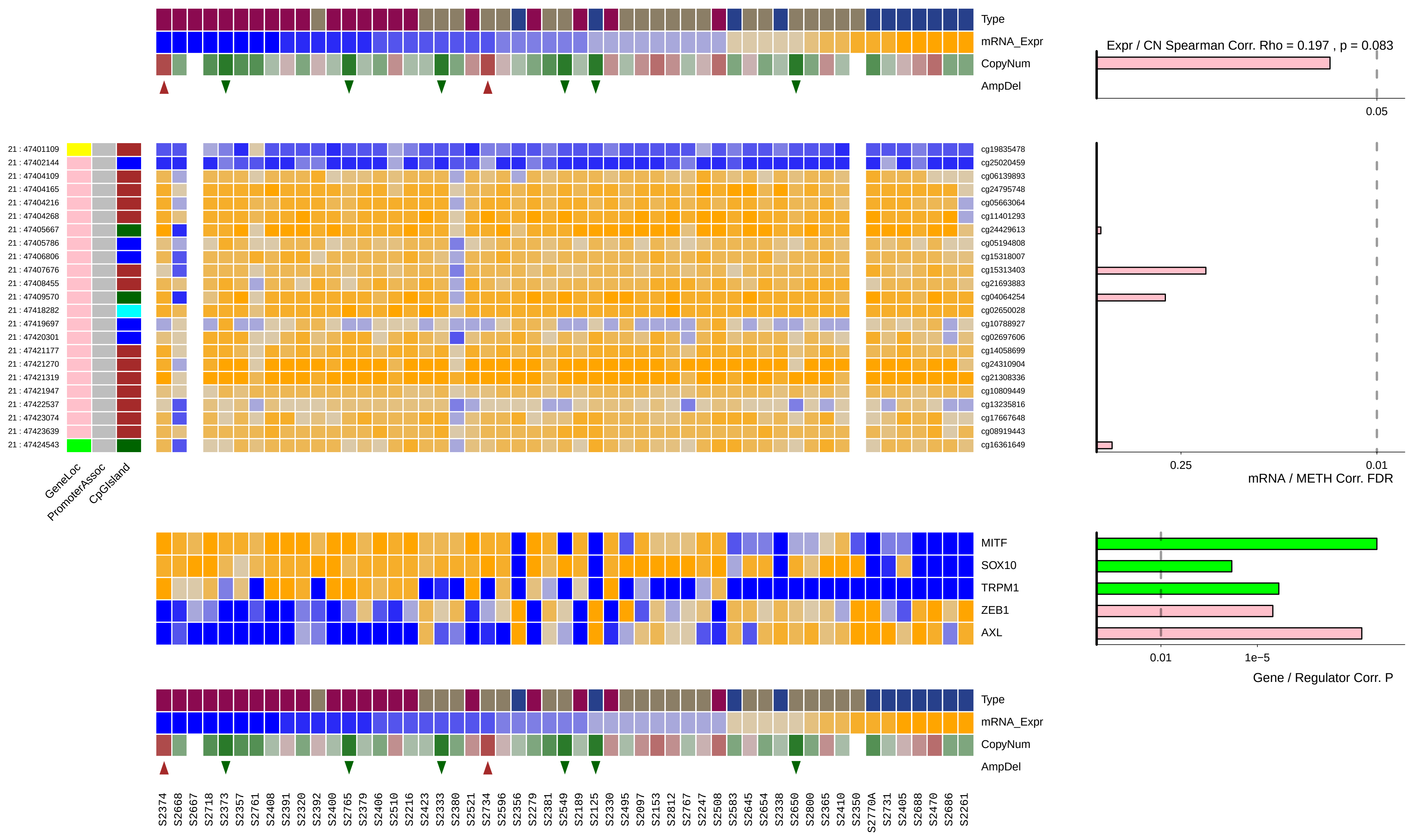

Type
mRNA_Expr
CopyNum
AmpDel
Expr / CN Spearman Corr. Rho = 0.197 , p = 0.083
0.05
21 : 47401109
21 : 47402144
21 : 47404109
21 : 47404165
21 : 47404216
21 : 47404268
21 : 47405667
21 : 47405786
21 : 47406806
21 : 47407676
21 : 47408455
21 : 47409570
21 : 47418282
21 : 47419697
21 : 47420301
21 : 47421177
21 : 47421270
21 : 47421319
21 : 47421947
21 : 47422537
21 : 47423074
21 : 47423639
21 : 47424543
GeneLoc
PromoterAssoc
CpGIsland
cg19835478
cg25020459
cg06139893
cg24795748
cg05663064
cg11401293
cg24429613
cg05194808
cg15318007
cg15313403
cg21693883
cg04064254
cg02650028
cg10788927
cg02697606
cg14058699
cg24310904
cg21308336
cg10809449
cg13235816
cg17667648
cg08919443
cg16361649
0.25
0.01
mRNA / METH Corr. FDR
MITF
SOX10
TRPM1
ZEB1
AXL
0.01
1e−5
Gene / Regulator Corr. P
Type
mRNA_Expr
CopyNum
AmpDel
S2374
S2668
S2667
S2718
S2373
S2357
S2761
S2408
S2391
S2320
S2392
S2400
S2765
S2379
S2406
S2510
S2216
S2423
S2333
S2380
S2521
S2734
S2596
S2356
S2279
S2381
S2549
S2189
S2125
S2330
S2495
S2097
S2153
S2812
S2767
S2247
S2508
S2583
S2645
S2654
S2338
S2650
S2800
S2365
S2410
S2350
S2770A
S2731
S2405
S2688
S2470
S2686
S2261

ZEB1

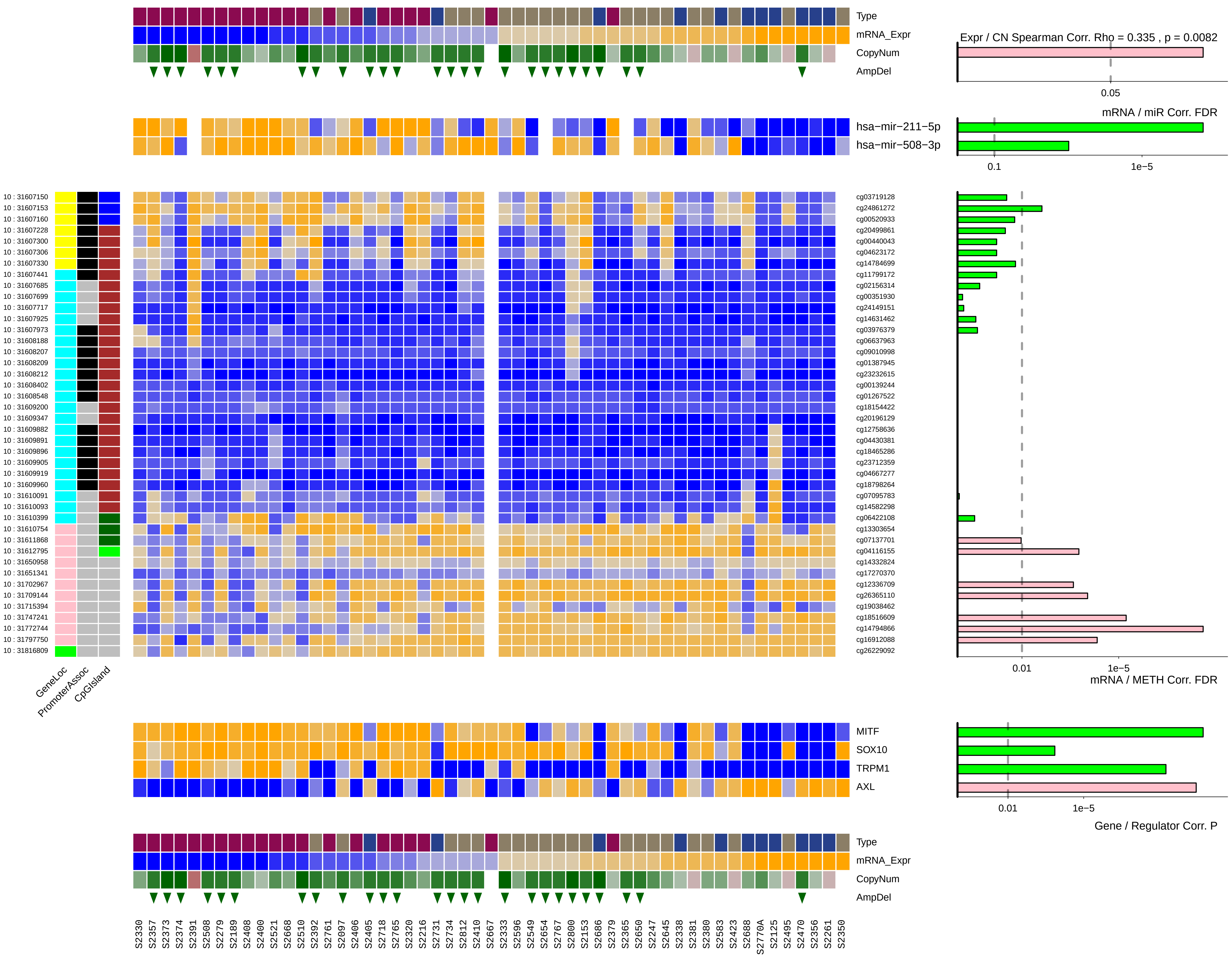
Type
mRNA_Expr
CopyNum
AmpDel
Expr / CN Spearman Corr. Rho = 0.335 , p = 0.0082
0.05
mRNA / miR Corr. FDR
hsa−mir−211−5p
hsa−mir−508−3p
0.1
1e−5
10 : 31607150
10 : 31607153
10 : 31607160
10 : 31607228
10 : 31607300
10 : 31607306
10 : 31607330
10 : 31607441
10 : 31607685
10 : 31607699
10 : 31607717
10 : 31607925
10 : 31607973
10 : 31608188
10 : 31608207
10 : 31608209
10 : 31608212
10 : 31608402
10 : 31608548
10 : 31609200
10 : 31609347
10 : 31609882
10 : 31609891
10 : 31609896
10 : 31609905
10 : 31609919
10 : 31609960
10 : 31610091
10 : 31610093
10 : 31610399
10 : 31610754
10 : 31611868
10 : 31612795
10 : 31650958
10 : 31651341
10 : 31702967
10 : 31709144
10 : 31715394
10 : 31747241
10 : 31772744
10 : 31797750
10 : 31816809
cg03719128
cg24861272
cg00520933
cg20499861
cg00440043
cg04623172
cg14784699
cg11799172
cg02156314
cg00351930
cg24149151
cg14631462
cg03976379
cg06637963
cg09010998
cg01387945
cg23232615
cg00139244
cg01267522
cg18154422
cg20196129
cg12758636
cg04430381
cg18465286
cg23712359
cg04667277
cg18798264
cg07095783
cg14582298
cg06422108
cg13303654
cg07137701
cg04116155
cg14332824
cg17270370
cg12336709
cg26365110
cg19038462
cg18516609
cg14794866
cg16912088
cg26229092
0.01
1e−5
mRNA / METH Corr. FDR
GeneLoc
PromoterAssoc
CpGIsland
MITF
SOX10
TRPM1
AXL
0.01
1e−5
Gene / Regulator Corr. P
Type
mRNA_Expr
CopyNum
AmpDel
S2330 S2357 S2373 S2374 S2391 S2508 S2279 S2189 S2408 S2400 S2521 S2668 S2510 S2392 S2761 S2097 S2406 S2405 S2718 S2765 S2320 S2216 S2731 S2734 S2812 S2410 S2667 S2333 S2596 S2549 S2654 S2767 S2800 S2153 S2686 S2379 S2365 S2650 S2247 S2645 S2338 S2381 S2380 S2583 S2423 S2688 S2770A S2125 S2495 S2470 S2356 S2261 S2350

CYR61

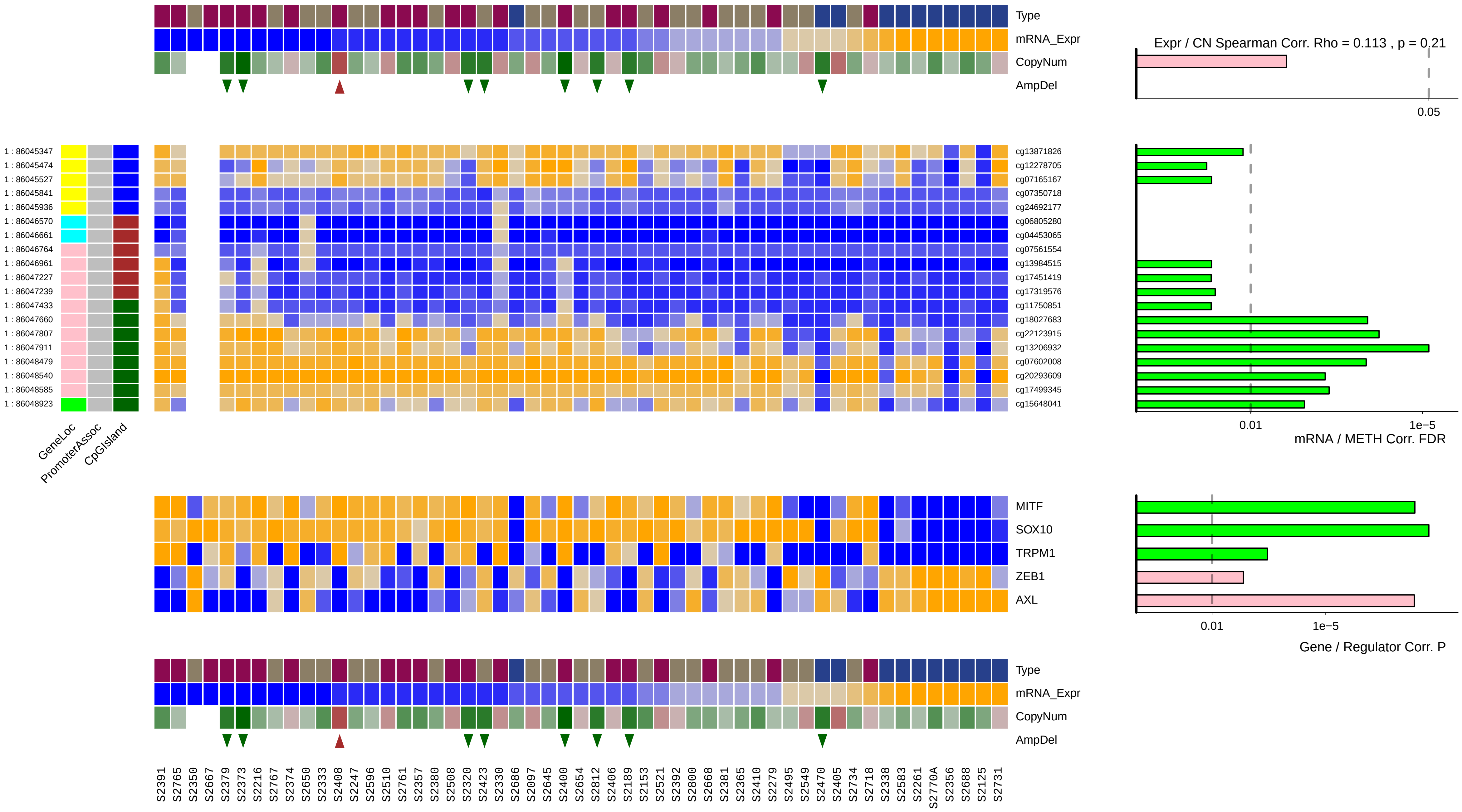
Type
mRNA_Expr
CopyNum
AmpDel
Expr / CN Spearman Corr. Rho = 0.113 , p = 0.21
0.05
1 : 86045347
1 : 86045474
1 : 86045527
1 : 86045841
1 : 86045936
1 : 86046570
1 : 86046661
1 : 86046764
1 : 86046961
1 : 86047227
1 : 86047239
1 : 86047433
1 : 86047660
1 : 86047807
1 : 86047911
1 : 86048479
1 : 86048540
1 : 86048585
1 : 86048923
GeneLoc
PromoterAssoc
CpGIsland
cg13871826
cg12278705
cg07165167
cg07350718
cg24692177
cg06805280
cg04453065
cg07561554
cg13984515
cg17451419
cg17319576
cg11750851
cg18027683
cg22123915
cg13206932
cg07602008
cg20293609
cg17499345
cg15648041
0.01
1e−5
mRNA / METH Corr. FDR
MITF
SOX10
TRPM1
ZEB1
AXL
0.01
1e−5
Gene / Regulator Corr. P
Type
mRNA_Expr
CopyNum
AmpDel
S2391 S2765 S2350 S2667 S2379 S2373 S2216 S2767 S2374 S2650 S2333 S2408 S2247 S2596 S2510 S2761 S2357 S2380 S2508 S2320 S2423 S2330 S2686 S2097 S2645 S2400 S2654 S2812 S2406 S2189 S2153 S2521 S2392 S2800 S2668 S2381 S2365 S2410 S2279 S2495 S2549 S2470 S2405 S2734 S2718 S2338 S2583 S2261 S2770A S2356 S2688 S2125 S2731

LIFR

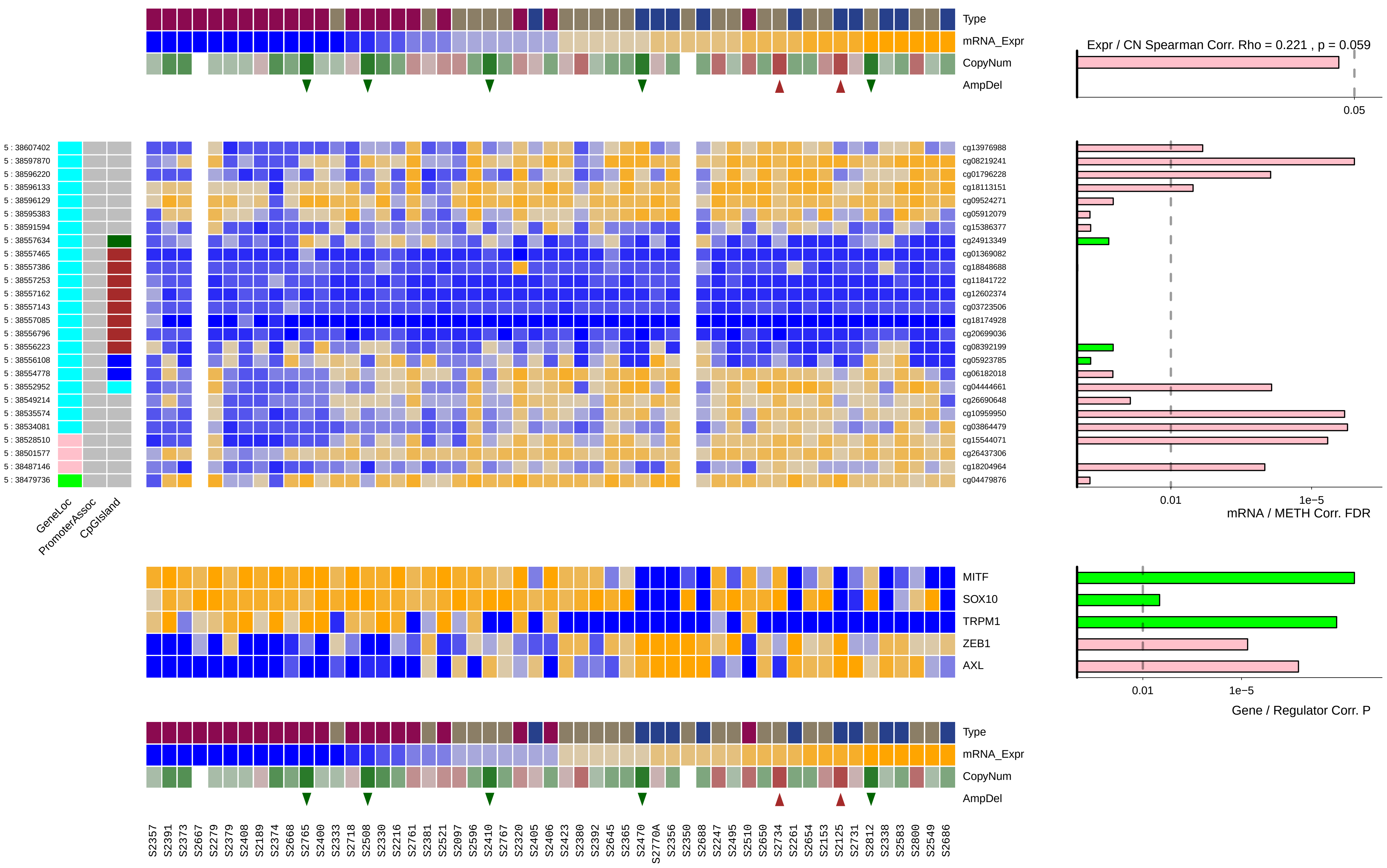

Type
mRNA_Expr
CopyNum
AmpDel
Expr / CN Spearman Corr. Rho = 0.221 , p = 0.059
0.05
5 : 38607402
5 : 38597870
5 : 38596220
5 : 38596133
5 : 38596129
5 : 38595383
5 : 38591594
5 : 38557634
5 : 38557465
5 : 38557386
5 : 38557253
5 : 38557162
5 : 38557143
5 : 38557085
5 : 38556796
5 : 38556223
5 : 38556108
5 : 38554778
5 : 38552952
5 : 38549214
5 : 38535574
5 : 38534081
5 : 38528510
5 : 38501577
5 : 38487146
5 : 38479736
cg13976988
cg08219241
cg01796228
cg18113151
cg09524271
cg05912079
cg15386377
cg24913349
cg01369082
cg18848688
cg11841722
cg12602374
cg03723506
cg18174928
cg20699036
cg08392199
cg05923785
cg06182018
cg04444661
cg26690648
cg10959950
cg03864479
cg15544071
cg26437306
cg18204964
cg04479876
0.01
1e−5
mRNA / METH Corr. FDR
GeneLoc
PromoterAssoc
CpGIsland
MITF
SOX10
TRPM1
ZEB1
AXL
0.01
1e−5
Gene / Regulator Corr. P
Type
mRNA_Expr
CopyNum
AmpDel
S2357
S2391
S2373
S2667
S2279
S2379
S2408
S2189
S2374
S2668
S2765
S2400
S2333
S2718
S2508
S2330
S2216
S2761
S2381
S2521
S2097
S2596
S2410
S2767
S2320
S2405
S2406
S2423
S2380
S2392
S2645
S2365
S2470
S2770A
S2356
S2350
S2688
S2247
S2495
S2510
S2650
S2734
S2261
S2654
S2153
S2125
S2731
S2812
S2338
S2583
S2800
S2549
S2686

KRT18

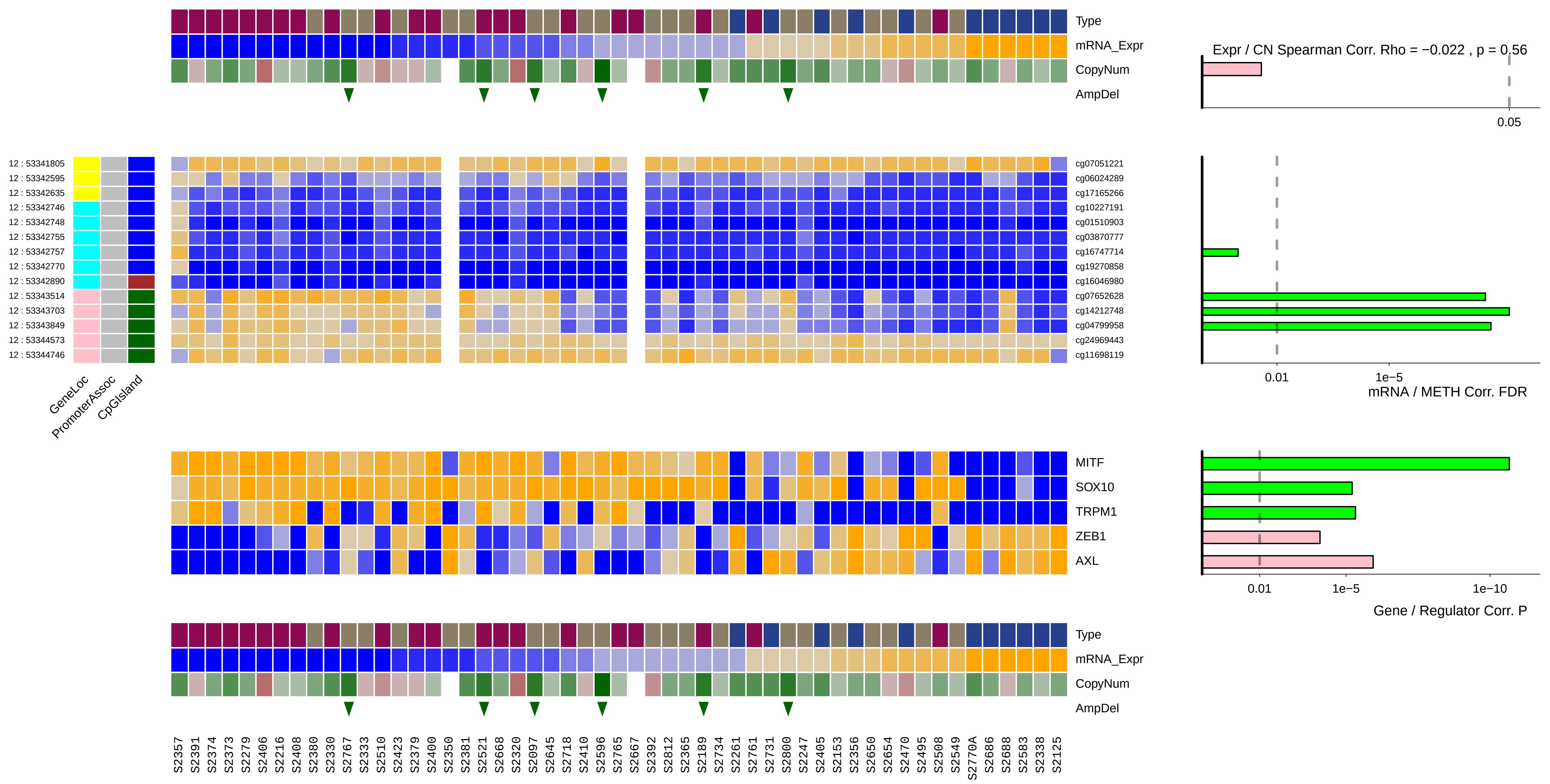
Type
mRNA_Expr
CopyNum
AmpDel
Expr / CN Spearman Corr. Rho = −0.022 , p = 0.56
0.05
12 : 53341805
12 : 53342595
12 : 53342635
12 : 53342746
12 : 53342748
12 : 53342755
12 : 53342757
12 : 53342770
12 : 53342890
12 : 53343514
12 : 53343703
12 : 53343849
12 : 53344573
12 : 53344746
cg07051221
cg06024289
cg17165266
cg10227191
cg01510903
cg03870777
cg16747714
cg19270858
cg16046980
cg07652628
cg14212748
cg04799958
cg24969443
cg11698119
GeneLoc
PromoterAssoc
CpGIsland
0.01
1e−5
mRNA / METH Corr. FDR
MITF
SOX10
TRPM1
ZEB1
AXL
0.01
1e−5
1e−10
Gene / Regulator Corr. P
Type
mRNA_Expr
CopyNum
AmpDel
S2357 S2391 S2374 S2373 S2279 S2406 S2216 S2408 S2380 S2330 S2767 S2333 S2510 S2423 S2379 S2400 S2350 S2381 S2521 S2668 S2320 S2097 S2645 S2718 S2410 S2596 S2765 S2667 S2392 S2812 S2365 S2189 S2734 S2261 S2761 S2731 S2800 S2247 S2405 S2153 S2356 S2650 S2654 S2470 S2495 S2508 S2549 S2770A S2686 S2688 S2583 S2338 S2125

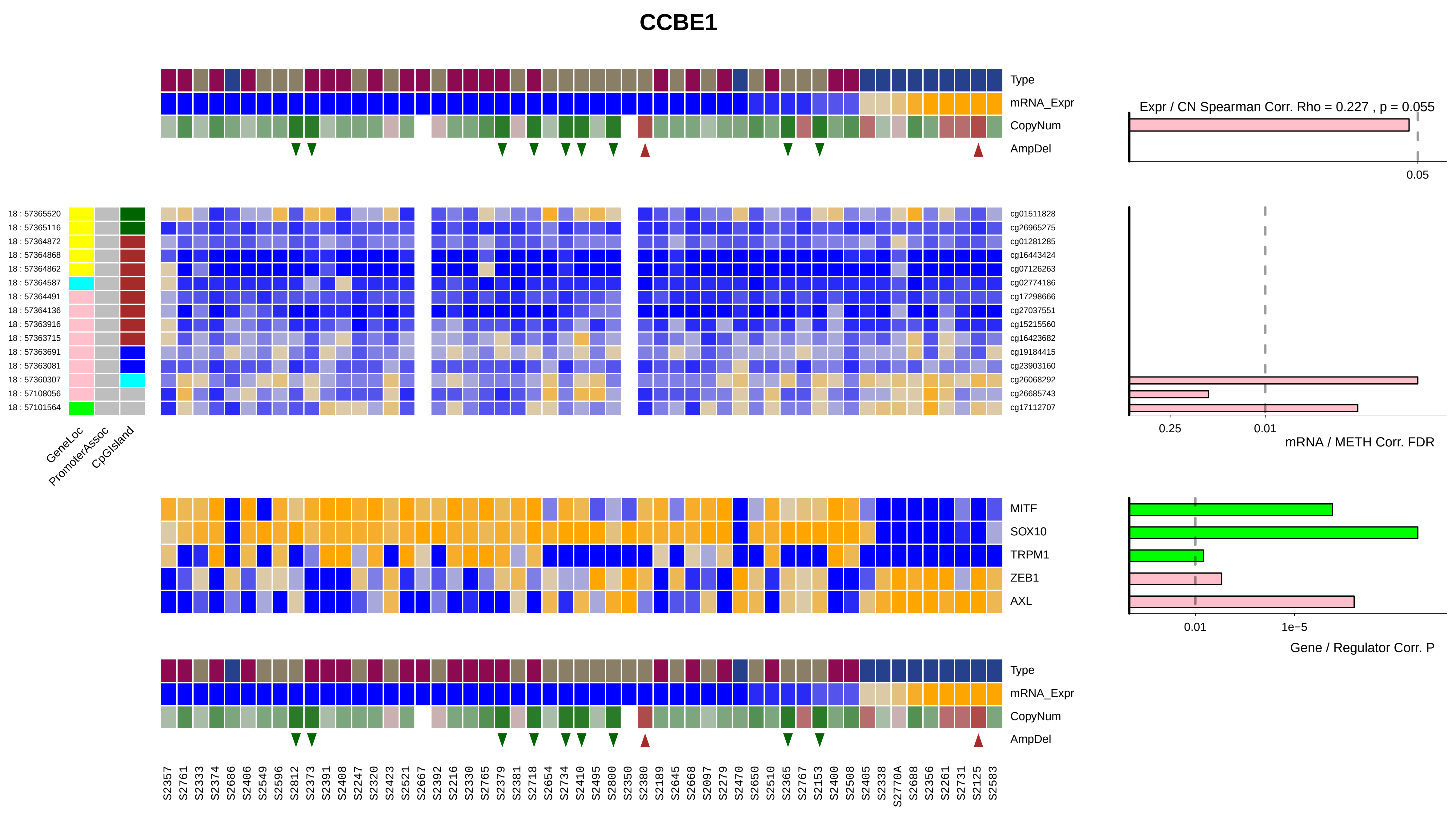
CCBE1
Type
mRNA_Expr
CopyNum
AmpDel
Expr / CN Spearman Corr. Rho = 0.227 , p = 0.055
0.05
18 : 57365520
18 : 57365116
18 : 57364872
18 : 57364868
18 : 57364862
18 : 57364587
18 : 57364491
18 : 57364136
18 : 57363916
18 : 57363715
18 : 57363691
18 : 57363081
18 : 57360307
18 : 57108056
18 : 57101564
cg01511828
cg26965275
cg01281285
cg16443424
cg07126263
cg02774186
cg17298666
cg27037551
cg15215560
cg16423682
cg19184415
cg23903160
cg26068292
cg26685743
cg17112707
GeneLoc
PromoterAssoc
CpGIsland
0.25
0.01
mRNA / METH Corr. FDR
MITF
SOX10
TRPM1
ZEB1
AXL
0.01
1e−5
Gene / Regulator Corr. P
S2357 S2761 S2333 S2374 S2686 S2406 S2549 S2596 S2812 S2373 S2391 S2408 S2247 S2320 S2423 S2521 S2667 S2392 S2216 S2330 S2765 S2379 S2381 S2718 S2654 S2734 S2410 S2495 S2800 S2350 S2380 S2189 S2645 S2668 S2097 S2279 S2470 S2650 S2510 S2365 S2767 S2153 S2400 S2508 S2405 S2338 S2770A S2688 S2356 S2261 S2731 S2125 S2583

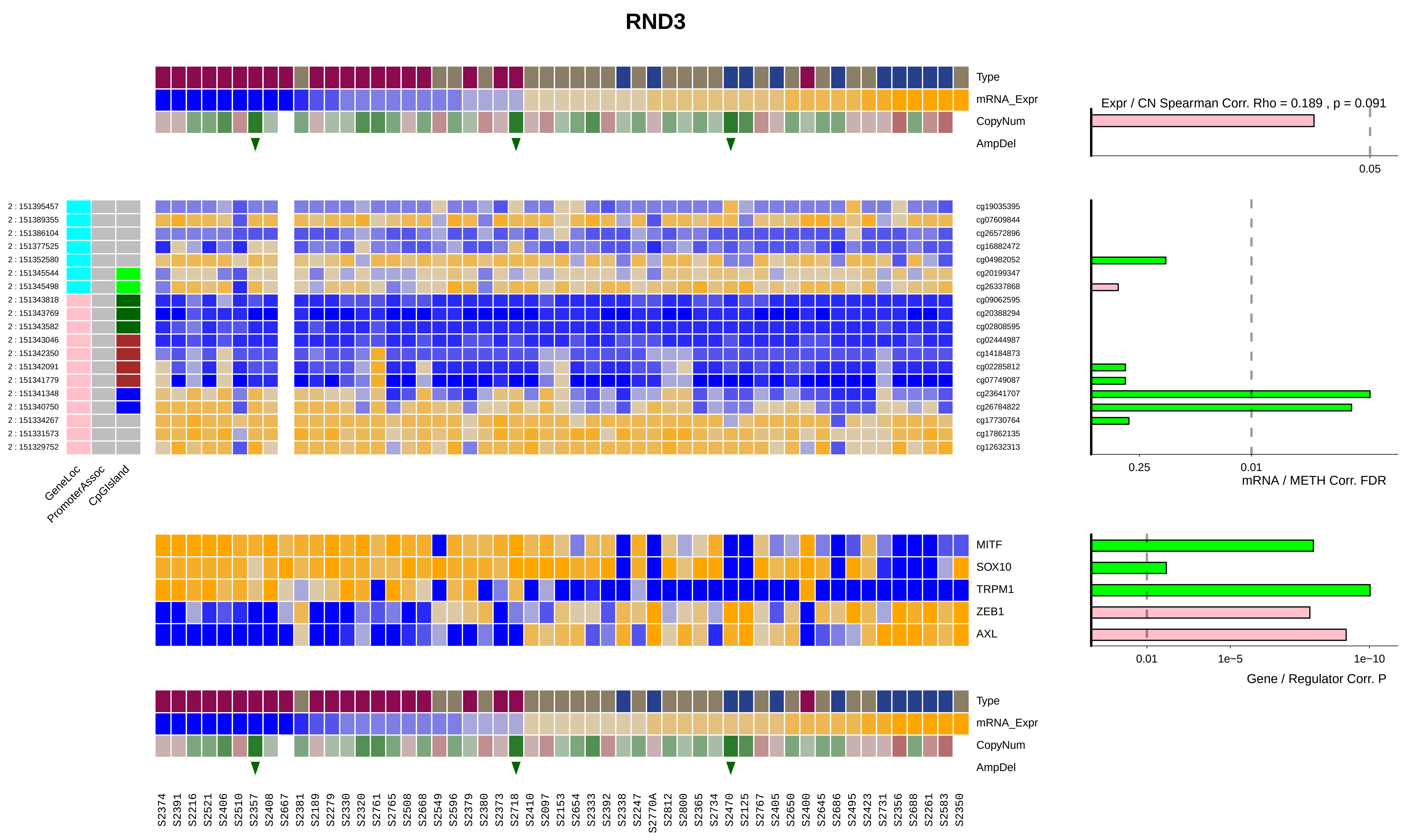
RND3
Type
mRNA_Expr
CopyNum
AmpDel
Expr / CN Spearman Corr. Rho = 0.189 , p = 0.091
0.05
2 : 151395457
2 : 151389355
2 : 151386104
2 : 151377525
2 : 151352580
2 : 151345544
2 : 151345498
2 : 151343818
2 : 151343769
2 : 151343582
2 : 151343046
2 : 151342350
2 : 151342091
2 : 151341779
2 : 151341348
2 : 151340750
2 : 151334267
2 : 151331573
2 : 151329752
cg19035395
cg07609844
cg26572896
cg16882472
cg04982052
cg20199347
cg26337868
cg09062595
cg20388294
cg02808595
cg02444987
cg14184873
cg02285812
cg07749087
cg23641707
cg26784822
cg17730764
cg17862135
cg12632313
GeneLoc
PromoterAssoc
CpGIsland
0.25
0.01
mRNA / METH Corr. FDR
MITF
SOX10
TRPM1
ZEB1
AXL
0.01
1e−5
1e−10
Gene / Regulator Corr. P
Type
mRNA_Expr
CopyNum
AmpDel
S2374 S2391 S2216 S2521 S2406 S2510 S2357 S2408 S2667 S2381 S2189 S2279 S2330 S2320 S2761 S2765 S2508 S2668 S2549 S2596 S2379 S2380 S2373 S2718 S2410 S2097 S2153 S2654 S2333 S2392 S2338 S2247 S2770A S2812 S2800 S2365 S2734 S2470 S2125 S2767 S2405 S2650 S2400 S2645 S2686 S2495 S2423 S2731 S2356 S2688 S2261 S2583 S2350

CCL2

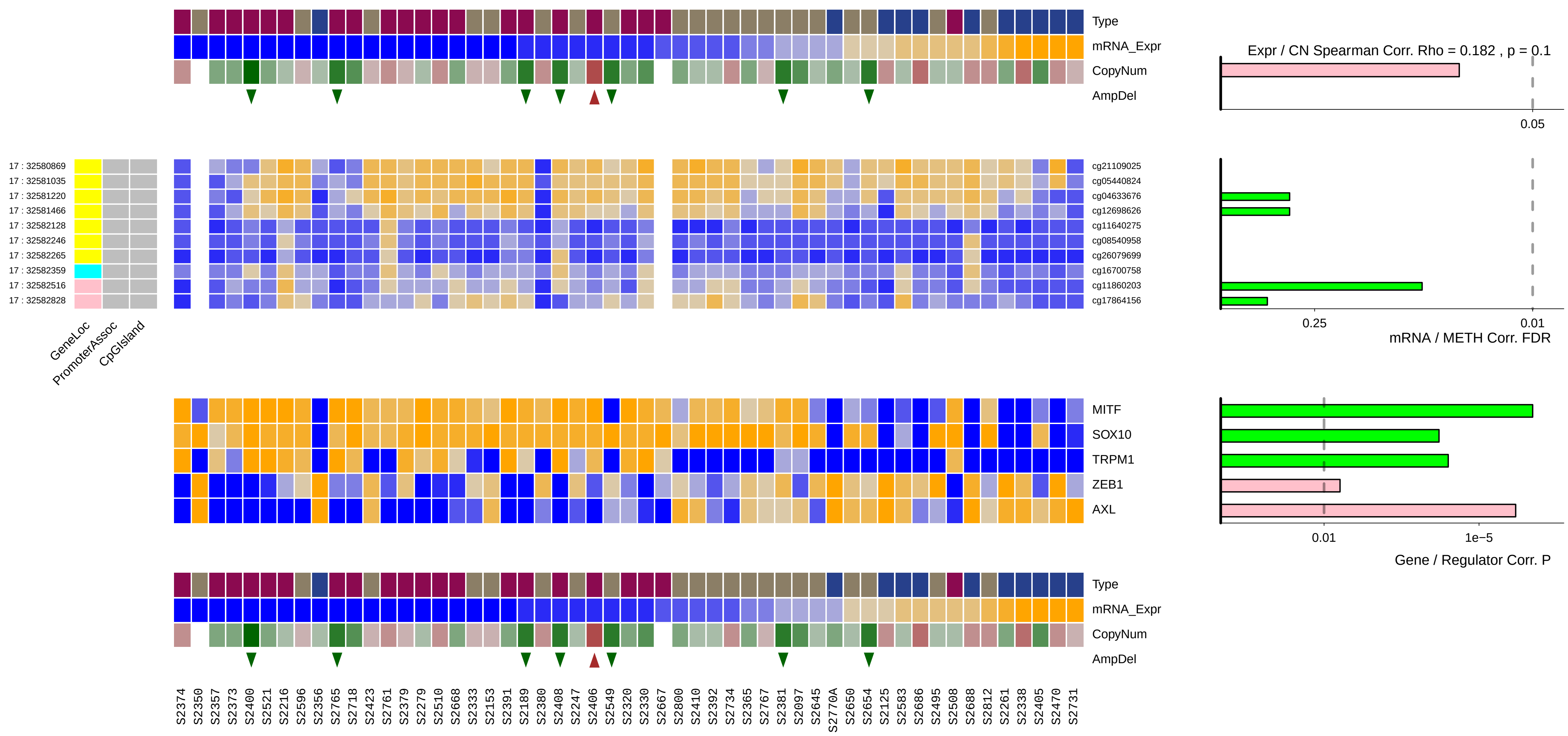
Type
mRNA_Expr
CopyNum
AmpDel
Expr / CN Spearman Corr. Rho = 0.182 , p = 0.1
0.05
17 : 32580869
17 : 32581035
17 : 32581220
17 : 32581466
17 : 32582128
17 : 32582246
17 : 32582265
17 : 32582359
17 : 32582516
17 : 32582828
GeneLoc
PromoterAssoc
CpGIsland
cg21109025
cg05440824
cg04633676
cg12698626
cg11640275
cg08540958
cg26079699
cg16700758
cg11860203
cg17864156
0.25
0.01
mRNA / METH Corr. FDR
MITF
SOX10
TRPM1
ZEB1
AXL
0.01
1e−5
Gene / Regulator Corr. P
Type
mRNA_Expr
CopyNum
AmpDel
S2374 S2350 S2357 S2373 S2400 S2521 S2216 S2596 S2356 S2765 S2718 S2423 S2761 S2379 S2279 S2510 S2668 S2333 S2153 S2391 S2189 S2380 S2408 S2247 S2406 S2549 S2320 S2330 S2667 S2800 S2410 S2392 S2734 S2365 S2767 S2381 S2097 S2645 S2770A S2650 S2654 S2125 S2583 S2686 S2495 S2508 S2688 S2812 S2261 S2338 S2405 S2470 S2731

OSMR

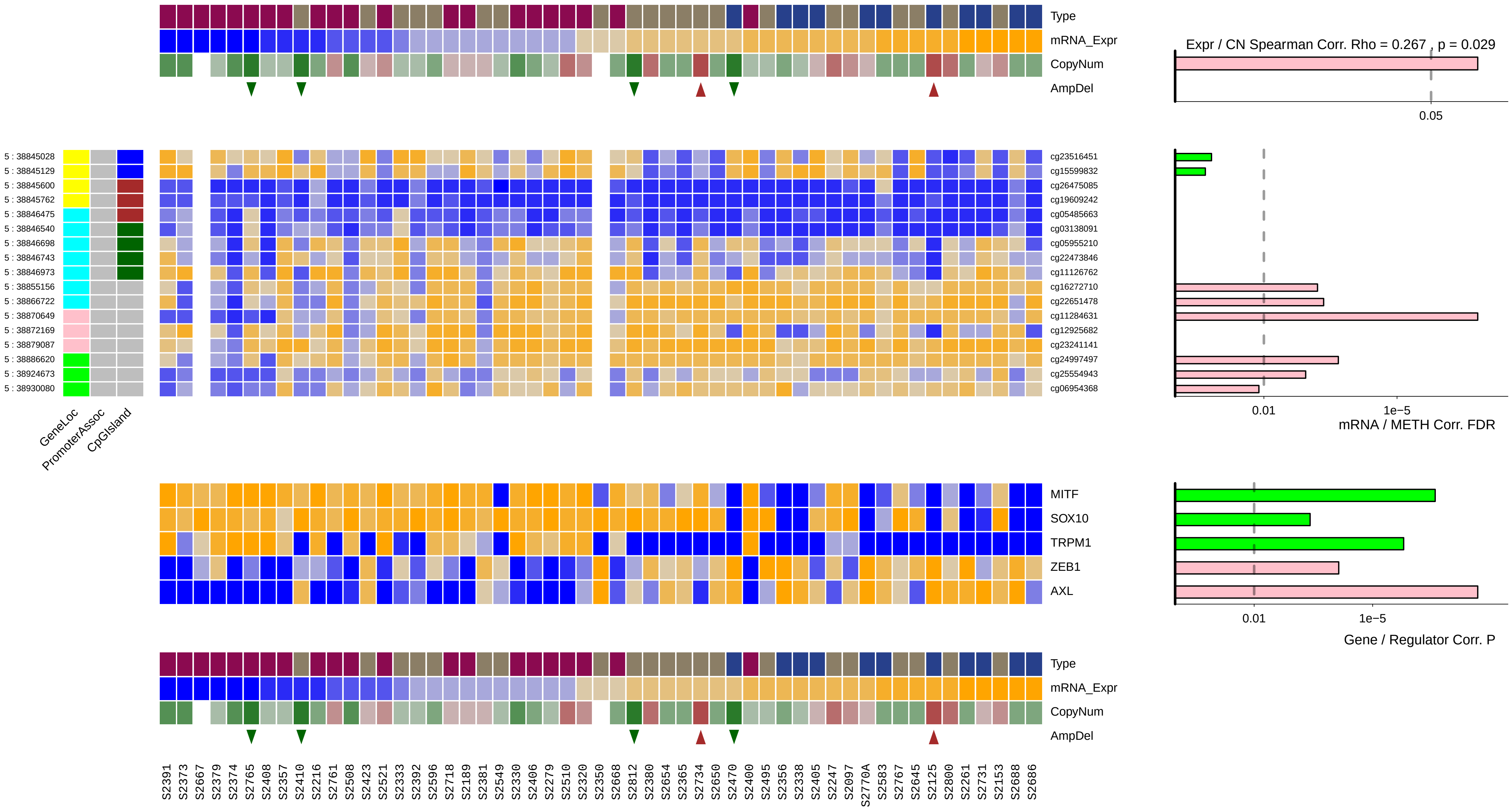
Type
mRNA_Expr
CopyNum
AmpDel
Expr / CN Spearman Corr. Rho = 0.267 , p = 0.029
0.05
5 : 38845028
5 : 38845129
5 : 38845600
5 : 38845762
5 : 38846475
5 : 38846540
5 : 38846698
5 : 38846743
5 : 38846973
5 : 38855156
5 : 38866722
5 : 38870649
5 : 38872169
5 : 38879087
5 : 38886620
5 : 38924673
5 : 38930080
GeneLoc
PromoterAssoc
CpGIsland
cg23516451
cg15599832
cg26475085
cg19609242
cg05485663
cg03138091
cg05955210
cg22473846
cg11126762
cg16272710
cg22651478
cg11284631
cg12925682
cg23241141
cg24997497
cg25554943
cg06954368
0.01
1e−5
mRNA / METH Corr. FDR
MITF
SOX10
TRPM1
ZEB1
AXL
0.01
1e−5
Gene / Regulator Corr. P
Type
mRNA_Expr
CopyNum
AmpDel
S2391
S2373
S2667
S2379
S2374
S2765
S2408
S2357
S2410
S2216
S2761
S2508
S2423
S2521
S2333
S2392
S2596
S2718
S2189
S2381
S2549
S2330
S2406
S2279
S2510
S2320
S2350
S2668
S2812
S2380
S2654
S2365
S2734
S2650
S2470
S2400
S2495
S2356
S2338
S2405
S2247
S2097
S2770A
S2583
S2767
S2645
S2125
S2800
S2261
S2731
S2153
S2688
S2686

EPHB2

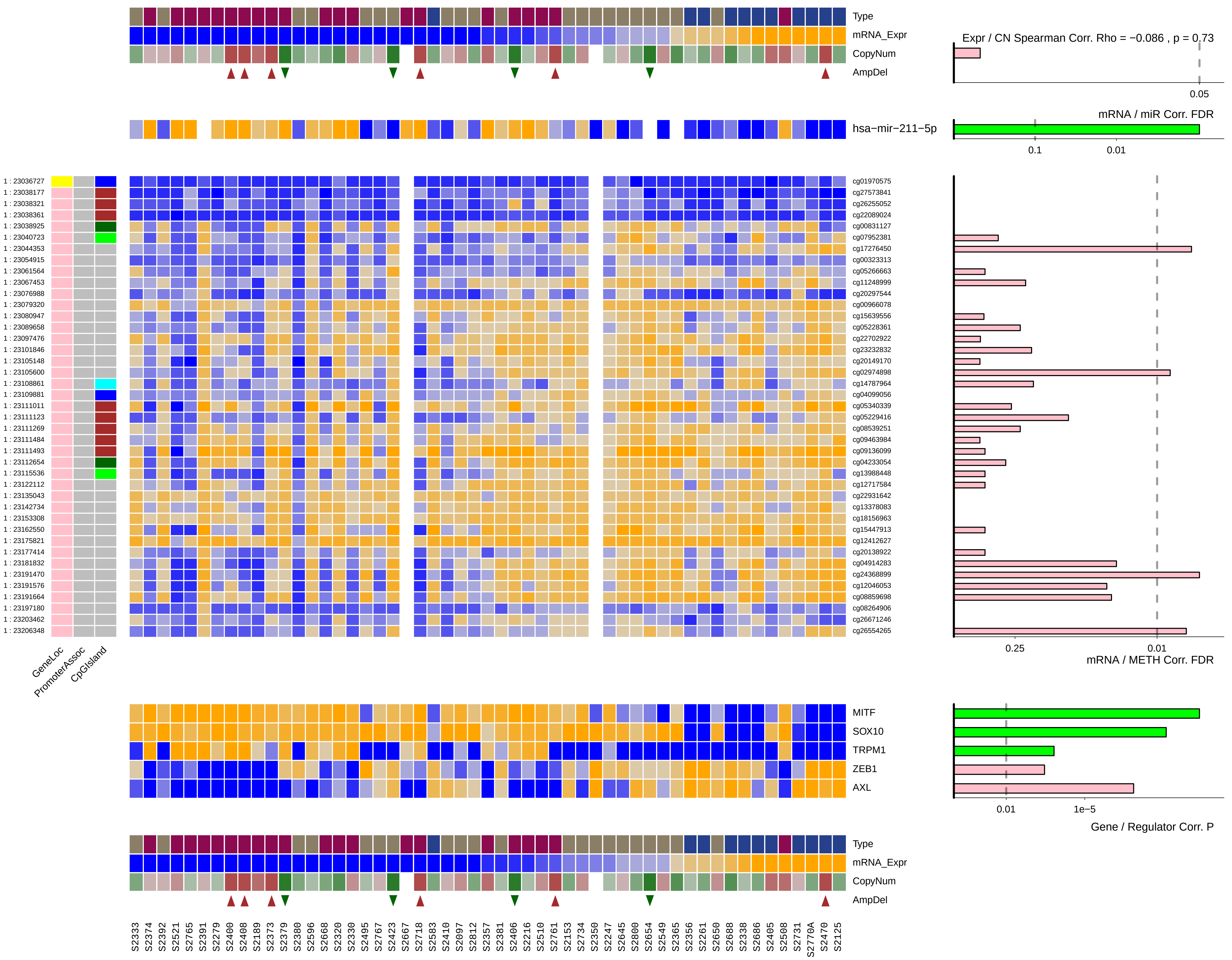

Type
mRNA_Expr
CopyNum
AmpDel
Expr / CN Spearman Corr. Rho = −0.086 , p = 0.73
0.05
hsa−mir−211−5p
mRNA / miR Corr. FDR
0.1
0.01
GeneLoc
PromoterAssoc
CpGIsland
0.25
0.01
mRNA / METH Corr. FDR
MITF
SOX10
TRPM1
ZEB1
AXL
0.01
1e−5
Gene / Regulator Corr. P
S2333
S2374
S2392
S2521
S2765
S2391
S2279
S2400
S2408
S2189
S2373
S2379
S2380
S2596
S2668
S2320
S2330
S2495
S2767
S2423
S2667
S2718
S2583
S2410
S2097
S2812
S2357
S2381
S2406
S2216
S2510
S2761
S2153
S2734
S2350
S2247
S2645
S2800
S2654
S2549
S2365
S2356
S2261
S2650
S2688
S2338
S2686
S2405
S2508
S2731
S2770A
S2470
S2125

TFPI

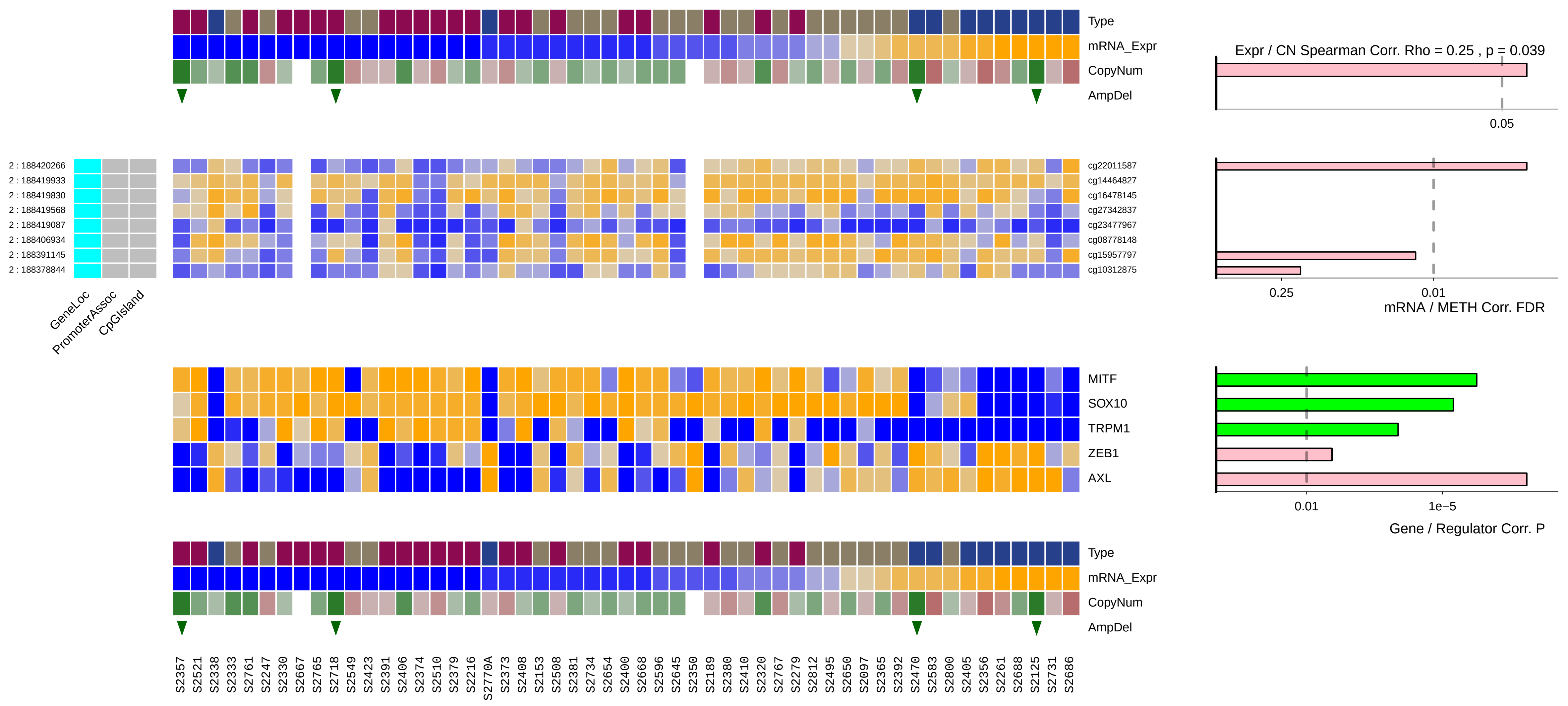

Type
mRNA_Expr
CopyNum
AmpDel
Expr / CN Spearman Corr. Rho = 0.25 , p = 0.039
0.05
2 : 188420266
2 : 188419933
2 : 188419830
2 : 188419568
2 : 188419087
2 : 188406934
2 : 188391145
2 : 188378844
GeneLoc
PromoterAssoc
CpGIsland
cg22011587
cg14464827
cg16478145
cg27342837
cg23477967
cg08778148
cg15957797
cg10312875
0.25
0.01
mRNA / METH Corr. FDR
MITF
SOX10
TRPM1
ZEB1
AXL
0.01
1e−5
Gene / Regulator Corr. P
S2357 S2521 S2338 S2333 S2761 S2247 S2330 S2667 S2765 S2718 S2549 S2423 S2391 S2406 S2374 S2510 S2379 S2216 S2770A S2373 S2408 S2153 S2508 S2381 S2734 S2654 S2400 S2668 S2596 S2645 S2350 S2189 S2380 S2410 S2320 S2767 S2279 S2812 S2495 S2650 S2097 S2365 S2392 S2470 S2583 S2800 S2405 S2356 S2261 S2688 S2125 S2731 S2686

NTM

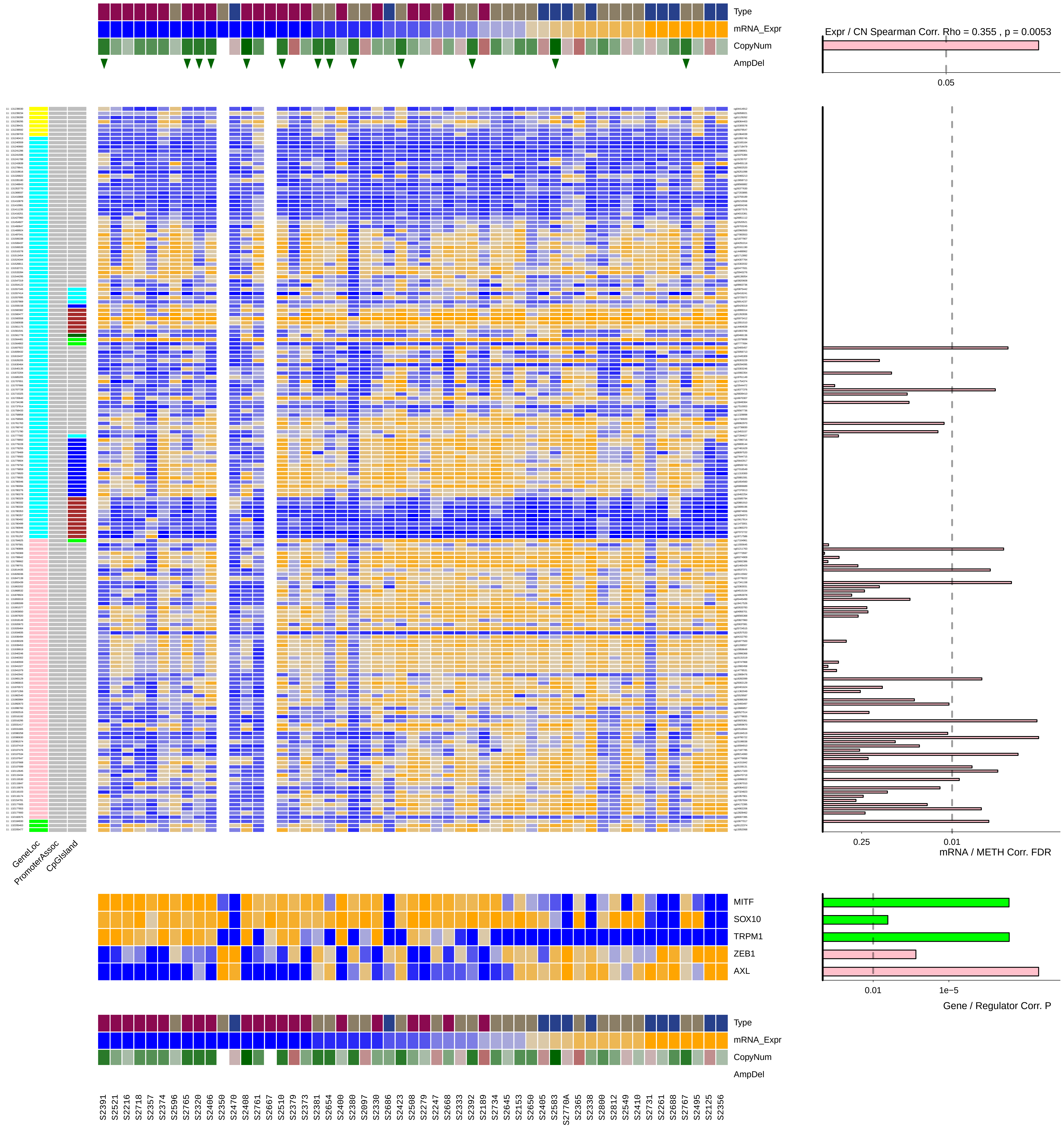

Type
mRNA_Expr
CopyNum
AmpDel
Expr / CN Spearman Corr. Rho = 0.355 , p = 0.0053
0.05
GeneLoc
PromoterAssoc
CpGIsland
0.25
0.01
mRNA / METH Corr. FDR
MITF
SOX10
TRPM1
ZEB1
AXL
0.01
1e−5
Gene / Regulator Corr. P
S2391 S2521 S2216 S2718 S2357 S2374 S2596 S2765 S2320 S2406 S2350 S2470 S2408 S2761 S2667 S2510 S2379 S2373 S2381 S2654 S2400 S2380 S2097 S2330 S2686 S2423 S2508 S2279 S2247 S2668 S2333 S2392 S2189 S2734 S2645 S2153 S2650 S2405 S2583 S2770A S2365 S2338 S2800 S2812 S2549 S2410 S2731 S2261 S2688 S2767 S2495 S2125 S2356

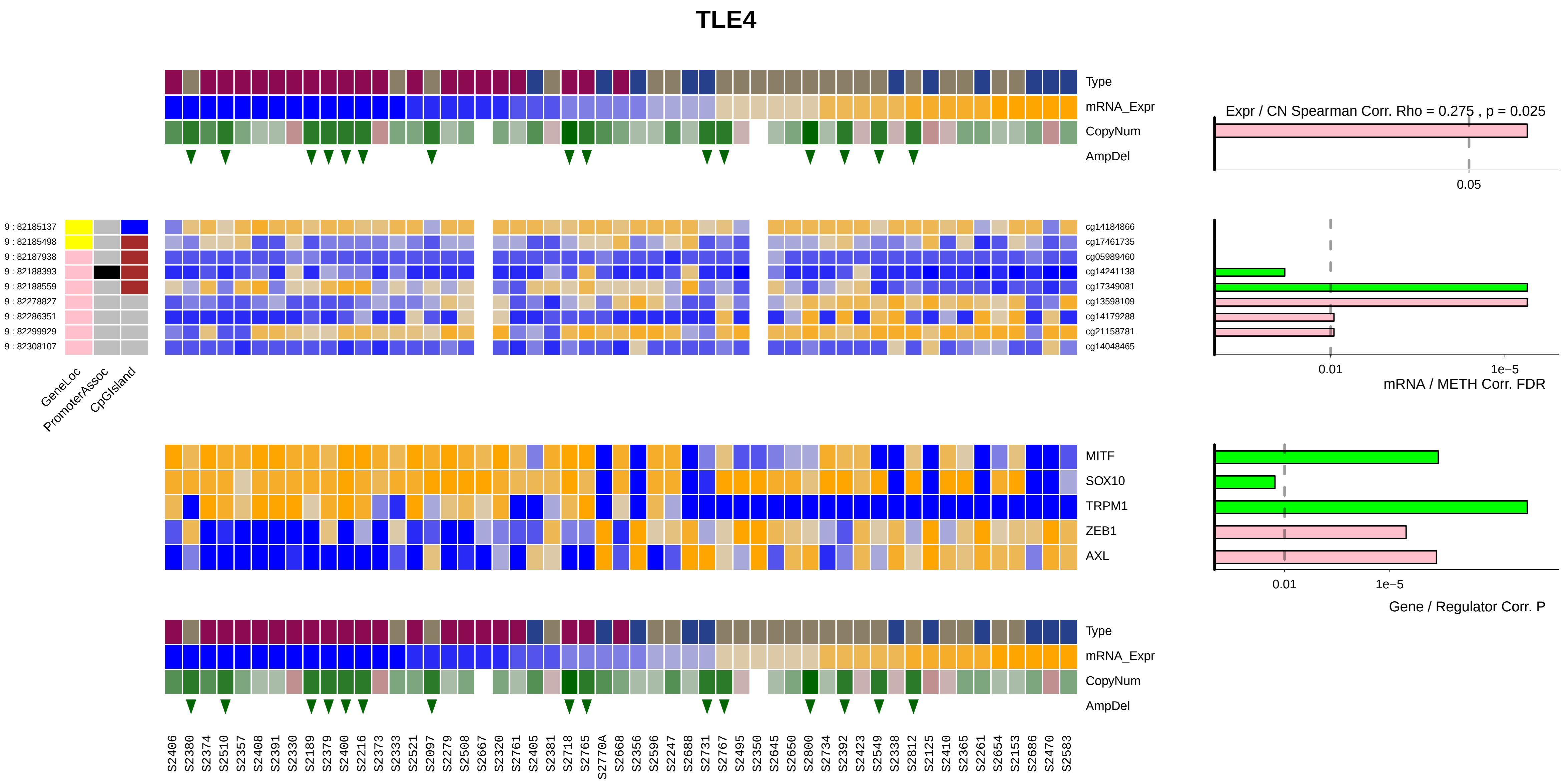
TLE4
Type
mRNA_Expr
CopyNum
AmpDel
Expr / CN Spearman Corr. Rho = 0.275 , p = 0.025
0.05
9 : 82185137
9 : 82185498
9 : 82187938
9 : 82188393
9 : 82188559
9 : 82278827
9 : 82286351
9 : 82299929
9 : 82308107
GeneLoc
PromoterAssoc
CpGIsland
cg14184866
cg17461735
cg05989460
cg14241138
cg17349081
cg13598109
cg14179288
cg21158781
cg14048465
0.01
1e−5
mRNA / METH Corr. FDR
MITF
SOX10
TRPM1
ZEB1
AXL
Gene / Regulator Corr. P
S2406 S2380 S2374 S2510 S2357 S2408 S2391 S2330 S2189 S2379 S2400 S2216 S2373 S2333 S2521 S2097 S2279 S2508 S2667 S2320 S2761 S2405 S2381 S2718 S2765 S2770A S2668 S2356 S2596 S2247 S2688 S2731 S2767 S2495 S2350 S2645 S2650 S2800 S2734 S2392 S2423 S2549 S2338 S2812 S2125 S2410 S2365 S2261 S2654 S2153 S2686 S2470 S2583

COL5A1

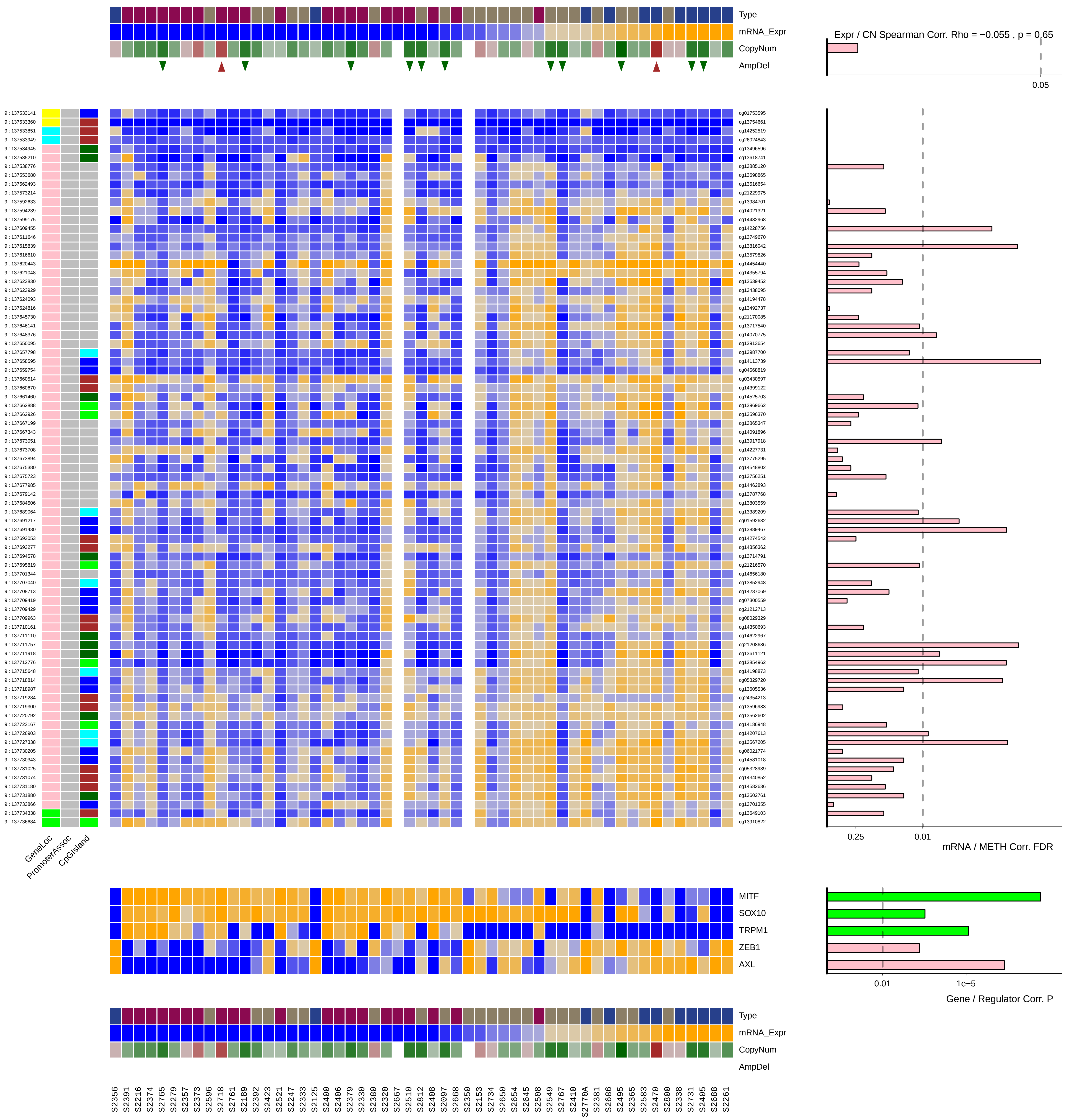
Type
mRNA_Expr
CopyNum
AmpDel
Expr / CN Spearman Corr. Rho = −0.055 , p = 0.65
0.05
GeneLoc
PromoterAssoc
CpGIsland
0.25
0.01
mRNA / METH Corr. FDR
MITF
SOX10
TRPM1
ZEB1
AXL
0.01
1e−5
Gene / Regulator Corr. P
Type
mRNA_Expr
CopyNum
AmpDel
S2356 S2391 S2216 S2374 S2765 S2279 S2357 S2373 S2596 S2718 S2761 S2189 S2392 S2423 S2521 S2247 S2333 S2125 S2400 S2406 S2379 S2330 S2380 S2320 S2667 S2510 S2812 S2408 S2097 S2668 S2350 S2153 S2734 S2650 S2654 S2645 S2508 S2549 S2767 S2410 S2770A S2381 S2686 S2495 S2365 S2583 S2470 S2800 S2338 S2731 S2405 S2688 S2261

C12ORF75

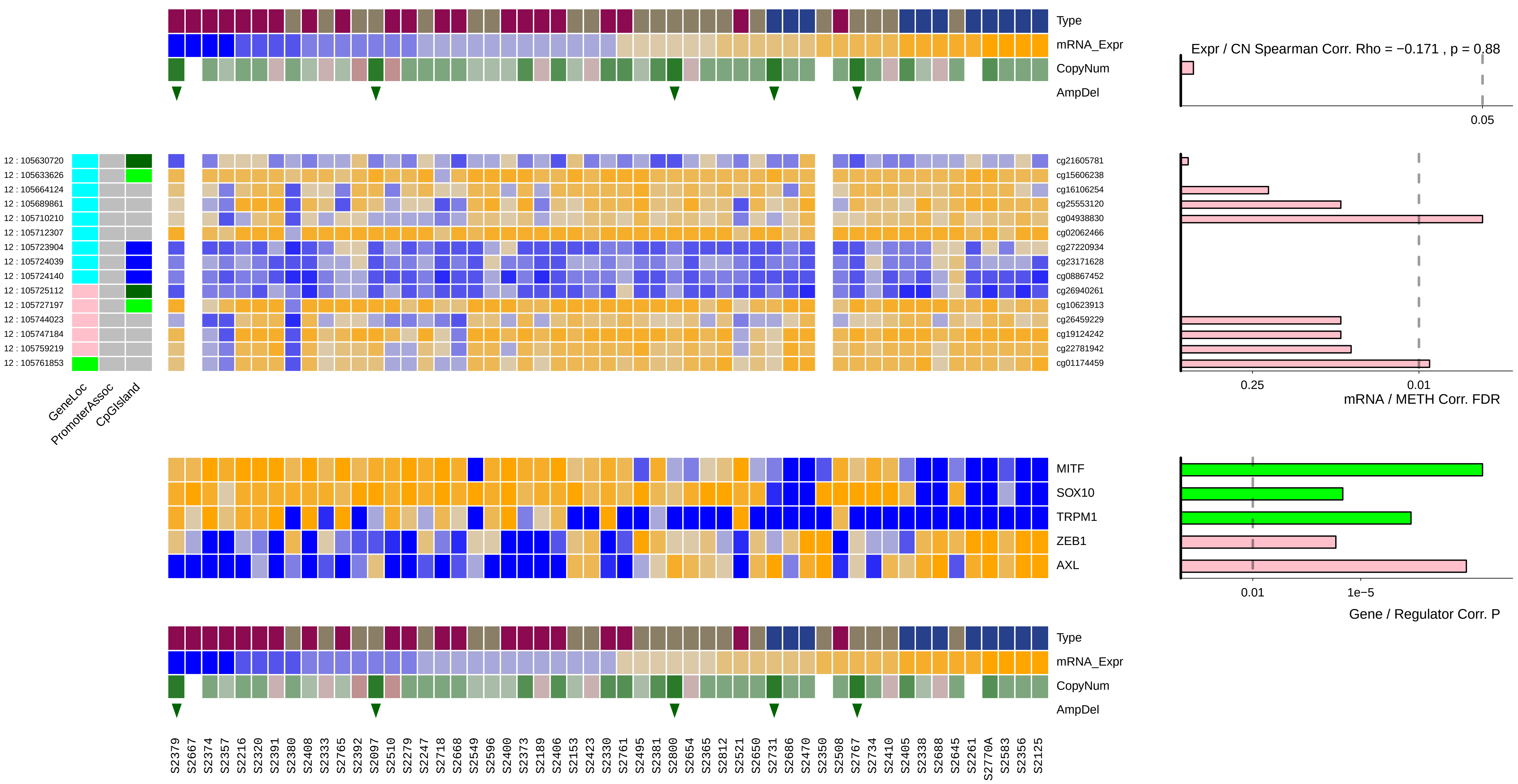
Type
mRNA_Expr
CopyNum
AmpDel
Expr / CN Spearman Corr. Rho = −0.171 , p = 0.88
0.05
12 : 105630720
12 : 105633626
12 : 105664124
12 : 105689861
12 : 105710210
12 : 105712307
12 : 105723904
12 : 105724039
12 : 105724140
12 : 105725112
12 : 105727197
12 : 105744023
12 : 105747184
12 : 105759219
12 : 105761853
GeneLoc
PromoterAssoc
CpGIsland
cg21605781
cg15606238
cg16106254
cg25553120
cg04938830
cg02062466
cg27220934
cg23171628
cg08867452
cg26940261
cg10623913
cg26459229
cg19124242
cg22781942
cg01174459
0.25
0.01
mRNA / METH Corr. FDR
MITF
SOX10
TRPM1
ZEB1
AXL
0.01
1e−5
Gene / Regulator Corr. P
Type
mRNA_Expr
CopyNum
AmpDel
S2379 S2667 S2374 S2357 S2216 S2320 S2391 S2380 S2408 S2333 S2765 S2392 S2097 S2510 S2279 S2247 S2718 S2668 S2549 S2596 S2400 S2373 S2189 S2406 S2153 S2423 S2330 S2761 S2495 S2381 S2800 S2654 S2365 S2812 S2521 S2650 S2731 S2686 S2470 S2350 S2508 S2767 S2734 S2410 S2405 S2338 S2688 S2645 S2261 S2770A S2583 S2356 S2125

RRAS2

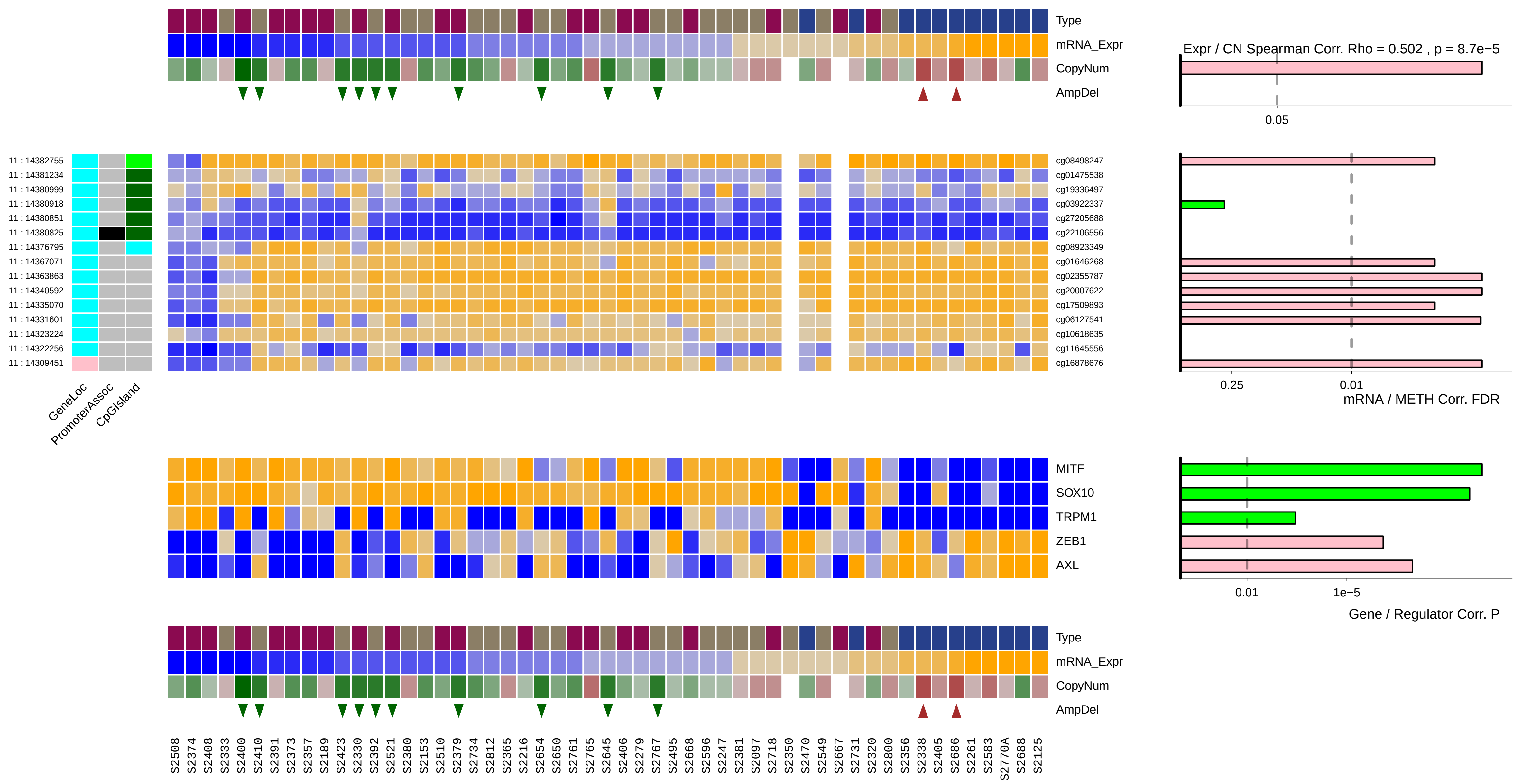
Type
mRNA_Expr
CopyNum
AmpDel
Expr / CN Spearman Corr. Rho = 0.502 , p = 8.7e−5
0.05
11 : 14382755
11 : 14381234
11 : 14380999
11 : 14380918
11 : 14380851
11 : 14380825
11 : 14376795
11 : 14367071
11 : 14363863
11 : 14340592
11 : 14335070
11 : 14331601
11 : 14323224
11 : 14322256
11 : 14309451
GeneLoc
PromoterAssoc
CpGIsland
cg08498247
cg01475538
cg19336497
cg03922337
cg27205688
cg22106556
cg08923349
cg01646268
cg02355787
cg20007622
cg17509893
cg06127541
cg10618635
cg11645556
cg16878676
0.25
0.01
mRNA / METH Corr. FDR
MITF
SOX10
TRPM1
ZEB1
AXL
0.01
1e−5
Gene / Regulator Corr. P
S2508 S2374 S2408 S2333 S2400 S2410 S2391 S2373 S2357 S2189 S2423 S2330 S2392 S2521 S2380 S2153 S2510 S2379 S2734 S2812 S2365 S2216 S2654 S2650 S2761 S2765 S2645 S2406 S2279 S2767 S2495 S2668 S2596 S2247 S2381 S2097 S2718 S2350 S2470 S2549 S2667 S2731 S2320 S2800 S2356 S2338 S2405 S2686 S2261 S2583 S2770A S2688 S2125

MYLK

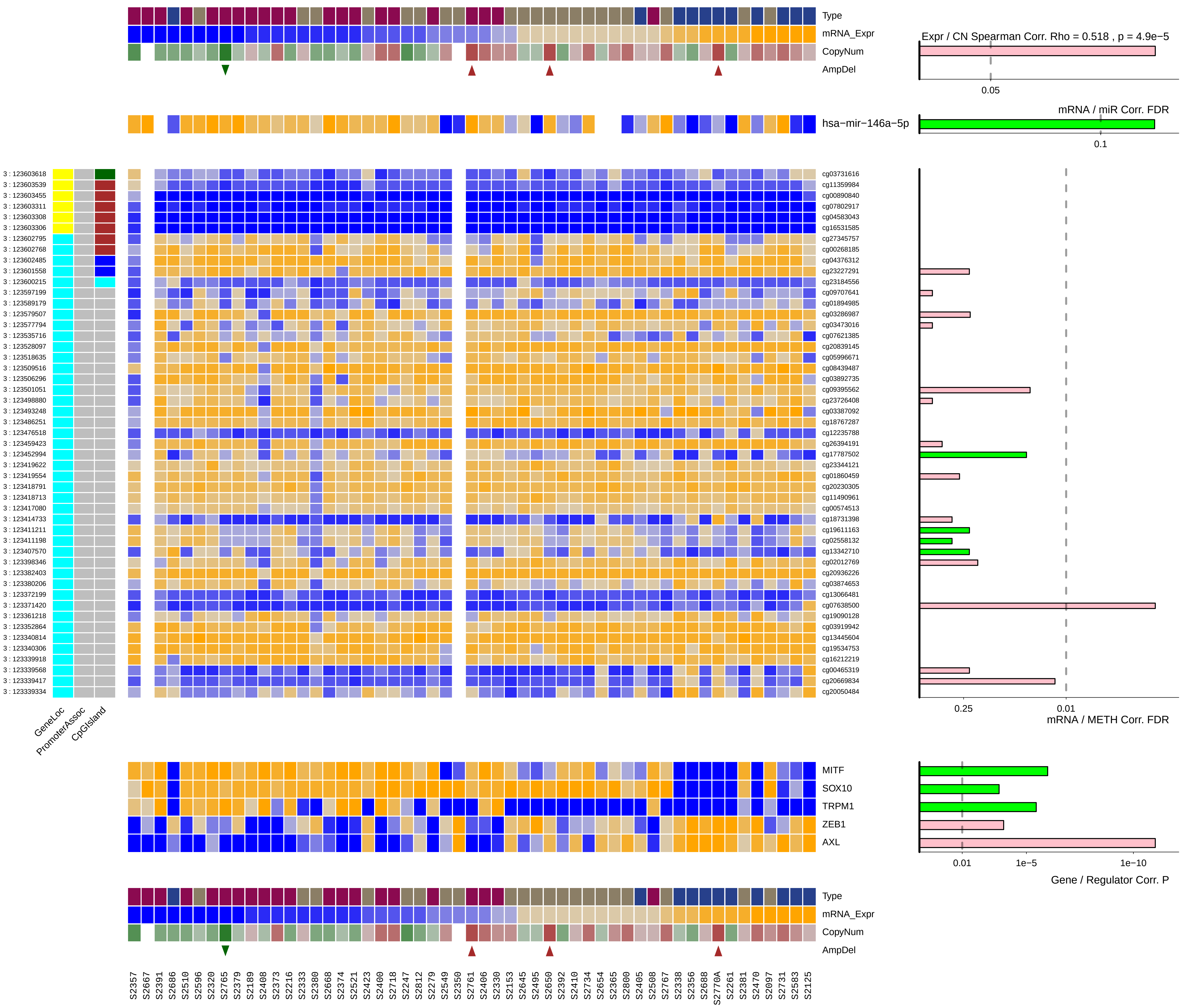

Type
mRNA_Expr
CopyNum
AmpDel
Expr / CN Spearman Corr. Rho = 0.518 , p = 4.9e−5
0.05
hsa−mir−146a−5p
mRNA / miR Corr. FDR
0.1
3 : 123603618
3 : 123603539
3 : 123603455
3 : 123603311
3 : 123603308
3 : 123603306
3 : 123602795
3 : 123602768
3 : 123602485
3 : 123601558
3 : 123600215
3 : 123597199
3 : 123589179
3 : 123579507
3 : 123577794
3 : 123535716
3 : 123528097
3 : 123518635
3 : 123509516
3 : 123506296
3 : 123501051
3 : 123498880
3 : 123493248
3 : 123486251
3 : 123476518
3 : 123459423
3 : 123452994
3 : 123419622
3 : 123419554
3 : 123418791
3 : 123418713
3 : 123417080
3 : 123414733
3 : 123411211
3 : 123411198
3 : 123407570
3 : 123398346
3 : 123382403
3 : 123380206
3 : 123372199
3 : 123371420
3 : 123361218
3 : 123352864
3 : 123340814
3 : 123340306
3 : 123339918
3 : 123339568
3 : 123339417
3 : 123339334
cg03731616
cg11359984
cg00890840
cg07802917
cg04583043
cg16531585
cg27345757
cg00268185
cg04376312
cg23227291
cg23184556
cg09707641
cg01894985
cg03286987
cg03473016
cg07621385
cg20839145
cg05996671
cg08439487
cg03892735
cg09395562
cg23726408
cg03387092
cg18767287
cg12235788
cg26394191
cg17787502
cg23344121
cg01860459
cg20230305
cg11490961
cg00574513
cg18731398
cg19611163
cg02558132
cg13342710
cg02012769
cg20936226
cg03874653
cg13066481
cg07638500
cg19090128
cg03919942
cg13445604
cg19534753
cg16212219
cg00465319
cg20669834
cg20050484
0.25
0.01
mRNA / METH Corr. FDR
GeneLoc
PromoterAssoc
CpGIsland
MITF
SOX10
TRPM1
ZEB1
AXL
0.01
1e−5
1e−10
Gene / Regulator Corr. P
Type
mRNA_Expr
CopyNum
AmpDel
S2357
S2667
S2391
S2686
S2510
S2596
S2320
S2765
S2379
S2189
S2408
S2373
S2216
S2333
S2380
S2668
S2374
S2521
S2423
S2400
S2718
S2247
S2812
S2279
S2549
S2350
S2761
S2406
S2330
S2153
S2645
S2495
S2650
S2392
S2410
S2734
S2654
S2365
S2800
S2405
S2508
S2767
S2338
S2356
S2688
S2770A
S2261
S2381
S2470
S2097
S2731
S2583
S2125

CPA4

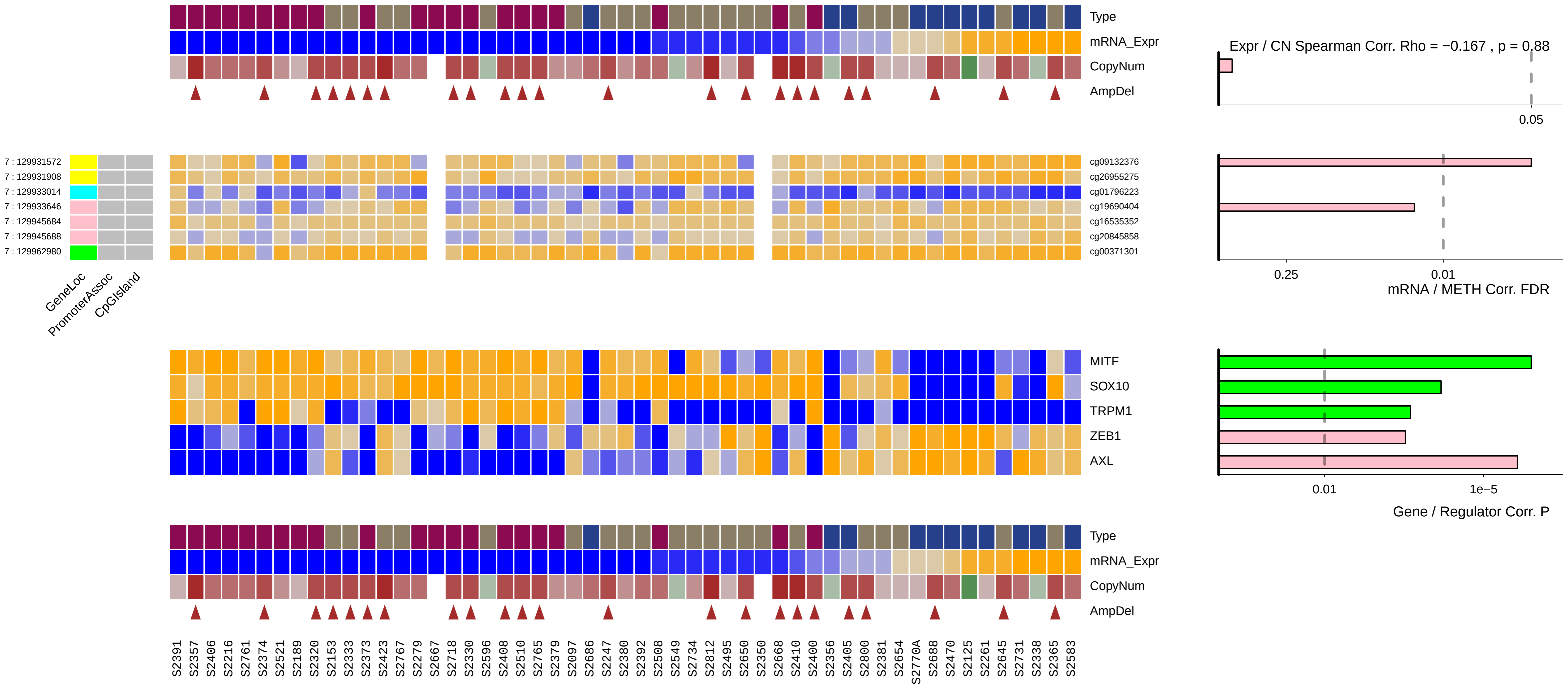
Type
mRNA_Expr
CopyNum
AmpDel
Expr / CN Spearman Corr. Rho = −0.167 , p = 0.88
0.05
7 : 129931572
7 : 129931908
7 : 129933014
7 : 129933646
7 : 129945684
7 : 129945688
7 : 129962980
GeneLoc
PromoterAssoc
CpGIsland
cg09132376
cg26955275
cg01796223
cg19690404
cg16535352
cg20845858
cg00371301
0.25
0.01
mRNA / METH Corr. FDR
MITF
SOX10
TRPM1
ZEB1
AXL
0.01
1e−5
Gene / Regulator Corr. P
S2391 S2357 S2406 S2216 S2761 S2374 S2521 S2189 S2320 S2153 S2333 S2373 S2423 S2767 S2279 S2667 S2718 S2330 S2596 S2408 S2510 S2765 S2379 S2097 S2686 S2247 S2380 S2392 S2508 S2549 S2734 S2812 S2495 S2650 S2350 S2668 S2410 S2400 S2356 S2405 S2800 S2381 S2654 S2770A S2688 S2470 S2125 S2261 S2645 S2731 S2338 S2365 S2583

CCDC80

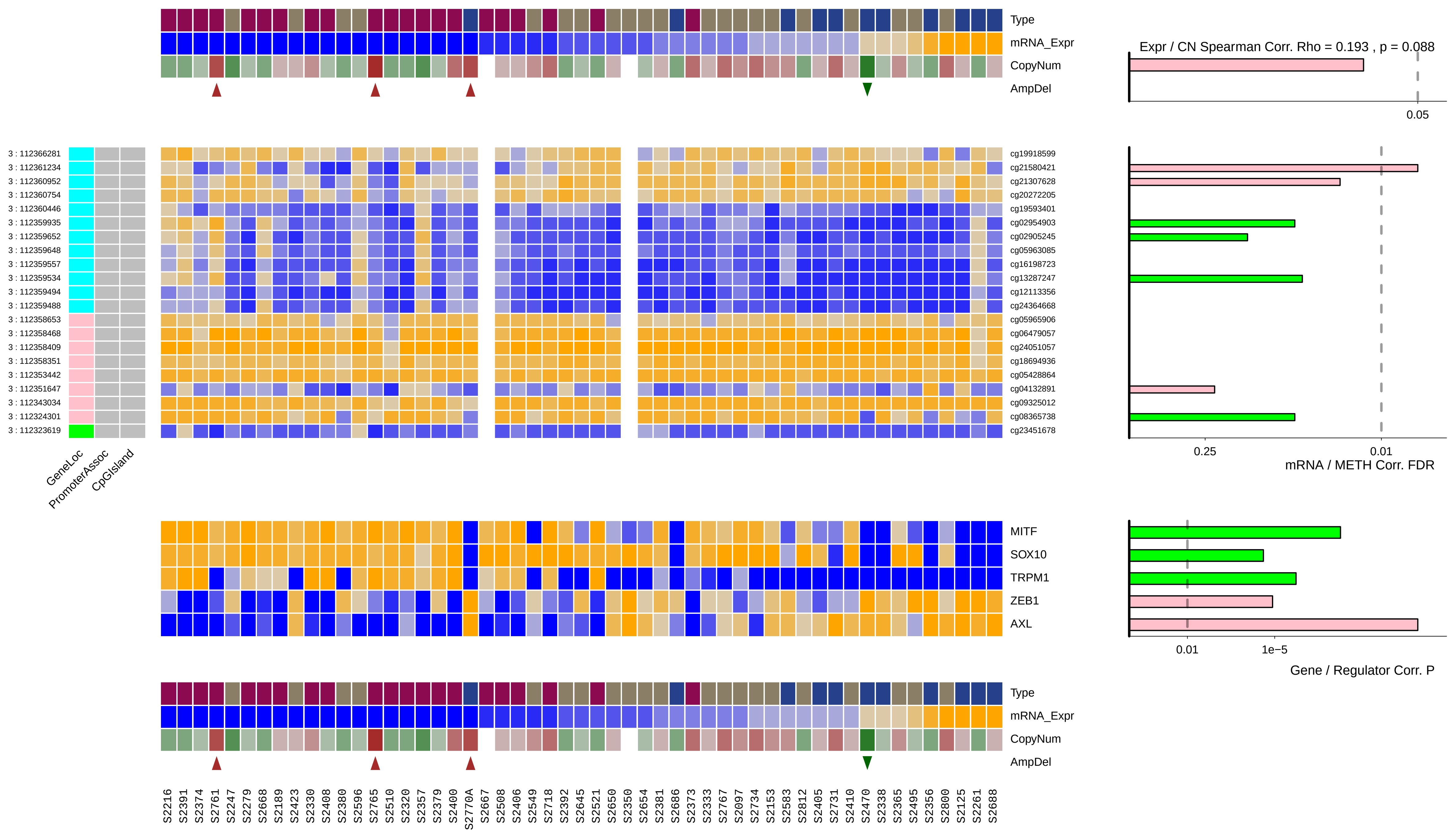

Type
mRNA_Expr
CopyNum
AmpDel
Expr / CN Spearman Corr. Rho = 0.193 , p = 0.088
0.05
GeneLoc
PromoterAssoc
CpGIsland
mRNA / METH Corr. FDR
0.25
0.01
MITF
SOX10
TRPM1
ZEB1
AXL
Gene / Regulator Corr. P
1e−5

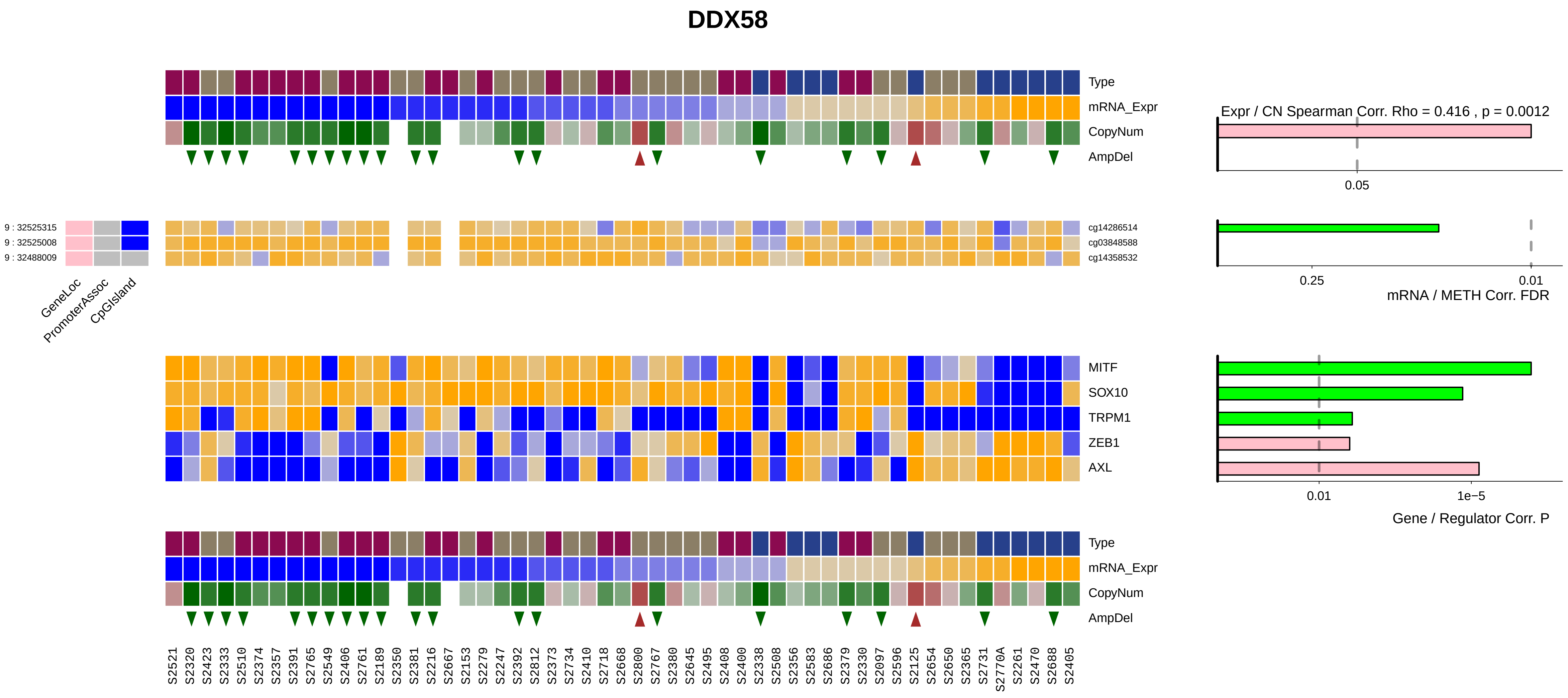

DDX58
Type
mRNA_Expr
CopyNum
AmpDel
Expr / CN Spearman Corr. Rho = 0.416 , p = 0.0012
0.05
9 : 32525315
9 : 32525008
9 : 32488009
cg14286514
cg03848588
cg14358532
GeneLoc
PromoterAssoc
CpGIsland
0.25
0.01
mRNA / METH Corr. FDR
MITF
SOX10
TRPM1
ZEB1
AXL
0.01
1e−5
Gene / Regulator Corr. P
S2521 S2320 S2423 S2333 S2510 S2374 S2357 S2391 S2765 S2549 S2406 S2761 S2189 S2350 S2381 S2216 S2667 S2153 S2279 S2247 S2392 S2812 S2373 S2734 S2410 S2718 S2668 S2800 S2767 S2380 S2645 S2495 S2408 S2400 S2338 S2508 S2356 S2583 S2686 S2379 S2330 S2097 S2596 S2125 S2654 S2650 S2365 S2731 S2770A S2261 S2470 S2688 S2405

SLC9A7

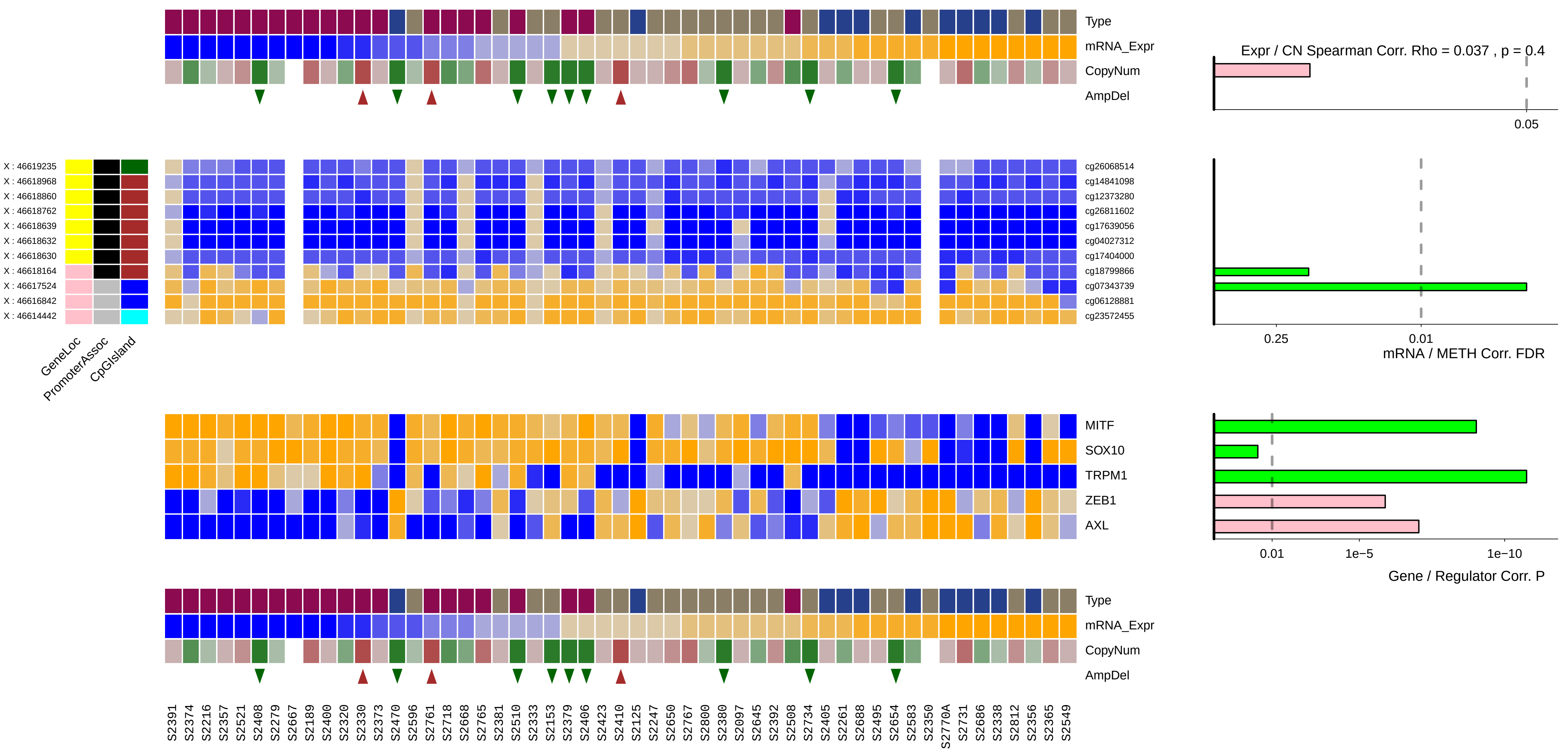

Type
mRNA_Expr
CopyNum
AmpDel
Expr / CN Spearman Corr. Rho = 0.037 , p = 0.4
0.05
X : 46619235
X : 46618968
X : 46618860
X : 46618762
X : 46618639
X : 46618632
X : 46618630
X : 46618164
X : 46617524
X : 46616842
X : 46614442
GeneLoc
PromoterAssoc
CpGIsland
cg26068514
cg14841098
cg12373280
cg26811602
cg17639056
cg04027312
cg17404000
cg18799866
cg07343739
cg06128881
cg23572455
0.25
0.01
mRNA / METH Corr. FDR
MITF
SOX10
TRPM1
ZEB1
AXL
0.01
1e−5
1e−10
Gene / Regulator Corr. P
Type
mRNA_Expr
CopyNum
AmpDel
S2391 S2374 S2216 S2357 S2521 S2408 S2279 S2667 S2189 S2400 S2320 S2330 S2373 S2470 S2596 S2761 S2718 S2668 S2765 S2381 S2510 S2333 S2153 S2379 S2406 S2423 S2410 S2125 S2247 S2650 S2767 S2800 S2380 S2097 S2645 S2392 S2508 S2734 S2405 S2261 S2688 S2495 S2654 S2583 S2350 S2770A S2731 S2686 S2338 S2812 S2356 S2365 S2549

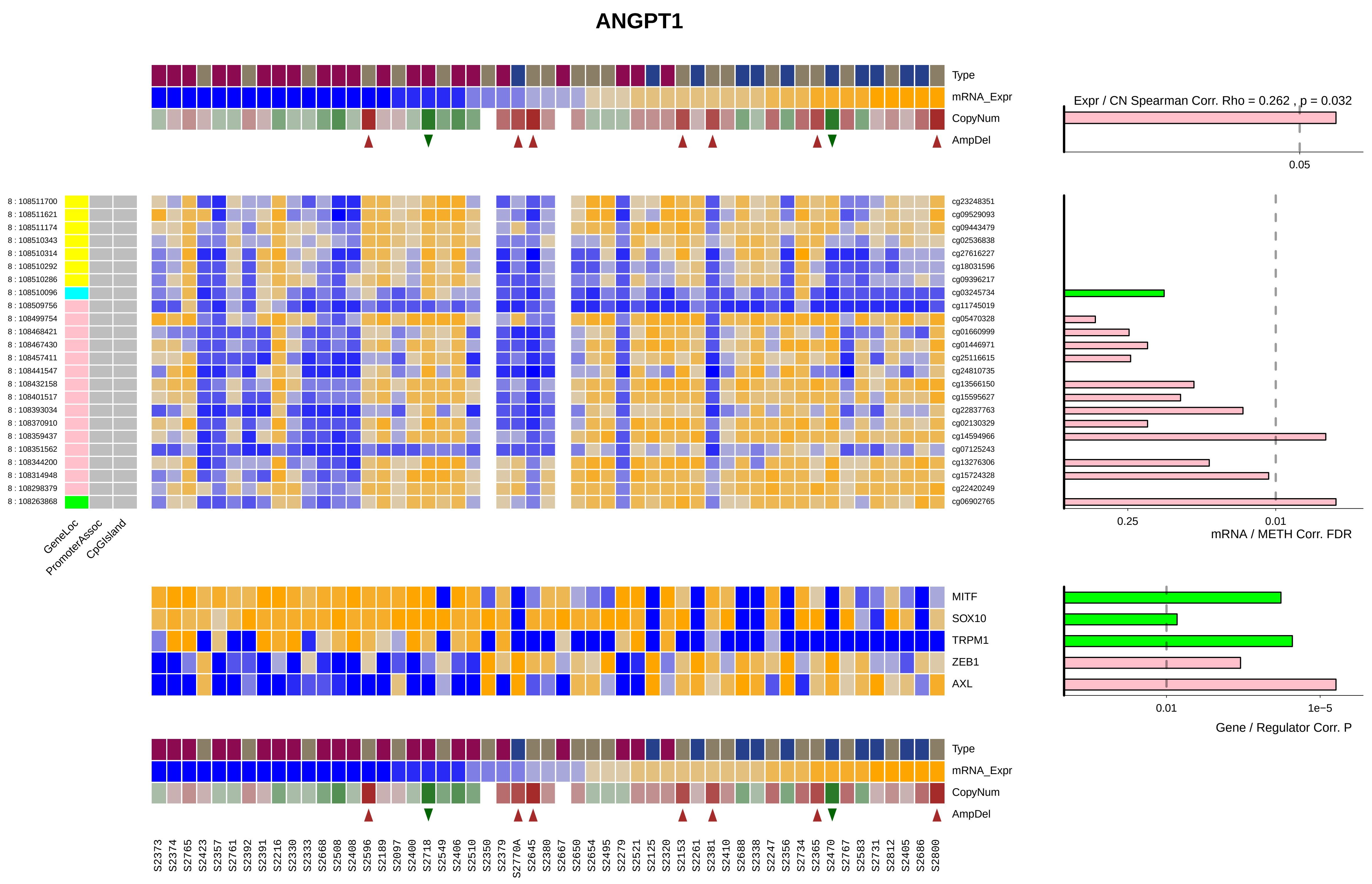
ANGPT1
Type
mRNA_Expr
CopyNum
AmpDel
Expr / CN Spearman Corr. Rho = 0.262 , p = 0.032
0.05
GeneLoc
PromoterAssoc
CpGIsland
mRNA / METH Corr. FDR
0.25
0.01
MITF
SOX10
TRPM1
ZEB1
AXL
Gene / Regulator Corr. P
1e−5

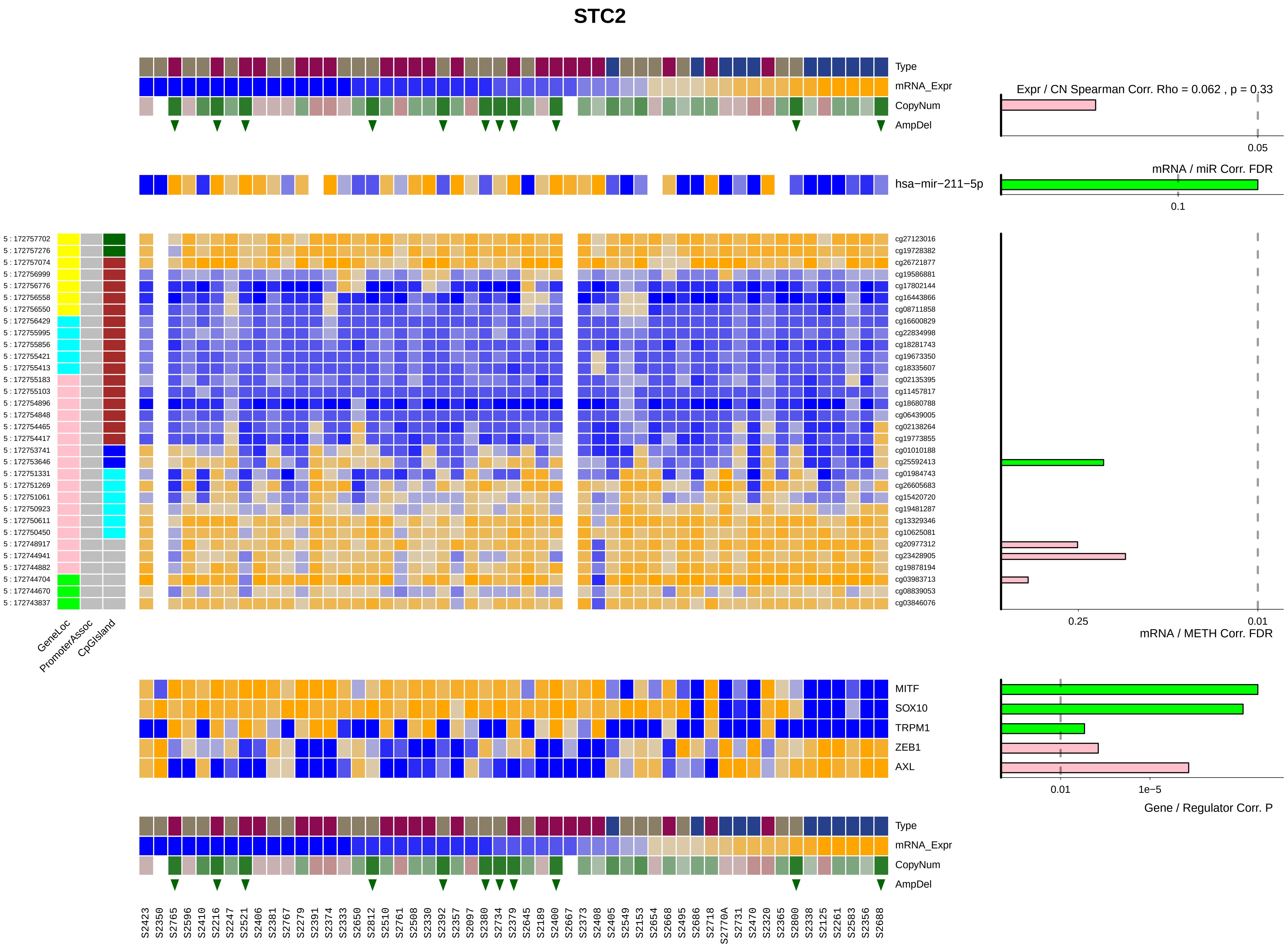
STC2
Type
mRNA_Expr
CopyNum
AmpDel
Expr / CN Spearman Corr. Rho = 0.062 , p = 0.33
0.05
mRNA / miR Corr. FDR
hsa−mir−211−5p
0.1
mRNA / METH Corr. FDR
0.25
0.01
GeneLoc
PromoterAssoc
CpGIsland
MITF
SOX10
TRPM1
ZEB1
AXL
Gene / Regulator Corr. P
1e−5

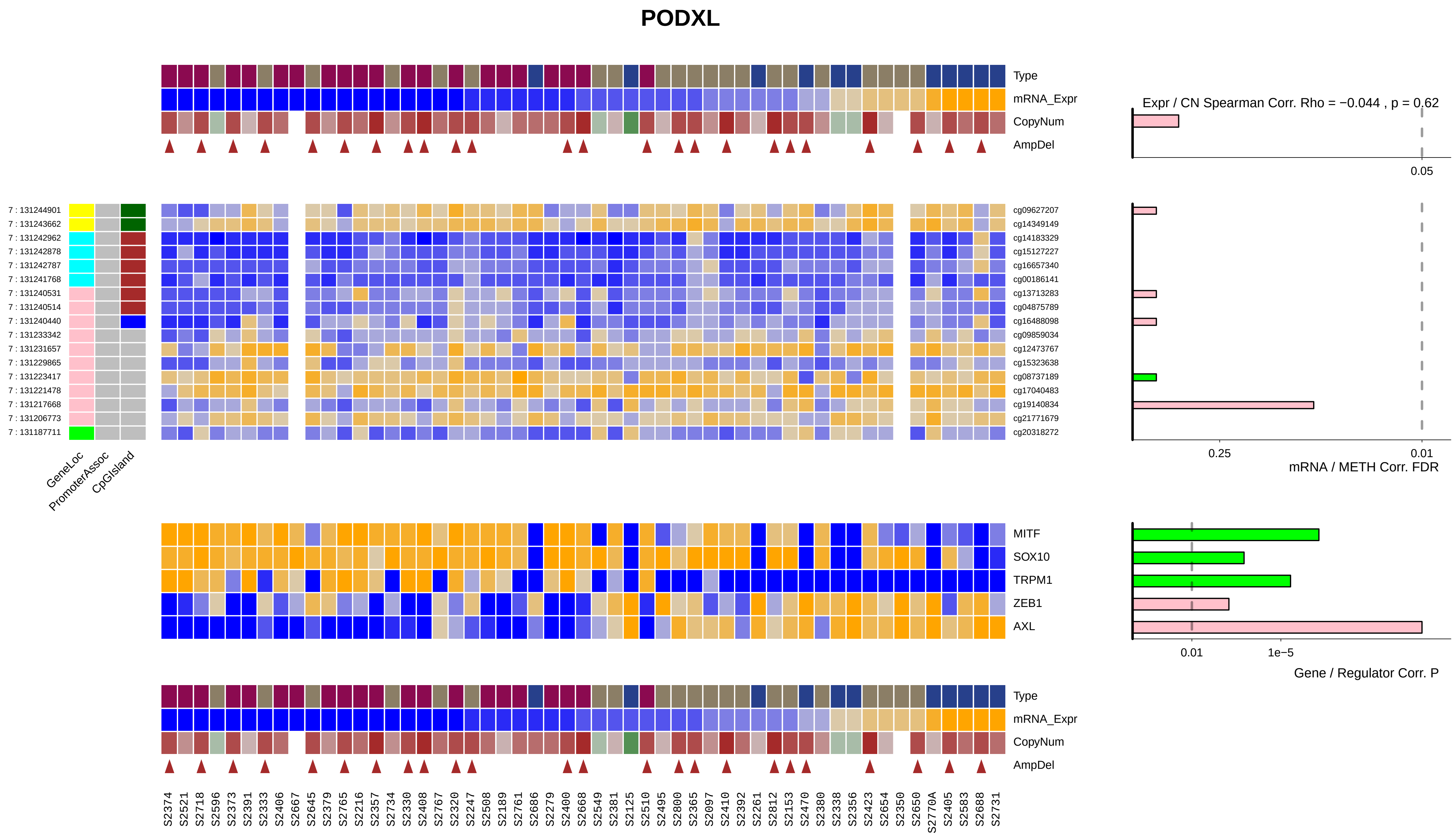
PODXL
Type
mRNA_Expr
CopyNum
AmpDel
Expr / CN Spearman Corr. Rho = −0.044 , p = 0.62
0.05
7 : 131244901
7 : 131243662
7 : 131242962
7 : 131242878
7 : 131242787
7 : 131241768
7 : 131240531
7 : 131240514
7 : 131240440
7 : 131233342
7 : 131231657
7 : 131229865
7 : 131223417
7 : 131221478
7 : 131217668
7 : 131206773
7 : 131187711
cg09627207
cg14349149
cg14183329
cg15127227
cg16657340
cg00186141
cg13713283
cg04875789
cg16488098
cg09859034
cg12473767
cg15323638
cg08737189
cg17040483
cg19140834
cg21771679
cg20318272
GeneLoc
PromoterAssoc
CpGIsland
0.25
0.01
mRNA / METH Corr. FDR
MITF
SOX10
TRPM1
ZEB1
AXL
0.01
1e−5
Gene / Regulator Corr. P
S2374 S2521 S2718 S2596 S2373 S2391 S2333 S2406 S2667 S2645 S2379 S2765 S2216 S2357 S2734 S2330 S2408 S2767 S2320 S2247 S2508 S2189 S2761 S2686 S2279 S2400 S2668 S2549 S2381 S2125 S2510 S2495 S2800 S2365 S2097 S2410 S2392 S2261 S2812 S2153 S2470 S2380 S2338 S2356 S2423 S2654 S2350 S2650 S2770A S2405 S2583 S2688 S2731

ARNTL2

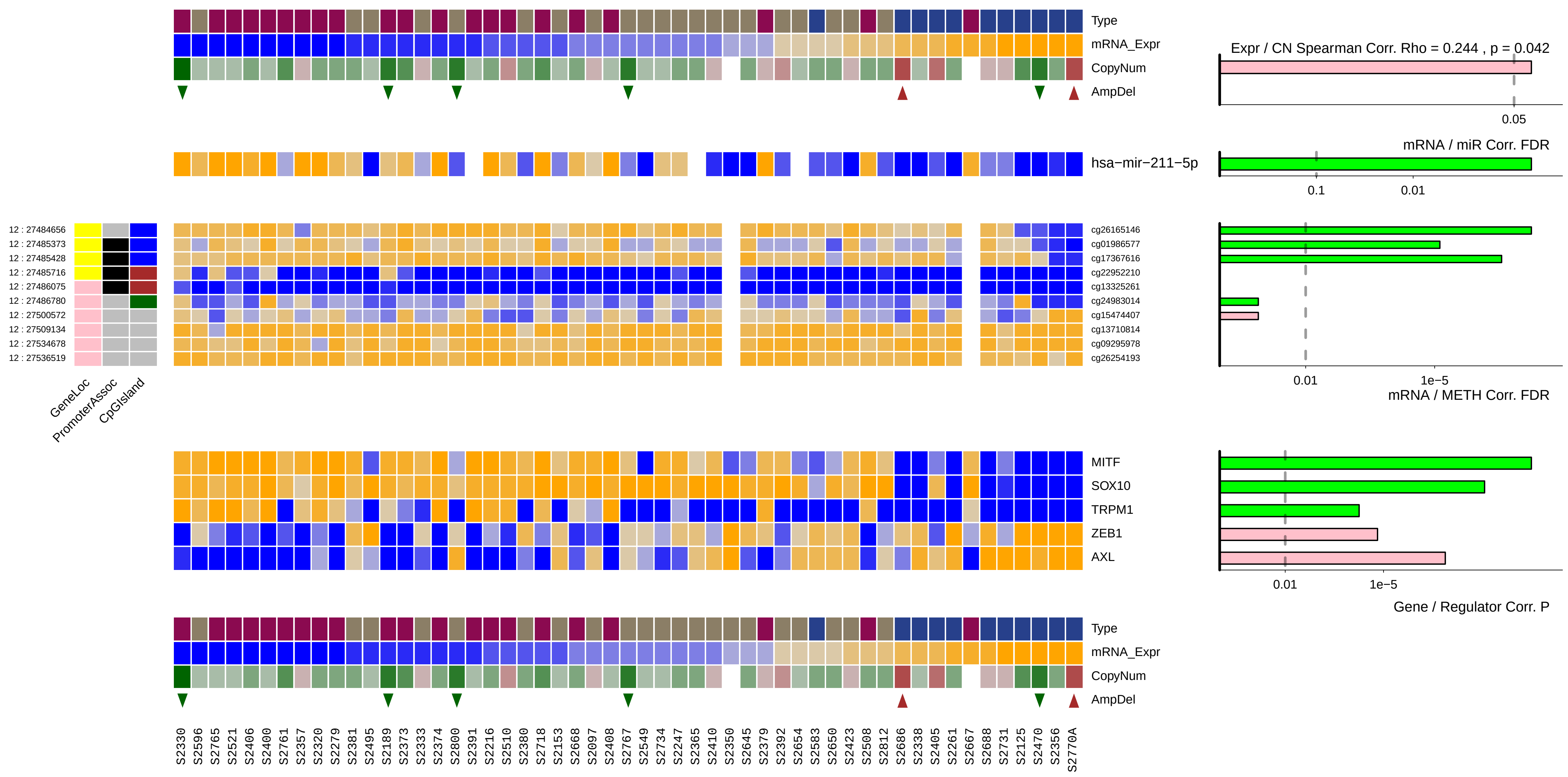
Type
mRNA_Expr
CopyNum
AmpDel
Expr / CN Spearman Corr. Rho = 0.244 , p = 0.042
0.05
hsa−mir−211−5p
mRNA / miR Corr. FDR
0.1
0.01
12 : 27484656
12 : 27485373
12 : 27485428
12 : 27485716
12 : 27486075
12 : 27486780
12 : 27500572
12 : 27509134
12 : 27534678
12 : 27536519
GeneLoc
PromoterAssoc
CpGIsland
cg26165146
cg01986577
cg17367616
cg22952210
cg13325261
cg24983014
cg15474407
cg13710814
cg09295978
cg26254193
0.01
1e−5
mRNA / METH Corr. FDR
MITF
SOX10
TRPM1
ZEB1
AXL
0.01
1e−5
Gene / Regulator Corr. P
Type
mRNA_Expr
CopyNum
AmpDel
S2330 S2596 S2765 S2521 S2406 S2400 S2761 S2357 S2320 S2279 S2381 S2495 S2189 S2373 S2333 S2374 S2800 S2391 S2216 S2510 S2380 S2718 S2153 S2668 S2097 S2408 S2767 S2549 S2734 S2247 S2365 S2410 S2350 S2645 S2379 S2392 S2654 S2583 S2650 S2423 S2508 S2812 S2686 S2338 S2405 S2261 S2667 S2688 S2731 S2125 S2470 S2356 S2770A

GPR176

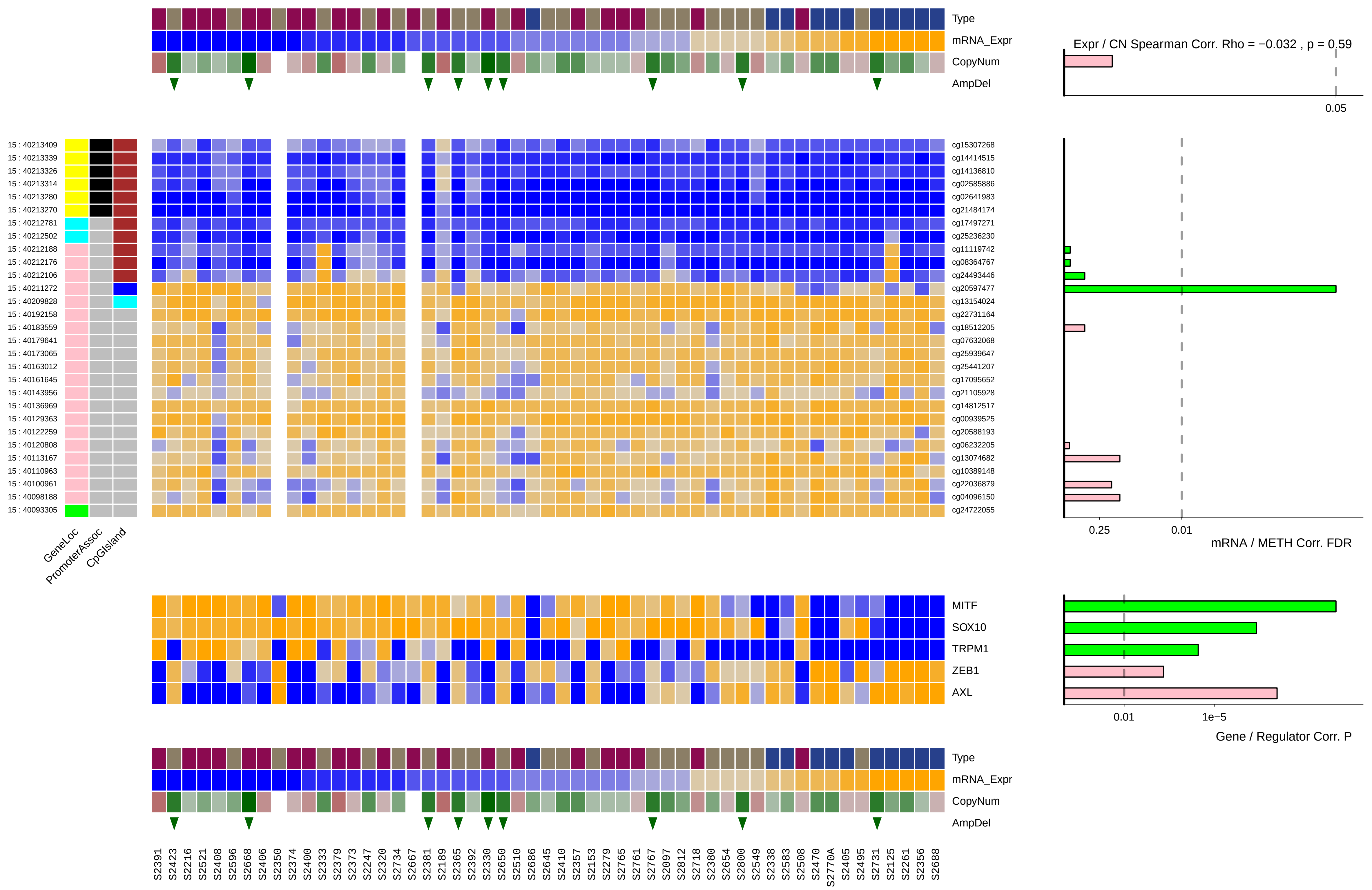

Type
mRNA_Expr
CopyNum
AmpDel
Expr / CN Spearman Corr. Rho = −0.032 , p = 0.59
0.05
15 : 40213409 cg15307268
15 : 40213339 cg14414515
15 : 40213326 cg14136810
15 : 40213314 cg02585886
15 : 40213280 cg02641983
15 : 40213270 cg21484174
15 : 40212781 cg17497271
15 : 40212502 cg25236230
15 : 40212188 cg11119742
15 : 40212176 cg08364767
15 : 40212106 cg24493446
15 : 40211272 cg20597477
15 : 40209828 cg13154024
15 : 40192158 cg22731164
15 : 40183559 cg18512205
15 : 40179641 cg07632068
15 : 40173065 cg25939647
15 : 40163012 cg25441207
15 : 40161645 cg17095652
15 : 40143956 cg21105928
15 : 40136969 cg14812517
15 : 40129363 cg00939525
15 : 40122259 cg20588193
15 : 40120808 cg06232205
15 : 40113167 cg13074682
15 : 40110963 cg10389148
15 : 40100961 cg22036879
15 : 40098188 cg04096150
15 : 40093305 cg24722055
GeneLoc
PromoterAssoc
CpGIsland
0.25
0.01
mRNA / METH Corr. FDR
MITF
SOX10
TRPM1
ZEB1
AXL
0.01
1e−5
Gene / Regulator Corr. P
S2391 S2423 S2216 S2521 S2408 S2596 S2668 S2406 S2350 S2374 S2400 S2333 S2379 S2373 S2247 S2320 S2734 S2667 S2381 S2189 S2365 S2392 S2330 S2650 S2510 S2686 S2645 S2410 S2357 S2153 S2279 S2765 S2761 S2767 S2097 S2812 S2718 S2380 S2654 S2800 S2549 S2338 S2583 S2508 S2470 S2770A S2405 S2495 S2731 S2125 S2261 S2356 S2688

FLNC

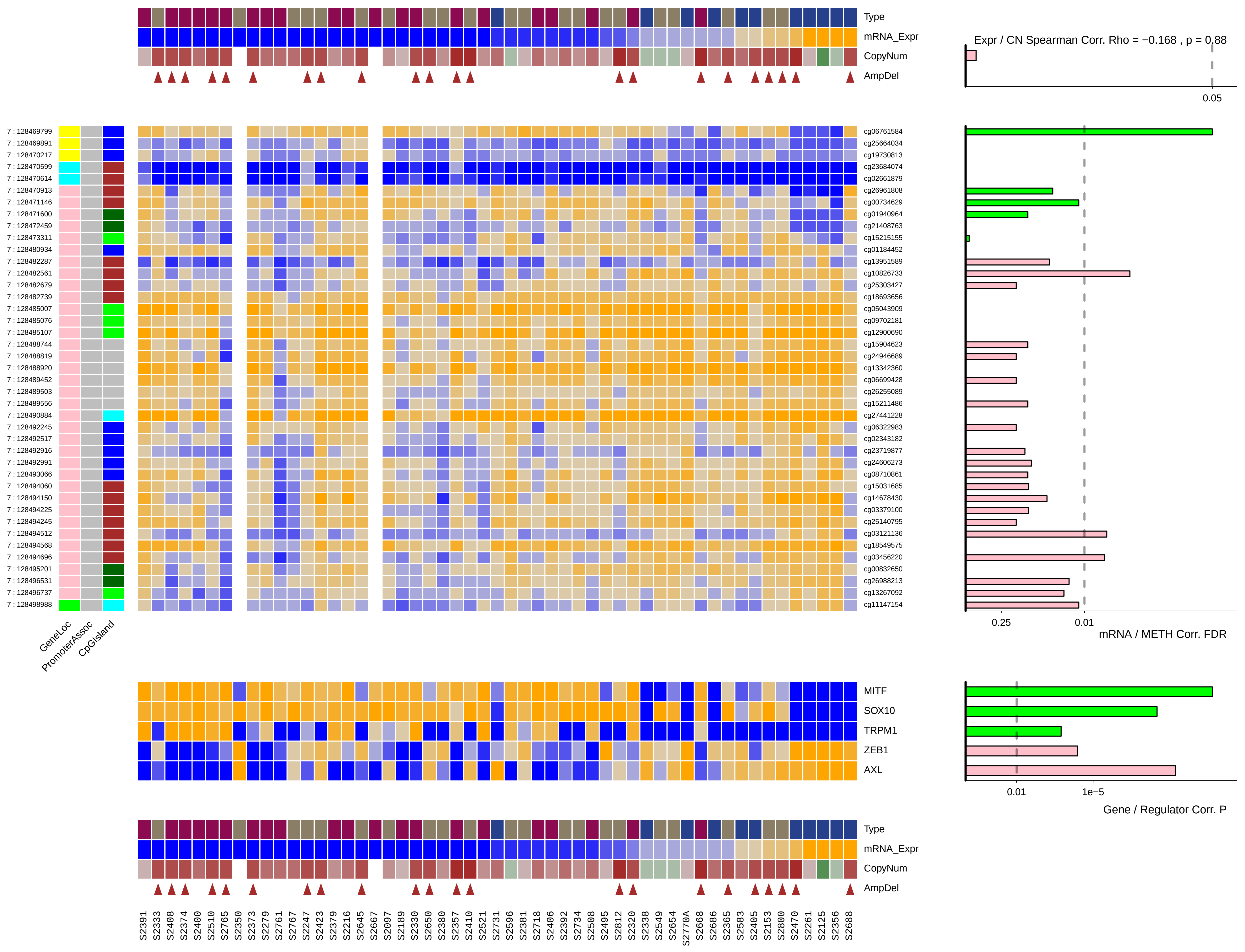

Type
mRNA_Expr
CopyNum
AmpDel
Expr / CN Spearman Corr. Rho = −0.168 , p = 0.88
0.05
7 : 128469799
7 : 128469891
7 : 128470217
7 : 128470599
7 : 128470614
7 : 128470913
7 : 128471146
7 : 128471600
7 : 128472459
7 : 128473311
7 : 128480934
7 : 128482287
7 : 128482561
7 : 128482679
7 : 128482739
7 : 128485007
7 : 128485076
7 : 128485107
7 : 128488744
7 : 128488819
7 : 128488920
7 : 128489452
7 : 128489503
7 : 128489556
7 : 128490884
7 : 128492245
7 : 128492517
7 : 128492916
7 : 128492991
7 : 128493066
7 : 128494060
7 : 128494150
7 : 128494225
7 : 128494245
7 : 128494512
7 : 128494568
7 : 128494696
7 : 128495201
7 : 128496531
7 : 128496737
7 : 128498988
cg06761584
cg25664034
cg19730813
cg23684074
cg02661879
cg26961808
cg00734629
cg01940964
cg21408763
cg15215155
cg01184452
cg13951589
cg10826733
cg25303427
cg18693656
cg05043909
cg09702181
cg12900690
cg15904623
cg24946689
cg13342360
cg06699428
cg26255089
cg15211486
cg27441228
cg06322983
cg02343182
cg23719877
cg24606273
cg08710861
cg15031685
cg14678430
cg03379100
cg25140795
cg03121136
cg18549575
cg03456220
cg00832650
cg26988213
cg13267092
cg11147154
GeneLoc
PromoterAssoc
CpGIsland
0.25
0.01
mRNA / METH Corr. FDR
MITF
SOX10
TRPM1
ZEB1
AXL
0.01
1e−5
Gene / Regulator Corr. P
Type
mRNA_Expr
CopyNum
AmpDel
S2391
S2333
S2408
S2374
S2400
S2510
S2765
S2350
S2373
S2279
S2761
S2767
S2247
S2423
S2379
S2216
S2645
S2667
S2097
S2189
S2330
S2650
S2380
S2357
S2410
S2521
S2731
S2596
S2381
S2718
S2406
S2392
S2734
S2508
S2495
S2812
S2320
S2338
S2549
S2654
S2770A
S2668
S2686
S2365
S2583
S2405
S2153
S2800
S2470
S2261
S2125
S2356
S2688

COL8A1

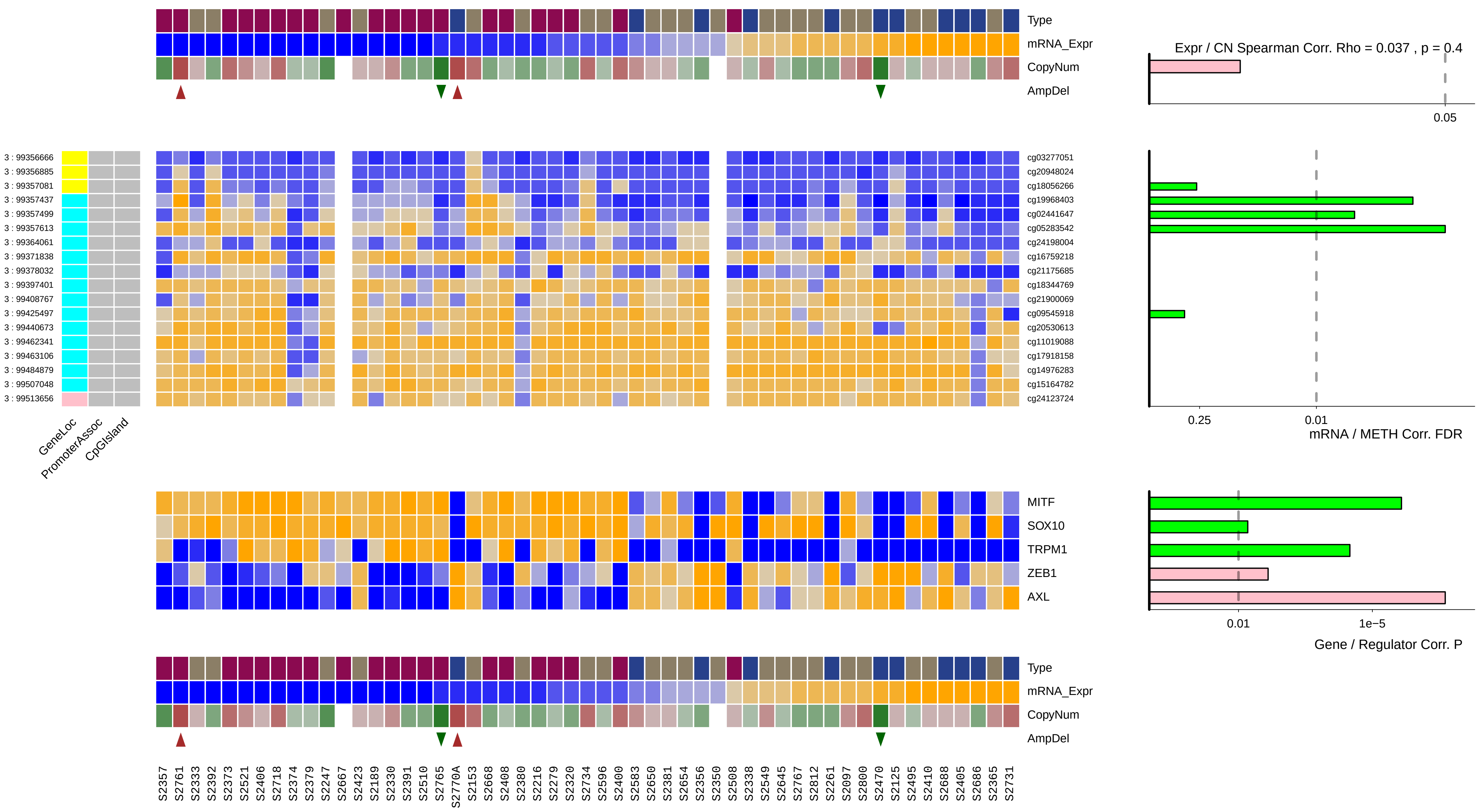

Type
mRNA_Expr
CopyNum
AmpDel
Expr / CN Spearman Corr. Rho = 0.037 , p = 0.4
0.05
3 : 99356666
3 : 99356885
3 : 99357081
3 : 99357437
3 : 99357499
3 : 99357613
3 : 99364061
3 : 99371838
3 : 99378032
3 : 99397401
3 : 99408767
3 : 99425497
3 : 99440673
3 : 99462341
3 : 99463106
3 : 99484879
3 : 99507048
3 : 99513656
cg03277051
cg20948024
cg18056266
cg19968403
cg02441647
cg05283542
cg24198004
cg16759218
cg21175685
cg18344769
cg21900069
cg09545918
cg20530613
cg11019088
cg17918158
cg14976283
cg15164782
cg24123724
GeneLoc
PromoterAssoc
CpGIsland
0.25
0.01
mRNA / METH Corr. FDR
MITF
SOX10
TRPM1
ZEB1
AXL
0.01
1e−5
Gene / Regulator Corr. P
S2357 S2761 S2333 S2392 S2373 S2521 S2406 S2718 S2374 S2379 S2247 S2667 S2423 S2189 S2330 S2391 S2510 S2765 S2770A S2153 S2668 S2408 S2380 S2216 S2279 S2320 S2734 S2596 S2400 S2583 S2650 S2381 S2654 S2356 S2350 S2508 S2338 S2549 S2645 S2767 S2812 S2261 S2097 S2800 S2470 S2125 S2495 S2410 S2688 S2405 S2686 S2365 S2731

CAV1

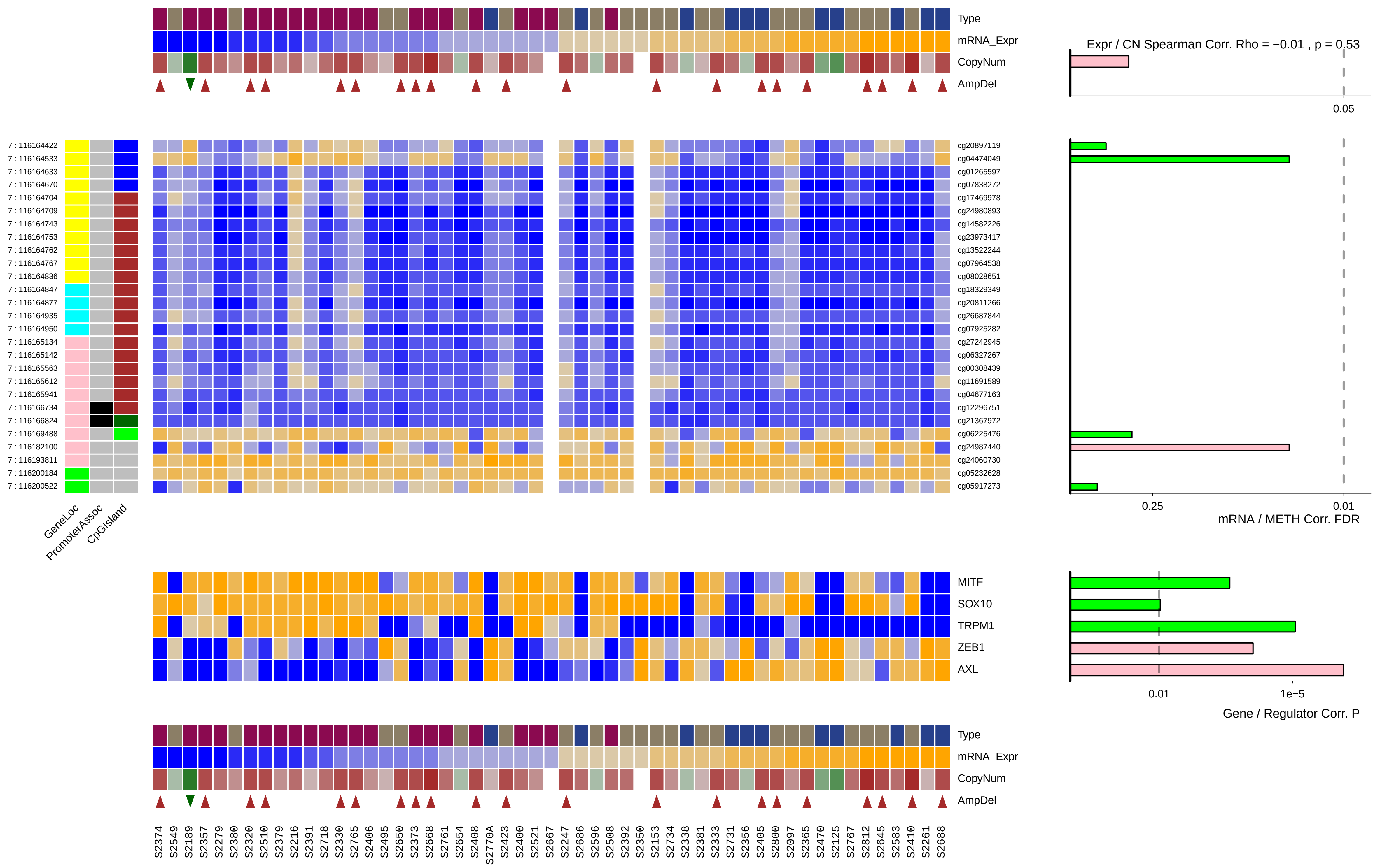

Type
mRNA_Expr
CopyNum
AmpDel
Expr / CN Spearman Corr. Rho = −0.01 , p = 0.53
0.05
GeneLoc
PromoterAssoc
CpGIsland
cg20897119
cg04474049
cg01265597
cg07838272
cg17469978
cg24980893
cg14582226
cg23973417
cg13522244
cg07964538
cg08028651
cg18329349
cg20811266
cg26687844
cg07925282
cg27242945
cg06327267
cg00308439
cg11691589
cg04677163
cg12296751
cg21367972
cg06225476
cg24987440
cg24060730
cg05232628
cg05917273
0.25
0.01
mRNA / METH Corr. FDR
MITF
SOX10
TRPM1
ZEB1
AXL
0.01
1e−5
Gene / Regulator Corr. P

AOX1

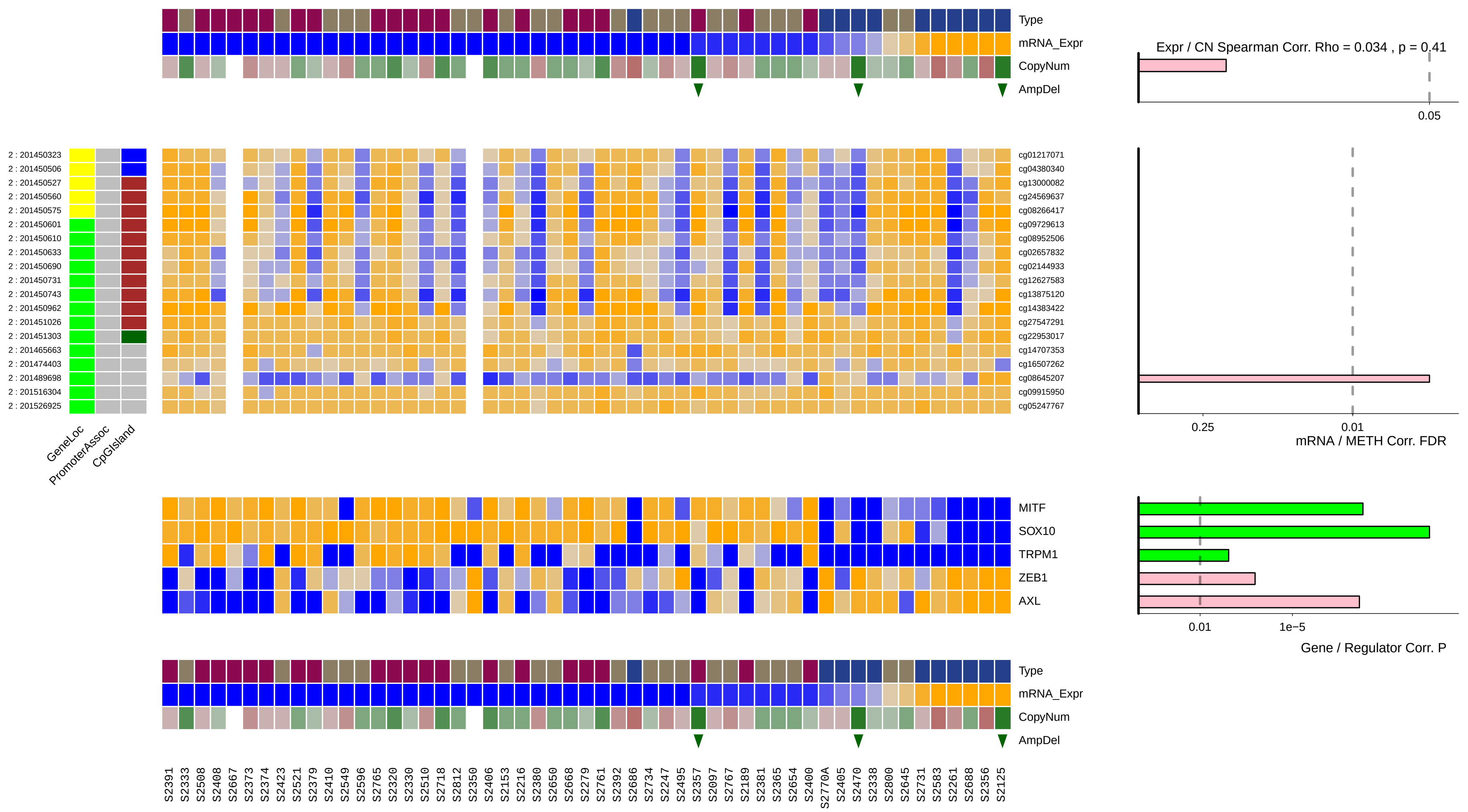

Type
mRNA_Expr
CopyNum
AmpDel
Expr / CN Spearman Corr. Rho = 0.034 , p = 0.41
0.05
2 : 201450323
2 : 201450506
2 : 201450527
2 : 201450560
2 : 201450575
2 : 201450601
2 : 201450610
2 : 201450633
2 : 201450690
2 : 201450731
2 : 201450743
2 : 201450962
2 : 201451026
2 : 201451303
2 : 201465663
2 : 201474403
2 : 201489698
2 : 201516304
2 : 201526925
cg01217071
cg04380340
cg13000082
cg24569637
cg08266417
cg09729613
cg08952506
cg02657832
cg02144933
cg12627583
cg13875120
cg14383422
cg27547291
cg22953017
cg14707353
cg16507262
cg08645207
cg09915950
cg05247767
0.25
0.01
mRNA / METH Corr. FDR
GeneLoc
PromoterAssoc
CpGIsland
MITF
SOX10
TRPM1
ZEB1
AXL
0.01
1e−5
Gene / Regulator Corr. P
Type
mRNA_Expr
CopyNum
AmpDel
S2391 S2333 S2508 S2408 S2667 S2373 S2374 S2423 S2521 S2379 S2410 S2549 S2596 S2765 S2320 S2330 S2510 S2718 S2812 S2350 S2406 S2153 S2216 S2380 S2650 S2668 S2279 S2761 S2392 S2686 S2734 S2247 S2495 S2357 S2097 S2767 S2189 S2381 S2365 S2654 S2400 S2770A S2405 S2470 S2338 S2800 S2645 S2731 S2583 S2261 S2688 S2356 S2125

PTGFR

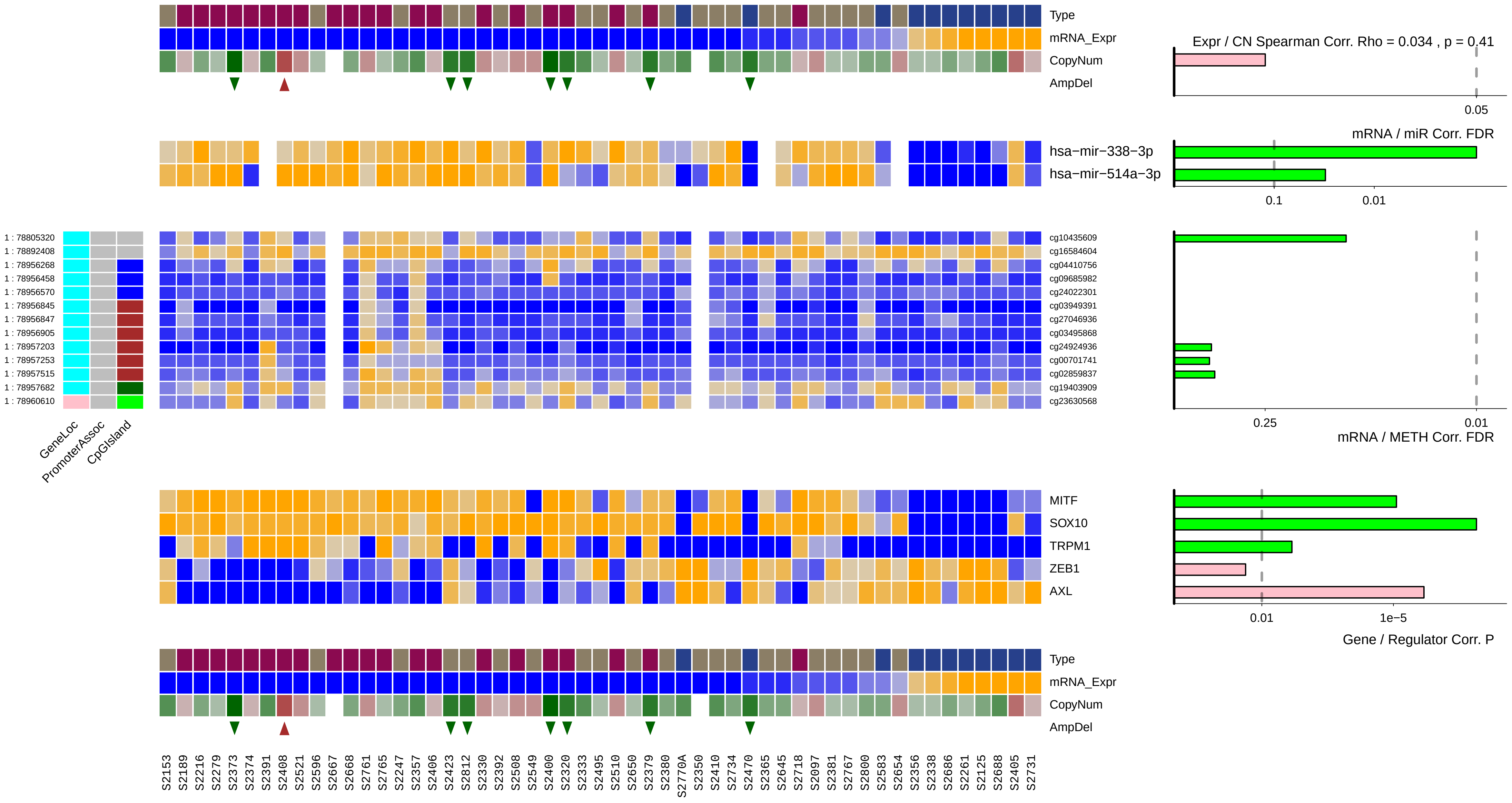

Type
mRNA_Expr
CopyNum
AmpDel
Expr / CN Spearman Corr. Rho = 0.034 , p = 0.41
0.05
mRNA / miR Corr. FDR
hsa−mir−338−3p
hsa−mir−514a−3p
0.1
0.01
1 : 78805320
1 : 78892408
1 : 78956268
1 : 78956458
1 : 78956570
1 : 78956845
1 : 78956847
1 : 78956905
1 : 78957203
1 : 78957253
1 : 78957515
1 : 78957682
1 : 78960610
GeneLoc
PromoterAssoc
CpGIsland
cg10435609
cg16584604
cg04410756
cg09685982
cg24022301
cg03949391
cg27046936
cg03495868
cg24924936
cg00701741
cg02859837
cg19403909
cg23630568
0.25
0.01
mRNA / METH Corr. FDR
MITF
SOX10
TRPM1
ZEB1
AXL
0.01
1e−5
Gene / Regulator Corr. P
Type
mRNA_Expr
CopyNum
AmpDel
S2153 S2189 S2216 S2279 S2373 S2374 S2391 S2408 S2521 S2596 S2667 S2668 S2761 S2765 S2247 S2357 S2406 S2423 S2812 S2330 S2392 S2508 S2549 S2400 S2320 S2333 S2495 S2510 S2650 S2379 S2380 S2770A S2350 S2410 S2734 S2470 S2365 S2645 S2718 S2097 S2381 S2767 S2800 S2583 S2654 S2356 S2338 S2686 S2261 S2125 S2688 S2405 S2731

ALPK2

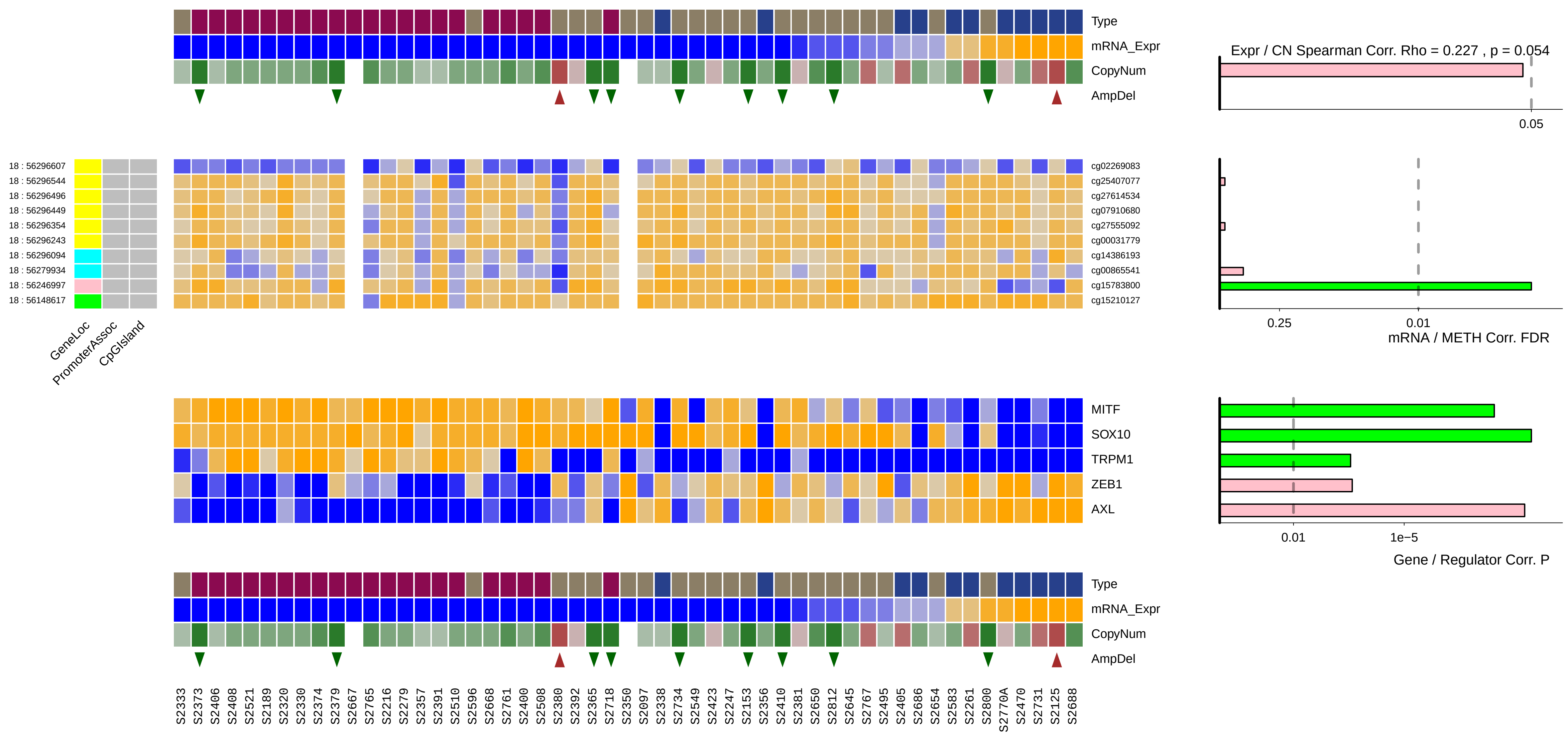

Type
mRNA_Expr
CopyNum
AmpDel
Expr / CN Spearman Corr. Rho = 0.227 , p = 0.054
0.05
18 : 56296607
18 : 56296544
18 : 56296496
18 : 56296449
18 : 56296354
18 : 56296243
18 : 56296094
18 : 56279934
18 : 56246997
18 : 56148617
GeneLoc
PromoterAssoc
CpGIsland
cg02269083
cg25407077
cg27614534
cg07910680
cg27555092
cg00031779
cg14386193
cg00865541
cg15783800
cg15210127
0.25
0.01
mRNA / METH Corr. FDR
MITF
SOX10
TRPM1
ZEB1
AXL
0.01
1e−5
Gene / Regulator Corr. P
Type
mRNA_Expr
CopyNum
AmpDel
S2333 S2373 S2406 S2408 S2521 S2189 S2320 S2330 S2374 S2379 S2667 S2765 S2216 S2279 S2357 S2391 S2510 S2596 S2668 S2761 S2400 S2508 S2380 S2392 S2365 S2718 S2350 S2097 S2338 S2734 S2549 S2423 S2247 S2153 S2356 S2410 S2381 S2650 S2812 S2645 S2767 S2495 S2405 S2686 S2654 S2583 S2261 S2800 S2770A S2470 S2731 S2125 S2688

CFH

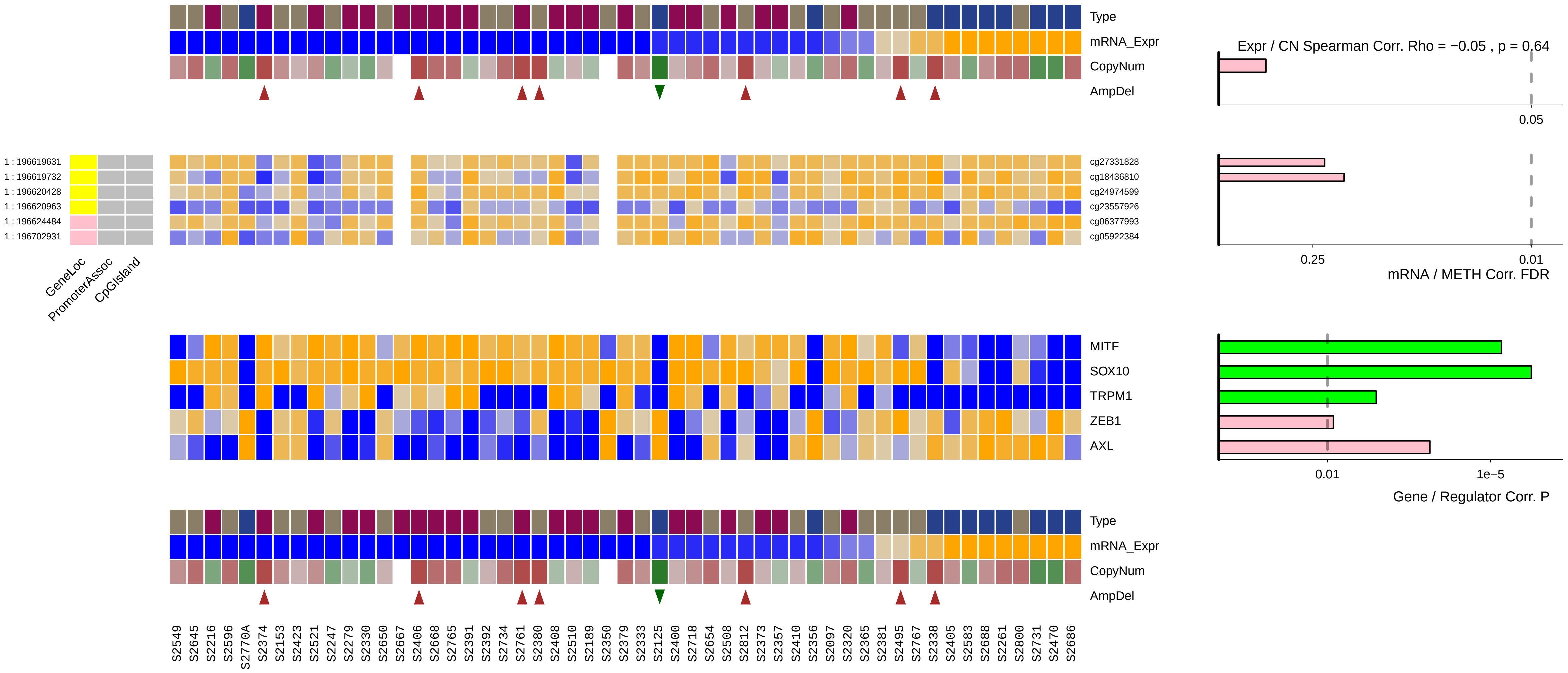

Type
mRNA_Expr
CopyNum
AmpDel
Expr / CN Spearman Corr. Rho = −0.05 , p = 0.64
0.05
1 : 196619631
1 : 196619732
1 : 196620428
1 : 196620963
1 : 196624484
1 : 196702931
GeneLoc
PromoterAssoc
CpGIsland
cg27331828
cg18436810
cg24974599
cg23557926
cg06377993
cg05922384
0.25
0.01
mRNA / METH Corr. FDR
MITF
SOX10
TRPM1
ZEB1
AXL
0.01
1e−5
Gene / Regulator Corr. P
Type
mRNA_Expr
CopyNum
AmpDel
S2549 S2645 S2216 S2596 S2770A S2374 S2153 S2423 S2521 S2247 S2279 S2330 S2650 S2667 S2406 S2668 S2765 S2391 S2392 S2734 S2761 S2380 S2408 S2510 S2189 S2350 S2379 S2333 S2125 S2400 S2718 S2654 S2508 S2812 S2373 S2357 S2410 S2356 S2097 S2320 S2365 S2381 S2495 S2767 S2338 S2405 S2583 S2688 S2261 S2800 S2731 S2470 S2686

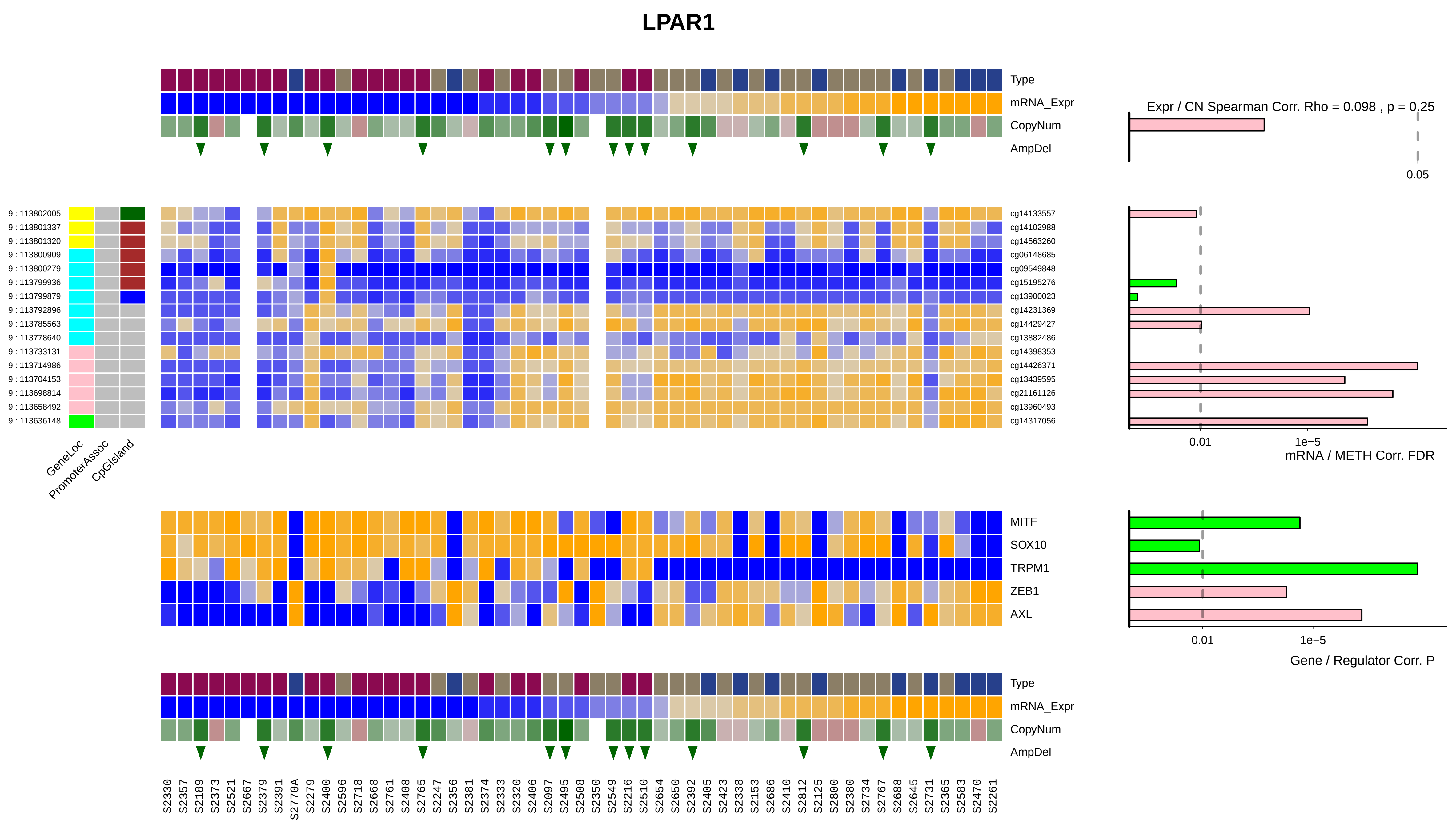
LPAR1
Type
mRNA_Expr
CopyNum
AmpDel
Expr / CN Spearman Corr. Rho = 0.098 , p = 0.25
0.05
GeneLoc
PromoterAssoc
CpGIsland
mRNA / METH Corr. FDR
0.01
1e−5
MITF
SOX10
TRPM1
ZEB1
AXL
Gene / Regulator Corr. P

F3

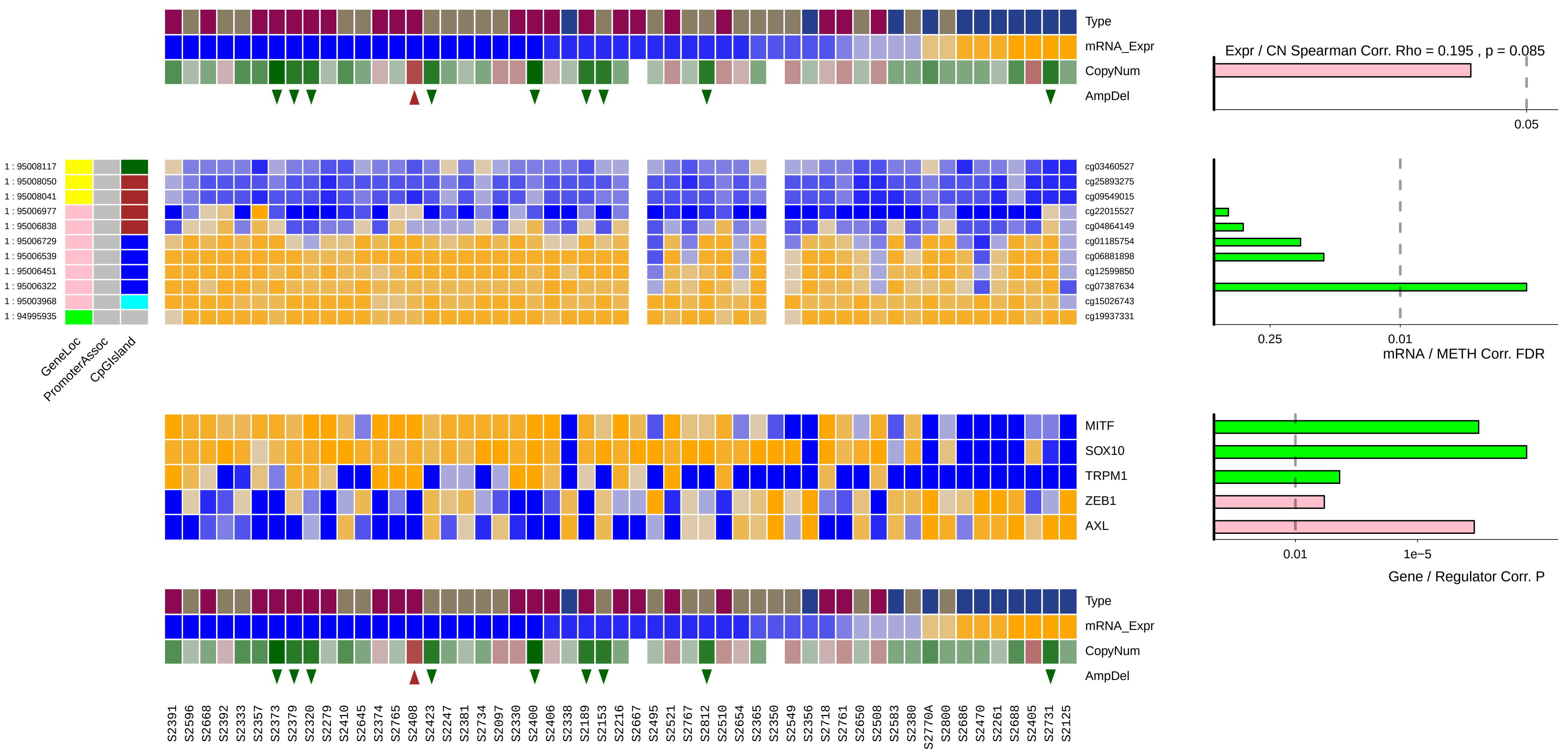
Type
mRNA_Expr
CopyNum
AmpDel
Expr / CN Spearman Corr. Rho = 0.195 , p = 0.085
0.05
1 : 95008117
1 : 95008050
1 : 95008041
1 : 95006977
1 : 95006838
1 : 95006729
1 : 95006539
1 : 95006451
1 : 95006322
1 : 95003968
1 : 94995935
GeneLoc
PromoterAssoc
CpGIsland
cg03460527
cg25893275
cg09549015
cg22015527
cg04864149
cg01185754
cg06881898
cg12599850
cg07387634
cg15026743
cg19937331
0.25
0.01
mRNA / METH Corr. FDR
MITF
SOX10
TRPM1
ZEB1
AXL
0.01
1e−5
Gene / Regulator Corr. P
Type
mRNA_Expr
CopyNum
AmpDel
S2391 S2596 S2668 S2392 S2333 S2357 S2373 S2379 S2320 S2279 S2410 S2645 S2374 S2765 S2408 S2423 S2247 S2381 S2734 S2097 S2330 S2400 S2406 S2338 S2189 S2153 S2216 S2667 S2495 S2521 S2767 S2812 S2510 S2654 S2365 S2350 S2549 S2356 S2718 S2761 S2650 S2508 S2583 S2380 S2770A S2800 S2686 S2470 S2261 S2688 S2405 S2731 S2125

CNN2

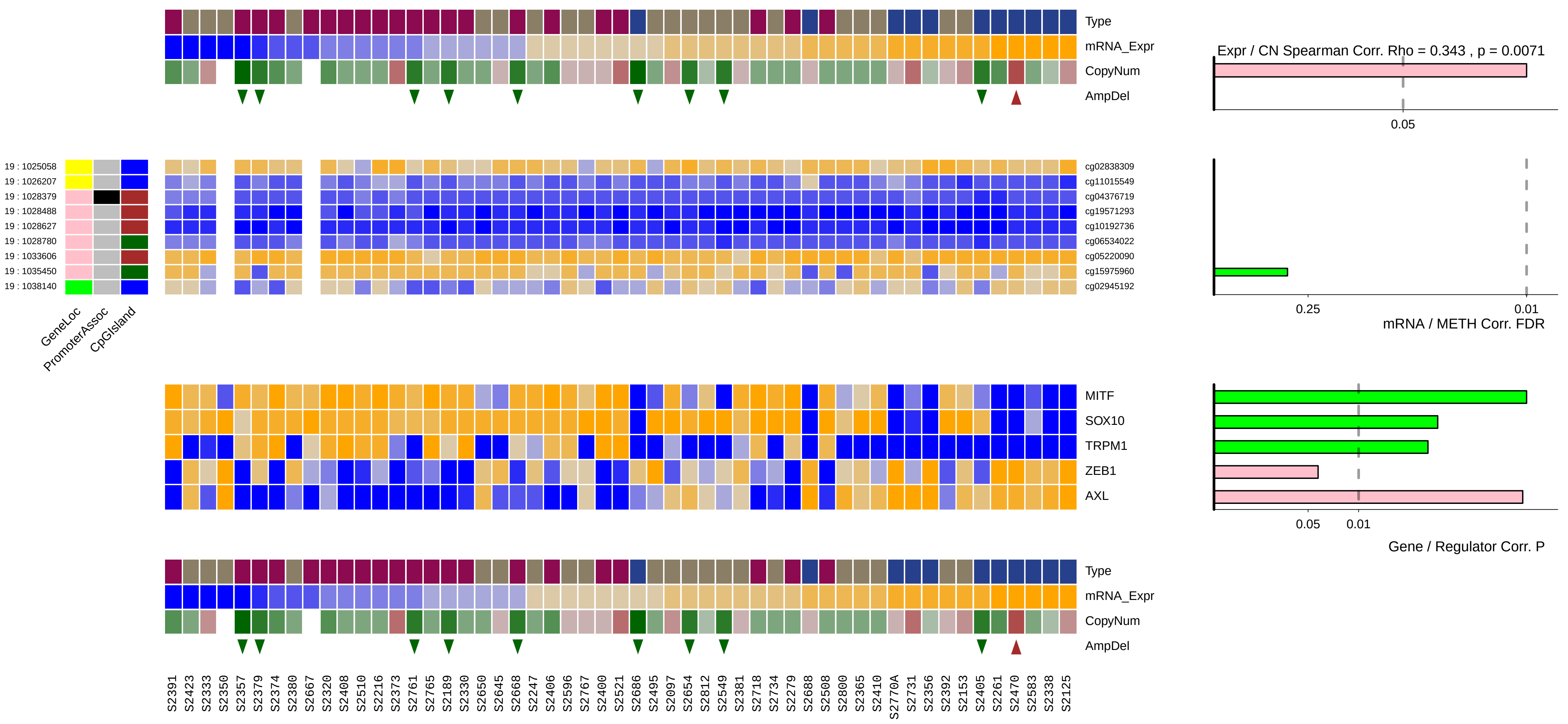

Type
mRNA_Expr
CopyNum
AmpDel
Expr / CN Spearman Corr. Rho = 0.343 , p = 0.0071
0.05
19 : 1025058
19 : 1026207
19 : 1028379
19 : 1028488
19 : 1028627
19 : 1028780
19 : 1033606
19 : 1035450
19 : 1038140
GeneLoc
PromoterAssoc
CpGIsland
cg02838309
cg11015549
cg04376719
cg19571293
cg10192736
cg06534022
cg05220090
cg15975960
cg02945192
0.25
0.01
mRNA / METH Corr. FDR
MITF
SOX10
TRPM1
ZEB1
AXL
0.05
0.01
Gene / Regulator Corr. P
S2391 S2423 S2333 S2350 S2357 S2379 S2374 S2380 S2667 S2320 S2408 S2510 S2216 S2373 S2761 S2765 S2189 S2330 S2650 S2645 S2668 S2247 S2406 S2596 S2767 S2400 S2521 S2686 S2495 S2097 S2654 S2812 S2549 S2381 S2718 S2734 S2279 S2688 S2508 S2800 S2365 S2410 S2770A S2731 S2356 S2392 S2153 S2405 S2261 S2470 S2583 S2338 S2125

MAP1B

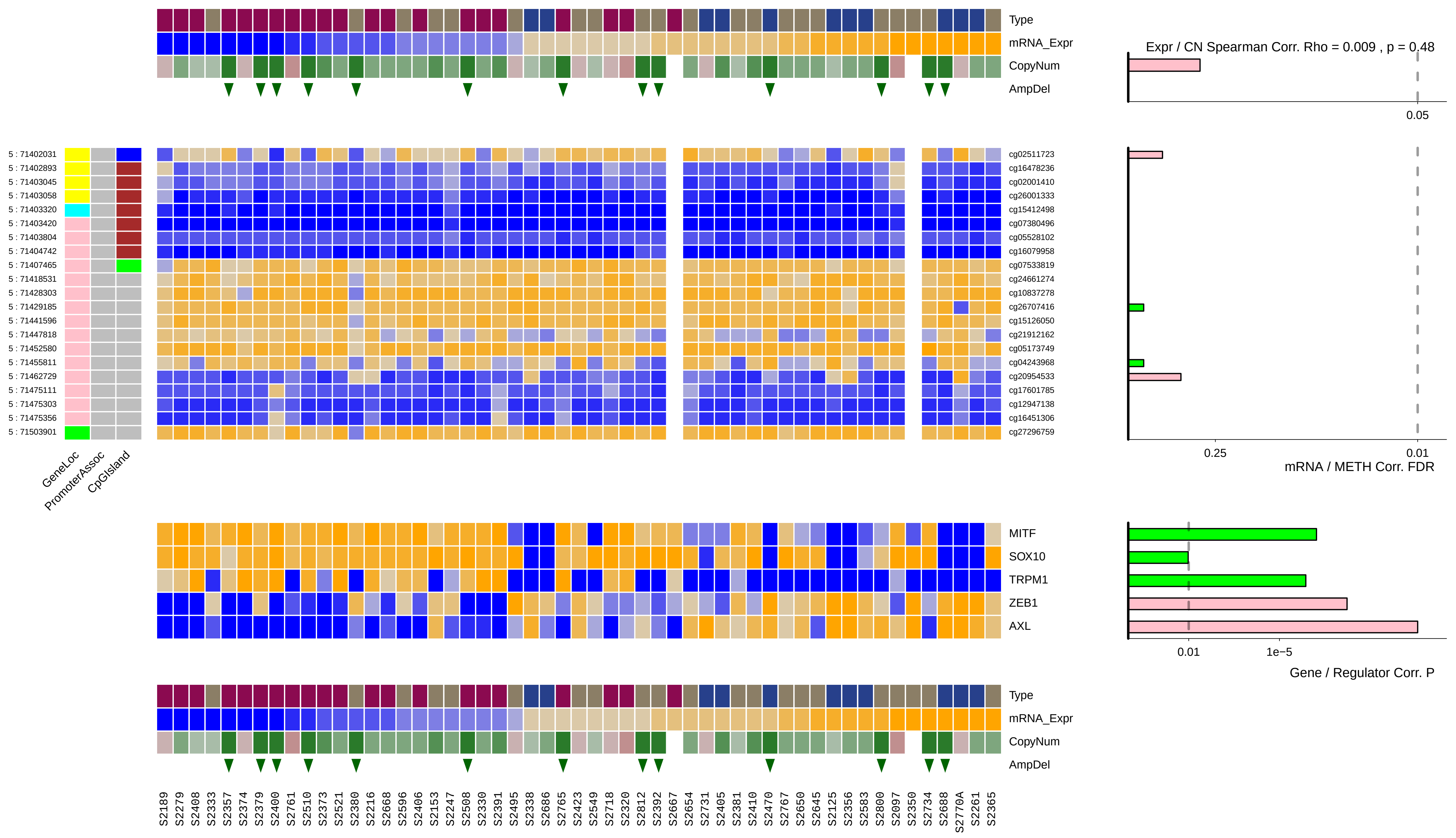
Type
mRNA_Expr
CopyNum
AmpDel
Expr / CN Spearman Corr. Rho = 0.009 , p = 0.48
0.05
5 : 71402031
5 : 71402893
5 : 71403045
5 : 71403058
5 : 71403320
5 : 71403420
5 : 71403804
5 : 71404742
5 : 71407465
5 : 71418531
5 : 71428303
5 : 71429185
5 : 71441596
5 : 71447818
5 : 71452580
5 : 71455811
5 : 71462729
5 : 71475111
5 : 71475303
5 : 71475356
5 : 71503901
cg02511723
cg16478236
cg02001410
cg26001333
cg15412498
cg07380496
cg05528102
cg16079958
cg07533819
cg24661274
cg10837278
cg26707416
cg15126050
cg21912162
cg05173749
cg04243968
cg20954533
cg17601785
cg12947138
cg16451306
cg27296759
GeneLoc
PromoterAssoc
CpGIsland
0.25
0.01
mRNA / METH Corr. FDR
MITF
SOX10
TRPM1
ZEB1
AXL
0.01
1e−5
Gene / Regulator Corr. P
Type
mRNA_Expr
CopyNum
AmpDel
S2189 S2279 S2408 S2333 S2357 S2374 S2379 S2400 S2761 S2510 S2373 S2521 S2380 S2216 S2668 S2596 S2406 S2153 S2247 S2508 S2330 S2391 S2495 S2338 S2686 S2765 S2423 S2549 S2718 S2320 S2812 S2392 S2667 S2654 S2731 S2405 S2381 S2410 S2470 S2767 S2650 S2645 S2125 S2356 S2583 S2800 S2097 S2350 S2734 S2688 S2770A S2261 S2365

ITGB1

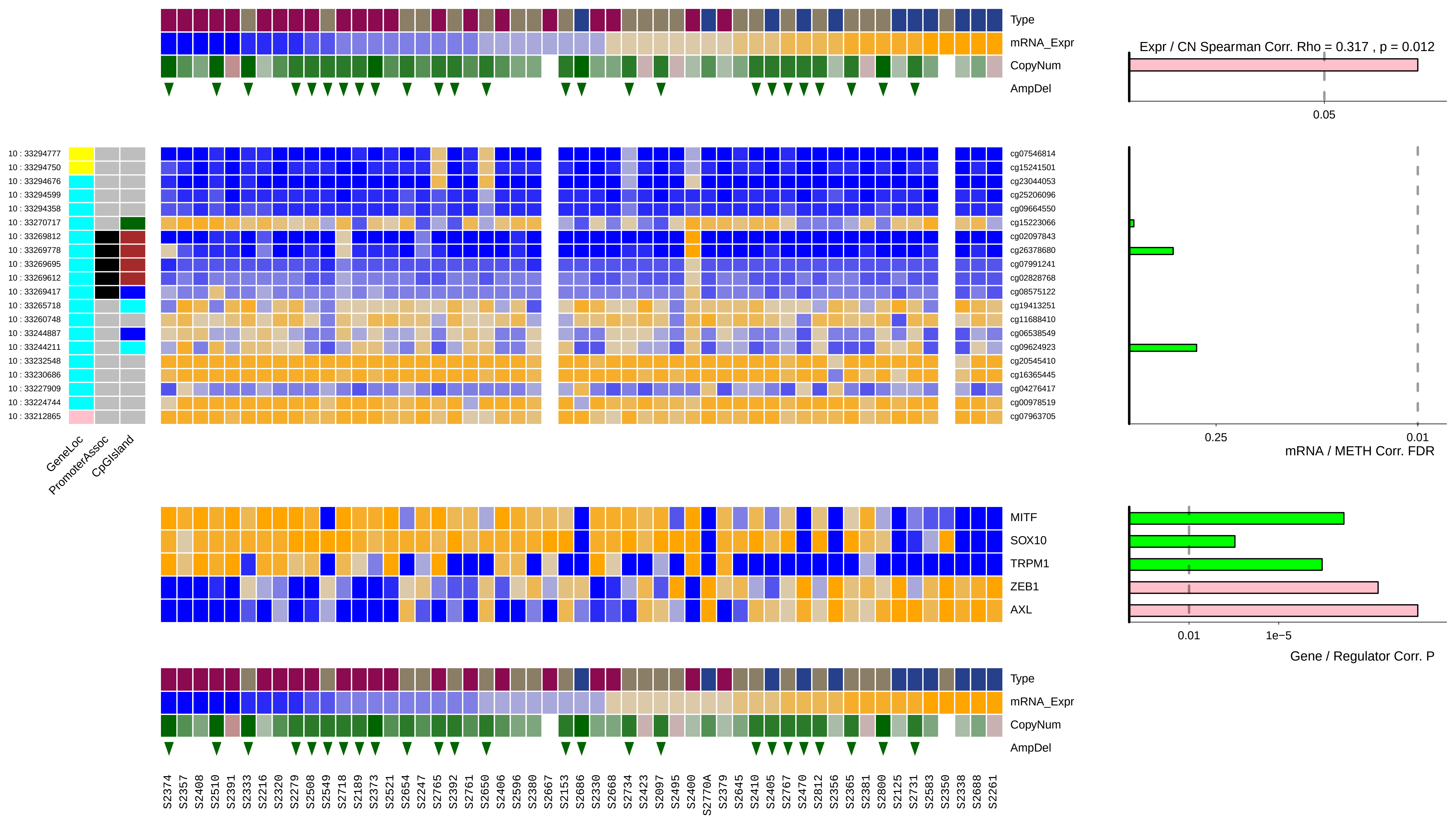
Type
mRNA_Expr
CopyNum
AmpDel
Expr / CN Spearman Corr. Rho = 0.317 , p = 0.012
0.05
10 : 33294777
10 : 33294750
10 : 33294676
10 : 33294599
10 : 33294358
10 : 33270717
10 : 33269812
10 : 33269778
10 : 33269695
10 : 33269612
10 : 33269417
10 : 33265718
10 : 33260748
10 : 33244887
10 : 33244211
10 : 33232548
10 : 33230686
10 : 33227909
10 : 33224744
10 : 33212865
GeneLoc
PromoterAssoc
CpGIsland
cg07546814
cg15241501
cg23044053
cg25206096
cg09664550
cg15223066
cg02097843
cg26378680
cg07991241
cg02828768
cg08575122
cg19413251
cg11688410
cg06538549
cg09624923
cg20545410
cg16365445
cg04276417
cg00978519
cg07963705
0.25
0.01
mRNA / METH Corr. FDR
MITF
SOX10
TRPM1
ZEB1
AXL
0.01
1e−5
Gene / Regulator Corr. P
Type
mRNA_Expr
CopyNum
AmpDel
S2374
S2357
S2408
S2510
S2391
S2333
S2216
S2320
S2279
S2508
S2549
S2718
S2189
S2373
S2521
S2654
S2247
S2765
S2392
S2761
S2650
S2406
S2596
S2380
S2667
S2153
S2686
S2330
S2668
S2734
S2423
S2097
S2495
S2400
S2770A
S2379
S2645
S2410
S2405
S2767
S2470
S2812
S2356
S2365
S2381
S2800
S2125
S2731
S2583
S2350
S2338
S2688
S2261

VCAN

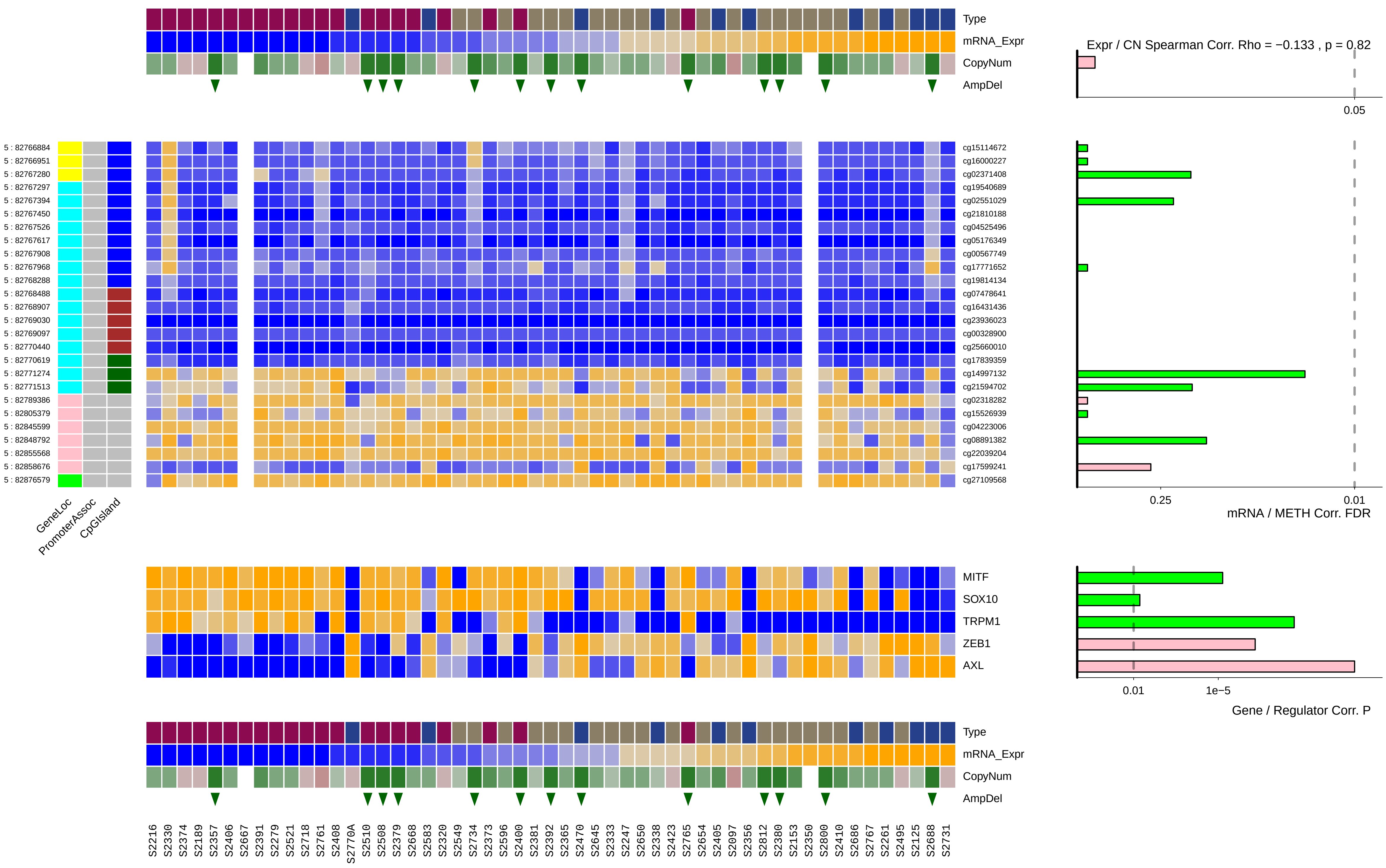
Type
mRNA_Expr
CopyNum
AmpDel
Expr / CN Spearman Corr. Rho = −0.133 , p = 0.82
0.05
mRNA / METH Corr. FDR
0.25
0.01
GeneLoc
PromoterAssoc
CpGIsland
MITF
SOX10
TRPM1
ZEB1
AXL
Gene / Regulator Corr. P
0.01
1e−5

NNMT

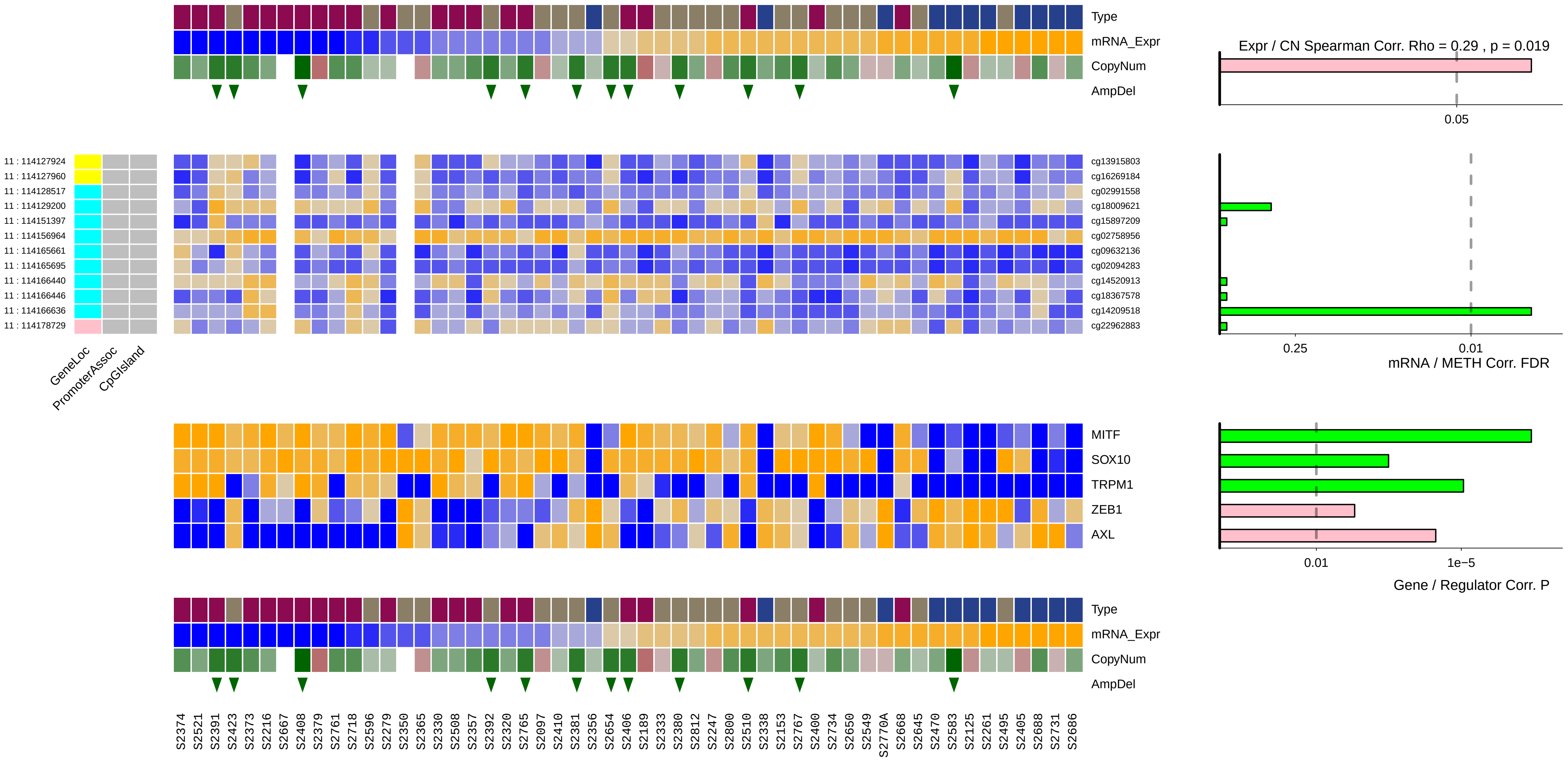

Type
mRNA_Expr
CopyNum
AmpDel
Expr / CN Spearman Corr. Rho = 0.29 , p = 0.019
0.05
11 : 114127924
11 : 114127960
11 : 114128517
11 : 114129200
11 : 114151397
11 : 114156964
11 : 114165661
11 : 114165695
11 : 114166440
11 : 114166446
11 : 114166636
11 : 114178729
GeneLoc
PromoterAssoc
CpGIsland
cg13915803
cg16269184
cg02991558
cg18009621
cg15897209
cg02758956
cg09632136
cg02094283
cg14520913
cg18367578
cg14209518
cg22962883
0.25
0.01
mRNA / METH Corr. FDR
MITF
SOX10
TRPM1
ZEB1
AXL
0.01
1e−5
Gene / Regulator Corr. P
Type
mRNA_Expr
CopyNum
AmpDel
S2374 S2521 S2391 S2423 S2373 S2216 S2667 S2408 S2379 S2761 S2718 S2596 S2279 S2350 S2365 S2330 S2508 S2357 S2392 S2320 S2765 S2097 S2410 S2381 S2356 S2654 S2406 S2189 S2333 S2380 S2812 S2247 S2800 S2510 S2338 S2153 S2767 S2400 S2734 S2650 S2549 S2770A S2668 S2645 S2470 S2583 S2125 S2261 S2495 S2405 S2688 S2731 S2686

TNFAIP2

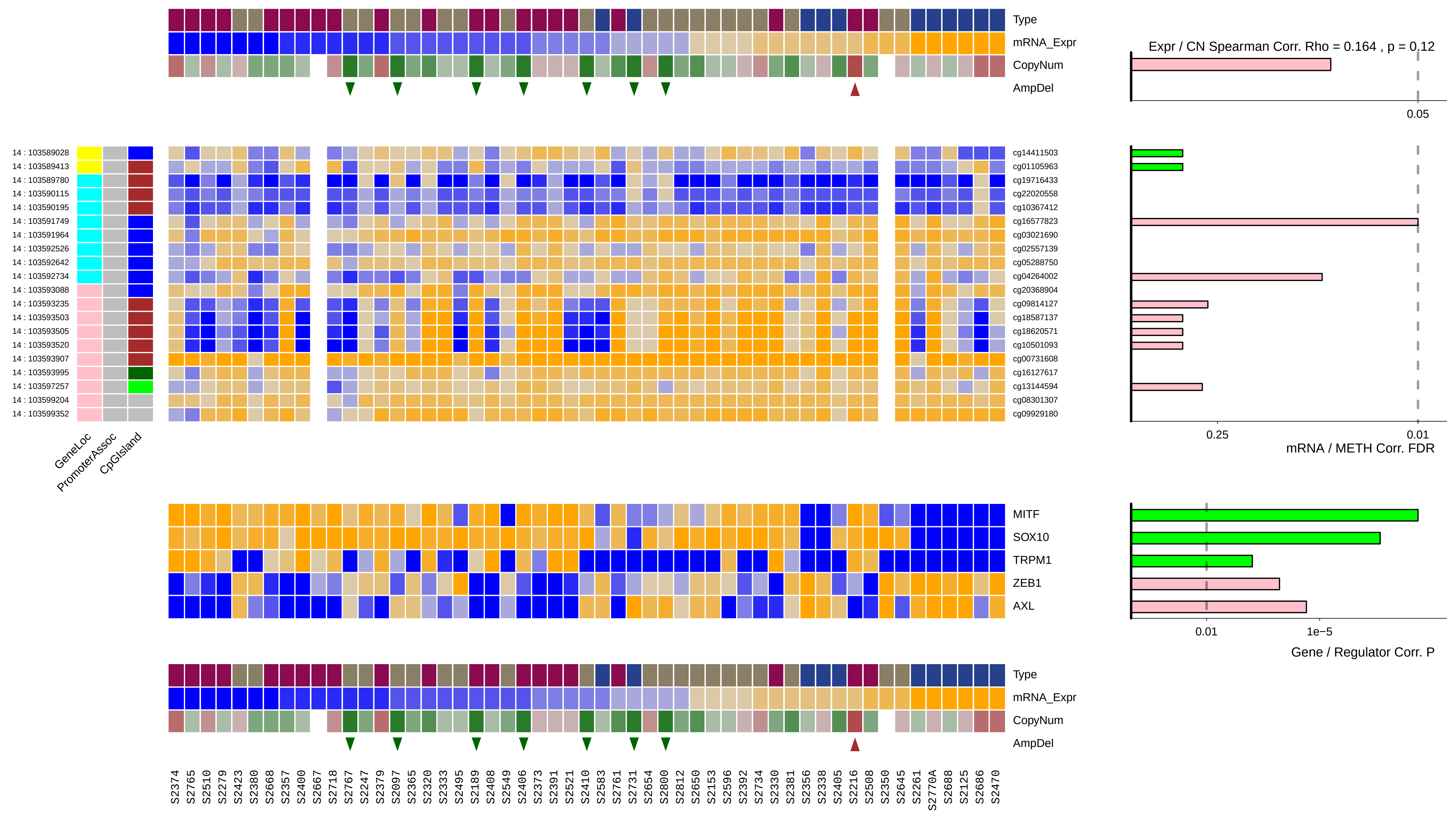

Type
mRNA_Expr
CopyNum
AmpDel
Expr / CN Spearman Corr. Rho = 0.164 , p = 0.12
0.05
14 : 103589028
14 : 103589413
14 : 103589780
14 : 103590115
14 : 103590195
14 : 103591749
14 : 103591964
14 : 103592526
14 : 103592642
14 : 103592734
14 : 103593088
14 : 103593235
14 : 103593503
14 : 103593505
14 : 103593520
14 : 103593907
14 : 103593995
14 : 103597257
14 : 103599204
14 : 103599352
cg14411503
cg01105963
cg19716433
cg22020558
cg10367412
cg16577823
cg03021690
cg02557139
cg05288750
cg04264002
cg20368904
cg09814127
cg18587137
cg18620571
cg10501093
cg00731608
cg16127617
cg13144594
cg08301307
cg09929180
0.25
0.01
mRNA / METH Corr. FDR
GeneLoc
PromoterAssoc
CpGIsland
MITF
SOX10
TRPM1
ZEB1
AXL
0.01
1e−5
Gene / Regulator Corr. P
Type
mRNA_Expr
CopyNum
AmpDel
S2374 S2765 S2510 S2279 S2423 S2380 S2668 S2357 S2400 S2667 S2718 S2767 S2247 S2379 S2097 S2365 S2320 S2333 S2495 S2189 S2408 S2549 S2406 S2373 S2391 S2521 S2410 S2583 S2761 S2731 S2654 S2800 S2812 S2650 S2153 S2596 S2392 S2734 S2330 S2381 S2356 S2338 S2405 S2216 S2508 S2350 S2645 S2261 S2770A S2688 S2125 S2686 S2470

ITGA5

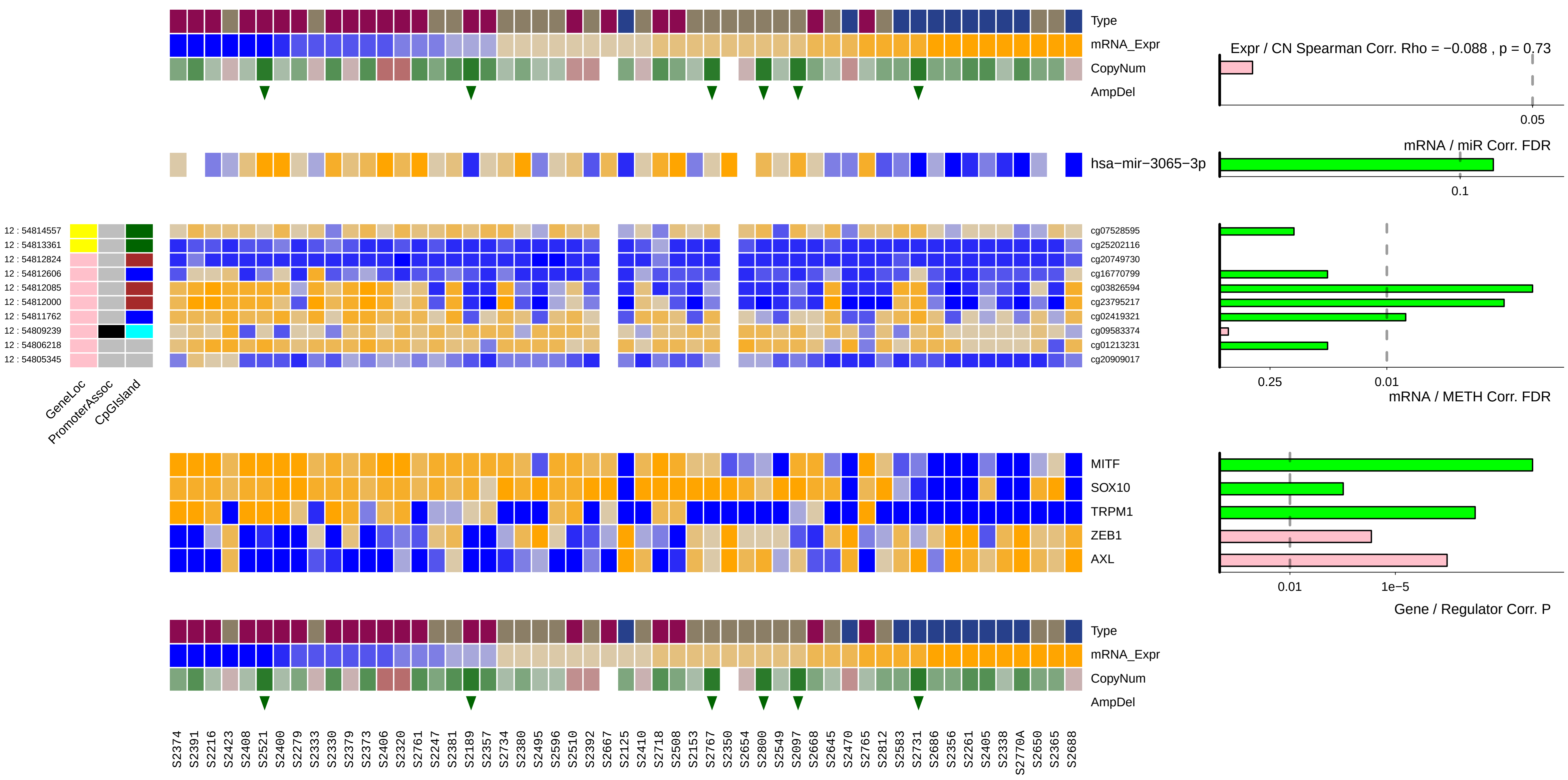
Type
mRNA_Expr
CopyNum
AmpDel
Expr / CN Spearman Corr. Rho = −0.088 , p = 0.73
0.05
mRNA / miR Corr. FDR
hsa−mir−3065−3p
0.1
12 : 54814557
12 : 54813361
12 : 54812824
12 : 54812606
12 : 54812085
12 : 54812000
12 : 54811762
12 : 54809239
12 : 54806218
12 : 54805345
GeneLoc
PromoterAssoc
CpGIsland
cg07528595
cg25202116
cg20749730
cg16770799
cg03826594
cg23795217
cg02419321
cg09583374
cg01213231
cg20909017
0.25
0.01
mRNA / METH Corr. FDR
MITF
SOX10
TRPM1
ZEB1
AXL
0.01
1e−5
Gene / Regulator Corr. P
Type
mRNA_Expr
CopyNum
AmpDel
S2374 S2391 S2216 S2423 S2408 S2521 S2400 S2279 S2333 S2330 S2379 S2373 S2406 S2320 S2761 S2247 S2381 S2189 S2357 S2734 S2380 S2495 S2596 S2510 S2392 S2667 S2125 S2410 S2718 S2508 S2153 S2767 S2350 S2654 S2800 S2549 S2097 S2668 S2645 S2470 S2765 S2812 S2583 S2731 S2686 S2356 S2261 S2405 S2338 S2770A S2650 S2365 S2688

FSCN1

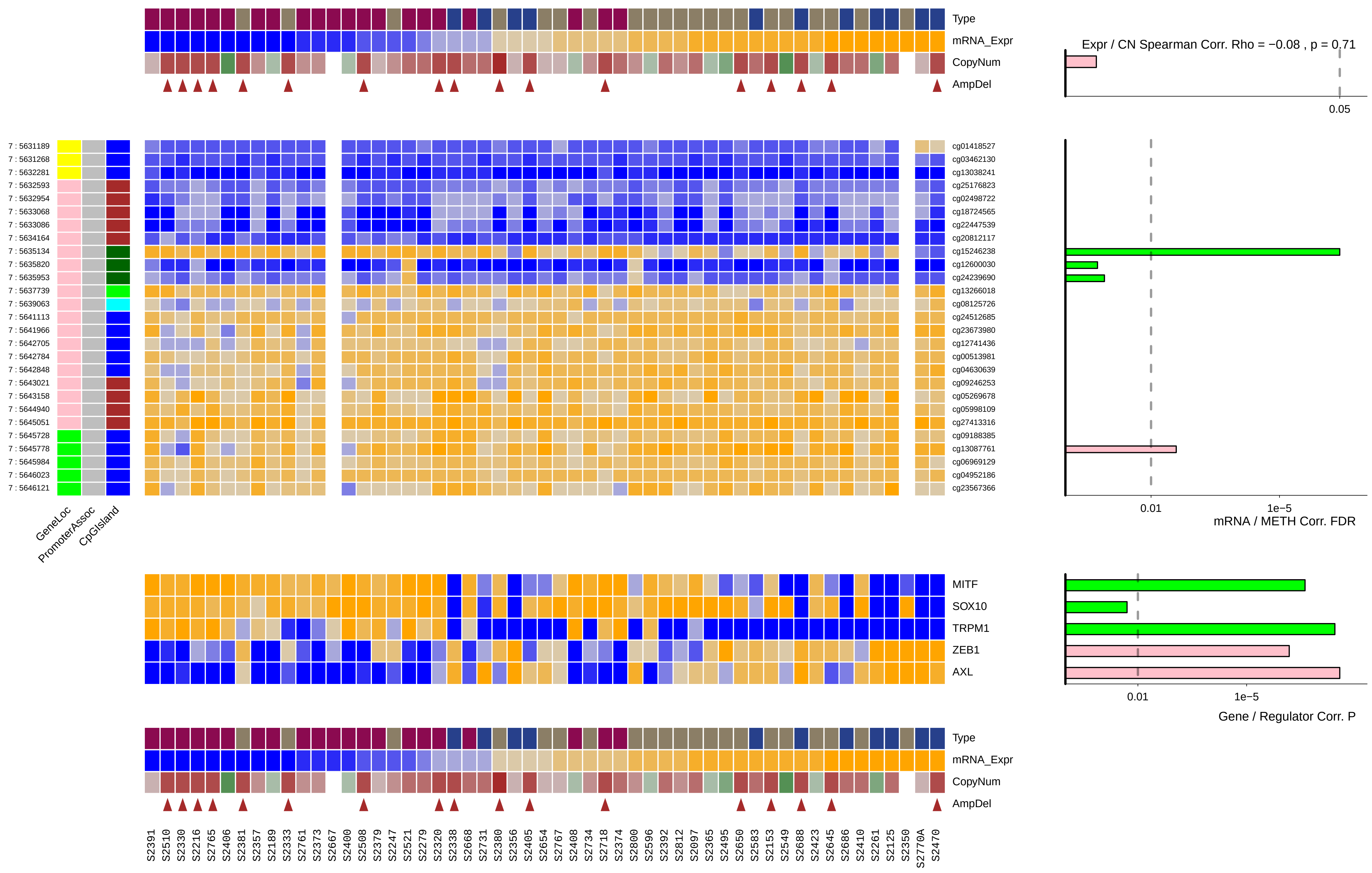

Type
mRNA_Expr
CopyNum
AmpDel
Expr / CN Spearman Corr. Rho = −0.08 , p = 0.71
0.05
7 : 5631189
7 : 5631268
7 : 5632281
7 : 5632593
7 : 5632954
7 : 5633068
7 : 5633086
7 : 5634164
7 : 5635134
7 : 5635820
7 : 5635953
7 : 5637739
7 : 5639063
7 : 5641113
7 : 5641966
7 : 5642705
7 : 5642784
7 : 5642848
7 : 5643021
7 : 5643158
7 : 5644940
7 : 5645051
7 : 5645728
7 : 5645778
7 : 5645984
7 : 5646023
7 : 5646121
cg01418527
cg03462130
cg13038241
cg25176823
cg02498722
cg18724565
cg22447539
cg20812117
cg15246238
cg12600030
cg24239690
cg13266018
cg08125726
cg24512685
cg23673980
cg12741436
cg00513981
cg04630639
cg09246253
cg05269678
cg05998109
cg27413316
cg09188385
cg13087761
cg06969129
cg04952186
cg23567366
GeneLoc
PromoterAssoc
CpGIsland
0.01
1e−5
mRNA / METH Corr. FDR
MITF
SOX10
TRPM1
ZEB1
AXL
0.01
1e−5
Gene / Regulator Corr. P
Type
mRNA_Expr
CopyNum
AmpDel
S2391
S2510
S2330
S2216
S2765
S2406
S2381
S2357
S2189
S2333
S2761
S2373
S2667
S2400
S2508
S2379
S2247
S2521
S2279
S2320
S2338
S2668
S2731
S2380
S2356
S2405
S2654
S2767
S2408
S2734
S2718
S2374
S2800
S2596
S2392
S2812
S2097
S2365
S2495
S2650
S2583
S2153
S2549
S2688
S2423
S2645
S2686
S2410
S2261
S2125
S2350
S2770A
S2470

EDIL3

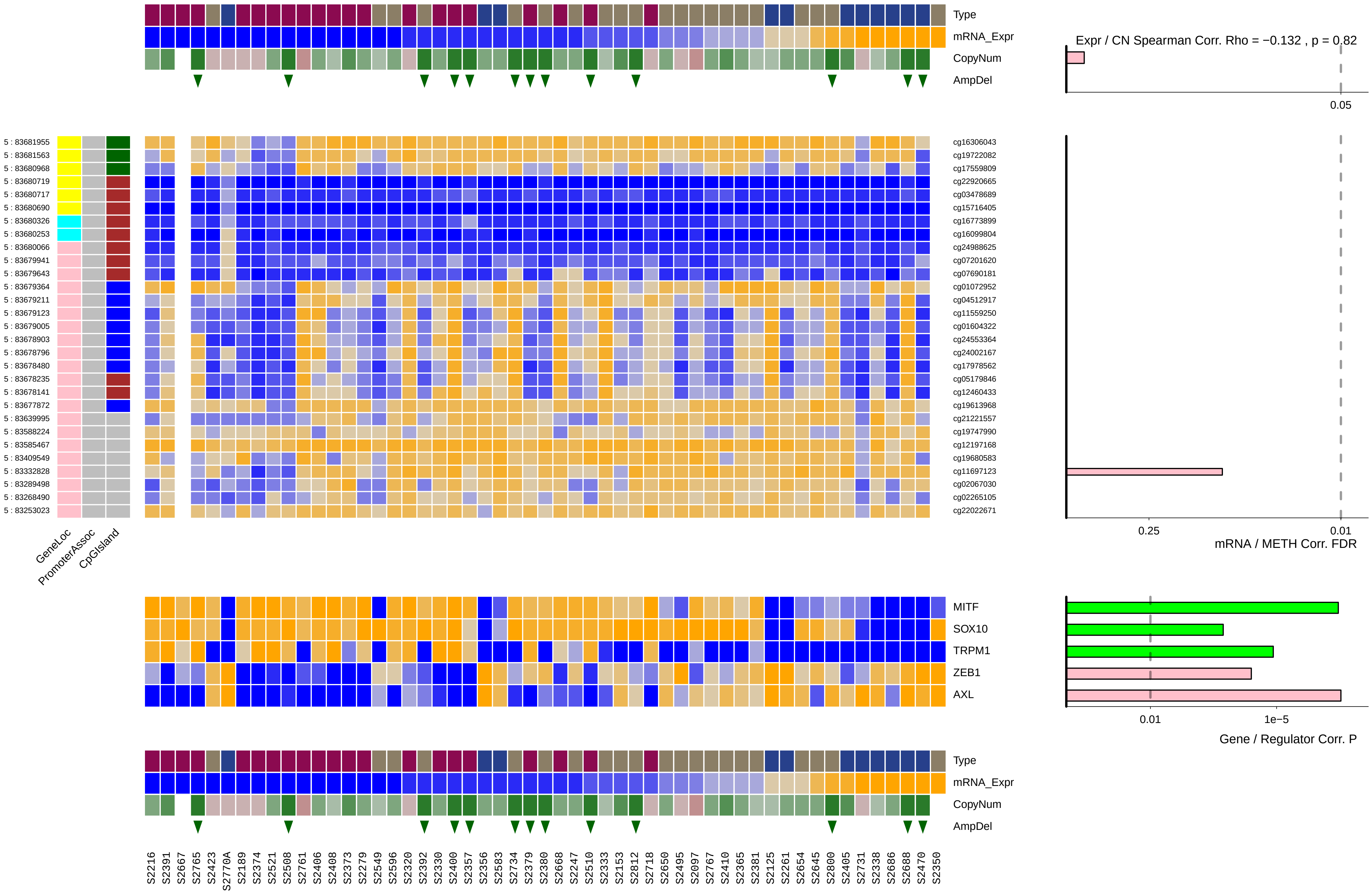

Type
mRNA_Expr
CopyNum
AmpDel
Expr / CN Spearman Corr. Rho = −0.132 , p = 0.82
0.05
5 : 83681955
5 : 83681563
5 : 83680968
5 : 83680719
5 : 83680717
5 : 83680690
5 : 83680326
5 : 83680253
5 : 83680066
5 : 83679941
5 : 83679643
5 : 83679364
5 : 83679211
5 : 83679123
5 : 83679005
5 : 83678903
5 : 83678796
5 : 83678480
5 : 83678235
5 : 83678141
5 : 83677872
5 : 83639995
5 : 83588224
5 : 83585467
5 : 83409549
5 : 83332828
5 : 83289498
5 : 83268490
5 : 83253023
cg16306043
cg19722082
cg17559809
cg22920665
cg03478689
cg15716405
cg16773899
cg16099804
cg24988625
cg07201620
cg07690181
cg01072952
cg04512917
cg11559250
cg01604322
cg24553364
cg24002167
cg17978562
cg05179846
cg12460433
cg19613968
cg21221557
cg19747990
cg12197168
cg19680583
cg11697123
cg02067030
cg02265105
cg22022671
GeneLoc
PromoterAssoc
CpGIsland
0.25
0.01
mRNA / METH Corr. FDR
MITF
SOX10
TRPM1
ZEB1
AXL
0.01
1e−5
Gene / Regulator Corr. P
Type
mRNA_Expr
CopyNum
AmpDel
S2216 S2391 S2667 S2765 S2423 S2770A S2189 S2374 S2521 S2508 S2761 S2406 S2408 S2373 S2279 S2549 S2596 S2320 S2392 S2330 S2400 S2357 S2356 S2583 S2734 S2379 S2380 S2668 S2247 S2510 S2333 S2153 S2812 S2718 S2650 S2495 S2097 S2767 S2410 S2365 S2381 S2125 S2261 S2654 S2645 S2800 S2405 S2731 S2338 S2686 S2688 S2470 S2350

VGLL3

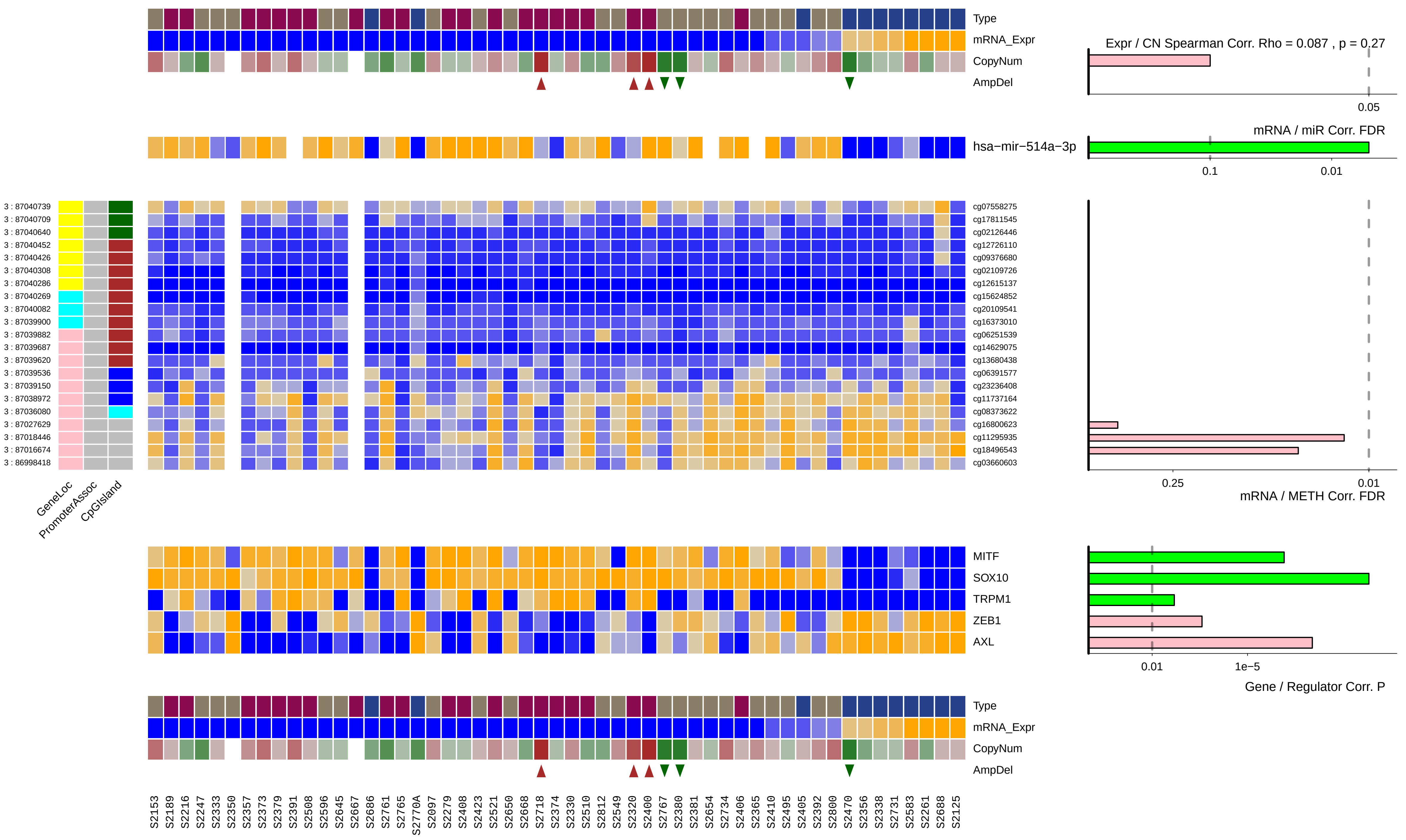

Type
mRNA_Expr
CopyNum
AmpDel
Expr / CN Spearman Corr. Rho = 0.087 , p = 0.27
0.05
hsa−mir−514a−3p
mRNA / miR Corr. FDR
0.1
0.01
3 : 87040739
3 : 87040709
3 : 87040640
3 : 87040452
3 : 87040426
3 : 87040308
3 : 87040286
3 : 87040269
3 : 87040082
3 : 87039900
3 : 87039882
3 : 87039687
3 : 87039620
3 : 87039536
3 : 87039150
3 : 87038972
3 : 87036080
3 : 87027629
3 : 87018446
3 : 87016674
3 : 86998418
cg07558275
cg17811545
cg02126446
cg12726110
cg09376680
cg02109726
cg12615137
cg15624852
cg20109541
cg16373010
cg06251539
cg14629075
cg13680438
cg06391577
cg23236408
cg11737164
cg08373622
cg16800623
cg11295935
cg18496543
cg03660603
0.25
0.01
mRNA / METH Corr. FDR
GeneLoc
PromoterAssoc
CpGIsland
MITF
SOX10
TRPM1
ZEB1
AXL
0.01
1e−5
Gene / Regulator Corr. P
Type
mRNA_Expr
CopyNum
AmpDel
S2153
S2189
S2216
S2247
S2333
S2350
S2357
S2373
S2379
S2391
S2508
S2596
S2645
S2667
S2686
S2761
S2765
S2770A
S2097
S2279
S2408
S2423
S2521
S2650
S2668
S2718
S2374
S2330
S2510
S2812
S2549
S2320
S2400
S2767
S2380
S2381
S2654
S2734
S2406
S2365
S2410
S2495
S2405
S2392
S2800
S2470
S2356
S2338
S2731
S2583
S2261
S2688
S2125

DDAH1

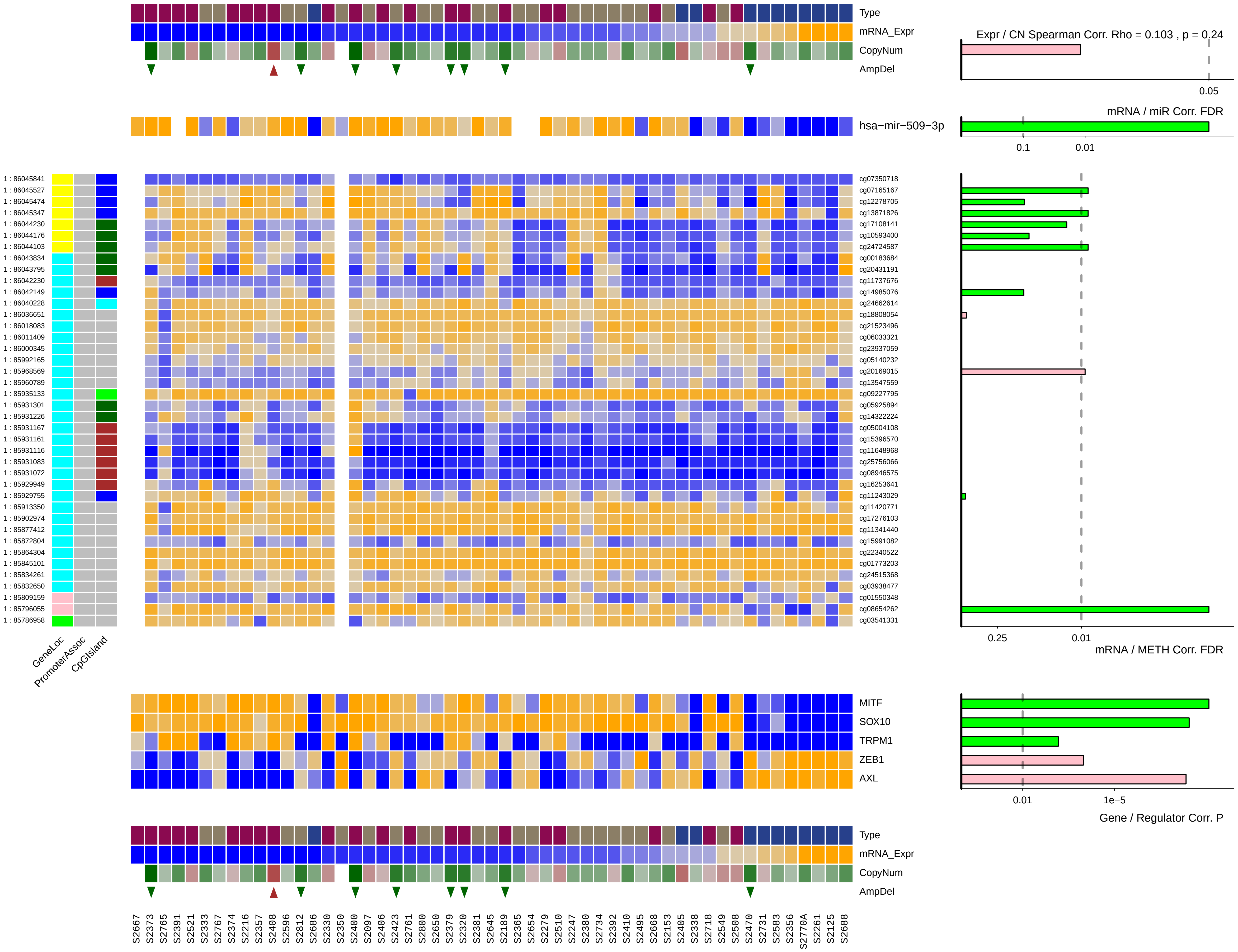

Type
mRNA_Expr
CopyNum
AmpDel
Expr / CN Spearman Corr. Rho = 0.103 , p = 0.24
0.05
hsa−mir−509−3p
mRNA / miR Corr. FDR
0.1
0.01
1 : 86045841
1 : 86045527
1 : 86045474
1 : 86045347
1 : 86044230
1 : 86044176
1 : 86044103
1 : 86043834
1 : 86043795
1 : 86042230
1 : 86042149
1 : 86040228
1 : 86036651
1 : 86018083
1 : 86011409
1 : 86000345
1 : 85992165
1 : 85968569
1 : 85960789
1 : 85935133
1 : 85931301
1 : 85931226
1 : 85931167
1 : 85931161
1 : 85931116
1 : 85931083
1 : 85931072
1 : 85929949
1 : 85929755
1 : 85913350
1 : 85902974
1 : 85877412
1 : 85872804
1 : 85864304
1 : 85845101
1 : 85834261
1 : 85832650
1 : 85809159
1 : 85796055
1 : 85786958
cg07350718
cg07165167
cg12278705
cg13871826
cg17108141
cg10593400
cg24724587
cg00183684
cg20431191
cg11737676
cg14985076
cg24662614
cg18808054
cg21523496
cg06033321
cg23937059
cg05140232
cg20169015
cg13547559
cg09227795
cg05925894
cg14322224
cg05004108
cg15396570
cg11648968
cg25756066
cg08946575
cg16253641
cg11243029
cg11420771
cg17276103
cg11341440
cg15991082
cg22340522
cg01773203
cg24515368
cg03938477
cg01550348
cg08654262
cg03541331
GeneLoc
PromoterAssoc
CpGIsland
0.25
0.01
mRNA / METH Corr. FDR
MITF
SOX10
TRPM1
ZEB1
AXL
0.01
1e−5
Gene / Regulator Corr. P
Type
mRNA_Expr
CopyNum
AmpDel
S2667 S2373 S2765 S2391 S2521 S2333 S2767 S2374 S2216 S2357 S2408 S2596 S2812 S2686 S2330 S2350 S2400 S2097 S2406 S2423 S2761 S2800 S2650 S2379 S2320 S2381 S2645 S2189 S2365 S2654 S2279 S2510 S2247 S2380 S2734 S2392 S2410 S2495 S2668 S2153 S2405 S2338 S2718 S2549 S2508 S2470 S2731 S2583 S2356 S2770A S2261 S2125 S2688

ATP8B1

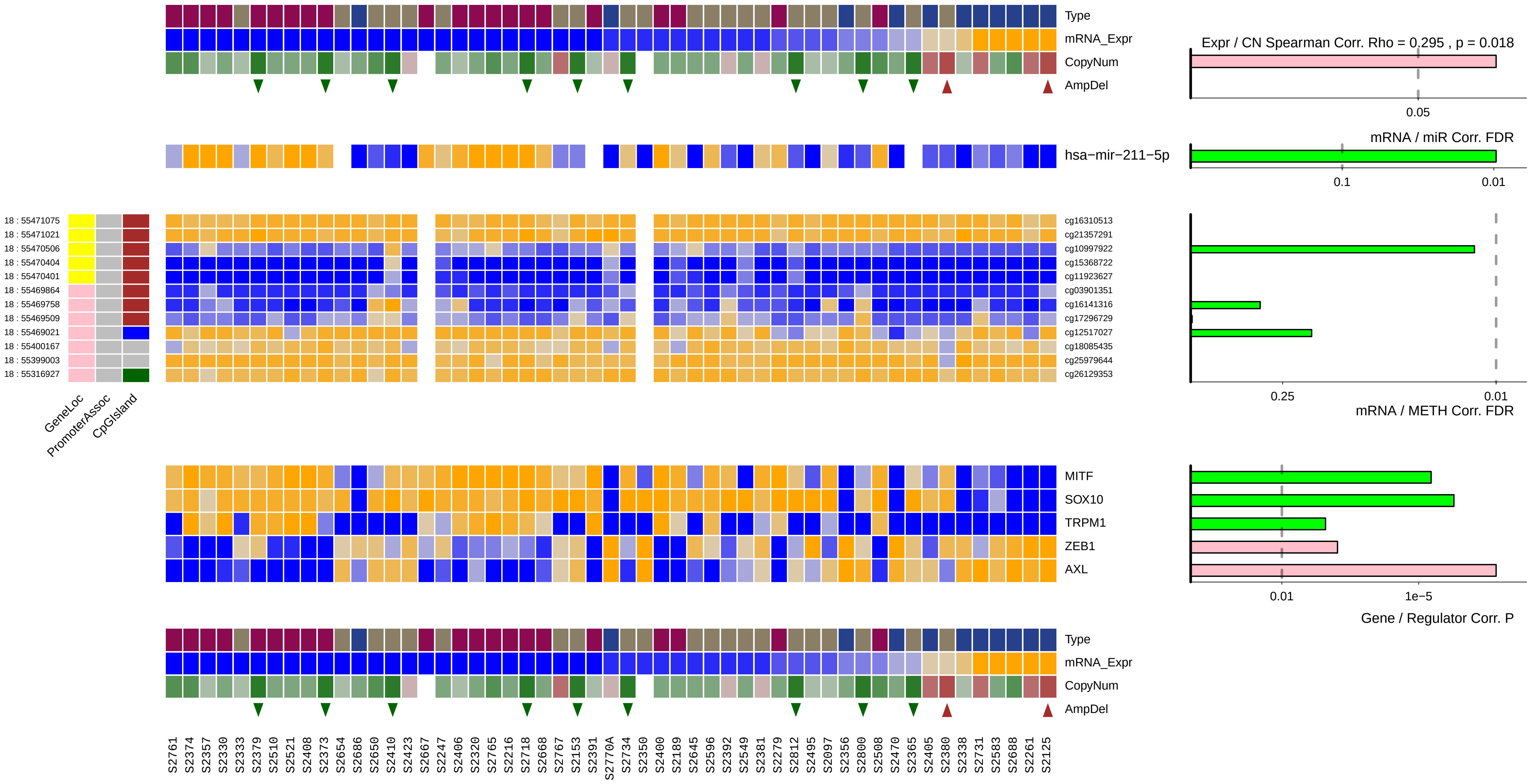
Type
mRNA_Expr
CopyNum
AmpDel
Expr / CN Spearman Corr. Rho = 0.295 , p = 0.018
0.05
hsa−mir−211−5p
mRNA / miR Corr. FDR
0.1
0.01
18 : 55471075
18 : 55471021
18 : 55470506
18 : 55470404
18 : 55470401
18 : 55469864
18 : 55469758
18 : 55469509
18 : 55469021
18 : 55400167
18 : 55399003
18 : 55316927
GeneLoc
PromoterAssoc
CpGIsland
cg16310513
cg21357291
cg10997922
cg15368722
cg11923627
cg03901351
cg16141316
cg17296729
cg12517027
cg18085435
cg25979644
cg26129353
0.25
0.01
mRNA / METH Corr. FDR
MITF
SOX10
TRPM1
ZEB1
AXL
0.01
1e−5
Gene / Regulator Corr. P
Type
mRNA_Expr
CopyNum
AmpDel
S2761 S2374 S2357 S2330 S2333 S2379 S2510 S2521 S2408 S2373 S2654 S2686 S2650 S2410 S2423 S2667 S2247 S2406 S2320 S2765 S2216 S2718 S2668 S2767 S2153 S2391 S2770A S2734 S2350 S2400 S2189 S2645 S2596 S2392 S2549 S2381 S2279 S2812 S2495 S2097 S2356 S2800 S2508 S2470 S2365 S2405 S2380 S2338 S2731 S2583 S2688 S2261 S2125

AMOTL2

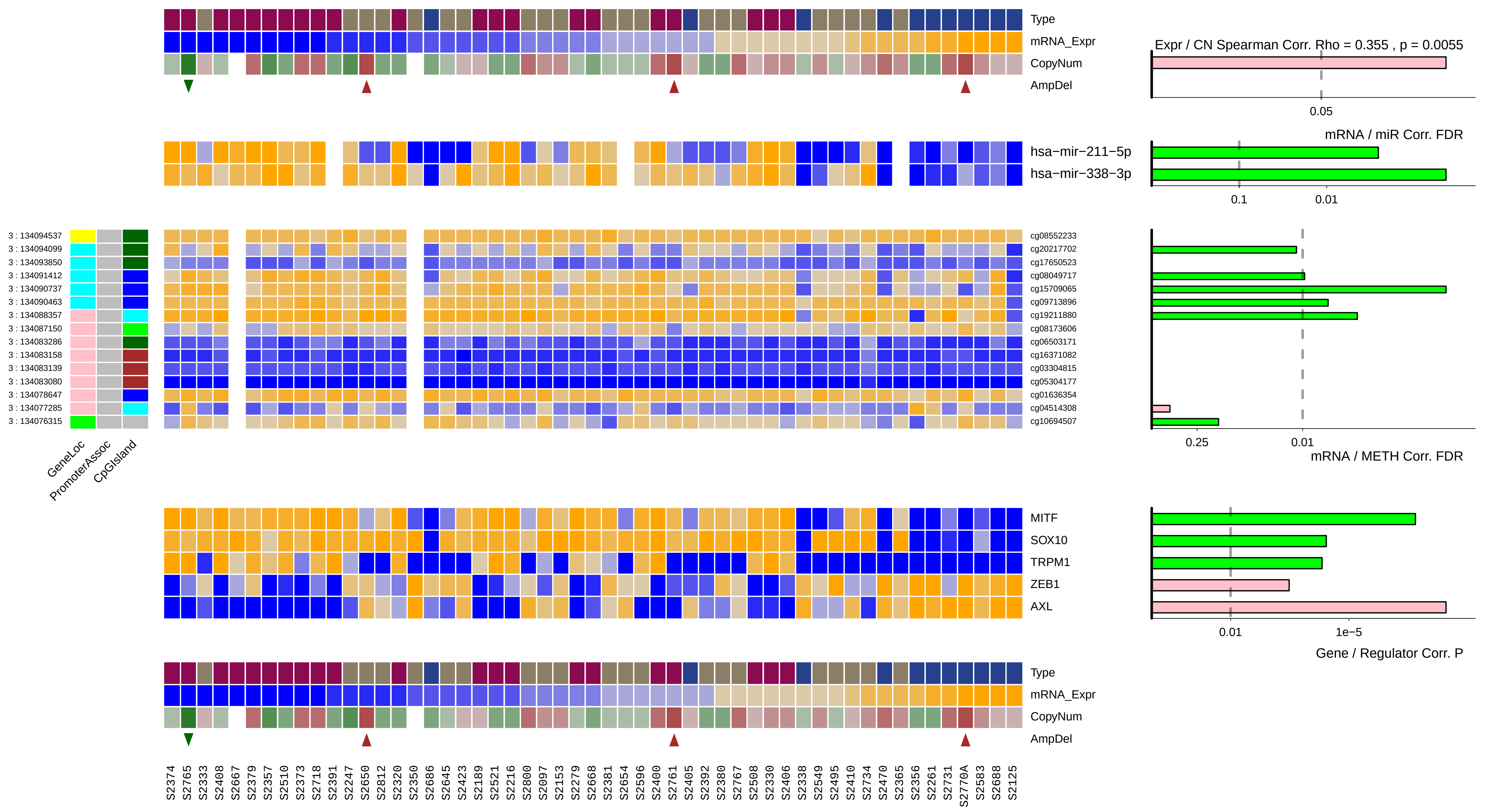

Type
mRNA_Expr
CopyNum
AmpDel
Expr / CN Spearman Corr. Rho = 0.355 , p = 0.0055
0.05
mRNA / miR Corr. FDR
hsa−mir−211−5p
hsa−mir−338−3p
0.1
0.01
GeneLoc
PromoterAssoc
CpGIsland
0.25
0.01
mRNA / METH Corr. FDR
MITF
SOX10
TRPM1
ZEB1
AXL
0.01
1e−5
Gene / Regulator Corr. P
Type
mRNA_Expr
CopyNum
AmpDel

LMO7

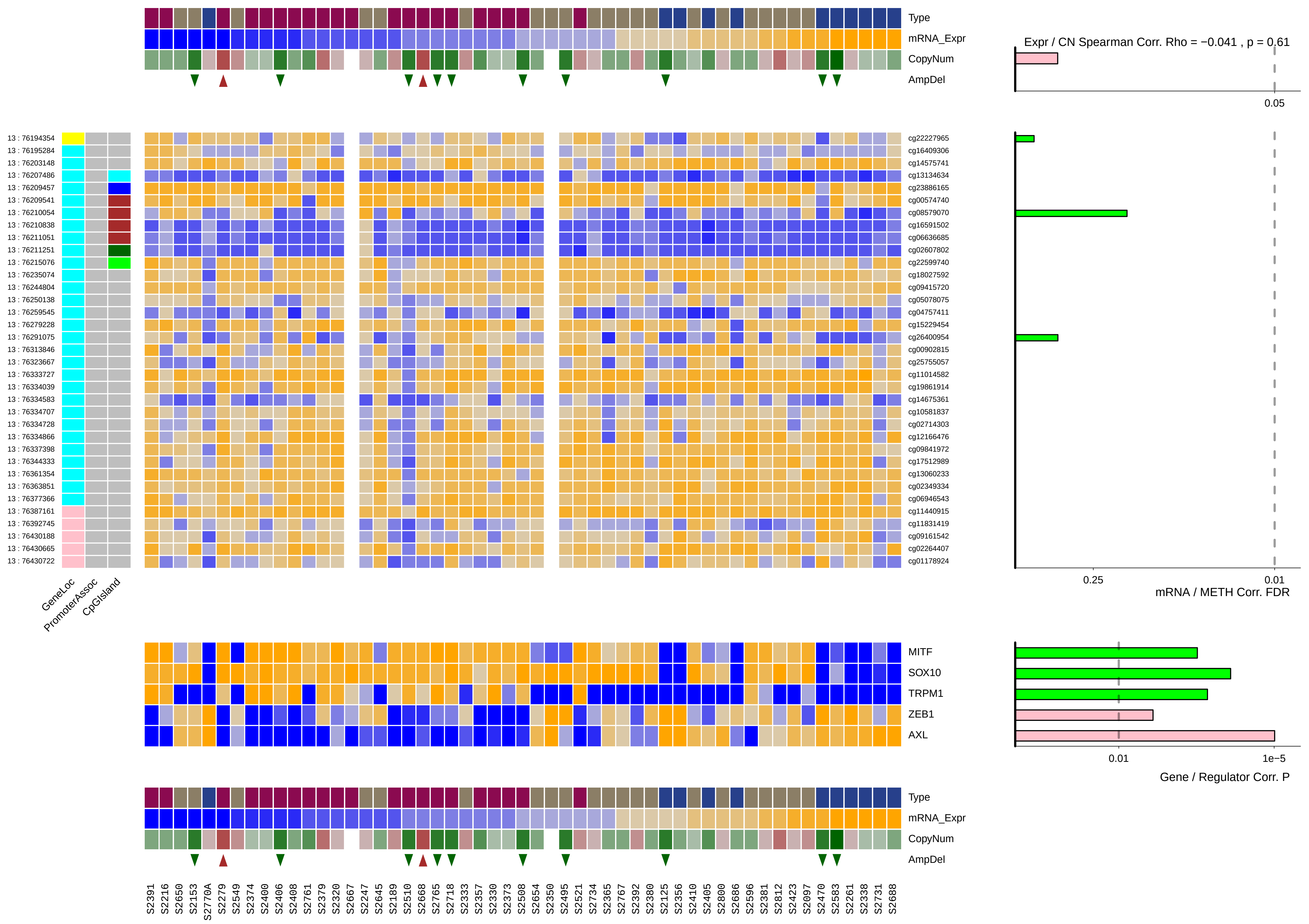

Type
mRNA_Expr
CopyNum
AmpDel
Expr / CN Spearman Corr. Rho = −0.041 , p = 0.61
0.05
13 : 76194354
13 : 76195284
13 : 76203148
13 : 76207486
13 : 76209457
13 : 76209541
13 : 76210054
13 : 76210838
13 : 76211051
13 : 76211251
13 : 76215076
13 : 76235074
13 : 76244804
13 : 76250138
13 : 76259545
13 : 76279228
13 : 76291075
13 : 76313846
13 : 76323667
13 : 76333727
13 : 76334039
13 : 76334583
13 : 76334707
13 : 76334728
13 : 76334866
13 : 76337398
13 : 76344333
13 : 76361354
13 : 76363851
13 : 76377366
13 : 76387161
13 : 76392745
13 : 76430188
13 : 76430665
13 : 76430722
cg22227965
cg16409306
cg14575741
cg13134634
cg23886165
cg00574740
cg08579070
cg16591502
cg06636685
cg02607802
cg22599740
cg18027592
cg09415720
cg05078075
cg04757411
cg15229454
cg26400954
cg00902815
cg25755057
cg11014582
cg19861914
cg14675361
cg10581837
cg02714303
cg12166476
cg09841972
cg17512989
cg13060233
cg02349334
cg06946543
cg11440915
cg11831419
cg09161542
cg02264407
cg01178924
0.25
0.01
mRNA / METH Corr. FDR
GeneLoc
PromoterAssoc
CpGIsland
MITF
SOX10
TRPM1
ZEB1
AXL
0.01
1e−5
Gene / Regulator Corr. P
Type
mRNA_Expr
CopyNum
AmpDel
S2391 S2216 S2650 S2153 S2770A S2279 S2549 S2374 S2400 S2406 S2408 S2761 S2379 S2320 S2667 S2247 S2645 S2189 S2510 S2668 S2765 S2718 S2333 S2357 S2330 S2373 S2508 S2654 S2350 S2495 S2521 S2734 S2365 S2767 S2392 S2380 S2125 S2356 S2410 S2405 S2800 S2686 S2596 S2381 S2812 S2423 S2097 S2470 S2583 S2261 S2338 S2731 S2688

RAC2

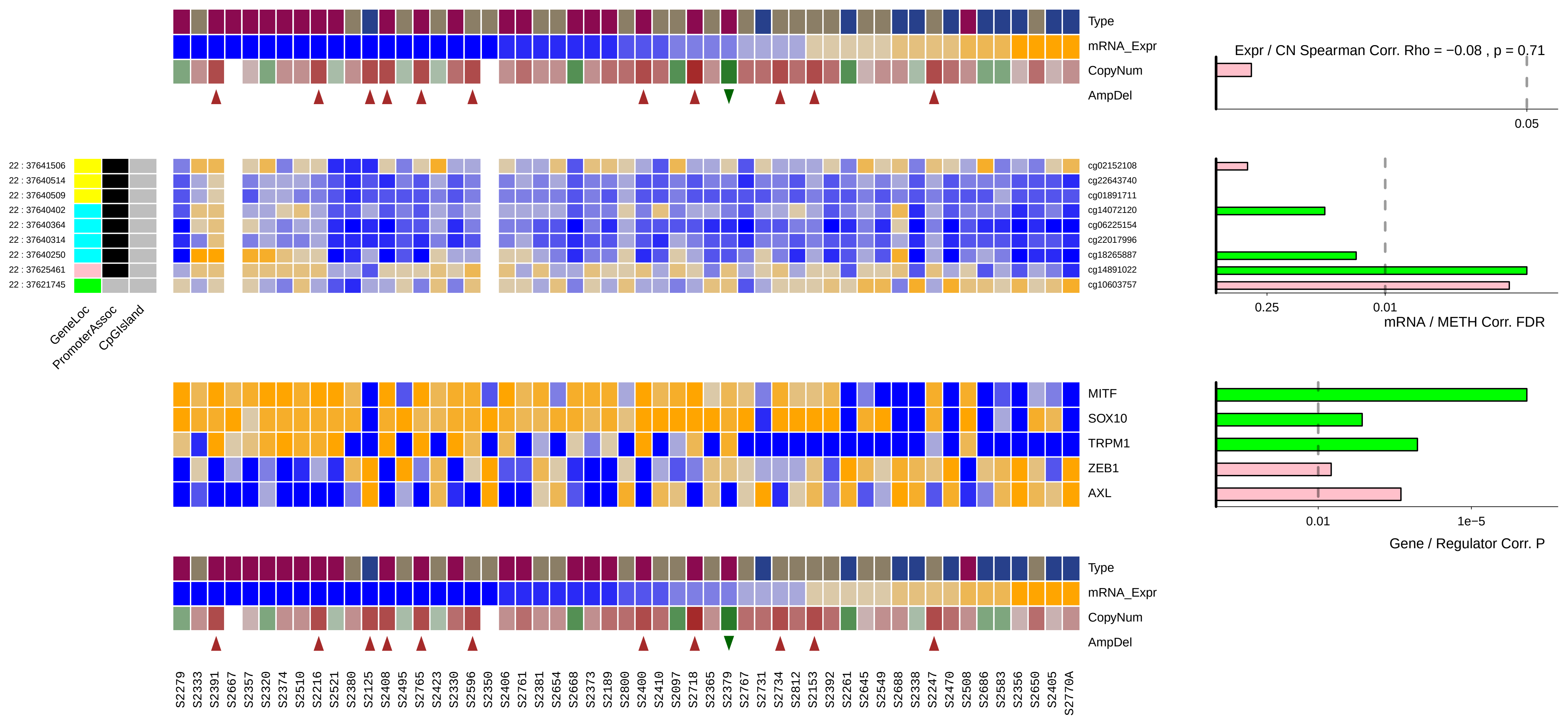

Type
mRNA_Expr
CopyNum
AmpDel
Expr / CN Spearman Corr. Rho = −0.08 , p = 0.71
0.05
22 : 37641506
22 : 37640514
22 : 37640509
22 : 37640402
22 : 37640364
22 : 37640314
22 : 37640250
22 : 37625461
22 : 37621745
GeneLoc
PromoterAssoc
CpGIsland
cg02152108
cg22643740
cg01891711
cg14072120
cg06225154
cg22017996
cg18265887
cg14891022
cg10603757
0.25
0.01
mRNA / METH Corr. FDR
MITF
SOX10
TRPM1
ZEB1
AXL
0.01
1e−5
Gene / Regulator Corr. P
S2279 S2333 S2391 S2667 S2357 S2320 S2374 S2510 S2216 S2521 S2380 S2125 S2408 S2495 S2765 S2423 S2330 S2596 S2350 S2406 S2761 S2381 S2654 S2668 S2373 S2189 S2800 S2400 S2410 S2097 S2718 S2365 S2379 S2767 S2731 S2734 S2812 S2153 S2392 S2261 S2645 S2549 S2688 S2338 S2247 S2470 S2508 S2686 S2583 S2356 S2650 S2405 S2770A

ARHGEF28

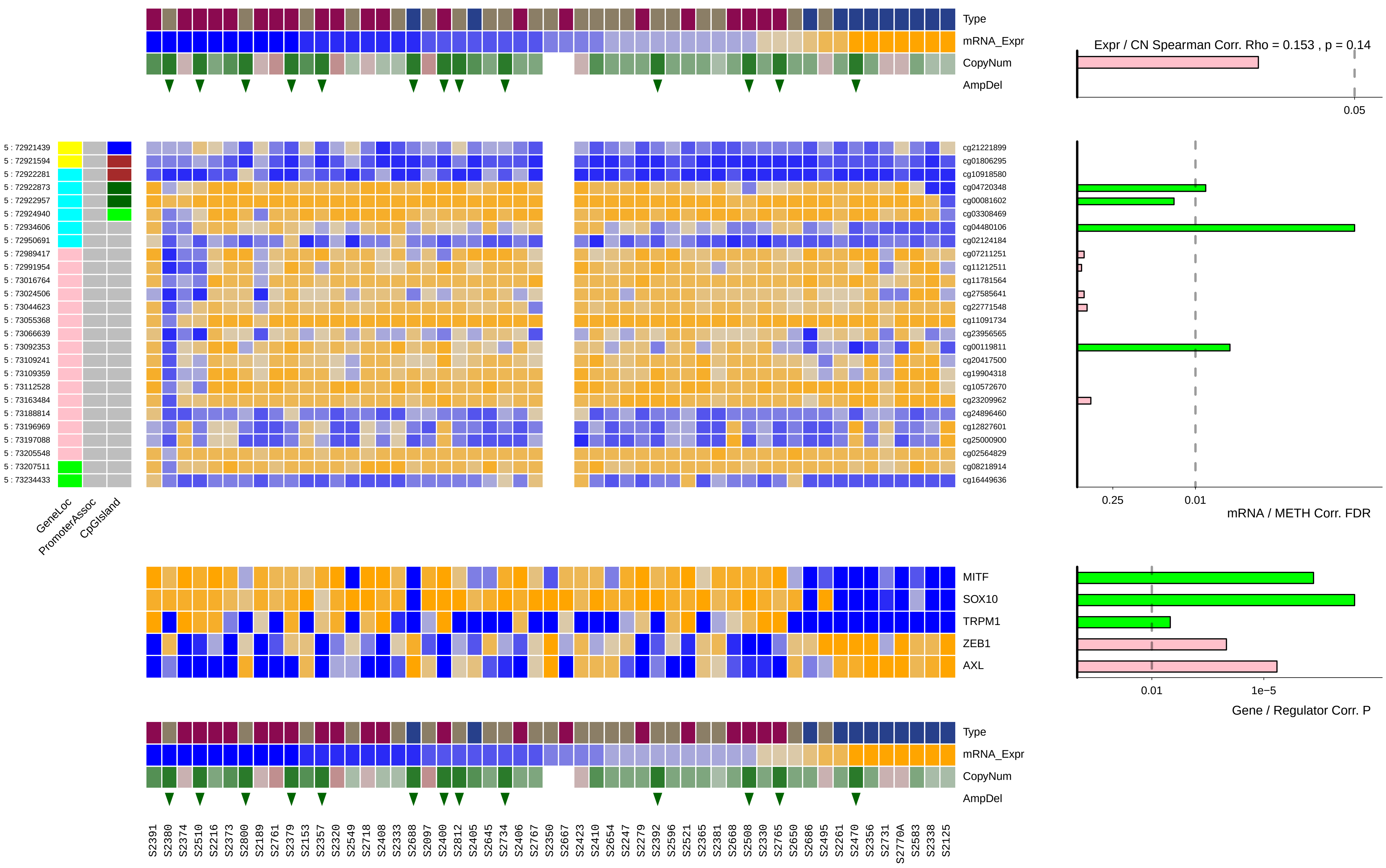

Type
mRNA_Expr
CopyNum
AmpDel
Expr / CN Spearman Corr. Rho = 0.153 , p = 0.14
0.05
GeneLoc
PromoterAssoc
CpGIsland
0.25
0.01
mRNA / METH Corr. FDR
MITF
SOX10
TRPM1
ZEB1
AXL
0.01
1e−5
Gene / Regulator Corr. P

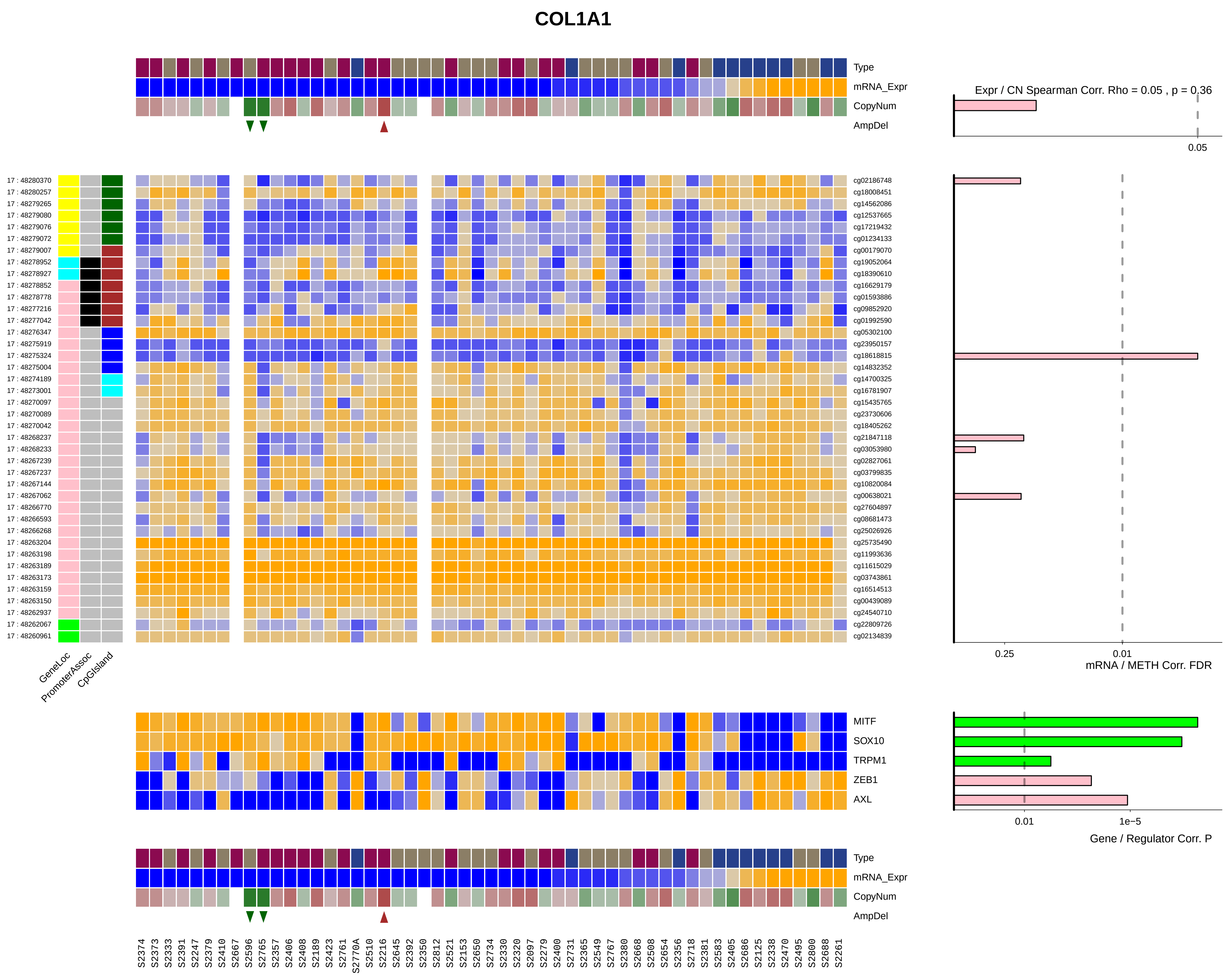
COL1A1
Type
mRNA_Expr
CopyNum
AmpDel
Expr / CN Spearman Corr. Rho = 0.05 , p = 0.36
0.05
17 : 48280370
17 : 48280257
17 : 48279265
17 : 48279080
17 : 48279076
17 : 48279072
17 : 48279007
17 : 48278952
17 : 48278927
17 : 48278852
17 : 48278778
17 : 48277216
17 : 48277042
17 : 48276347
17 : 48275919
17 : 48275324
17 : 48275004
17 : 48274189
17 : 48273001
17 : 48270097
17 : 48270089
17 : 48270042
17 : 48268237
17 : 48268233
17 : 48267239
17 : 48267237
17 : 48267144
17 : 48267062
17 : 48266770
17 : 48266593
17 : 48266268
17 : 48263204
17 : 48263198
17 : 48263189
17 : 48263173
17 : 48263159
17 : 48263150
17 : 48262937
17 : 48262067
17 : 48260961
cg02186748
cg18008451
cg14562086
cg12537665
cg17219432
cg01234133
cg00179070
cg19052064
cg18390610
cg16629179
cg01593886
cg09852920
cg01992590
cg05302100
cg23950157
cg18618815
cg14832352
cg14700325
cg16781907
cg15435765
cg23730606
cg18405262
cg21847118
cg03053980
cg02827061
cg03799835
cg10820084
cg00638021
cg27604897
cg08681473
cg25026926
cg25735490
cg11993636
cg11615029
cg03743861
cg16514513
cg00439089
cg24540710
cg22809726
cg02134839
0.25
0.01
mRNA / METH Corr. FDR
GeneLoc
PromoterAssoc
CpGIsland
MITF
SOX10
TRPM1
ZEB1
AXL
0.01
1e−5
Gene / Regulator Corr. P
Type
mRNA_Expr
CopyNum
AmpDel
S2374
S2373
S2333
S2391
S2247
S2379
S2410
S2667
S2596
S2765
S2357
S2406
S2408
S2189
S2423
S2761
S2770A
S2510
S2216
S2645
S2392
S2350
S2812
S2521
S2153
S2650
S2734
S2330
S2320
S2097
S2279
S2400
S2731
S2365
S2549
S2767
S2380
S2668
S2508
S2654
S2356
S2718
S2381
S2583
S2405
S2686
S2125
S2338
S2470
S2495
S2800
S2688
S2261

LOX

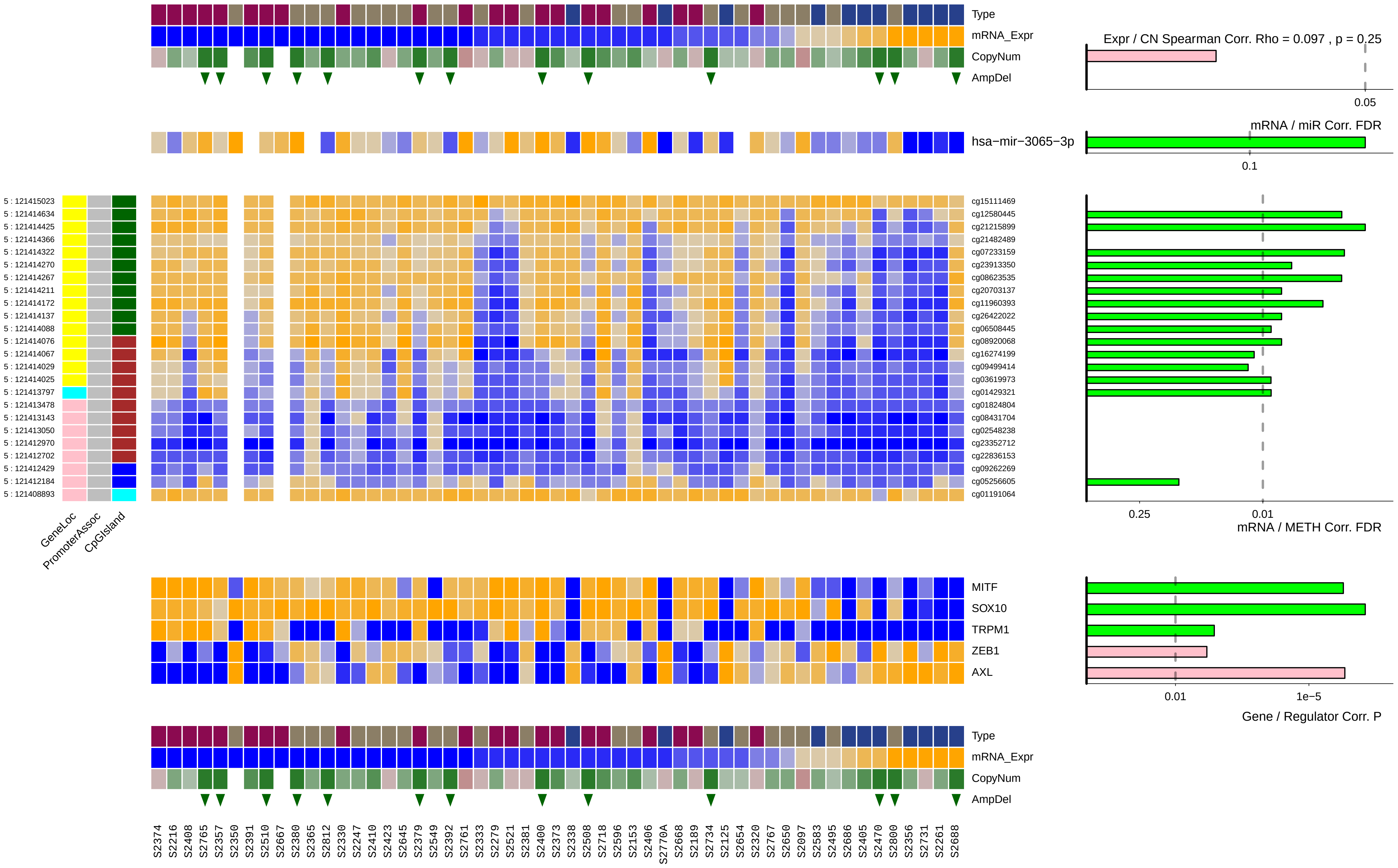

Type
mRNA_Expr
CopyNum
AmpDel
Expr / CN Spearman Corr. Rho = 0.097 , p = 0.25
0.05
hsa−mir−3065−3p
mRNA / miR Corr. FDR
0.1
mRNA / METH Corr. FDR
0.25
0.01
GeneLoc
PromoterAssoc
CpGIsland
MITF
SOX10
TRPM1
ZEB1
AXL
Gene / Regulator Corr. P
0.01
1e−5

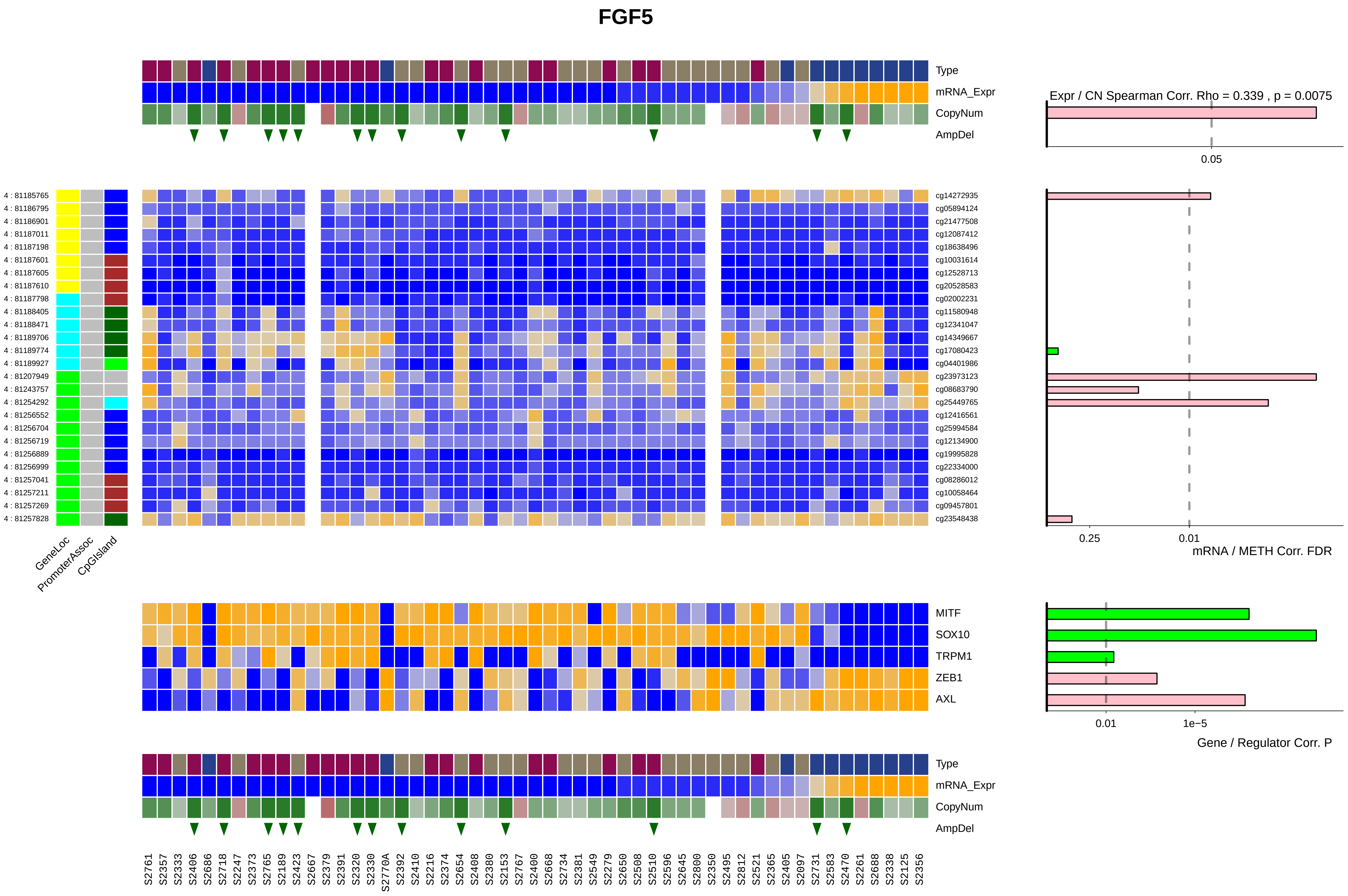
FGF5
Type
mRNA_Expr
CopyNum
AmpDel
Expr / CN Spearman Corr. Rho = 0.339 , p = 0.0075
0.05
4 : 81185765
4 : 81186795
4 : 81186901
4 : 81187011
4 : 81187198
4 : 81187601
4 : 81187605
4 : 81187610
4 : 81187798
4 : 81188405
4 : 81188471
4 : 81189706
4 : 81189774
4 : 81189927
4 : 81207949
4 : 81243757
4 : 81254292
4 : 81256552
4 : 81256704
4 : 81256719
4 : 81256889
4 : 81256999
4 : 81257041
4 : 81257211
4 : 81257269
4 : 81257828
cg14272935
cg05894124
cg21477508
cg12087412
cg18638496
cg10031614
cg12528713
cg20528583
cg02002231
cg11580948
cg12341047
cg14349667
cg17080423
cg04401986
cg23973123
cg08683790
cg25449765
cg12416561
cg25994584
cg12134900
cg19995828
cg22334000
cg08286012
cg10058464
cg09457801
cg23548438
0.25
0.01
mRNA / METH Corr. FDR
GeneLoc
PromoterAssoc
CpGIsland
MITF
SOX10
TRPM1
ZEB1
AXL
0.01
1e−5
Gene / Regulator Corr. P
Type
mRNA_Expr
CopyNum
AmpDel
S2761 S2357 S2333 S2406 S2686 S2718 S2247 S2373 S2765 S2189 S2423 S2667 S2379 S2391 S2320 S2330 S2770A S2392 S2410 S2216 S2374 S2654 S2408 S2380 S2153 S2767 S2400 S2668 S2734 S2381 S2549 S2279 S2650 S2508 S2510 S2596 S2645 S2800 S2350 S2495 S2812 S2521 S2365 S2405 S2097 S2731 S2583 S2470 S2261 S2688 S2338 S2125 S2356

LRIG1

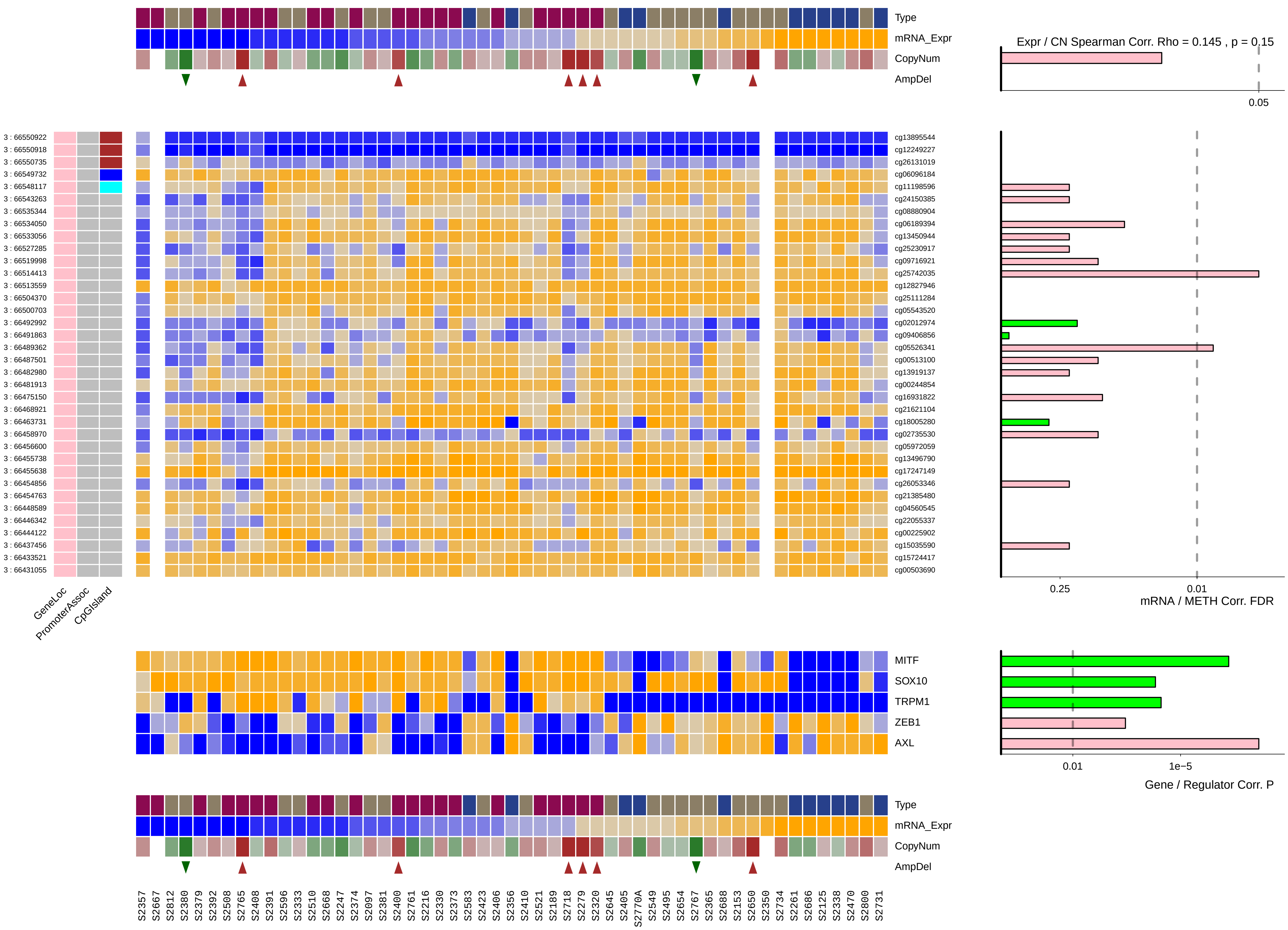
Type
mRNA_Expr
CopyNum
AmpDel
Expr / CN Spearman Corr. Rho = 0.145 , p = 0.15
0.05
GeneLoc
PromoterAssoc
CpGIsland
0.25
0.01
mRNA / METH Corr. FDR
MITF
SOX10
TRPM1
ZEB1
AXL
0.01
1e−5
Gene / Regulator Corr. P

SLC38A1

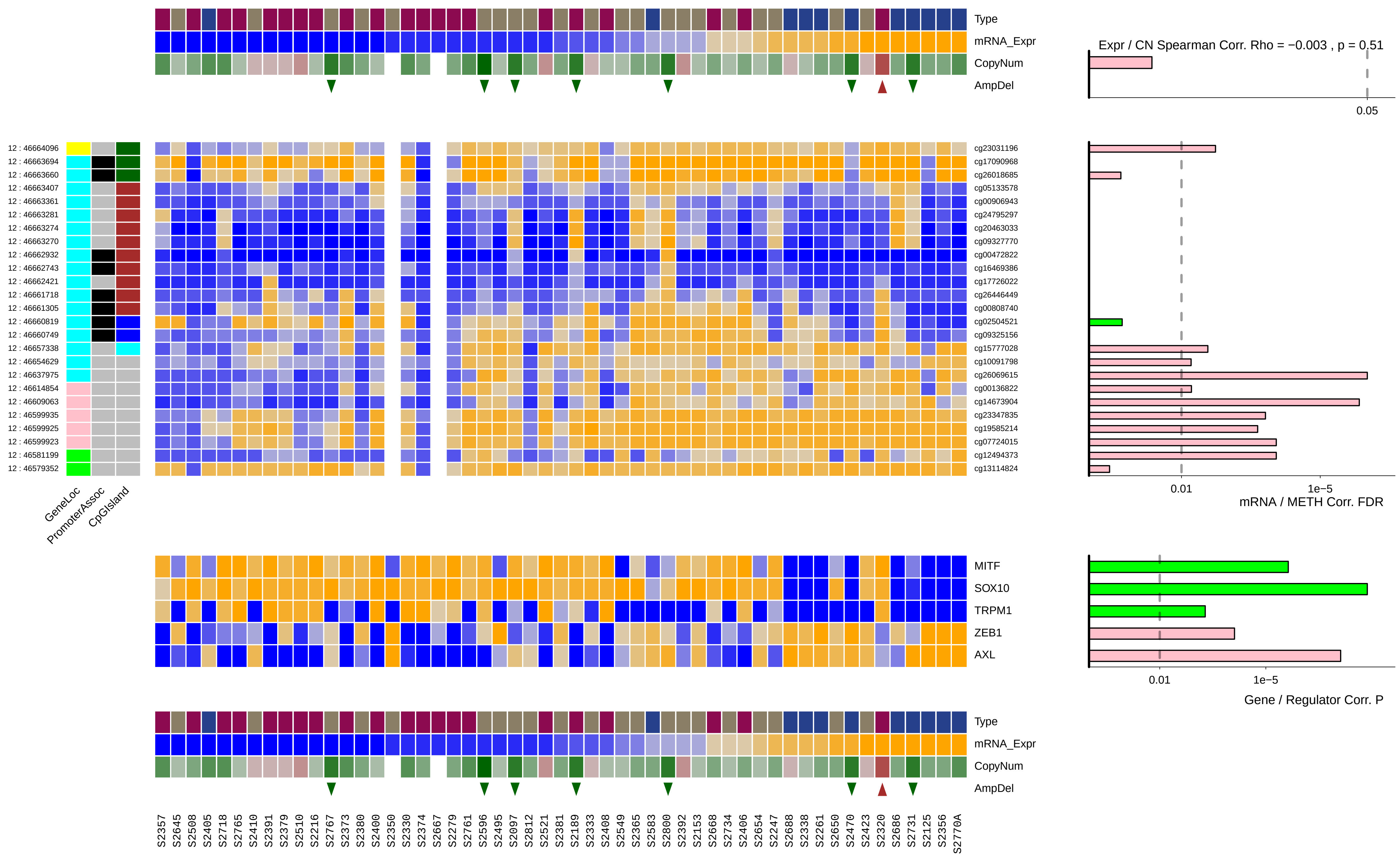

Type
mRNA_Expr
CopyNum
AmpDel
Expr / CN Spearman Corr. Rho = −0.003 , p = 0.51
0.05
12 : 46664096
12 : 46663694
12 : 46663660
12 : 46663407
12 : 46663361
12 : 46663281
12 : 46663274
12 : 46663270
12 : 46662932
12 : 46662743
12 : 46662421
12 : 46661718
12 : 46661305
12 : 46660819
12 : 46660749
12 : 46657338
12 : 46654629
12 : 46637975
12 : 46614854
12 : 46609063
12 : 46599935
12 : 46599925
12 : 46599923
12 : 46581199
12 : 46579352
cg23031196
cg17090968
cg26018685
cg05133578
cg00906943
cg24795297
cg20463033
cg09327770
cg00472822
cg16469386
cg17726022
cg26446449
cg00808740
cg02504521
cg09325156
cg15777028
cg10091798
cg26069615
cg00136822
cg14673904
cg23347835
cg19585214
cg07724015
cg12494373
cg13114824
0.01
1e−5
mRNA / METH Corr. FDR
GeneLoc
PromoterAssoc
CpGIsland
MITF
SOX10
TRPM1
ZEB1
AXL
0.01
1e−5
Gene / Regulator Corr. P
Type
mRNA_Expr
CopyNum
AmpDel
S2357
S2645
S2508
S2405
S2718
S2765
S2410
S2391
S2379
S2510
S2216
S2767
S2373
S2380
S2400
S2350
S2330
S2374
S2667
S2279
S2761
S2596
S2495
S2097
S2812
S2521
S2381
S2189
S2333
S2408
S2549
S2365
S2583
S2800
S2392
S2153
S2668
S2734
S2406
S2654
S2247
S2688
S2338
S2261
S2650
S2470
S2423
S2320
S2686
S2731
S2125
S2356
S2770A

CITED2

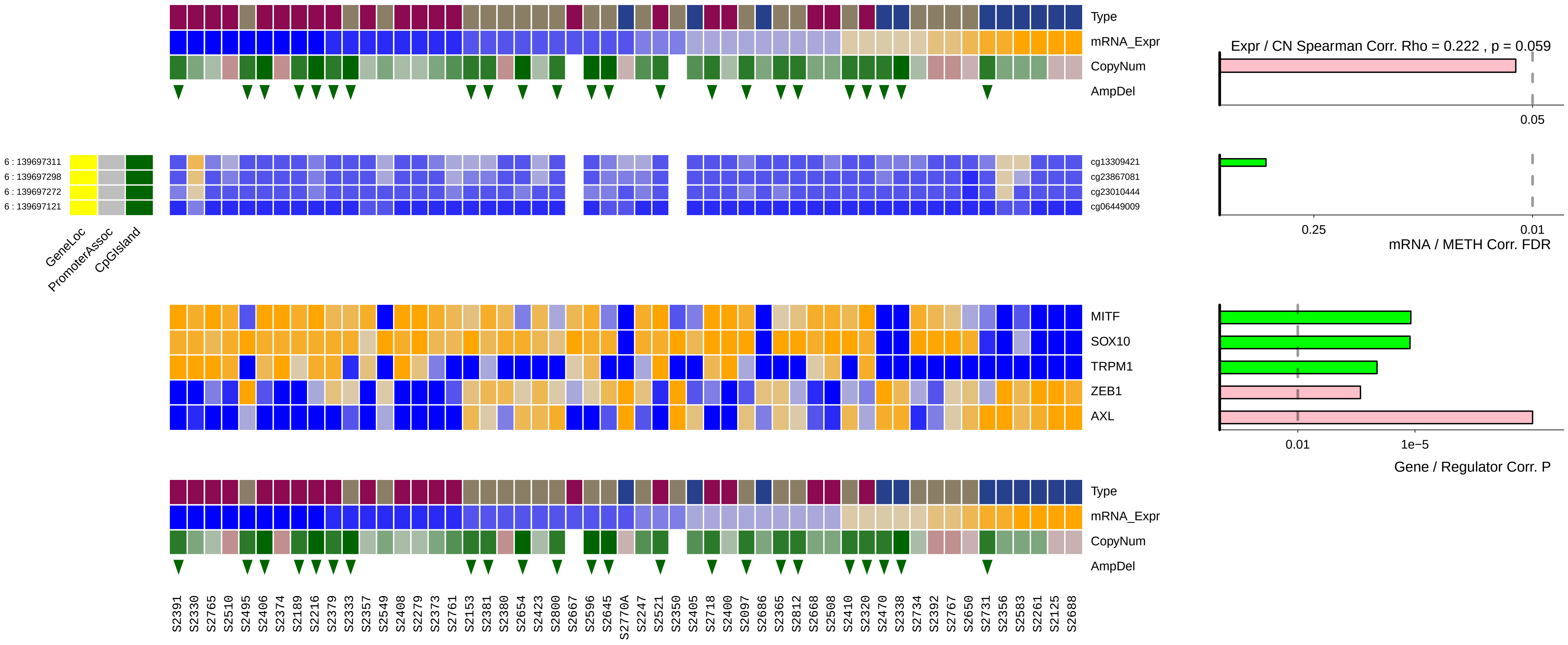

Type
mRNA_Expr
CopyNum
AmpDel
Expr / CN Spearman Corr. Rho = 0.222 , p = 0.059
0.05
6 : 139697311
6 : 139697298
6 : 139697272
6 : 139697121
cg13309421
cg23867081
cg23010444
cg06449009
GeneLoc
PromoterAssoc
CpGIsland
0.25
0.01
mRNA / METH Corr. FDR
MITF
SOX10
TRPM1
ZEB1
AXL
0.01
1e−5
Gene / Regulator Corr. P
Type
mRNA_Expr
CopyNum
AmpDel
S2391 S2330 S2765 S2510 S2495 S2406 S2374 S2189 S2216 S2379 S2333 S2357 S2549 S2408 S2279 S2373 S2761 S2153 S2381 S2380 S2654 S2423 S2800 S2667 S2596 S2645 S2770A S2247 S2521 S2350 S2405 S2718 S2400 S2097 S2686 S2365 S2812 S2668 S2508 S2410 S2320 S2470 S2338 S2734 S2392 S2767 S2650 S2731 S2356 S2583 S2261 S2125 S2688

PDE1C

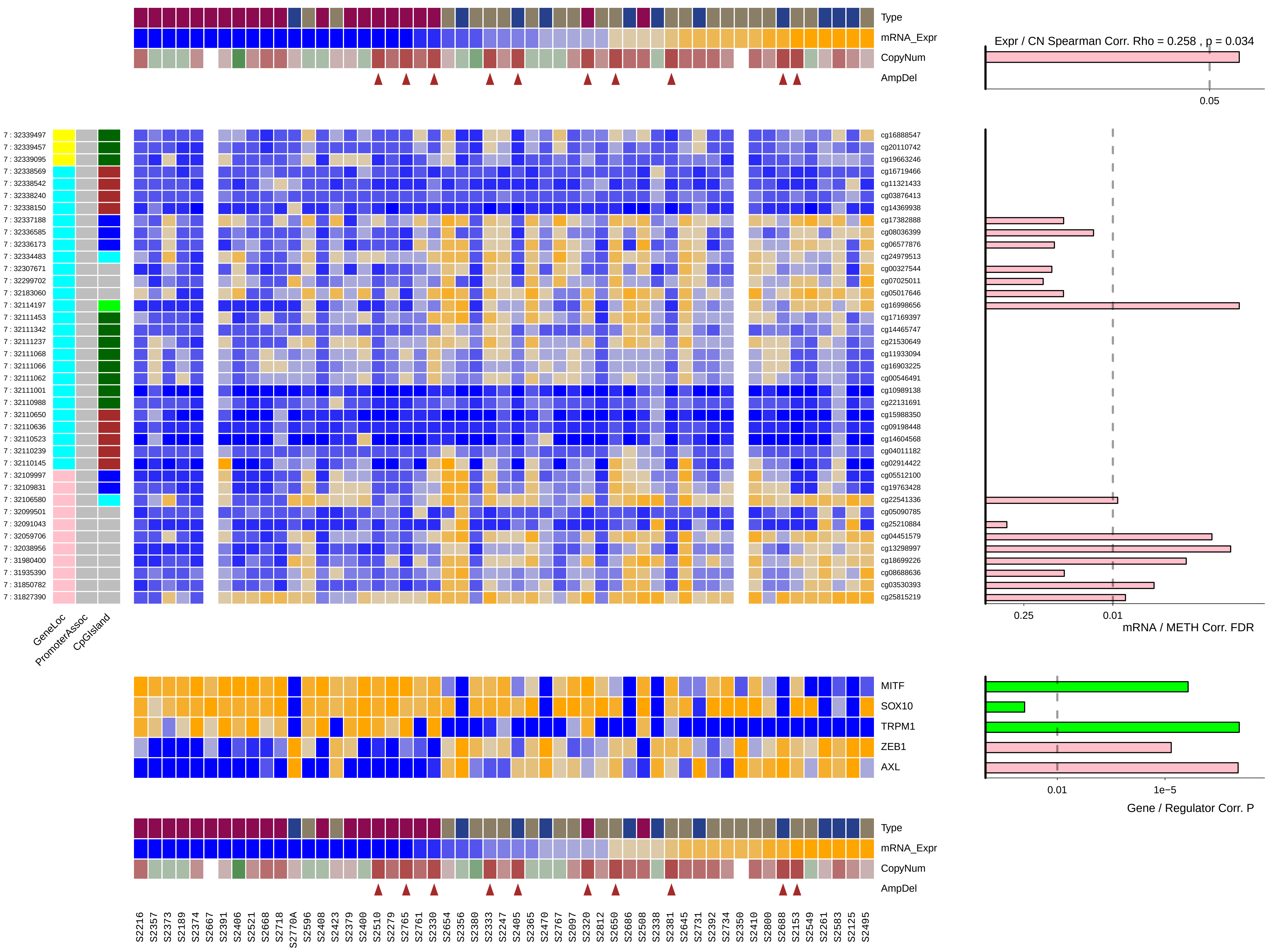

Type
mRNA_Expr
CopyNum
AmpDel
Expr / CN Spearman Corr. Rho = 0.258 , p = 0.034
0.05
GeneLoc
PromoterAssoc
CpGIsland
0.25
0.01
mRNA / METH Corr. FDR
MITF
SOX10
TRPM1
ZEB1
AXL
0.01
1e−5
Gene / Regulator Corr. P
S2216 S2357 S2373 S2189 S2374 S2667 S2391 S2406 S2521 S2668 S2718 S2770A S2596 S2408 S2423 S2379 S2400 S2510 S2279 S2765 S2761 S2330 S2654 S2356 S2380 S2333 S2247 S2405 S2365 S2470 S2767 S2097 S2320 S2812 S2650 S2686 S2508 S2338 S2381 S2645 S2731 S2392 S2734 S2350 S2410 S2800 S2688 S2153 S2549 S2261 S2583 S2125 S2495

CDH2

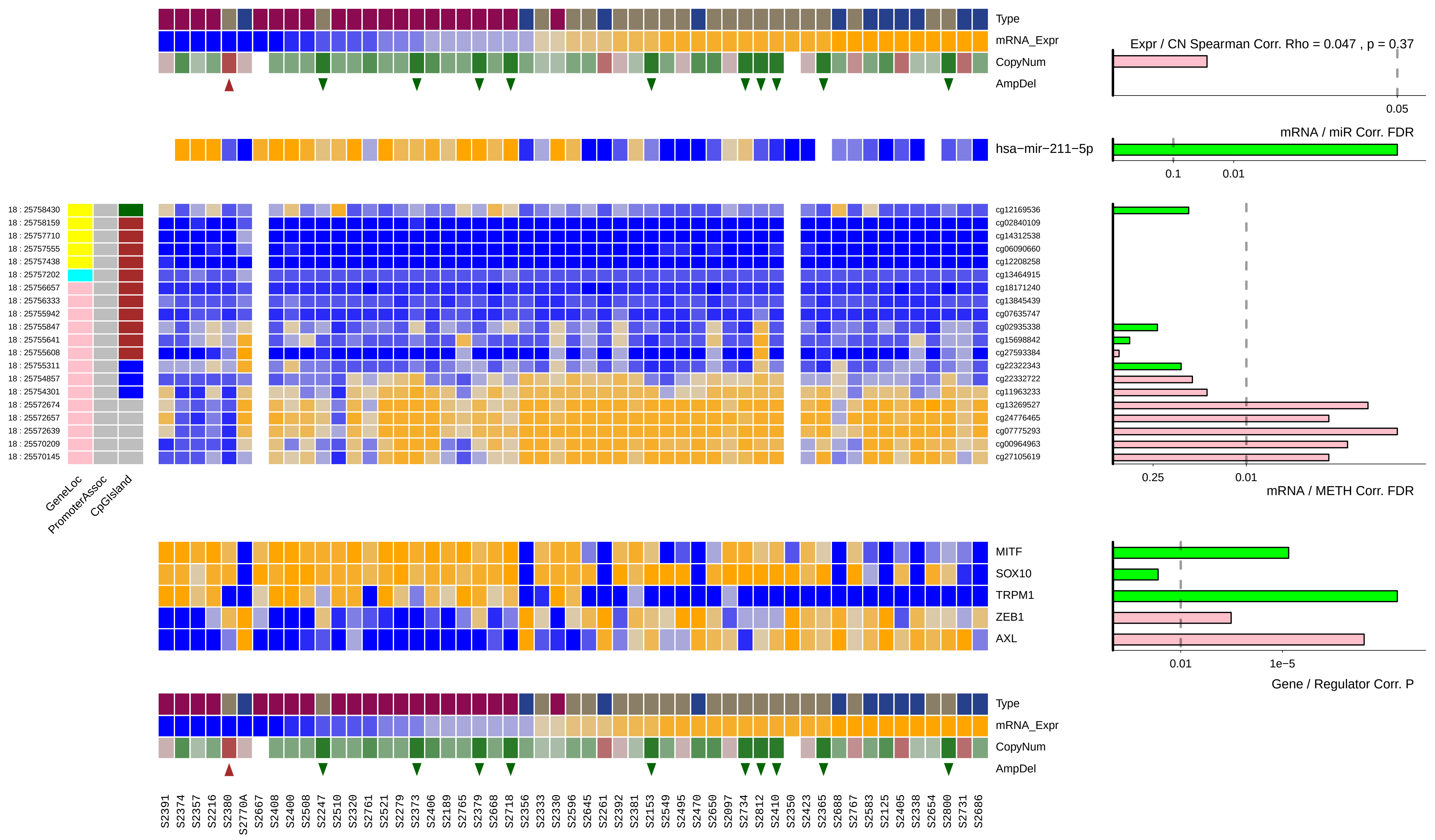

Type
mRNA_Expr
CopyNum
AmpDel
Expr / CN Spearman Corr. Rho = 0.047 , p = 0.37
0.05
hsa−mir−211−5p
mRNA / miR Corr. FDR
0.1
0.01
18 : 25758430
18 : 25758159
18 : 25757710
18 : 25757555
18 : 25757438
18 : 25757202
18 : 25756657
18 : 25756333
18 : 25755942
18 : 25755847
18 : 25755641
18 : 25755608
18 : 25755311
18 : 25754857
18 : 25754301
18 : 25572674
18 : 25572657
18 : 25572639
18 : 25570209
18 : 25570145
cg12169536
cg02840109
cg14312538
cg06090660
cg12208258
cg13464915
cg18171240
cg13845439
cg07635747
cg02935338
cg15698842
cg27593384
cg22322343
cg22332722
cg11963233
cg13269527
cg24776465
cg07775293
cg00964963
cg27105619
GeneLoc
PromoterAssoc
CpGIsland
0.25
0.01
mRNA / METH Corr. FDR
MITF
SOX10
TRPM1
ZEB1
AXL
0.01
1e−5
Gene / Regulator Corr. P
S2391 S2374 S2357 S2216 S2380 S2770A S2667 S2408 S2400 S2508 S2247 S2510 S2320 S2761 S2521 S2279 S2373 S2406 S2189 S2765 S2379 S2668 S2718 S2356 S2333 S2330 S2596 S2645 S2261 S2392 S2381 S2153 S2549 S2495 S2470 S2650 S2097 S2734 S2812 S2410 S2350 S2423 S2365 S2688 S2767 S2583 S2125 S2405 S2338 S2654 S2800 S2731 S2686

RGS4

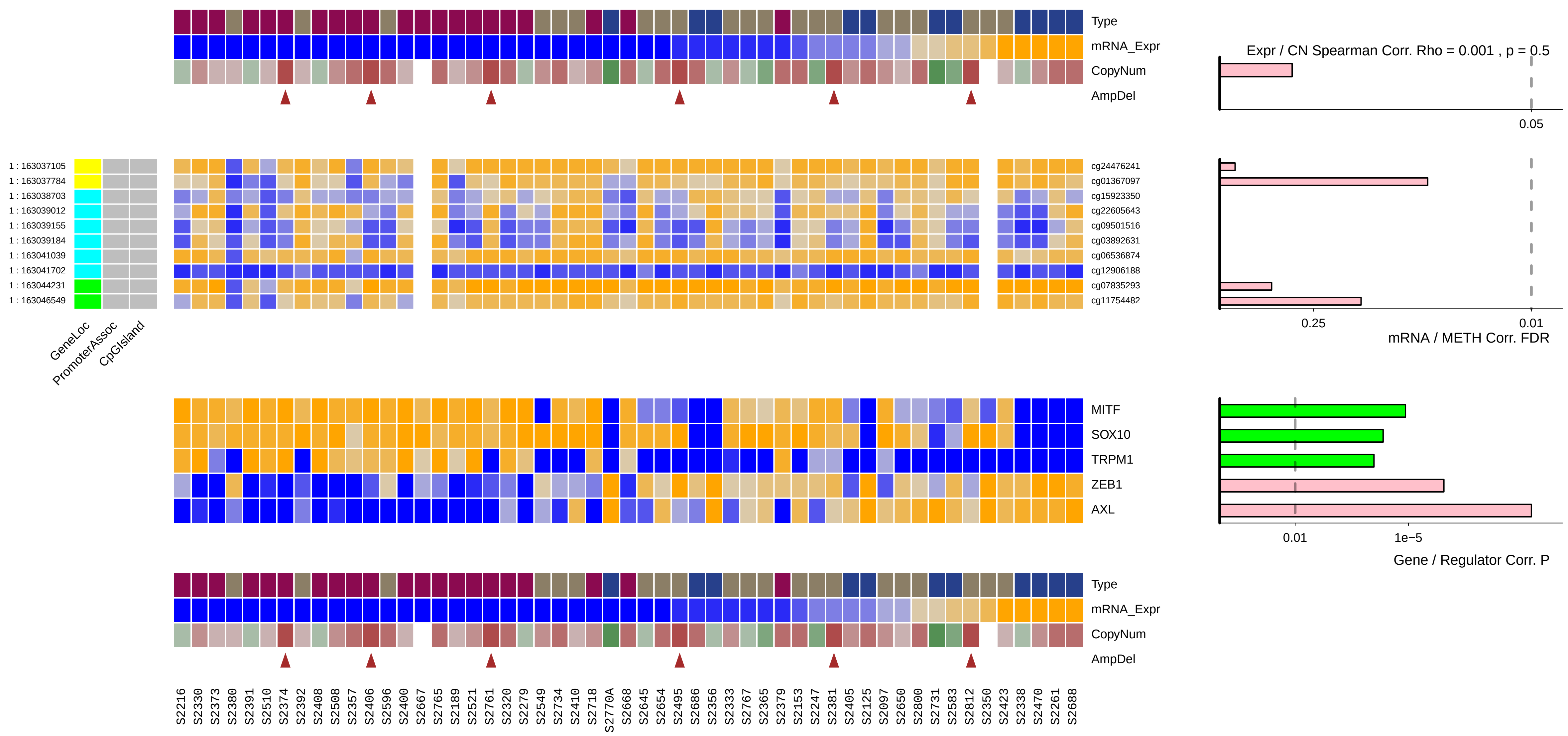

Type
mRNA_Expr
CopyNum
AmpDel
Expr / CN Spearman Corr. Rho = 0.001 , p = 0.5
0.05
1 : 163037105
1 : 163037784
1 : 163038703
1 : 163039012
1 : 163039155
1 : 163039184
1 : 163041039
1 : 163041702
1 : 163044231
1 : 163046549
GeneLoc
PromoterAssoc
CpGIsland
cg24476241
cg01367097
cg15923350
cg22605643
cg09501516
cg03892631
cg06536874
cg12906188
cg07835293
cg11754482
0.25
0.01
mRNA / METH Corr. FDR
MITF
SOX10
TRPM1
ZEB1
AXL
0.01
1e−5
Gene / Regulator Corr. P
Type
mRNA_Expr
CopyNum
AmpDel
S2216 S2330 S2373 S2380 S2391 S2510 S2374 S2392 S2408 S2508 S2357 S2406 S2596 S2400 S2667 S2765 S2189 S2521 S2761 S2320 S2279 S2549 S2734 S2410 S2718 S2770A S2668 S2645 S2654 S2495 S2686 S2356 S2333 S2767 S2365 S2379 S2153 S2247 S2381 S2405 S2125 S2097 S2650 S2800 S2731 S2583 S2812 S2350 S2423 S2338 S2470 S2261 S2688

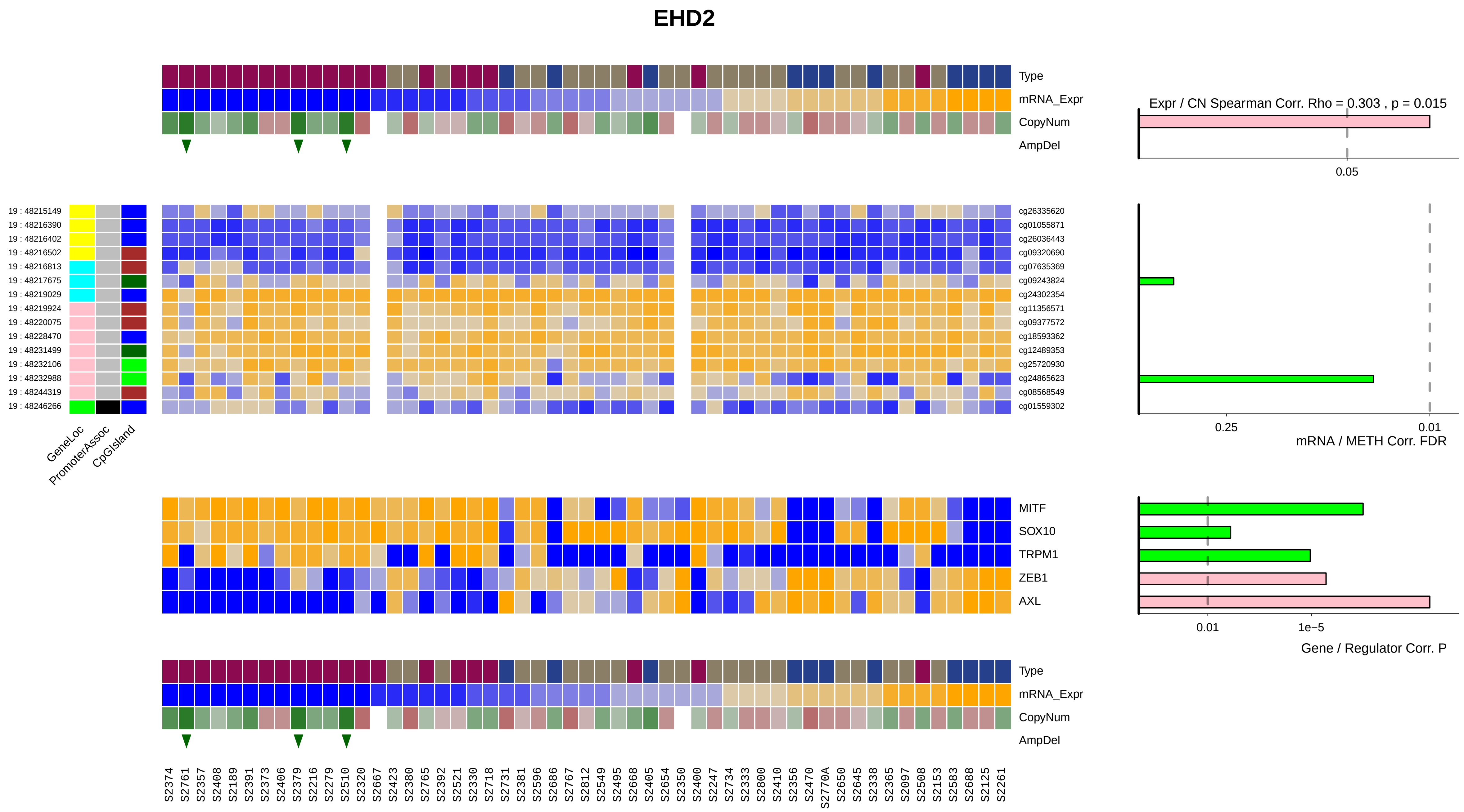

EHD2
Type
mRNA_Expr
CopyNum
AmpDel
Expr / CN Spearman Corr. Rho = 0.303 , p = 0.015
0.05
19 : 48215149
19 : 48216390
19 : 48216402
19 : 48216502
19 : 48216813
19 : 48217675
19 : 48219029
19 : 48219924
19 : 48220075
19 : 48228470
19 : 48231499
19 : 48232106
19 : 48232988
19 : 48244319
19 : 48246266
GeneLoc
PromoterAssoc
CpGIsland
cg26335620
cg01055871
cg26036443
cg09320690
cg07635369
cg09243824
cg24302354
cg11356571
cg09377572
cg18593362
cg12489353
cg25720930
cg24865623
cg08568549
cg01559302
0.25
0.01
mRNA / METH Corr. FDR
MITF
SOX10
TRPM1
ZEB1
AXL
0.01
1e−5
Gene / Regulator Corr. P
Type
mRNA_Expr
CopyNum
AmpDel
S2374 S2761 S2357 S2408 S2189 S2391 S2373 S2406 S2379 S2216 S2279 S2510 S2320 S2667 S2423 S2380 S2765 S2392 S2521 S2330 S2718 S2731 S2381 S2596 S2686 S2767 S2812 S2549 S2495 S2668 S2405 S2654 S2350 S2400 S2247 S2734 S2333 S2800 S2410 S2356 S2470 S2770A S2650 S2645 S2338 S2365 S2097 S2508 S2153 S2583 S2688 S2125 S2261

COL12A1

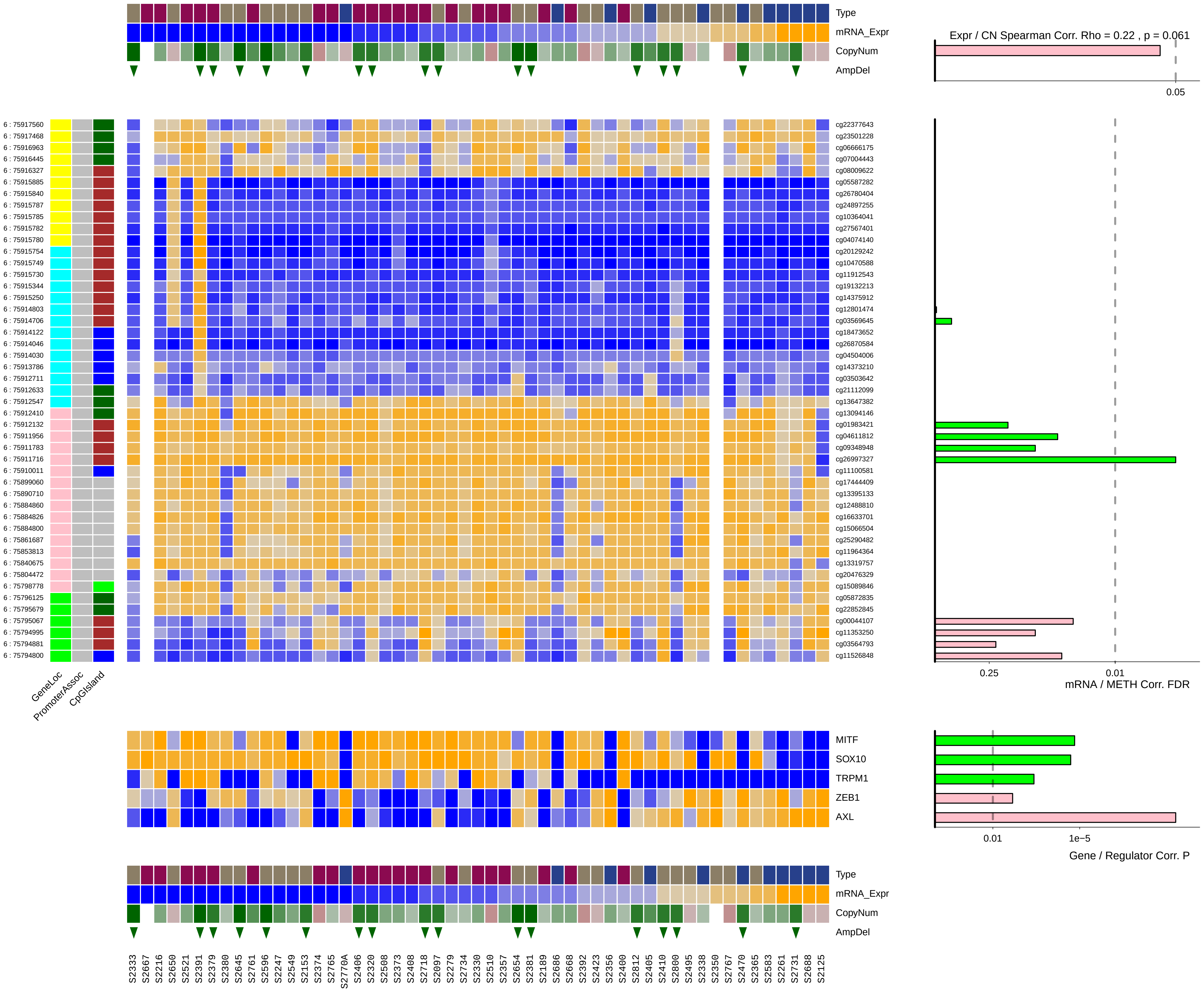

Type
mRNA_Expr
CopyNum
AmpDel
Expr / CN Spearman Corr. Rho = 0.22 , p = 0.061
0.05
0.25
0.01
mRNA / METH Corr. FDR
GeneLoc
PromoterAssoc
CpGIsland
MITF
SOX10
TRPM1
ZEB1
AXL
0.01
1e−5
Gene / Regulator Corr. P

MICAL2

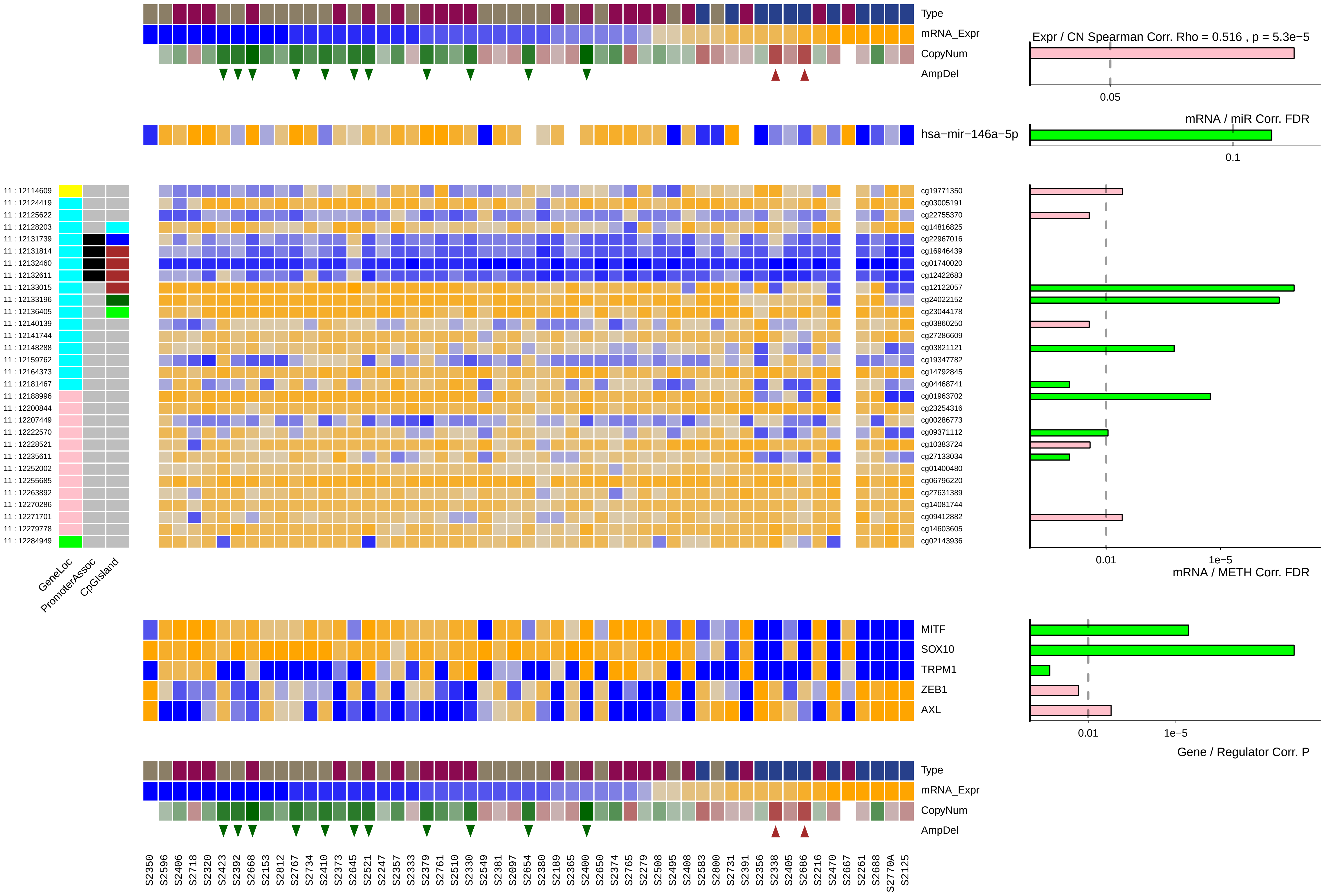

Type
mRNA_Expr
CopyNum
AmpDel
Expr / CN Spearman Corr. Rho = 0.516 , p = 5.3e−5
0.05
mRNA / miR Corr. FDR
0.1
hsa−mir−146a−5p
11 : 12114609
11 : 12124419
11 : 12125622
11 : 12128203
11 : 12131739
11 : 12131814
11 : 12132460
11 : 12132611
11 : 12133015
11 : 12133196
11 : 12136405
11 : 12140139
11 : 12141744
11 : 12148288
11 : 12159762
11 : 12164373
11 : 12181467
11 : 12188996
11 : 12200844
11 : 12207449
11 : 12222570
11 : 12228521
11 : 12235611
11 : 12252002
11 : 12255685
11 : 12263892
11 : 12270286
11 : 12271701
11 : 12279778
11 : 12284949
cg19771350
cg03005191
cg22755370
cg14816825
cg22967016
cg16946439
cg01740020
cg12422683
cg12122057
cg24022152
cg23044178
cg03860250
cg27286609
cg03821121
cg19347782
cg14792845
cg04468741
cg01963702
cg23254316
cg00286773
cg09371112
cg10383724
cg27133034
cg01400480
cg06796220
cg27631389
cg14081744
cg09412882
cg14603605
cg02143936
0.01
1e−5
mRNA / METH Corr. FDR
GeneLoc
PromoterAssoc
CpGIsland
MITF
SOX10
TRPM1
ZEB1
AXL
0.01
1e−5
Gene / Regulator Corr. P
Type
mRNA_Expr
CopyNum
AmpDel
S2350 S2596 S2406 S2718 S2320 S2423 S2392 S2668 S2153 S2812 S2767 S2734 S2410 S2373 S2645 S2521 S2247 S2357 S2333 S2379 S2761 S2510 S2330 S2549 S2381 S2097 S2654 S2380 S2189 S2365 S2400 S2650 S2374 S2765 S2279 S2508 S2495 S2408 S2583 S2800 S2731 S2391 S2356 S2338 S2405 S2686 S2216 S2470 S2667 S2261 S2688 S2770A S2125

ERRFI1

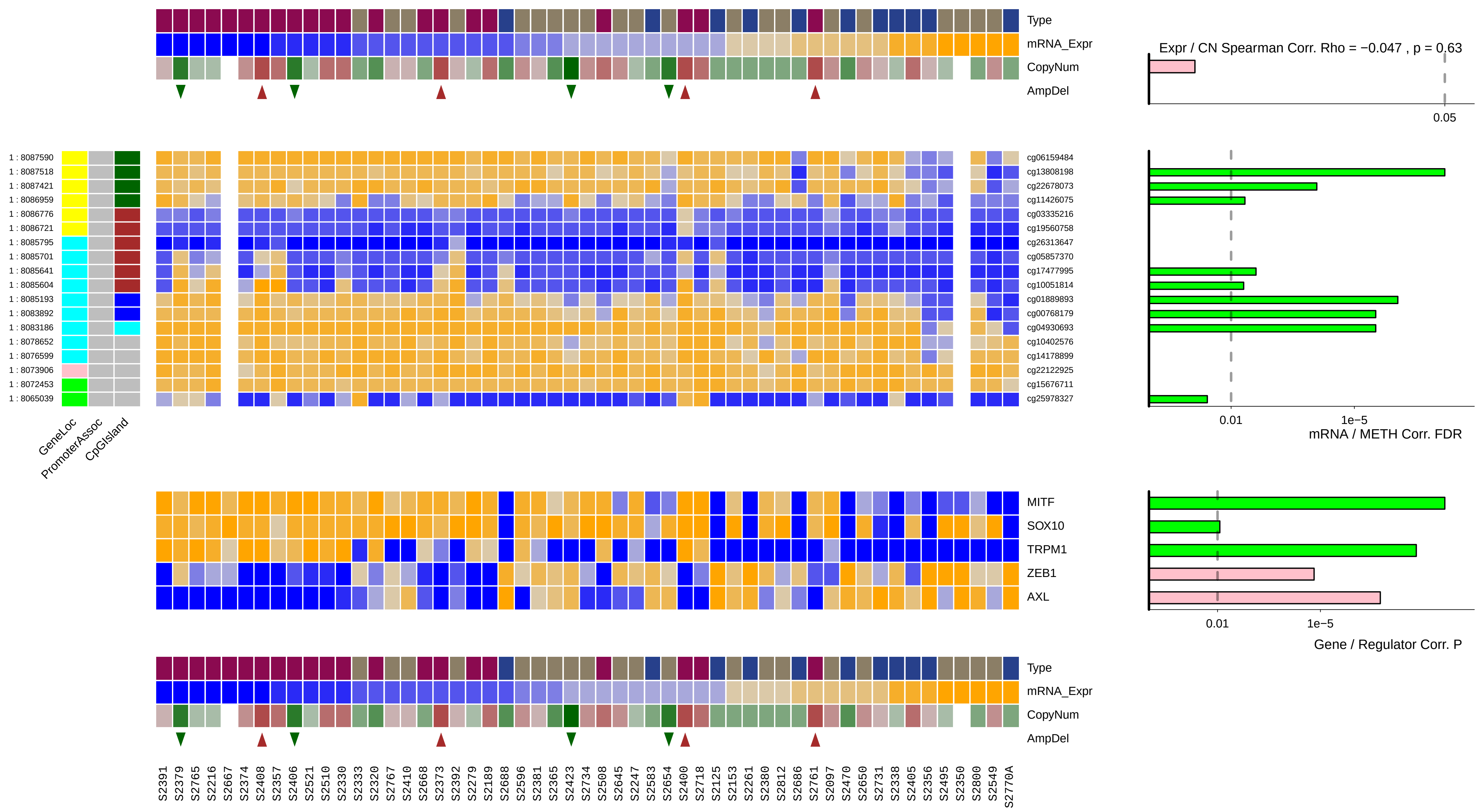
Type
mRNA_Expr
CopyNum
AmpDel
Expr / CN Spearman Corr. Rho = −0.047 , p = 0.63
0.05
1 : 8087590
1 : 8087518
1 : 8087421
1 : 8086959
1 : 8086776
1 : 8086721
1 : 8085795
1 : 8085701
1 : 8085641
1 : 8085604
1 : 8085193
1 : 8083892
1 : 8083186
1 : 8078652
1 : 8076599
1 : 8073906
1 : 8072453
1 : 8065039
GeneLoc
PromoterAssoc
CpGIsland
cg06159484
cg13808198
cg22678073
cg11426075
cg03335216
cg19560758
cg26313647
cg05857370
cg17477995
cg10051814
cg01889893
cg00768179
cg04930693
cg10402576
cg14178899
cg22122925
cg15676711
cg25978327
0.01
1e−5
mRNA / METH Corr. FDR
MITF
SOX10
TRPM1
ZEB1
AXL
0.01
1e−5
Gene / Regulator Corr. P
Type
mRNA_Expr
CopyNum
AmpDel
S2391
S2379
S2765
S2216
S2667
S2374
S2408
S2357
S2406
S2521
S2510
S2330
S2333
S2320
S2767
S2410
S2668
S2373
S2392
S2279
S2189
S2688
S2596
S2381
S2365
S2423
S2734
S2508
S2645
S2247
S2583
S2654
S2400
S2718
S2125
S2153
S2261
S2380
S2812
S2686
S2761
S2097
S2470
S2650
S2731
S2338
S2405
S2356
S2495
S2350
S2800
S2549
S2770A

SLC14A1

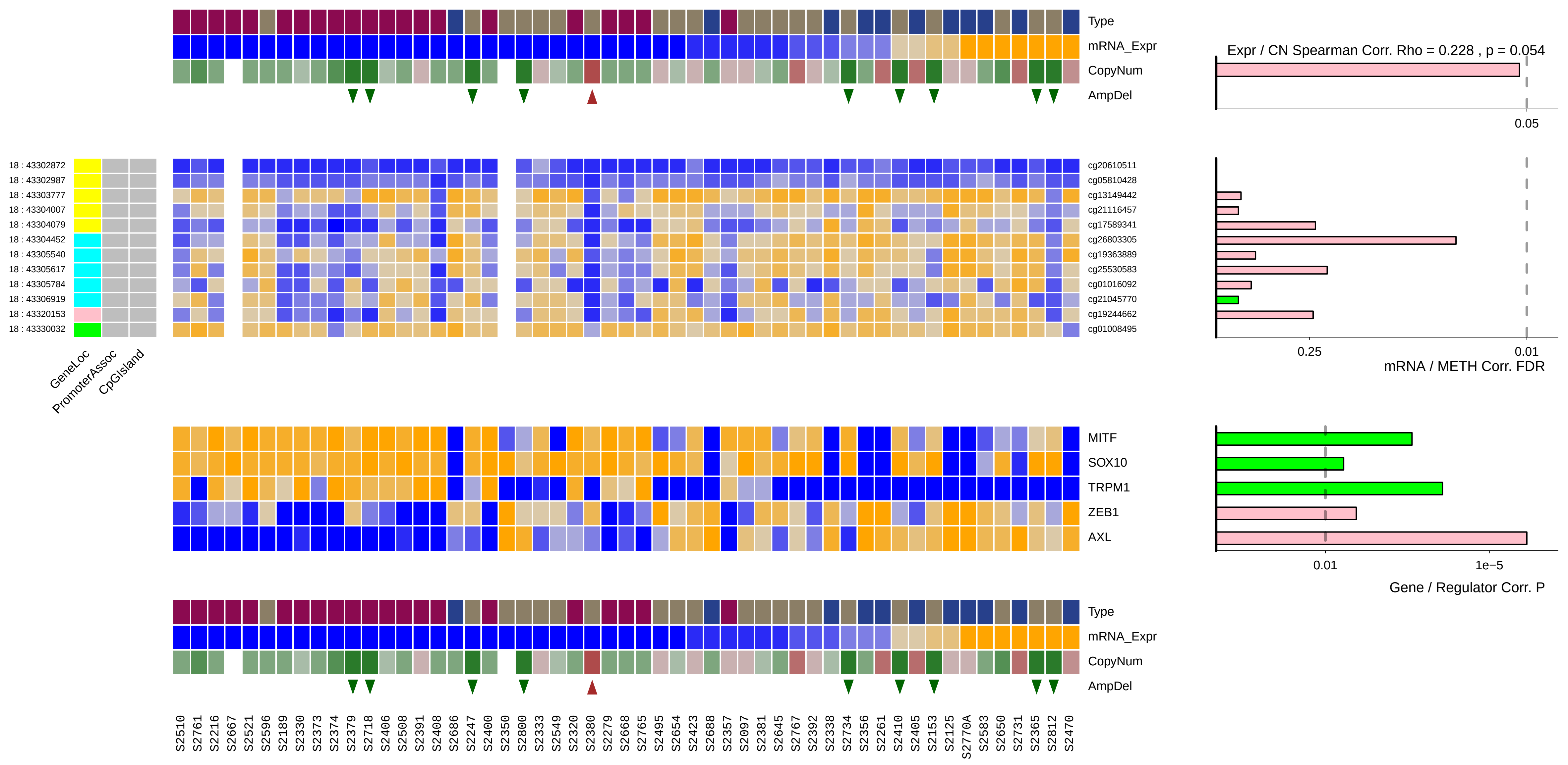

Type
mRNA_Expr
CopyNum
AmpDel
Expr / CN Spearman Corr. Rho = 0.228 , p = 0.054
0.05
18 : 43302872
18 : 43302987
18 : 43303777
18 : 43304007
18 : 43304079
18 : 43304452
18 : 43305540
18 : 43305617
18 : 43305784
18 : 43306919
18 : 43320153
18 : 43330032
GeneLoc
PromoterAssoc
CpGIsland
cg20610511
cg05810428
cg13149442
cg21116457
cg17589341
cg26803305
cg19363889
cg25530583
cg01016092
cg21045770
cg19244662
cg01008495
0.25
0.01
mRNA / METH Corr. FDR
MITF
SOX10
TRPM1
ZEB1
AXL
0.01
1e−5
Gene / Regulator Corr. P
Type
mRNA_Expr
CopyNum
AmpDel
S2510 S2761 S2216 S2667 S2521 S2596 S2189 S2330 S2373 S2374 S2379 S2718 S2406 S2508 S2391 S2408 S2686 S2247 S2400 S2350 S2800 S2333 S2549 S2320 S2380 S2279 S2668 S2765 S2495 S2654 S2423 S2688 S2357 S2097 S2381 S2645 S2767 S2392 S2338 S2734 S2356 S2261 S2410 S2405 S2153 S2125 S2770A S2583 S2650 S2731 S2365 S2812 S2470

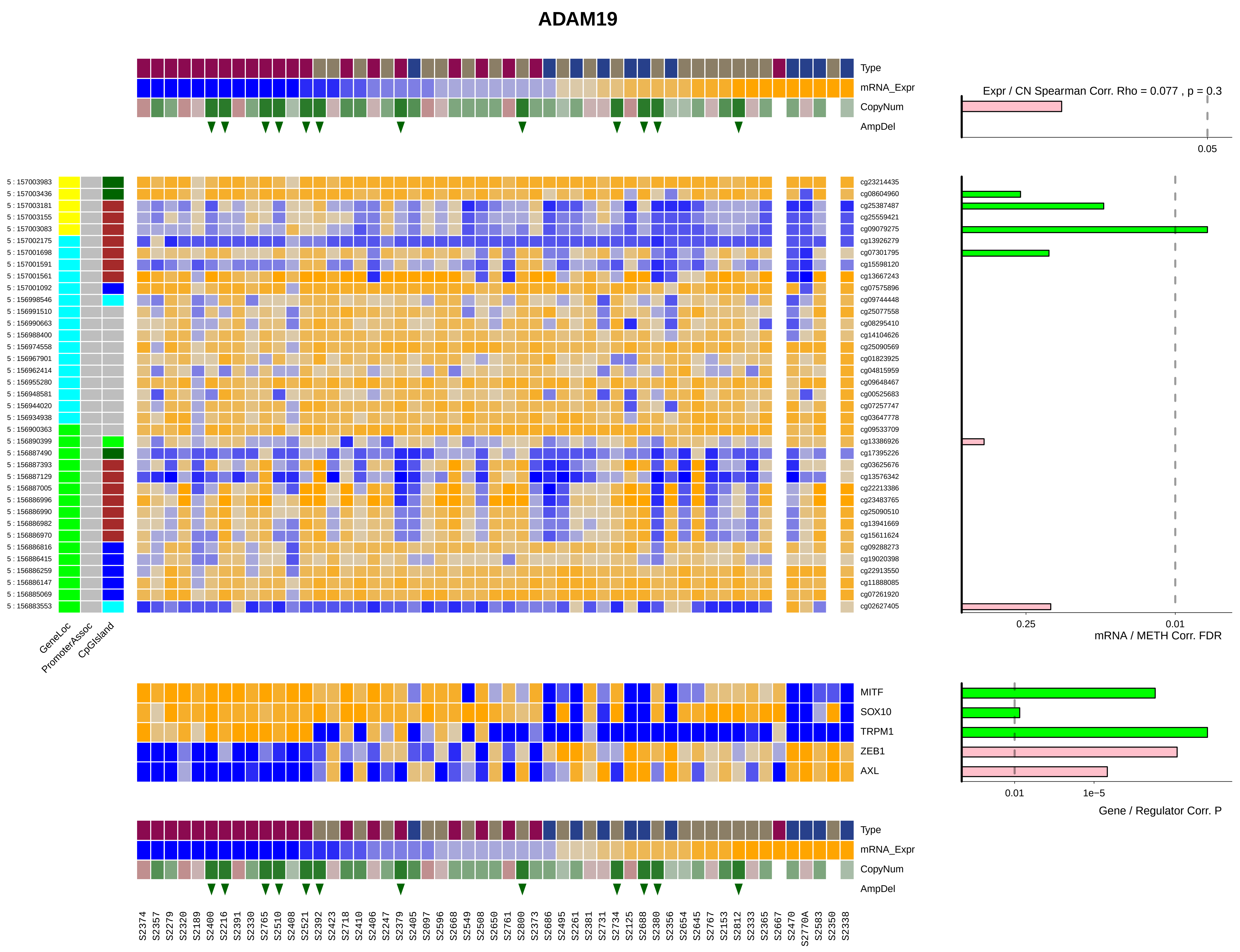
ADAM19
Type
mRNA_Expr
CopyNum
AmpDel
Expr / CN Spearman Corr. Rho = 0.077 , p = 0.3
0.05
5 : 157003983
5 : 157003436
5 : 157003181
5 : 157003155
5 : 157003083
5 : 157002175
5 : 157001698
5 : 157001591
5 : 157001561
5 : 157001092
5 : 156998546
5 : 156991510
5 : 156990663
5 : 156988400
5 : 156974558
5 : 156967901
5 : 156962414
5 : 156955280
5 : 156948581
5 : 156944020
5 : 156934938
5 : 156900363
5 : 156890399
5 : 156887490
5 : 156887393
5 : 156887129
5 : 156887005
5 : 156886996
5 : 156886990
5 : 156886982
5 : 156886970
5 : 156886816
5 : 156886415
5 : 156886259
5 : 156886147
5 : 156885069
5 : 156883553
cg23214435
cg08604960
cg25387487
cg25559421
cg09079275
cg13926279
cg07301795
cg15598120
cg13667243
cg07575896
cg09744448
cg25077558
cg08295410
cg14104626
cg25090569
cg01823925
cg04815959
cg09648467
cg00525683
cg07257747
cg03647778
cg09533709
cg13386926
cg17395226
cg03625676
cg13576342
cg22213386
cg23483765
cg25090510
cg13941669
cg15611624
cg09288273
cg19020398
cg22913550
cg11888085
cg07261920
cg02627405
GeneLoc
PromoterAssoc
CpGIsland
0.25
0.01
mRNA / METH Corr. FDR
MITF
SOX10
TRPM1
ZEB1
AXL
0.01
1e−5
Gene / Regulator Corr. P
Type
mRNA_Expr
CopyNum
AmpDel
S2374 S2357 S2279 S2320 S2189 S2400 S2216 S2391 S2330 S2765 S2510 S2408 S2521 S2392 S2423 S2718 S2410 S2406 S2247 S2379 S2405 S2097 S2596 S2668 S2549 S2508 S2650 S2761 S2800 S2373 S2686 S2495 S2261 S2381 S2731 S2734 S2125 S2688 S2380 S2356 S2654 S2645 S2767 S2153 S2812 S2333 S2365 S2667 S2470 S2770A S2583 S2350 S2338

KRT8

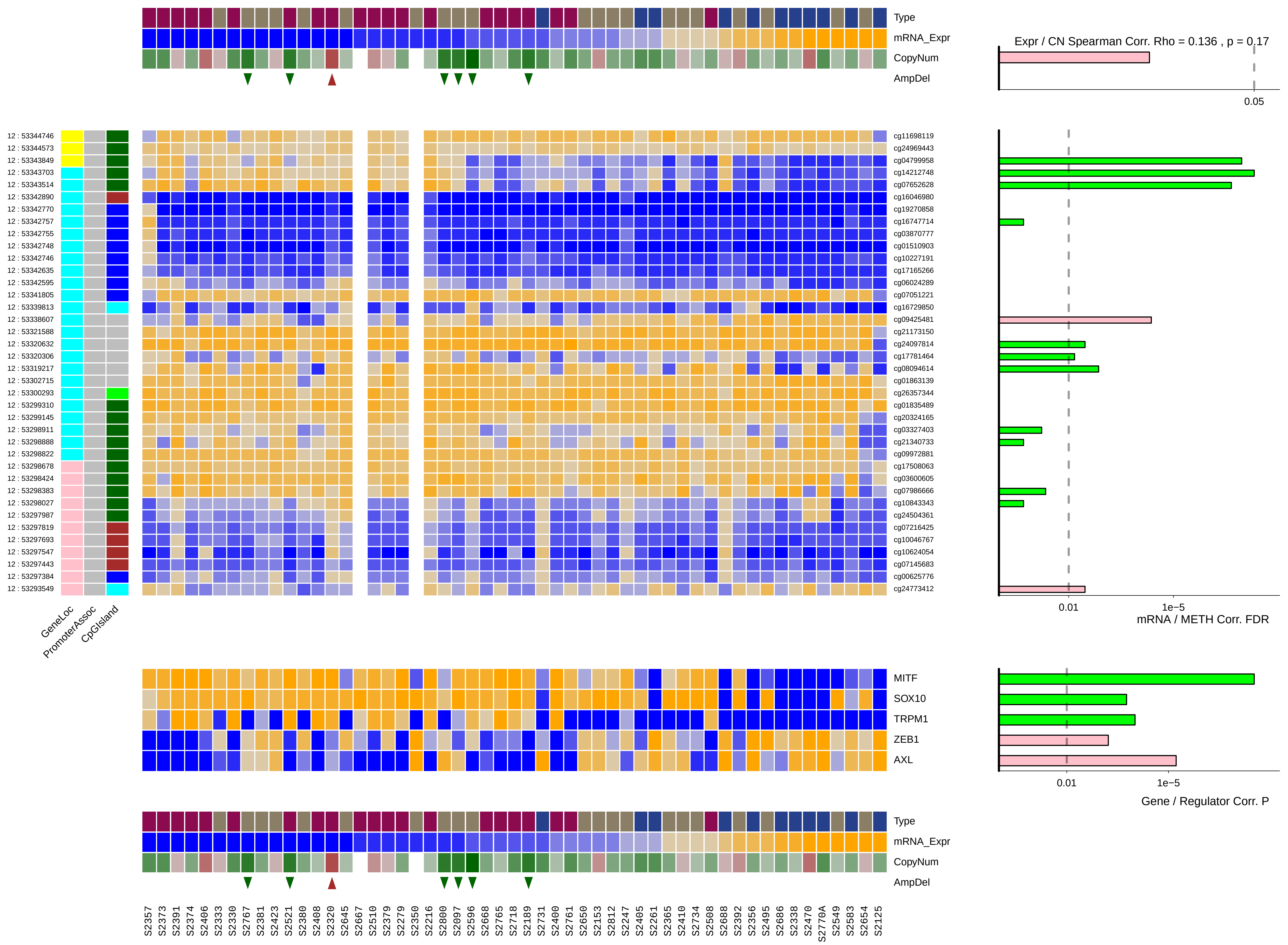

Type
mRNA_Expr
CopyNum
AmpDel
Expr / CN Spearman Corr. Rho = 0.136 , p = 0.17
0.05
12 : 53344746
12 : 53344573
12 : 53343849
12 : 53343703
12 : 53343514
12 : 53342890
12 : 53342770
12 : 53342757
12 : 53342755
12 : 53342748
12 : 53342746
12 : 53342635
12 : 53342595
12 : 53341805
12 : 53339813
12 : 53338607
12 : 53321588
12 : 53320632
12 : 53320306
12 : 53319217
12 : 53302715
12 : 53300293
12 : 53299310
12 : 53299145
12 : 53298911
12 : 53298888
12 : 53298822
12 : 53298678
12 : 53298424
12 : 53298383
12 : 53298027
12 : 53297987
12 : 53297819
12 : 53297693
12 : 53297547
12 : 53297443
12 : 53297384
12 : 53293549
cg11698119
cg24969443
cg04799958
cg14212748
cg07652628
cg16046980
cg19270858
cg16747714
cg03870777
cg01510903
cg10227191
cg17165266
cg06024289
cg07051221
cg16729850
cg09425481
cg21173150
cg24097814
cg17781464
cg08094614
cg01863139
cg26357344
cg01835489
cg20324165
cg03327403
cg21340733
cg09972881
cg17508063
cg03600605
cg07986666
cg10843343
cg24504361
cg07216425
cg10046767
cg10624054
cg07145683
cg00625776
cg24773412
0.01
1e−5
mRNA / METH Corr. FDR
GeneLoc
PromoterAssoc
CpGIsland
MITF
SOX10
TRPM1
ZEB1
AXL
0.01
1e−5
Gene / Regulator Corr. P
Type
mRNA_Expr
CopyNum
AmpDel
S2357
S2373
S2391
S2374
S2406
S2333
S2330
S2767
S2381
S2423
S2521
S2380
S2408
S2320
S2645
S2667
S2510
S2379
S2279
S2350
S2216
S2800
S2097
S2596
S2668
S2765
S2718
S2189
S2731
S2400
S2761
S2650
S2153
S2812
S2247
S2405
S2261
S2365
S2410
S2734
S2508
S2688
S2392
S2356
S2495
S2686
S2338
S2470
S2770A
S2549
S2583
S2654
S2125

CLMP

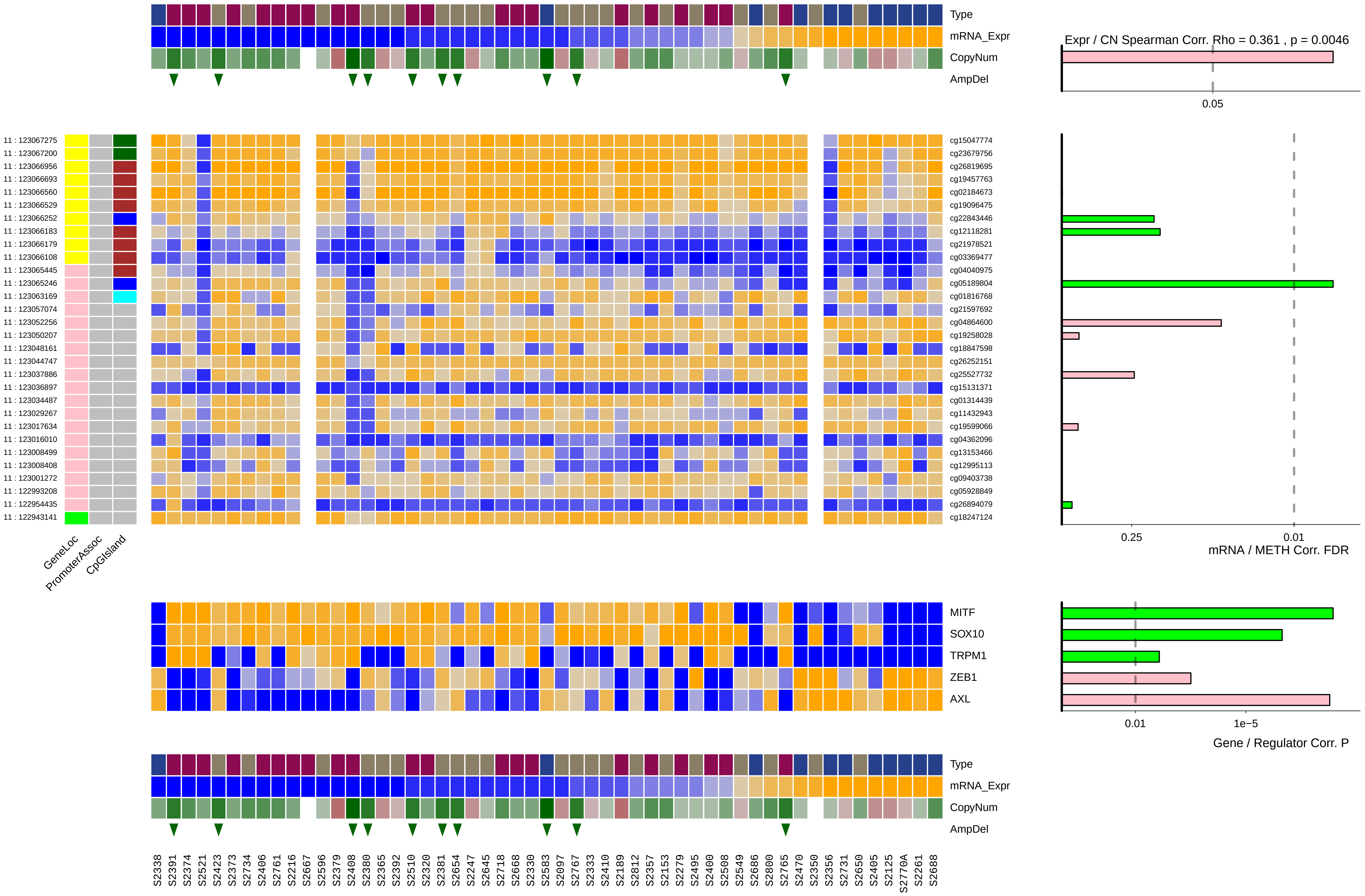
Type
mRNA_Expr
CopyNum
AmpDel
Expr / CN Spearman Corr. Rho = 0.361 , p = 0.0046
0.05
11 : 123067275
11 : 123067200
11 : 123066956
11 : 123066693
11 : 123066560
11 : 123066529
11 : 123066252
11 : 123066183
11 : 123066179
11 : 123066108
11 : 123065445
11 : 123065246
11 : 123063169
11 : 123057074
11 : 123052256
11 : 123050207
11 : 123048161
11 : 123044747
11 : 123037886
11 : 123036897
11 : 123034487
11 : 123029267
11 : 123017634
11 : 123016010
11 : 123008499
11 : 123008408
11 : 123001272
11 : 122993208
11 : 122954435
11 : 122943141
GeneLoc
PromoterAssoc
CpGIsland
cg15047774
cg23679756
cg26819695
cg19457763
cg02184673
cg19096475
cg22843446
cg12118281
cg21978521
cg03369477
cg04040975
cg05189804
cg01816768
cg21597692
cg04864600
cg19258028
cg18847598
cg26252151
cg25527732
cg15131371
cg01314439
cg11432943
cg19599066
cg04362096
cg13153466
cg12995113
cg09403738
cg05928849
cg26894079
cg18247124
0.25
0.01
mRNA / METH Corr. FDR
MITF
SOX10
TRPM1
ZEB1
AXL
0.01
1e−5
Gene / Regulator Corr. P
Type
mRNA_Expr
CopyNum
AmpDel
S2338 S2391 S2374 S2521 S2423 S2373 S2734 S2406 S2761 S2216 S2667 S2596 S2379 S2408 S2380 S2365 S2392 S2510 S2320 S2381 S2654 S2247 S2645 S2718 S2668 S2330 S2583 S2097 S2767 S2333 S2410 S2189 S2812 S2357 S2153 S2279 S2495 S2400 S2508 S2549 S2686 S2800 S2765 S2470 S2350 S2356 S2731 S2650 S2405 S2125 S2770A S2261 S2688

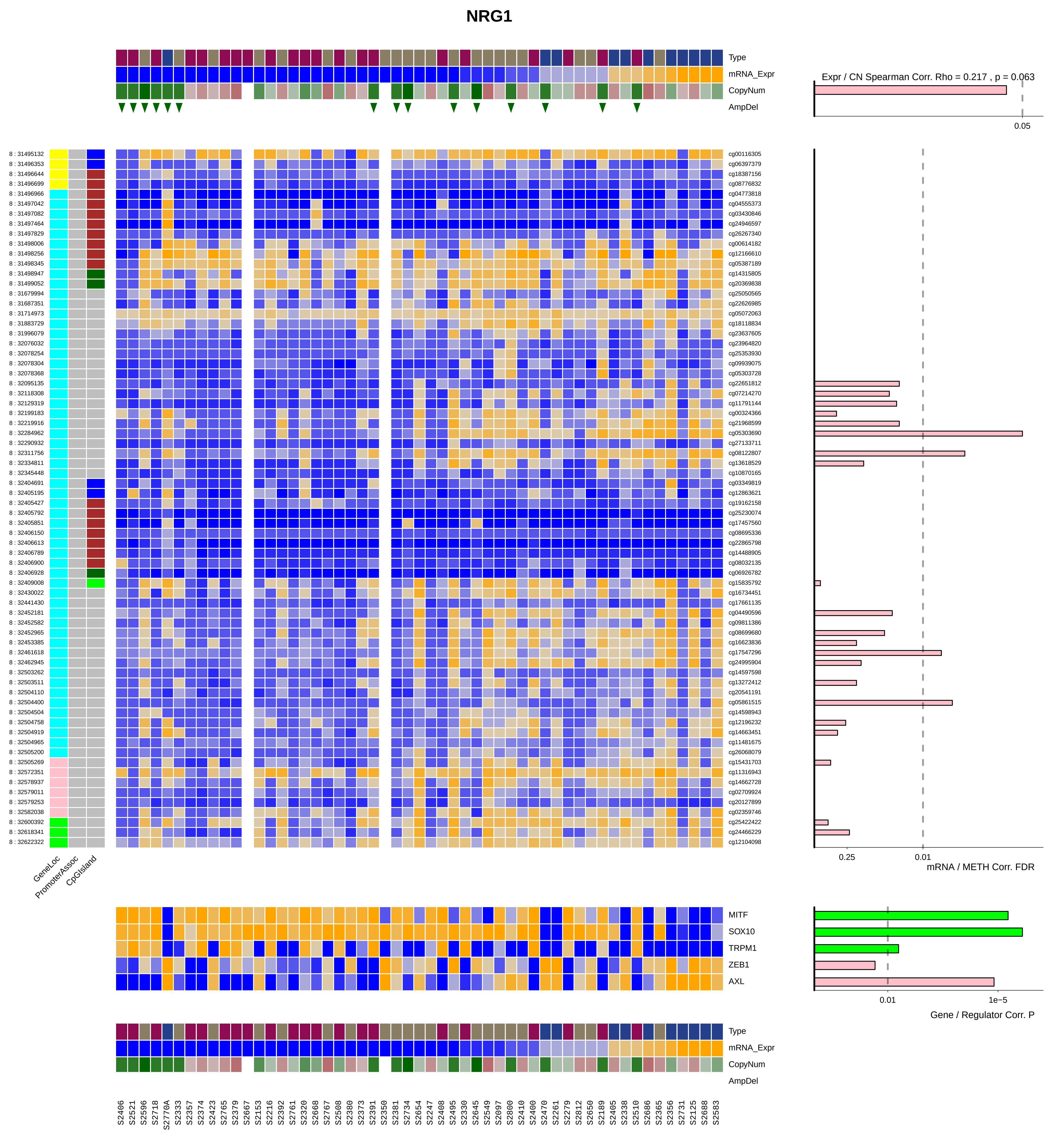
NRG1
Type
mRNA_Expr
CopyNum
AmpDel
Expr / CN Spearman Corr. Rho = 0.217 , p = 0.063
0.05
8 : 31495132
8 : 31496353
8 : 31496644
8 : 31496699
8 : 31496966
8 : 31497042
8 : 31497082
8 : 31497464
8 : 31497829
8 : 31498006
8 : 31498256
8 : 31498345
8 : 31498947
8 : 31499052
8 : 31679994
8 : 31687351
8 : 31714973
8 : 31883729
8 : 31996079
8 : 32076032
8 : 32078254
8 : 32078304
8 : 32078368
8 : 32095135
8 : 32118308
8 : 32129319
8 : 32199183
8 : 32219916
8 : 32284962
8 : 32290932
8 : 32311756
8 : 32334811
8 : 32345448
8 : 32404691
8 : 32405195
8 : 32405427
8 : 32405792
8 : 32405851
8 : 32406150
8 : 32406613
8 : 32406789
8 : 32406900
8 : 32406928
8 : 32409008
8 : 32430022
8 : 32441430
8 : 32452181
8 : 32452582
8 : 32452965
8 : 32453385
8 : 32461618
8 : 32462945
8 : 32503262
8 : 32503511
8 : 32504110
8 : 32504400
8 : 32504504
8 : 32504758
8 : 32504919
8 : 32504965
8 : 32505200
8 : 32505269
8 : 32572351
8 : 32578937
8 : 32579011
8 : 32579253
8 : 32582038
8 : 32600392
8 : 32618341
8 : 32622322
cg00116305
cg06397379
cg18387156
cg08776832
cg04773818
cg04555373
cg03430846
cg24946597
cg26267340
cg00614182
cg12166610
cg05387189
cg14315805
cg20369838
cg25050565
cg22626985
cg05072063
cg18118834
cg23637605
cg23964820
cg25353930
cg09939075
cg05303728
cg22651812
cg07214270
cg11791144
cg00324366
cg21968599
cg05303690
cg27133711
cg08122807
cg13618529
cg10870165
cg03349819
cg12863621
cg19162158
cg25230074
cg17457560
cg08695336
cg22865798
cg14488905
cg08032135
cg06926782
cg15835792
cg16734451
cg17661135
cg04490596
cg09811386
cg08699680
cg16623836
cg17547296
cg24995904
cg14597598
cg13272412
cg20541191
cg05861515
cg14598943
cg12196232
cg14663451
cg11481675
cg26068079
cg15431703
cg11316943
cg14662728
cg02709924
cg20127899
cg02359746
cg25422422
cg24466229
cg12104098
0.25
0.01
mRNA / METH Corr. FDR
GeneLoc
PromoterAssoc
CpGIsland
MITF
SOX10
TRPM1
ZEB1
AXL
0.01
1e−5
Gene / Regulator Corr. P
Type
mRNA_Expr
CopyNum
AmpDel
S2406
S2521
S2596
S2718
S2770A
S2333
S2357
S2374
S2423
S2765
S2379
S2667
S2153
S2216
S2392
S2761
S2320
S2668
S2767
S2508
S2380
S2373
S2391
S2350
S2381
S2734
S2654
S2247
S2408
S2495
S2330
S2645
S2549
S2097
S2800
S2410
S2400
S2470
S2261
S2279
S2812
S2650
S2189
S2405
S2338
S2510
S2686
S2365
S2356
S2731
S2125
S2688
S2583

ITGA11

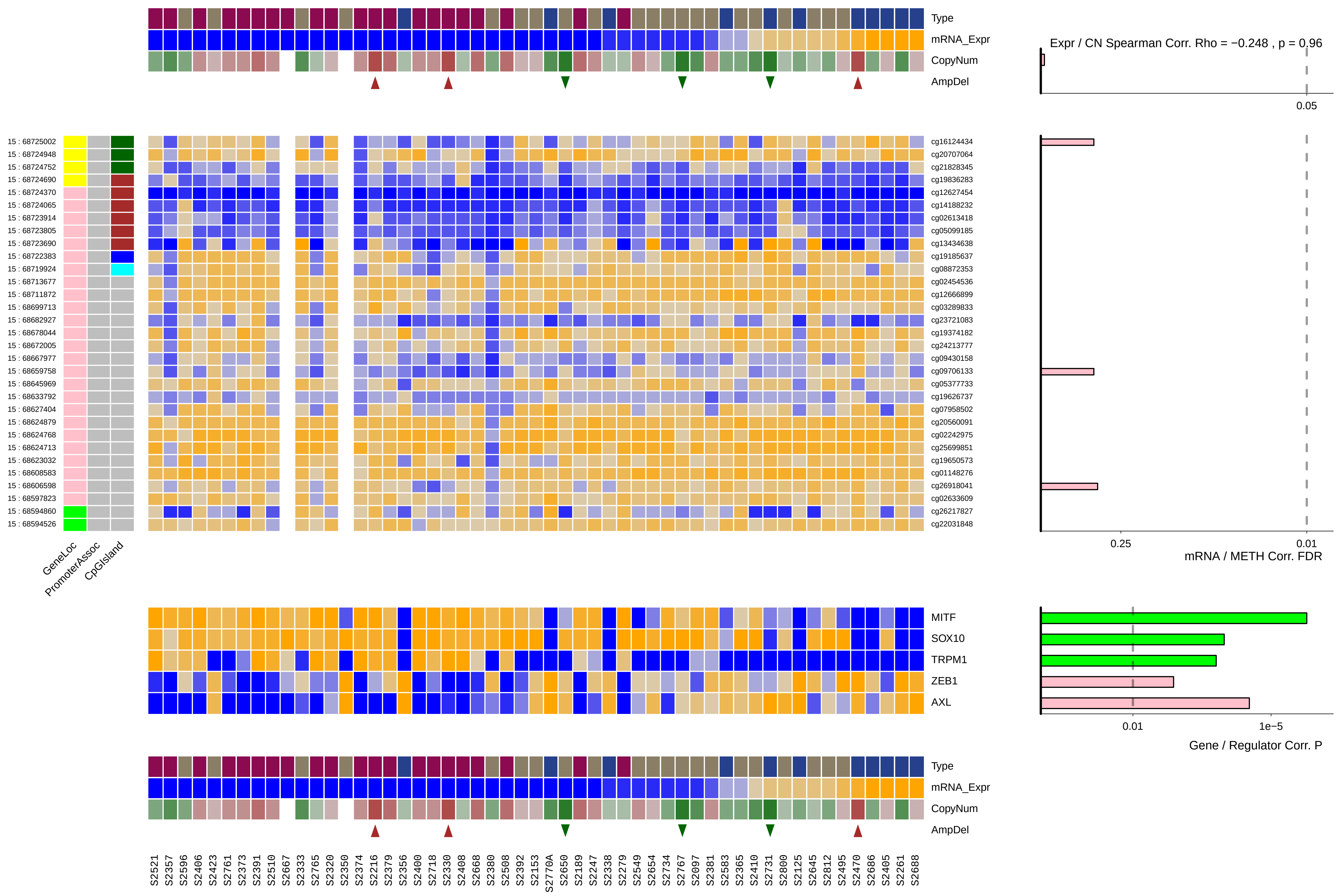

Type
mRNA_Expr
CopyNum
AmpDel
Expr / CN Spearman Corr. Rho = −0.248 , p = 0.96
0.05
15 : 68725002
15 : 68724948
15 : 68724752
15 : 68724690
15 : 68724370
15 : 68724065
15 : 68723914
15 : 68723805
15 : 68723690
15 : 68722383
15 : 68719924
15 : 68713677
15 : 68711872
15 : 68699713
15 : 68682927
15 : 68678044
15 : 68672005
15 : 68667977
15 : 68659758
15 : 68645969
15 : 68633792
15 : 68627404
15 : 68624879
15 : 68624768
15 : 68624713
15 : 68623032
15 : 68608583
15 : 68606598
15 : 68597823
15 : 68594860
15 : 68594526
cg16124434
cg20707064
cg21828345
cg19836283
cg12627454
cg14188232
cg02613418
cg05099185
cg13434638
cg19185637
cg08872353
cg02454536
cg12666899
cg03289833
cg23721083
cg19374182
cg24213777
cg09430158
cg09706133
cg05377733
cg19626737
cg07958502
cg20560091
cg02242975
cg25699851
cg19650573
cg01148276
cg26918041
cg02633609
cg26217827
cg22031848
0.25
0.01
mRNA / METH Corr. FDR
GeneLoc
PromoterAssoc
CpGIsland
MITF
SOX10
TRPM1
ZEB1
AXL
0.01
1e−5
Gene / Regulator Corr. P
Type
mRNA_Expr
CopyNum
AmpDel
S2521
S2357
S2596
S2406
S2423
S2761
S2373
S2391
S2510
S2667
S2333
S2765
S2320
S2350
S2374
S2216
S2379
S2356
S2400
S2718
S2330
S2408
S2668
S2380
S2508
S2392
S2153
S2770A
S2650
S2189
S2247
S2338
S2279
S2549
S2654
S2734
S2767
S2097
S2381
S2583
S2365
S2410
S2731
S2800
S2125
S2645
S2812
S2495
S2470
S2686
S2405
S2261
S2688

RARRES3

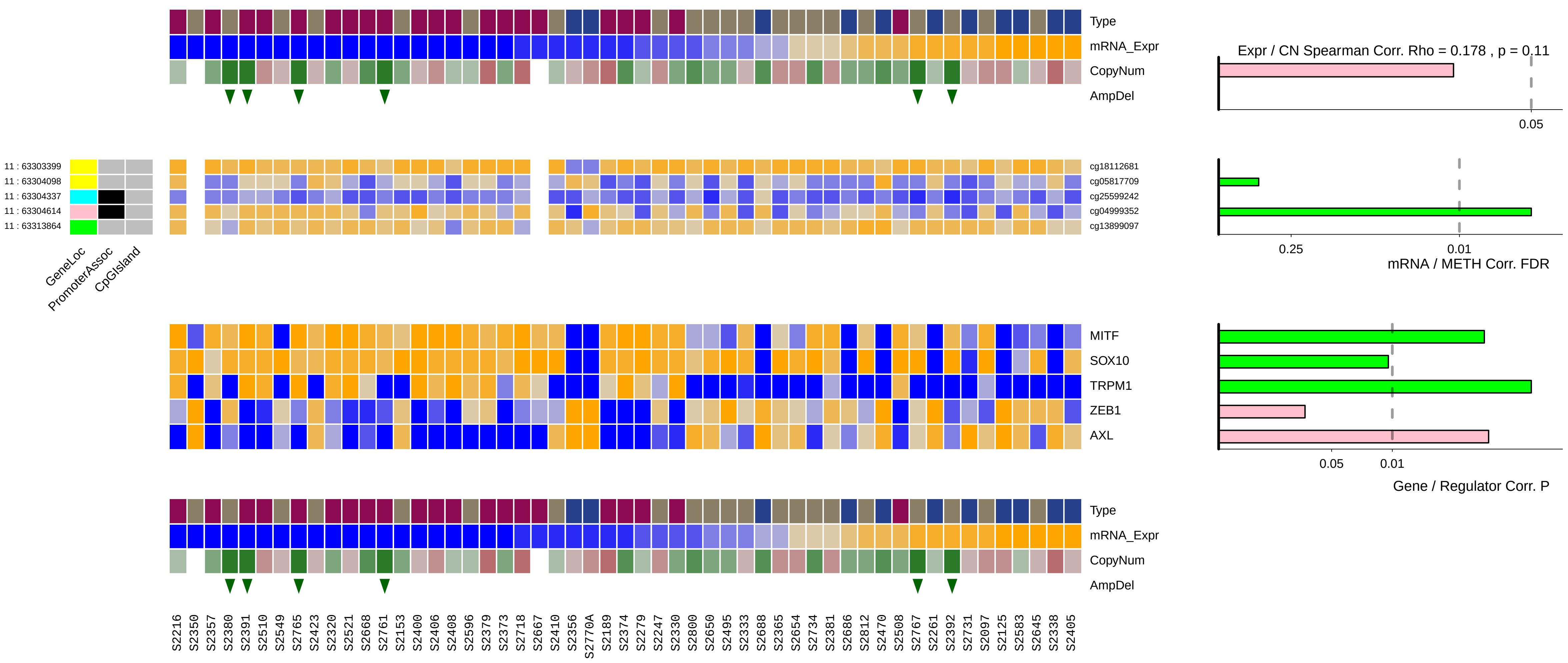
Type
mRNA_Expr
CopyNum
AmpDel
Expr / CN Spearman Corr. Rho = 0.178 , p = 0.11
0.05
11 : 63303399
11 : 63304098
11 : 63304337
11 : 63304614
11 : 63313864
GeneLoc
PromoterAssoc
CpGIsland
cg18112681
cg05817709
cg25599242
cg04999352
cg13899097
0.25
0.01
mRNA / METH Corr. FDR
MITF
SOX10
TRPM1
ZEB1
AXL
0.05
0.01
Gene / Regulator Corr. P
Type
mRNA_Expr
CopyNum
AmpDel
S2216 S2350 S2357 S2380 S2391 S2510 S2549 S2765 S2423 S2320 S2521 S2668 S2761 S2153 S2400 S2406 S2408 S2596 S2379 S2373 S2718 S2667 S2410 S2356 S2770A S2189 S2374 S2279 S2247 S2330 S2800 S2650 S2495 S2333 S2688 S2365 S2654 S2734 S2381 S2686 S2812 S2470 S2508 S2767 S2261 S2392 S2731 S2097 S2125 S2583 S2645 S2338 S2405

FHL1

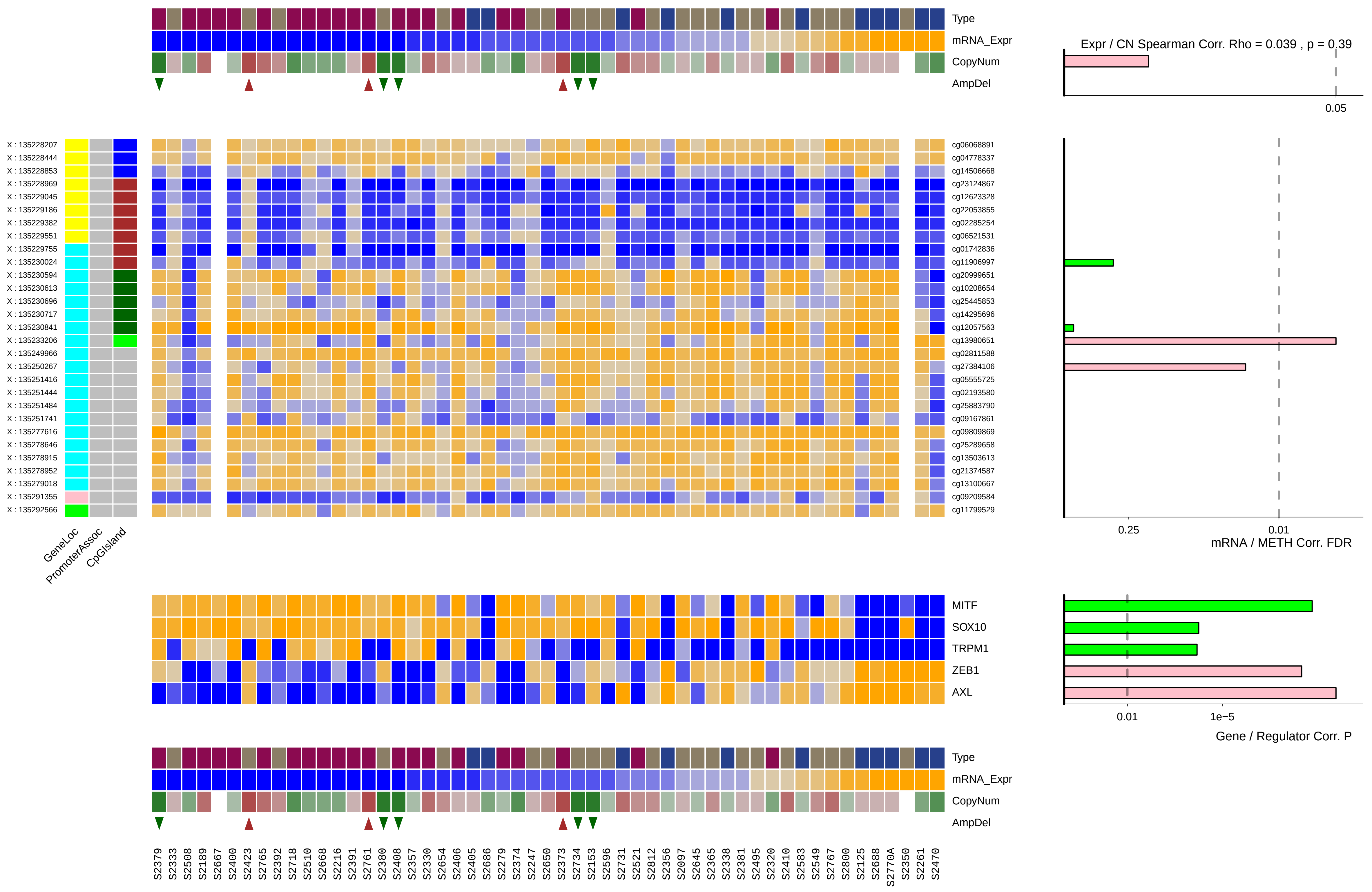
Type
mRNA_Expr
CopyNum
AmpDel
Expr / CN Spearman Corr. Rho = 0.039 , p = 0.39
0.05
mRNA / METH Corr. FDR
0.25
0.01
GeneLoc
PromoterAssoc
CpGIsland
MITF
SOX10
TRPM1
ZEB1
AXL
Gene / Regulator Corr. P
1e−5

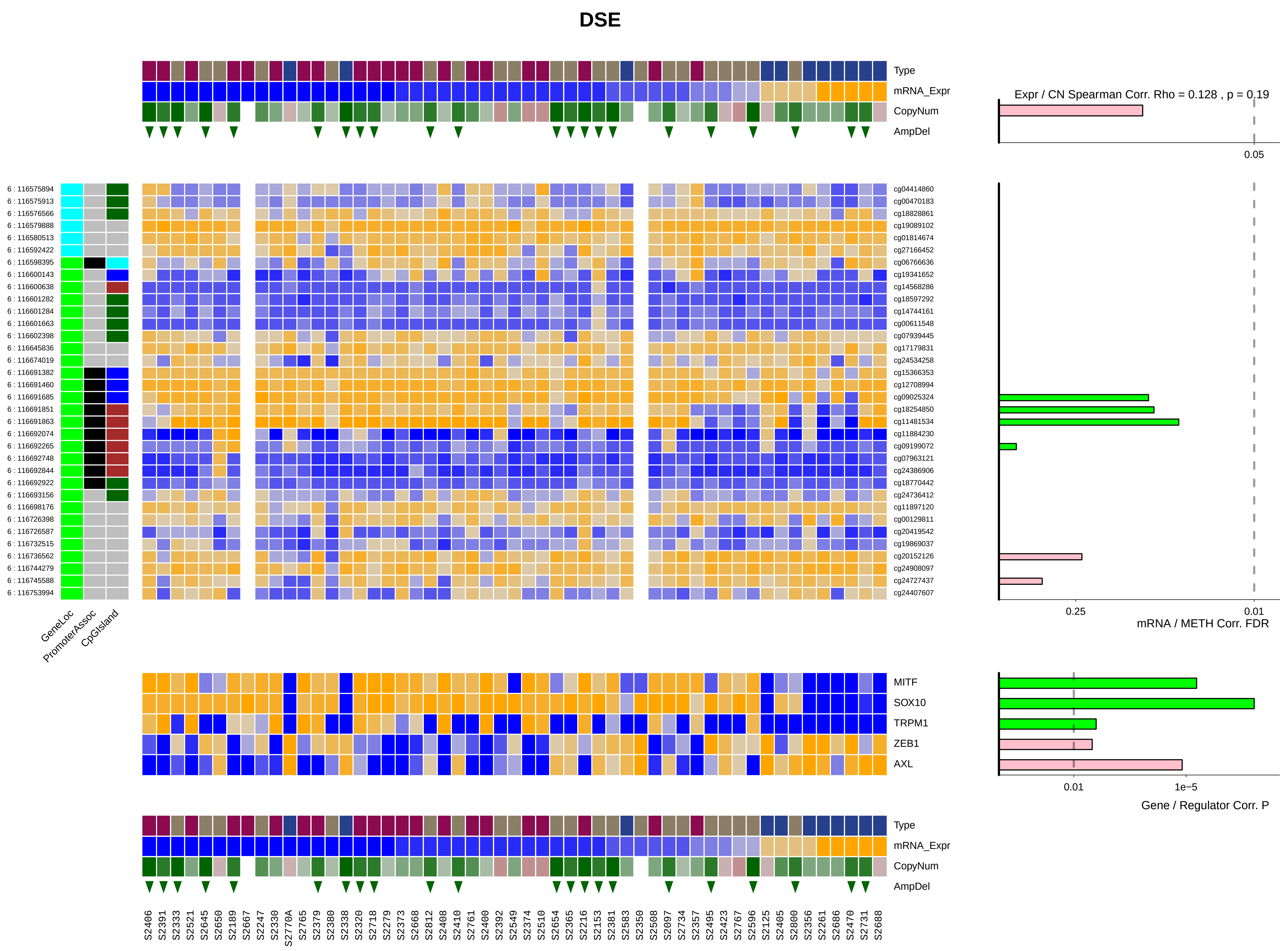

DSE
Type
mRNA_Expr
CopyNum
AmpDel
Expr / CN Spearman Corr. Rho = 0.128 , p = 0.19
0.05
6 : 116575894
6 : 116575913
6 : 116576566
6 : 116579888
6 : 116580513
6 : 116592422
6 : 116598395
6 : 116600143
6 : 116600638
6 : 116601282
6 : 116601284
6 : 116601663
6 : 116602398
6 : 116645836
6 : 116674019
6 : 116691382
6 : 116691460
6 : 116691685
6 : 116691851
6 : 116691863
6 : 116692074
6 : 116692265
6 : 116692748
6 : 116692844
6 : 116692922
6 : 116693156
6 : 116698176
6 : 116726398
6 : 116726587
6 : 116732515
6 : 116736562
6 : 116744279
6 : 116745588
6 : 116753994
cg04414860
cg00470183
cg18828861
cg19089102
cg01814674
cg27166452
cg06766636
cg19341652
cg14568286
cg18597292
cg14744161
cg00611548
cg07939445
cg17179831
cg24534258
cg15366353
cg12708994
cg09025324
cg18254850
cg11481534
cg11884230
cg09199072
cg07963121
cg24386906
cg18770442
cg24736412
cg11897120
cg00129811
cg20419542
cg19869037
cg20152126
cg24908097
cg24727437
cg24407607
0.25
0.01
mRNA / METH Corr. FDR
GeneLoc
PromoterAssoc
CpGIsland
MITF
SOX10
TRPM1
ZEB1
AXL
0.01
1e−5
Gene / Regulator Corr. P
Type
mRNA_Expr
CopyNum
AmpDel
S2406
S2391
S2333
S2521
S2645
S2650
S2189
S2667
S2247
S2330
S2770A
S2765
S2379
S2380
S2338
S2320
S2718
S2279
S2373
S2668
S2812
S2408
S2410
S2761
S2400
S2392
S2549
S2374
S2510
S2654
S2365
S2216
S2153
S2381
S2583
S2350
S2508
S2097
S2734
S2357
S2495
S2423
S2767
S2596
S2125
S2405
S2800
S2356
S2261
S2686
S2470
S2731
S2688

ESM1

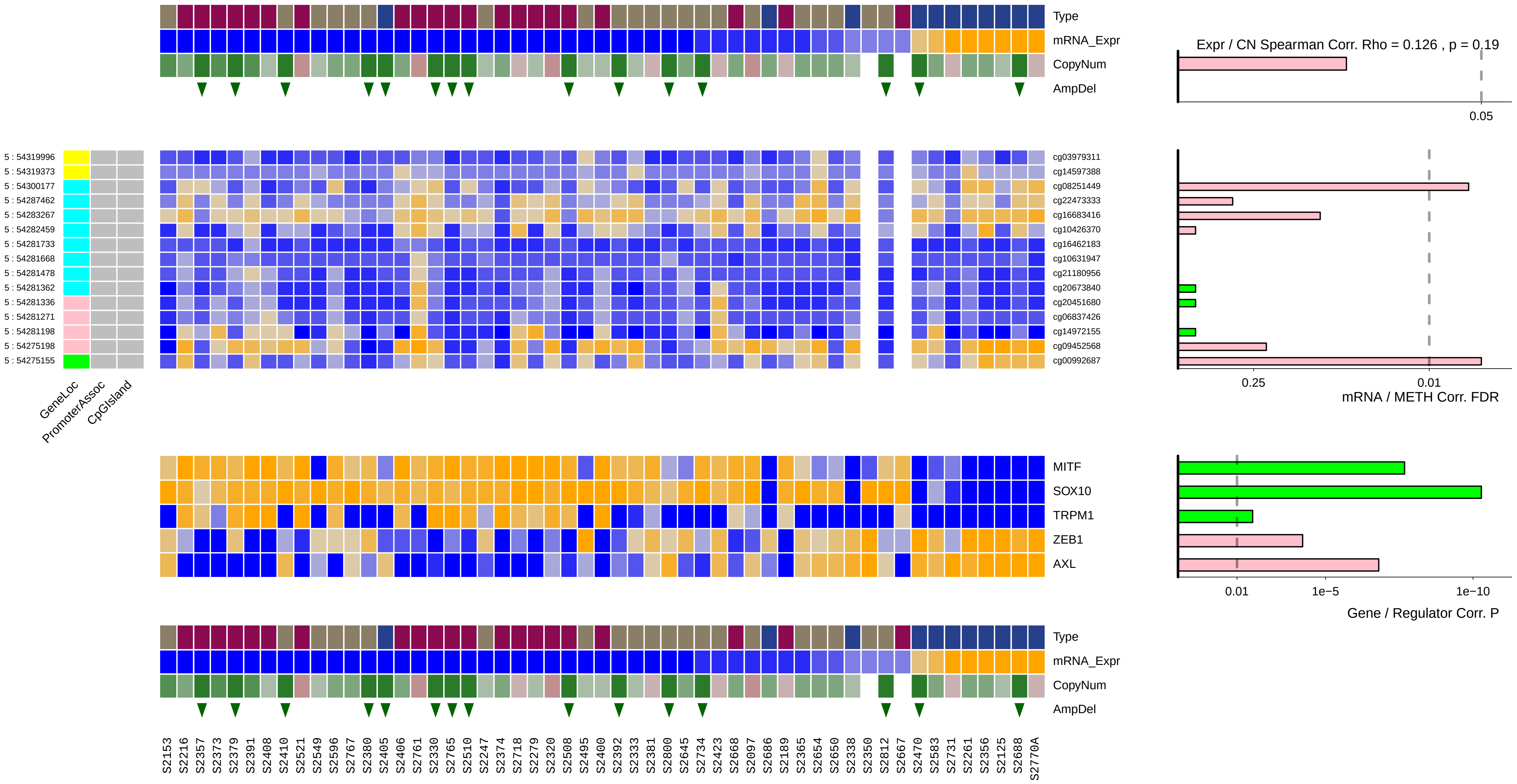
Type
mRNA_Expr
CopyNum
AmpDel
Expr / CN Spearman Corr. Rho = 0.126 , p = 0.19
0.05
5 : 54319996
5 : 54319373
5 : 54300177
5 : 54287462
5 : 54283267
5 : 54282459
5 : 54281733
5 : 54281668
5 : 54281478
5 : 54281362
5 : 54281336
5 : 54281271
5 : 54281198
5 : 54275198
5 : 54275155
GeneLoc
PromoterAssoc
CpGIsland
cg03979311
cg14597388
cg08251449
cg22473333
cg16683416
cg10426370
cg16462183
cg10631947
cg21180956
cg20673840
cg20451680
cg06837426
cg14972155
cg09452568
cg00992687
0.25
0.01
mRNA / METH Corr. FDR
MITF
SOX10
TRPM1
ZEB1
AXL
0.01
1e−5
1e−10
Gene / Regulator Corr. P
Type
mRNA_Expr
CopyNum
AmpDel
S2153 S2216 S2357 S2373 S2379 S2391 S2408 S2410 S2521 S2549 S2596 S2767 S2380 S2405 S2406 S2761 S2330 S2765 S2510 S2247 S2374 S2718 S2279 S2320 S2508 S2495 S2400 S2392 S2333 S2381 S2800 S2645 S2734 S2423 S2668 S2097 S2686 S2189 S2365 S2654 S2650 S2338 S2350 S2812 S2667 S2470 S2583 S2731 S2261 S2356 S2125 S2688 S2770A

SERPINB7

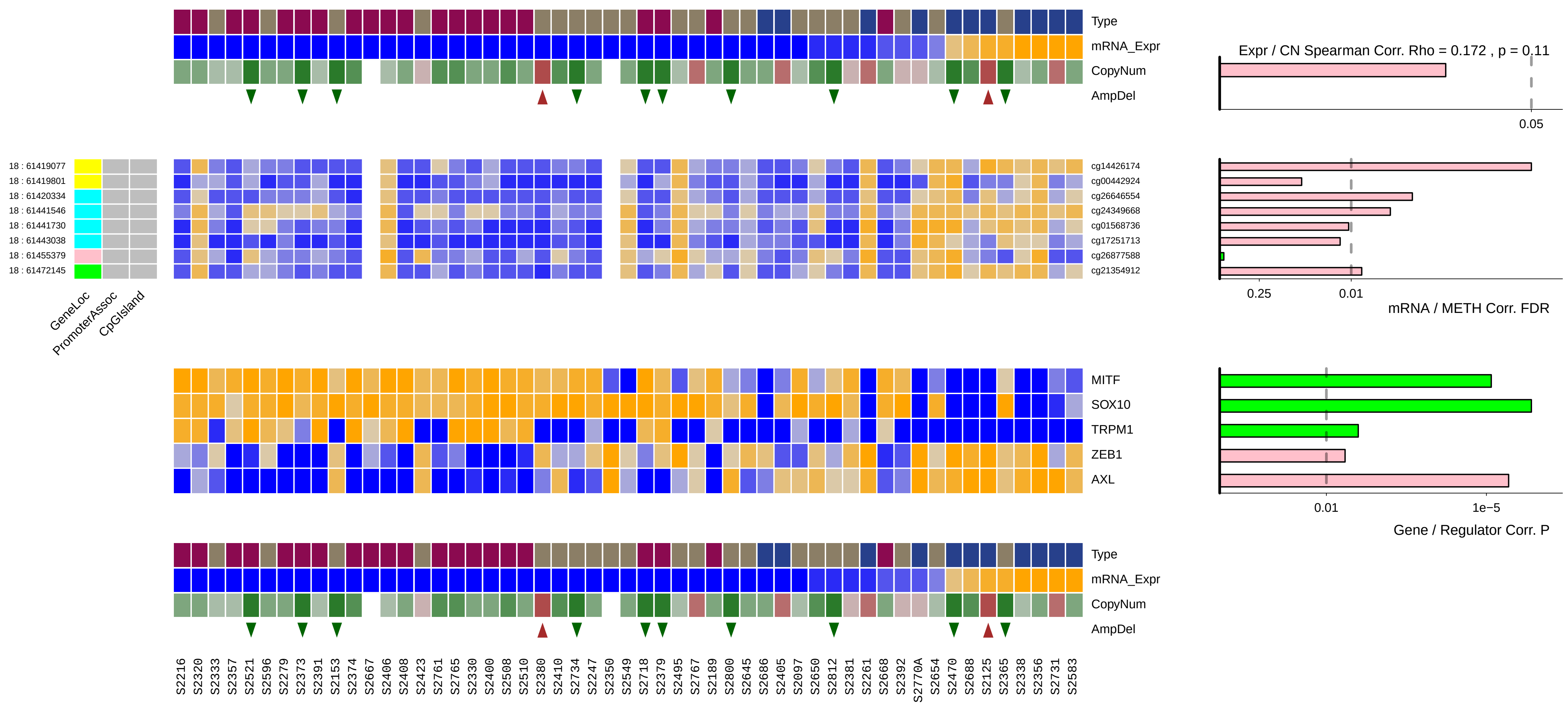

Type
mRNA_Expr
CopyNum
AmpDel
Expr / CN Spearman Corr. Rho = 0.172 , p = 0.11
0.05
18 : 61419077
18 : 61419801
18 : 61420334
18 : 61441546
18 : 61441730
18 : 61443038
18 : 61455379
18 : 61472145
GeneLoc
PromoterAssoc
CpGIsland
cg14426174
cg00442924
cg26646554
cg24349668
cg01568736
cg17251713
cg26877588
cg21354912
0.25
0.01
mRNA / METH Corr. FDR
MITF
SOX10
TRPM1
ZEB1
AXL
0.01
1e−5
Gene / Regulator Corr. P
S2216 S2320 S2333 S2357 S2521 S2596 S2279 S2373 S2391 S2153 S2374 S2667 S2406 S2408 S2423 S2761 S2765 S2330 S2400 S2508 S2510 S2380 S2410 S2734 S2247 S2350 S2549 S2718 S2379 S2495 S2767 S2189 S2800 S2645 S2686 S2405 S2097 S2650 S2812 S2381 S2261 S2668 S2392 S2770A S2654 S2470 S2688 S2125 S2365 S2338 S2356 S2731 S2583

PLPP3

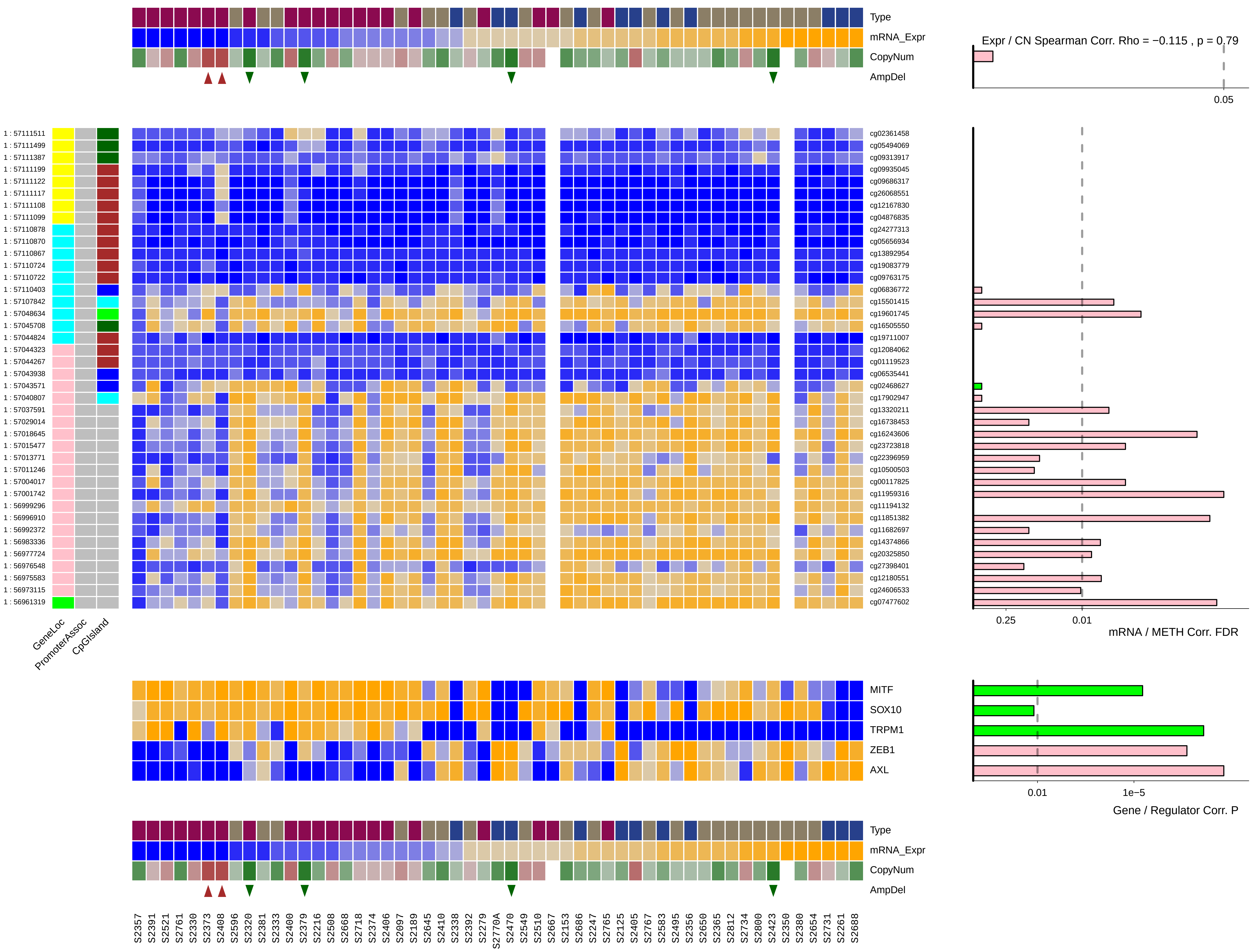
Type
mRNA_Expr
CopyNum
AmpDel
Expr / CN Spearman Corr. Rho = −0.115 , p = 0.79
0.05
GeneLoc
PromoterAssoc
CpGIsland
mRNA / METH Corr. FDR
0.25
0.01
MITF
SOX10
TRPM1
ZEB1
AXL
1e−5
Gene / Regulator Corr. P

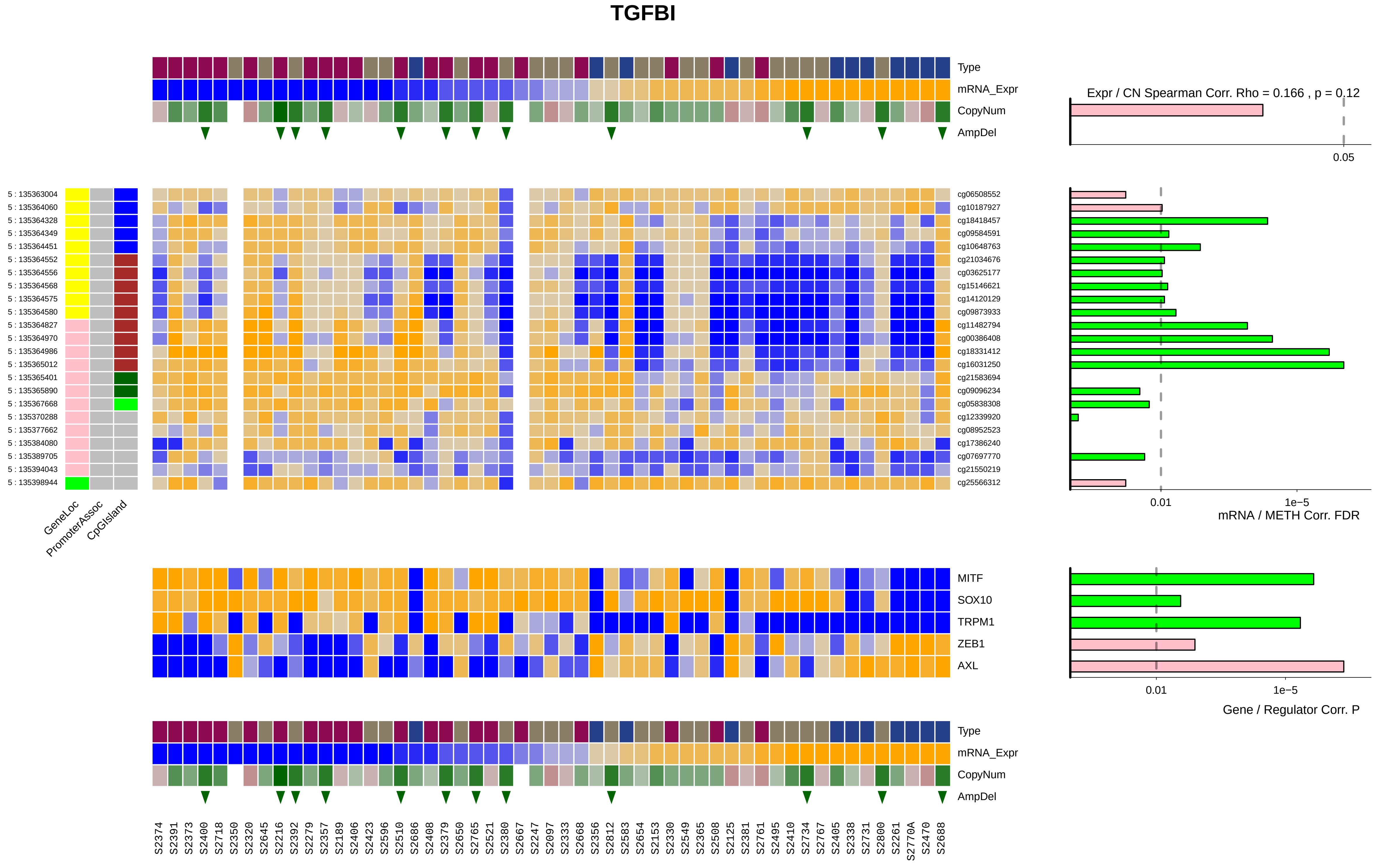
TGFBI
Type
mRNA_Expr
CopyNum
AmpDel
Expr / CN Spearman Corr. Rho = 0.166 , p = 0.12
0.05
mRNA / METH Corr. FDR
0.01
1e−5
GeneLoc
PromoterAssoc
CpGIsland
MITF
SOX10
TRPM1
ZEB1
AXL
Gene / Regulator Corr. P
