# Supplementary material for: Multi-modal molecular programs regulate melanoma cell state
Source: Nat Commun. 2022 Jul 9;13:4000. doi: 10.1038/s41467-022-31510-1 (PMC9271073; doi:10.1038/s41467-022-31510-1)
Supplement: Supplementary file 13 — Supplementary Data 10 [file 41467_2022_31510_MOESM13_ESM.pdf]

Supplementary Data 10: MES gene regulatory influences - TCGA melanomas.

EGFR

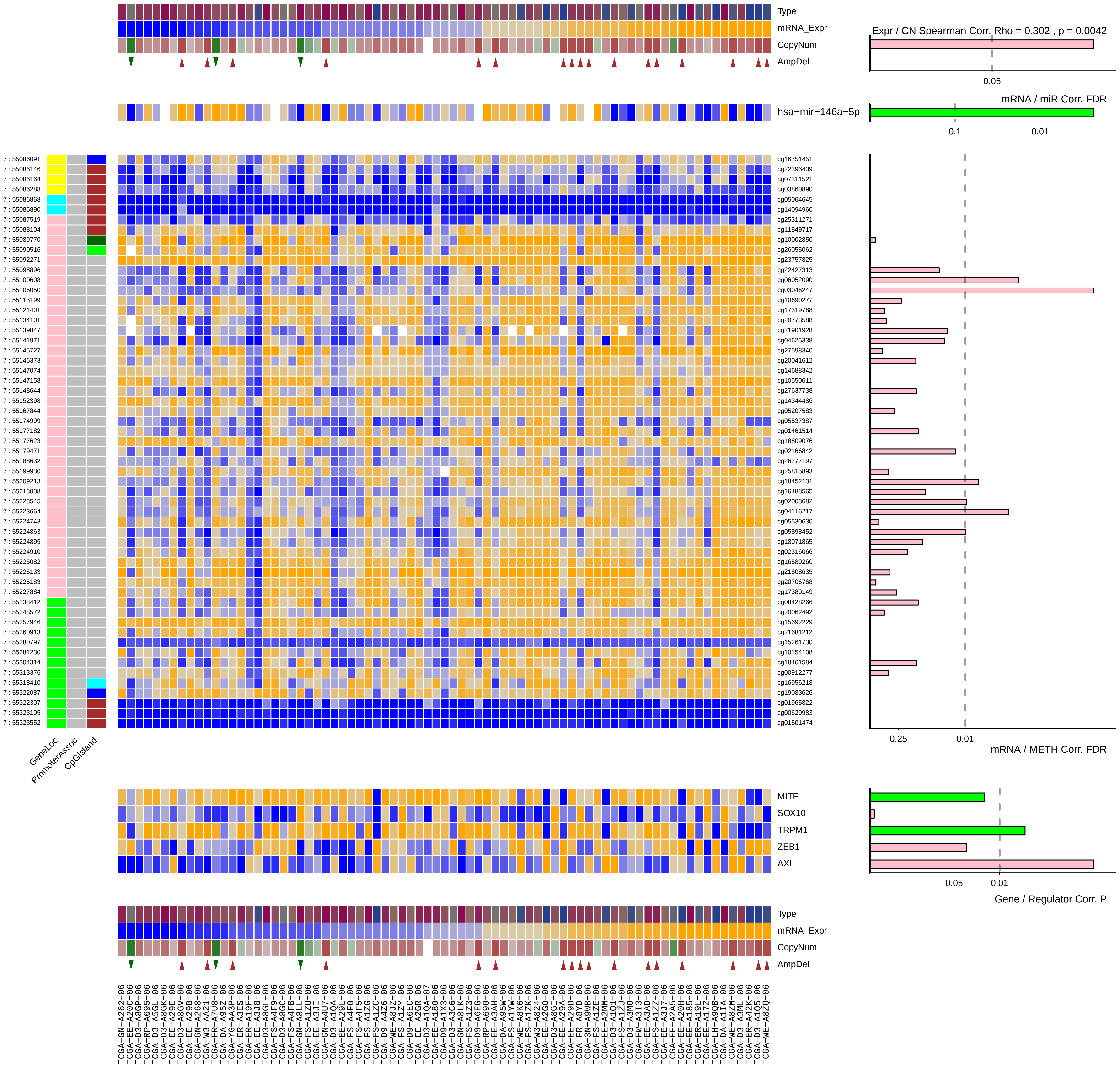

Type
mRNA_Expr
CopyNum
AmpDel
Expr / CN Spearman Corr. Rho = 0.302 , p = 0.0042
0.05
mRNA / miR Corr. FDR
hsa−mir−146a−5p
0.1
0.01
GeneLoc
PromoterAssoc
CpGIsland
0.25
0.01
mRNA / METH Corr. FDR
MITF
SOX10
TRPM1
ZEB1
AXL
0.05
0.01
Gene / Regulator Corr. P

TSPAN5

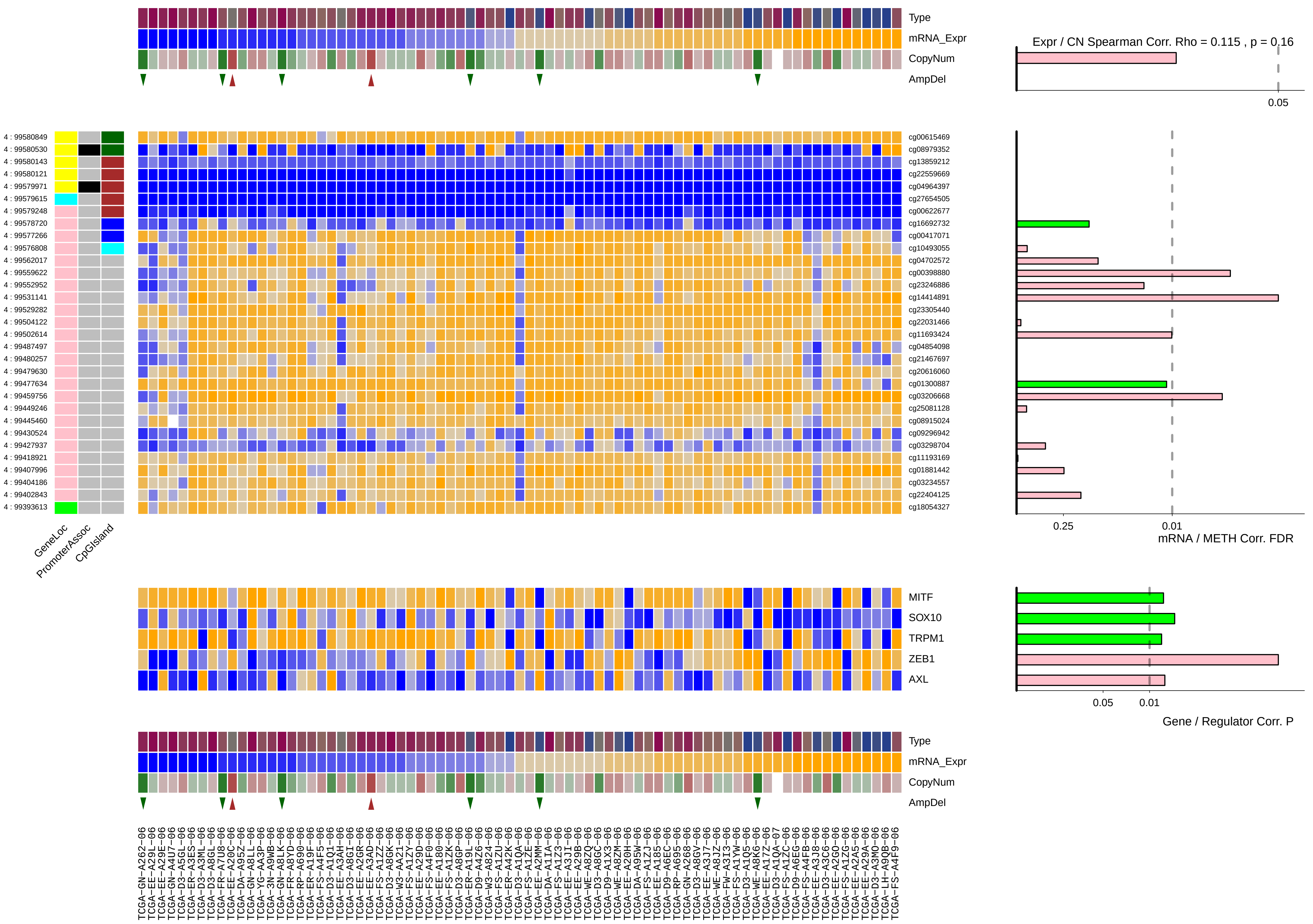
Type
mRNA_Expr
CopyNum
AmpDel
Expr / CN Spearman Corr. Rho = 0.115 , p = 0.16
0.05
GeneLoc
PromoterAssoc
CpGIsland
mRNA / METH Corr. FDR
0.25
0.01
MITF
SOX10
TRPM1
ZEB1
AXL
Gene / Regulator Corr. P
0.05
0.01

NRP1

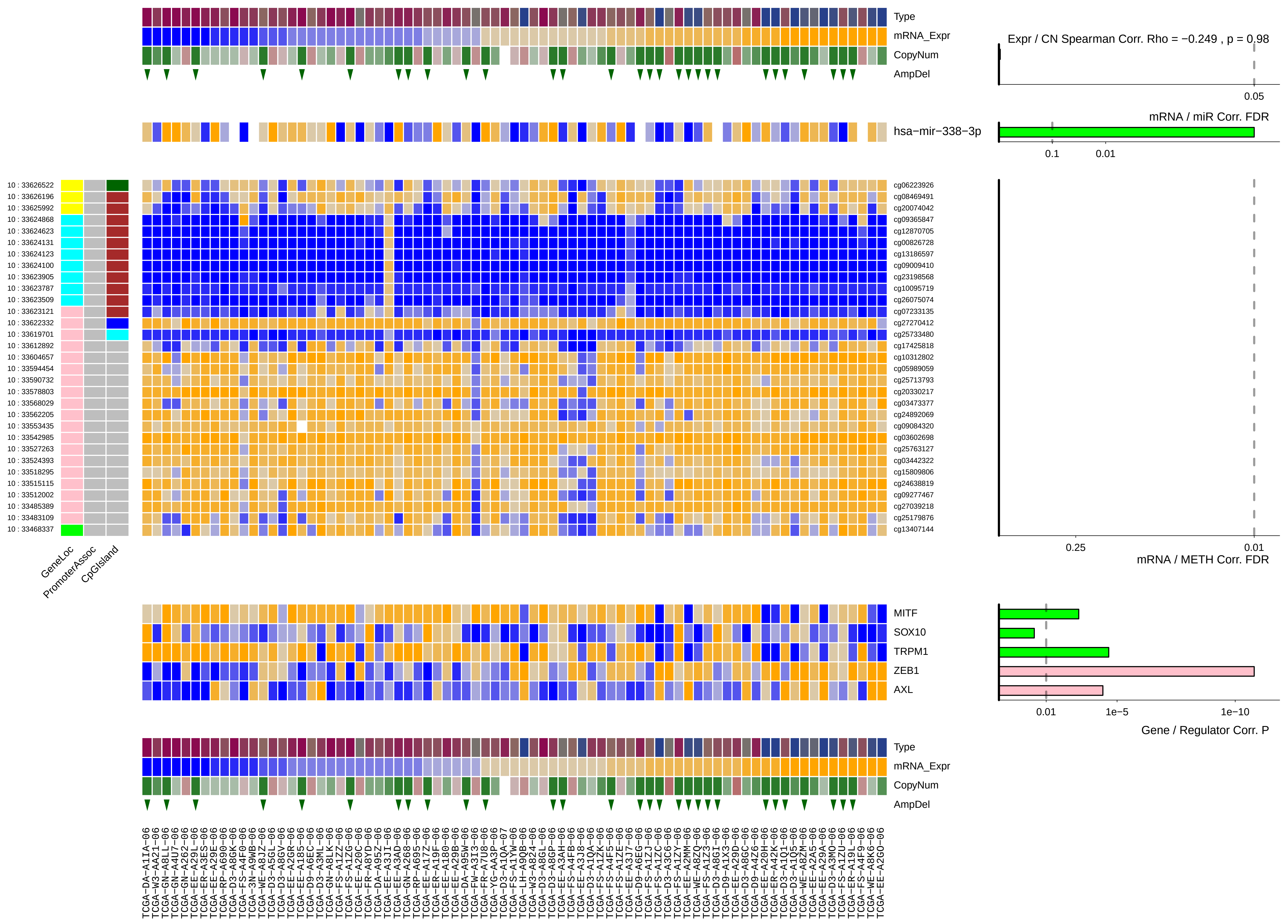
Type
mRNA_Expr
CopyNum
AmpDel
Expr / CN Spearman Corr. Rho = −0.249 , p = 0.98
0.05
hsa−mir−338−3p
mRNA / miR Corr. FDR
0.1
0.01
GeneLoc
PromoterAssoc
CpGIsland
0.25
0.01
mRNA / METH Corr. FDR
MITF
SOX10
TRPM1
ZEB1
AXL
0.01
1e−5
1e−10
Gene / Regulator Corr. P

BASP1

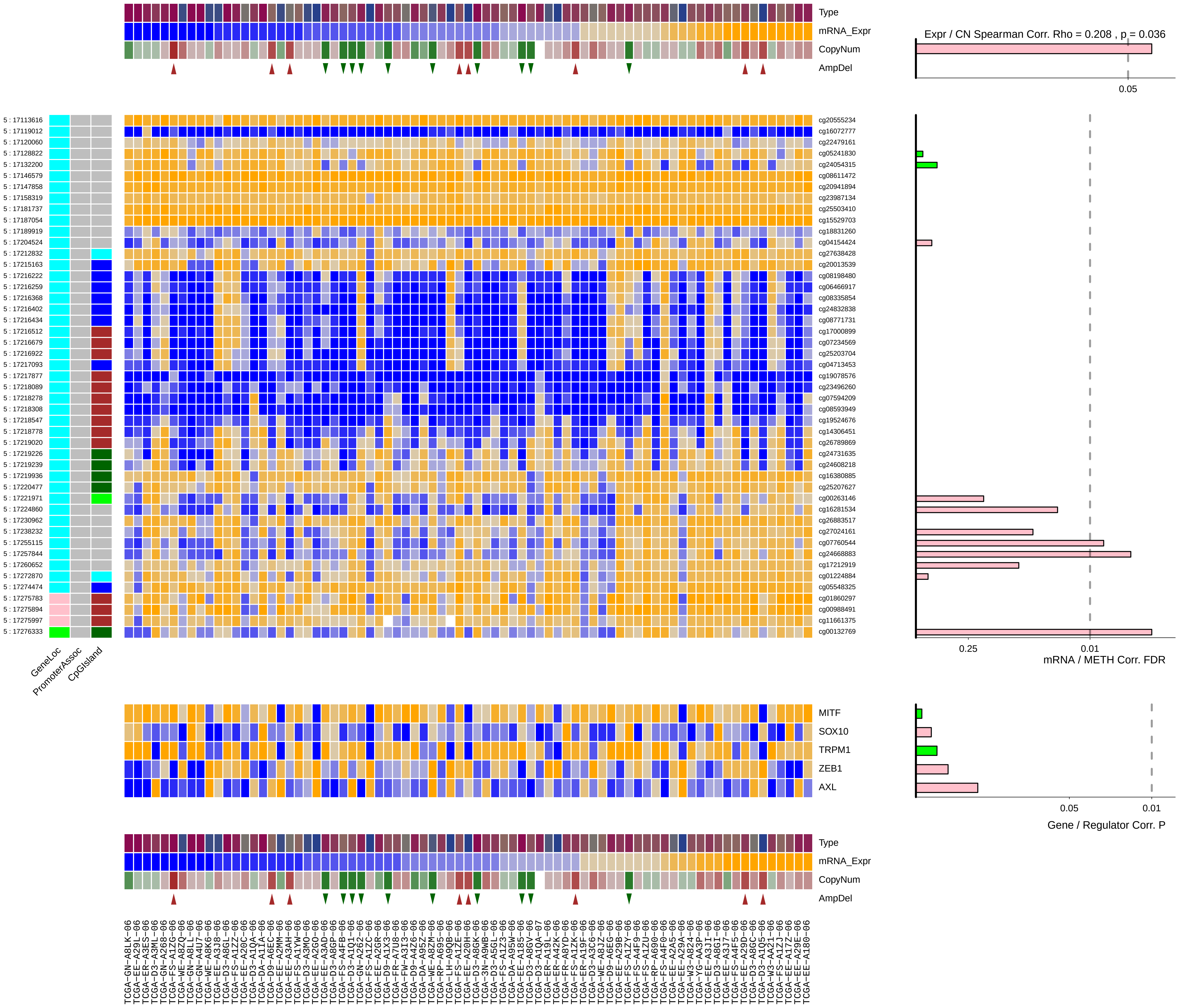

Type
mRNA_Expr
CopyNum
AmpDel
Expr / CN Spearman Corr. Rho = 0.208 , p = 0.036
0.05
5 : 17113616 cg20555234
5 : 17119012 cg16072777
5 : 17120060 cg22479161
5 : 17128822 cg05241830
5 : 17132200 cg24054315
5 : 17146579 cg08611472
5 : 17147858 cg20941894
5 : 17158319 cg23987134
5 : 17181737 cg25503410
5 : 17187054 cg15529703
5 : 17189919 cg18831260
5 : 17204524 cg04154424
5 : 17212832 cg27638428
5 : 17215163 cg20013539
5 : 17216222 cg08198480
5 : 17216259 cg06466917
5 : 17216368 cg08335854
5 : 17216402 cg24832838
5 : 17216434 cg08771731
5 : 17216512 cg17000899
5 : 17216679 cg07234569
5 : 17216922 cg25203704
5 : 17217093 cg04713453
5 : 17217877 cg19078576
5 : 17218089 cg23496260
5 : 17218278 cg07594209
5 : 17218308 cg08593949
5 : 17218547 cg19524676
5 : 17218778 cg14306451
5 : 17219020 cg26789869
5 : 17219226 cg24731635
5 : 17219239 cg24608218
5 : 17219936 cg16380885
5 : 17220477 cg25207627
5 : 17221971 cg00263146
5 : 17224860 cg16281534
5 : 17230962 cg26883517
5 : 17238232 cg27024161
5 : 17255115 cg07760544
5 : 17257844 cg24668883
5 : 17260652 cg17212919
5 : 17272870 cg01224884
5 : 17274474 cg05548325
5 : 17275783 cg01860297
5 : 17275894 cg00988491
5 : 17275997 cg11661375
5 : 17276333 cg00132769
GeneLoc
PromoterAssoc
CpGIsland
0.25
0.01
mRNA / METH Corr. FDR
MITF
SOX10
TRPM1
ZEB1
AXL
0.05
0.01
Gene / Regulator Corr. P
Type
mRNA_Expr
CopyNum
AmpDel

TMEM200A

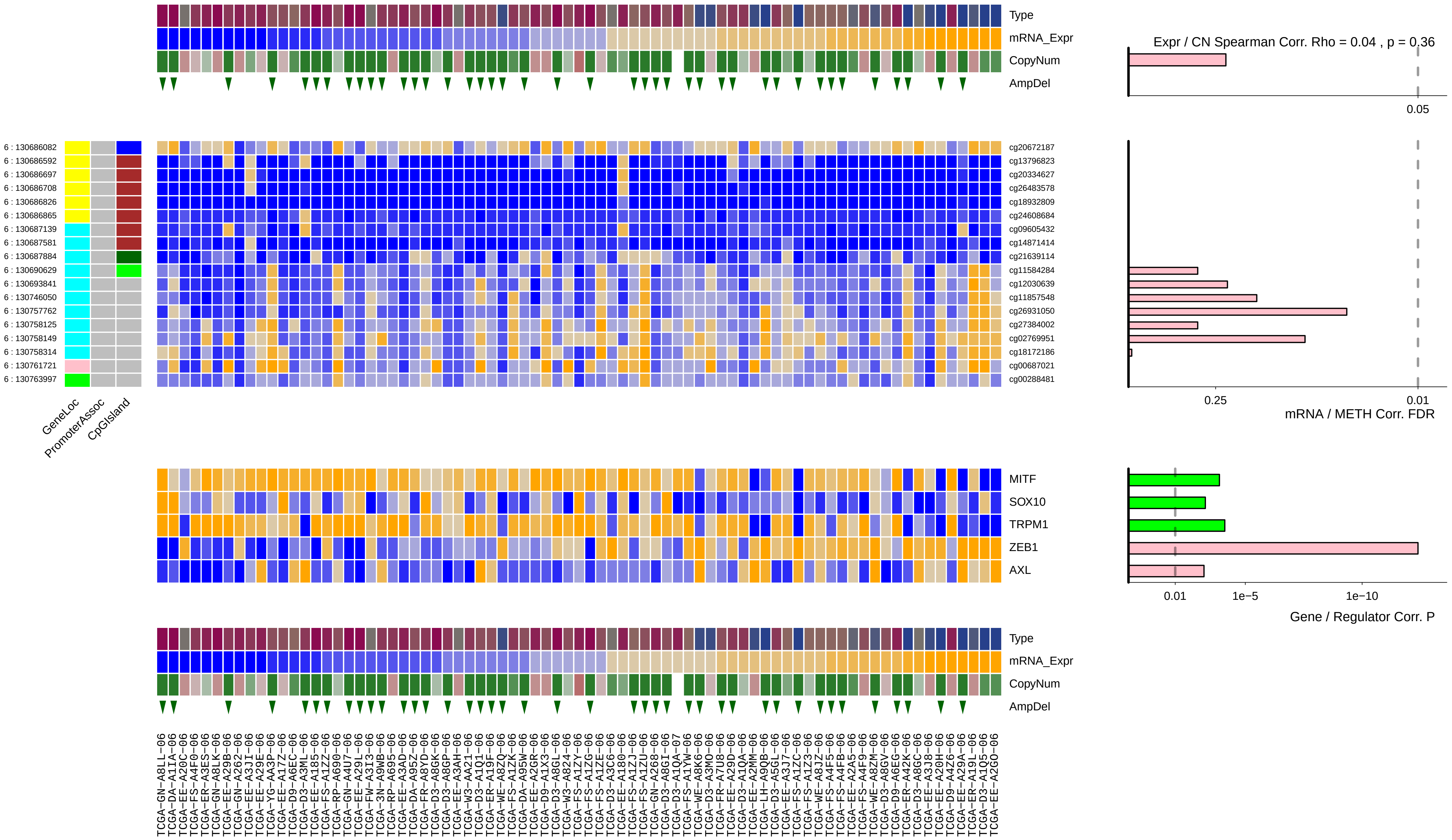

Type
mRNA_Expr
CopyNum
AmpDel
Expr / CN Spearman Corr. Rho = 0.04 , p = 0.36
0.05
mRNA / METH Corr. FDR
0.25
0.01
GeneLoc
PromoterAssoc
CpGIsland
MITF
SOX10
TRPM1
ZEB1
AXL
Gene / Regulator Corr. P
0.01
1e−5
1e−10

LOXL2

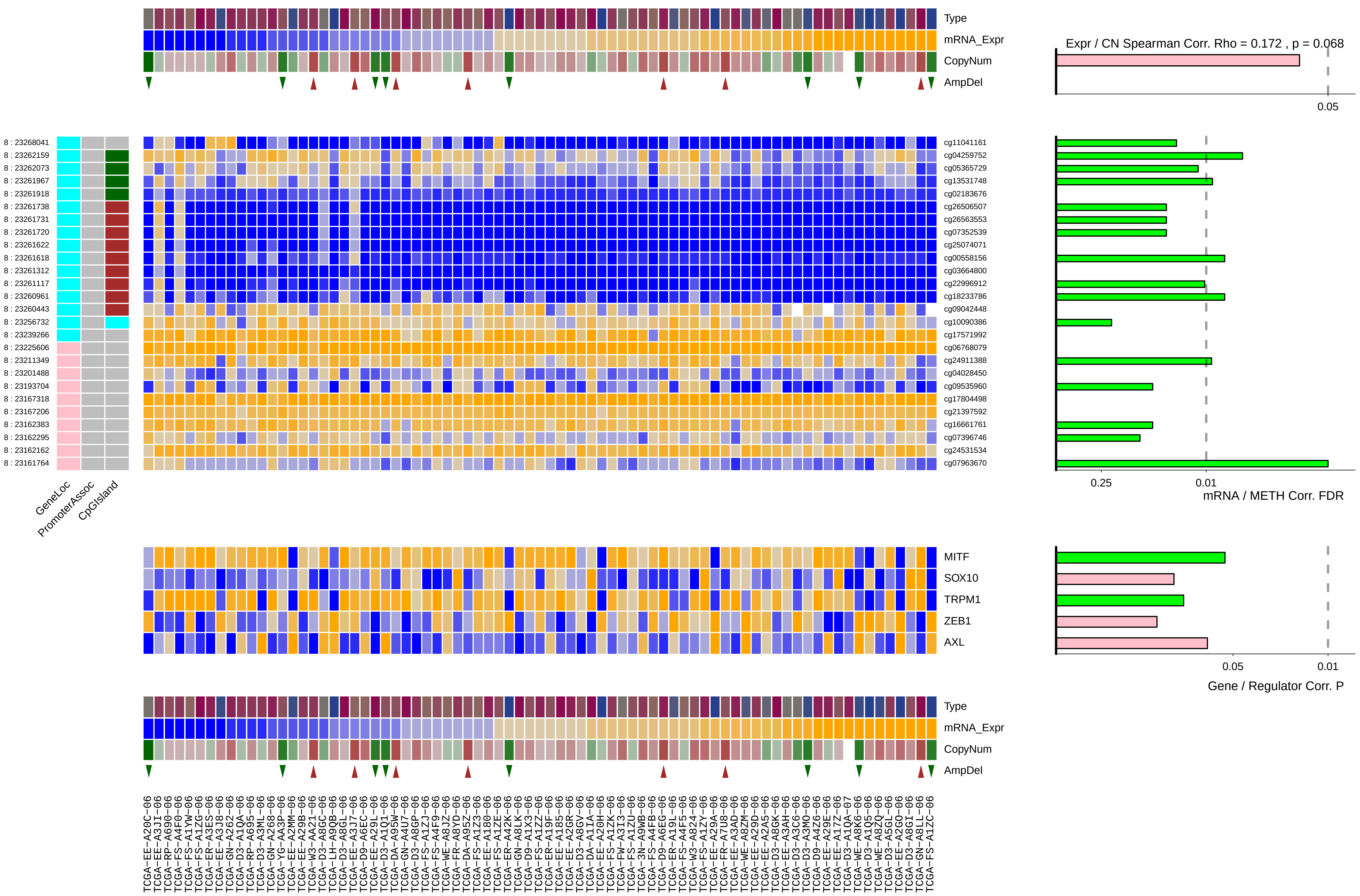
Type
mRNA_Expr
CopyNum
AmpDel
Expr / CN Spearman Corr. Rho = 0.172 , p = 0.068
0.05
GeneLoc
PromoterAssoc
CpGIsland
0.25
0.01
mRNA / METH Corr. FDR
MITF
SOX10
TRPM1
ZEB1
AXL
0.05
0.01
Gene / Regulator Corr. P

CRIM1

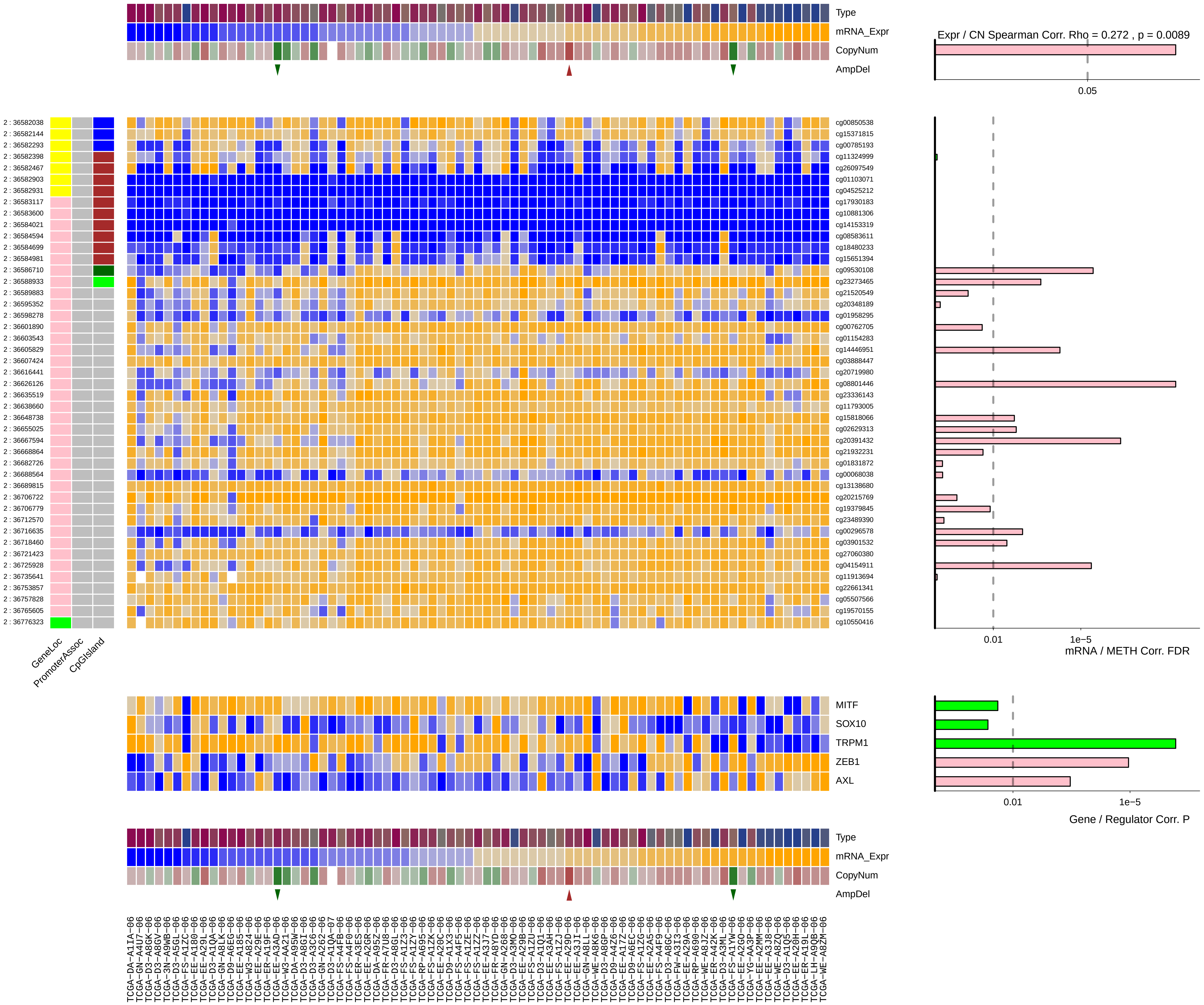
Type
mRNA_Expr
CopyNum
AmpDel
Expr / CN Spearman Corr. Rho = 0.272 , p = 0.0089
0.05
cg00850538
cg15371815
cg00785193
cg11324999
cg26097549
cg01103071
cg04525212
cg17930183
cg10881306
cg14153319
cg08583611
cg18480233
cg15651394
cg09530108
cg23273465
cg21520549
cg20348189
cg01958295
cg00762705
cg01154283
cg14446951
cg03888447
cg20719980
cg08801446
cg23336143
cg11793005
cg15818066
cg02629313
cg20391432
cg21932231
cg01831872
cg00068038
cg13138680
cg20215769
cg19379845
cg23489390
cg00296578
cg03901532
cg27060380
cg04154911
cg11913694
cg22661341
cg05507566
cg19570155
cg10550416
0.01
1e−5
mRNA / METH Corr. FDR
GeneLoc
PromoterAssoc
CpGIsland
MITF
SOX10
TRPM1
ZEB1
AXL
0.01
1e−5
Gene / Regulator Corr. P
Type
mRNA_Expr
CopyNum
AmpDel

ANTXR2

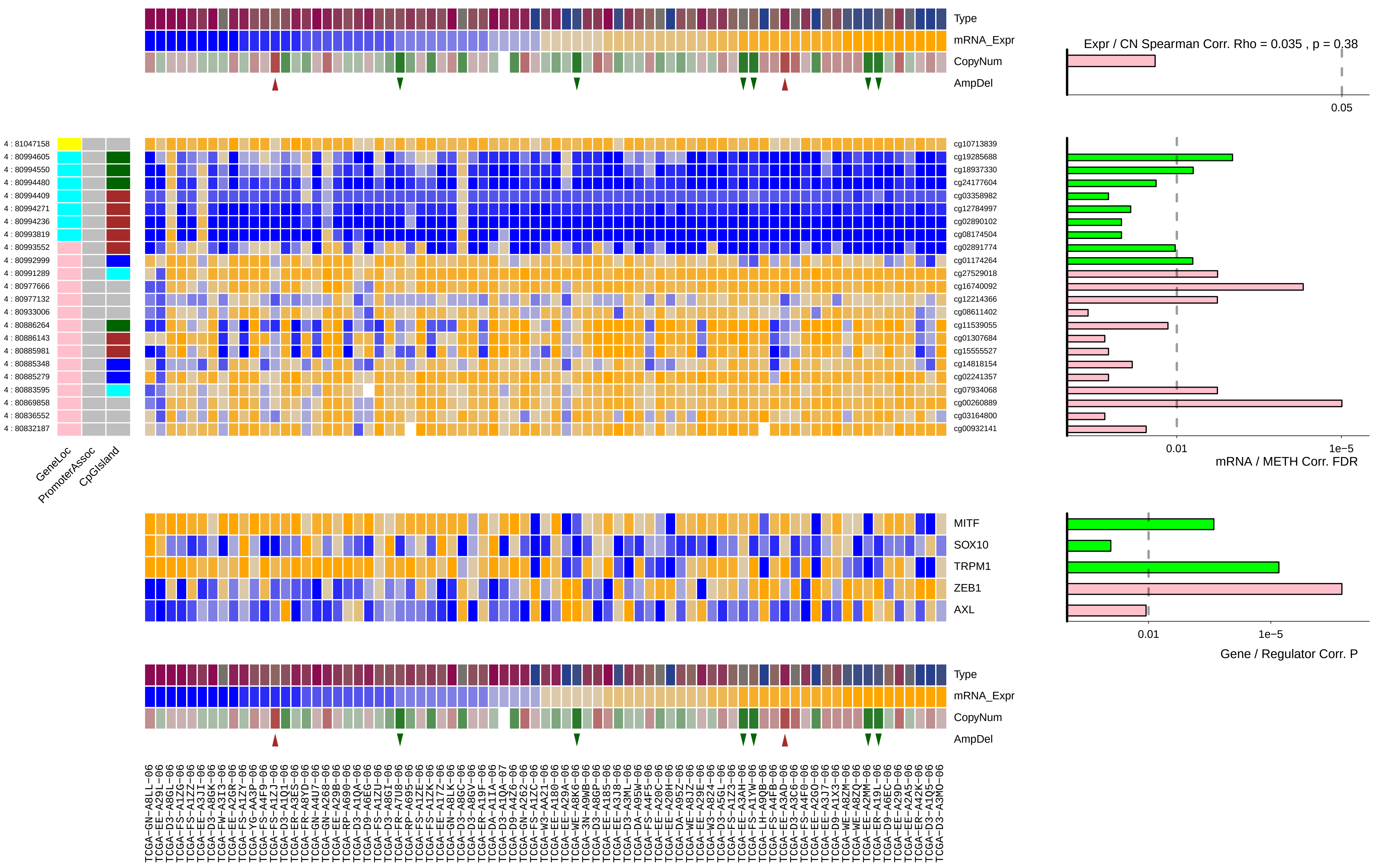
Type
mRNA_Expr
CopyNum
AmpDel
Expr / CN Spearman Corr. Rho = 0.035 , p = 0.38
0.05
GeneLoc
PromoterAssoc
CpGIsland
0.01
1e−5
mRNA / METH Corr. FDR
MITF
SOX10
TRPM1
ZEB1
AXL
Gene / Regulator Corr. P

KCNMA1

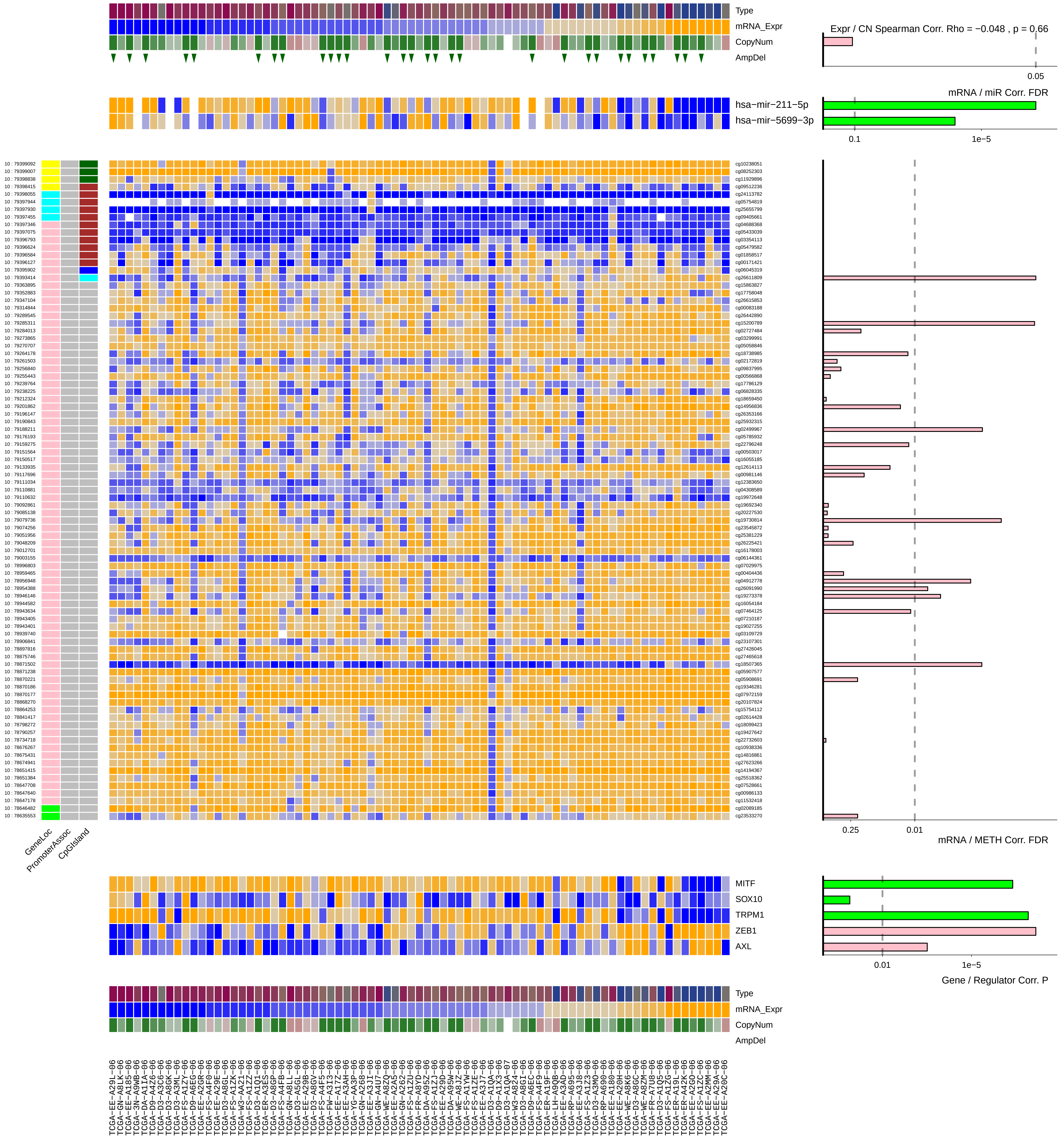

Type
mRNA_Expr
CopyNum
AmpDel
Expr / CN Spearman Corr. Rho = −0.048 , p = 0.66
0.05
mRNA / miR Corr. FDR
hsa−mir−211−5p
hsa−mir−5699−3p
0.1
1e−5
GeneLoc
PromoterAssoc
CpGIsland
0.25
0.01
mRNA / METH Corr. FDR
MITF
SOX10
TRPM1
ZEB1
AXL
0.01
1e−5
Gene / Regulator Corr. P

AXL

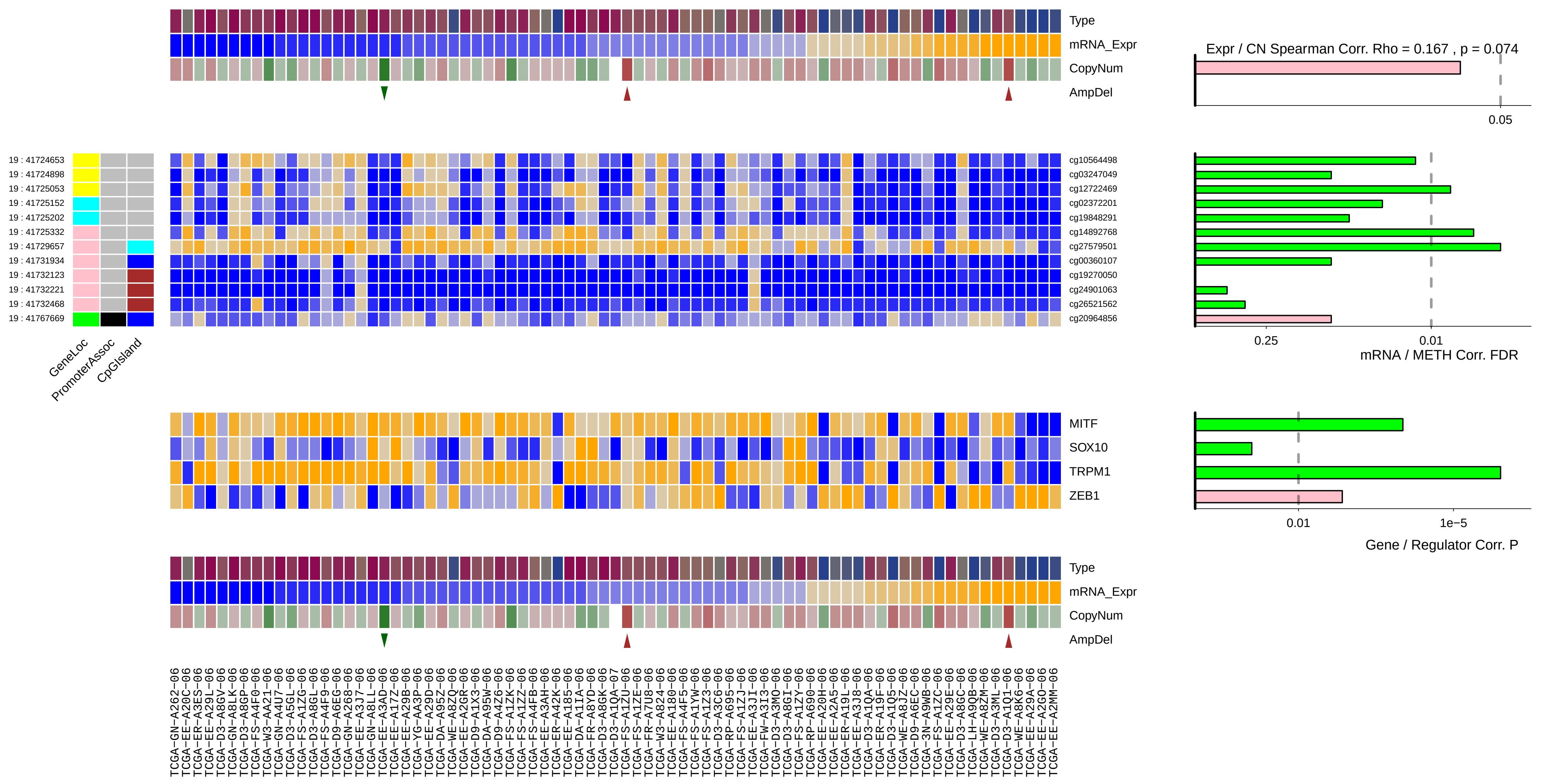
Type
mRNA_Expr
CopyNum
AmpDel
Expr / CN Spearman Corr. Rho = 0.167 , p = 0.074
0.05
19 : 41724653
19 : 41724898
19 : 41725053
19 : 41725152
19 : 41725202
19 : 41725332
19 : 41729657
19 : 41731934
19 : 41732123
19 : 41732221
19 : 41732468
19 : 41767669
GeneLoc
PromoterAssoc
CpGIsland
cg10564498
cg03247049
cg12722469
cg02372201
cg19848291
cg14892768
cg27579501
cg00360107
cg19270050
cg24901063
cg26521562
cg20964856
0.25
0.01
mRNA / METH Corr. FDR
MITF
SOX10
TRPM1
ZEB1
0.01
1e−5
Gene / Regulator Corr. P
Type
mRNA_Expr
CopyNum
AmpDel

COL13A1

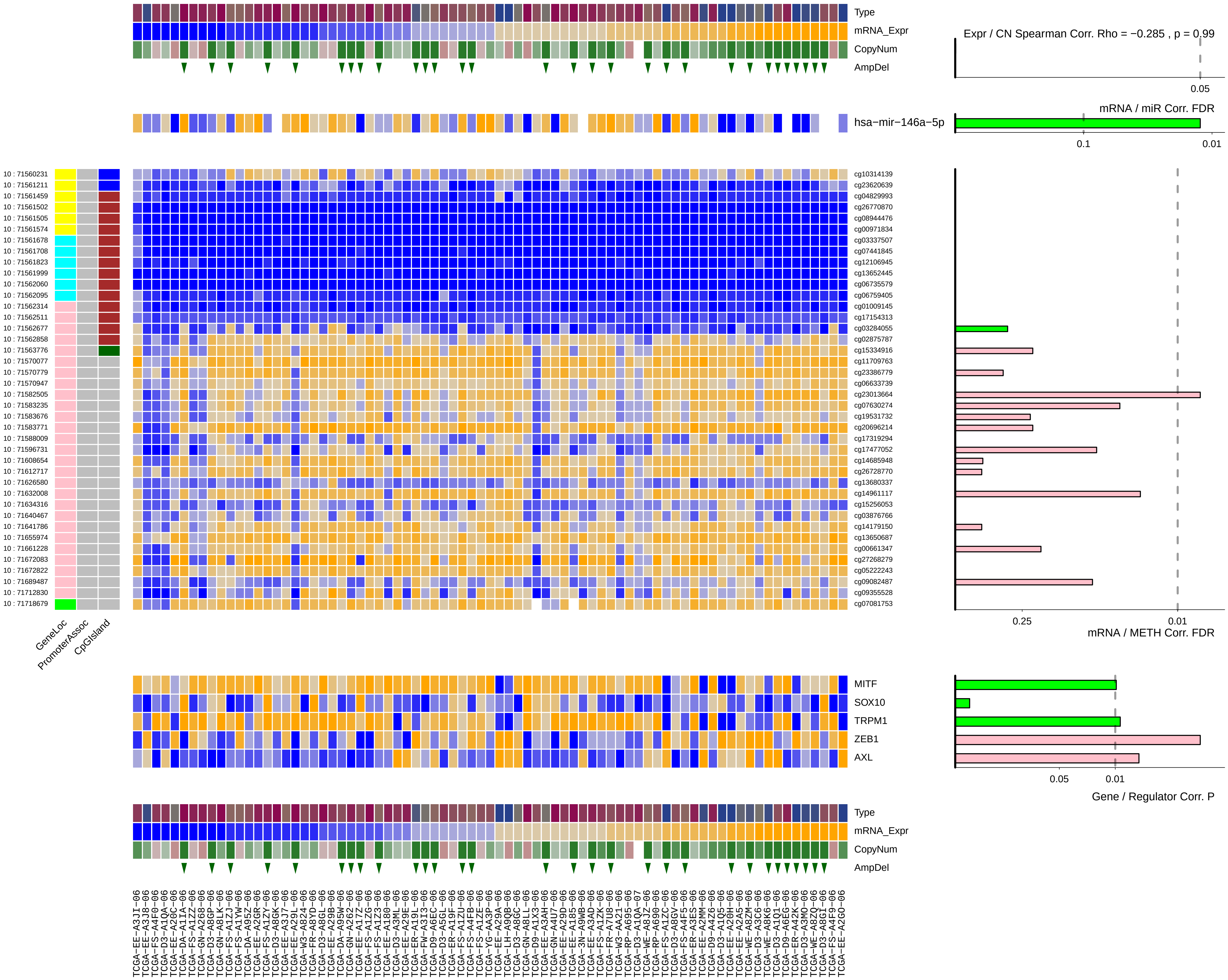

Type
mRNA_Expr
CopyNum
AmpDel
Expr / CN Spearman Corr. Rho = −0.285 , p = 0.99
0.05
hsa−mir−146a−5p
mRNA / miR Corr. FDR
0.1
0.01
GeneLoc
PromoterAssoc
CpGIsland
0.25
0.01
mRNA / METH Corr. FDR
MITF
SOX10
TRPM1
ZEB1
AXL
0.05
0.01
Gene / Regulator Corr. P
Type
mRNA_Expr
CopyNum
AmpDel

RIN2

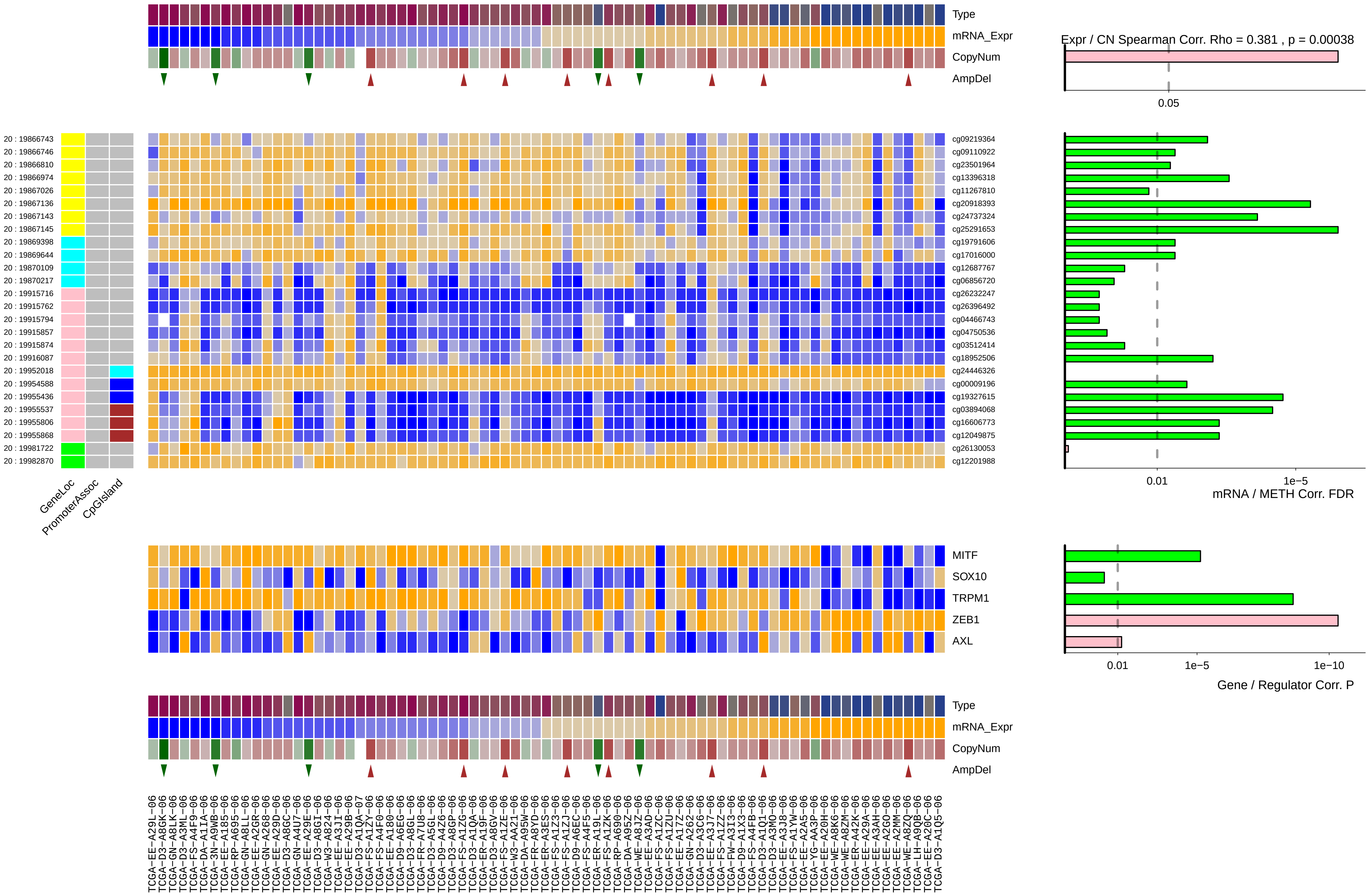
Type
mRNA_Expr
CopyNum
AmpDel
Expr / CN Spearman Corr. Rho = 0.381 , p = 0.00038
0.05
20 : 19866743
20 : 19866746
20 : 19866810
20 : 19866974
20 : 19867026
20 : 19867136
20 : 19867143
20 : 19867145
20 : 19869398
20 : 19869644
20 : 19870109
20 : 19870217
20 : 19915716
20 : 19915762
20 : 19915794
20 : 19915857
20 : 19915874
20 : 19916087
20 : 19952018
20 : 19954588
20 : 19955436
20 : 19955537
20 : 19955806
20 : 19955868
20 : 19981722
20 : 19982870
cg09219364
cg09110922
cg23501964
cg13396318
cg11267810
cg20918393
cg24737324
cg25291653
cg19791606
cg17016000
cg12687767
cg06856720
cg26232247
cg26396492
cg04466743
cg04750536
cg03512414
cg18952506
cg24446326
cg00009196
cg19327615
cg03894068
cg16606773
cg12049875
cg26130053
cg12201988
GeneLoc
PromoterAssoc
CpGIsland
0.01
1e−5
mRNA / METH Corr. FDR
MITF
SOX10
TRPM1
ZEB1
AXL
0.01
1e−5
1e−10
Gene / Regulator Corr. P
Type
mRNA_Expr
CopyNum
AmpDel

ADAMTS12

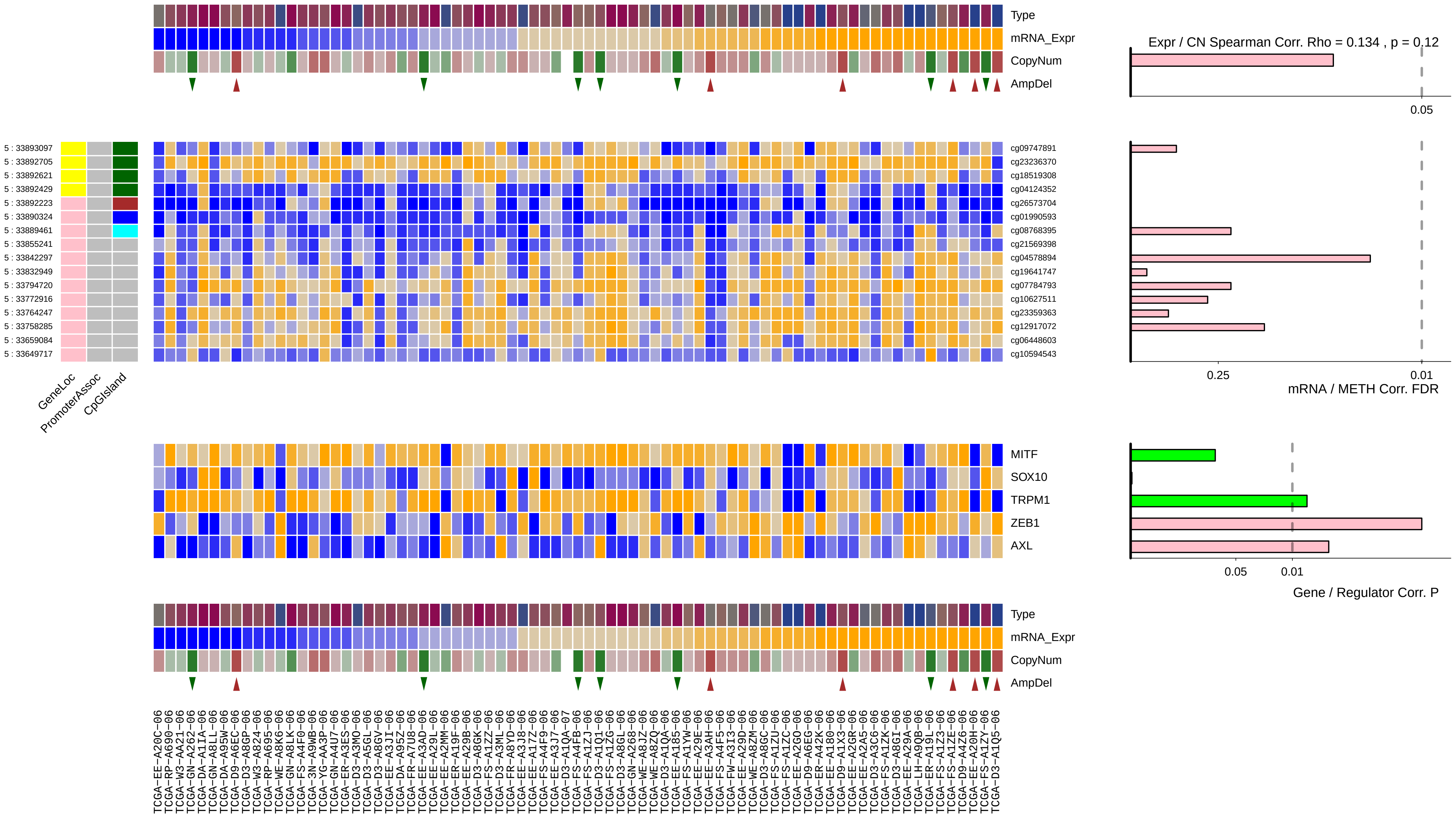
Type
mRNA_Expr
CopyNum
AmpDel
Expr / CN Spearman Corr. Rho = 0.134 , p = 0.12
0.05
5 : 33893097
5 : 33892705
5 : 33892621
5 : 33892429
5 : 33892223
5 : 33890324
5 : 33889461
5 : 33855241
5 : 33842297
5 : 33832949
5 : 33794720
5 : 33772916
5 : 33764247
5 : 33758285
5 : 33659084
5 : 33649717
GeneLoc
PromoterAssoc
CpGIsland
cg09747891
cg23236370
cg18519308
cg04124352
cg26573704
cg01990593
cg08768395
cg21569398
cg04578894
cg19641747
cg07784793
cg10627511
cg23359363
cg12917072
cg06448603
cg10594543
0.25
0.01
mRNA / METH Corr. FDR
MITF
SOX10
TRPM1
ZEB1
AXL
0.05
0.01
Gene / Regulator Corr. P
Type
mRNA_Expr
CopyNum
AmpDel

GPR39

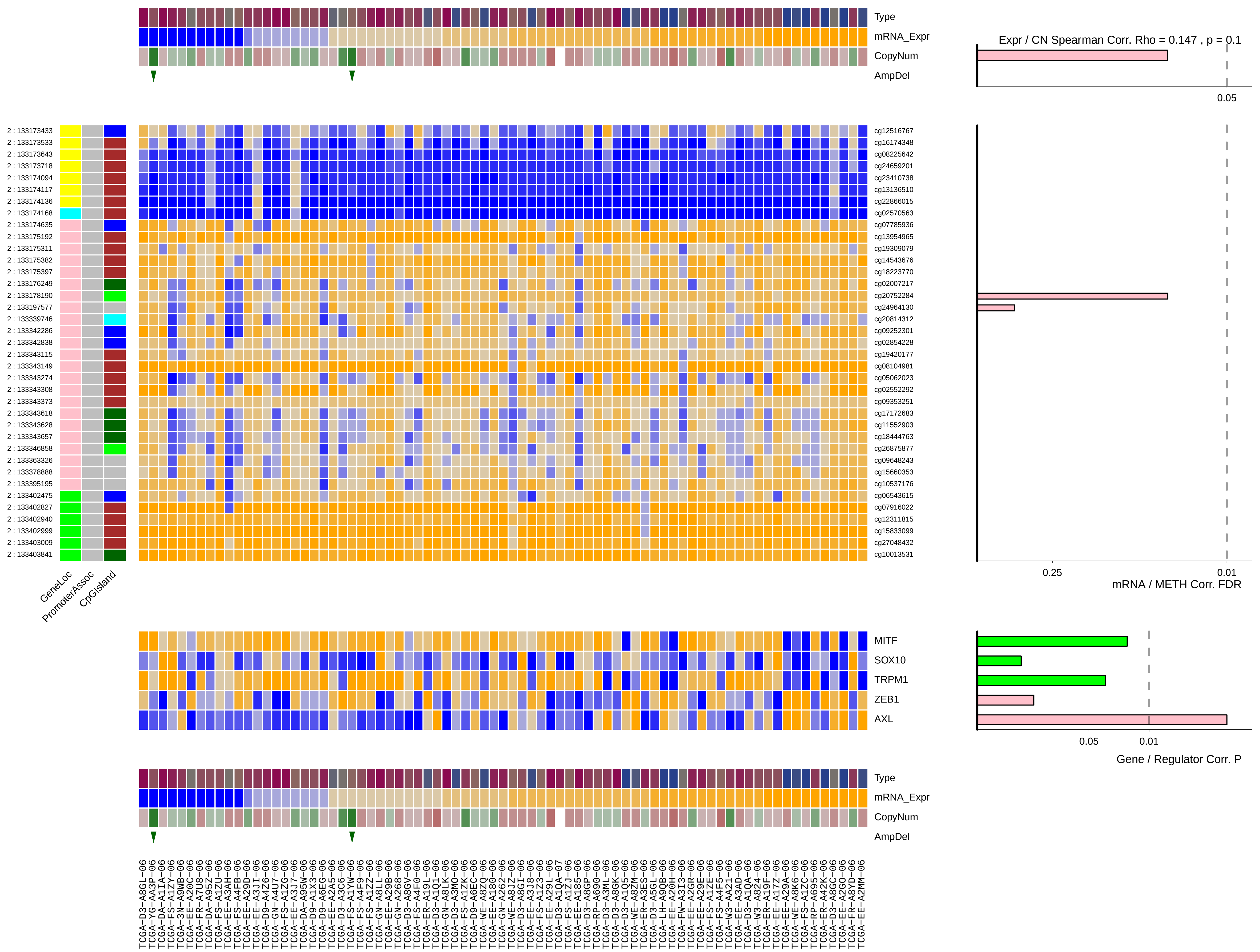
Type
mRNA_Expr
CopyNum
AmpDel
Expr / CN Spearman Corr. Rho = 0.147 , p = 0.1
0.05
2 : 133173433 cg12516767
2 : 133173533 cg16174348
2 : 133173643 cg08225642
2 : 133173718 cg24659201
2 : 133174094 cg23410738
2 : 133174117 cg13136510
2 : 133174136 cg22866015
2 : 133174168 cg02570563
2 : 133174635 cg07785936
2 : 133175192 cg13954965
2 : 133175311 cg19309079
2 : 133175382 cg14543676
2 : 133175397 cg18223770
2 : 133176249 cg02007217
2 : 133178190 cg20752284
2 : 133197577 cg24964130
2 : 133339746 cg20814312
2 : 133342286 cg09252301
2 : 133342838 cg02854228
2 : 133343115 cg19420177
2 : 133343149 cg08104981
2 : 133343274 cg05062023
2 : 133343308 cg02552292
2 : 133343373 cg09353251
2 : 133343618 cg17172683
2 : 133343628 cg11552903
2 : 133343657 cg18444763
2 : 133346858 cg26875877
2 : 133363326 cg09648243
2 : 133378888 cg15660353
2 : 133395195 cg10537176
2 : 133402475 cg06543615
2 : 133402827 cg07916022
2 : 133402940 cg12311815
2 : 133402999 cg15833099
2 : 133403009 cg27048432
2 : 133403841 cg10013531
GeneLoc
PromoterAssoc
CpGIsland
0.25
0.01
mRNA / METH Corr. FDR
MITF
SOX10
TRPM1
ZEB1
AXL
0.05
0.01
Gene / Regulator Corr. P
Type
mRNA_Expr
CopyNum
AmpDel

SERPINE1

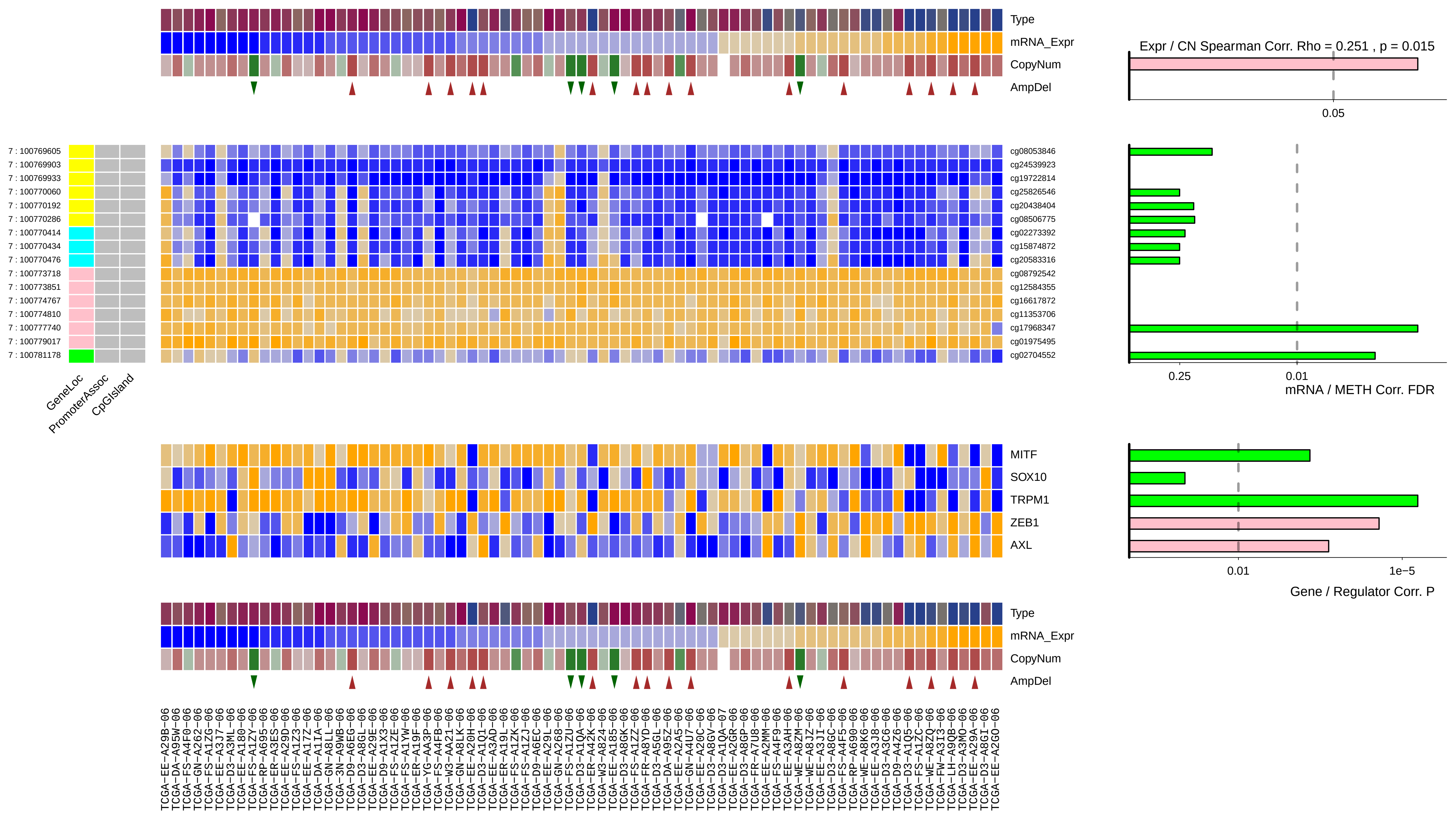
Type
mRNA_Expr
CopyNum
AmpDel
Expr / CN Spearman Corr. Rho = 0.251 , p = 0.015
0.05
7 : 100769605
7 : 100769903
7 : 100769933
7 : 100770060
7 : 100770192
7 : 100770286
7 : 100770414
7 : 100770434
7 : 100770476
7 : 100773718
7 : 100773851
7 : 100774767
7 : 100774810
7 : 100777740
7 : 100779017
7 : 100781178
GeneLoc
PromoterAssoc
CpGIsland
cg08053846
cg24539923
cg19722814
cg25826546
cg20438404
cg08506775
cg02273392
cg15874872
cg20583316
cg08792542
cg12584355
cg16617872
cg11353706
cg17968347
cg01975495
cg02704552
0.25
0.01
mRNA / METH Corr. FDR
MITF
SOX10
TRPM1
ZEB1
AXL
0.01
1e−5
Gene / Regulator Corr. P
Type
mRNA_Expr
CopyNum
AmpDel

LTBP1

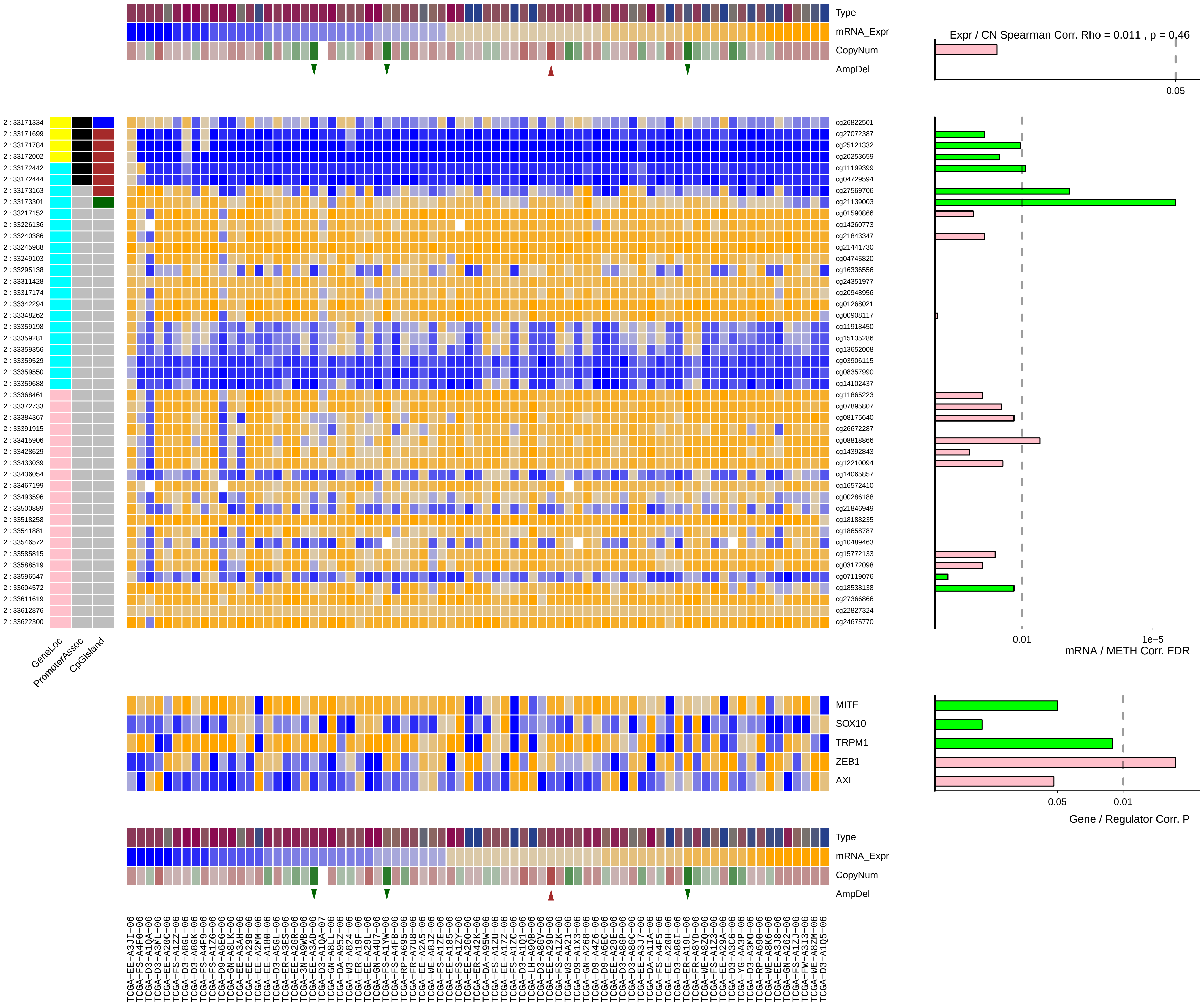
Type
mRNA_Expr
CopyNum
AmpDel
Expr / CN Spearman Corr. Rho = 0.011 , p = 0.46
0.05
GeneLoc
PromoterAssoc
CpGIsland
0.01
1e−5
mRNA / METH Corr. FDR
MITF
SOX10
TRPM1
ZEB1
AXL
0.05
0.01
Gene / Regulator Corr. P
Type
mRNA_Expr
CopyNum
AmpDel

TPBG

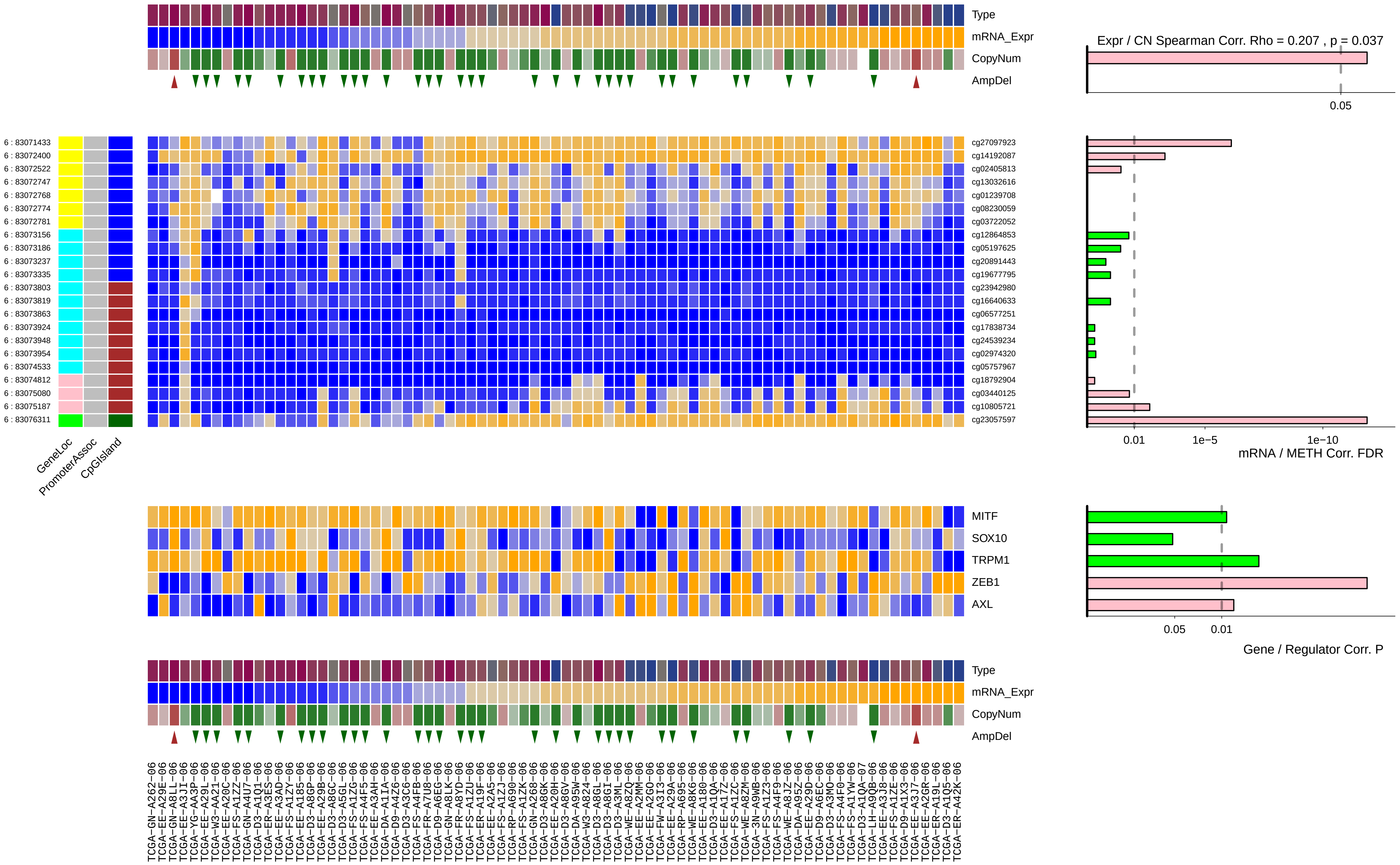

Type
mRNA_Expr
CopyNum
AmpDel
Expr / CN Spearman Corr. Rho = 0.207 , p = 0.037
0.05
6 : 83071433
6 : 83072400
6 : 83072522
6 : 83072747
6 : 83072768
6 : 83072774
6 : 83072781
6 : 83073156
6 : 83073186
6 : 83073237
6 : 83073335
6 : 83073803
6 : 83073819
6 : 83073863
6 : 83073924
6 : 83073948
6 : 83073954
6 : 83074533
6 : 83074812
6 : 83075080
6 : 83075187
6 : 83076311
cg27097923
cg14192087
cg02405813
cg13032616
cg01239708
cg08230059
cg03722052
cg12864853
cg05197625
cg20891443
cg19677795
cg23942980
cg16640633
cg06577251
cg17838734
cg24539234
cg02974320
cg05757967
cg18792904
cg03440125
cg10805721
cg23057597
GeneLoc
PromoterAssoc
CpGIsland
0.01
1e−5
1e−10
mRNA / METH Corr. FDR
MITF
SOX10
TRPM1
ZEB1
AXL
0.05
0.01
Gene / Regulator Corr. P
Type
mRNA_Expr
CopyNum
AmpDel

CDH13

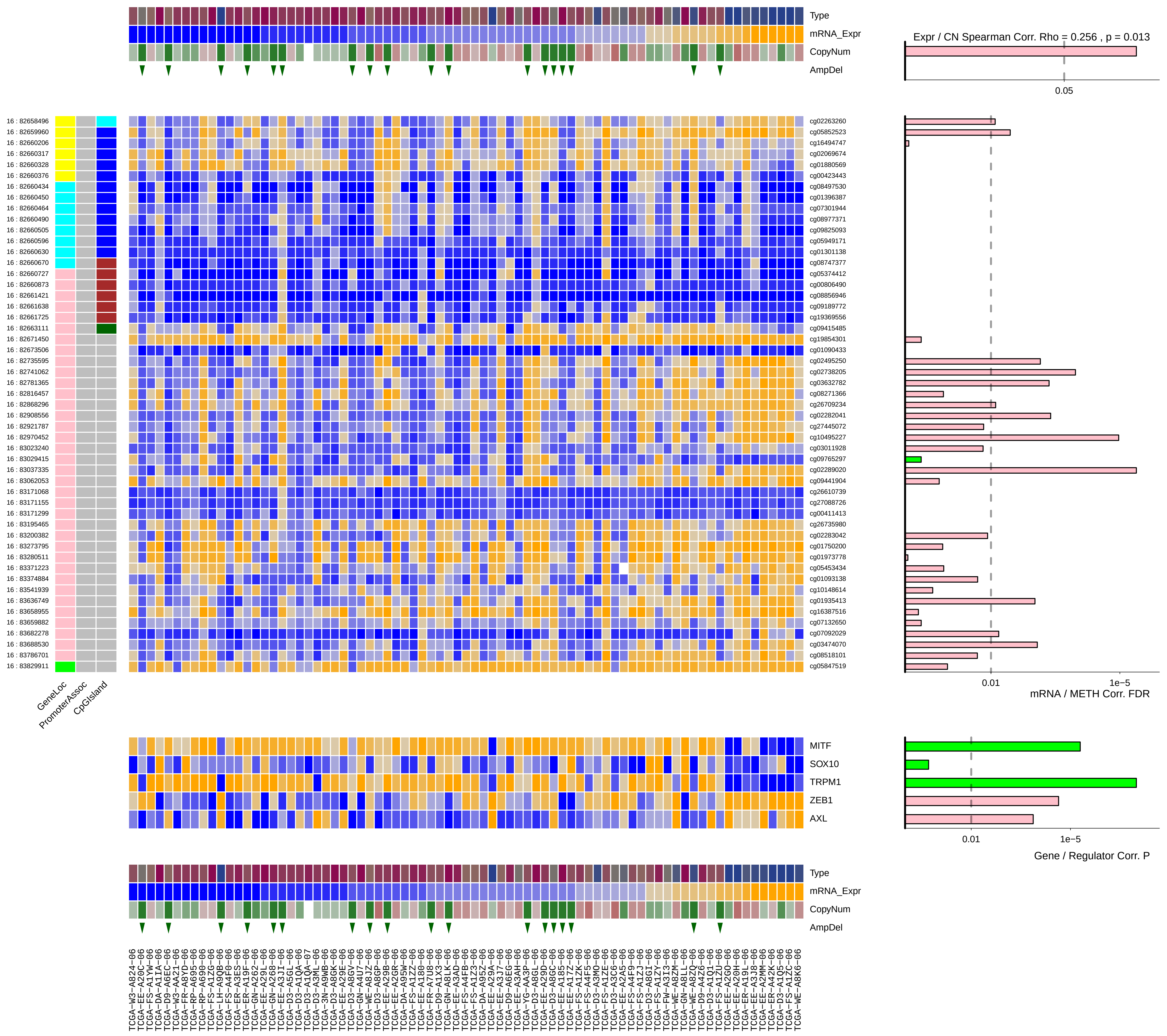

Type
mRNA_Expr
CopyNum
AmpDel
Expr / CN Spearman Corr. Rho = 0.256 , p = 0.013
0.05
GeneLoc
PromoterAssoc
CpGIsland
0.01
1e−5
mRNA / METH Corr. FDR
MITF
SOX10
TRPM1
ZEB1
AXL
0.01
1e−5
Gene / Regulator Corr. P

RAB27B

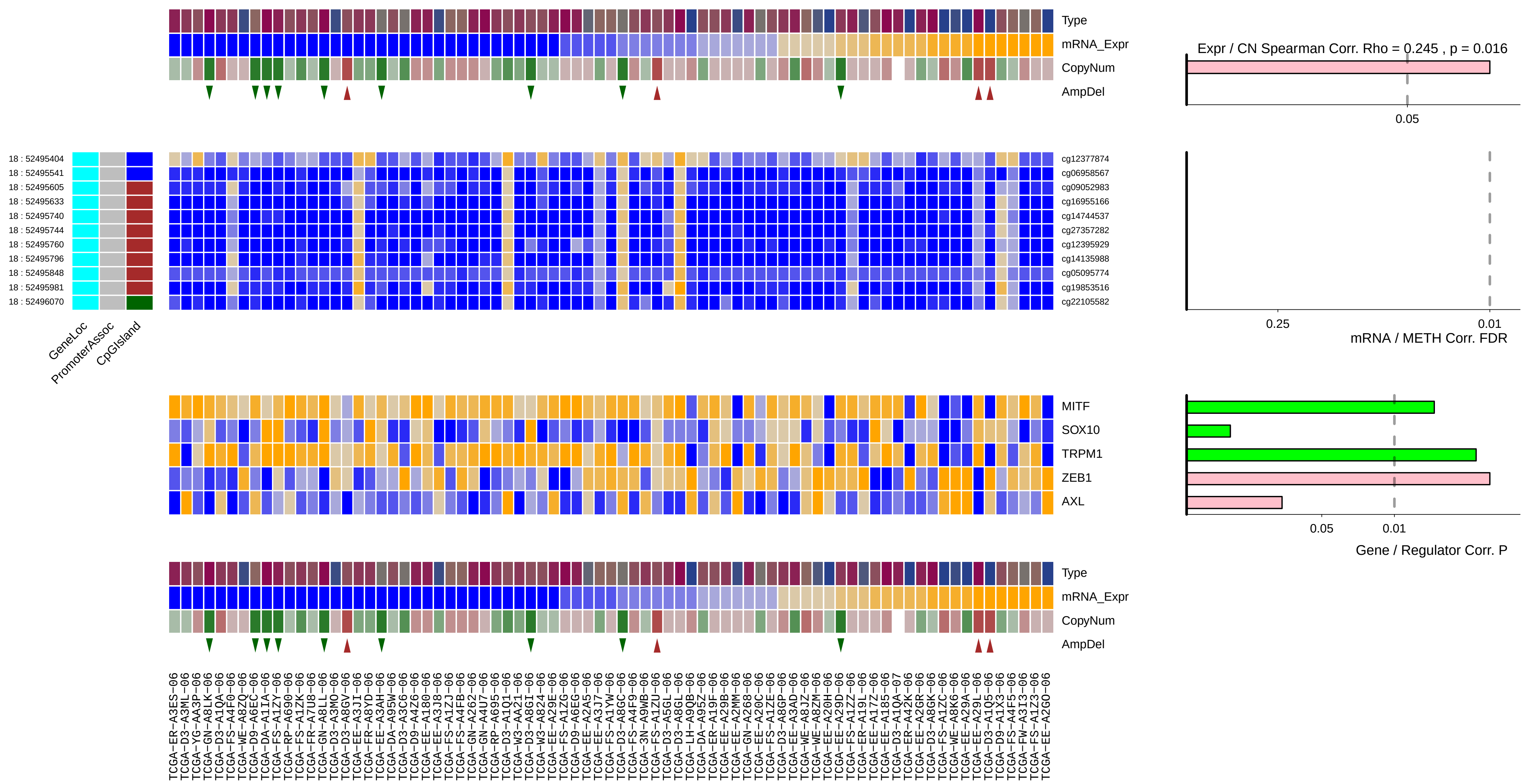

Type
mRNA_Expr
CopyNum
AmpDel
Expr / CN Spearman Corr. Rho = 0.245 , p = 0.016
0.05
18 : 52495404
18 : 52495541
18 : 52495605
18 : 52495633
18 : 52495740
18 : 52495744
18 : 52495760
18 : 52495796
18 : 52495848
18 : 52495981
18 : 52496070
cg12377874
cg06958567
cg09052983
cg16955166
cg14744537
cg27357282
cg12395929
cg14135988
cg05095774
cg19853516
cg22105582
0.25
0.01
mRNA / METH Corr. FDR
GeneLoc
PromoterAssoc
CpGIsland
MITF
SOX10
TRPM1
ZEB1
AXL
0.05
0.01
Gene / Regulator Corr. P
Type
mRNA_Expr
CopyNum
AmpDel

VEGFC

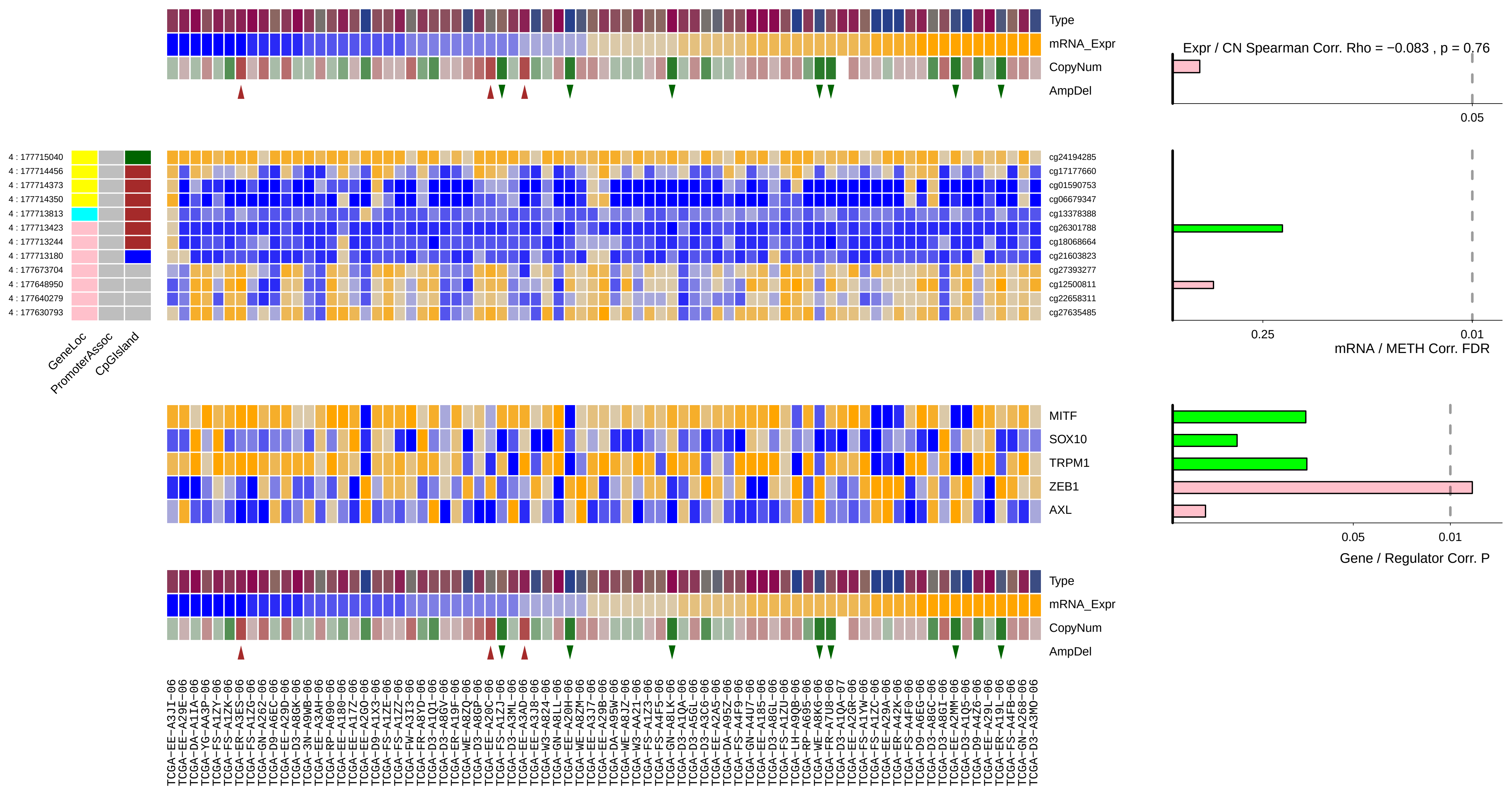
Type
mRNA_Expr
CopyNum
AmpDel
Expr / CN Spearman Corr. Rho = −0.083 , p = 0.76
0.05
mRNA / METH Corr. FDR
0.25
0.01
GeneLoc
PromoterAssoc
CpGIsland
MITF
SOX10
TRPM1
ZEB1
AXL
Gene / Regulator Corr. P

DPYD

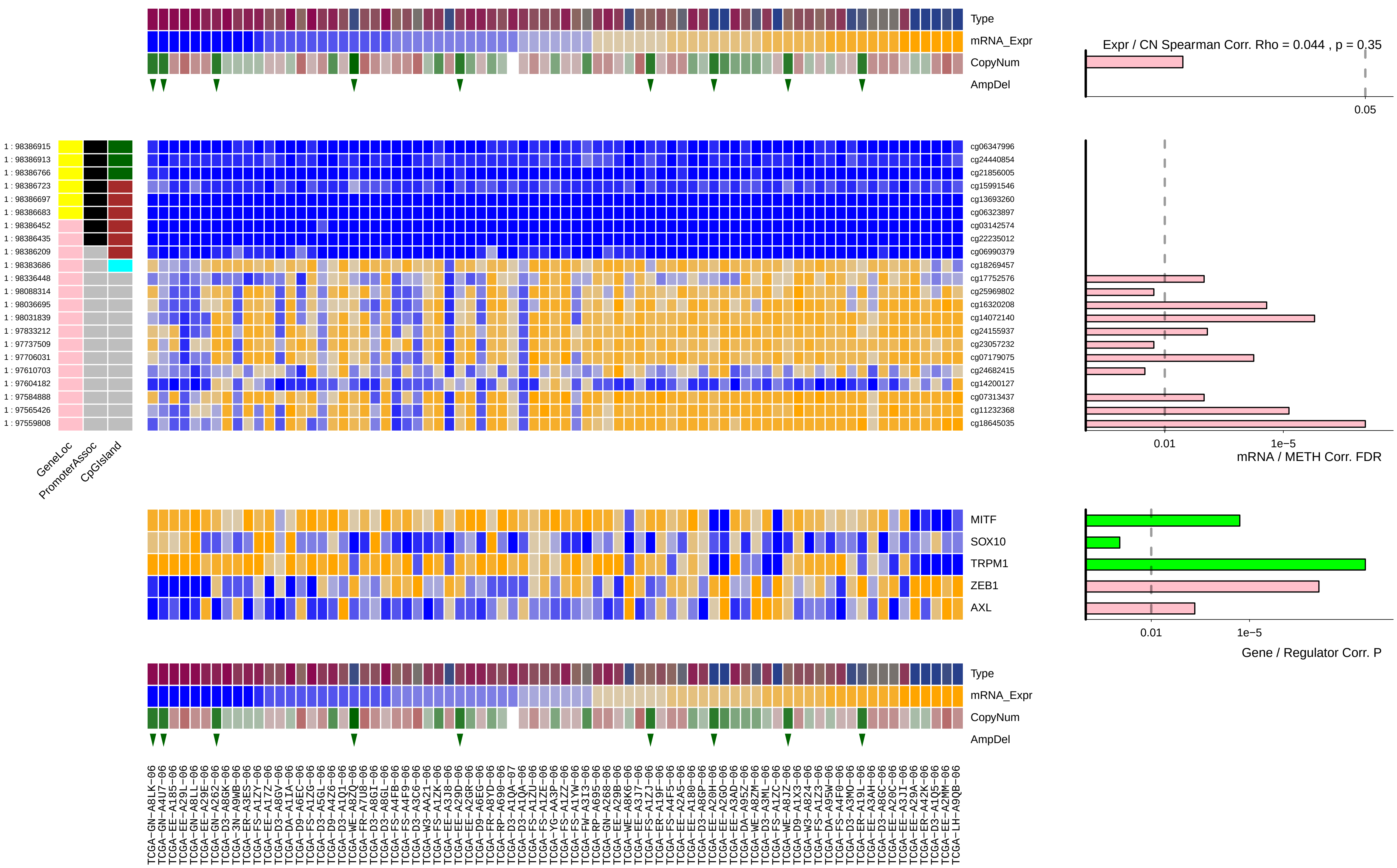
Type
mRNA_Expr
CopyNum
AmpDel
Expr / CN Spearman Corr. Rho = 0.044 , p = 0.35
0.05
1 : 98386915
1 : 98386913
1 : 98386766
1 : 98386723
1 : 98386697
1 : 98386683
1 : 98386452
1 : 98386435
1 : 98386209
1 : 98383686
1 : 98336448
1 : 98088314
1 : 98036695
1 : 98031839
1 : 97833212
1 : 97737509
1 : 97706031
1 : 97610703
1 : 97604182
1 : 97584888
1 : 97565426
1 : 97559808
cg06347996
cg24440854
cg21856005
cg15991546
cg13693260
cg06323897
cg03142574
cg22235012
cg06990379
cg18269457
cg17752576
cg25969802
cg16320208
cg14072140
cg24155937
cg23057232
cg07179075
cg24682415
cg14200127
cg07313437
cg11232368
cg18645035
GeneLoc
PromoterAssoc
CpGIsland
0.01
1e−5
mRNA / METH Corr. FDR
MITF
SOX10
TRPM1
ZEB1
AXL
0.01
1e−5
Gene / Regulator Corr. P
Type
mRNA_Expr
CopyNum
AmpDel

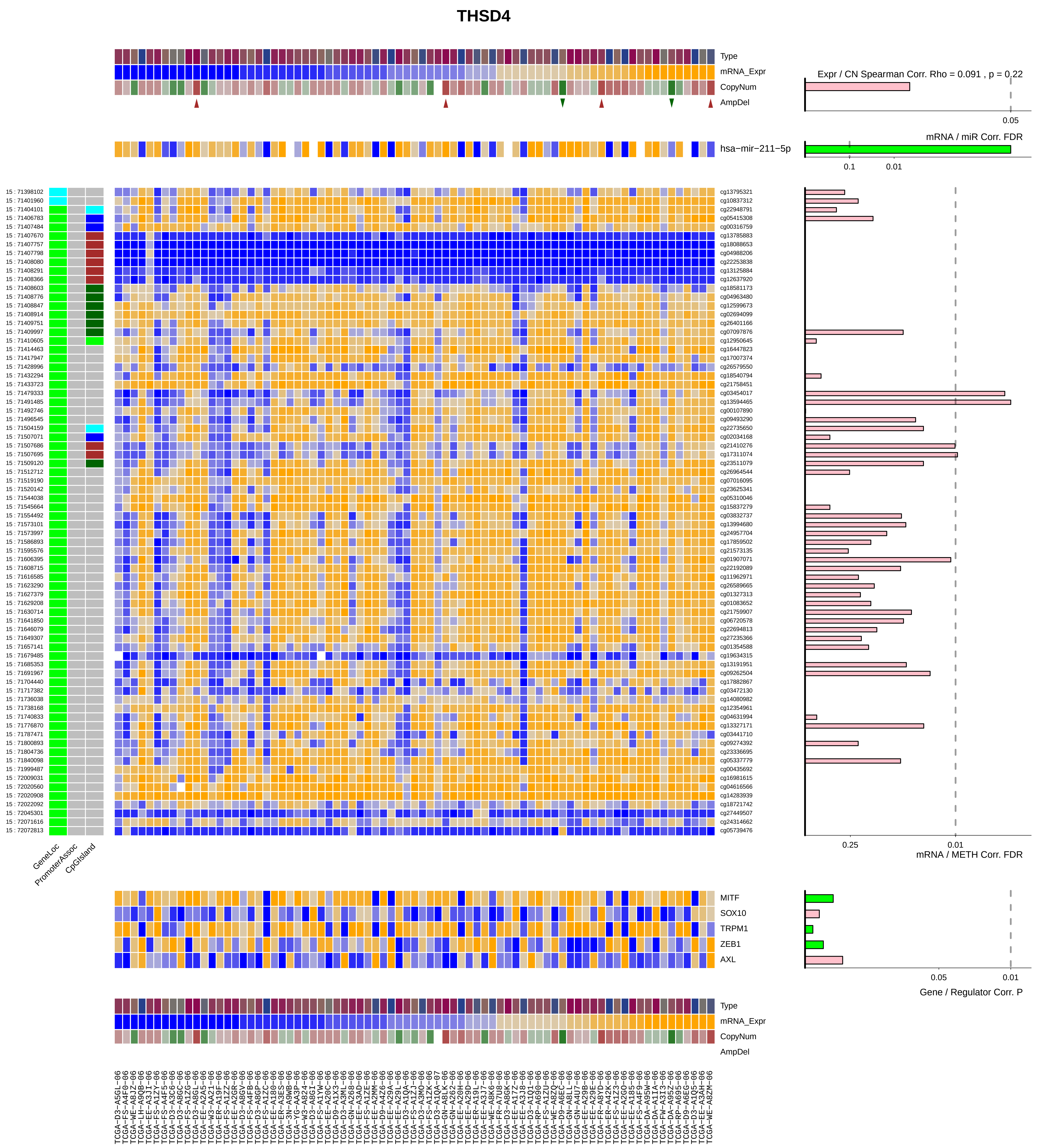
THSD4
Type
mRNA_Expr
CopyNum
AmpDel
Expr / CN Spearman Corr. Rho = 0.091 , p = 0.22
0.05
mRNA / miR Corr. FDR
hsa−mir−211−5p
0.1
0.01
0.25
0.01
mRNA / METH Corr. FDR
GeneLoc
PromoterAssoc
CpGIsland
MITF
SOX10
TRPM1
ZEB1
AXL
0.05
0.01
Gene / Regulator Corr. P

COL6A2

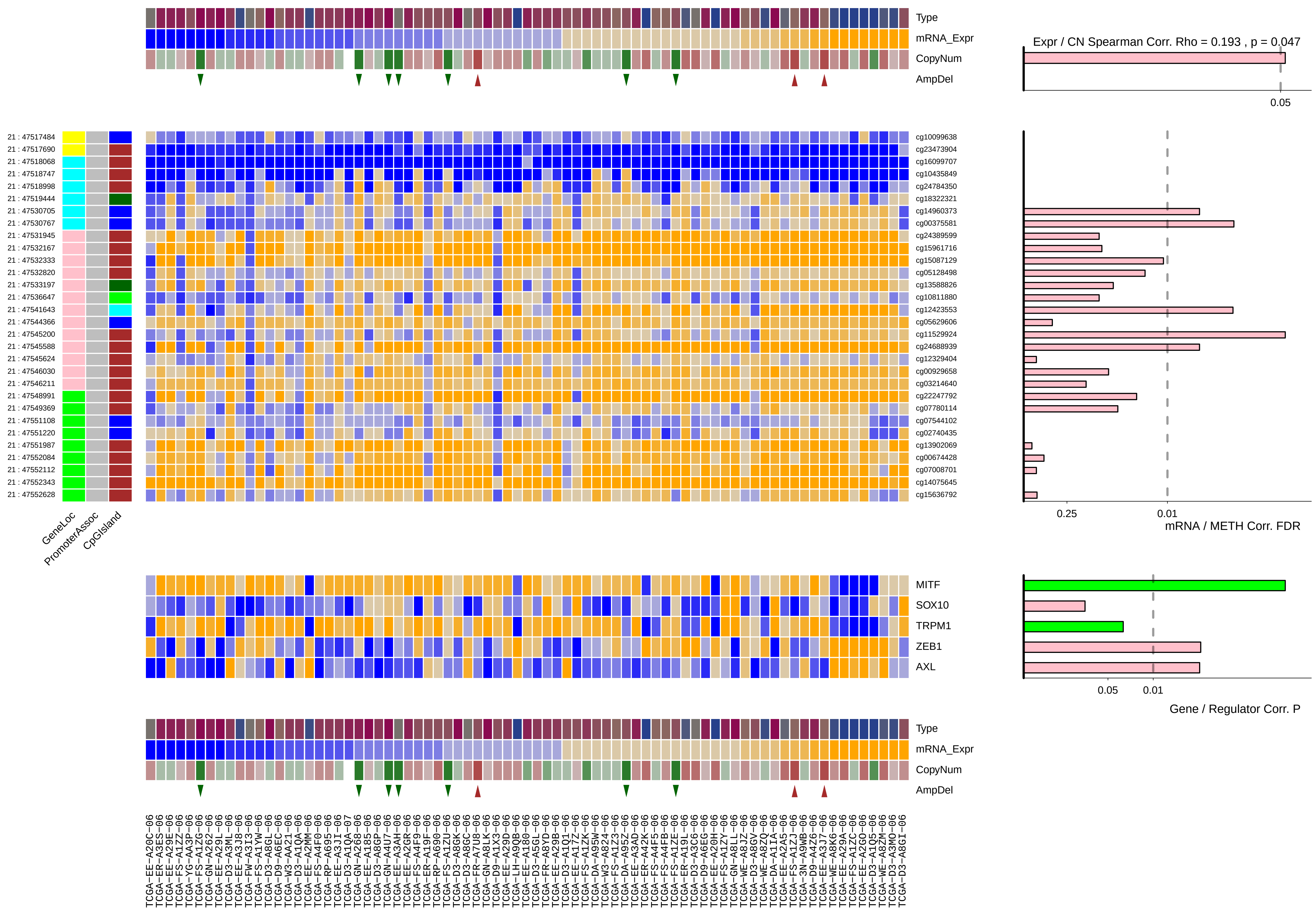

Type
mRNA_Expr
CopyNum
AmpDel
Expr / CN Spearman Corr. Rho = 0.193 , p = 0.047
0.05
21 : 47517484
21 : 47517690
21 : 47518068
21 : 47518747
21 : 47518998
21 : 47519444
21 : 47530705
21 : 47530767
21 : 47531945
21 : 47532167
21 : 47532333
21 : 47532820
21 : 47533197
21 : 47536647
21 : 47541643
21 : 47544366
21 : 47545200
21 : 47545588
21 : 47545624
21 : 47546030
21 : 47546211
21 : 47548991
21 : 47549369
21 : 47551108
21 : 47551220
21 : 47551987
21 : 47552084
21 : 47552112
21 : 47552343
21 : 47552628
GeneLoc
PromoterAssoc
CpGIsland
cg10099638
cg23473904
cg16099707
cg10435849
cg24784350
cg18322321
cg14960373
cg00375581
cg24389599
cg15961716
cg15087129
cg05128498
cg13588826
cg10811880
cg12423553
cg05629606
cg11529924
cg24688939
cg12329404
cg00929658
cg03214640
cg22247792
cg07780114
cg07544102
cg02740435
cg13902069
cg00674428
cg07008701
cg14075645
cg15636792
0.25
0.01
mRNA / METH Corr. FDR
MITF
SOX10
TRPM1
ZEB1
AXL
0.05
0.01
Gene / Regulator Corr. P
Type
mRNA_Expr
CopyNum
AmpDel

DKK3

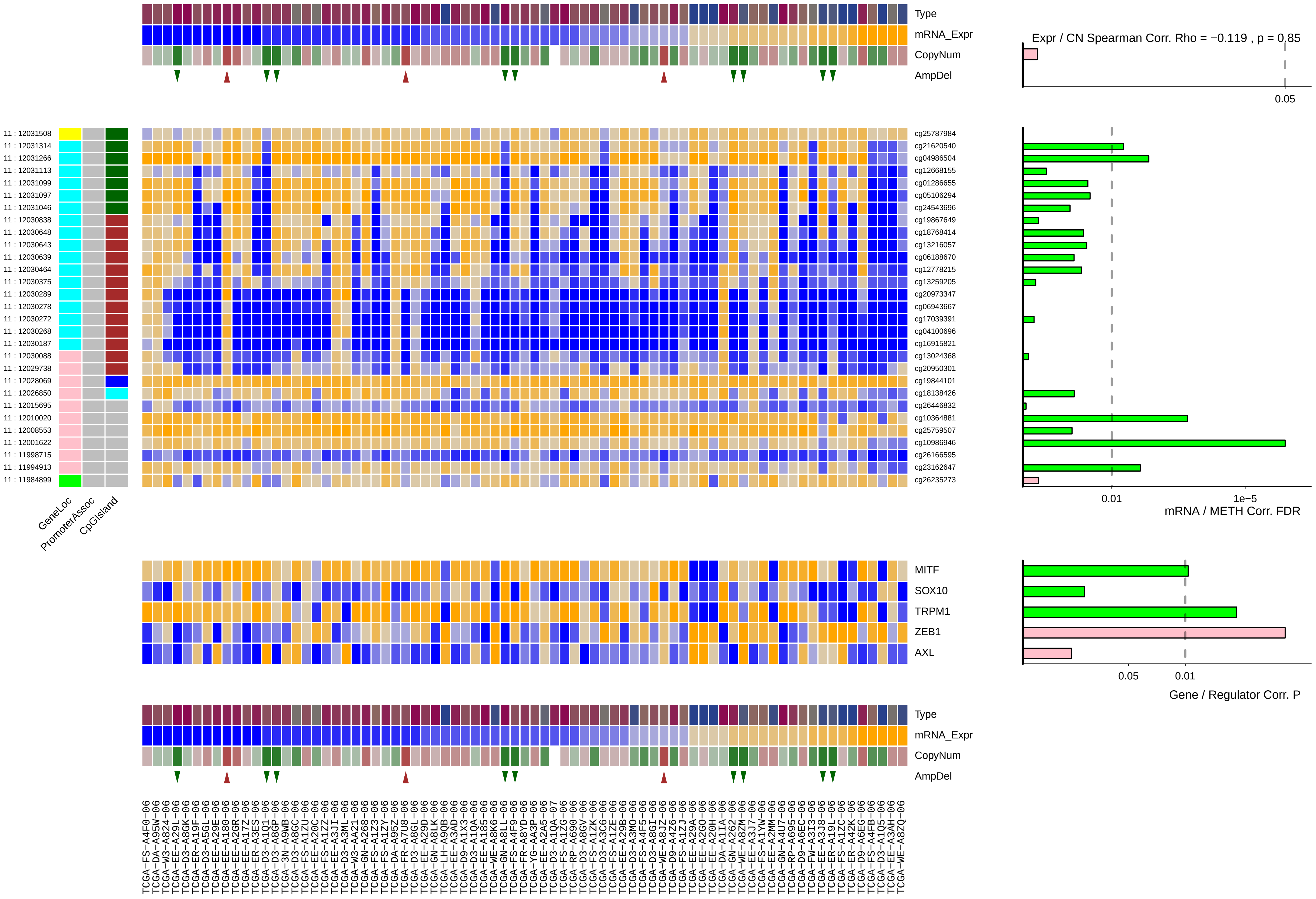
Type
mRNA_Expr
CopyNum
AmpDel
Expr / CN Spearman Corr. Rho = −0.119 , p = 0.85
0.05
mRNA / METH Corr. FDR
0.01
1e−5
GeneLoc
PromoterAssoc
CpGIsland
MITF
SOX10
TRPM1
ZEB1
AXL
Gene / Regulator Corr. P
0.05
0.01

SOX9

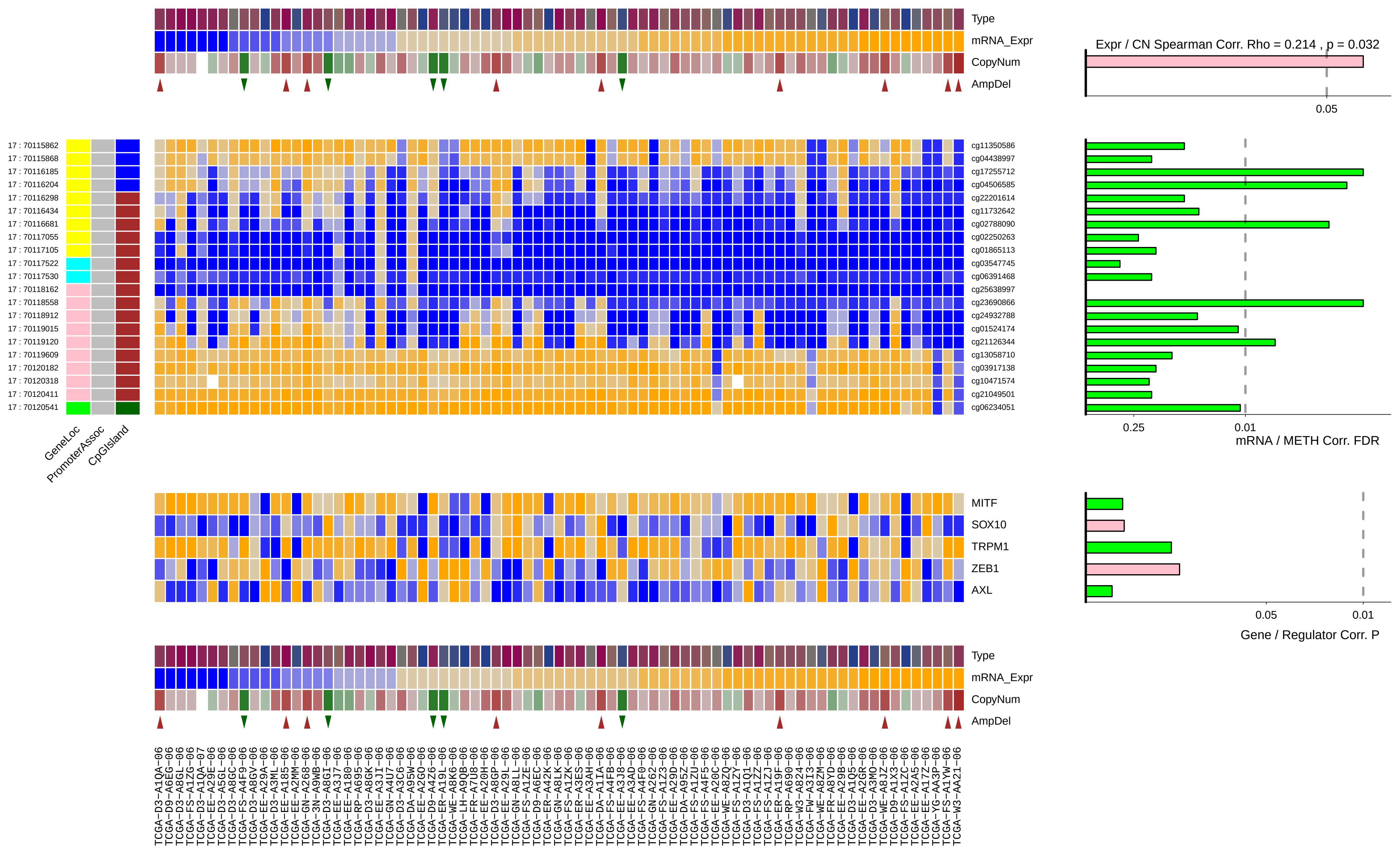
Type
mRNA_Expr
CopyNum
AmpDel
Expr / CN Spearman Corr. Rho = 0.214 , p = 0.032
0.05
mRNA / METH Corr. FDR
Gene / Regulator Corr. P
MITF
SOX10
TRPM1
ZEB1
AXL
GeneLoc
PromoterAssoc
CpGIsland

PDGFC

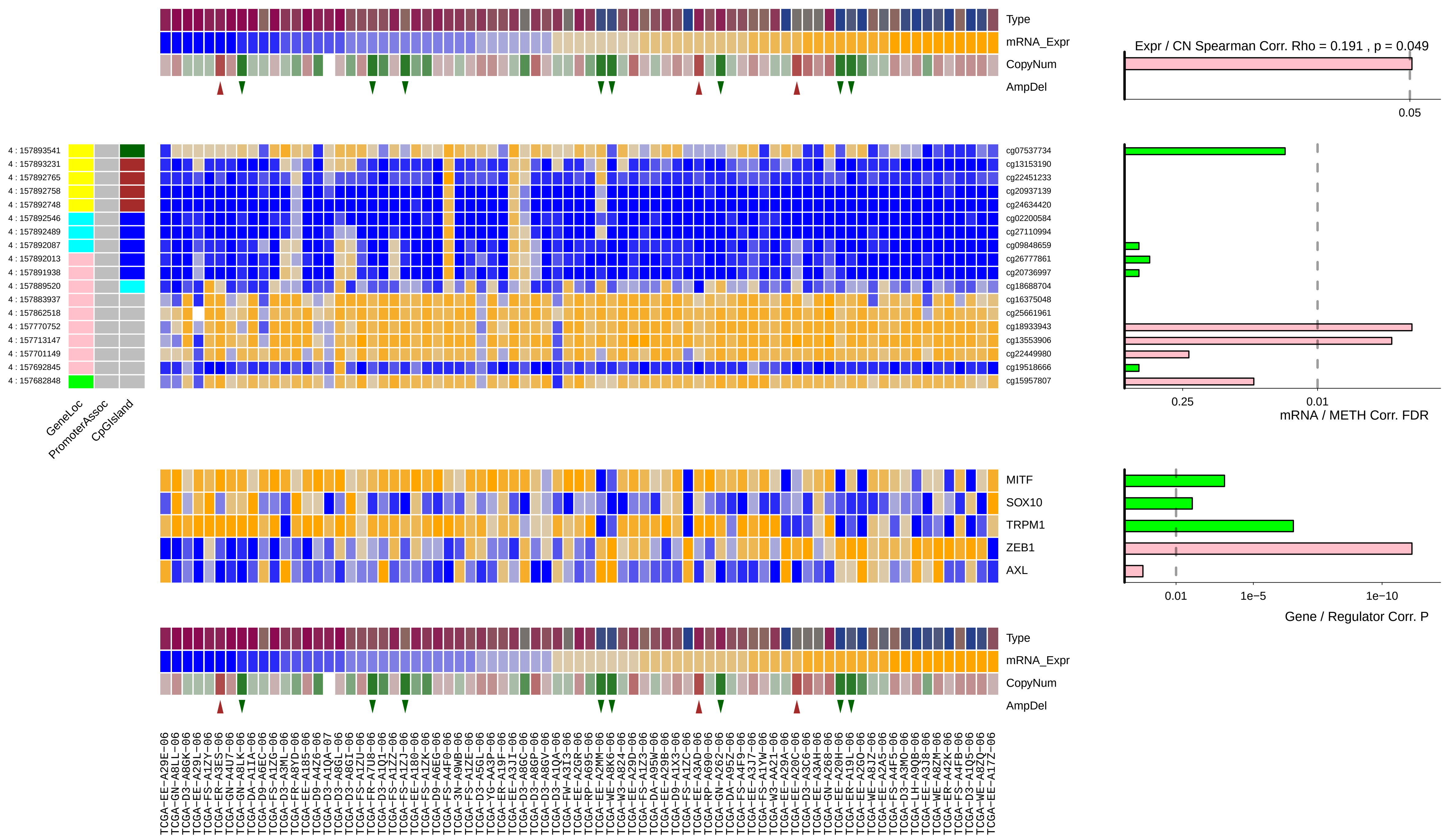

Type
mRNA_Expr
CopyNum
AmpDel
Expr / CN Spearman Corr. Rho = 0.191 , p = 0.049
0.05
mRNA / METH Corr. FDR
0.25
0.01
MITF
SOX10
TRPM1
ZEB1
AXL
Gene / Regulator Corr. P
1e−5
1e−10
GeneLoc
PromoterAssoc
CpGIsland

HECW2

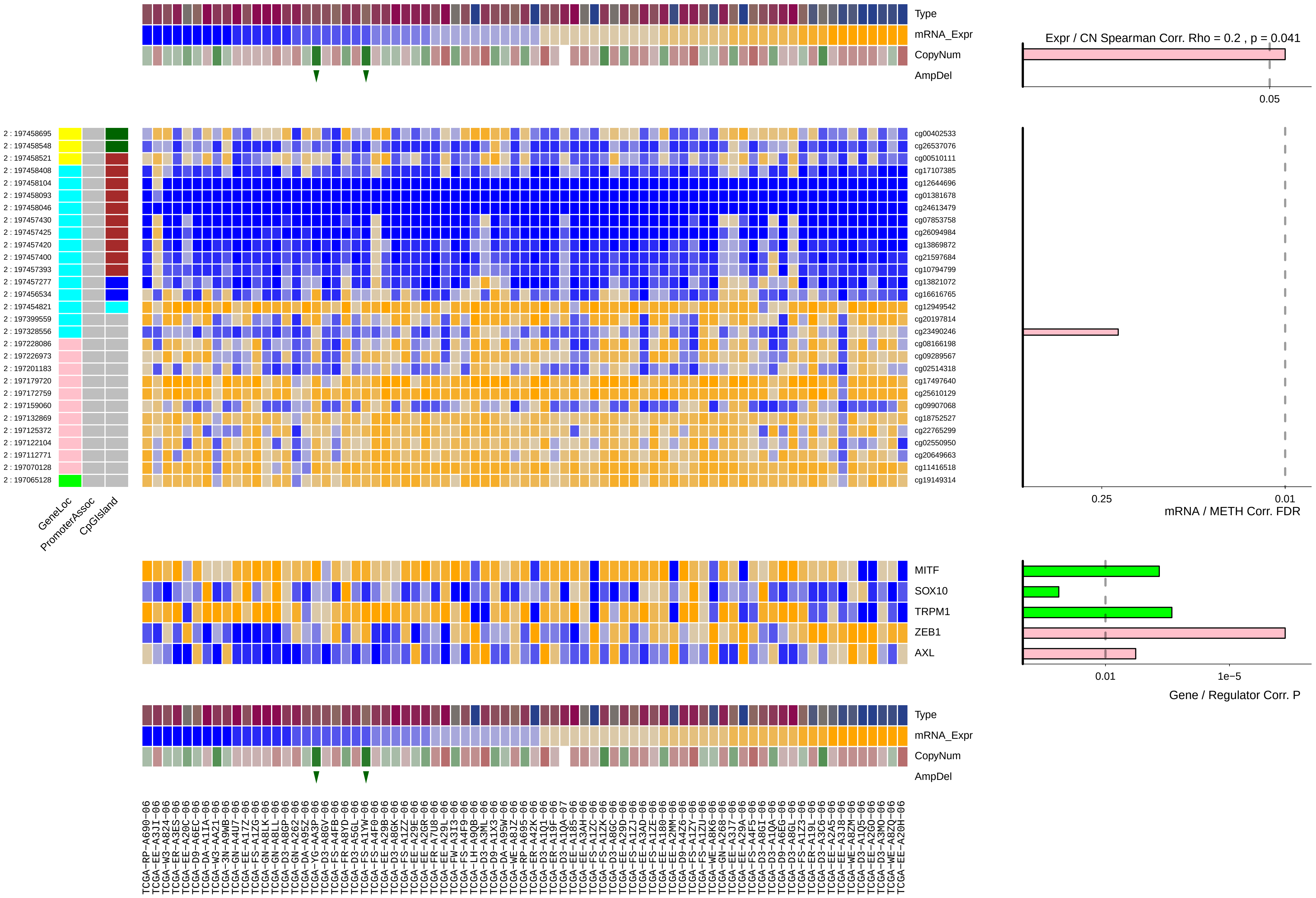

Type
mRNA_Expr
CopyNum
AmpDel
Expr / CN Spearman Corr. Rho = 0.2 , p = 0.041
0.05
2 : 197458695
2 : 197458548
2 : 197458521
2 : 197458408
2 : 197458104
2 : 197458093
2 : 197458046
2 : 197457430
2 : 197457425
2 : 197457420
2 : 197457400
2 : 197457393
2 : 197457277
2 : 197456534
2 : 197454821
2 : 197399559
2 : 197328556
2 : 197228086
2 : 197226973
2 : 197201183
2 : 197179720
2 : 197172759
2 : 197159060
2 : 197132869
2 : 197125372
2 : 197122104
2 : 197112771
2 : 197070128
2 : 197065128
cg00402533
cg26537076
cg00510111
cg17107385
cg12644696
cg01381678
cg24613479
cg07853758
cg26094984
cg13869872
cg21597684
cg10794799
cg13821072
cg16616765
cg12949542
cg20197814
cg23490246
cg08166198
cg09289567
cg02514318
cg17497640
cg25610129
cg09907068
cg18752527
cg22765299
cg02550950
cg20649663
cg11416518
cg19149314
GeneLoc
PromoterAssoc
CpGIsland
0.25
0.01
mRNA / METH Corr. FDR
MITF
SOX10
TRPM1
ZEB1
AXL
0.01
1e−5
Gene / Regulator Corr. P
Type
mRNA_Expr
CopyNum
AmpDel

FZD2

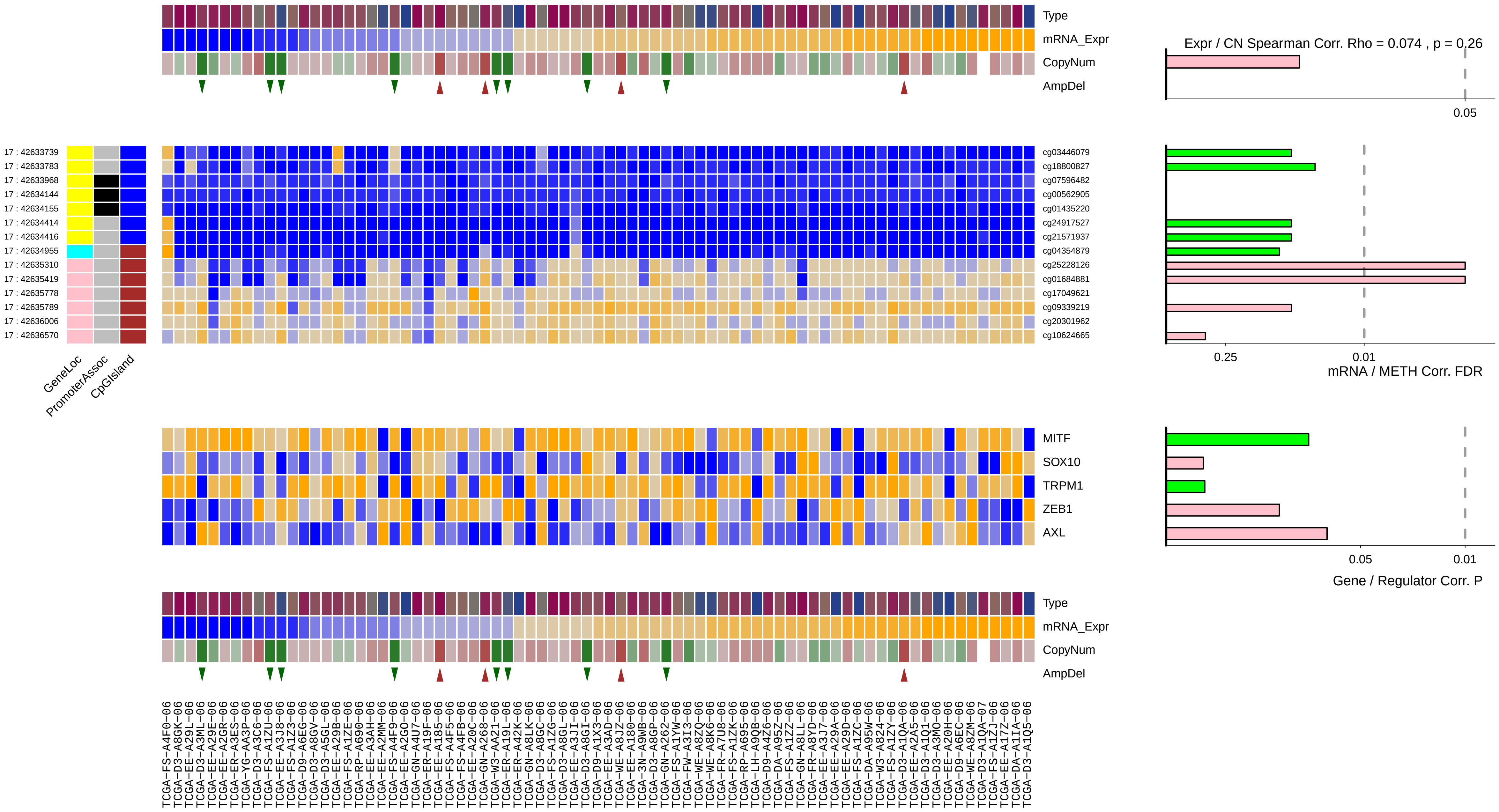

Type
mRNA_Expr
CopyNum
AmpDel
Expr / CN Spearman Corr. Rho = 0.074 , p = 0.26
0.05
17 : 42633739
17 : 42633783
17 : 42633968
17 : 42634144
17 : 42634155
17 : 42634414
17 : 42634416
17 : 42634955
17 : 42635310
17 : 42635419
17 : 42635778
17 : 42635789
17 : 42636006
17 : 42636570
cg03446079
cg18800827
cg07596482
cg00562905
cg01435220
cg24917527
cg21571937
cg04354879
cg25228126
cg01684881
cg17049621
cg09339219
cg20301962
cg10624665
0.25
0.01
mRNA / METH Corr. FDR
GeneLoc
PromoterAssoc
CpGIsland
MITF
SOX10
TRPM1
ZEB1
AXL
0.05
0.01
Gene / Regulator Corr. P
Type
mRNA_Expr
CopyNum
AmpDel

PAPPA

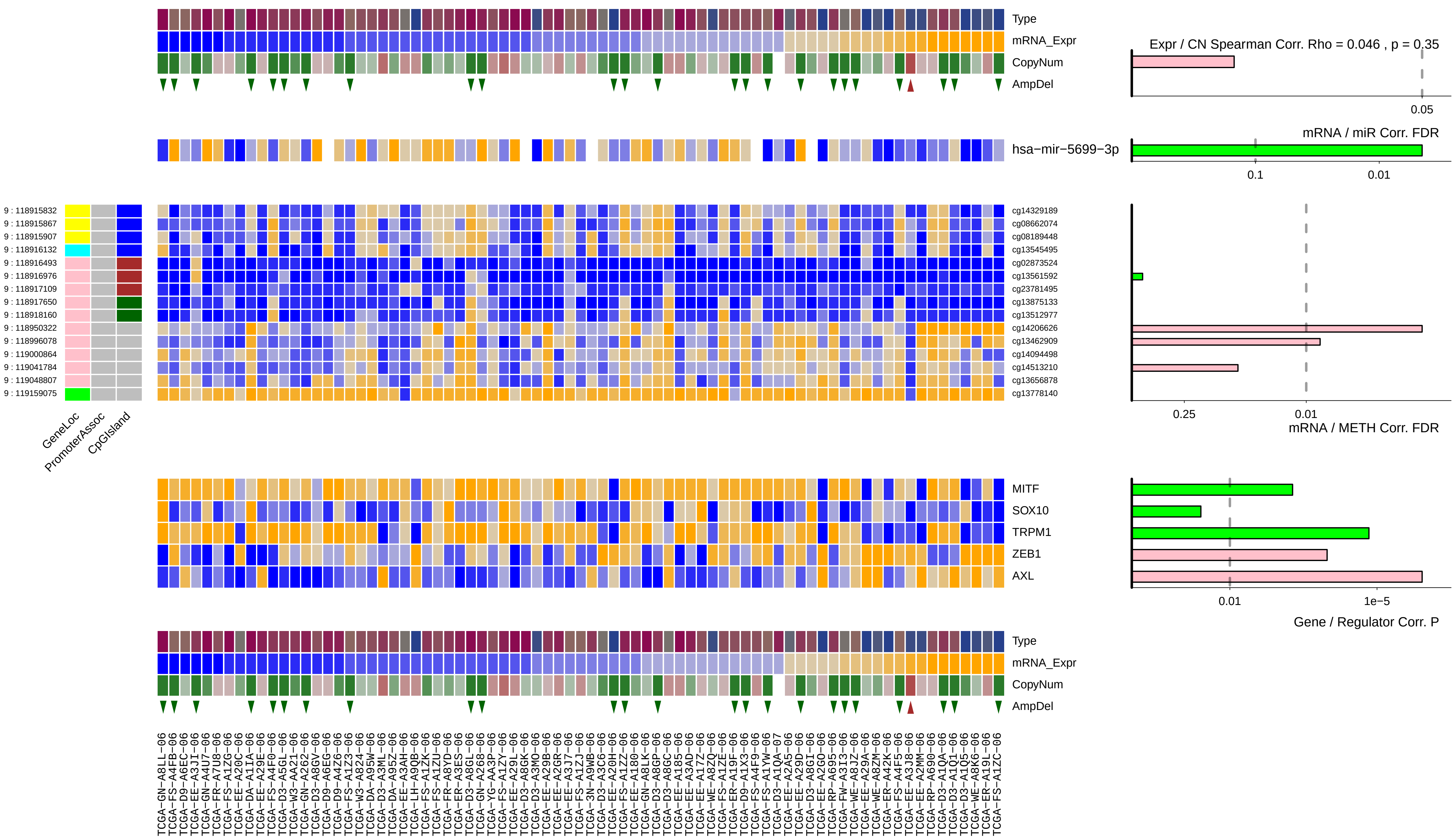
Type
mRNA_Expr
CopyNum
AmpDel
Expr / CN Spearman Corr. Rho = 0.046 , p = 0.35
0.05
hsa−mir−5699−3p
mRNA / miR Corr. FDR
0.1
0.01
9 : 118915832
9 : 118915867
9 : 118915907
9 : 118916132
9 : 118916493
9 : 118916976
9 : 118917109
9 : 118917650
9 : 118918160
9 : 118950322
9 : 118996078
9 : 119000864
9 : 119041784
9 : 119048807
9 : 119159075
cg14329189
cg08662074
cg08189448
cg13545495
cg02873524
cg13561592
cg23781495
cg13875133
cg13512977
cg14206626
cg13462909
cg14094498
cg14513210
cg13656878
cg13778140
GeneLoc
PromoterAssoc
CpGIsland
0.25
0.01
mRNA / METH Corr. FDR
MITF
SOX10
TRPM1
ZEB1
AXL
0.01
1e−5
Gene / Regulator Corr. P
Type
mRNA_Expr
CopyNum
AmpDel

WNT5A

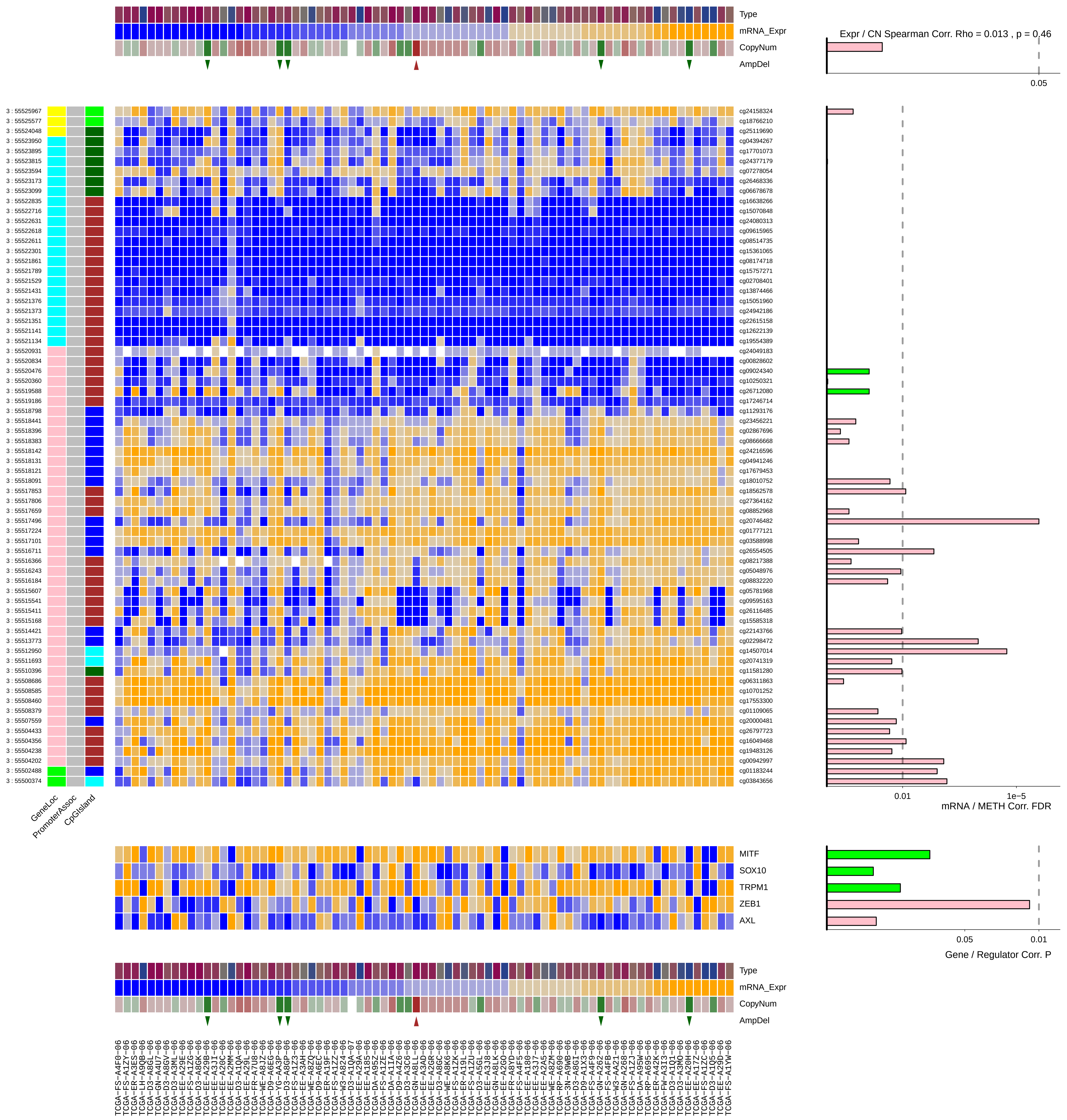
Type
mRNA_Expr
CopyNum
AmpDel
Expr / CN Spearman Corr. Rho = 0.013 , p = 0.46
0.05
0.01
1e−5
mRNA / METH Corr. FDR
GeneLoc
PromoterAssoc
CpGIsland
MITF
SOX10
TRPM1
ZEB1
AXL
0.05
0.01
Gene / Regulator Corr. P

TGM2

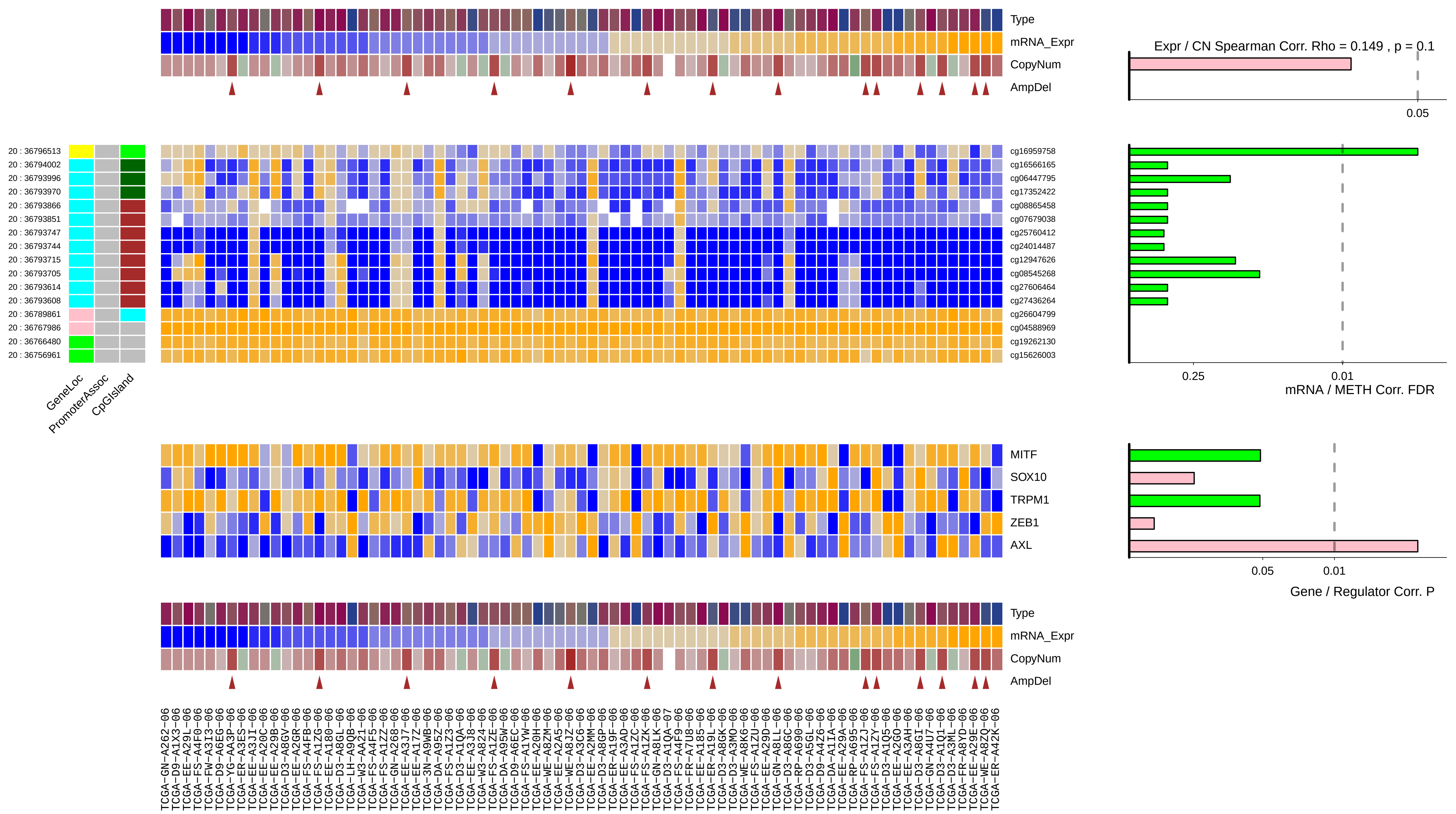

Type
mRNA_Expr
CopyNum
AmpDel
Expr / CN Spearman Corr. Rho = 0.149 , p = 0.1
0.05
20 : 36796513
20 : 36794002
20 : 36793996
20 : 36793970
20 : 36793866
20 : 36793851
20 : 36793747
20 : 36793744
20 : 36793715
20 : 36793705
20 : 36793614
20 : 36793608
20 : 36789861
20 : 36767986
20 : 36766480
20 : 36756961
GeneLoc
PromoterAssoc
CpGIsland
cg16959758
cg16566165
cg06447795
cg17352422
cg08865458
cg07679038
cg25760412
cg24014487
cg12947626
cg08545268
cg27606464
cg27436264
cg26604799
cg04588969
cg19262130
cg15626003
0.25
0.01
mRNA / METH Corr. FDR
MITF
SOX10
TRPM1
ZEB1
AXL
0.05
0.01
Gene / Regulator Corr. P
Type
mRNA_Expr
CopyNum
AmpDel

SQRDL

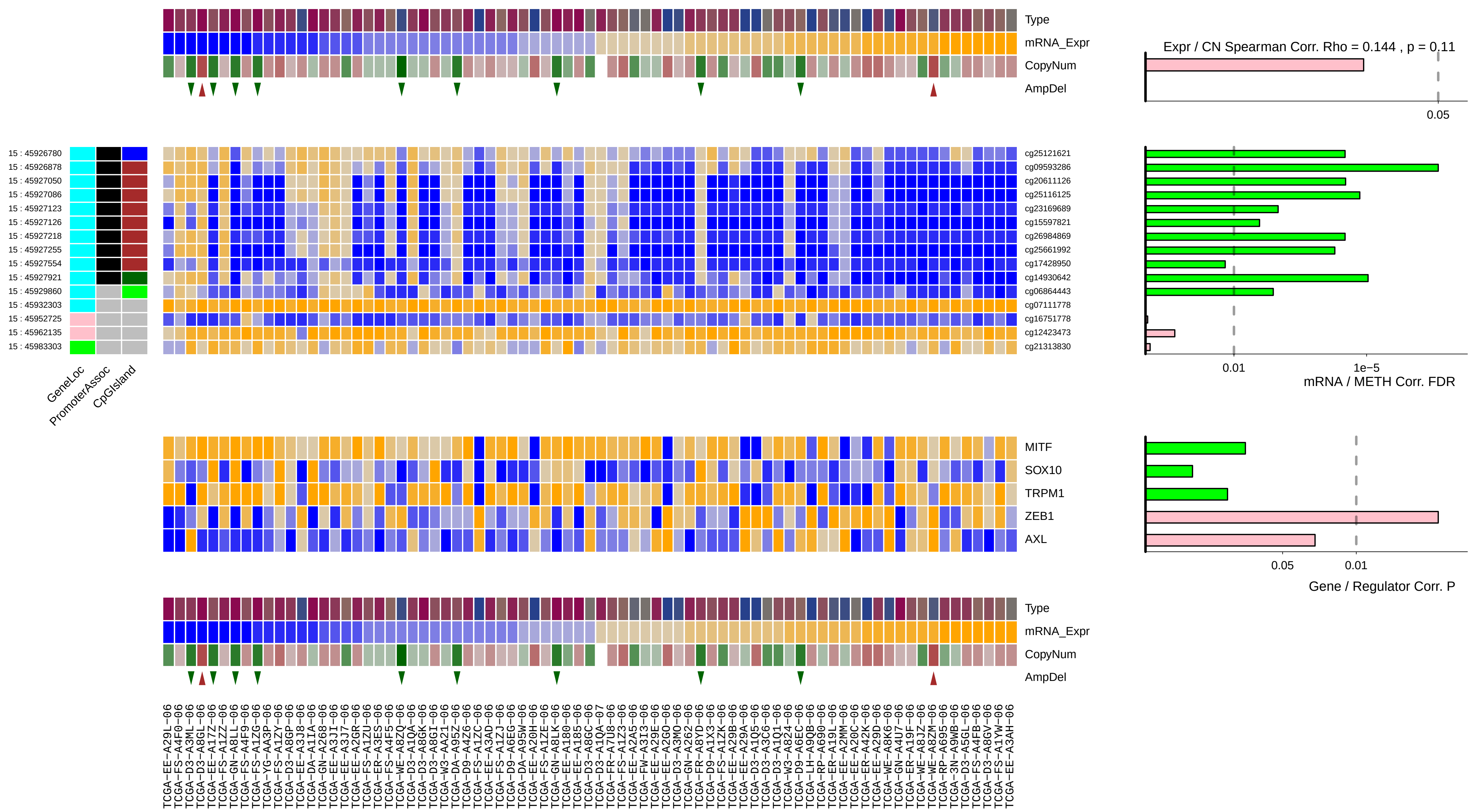
Type
mRNA_Expr
CopyNum
AmpDel
Expr / CN Spearman Corr. Rho = 0.144 , p = 0.11
0.05
GeneLoc
PromoterAssoc
CpGIsland
0.01
1e−5
mRNA / METH Corr. FDR
MITF
SOX10
TRPM1
ZEB1
AXL
0.05
0.01
Gene / Regulator Corr. P

IRF1

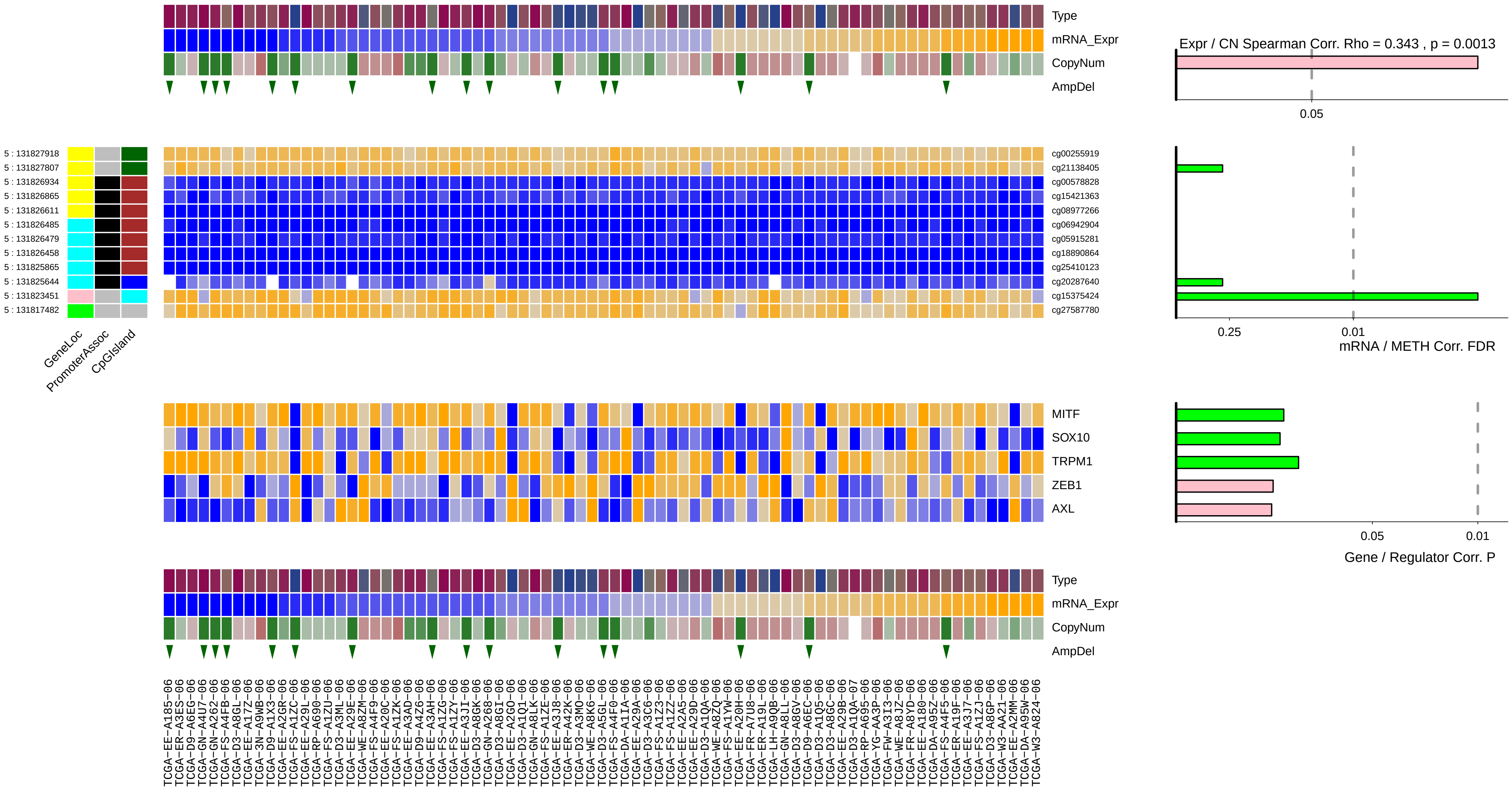

Type
mRNA_Expr
CopyNum
AmpDel
Expr / CN Spearman Corr. Rho = 0.343 , p = 0.0013
0.05
5 : 131827918
5 : 131827807
5 : 131826934
5 : 131826865
5 : 131826611
5 : 131826485
5 : 131826479
5 : 131826458
5 : 131825865
5 : 131825644
5 : 131823451
5 : 131817482
GeneLoc
PromoterAssoc
CpGIsland
cg00255919
cg21138405
cg00578828
cg15421363
cg08977266
cg06942904
cg05915281
cg18890864
cg25410123
cg20287640
cg15375424
cg27587780
0.25
0.01
mRNA / METH Corr. FDR
MITF
SOX10
TRPM1
ZEB1
AXL
0.05
0.01
Gene / Regulator Corr. P

SMURF2

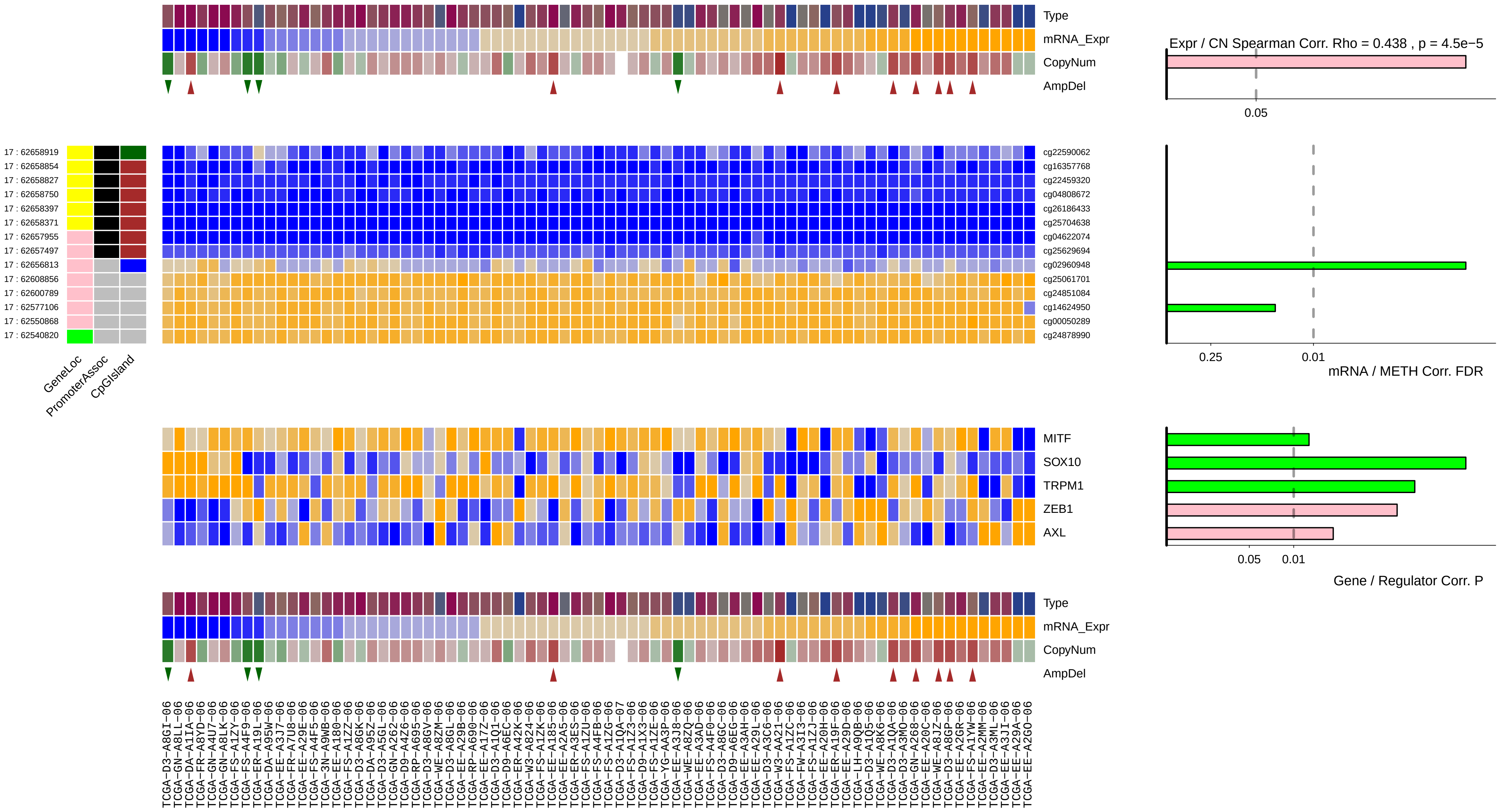
Type
mRNA_Expr
CopyNum
AmpDel
Expr / CN Spearman Corr. Rho = 0.438 , p = 4.5e−5
0.05
17 : 62658919
17 : 62658854
17 : 62658827
17 : 62658750
17 : 62658397
17 : 62658371
17 : 62657955
17 : 62657497
17 : 62656813
17 : 62608856
17 : 62600789
17 : 62577106
17 : 62550868
17 : 62540820
cg22590062
cg16357768
cg22459320
cg04808672
cg26186433
cg25704638
cg04622074
cg25629694
cg02960948
cg25061701
cg24851084
cg14624950
cg00050289
cg24878990
GeneLoc
PromoterAssoc
CpGIsland
0.25
0.01
mRNA / METH Corr. FDR
MITF
SOX10
TRPM1
ZEB1
AXL
0.05
0.01
Gene / Regulator Corr. P
Type
mRNA_Expr
CopyNum
AmpDel

IGFBP6

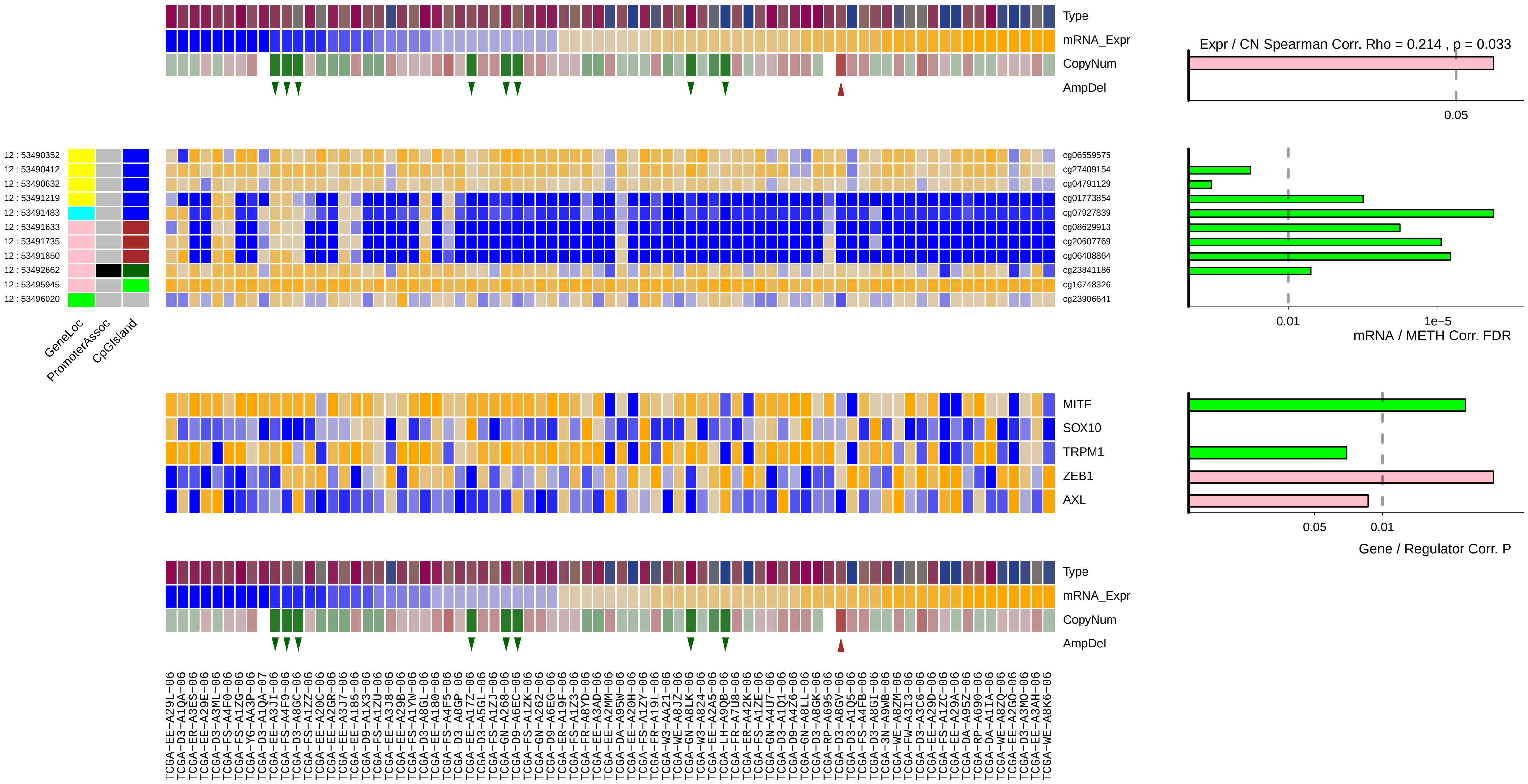

Type
mRNA_Expr
CopyNum
AmpDel
Expr / CN Spearman Corr. Rho = 0.214 , p = 0.033
0.05
12 : 53490352
12 : 53490412
12 : 53490632
12 : 53491219
12 : 53491483
12 : 53491633
12 : 53491735
12 : 53491850
12 : 53492662
12 : 53495945
12 : 53496020
cg06559575
cg27409154
cg04791129
cg01773854
cg07927839
cg08629913
cg20607769
cg06408864
cg23841186
cg16748326
cg23906641
0.01
1e−5
mRNA / METH Corr. FDR
GeneLoc
PromoterAssoc
CpGIsland
MITF
SOX10
TRPM1
ZEB1
AXL
0.05
0.01
Gene / Regulator Corr. P

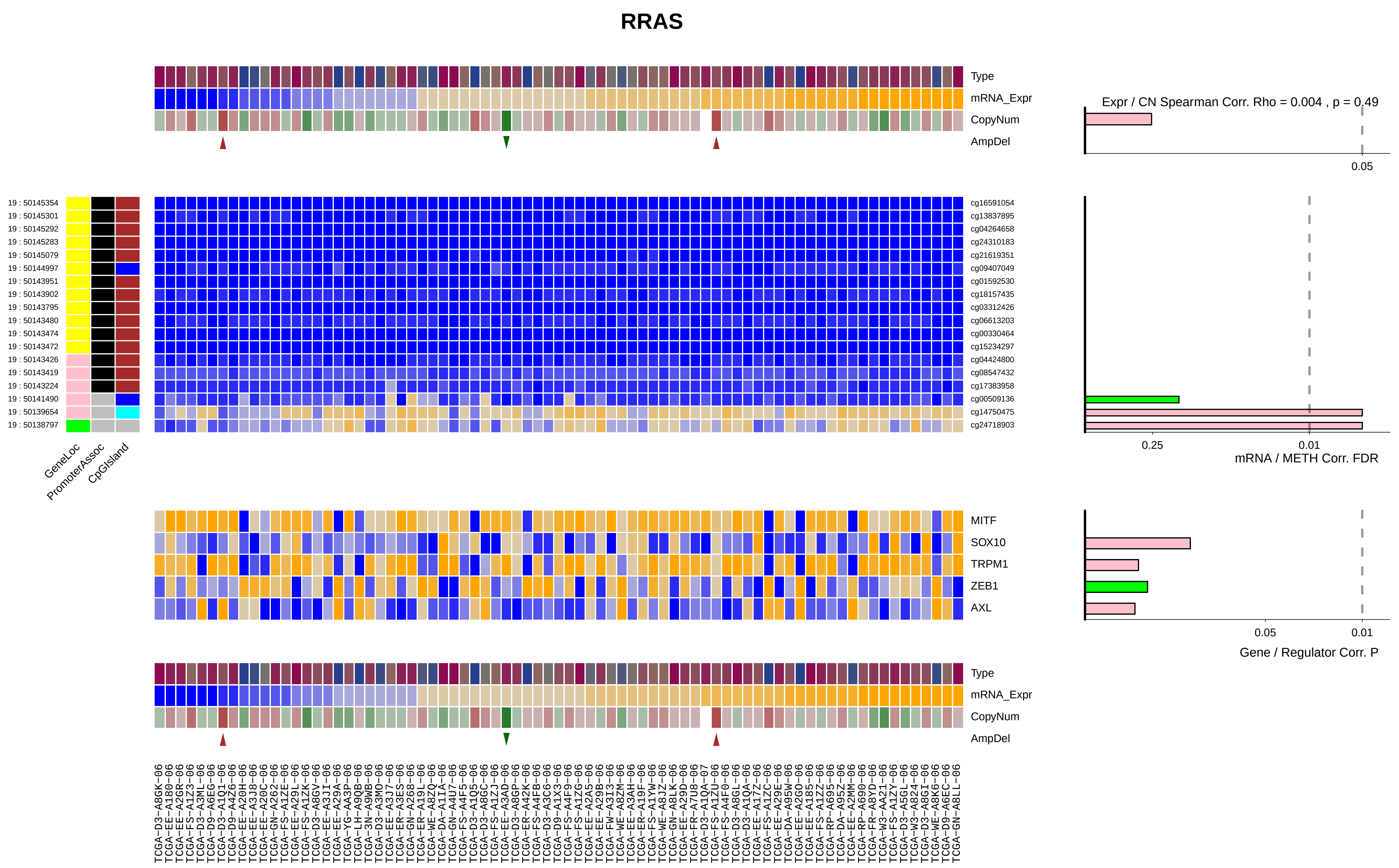

RRAS
Type
mRNA_Expr
CopyNum
AmpDel
Expr / CN Spearman Corr. Rho = 0.004 , p = 0.49
0.05
19 : 50145354
19 : 50145301
19 : 50145292
19 : 50145283
19 : 50145079
19 : 50144997
19 : 50143951
19 : 50143902
19 : 50143795
19 : 50143480
19 : 50143474
19 : 50143472
19 : 50143426
19 : 50143419
19 : 50143224
19 : 50141490
19 : 50139654
19 : 50138797
cg16591054
cg13837895
cg04264658
cg24310183
cg21619351
cg09407049
cg01592530
cg18157435
cg03312426
cg06613203
cg00330464
cg15234297
cg04424800
cg08547432
cg17383958
cg00509136
cg14750475
cg24718903
GeneLoc
PromoterAssoc
CpGIsland
0.25
0.01
mRNA / METH Corr. FDR
MITF
SOX10
TRPM1
ZEB1
AXL
0.05
0.01
Gene / Regulator Corr. P
Type
mRNA_Expr
CopyNum
AmpDel

TGFBR2

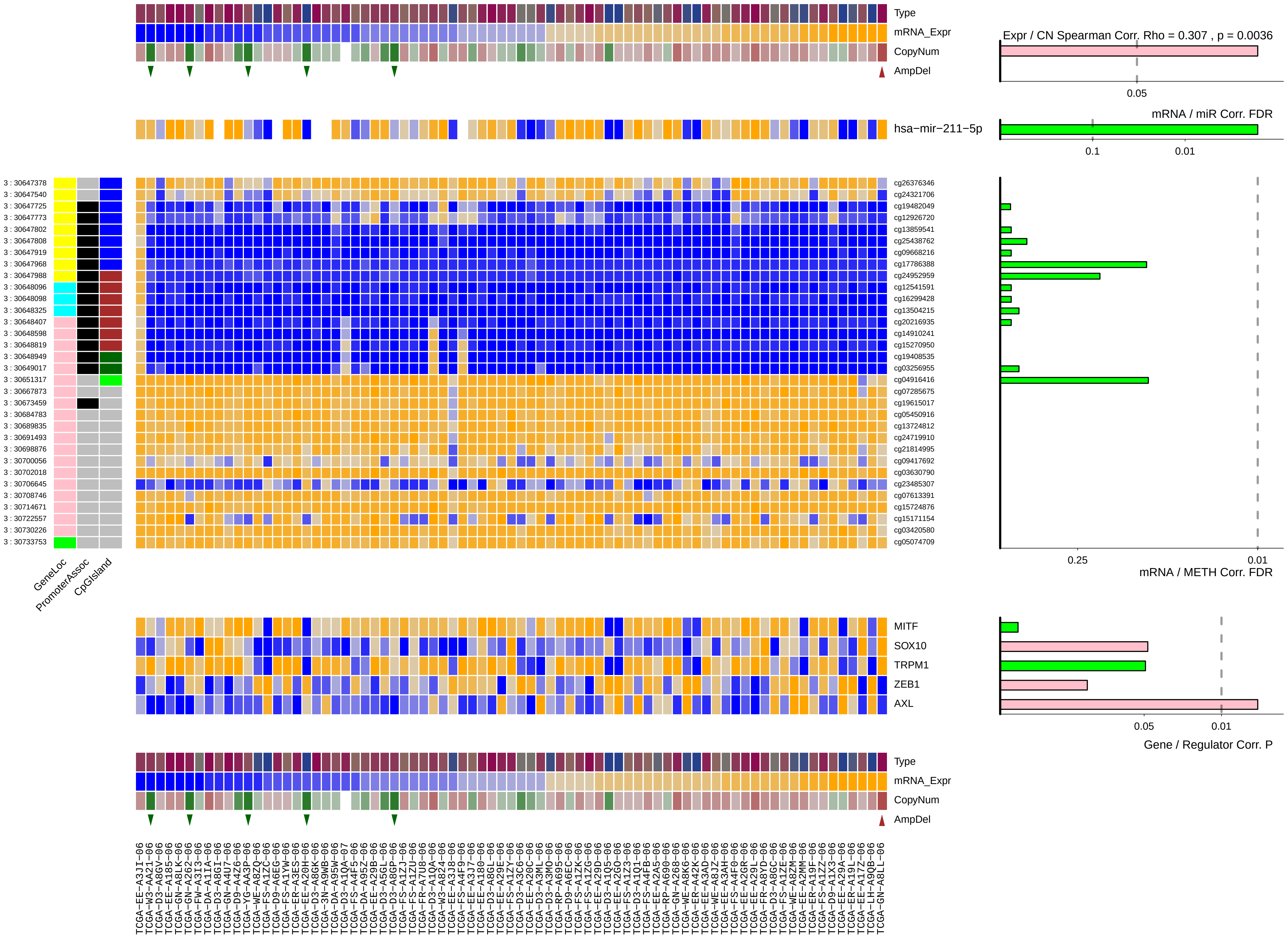

Type
mRNA_Expr
CopyNum
AmpDel
Expr / CN Spearman Corr. Rho = 0.307 , p = 0.0036
hsa−mir−211−5p
mRNA / miR Corr. FDR
mRNA / METH Corr. FDR
GeneLoc
PromoterAssoc
CpGIsland
cg26376346
cg24321706
cg19482049
cg12926720
cg13859541
cg25438762
cg09668216
cg17786388
cg24952959
cg12541591
cg16299428
cg13504215
cg20216935
cg14910241
cg15270950
cg19408535
cg03256955
cg04916416
cg07285675
cg19615017
cg05450916
cg13724812
cg24719910
cg21814995
cg09417692
cg03630790
cg23485307
cg07613391
cg15724876
cg15171154
cg03420580
cg05074709
MITF
SOX10
TRPM1
ZEB1
AXL
Gene / Regulator Corr. P

TPM1

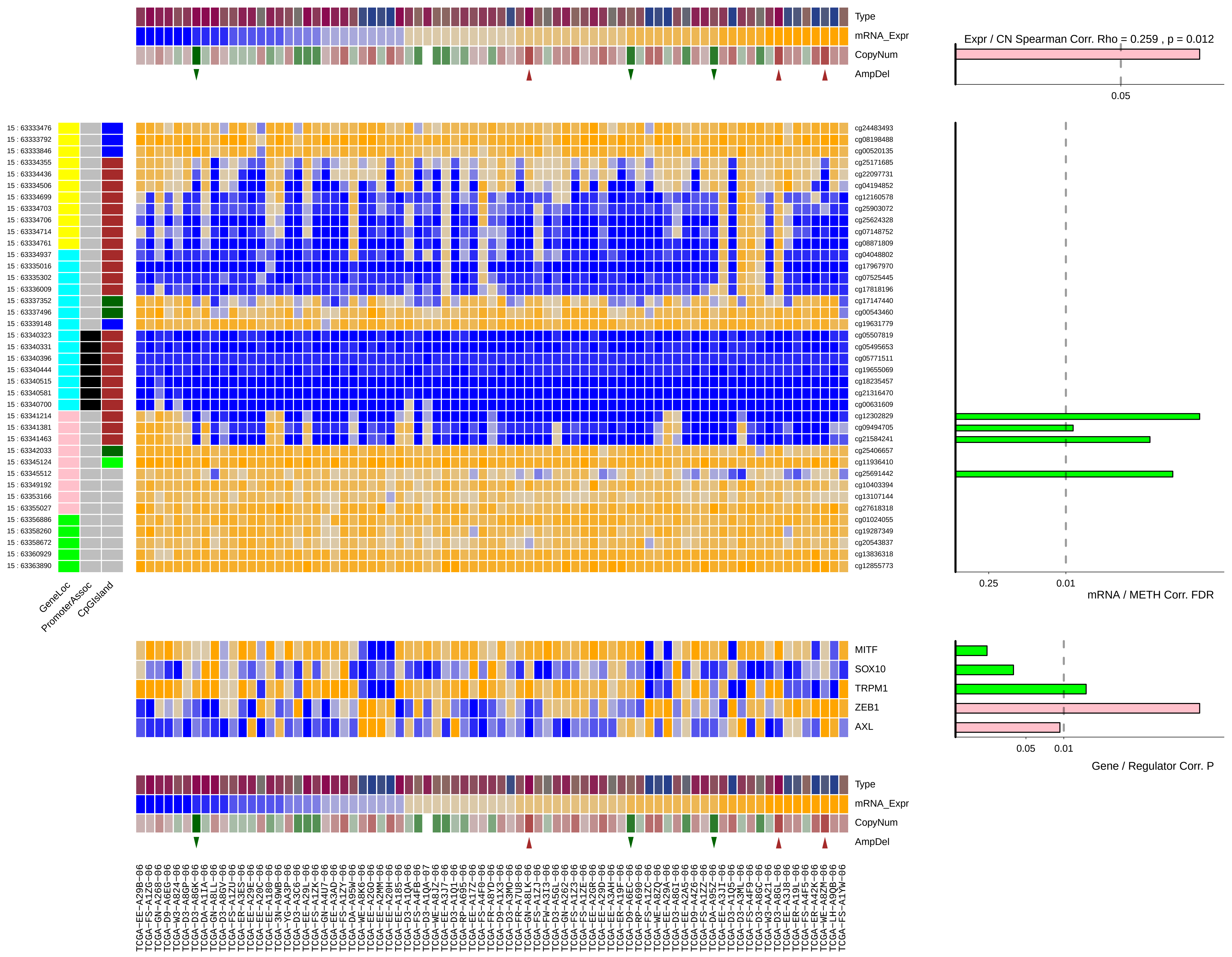
Type
mRNA_Expr
CopyNum
AmpDel
Expr / CN Spearman Corr. Rho = 0.259 , p = 0.012
0.05
15 : 63333476
15 : 63333792
15 : 63333846
15 : 63334355
15 : 63334436
15 : 63334506
15 : 63334699
15 : 63334703
15 : 63334706
15 : 63334714
15 : 63334761
15 : 63334937
15 : 63335016
15 : 63335302
15 : 63336009
15 : 63337352
15 : 63337496
15 : 63339148
15 : 63340323
15 : 63340331
15 : 63340396
15 : 63340444
15 : 63340515
15 : 63340581
15 : 63340700
15 : 63341214
15 : 63341381
15 : 63341463
15 : 63342033
15 : 63345124
15 : 63345512
15 : 63349192
15 : 63353166
15 : 63355027
15 : 63356886
15 : 63358260
15 : 63358672
15 : 63360929
15 : 63363890
cg24483493
cg08198488
cg00520135
cg25171685
cg22097731
cg04194852
cg12160578
cg25903072
cg25624328
cg07148752
cg08871809
cg04048802
cg17967970
cg07525445
cg17818196
cg17147440
cg00543460
cg19631779
cg05507819
cg05495653
cg05771511
cg19655069
cg18235457
cg21316470
cg00631609
cg12302829
cg09494705
cg21584241
cg25406657
cg11936410
cg25691442
cg10403394
cg13107144
cg27618318
cg01024055
cg19287349
cg20543837
cg13836318
cg12855773
0.25
0.01
mRNA / METH Corr. FDR
GeneLoc
PromoterAssoc
CpGIsland
MITF
SOX10
TRPM1
ZEB1
AXL
0.05
0.01
Gene / Regulator Corr. P
Type
mRNA_Expr
CopyNum
AmpDel

BDNF

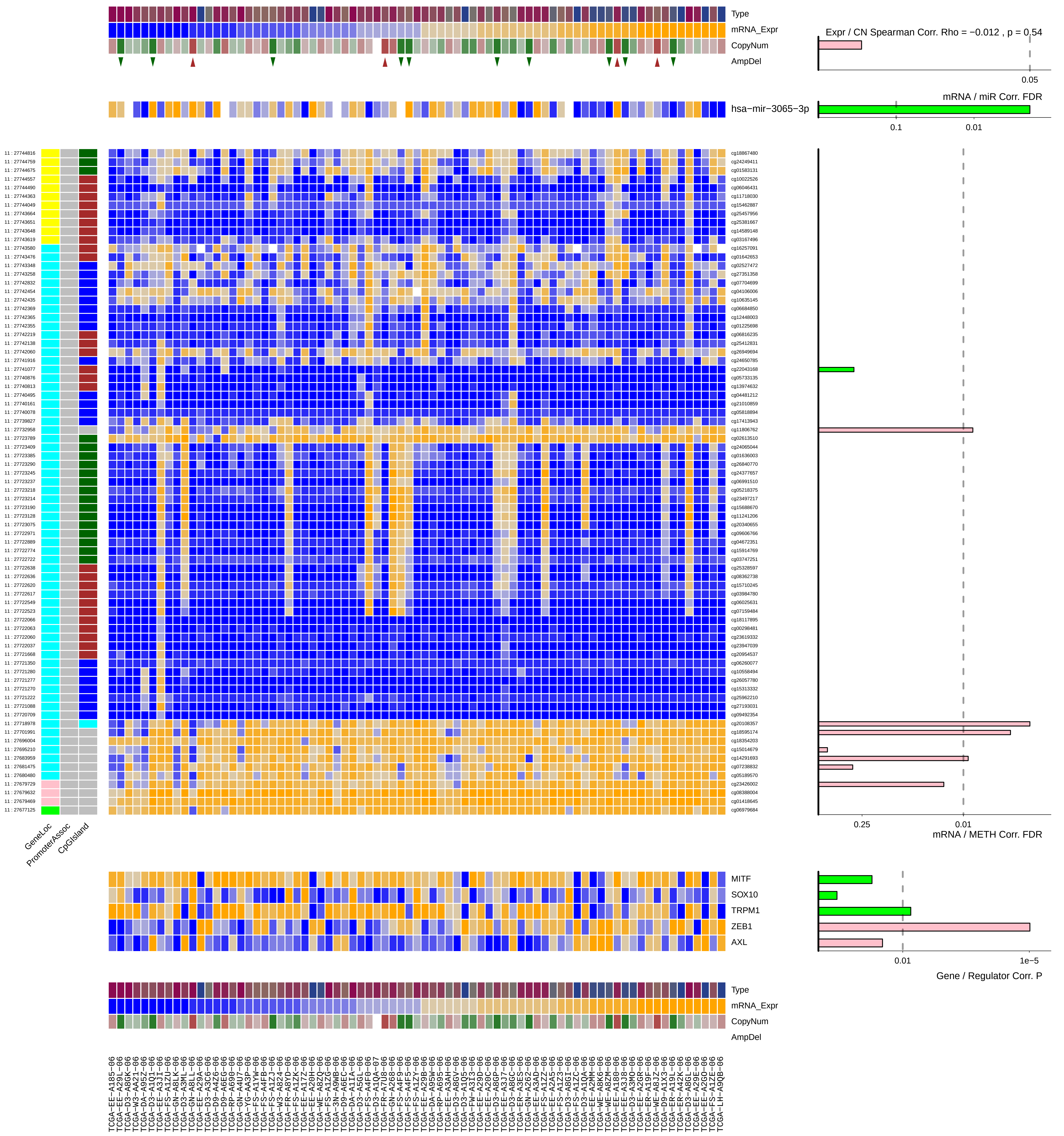
Type
mRNA_Expr
CopyNum
AmpDel
Expr / CN Spearman Corr. Rho = −0.012 , p = 0.54
0.05
mRNA / miR Corr. FDR
hsa−mir−3065−3p
0.1
0.01
0.25
0.01
mRNA / METH Corr. FDR
GeneLoc
PromoterAssoc
CpGIsland
MITF
SOX10
TRPM1
ZEB1
AXL
0.01
1e−5
Gene / Regulator Corr. P

GBP1

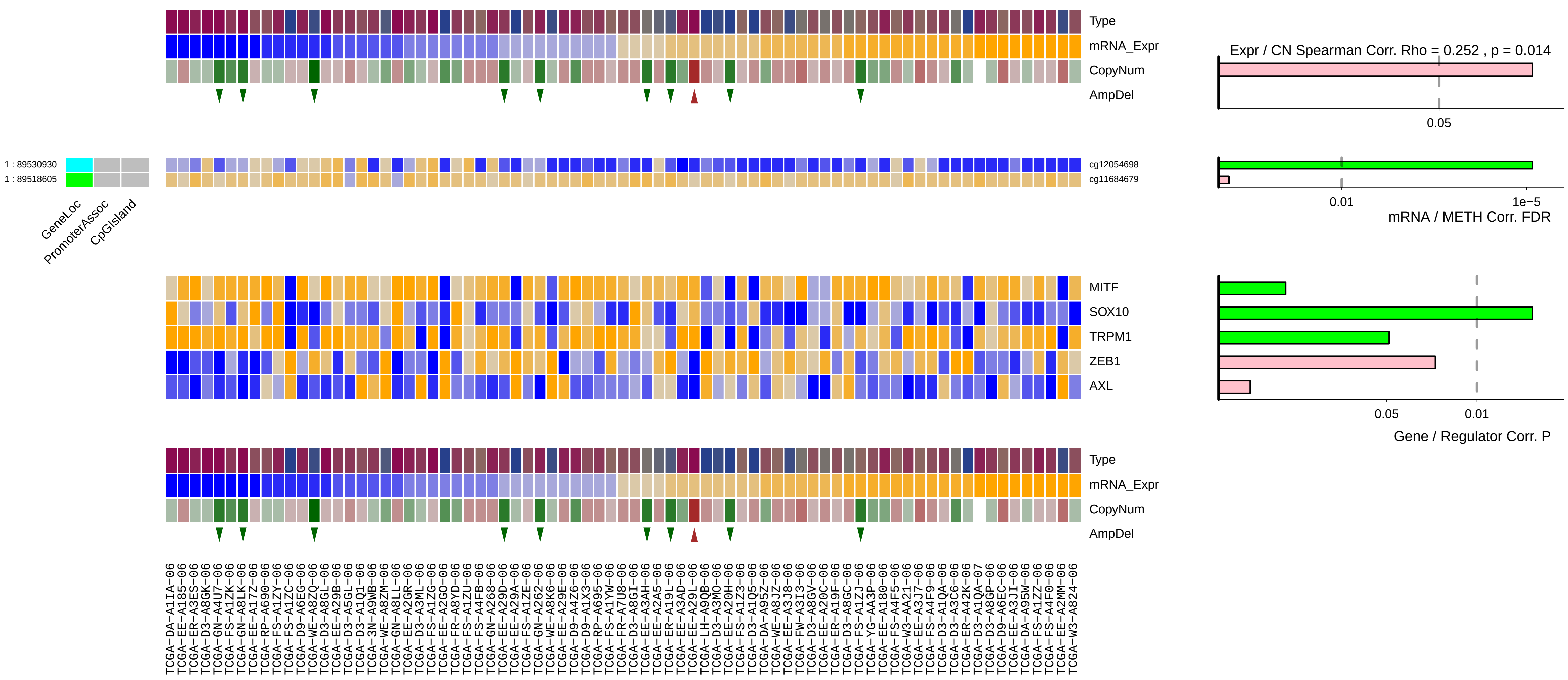

Type
mRNA_Expr
CopyNum
AmpDel
Expr / CN Spearman Corr. Rho = 0.252 , p = 0.014
0.05
1 : 89530930
1 : 89518605
GeneLoc
PromoterAssoc
CpGIsland
cg12054698
cg11684679
0.01
1e−5
mRNA / METH Corr. FDR
MITF
SOX10
TRPM1
ZEB1
AXL
0.05
0.01
Gene / Regulator Corr. P

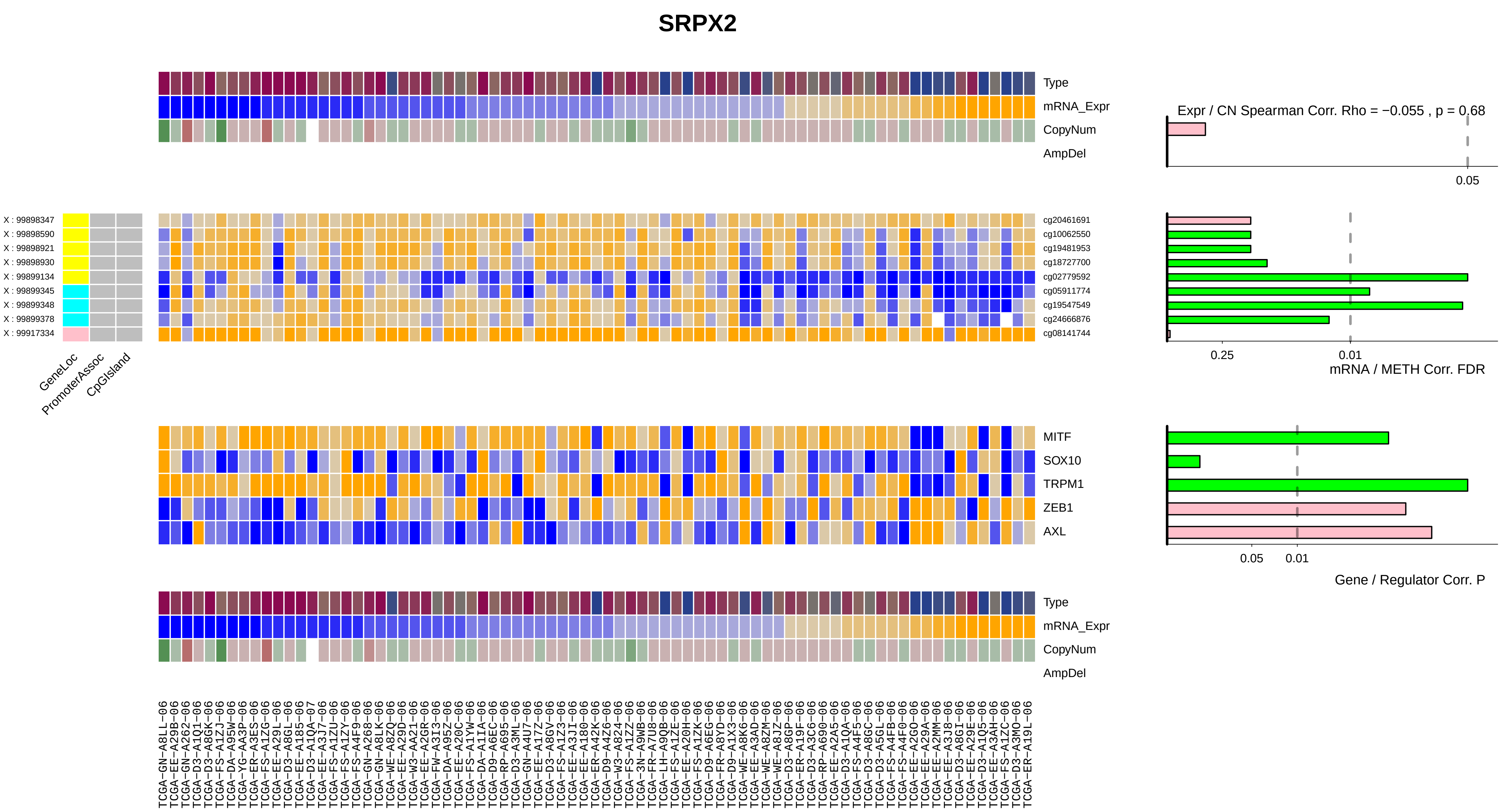
SRPX2
Type
mRNA_Expr
CopyNum
AmpDel
Expr / CN Spearman Corr. Rho = −0.055 , p = 0.68
0.05
X : 99898347
X : 99898590
X : 99898921
X : 99898930
X : 99899134
X : 99899345
X : 99899348
X : 99899378
X : 99917334
GeneLoc
PromoterAssoc
CpGIsland
cg20461691
cg10062550
cg19481953
cg18727700
cg02779592
cg05911774
cg19547549
cg24666876
cg08141744
0.25
0.01
mRNA / METH Corr. FDR
MITF
SOX10
TRPM1
ZEB1
AXL
0.05
0.01
Gene / Regulator Corr. P
Type
mRNA_Expr
CopyNum
AmpDel

SMAGP

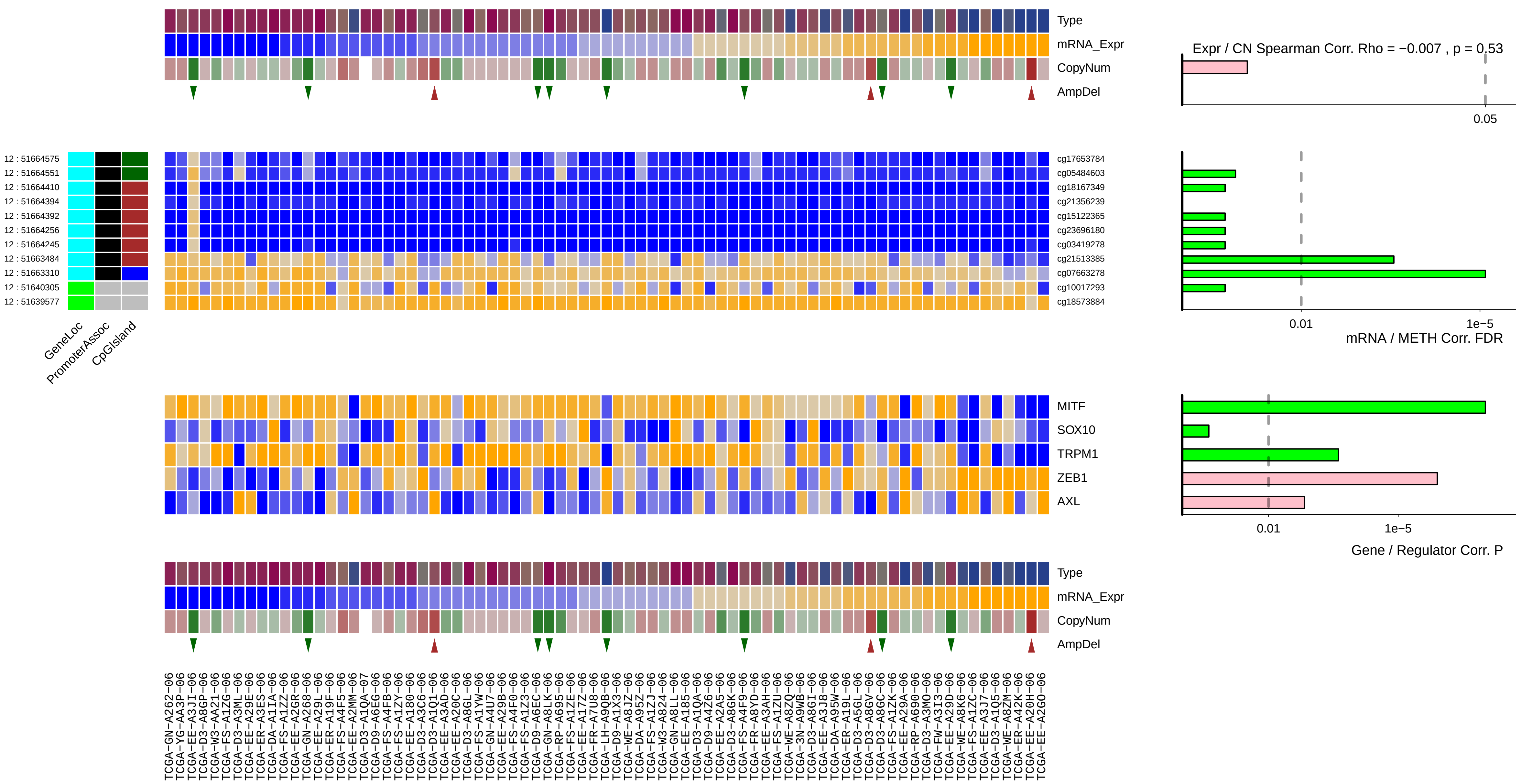

Type
mRNA_Expr
CopyNum
AmpDel
Expr / CN Spearman Corr. Rho = −0.007 , p = 0.53
0.05
12 : 51664575
12 : 51664551
12 : 51664410
12 : 51664394
12 : 51664392
12 : 51664256
12 : 51664245
12 : 51663484
12 : 51663310
12 : 51640305
12 : 51639577
cg17653784
cg05484603
cg18167349
cg21356239
cg15122365
cg23696180
cg03419278
cg21513385
cg07663278
cg10017293
cg18573884
GeneLoc
PromoterAssoc
CpGIsland
0.01
1e−5
mRNA / METH Corr. FDR
MITF
SOX10
TRPM1
ZEB1
AXL
Gene / Regulator Corr. P

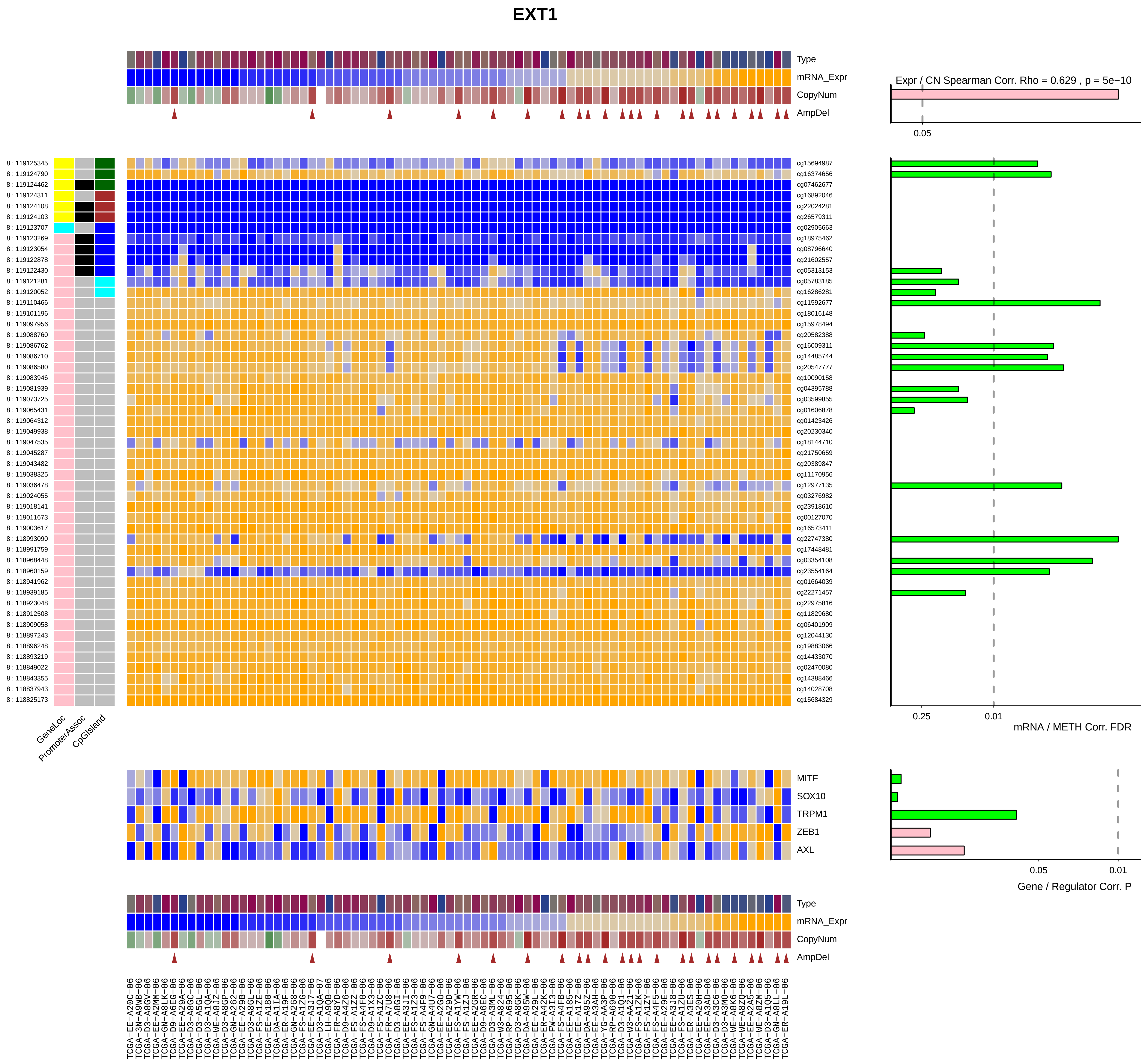
EXT1
Type
mRNA_Expr
CopyNum
AmpDel
Expr / CN Spearman Corr. Rho = 0.629 , p = 5e−10
0.05
0.25
0.01
mRNA / METH Corr. FDR
GeneLoc
PromoterAssoc
CpGIsland
MITF
SOX10
TRPM1
ZEB1
AXL
0.05
0.01
Gene / Regulator Corr. P
Type
mRNA_Expr
CopyNum
AmpDel

TMEM158

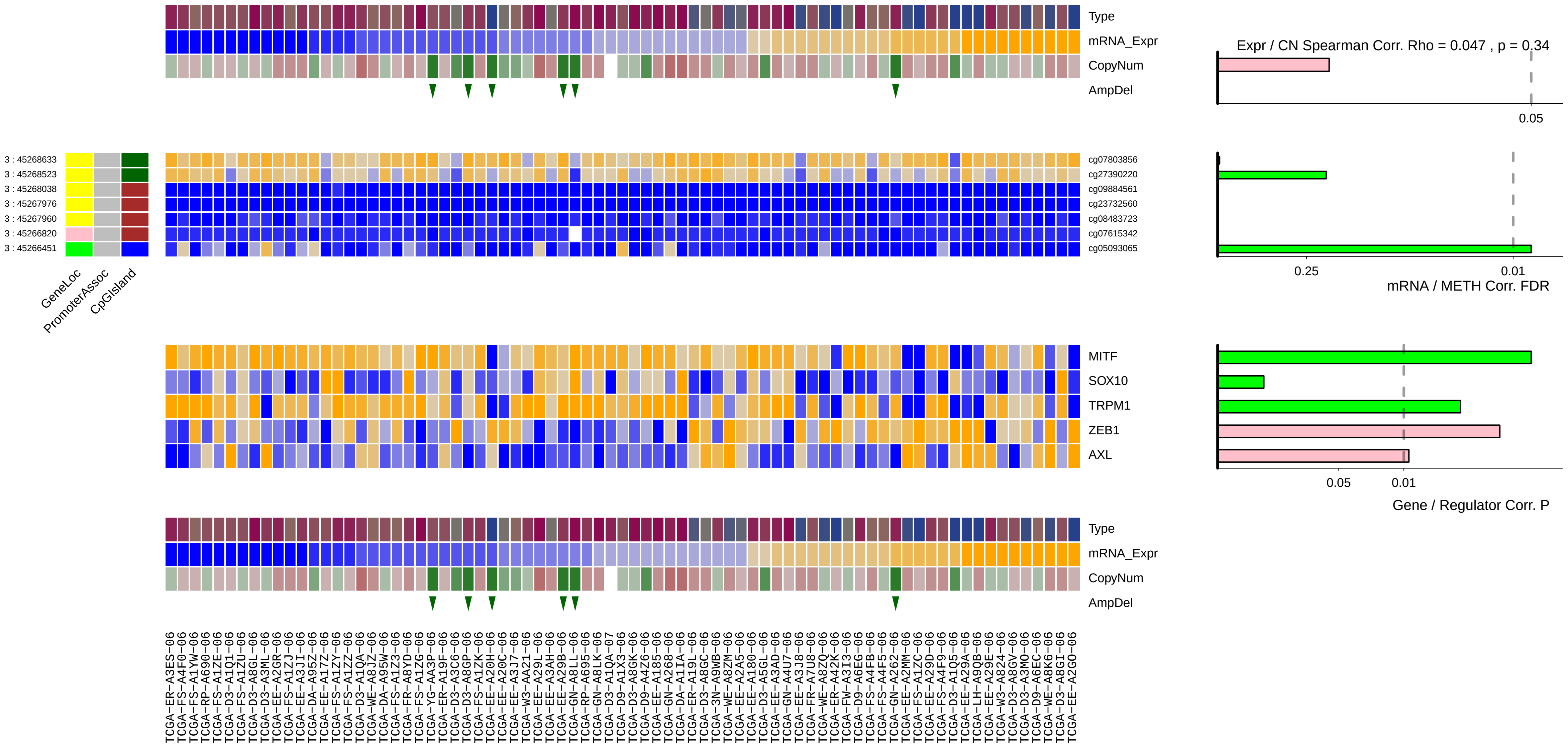

Type
mRNA_Expr
CopyNum
AmpDel
Expr / CN Spearman Corr. Rho = 0.047 , p = 0.34
0.05
3 : 45268633
3 : 45268523
3 : 45268038
3 : 45267976
3 : 45267960
3 : 45266820
3 : 45266451
cg07803856
cg27390220
cg09884561
cg23732560
cg08483723
cg07615342
cg05093065
0.25
0.01
mRNA / METH Corr. FDR
GeneLoc
PromoterAssoc
CpGIsland
MITF
SOX10
TRPM1
ZEB1
AXL
0.05
0.01
Gene / Regulator Corr. P

AFAP1

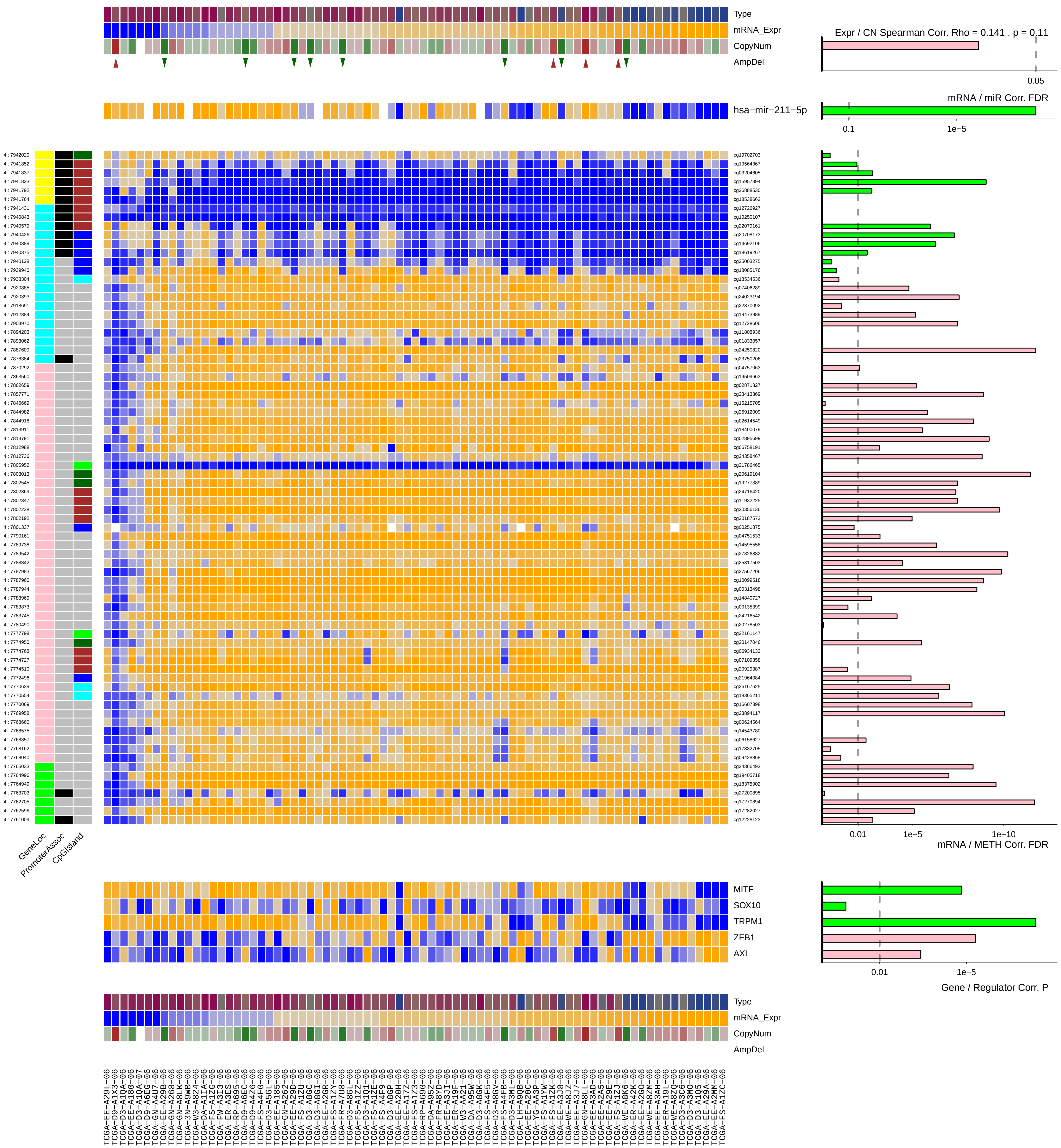

Type
mRNA_Expr
CopyNum
AmpDel
Expr / CN Spearman Corr. Rho = 0.141 , p = 0.11
0.05
hsa−mir−211−5p
mRNA / miR Corr. FDR
0.1
1e−5
0.01
1e−5
1e−10
mRNA / METH Corr. FDR
GeneLoc
PromoterAssoc
CpGIsland
MITF
SOX10
TRPM1
ZEB1
AXL
0.01
1e−5
Gene / Regulator Corr. P

ITGA2

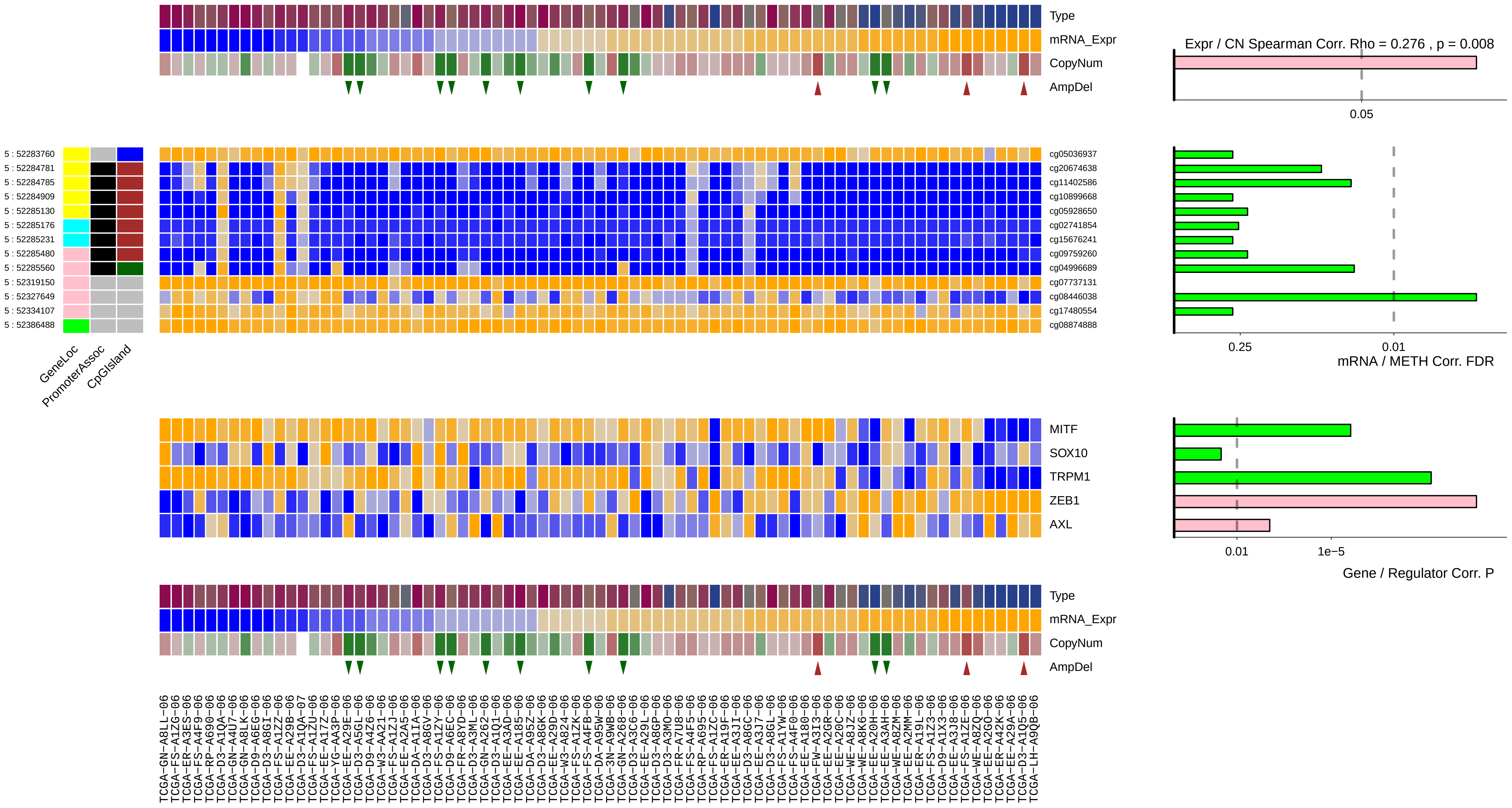
Type
mRNA_Expr
CopyNum
AmpDel
Expr / CN Spearman Corr. Rho = 0.276 , p = 0.008
0.05
5 : 52283760
5 : 52284781
5 : 52284785
5 : 52284909
5 : 52285130
5 : 52285176
5 : 52285231
5 : 52285480
5 : 52285560
5 : 52319150
5 : 52327649
5 : 52334107
5 : 52386488
GeneLoc
PromoterAssoc
CpGIsland
cg05036937
cg20674638
cg11402586
cg10899668
cg05928650
cg02741854
cg15676241
cg09759260
cg04996689
cg07737131
cg08446038
cg17480554
cg08874888
0.25
0.01
mRNA / METH Corr. FDR
MITF
SOX10
TRPM1
ZEB1
AXL
0.01
1e−5
Gene / Regulator Corr. P
Type
mRNA_Expr
CopyNum
AmpDel

AKR1C3

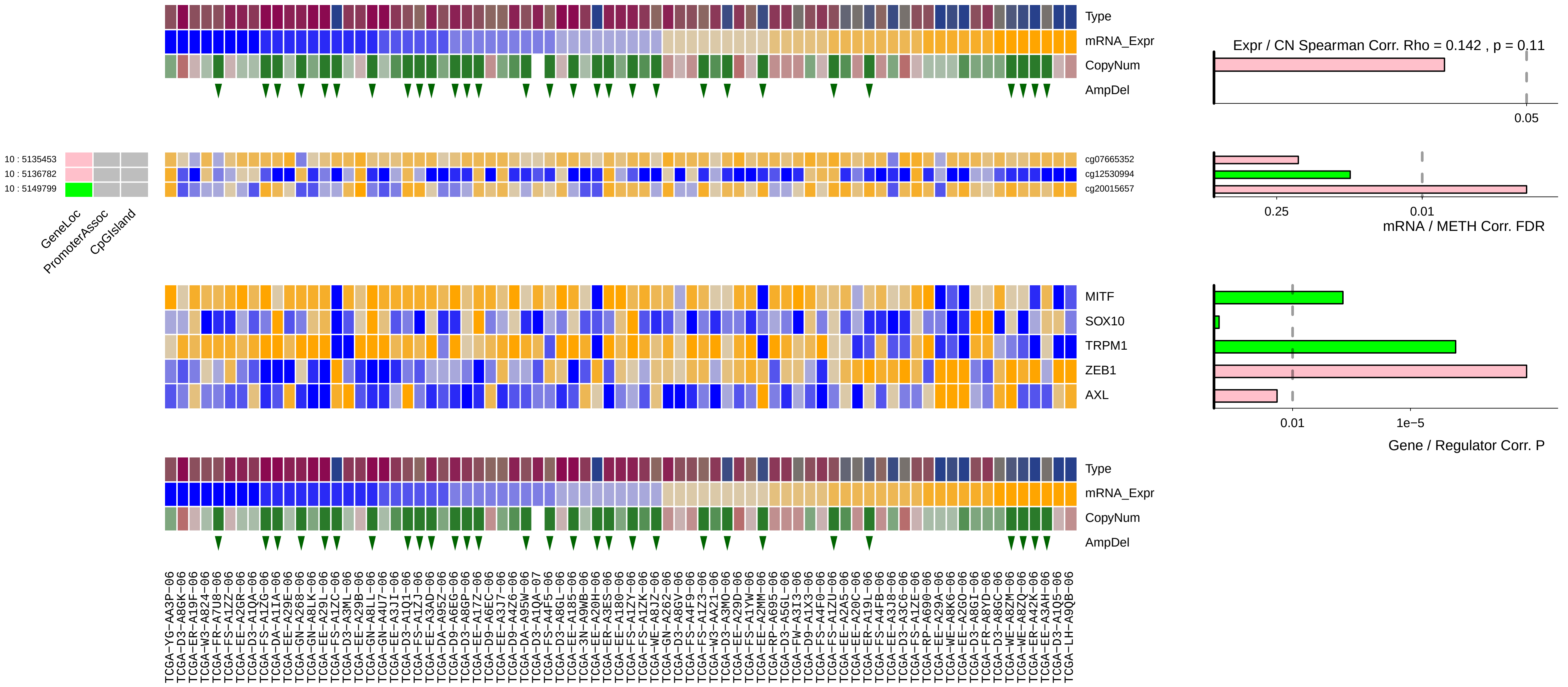
Type
mRNA_Expr
CopyNum
AmpDel
Expr / CN Spearman Corr. Rho = 0.142 , p = 0.11
0.05
10 : 5135453
10 : 5136782
10 : 5149799
GeneLoc
PromoterAssoc
CpGIsland
cg07665352
cg12530994
cg20015657
0.25
0.01
mRNA / METH Corr. FDR
MITF
SOX10
TRPM1
ZEB1
AXL
0.01
1e−5
Gene / Regulator Corr. P

ARL4C

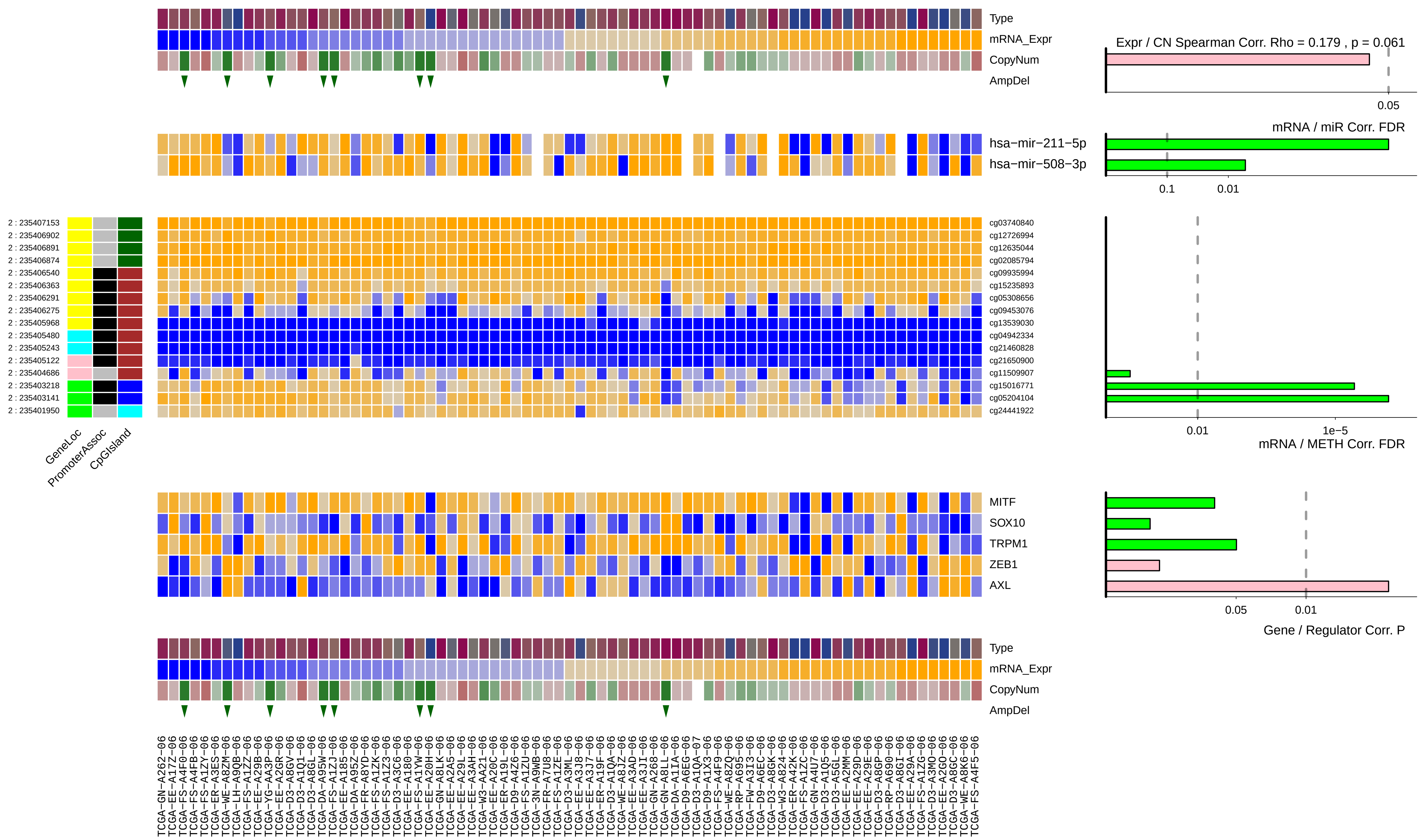

Type
mRNA_Expr
CopyNum
AmpDel
Expr / CN Spearman Corr. Rho = 0.179 , p = 0.061
0.05
mRNA / miR Corr. FDR
hsa−mir−211−5p
hsa−mir−508−3p
0.1
0.01
cg03740840
cg12726994
cg12635044
cg02085794
cg09935994
cg15235893
cg05308656
cg09453076
cg13539030
cg04942334
cg21460828
cg21650900
cg11509907
cg15016771
cg05204104
cg24441922
0.01
1e−5
mRNA / METH Corr. FDR
GeneLoc
PromoterAssoc
CpGIsland
MITF
SOX10
TRPM1
ZEB1
AXL
0.05
0.01
Gene / Regulator Corr. P

HRH1

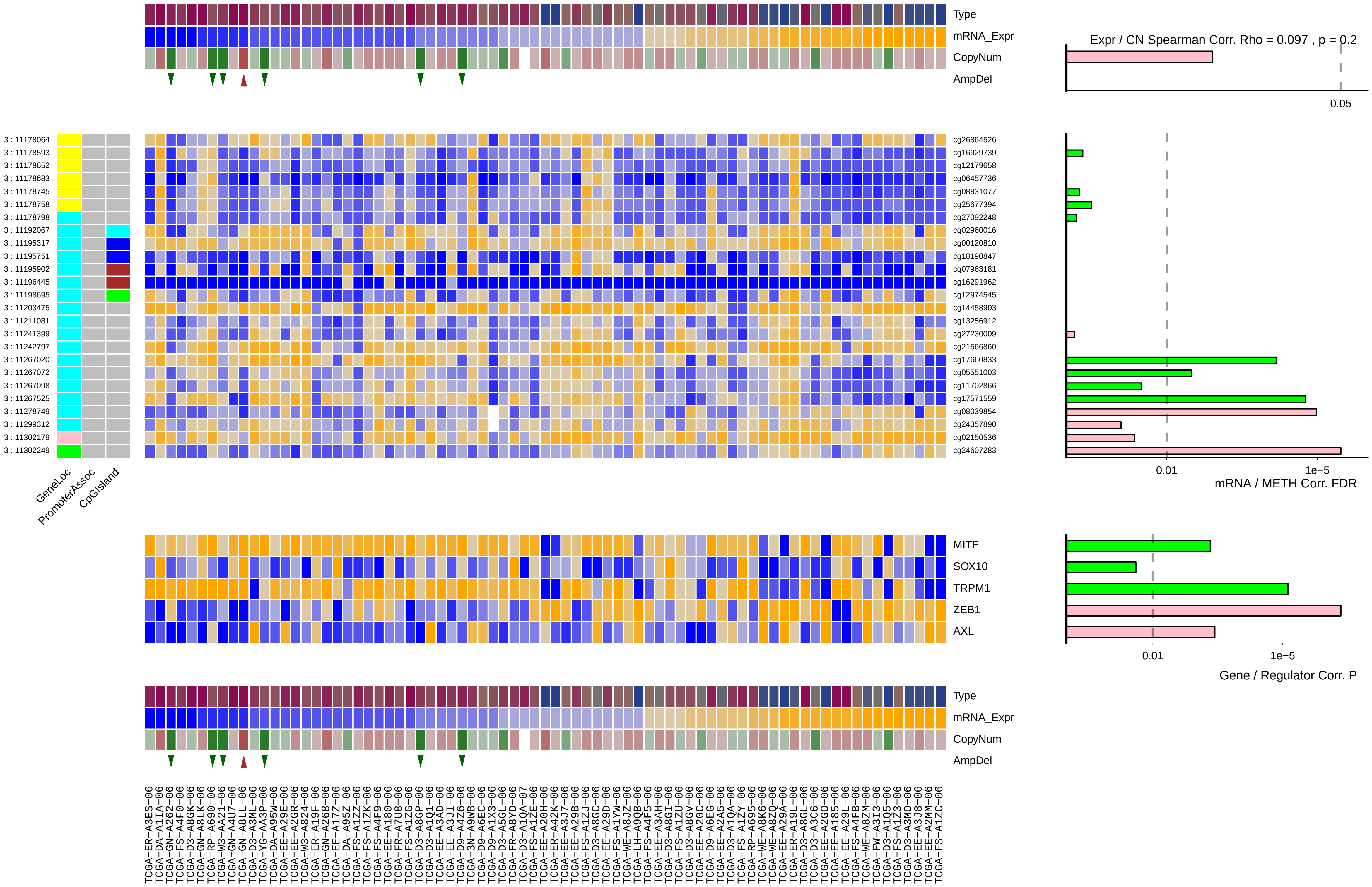
Type
mRNA_Expr
CopyNum
AmpDel
Expr / CN Spearman Corr. Rho = 0.097 , p = 0.2
0.05
3 : 11178064
3 : 11178593
3 : 11178652
3 : 11178683
3 : 11178745
3 : 11178758
3 : 11178798
3 : 11192067
3 : 11195317
3 : 11195751
3 : 11195902
3 : 11196445
3 : 11198695
3 : 11203475
3 : 11211081
3 : 11241399
3 : 11242797
3 : 11267020
3 : 11267072
3 : 11267098
3 : 11267525
3 : 11278749
3 : 11299312
3 : 11302179
3 : 11302249
cg26864526
cg16929739
cg12179658
cg06457736
cg08831077
cg25677394
cg27092248
cg02960016
cg00120810
cg18190847
cg07963181
cg16291962
cg12974545
cg14458903
cg13256912
cg27230009
cg21566860
cg17660833
cg05551003
cg11702866
cg17571559
cg08039854
cg24357890
cg02150536
cg24607283
GeneLoc
PromoterAssoc
CpGIsland
0.01
1e−5
mRNA / METH Corr. FDR
MITF
SOX10
TRPM1
ZEB1
AXL
0.01
1e−5
Gene / Regulator Corr. P
Type
mRNA_Expr
CopyNum
AmpDel

CLU

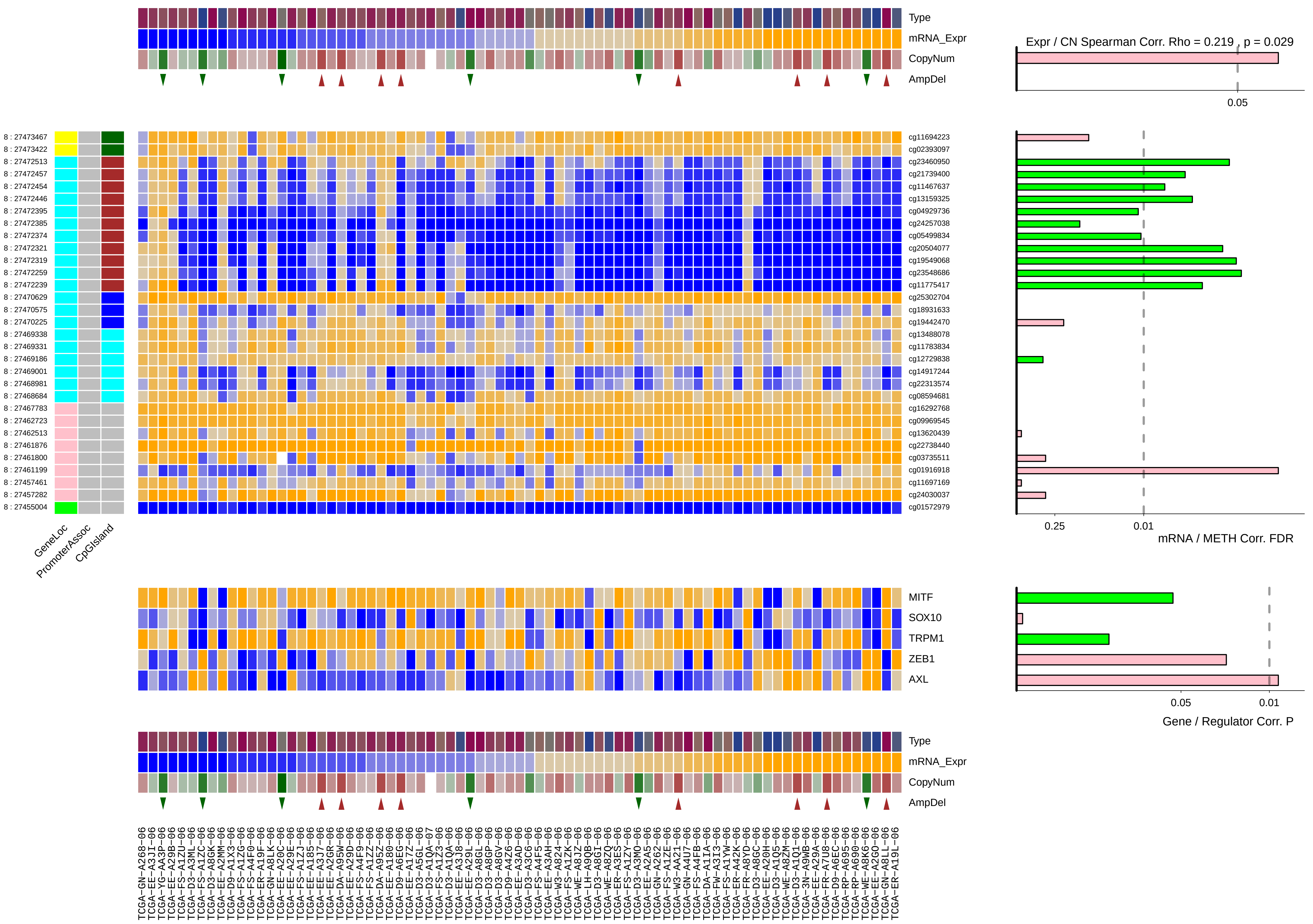
Type
mRNA_Expr
CopyNum
AmpDel
Expr / CN Spearman Corr. Rho = 0.219 , p = 0.029
0.05
GeneLoc
PromoterAssoc
CpGIsland
0.25
0.01
mRNA / METH Corr. FDR
MITF
SOX10
TRPM1
ZEB1
AXL
0.05
0.01
Gene / Regulator Corr. P

ADAMTS6

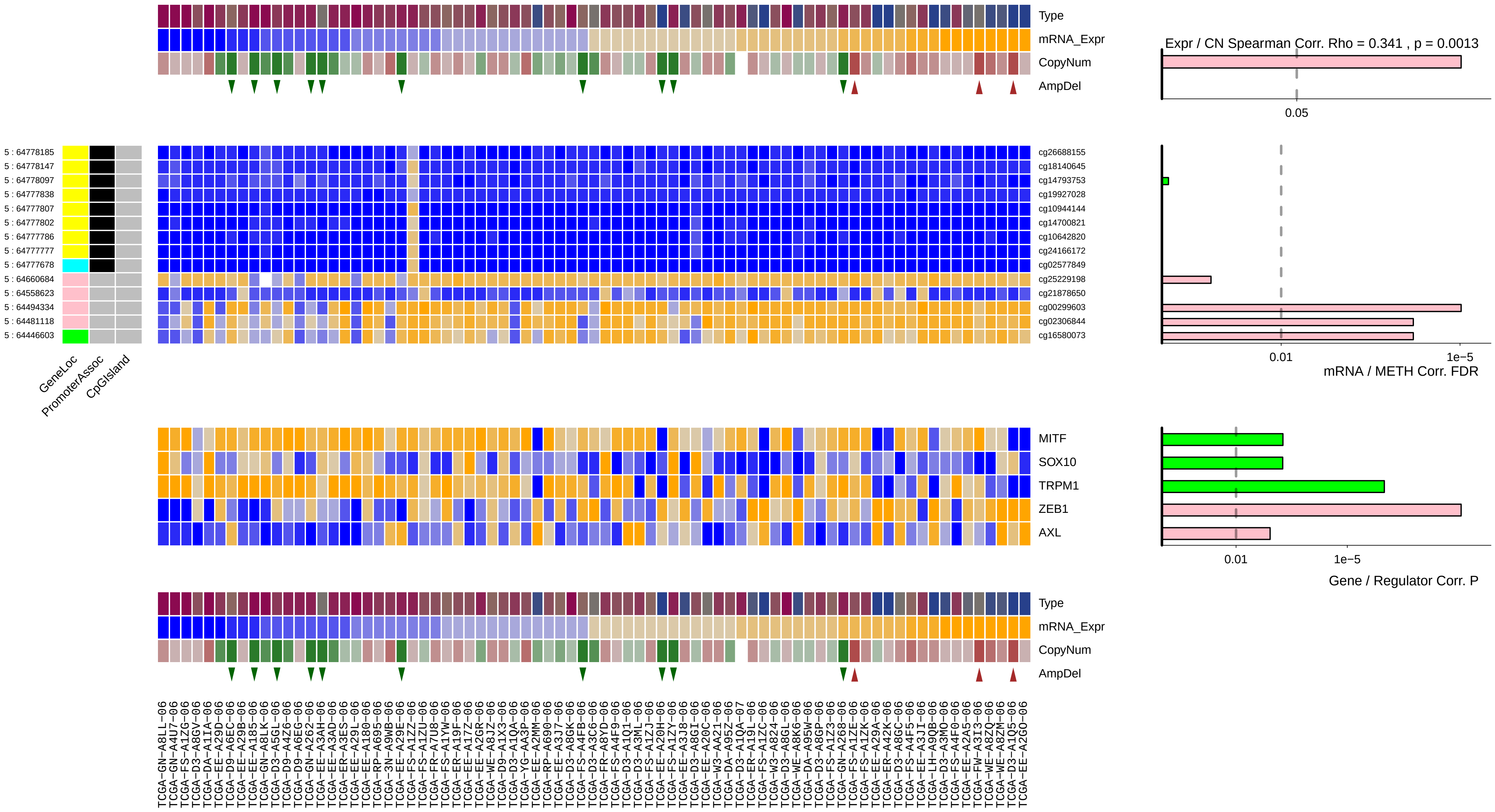
Type
mRNA_Expr
CopyNum
AmpDel
Expr / CN Spearman Corr. Rho = 0.341 , p = 0.0013
0.05
cg26688155
cg18140645
cg14793753
cg19927028
cg10944144
cg14700821
cg10642820
cg24166172
cg02577849
cg25229198
cg21878650
cg00299603
cg02306844
cg16580073
5 : 64778185
5 : 64778147
5 : 64778097
5 : 64777838
5 : 64777807
5 : 64777802
5 : 64777786
5 : 64777777
5 : 64777678
5 : 64660684
5 : 64558623
5 : 64494334
5 : 64481118
5 : 64446603
GeneLoc
PromoterAssoc
CpGIsland
0.01
1e−5
mRNA / METH Corr. FDR
MITF
SOX10
TRPM1
ZEB1
AXL
0.01
1e−5
Gene / Regulator Corr. P

ABCC3

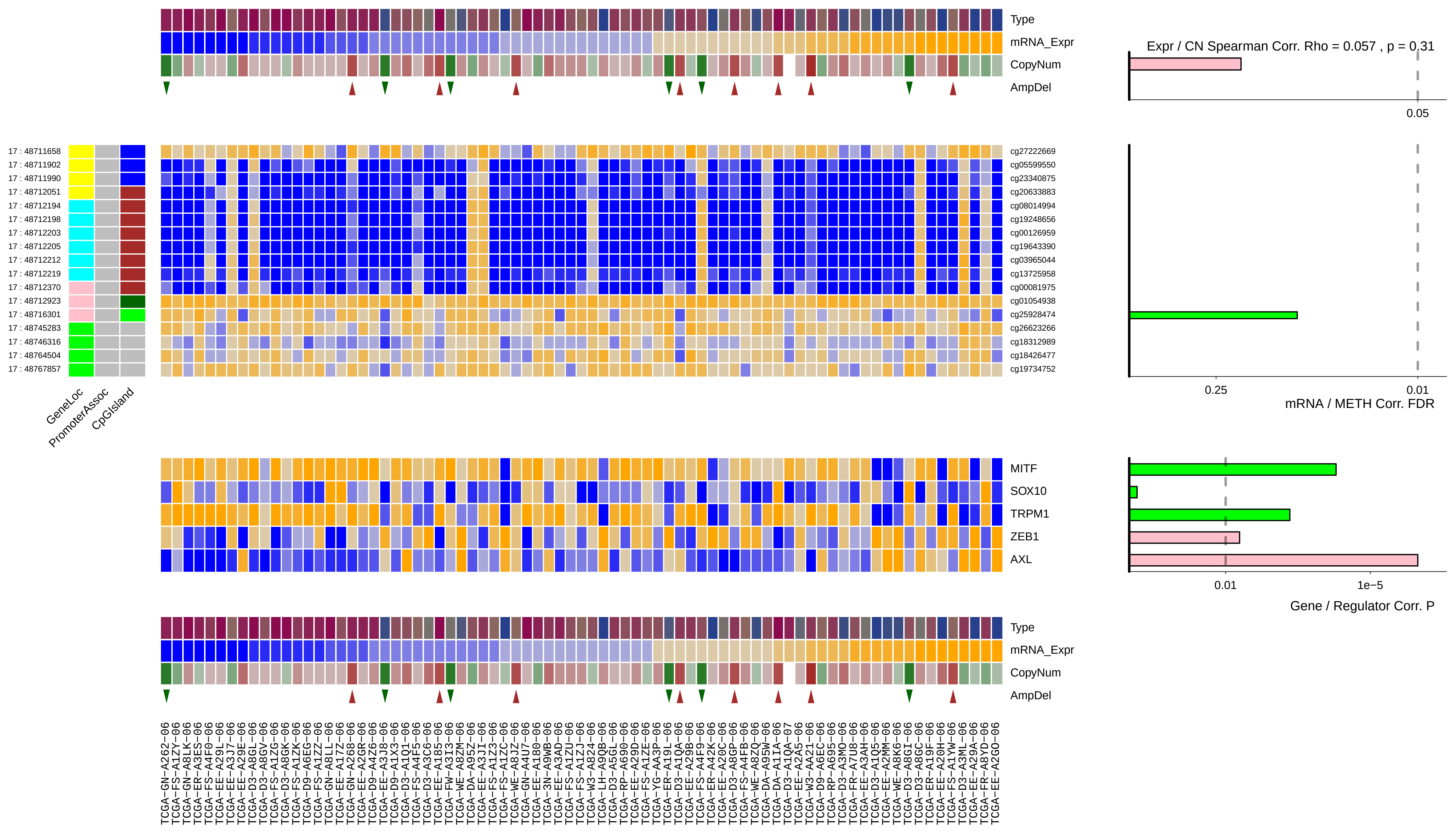

Type
mRNA_Expr
CopyNum
AmpDel
Expr / CN Spearman Corr. Rho = 0.057 , p = 0.31
0.05
GeneLoc
PromoterAssoc
CpGIsland
0.25
0.01
mRNA / METH Corr. FDR
MITF
SOX10
TRPM1
ZEB1
AXL
0.01
1e−5
Gene / Regulator Corr. P

CSF1

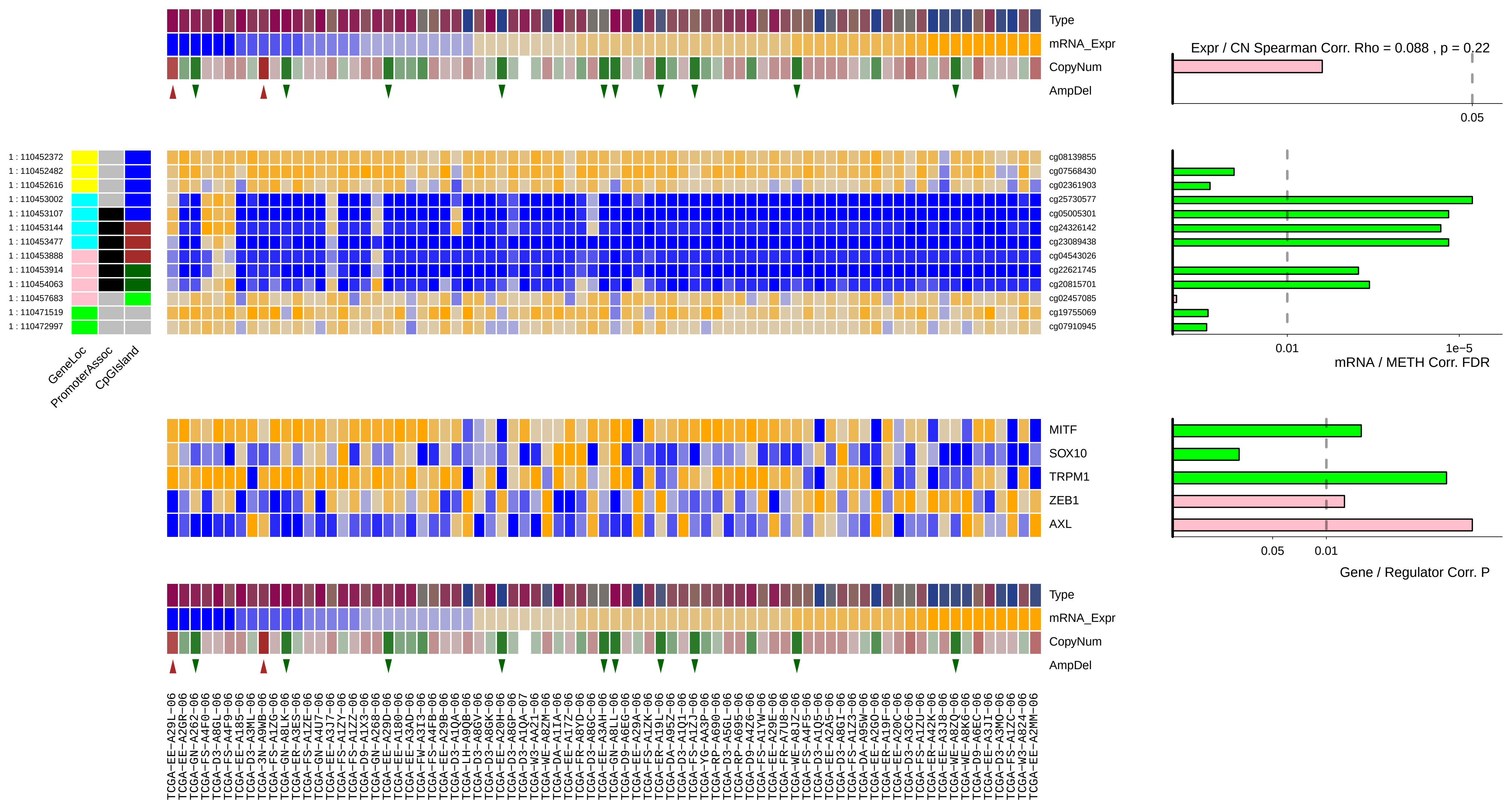

Type
mRNA_Expr
CopyNum
AmpDel
Expr / CN Spearman Corr. Rho = 0.088 , p = 0.22
0.05
cg08139855
cg07568430
cg02361903
cg25730577
cg05005301
cg24326142
cg23089438
cg04543026
cg22621745
cg20815701
cg02457085
cg19755069
cg07910945
1 : 110452372
1 : 110452482
1 : 110452616
1 : 110453002
1 : 110453107
1 : 110453144
1 : 110453477
1 : 110453888
1 : 110453914
1 : 110454063
1 : 110457683
1 : 110471519
1 : 110472997
GeneLoc
PromoterAssoc
CpGIsland
0.01
1e−5
mRNA / METH Corr. FDR
MITF
SOX10
TRPM1
ZEB1
AXL
0.05
0.01
Gene / Regulator Corr. P

HEG1

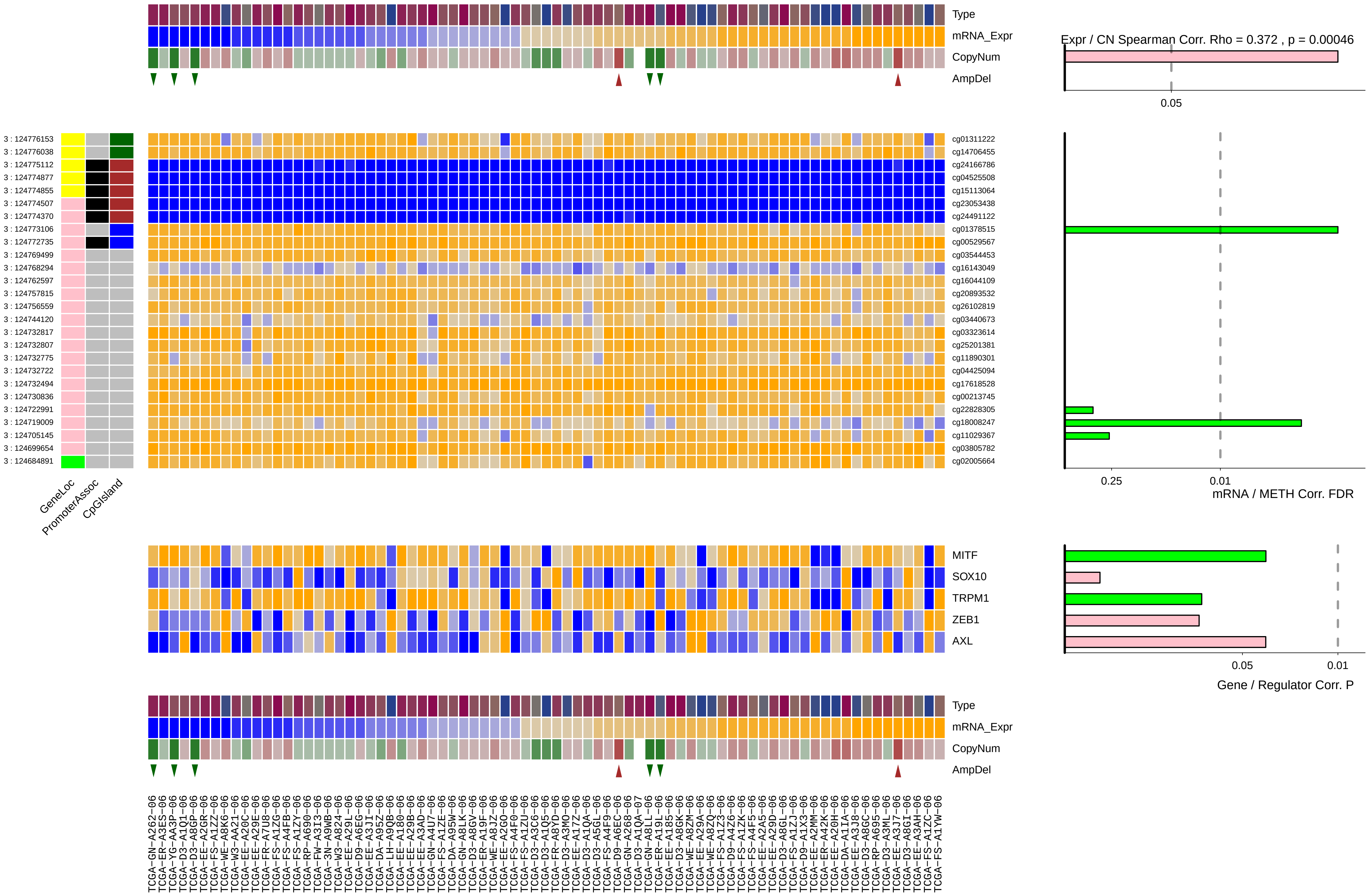
Type
mRNA_Expr
CopyNum
AmpDel
Expr / CN Spearman Corr. Rho = 0.372 , p = 0.00046
0.05
GeneLoc
PromoterAssoc
CpGIsland
0.25
0.01
mRNA / METH Corr. FDR
MITF
SOX10
TRPM1
ZEB1
AXL
Gene / Regulator Corr. P

SPEG

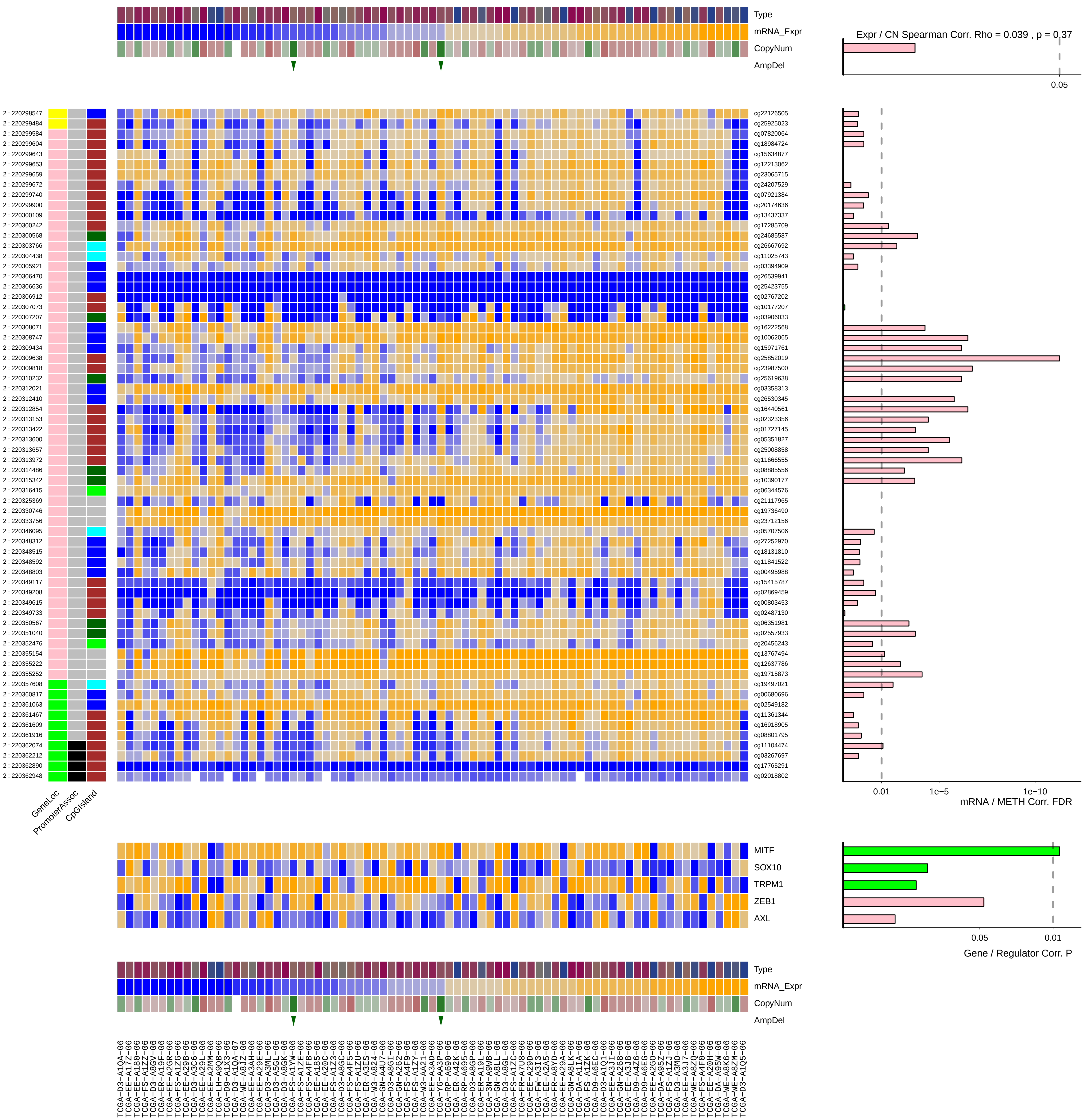

Type
mRNA_Expr
CopyNum
AmpDel
Expr / CN Spearman Corr. Rho = 0.039 , p = 0.37
0.05
0.01
1e−5
1e−10
mRNA / METH Corr. FDR
GeneLoc
PromoterAssoc
CpGIsland
MITF
SOX10
TRPM1
ZEB1
AXL
0.05
0.01
Gene / Regulator Corr. P

FBN1

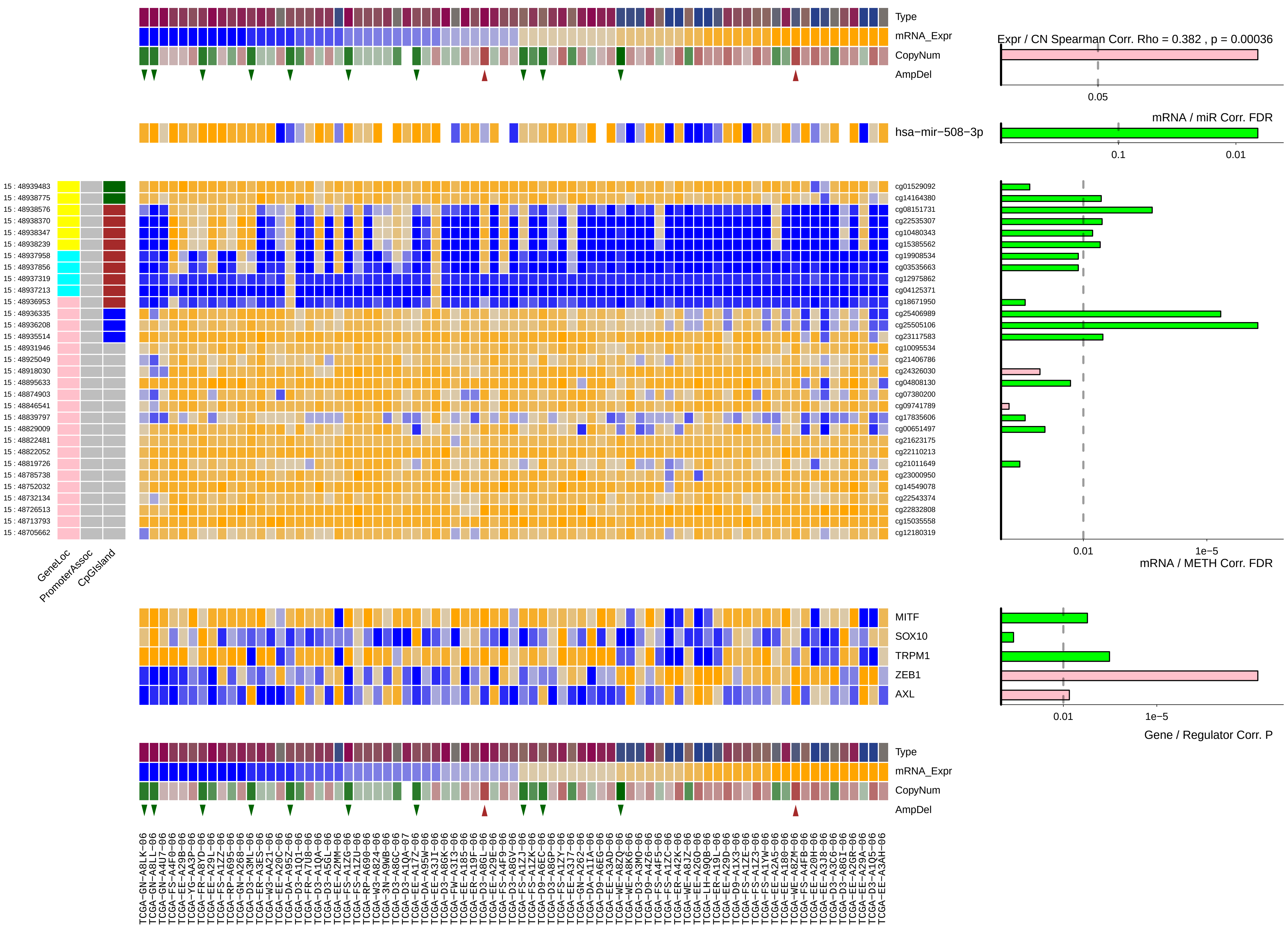
Type
mRNA_Expr
CopyNum
AmpDel
Expr / CN Spearman Corr. Rho = 0.382 , p = 0.00036
0.05
hsa−mir−508−3p
mRNA / miR Corr. FDR
0.1
0.01
GeneLoc
PromoterAssoc
CpGIsland
mRNA / METH Corr. FDR
0.01
1e−5
MITF
SOX10
TRPM1
ZEB1
AXL
Gene / Regulator Corr. P
0.01
1e−5

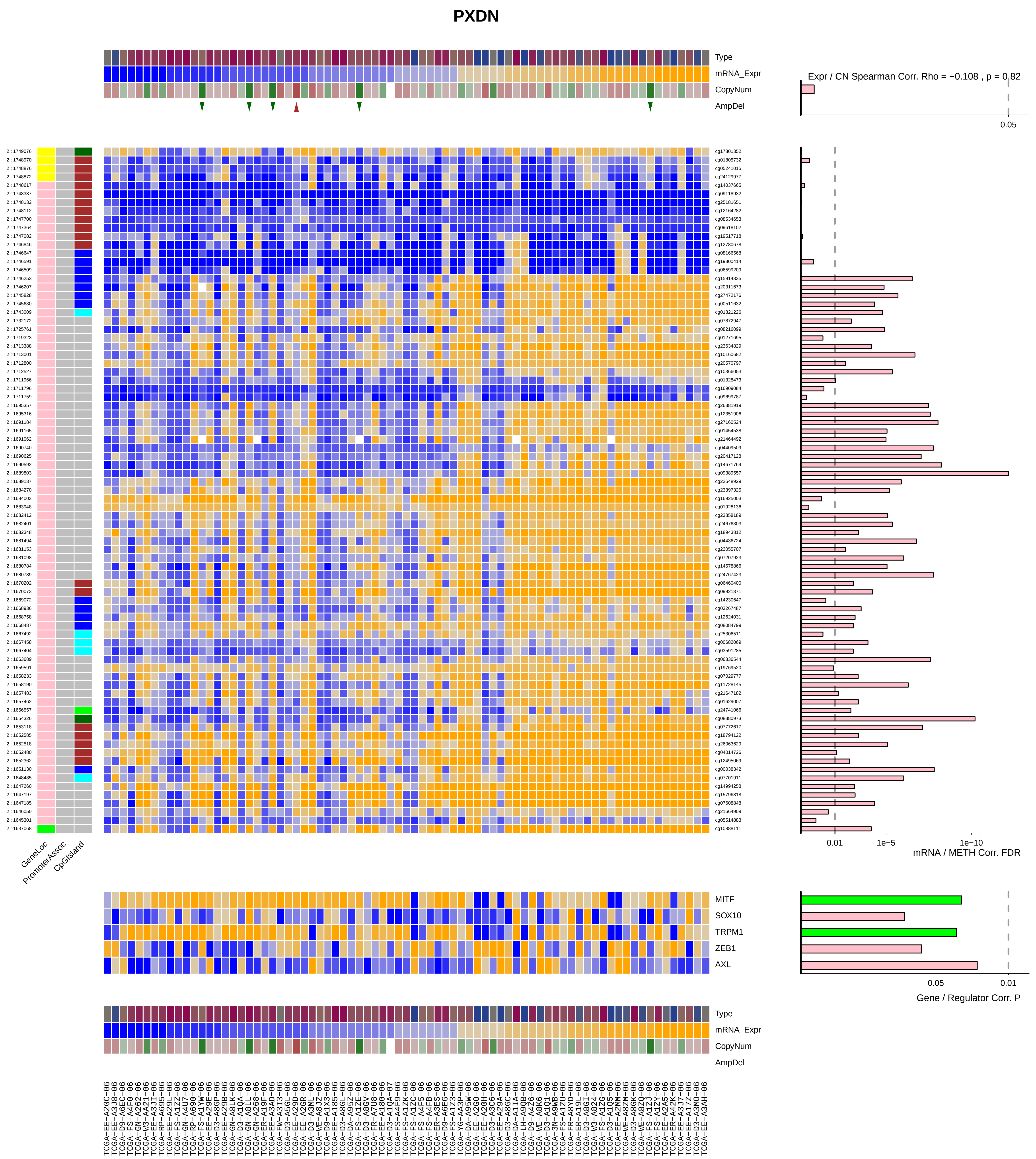
PXDN
Type
mRNA_Expr
CopyNum
AmpDel
Expr / CN Spearman Corr. Rho = −0.108 , p = 0.82
0.05
GeneLoc
PromoterAssoc
CpGIsland
0.01
1e−5
1e−10
mRNA / METH Corr. FDR
MITF
SOX10
TRPM1
ZEB1
AXL
0.05
0.01
Gene / Regulator Corr. P

ALCAM

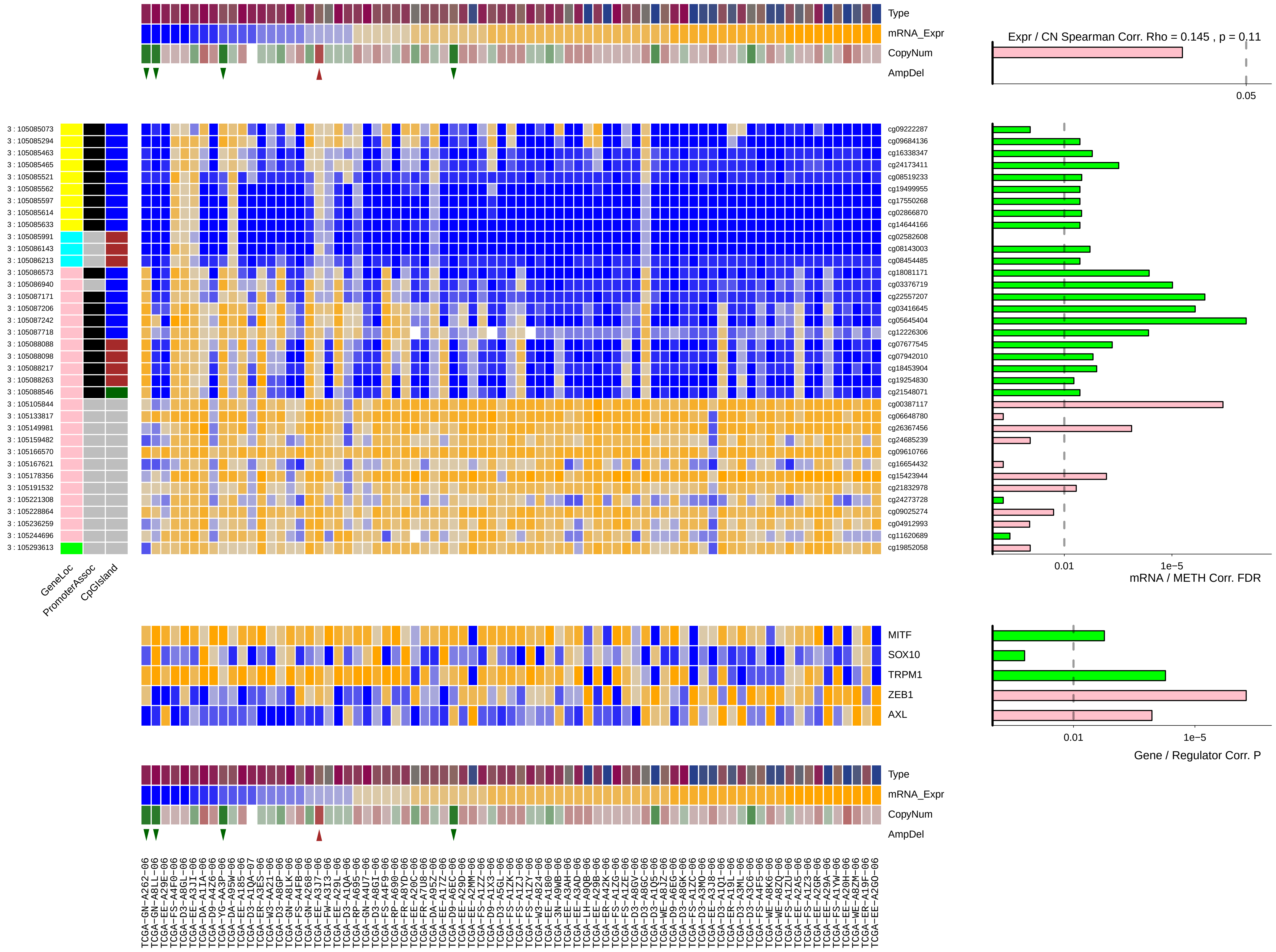
Type
mRNA_Expr
CopyNum
AmpDel
Expr / CN Spearman Corr. Rho = 0.145 , p = 0.11
0.05
GeneLoc
PromoterAssoc
CpGIsland
mRNA / METH Corr. FDR
0.01
1e−5
MITF
SOX10
TRPM1
ZEB1
AXL
Gene / Regulator Corr. P

GLIS3

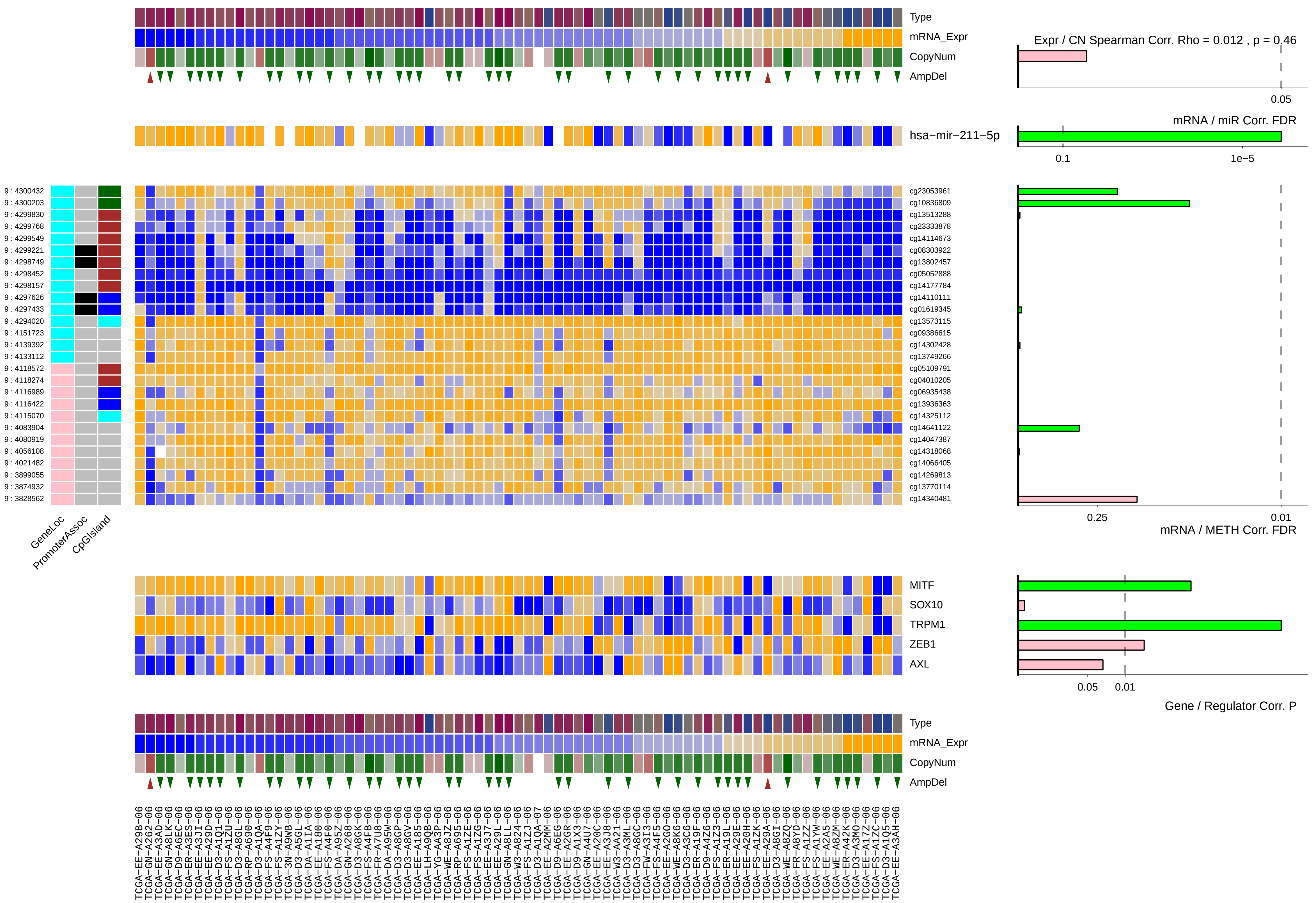

Type
mRNA_Expr
CopyNum
AmpDel
Expr / CN Spearman Corr. Rho = 0.012 , p = 0.46
0.05
hsa−mir−211−5p
mRNA / miR Corr. FDR
0.1
1e−5
mRNA / METH Corr. FDR
0.25
0.01
GeneLoc
PromoterAssoc
CpGIsland
MITF
SOX10
TRPM1
ZEB1
AXL
Gene / Regulator Corr. P

SPOCK1

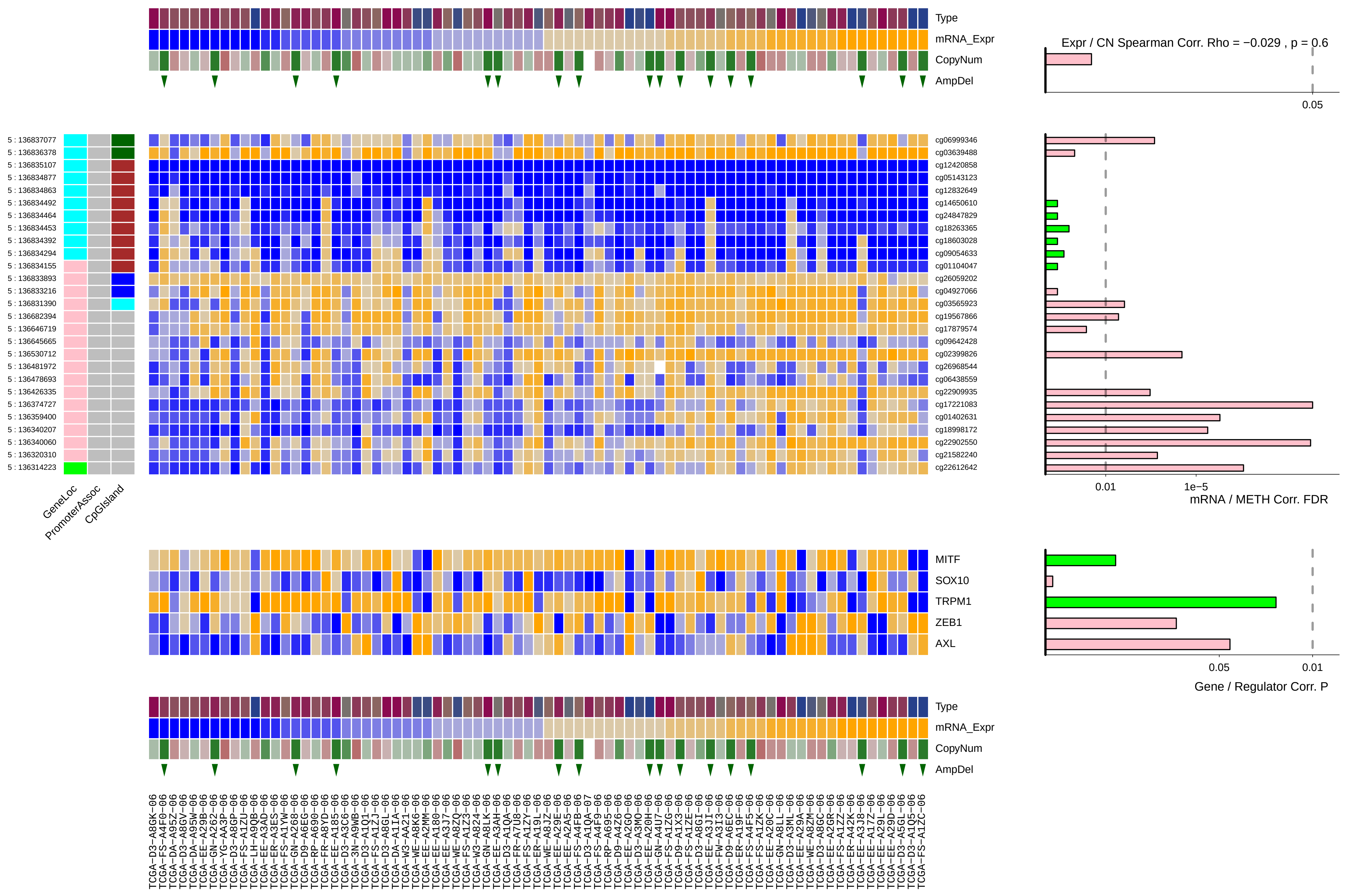

Type
mRNA_Expr
CopyNum
AmpDel
Expr / CN Spearman Corr. Rho = −0.029 , p = 0.6
0.05
GeneLoc
PromoterAssoc
CpGIsland
mRNA / METH Corr. FDR
0.01
1e−5
MITF
SOX10
TRPM1
ZEB1
AXL
Gene / Regulator Corr. P
0.05
0.01

WNT5B

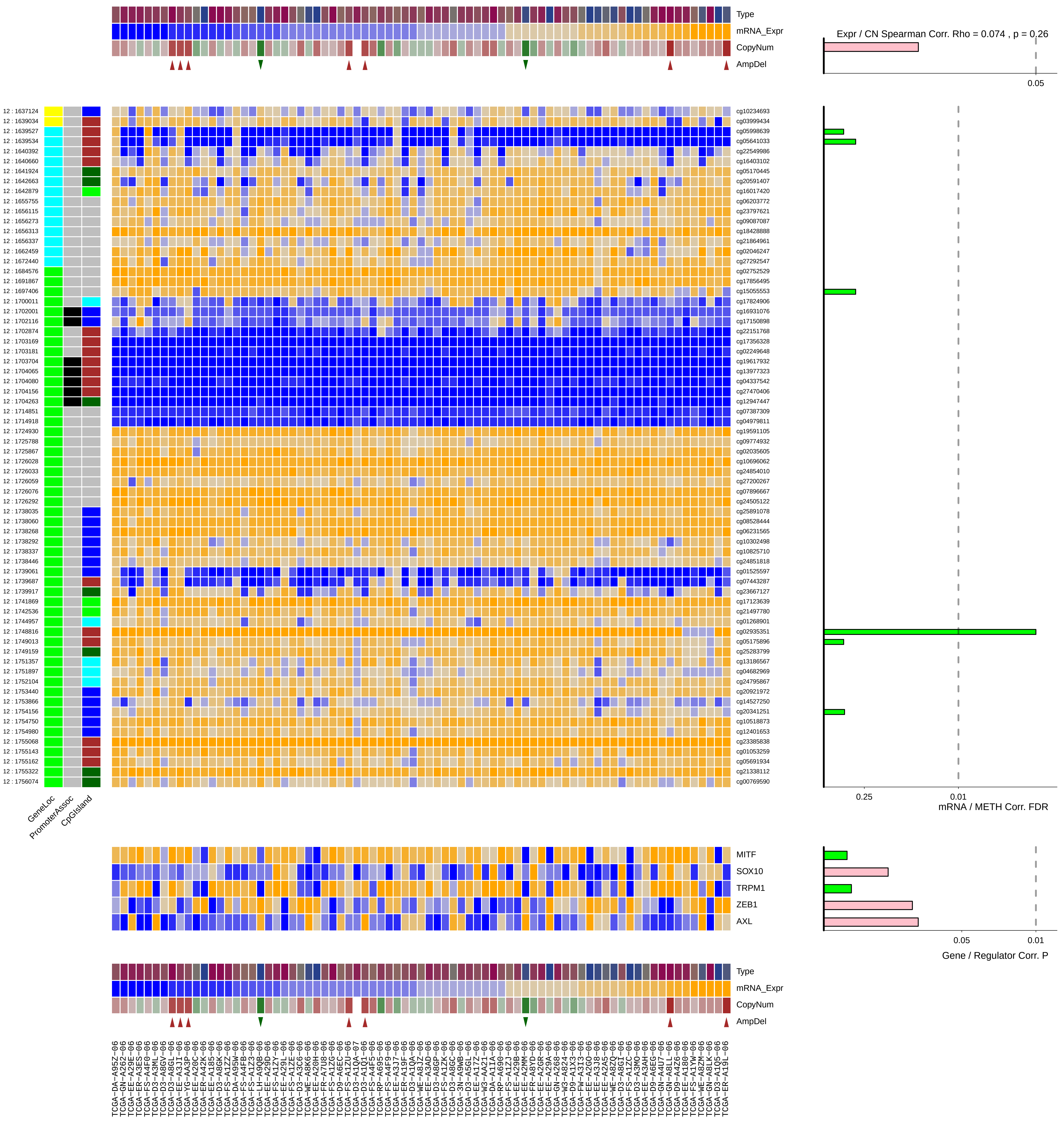
Type
mRNA_Expr
CopyNum
AmpDel
Expr / CN Spearman Corr. Rho = 0.074 , p = 0.26
0.05
GeneLoc
PromoterAssoc
CpGIsland
0.25
0.01
mRNA / METH Corr. FDR
MITF
SOX10
TRPM1
ZEB1
AXL
0.05
0.01
Gene / Regulator Corr. P

SLIT2

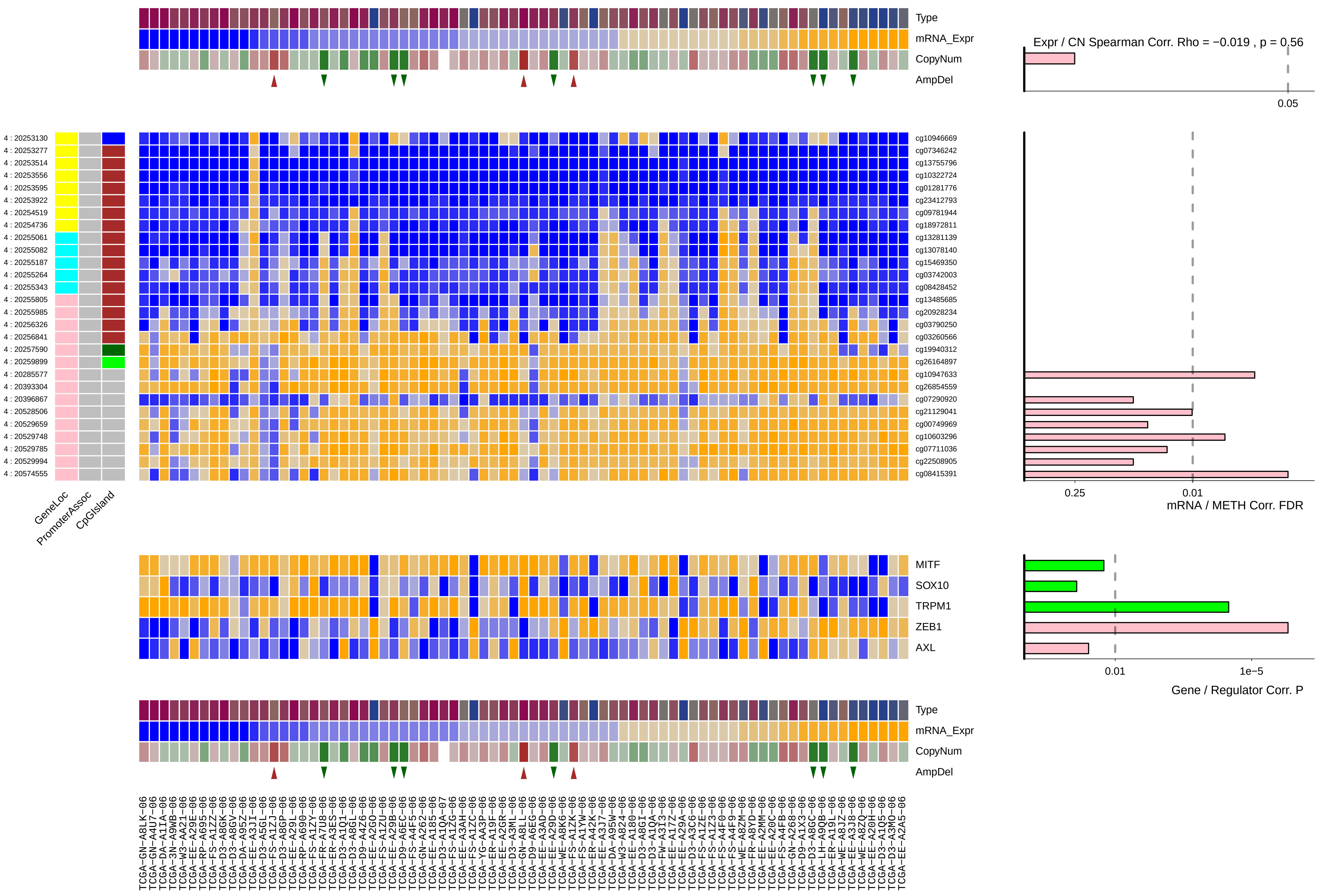

Type
mRNA_Expr
CopyNum
AmpDel
Expr / CN Spearman Corr. Rho = −0.019 , p = 0.56
0.05
GeneLoc
PromoterAssoc
CpGIsland
0.25
0.01
mRNA / METH Corr. FDR
MITF
SOX10
TRPM1
ZEB1
AXL
0.01
1e−5
Gene / Regulator Corr. P

LTBP2

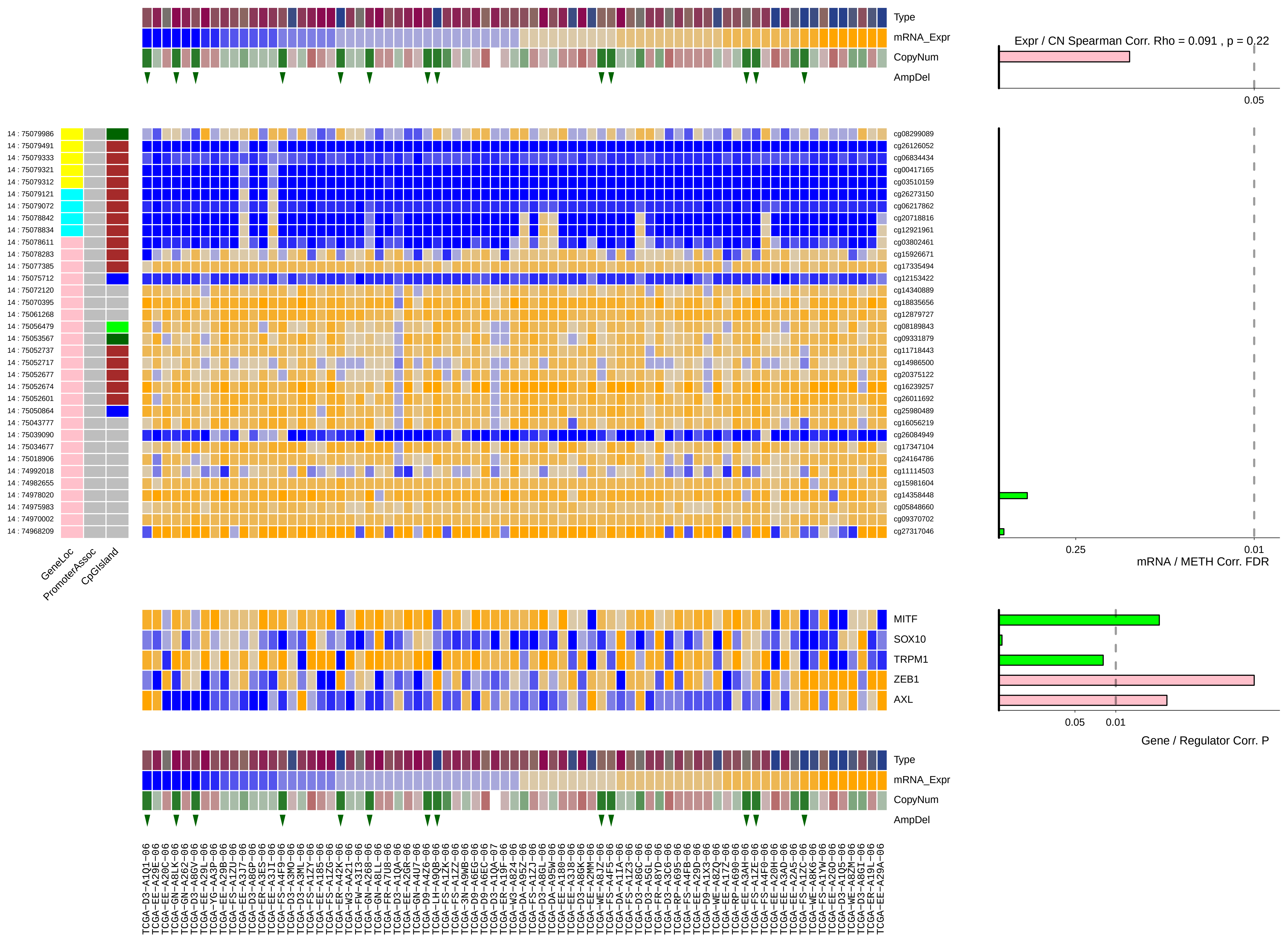
Type
mRNA_Expr
CopyNum
AmpDel
Expr / CN Spearman Corr. Rho = 0.091 , p = 0.22
0.05
14 : 75079986
14 : 75079491
14 : 75079333
14 : 75079321
14 : 75079312
14 : 75079121
14 : 75079072
14 : 75078842
14 : 75078834
14 : 75078611
14 : 75078283
14 : 75077385
14 : 75075712
14 : 75072120
14 : 75070395
14 : 75061268
14 : 75056479
14 : 75053567
14 : 75052737
14 : 75052717
14 : 75052677
14 : 75052674
14 : 75052601
14 : 75050864
14 : 75043777
14 : 75039090
14 : 75034677
14 : 75018906
14 : 74992018
14 : 74982655
14 : 74978020
14 : 74975983
14 : 74970002
14 : 74968209
cg08299089
cg26126052
cg06834434
cg00417165
cg03510159
cg26273150
cg06217862
cg20718816
cg12921961
cg03802461
cg15926671
cg17335494
cg12153422
cg14340889
cg18835656
cg12879727
cg08189843
cg09331879
cg11718443
cg14986500
cg20375122
cg16239257
cg26011692
cg25980489
cg16056219
cg26084949
cg17347104
cg24164786
cg11114503
cg15981604
cg14358448
cg05848660
cg09370702
cg27317046
GeneLoc
PromoterAssoc
CpGIsland
0.25
0.01
mRNA / METH Corr. FDR
MITF
SOX10
TRPM1
ZEB1
AXL
0.05
0.01
Gene / Regulator Corr. P
Type
mRNA_Expr
CopyNum
AmpDel

PLAU

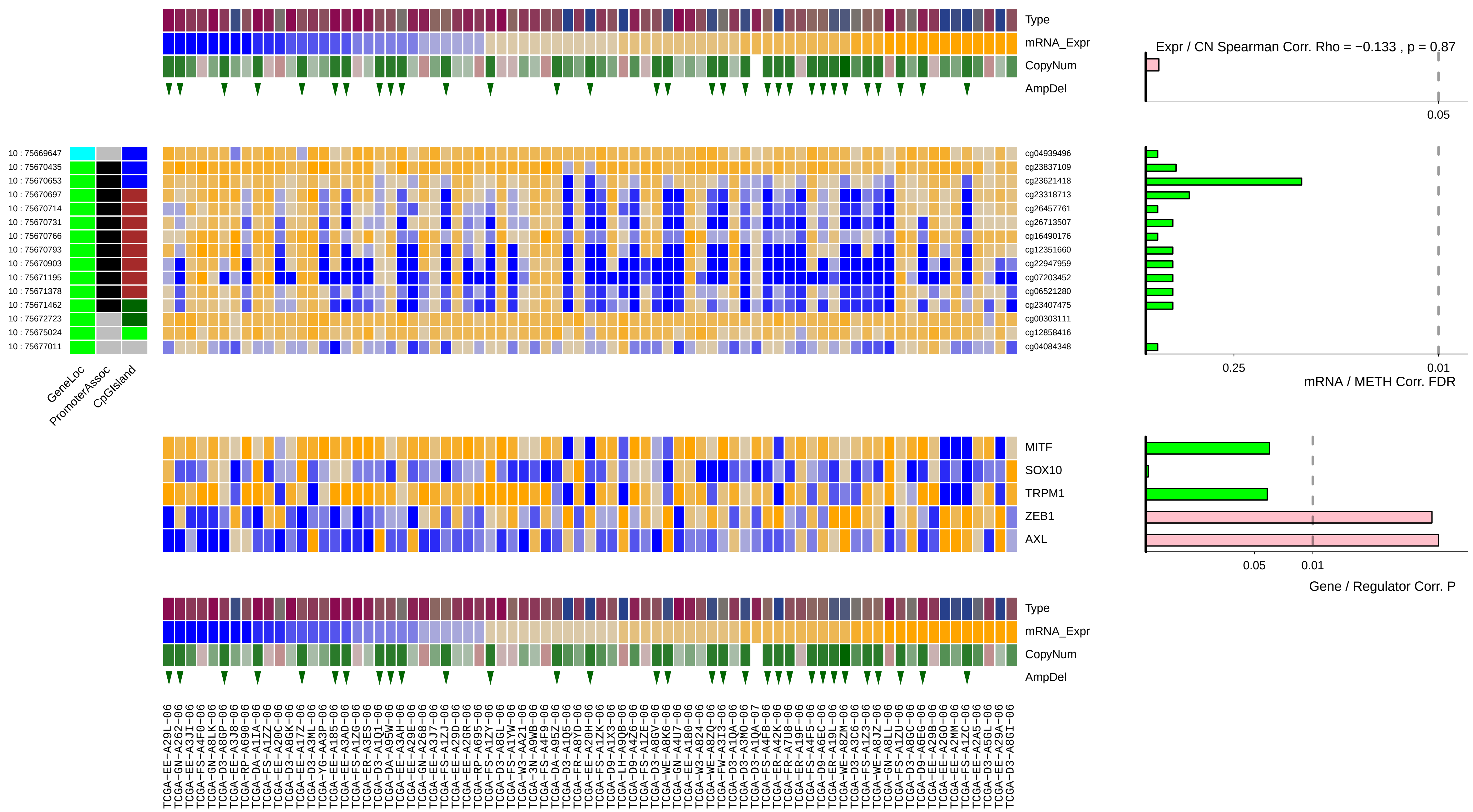

Type
mRNA_Expr
CopyNum
AmpDel
Expr / CN Spearman Corr. Rho = −0.133 , p = 0.87
0.05
mRNA / METH Corr. FDR
0.25
0.01
GeneLoc
PromoterAssoc
CpGIsland
MITF
SOX10
TRPM1
ZEB1
AXL
Gene / Regulator Corr. P

ADAM12

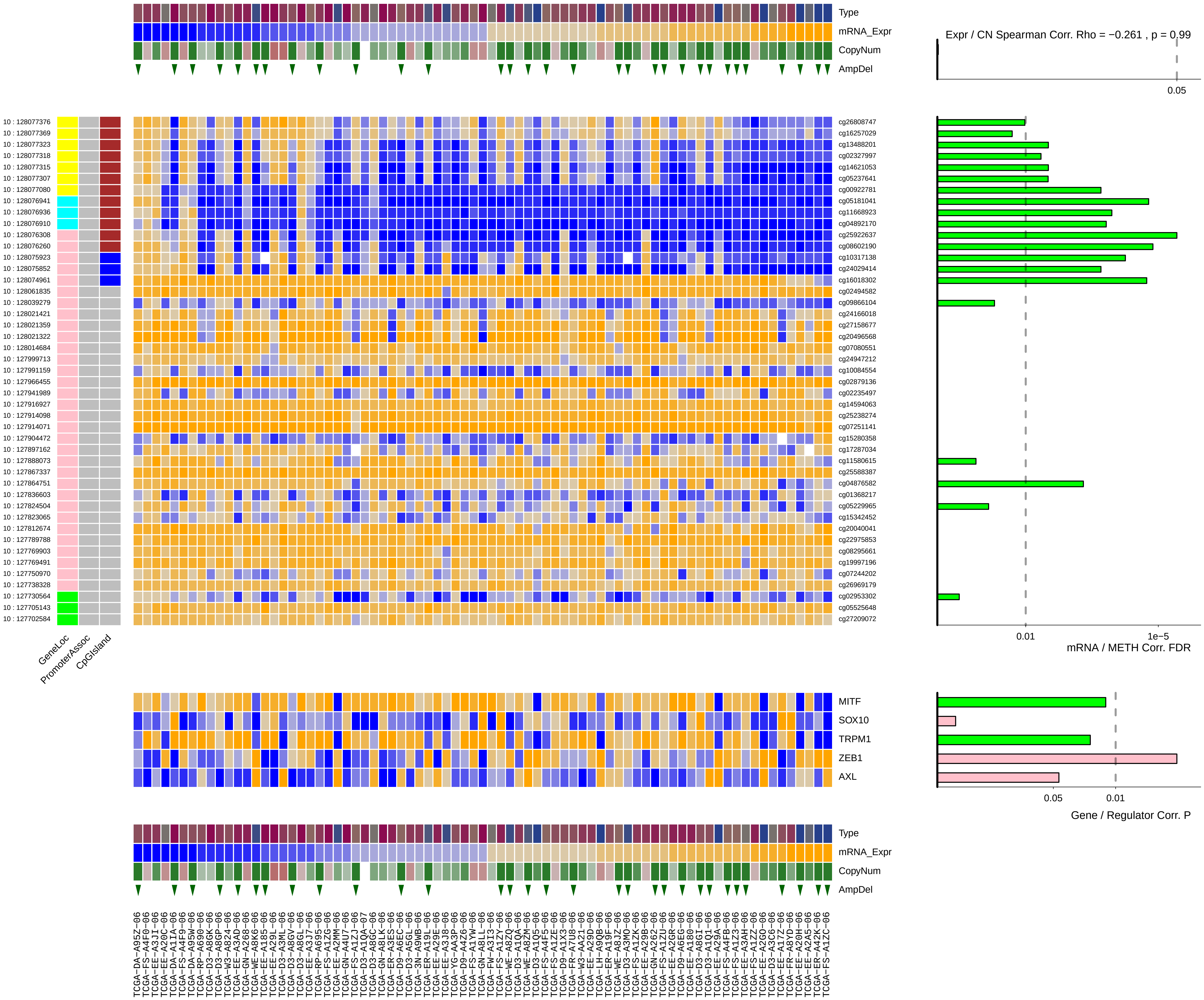
Type
mRNA_Expr
CopyNum
AmpDel
Expr / CN Spearman Corr. Rho = −0.261 , p = 0.99
0.05
GeneLoc
PromoterAssoc
CpGIsland
mRNA / METH Corr. FDR
0.01
1e−5
MITF
SOX10
TRPM1
ZEB1
AXL
Gene / Regulator Corr. P
0.05
0.01

SDC4

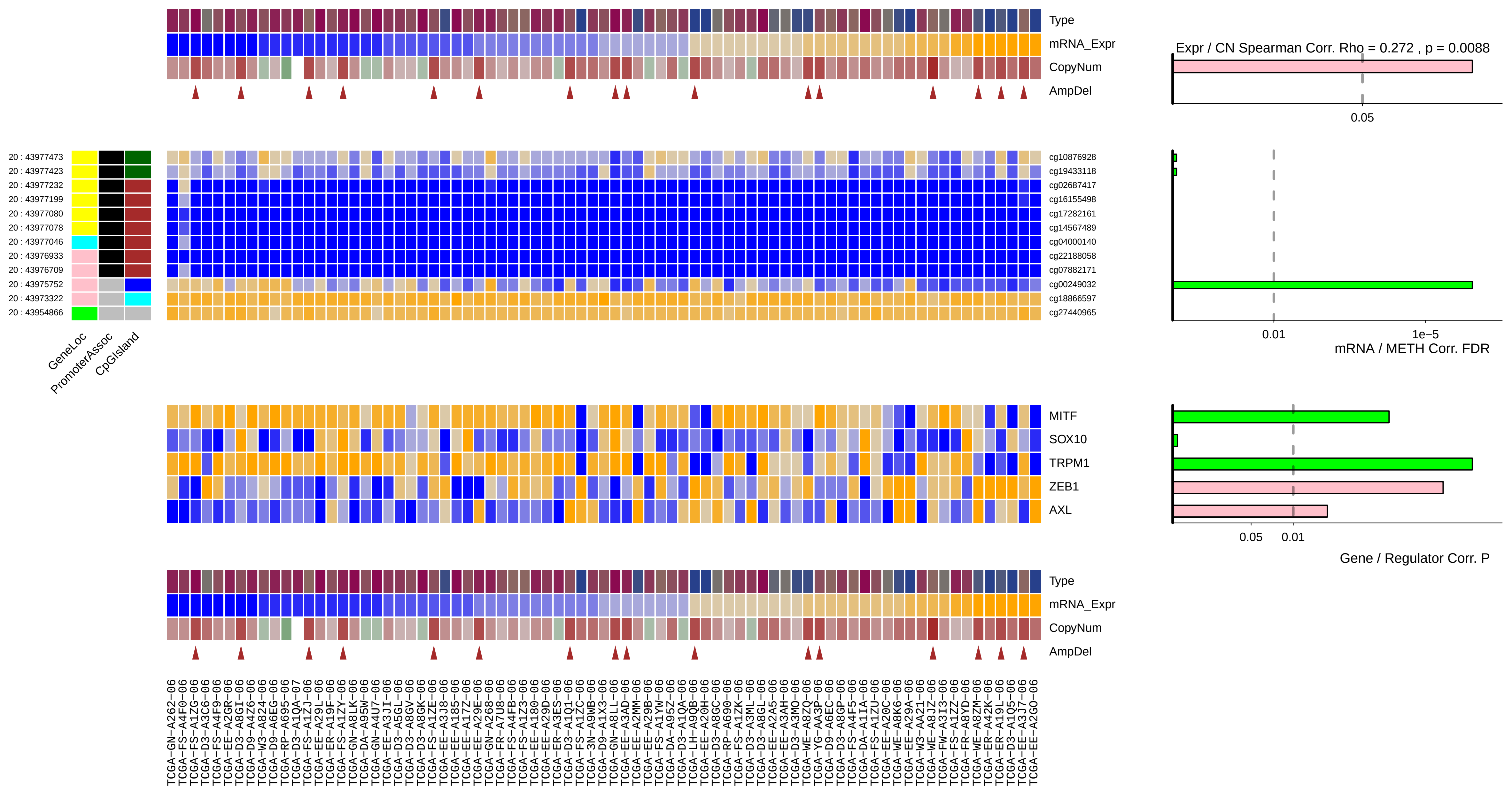

Type
mRNA_Expr
CopyNum
AmpDel
Expr / CN Spearman Corr. Rho = 0.272 , p = 0.0088
0.05
cg10876928
cg19433118
cg02687417
cg16155498
cg17282161
cg14567489
cg04000140
cg22188058
cg07882171
cg00249032
cg18866597
cg27440965
20 : 43977473
20 : 43977423
20 : 43977232
20 : 43977199
20 : 43977080
20 : 43977078
20 : 43977046
20 : 43976933
20 : 43976709
20 : 43975752
20 : 43973322
20 : 43954866
GeneLoc
PromoterAssoc
CpGIsland
0.01
1e−5
mRNA / METH Corr. FDR
MITF
SOX10
TRPM1
ZEB1
AXL
0.05
0.01
Gene / Regulator Corr. P

GLIPR1

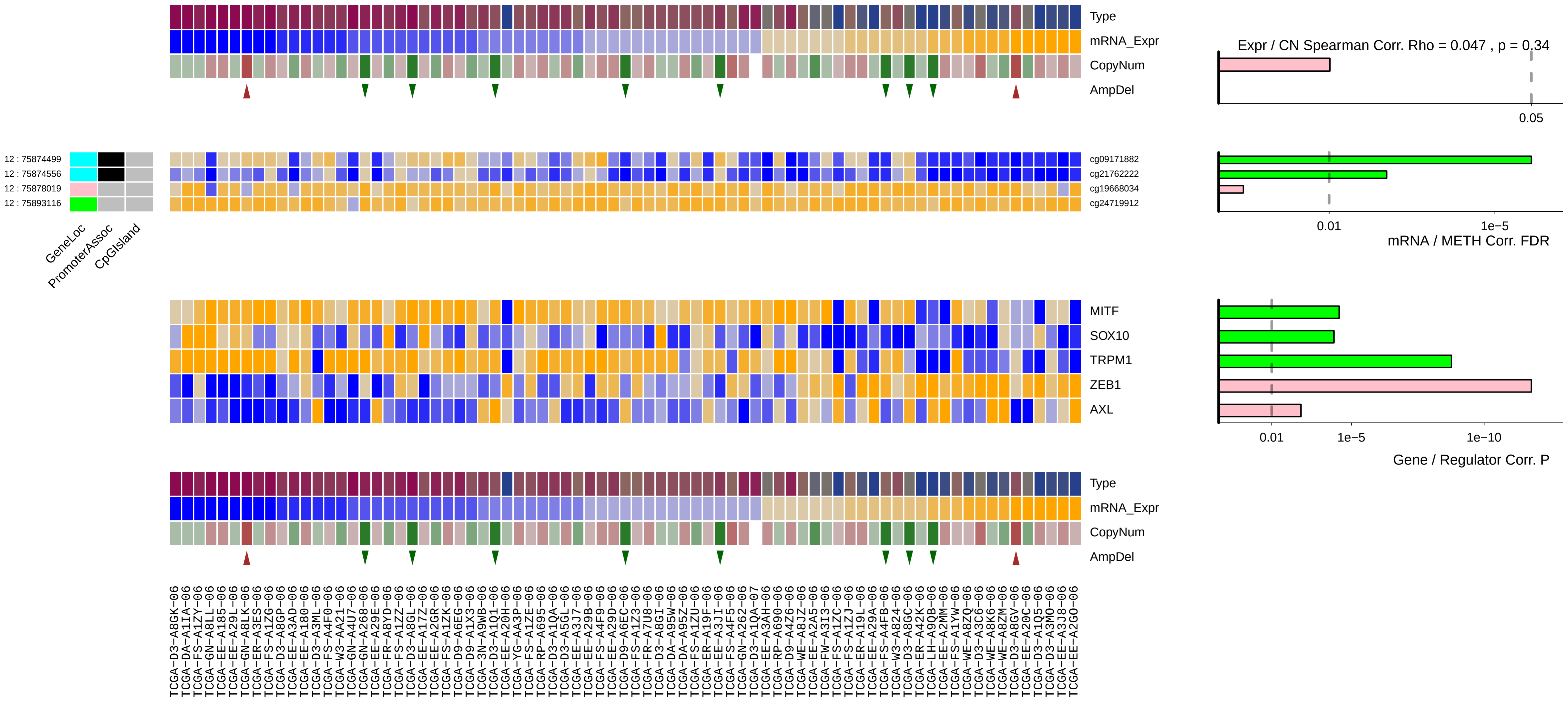

Type
mRNA_Expr
CopyNum
AmpDel
Expr / CN Spearman Corr. Rho = 0.047 , p = 0.34
0.05
12 : 75874499
12 : 75874556
12 : 75878019
12 : 75893116
GeneLoc
PromoterAssoc
CpGIsland
cg09171882
cg21762222
cg19668034
cg24719912
0.01
1e−5
mRNA / METH Corr. FDR
MITF
SOX10
TRPM1
ZEB1
AXL
0.01
1e−5
1e−10
Gene / Regulator Corr. P

NTN4

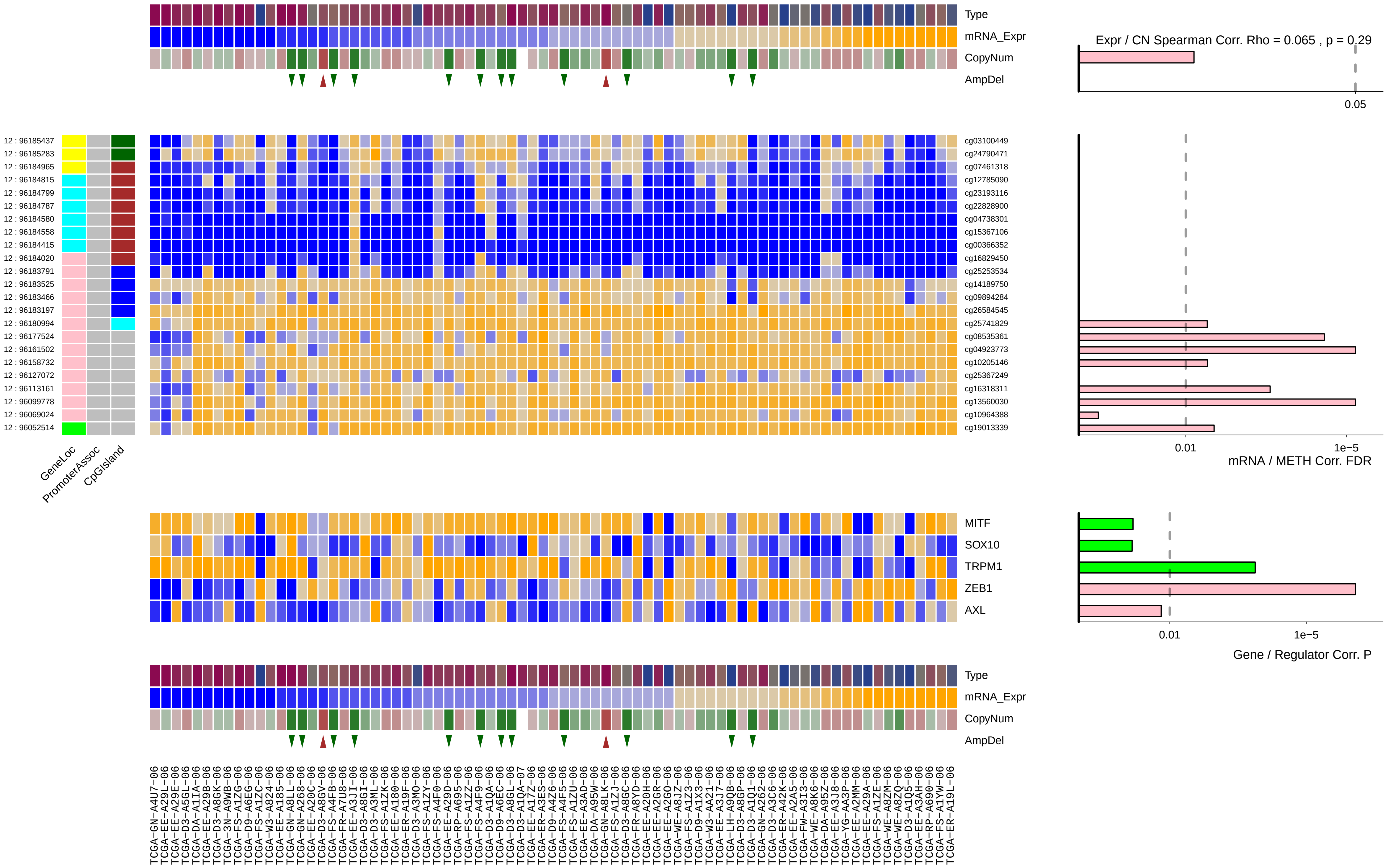

Type
mRNA_Expr
CopyNum
AmpDel
Expr / CN Spearman Corr. Rho = 0.065 , p = 0.29
0.05
GeneLoc
PromoterAssoc
CpGIsland
mRNA / METH Corr. FDR
0.01
1e−5
MITF
SOX10
TRPM1
ZEB1
AXL
Gene / Regulator Corr. P

TCF4

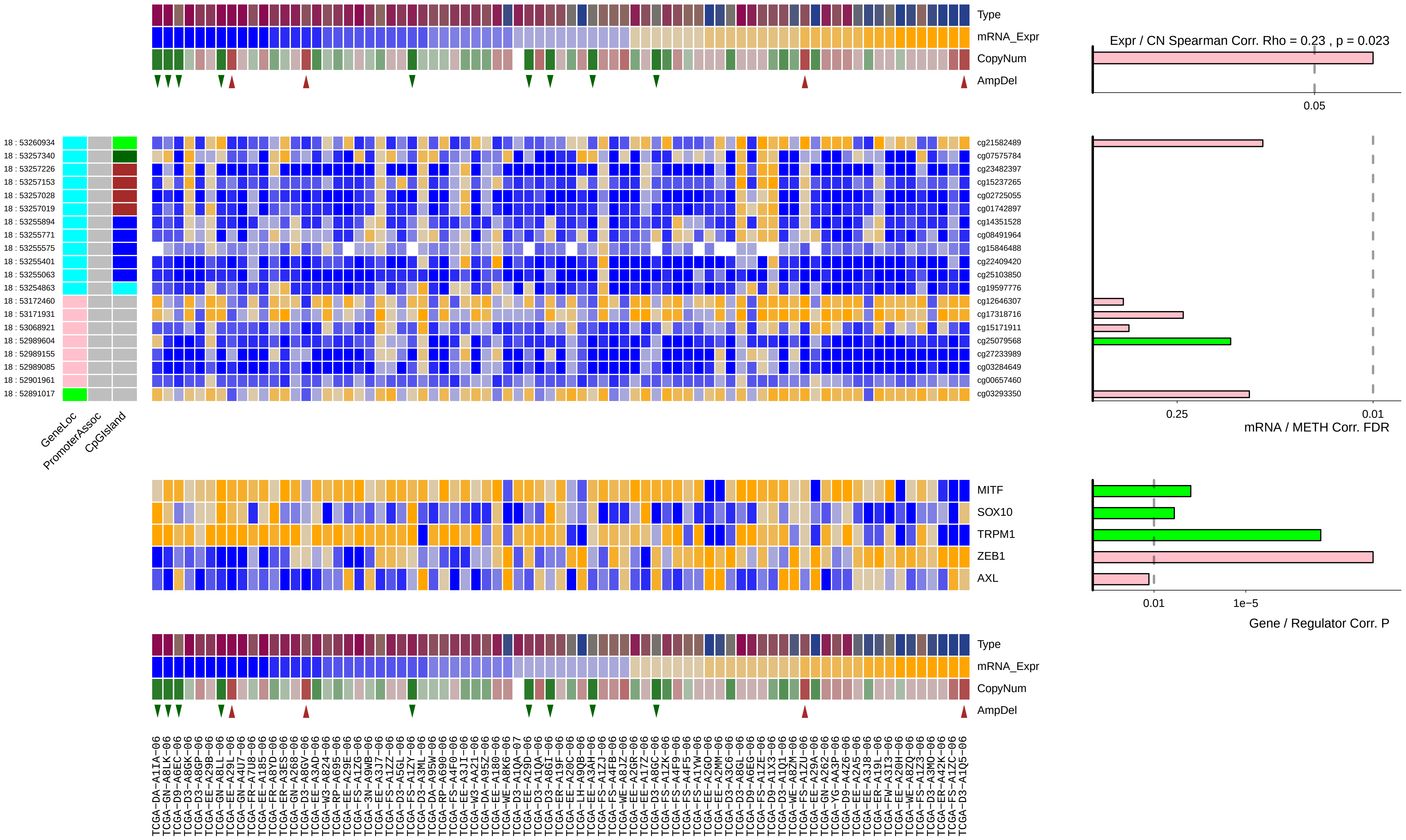

Type
mRNA_Expr
CopyNum
AmpDel
Expr / CN Spearman Corr. Rho = 0.23 , p = 0.023
0.05
18 : 53260934 cg21582489
18 : 53257340 cg07575784
18 : 53257226 cg23482397
18 : 53257153 cg15237265
18 : 53257028 cg02725055
18 : 53257019 cg01742897
18 : 53255894 cg14351528
18 : 53255771 cg08491964
18 : 53255575 cg15846488
18 : 53255401 cg22409420
18 : 53255063 cg25103850
18 : 53254863 cg19597776
18 : 53172460 cg12646307
18 : 53171931 cg17318716
18 : 53068921 cg15171911
18 : 52989604 cg25079568
18 : 52989155 cg27233989
18 : 52989085 cg03284649
18 : 52901961 cg00657460
18 : 52891017 cg03293350
GeneLoc
PromoterAssoc
CpGIsland
0.25
0.01
mRNA / METH Corr. FDR
MITF
SOX10
TRPM1
ZEB1
AXL
0.01
1e−5
Gene / Regulator Corr. P

EPHA2

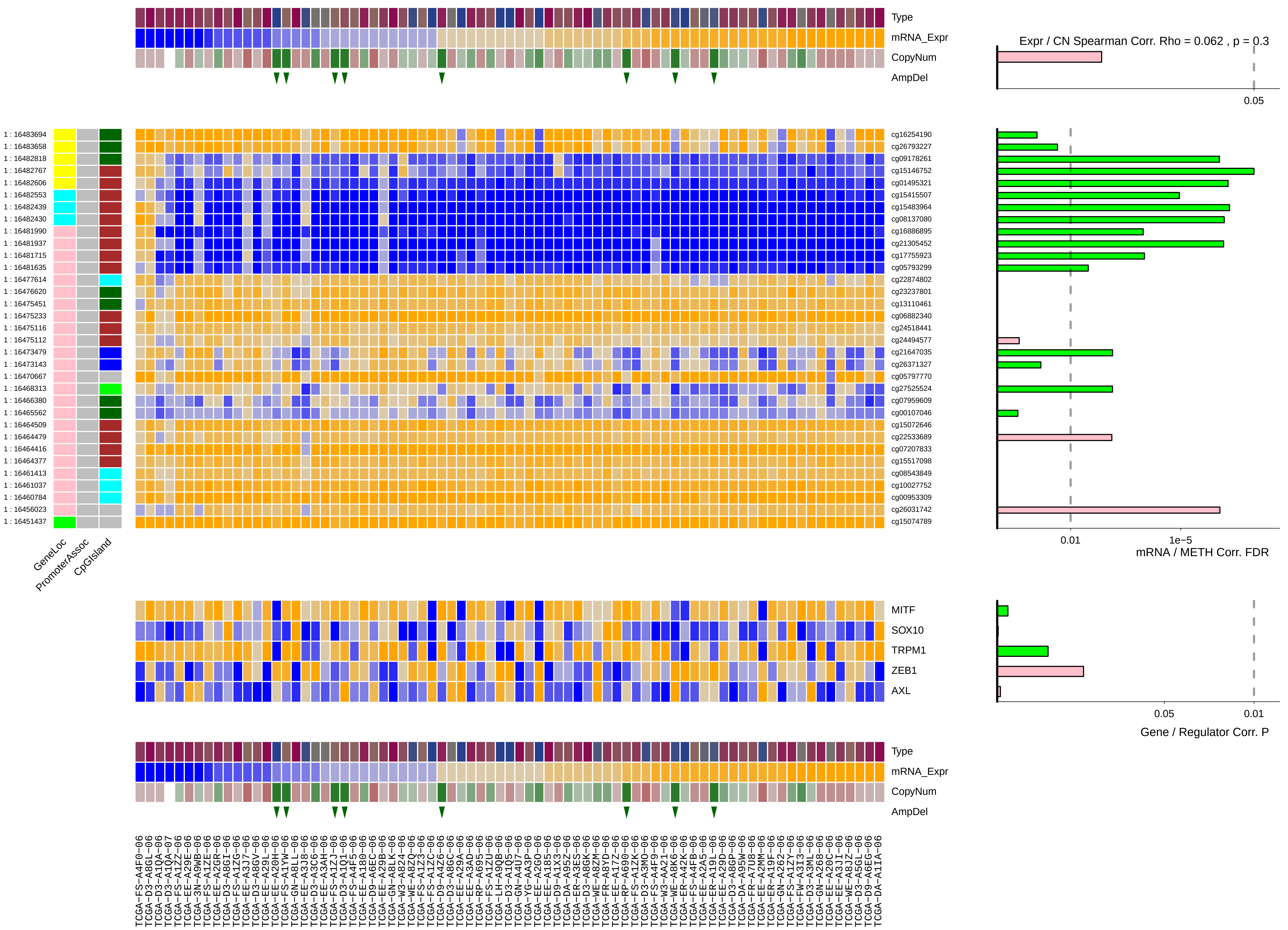

Type
mRNA_Expr
CopyNum
AmpDel
Expr / CN Spearman Corr. Rho = 0.062 , p = 0.3
0.05
mRNA / METH Corr. FDR
0.01
1e−5
GeneLoc
PromoterAssoc
CpGIsland
MITF
SOX10
TRPM1
ZEB1
AXL
Gene / Regulator Corr. P

GBP3

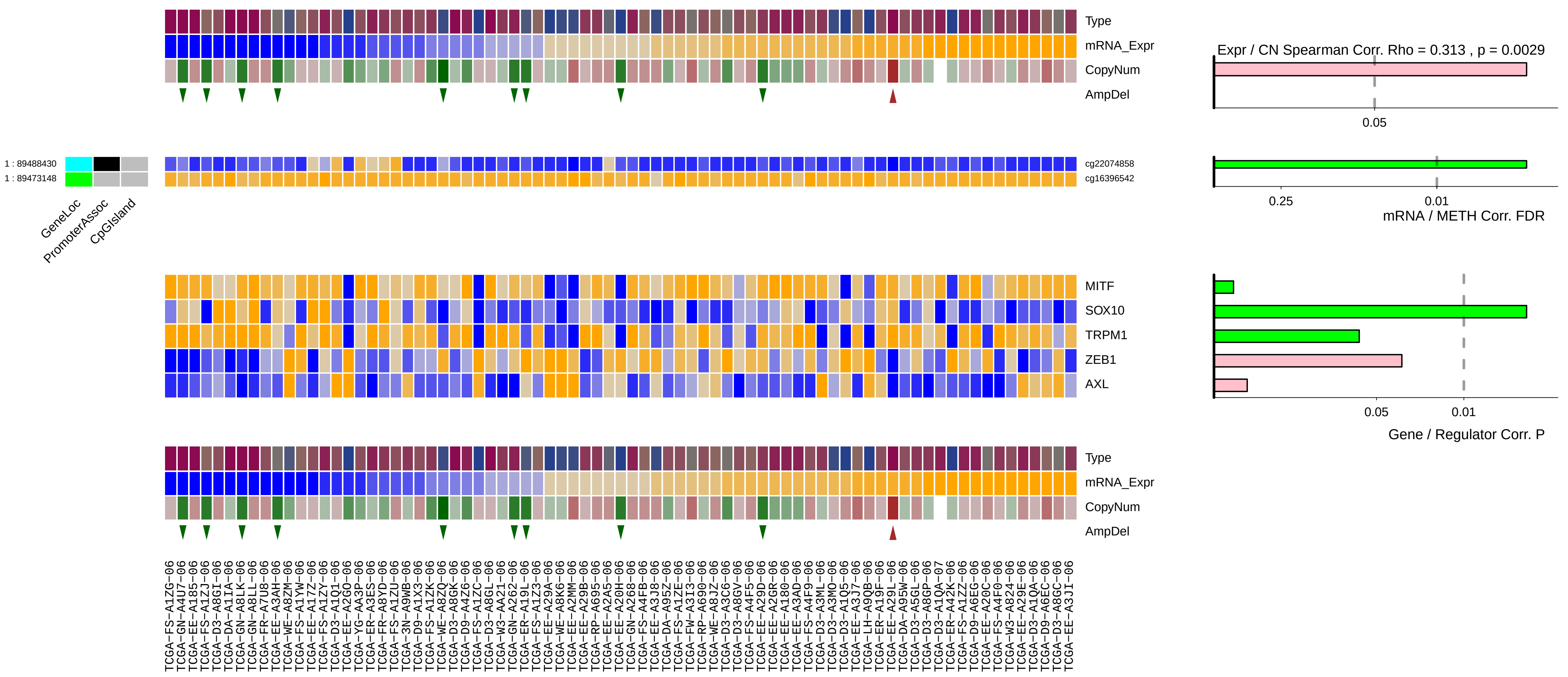

Type
mRNA_Expr
CopyNum
AmpDel
Expr / CN Spearman Corr. Rho = 0.313 , p = 0.0029
0.05
1 : 89488430
1 : 89473148
GeneLoc
PromoterAssoc
CpGIsland
cg22074858
cg16396542
0.25
0.01
mRNA / METH Corr. FDR
MITF
SOX10
TRPM1
ZEB1
AXL
0.05
0.01
Gene / Regulator Corr. P
Type
mRNA_Expr
CopyNum
AmpDel

COL6A1

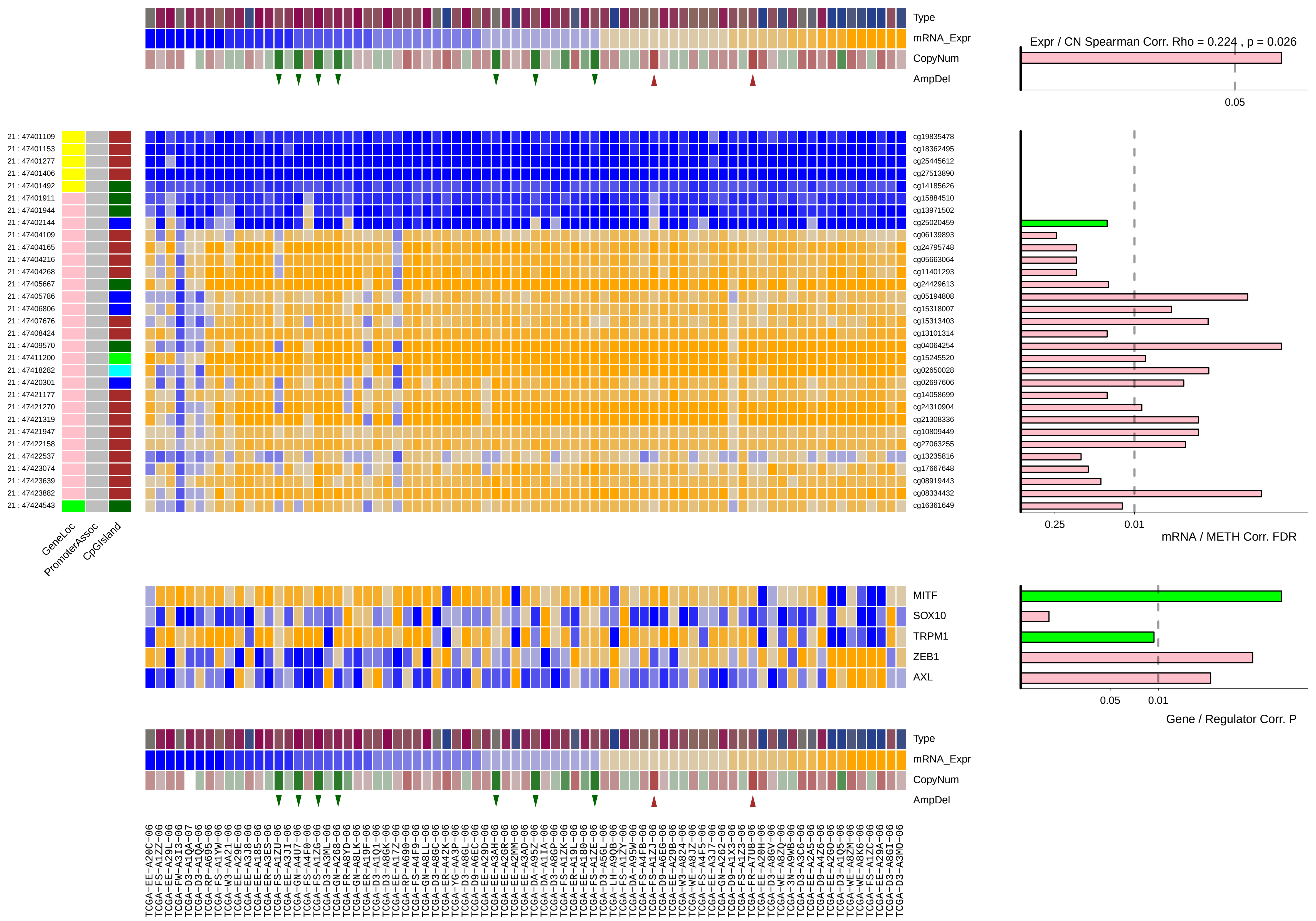
Type
mRNA_Expr
CopyNum
AmpDel
Expr / CN Spearman Corr. Rho = 0.224 , p = 0.026
0.05
GeneLoc
PromoterAssoc
CpGIsland
mRNA / METH Corr. FDR
0.25
0.01
MITF
SOX10
TRPM1
ZEB1
AXL
Gene / Regulator Corr. P

ZEB1

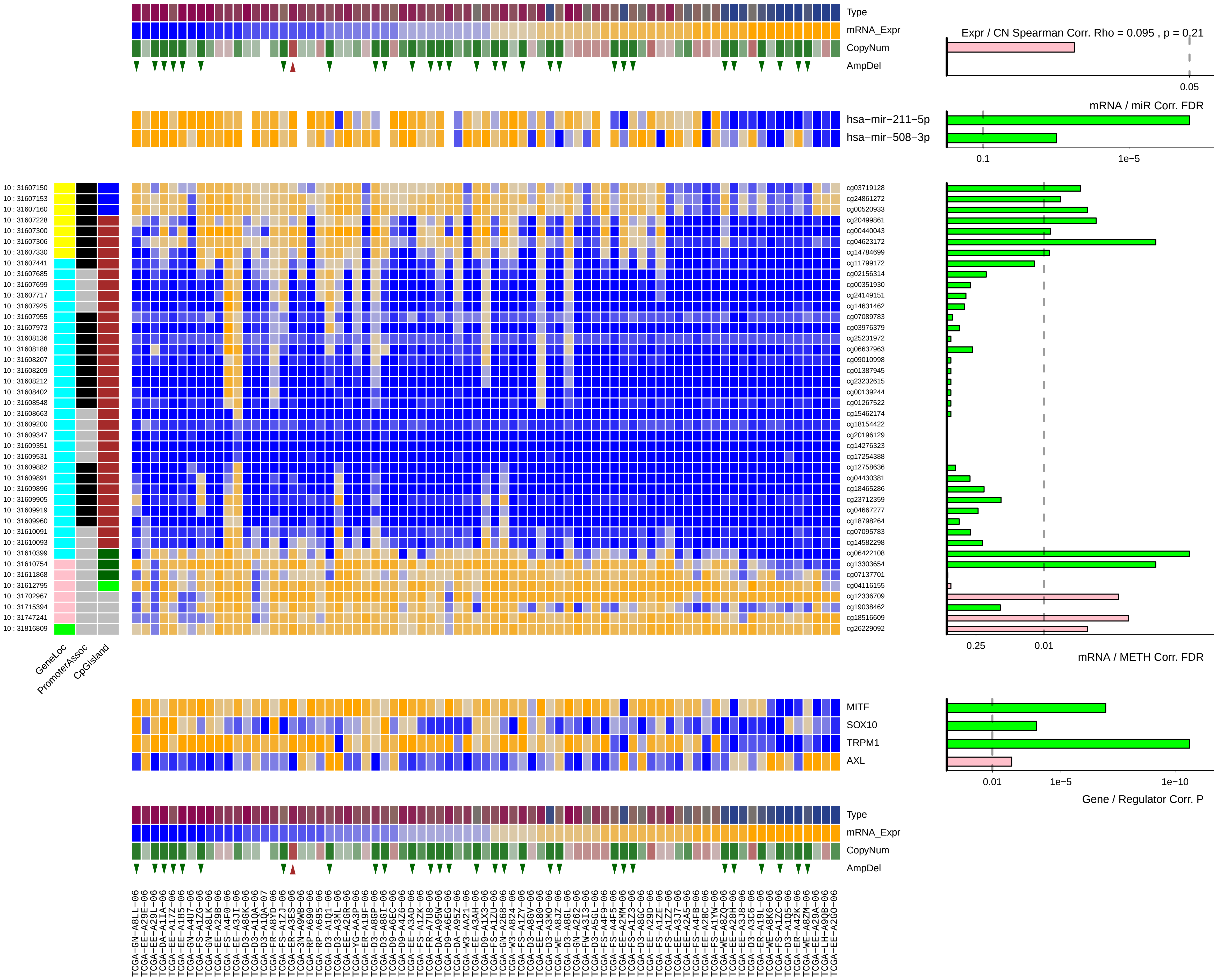
Type
mRNA_Expr
CopyNum
AmpDel
Expr / CN Spearman Corr. Rho = 0.095 , p = 0.21
0.05
hsa−mir−211−5p
hsa−mir−508−3p
mRNA / miR Corr. FDR
0.1
1e−5
GeneLoc
PromoterAssoc
CpGIsland
0.25
0.01
mRNA / METH Corr. FDR
MITF
SOX10
TRPM1
AXL
0.01
1e−5
1e−10
Gene / Regulator Corr. P

CYR61

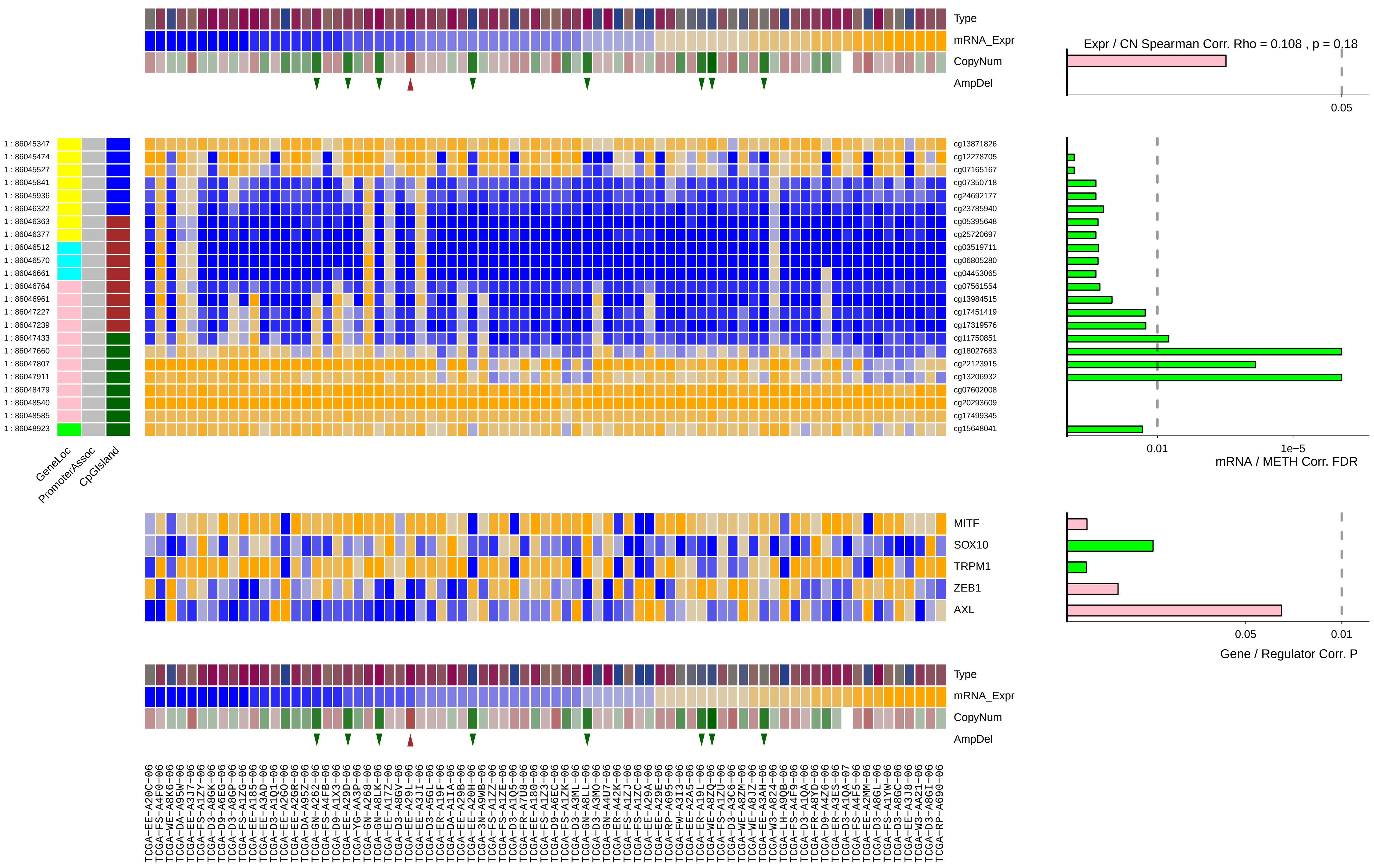

Type
mRNA_Expr
CopyNum
AmpDel
Expr / CN Spearman Corr. Rho = 0.108 , p = 0.18
0.05
GeneLoc
PromoterAssoc
CpGIsland
0.01
1e−5
mRNA / METH Corr. FDR
MITF
SOX10
TRPM1
ZEB1
AXL
0.05
0.01
Gene / Regulator Corr. P

LIFR

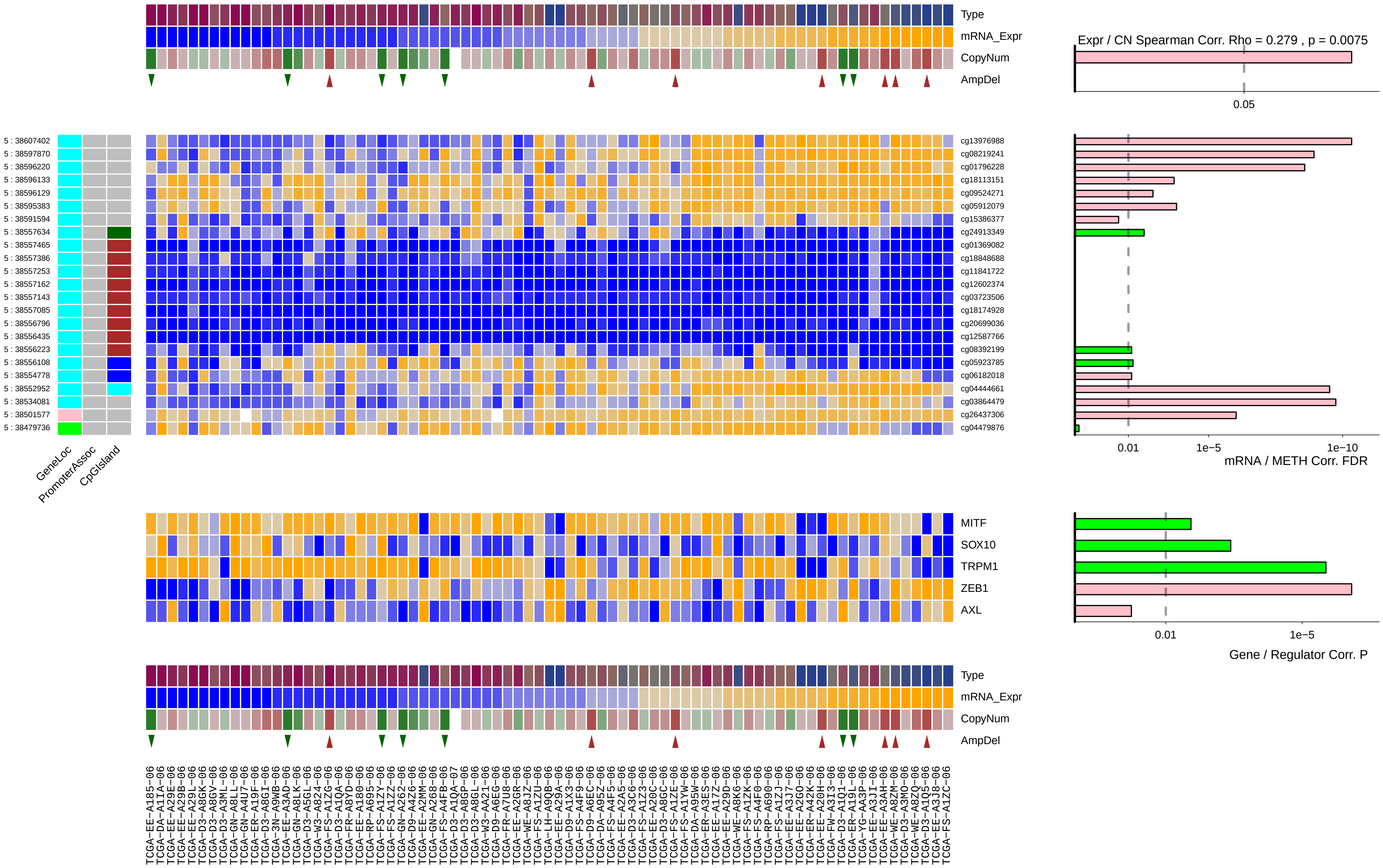
Type
mRNA_Expr
CopyNum
AmpDel
Expr / CN Spearman Corr. Rho = 0.279 , p = 0.0075
0.05
5 : 38607402
5 : 38597870
5 : 38596220
5 : 38596133
5 : 38596129
5 : 38595383
5 : 38591594
5 : 38557634
5 : 38557465
5 : 38557386
5 : 38557253
5 : 38557162
5 : 38557143
5 : 38557085
5 : 38556796
5 : 38556435
5 : 38556223
5 : 38556108
5 : 38554778
5 : 38552952
5 : 38534081
5 : 38501577
5 : 38479736
GeneLoc
PromoterAssoc
CpGIsland
cg13976988
cg08219241
cg01796228
cg18113151
cg09524271
cg05912079
cg15386377
cg24913349
cg01369082
cg18848688
cg11841722
cg12602374
cg03723506
cg18174928
cg20699036
cg12587766
cg08392199
cg05923785
cg06182018
cg04444661
cg03864479
cg26437306
cg04479876
0.01
1e−5
1e−10
mRNA / METH Corr. FDR
MITF
SOX10
TRPM1
ZEB1
AXL
0.01
1e−5
Gene / Regulator Corr. P
Type
mRNA_Expr
CopyNum
AmpDel

KRT18

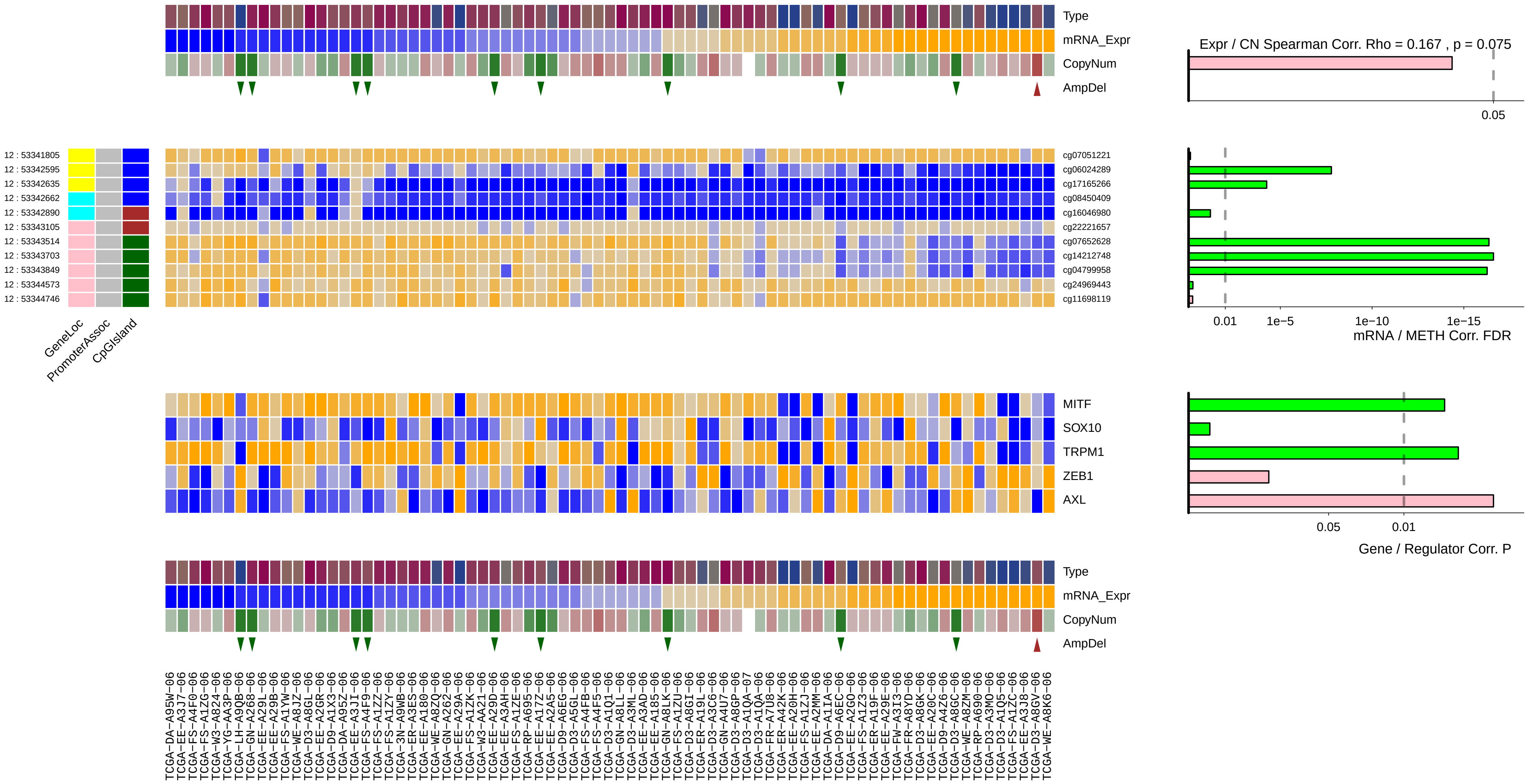

Type
mRNA_Expr
CopyNum
AmpDel
Expr / CN Spearman Corr. Rho = 0.167 , p = 0.075
0.05
12 : 53341805
12 : 53342595
12 : 53342635
12 : 53342662
12 : 53342890
12 : 53343105
12 : 53343514
12 : 53343703
12 : 53343849
12 : 53344573
12 : 53344746
cg07051221
cg06024289
cg17165266
cg08450409
cg16046980
cg22221657
cg07652628
cg14212748
cg04799958
cg24969443
cg11698119
0.01
1e−5
1e−10
1e−15
mRNA / METH Corr. FDR
GeneLoc
PromoterAssoc
CpGIsland
MITF
SOX10
TRPM1
ZEB1
AXL
0.05
0.01
Gene / Regulator Corr. P
Type
mRNA_Expr
CopyNum
AmpDel

CCBE1

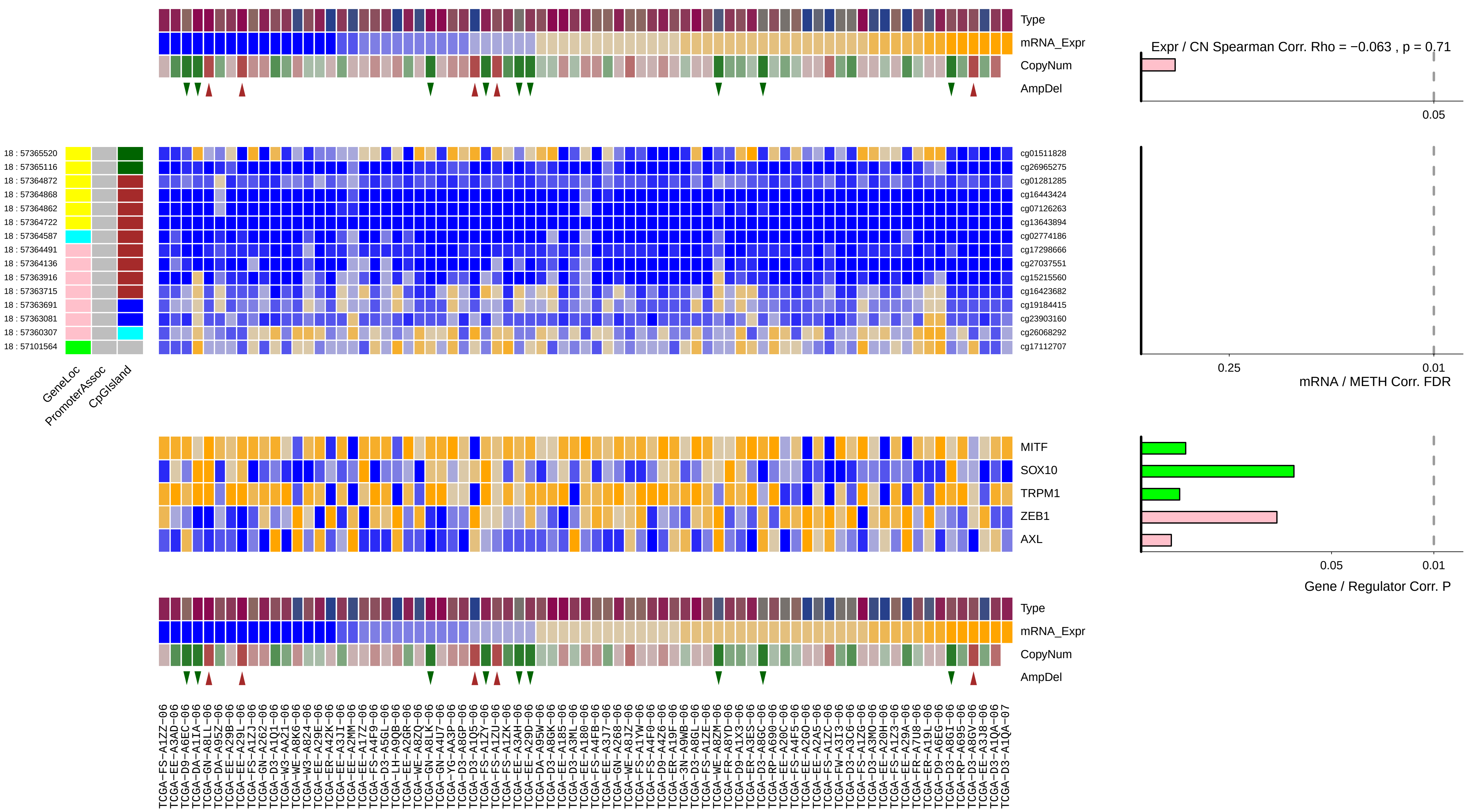

Type
mRNA_Expr
CopyNum
AmpDel
Expr / CN Spearman Corr. Rho = −0.063 , p = 0.71
0.05
18 : 57365520
18 : 57365116
18 : 57364872
18 : 57364868
18 : 57364862
18 : 57364722
18 : 57364587
18 : 57364491
18 : 57364136
18 : 57363916
18 : 57363715
18 : 57363691
18 : 57363081
18 : 57360307
18 : 57101564
cg01511828
cg26965275
cg01281285
cg16443424
cg07126263
cg13643894
cg02774186
cg17298666
cg27037551
cg15215560
cg16423682
cg19184415
cg23903160
cg26068292
cg17112707
GeneLoc
PromoterAssoc
CpGIsland
0.25
0.01
mRNA / METH Corr. FDR
MITF
SOX10
TRPM1
ZEB1
AXL
0.05
0.01
Gene / Regulator Corr. P
Type
mRNA_Expr
CopyNum
AmpDel

RND3

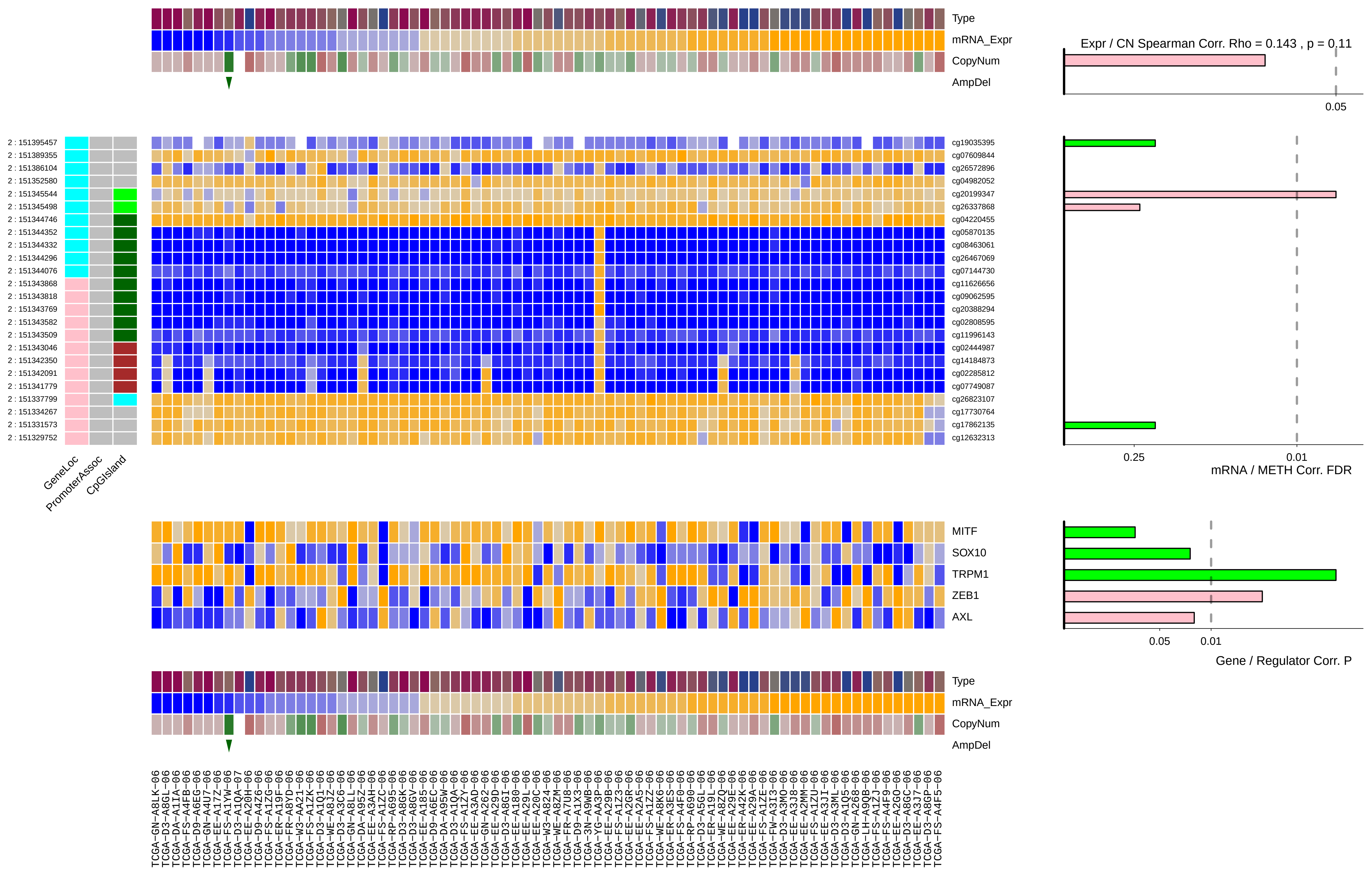

Type
mRNA_Expr
CopyNum
AmpDel
Expr / CN Spearman Corr. Rho = 0.143 , p = 0.11
0.05
2 : 151395457
2 : 151389355
2 : 151386104
2 : 151352580
2 : 151345544
2 : 151345498
2 : 151344746
2 : 151344352
2 : 151344332
2 : 151344296
2 : 151344076
2 : 151343868
2 : 151343818
2 : 151343769
2 : 151343582
2 : 151343509
2 : 151343046
2 : 151342350
2 : 151342091
2 : 151341779
2 : 151337799
2 : 151334267
2 : 151331573
2 : 151329752
GeneLoc
PromoterAssoc
CpGIsland
cg19035395
cg07609844
cg26572896
cg04982052
cg20199347
cg26337868
cg04220455
cg05870135
cg08463061
cg26467069
cg07144730
cg11626656
cg09062595
cg20388294
cg02808595
cg11996143
cg02444987
cg14184873
cg02285812
cg07749087
cg26823107
cg17730764
cg17862135
cg12632313
0.25
0.01
mRNA / METH Corr. FDR
MITF
SOX10
TRPM1
ZEB1
AXL
0.05
0.01
Gene / Regulator Corr. P
Type
mRNA_Expr
CopyNum
AmpDel
TCGA-GN-A8LK-06
TCGA-D3-A8GL-06
TCGA-DA-A1IA-06
TCGA-FS-A4FB-06
TCGA-D9-A6EG-06
TCGA-GN-A4U7-06
TCGA-EE-A17Z-06
TCGA-FS-A1YW-06
TCGA-D3-A1QA-07
TCGA-EE-A20H-06
TCGA-D9-A4Z6-06
TCGA-FS-A1ZG-06
TCGA-ER-A19F-06
TCGA-FR-A8YD-06
TCGA-W3-AA21-06
TCGA-FS-A1ZK-06
TCGA-D3-A1Q1-06
TCGA-WE-A8JZ-06
TCGA-D3-A3C6-06
TCGA-GN-A8LL-06
TCGA-DA-A95Z-06
TCGA-EE-A3AH-06
TCGA-FS-A1ZC-06
TCGA-RP-A695-06
TCGA-D3-A8GK-06
TCGA-D3-A8GV-06
TCGA-EE-A185-06
TCGA-D9-A6EC-06
TCGA-DA-A95W-06
TCGA-D3-A1QA-06
TCGA-FS-A1ZY-06
TCGA-EE-A3AD-06
TCGA-GN-A262-06
TCGA-EE-A29D-06
TCGA-D3-A8GI-06
TCGA-EE-A180-06
TCGA-EE-A29L-06
TCGA-EE-A20C-06
TCGA-W3-A824-06
TCGA-WE-A8ZM-06
TCGA-FR-A7U8-06
TCGA-D9-A1X3-06
TCGA-3N-A9WB-06
TCGA-YG-AA3P-06
TCGA-EE-A29B-06
TCGA-FS-A1Z3-06
TCGA-EE-A2GR-06
TCGA-EE-A2A5-06
TCGA-FS-A1ZZ-06
TCGA-WE-A8K6-06
TCGA-ER-A3ES-06
TCGA-FS-A4F0-06
TCGA-RP-A690-06
TCGA-D3-A5GL-06
TCGA-ER-A19L-06
TCGA-WE-A8ZQ-06
TCGA-EE-A29E-06
TCGA-ER-A42K-06
TCGA-EE-A29A-06
TCGA-FS-A1ZE-06
TCGA-FW-A3I3-06
TCGA-D3-A3MO-06
TCGA-EE-A3J8-06
TCGA-EE-A2MM-06
TCGA-FS-A1ZU-06
TCGA-EE-A3JI-06
TCGA-D3-A3ML-06
TCGA-D3-A1Q5-06
TCGA-GN-A268-06
TCGA-LH-A9QB-06
TCGA-FS-A1ZJ-06
TCGA-FS-A4F9-06
TCGA-EE-A2GO-06
TCGA-D3-A8GC-06
TCGA-EE-A3J7-06
TCGA-D3-A8GP-06
TCGA-FS-A4F5-06

CCL2

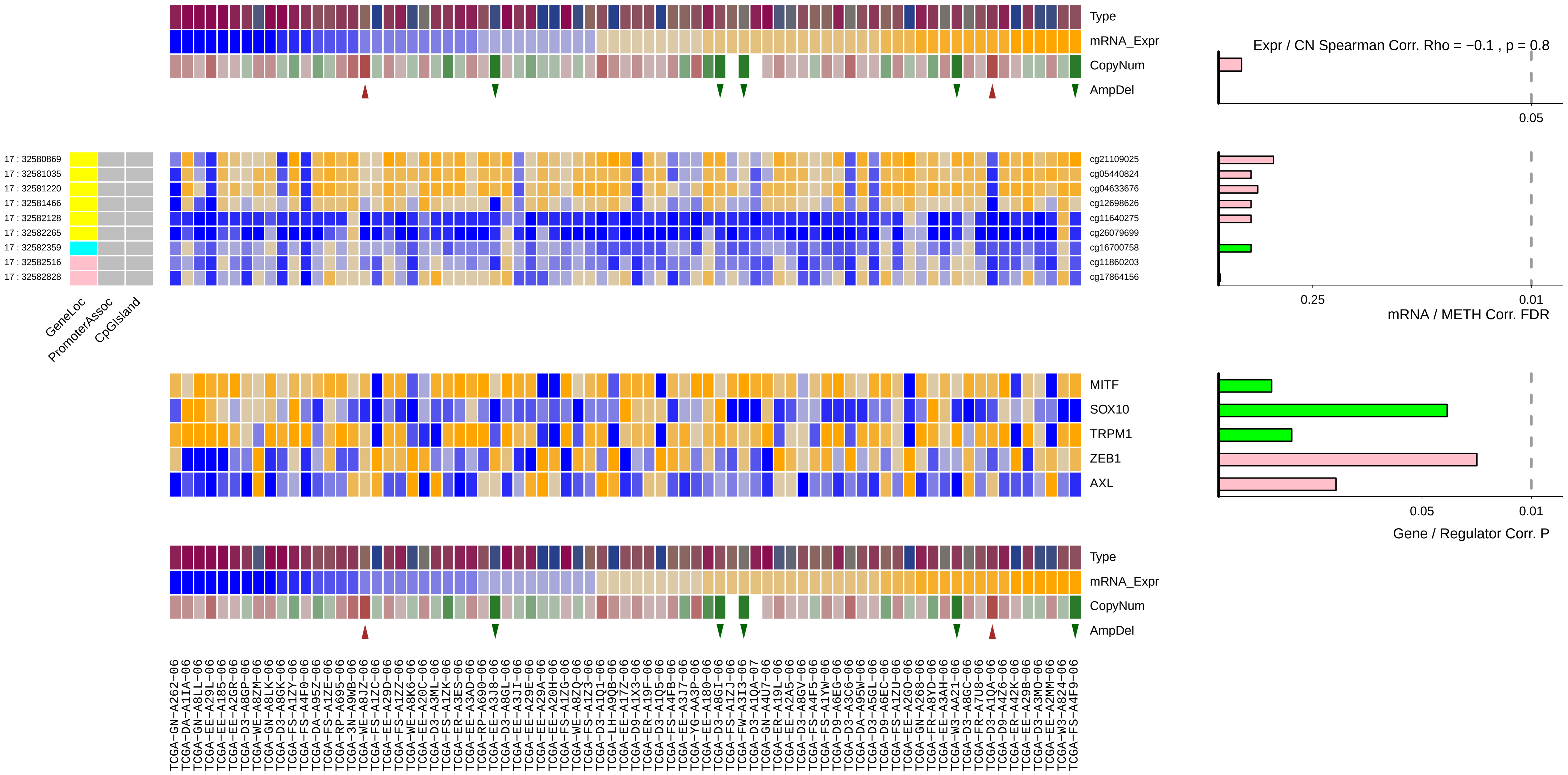

Type
mRNA_Expr
CopyNum
AmpDel
Expr / CN Spearman Corr. Rho = −0.1 , p = 0.8
0.05
17 : 32580869
17 : 32581035
17 : 32581220
17 : 32581466
17 : 32582128
17 : 32582265
17 : 32582359
17 : 32582516
17 : 32582828
cg21109025
cg05440824
cg04633676
cg12698626
cg11640275
cg26079699
cg16700758
cg11860203
cg17864156
GeneLoc
PromoterAssoc
CpGIsland
0.25
0.01
mRNA / METH Corr. FDR
MITF
SOX10
TRPM1
ZEB1
AXL
0.05
0.01
Gene / Regulator Corr. P

OSMR

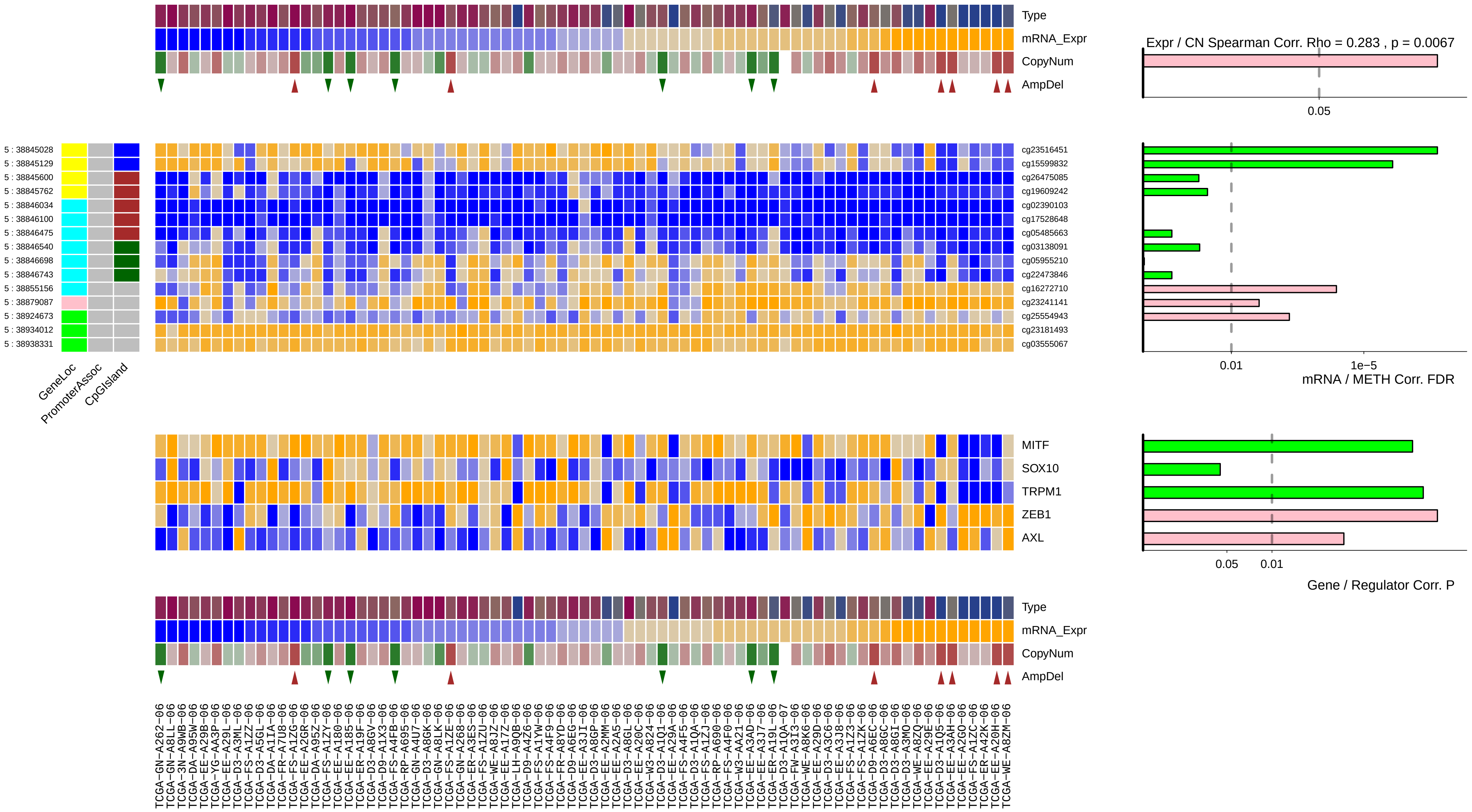
Type
mRNA_Expr
CopyNum
AmpDel
Expr / CN Spearman Corr. Rho = 0.283 , p = 0.0067
0.05
GeneLoc
PromoterAssoc
CpGIsland
5 : 38845028
5 : 38845129
5 : 38845600
5 : 38845762
5 : 38846034
5 : 38846100
5 : 38846475
5 : 38846540
5 : 38846698
5 : 38846743
5 : 38855156
5 : 38879087
5 : 38924673
5 : 38934012
5 : 38938331
cg23516451
cg15599832
cg26475085
cg19609242
cg02390103
cg17528648
cg05485663
cg03138091
cg05955210
cg22473846
cg16272710
cg23241141
cg25554943
cg23181493
cg03555067
0.01
1e−5
mRNA / METH Corr. FDR
MITF
SOX10
TRPM1
ZEB1
AXL
0.05
0.01
Gene / Regulator Corr. P

EPHB2

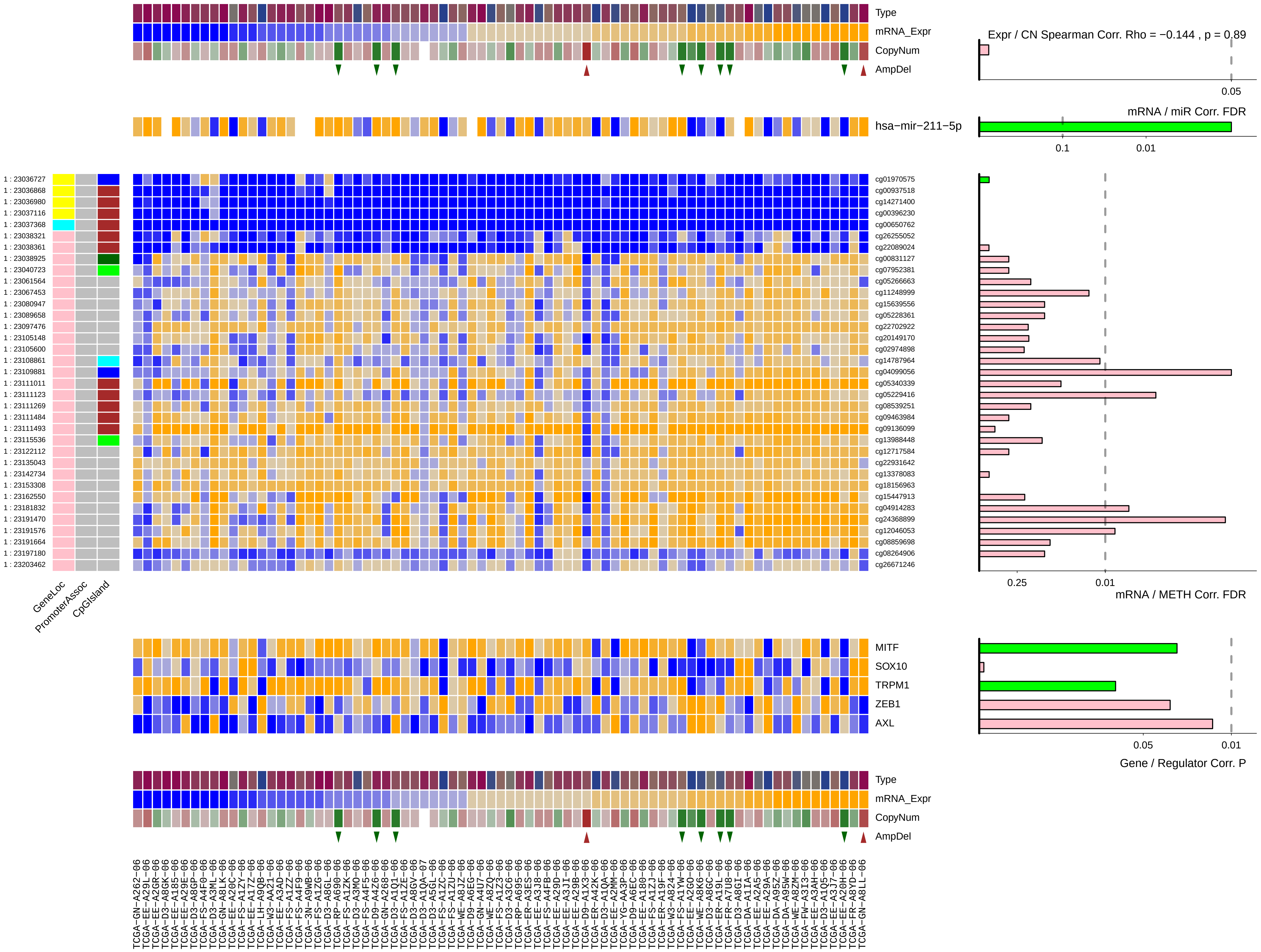

Type
mRNA_Expr
CopyNum
AmpDel
Expr / CN Spearman Corr. Rho = −0.144 , p = 0.89
0.05
hsa−mir−211−5p
mRNA / miR Corr. FDR
0.1
0.01
cg01970575
cg00937518
cg14271400
cg00396230
cg00650762
cg26255052
cg22089024
cg00831127
cg07952381
cg05266663
cg11248999
cg15639556
cg05228361
cg22702922
cg20149170
cg02974898
cg14787964
cg04099056
cg05340339
cg05229416
cg08539251
cg09463984
cg09136099
cg13988448
cg12717584
cg22931642
cg13378083
cg18156963
cg15447913
cg04914283
cg24368899
cg12046053
cg08859698
cg08264906
cg26671246
1 : 23036727
1 : 23036868
1 : 23036980
1 : 23037116
1 : 23037368
1 : 23038321
1 : 23038361
1 : 23038925
1 : 23040723
1 : 23061564
1 : 23067453
1 : 23080947
1 : 23089658
1 : 23097476
1 : 23105148
1 : 23105600
1 : 23108861
1 : 23109881
1 : 23111011
1 : 23111123
1 : 23111269
1 : 23111484
1 : 23111493
1 : 23115536
1 : 23122112
1 : 23135043
1 : 23142734
1 : 23153308
1 : 23162550
1 : 23181832
1 : 23191470
1 : 23191576
1 : 23191664
1 : 23197180
1 : 23203462
GeneLoc
PromoterAssoc
CpGIsland
0.25
0.01
mRNA / METH Corr. FDR
MITF
SOX10
TRPM1
ZEB1
AXL
0.05
0.01
Gene / Regulator Corr. P
Type
mRNA_Expr
CopyNum
AmpDel

TFPI

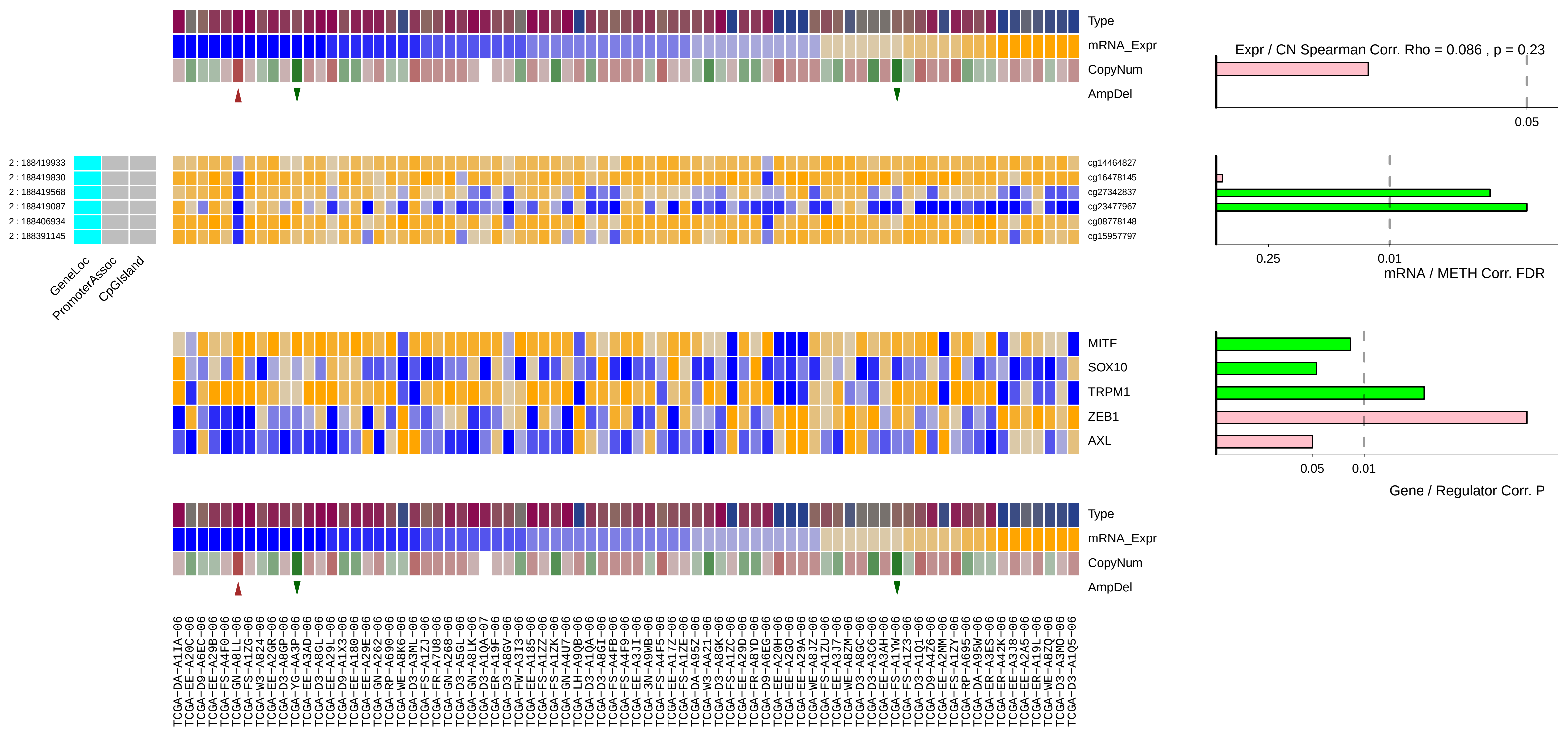

Type
mRNA_Expr
CopyNum
AmpDel
Expr / CN Spearman Corr. Rho = 0.086 , p = 0.23
0.05
cg14464827
cg16478145
cg27342837
cg23477967
cg08778148
cg15957797
2 : 188419933
2 : 188419830
2 : 188419568
2 : 188419087
2 : 188406934
2 : 188391145
GeneLoc
PromoterAssoc
CpGIsland
0.25
0.01
mRNA / METH Corr. FDR
MITF
SOX10
TRPM1
ZEB1
AXL
0.05
0.01
Gene / Regulator Corr. P

NTM

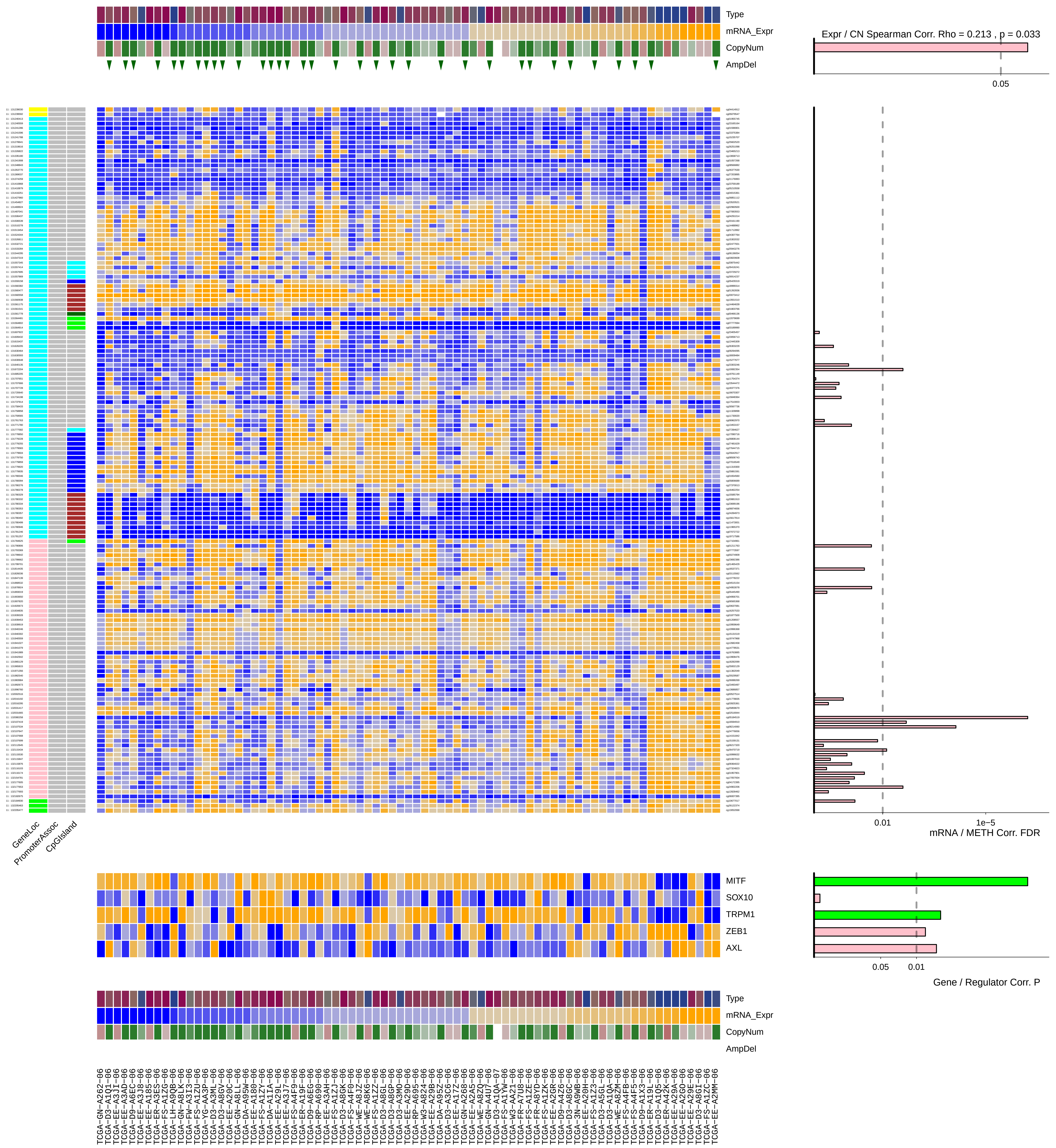

Type
mRNA_Expr
CopyNum
AmpDel
Expr / CN Spearman Corr. Rho = 0.213 , p = 0.033
0.05
GeneLoc
PromoterAssoc
CpGIsland
0.01
1e−5
mRNA / METH Corr. FDR
MITF
SOX10
TRPM1
ZEB1
AXL
0.05
0.01
Gene / Regulator Corr. P

TLE4

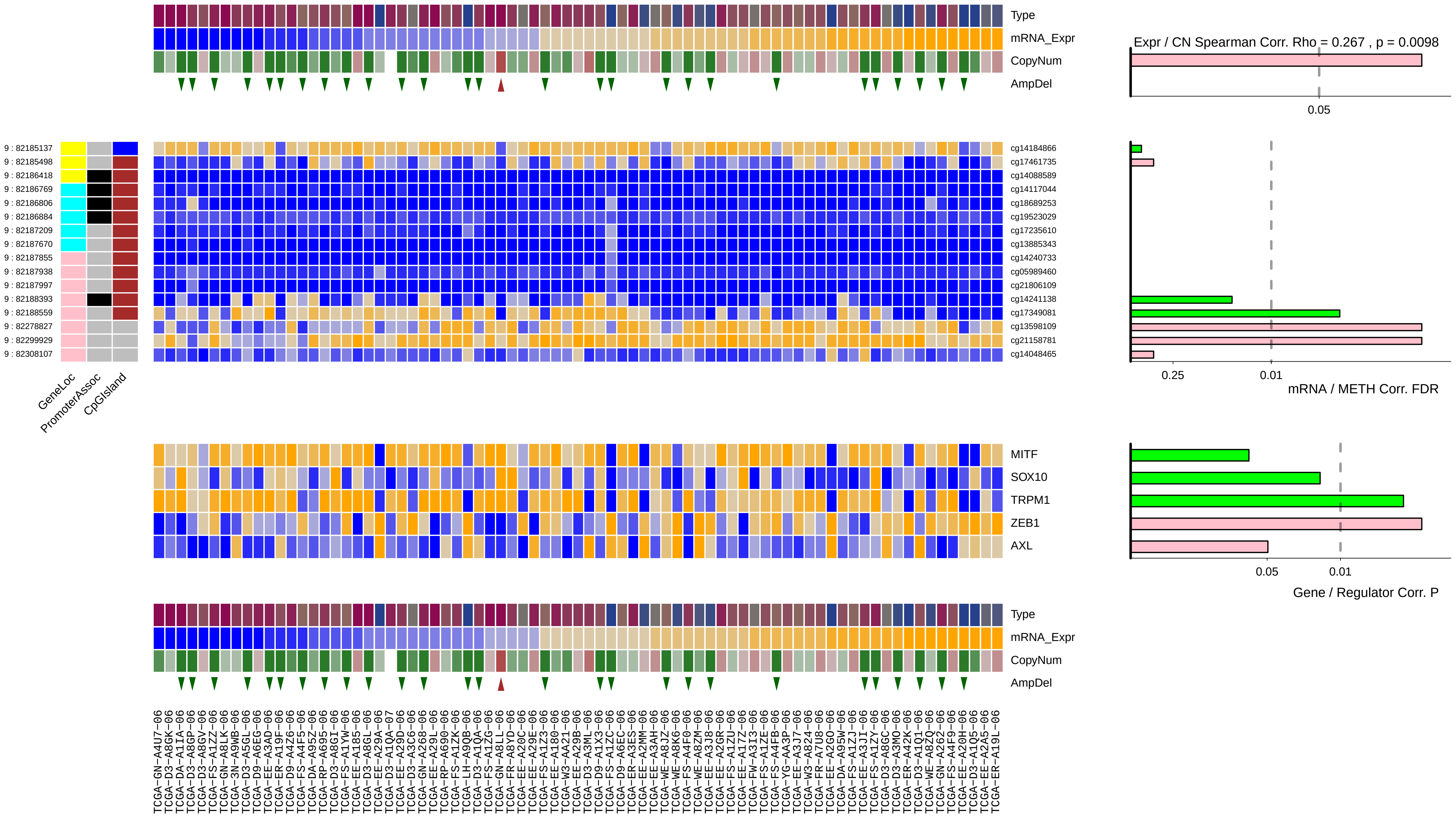

Type
mRNA_Expr
CopyNum
AmpDel
Expr / CN Spearman Corr. Rho = 0.267 , p = 0.0098
0.05
9 : 82185137
9 : 82185498
9 : 82186418
9 : 82186769
9 : 82186806
9 : 82186884
9 : 82187209
9 : 82187670
9 : 82187855
9 : 82187938
9 : 82187997
9 : 82188393
9 : 82188559
9 : 82278827
9 : 82299929
9 : 82308107
cg14184866
cg17461735
cg14088589
cg14117044
cg18689253
cg19523029
cg17235610
cg13885343
cg14240733
cg05989460
cg21806109
cg14241138
cg17349081
cg13598109
cg21158781
cg14048465
0.25
0.01
mRNA / METH Corr. FDR
GeneLoc
PromoterAssoc
CpGIsland
MITF
SOX10
TRPM1
ZEB1
AXL
0.05
0.01
Gene / Regulator Corr. P
Type
mRNA_Expr
CopyNum
AmpDel

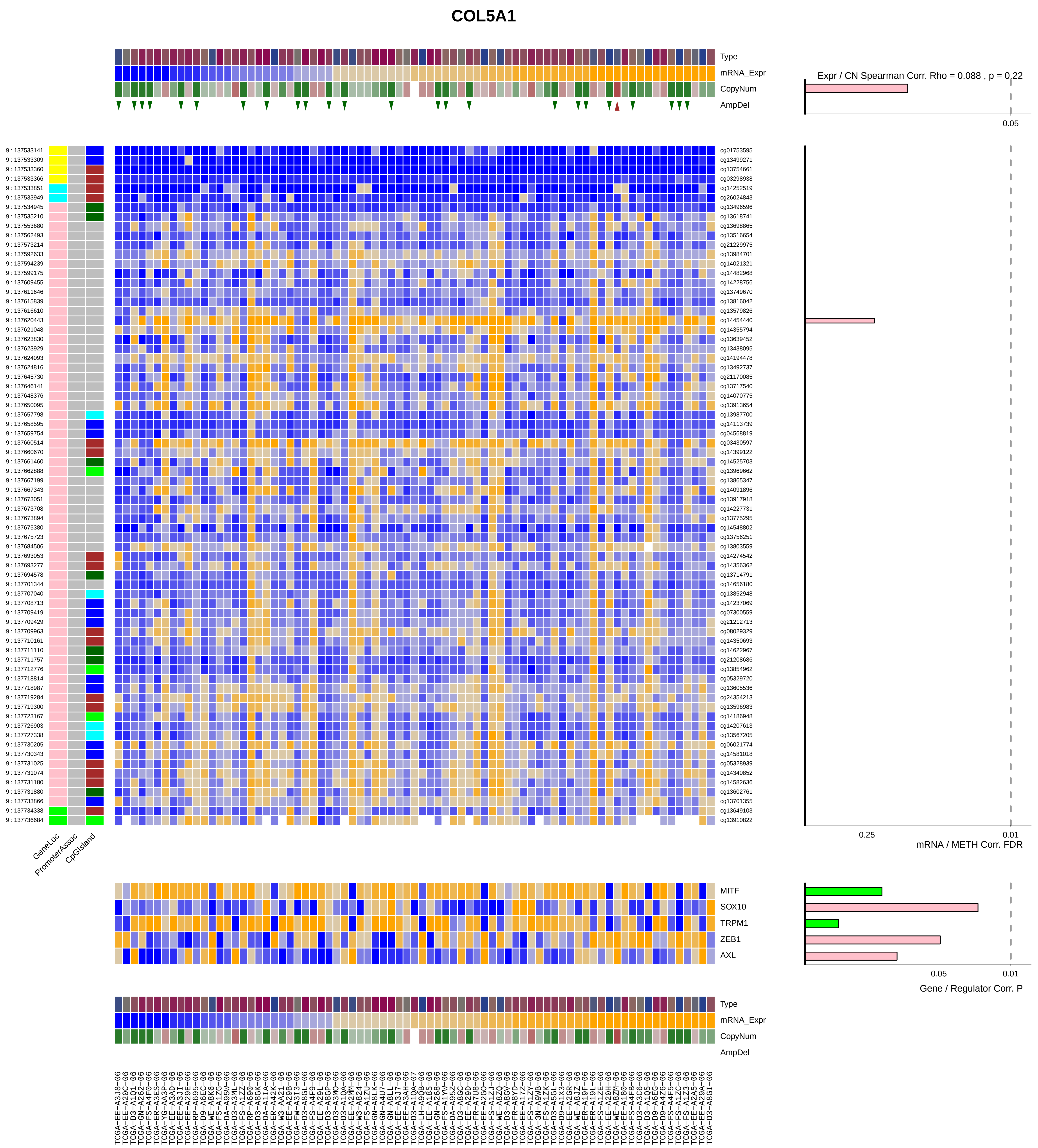

COL5A1
Type
mRNA_Expr
CopyNum
AmpDel
Expr / CN Spearman Corr. Rho = 0.088 , p = 0.22
0.05
9 : 137533141
9 : 137533309
9 : 137533360
9 : 137533366
9 : 137533851
9 : 137533949
9 : 137534945
9 : 137535210
9 : 137553680
9 : 137562493
9 : 137573214
9 : 137592633
9 : 137594239
9 : 137599175
9 : 137609455
9 : 137611646
9 : 137615839
9 : 137616610
9 : 137620443
9 : 137621048
9 : 137623830
9 : 137623929
9 : 137624093
9 : 137624816
9 : 137645730
9 : 137646141
9 : 137648376
9 : 137650095
9 : 137657798
9 : 137658595
9 : 137659754
9 : 137660514
9 : 137660670
9 : 137661460
9 : 137662888
9 : 137667199
9 : 137667343
9 : 137673051
9 : 137673708
9 : 137673894
9 : 137675380
9 : 137675723
9 : 137684506
9 : 137693053
9 : 137693277
9 : 137694578
9 : 137701344
9 : 137707040
9 : 137708713
9 : 137709419
9 : 137709429
9 : 137709963
9 : 137710161
9 : 137711110
9 : 137711757
9 : 137712776
9 : 137718814
9 : 137718987
9 : 137719284
9 : 137719300
9 : 137723167
9 : 137726903
9 : 137727338
9 : 137730205
9 : 137730343
9 : 137731025
9 : 137731074
9 : 137731180
9 : 137731880
9 : 137733866
9 : 137734338
9 : 137736684
cg01753595
cg13499271
cg13754661
cg03298938
cg14252519
cg26024843
cg13496596
cg13618741
cg13698865
cg13516654
cg21229975
cg13984701
cg14021321
cg14482968
cg14228756
cg13749670
cg13816042
cg13579826
cg14454440
cg14355794
cg13639452
cg13438095
cg14194478
cg13492737
cg21170085
cg13717540
cg14070775
cg13913654
cg13987700
cg14113739
cg04568819
cg03430597
cg14399122
cg14525703
cg13969662
cg13865347
cg14091896
cg13917918
cg14227731
cg13775295
cg14548802
cg13756251
cg13803559
cg14274542
cg14356362
cg13714791
cg14656180
cg13852948
cg14237069
cg07300559
cg21212713
cg08029329
cg14350693
cg14622967
cg21208686
cg13854962
cg05329720
cg13605536
cg24354213
cg13596983
cg14186948
cg14207613
cg13567205
cg06021774
cg14581018
cg05328939
cg14340852
cg14582636
cg13602761
cg13701355
cg13649103
cg13910822
0.25
0.01
mRNA / METH Corr. FDR
GeneLoc
PromoterAssoc
CpGIsland
MITF
SOX10
TRPM1
ZEB1
AXL
0.05
0.01
Gene / Regulator Corr. P
Type
mRNA_Expr
CopyNum
AmpDel

C12ORF75

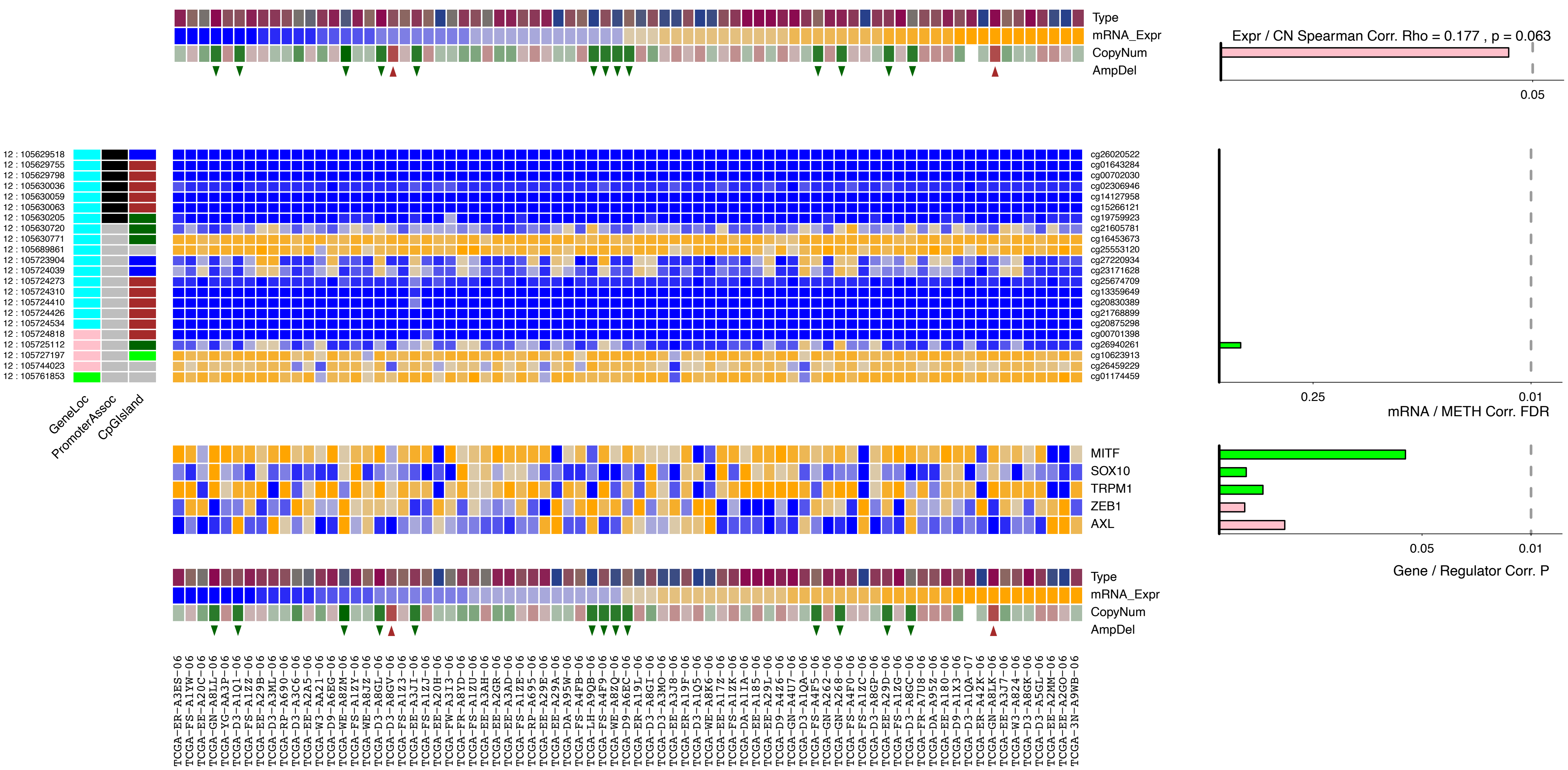

Type
mRNA_Expr
CopyNum
AmpDel
Expr / CN Spearman Corr. Rho = 0.177 , p = 0.063
0.05
12 : 105629518
12 : 105629755
12 : 105629798
12 : 105630036
12 : 105630059
12 : 105630063
12 : 105630205
12 : 105630720
12 : 105630771
12 : 105689861
12 : 105723904
12 : 105724039
12 : 105724273
12 : 105724310
12 : 105724410
12 : 105724426
12 : 105724534
12 : 105724818
12 : 105725112
12 : 105727197
12 : 105744023
12 : 105761853
cg26020522
cg01643284
cg00702030
cg02306946
cg14127958
cg15266121
cg19759923
cg21605781
cg16453673
cg25553120
cg27220934
cg23171628
cg25674709
cg13359649
cg20830389
cg21768899
cg20875298
cg00701398
cg26940261
cg10623913
cg26459229
cg01174459
GeneLoc
PromoterAssoc
CpGIsland
0.25
0.01
mRNA / METH Corr. FDR
MITF
SOX10
TRPM1
ZEB1
AXL
0.05
0.01
Gene / Regulator Corr. P
Type
mRNA_Expr
CopyNum
AmpDel

RRAS2

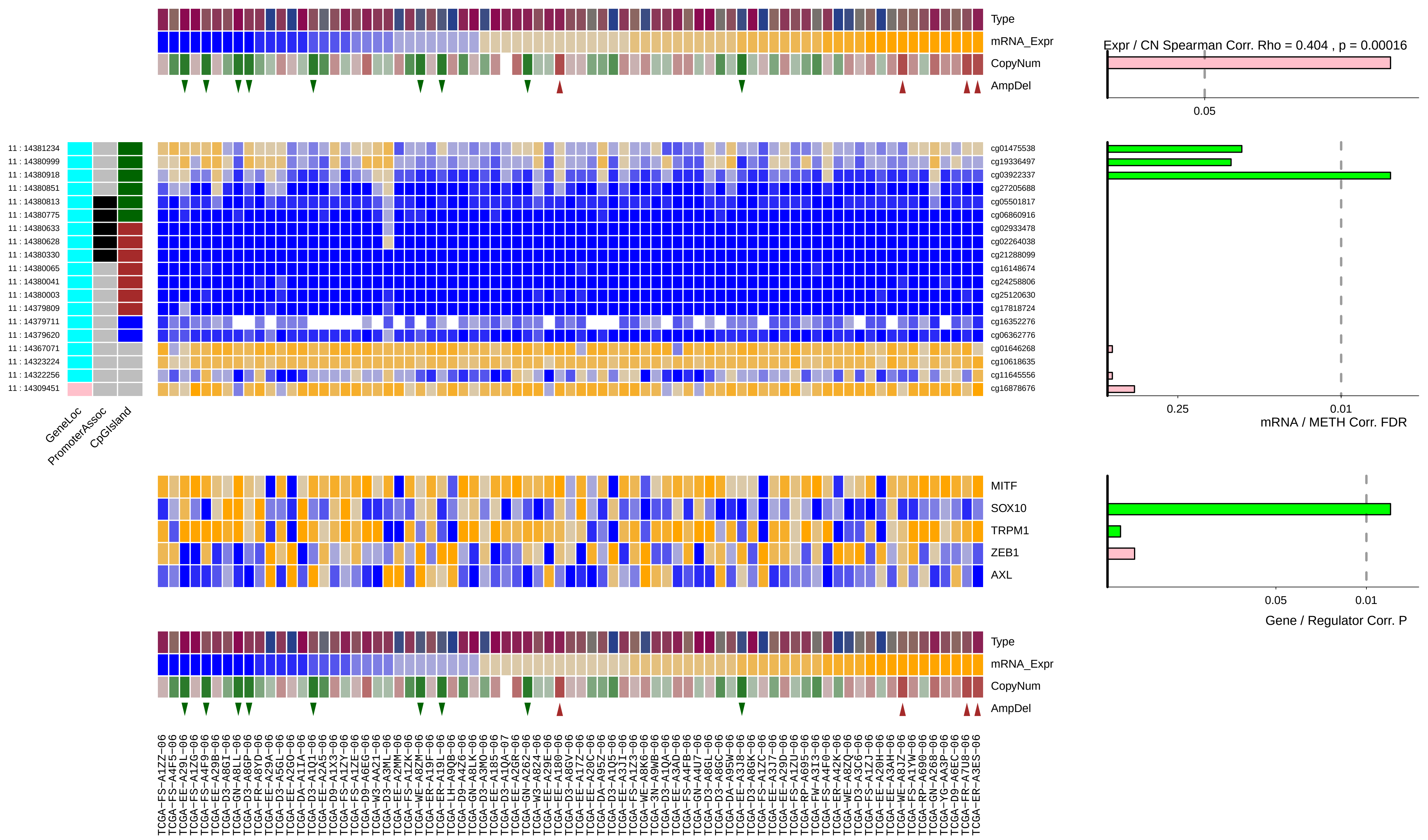
Type
mRNA_Expr
CopyNum
AmpDel
Expr / CN Spearman Corr. Rho = 0.404 , p = 0.00016
0.05
11 : 14381234
11 : 14380999
11 : 14380918
11 : 14380851
11 : 14380813
11 : 14380775
11 : 14380633
11 : 14380628
11 : 14380330
11 : 14380065
11 : 14380041
11 : 14380003
11 : 14379809
11 : 14379711
11 : 14379620
11 : 14367071
11 : 14323224
11 : 14322256
11 : 14309451
cg01475538
cg19336497
cg03922337
cg27205688
cg05501817
cg06860916
cg02933478
cg02264038
cg21288099
cg16148674
cg24258806
cg25120630
cg17818724
cg16352276
cg06362776
cg01646268
cg10618635
cg11645556
cg16878676
0.25
0.01
mRNA / METH Corr. FDR
GeneLoc
PromoterAssoc
CpGIsland
MITF
SOX10
TRPM1
ZEB1
AXL
0.05
0.01
Gene / Regulator Corr. P
Type
mRNA_Expr
CopyNum
AmpDel

MYLK

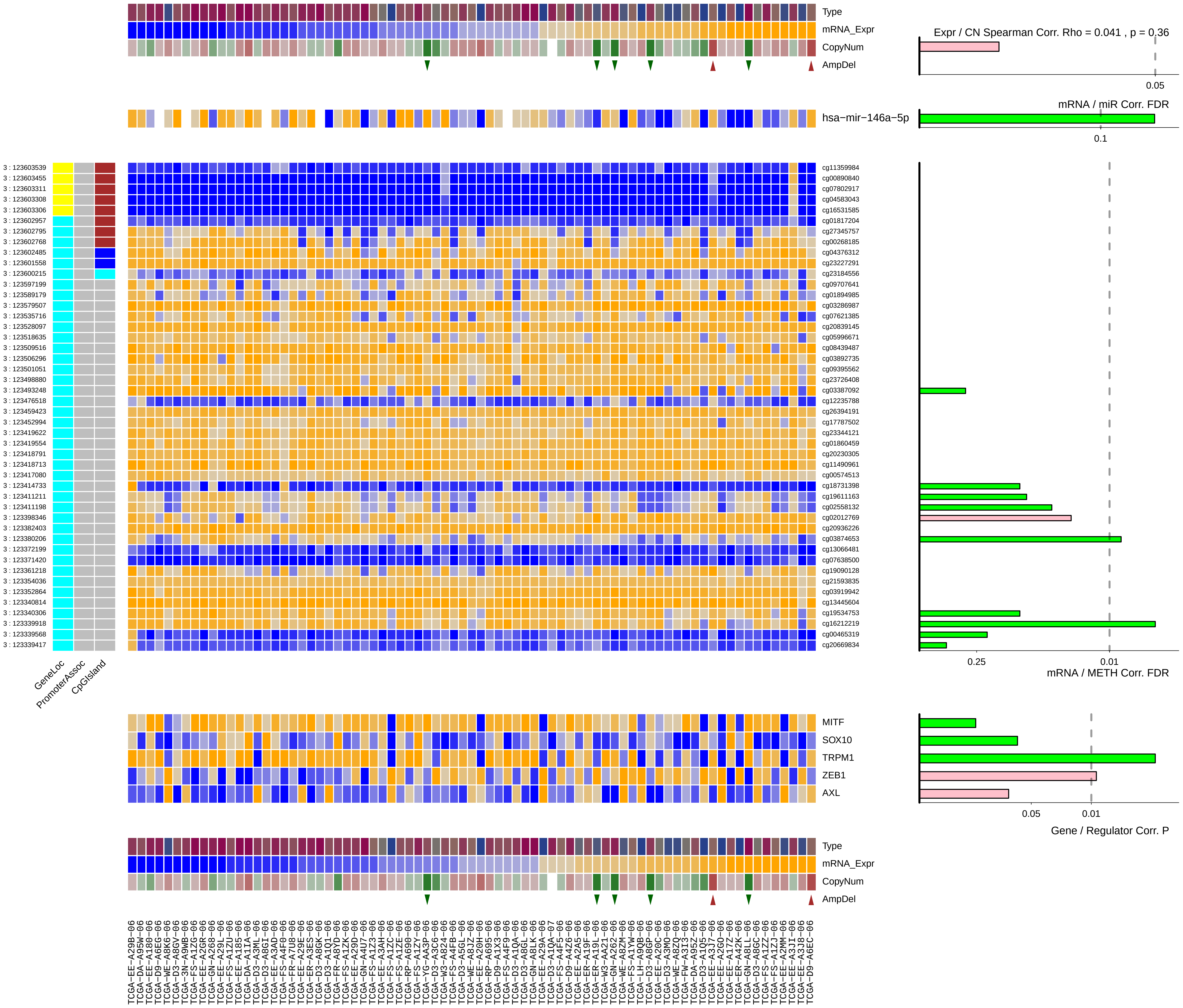

Type
mRNA_Expr
CopyNum
AmpDel
Expr / CN Spearman Corr. Rho = 0.041 , p = 0.36
0.05
hsa−mir−146a−5p
mRNA / miR Corr. FDR
0.1
cg11359984
cg00890840
cg07802917
cg04583043
cg16531585
cg01817204
cg27345757
cg00268185
cg04376312
cg23227291
cg23184556
cg09707641
cg01894985
cg03286987
cg07621385
cg20839145
cg05996671
cg08439487
cg03892735
cg09395562
cg23726408
cg03387092
cg12235788
cg26394191
cg17787502
cg23344121
cg01860459
cg20230305
cg11490961
cg00574513
cg18731398
cg19611163
cg02558132
cg02012769
cg20936226
cg03874653
cg13066481
cg07638500
cg19090128
cg21593835
cg03919942
cg13445604
cg19534753
cg16212219
cg00465319
cg20669834
GeneLoc
PromoterAssoc
CpGIsland
0.25
0.01
mRNA / METH Corr. FDR
MITF
SOX10
TRPM1
ZEB1
AXL
0.05
0.01
Gene / Regulator Corr. P

CPA4

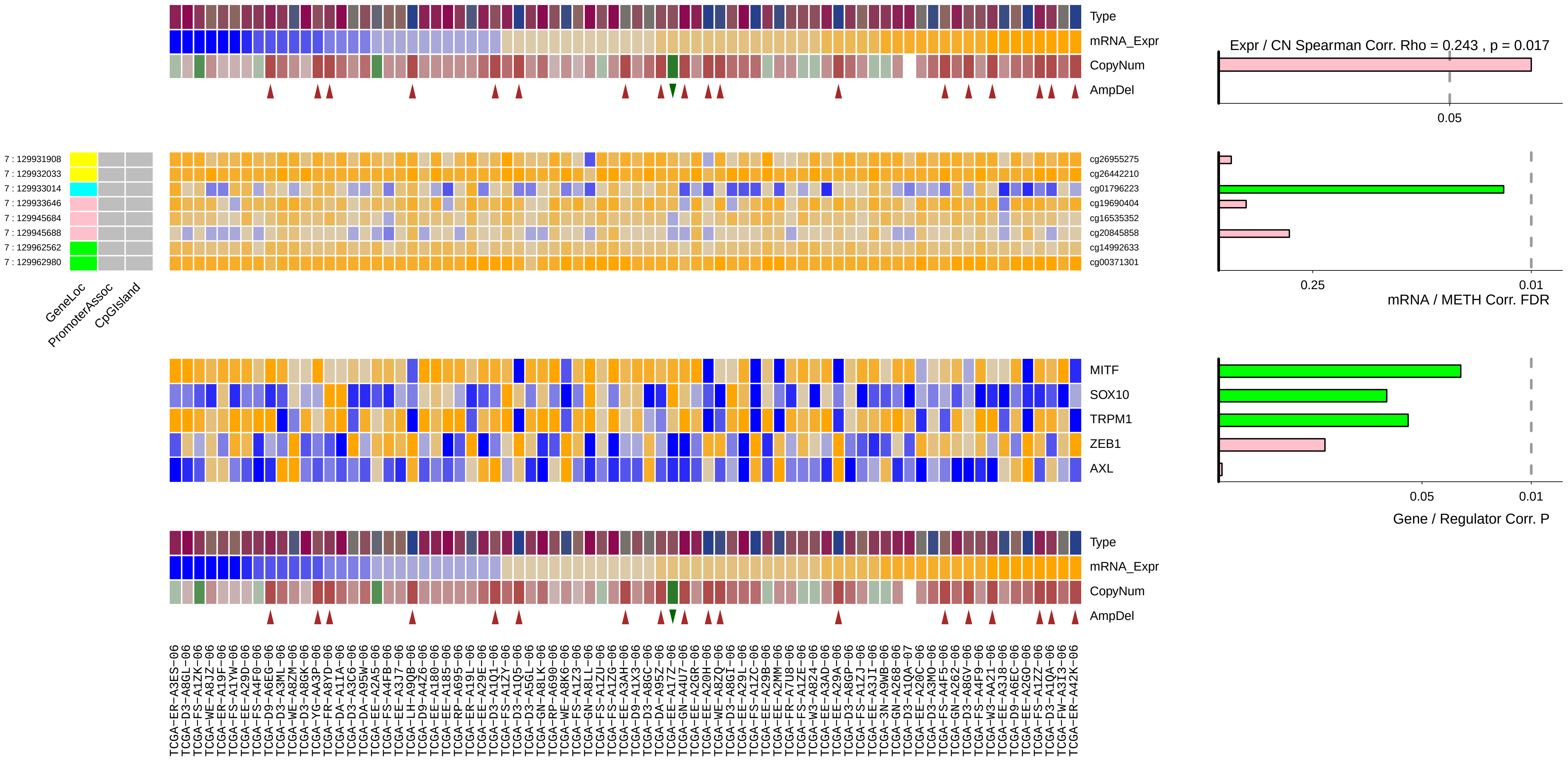
Type
mRNA_Expr
CopyNum
AmpDel
Expr / CN Spearman Corr. Rho = 0.243 , p = 0.017
0.05
7 : 129931908
7 : 129932033
7 : 129933014
7 : 129933646
7 : 129945684
7 : 129945688
7 : 129962562
7 : 129962980
GeneLoc
PromoterAssoc
CpGIsland
cg26955275
cg26442210
cg01796223
cg19690404
cg16535352
cg20845858
cg14992633
cg00371301
0.25
0.01
mRNA / METH Corr. FDR
MITF
SOX10
TRPM1
ZEB1
AXL
0.05
0.01
Gene / Regulator Corr. P
Type
mRNA_Expr
CopyNum
AmpDel

CCDC80

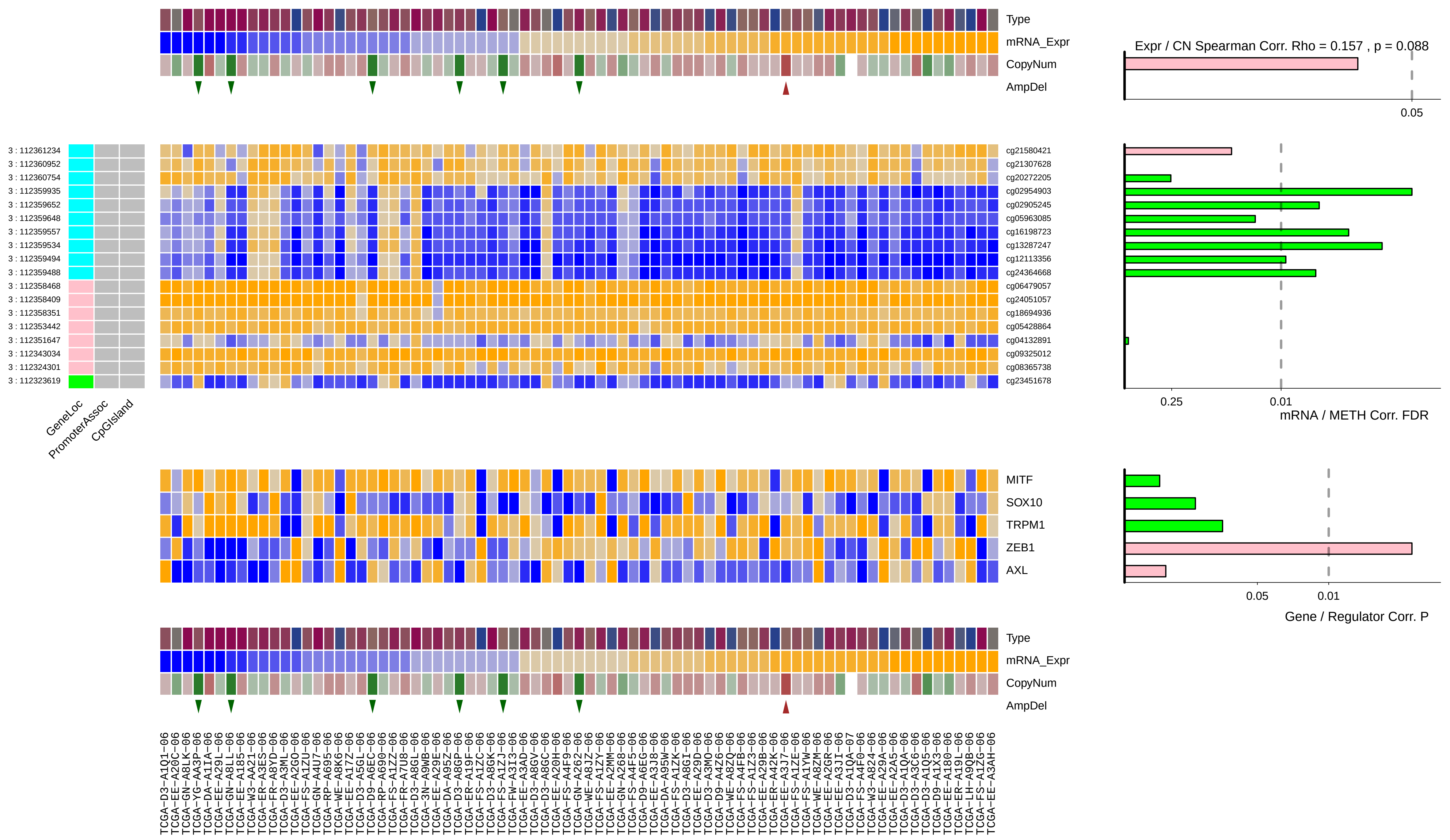

Type
mRNA_Expr
CopyNum
AmpDel
Expr / CN Spearman Corr. Rho = 0.157 , p = 0.088
0.05
3 : 112361234
3 : 112360952
3 : 112360754
3 : 112359935
3 : 112359652
3 : 112359648
3 : 112359557
3 : 112359534
3 : 112359494
3 : 112359488
3 : 112358468
3 : 112358409
3 : 112358351
3 : 112353442
3 : 112351647
3 : 112343034
3 : 112324301
3 : 112323619
GeneLoc
PromoterAssoc
CpGIsland
cg21580421
cg21307628
cg20272205
cg02954903
cg02905245
cg05963085
cg16198723
cg13287247
cg12113356
cg24364668
cg06479057
cg24051057
cg18694936
cg05428864
cg04132891
cg09325012
cg08365738
cg23451678
0.25
0.01
mRNA / METH Corr. FDR
MITF
SOX10
TRPM1
ZEB1
AXL
0.05
0.01
Gene / Regulator Corr. P
Type
mRNA_Expr
CopyNum
AmpDel

DDX58

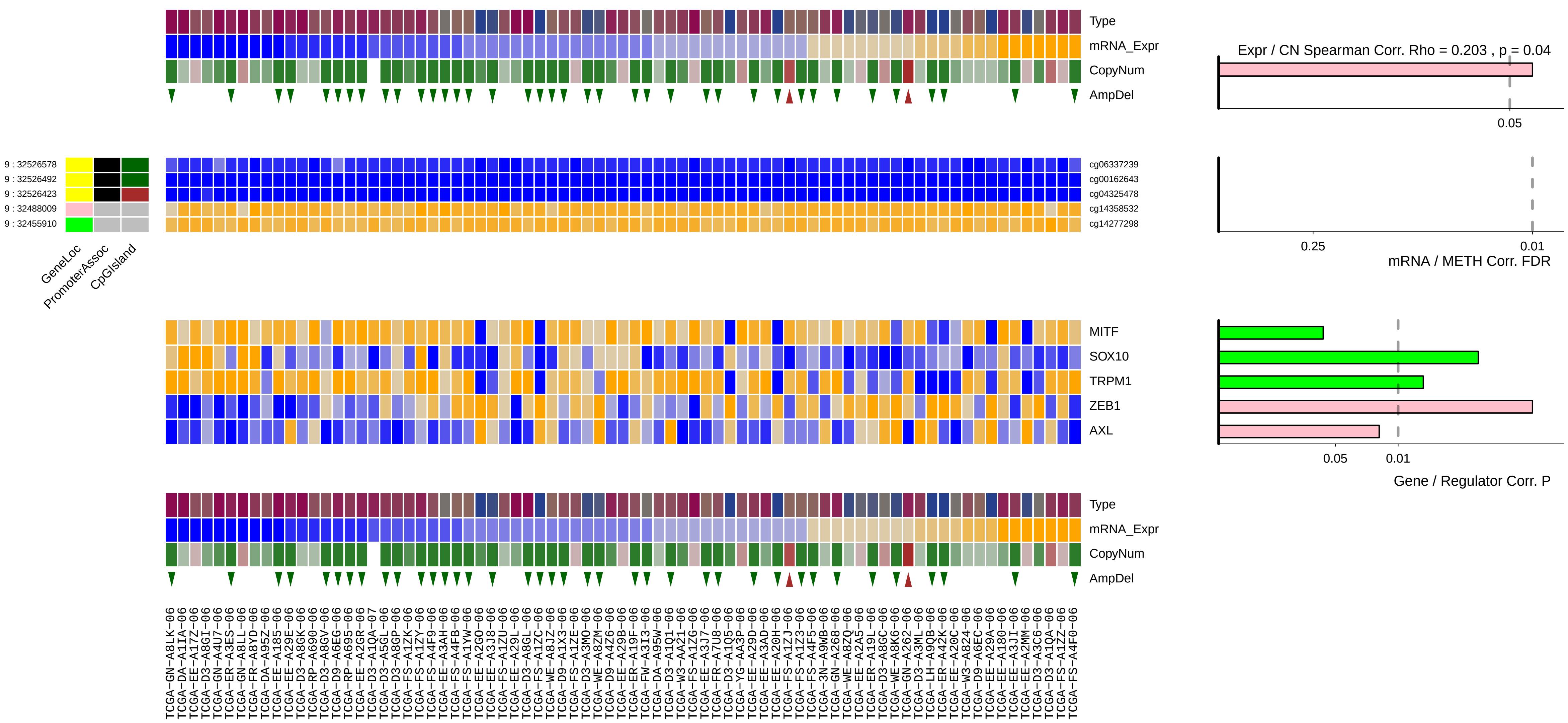
Type
mRNA_Expr
CopyNum
AmpDel
Expr / CN Spearman Corr. Rho = 0.203 , p = 0.04
0.05
9 : 32526578
9 : 32526492
9 : 32526423
9 : 32488009
9 : 32455910
GeneLoc
PromoterAssoc
CpGIsland
cg06337239
cg00162643
cg04325478
cg14358532
cg14277298
0.25
0.01
mRNA / METH Corr. FDR
MITF
SOX10
TRPM1
ZEB1
AXL
0.05
0.01
Gene / Regulator Corr. P
Type
mRNA_Expr
CopyNum
AmpDel

SLC9A7

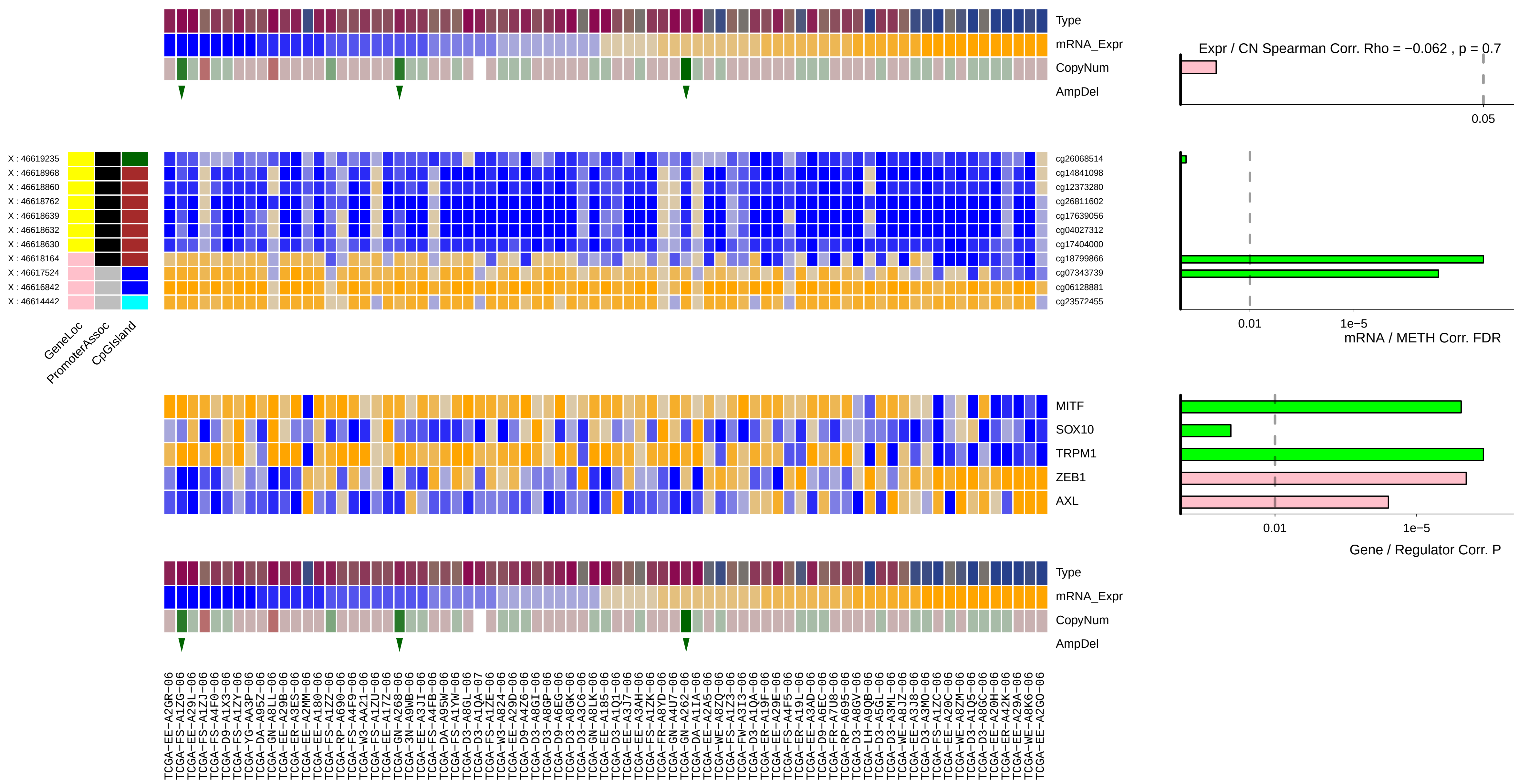

Type
mRNA_Expr
CopyNum
AmpDel
Expr / CN Spearman Corr. Rho = −0.062 , p = 0.7
0.05
X : 46619235
X : 46618968
X : 46618860
X : 46618762
X : 46618639
X : 46618632
X : 46618630
X : 46618164
X : 46617524
X : 46616842
X : 46614442
GeneLoc
PromoterAssoc
CpGIsland
cg26068514
cg14841098
cg12373280
cg26811602
cg17639056
cg04027312
cg17404000
cg18799866
cg07343739
cg06128881
cg23572455
0.01
1e−5
mRNA / METH Corr. FDR
MITF
SOX10
TRPM1
ZEB1
AXL
0.01
1e−5
Gene / Regulator Corr. P
Type
mRNA_Expr
CopyNum
AmpDel

ANGPT1

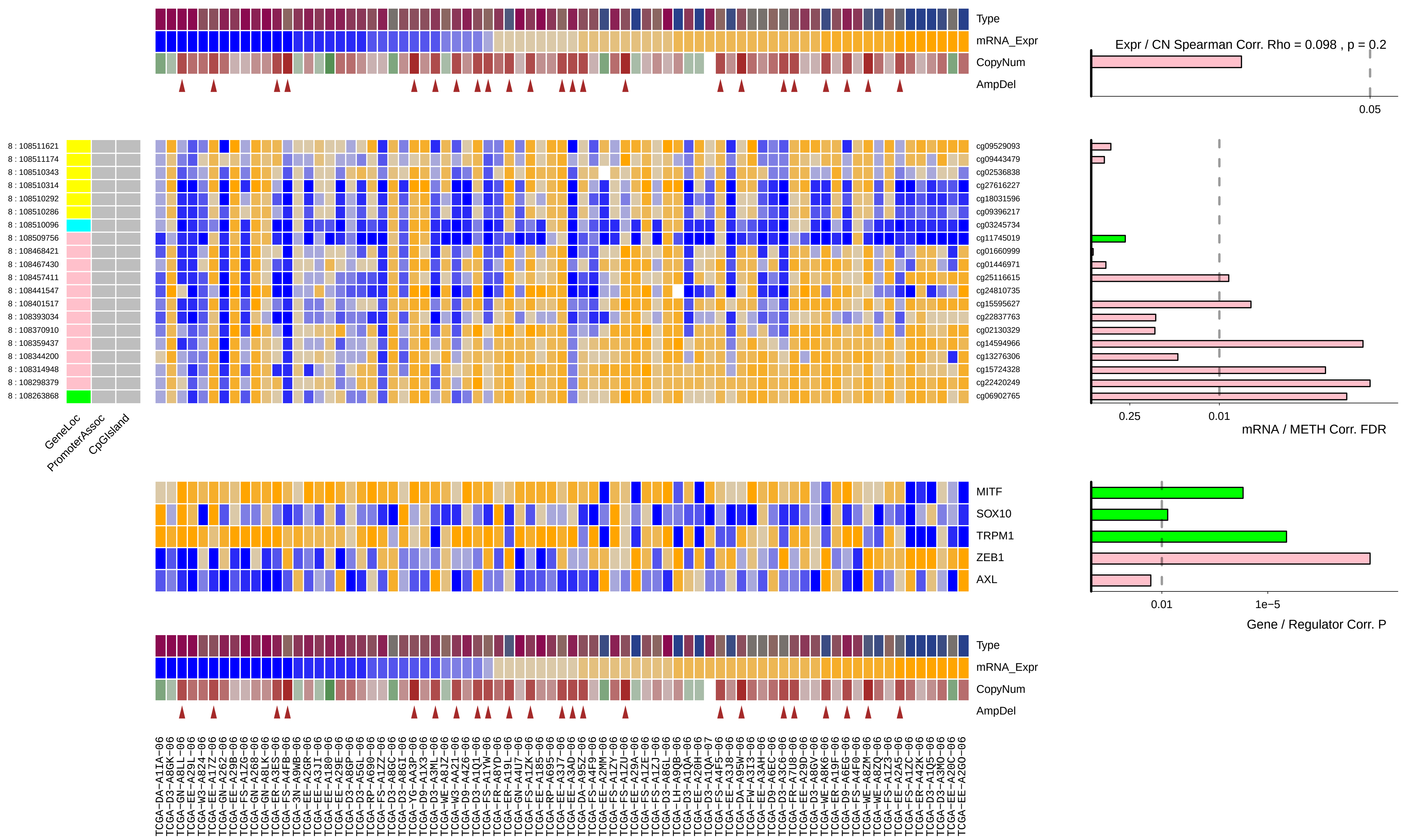
Type
mRNA_Expr
CopyNum
AmpDel
Expr / CN Spearman Corr. Rho = 0.098 , p = 0.2
0.05
8 : 108511621
8 : 108511174
8 : 108510343
8 : 108510314
8 : 108510292
8 : 108510286
8 : 108510096
8 : 108509756
8 : 108468421
8 : 108467430
8 : 108457411
8 : 108441547
8 : 108401517
8 : 108393034
8 : 108370910
8 : 108359437
8 : 108344200
8 : 108314948
8 : 108298379
8 : 108263868
cg09529093
cg09443479
cg02536838
cg27616227
cg18031596
cg09396217
cg03245734
cg11745019
cg01660999
cg01446971
cg25116615
cg24810735
cg15595627
cg22837763
cg02130329
cg14594966
cg13276306
cg15724328
cg22420249
cg06902765
GeneLoc
PromoterAssoc
CpGIsland
0.25
0.01
mRNA / METH Corr. FDR
MITF
SOX10
TRPM1
ZEB1
AXL
0.01
1e−5
Gene / Regulator Corr. P
Type
mRNA_Expr
CopyNum
AmpDel

STC2

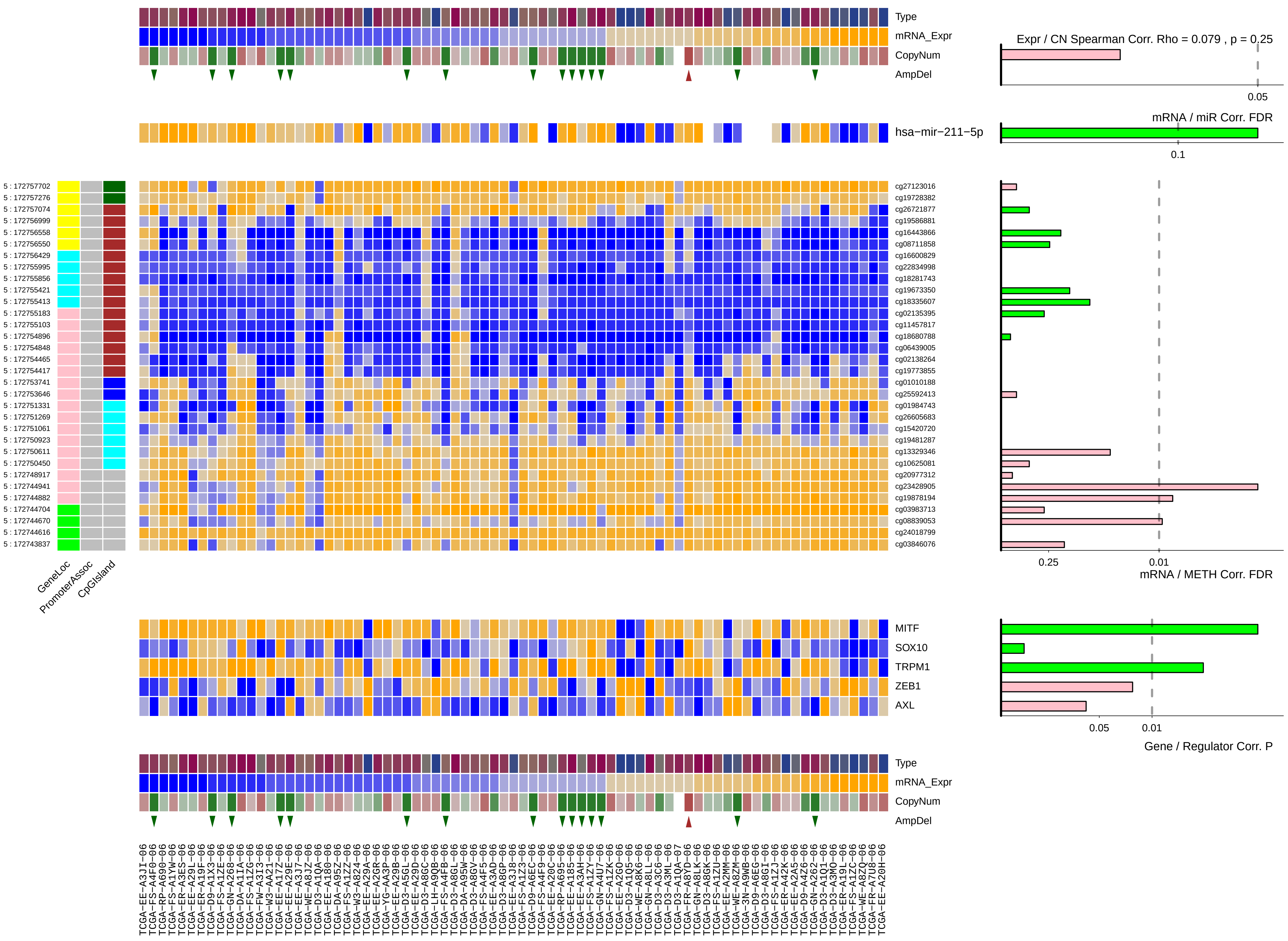

Type
mRNA_Expr
CopyNum
AmpDel
Expr / CN Spearman Corr. Rho = 0.079 , p = 0.25
0.05
hsa−mir−211−5p
mRNA / miR Corr. FDR
0.1
cg27123016
cg19728382
cg26721877
cg19586881
cg16443866
cg08711858
cg16600829
cg22834998
cg18281743
cg19673350
cg18335607
cg02135395
cg11457817
cg18680788
cg06439005
cg02138264
cg19773855
cg01010188
cg25592413
cg01984743
cg26605683
cg15420720
cg19481287
cg13329346
cg10625081
cg20977312
cg23428905
cg19878194
cg03983713
cg08839053
cg24018799
cg03846076
0.25
0.01
mRNA / METH Corr. FDR
GeneLoc
PromoterAssoc
CpGIsland
MITF
SOX10
TRPM1
ZEB1
AXL
0.05
0.01
Gene / Regulator Corr. P
Type
mRNA_Expr
CopyNum
AmpDel

PODXL

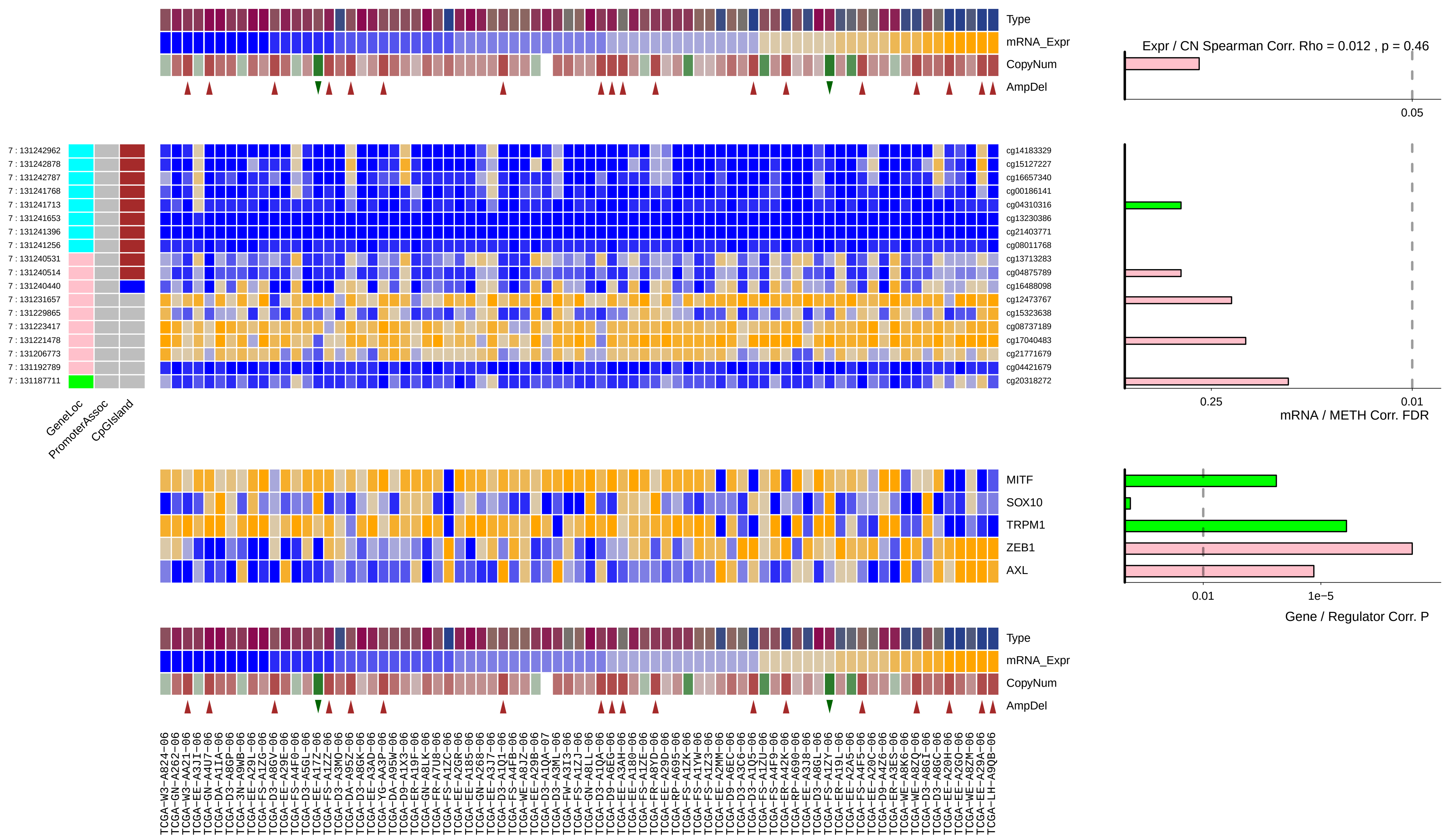

Type
mRNA_Expr
CopyNum
AmpDel
Expr / CN Spearman Corr. Rho = 0.012 , p = 0.46
0.05
7 : 131242962
7 : 131242878
7 : 131242787
7 : 131241768
7 : 131241713
7 : 131241653
7 : 131241396
7 : 131241256
7 : 131240531
7 : 131240514
7 : 131240440
7 : 131231657
7 : 131229865
7 : 131223417
7 : 131221478
7 : 131206773
7 : 131192789
7 : 131187711
GeneLoc
PromoterAssoc
CpGIsland
cg14183329
cg15127227
cg16657340
cg00186141
cg04310316
cg13230386
cg21403771
cg08011768
cg13713283
cg04875789
cg16488098
cg12473767
cg15323638
cg08737189
cg17040483
cg21771679
cg04421679
cg20318272
0.25
0.01
mRNA / METH Corr. FDR
MITF
SOX10
TRPM1
ZEB1
AXL
0.01
1e−5
Gene / Regulator Corr. P
Type
mRNA_Expr
CopyNum
AmpDel

ARNTL2

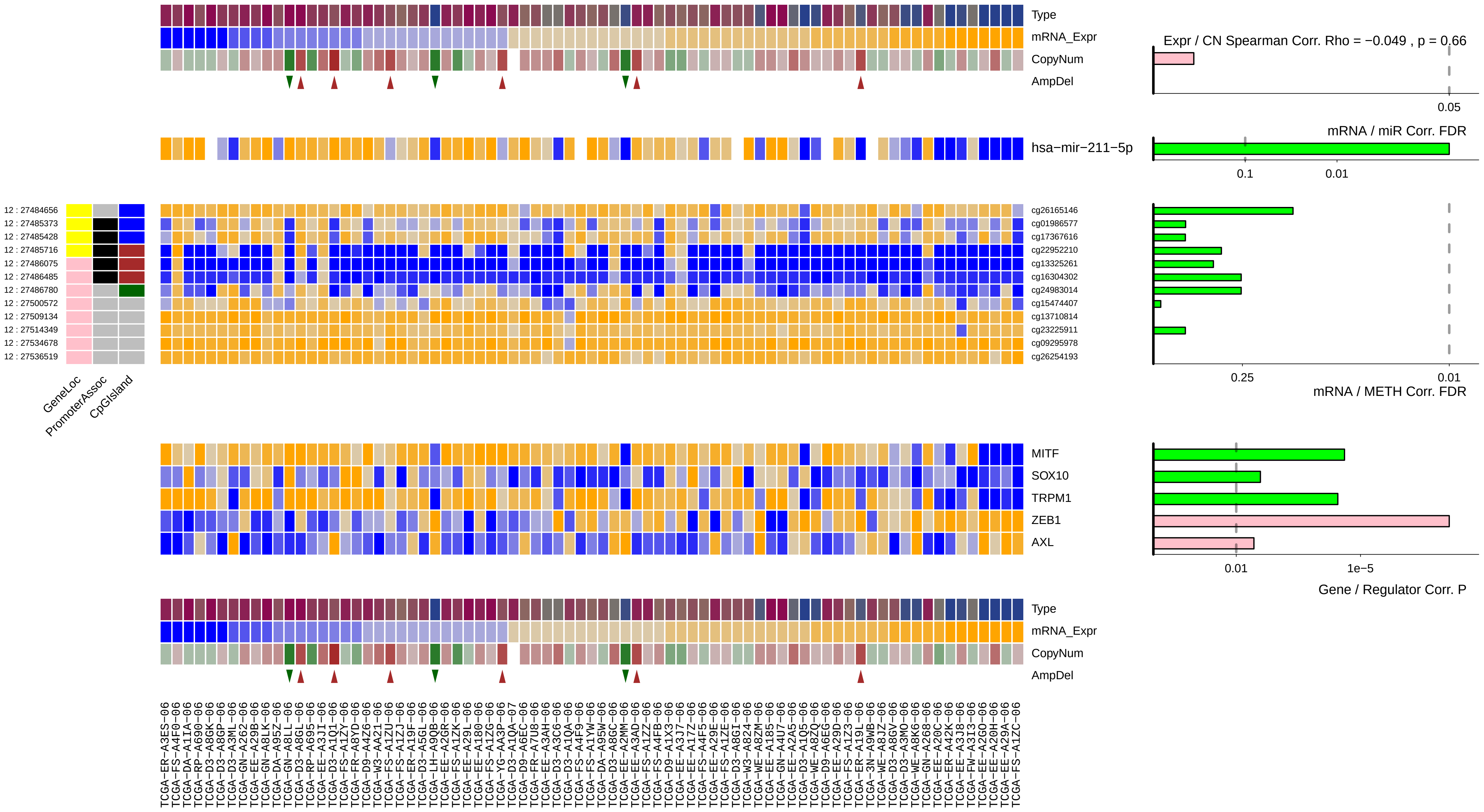
Type
mRNA_Expr
CopyNum
AmpDel
Expr / CN Spearman Corr. Rho = −0.049 , p = 0.66
0.05
hsa−mir−211−5p
mRNA / miR Corr. FDR
0.1
0.01
12 : 27484656
12 : 27485373
12 : 27485428
12 : 27485716
12 : 27486075
12 : 27486485
12 : 27486780
12 : 27500572
12 : 27509134
12 : 27514349
12 : 27534678
12 : 27536519
GeneLoc
PromoterAssoc
CpGIsland
cg26165146
cg01986577
cg17367616
cg22952210
cg13325261
cg16304302
cg24983014
cg15474407
cg13710814
cg23225911
cg09295978
cg26254193
0.25
0.01
mRNA / METH Corr. FDR
MITF
SOX10
TRPM1
ZEB1
AXL
0.01
1e−5
Gene / Regulator Corr. P
Type
mRNA_Expr
CopyNum
AmpDel

GPR176

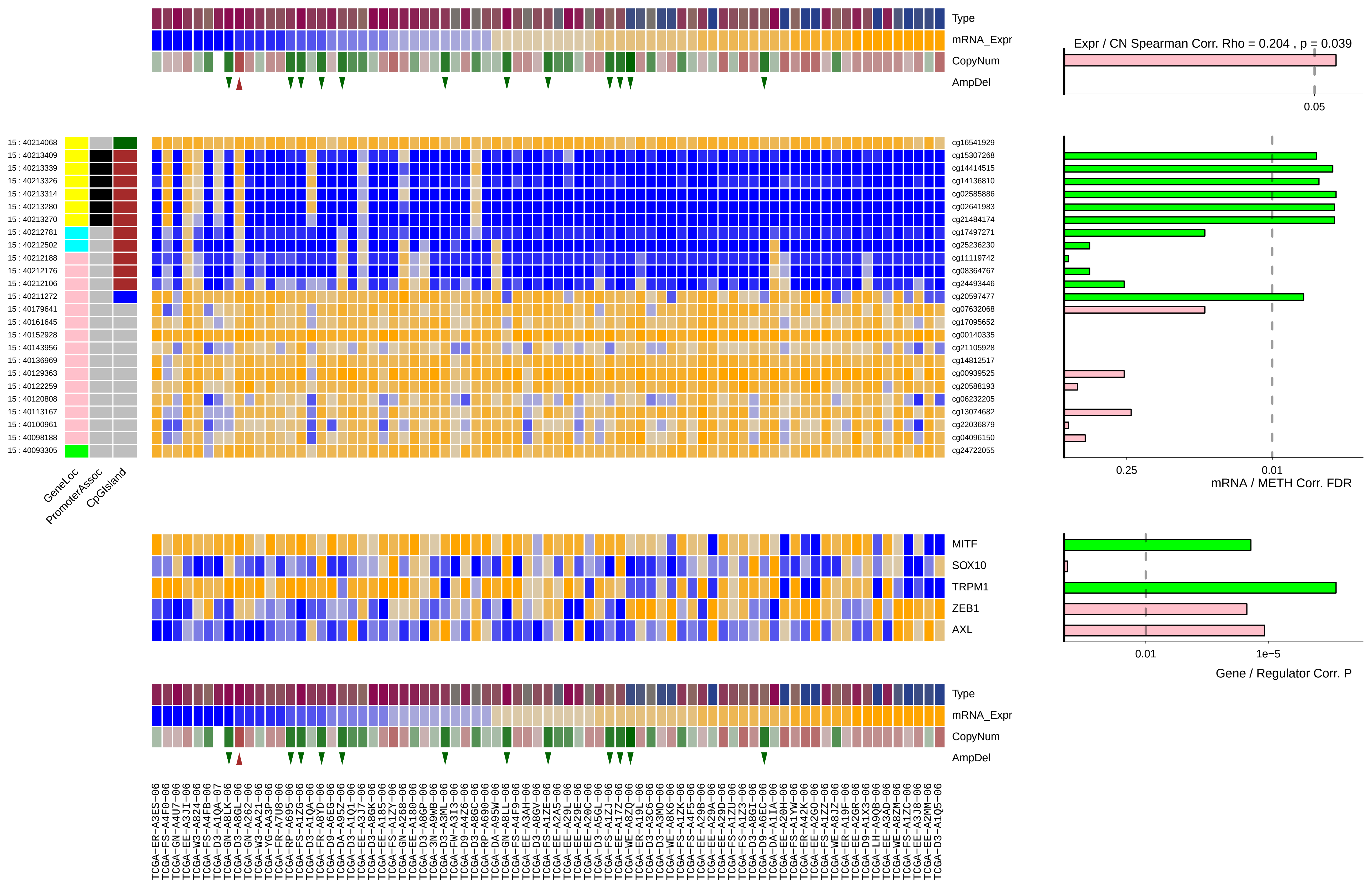

Type
mRNA_Expr
CopyNum
AmpDel
Expr / CN Spearman Corr. Rho = 0.204 , p = 0.039
0.05
GeneLoc
PromoterAssoc
CpGIsland
0.25
0.01
mRNA / METH Corr. FDR
MITF
SOX10
TRPM1
ZEB1
AXL
0.01
1e−5
Gene / Regulator Corr. P

FLNC

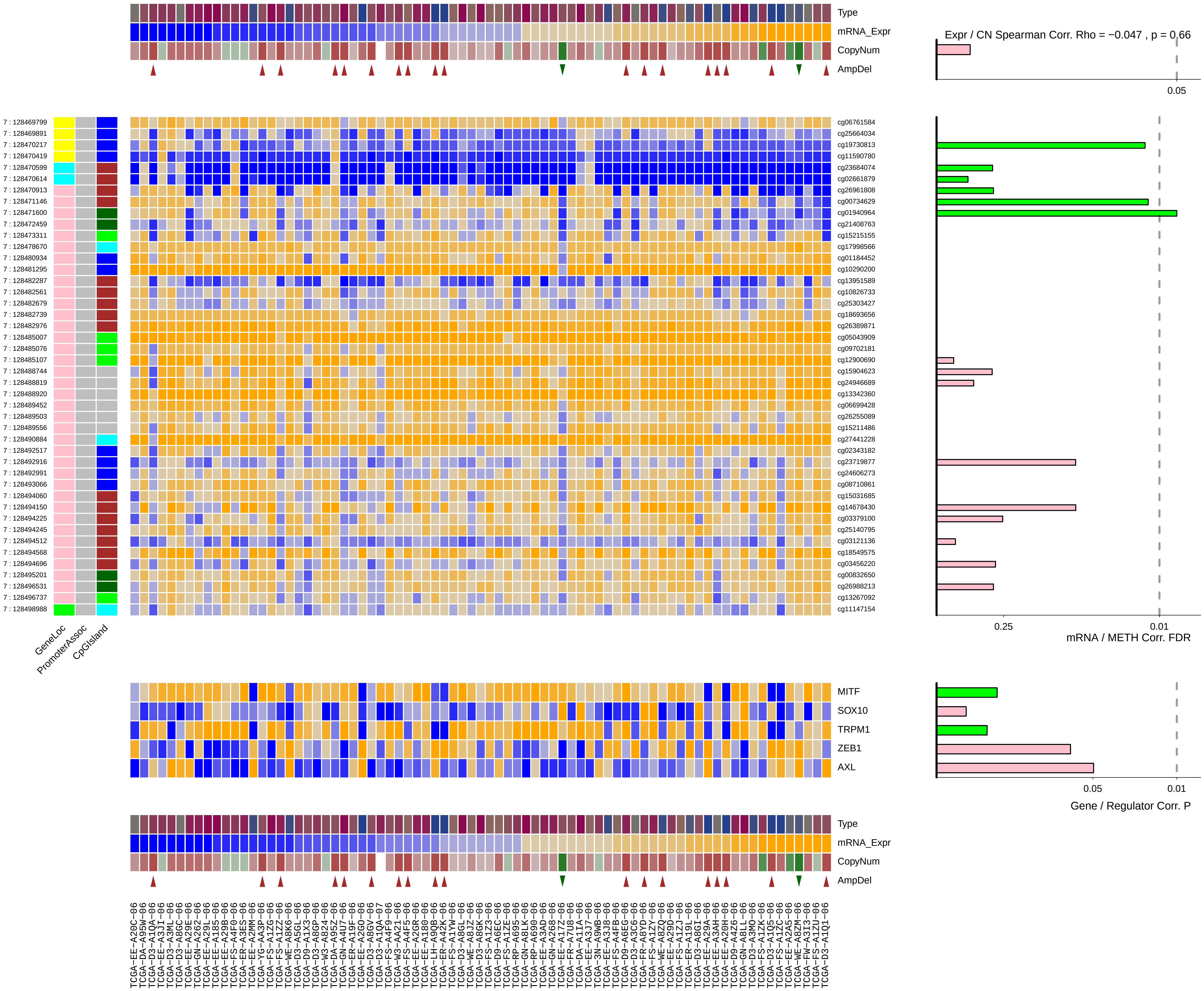
Type
mRNA_Expr
CopyNum
AmpDel
Expr / CN Spearman Corr. Rho = −0.047 , p = 0.66
0.05
mRNA / METH Corr. FDR
0.25
0.01
GeneLoc
PromoterAssoc
CpGIsland
MITF
SOX10
TRPM1
ZEB1
AXL
Gene / Regulator Corr. P
0.05
0.01

COL8A1

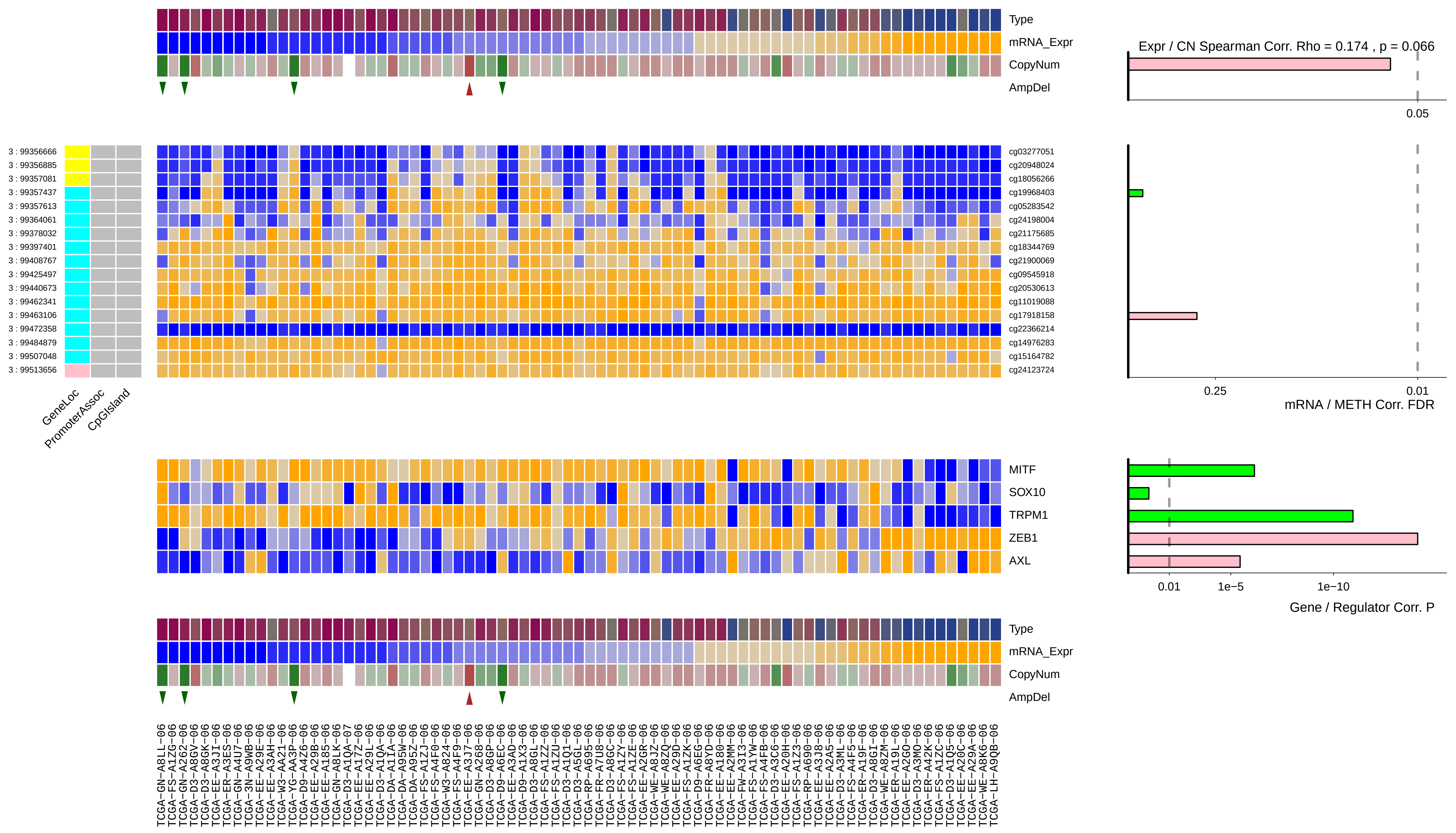
Type
mRNA_Expr
CopyNum
AmpDel
Expr / CN Spearman Corr. Rho = 0.174 , p = 0.066
0.05
GeneLoc
PromoterAssoc
CpGIsland
0.25
0.01
mRNA / METH Corr. FDR
MITF
SOX10
TRPM1
ZEB1
AXL
0.01
1e−5
1e−10
Gene / Regulator Corr. P
Type
mRNA_Expr
CopyNum
AmpDel

CAV1

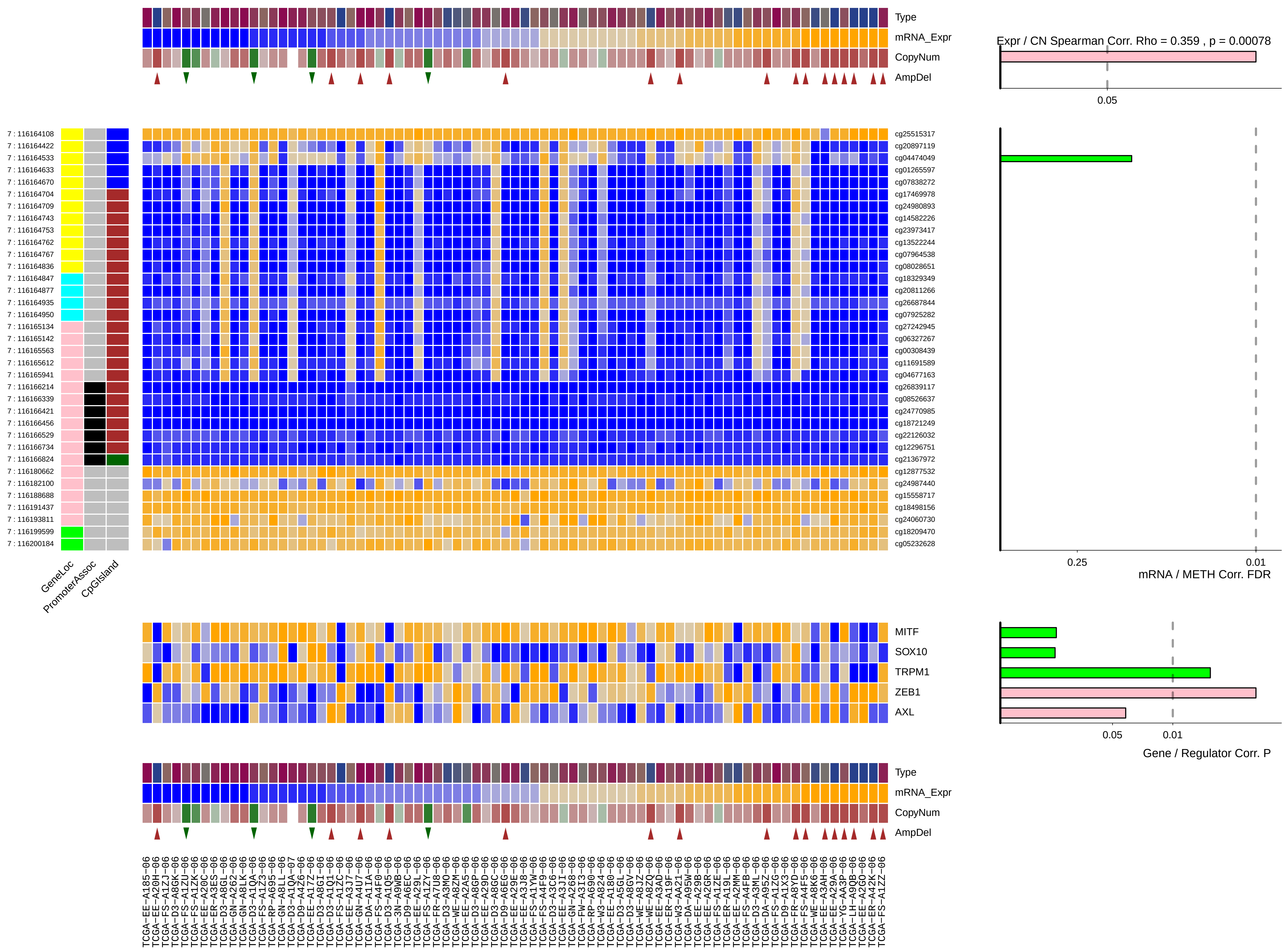

Type
mRNA_Expr
CopyNum
AmpDel
Expr / CN Spearman Corr. Rho = 0.359 , p = 0.00078
0.05
7 : 116164108
7 : 116164422
7 : 116164533
7 : 116164633
7 : 116164670
7 : 116164704
7 : 116164709
7 : 116164743
7 : 116164753
7 : 116164762
7 : 116164767
7 : 116164836
7 : 116164847
7 : 116164877
7 : 116164935
7 : 116164950
7 : 116165134
7 : 116165142
7 : 116165563
7 : 116165612
7 : 116165941
7 : 116166214
7 : 116166339
7 : 116166421
7 : 116166456
7 : 116166529
7 : 116166734
7 : 116166824
7 : 116180662
7 : 116182100
7 : 116188688
7 : 116191437
7 : 116193811
7 : 116199599
7 : 116200184
cg25515317
cg20897119
cg04474049
cg01265597
cg07838272
cg17469978
cg24980893
cg14582226
cg23973417
cg13522244
cg07964538
cg08028651
cg18329349
cg20811266
cg26687844
cg07925282
cg27242945
cg06327267
cg00308439
cg11691589
cg04677163
cg26839117
cg08526637
cg24770985
cg18721249
cg22126032
cg12296751
cg21367972
cg12877532
cg24987440
cg15558717
cg18498156
cg24060730
cg18209470
cg05232628
GeneLoc
PromoterAssoc
CpGIsland
0.25
0.01
mRNA / METH Corr. FDR
MITF
SOX10
TRPM1
ZEB1
AXL
0.05
0.01
Gene / Regulator Corr. P
Type
mRNA_Expr
CopyNum
AmpDel

AOX1

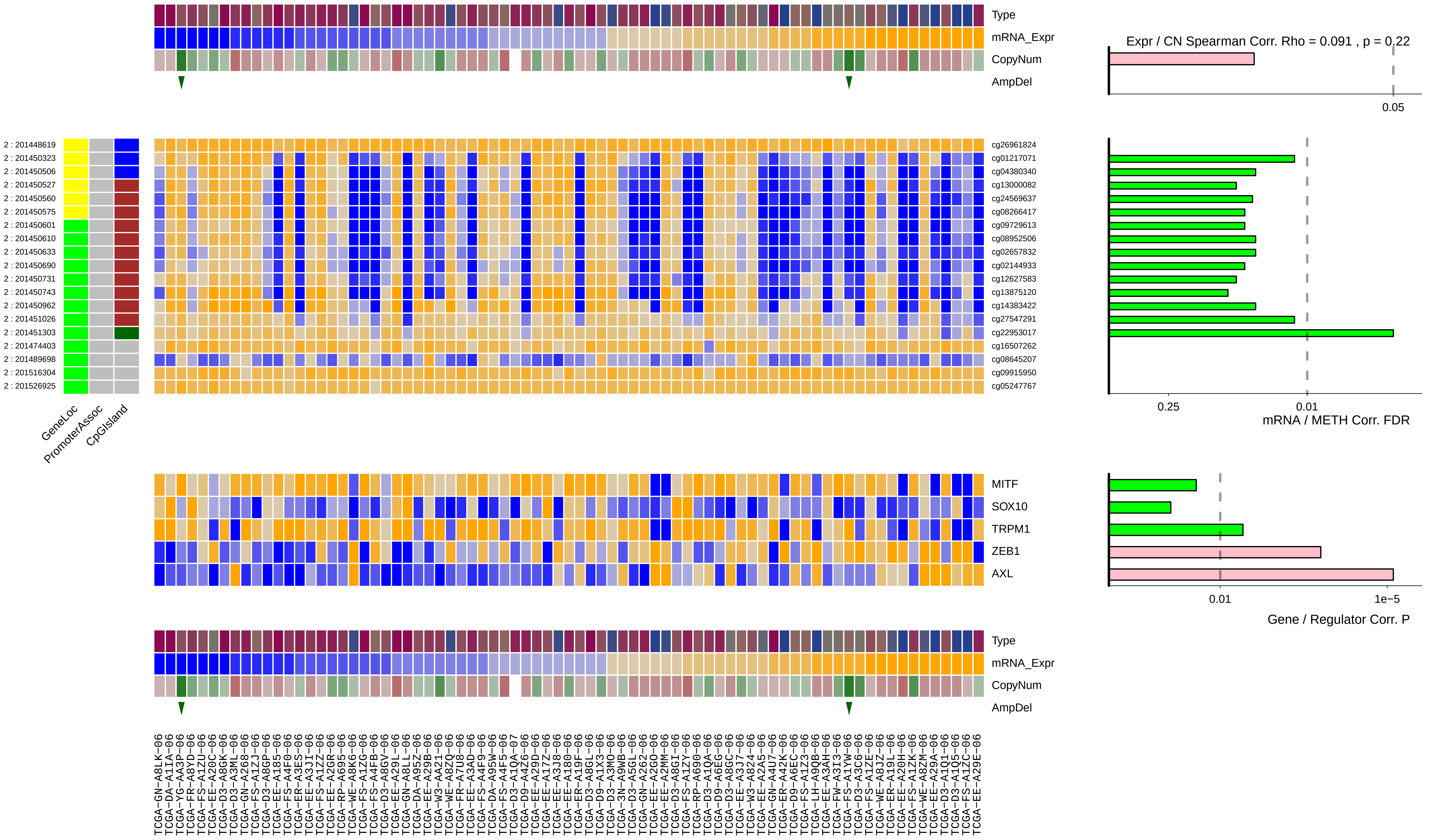

Type
mRNA_Expr
CopyNum
AmpDel
Expr / CN Spearman Corr. Rho = 0.091 , p = 0.22
0.05
cg26961824
cg01217071
cg04380340
cg13000082
cg24569637
cg08266417
cg09729613
cg08952506
cg02657832
cg02144933
cg12627583
cg13875120
cg14383422
cg27547291
cg22953017
cg16507262
cg08645207
cg09915950
cg05247767
2 : 201448619
2 : 201450323
2 : 201450506
2 : 201450527
2 : 201450560
2 : 201450575
2 : 201450601
2 : 201450610
2 : 201450633
2 : 201450690
2 : 201450731
2 : 201450743
2 : 201450962
2 : 201451026
2 : 201451303
2 : 201474403
2 : 201489698
2 : 201516304
2 : 201526925
GeneLoc
PromoterAssoc
CpGIsland
0.25
0.01
mRNA / METH Corr. FDR
MITF
SOX10
TRPM1
ZEB1
AXL
0.01
1e−5
Gene / Regulator Corr. P

PTGFR

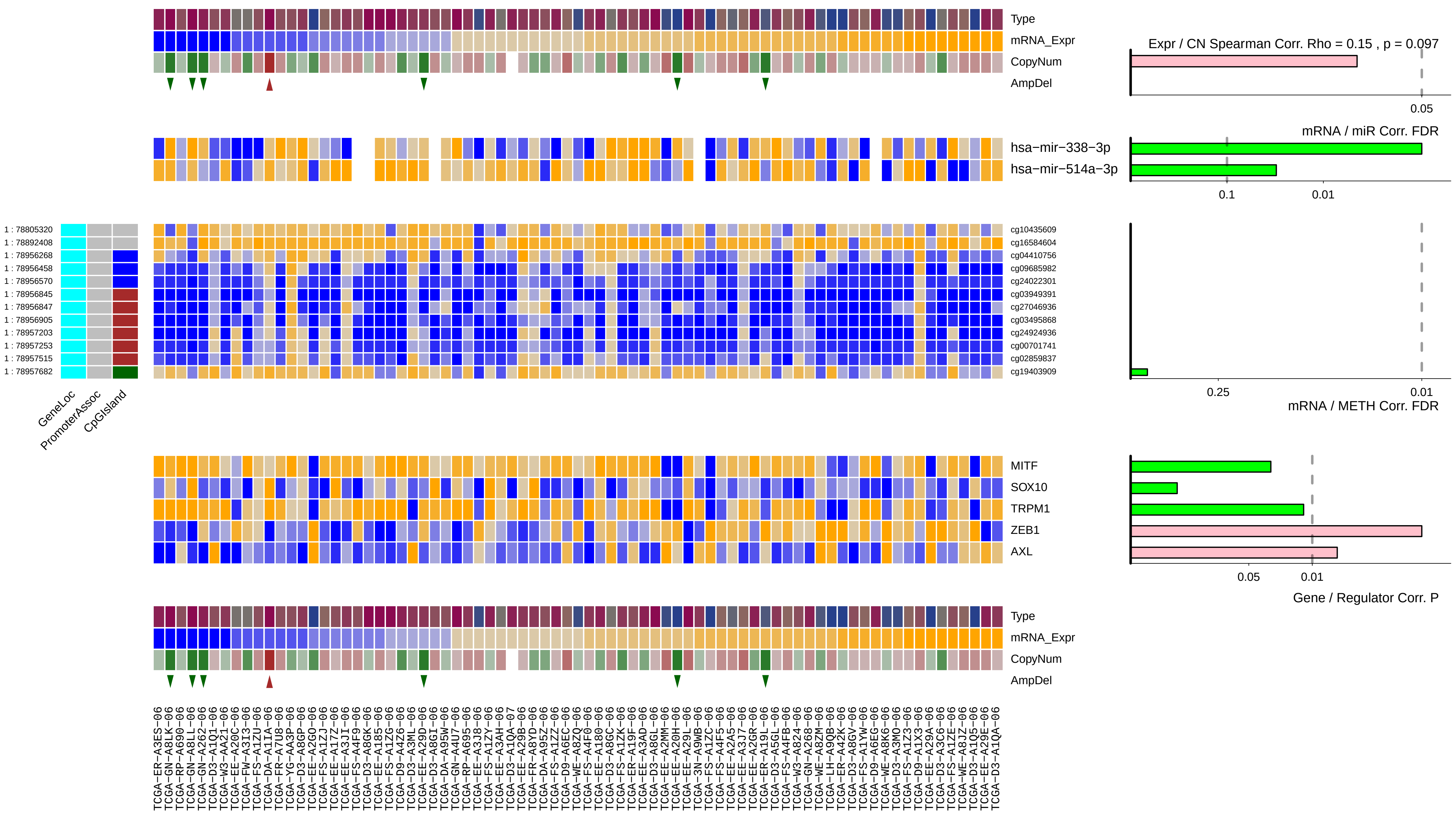
Type
mRNA_Expr
CopyNum
AmpDel
Expr / CN Spearman Corr. Rho = 0.15 , p = 0.097
0.05
mRNA / miR Corr. FDR
hsa−mir−338−3p
hsa−mir−514a−3p
0.1
0.01
1 : 78805320
1 : 78892408
1 : 78956268
1 : 78956458
1 : 78956570
1 : 78956845
1 : 78956847
1 : 78956905
1 : 78957203
1 : 78957253
1 : 78957515
1 : 78957682
cg10435609
cg16584604
cg04410756
cg09685982
cg24022301
cg03949391
cg27046936
cg03495868
cg24924936
cg00701741
cg02859837
cg19403909
GeneLoc
PromoterAssoc
CpGIsland
0.25
0.01
mRNA / METH Corr. FDR
MITF
SOX10
TRPM1
ZEB1
AXL
0.05
0.01
Gene / Regulator Corr. P
Type
mRNA_Expr
CopyNum
AmpDel

ALPK2

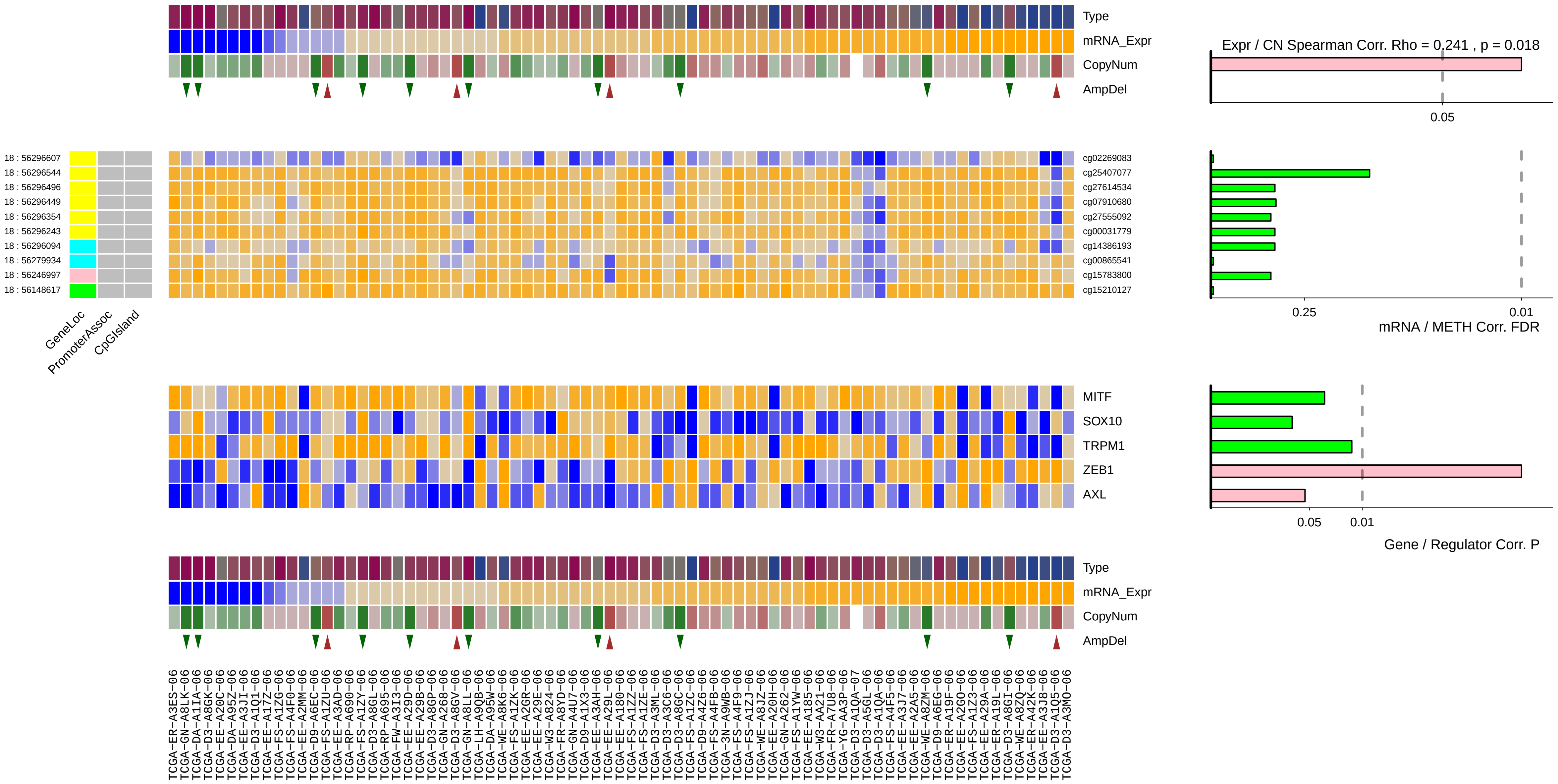

Type
mRNA_Expr
CopyNum
AmpDel
Expr / CN Spearman Corr. Rho = 0.241 , p = 0.018
0.05
18 : 56296607
18 : 56296544
18 : 56296496
18 : 56296449
18 : 56296354
18 : 56296243
18 : 56296094
18 : 56279934
18 : 56246997
18 : 56148617
GeneLoc
PromoterAssoc
CpGIsland
cg02269083
cg25407077
cg27614534
cg07910680
cg27555092
cg00031779
cg14386193
cg00865541
cg15783800
cg15210127
0.25
0.01
mRNA / METH Corr. FDR
MITF
SOX10
TRPM1
ZEB1
AXL
0.05
0.01
Gene / Regulator Corr. P

CFH

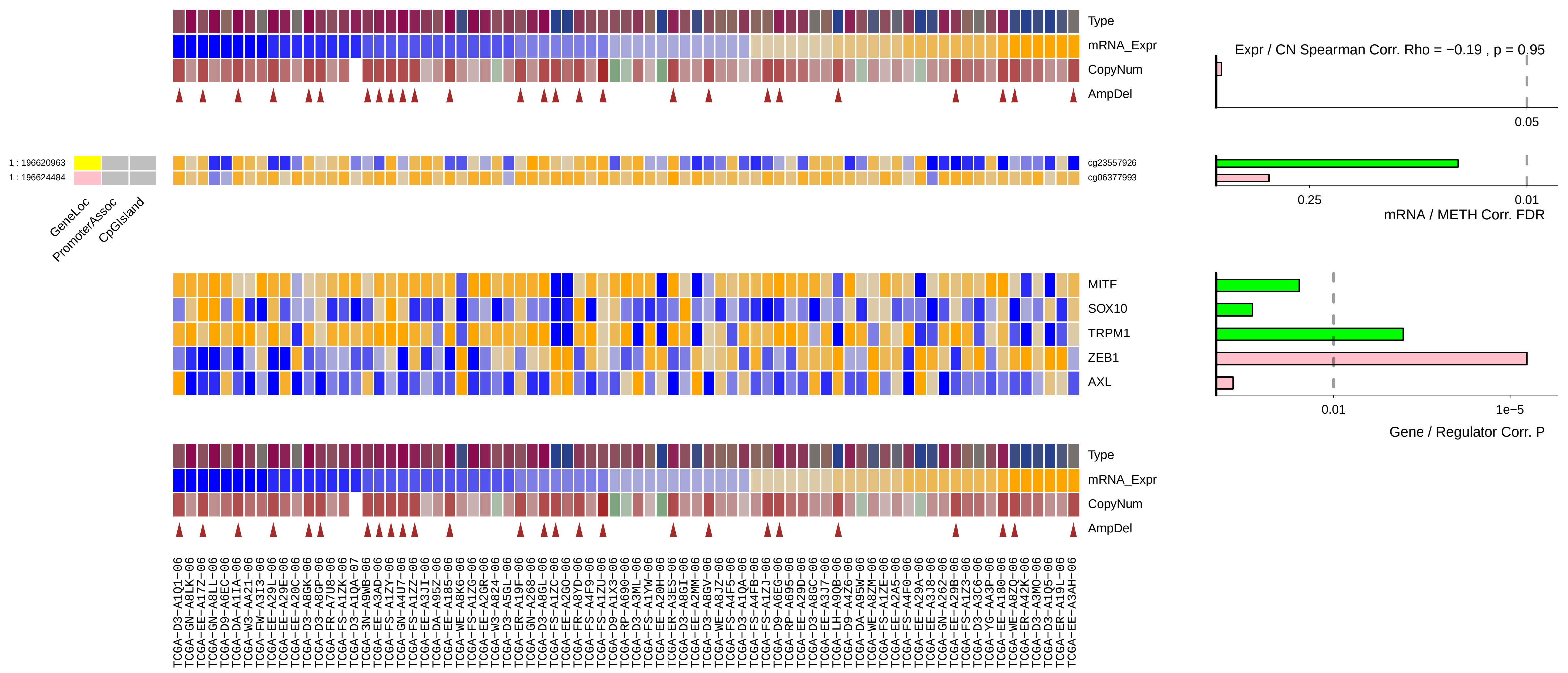

Type
mRNA_Expr
CopyNum
AmpDel
Expr / CN Spearman Corr. Rho = −0.19 , p = 0.95
0.05
1 : 196620963
1 : 196624484
GeneLoc
PromoterAssoc
CpGIsland
cg23557926
cg06377993
0.25
0.01
mRNA / METH Corr. FDR
MITF
SOX10
TRPM1
ZEB1
AXL
0.01
1e−5
Gene / Regulator Corr. P

LPAR1

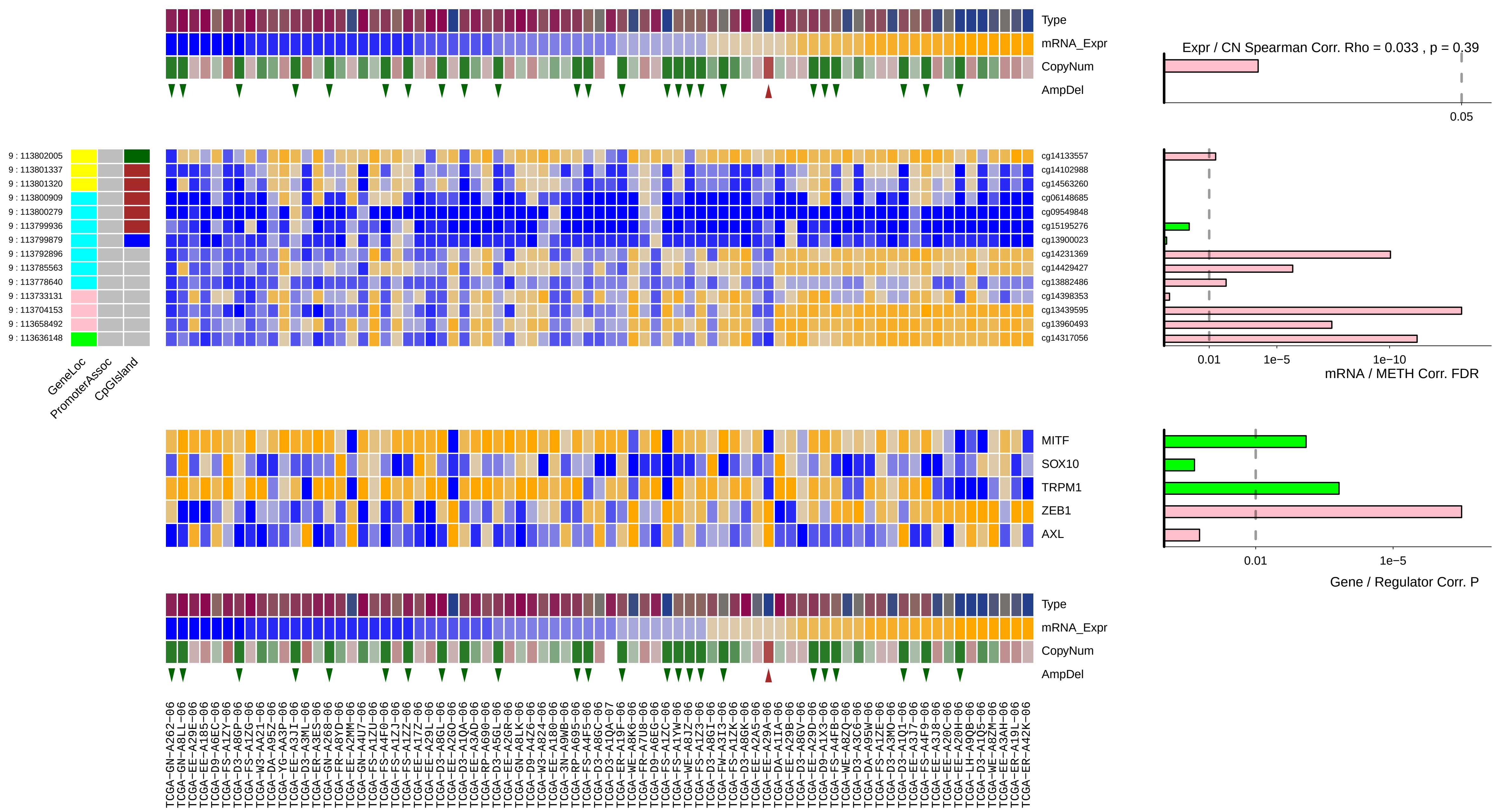
Type
mRNA_Expr
CopyNum
AmpDel
Expr / CN Spearman Corr. Rho = 0.033 , p = 0.39
0.05
9 : 113802005
9 : 113801337
9 : 113801320
9 : 113800909
9 : 113800279
9 : 113799936
9 : 113799879
9 : 113792896
9 : 113785563
9 : 113778640
9 : 113733131
9 : 113704153
9 : 113658492
9 : 113636148
GeneLoc
PromoterAssoc
CpGIsland
cg14133557
cg14102988
cg14563260
cg06148685
cg09549848
cg15195276
cg13900023
cg14231369
cg14429427
cg13882486
cg14398353
cg13439595
cg13960493
cg14317056
0.01
1e−5
1e−10
mRNA / METH Corr. FDR
MITF
SOX10
TRPM1
ZEB1
AXL
0.01
1e−5
Gene / Regulator Corr. P
Type
mRNA_Expr
CopyNum
AmpDel

F3

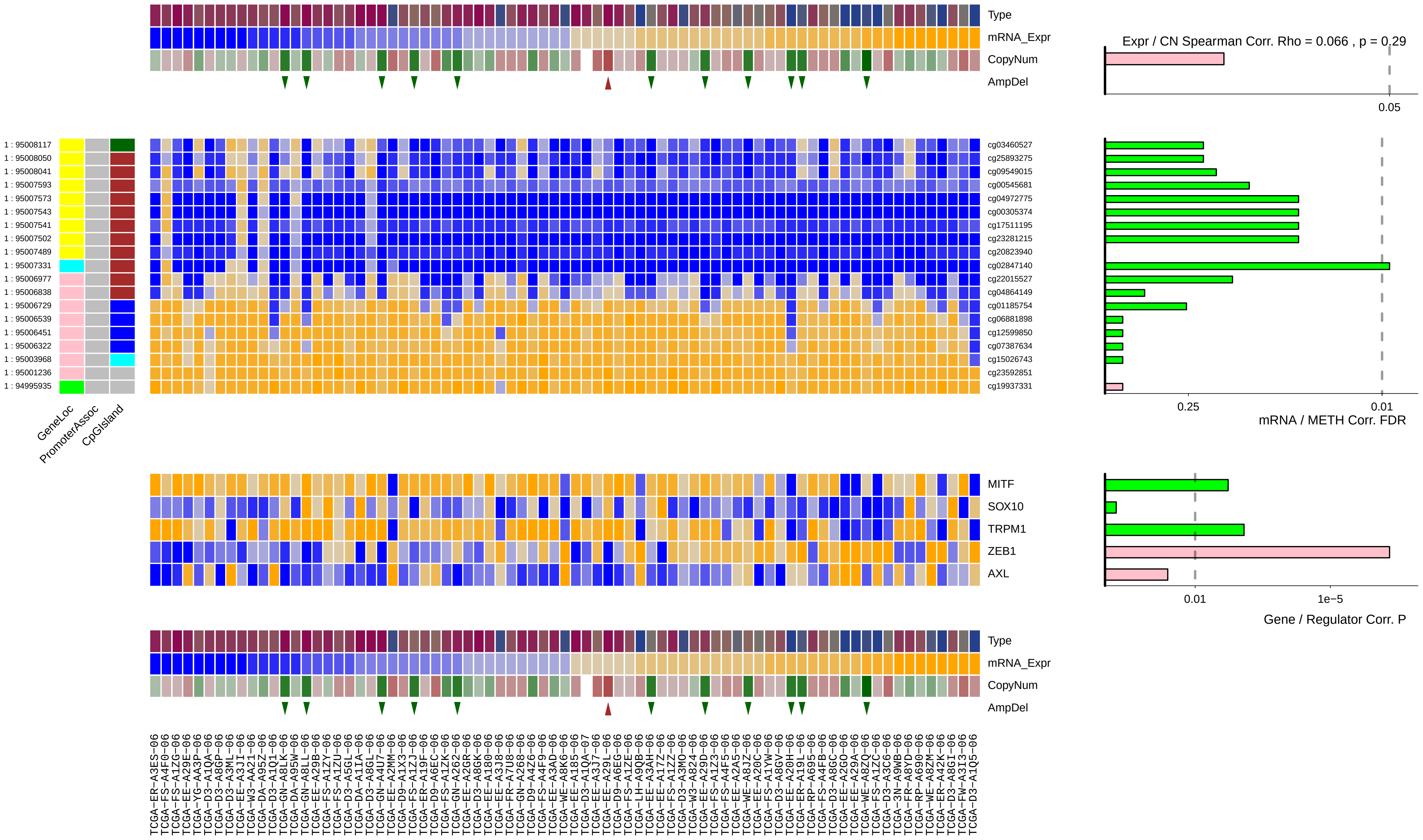
Type
mRNA_Expr
CopyNum
AmpDel
Expr / CN Spearman Corr. Rho = 0.066 , p = 0.29
0.05
1 : 95008117
1 : 95008050
1 : 95008041
1 : 95007593
1 : 95007573
1 : 95007543
1 : 95007541
1 : 95007502
1 : 95007489
1 : 95007331
1 : 95006977
1 : 95006838
1 : 95006729
1 : 95006539
1 : 95006451
1 : 95006322
1 : 95003968
1 : 95001236
1 : 94995935
GeneLoc
PromoterAssoc
CpGIsland
cg03460527
cg25893275
cg09549015
cg00545681
cg04972775
cg00305374
cg17511195
cg23281215
cg20823940
cg02847140
cg22015527
cg04864149
cg01185754
cg06881898
cg12599850
cg07387634
cg15026743
cg23592851
cg19937331
0.25
0.01
mRNA / METH Corr. FDR
MITF
SOX10
TRPM1
ZEB1
AXL
0.01
1e−5
Gene / Regulator Corr. P

CNN2

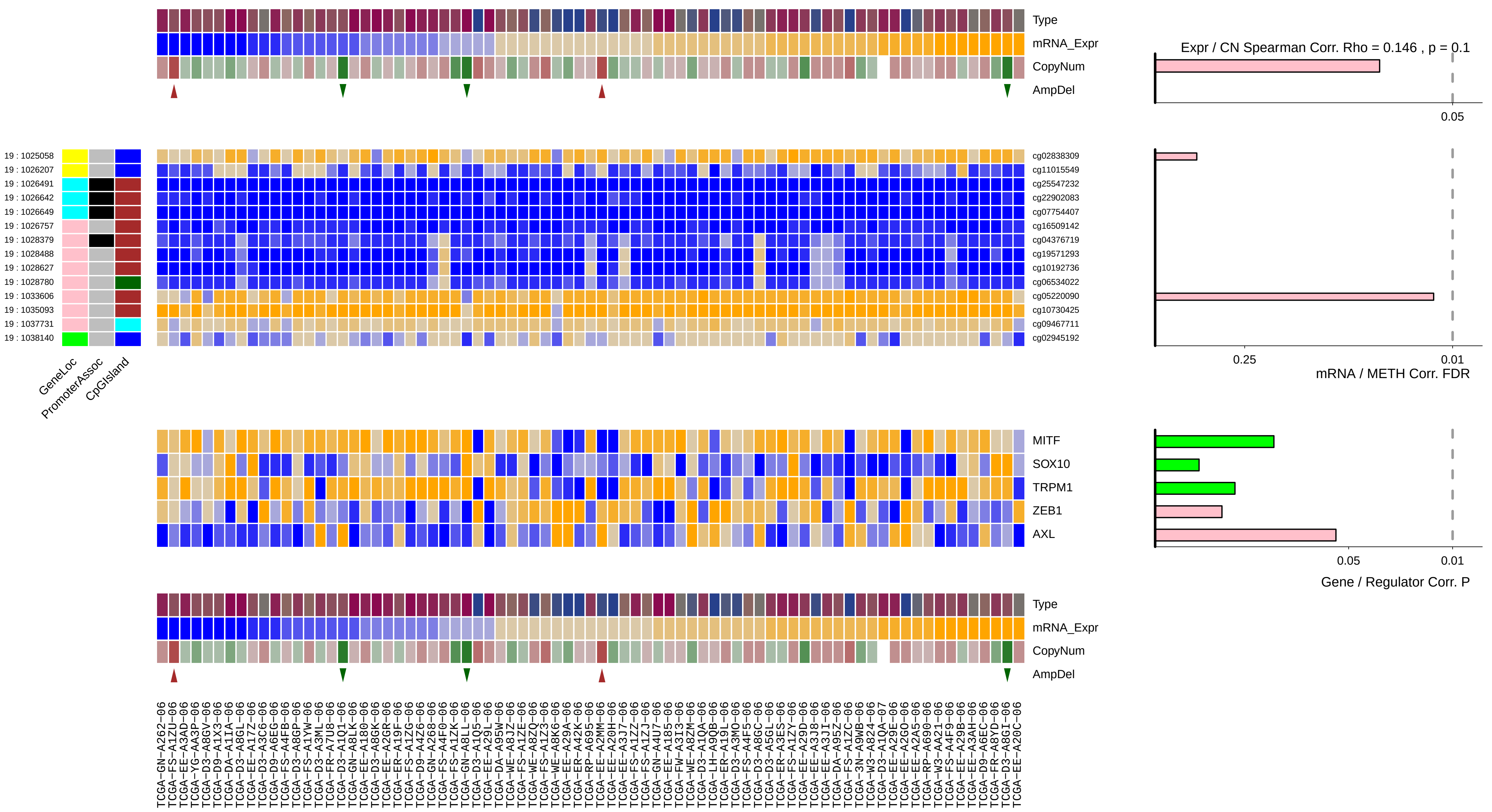
Type
mRNA_Expr
CopyNum
AmpDel
Expr / CN Spearman Corr. Rho = 0.146 , p = 0.1
0.05
19 : 1025058
19 : 1026207
19 : 1026491
19 : 1026642
19 : 1026649
19 : 1026757
19 : 1028379
19 : 1028488
19 : 1028627
19 : 1028780
19 : 1033606
19 : 1035093
19 : 1037731
19 : 1038140
cg02838309
cg11015549
cg25547232
cg22902083
cg07754407
cg16509142
cg04376719
cg19571293
cg10192736
cg06534022
cg05220090
cg10730425
cg09467711
cg02945192
GeneLoc
PromoterAssoc
CpGIsland
0.25
0.01
mRNA / METH Corr. FDR
MITF
SOX10
TRPM1
ZEB1
AXL
0.05
0.01
Gene / Regulator Corr. P
Type
mRNA_Expr
CopyNum
AmpDel

MAP1B

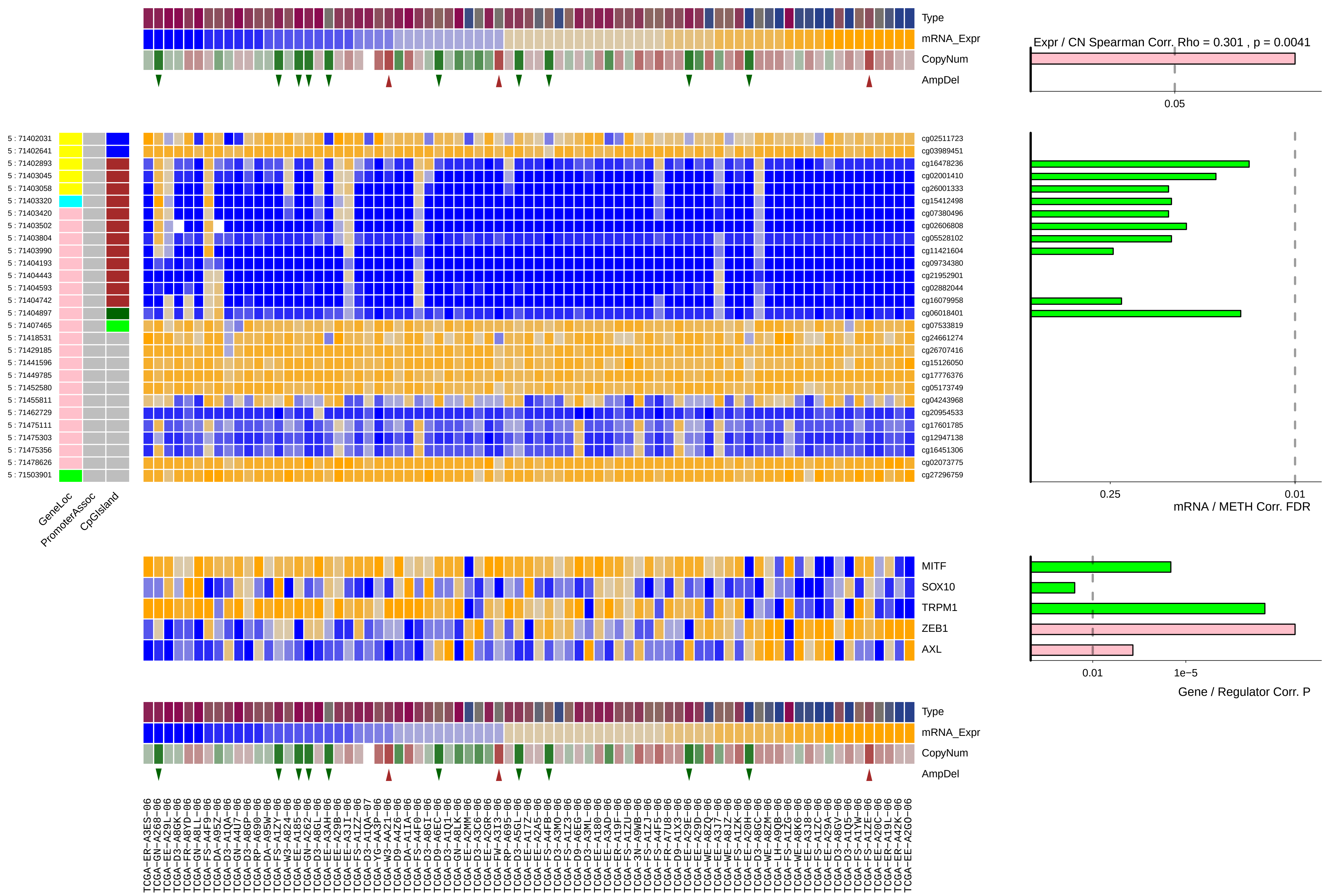

Type
mRNA_Expr
CopyNum
AmpDel
Expr / CN Spearman Corr. Rho = 0.301 , p = 0.0041
0.05
5 : 71402031
5 : 71402641
5 : 71402893
5 : 71403045
5 : 71403058
5 : 71403320
5 : 71403420
5 : 71403502
5 : 71403804
5 : 71403990
5 : 71404193
5 : 71404443
5 : 71404593
5 : 71404742
5 : 71404897
5 : 71407465
5 : 71418531
5 : 71429185
5 : 71441596
5 : 71449785
5 : 71452580
5 : 71455811
5 : 71462729
5 : 71475111
5 : 71475303
5 : 71475356
5 : 71478626
5 : 71503901
cg02511723
cg03989451
cg16478236
cg02001410
cg26001333
cg15412498
cg07380496
cg02606808
cg05528102
cg11421604
cg09734380
cg21952901
cg02882044
cg16079958
cg06018401
cg07533819
cg24661274
cg26707416
cg15126050
cg17776376
cg05173749
cg04243968
cg20954533
cg17601785
cg12947138
cg16451306
cg02073775
cg27296759
GeneLoc
PromoterAssoc
CpGIsland
0.25
0.01
mRNA / METH Corr. FDR
MITF
SOX10
TRPM1
ZEB1
AXL
0.01
1e−5
Gene / Regulator Corr. P
Type
mRNA_Expr
CopyNum
AmpDel

ITGB1

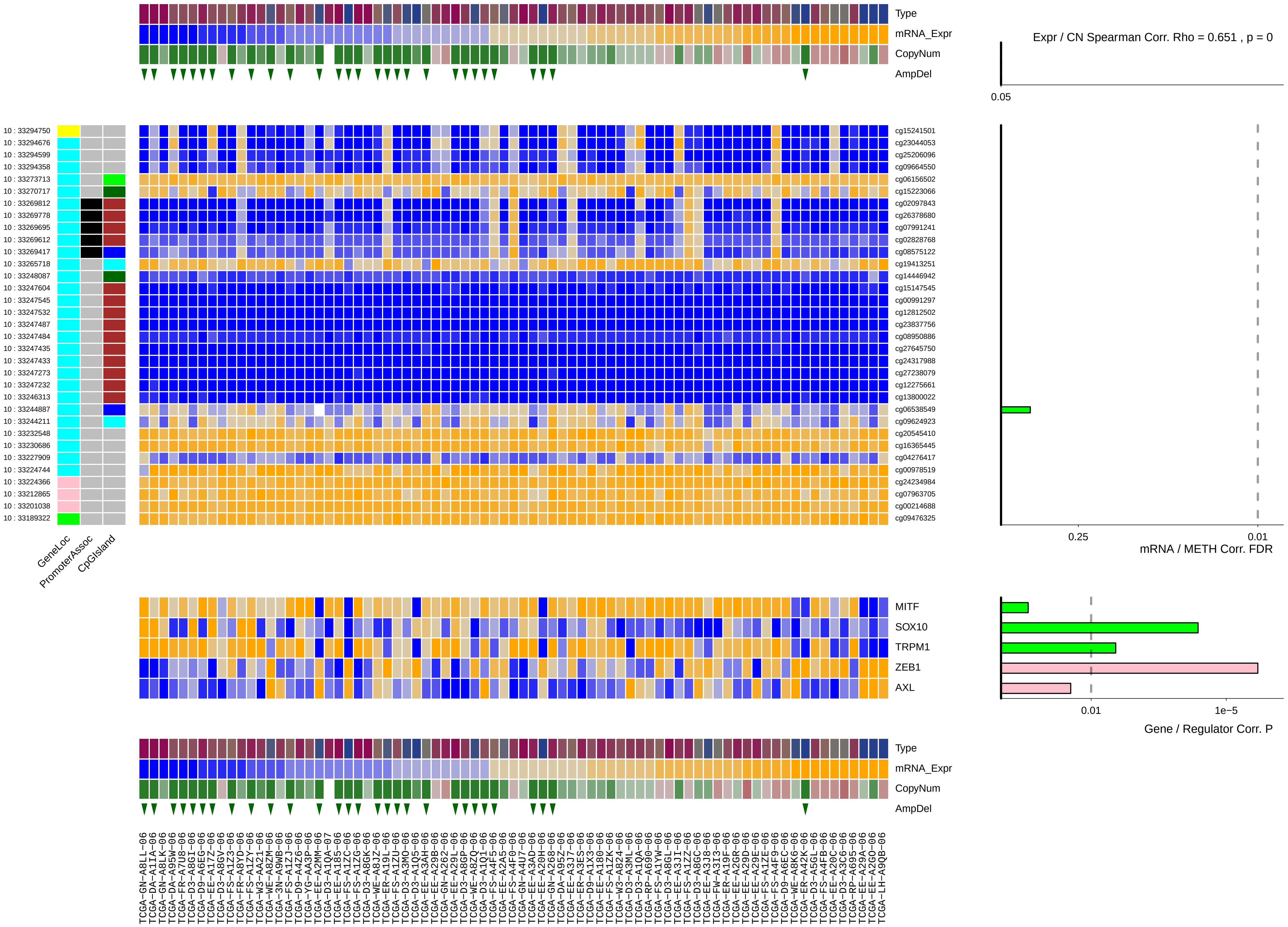
Type
mRNA_Expr
CopyNum
AmpDel
Expr / CN Spearman Corr. Rho = 0.651 , p = 0
0.05
10 : 33294750
10 : 33294676
10 : 33294599
10 : 33294358
10 : 33273713
10 : 33270717
10 : 33269812
10 : 33269778
10 : 33269695
10 : 33269612
10 : 33269417
10 : 33265718
10 : 33248087
10 : 33247604
10 : 33247545
10 : 33247532
10 : 33247487
10 : 33247484
10 : 33247435
10 : 33247433
10 : 33247273
10 : 33247232
10 : 33246313
10 : 33244887
10 : 33244211
10 : 33232548
10 : 33230686
10 : 33227909
10 : 33224744
10 : 33224366
10 : 33212865
10 : 33201038
10 : 33189322
cg15241501
cg23044053
cg25206096
cg09664550
cg06156502
cg15223066
cg02097843
cg26378680
cg07991241
cg02828768
cg08575122
cg19413251
cg14446942
cg15147545
cg00991297
cg12812502
cg23837756
cg08950886
cg27645750
cg24317988
cg27238079
cg12275661
cg13800022
cg06538549
cg09624923
cg20545410
cg16365445
cg04276417
cg00978519
cg24234984
cg07963705
cg00214688
cg09476325
GeneLoc
PromoterAssoc
CpGIsland
0.25
0.01
mRNA / METH Corr. FDR
MITF
SOX10
TRPM1
ZEB1
AXL
0.01
1e−5
Gene / Regulator Corr. P
Type
mRNA_Expr
CopyNum
AmpDel

VCAN

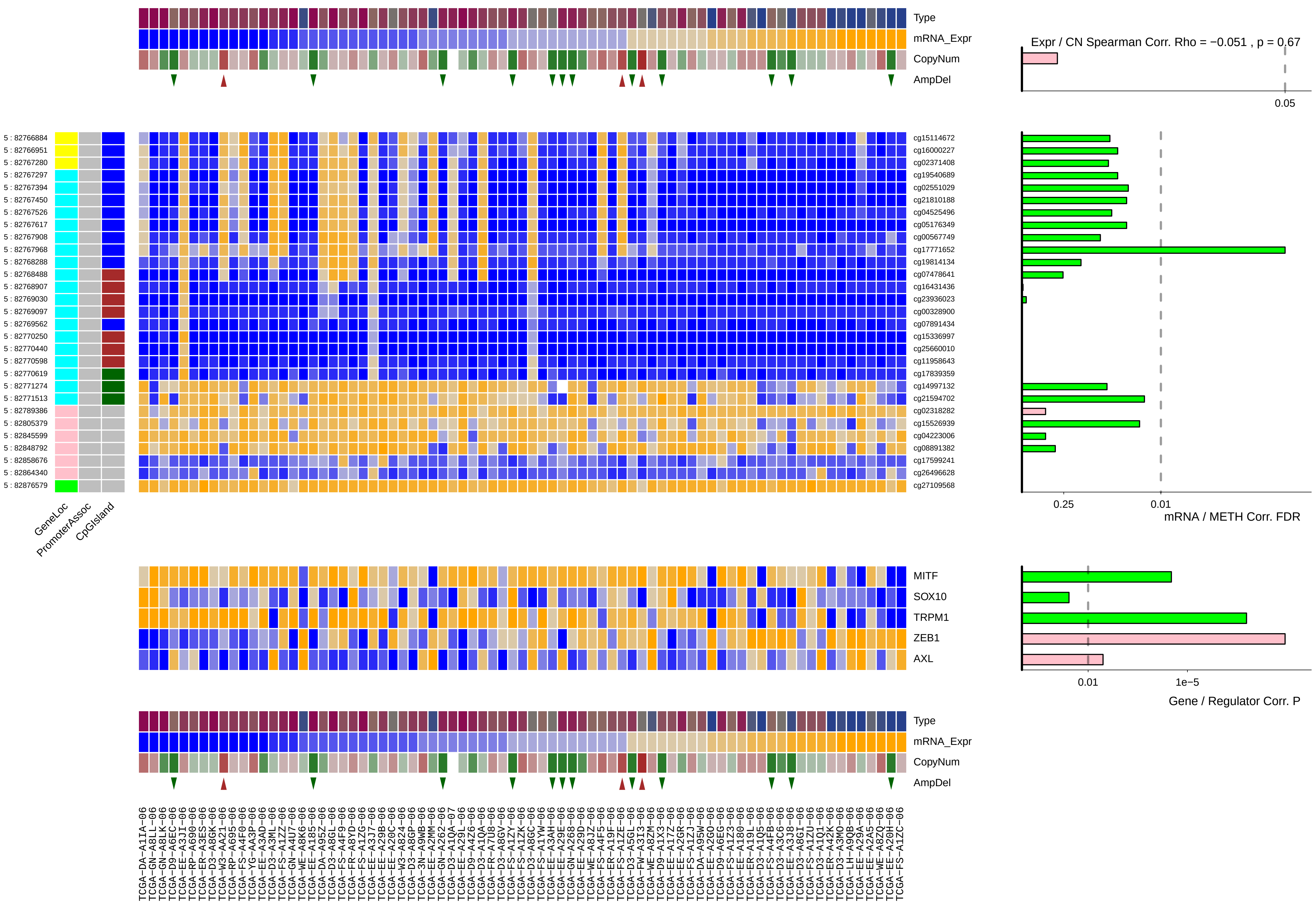

Type
mRNA_Expr
CopyNum
AmpDel
Expr / CN Spearman Corr. Rho = −0.051 , p = 0.67
0.05
5 : 82766884
5 : 82766951
5 : 82767280
5 : 82767297
5 : 82767394
5 : 82767450
5 : 82767526
5 : 82767617
5 : 82767908
5 : 82767968
5 : 82768288
5 : 82768488
5 : 82768907
5 : 82769030
5 : 82769097
5 : 82769562
5 : 82770250
5 : 82770440
5 : 82770598
5 : 82770619
5 : 82771274
5 : 82771513
5 : 82789386
5 : 82805379
5 : 82845599
5 : 82848792
5 : 82858676
5 : 82864340
5 : 82876579
cg15114672
cg16000227
cg02371408
cg19540689
cg02551029
cg21810188
cg04525496
cg05176349
cg00567749
cg17771652
cg19814134
cg07478641
cg16431436
cg23936023
cg00328900
cg07891434
cg15336997
cg25660010
cg11958643
cg17839359
cg14997132
cg21594702
cg02318282
cg15526939
cg04223006
cg08891382
cg17599241
cg26496628
cg27109568
0.25
0.01
mRNA / METH Corr. FDR
GeneLoc
PromoterAssoc
CpGIsland
MITF
SOX10
TRPM1
ZEB1
AXL
0.01
1e−5
Gene / Regulator Corr. P
Type
mRNA_Expr
CopyNum
AmpDel

NNMT

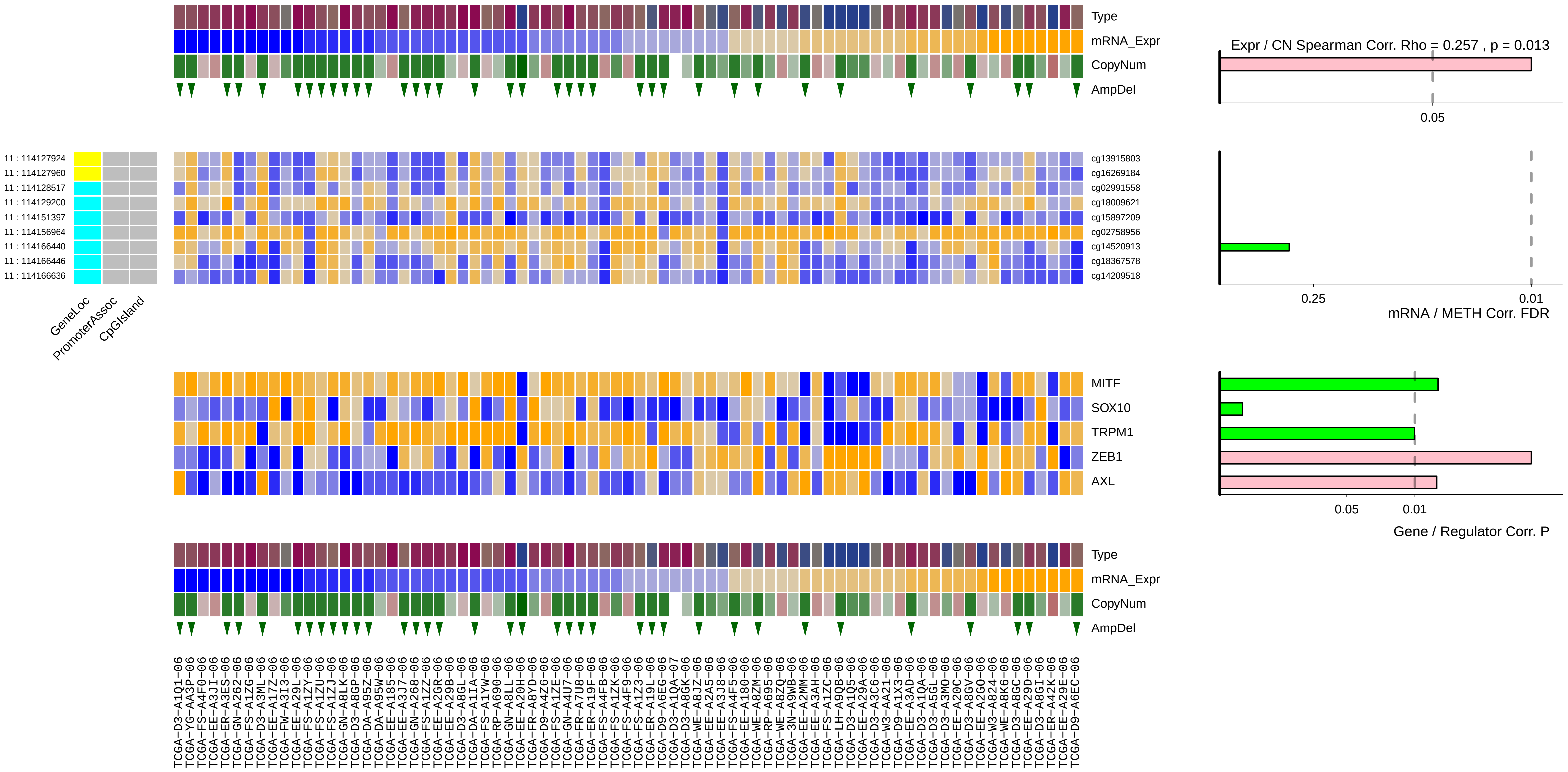

Type
mRNA_Expr
CopyNum
AmpDel
Expr / CN Spearman Corr. Rho = 0.257 , p = 0.013
0.05
11 : 114127924
11 : 114127960
11 : 114128517
11 : 114129200
11 : 114151397
11 : 114156964
11 : 114166440
11 : 114166446
11 : 114166636
GeneLoc
PromoterAssoc
CpGIsland
cg13915803
cg16269184
cg02991558
cg18009621
cg15897209
cg02758956
cg14520913
cg18367578
cg14209518
0.25
0.01
mRNA / METH Corr. FDR
MITF
SOX10
TRPM1
ZEB1
AXL
0.05
0.01
Gene / Regulator Corr. P

TNFAIP2

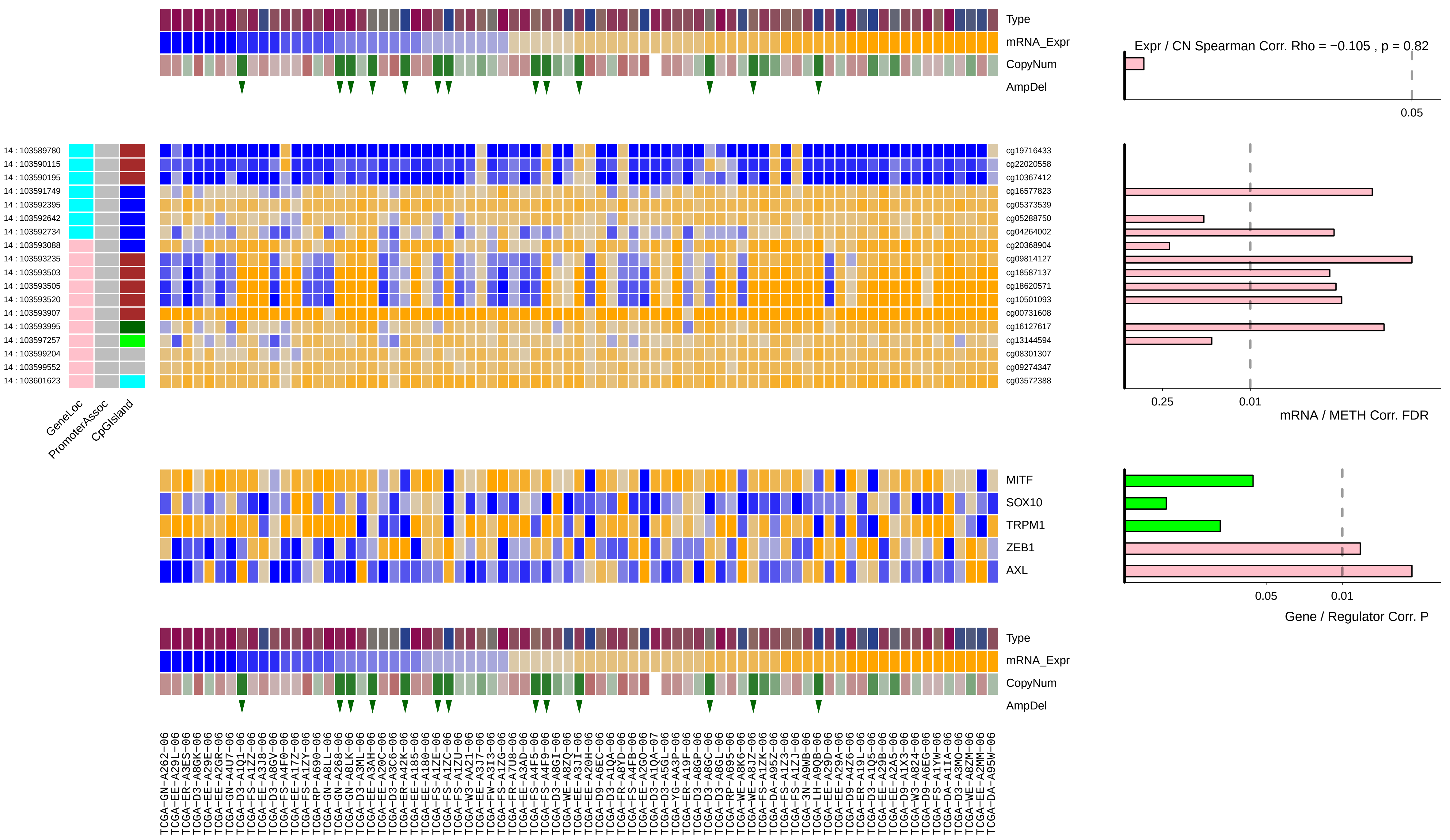
Type
mRNA_Expr
CopyNum
AmpDel
Expr / CN Spearman Corr. Rho = −0.105 , p = 0.82
0.05
cg19716433
cg22020558
cg10367412
cg16577823
cg05373539
cg05288750
cg04264002
cg20368904
cg09814127
cg18587137
cg18620571
cg10501093
cg00731608
cg16127617
cg13144594
cg08301307
cg09274347
cg03572388
14 : 103589780
14 : 103590115
14 : 103590195
14 : 103591749
14 : 103592395
14 : 103592642
14 : 103592734
14 : 103593088
14 : 103593235
14 : 103593503
14 : 103593505
14 : 103593520
14 : 103593907
14 : 103593995
14 : 103597257
14 : 103599204
14 : 103599552
14 : 103601623
GeneLoc
PromoterAssoc
CpGIsland
0.25
0.01
mRNA / METH Corr. FDR
MITF
SOX10
TRPM1
ZEB1
AXL
0.05
0.01
Gene / Regulator Corr. P

ITGA5

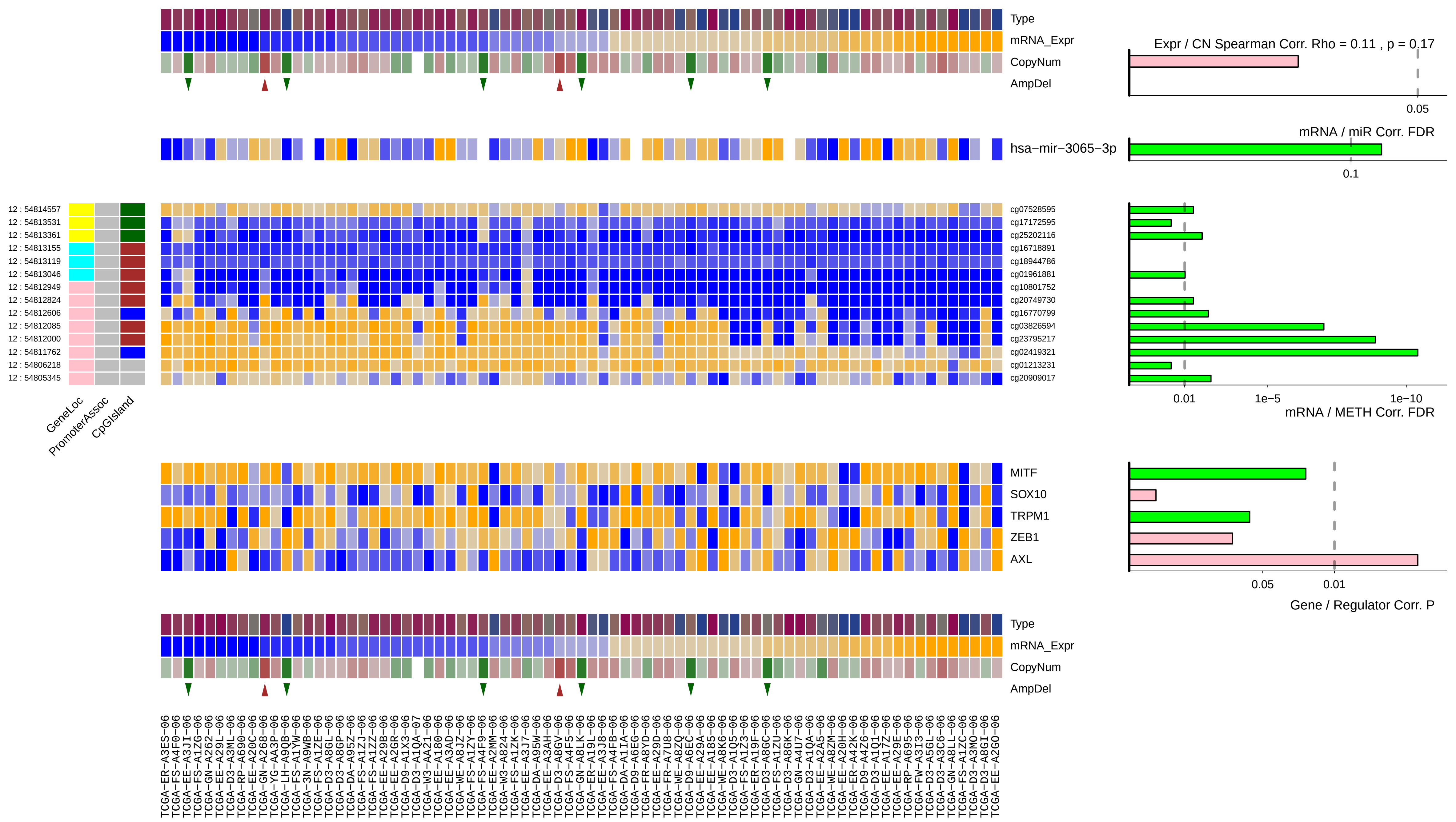
Type
mRNA_Expr
CopyNum
AmpDel
Expr / CN Spearman Corr. Rho = 0.11 , p = 0.17
0.05
hsa−mir−3065−3p
mRNA / miR Corr. FDR
0.1
cg07528595
cg17172595
cg25202116
cg16718891
cg18944786
cg01961881
cg10801752
cg20749730
cg16770799
cg03826594
cg23795217
cg02419321
cg01213231
cg20909017
12 : 54814557
12 : 54813531
12 : 54813361
12 : 54813155
12 : 54813119
12 : 54813046
12 : 54812949
12 : 54812824
12 : 54812606
12 : 54812085
12 : 54812000
12 : 54811762
12 : 54806218
12 : 54805345
GeneLoc
PromoterAssoc
CpGIsland
0.01
1e−5
1e−10
mRNA / METH Corr. FDR
MITF
SOX10
TRPM1
ZEB1
AXL
0.05
0.01
Gene / Regulator Corr. P
Type
mRNA_Expr
CopyNum
AmpDel

FSCN1

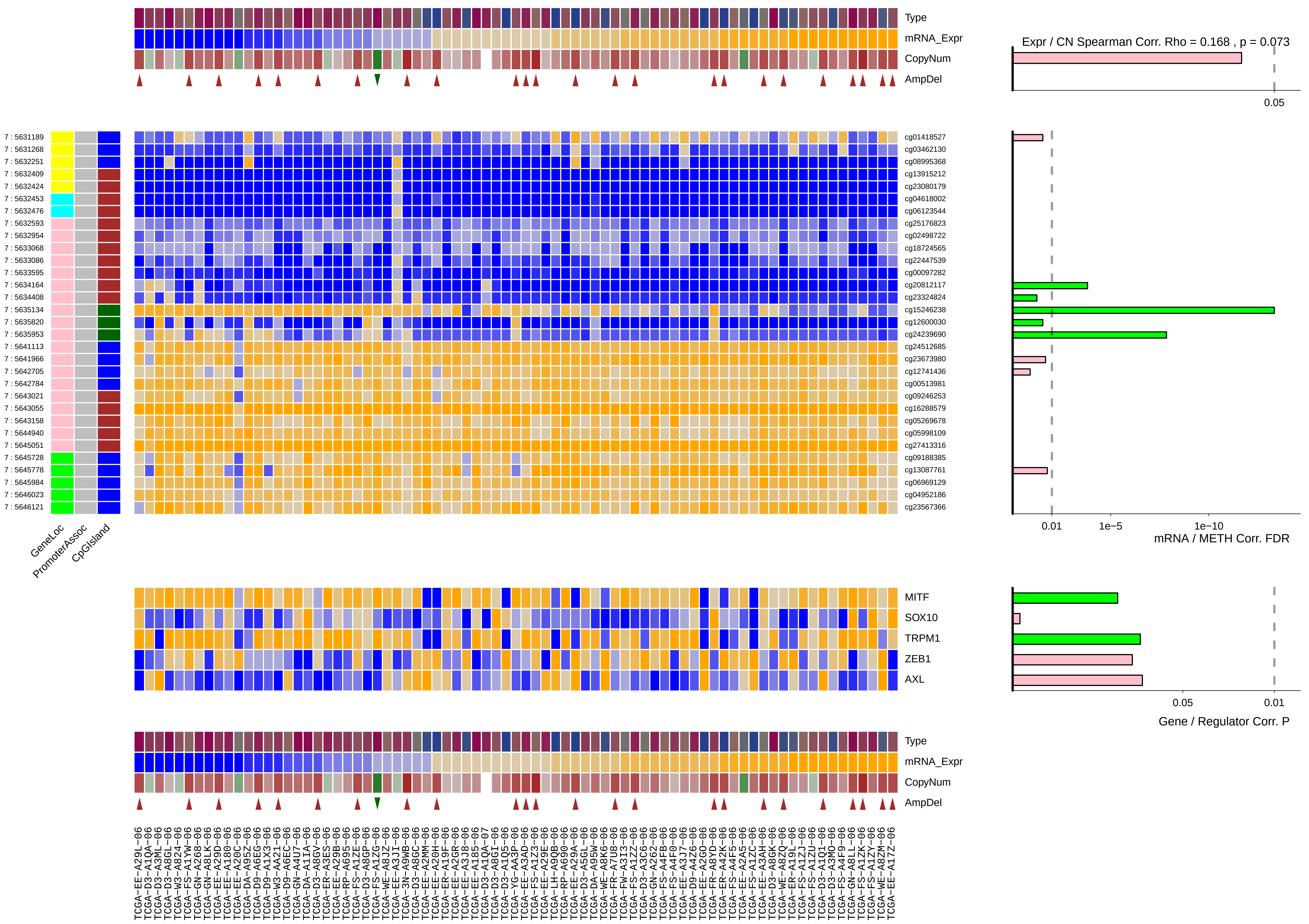
Type
mRNA_Expr
CopyNum
AmpDel
Expr / CN Spearman Corr. Rho = 0.168 , p = 0.073
0.05
GeneLoc
PromoterAssoc
CpGIsland
mRNA / METH Corr. FDR
0.01
1e−5
1e−10
MITF
SOX10
TRPM1
ZEB1
AXL
Gene / Regulator Corr. P

EDIL3

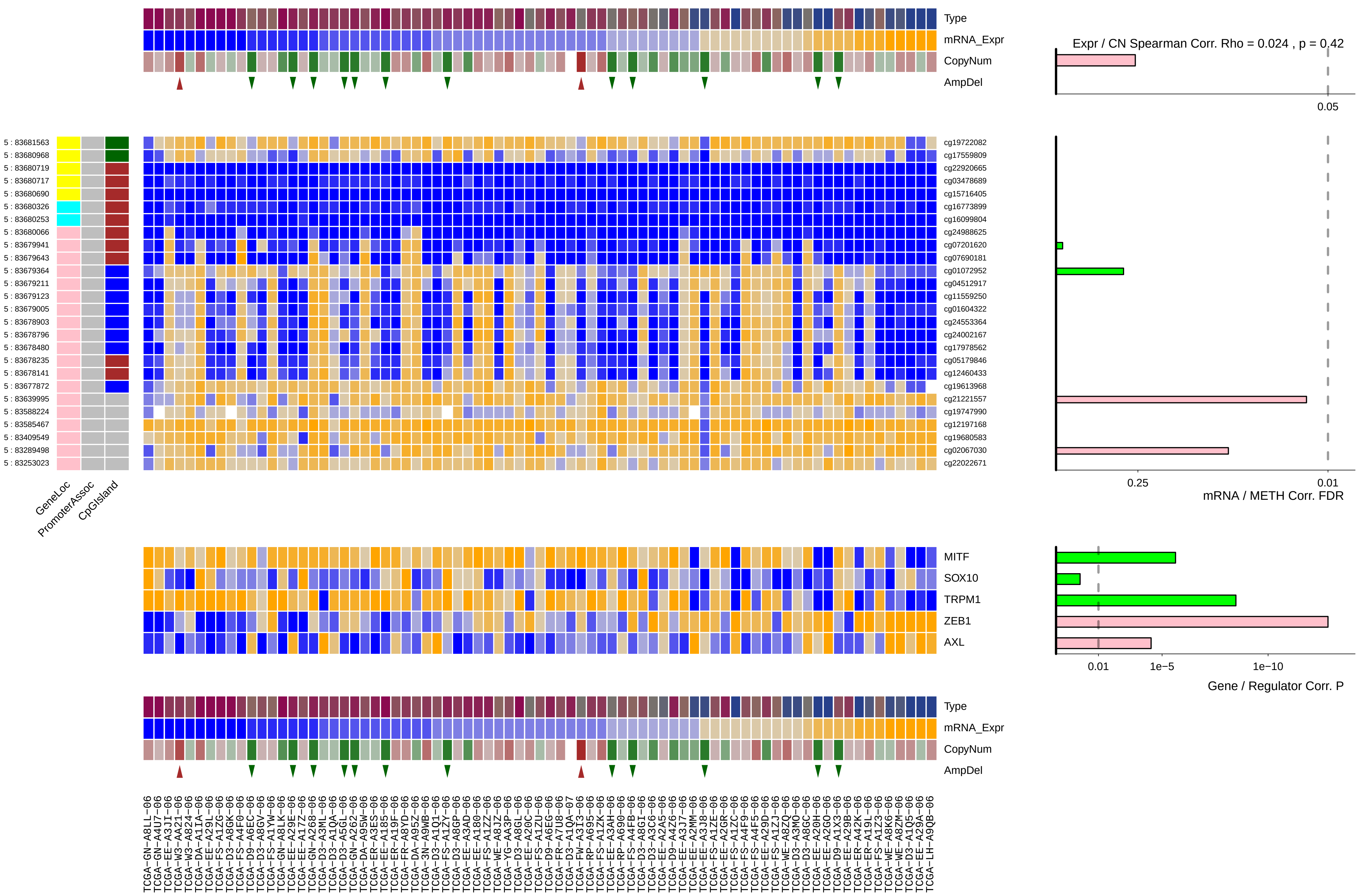

Type
mRNA_Expr
CopyNum
AmpDel
Expr / CN Spearman Corr. Rho = 0.024 , p = 0.42
0.05
GeneLoc
PromoterAssoc
CpGIsland
0.25
0.01
mRNA / METH Corr. FDR
MITF
SOX10
TRPM1
ZEB1
AXL
0.01
1e−5
1e−10
Gene / Regulator Corr. P

VGLL3

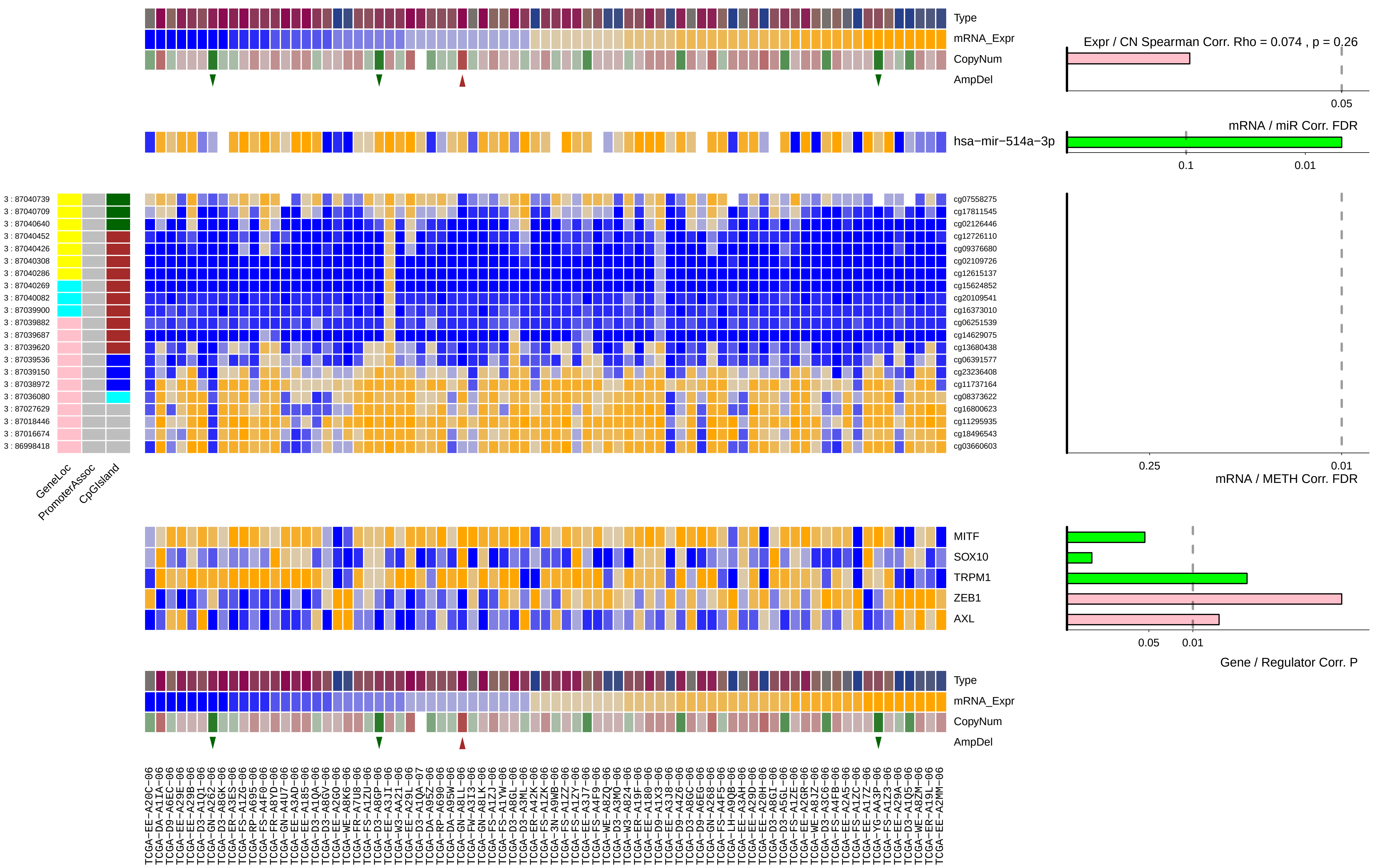
Type
mRNA_Expr
CopyNum
AmpDel
Expr / CN Spearman Corr. Rho = 0.074 , p = 0.26
0.05
hsa−mir−514a−3p
mRNA / miR Corr. FDR
0.1
0.01
GeneLoc
PromoterAssoc
CpGIsland
0.25
0.01
mRNA / METH Corr. FDR
MITF
SOX10
TRPM1
ZEB1
AXL
0.05
0.01
Gene / Regulator Corr. P

DDAH1

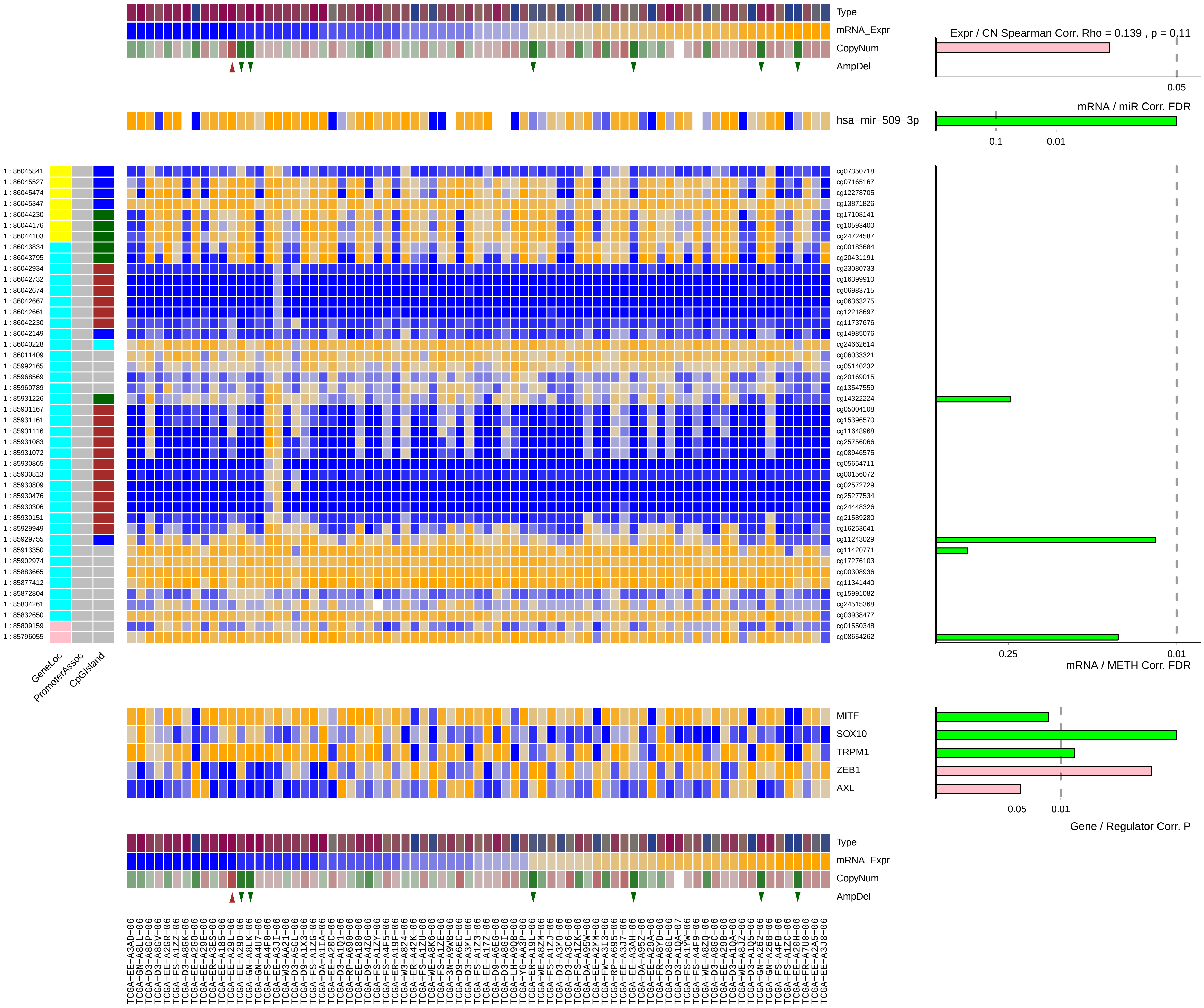

Type
mRNA_Expr
CopyNum
AmpDel
Expr / CN Spearman Corr. Rho = 0.139 , p = 0.11
0.05
hsa−mir−509−3p
mRNA / miR Corr. FDR
0.1
0.01
0.25
0.01
mRNA / METH Corr. FDR
GeneLoc
PromoterAssoc
CpGIsland
MITF
SOX10
TRPM1
ZEB1
AXL
0.05
0.01
Gene / Regulator Corr. P

ATP8B1

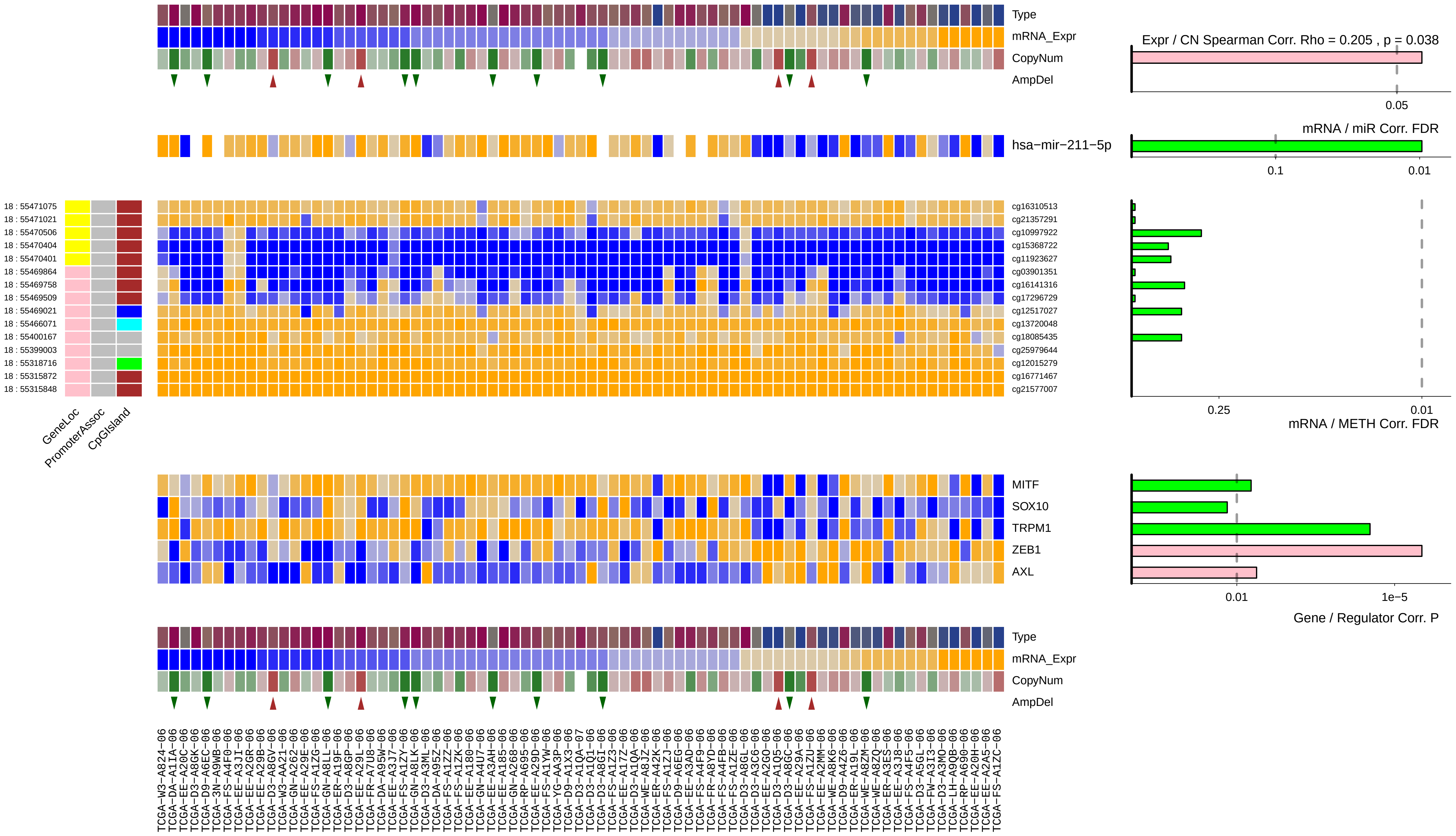

Type
mRNA_Expr
CopyNum
AmpDel
Expr / CN Spearman Corr. Rho = 0.205 , p = 0.038
0.05
hsa−mir−211−5p
mRNA / miR Corr. FDR
0.1
0.01
18 : 55471075
18 : 55471021
18 : 55470506
18 : 55470404
18 : 55470401
18 : 55469864
18 : 55469758
18 : 55469509
18 : 55469021
18 : 55466071
18 : 55400167
18 : 55399003
18 : 55318716
18 : 55315872
18 : 55315848
GeneLoc
PromoterAssoc
CpGIsland
cg16310513
cg21357291
cg10997922
cg15368722
cg11923627
cg03901351
cg16141316
cg17296729
cg12517027
cg13720048
cg18085435
cg25979644
cg12015279
cg16771467
cg21577007
0.25
0.01
mRNA / METH Corr. FDR
MITF
SOX10
TRPM1
ZEB1
AXL
0.01
1e−5
Gene / Regulator Corr. P

AMOTL2

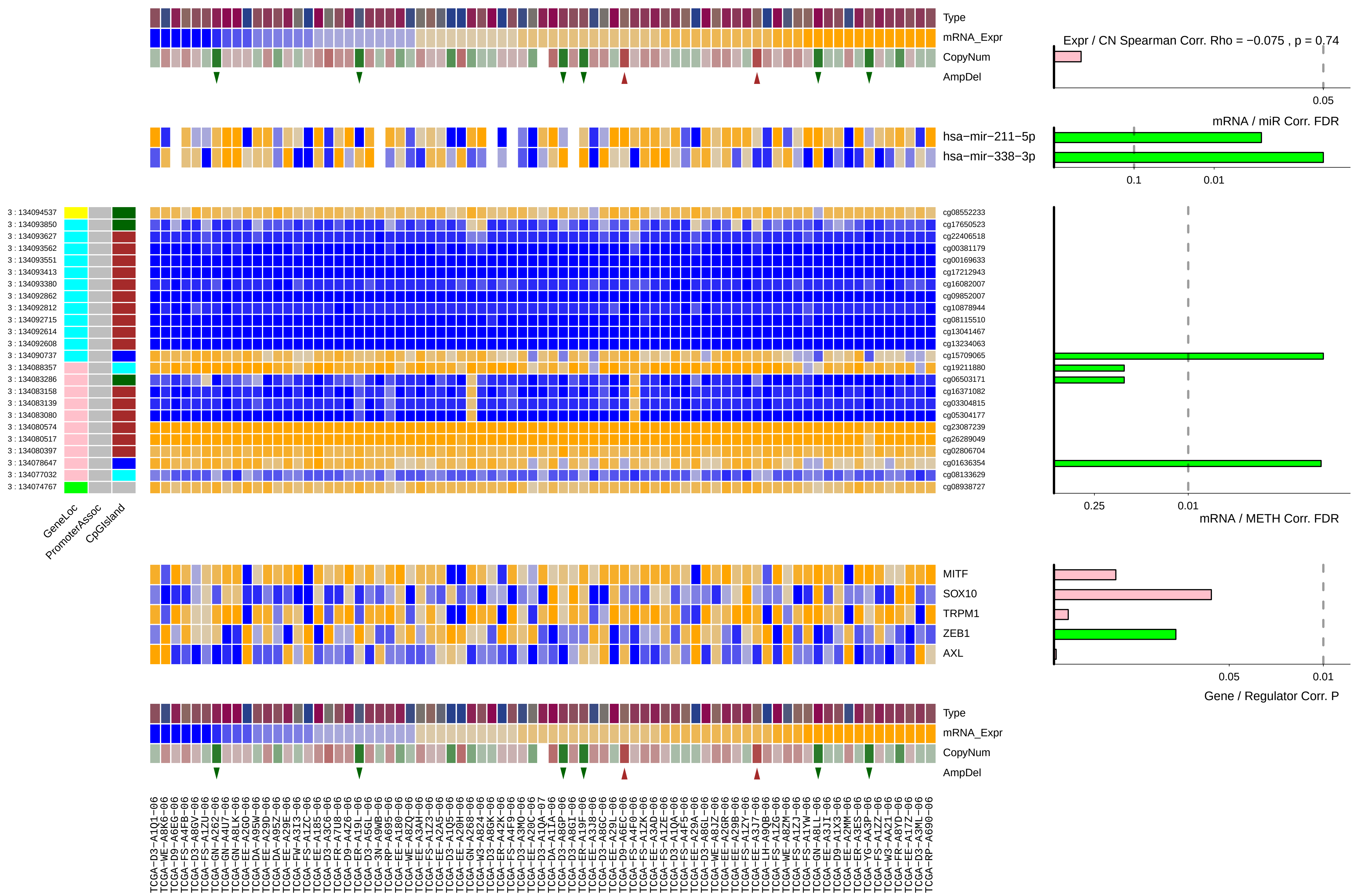

Type
mRNA_Expr
CopyNum
AmpDel
Expr / CN Spearman Corr. Rho = −0.075 , p = 0.74
0.05
mRNA / miR Corr. FDR
hsa−mir−211−5p
hsa−mir−338−3p
0.1
0.01
GeneLoc
PromoterAssoc
CpGIsland
mRNA / METH Corr. FDR
0.25
0.01
MITF
SOX10
TRPM1
ZEB1
AXL
0.05
0.01
Gene / Regulator Corr. P

LMO7

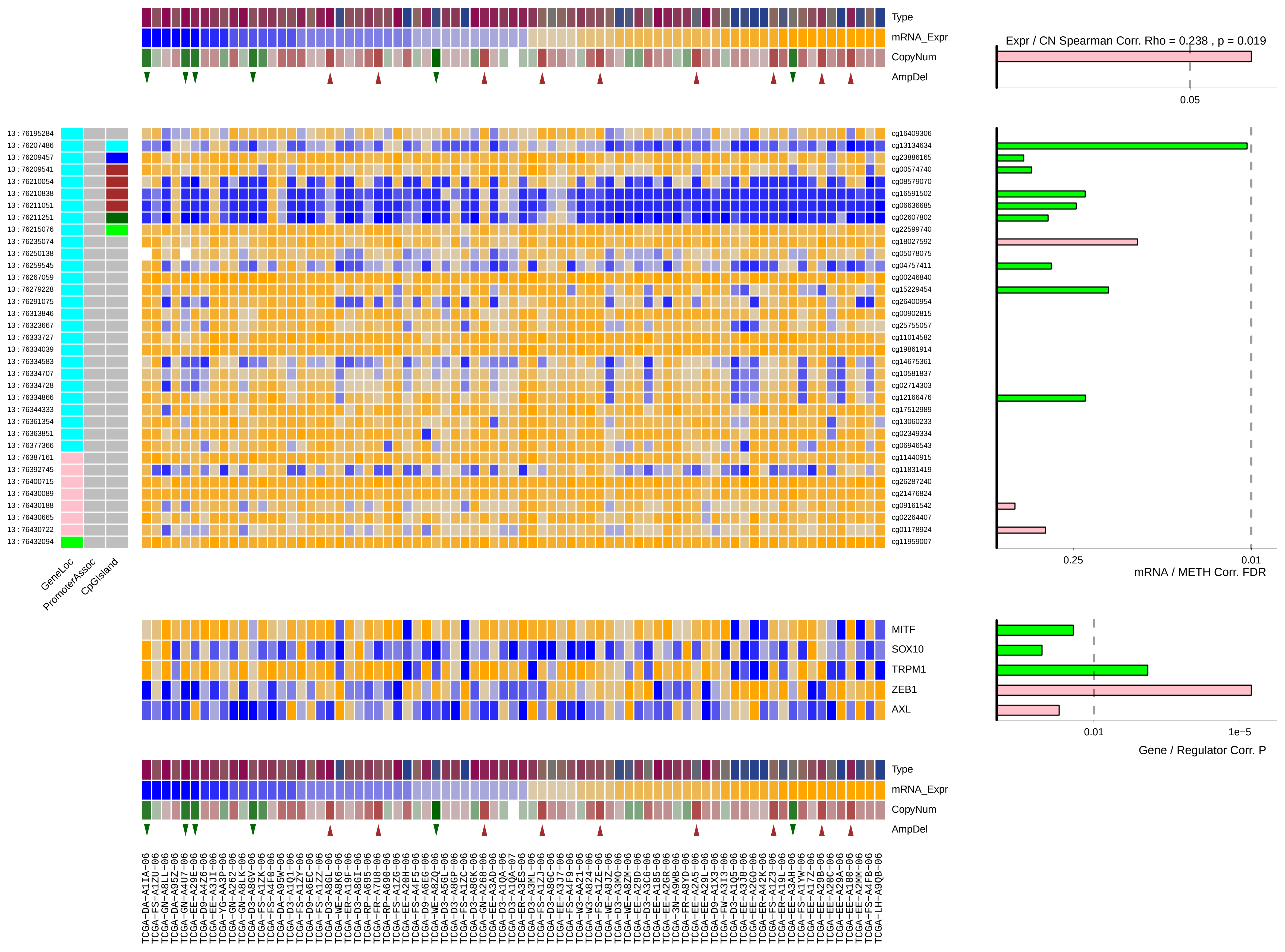

Type
mRNA_Expr
CopyNum
AmpDel
Expr / CN Spearman Corr. Rho = 0.238 , p = 0.019
0.05
GeneLoc
PromoterAssoc
CpGIsland
mRNA / METH Corr. FDR
0.25
0.01
MITF
SOX10
TRPM1
ZEB1
AXL
Gene / Regulator Corr. P
1e−5

RAC2

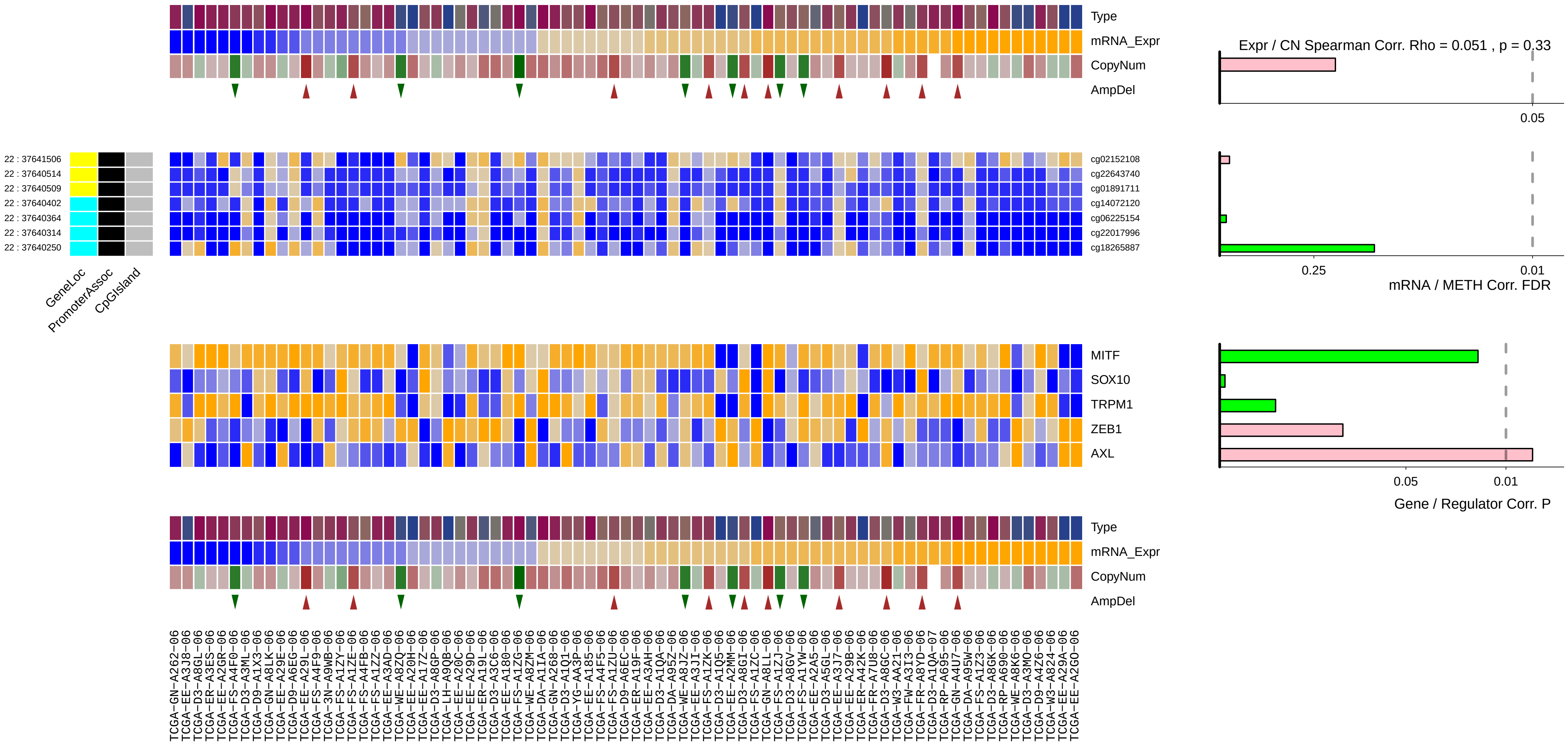
Type
mRNA_Expr
CopyNum
AmpDel
Expr / CN Spearman Corr. Rho = 0.051 , p = 0.33
0.05
22 : 37641506
22 : 37640514
22 : 37640509
22 : 37640402
22 : 37640364
22 : 37640314
22 : 37640250
GeneLoc
PromoterAssoc
CpGIsland
cg02152108
cg22643740
cg01891711
cg14072120
cg06225154
cg22017996
cg18265887
0.25
0.01
mRNA / METH Corr. FDR
MITF
SOX10
TRPM1
ZEB1
AXL
0.05
0.01
Gene / Regulator Corr. P

ARHGEF28

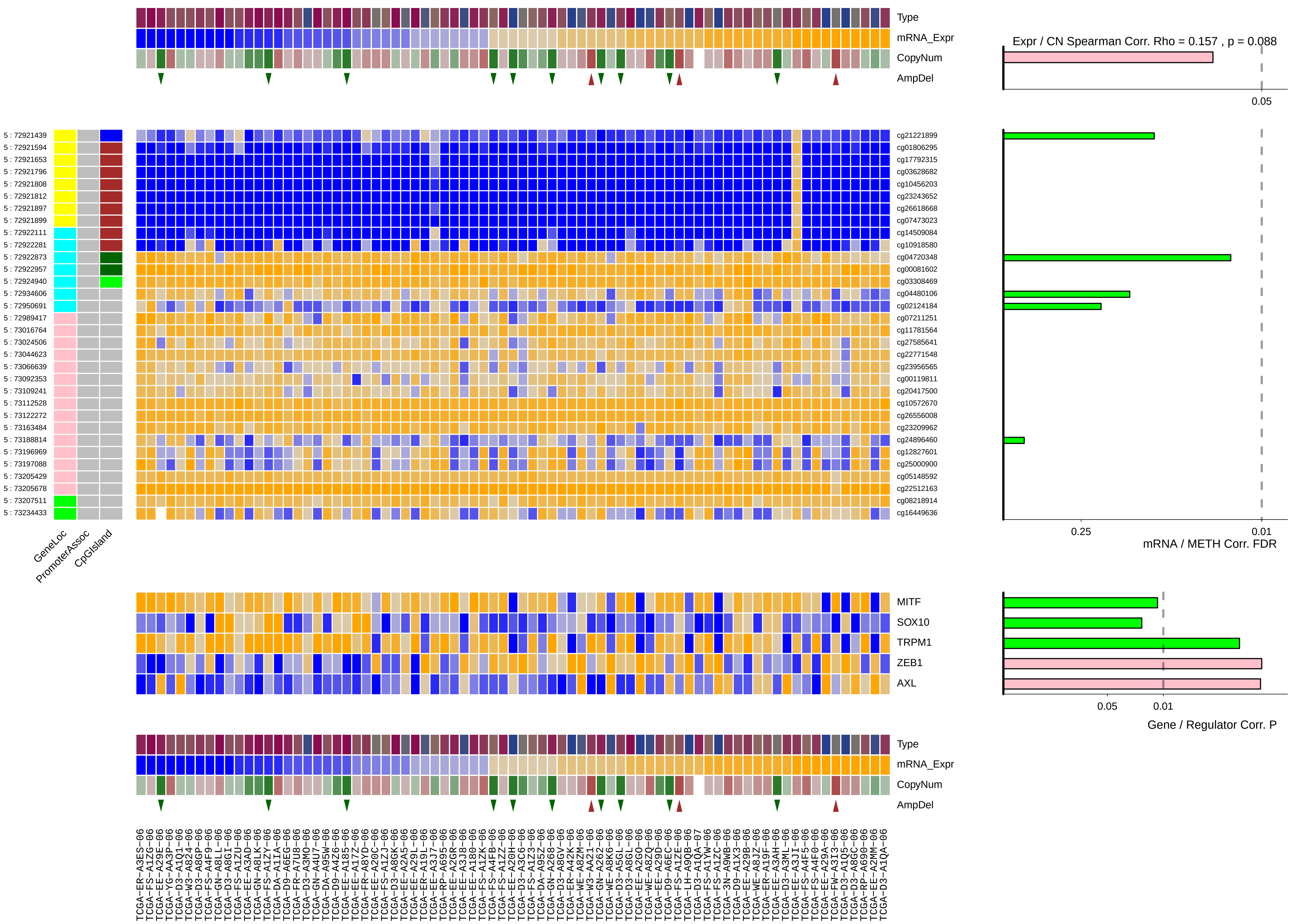

Type
mRNA_Expr
CopyNum
AmpDel
Expr / CN Spearman Corr. Rho = 0.157 , p = 0.088
0.05
5 : 72921439
5 : 72921594
5 : 72921653
5 : 72921796
5 : 72921808
5 : 72921812
5 : 72921897
5 : 72921899
5 : 72922111
5 : 72922281
5 : 72922873
5 : 72922957
5 : 72924940
5 : 72934606
5 : 72950691
5 : 72989417
5 : 73016764
5 : 73024506
5 : 73044623
5 : 73066639
5 : 73092353
5 : 73109241
5 : 73112528
5 : 73122272
5 : 73163484
5 : 73188814
5 : 73196969
5 : 73197088
5 : 73205429
5 : 73205678
5 : 73207511
5 : 73234433
cg21221899
cg01806295
cg17792315
cg03628682
cg10456203
cg23243652
cg26618668
cg07473023
cg14509084
cg10918580
cg04720348
cg00081602
cg03308469
cg04480106
cg02124184
cg07211251
cg11781564
cg27585641
cg22771548
cg23956565
cg00119811
cg20417500
cg10572670
cg26556008
cg23209962
cg24896460
cg12827601
cg25000900
cg05148592
cg22512163
cg08218914
cg16449636
GeneLoc
PromoterAssoc
CpGIsland
0.25
0.01
mRNA / METH Corr. FDR
MITF
SOX10
TRPM1
ZEB1
AXL
0.05
0.01
Gene / Regulator Corr. P
Type
mRNA_Expr
CopyNum
AmpDel

COL1A1

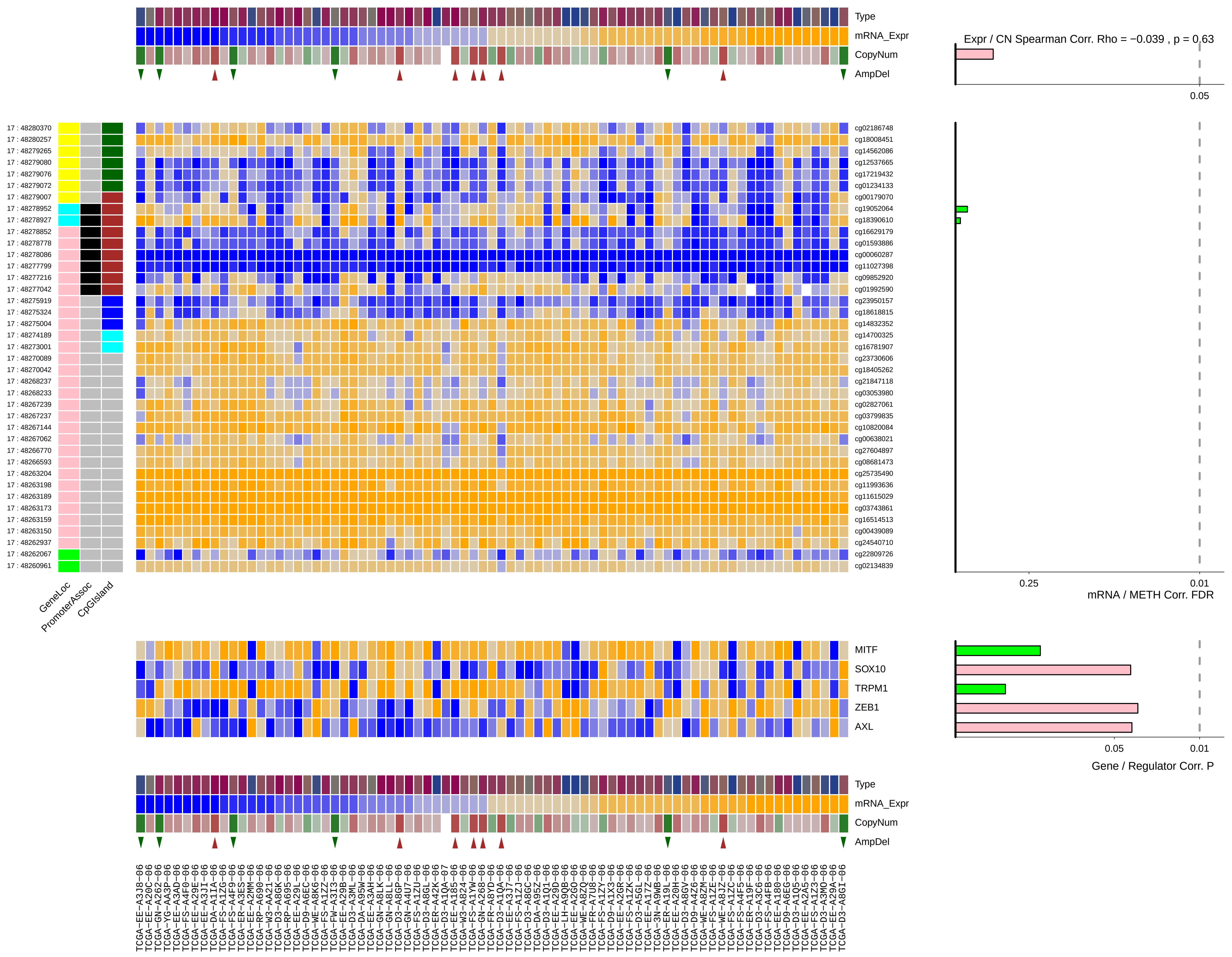

Type
mRNA_Expr
CopyNum
AmpDel
Expr / CN Spearman Corr. Rho = −0.039 , p = 0.63
0.05
0.25
0.01
mRNA / METH Corr. FDR
GeneLoc
PromoterAssoc
CpGIsland
MITF
SOX10
TRPM1
ZEB1
AXL
Gene / Regulator Corr. P

LOX

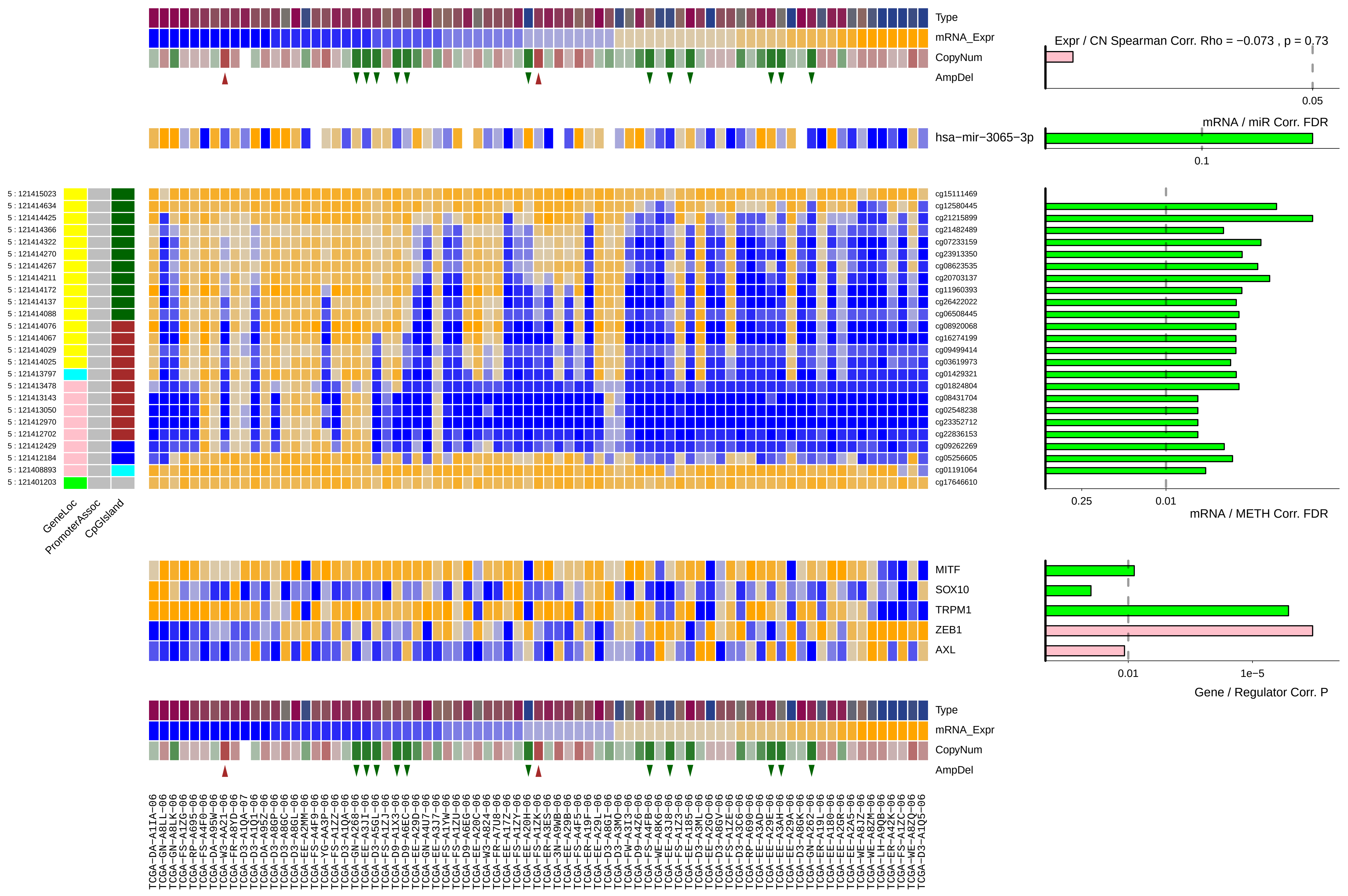

Type
mRNA_Expr
CopyNum
AmpDel
Expr / CN Spearman Corr. Rho = −0.073 , p = 0.73
hsa−mir−3065−3p
mRNA / miR Corr. FDR
mRNA / METH Corr. FDR
Gene / Regulator Corr. P
GeneLoc
PromoterAssoc
CpGIsland
MITF
SOX10
TRPM1
ZEB1
AXL

FGF5

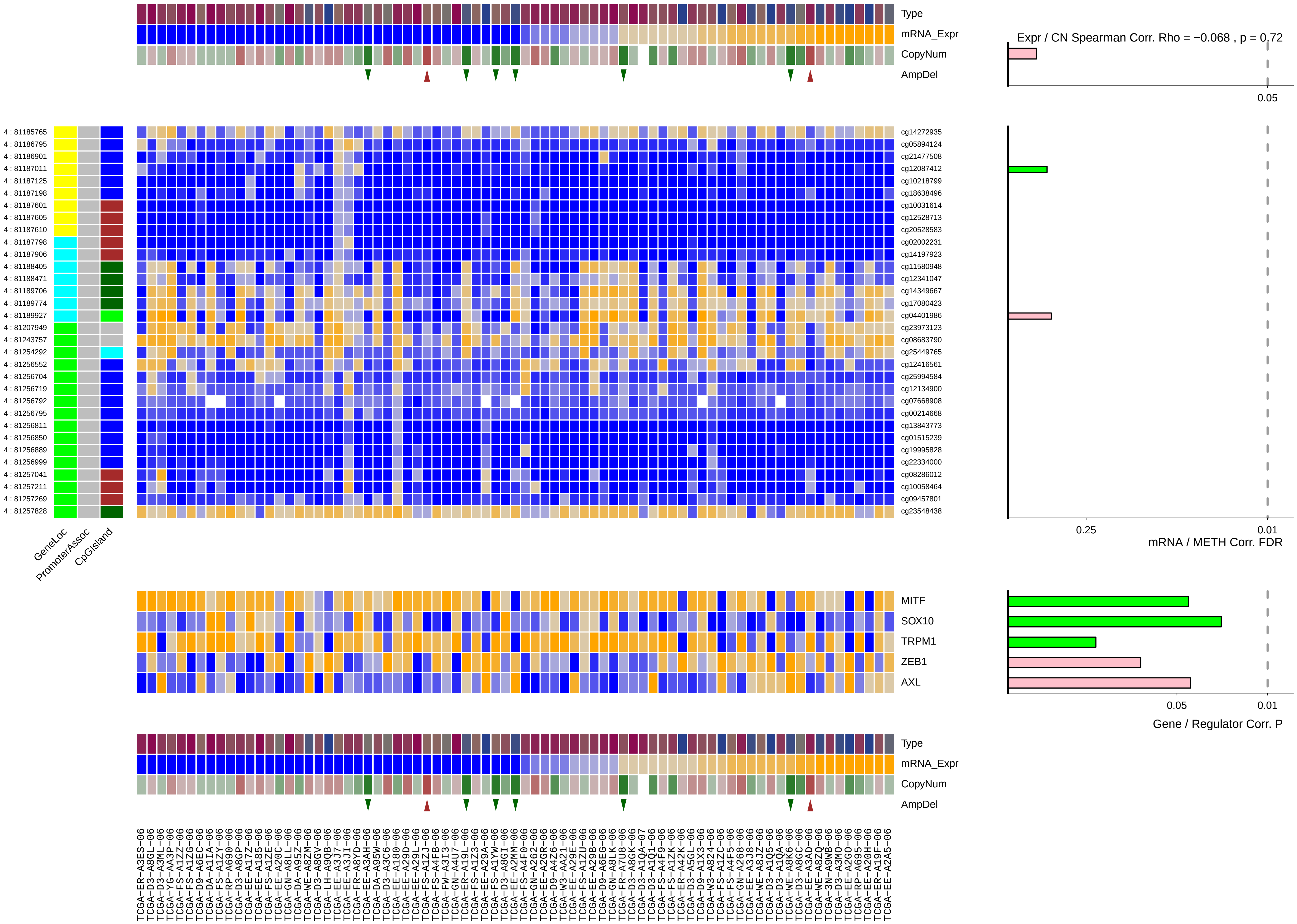
Type
mRNA_Expr
CopyNum
AmpDel
Expr / CN Spearman Corr. Rho = −0.068 , p = 0.72
0.05
GeneLoc
PromoterAssoc
CpGIsland
mRNA / METH Corr. FDR
0.25
0.01
MITF
SOX10
TRPM1
ZEB1
AXL
Gene / Regulator Corr. P

LRIG1

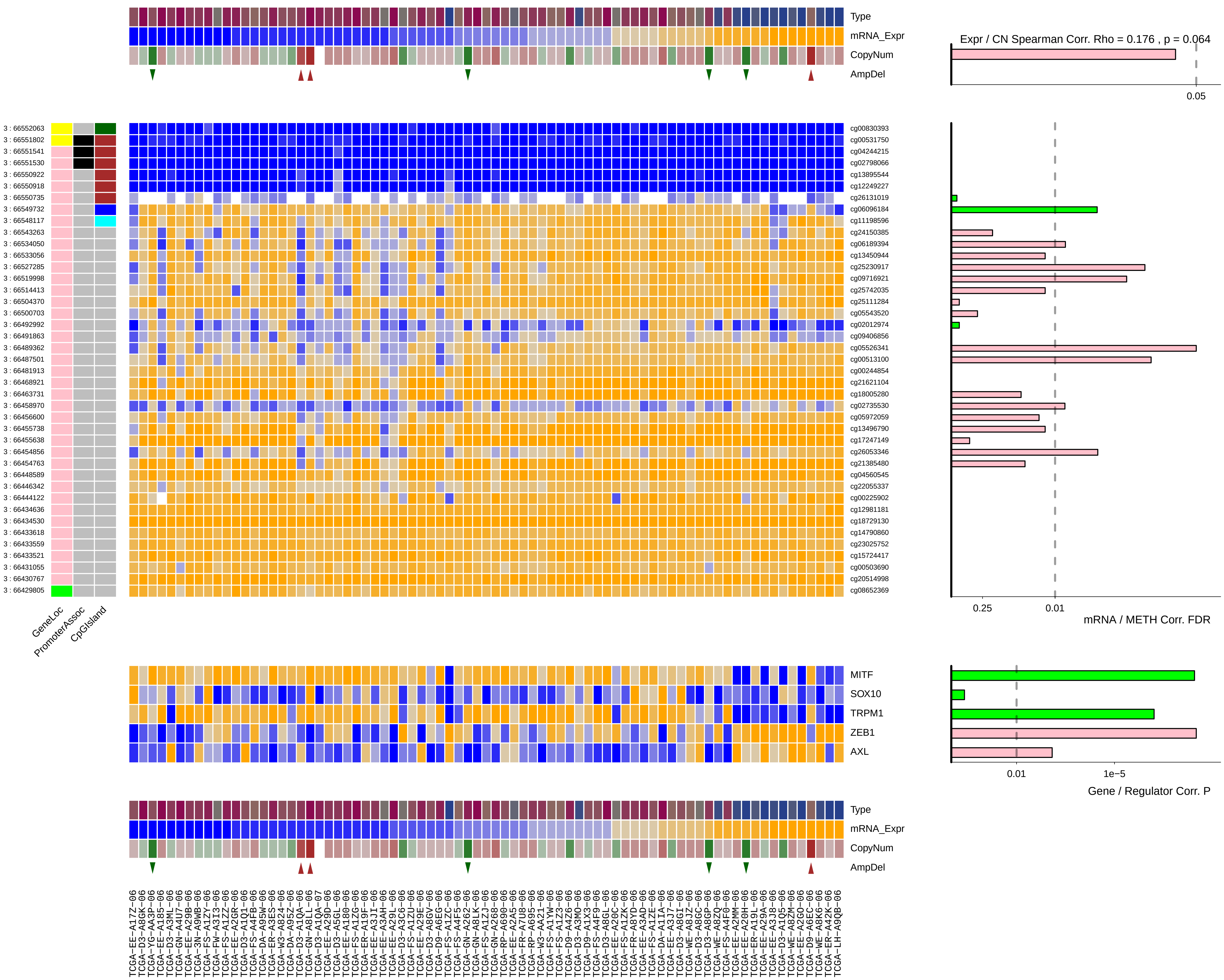

Type
mRNA_Expr
CopyNum
AmpDel
Expr / CN Spearman Corr. Rho = 0.176 , p = 0.064
0.05
3 : 66552063
3 : 66551802
3 : 66551541
3 : 66551530
3 : 66550922
3 : 66550918
3 : 66550735
3 : 66549732
3 : 66548117
3 : 66543263
3 : 66534050
3 : 66533056
3 : 66527285
3 : 66519998
3 : 66514413
3 : 66504370
3 : 66500703
3 : 66492992
3 : 66491863
3 : 66489362
3 : 66487501
3 : 66481913
3 : 66468921
3 : 66463731
3 : 66458970
3 : 66456600
3 : 66455738
3 : 66455638
3 : 66454856
3 : 66454763
3 : 66448589
3 : 66446342
3 : 66444122
3 : 66434636
3 : 66434530
3 : 66433618
3 : 66433559
3 : 66433521
3 : 66431055
3 : 66430767
3 : 66429805
cg00830393
cg00531750
cg04244215
cg02798066
cg13895544
cg12249227
cg26131019
cg06096184
cg11198596
cg24150385
cg06189394
cg13450944
cg25230917
cg09716921
cg25742035
cg25111284
cg05543520
cg02012974
cg09406856
cg05526341
cg00513100
cg00244854
cg21621104
cg18005280
cg02735530
cg05972059
cg13496790
cg17247149
cg26053346
cg21385480
cg04560545
cg22055337
cg00225902
cg12981181
cg18729130
cg14790860
cg23025752
cg15724417
cg00503690
cg20514998
cg08652369
0.25
0.01
mRNA / METH Corr. FDR
GeneLoc
PromoterAssoc
CpGIsland
MITF
SOX10
TRPM1
ZEB1
AXL
0.01
1e−5
Gene / Regulator Corr. P
Type
mRNA_Expr
CopyNum
AmpDel

SLC38A1

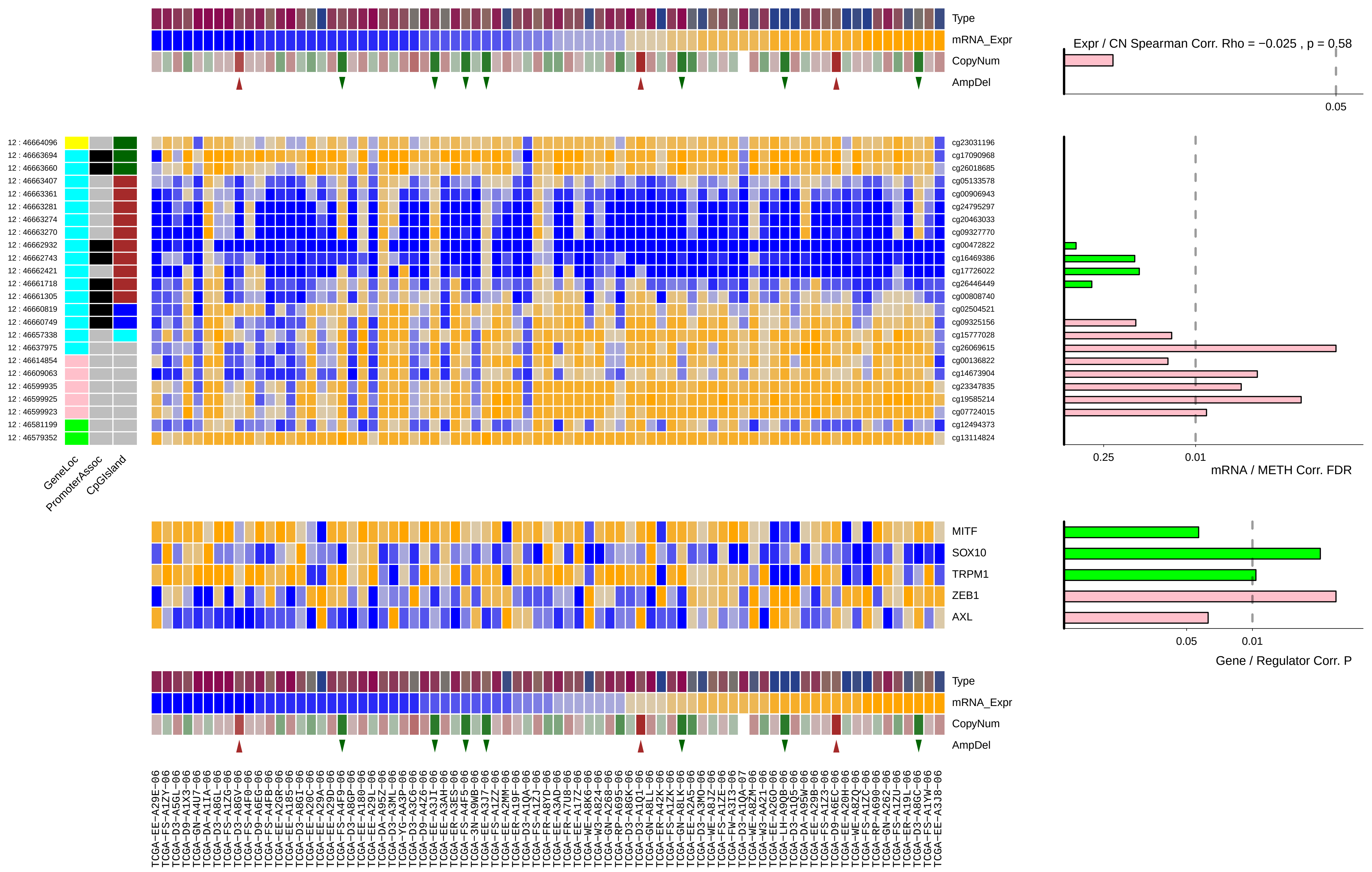

Type
mRNA_Expr
CopyNum
AmpDel
Expr / CN Spearman Corr. Rho = −0.025 , p = 0.58
0.05
GeneLoc
PromoterAssoc
CpGIsland
0.25
0.01
mRNA / METH Corr. FDR
MITF
SOX10
TRPM1
ZEB1
AXL
0.05
0.01
Gene / Regulator Corr. P

CITED2

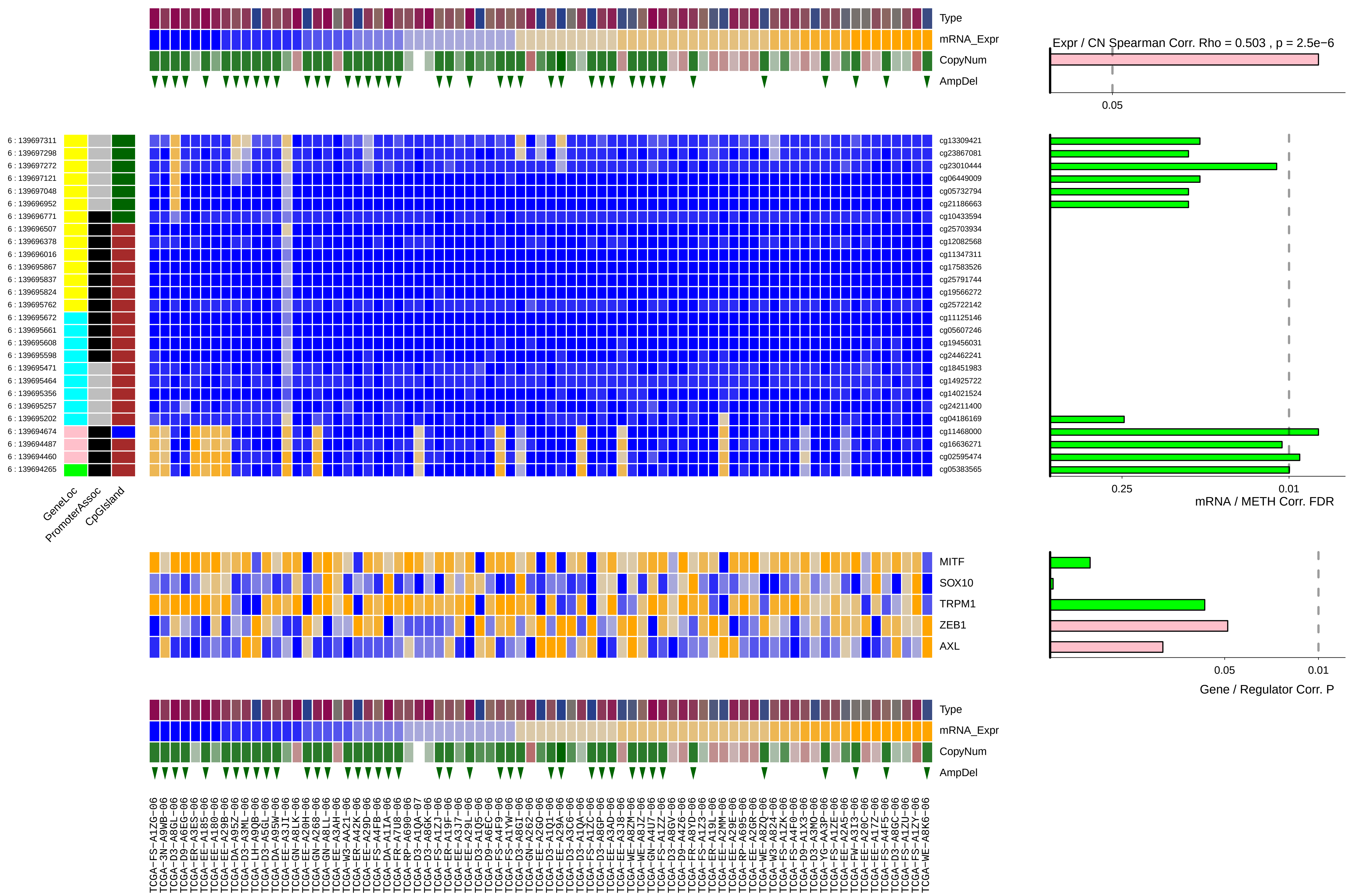
Type
mRNA_Expr
CopyNum
AmpDel
Expr / CN Spearman Corr. Rho = 0.503 , p = 2.5e−6
0.05
GeneLoc
PromoterAssoc
CpGIsland
0.25
0.01
mRNA / METH Corr. FDR
MITF
SOX10
TRPM1
ZEB1
AXL
0.05
0.01
Gene / Regulator Corr. P

PDE1C

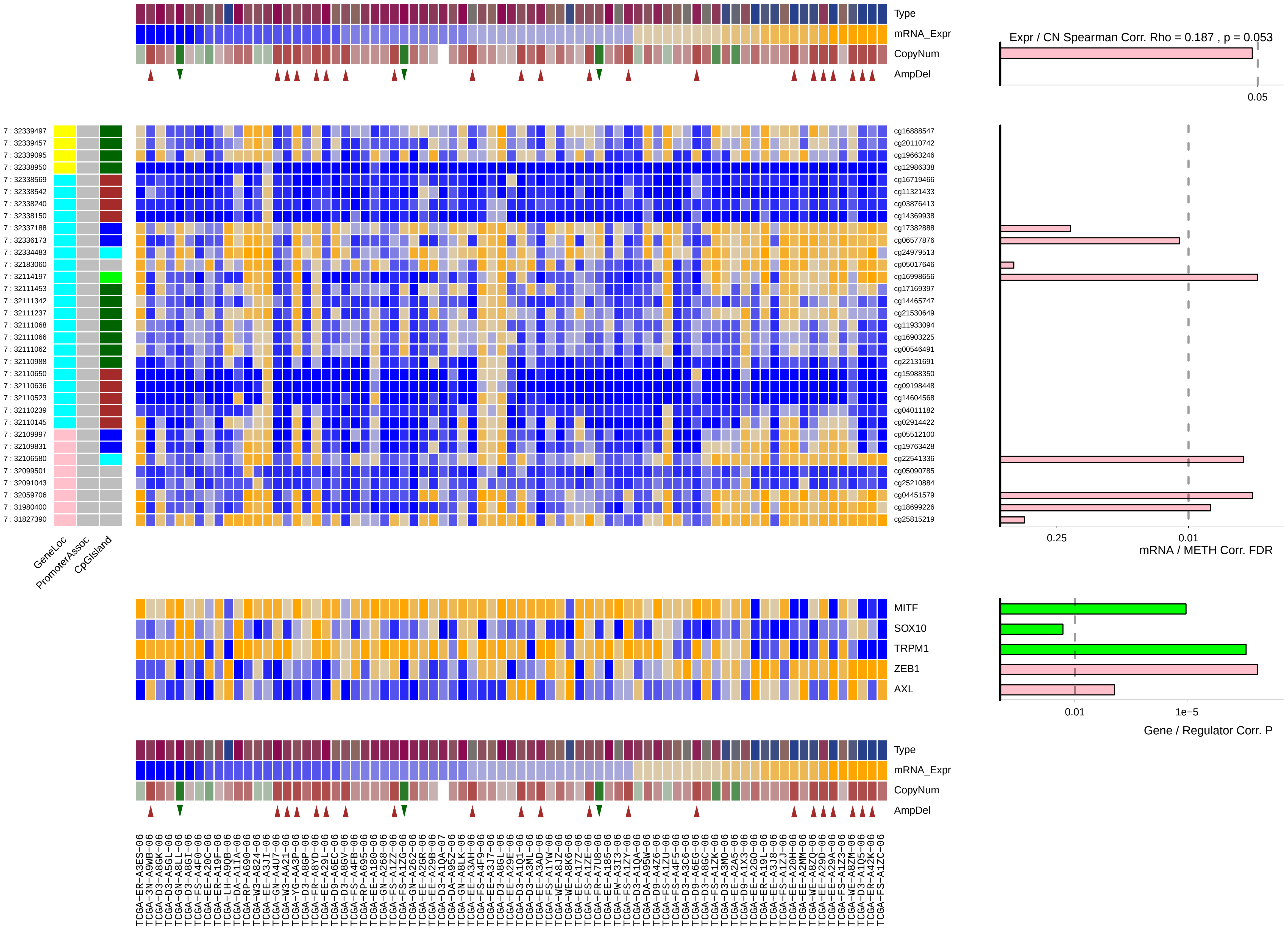
Type
mRNA_Expr
CopyNum
AmpDel
Expr / CN Spearman Corr. Rho = 0.187 , p = 0.053
0.05
7 : 32339497
7 : 32339457
7 : 32339095
7 : 32338950
7 : 32338569
7 : 32338542
7 : 32338240
7 : 32338150
7 : 32337188
7 : 32336173
7 : 32334483
7 : 32183060
7 : 32114197
7 : 32111453
7 : 32111342
7 : 32111237
7 : 32111068
7 : 32111066
7 : 32111062
7 : 32110988
7 : 32110650
7 : 32110636
7 : 32110523
7 : 32110239
7 : 32110145
7 : 32109997
7 : 32109831
7 : 32106580
7 : 32099501
7 : 32091043
7 : 32059706
7 : 31980400
7 : 31827390
cg16888547
cg20110742
cg19663246
cg12986338
cg16719466
cg11321433
cg03876413
cg14369938
cg17382888
cg06577876
cg24979513
cg05017646
cg16998656
cg17169397
cg14465747
cg21530649
cg11933094
cg16903225
cg00546491
cg22131691
cg15988350
cg09198448
cg14604568
cg04011182
cg02914422
cg05512100
cg19763428
cg22541336
cg05090785
cg25210884
cg04451579
cg18699226
cg25815219
0.25
0.01
mRNA / METH Corr. FDR
GeneLoc
PromoterAssoc
CpGIsland
MITF
SOX10
TRPM1
ZEB1
AXL
0.01
1e−5
Gene / Regulator Corr. P
Type
mRNA_Expr
CopyNum
AmpDel

CDH2

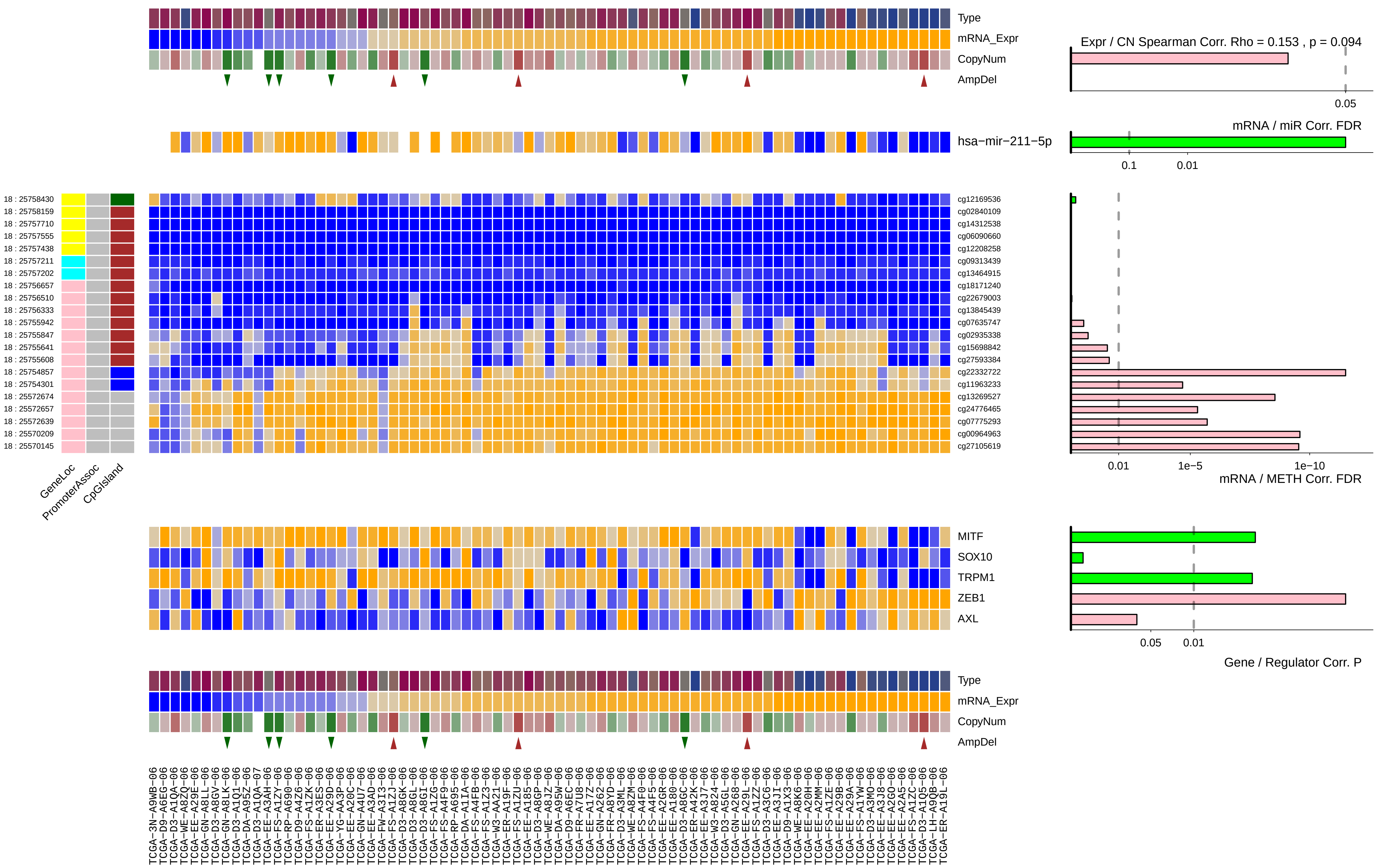

Type
mRNA_Expr
CopyNum
AmpDel
Expr / CN Spearman Corr. Rho = 0.153 , p = 0.094
0.05
hsa−mir−211−5p
mRNA / miR Corr. FDR
0.1
0.01
GeneLoc
PromoterAssoc
CpGIsland
0.01
1e−5
1e−10
mRNA / METH Corr. FDR
MITF
SOX10
TRPM1
ZEB1
AXL
0.05
0.01
Gene / Regulator Corr. P

RGS4

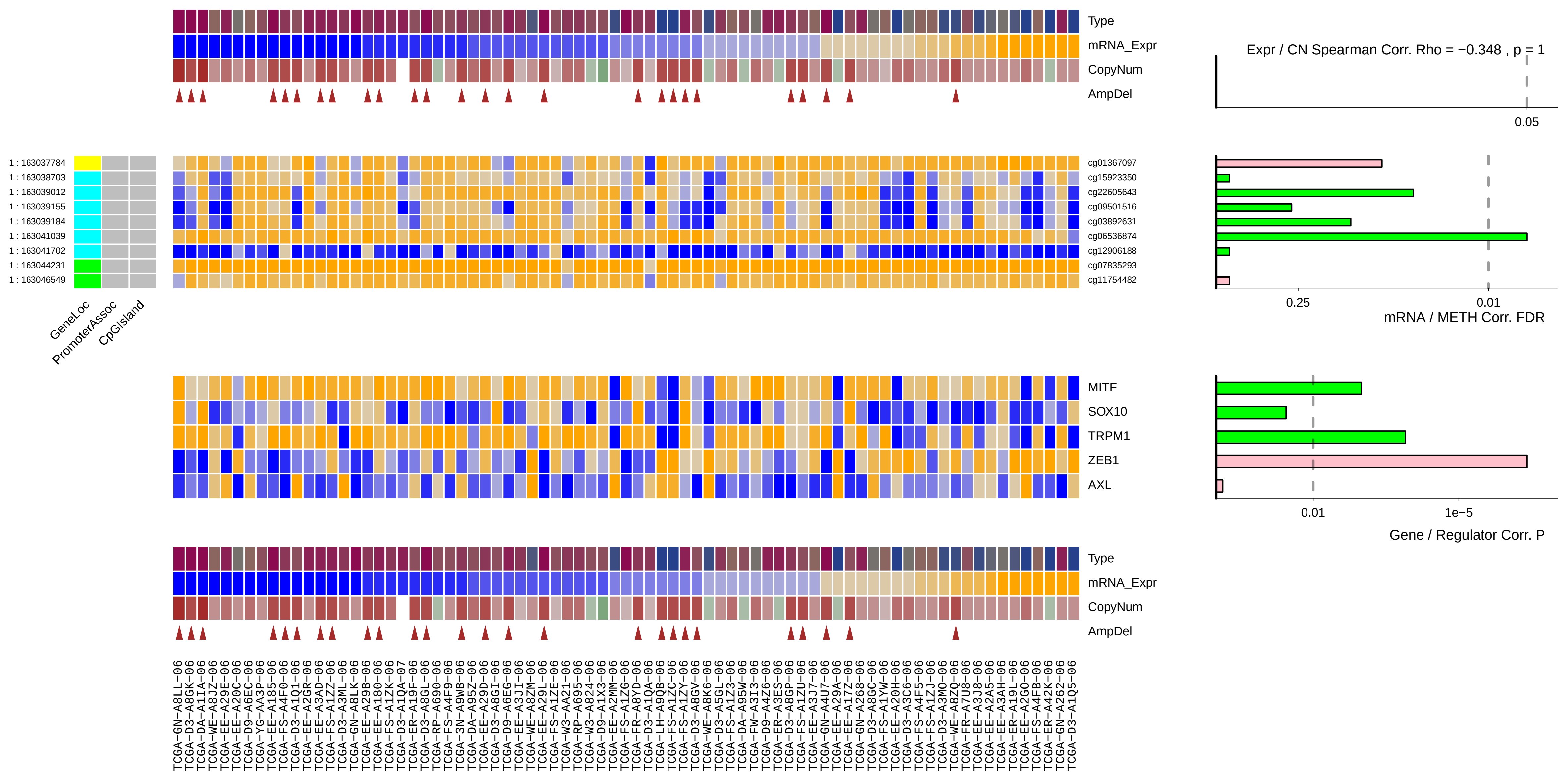

Type
mRNA_Expr
CopyNum
AmpDel
Expr / CN Spearman Corr. Rho = −0.348 , p = 1
0.05
1 : 163037784
1 : 163038703
1 : 163039012
1 : 163039155
1 : 163039184
1 : 163041039
1 : 163041702
1 : 163044231
1 : 163046549
cg01367097
cg15923350
cg22605643
cg09501516
cg03892631
cg06536874
cg12906188
cg07835293
cg11754482
GeneLoc
PromoterAssoc
CpGIsland
0.25
0.01
mRNA / METH Corr. FDR
MITF
SOX10
TRPM1
ZEB1
AXL
0.01
1e−5
Gene / Regulator Corr. P

EHD2

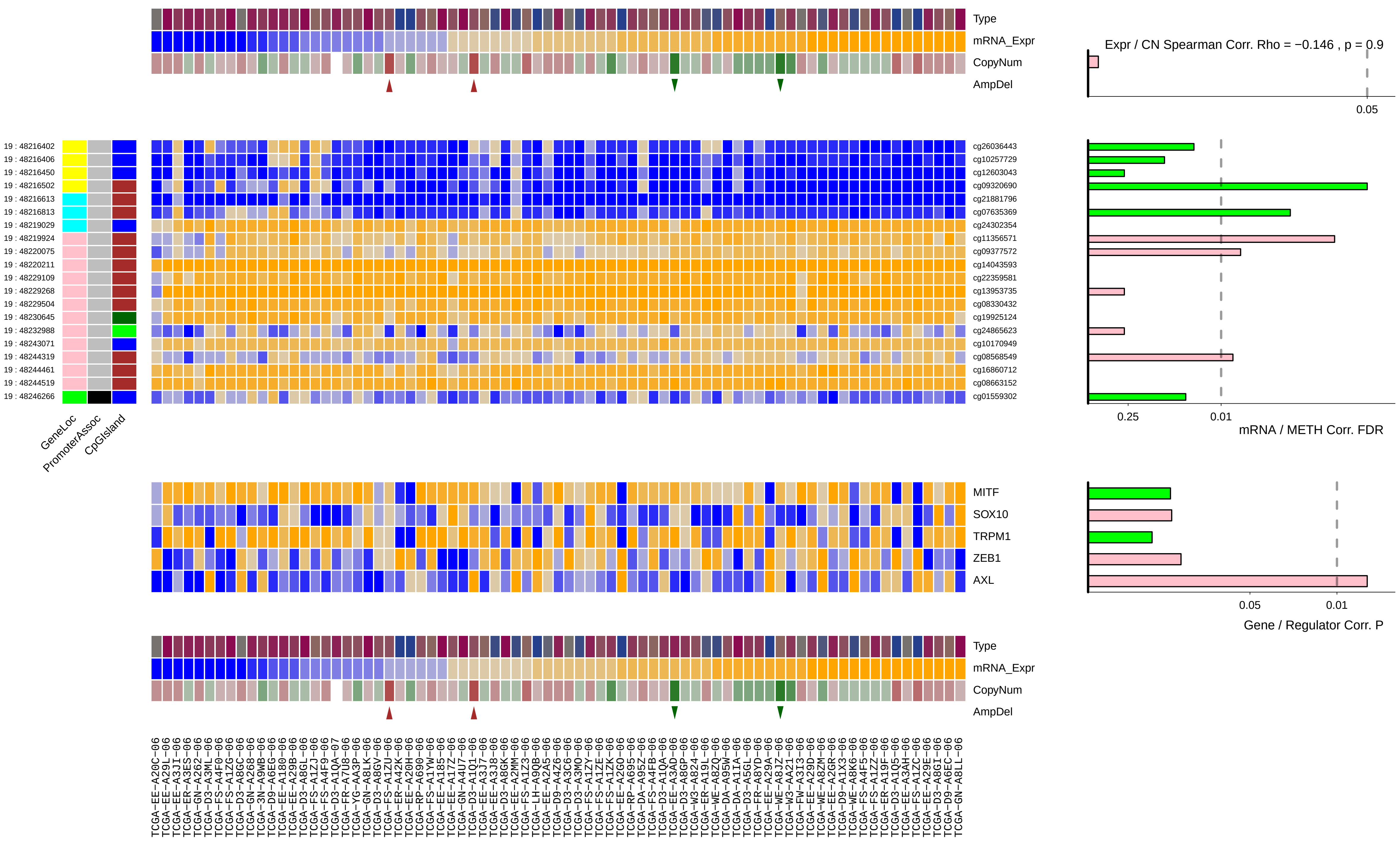
Type
mRNA_Expr
CopyNum
AmpDel
Expr / CN Spearman Corr. Rho = −0.146 , p = 0.9
0.05
cg26036443
cg10257729
cg12603043
cg09320690
cg21881796
cg07635369
cg24302354
cg11356571
cg09377572
cg14043593
cg22359581
cg13953735
cg08330432
cg19925124
cg24865623
cg10170949
cg08568549
cg16860712
cg08663152
cg01559302
GeneLoc
PromoterAssoc
CpGIsland
0.25
0.01
mRNA / METH Corr. FDR
MITF
SOX10
TRPM1
ZEB1
AXL
0.05
0.01
Gene / Regulator Corr. P

COL12A1

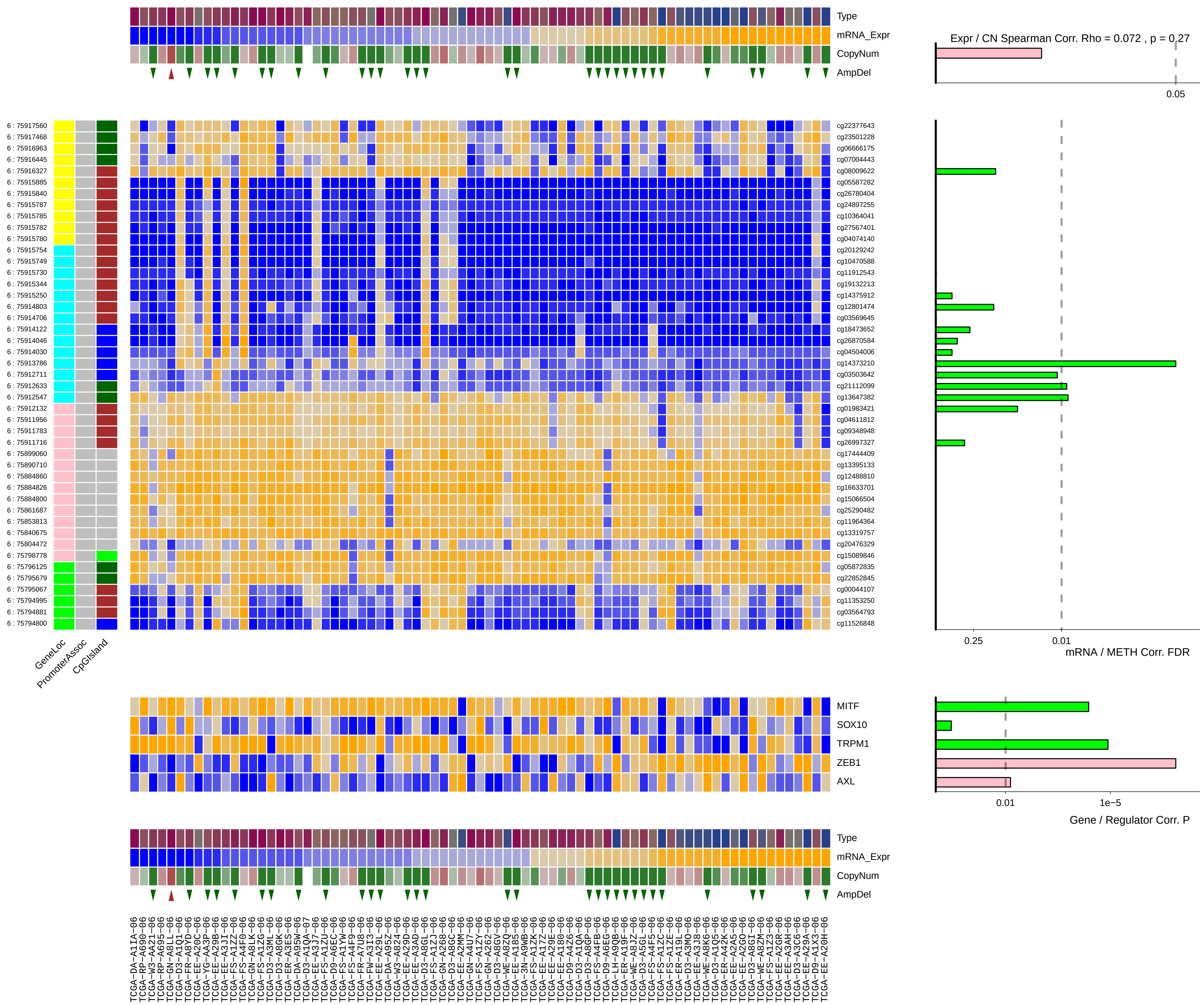
Type
mRNA_Expr
CopyNum
AmpDel
Expr / CN Spearman Corr. Rho = 0.072 , p = 0.27
0.05
GeneLoc
PromoterAssoc
CpGIsland
mRNA / METH Corr. FDR
0.25
0.01
MITF
SOX10
TRPM1
ZEB1
AXL
Gene / Regulator Corr. P
0.01
1e−5

MICAL2

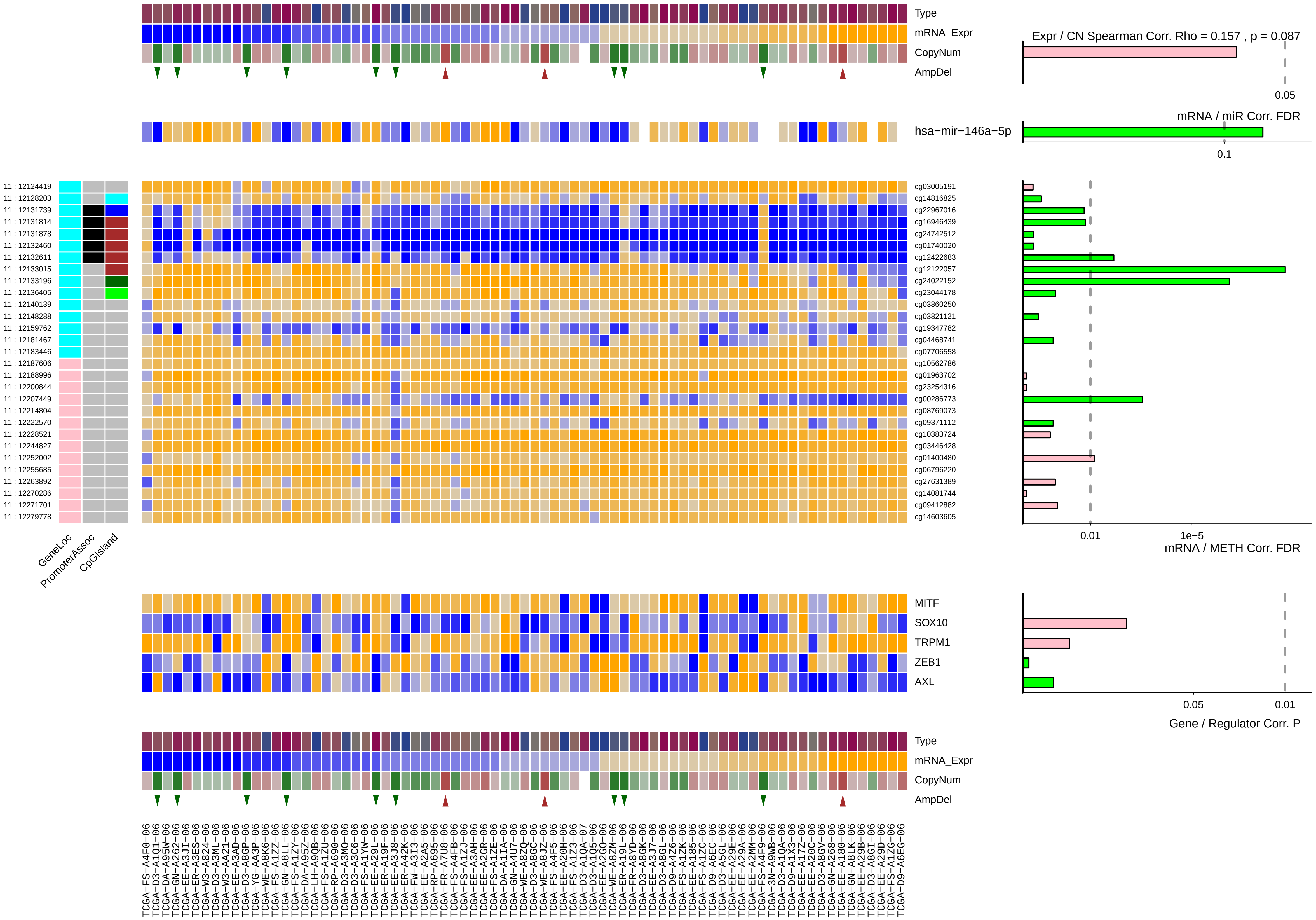

Type
mRNA_Expr
CopyNum
AmpDel
Expr / CN Spearman Corr. Rho = 0.157 , p = 0.087
0.05
hsa−mir−146a−5p
mRNA / miR Corr. FDR
0.1
0.01
1e−5
mRNA / METH Corr. FDR
GeneLoc
PromoterAssoc
CpGIsland
MITF
SOX10
TRPM1
ZEB1
AXL
0.05
0.01
Gene / Regulator Corr. P

ERRFI1

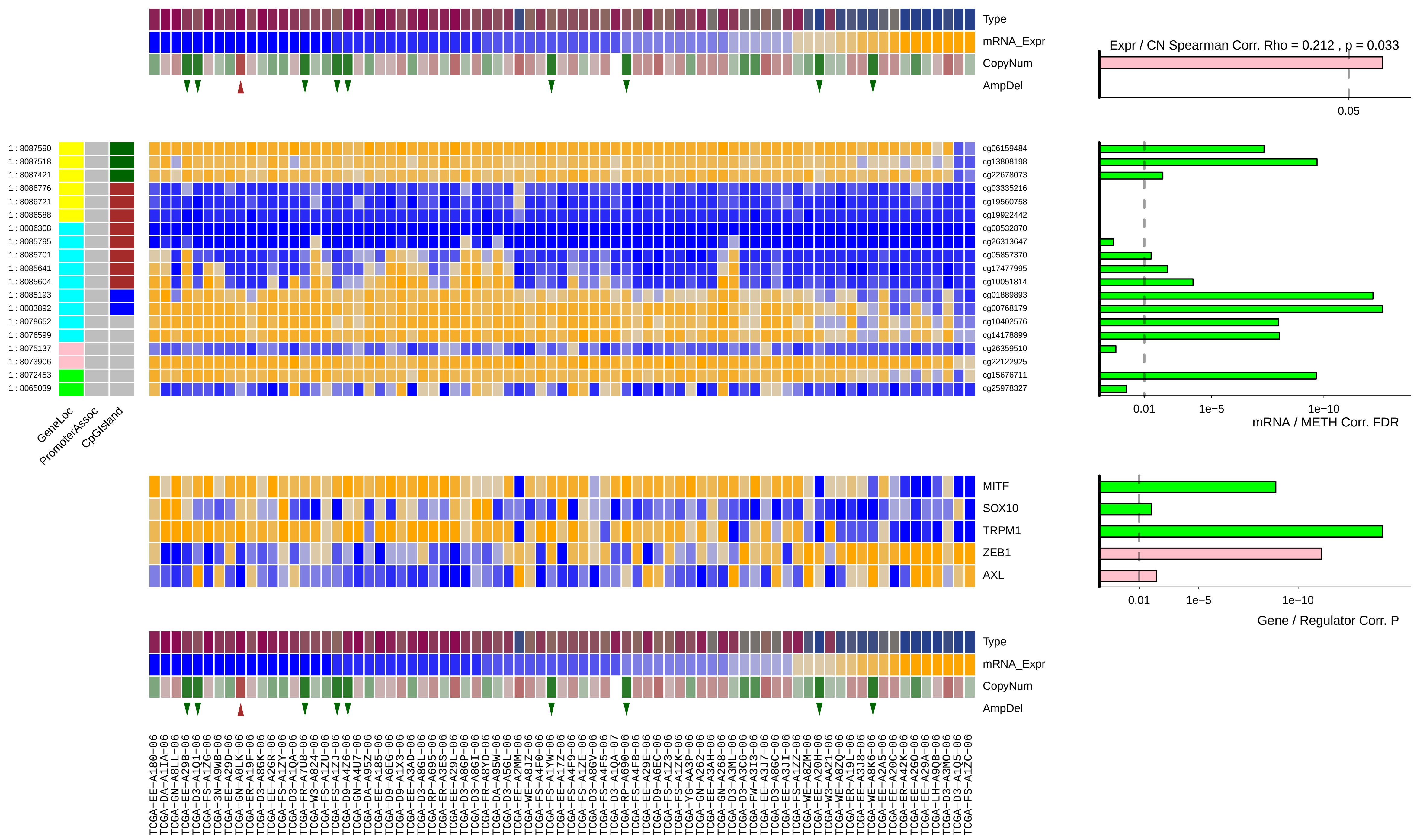

Type
mRNA_Expr
CopyNum
AmpDel
Expr / CN Spearman Corr. Rho = 0.212 , p = 0.033
0.05
1 : 8087590
1 : 8087518
1 : 8087421
1 : 8086776
1 : 8086721
1 : 8086588
1 : 8086308
1 : 8085795
1 : 8085701
1 : 8085641
1 : 8085604
1 : 8085193
1 : 8083892
1 : 8078652
1 : 8076599
1 : 8075137
1 : 8073906
1 : 8072453
1 : 8065039
cg06159484
cg13808198
cg22678073
cg03335216
cg19560758
cg19922442
cg08532870
cg26313647
cg05857370
cg17477995
cg10051814
cg01889893
cg00768179
cg10402576
cg14178899
cg26359510
cg22122925
cg15676711
cg25978327
GeneLoc
PromoterAssoc
CpGIsland
0.01
1e−5
1e−10
mRNA / METH Corr. FDR
MITF
SOX10
TRPM1
ZEB1
AXL
0.01
1e−5
1e−10
Gene / Regulator Corr. P
Type
mRNA_Expr
CopyNum
AmpDel

SLC14A1

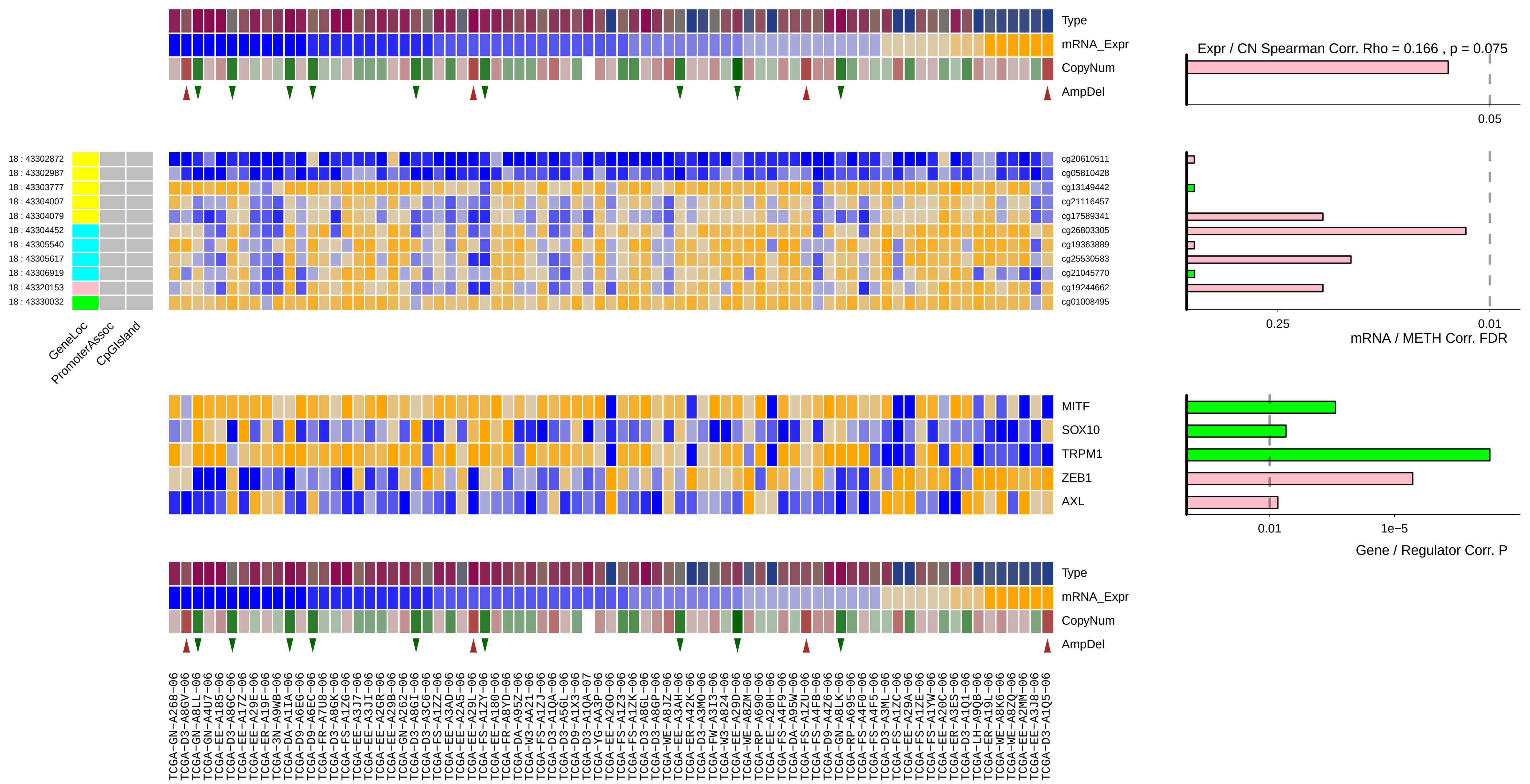

Type
mRNA_Expr
CopyNum
AmpDel
Expr / CN Spearman Corr. Rho = 0.166 , p = 0.075
0.05
cg20610511
cg05810428
cg13149442
cg21116457
cg17589341
cg26803305
cg19363889
cg25530583
cg21045770
cg19244662
cg01008495
GeneLoc
PromoterAssoc
CpGIsland
0.25
0.01
mRNA / METH Corr. FDR
MITF
SOX10
TRPM1
ZEB1
AXL
0.01
1e−5
Gene / Regulator Corr. P

ADAM19

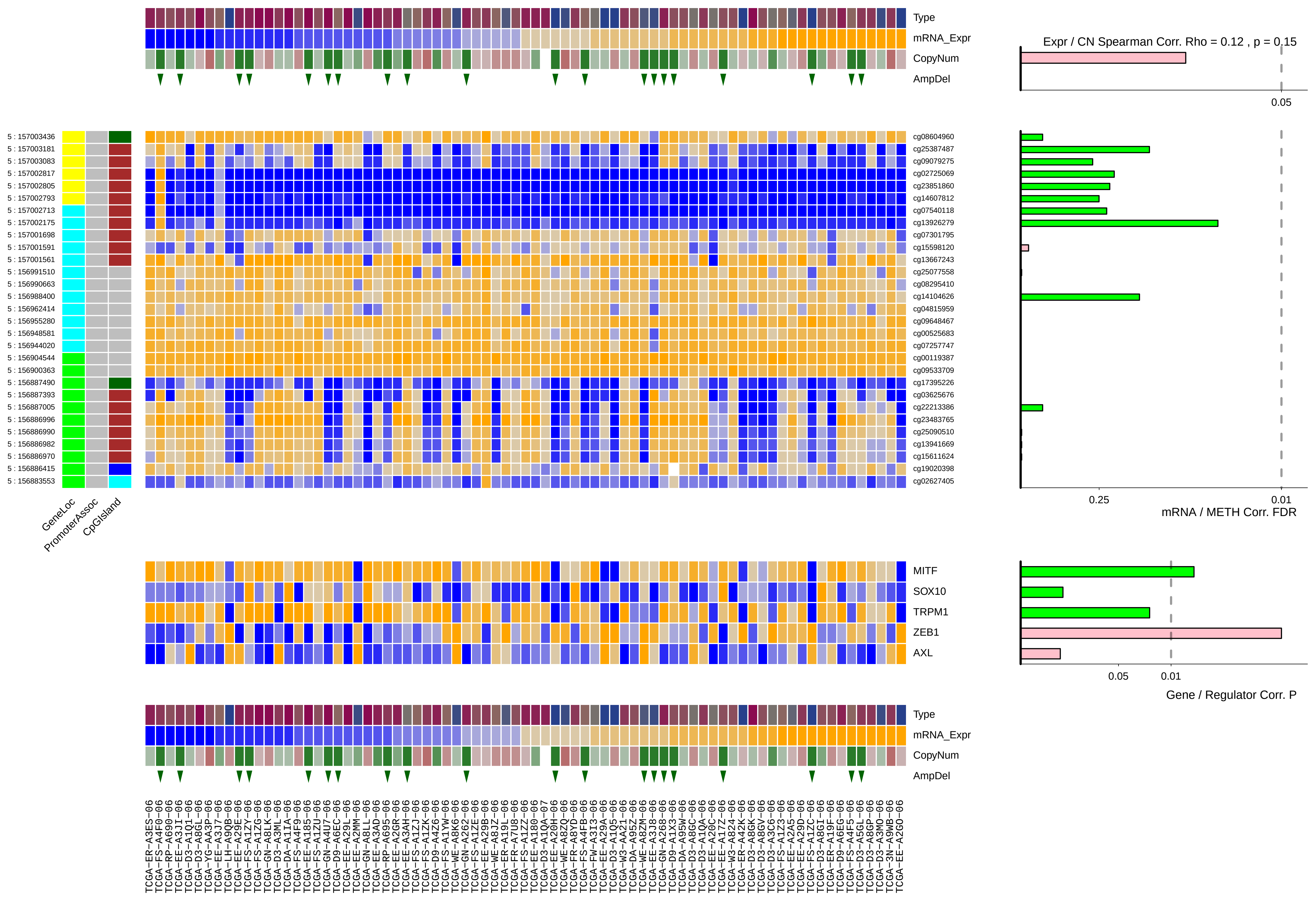

Type
mRNA_Expr
CopyNum
AmpDel
Expr / CN Spearman Corr. Rho = 0.12 , p = 0.15
0.05
5 : 157003436
5 : 157003181
5 : 157003083
5 : 157002817
5 : 157002805
5 : 157002793
5 : 157002713
5 : 157002175
5 : 157001698
5 : 157001591
5 : 157001561
5 : 156991510
5 : 156990663
5 : 156988400
5 : 156962414
5 : 156955280
5 : 156948581
5 : 156944020
5 : 156904544
5 : 156900363
5 : 156887490
5 : 156887393
5 : 156887005
5 : 156886996
5 : 156886990
5 : 156886982
5 : 156886970
5 : 156886415
5 : 156883553
GeneLoc
PromoterAssoc
CpGIsland
cg08604960
cg25387487
cg09079275
cg02725069
cg23851860
cg14607812
cg07540118
cg13926279
cg07301795
cg15598120
cg13667243
cg25077558
cg08295410
cg14104626
cg04815959
cg09648467
cg00525683
cg07257747
cg00119387
cg09533709
cg17395226
cg03625676
cg22213386
cg23483765
cg25090510
cg13941669
cg15611624
cg19020398
cg02627405
0.25
0.01
mRNA / METH Corr. FDR
MITF
SOX10
TRPM1
ZEB1
AXL
0.05
0.01
Gene / Regulator Corr. P
Type
mRNA_Expr
CopyNum
AmpDel

KRT8

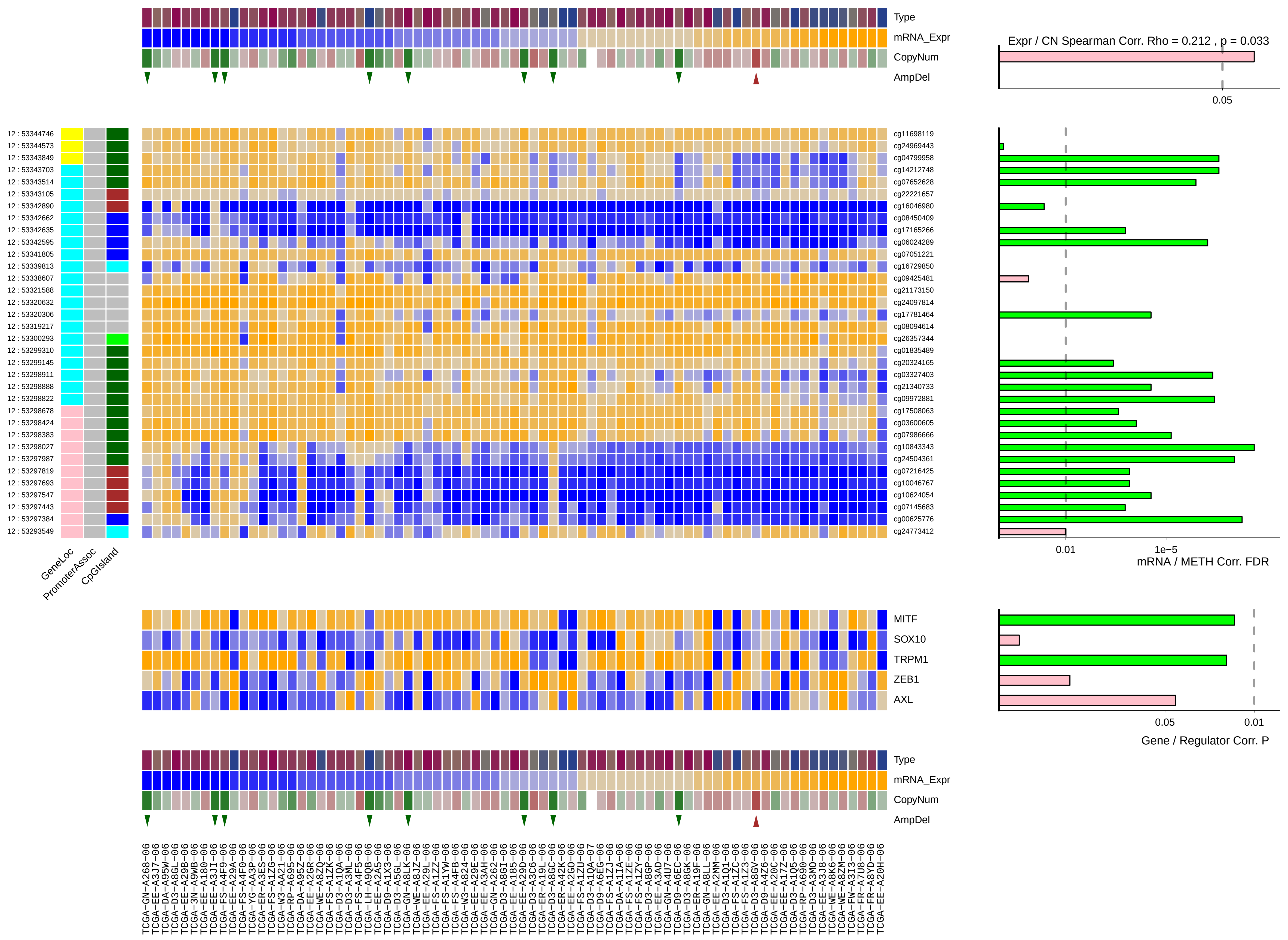
Type
mRNA_Expr
CopyNum
AmpDel
Expr / CN Spearman Corr. Rho = 0.212 , p = 0.033
0.05
GeneLoc
PromoterAssoc
CpGIsland
mRNA / METH Corr. FDR
0.01
1e−5
MITF
SOX10
TRPM1
ZEB1
AXL
Gene / Regulator Corr. P
0.05
0.01

CLMP

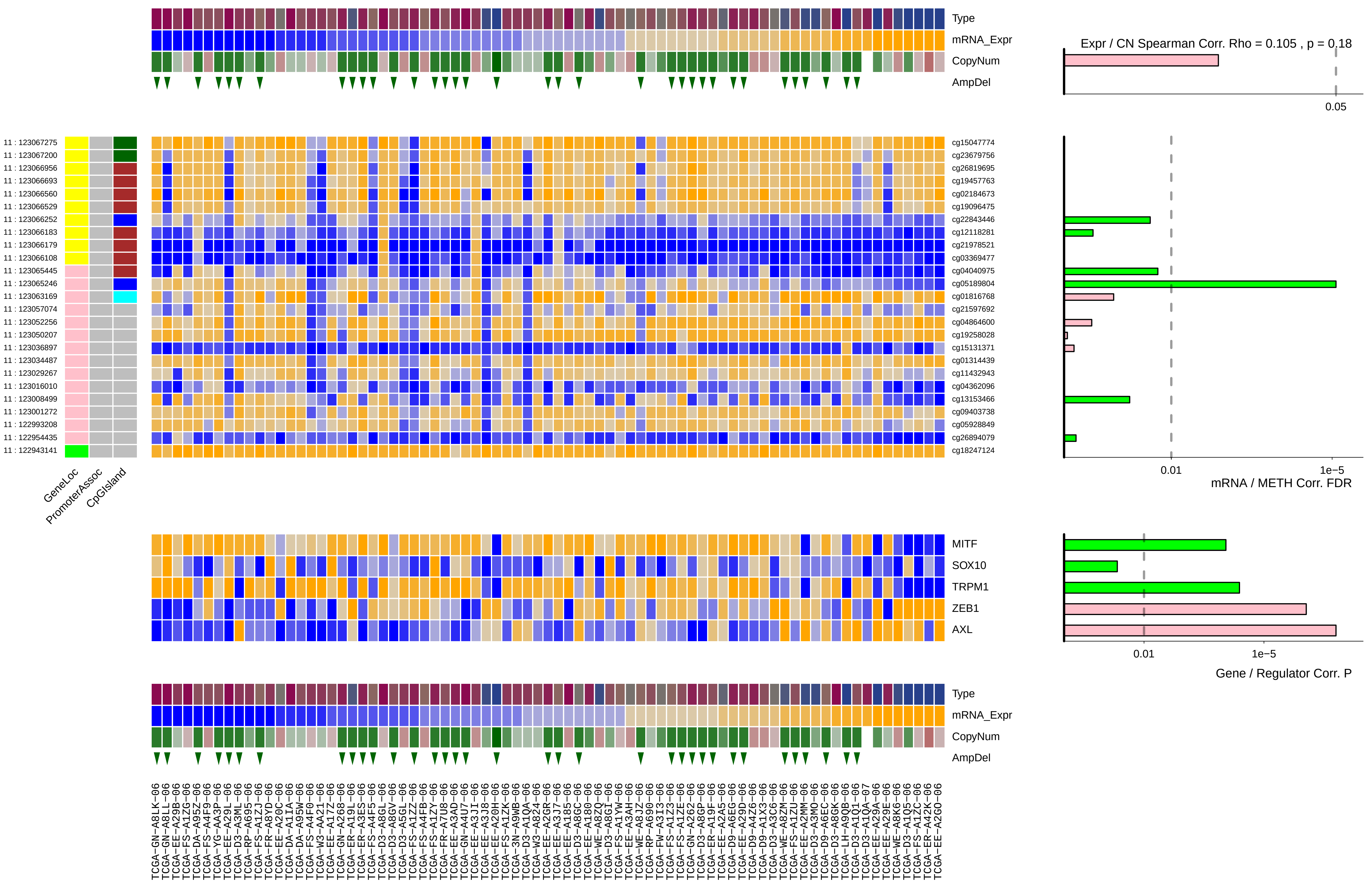

NRG1

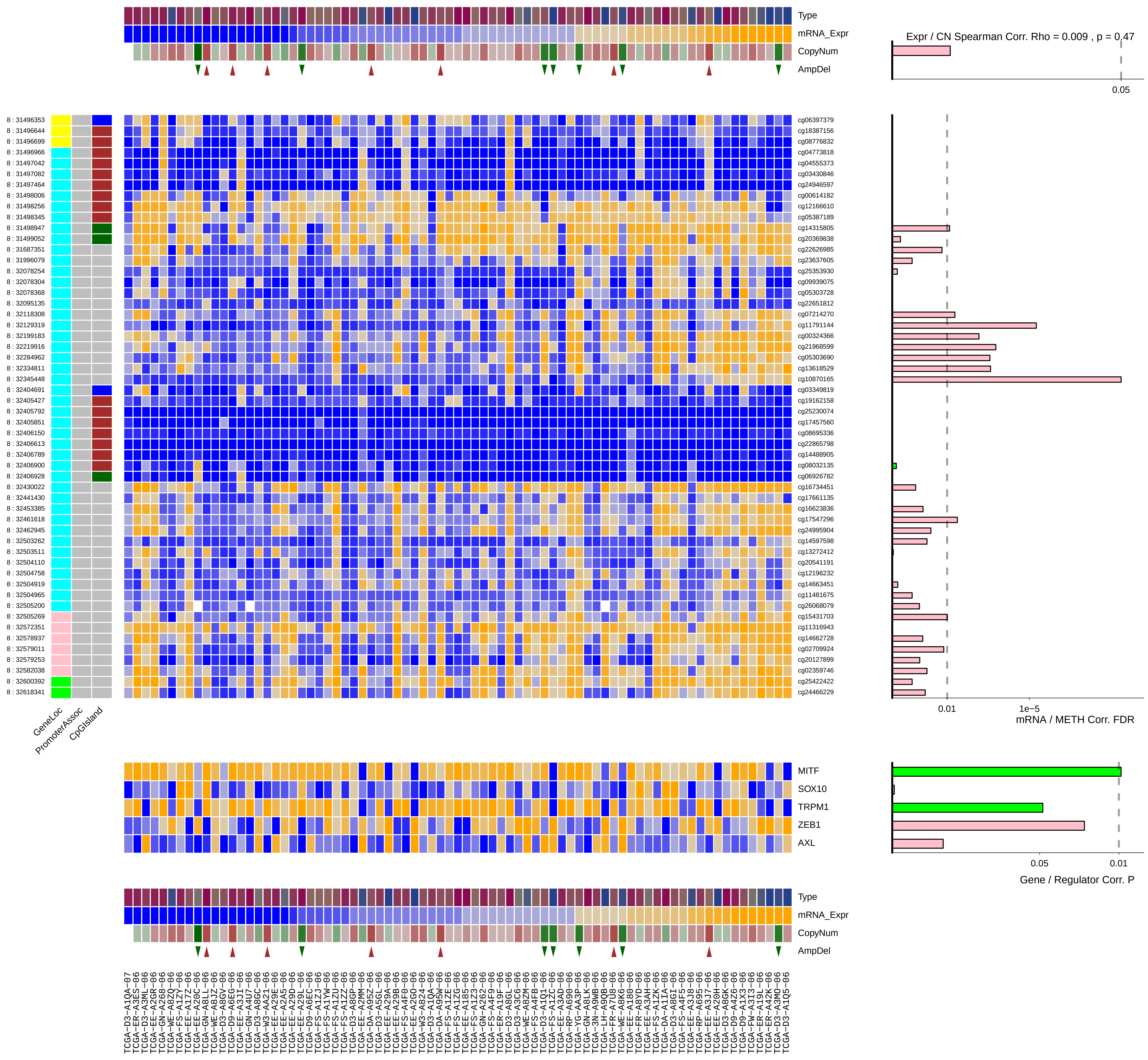

Type
mRNA_Expr
CopyNum
AmpDel
Expr / CN Spearman Corr. Rho = 0.009 , p = 0.47
0.05
GeneLoc
PromoterAssoc
CpGIsland
0.01
1e−5
mRNA / METH Corr. FDR
MITF
SOX10
TRPM1
ZEB1
AXL
0.05
0.01
Gene / Regulator Corr. P

ITGA11

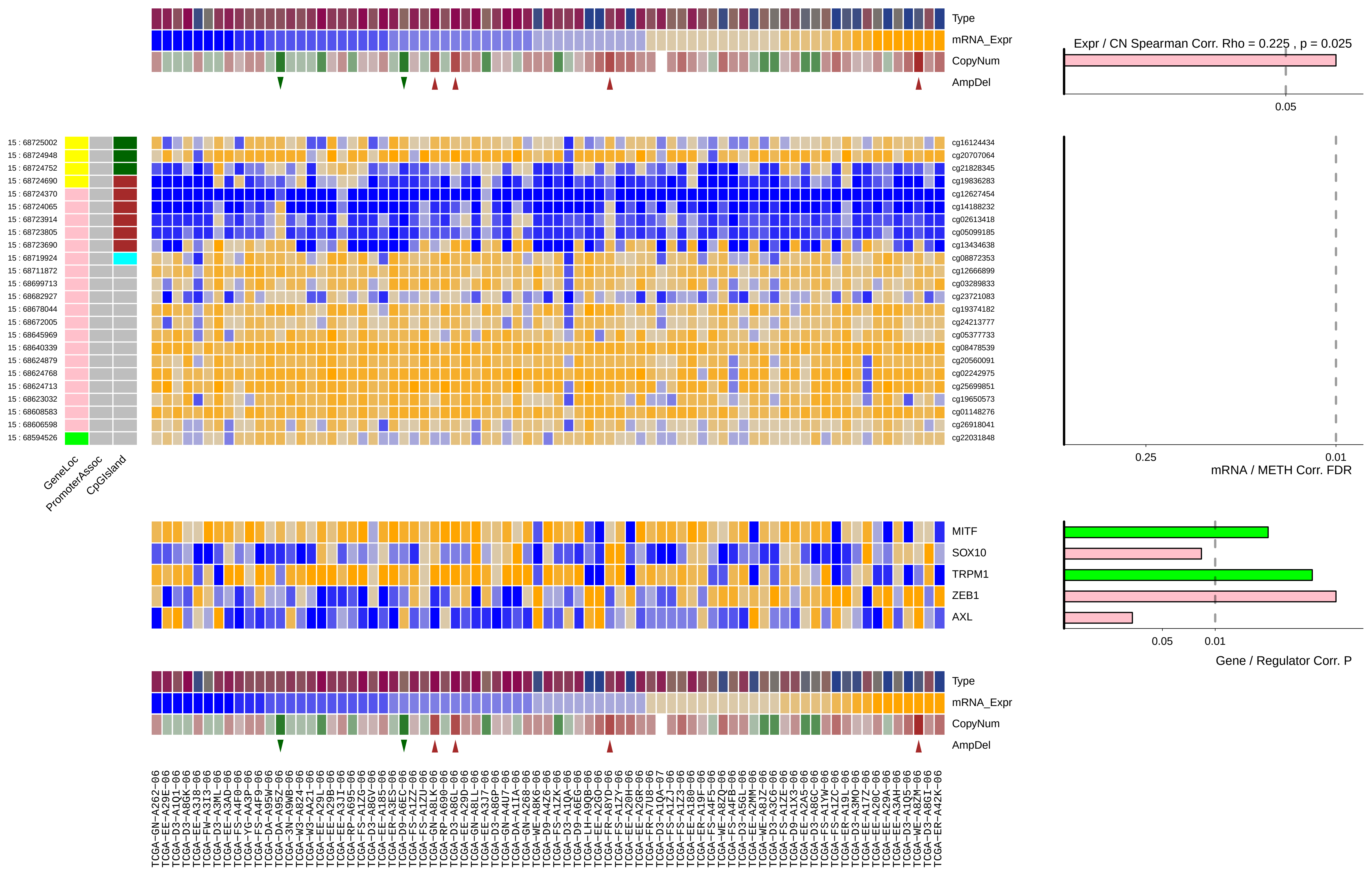
Type
mRNA_Expr
CopyNum
AmpDel
Expr / CN Spearman Corr. Rho = 0.225 , p = 0.025
0.05
mRNA / METH Corr. FDR
0.25
0.01
GeneLoc
PromoterAssoc
CpGIsland
MITF
SOX10
TRPM1
ZEB1
AXL
Gene / Regulator Corr. P

RARRES3

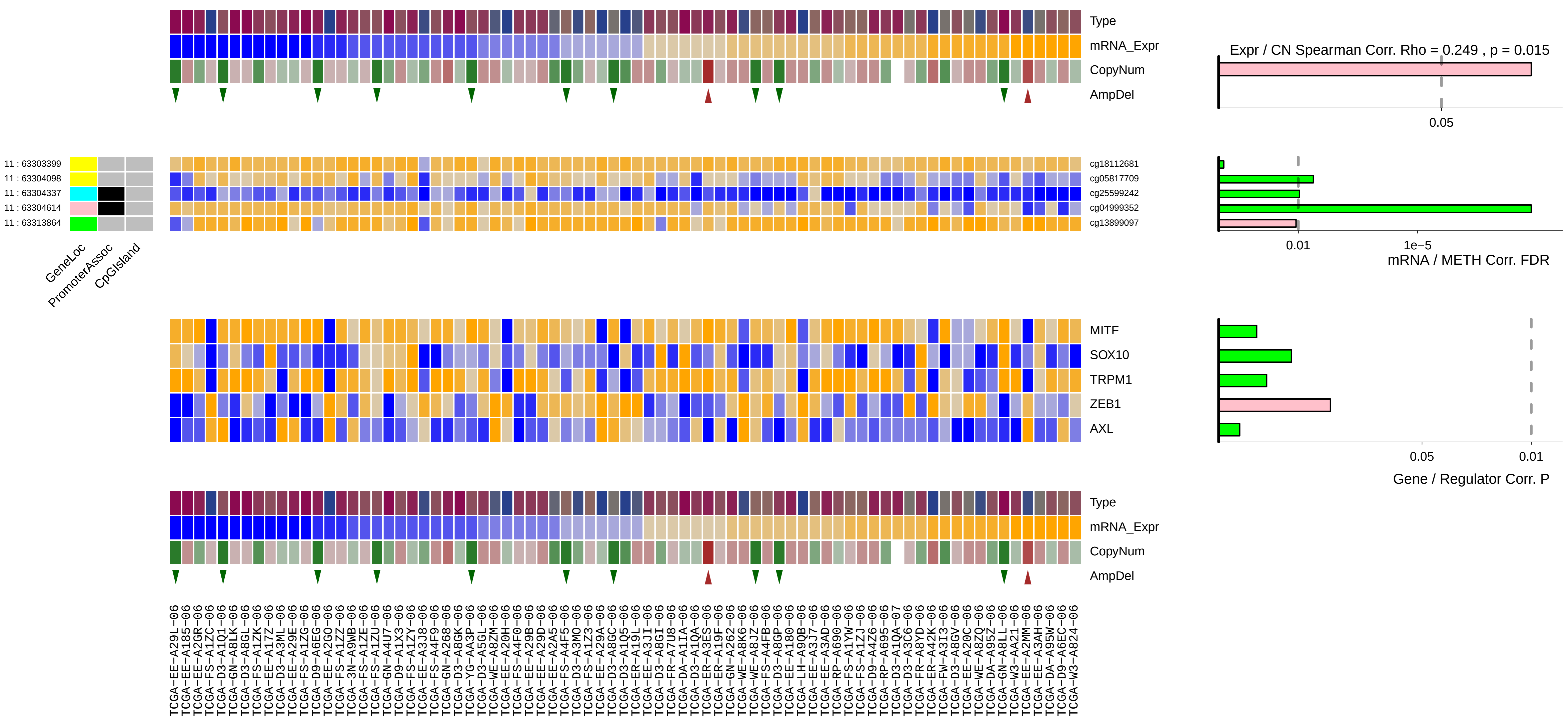

Type
mRNA_Expr
CopyNum
AmpDel
Expr / CN Spearman Corr. Rho = 0.249 , p = 0.015
0.05
11 : 63303399
11 : 63304098
11 : 63304337
11 : 63304614
11 : 63313864
GeneLoc
PromoterAssoc
CpGIsland
cg18112681
cg05817709
cg25599242
cg04999352
cg13899097
0.01
1e−5
mRNA / METH Corr. FDR
MITF
SOX10
TRPM1
ZEB1
AXL
0.05
0.01
Gene / Regulator Corr. P

FHL1

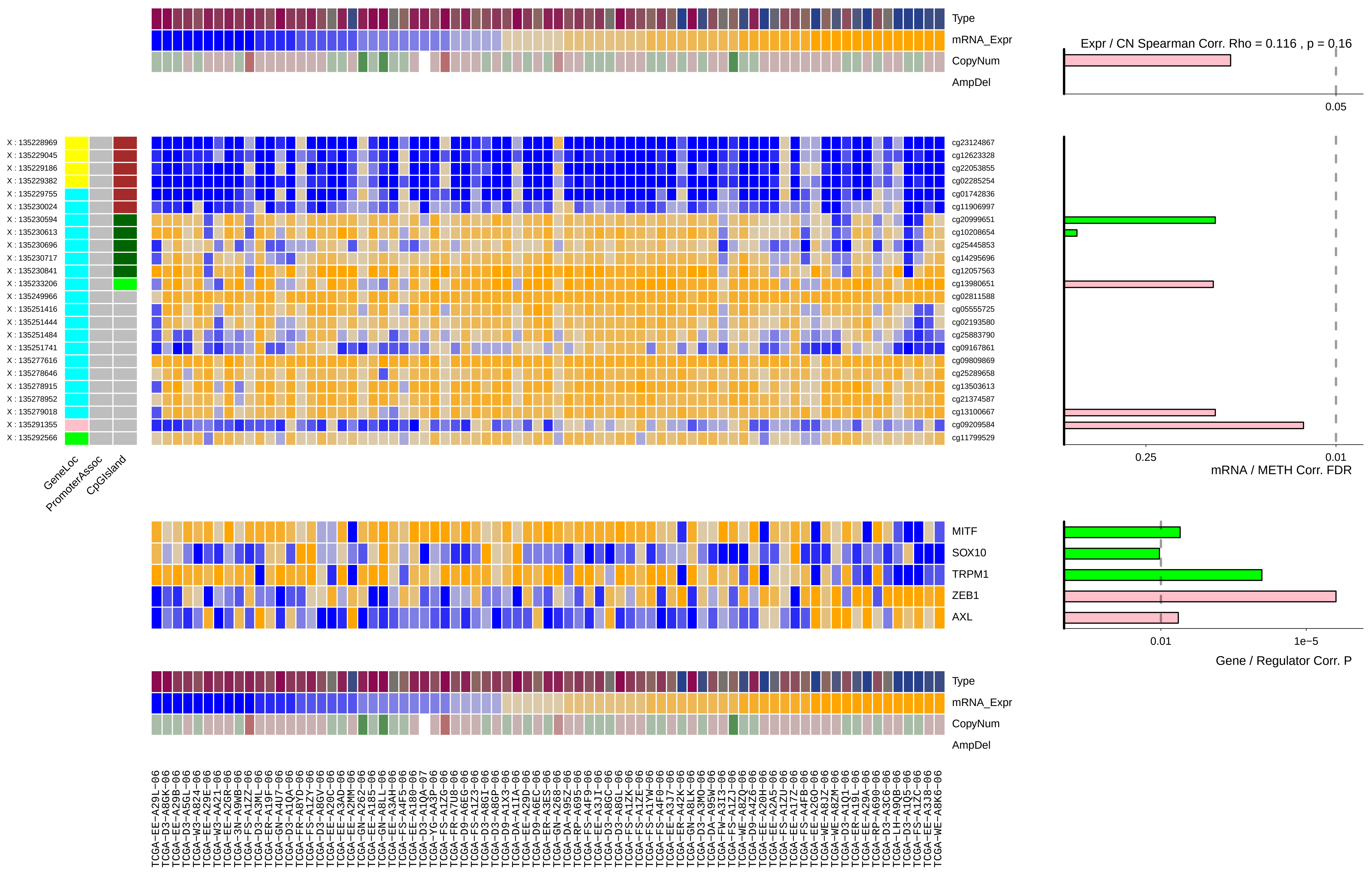

Type
mRNA_Expr
CopyNum
AmpDel
Expr / CN Spearman Corr. Rho = 0.116 , p = 0.16
0.05
0.25
0.01
mRNA / METH Corr. FDR
GeneLoc
PromoterAssoc
CpGIsland
MITF
SOX10
TRPM1
ZEB1
AXL
0.01
1e−5
Gene / Regulator Corr. P

DSE

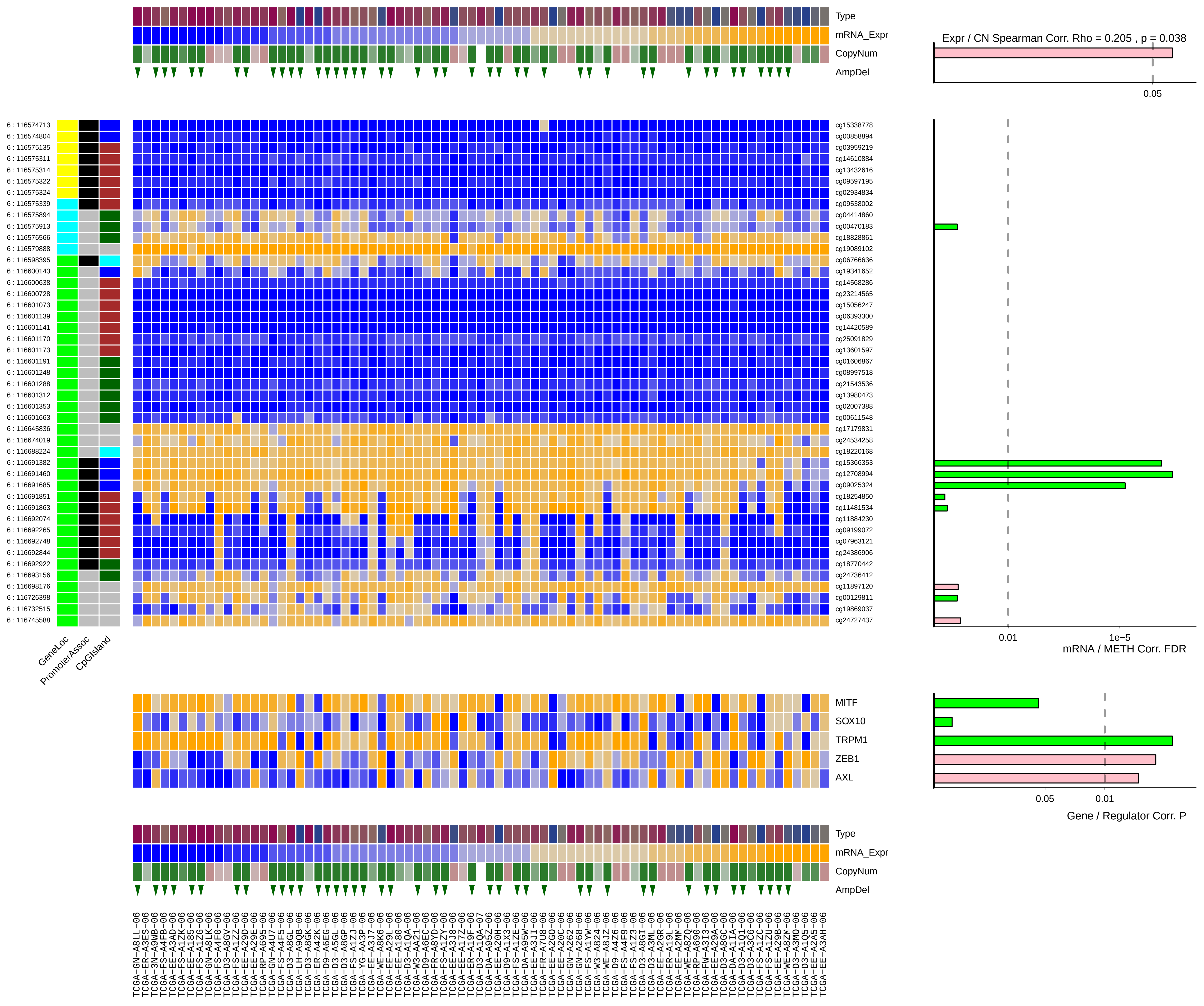

Type
mRNA_Expr
CopyNum
AmpDel
Expr / CN Spearman Corr. Rho = 0.205 , p = 0.038
0.05
GeneLoc
PromoterAssoc
CpGIsland
0.01
1e−5
mRNA / METH Corr. FDR
MITF
SOX10
TRPM1
ZEB1
AXL
0.05
0.01
Gene / Regulator Corr. P

ESM1

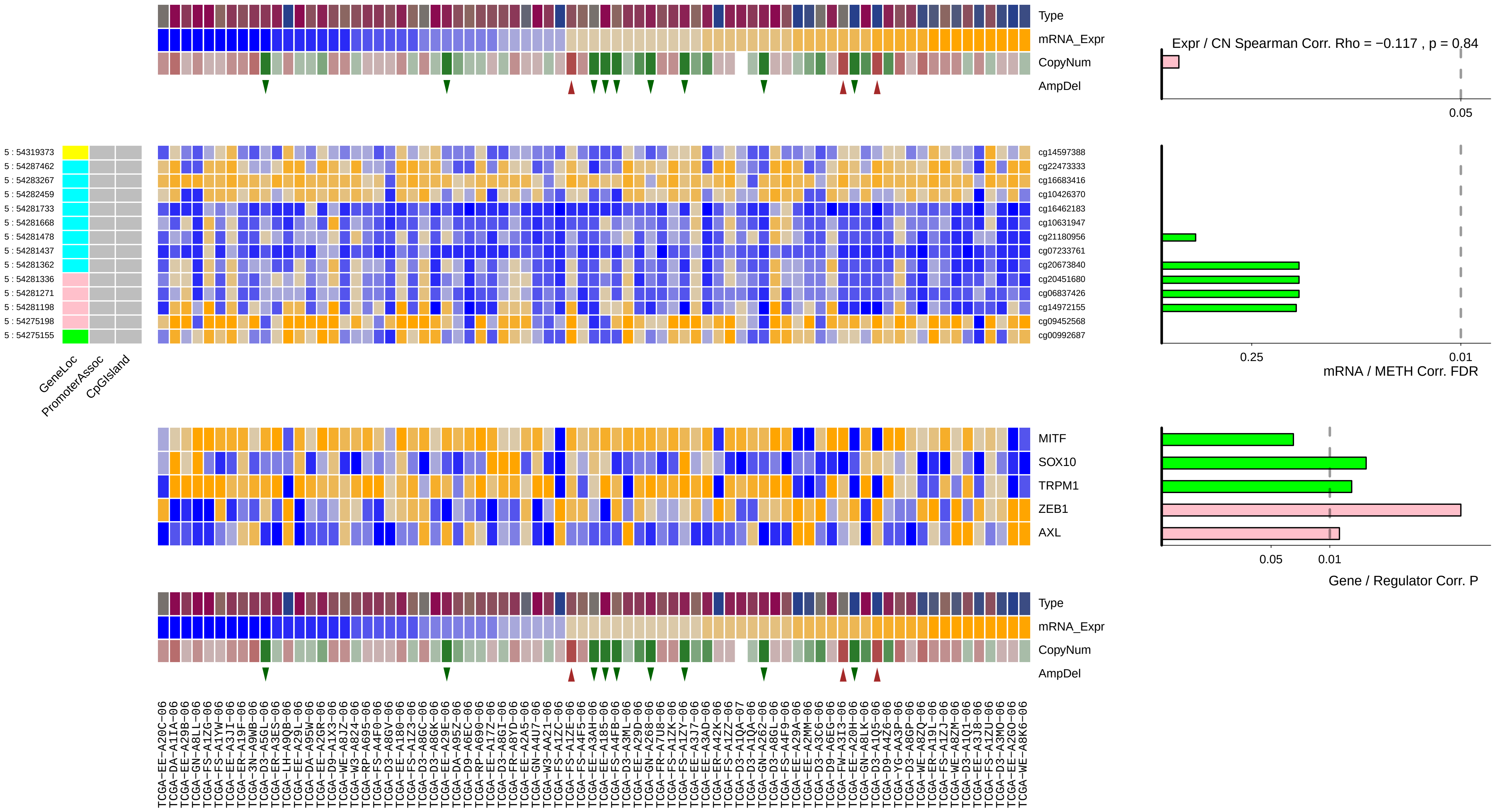
Type
mRNA_Expr
CopyNum
AmpDel
Expr / CN Spearman Corr. Rho = −0.117 , p = 0.84
0.05
5 : 54319373
5 : 54287462
5 : 54283267
5 : 54282459
5 : 54281733
5 : 54281668
5 : 54281478
5 : 54281437
5 : 54281362
5 : 54281336
5 : 54281271
5 : 54281198
5 : 54275198
5 : 54275155
GeneLoc
PromoterAssoc
CpGIsland
cg14597388
cg22473333
cg16683416
cg10426370
cg16462183
cg10631947
cg21180956
cg07233761
cg20673840
cg20451680
cg06837426
cg14972155
cg09452568
cg00992687
0.25
0.01
mRNA / METH Corr. FDR
MITF
SOX10
TRPM1
ZEB1
AXL
0.05
0.01
Gene / Regulator Corr. P
Type
mRNA_Expr
CopyNum
AmpDel

SERPINB7

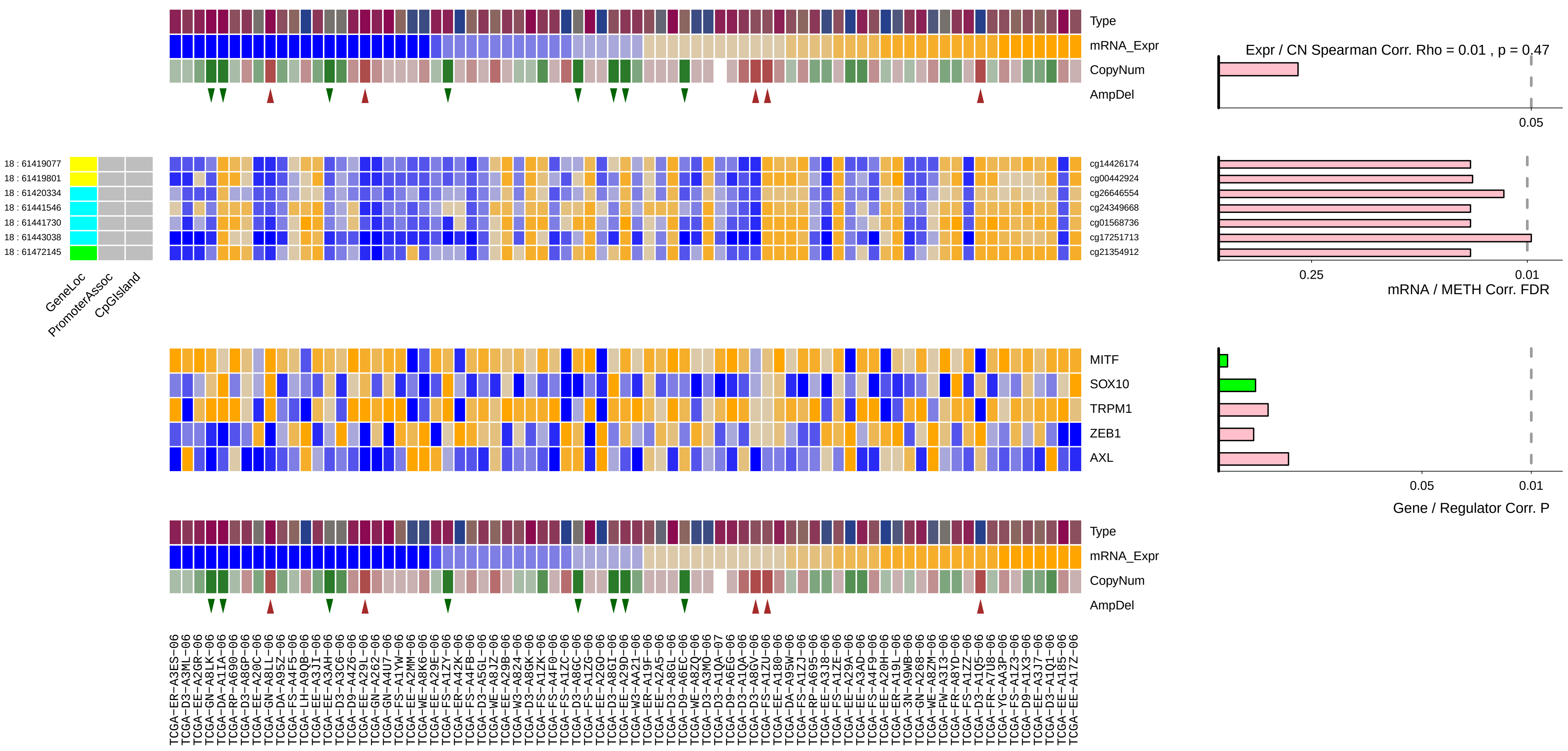

Type
mRNA_Expr
CopyNum
AmpDel
Expr / CN Spearman Corr. Rho = 0.01 , p = 0.47
0.05
18 : 61419077
18 : 61419801
18 : 61420334
18 : 61441546
18 : 61441730
18 : 61443038
18 : 61472145
GeneLoc
PromoterAssoc
CpGIsland
cg14426174
cg00442924
cg26646554
cg24349668
cg01568736
cg17251713
cg21354912
0.25
0.01
mRNA / METH Corr. FDR
MITF
SOX10
TRPM1
ZEB1
AXL
0.05
0.01
Gene / Regulator Corr. P

PLPP3

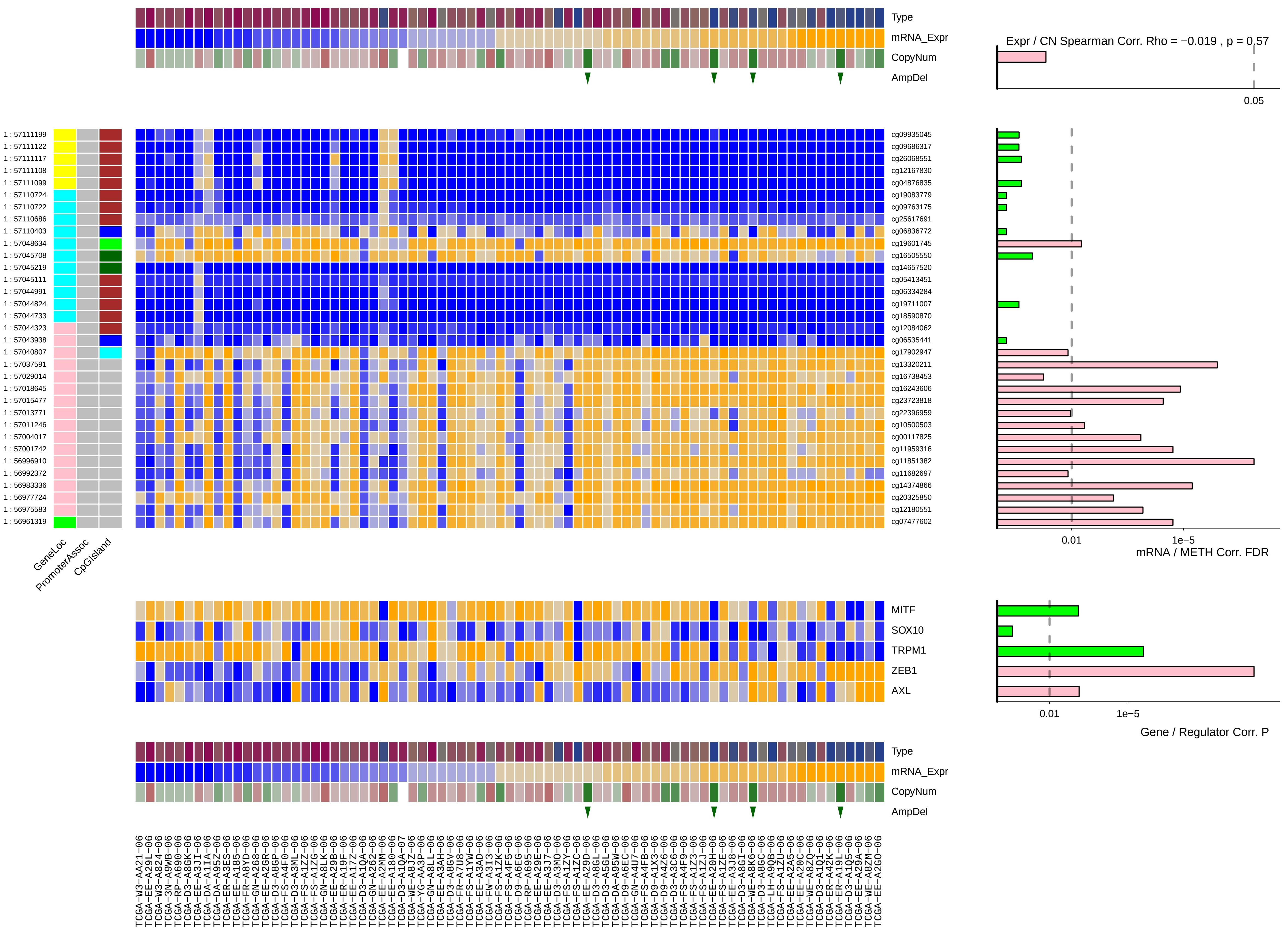
Type
mRNA_Expr
CopyNum
AmpDel
Expr / CN Spearman Corr. Rho = −0.019 , p = 0.57
0.05
GeneLoc
PromoterAssoc
CpGIsland
0.01
1e−5
mRNA / METH Corr. FDR
MITF
SOX10
TRPM1
ZEB1
AXL
Gene / Regulator Corr. P

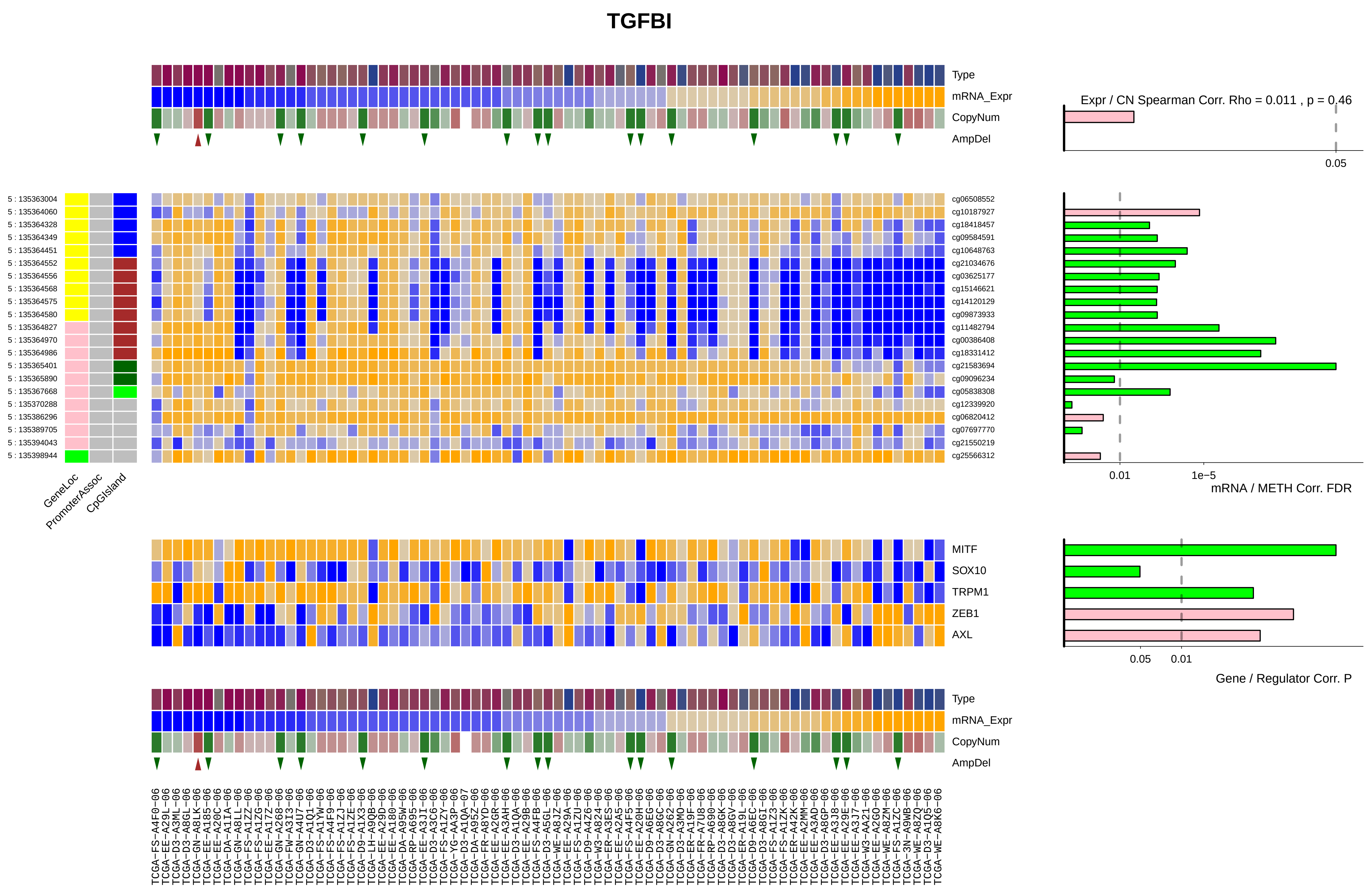

TGFBI
Type
mRNA_Expr
CopyNum
AmpDel
Expr / CN Spearman Corr. Rho = 0.011 , p = 0.46
0.05
5 : 135363004
5 : 135364060
5 : 135364328
5 : 135364349
5 : 135364451
5 : 135364552
5 : 135364556
5 : 135364568
5 : 135364575
5 : 135364580
5 : 135364827
5 : 135364970
5 : 135364986
5 : 135365401
5 : 135365890
5 : 135367668
5 : 135370288
5 : 135386296
5 : 135389705
5 : 135394043
5 : 135398944
cg06508552
cg10187927
cg18418457
cg09584591
cg10648763
cg21034676
cg03625177
cg15146621
cg14120129
cg09873933
cg11482794
cg00386408
cg18331412
cg21583694
cg09096234
cg05838308
cg12339920
cg06820412
cg07697770
cg21550219
cg25566312
0.01
1e−5
mRNA / METH Corr. FDR
GeneLoc
PromoterAssoc
CpGIsland
MITF
SOX10
TRPM1
ZEB1
AXL
0.05
0.01
Gene / Regulator Corr. P
