# Supplementary material for: Multi-modal molecular programs regulate melanoma cell state
Source: Nat Commun. 2022 Jul 9;13:4000. doi: 10.1038/s41467-022-31510-1 (PMC9271073; doi:10.1038/s41467-022-31510-1)
Supplement: Supplementary file 14 — Reporting Summary [file 41467_2022_31510_MOESM14_ESM.pdf]

nature portfolio

Corresponding author(s): Scott E. Woodman, MD, PhD

Last updated by author(s): June 17, 2022

Reporting Summary

Nature Portfolio wishes to improve the reproducibility of the work that we publish. This form provides structure for consistency and transparency in reporting. For further information on Nature Portfolio policies, see our Editorial Policies and the Editorial Policy Checklist.

Statistics

For all statistical analyses, confirm that the following items are present in the figure legend, table legend, main text, or Methods section.

n/a	Confirmed	
☐	☒	The exact sample size (*n*) for each experimental group/condition, given as a discrete number and unit of measurement
☐	☒	A statement on whether measurements were taken from distinct samples or whether the same sample was measured repeatedly
☐	☒	The statistical test(s) used AND whether they are one- or two-sided<br>*Only common tests should be described solely by name; describe more complex techniques in the Methods section.*
☐	☒	A description of all covariates tested
☐	☒	A description of any assumptions or corrections, such as tests of normality and adjustment for multiple comparisons
☐	☒	A full description of the statistical parameters including central tendency (e.g. means) or other basic estimates (e.g. regression coefficient) AND variation (e.g. standard deviation) or associated estimates of uncertainty (e.g. confidence intervals)
☐	☒	For null hypothesis testing, the test statistic (e.g. *F*, *t*, *r*) with confidence intervals, effect sizes, degrees of freedom and *P* value noted<br>*Give P values as exact values whenever suitable.*
☒	☐	For Bayesian analysis, information on the choice of priors and Markov chain Monte Carlo settings
☒	☐	For hierarchical and complex designs, identification of the appropriate level for tests and full reporting of outcomes
☐	☒	Estimates of effect sizes (e.g. Cohen's *d*, Pearson's *r*), indicating how they were calculated

Our web collection on statistics for biologists contains articles on many of the points above.

Software and code

Policy information about availability of computer code

Data collection	Data were collected/collated directly in tabulated form using Microsoft Excel (Excel 2016 and later versions) or R (v4.1.0). No custom software were created/used for data collection in this study. "Custom" implementation of existing software is explained in the methods including details of parameters used.
Data analysis	- General analyses (clinical metadata, basic statistics): R including stats (v4.1.0), dplyr (v1.0.7), dunn.test v1.3.5, Hmisc v4.5-0, reshape2 v1.4.4; Microsoft Excel<br>- RNA sequencing: bcl2fastq2 (v2.17.1.14), STAR (v2.3.0), htseq-count, R package DESeq2 (v1.32.0)<br>- Clustering analyses: R including stats (v4.1.0), cluster (v2.1.2), dendsort v0.3.4; cNMF via GenePattern NMFConsensus module (Broad Institute) and R package NMF (v0.23.0), SubMap code provided in "Submap.R", Hoshida Y, et al., dated 14 Oct 2008 downloaded from the Broad Institute GenePattern module archive 29 July 2021<br>- Feature selection and prediction: R packages caret (v6.0-88), e1071 (v1.7-7), randomForest (v4.6-14)<br>- Gene expression analyses and annotation: GO/KEGG analysis via DAVID (v6.8, http://David.ncifcrf.gov), Broad Institute GSEA desktop module v4.1.0, R package biomaRt (v2.48.2)<br>- Whole exome sequencing: Illumina CASAVA toolkit, BWA-aln, Picard (Broad Institute), GATK (v3.8.1.0), MuTect2, Annovar (rev 521, May 2013), ABSOLUTE<br>- Copy number and purity analysis: ExomeCNV, ExomeLyzer (v1.6.2), HMMCopy (v1.24.0), GISTIC2 (v2.0.21), Sequenza (v2.1.0, Oct 2014), GenVisR (v1.24.0), ABSOLUTE, CPE (Aran D, et al.)<br>- DNA methylation analysis: R package lumi, DNAmAge analysis as per Horvath (Genome Biology 2013 doi:10.1186/gb-2013-14-10-r115)<br>- RPPA analysis: Microsoft Excel (Excel 2016 and later versions), R (v4.1.0), SuperCurve<br>- microRNA analysis: bowtie2, miRDB (v5.0), TargetMiner (2012), TargetScan (v7.1), miRTarBase (v6.1), miRWalk (v2.0)<br>- lncRNA analysis: GENCODE (release 19), RSEM (v1.2.12), SAM (R package samr, v2.0)<br>- Immune deconvolution: MCPcounter (v1.2.0), immunedeconv (v2.0.4)

- Integrative analysis: R package cluster (v2.0.7-1)
- Visualizations: ggplot2 (v3.3.5), circlize (v0.4.13), ComplexHeatmap (v2.8.0), ggVennDiagram (v1.1.4)

For manuscripts utilizing custom algorithms or software that are central to the research but not yet described in published literature, software must be made available to editors and reviewers. We strongly encourage code deposition in a community repository (e.g. GitHub). See the Nature Portfolio guidelines for submitting code & software for further information.

Data

Policy information about availability of data

All manuscripts must include a data availability statement. This statement should provide the following information, where applicable:

- Accession codes, unique identifiers, or web links for publicly available datasets
- A description of any restrictions on data availability
- For clinical datasets or third party data, please ensure that the statement adheres to our policy

The authors declare that the data supporting the findings of this study are available within the paper, its supplementary information files, or in public repositories as indicated in the Data and Software Availability statement:

The genomic data generated in this study have been deposited in the European Genome-Phenome Archive (EGA) under accession EGAS00001004536 (https://ega-archive.org/studies/EGAS00001004536). These data are available upon request to the corresponding author for academic cancer research purposes in accordance with the conditions of consent agreed to by the source participants. Requests will be addressed within 8 weeks and, if approved, access will be made available for a one-year period, renewable upon additional request. Relevant non-identifiable clinical metadata and processed data generated in this study are provided in the Supplementary Data and Source Data. The Cancer Genome Atlas (TCGA) melanoma ("SKCM") cohort publicly available gene expression data used in this study are available from http://gdac.broadinstitute.org/ as the "Broad Firehose 2016" version. Publicly available Cancer Cell Line Encylopedia (CCLE) gene expression (TPM), segmented copy number profile data (segmeans) and annotations are available via the CCLE portal at https://portals.broadinstitute.org/ccle (registration required). Publicly available transcriptome data (raw counts) from the PD-1 inhibitor-treated melanoma clinical and gene expression dataset of Riaz et al. are available from https://github.com/riazn/bms038_analysis/tree/master/. Source data are provided with this paper. The remaining data are available within the Article, Supplementary Information or Source Data file.

Field-specific reporting

Please select the one below that is the best fit for your research. If you are not sure, read the appropriate sections before making your selection.

☒ Life sciences ☐ Behavioural & social sciences ☐ Ecological, evolutionary & environmental sciences

For a reference copy of the document with all sections, see nature.com/documents/nr-reporting-summary-flat.pdf

Life sciences study design

All studies must disclose on these points even when the disclosure is negative.

Sample size	All available early-passage cell lines derived from clinical samples during the period of data generation of this multi-omic platform study were used.
Data exclusions	No data were excluded from the initial transcriptomic analysis and consensus clustering, however the cohort taken for further evaluation with all platforms was refined by removal of samples generated from rarer melanoma sub-types (uveal, mucosal, acral) and only one sample per individual source patient was retained. Not all omic platform data types were able to be generated for all samples included in the study due to technical limitations, as indicated in the methods for each data type.
Replication	The clinical findings cannot be replicated pending further multi-omic studies of similar patient populations. All omic analyses (sequencing, RPPA, etc) were performed with appropriate technical replication; sequencing replicates were collapsed if appropriate following QC analysis. The use of as large a cohort size as was available provides sufficient biological replicates for the cluster types identified; these were further validated using external datasets (e.g. TCGA melanoma "SKCM" dataset).
Randomization	Randomization was not relevant to this observational cohort.
Blinding	Blinding was not possible due to the retrospective observational nature of this cohort including documented clinical outcomes. Primary data collection and processing was performed without knowledge of endpoint data.

Reporting for specific materials, systems and methods

We require information from authors about some types of materials, experimental systems and methods used in many studies. Here, indicate whether each material, system or method listed is relevant to your study. If you are not sure if a list item applies to your research, read the appropriate section before selecting a response.

Materials & experimental systems

n/a	Involved in the study
☐	☒ Antibodies
☐	☒ Eukaryotic cell lines
☒	☐ Palaeontology and archaeology
☒	☐ Animals and other organisms
☐	☒ Human research participants
☒	☐ Clinical data
☒	☐ Dual use research of concern

Methods

n/a	Involved in the study
☒	☐ ChIP-seq
☒	☐ Flow cytometry
☒	☐ MRI-based neuroimaging

Antibodies

Antibodies used	Reverse-phase protein array technology was used in this study to profile sample content of 287 proteins using established methodology provided by the University of Texas MD Anderson Cancer Center Functional Proteomics RPPA Core Facility; detailed information are available at https://www.mdanderson.org/research/research-resources/core-facilities/functional-proteomics-rppa-core.html. Due to the number of antibodies used, details are included together with the RPPA data in Supplementary Data5.<br>Cell cultures derived from metastatic melanoma tumors were stained with a melanoma tumor marker (MCSP, 1:50, Miltenyi, cat# 130-117-347) and with a fibroblast marker (CD90, 1: 50, BD Pharmingen, cat# 561558).<br>ZEB1 protein level was measured by Western Blot using the rabbit anti-human ZEB1 (H-102) polyclonal IgG (1:500, Cat. No. sc-25388, Santa Cruz Biotechnology, CA, USA), and mouse anti-GAPDH monoclonal IgM (1:10000, clone GAPDH-71.1, Cat. No. G9295, Sigma Aldrich, MO, USA) as house-keeping control.
Validation	All antibodies were used in accordance with the stated suitable target organism (Homo sapiens) and underwent extensive validation at the UT MD Anderson Cancer Center Functional Proteomics RPPA Core Facility.

Eukaryotic cell lines

Policy information about cell lines

Cell line source(s)	All early-passage melanoma cell lines were derived from human metastatic tumors at the University of Texas, MD Anderson Cancer Center. Commercially available human melanoma cell lines MEL888, SKMEL23, WM902B, and WM115 were generously provided by Dr. Dr. Michael A. Davies at the University of Texas, MD Anderson Cancer Center.
Authentication	All melanoma cell lines were authenticated using the short-tandem repeat (STR) methodology through the University of Texas, MD Anderson Cancer Center Cell Line Verification Core Facility
Mycoplasma contamination	All melanoma cell lines tested negative for mycoplasma contamination using the MyoAlert detection test
Commonly misidentified lines (See ICLAC register)	No commonly misidentified cell lines were used in this study.

Human research participants

Policy information about studies involving human research participants

Population characteristics	Patients with advanced melanoma of any sub-type were enrolled in the University of Texas MD Anderson Cancer Center's Adoptive T cell Therapy Clinical Program prior to harvest of tumor material from which cell lines were generated. All subsequent analyses presented in this study were derived from this material. All relevant clinical characteristics for each patient-generated cell line are provided in Supplementary Data1.
Recruitment	Patients provided written informed consent to participation in the Adoptive T cell Therapy Clinical Program under local protocols LAB06-0755 and 2004-0069 (NCT00338377: Lymphodepletion plus adoptive cell transfer with or without dendritic cell immunization in patients with metastatic melanoma), including consent to harvest of tumor material and use in parallel cancer-related research as presented in this study. Participants did not receive compensation for their participation.
Ethics oversight	This study was approved by the University of Texas MD Anderson Cancer Center Institutional Review Board. All participants provided written informed consent prior to enrollment. All experimental methods abided by the Declaration of Helsinki.

Note that full information on the approval of the study protocol must also be provided in the manuscript.
